# Proceedings of the 29th European Paediatric Rheumatology Congress

**DOI:** 10.1186/s12969-023-00879-8

**Published:** 2023-10-17

## O01 The childhood arthritis and rheumatology research alliance start time optimization of biologic therapy in polyarticular JIA (STOP-JIA) study: 2- and 3-year outcomes

### S. Ringold^1^, G. Tomlinson^2^, L. E. Schanberg^3,4^, V. Del Gaizo^5^, K. L. Murphy^6^, B. Feldman^7^, M.-S. Ong^8^, M. D. Natter^9^, Y. Kimura^10,11^ on behalf of STOP-JIA CARRA Registry Investigators

#### ^1^Seattle Children's, Seattle, United States; ^2^University of Toronto, Toronto, Canada; ^3^Duke University Medical Center; ^4^Duke Clinical Research Institute, Durham; ^5^Childhood Arthritis and Rheumatology Research Alliance, White House Station; ^6^Louisiana Department of Public Health, New Orleans; ^7^The Hospital for Sick Children, Toronto; ^8^Department of Population Medicine, Harvard Medical School & Harvard Pilgrim Health Care Institute; ^9^Computational Health Informatics Program, Boston Children's Hospital, Boston; ^10^Joseph M. Sanzari Children's Hospital; ^11^Hackensack Meridian School of Medicine, Hackensack, United States

##### **Correspondence:** S. Ringold

*Pediatric Rheumatology 2023*, **21(Suppl 2):**O01

**Introduction:** The STOP-JIA study was designed to compare the effectiveness of the Childhood Arthritis and Rheumatology Research Alliance (CARRA) Consensus Treatment Plans (CTPs) for untreated polyarticular JIA (pJIA) in achieving ACR clinically inactive disease (CID) at 1 year. The CTPs differ in the timing of initiation of biologic disease modifying anti-rheumatic drug therapy (bDMARD).

**Objectives:** To measure the impact of CARRA STOP-JIA CTPs on clinical outcomes at 2 and 3 years.

**Methods:** STOP-JIA compared 3 CARRA CTPs in 400 children with pJIA: 1) Step-Up (SU) – starting conventional, synthetic DMARD monotherapy (csDMARD), adding bDMARD if needed after 3 months; 2) Early Combination (EC) – starting csDMARD and bDMARD within the first 3 months; and 3) Biologic First (BF) – starting bDMARD monotherapy and adding csDMARD if needed after 3 months. There was no randomization. Data were collected using the CARRA Registry approximately every 3 months for the first 12 months and every 6 months thereafter. Patients with 2 to 3 years of follow-up were included. The primary outcome was the percentage of children achieving CID off glucocorticoids at 2 and/or 3 years. Propensity score (PS) weighting was used to balance baseline differences in potential confounders between CTPs. Secondary outcomes included comparison of proportions of patients with clinical Juvenile Arthritis Disease Activity Score based on 10 joints inactive disease (cJADAS10-ID ≤ 2.5), clinical remission on medications (CRM; consecutive visits with CID ≥ 6 months), and proportion of time spent in CID or cJADAS10-ID.

**Results:** 325 participants had a 2- and/or 3-year visit (210 SU, 83 EC, 32 BF). Percentage of patients in CID at 2 years was 42% for SU, 58% EC, and 52% BF (p=0.03 for SU versus EC). CID differences were not statistically significant at 3 years. Likewise, there was no significant difference between CTPs for JADAS10-ID at 2 or 3 years. However, there were significant percentage differences in CRM, which were higher for EC compared to SU at 2 years (46.3% versus 28.8% [p=0.02]) and 3 years (66.1% versus 40.1% [p<0.01]), and for percentages of time spent in CID (42.8% versus 28.8% [p<0.01]) and cJADAS10-ID (52.5% versus 39.4% [p<0.01]) up to 3 years.

**Conclusion:** These data support improved effectiveness of EC versus SU and BF at 2 and 3 years for most outcomes. There were significant differences favoring EC versus SU in outcomes that reflected duration of time spent with less disease activity at 2 and 3 years, which may be most important in limiting disease burden. However, benefits of EC did not reach statistical significance for all comparisons. More research is needed to improve understanding of which pJIA patients will respond to a particular CTP in order to optimize outcomes.

**Trial registration identifying number:** NCT02593006

**Patient Consent**

Not applicable (there are no patient data)

**Disclosure of Interest**

S. Ringold Employee with: Janssen Research & Development, LLC, G. Tomlinson: None declared, L. Schanberg Grant / Research Support with: BMS, Consultant with: Sanofi (DSMB), UCB (DSMB), V. Del Gaizo: None declared, K. Murphy: None declared, B. Feldman Consultant with: AB2Bio (DSMB), Pfizer (DSMB & Advisory Board), Janssen, Novo Nordisk, M.-S. Ong: None declared, M. Natter: None declared, Y. Kimura: None declared

**Reference**

1. Kimura Y, Schanberg LE, Tomlinson GA, Weiss PF, Riordan ME, Dennos AC, Del Gaizo V, Murphy K, Natter M, Feldman BM, Ringold S. The Childhood Arthritis & Rheumatology Research Alliance Start Time Optimization of Biologics in Polyarticular Juvenile Idiopathic Arthritis Study. Arthritis Rheumatol. 2021 Oct;73(10):1898-1909. PMID:34105312

## O02 Recombinant interleukin-1 receptor antagonist is an effective first-line treatment strategy in new-onset systemic juvenile idiopathic arthritis, irrespective of HLA-DRB1 background and IL1RN variants

### R. Erkens^1,2^, J. Calis^1^, A. Verwoerd^1^, S. De Roock^1,2^, N. Ter Haar^1,2^, L. Van der Veken^3^, R. Ernst^3^, H. Van Deutekom^3^, A. Pickering^4^, R. Scholman^1^, M. Jansen^2^, J. Swart^2^, R. Sinha^5^, J. Roth^6^, G. Schulert^7^, A. Grom^7^, J. Van Loosdregt^1^, B. Vastert^1,2^

#### ^1^Center for Translational Immunology, University Medical Center Utrecht; ^2^Department of Pediatric Rheumatology and immunology, Wilhelmina Children's Hospital; ^3^Department of Genetics, Division Laboratories, Pharmacy and Biomedical Genetics, University Medical Center Utrecht, Utrecht, Netherlands; ^4^Department of Biomedical Informatics, Harvard Medical School, Boston; ^5^Systemic JIA foundation, Cincinnati, United States; ^6^Institute of Immunology, University of Münster, Münster, Germany; ^7^Division of Rheumatology, Cincinnati Children’s Hospital and Department of Pediatrics, University of Cincinnati College of Medicine, Cincinnati, United States

##### **Correspondence:** R. Erkens

*Pediatric Rheumatology 2023*, **21(Suppl 2):**O02

**Introduction:** HLA-DRB1*15:01 has been recently associated with interstitial lung disease (LD), eosinophilia, and adverse drug reactions to biological therpay in systemic juvenile idiopathic arthritis (sJIA). Additionally, genetic variants in *IL1RN* have been linked to poor response to anakinra. These findings have spurred a debate among pediatric rheumatologists about the utility of pre-prescription HLA-typing to guide medication decisions for new-onset sJIA patients. Such decisions may include postponing and even forgoing highly effective biological therapy in new-onset sJIA patients who carry the commonly occurring HLA-DRB1*15 haplotypes.

**Objectives:** Here, we present HLA-DRB1 and *IL1RN* variant genotyping data from our prospective cohort of new-onset sJIA patients treated in a standardized manner with the recombinant IL-1 Receptor antagonist anakinra as first-line therapy. The objectives of the study were to describe the HLA-DRB1 background of our sJIA cohort in relation to disease course and to determine clinical inactive disease rates in the first 2 years of disease based on HLA-DRB1 background and *IL1RN* genetic variants.

**Methods:** HLA and *IL1RN* risk alleles were identified via whole genome sequencing. Treatment responses and complications were compared between carriers versus non-carriers.

**Results:** Seventeen of 65 patients (26%) carried HLA-DRB1*15:01, comparable to the general Dutch and European population. Furthermore we found enrichment for HLA-DRB1*11:01 (28%), a known risk locus for sJIA. The rates of clinical inactive disease (CID) at 6 months, 1 and 2 years were high (>80%), irrespective of HLA-DRB1 or *IL1RN* variants. One patient, an HLA-DRB1*15:01 carrier, developed sJIA-LD. Of the three patients with severe drug reactions to biologics, one carried HLA-DRB1*15:01. The prevalence of eosinophilia is common and did not significantly differ between HLA-DRB1*15:01 carriers and non-carriers at disease-onset (6.2% vs 14.9%, p=0.67) nor after the start of anakinra (35.3% versus 37.5% in the first 2 years of disease).

**Conclusion:** We observed high rates of CID using anakinra as first-line treatment irrespective of HLA-DRB1 or *IL1RN* variants. Only one of the 17 HLA-DRB1*15:01 carriers developed sJIA-LD, and of the 3 patients with drug reactions to biologics, only one carried HLA-DRB1*15:01. Although thorough monitoring for sJIA-LD and drug hypersensitivity in sJIA remains important, withholding effective biological therapy in new patients based solely on HLA-DRB1 or genetic *IL1RN* variants is not warranted.

**Patient Consent**

Yes, I received consent

**Disclosure of Interest**

R. Erkens: None declared, J. Calis: None declared, A. Verwoerd: None declared, S. De Roock: None declared, N. Ter Haar: None declared, L. Van der Veken: None declared, R. Ernst: None declared, H. Van Deutekom: None declared, A. Pickering Grant / Research Support with: SJIA Foundation, R. Scholman: None declared, M. Jansen: None declared, J. Swart Consultant with: Amgen, R. Sinha Employee with: President, Systemic JIA Foundation (unpaid), J. Roth: None declared, G. Schulert Consultant with: Novartis and SOBI, A. Grom Consultant with: Novartis, SOBI and AB2Bio, J. Van Loosdregt: None declared, B. Vastert Grant / Research Support with: SOBI, Consultant with: SOBi and Novartis

## O03 Extended report on the long-term prognostic evaluation subsequent to a clinical trial of tocilizumab as first-line biologic therapy in patients with refractory systemic onset Juvenile idiopathic arthritis

### T. Miyamae^1^, T. Kawabe^1^, K. Nishimura^2^, S. Hattori^2^, T. Imagawa^3^, T. Ishii^4^, S. Ito^2^, N. Iwata^5^, Y. Kamata^6^, Y. Kamiyama^2^, M. Mizuta^7^, M. Mori^8,9^, A. Murase^2^, Y. Nakagishi^7^, T. Nakano^10^, S. Nakayamada^11^, T. Nozawa^2^, T. Ohya^2^, N. Okamoto^12,13^, K. Sato^14^, Y. Sugita^12^, S. Takei^15^, S. Tanaka^16^, Y. Tanaka^17^, M. Tomiita^18^, H. Umebayashi^19^, Y. Yamasaki^15^, N. Nishimoto^20,21^, S. Yokota^2^

#### ^1^Department of Pediatric Rheumatology, Institute of Rheumatology, Tokyo Women's Medical University, Tokyo; ^2^Department of Pediatrics, Yokohama City University Graduate School of Medicine; ^3^Infectious Diseases & Immunology, Kanagawa Children's Medical Center, Yokohama; ^4^Clinical Research, Education and Innovation Center, Tohoku University Hospital, Sendai; ^5^Department of Infection and Immunology, Aichi Children’s Health and Medical Center, Obu; ^6^Division of Rhematology and Clinical Immunology, Jichi Medical University, uShimotsuke-shi, Tochigi; ^7^Department of Pediatric Rheumatology, Hyogo Prefectual Kobe Children's Hospital, Kobe; ^8^Department of Lifetime Clinical Immunology, Tokyo Medical and Dental University, Tokyo; ^9^Division of Rheumatology and Allergology, Department of Internal Medicine, St. Marianna University School of Medicine, Kawasaki; ^10^Department of Pediatrics, Graduate School of Medicine, Chiba University, Chiba; ^11^The First Department of Internal Medicine, Japan, University of Occupational and Environmental Health, Kitakyushu; ^12^Department of Pediatrics, Osaka Medical and Pharmaceutical University, Takatsuki; ^13^Department of Pediatrics, Osaka Rosai Hospital, Sakai; ^14^Division of Rheumatology and Clinical Immunology, Jichi Medical University, Shimotsuke-shi, Tochigi; ^15^Department of Pediatrics, Kagoshima University, Kagoshima; ^16^Department of Pediatrics and Child Health, Kurume University School of Medicine, Kurume; ^17^The First Department of Internal Medicine, University of Occupational and Environmental Health, Japan, Kitakyushu; ^18^Department of Allergy and Rheumatology, Chiba Children's Hospital, Chiba; ^19^Deptment of Rheumatism, Infection Disease, Miyagi Children's Hospital, Sendai, ^20^Osaka Rheumatology Clinic; ^21^Department of Molecular Regulation for Intractable Diseases Institute of Medical Science, Tokyo Medical University, Osaka, Japan

##### **Correspondence:** T. Miyamae

*Pediatric Rheumatology 2023*, **21(Suppl 2):**O03

**Introduction:** In 2008, Tocilizumab (TCZ) was approved for systemic juvenile idiopathic arthritis (sJIA) as an intravenous formulation after its efficacy and safety were demonstrated in a clinical trial conducted in Japan between 2002 and 2008.

**Objectives:** This study aimed to comprehend the long-term prognosis of patients who participated in phases II (MRA011JP), III (MRA316JP), and III/IV (MRA324JP) of the clinical trial. A total of 149 participants were included in the study.

**Methods:** The primary endpoint of this study was the sustained frequency of TCZ administration throughout the prolonged observation period of sJIA. Secondary endpoints encompassed additional long-term prognostic determinants, such as therapeutic conditions, disease status including remission rate and clinical phenotype, complications, social adaptation, employment status, and quantification of health-related quality of life (HRQOL).

**Results:** Results were collected for 147 cases by April 2023. Among the 135 patients (61 male and 74 female) whose medical records were available and final diagnosis was sJIA, the median age of the study participants was 26.7 years at the time of the study, 4.3 years at the onset of sJIA, 9.2 years at the first administration of TCZ, 4.0 years from the onset to the first TCZ administration, and 9.8 years from the initiation of TCZ (all median values). Thirty-four (25.2%) were in medication-free remission, and 97 (71.9%) continued therapy. Of the 97 patients, 91 were treated with b-/ts-DMARDs. Tocilizumab was continued in 83 patients (61.5%), and six were switched to canakinumab (CAN) due to TCZ failure, side effects, or other reasons. Except for an isolated incident of sudden death, three cases resulted in a fatal outcome. Specifically, the causes of mortality were macrophage activation syndrome, sJIA-associated interstitial pneumonia, and disseminated aspergillosis. Forty-four (32.6%) transitioned from acute febrile sJIA to chronic arthritic sJIA, in which chronic arthritis was the primary pathology without systemic inflammation. Macrophage activation syndrome was developed in 33 patients (24.4%) during the clinical course. The most commonly observed complications were osteoporosis in 74 (54.8%), infection requiring hospitalization in 43 (31.8%), and hypertension in 28 (20.7%). The EQ-5D-5L score was 0.89 (mean). The final mean height was 156.6 cm for males and 144.0 cm for females, showing a significant short stature. The college/university enrollment rate was as high as 65.4%, and all but five students were employed.

**Conclusion:** After approximately a decade of observation, 71.9% of the patients remained under treatment, with 61.5% electing to receive TCZ. Conversion to chronic arthritic sJIA was detected in 32.6% of the patient population. Despite the presence of primary disease activity and associated complications, the level of social adjustment observed was satisfactory, implying a favorable impact of the administered treatment.

**Patient Consent**

Yes, I received consent

**Disclosure of Interest**

T. Miyamae Shareholder with: none, Grant / Research Support with: none, Consultant with: none, Employee with: none, Paid Instructor with: none, Speaker Bureau with: Chugai Pharma, Novartis Farma, T. Kawabe Shareholder with: none, Grant / Research Support with: none, Consultant with: none, Employee with: none, Paid Instructor with: none, Speaker Bureau with: none, K. Nishimura Shareholder with: none, Grant / Research Support with: Chugai Pharma, Consultant with: none, Employee with: none, Paid Instructor with: none, Speaker Bureau with: Novaritis Pharma, S. Hattori Shareholder with: none, Grant / Research Support with: Chugai Pharma, Consultant with: none, Employee with: none, Paid Instructor with: none, Speaker Bureau with: none, T. Imagawa Shareholder with: none, Grant / Research Support with: none, Consultant with: none, Employee with: none, Paid Instructor with: none, Speaker Bureau with: none, T. Ishii Shareholder with: none, Grant / Research Support with: none, Consultant with: none, Employee with: none, Paid Instructor with: none, Speaker Bureau with: Chugai Pharma, S. Ito Shareholder with: none, Grant / Research Support with: Chugai Pharma, Consultant with: none, Employee with: none, Paid Instructor with: none, Speaker Bureau with: Novaritis Pharma, Chugai Pharma, N. Iwata Shareholder with: none, Grant / Research Support with: none, Consultant with: none, Employee with: none, Paid Instructor with: none, Speaker Bureau with: ONO PHARMACEUTICAL CO., LTD., GlaxoSmithKline Plc., Y. Kamata Shareholder with: none, Grant / Research Support with: none, Consultant with: none, Employee with: none, Paid Instructor with: none, Speaker Bureau with: none, Y. Kamiyama Shareholder with: none, Grant / Research Support with: Chugai Pharma, Consultant with: none, Employee with: none, Paid Instructor with: none, Speaker Bureau with: none, M. Mizuta Shareholder with: none, Grant / Research Support with: none, Consultant with: none, Employee with: none, Paid Instructor with: none, Speaker Bureau with: Novaritis Pharma, M. Mori Shareholder with: none, Grant / Research Support with: I belong to the department that is financially supported by Chugai, UCB Japan, CSL Behring, Abbvie Japan, Japan Blood Products Organization, AYUMI, Nippon Kayaku, and Asahi-Kasei. , Consultant with: none, Employee with: none, Paid Instructor with: none, Speaker Bureau with: I have received lecture fees from MSD, Chugai, UCB Japan, Abbvie Japan, Japan Blood Products Organization, AYUMI, and Asahi-Kasei., A. Murase Shareholder with: none, Grant / Research Support with: Chugai Pharma, Consultant with: none, Employee with: none, Paid Instructor with: none, Speaker Bureau with: none, Y. Nakagishi Shareholder with: none, Grant / Research Support with: none, Consultant with: none, Employee with: none, Paid Instructor with: none, Speaker Bureau with: CHUGAI PHARMACEUTICAL CO.,LTD. Novartis Pharma K.K. AstraZeneca plc, T. Nakano Shareholder with: none, Grant / Research Support with: Maruho Co., Ltd 500,000 yen, Torii Pharmaceutical Co., Ltd 300,000 yen, Consultant with: none, Employee with: none, Paid Instructor with: none, Speaker Bureau with: none, S. Nakayamada Shareholder with: none, Grant / Research Support with: S. Nakayamada has received research grants from Mitsubishi-Tanabe, Consultant with: none, Employee with: none, Paid Instructor with: none, Speaker Bureau with: S Nakayamada has received consulting fees, lecture fees, and/or honoraria from Bristol-Myers, AstraZeneca, Pfizer, GlaxoSmithKline, AbbVie, Astellas, Asahi-kasei, Sanofi, Chugai, Eisai, Gilead Sciences, Boehringer Ingelheim., T. Nozawa Shareholder with: none, Grant / Research Support with: Chugai Pharma, Consultant with: none, Employee with: none, Paid Instructor with: none, Speaker Bureau with: none, T. Ohya Shareholder with: none, Grant / Research Support with: Chugai Pharma, Consultant with: none, Employee with: none, Paid Instructor with: none, Speaker Bureau with: none, N. Okamoto Shareholder with: none, Grant / Research Support with: none, Consultant with: none, Employee with: none, Paid Instructor with: N.O. has has received consulting fees from Daiichi Sankyo Company Limited; Eli Lilly Japan K.K.; and Swedish Orphan Biovitrum AB, Speaker Bureau with: N.O. has received honoraria or lecture fees from AbbVie Inc.; Amgen Inc.; Asahi Kasei Pharma Corporation; Astellas Pharma Inc.; AstraZeneca PLC; AYUMI Pharmaceutical Co., Ltd.; Bristol Myers Squibb; Chugai Pharmaceutical Co., Ltd.; Eisai Co., Ltd.; Eli Lilly Japan K.K.; GlaxoSmithKline PLC; Mitsubishi Tanabe Pharma Corporation; Novartis Pharma K.K.; Pfizer Inc.; and Teijin Pharma Limited; ., K. Sato Shareholder with: none, Grant / Research Support with: Chugai Pharmaceutical Co., Ltd, Four times, average 1,800,000 yen, Consultant with: none, Employee with: none, Paid Instructor with: none, Speaker Bureau with: Chugai Pharmaceutical Co., Ltd, One time, 100,000 yen, Y. Sugita Shareholder with: none, Grant / Research Support with: none, Consultant with: none, Employee with: none, Paid Instructor with: none, Speaker Bureau with: none, S. Takei Shareholder with: none, Grant / Research Support with: Chugai Pharcaceutical Co.Ltd., Eisai Co.Ltd., Consultant with: none, Employee with: none, Paid Instructor with: none, Speaker Bureau with: Glaxo Smith Klein Pharmaceuticals Ltd, Novartis Pharma K.K., AbbVie, Mitsubishi Tanabe Pharma Corporation, Ayumimi Pharmaceutical Co, Bristol-Myers Squibb, Eli-Lilly,K.K., Asahi Kasei Medical Co.,, Sanofi, K.K. , S. Tanaka Shareholder with: none, Grant / Research Support with: none, Consultant with: none, Employee with: none, Paid Instructor with: none, Speaker Bureau with: none, Y. Tanaka Shareholder with: none, Grant / Research Support with: Y. Tanaka has received research grants from Asahi-Kasei, Abbvie, Chugai, Eisai, Takeda, Daiichi-Sankyo, Behringer-Ingelheim., Consultant with: none, Employee with: none, Paid Instructor with: none, Speaker Bureau with: Y. Tanaka has received speaking fees and/or honoraria from Behringer-Ingelheim, Eli Lilly, Abbvie, Gilead, AstraZeneca, Bristol-Myers, Chugai, Daiichi-Sankyo, Eisai, Pfizer, Mitsubishi-Tanabe, GlaxoSmithKline, M. Tomiita Shareholder with: none, Grant / Research Support with: none, Consultant with: none, Employee with: none, Paid Instructor with: none, Speaker Bureau with: none, H. Umebayashi Shareholder with: none, Grant / Research Support with: none, Consultant with: none, Employee with: none, Paid Instructor with: none, Speaker Bureau with: Novaritis Pharma, Y. Yamasaki Shareholder with: none, Grant / Research Support with: none, Consultant with: none, Employee with: none, Paid Instructor with: none, Speaker Bureau with: none, N. Nishimoto Shareholder with: none, Grant / Research Support with: I have received financial grants from Chugai Pharmaceutical Co. Ltd.,the product company of tocilizumab., Consultant with: I have been work as a paid consultant for Chugai Pharmaceutical Co. Ltd. ,the product company of tocilizumab., Employee with: none, Paid Instructor with: I have been a paid instructor for Chugai Pharmaceutical Co. Ltd. ,the product company of tocilizumab., Speaker Bureau with: I have been paid as a speaker for Chugai Pharmaceutical Co. Ltd. ,the product company of tocilizumab., S. Yokota Shareholder with: none, Grant / Research Support with: none, Consultant with: none, Employee with: none, Paid Instructor with: none, Speaker Bureau with: none

**Reference**

1. Yokota S, et al. Efficacy and safety of tocilizumab in patients with systemic-onset juvenile idiopathic arthritis: a randomized, double-blind, placebo-controlled, withdrawal phase III trial. Lancet. 2008 Mar 22;371(9617):998-1006.

## O04 Non-systemic Juvenile idiopathic arthritis - is the treatment goal achieved?

### K. Vollbach^1^, J. Klotsche^2^, K. Tenbrock^1^, G. Horneff^3^, D. Föll^4^, J. P. Haas^5^, D. Windschall^6^, T. Kallinich^7^, F. Weller^8^, S. Mrusek^9^, K. Mönkemöller^10^, M. Hufnagel^11^, I. Földvari^12^, A. Hospach^13^, R. Trauzeddel^14^, C. Schütz^15^, N. Brück^15^, J. Kümmerle-Deschner^16^, P. Oommen^17^, J. Brunner^18^, F. Dressler^19^, A. Klein^3^, C. Rietschel^20^, M. Klaas^21^, M. Rühlmann^22^, K. Minden^2,7^

#### ^1^Klinik für Kinder- und Jugendmedizin, Universitätsklinikum Aachen, Aachen; ^2^PA Epidemiology, Deutsches Rheuma-Forschungszentrum, Berlin; ^3^Asklepios Kinderklinik Sankt Ausgustin, Sankt Ausgustin; ^4^Klinik für Pädiatrische Rheumatologie und Immunologie, Universitätsklinik Münster, Münster; ^5^Deutsches Zentrum für Kinder- und Jugend-rheumatologie, Garmisch-Partenkirchen; ^6^Klinik für Kinder- und Jugendrheumatologie, St. Josef-Stift Sendenhorst, Sendenhorst; ^7^Department of Pediatric Respiratory Medicine, Immunology and Critical Care Medicine, Charité Universitätsmedizin Berlin, Berlin; ^8^Eltern-Kind-Zentrum Prof.Hess, Klinikum Bremen-Mitte , Bremen; ^9^Kinderarztpraxis, Baden-Baden, ^10^Kinderkrankenhaus der Stadt Köln, Köln; ^11^Sektion Päd. Infektiologie und Rheumatologie, Universitätsklinik Freiburg, Freiburg; ^12^Hamburger Zentrum für Kinder- und Jugendrheumatologie, Hamburg; ^13^Kinderrheumatologie, Klinikum Stuttgart - Olgahospital, Stuttgart; ^14^Klinik für Kinder- und Jugendmedizin, Helios Klinkum Berlin-Buch, Berlin; ^15^Klinik für Kinder- und Jugendmedizin, Universitätsklinikum Carl Gustav-Carus, Dresden; ^16^Klinik für Kinder- und Jugendmedizin, Zentrum für Kinder- und Jugendrheumatologie, arcT, Universitätsklinik Tübingen, Tübingen; ^17^Klinik für Kinder- und Jugendmedizin, Med. Einrichtungen der Heinrich-Heine-Universität Düsseldorf, Düsseldorf, Germany; ^18^Kinder- und Jugendheilkunde, Medizinische Universität Innsbruck, Innsbruck, Austria, ^19^Kinderklinik - Rheumaambulanz, Medizinische Hochschule Hannover, Hannover; ^20^Klinik für Kinder- und Jugendmedizin, Clementine Kinderhospital, Frankfurt; ^21^Klinik für Kinder- und Jugendmedizin, Vivantes Klinkum Friedrichshain, Berlin; ^22^Kinderarztpraxis, Göttingen, Germany

##### **Correspondence:** K. Tenbrock

*Pediatric Rheumatology 2023*, **21(Suppl 2):**O04

**Introduction:** A treat-to-target approach is recommended for all forms of juvenile idiopathic arthritis (JIA), with the goal of achieving inactive disease within the first six months of treatment [1]. Treatments and outcomes in newly diagnosed children and adolescents with JIA are currently being studied as part of the ProKind-Rheuma project.

**Objectives:** To investigate whether the treatment goal of inactive disease is achieved in non-systemic JIA and what factors are associated with failure to achieve the goal.

**Methods:** ProKind-Rheuma is an ongoing multicentre, prospective, non-interventional observational study. Patients with newly diagnosed JIA were enrolled from January 2020 to June 2022 and are being followed prospectively. Physician- and parent-reported data are collected in a standardized way (e.g., disease activity with the cJADAS-10, functional limitation with the Childhood Health Assessment Questionnaire (CHAQ), quality of life with the PedsQL 4.0). Data from patients with non-systemic JIA and a follow-up (FU) at 6 months ± 6 weeks were included. Chi2-Test was performed for categorical variables, Students t-test for continuously distributed variables.

**Results:** Six-month FU data were available for 325 patients with non-systemic JIA (42% oligoarthritis, 35% polyarthritis, 8% enthesitis-related arthritis, 3% psoriatic arthritis, 3% other arthritis) recruited 1.2 (±2.1) months after diagnosis from 17 paediatric rheumatology centres.

At the FU, 43% had reached inactive disease according to the 2021 cJADAS cutoffs [2]. Conversely, more than half had not reached the treatment target, including 69% of oligoarthritis, 48% of polyarthritis, 53% of enthesitis-related arthritis and 43% of psoriatic arthritis patients. One third (35%) of patients still had moderate or high disease activity at FU.

There were no significant differences in age at onset, frequency of ANA or HLA-B27 positivity, cJADAS-10 or PedsQL 4.0 score at baseline, parental education level (>10 years), or time from symptom onset to diagnosis between those who did not reach treatment goal and those who did. However, those who did not reach the treatment target were more likely to have oligoarthritis (50% versus 30%, p=0.025), to receive DMARDs later (2.8±3.7 months versus 1.0±1.3, p=0.001) and were less likely to have a 50% decrease in cJADAS-10 score in the first three months of treatment (61% versus 80%, p=0.049) than those who achieved the treatment target.

**Conclusion:** Under current treatment conditions, less than half of patients achieve the goal of inactive disease after six months of treatment. It seems that especially patients with oligoarthritis need to be treated more effectively.

**Patient Consent**

Yes, I received consent

**Disclosure of Interest**

K. Vollbach: None declared, J. Klotsche: None declared, K. Tenbrock: None declared, G. Horneff Speaker Bureau with: Novartis, MSD, Pfizer, Roche, Sanofi, Sobi, Biogen, D. Föll: None declared, J. Haas: None declared, D. Windschall: None declared, T. Kallinich: None declared, F. Weller: None declared, S. Mrusek: None declared, K. Mönkemöller: None declared, M. Hufnagel: None declared, I. Földvari: None declared, A. Hospach: None declared, R. Trauzeddel: None declared, C. Schütz: None declared, N. Brück: None declared, J. Kümmerle-Deschner: None declared, P. Oommen: None declared, J. Brunner: None declared, F. Dressler: None declared, A. Klein: None declared, C. Rietschel: None declared, M. Klaas: None declared, M. Rühlmann: None declared, K. Minden Speaker Bureau with: Amgen, Novartis

**References**

1. Ravelli A, Consolaro A, Horneff G, et al. Ann Rheum Dis 2018;77:819-28.
2. Trincianti C, Van Dijkhuizen EHP, Alongi A, et al.; PRINTO. Arthritis Rheumatol 2021;73:1966-75.

## O05 Incidence of uveitis and uveitis related complications in children with juvenile idiopathic arthritis: results from the childhood arthritis prospective study

### J. Jennycloss^1^, L. Kearsley-Fleet^1^, K. L. Hyrich^1,2^, C. Ciurtin^3^, F. McErlane^4^, S. Lawson-Tovey^2,5^, A. L. Solebo^6,7,8^ on behalf of on behalf of the CAPS Investigators group

#### ^1^Centre for Epidemiology Versus Arthritis, The University of Manchester; ^2^NIHR Manchester Biomedical Research Centre, Manchester University NHS Foundation Trust, Manchester; ^3^Centre for Adolescent Rheumatology, 5 University Street, University College London, London; ^4^Paediatric Rheumatology, Great North Children's Hospital, Newcastle Hospitals NHS Foundation Trust, Newcastle upon Tyne; ^5^Centre for Genetics and Genomics Versus Arthritis, The University of Manchester, Manchester; ^6^Population, Policy and Practice Department, UCL GOS Institute of Child Health, University College London; ^7^NIHR Biomedical Research Centre at UCL GOS Institute of Child Health and Great Ormond Street Hospital; ^8^Great Ormond Street Hospital for Children NHS Trust, London, United Kingdom

##### **Correspondence:** L. Kearsley-Fleet

*Pediatric Rheumatology 2023*, **21(Suppl 2):**O05

**Introduction:** Juvenile idiopathic arthritis (JIA) is the most prevalent inflammatory rheumatic disease in children and young people (CYP) (1, 2). Uveitis, or intraocular inflammation, is the most common extra-articular manifestation of JIA. If JIA-uveitis (JIA-U) is not diagnosed early and thus left untreated, major ocular complications such as cataracts, glaucoma, and blindness can occur (1, 2, 3).

**Objectives:** To describe the incidence and characteristics of JIA-U among a representative inception cohort of CYP with JIA enrolled in the Childhood Arthritis Prospective Study (CAPS).

**Methods:** CAPS, a prospective inception cohort study, recruited CYP aged <16 years with newly diagnosed inflammatory arthritis across seven UK rheumatology centres between January 2001 (4) and July 2019. Analysis included descriptive statistics of all children recruited from the five centres at which ophthalmic data were available. Detailed ophthalmic data were extracted from clinical records by a paediatric ophthalmologist and comprised visual acuity, date of detection of uveitis, inflammation severity at onset, ophthalmic treatment use, and the presence of and date of detection of ocular structural complications.

**Results:** Ophthalmic information was available for 1169 (66%) CYP with JIA recruited to CAPS, of whom 158 (14%) were identified as having uveitis. Most patients with JIA-U (N=158) were female (72%), of white ethnicity (76%), had oligoarticular JIA (58%), and had a history of a positive ANA blood test result (69%). The median time from JIA diagnosis to JIA-U diagnosis was 0.9 years [IQR: 0, 2.5] and the median age at JIA-U diagnosis was 5.7 years [IQR: 3.7, 8.8].

Of the 158 patients reporting uveitis, 94% had anterior uveitis and 6% had anterior and intermediate uveitis at presentation. Disease presented bilaterally in 107 children (68%), and of the 51 with initially unilateral disease, seven progressed to having bilateral disease. At detection of uveitis, complications (cataract, glaucoma, macular oedema, posterior synechiae, band keratopathy and or visual impairment) were present in 23 children (15%) and a further 30% (48/158) went on to develop complications [follow up range 2-10 years, IQR 5, 10]; 8 of these patients had complications at both baseline and follow-up.

**Conclusion:** This is the first analysis on ophthalmic data collected by the CAPS study and provides an opportunity to examine the characteristics of JIA-U in JIA patients in greater detail, with 40% of children with uveitis having sight-threatening complications.

**Patient Consent**

Not applicable (there are no patient data)

**Disclosure of Interest**

J. Jennycloss: None declared, L. Kearsley-Fleet: None declared, K. Hyrich Grant / Research Support with: Grants from Pfizer and BMS outside the submitted work., Speaker Bureau with: Honoraria from Abbvie , C. Ciurtin: None declared, F. McErlane: None declared, S. Lawson-Tovey: None declared, A. L. Solebo: None declared

**References**

1. Carvounis PE, Herman DC, Cha S, Burke JP. Incidence and outcomes of uveitis in juvenile rheumatoid arthritis, a synthesis of the literature. Graefes Arch Clin Exp Ophthalmol. 2006;244(3):281-90. 10.1007/s00417-005-0087-3.
2. Clarke SL, Sen ES, Ramanan AV. Juvenile idiopathic arthritis-associated uveitis. Pediatr Rheumatol Online J. 2016;14(1):27. 10.1186/s12969-016-0088-2.
3. Carlsson E, Beresford MW, Ramanan AV, Dick AD, Hedrich CM. Juvenile Idiopathic Arthritis Associated Uveitis. Children (Basel). 2021;8(8). 10.3390/children8080646.
4. Adib N, Hyrich K, Thornton J, Lunt M, Davidson J, Gardner-Medwin J, et al. Association between duration of symptoms and severity of disease at first presentation to paediatric rheumatology: results from the Childhood Arthritis Prospective Study. Rheumatology (Oxford). 2008;47(7):991-5. 10.1093/rheumatology/ken085.

## O06 Increased incidence of pediatric uveitis associated with the COVID-19 pandemic occurring before COVID-19 vaccine implementation - a time-series analysis

### C. Lafay^1^, Z. ASSAD^1^, N. Ouldali^1^, E. Bui quoc^1^, A. Clement^2^, C. Durand^1^, S. Fares^2^, A. Faye^1^, L.-A. L.-A. Eveillard^1^, F. Kaguelidou^1^, C. Titah^2^, Z. Valtuille^1^, C. Vinit^1^, U. Meinzer^1^, C. Dumaine^1^

#### ^1^Robert-Debré University Hospital; ^2^Adolph Rothschild Hospital, Paris, France

##### **Correspondence:** C. Lafay

*Pediatric Rheumatology 2023*, **21(Suppl 2):**O06

**Introduction:** Viral infections have been suggested as a potential trigger in pediatric uveitis, a rare disease. This led us to suggest that the coronavirus disease 2019 (COVID-19) pandemic could have been a potential cause for the emergence of newly diagnosed uveitis.

**Objectives:** We sought to examine whether the COVID-19 pandemic was associated with an increased incidence of uveitis in children and if this increased incidence was correlated with the beginning of the campain vaccination in june 2021. We then studied children with newly diagnosed uveitis prior to the COVID-19 pandemic to those who were diagnosed after March 2020, to see if there were any clinical or biological differences.

**Methods:** We performed a time-series analysis of patient records from a national hospital-based French surveillance system. All children younger than 18 years hospitalized for uveitis in France between January 2012 and March 2022 were included. The incidence of newly diagnosed uveitis per 100,000 children per trimester in France was analyzed by a quasi-Poisson regression. The incidence of sarcoidosis and acute tubulo-interstitial nephritis over the same period were used as control outcomes. A cohort of children diagnosed with uveitis at Robert-Debré Hospital was described to evaluate wether one specific type of uveitis increased and to compare the clinical and biological characteristics of uveitis diagnosed before and after the onset of the pandemic.

**Results:** During the study period, 2492 children were hospitalized for uveitis in France. The COVID-19 pandemic, which started in March 2020, was associated with a significant increase in the occurrence of uveitis (estimated cumulative change, 44.9%; 95% CI 11.4 to 78.4; *P* < .001). The increase in the incidence of pediatric uveitis started in October 2020, while the national immunization program targeting children aged less than 18 years began in June 2021. This increase involved all forms of uveitis, regardless of location, and their characteristics were similar to those diagnosed before the pandemic.

**Conclusion:** Our study evidenced a significant increase in the incidence of pediatric uveitis following the COVID-19 pandemic. This increase, occurring 6 months before the implementation of the COVID-19 national immunization program in children, suggests the independence of the vaccine regarding the resurgence of this rare disease.

**Patient Consent**

Yes, I received consent

**Disclosure of Interest**

None declared

**References**

1. Tugal-Tutkun I. Pediatric uveitis. *J Ophthalmic Vis Res* 2011; **6**: 259–69.
2. Cunningham ET. Uveitis in children. *Ocul Immunol Inflamm* 2000; **8**: 251–61.
3. Kanski JJ, Shun-Shin GA. Systemic Uveitis Syndromes in Childhood: An Analysis of 340 Cases. *Ophthalmology* 1984; **91**: 1247–52.
4. Pivetti-Pezzi P. Uveitis in Children. *Eur J Ophthalmol* 1996; **6**: 293–8.
5. Soylu M, Özdemir G, Anli A. Pediatric uveitis in Southern Turkey. *Ocul Immunol Inflamm* 1997; **5**: 197–202.
6. Hamade IH, Shamsi HNA, Dhibi HA, Chacra CB, El-Asrar AMA, Tabbara KF. Uveitis survey in children. *Br J Ophthalmol* 2009; **93**: 569–72.
7. Standardization of Uveitis Nomenclature for Reporting Clinical Data. Results of the First International Workshop. *Am J Ophthalmol* 2005; **140**: 509–16.
8. De Groot-Mijnes JDF, Chan ASY, Chee S-P, Verjans GMGM. Immunopathology of Virus-Induced Anterior Uveitis. *Ocul Immunol Inflamm* 2018; **26**: 338–46.
9. Groen-Hakan F, Babu K, Tugal-Tutkun I, *et al.* Challenges of Diagnosing Viral Anterior Uveitis. *Ocul Immunol Inflamm* 2017; **25**: 715–25.
10. Babu K, Murthy G. Chikungunya virus iridocyclitis in Fuchs′ heterochromic iridocyclitis. *Indian J Ophthalmol* 2012; **60**: 73.

## O07 Elevated serum interferon-alpha associates with flare risk in juvenile-onset systemic lupus erythematosus

### V. Natoli^1,2^, Y. J. Crow^3,4^, E. Carter^3^, K. Tharmaratnam^5^, A. J. Jorgensen^5^, M. W. Beresford^1,6^, C. M. Hedrich^1,6^, E. M. Smith^1,6^

#### ^1^Department of Women’s and Children’s Health, Institute of Life Course and Medical Sciences, University of Liverpool, Liverpool, United Kingdom; ^2^Dipartimento di Neuroscienze, Riabilitazione, Oftalmologia, Genetica e Scienze Materno-Infantili, Università degli Studi di Genova, Genoa, Italy; ^3^MRC Human Genetics Unit, Institute of Genetics and Cancer, University of Edinburgh, Edinburgh, United Kingdom; ^4^Laboratory of Neurogenetics and Neuroinflammation, Institut Imagine, Université de Paris, Paris, France; ^5^Department of Health Data Science, University of Liverpool Faculty of Health and Life Sciences; ^6^Department of Paediatric Rheumatology, Alder Hey Children's NHS Foundation Trust Hospital, Liverpool, United Kingdom

##### **Correspondence:** V. Natoli

*Pediatric Rheumatology 2023*, **21(Suppl 2):**O07

**Introduction:** Despite the established role of IFN-α in the pathophysiology of Juvenile-onset Systemic Lupus Erythematosus (JSLE), its utility as a tool for monitoring disease activity has not been explored extensively.

**Objectives:** To assess the utility of serum IFN-α as a potential marker of disease activity, and a predictor of disease flare in JSLE patients who have reached a low disease activity state (LDAS) or remission.

**Methods:** Serum samples from 291 participants were analysed, including 49 healthy controls (HCs), 95 JSLE, and 52 juvenile idiopathic arthritis (JIA) patients. IFN-α levels were determined using ultra-sensitive Single-molecule array (Simoa) digital ELISA. All serum samples were analysed in duplicates, the coefficient of variation (CV) was calculated, and samples with a CV>15 were excluded. Thus, 88 serum samples were excluded and 203 samples were included in the analysis (25 HCs, 85 JSLE (148 samples), 30 JIA patients). At each visit, JSLE patients were classified as either being in: a) remission, b) LDAS, or c) having intermediate or active disease. Clinical characteristics, demographics and disease activity scores were collected. Median IFN-α levels were compared between patient groups and disease activity state sub-groups, cross-sectionally. Time-to-flare was analysed cross-sectionally by linear regression, and the ability of the IFN-α and other traditional biomarkers (erythrocyte sedimentation rate/ESR, low C3, anti-dsDNA antibodies) in predicting flare at the following visit was assessed longitudinally by generalised linear mixed model.

**Results:** Median IFN-α levels were higher in the combined active/intermediate group (median 3,184 fg/mL, IQR 69-14,878) as compared to both the LDAS (586 fg/mL, IQR 52-1,317 fg/mL, p=0.036) and remission sub-groups (271 fg/mL, IQR 3-56, p <0.001). IFN-α levels were comparable between JSLE patients in remission and HCs (23 fg/mL, IQR 3-277, p=0.5). IFN-α concentrations were higher in all JSLE patients (median 603 fg/mL, IQR 11-2,643) as compared to JIA patients (median 3 fg/mL, IQR 3-103, p=0.001) and HCs (p=0.016). Abnormal serum IFN-α levels were defined as >871 fg/mL (mean serum HC IFN-α level + three standard deviations). Cross-sectional JSLE patients in remission or LDAS with abnormal IFN-α levels had a shorter time-to-flare over the subsequent six months (p=0.038), compared to patients with normal IFN-α levels. Longitudinally, multivariable analysis demonstrated high IFN-α to be the only predictor of flare at the next visit (p=0.040), whereas elevated ESR, low C3, and anti-dsDNA antibodies did not predict flares.

**Conclusion:** Serum IFN-α levels correlate with JSLE disease activity and facilitate identification of a sub-group of patients in remission or LDAS who are at increased risk of flare.

**Patient Consent**

Yes, I received consent

**Disclosure of Interest**

None declared

## O08 Paediatric rheumatologists’ perspective on cardiovascular risk assessment and management in young people with childhood onset systemic lupus erythematosus – a pres/carra survey

### C. Ciurtin^1^, M. Butt^1^, G. A. Robinson^1^, J. Peng^1^, S. Ardoin^2^, L. Schanberg^3^, A. Boteanu^4^, K. Bouchalova^5^, S. Demir^6^, E. Moraitis^7^, A. Migowa^8^, Y. Glackin^7^, J. Ainsworth^9^, E. Smith^9^, S. Sahin^10^, S. Kamphuis^11^, E. C. Jury^12^, L. Lewandowski^13^ on behalf of on behalf of the Paediatric Rheumatology European Society (PReS) Lupus Working Party and the Childhood Arthritis and Rheumatology Research Alliance (CARRA).

#### ^1^Centre for Adolescent Rheumatology, University College London, London, United Kingdom; ^2^Department of Pediatrics, Nationwide Children's Hospital, Ohio, ^3^Duke Clinical Research Institute, Department of Pediatrics, Duke University School of Medicine, Durham, United States; ^4^Department of Rheumatology, Ramon y Cajal University Hospital, Madrid, Spain; ^5^Paediatric Rheumatology, Department of Paediatrics, Faculty of Medicine and Dentistry, Palacky University Olomouc and University Hospital, Olomouc, Czech Republic; ^6^Department of Pediatrics, Division of Rheumatology, Hacettepe University Faculty of Medicine, Ankara, Türkiye; ^7^Department of Paediatric Rheumatology, Great Ormond Street Hospital, London, United Kingdom; ^8^Department of Paediatric Rheumatology, Aga Khan University Hospital, Nairobi, Kenya; ^9^Department of Women’s & Children’s Health, Institute of Life Course and Medical Sciences, University of Liverpool, Liverpool, United Kingdom; ^10^Department of Pediatric Rheumatology, Istanbul University Cerrahpasa, Istanbul, Türkiye; ^11^Department of Paediatric Rheumatology, Erasmus MC Sophia Children's Hospital, Rotterdam, Netherlands; ^12^Centre for Rheumatology, Division of Medicine, University College London, London, United Kingdom; ^13^Lupus Genomics and Global Health Disparities Unit, National Institute of Arthritis and Musculoskeletal and Skin Diseases, National Institutes of Health, Bethesda, United States

##### **Correspondence:** C. Ciurtin

*Pediatric Rheumatology 2023*, **21(Suppl 2):**O08

**Introduction:** Atherosclerosis is an early manifestation of cardiovascular disease (CVD) which can be detected in young people (YP), suggesting that cardiovascular risk (CVR) management should start earlier in life. Childhood onset systemic lupus erythematosus (cSLE) has a 100-300-fold increased CVD mortality vs. age-matched population. Although progress has been made in assessing CVR for primary prevention of CVD, there is less guidance for CVR assessment in YP.

**Objectives:** The survey aimed to explore paediatric rheumatologists' perspective on CVR assessment/management in YP with cSLE, as well as potential geographical differences.

**Methods:** A 17-question survey adressing the objectives above was distributed electronically through contact lists to the Paediatric Rheumatology European Society (PReS) and the Childhood Arthritis and Rheumatology Research Alliance (CARRA) members. Respondents were asked to rate some of their choices from 5 - “very important” to 1 - “unimportant”. Findings are reported using descriptive statistics.

**Results:** Out of 170 respondents, 161 (95%) completed the survey (62% were from Europe, 34% from US/Canada and 4% from other countries). The majority (67%) were fully trained paediatric rheumatologists, 9% dually trained, 23% were paediatric rheumatology trainees and 1% allied health professionals; 72% respondents reported seeing up to 10 cSLE patients/month. There was a significant agreement that YP with cSLE have a higher CVR compared to age-matched population (95%), and that CVR assessment in cSLE is warranted (95%), with the annual assessment being the most preferred option (70%). Despite 50% of respondents not being aware of any CVD-risk scores, 90% agreed with the need of validating a CVR score in YP with cSLE, and 70% of respondents indicated that they would use such a tool to guide lifestyle changes, while 50% would use it to either assess response to CVR management interventions or calculate the patients’ theoretical CVR. Previous CVD events (99%), smoking (98%), obesity and hyperglycaemia (87%), atherosclerosis lesions on vascular scans (86%), hypertension and BMI (85%), and increased LDL-cholesterol level (79%) were the top factors rated as "very” or “moderately important" for CVR assessment in cSLE. The most preferred CVR management interventions were tight control of cSLE and diabetes (78%), increase in physical activity (77%), followed by diet (72%) and tapering steroids (65%), with 51% respondents supporting the use of statins. Interestingly, 66% rated deprivation (1) as "very” or “moderately important" in determining CVR (98% from US vs. 21% from Europe p<0.001) with no differences between trainees and consultants (78% vs. 80%, p=0.89). No other significant geographical or training level differences were noted.

**Conclusion:** This is the first worldwide survey investigating paediatric rheumatologists' perspective on CVR in cSLE, which provided preliminary evidence for good consensus for most of the proposed strategies for CVR assessment and management in cSLE. There was a significant geographical difference in recognising the role of patient’s deprivation in determining CVR, which may require further exploration.

**Patient Consent**

Not applicable (there are no patient data)

**Disclosure of Interest**

None declared

**Reference**

1. Kimenai DM, et al. Circulation. 2022 Jul 19;146(3):240-248

## O09 Phosphomevalonate kinase deficiency expands the genetic spectrum of systemic autoinflammatory diseases

### J. Brunner^1^, J. Berner^2^, R. J. Heredia^2^, C. van de Wetering^3^, C. R. Rashkova^4^, J. Weiss^1^, A. Frohne^4^, S. Giuliani^4^, S. Ferdinandusse, ^5^, H. Waterham^5^, I. Castanon^4^, K. Boztug^4^

#### ^1^Department of Pediatrics, Medical University Innsbruck, Innsbruck; ^2^Department of Pediatrics and Adolescent Medicine, Medical University Vienna; ^3^Department of Pediatrics; ^4^Department of Pediatrics and Adolescent Medicine, St. Anna Children’s Cancer Research Institute , Vienna, Austria; ^5^Department of Clinical Chemistry, Amsterdam UMC location University of Amsterdam, Amsterdam, Netherlands

##### **Correspondence:** J. Brunner

*Pediatric Rheumatology 2023*, **21(Suppl 2):**O09

**Introduction:** In the isoprenoid biosynthesis pathway, mevalonate is phosphorylated in two subsequent enzyme steps by mevalonate kinase (MVK) and phosphomevalonate kinase (PMVK) to generate mevalonate pyrophosphate that is further metabolized to produce sterol and non-sterol isoprenoids. Biallelic pathogenic variants in the *MVK* gene result in the autoinflammatory metabolic disorder MVK Deficiency (MKD). So far, however, no patients with PMVK deficiency due to biallelic pathogenic variants in the *PMVK* gene have been reported.

**Objectives:** This study aims to report the first patient with proven PMVK deficiency, including the clinical, biochemical and immunological consequences of a homozygous pathogenic variant in the *PMVK* gene.

**Methods:** We performed whole exome sequencing and functional studies in cells from a patient who, upon clinical and immunological evaluation, was suspected of an autoinflammatory disease*.*

**Results:** We identified a biallelic homozygous variant in the *PMVK* gene of the patient *(*NM_006556.4: c.392T>C, p.Val131Ala). Pathogenicity was confirmed by functional studies in patient cells, which revealed a markedly reduced PMVK enzyme activity due to a virtually complete absence of PMVK protein. Clinically, the patient showed various similarities but also distinct features compared to MKD patients, and responded well to therapeutic IL-1 inhibition.

**Conclusion:** In this study we report the first patient with proven PMVK deficiency due to a homozygous loss-of-function variant in *PMVK* leading to an autoinflammatory disease. PMVK deficiency expands the genetic spectrum of the systemic autoinflammatory diseases (SAID), characterized by recurrent fevers, arthritis and cytopenia and thus should be included in the differential diagnosis and genetic testing for SAIDs.

**Trial registration identifying number:** -

**Patient Consent**

Yes, I received consent

**Disclosure of Interest**

None declared

**References**

1. Hansmann S, Lainka E, Horneff G, Holzinger D, Rieber N, Jansson AF, et al. Consensus protocols for the diagnosis and management of the hereditary autoinflammatory syndromes CAPS, TRAPS and MKD/HIDS: a German PRO-KIND initiative. Pediatr Rheumatol Online J. 2020 Feb;18(1):17.
2. van der Hilst JCH, Bodar EJ, Barron KS, Frenkel J, Drenth JPH, van der Meer JWM, et al. Long-term follow-up, clinical features, and quality of life in a series of 103 patients with hyperimmunoglobulinemia D syndrome. Medicine (Baltimore). 2008 Nov;87(6):301–10.
3. ter Haar NM, Jeyaratnam J, Lachmann HJ, Simon A, Brogan PA, Doglio M, et al. The Phenotype and Genotype of Mevalonate Kinase Deficiency: A Series of 114 Cases From the Eurofever Registry. Arthritis Rheumatol. 2016;68(11):2795–805.
4. Yıldız Ç, Gezgin Yıldırım D, Inci A, Tümer L, Cengiz Ergin FB, Sunar Yayla ENS, et al. A possibly new autoinflammatory disease due to compound heterozygous phosphomevalonate kinase gene mutation. Jt Bone Spine. 2023;90(1):2022–4.

## O10 Evidence of sex dimorphism within the hla region in a cohort of JIA patients

### M. Tordoff^1^, S. L. Smith^1^, A. P. Morris^1,2^, S. Eyre^1,2^, J. Bowes^1,2^

#### ^1^Centre for Genetics and Genomics Versus Arthritis, The University of Manchester; ^2^Manchester University NHS Foundation Trust, National Institute of Health Research Manchester Biomedical Research Centre, Manchester, United Kingdom

##### **Correspondence:** M. Tordoff

*Pediatric Rheumatology 2023*, **21(Suppl 2):**O10

**Introduction:** Juvenile idiopathic arthritis (JIA) encompasses a group of heterogenous diseases characterised by joint pain and swelling where symptom onset is before the age of 16. The disease occurs unequally in female and male patients with a ratio of 2:1 but incidence differs between JIA subtypes. The *HLA* region is reported to have a role within the immune response and has been associated with several autoimmune conditions, including JIA.

**Objectives:** To investigate the role of variants within the *HLA* region for JIA onset in females and males, using sex dimorphism analysis.

**Methods:** Genotyping data was available on 2052 females and 961 males with JIA, excluding systemic JIA patients, and 9196 controls (female = 5137, males = 4059). Amino acids, alleles and SNPs within the *HLA* region were imputed using SNP2HLA with a total of 7773 *HLA* markers available for analysis. The *HLA* sex dimorphism analysis combined sex-specific GWAS summary statistics using the GWAMA software package. Sex-specific summary statistics for this analysis were calculated using a logistic regression with three principal components as covariates within PLINK. This analysis provided a sex-heterogeneity p-value (p_het_), which provides evidence to support the heterogeneity between effect estimates of females and males.

**Results:** In total, 139 variants within the *HLA* region passed the threshold (5x10^-8^) for significant sex dimorphism. A large proportion of markers that were significant for sex dimorphism were located within *HLA-B*, including *HLA-B27* (p_het_ = 9.7x10^-15^), which is a well-established risk locus for enthesitis related arthritis (ERA). ERA is reported to occur more frequently in male patients and effect sizes at *HLA-B27* suggest a male specific effect in this cohort (OR_female_ = 1.6, OR_male_ = 4.1). Amino acids within the YST motif of the *HLA-DRB1* binding groove were also significantly sex dimorphic. Tyrosine at position 10 (p_het_ = 4.7x10^-19^, OR_female_ = 1.9, OR_male_ = 1.2), serine at position 11 (p_het_ = 2.3x10^-11^, OR_female_ = 1.9, OR_male_ = 1.2) and threonine at position 12 (p_het_ = 2.3x10^-11^, OR_female_ = 1.9, OR_male_ = 1.2), make up the YST motif of *DRB1.* The effect sizes of the YST motif markers suggest that these markers contribute to JIA onset only in females. *HLA-DRB1* at position 11 has been previously associated with JIA onset in a cohort of oligoarthritis and rheumatoid factor negative polyarthritis; these subtypes are more common in females.

**Conclusion:** In conclusion, this is the first fine-mapping of the *HLA* region in a sex dimorphism analysis for JIA susceptibility. This research has provided evidence of differing genetic risk factors for female and male JIA onset that align with clinical observations. However, further research is now required to understand the mechanism behind these findings. Defining the genetic architecture to JIA will aid disease classification and diagnosis of patients in the future.

**Patient Consent**

Not applicable (there are no patient data)

**Disclosure of Interest**

None declared

## O11 P2RX7 gene variants associate with altered inflammasome assembly and reduced pyroptosis in Chronic Nonbacterial Osteomyelitis (CNO)

### A. Charras^1^, S. R. Hofmann^2^, A. J. Cox^3^, F. Schulze^2^, S. Russ^2^, S. Northey^1^, X. Liu^4^, Y. Fang^4^, S. Haldenby^4^, H. Hartmann^5^, A. G. Bassuk^6^, A. Carvalho^1^, F. Sposito^1^, L. Grinstein^7^, A. Rösen-Wolff^2^, A. Meyer-Bahlburg^8^, M. W. Beresford^1,9^, E. Lainka^10^ on behalf of the German Autoinflammatory Disease Network (AID Net), D. Foell^11^ on behalf of the German Autoinflammatory Disease Network (AID Net), H. Wittkowski^10^ on behalf of the German Autoinflammatory Disease Network (AID Net), H. Girschick^11^ on behalf of the German Autoinflammatory Disease Network (AID Net), H. Morbach^12^, S. Uebe^13^, U. Hüffmeier^13^, P. J. Ferguson^6^, C. M. Hedrich ^1,9^

#### ^1^Department of Women’s and Children’s Health, Institute of Life Course and Medical Sciences, University of Liverpool, Liverpool, United Kingdom; ^2^Department of Paediatrics, Medical Faculty Carl Gustav Carus, Technische Universität Dresden, Dresden, Germany; ^3^Stead Family Department of Pediatrics, University of Iowa Carver College of Medicine, Iowa; ^4^Centre of Genome Research, Institute of Infection, Veterinary & Ecological Sciences, University of Liverpool, Liverpool, United Kingdom; ^5^Light Microscopy Facility, Centre for Regenerative Therapies, Technische Universität Dresden, Dresden, Germany; ^6^Stead Family Department of Pediatrics, University of Iowa Carver College of Medicine, Iowa, United States; ^7^Department of Pediatrics, University Medical Centre Hamburg-Eppendorf, Hamburg; ^8^Pediatric Rheumatology and Immunology, Department of Pediatrics, University Medicine Greifswald, Greifswald, Germany; ^9^Department of Rheumatology, Alder Hey Children’s NHS Foundation Trust, Liverpool, United Kingdom; ^10^Department of Paediatrics II, University Hospital Essen, University of Duisburg-Essen, Essen; ^11^Department for Pediatric Rheumatology & Immunology, University Hospital Münster, Münster; ^12^Department of Pediatrics, Vivantes Hospital Friedrichshain, Berlin; ^13^Institute of Human Genetics, University Hospital Erlangen, Friedrich-Alexander-Universität Erlangen-Nürnberg, Erlangen, Germany

##### **Correspondence:** A. Charras

*Pediatric Rheumatology 2023*, **21(Suppl 2):**O11

**Introduction:** Chronic nonbacterial osteomyelitis (CNO) is an autoinflammatory bone disease primarily affecting children and adolescents. It can cause pain, hyperostosis and fractures, affecting quality-of-life and psychomotor development. The exact pathophysiology remains unknown, and no disease-specific biomarkers exist.

**Objectives:** This study aimed to investigate CNO-associated variants in *P2RX7*, encoding the ATP-dependent trans-membrane K^+^ channel *P2RX7*, and their effects on NLRP3 inflammasome assembly, to explore potential for patient stratification and individualized care.

**Methods:** Whole exome sequencing in two CNO patients from the same family (mother and daughter), and target sequencing of *P2RX7* in a large CNO cohort (N=190) were conducted. Results were compared with publicly available datasets and regional controls (N=1873). Findings were integrated with demographic and clinical data. Patient-derived monocytes and genetically modified THP-1 cells were used to investigate potassium flux, inflammasome assembly, pyroptosis, and cytokine release.

**Results:** Rare damaging mutations in *P2RX7* were identified in two related CNO patients. Targeted *P2RX7* sequencing identified 11 additional CNO patients with rare damaging variants. Across the CNO cohort, rare variants unique to one (Median: 42 versus 3.7) or more (up to 11 CNO patients) participants were over-represented when compared to 190 randomly selected healthy controls. Patients with rare damaging variants were younger and more frequently required treatment with 2^nd^-line agents (DMARDs and/or bisphosphonates). Monocyte-derived macrophages from patients, and genetically modified THP-1-derived macrophages expressing variant P2X7 exhibited altered potassium flux, inflammasome assembly, IL-1β and IL-18 release, and pyroptosis.

**Conclusion:** Rare damaging *P2RX7* variants occur in a small subset of the here investigated CNO patients (5·7%). The genetically variable *P2RX7* gene may represent a CNO risk allele. Observations argue for inhibition of inflammasome activation and/or cytokine blocking strategies and may allow future patient stratification and individualized care.

**Patient Consent**

Yes, I received consent

**Disclosure of Interest**

None declared

## O12 Insights from a novel monogenic autoimmune disease: overview of a multicentric European cohort of 27 patients with copa syndrome

### C. David-Gabarre^1^, B. Bader-Meunier^2^, A. Belot^3^, G. Labouret^4^, M. Brennan^5^, E. Al-abadi^6^, P. Arkwright^7^, W. Newman^7^, M. Gattorno^8^, S. Volpi^8^, A. Tommasini^9^, R. Manna^8^, A. Taddio^9^, B. Lopez Montesinos^10^, T. Clavaguera Poch^11^, M. Gispert-Saüch^11^, A. Mensa^12^, S. El Khalifi-Boulisfane^13^, C. Thumerelle^14^, J. Cadranel^15^, F. Maurier^16^, M. Wislez^17^, Y. Crow^1^, N. Nathan^18^, M.-L. Frémond^1,19^

#### ^1^Laboratory of Neurogenetics and Neuroinflammation, Imagine Institute; ^2^Department of Paediatric Hematology-Immunology and Rheumatology, Necker-Enfants Malades Hospital, AP-HP, Paris, France, Reference center for Rheumatic, AutoImmune and Systemic diseases in children (RAISE); Paris cité University, Paris; ^3^National Reference Center for Rheumatic, Autoimmune and Systemic Diseases in Children (RAISE), Pediatric Nephrology, Rheumatology, Dermatology Unit, Hospital of Mother and Child, Hospices Civils of Lyon, Lyon; ^4^Paediatric Pulmonology Department, University Hospital for Children, Toulouse, France; ^5^Paediatric & Adolescent Rheumatology, Royal Hospital for Children & Young People, Edinburgh; ^6^Childhood Arthritis and Rheumatic Diseases Unit, Birmingham Women’s and Children’s Hospital NHSFT, Birmingham; ^7^Department of Paediatric Allergy & Immunology, Royal Manchester Children’s Hospital, Manchester, United Kingdom; ^8^Center of autoinflammatory diseases and immunodeficiencies, Department of Pediatrics and Rheumatology, IRCCS Istituto G. Gaslini, Genova; ^9^Institute of Child and Maternal Health - IRCCS "Burlo Garofolo" University of Trieste, Trieste, Italy; ^10^Unidad de Reumatologia Pediatrica, HUIP la Fe, Valencia; ^11^UF Pediatric Rheumatology Department. Hospital Doctor Trueta, Girona; ^12^Department of Immunology-CDB, Hospital Clínic-IDIBAPS, Barcelona, Spain; ^13^Emergency, Infectious Disease and Pediatric Rheumatology Department, Centre Hospitalier Régional Universitaire Lille, University of Lille; ^14^Paediatric Pulmonology and Allergy Department, Hôpital Jeanne de Flandre, CHU Lille, Univ. Lille, Lille; ^15^Department of Pneumology and Thoracic Oncology, Tenon Hospital, Assistance Publique-Hôpitaux de Paris (AP-HP) Sorbonne Université, Paris; ^16^Internal Medicine Unit, Hôpitaux Privés de Metz Site Belle Isle,, Metz; ^17^Pulmonology department, Cochin hospital, Assistance Publique - Hopitaux de Paris; ^18^Paediatric Pulmonology Department and Reference Centre for Rare Lung Diseases RespiRare, INSERM UMR_S933 Laboratory of Childhood Genetic Diseases, Armand Trousseau Hospital, Sorbonne University and APHP; ^19^Department of Paediatric Hematology-Immunology and Rheumatology, Necker-Enfants Malades Hospital, AP-HP, Paris, France, Reference center for Rheumatic, AutoImmune and Systemic diseases in children (RAISE); Paris cité University, Paris, France

##### **Correspondence:** M.-L. Frémond

*Pediatric Rheumatology 2023*, **21(Suppl 2):**O12

**Introduction:** COPA syndrome is a recently described monogenic autoimmune disease due to heterozygous mutations in *COPA*. COPA syndrome demonstrates considerable phenotypic overlap with SAVI (STING-associated vasculopathy with onset in infancy) due to gain-of-function mutations in STING.

**Objectives:** Our aim was to gather a European cohort of COPA patients to better delineate the clinical phenotype of this rare monogenic disorder

**Methods:** Assessment of clinical, radiological, immunological and therapeutic data from 27 patients (13 families) with molecularly confirmed COPA syndrome.

**Results:** Twenty-seven individuals with pathogenic COPA mutations were included. Among them, 20 patients presented with at least one clinical manifestation evocative of COPA syndrome (clinical penetrance of 74.1%). Symptomatic patients were female in 13 (65%) cases with a median age at disease onset of 4 years (0-50). All COPA mutations were inherited in an autosomal dominant pattern except for one that occurred *de novo*. Pulmonary involvement was observed in 16 (80%) patients, with interstitial lung disease (ILD) in most cases (n=13, 65%), diffuse alveolar haemorrhage (DAH) in 5 (25%) individuals and the association of ILD and DAH in 3 (15%) patients. Twelve (60%) patients demonstrated joint involvement of variable severity: 4 (20%) individuals experiencing deforming arthritis including one requiring bilateral knee arthroplasty, 6 (30%) patients had polyarticular arthritis and two (10%) patients presented with isolated arthralgias. Renal disease was observed in three (15%) individuals, manifesting as either proliferative glomerulonephritis (n=2) or membranous glomerulonephritis (n=1). Previously undescribed features were noted i.e. cutaneous involvement - acral ulcers, vitiligo and nasal perforation (n=3, 15%), cardiac disease (n=2, 10%), gastrointestinal dysfunction (n=2, 10%), and cytolytic hepatitis (n=1). When tested, 14 (93.9%) patients had positive autoantibodies. When assessed, immunophenotyping showed a mild T-cell lymphopenia, with an excess of naive T CD8+ cells and a defect of memory T CD8+ cells. All patients explored exhibited elevated IFN alpha protein levels and high IFN signature scores. The IFN signature was mildly positive in half of the clinically asymptomatic individuals assessed. The majority (60%) of patients were treated with corticosteroids and immunosuppressants, ten (50%) received biotherapies and eight (40%) patients are currently under JAK1/2 inhibition.

**Conclusion:** We report the first European cohort of COPA patients. While confirming the core organ features (lung, joint and kidney) of COPA syndrome, our data expand the phenotype to include cardiac, skin and digestive features, further demonstrating the clinical overlap with SAVI and other type I interferonopathies.In view of current (JAK inhibitors) and potential future targeted therapies, we suggest a requirement to assess IFN pathway status and/or perform sequencing in the case of suggestive features, even in the absence of a familial history.

**Patient Consent**

Yes, I received consent

**Disclosure of Interest**

None declared

## O13 Whole exome sequencing enables a molecular diagnosis in >10% of early onset or familial SLE

### M. Tusseau^1^, Q. Riller^2^, H. Reumaux^3^, E. Hachulla^4^, B. Bader-Meunier^5^, F. Rieux-Laucat^2^, A. Belot^6^

#### ^1^Laboratoire de génétique, Hospices civils de Lyon, Lyon; ^2^Immunogénétique des maladies auto-immunes pédiatriques, Institut Imagine, Université Paris Cité, Paris; ^3^Service de Rhumatologie pédiatrique, Hôpital Jeanne de Flandre; ^4^Service de médecine interne, Hôpital Claude-Huriez, Lille; ^5^Service d’Immuno-Hématologie et Rhumatologie pédiatriques, Hôpital Necker Enfants Malades, Paris; ^6^Service de néphrologie-rhumatologie-dermatologie pédiatriques, Hospices civils de Lyon, Lyon, France

##### **Correspondence:** M. Tusseau

*Pediatric Rheumatology 2023*, **21(Suppl 2):**O13

**Introduction:** Systemic lupus erythematosus is an autoimmune disease characterized by the production of antinuclear antibodies and an increase in type I interferons. The exact cause of the disease is unknown, but genetic and environmental factors are thought to play a role. Over the past decade, we have been able to explore the Mendelian contribution to juvenile-onset SLE (jSLE) and show that 7% of jSLE is monogenic using a panel approach^1,2^.

**Objectives:** The aim of this study was to explore a cohort of juvenile or familial lupus with a pangenomic approach to assess the diversity of genes involved in lupus and to evaluate the diagnostic rate of exome sequencing.

**Methods:** We selected patients from the National Lupus Biobank who met at least one of the following criteria: (1) male sex, (2) disease onset < 12 years, (3) family history of autoimmune disease, and performed whole exome sequencing in 118 families. In a diagnostic approach, we used in silico panels and then explored the dataset to identify novel genes involved in lupus.

**Results:** We identified pathogenic or probable pathogenic variations according to the American College of Medical Genetics classification in genes associated with inborn errors of immunity in 7 patients (ADAR, C1QA, PSTPIP1, IRAK4, PTPN11, COPA, IKZF3). A genetic diagnosis involving a gene never associated with lupus (MAN1B1, ETV6) was identified in 2 patients, explaining part of the phenotype but not the lupus. In addition, a research approach revealed numerous candidate genes, including SOCS1, PTPN2, and DOCK11, which were confirmed as responsible for the disease by collaborative and functional studies^3,4^.

**Conclusion:** This study confirms the value of exome sequencing in pre-selected lupus patients with a diagnosis rate of more than 10% of monogenic SLE. It demonstrates the superiority of exome over panel in lupus, with genetic diagnosis of unexpected genes and the possibility of discovery-based approaches. Finally, the striking element is the high proportion of novel genes involved, demonstrating the dynamics of genetic discovery in this field.

**Patient Consent**

Yes, I received consent

**Disclosure of Interest**

None declared

**References**

1. Belot, A. *et al.* Contribution of rare and predicted pathogenic gene variants to childhood-onset lupus: a large, genetic panel analysis of British and French cohorts. *The Lancet Rheumatology* **2**, e99–e109 (2020).
2. Omarjee, O. *et al.* Monogenic lupus: Dissecting heterogeneity. *Autoimmunity Reviews* **18**, 102361 (2019).
3. Hadjadj, J. *et al.* Early-onset autoimmunity associated with SOCS1 haploinsufficiency. *Nat Commun* **11**, 5341 (2020).
4. Boussard, C. *et al.* DOCK11 deficiency in patients with X-linked actinopathy and autoimmunity. *Blood* blood.2022018486 (2023) doi:10.1182/blood.2022018486.

## O14 Patients with SLE have unique changes in serum metabolomic profiles across age associated with cardiometabolic risk

### A. Van Vijfeijken, J. Peng, L. Martin Gutierrez, C. Ciurtin, E. C. Jury, G. A. Robinson

#### Rheumatology, University College London, London, United Kingdom

##### **Correspondence:** G. A. Robinson

*Pediatric Rheumatology 2023*, **21(Suppl 2):**O14

**Introduction:** Cardiovascular disease is a leading cause of mortality for patients with systemic lupus erythematosus (SLE) through accelerated atherosclerosis. This is likely due to chronic inflammation and cardiometabolic defects that exacerbate with age. Mechanisms of atherosclerosis begin from an early age, particularly in young patients with juvenile-onset SLE, highlighting the importance of studying cardiometabolic risk over age in SLE.

**Objectives:** This study investigated detailed age-associated changes in the circulating metabolomic profiles of SLE patients and healthy controls (HCs).

**Methods:** Serum NMR metabolomics (>250 metabolites) of female SLE patients (n=164, age=13-72, mean age=37) and matched HCs (n=123, age=15-76, mean age=37) was assessed by linear regression and Venn analysis. Multiple t-tests (FDR-corrected) and MetaboAnalyst assessed unique metabolic changes and pathways by age group between patients/HCs (≤25, n=62/46; 26-49, n=50/46; ≥50, n=52/31). The impact of inflammation, SLE disease activity, and treatments on metabolites were also investigated. Disease-wide association analysis of metabolites of interest was performed using the Nightingale Atlas web-tool (data from the UK Biobank cohort).

**Results:** Twenty-five metabolites were significantly altered in all SLE age groups vs HCs, dominated by decreased atheroprotective high-density lipoprotein (HDL) subsets and HDL-associated apolipoprotein(Apo)A1 (p<0.0001). Importantly, ApoA1 correlated negatively with disease activity measures (SLEDAI, p=0.005; BILAG, p=0.0009; dsDNA, p=0.003). Strikingly, the metabolite signature was significantly associated with both atherosclerosis incidence and myocardial infarction (MI) mortality through disease-wide association analysis. Altered metabolites unique to different age groups in SLE vs HCs included reduced amino acids (≤25), increased very-low-density lipoproteins (26-49), and increased low-density lipoproteins (≥50). Separately, metabolites in the glycolysis pathway (p=0.004), including acetone, citrate, creatinine, glycerol, lactate, and pyruvate, had positive correlations with age in SLE patients, but not in HCs. Pyruvate (p=0.01) and lactate (p=0.009) were upregulated in prednisolone-treated patients, whilst citrate (p=0.002) and creatinine (p= 0.005) were downregulated in hydroxychloroquine-treated patients. Importantly, all of these SLE age-associated glycolysis metabolites had a significant disease-wide association with both type 1 and type 2 diabetes.

**Conclusion:** Increasing HDL (ApoA1) levels through therapeutic/nutritional intervention, whilst maintaining low disease activity, in SLE patients from a young age could improve disease and cardiometabolic outcomes. Biomarkers from the glycolytic pathway could decrease the adverse metabolic effects of current therapies.

**Patient Consent**

Yes, I received consent

**Disclosure of Interest**

None declared

## O15 Speaking the same language: international cross-validation of emerging biomarkers for Juvenile idiopathic arthritis

### C. Kessel^1^, R. Marsh^2^, C. Wouters^3^, P. Matthys^4^, P. Proost^4^, D. Foell^1^, F. Minoia^5^, S. Canna^6^, G. Prencipe^7^, C. Bracaglia^8^, F. De Benedetti^8^, D. Dissanayake^9^, R. Laxer^9^, S. Vastert^10^, K. Brown^11^, D. A. Cabral^12^, G. Schulert^13^ on behalf of PReS sJIA/MAS working party

#### ^1^Pediatric Rheumatology & Immunology, University Hospital Muenster, Muenster, Germany; ^2^Divison of Bone Marrow Transplantation and Immune Deficiency, Cincinnati Children's Hospital, Cincinnati, United States; ^3^Department of Pediatrics, University Hospitals Leuven; ^4^Microbiology, Immunology and Transplantation, Rega Institute KU Leuven, Leuven, Belgium; ^5^Pediatric Immuno-Rheumatology Unit, Fondazione IRCSS Ca' Granda Ospedale Maggiore Policlinico, Milan, Italy; ^6^Division of Rheumatology, Department of Pediatrics, Children's Hospital of Philadelphia and University of Philadelphia Perelman School of Medicine, Philadelphia, United States; ^7^Laboratory of Immuno-Rheumatology; ^8^Division of Rheumatology, Bambino Gesù Children's Hospital, Rome, Italy; ^9^Department of Pediatrics, Hospital for Sick Children, Toronto, Canada; ^10^Pediatric Rheumatology and Immunology, University Medical Center Utrecht, Utrecht, Netherlands; ^11^Department of Pediatrics, British Columbia Children's Hospital Research Institute; ^12^Division of Pediatric Rheumatology, BC Children's Hospital, University of British Columbia, Vancouver, Canada; ^13^Department of Pediatrics, Division of Rheumatology, Cincinnati Children's Hospital, Cincinnati, United States

##### **Correspondence:** C. Kessel

*Pediatric Rheumatology 2023*, **21(Suppl 2):**O15

**Introduction:** To date, several studies have validated the use of key biomarkers such as IL-18, CXCL9 and S100 proteins in diagnosis and monitoring of treatment response of systemic juvenile idiopathic arthritis (sJIA). Despite the promise of these biomarkers, their clinical utility is still limited by their overall lack of standardization.

**Objectives:** In this project we set out to cross-validate emerging systemic JIA biomarkers across different measurement platforms and different international centers to facilitate their wider introduction into routine clinical care.

**Methods:** In a first step healthy donor serum samples spiked with defined concentrations of recombinant S100 proteins, CXCL9, CXCL10, IL-18 and sCD25 were distributed in blinded manner among all participating centers (CARRA member sites: Cincinnati, Philadelphia, Toronto, Vancouver; PReS centers: Leuven, Muenster, Rome, Utrecht). Individual spiked protein levels were determined using locally established platforms including commercial ELISA, commercial/custom luminex, Ella and Mesoscale. In a second step patients’ samples enrolled in the FROST study will be distributed for respective biomarker analyses. All data will be analyzed for variances across different platforms and agreement across identical platforms in different labs.

**Results:** We observe extremely tight correlation of spiked IL-18 and CXCL9 levels with the amounts quantified by the employed measurement platforms (IL-18 R^2^=0.744-0.999, *P*<0.0001; CXCL9 R^2^=0.924-0.999, *P*<0.0001). However, the actual spike recovery of the individual assays varied substantially. While some assays met 90-100% spike recovery over almost the entire tested concentration range (1pg-500ng/mL), others consistently yielded high (approx. 500%) or low (approx. 60-70%) spike recovery. Further, our data determined the lower level of detection for each assay to provide consistent performance. At present, this analysis is extended to other spiked parameters and measurements in patients’ samples are in preparation.

**Conclusion:** Our spike recovery approach demonstrates - as expected - high correlation of individual assay results but widely divergent absolute concentrations measured. From our data we can now clearly identify assays with almost perfect spike recovery and calculate conversion factors for those that over- or underperform in their concentration output. This may allow for correction factors for IL-18 and CXCL9 levels (and others) quantified in future studies using the tested assay systems. We will further expand to utilize patient samples from the FROST study to validate the utility of correction factors. Altogether, the results from our study will enable wide interpretation and translation of respective biomarker data and pave the way towards their wider use in routine clinical practice and international collaborative studies.

**Patient Consent**

Not applicable (there are no patient data)

**Disclosure of Interest**

C. Kessel Grant / Research Support with: Novartis, Consultant with: Novartis and SOBI, R. Marsh: None declared, C. Wouters: None declared, P. Matthys: None declared, P. Proost: None declared, D. Foell Grant / Research Support with: Novartis, Pfizer and SOBI, Consultant with: Chugai-Roche, Novartis and SOBI, F. Minoia Consultant with: Novartis and SOBI, S. Canna Consultant with: AB2Bio and Novartis, G. Prencipe: None declared, C. Bracaglia Consultant with: Novartis and SOBI, F. De Benedetti Grant / Research Support with: AbbVie, Sobi, Pfizer, Roche, Sanofi, Novartis, Novimmune, Consultant with: AbbVie, Sobi, Pfizer, Roche, Sanofi, Novartis, Novimmune, D. Dissanayake: None declared, R. Laxer: None declared, S. Vastert Grant / Research Support with: Novartis and SOBI, Consultant with: Novartis and SOBI, K. Brown: None declared, D. Cabral: None declared, G. Schulert Grant / Research Support with: IpiNovyx, Consultant with: SOBI

## O16 In a multi-mediator inflammatory environment Il-1 signaling acts as paramount driver of human coronary artery endothelial activation and endothelial-to-mesenchymal transition

### P. Buthe^1^, M. C. Limburg^1^, S. Fuehner^1^, J. K. Kuehn^1^, A. Jakob^2^, I. Koné-Paut^3^, S. Tellier^4^, A. Belot^5^, L. Rossi-Semerano^3^, P. Dusser^3^, I. Marie^3^, K. Masjosthusmann^6^, J. Merfort^6^, C. Hinze^1^, H. Wittkowski^1^, D. Foell^1^, C. Kessel^1^

#### ^1^Pediatric Rheumatology & Immunology, University Hospital Muenster, Muenster; ^2^Pediatric Cardiology and Pediatric Intensive Care, Ludwig-Maximilians-University, Munich, Germany; ^3^Pediatric Rheumatology and CEREMAIA, University of Paris Saclay, Le Kremlin-Bicêtre, France; ^4^Department of Pediatrics, Divisions of Nephrology, Rheumatology and Internal Medicine, University of Toulouse, Toulouse, Germany; ^5^Departments of Pediatrics, Division of Rheumatology, Dermatology and Nephrology, University of Lyon, Lyon, France; ^6^General Pediatrics, University Hospital Muenster, Muenster, Germany

##### **Correspondence:** P. Buthe

*Pediatric Rheumatology 2023*, **21(Suppl 2):**O16

**Introduction:** Kawasaki disease (KD) is an acute systemic vasculitis of unknown etiology that affects small- and medium-sized arteries of infants and children. Using biosamples from a phase II open-label study of the interleukin 1 (IL-1) receptor antagonist (IL-1Ra) anakinra in treating IVIG-resistant Kawasaki Disease (KD) patients, we recently identified leucin-rich-α2-glycoprotein-1 (LRG-1) as known trigger of endothelial activation and cardiac re-modelling to associate with IL-1β signaling in KD.

**Objectives:** In the present study we aimed to assess a potential role of LRG-1 in activation of human coronary artery endothelium in context of a complex inflammatory environment as in KD.

**Methods:** To mimic a multi-mediator inflammatory interplay, primary human coronary artery endothelial cells (HCAECs) were treated with patients’ (KD (n=8), sJIA (n=4), MIS-C (n=3)) serum conditioned medium or an inflammatory matrix (IM) from stimulated healthy control whole blood, with or without IL-1R1 (anakinra), IL-6R (tocilizumab), TNFa (adalimumab) or LRG-1 (magacizumab) neutralizing drugs or IVIGs and were analyzed for inflammatory activation or endothelial-mesenchymal transition (EndoMT) on gene expression level. IM samples (n=8), treatment naïve KD (n=10) or HC sera (n=10) were subjected to proximity extension proteomic analysis for cardiovascular and/or inflammatory markers (n=184).

**Results:** Proteomic analysis of KD sera (n=10) and IM (n=8) revealed elevation of 32 versus 45 inflammatory proteins, respectively, and shared 19 significantly upregulated markers. HCAEC culture with IM or patient sera resulted in inflammatory endothelial activation, which differed between KD, MIS-C and sJIA. Upon exposure to IM this was most efficiently abrogated by IL-1R1 inhibition, while particularly IL-6R and LRG-1 targeting as well as IVIG-treatment revealed no effect. However, inflammatory endothelial activation is closely linked to endothelial-to-mesenchymal transition (EndoMT), which is supported by respective signatures in our proteomic analysis of cardiovascular activation in KD sera (n=10). Among others, EndoMT can be mediated by TGFβ1-signaling. Yet, inhibition of LRG-1 as a modulator of TGFβ1-signaling did not impact EndoMT of HCAECs. Instead, particularly on the level of transition as well as mesenchymal markers, EndoMT was most consistently abrogated by IL-1R1 inhibition compared to other drugs or combinations of those.

**Conclusion:** While targeted LRG-1 inhibition had no effect on inflammatory HCAEC activation or EndoMT, both processes were profoundly impacted by anakinra treatment. These observations highlight a superior role of IL-1 signaling in EndoMT, particularly in context of a high-dimensional inflammatory environment, and complement our understanding of the cytokine’s prominent role in context of cardiovascular inflammation, arteriosclerosis, and myocardial fibrosis.

**Patient Consent**

Yes, I received consent

**Disclosure of Interest**

None declared

## O17 Immunological indicators of poor outcome in oligoarticular Juvenile idiopathic arthritis

### F. Raggi^1^, S. Pelassa^1^, C. Rossi^1^, F. Antonini^2^, C. Trincianti^3^, G. Filocamo^4^, A. Civino^5^, M. Gattorno^1^, A. Ravelli^6^, A. Consolaro^7^, M. C. Bosco^1^

#### ^1^Unit of Autoinflammatory Diseases and Immunodeficiencies, Pediatric Rheumatology Clinic; ^2^Core Facilities, Department of Research and Diagnostics, IRCCS Istituto Giannina Gaslini; ^3^Department of Neurosciences, Rehabilitation, Ophthalmology, Genetics and Maternal-Infantile Sciences (DiNOGMI), University of Genova, Genova; ^4^SC Pediatric Immunorheumatology, Fondazione IRCCS Ca' Granda Ospedale Maggiore Policlinico, Milano; ^5^Pediatric Rheumatology and Immunology, Ospedale Vito Fazzi, Lecce; ^6^Scientific Direction; ^7^Pediatric Rheumatology Clinic, IRCCS Istituto Giannina Gaslini, Genova, Italy

##### **Correspondence:** F. Raggi

*Pediatric Rheumatology 2023*, **21(Suppl 2):**O17

**Introduction:** In Oligoarticular Juvenile Idiopathic Arthritis (OJIA), the mechanisms underlying the inflammatory processes within the joints and leading to polyarticular extension are poorly understood. However, the pathogenetic role of immune cells infiltrating the synovial environment is well known, and the involvement of extracellular vesicles (EVs) released by immune cells is being increasingly recognized. Many studies have focused on the effects of EVs released in synovial fluid (SF) of patients affected by adult rheumatic arthritis on disease progression. Immunologically active EVs are mainly released by macrophages (Mf) and T cells, participating in antigen presentation and immune regulation. A better understanding of the immunophenotypic profile of circulating and in situ infiltrated inflammatory cells in OJIA patients and of the surface protein cargo of released EVs may help earlier disease diagnosis and new therapeutic approaches.

**Objectives:** This study aimed at identifying specific immune cell subsets from samples of OJIA patients at disease onset, their activation markers, and expression of specific EV surface molecules, which could be used as indicators to predict polyarticular extension.

**Methods:** A total of 50 treatment-naïve OJIA patients was enrolled at the onset and followed-up for two years. Plasma (PL) and SF samples from 10 patients who presented an oligoarticular course (oOJIA) and 10 who developed a polyarticular course (pOJIA) were considered for the analysis. Mf and T cell subsets from PBMCs and SF mononuclear cells (SFMCs) were characterized by cytofluorimetry. Characterization of surface molecules of EVs isolated from SF and PL was carried out.

**Results:** SF-derived Mf in active joints of patients who developed pOJIA exhibit polarization toward the M1-like phenotype, as shown by predominance of the CD80 and the coexpressing CD80+/CD206+ or CD80+/CD163+ subsets respect to oOJIA patients associated with higher expression of the immunoregulatory receptor TREM1. No differences were observed in the M2 subsets between the groups of patients presenting the oOJIA and pOJIA course. PBMCs displayed the same percentage of M1 and M2, but higher presence of the CD206^+^/CD163^+^ subset in patients who developed pOJIA. A different state of T cell activation (HLADR^+^) in both PBMCs and SFMCs and a different ratio of Treg in the SMFCs were also observed in oOJIA and pOJIA patients providing discrimination between outcome groups, whereas no differences were detectable in the percentages of naive, central memory, effector memory, and terminally differentiated effector memory CD4^+^ and CD8^+^ subsets derived from SFMCs and PBMCs. Interestingly, analysis on EV surface cargo showed higher expression in pOJIA of the glycoproteins CD9, CD44, and CD11c whose relevance was demonstrated in adult arthritis where their targeting is proposed as drug delivery strategy in disease treatment.

**Conclusion:** These data suggest that the number of CD80+/TREM-1+ Mf and the Treg subset in the SF of OJIA patients at disease onset might be helpful for early prediction of persistent/extended-to-be patients. Similarly, the expression levels of specific surface markers on SF-derived EVs might have a potential predictive value. These data provide novel mechanistic insights into OJIA pathophysiology and an important contribution in the search of new indicators for improving disease diagnostic accuracy.

**Patient Consent**

Not applicable (there are no patient data)

**Disclosure of Interest**

None declared

## O18 Population pharmacokinetic/pharmacodynamic analysis of emapalumab in patients with macrophage activation syndrome associated with systemic Juvenile idiopathic arthritis

### P. Brossard^1^, A. Facius^2^

#### ^1^Swedish Orphan Biovitrum, Basel; ^2^thinkQ2 AG, Baar, Switzerland

##### **Correspondence:** P. Brossard

*Pediatric Rheumatology 2023*, **21(Suppl 2):**O18

**Introduction:** Macrophage activation syndrome (MAS) is a rare, life-threatening complication of rheumatic diseases that occurs most frequently in patients with Still’s disease (systemic juvenile idiopathic arthritis [sJIA] and adult-onset Still’s disease [AOSD]). MAS is characterized by overproduction of interferon γ (IFNγ) and other cytokines. Emapalumab, a fully human anti-IFNγ monoclonal antibody, is being investigated as a treatment for patients with MAS in rheumatic diseases.

**Objectives:** To develop a population pharmacokinetic (PK)/pharmacodynamic (PD) model to describe the PD effect of emapalumab in patients with MAS associated with sJIA/AOSD.

**Methods:** A PK model was developed using pooled data from 3 studies (n=58; 2709 samples): (i) an open-label, single-arm, phase 2/3 clinical trial of 45 patients who received emapalumab for primary haemophagocytic lymphohistiocytosis; (ii) a pilot, open-label, single-arm, phase 2 study of 14 patients who received emapalumab for MAS in sJIA; and a 1-year, long-term, follow-up study of patients from both studies. PK analysis was performed using nonlinear mixed effects modelling (NONMEM® version 7.5). Three linked PK/PD models were then developed on data from the 14 patients in study (ii) to characterize the relationship between emapalumab exposure and laboratory parameters associated with MAS, namely C-X-C motif chemokine ligand 9 (CXCL9), soluble interleukin-2 receptor alpha (sIL-2Rα), and ferritin. These PD parameters and their treatment-induced changes were characterized by turnover models. Parameters were assumed to be in steady-state at baseline with exposure-induced inhibition after treatment start. Predictive performance of the model was assessed using goodness-of-fit (GOF) plots and visual predictive checks (VPCs).

**Results:** Emapalumab PK was adequately described by a two-compartment model with first-order elimination that was constant at total IFNγ concentrations <~10^4^ pg/mL. Emapalumab clearance increased proportionally with total IFNγ concentrations. Estimated baseline levels of CXCL9, sIL-2Rα, and ferritin were 8400 ng/L, 6550 ng/L, and 15300 mg/L, respectively. A rapid PD response to changes in emapalumab concentration was observed. Emapalumab almost completely suppressed CXCL9, sIL2-Rα, and ferritin production (estimated reduction in synthesis rate: 98.3%, 87%, and 99.6%, respectively). Standard errors of all parameters obtained by a bootstrap procedure were <40% of their respective bootstrap means indicating good model precision. GOF plots and VPCs indicated good alignment between model predictions and observed concentrations for all parameters.

**Conclusion:** Population PK/PD modelling indicated that emapalumab rapidly suppresses CXCL9, sIL2-Rα, and ferritin production in patients with MAS associated with sJIA/AOSD.

**Trial registration identifying number:** ClinicalTrials.gov identifier: NCT01818492, NCT02069899 and NCT03311854.

**Patient Consent**

Not applicable (there are no patient data)

**Disclosure of Interest**

P. Brossard Employee with: Sobi, A. Facius Consultant with: Sobi

## PT001 Artificial intelligence model 'maverik' for the diagnosis of Chronic Non-bacterial Osteomyelitis (CNO): preliminary results

### E. Aliyev^1,2^, Y. Uğur^3^, Y. Bayindir^1^, H. O. Basaran^1^, S. Ozen^1^

#### ^1^Pediatric Rheumatology, Hacettepe University School of Medicine; ^2^SEMBA Science, Education & Informatics Ltd. Co.; ^3^Keytech Electronic & Software Ltd. Co., Ankara, Türkiye

##### **Correspondence:** E. Aliyev

*Pediatric Rheumatology 2023*, **21(Suppl 2):**PT001

**Introduction:** Chronic Non-bacterial Osteomyelitis (CNO) is an inflammatory bone disease described in the last decade and frequently encountered in rheumatology practice. Since the condition is not well known and often confused with malignancy and growing pains, it can be easily overlooked in practice. Therefore, auxiliary means are needed.

**Objectives:** In this context, our study aims to develop computer-savvy physician-friendly modeling for detecting the disease with the help of Artificial Intelligence (AI) and to test it on real individuals.

**Methods:** Eighty-three patients with CNO, nine patients with growth pain, nine patients with bone tumor, nine patients with juvenile idiopathic arthritis (JIA), and 30 healthy controls (HCs) who were followed up in Hacettepe University Pediatric Rheumatology Outpatient Clinics were included in the study. Data sets, including clinical and laboratory findings of the individuals at the time of diagnosis, were given as input to the AI model. Python® software language and Tensorflow® AI library were preferred for model development. Neural Network (NN) and Recurrent Neural Network (RNN) models were preferred in the development of Maverik. Maverik started with eight neurons and evolved to a 1000-neuron model with 84 inner layers at the end of the training. The laboratory and clinical results of the cases were digitized as 1, -1 (abnormal), and 0 (normal range). The data belonging to a total of 140 cases were increased 20-fold to 2800 with repetitions, 80% of which were randomized by the AI as training data and 20% as validation data. The homogeneity of the variables was tested by analysis of covariance (p=0.881).

**Results:** During the first minutes of model training, the error rate was approximately 0.44. In the following generations of Maverik, the error rate decreased to 0.019. This error rate was considered sufficient, and the training phase was terminated. Training continued autonomously in the latent phase. The training lasted a total of 62 minutes. The study was designed to be single blind. Thirty case data (data completely unfamiliar to the model) were asked to Maverik and the expert clinician, and their interpretations were compared. Regression analyses were performed between both groups to evaluate the diagnostic prediction success. Maverik accepted 70% and above as a definitive diagnosis of CNO, with a mean of 88.1% (71.33-96.3%). This rate was almost identical to the diagnostic acuity of clinicians (88.2% (5-100%)). There was no difference between Maverik's predictions and etalon diagnoses (p=0.812), and CNO was 100% successfully recognized in the disease. However, clinicians correctly recognized 15 of 17 CNO patients (88.3%). Maverik was not as accurate as clinicians in recognizing patients with growth pain, malignancy, and JIA in the mixed data set.

**Conclusion:** Our study is the first investigation in the literature to develop and test an AI model as a CNO diagnostic tool. The developed AI model performed better than the clinician in differentiating the CNO patient from the aforementioned differential diagnoses. This study shows that AI can help clinicians in the diagnostic stage and differential diagnosis. Maverik could not clearly distinguish the differential diagnoses because there were only HCs and CNO case data in the training set. The model will achieve better results with more data sets. In addition to all these, it seems likely that the model can be used as an additional diagnostic tool for clinicians outside the disciplines.

**Patient Consent**

Not applicable (there are no patient data)

**Disclosure of Interest**

None declared

**References**

1. Yazdany J, Bansback N, Clowse M, et al. Rheumatology informatics system for effectiveness: a national informatics-enabled registry for quality improvement. *Arthritis Care Res (Hoboken)* 2016;**68**:1866–73.
2. McMaster, C., Bird, A., Liew, D. F. L., Buchanan, R. R., Owen, C. E., Chapman, W. W., & Pires, D. E. V. (2022). Artificial Intelligence and Deep Learning for Rheumatologists. *Arthritis & rheumatology (Hoboken, N.J.)*, *74*(12), 1893–1905.

## PT002 The impact of different MEFV genotypes on clinical phenotype of patients with familial Mediterranean fever: special emphasis on joint involvement

### E. Aslan, E. K. Konte, A. Gunalp, F. Haslak, M. Yildiz, A. Adrovic, S. Sahin, K. Barut, O. Kasapcopur

#### Pediatric Rheumatology, Istanbul University-Cerrahpasa, Cerrahpasa Medical School, Istanbul, Türkiye

##### **Correspondence:** E. Aslan

*Pediatric Rheumatology 2023*, **21(Suppl 2):**PT002

**Introduction:** Familial Mediterranean fever (FMF) is the most common monogenic autoinflammatory disease worldwide. It is characterized by recurrent episodes of fever and self-limiting polyserositis [1]. Joint involvement and erysipelas like erythema are other major features, which are not rare in the course of disease. [2, 3]. Joint involvement is mainly in the form of non-erosive monoarthritis, which is frequently seen in the large joints of the lower extremities [2]. Moreover, 3-5%% of patients may develop chronic arthritis in the hips or knees [2, 4].

**Objectives:** In our study, we aimed to collect the demographic, clinical, and genetic data of patients with FMF. In the second-step analysis, we investigated the impact of various genotypes on the clinical features of FMF patients, with a special focus on joint involvement patterns.

**Methods:** A total of 782 patients were consecutively enrolled to the study according to their order of admission to the outpatient clinic between September 2022 and May 2023. The clinical, genetic, and laboratory data of 782 patients were obtained from patient files retrospectively and these data were also confirmed by patients/parents at the study visit. The study group was categorized into 2 groups: Group 1 (Patients carrying the 4 pathogenic MEFV variants [M694V, M694I, M680I, V726A] in a homozygous or heterozygous state) and Group 2 (FMF patients with other variants or no mutation).

**Results:** Of the 782 patients (292 in Group 1 and 490 in Group 2), 384 (49.1%) were female and the median age at disease onset and diagnosis were 3.0 (0.5-16) and 5.5 years, respectively. The frequency of colchicine resistance (n=15, 5.1% *vs* n=1, 0.2%; p<0.001), chest pain (n=80, 27.4% *vs* n=84, 17.1%; p=0.001), arthritis (n=93, 31.8% *vs* n=124, 23.3%; p<0.01), erysipelas like erythema (n=20, 6.8% *vs* n=10, 2.0 %; p=0.001), corticosteroid usage (n=10, 3.4% *vs* n=6, 1.2%; p<0.05), synthetic DMARD (n=20, 6.8% *vs* n=16, 3.3%; p<0.05) and biologic agent (n=61, 20.9% *vs* n=30, 6.1%; p<0.001) requirement were significantly higher in Group 1 than Group 2. Both of the patients (n=2) with renal amyloidosis were in Group 1. Patients in Group 1 were found to have a longer duration of arthritis and higher doses of colchicine were required in this Group.

**Conclusion:** FMF Patients with a pathogenic exon 10 mutation either in a homozygous or compound heterozygous state not only have higher rates of serositis, erysipelas like erythema and amyloidosis, but also tend to have more frequent joint involvement with a longer remission period.

**Patient Consent**

Yes, I received consent

**Disclosure of Interest**

None declared

**References**

1. Barut, K., et al., *Familial Mediterranean fever in childhood: a single-center experience.* Rheumatol Int, 2018. **38**(1): p. 67-74.
2. Avar-Aydın, P.O., et al., *The expanded spectrum of arthritis in children with familial Mediterranean fever.* Clin Rheumatol, 2022. **41**(5): p. 1535-1541.
3. Sozeri, B. and O. Kasapcopur, *Biological agents in familial Mediterranean fever focusing on colchicine resistance and amyloidosis.* Curr Med Chem, 2015. **22**(16): p. 1986-91.
4. Özen, S., E.D. Batu, and S. Demir, *Familial Mediterranean Fever: Recent Developments in Pathogenesis and New Recommendations for Management.* Front Immunol, 2017. **8**: p. 253.
5. Yildiz, M., et al., *Evaluation of co-existing diseases in children with familial Mediterranean fever.* Rheumatol Int, 2020. **40**(1): p. 57-64.

## PT003 Clinical characteristics and treatment of 103 cases of cryopyrin-associated periodic syndrome in Japan

### K. Izawa^1^, T. Miyamoto^1^, Y. Honda^1^, E. Hiejima^1^, O. Ohara^2^, J. Takita^1^, T. Yasumi^1^, R. Nishikomori^3^ on behalf of The Japan CAPS working group

#### ^1^Pediatrics, Kyoto University Graduate School of Medicine, Kyoto; ^2^Kazusa DNA Research Institute, Kisarazu; ^3^Pediatrics and Child Health, Kurume University School of Medicine, Kurume, Japan

##### **Correspondence:** K. Izawa

*Pediatric Rheumatology 2023*, **21(Suppl 2):**PT003

**Introduction:** Cryopyrin-associated periodic syndrome (CAPS) comprises a spectrum of autoinflammatory disorders caused by gain-of-function mutations in *NLRP3. Since 2009, genetic testing for autoinflammatory diseases has been provided as part of the Primary Immunodeficiency Database in Japan (PIDJ) project in Japan. Canakinumab was subsequently approved in 2011 for CAPS treatment.*

**Objectives:** To assess the clinical characteristics of patients with cryopyrin-associated periodic syndrome (CAPS) in Japan.

**Methods:** Clinical information was collected retrospectively, and the serum concentrations of canakinumab and were analyzed.

**Results:** A total of 103 patients were included in this analysis; 86 and 15 patients carried heterozygous germline and somatic mosaic mutations, respectively, and two were mutation negative. We identified 39 mutation types, and the common CAPS-associated symptoms corresponded with previous reports. Notably, 74.4% of patients achieved complete remission with canakinumab, and early therapeutic intervention was associated with better auditory outcomes. In some patients, canakinumab treatment improved height gain, visual acuity, and renal function. However, 23.2% of patients did not achieve inflammatory remission with crucial deterioration of organ damage, and two fatal cases were recorded. Serological analysis of canakinumab and cytokine concentrations revealed that the poor response was not related to canakinumab shortage. Four inflammatory non-remitters developed inflammatory bowel disease unclassified (IBD-u) during canakinumab treatment. Dual biologic therapy with canakinumab and anti-TNF-α agents was effective for IBD- and CAPS-associated symptoms not resolved by canakinumab monotherapy.

**Conclusion:** This study provides one of the largest-scale epidemiologic datasets for CAPS. While early initiation of anti-IL-1 treatment is beneficial for improving disease prognosis, some patients do not achieve remission despite high serum concentration of canakinumab. Therefore, dual biological therapy may be an effective approach for such patients.

**Patient Consent**

Yes, I received consent

**Disclosure of Interest**

K. Izawa Consultant with: SOBI, Speaker Bureau with: Novartis, T. Miyamoto: None declared, Y. Honda: None declared, E. Hiejima: None declared, O. Ohara: None declared, J. Takita: None declared, T. Yasumi Speaker Bureau with: Novartis, Abbvie, R. Nishikomori Consultant with: Eli Lilly, Speaker Bureau with: Novartis

## PT004 Hematopoetic Stem Cell Transplantation (HSCT) results in patients with monogenic Autoinflammatory Diseases (AID)

### A. Kozlova, N. Kan, V. Bludova, V. Burlakov, Z. Nesterenko, M. Leontieva, Y. Rodina, D. Balashov, G. Novichkova, A. Shcherbina

#### Immunology, Dmitry Rogachev National Medical Research Center of Pediatric Hematology, Oncology and Immunology, Moscow, Russian Federation

##### **Correspondence:** A. Kozlova

*Pediatric Rheumatology 2023*, **21(Suppl 2):**PT004

**Introduction:** HSCT represents a well-characterized curative treatment option for classic inborn errors of immunity, affecting T and B lymphocytes. Yet, its applicability in monogenic autoinflammatory disorders, resistant to conservative treatment, is less studied, and its efficacy is not always obvious as many AID affect non-hematopoietic lineages as well

**Objectives:** To analyze HSCT results in a group of children with monogenic AID

**Methods:** We report HSCT results in 6 patients (2 males, 4 females), who underwent transplantation at our center to treat the following AIDs: PSTPIP1 defect – 3, MVK deficiency – 2, DNASE2 deficiency - 1. The latter patient underwent simultaneous kidney and hematopoietic stem cell transplantation. HSCT indications included lack of remission while on multiple lines of treatment in 3, myelodysplastic syndrome in 1, hemophagocytic lymphohistiocytosis development in 1, aplastic anemia and end-stage kidney disease in 1

**Results:** The age of disease onset was M 0 (0- 2,9) years, the age of HSCT M 10,1 (1,5- 12) years. The donors for the first HSCT were matched unrelated in 3 patients, haploidentical - in 3. Treosulfan-based conditioning was used in all patients. Graft rejection occurred in 3/6 patients, all of them underwent second HSCT: 2/3 - from matched unrelated, 1/3- from haploidentical donor. 1/3 died from HSCT complications after the second transplantation. Graft versus host disease (GVHD) occurred in 2/9 procedures. Currently 5 patients have 3,2 (2- 5,6) years follow-up, with full donor chimerism, full immune reconstitution and resolution of all AID symptoms

**Conclusion:** Based on our experience HSCT in AID represents challenging, yet potentially effective treatment. Conditioning and GVHD prophylaxis regiments require further investigation in a larger cohorts of patients

**Patient Consent**

Not applicable (there are no patient data)

**Disclosure of Interest**

None declared

## PT005 No cumulative effect of infection rates in children receiving long-term canakinumab treatment in autoinflammatory periodic fever syndromes − data from the reliance registry

### J. B. Kuemmerle-Deschner^1^, J. Henes^2^, B. Kortus-Goetze^3^, P. T. Oommen^4^, A. Pankow^5^, T. Kallinich^6,7^, T. Krickau^8,9,10^, C. Schuetz^11^, G. Horneff^12,13^, I. Foeldvari^14^, J. Rech^8,9,15^, F. Weller-Heinemann^16^, A. Janda^17^, M. Hufnagel^18^, F. M. Meier^19,20^, F. Dressler^21^, M. Borte^22^, I. Andreica^23^, P. Wasiliew^1^, M. Fiene^24^, D. Windschall^25^, J. Weber-Arden^26^, N. Blank^27^

#### ^1^Division of Pediatric Rheumatology and autoinflammation reference center Tuebingen, Department of Pediatrics; ^2^University Hospital Tuebingen, University Hospital Tuebingen, Tuebingen; ^3^Department of Internal Medicine, Division of Nephrology, University Hospital of Giessen and Marburg, Marburg; ^4^Department of Pediatric Oncology, Hematology and Clinical Immunology, Center for Child and Adolescent Health, Medical Faculty Heinrich-Heine-University Duesseldorf, Duesseldorf; ^5^Department of Rheumatology and Clinical Immunology, Charité-Universitätsmedizin Berlin; ^6^Department of Pediatric Respiratory Medicine, Immunology and Critical Care Medicine, Charité Universitätsmedizin Berlin; ^7^Deutsches Rheuma-Forschungszentrum (DRFZ) , Berlin; ^8^Centre for rare diseases Erlangen (ZSEER); ^9^DZI (Deutsches Zentrum für Immuntherapie); ^10^Department of Pediatrics, Friedrich-Alexander University Erlangen-Nuernberg (FAU), Erlangen; ^11^Department of Pediatrics, Medizinische Fakultät Carl Gustav Carus, Technische Universität Dresden, Dresden; ^12^Department of Pediatrics, Asklepios Kinderklinik Sankt Augustin, Sankt Augustin; ^13^Department of Pediatric and Adolescent Medicine, Medical Faculty, University Hospital of Cologne, Cologne; ^14^Hamburg Centre for Pediatric and Adolescence Rheumatology, Hamburg; ^15^Department of Rheumatology and Clinical Immunology, University Hospital Erlangen, Erlangen; ^16^Division of Pediatric Rheumatology, Prof. Hess Children's Hospital, Bremen; ^17^Department of Pediatrics and Adolescent Medicine, University Medical Center Ulm, Ulm; ^18^Division of Pediatric Infectious Diseases and Rheumatology, Department of Pediatrics and Adolescent Medicine, University Medical Center, Medical Faculty, University of Freiburg, Freiburg; ^19^Fraunhofer Institute for Translational Medicine and Pharmacology ITMP; ^20^Department of General Pharmacology and Toxicology, Goethe University Hospital and Goethe University Frankfurt, Frankfurt am Main; ^21^Department of Paediatric Pneumology, Allergology and Neonatology, Children's Hospital, Hannover Medical School, Hannover; ^22^Hospital for Children & Adolescents, St. Georg Hospital, Leipzig; ^23^Rheumazentrum Ruhrgebiet Herne, Ruhr-Universität Bochum, Herne; ^24^Rheumatology Center Greifswald, Greifswald; ^25^Clinic of Paediatric and Adolescent Rheumatology, St. Josef-Stift Sendenhorst, Northwest German Center for Rheumatology, Sendenhorst; ^26^Immunology, Novartis Pharma GmbH, Nuernberg; ^27^Division of Rheumatology, Department of Internal Medicine, Heidelberg University Hospital, Heidelberg, Germany

##### **Correspondence:** J. B. Kuemmerle-Deschner

*Pediatric Rheumatology 2023*, **21(Suppl 2):**PT005

**Introduction:** Autoinflammatory diseases (AID) have been treated safely and effectively with the interleukin-1β inhibitor canakinumab (CAN) in controlled trials and routine clinical practice. The most common adverse event reported were infections.

**Objectives:** In this study infections and infection rates in patients with cryopyrin-associated periodic syndromes (CAPS), familial Mediterranean fever (FMF), hyper-IgD syndrome/mevalonate kinase deficiency (HIDS/MKD) and tumor necrosis factor receptor-associated periodic syndrome (TRAPS) on CAN therapy were investigated in a real-world setting.

**Methods:** RELIANCE is a prospective, non-interventional, observational study in Germany enrolling pediatric (age ≥2 years) and adult patients with a clinically confirmed diagnosis of AID who routinely receive CAN. Efficacy and safety parameters are recorded at baseline and assessed at 6-month intervals.

**Results:** The present interim analysis is based on data from a total of n=232 patients including n=101 (44%) pediatric patients under 18 years diagnosed with autoinflammatory diseases enrolled in the RELIANCE registry. The median duration of CAN treatment before and during study in the pediatric cohort was 4 years (0−15 years).

Between 2017 and 2022, 898 adverse events (AE) were recorded in n=164 patients (71%). The incidence rate per 100 patient years (IR) was 163.82. Serious adverse events (SAE) were reported for n=35 patients (15%; 98 events, IR 17.88).

During the study, infections occurred in 54.5% of patients (55 patients, 131 AE, including 11 SAE). To closely monitor the impact of long-term CAN treatment on infection rates in pediatric patients, data from a total of n=53, 71, 83 and 80 pediatric patients enrolled in the study in 2019, 2020, 2021, and 2022 were compared. The IR of non-serious and serious infections in pediatric patients was 75.24 and 10.75 in 2019, and dropped to 44.90 and 4.99 in 2020, and 29.65 and 0.00 in 2021. The IR decrease might be caused by the periods of social distancing during the coronavirus pandemic in 2020 and 2021. In 2022, the IR of non-serious infections increased to 105.50 while the IR of serious infection stayed flat (IR 2.15). The course of upper respiratory tract infections IR in 2019, 2020, 2021, and 2022 was comparable to the IR of non-serious infections: 10.75, 8.32, 1.85, and 10.76. No cumulation of non-serious and serious infections under Canakinumab long-term treatment could be observed in the pediatric cohort.

**Conclusion:** Interim data of the RELIANCE study confirm that in the pediatric cohort the risk of infections including upper respiratory tract infections does not accumulate over 4 years under CAN treatment.

**Patient Consent**

Yes, I received consent

**Disclosure of Interest**

J. Kuemmerle-Deschner Grant / Research Support with: Novartis, AbbVie, Sobi, Consultant with: Novartis, AbbVie, Sobi, J. Henes Grant / Research Support with: Novartis, Roche, Consultant with: Novartis, AbbVie, Sobi, Roche, Janssen, Boehringer-Ingelheim, B. Kortus-Goetze Consultant with: Novartis, P. Oommen Grant / Research Support with: Novartis, A. Pankow: None declared, T. Kallinich Speaker Bureau with: Roche, T. Krickau Grant / Research Support with: Novartis, Consultant with: Novartis, Speaker Bureau with: Novartis, C. Schuetz Grant / Research Support with: Novartis, G. Horneff Grant / Research Support with: AbbVie, Chugai, Merck Sharp & Dohme, Novartis, Pfizer, Roche, Speaker Bureau with: AbbVie, Bayer, Chugai, Merck Sharp & Dohme, Novartis, Pfizer, Roche, I. Foeldvari Consultant with: Novartis, J. Rech Grant / Research Support with: Novartis, Sobi, Consultant with: AbbVie, Biogen, BMS, Chugai, GSK, Janssen, Lilly, MSD, Mylan, Novartis, Roche, Sanofi, Sobi, UCB, Speaker Bureau with: AbbVie, Biogen, BMS, Chugai, GSK, Janssen, Lilly, MSD; Mylan, Novartis, Roche, Sanofi, Sobi, UCB, F. Weller-Heinemann: None declared, A. Janda: None declared, M. Hufnagel Grant / Research Support with: Novartis, F. Meier Speaker Bureau with: Novartis, F. Dressler Grant / Research Support with: Novartis, Consultant with: Abbvie, Mylan, Novartis, Pfizer, M. Borte Grant / Research Support with: Pfizer, Shire, I. Andreica Consultant with: Abbvie, Chugai, Novartis, UCB, Galapagos, Takeda, Astrazeneca, Lilly, Boehringer Ingelheim, Amgen, Sobi, Paid Instructor with: Astrazeneca, UCB, Speaker Bureau with: Abbvie, Chugai, Novartis, UCB, MSD, Lilly, Sobi, Astrazeneca, Amgen, Pfizer, Gilead, P. Wasiliew: None declared, M. Fiene: None declared, D. Windschall: None declared, J. Weber-Arden Employee with: Novartis, N. Blank Grant / Research Support with: Novartis, Sobi, Consultant with: Novartis, Sobi, Lilly, Pfizer, Abbvie, BMS, MSD, Actelion, UCB, Boehringer-Ingelheim, Roche

## PT006 Clinical, immunologic, and genetic characteristics in patients with Syndrome of Undifferentiated Recurrent Fevers (SURF)

### M. Macaraeg^1^, M. Matt^1^, E. Baker^2^, E. Handorf^1^, G. Schulert^1^

#### ^1^Rheumatology; ^2^Genetics, Cincinnati Children's Hospital Medical Center, Cincinnati, United States

##### **Correspondence:** M. Macaraeg

*Pediatric Rheumatology 2023*, **21(Suppl 2):**PT006

**Introduction:** SURF represents a heterogeneous group of disorders characterized by self-limited recurrent fevers and systemic autoinflammation without confirmed molecular diagnosis of a Hereditary Recurrent Fever syndrome, and not fulfilling criteria for Periodic Fever, Adenitis, Pharyngitis, Aphthous stomatitis syndrome (PFAPA), and is a cause of significant burden to affected families due to days of daycare or school missed for the child, and days of work missed for the parent. Very little is currently known about SURF, and many different phenotypes are likely encompassed by this term.

**Objectives:** Our primary objectives are to define the clinical manifestations, treatment responses, cytokine signatures, and genetic variants of SURF. Our secondary objective is to compare clinical manifestations and cytokine signatures of SURF patients to PFAPA.

**Methods:** We enrolled 47 patients followed at the Cincinnati Children’s Hospital (CCHMC) Autoinflammatory Treatment and Research Center with recurrent fever who met criteria for SURF and did not satisfy EULAR/PRINTO PFAPA classification criteria. Clinical course was followed over time. Cytokines were run using a multiplex Luminex assay. In some patients, whole exome sequencing was done, with focused analysis of 394 genes implicated in known inflammatory disorders as well as primary immunodeficiency syndromes. This research was approved by the Institutional Review Board of CCHMC and all patients or parents/guardians provided informed consent.

**Results:** Pharyngitis and adenopathy were present in a minority of SURF patients compared to PFAPA, while rash and arthralgias were significantly more common in SURF. There were no significant differences in serum levels of proinflammatory cytokines between SURF and PFAPA patients; however, SURF had more outliers with marked elevations in IL1b, IL6, IL8, IFNg, IL17a, IL18, and IL23. Hierarchical clustering shows a distinct subgroup of SURF patients with elevated IFNg, IL17a, IL12p70, and IL23 compared to PFAPA. Successful treatment strategies in SURF patients ranged from self-resolution to the need for anti-IL1 therapy. Genetic variants of unknown significance (VUS) were frequently found in patients with SURF, particularly in genes implicated in T and B cell development and function, granulocyte/monocyte function, immunodeficiencies, and IBD risk.

**Conclusion:** Our preliminary findings suggest SURF is a heterogeneous group of disorders that have distinct clinical and immunologic features, and treatment responses. We also find frequent VUS in pathways which may have relevance to disease pathogenesis, and possible associations to SURF endotypes. Further collaborative research is necessary to understand these SURF endotypes, what drives the disorder, and how physicians can better predict which treatment will be most successful for each patient.

**Patient Consent**

Yes, I received consent

**Disclosure of Interest**

None declared

## PT007 Tonsillectomy in PFAPA – many are cured but pfapa-related symptoms are common at long-term follow-up

### T. Moberg^1^, K. Rydenman^1,2^, S. Berg^2,3^, A. Fasth^2,3^, P. Wekell^1,2,3^

#### ^1^Department of Pediatrics, NU Hospital Group, Uddevalla; ^2^Department of Pediatrics, Institute of Clinical Sciences, University of Gothenburg; ^3^Department of Pediatric Rheumatology and Immunology, Queen Silvia Children’s Hospital, Gothenburg, Sweden

##### **Correspondence:** T. Moberg

*Pediatric Rheumatology 2023*, **21(Suppl 2):**PT007

**Introduction:** Tonsillectomy is an established treatment for PFAPA today. Data on both short- and long-term impact are important when deciding if the child should undergo tonsillectomy or not. There is, however, a lack of data on follow-up, in particular on the long-term effects of the procedure.

**Objectives:** To describe the short- and long-term effects of tonsillectomy on PFAPA-episodes and PFAPA-related symptoms.

**Methods:** All patients diagnosed with PFAPA between 2006 and 2017 at the Queen Silvia Children's Hospital, NU Hospital Group and Skaraborg Hospital were identified. Medical records were reviewed and all patients that had undergone tonsillectomy after the onset of PFAPA were included. A structured telephone interview regarding PFAPA symptoms over time, the effect of tonsillectomy and symptoms at the time of follow-up was held with caregivers and patients ≥ 18 years of age. For patients <18 years of age at the time of follow-up, the interview was held with a caregiver only. Patients ≥18 years of age at follow-up were interviewed regarding present symptoms, and their previous caregiver regarding the effect of tonsillectomy and symptoms as a child.

**Results:** Out of 101 patients identified, 3 were excluded as they didn’t live in Sweden at follow-up. Of the remaining 98 patients, 86 (88%) accepted to participate in the study. Among patients ≥ 18 years of age that were interviewed, caregivers were unavailable in 4 cases, i.e., complete data set was available for 82 patients (84%).

Median follow-up time after tonsillectomy was 8.8 years (range 2.8-16.1) and median age at follow-up 14.8 years (range 6.0-28.8). Median age at onset of PFAPA was 1.8 years (range 0.1-16.0), median age at tonsillectomy 5.1 years (range 2.3-18.8) and median duration of symptoms at tonsillectomy 3.0 years (range 0.5-11.5). 58% of patients were male and 42% female.

Responses from caregivers showed that 36/82 (44%) of patients had complete resolution of all symptoms after tonsillectomy and symptoms did not return during the follow-up time. In 19/82 (23%) patients, the febrile episodes became milder or fewer after tonsillectomy, while in 17/82 (21%) patients, the febrile episodes disappeared, but other PFAPA-related symptoms persisted. In 9/82 (11%) patients, the febrile episodes disappeared but returned after a median time of 1 year (range 0,5-4,5 years). Finally, 1/82 patients (1%) did not have any effect of tonsillectomy.

Regarding symptoms at the time of follow-up, we found that 15/86 (17%) patients still had febrile episodes (>38°C). Out of them, twelve had episodes with regular intervals, two with irregular intervals and one had episodes triggered by exercise. Four patients were treated with colchicine, one with betamethasone and eight did not have any contact with healthcare providers. 17/86 (20%) still had regularly or irregularly recurring non-febrile PFAPA-related symptoms such as subfebrility, malaise, aphthae, pharyngitis, swollen lymph nodes, joint pain, abdominal pain and headache. Most described their episodes as similar to what they had experienced before tonsillectomy, but now milder and without fever. Twelve patients had recurring aphthae of whom nine had aphthae as their only symptom.

**Conclusion:** Tonsillectomy is an effective treatment for PFAPA. In some patients, however, the fever episodes reappeared and in some non-febrile symptoms of PFAPA continued. At long-term follow up, 17% still had febrile episodes and 20% still had other PFAPA-related symptoms. These results have implications when deciding if a child should undergo tonsillectomy or not, but also for the understanding of children with persisting or reappearing symptoms after tonsillectomy.

**Patient Consent**

Not applicable (there are no patient data)

**Disclosure of Interest**

None declared

## PT008 Decision tree analysis as preliminary evidence-based classification criteria for SURF (Syndrome of Undifferentiated Recurrent Fever)

### R. Papa^1^, F. Bovis^2^, S. Federici^3^, M. Bustaffa^1^, S. Palmeri^1^, C. Matucci-Cerinic^1^, D. Sutera^4^, R. Bertelli^5^, M. Cecconi^5^, S. Volpi^1^, R. Caorsi^1^, N. Ruperto^6^, M. Gattorno^1^

#### ^1^UOC Reumatologia e Malattie Autoinfiammatorie, IRCCS Istituto Giannina Gaslini; ^2^Dipartimento di Scienze della Salute - DISSAL, Università degli Studi di Genova, Genova; ^3^UOC Reumatologia, IRCCS Ospedale Pediatrico Bambino Gesù, Roma; ^4^Patologia Umana dell'adulto e del bambino Gaetano Barresi, Università di Messina, Messina; ^5^UOC Laboratorio di Genetica Umana; ^6^UOC Centro Trial, IRCCS Istituto Giannina Gaslini, Genova, Italy

##### **Correspondence:** S. Palmeri

*Pediatric Rheumatology 2023*, **21(Suppl 2):**PT008

**Introduction:** Syndrome of undifferentiated recurrent fever (SURF) comprises a group of patients characterized by: i) recurrent fever episodes, ii) negative molecular analysis for genes associated with hereditary recurrent fevers (HRF), iii) absence or marginal incidence of the typical clinical features of PFAPA syndrome, iv) complete or good response to colchicine treatment.

**Objectives:** Aim of this study was to compare SURF, HRF and PFAPA patients enrolled in the Eurofever registry in order to identify evidence-based criteria that could help in the classification of SURF patients.

**Methods:** 59 pediatric SURF patients followed in a single tertiary center for Autoinflammatory diseases were enrolled in the Eurofever registry according to the inclusion criteria already published. A group of 188 pediatric patients (< 18 years of age) affected with confounding diseases (32 FMF, 32 TRAPS, 56 MKD, 31 CAPS and 37 PFAPA) validated by international experts has been used as historical disease control group. A decision trees approach was tested randomly splitting the available data in a training set (70%) and in an internal validation set (30%). The recursive partitioning (rPART) method was used to identify the optimal split of the data that would best classify autoinflammatory diseases patients into SURF or not SURF. Two fully grown decision tree classifiers with no limit on the complexity parameter were developed using i) a combinations of genetic and clinical data; ii) clinical data only with the exclusion of the genetic information. We then calculated the area under the receiver operating characteristic (ROC) curve (AUC), accuracy, sensitivity, and specificity of the decision tree classifier for predicting SURF vs NON-SURF.

**Results:** A total of 247 patients were included in the analysis and randomly split into a training set (N=186) and a validation set (N=61). All the performance values obtained in the training cohort and in the validation set are summarized in the Table 1. To support the decision tree results, an alternative model was created using a logistic regression model. The models including genetic information yielded better performance measurements compared to those without genetic information. According to the decision tree including the genetic analysis, the probability of being classified as SURF having a positive genetic test is 0%, while the same probability increased to 92% if the genetic test is negative and the patient does not present exudative tonsillitis. In the absence of a genetic test, patients showed a higher probability of being classified as SURF in the absence of triggers for episodes and pain at lymph nodes, negative family history, age of disease onset > 2 years, mean duration of episodes > 2.5 days. An external validation is ongoing.

**Conclusion:** Preliminary evidence-based classification criteria to differentiate SURF patients from the main causes of recurrent fevers in childhood are available. A decision tree including the genetic analysis yielded the highest accuracy. However, the decision tree was able to discriminate SURF patients from confounding diseases even in the absence of information on genetic analysis.

**Patient Consent**

Not applicable (there are no patient data)

**Disclosure of Interest**

None declared

## PT009 Long-term follow-up of children with chronic non-bacterial osteomyelitis - assessment of disease activity and outcome using the proposed Carra disease activity score and adaptation

### C. Reiser^1,2^, J. Klotsche^3,4^, N. Grösch^3^, M. Niewerth^3^, K. Minden^3,4^, H. J. Girschick^5,6,7^

#### ^1^Pediatrics, LKH Bregenz, Bregenz, Austria; ^2^Pediatric Rheumatology, Department of Pediatrics and Autoinflammation reference Center Tuebingen (arcT), University Childrens' Hospital, Tuebingen; ^3^Deutsches Rheuma-Forschungszentrum Berlin, ein Institut der Leibniz-Gemeinschaft; ^4^Charité – Universitätsmedizin Berlin, corporate member of Freie Universität Berlin, Humboldt Universität zu Berlin, and Berlin Institute of Health; ^5^Children’s Hospital, Vivantes Klinikum Friedrichshain; ^6^German Center for Growth and Development DeuzWeg, Berlin; ^7^Pediatrics, University Childrens' Hospital, Wuerzburg, Germany

##### **Correspondence:** C. Reiser

*Pediatric Rheumatology 2023*, **21(Suppl 2):**PT009

**Introduction:** The National Pediatric Rheumatologic Database (NPRD) collects long-term data of children and adolescents including CNO. Scores are warranted to assess disease activity (DA) and inactive disease.

**Objectives:** To assess disease activity (DA) and outcome of CNO in the NPRD cohort with onset in childhood and adolescence using the recently proposed numeric composite disease activity score developed by the CARRA (1), in addition to a score adaptation including whole body MRI defined lesions.

**Methods:** From 2015-2021 patients with a confirmed diagnosis of CNO were included in this analysis and observed for up to 4 years. Here we report the **first-year outcome**, including denominators for active and inactive disease.

**Results:** 400 patients with a diagnosis of CNO were enrolled in the NRPD during the study period. The CARRA clinical DA score (CARRA CDAS) consists of the sum of three DA components (patient global DA and patient pain measured via numeric rating scale (NRS) + the number of clinical lesions). To underline the significance of the number of radiological lesions regarding long term outcome and therapeutic decisions, a score adaptation was considered, by exchanging the number of clinical lesions by MRI defined lesions: MRI CDAS (patient global DA, patient pain, MRI lesions). Inactive disease may be considered if each item of the scores shows a NRS below 1 and no lesions are noticed /detected. Compared to the CARRA Validation cohort, a mild to moderate DA could be shown. At baseline the CARRA CDAS is 6.7 (mean (standard deviation sd 5.4); median 5.5), compared to 4.9 ((4.9); 4) after one year.

Initial patient global DA (2.7), physician global DA (2.1) and clinical lesions (1.3) dropped to 2.0, 1.2, 0.9, respectively, MRI lesions, dropped from 2.2 to 1.8 after one year.

**Conclusion:** An improvement of DA was documented either in the individual score components, as well as in the newly set up CARRA CDAS as well as in the MRI CDAS, suggesting that the majority of CNO patients experiences an improvement in the first year of follow-up. Further score adaptation and validation, and the analysis of the complete follow up time of 4 years is planned. Composite numeric scores might help to describe CNO activity including inactive disease. The proposed composite CDAS/its variation might help to institute effective treatment modalities earlier.

**Patient Consent**

Yes, I received consent

**Disclosure of Interest**

None declared

**Reference**

1. Wu EY, Oliver M, Scheck J, Lapidus S, Akca UK, Yasin S, et al. Feasibility of Conducting Comparative Effectiveness Research and Validation of a Clinical Disease Activity Score for Chronic Nonbacterial Osteomyelitis. Rheumatology; 2022 Oct [cited 2023 May 12]. Available from: http://medrxiv.org/lookup/doi/10.1101/2022.10.03.22280351

## PT010 Can we predict the discontinuation of biological agents in colchicine-resistant pediatric familial Mediterranean fever patients?

### Ö. Taş^1^, F. Aydın^1^, M. Sezer^2^, B. Çelikel Acar^2^, O. Bahçeci^1^, N. Çakar^1^, B. Özçakar^1^

#### ^1^Ankara University of Medicine Department of Pediatric Rheumatology; ^2^Ankara Bilkent City Hospital Department of Pediatric Rheumatology, Ankara, Türkiye

##### **Correspondence:** Ö. Taş

*Pediatric Rheumatology 2023*, **21(Suppl 2):**PT010

**Introduction:** Familial Mediterranean fever (FMF) is a monogenic autoinflammatory disease characterized by recurrent, self-limiting episodes of fever and sterile serositis. Colchicine is the mainstay of treatment. Despite the maximum tolerated colchicine doses, approximately 5-10% of patients may respond inadequately to colchicine. Anti-IL-1 agents are important treatment options in these patients.

**Objectives:** Our aim is to investigate the characteristics of colchicine-resistant FMF patients who continue anti-IL-1 therapy and whose therapy can be discontinued.

**Methods:** Electronic medical records of colchicine-resistant FMF patients receiving anti-IL-1 therapy at two referral centers in Ankara were evaluated retrospectively. Demographic, clinical characteristics and disease severity of patients in whom anti-IL-1 treatment was continued and discontinued were compared. Disease severity was evaluated with the international FMF severity scoring system (ISSF).

**Results:** Among 64 colchicine-resistant FMF patients, 39 (61%) were female. The median age at the onset of symptoms was 3 years (6 months-15 years), age at the diagnosis was 5 years (6 months-16years), the mean follow-up duration was 117.05 ± 53.80 months and the mean duration of biological use was 44.65±30.53 months. All patients received colchicine therapy along with anti IL- 1 agent. Treatment of 26 (40.6%) patients was started with anakinra, 38 (59.4%) with canakinumab. Eighteen of the patients receiving anakinra were switched to canakinumab. During follow-up, anti-IL-1 treatment was discontinued in 23 (35.9%) patients. Mean duration of biological use in these patients was 35.39±24.18 months, and median follow-up period was 40 months (6-81) after the drug was discontinued. Re-treatment was initiated in 1 patient. All clinical and demographic characteristics, colchicine doses, pre and post-biological ISSF scores of both groups were compared. ISSF scores before biological treatment were significantly higher in whom biological therapy could not be discontinued (mean 4.03±1.59) compared to those who could be discontinued (mean 3.13±1.10) (p<0,009). Even it was not statistically significant, the age at disease onset was lower and leg pain was less common in the group of patients that biologic treatment could be discontinued (p=0,06).

**Conclusion:** There is no definitive recommendation for the optimal duration and discontinuation of biological therapy for colchicine-resistant FMF patients. Low ISSF scores before biological treatment may predict the discontinuation of biologic agents in colchicine-resistant pediatric FMF patients.

**Patient Consent**

Not applicable (there are no patient data)

**Disclosure of Interest**

None declared

## PT011 Open-label phase 3 study of intravenous golimumab in patients with polyarticular Juvenile idiopathic arthritis: pharmacokinetics, effectiveness, safety, and immunogenicity over 252 weeks

### H. I. Brunner^1^, D. J. Lovell^1^, S. Ringold^2^, X. L. Xu^2^, E. Lam^2^, Y. Wang^2^, J. H. Leu^2^, A. Martini^3^, N. Ruperto^4^ on behalf of PRCSG and PRINTO Investigators

#### ^1^Division of Rheumatology, Cincinnati Children’s Hospital Medical Center, Cincinnati; ^2^Janssen Research & Development, LLC, Spring House, United States; ^3^Dipartimento di Neuroscienze, Riabilitazione, Oftalmologia, Genetica e Scienze Materno-Infantili (DiNOGMI), Universita degli Studi di Genova, Genova; ^4^Clinica Pediatrica e Reumatologia, IRCCS Istituto Giannina Gaslini, Genoa, Italy

##### **Correspondence:** H. I. Brunner

*Pediatric Rheumatology 2023*, **21(Suppl 2):**PT011

**Introduction:** The phase 3, IV Golimumab in Pediatric Participants with Active Polyarticular-Course JIA Despite Methotrexate (GO-VIVA) study demonstrated that golimumab (GLM) 80 mg/m^2^ at Week 0, 4 and every 8 weeks thereafter is well-tolerated and effective in children 2 to < 18 years of age with active polyarticular-course juvenile idiopathic arthritis (pcJIA) despite methotrexate over 52 weeks (W).

**Objectives:** To evaluate the pharmacokinetics (PK), immunogenicity, efficacy, and safety of GLM in GO-VIVA participants (pts) who continued into the long-term extension (LTE).

**Methods:** Pts from GO-VIVA who continued GLM 80 mg/m^2^ IV q8w (max single dose 240 mg) after W52 were included. PK and immunogenicity were assessed through W244, and safety was assessed through W252. Efficacy measures included JIA ACR response from baseline (start of GLM), clinical Juvenile Arthritis Disease Activity Score based on 10 joints (cJADAS-10) minimal disease activity (cJADAS-10 MDA), inactive disease (cJADAS-10 ID) and remission (≥ 6 continuous months of cJADAS-10 ID). These were analyzed using an intent-to-treat (ITT) approach through W116, due to a protocol amendment instituted during the LTE that limited efficacy data collected after W116. Non-responder imputation was used for missing data.

**Results:** Of the 127 pts treated, 112 (88.2%) continued into the LTE, and 69 (54.3%) completed GLM through W244 and had a W252 assessment. W244 median steady-state trough GLM concentration was 0.61 μg/mL (mean ± SD: 0.66 ± 0.569μg/mL; N=31). Median steady-state trough serum GLM concentrations ranged from 0.29 to 0.61 μg/mL, indicating that exposure was maintained over time. Median trough GLM concentrations at W244 were similar across different age categories and body weight quartiles. At each visit for which efficacy was evaluable, the majority of pts had JIA ACR 30 (72%-77%), JIA ACR 50 (71%-76%), and JIA ACR 70 (62%-68%) responses, and approximately 50% had JIA ACR 90. The majority of pts had cJADAS-10 MDA, 41%-49% achieved cJADAS-10 ID, and 28-33% achieved remission. Antibodies to GLM were detected in 56 (44.8%) of pts. Of these 56 pts, 35 were positive for neutralizing antibodies (Nab) with an overall incidence of NAb of 31% (35/112). No new or unexpected safety events were reported. 92.1% of pts experienced ≥ 1 AE. SAEs were reported in 19.7% of pts. One death (septic shock) occurred.

**Conclusion:** PK exposure through the end of the LTE was consistent to that observed through W52. Although analyses were limited by protocol modification, data through W116 suggest an efficacy benefit with additional treatment and achievement of clinically important endpoints for pts who continued in the LTE. GLM was generally well tolerated with an acceptable long-term safety profile through W252.

**Trial registration identifying number:** NCT02277444

**Patient Consent**

Not applicable (there are no patient data)

**Disclosure of Interest**

H. Brunner Grant / Research Support with: The Cincinnati Children’s Hospital, where HBR works as a full-time public employee, has received contributions from the following industries in the past 3 years: Bristol-Myers Squibb, Eli Lilly, GlaxoSmithKline, F. Hoffmann-La Roche, Janssen, Novartis, and Pfizer. This funding has been reinvested for the research activities of the hospital in a fully independent manner, without any commitment to third parties, Consultant with: AbbVie, Astra Zeneca-Medimmune, Biogen, Boehringer, Bristol-Myers Squibb, Celgene, Eli Lilly, EMD Serono, Genzyme, GlaxoSmithKline, F. Hoffmann-La Roche, Janssen, Merck, Novartis, R-Pharm, Sanofi, Speaker Bureau with: Novartis, Roche, GlaxoSmithKline , D. Lovell Grant / Research Support with: Janssen Research & Development LLC, Bristol Meyers Squibb, Roche Laboratories, Consultant with: United Bioscience Corporation, AstraZeneca, GSK, Pfizer (Consultant & Member of Advisory Board), Novartis, Speaker Bureau with: Novartis, Pfizer, S. Ringold Employee with: Janssen Research & Development, LLC, X. Xu Shareholder with: Johnson & Johnson, Employee with: Janssen Research & Development, LLC, E. Lam Shareholder with: Johnson & Johnson, Employee with: Janssen Research & Development, LLC, Y. Wang Shareholder with: Johnson & Johnson, Employee with: Janssen Research & Development, LLC, J. Leu Shareholder with: Johnson & Johnson, Employee with: Janssen Research & Development, LLC, A. Martini Consultant with: Eli-Lilly, EMD Serono, Janssen, Novartis, Pfizer, AbbVie, Idorsia, Boheringer, N. Ruperto Grant / Research Support with: The IRCCS Istituto Giannina Gaslini (IGG), where NR works as full-time public employee has received contributions from the following industries: Bristol Myers and Squibb, Eli-Lilly, F Hoffmann-La Roche, Novartis, Pfizer, Sobi. This funding has been reinvested for the research activities of the hospital in a fully independent manner, without any commitment with third parties, Consultant with: 2 Bridge, AMGEN, AstraZeneca, Aurinia, Bayer, BMS, Celgene, Cambridge Healthcare Research (CHR), Domain Therapeutic, Eli Lilly, EMD, GSK, Idorsia, Janssen, Novartis, Pfizer, UCB, Speaker Bureau with: 2 Bridge, AMGEN, AstraZeneca, Aurinia, Bayer, BMS, Celgene, Cambridge Healthcare Research (CHR), Domain Therapeutic, Eli Lilly, EMD, GSK, Idorsia, Janssen, Novartis, Pfizer, UCB

**Reference**

1. Ruperto N, Brunner HI, Pacheco-Tena C, Louw I, Vega-Cornejo G, Spindler AJ, Kingsbury DJ, Schmeling H, Borzutzky A, Cuttica R, Inman CJ, Malievskiy V, Scott C, Keltsev V, Terreri MT, Viola DO, Xavier RM, Fernandes TAP, Velázquez MDRM, Henrickson M, Clark MB, Bensley KA, Li X, Lo KH, Leu JH, Hsu CH, Hsia EC, Xu Z, Martini A, Lovell DJ; Pediatric Rheumatology Collaborative Study Group (PRCSG) and the Paediatric Rheumatology International Trials Organisation (PRINTO). Open-label phase 3 study of intravenous golimumab in patients with polyarticular juvenile idiopathic arthritis. Rheumatology (Oxford). 2021 Oct 2;60(10):4495-4507. doi: 10.1093/rheumatology/keab021. PMID: 33493312; PMCID: PMC8487314.

## PT012 Open-label, phase 2B study of sarilumab in patients (PTS) with Polyarticular-Course Juvenile Idiopathic Arthritis (PCJIA): 1-year data

### F. De Benedetti^1^, I. Calvo Penadés^2^, I. Nikishina^3^, I. Foeldvari^4^, A. J. Spindler^5^, A. Kozlova^6^, N. Rubio-Pérez^7^, P. Quartier^8^, Z. Żuber^9^, R. Barria^10^, D. Clemente^11^, G. Vega Cornejo^12^, K. Marzan^13^, N. Liu^14^, C. Xu^14^, M. C. Nivens^15^, A. Giannelou^15^, B. Akinlade^15^, L. Baret-Cormel^16^

#### ^1^Ospedale Pediatrico Bambino Gesù , Rome, Italy; ^2^Instituto de Investigación Sanitaria La Fe, Valencia, Spain; ^3^V.A. Nasonova Research Institute of Rheumatology, Moscow, Russian Federation; ^4^Hamburg Center for Pediatric and Adolescent Rheumatology, Am Schoen Klinik Eilbek, Hamburg, Germany; ^5^Centro Médico Privado de Reumatologia, Tucumán, Argentina; ^6^Centre of Pediatric Hematology, Oncology and Immunology, Moscow, Russian Federation, ^7^University Hospital Dr. José Eleuterio González, Monterrey, Mexico; ^8^Necker Hospital, Paris, France; ^9^Andrzej Frycz Modrzewski Krakow University , Krakow, Poland; ^10^Bioreuma, Concepción, Chile; ^11^Hospital Infantil Universitario Niño Jesús , Madrid, Spain; ^12^CREA de Guadalajara, Jalisco, Mexico; ^13^Children's Hospital Los Angeles, Keck School of Medicine, University of Southern California, Los Angeles; ^14^Sanofi, Bridgewater; ^15^Regeneron, Tarrytown, United States; ^16^Sanofi, Paris, France

##### **Correspondence:** F. De Benedetti

*Pediatric Rheumatology 2023*, **21(Suppl 2):**PT012

**Introduction:** A 12-week (W) open-label, dose-finding study evaluated three subcutaneous doses of sarilumab in two weight groups of pts with pcJIA (Group A/B: body weight ≥30 kg/ ≥10 kg–<30 kg])^1^. Based on the results, Dose 2 (3/4 mg/kg every 2 weeks (q2w) for Group A/B) was selected for further evaluation in an extension phase and with additional pts.

**Objectives:** To present 1-year data of pcJIA pts who received Dose 2 from baseline (BL)

**Methods:** This phase 2b, open-label, multicentre study comprised a 12W core dose-finding phase and an extension phase. It enrolled pcJIA pts aged 2–17 years. Primary endpoint: sarilumab pharmacokinetic exposure from BL to W12. Secondary endpoints: JIA ACR response rate and safety

**Results:** Of 73 pts (Group A/B: n=42/31) treated, majority were female (79.5%) with mean (SD) age of 9.5 (4.7) years (Group A/B: 12.6 (3.0)/ 5.4 (3.1) years) at BL. Mean (SD) disease duration and cJADAS10 was 2.48 (3.28) and 20.07 (4.09), respectively. Among them, 17.8% had RF+ polyarticular JIA. Concomitant use of conventional synthetic DMARD (mainly methotrexate) and systemic glucocorticoids (GC) were noted in 84.9% and 13.7% of the pts, respectively; 19.2% pts had prior treatment with biologic DMARD (mainly TNFi).

The observed C_trough_ were comparable in both groups and were 7.49 to 9.47 mg/L at W12 and 11.6 to 14.2 mg/L at W48. JIA ACR 70/90 response rates were 80.9%/45.6% (Group A: 74.4%/43.6%; Group B: 89.7%/48.3%) at W12 and 93.8%/76.6% (Group A: 89.5%/68.4%; Group B: 100%/88.5%) at W48. Proportion of the pts who reported cJADAS-10≤2.5, clinically inactive disease (CID) as per Wallace criteria with no systemic GC use and clinical remission (CID for 6 consecutive months) at W48 were 73.4%, 60.9% and 51.6%, respectively.

Treatment-emergent adverse events (AEs) were reported in 95.9% of the pts at W48; infections (79.5%) were the most common. Grade 3/4 neutropenia (AE of special interest) were reported in 27.4% pts at W52; all of them recovered within a few days and were not associated with increased infection risk. Six (8.2%) pts experienced 9 serious AEs (acute sinusitis, bone tuberculosis, tonsillar hypertrophy, inguinal hernia, pancreatic pseudocyst, acute pancreatitis, JIA, ligament rupture, meniscus injury) which were deemed unrelated to study drug by investigators. AEs leading to permanent treatment discontinuation (9.6%, 7 pts) were mostly neutropenia. No deaths were reported.

**Conclusion:** At steady state in pcJIA pts with Dose 2, exposure was comparable among the two body weight groups and was similar to that with the 200 mg q2w in adult pts with RA. There was a clinically relevant improvement in disease activity, with more than half of pts achieving remission at W48. Safety was consistent with the known profile of sarilumab.

**Trial registration identifying number:** NCT02776735

**Patient Consent**

Not applicable (there are no patient data)

**Disclosure of Interest**

F. De Benedetti Grant / Research Support with: AbbVie, Novartis, Novimmune, Pfizer, Roche, Sanofi, SOBI and UCB, Consultant with: Roche, SOBI and Novartis, I. Calvo Penadés Grant / Research Support with: AbbVie, Bristol-Myers Squibb, Clementia, MSD, Novartis, Pfizer, Roche and Sanofi, Speaker Bureau with: AbbVie, Novartis, Roche and SOBI; and has participated in advisory boards for AbbVie and Novartis, I. Nikishina Speaker Bureau with: Novartis, MSD, Pfizer, AbbVie, Hoffmann-La Roche, Janssen and Ipsen, I. Foeldvari Speaker Bureau with: Pfizer (also advisor), A. J. Spindler Speaker Bureau with: Eli Lilly, A. Kozlova: None declared, N. Rubio-Pérez Speaker Bureau with: AbbVie and Roche, P. Quartier Consultant with: AbbVie, Chugai-Roche, Lilly, Novartis, Novimmune, Sanofi (also member of a data safety monitoring board), and SOBI, Speaker Bureau with: AbbVie, Bristol-Myers Squibb, Chugai-Roche, Novartis, Pfizer, and SOBI, Z. Żuber: None declared, R. Barria Consultant with: Tecnofarma, Speaker Bureau with: Pfizer and Roche, D. Clemente Speaker Bureau with: Novartis and Roche, G. Vega Cornejo Grant / Research Support with: Bristol-Myers Squibb, Parexel, and Sanofi, K. Marzan Grant / Research Support with: Sanofi, Novartis and Pfizer, N. Liu Shareholder with: Sanofi, Employee with: Sanofi, C. Xu Shareholder with: Sanofi, Employee with: Sanofi, M. C. Nivens Shareholder with: Regeneron , Employee with: Regeneron , A. Giannelou Shareholder with: Regeneron, Employee with: Regeneron, B. Akinlade Shareholder with: Regeneron, Employee with: Regeneron, L. Baret-Cormel Shareholder with: Sanofi, Employee with: Sanofi

**Reference**

1. Benedetti FD, et al. Annals of the Rheumatic Diseases 2019;78:969-970.

## PT013 Inflammatory biomarkers in relation to long-term remission and active disease. A population-based study of the Nordic JIA cohort

### M. Glerup^1^, C. Kessel^2^, D. Foell^2^, L. Berntson^3^, A. Fasth^4^, C. Myrup^5^, E. Nordal^6,7^, V. G. Rypdal^6,7^, M. Rygg^8,9^, E. D. Arnstad^8,10^, S. Peltoniemi^11^, K. Aalto^12^, M. Høllsberg^1^, A. E. Bilgrau^13^, T. Herlin^1^

#### ^1^Pediatrics, Aarhus University Hospital, Aarhus, Denmark; ^2^Pediatric Rheumatology and Immunology, University Hospital Münster, Münster, Germany; ^3^Women's and Children's Health, Uppsala University, Uppsala; ^4^Pediatrics, Institute of Clinical Sciences, Gothenburg, Sweden; ^5^Pediatrics, Rigshospitalet, Copenhagen, Denmark; ^6^Pediatrics, University Hospital of North Norway; ^7^Clinical Medicine, UiT Arctic University of Norway, Tromsø; ^8^Clinical and Molecular Medicine, NTNU Norwegian University of Science and Technology; ^9^Pediatrics, St. Olavs Hospital; ^10^Pediatrics, Levanger Hospital, Trondheim, Norway; ^11^Rheumatology, Helsinki University Hospital; ^12^Pediatric Research Center, Children's Hospital, Helsinki University Hospital, Helsinki, Finland; ^13^Mathematical Sciences, Aalborg University, Aalborg, Denmark

##### **Correspondence:** T. Herlin

*Pediatric Rheumatology 2023*, **21(Suppl 2):**PT013

**Introduction:** Inflammatory biomarkers have been suggested to reflect disease activity in juvenile idiopathic arthritis (JIA) and contribute to the prediction of clinical outcome.

**Objectives:** To evaluate serum biomarkers obtained early during disease as predictors for disease activity and remission status at long-term follow-up (FU) 18 years after disease onset.

**Methods:** Patients from the population-based Nordic JIA cohort study were recruited close to disease onset from defined regions of Nordic countries between 1997-2000. Clinical data and serum biomarkers were obtained at baseline (6 (-1/+2) months from disease onset) and at 18-yr FU. S100 proteins, and 14 other inflammatory biomarkers (cytokines, chemokines) were determined by multiplexed bead array assay. The analyzing laboratory in Münster was blinded for the patients’ clinical data. We estimated both univariate and multivariate logistic regression models on binary outcomes of disease activity and remission with baseline variables as explanatory variables.

**Results:** Of the 510 patients from the Nordic JIA cohort, serum samples from 236 patients at baseline were available. Median JADAS10 at baseline was 5.0 (IQR 2.0-11.0) compared to 2.0 (IQR 0.0-6.4) at FU. Inactive disease at 18-yr FU was observed in 58% and remission off medication in 39% of the patients. No significant difference in clinical characteristics between the group of patients with baseline serum samples and the remaining group without blood samples was found. Computing receiver operating characteristics (ROC) illustrating the area under the curve (AUC) for the prediction of active disease at 18-Y FU we found that significant levels of AUC were obtained for baseline levels of IL-1β, IL-6, IL-12p70, IL-13, MMP-3, S100A9 and S100A12 as well as for ESR, and number of active and cumulative joints at the baseline visit. Nested multivariate logistic regressions models were compared in analysis of variance. We compared a traditional clinical model (with the variables gender, age, joint counts, ESR/CRP) and a combined clinical and biomarker model (clinical variables as above plus 16 biomarkers obtained at baseline: IL-1ß, IL-4, IL-6, IL-10, IL-12, IL-13, IL-17A, IL-18, TNFα, MMP-3; CCL-2, sCD25, GM-CSF, MPO, S100A9, S100A12). We found that the biomarkers significantly added to the explanatory value predicting the outcome regarding inactive/active disease at 18-yr FU (AUC increased from 0.59 to 0.80, p=0.024). Multiple regression analysis revealed S100A9 as the strongest predictor for active/inactive disease at long-term outcome, controlling for the other variables.

**Conclusion:** Biomarkers of inflammation obtained within the first 6 months after JIA onset may complement the characterization of disease activity and contribute to improved future prediction models of long-term outcome.

**Patient Consent**

Yes, I received consent

**Disclosure of Interest**

None declared

## PT014 Management of Temporomandibular Joint Involvement(TMJ) in Juvenile Idiopathic Arthritis (JIA) in our Centre

### K. Mclellan^1^, N. Saeed^2^, S. Compeyrot-Lacassagne^1^

#### ^1^Paediatric Rheumatology; ^2^Paediatric Maxillofacial Surgery, Great Ormond Street Hospital, London, United Kingdom

##### **Correspondence:** K. Mclellan

*Pediatric Rheumatology 2023*, **21(Suppl 2):**PT014

**Introduction:** Temporomandibular joint(TMJ) synovitis remains challenging in juvenile idiopathic arthritis(JIA) management. The American College of Rheumatology(ACR) 2021 JIA management guidelines identified TMJ involvement as a risk factor for poor outcome and conditionally as an indication to escalate systemic treatment.

**Objectives:** To review management of TMJ synovitis in our centre; identify the demographic with radiologically-proven TMJ synovitis, presence of damage and referral to maxillofacial services.

**Methods:** Case note retrospective review of children with TMJ synovitis confirmed radiologically at Great Ormond Street Hospital over 12-month period.

**Results:** 57 patients with JIA had a TMJ MRI; 45(79%) female. 12(17%) had MRI at diagnosis, 10(14%) within a year and 14(20%) 1 to 5 years from diagnosis. Indications for TMJ MRI were pain (71%), restricted movement (38%) and jaw swing (58%). 60% had other active joints.

The most common ILAR subtype with TMJ synovitis was oligoarticular JIA 26(46%) (18 ANA+), followed by polyarticular rheumatoid factor(RF)-negative 12(21%), enthesitis-related 6(11%), polyarticular RF-positive 4(7%), systemic 4(7%), psoriatic 3(5%) and inflammatory bowel disease-related 2(4%).

Of 58 JIA TMJ scans, 45(78%) were abnormal; 42(75%) had active synovitis and a further 2(3%) had biomechanical changes. 29/45(64%) had bilateral changes.

Of the 45 abnormal TMJ JIA scans, 44(98%) had synovial enhancement, 24(52%) condyle flattening/irregularity, 17(37%) erosions, 10(22%) oedema and 8(18%) effusion.

36/44(82%) JIA patients with radiologically-proven TMJ synovitis had a change in treatment; 26(72%) had other active joints. Of the 18 patients with TMJ synovitis, without other active joints,11 had a change in their medication; 6 started a biologic, 1 started methotrexate, 1 switched biologic and 3 had doses optimised; 7 had no change in management.

Of 44 JIA with TMJ synovitis, 7 were already under maxillofacial services and 14 new patients (38%) referred. 5 patients had surgical intervention, including 1 new referral.

**Conclusion:** Diagnosis of TMJ synovitis, with absence of early clinical findings, is challenging. In our cohort, 53% of JIA TMJ MRI scans had joint damage. Furthermore 64% had bilateral changes.

With a high proportion of abnormal scans, erosions and bilateral involvement, we highlight the importance of early MRI. TMJ synovitis should be systematically screened for using the published tools. We also raise the question of screening TMJ MRIs in a subset of JIA patients; oligoarticular JIA accounted for the highest proportion of TMJ synovitis in our cohort. Identifying subclinical TMJ synovitis is especially important in this cohort as this would influence management.

Intra-articular TMJ steroid injections have been shown not to reduce joint damage and may worsen osseous changes. In our JIA cohort with isolated radiologically-confirmed TMJ synovitis, 61% had a change in management. We suggest that all young people with TMJ synovitis should be referred to maxillofacial services for interdisciplinary management.

**Patient Consent**

Not applicable (there are no patient data)

**Disclosure of Interest**

None declared

## PT015 Prevalence and correlates of anxiety and depression symptoms in adolescents and young adults with Juvenile idiopathic arthritis

### F. Milatz^1^, J. Klotsche^1^, M. Niewerth^1^, C. Sengler^1^, D. Windschall^2^, T. Kallinich^3,4^, F. Dressler^5^, R. Trauzeddel^6^, R. W. Holl^7,8^, I. Foeldvari^9^, N. Brueck^10^, S. Temming^3^, A. Hospach^11^, P. Warschburger^12^, R. Berendes^13^, G. Erbis^14^, J. B. Kuemmerle-Deschner^14^, F. Weller-Heinemann^15^, J.-P. Haas^16^, A. Mueller-Stierlin^17^, A. Mutter^18^, T. Meissner^19^, H. Baumeister^18^, K. Minden^1,3^

#### ^1^Epidemiology and Health Services Research, German Rheumatism Research Centre, Berlin; ^2^Clinic of Pediatric and Adolescent Rheumatology, St. Josef-Stift Sendenhorst, Sendenhorst; ^3^Department of Pediatric Respiratory Medicine, Immunology and Critical Care Medicine, Charité – Universitätsmedizin Berlin; ^4^Pathophysiology of Rheumatic Inflammation, German Rheumatism Research Centre, Berlin; ^5^Department of Paediatric Pneumology, Allergology and Neonatology, Hannover Medical School, Hannover; ^6^Department of Pediatrics, Helios Klinik Berlin-Buch, Berlin; ^7^Institute for Epidemiology and Medical Biometry, University of Ulm, Ulm, ^8^German Center for Diabetes Research, Munich; ^9^Hamburg Centre for Pediatric and Adolescent Rheumatology, Schön Klinik Hamburg Eilbek, Hamburg; ^10^Department of Pediatrics, Medizinische Fakultät Carl Gustav Carus, Dresden; ^11^Department of Pediatrics, Olgahospital, Klinikum Stuttgart, Stuttgart; ^12^Department of Psychology, University of Potsdam, Potsdam; ^13^Pediatric Rheumatology, Children's Hospital St. Marien, Landshut; ^14^Department of Pediatrics, University Hospital Tuebingen, Tuebingen; ^15^Division of Pediatric Rheumatology, Prof. Hess Children's Hospital, Bremen; ^16^German Center for Pediatric and Adolescent Rheumatology, Garmisch-Partenkirchen; ^17^Department of Psychiatry and Psychotherapy II; ^18^Department of Clinical Psychology and Psychotherapy, University of Ulm, Ulm; ^19^Department of General Pediatrics, Neonatology and Pediatric Cardiology, Heinrich-Heine-University Dusseldorf, Dusseldorf, Germany

##### **Correspondence:** F. Milatz

*Pediatric Rheumatology 2023*, **21(Suppl 2):**PT015

**Introduction:** Previous studies have shown that growing up with rheumatic conditions can fuel dissatisfaction and psychological distress, which in turn affects disease self-management and treatment adherence [1].

**Objectives:** Primary objective of this study was to estimate the prevalence of anxiety and depression symptoms in adolescents and young adults (AYA) with juvenile idiopathic arthritis (JIA) and to identify correlates of conspicuous screening.

**Methods:** Initiated as part of the COACH multicentre observational study, outpatients aged 12 to 21 years participating in the National Paediatric Rheumatological Database (NPRD) were prospectively screened for mental health using the Patient Health Questionnaire-9 (PHQ-9) and the Generalised Anxiety Disorder Scale-7 (GAD-7). Scores ≥7 on either instrument were considered conspicuous. In order to consider potential seasonal and pandemic influences in the study period, correlates were identified by regression analysis adjusted for screening date.

**Results:** Data from 1,150 adolescents with JIA (mean age 15.6 ± 2.2 years; mean disease duration 7.2 ± 4.9 years, 69% female, 43% oligoarthritis, 26% polyarthritis) from 48 paediatric rheumatology centres were analysed. Overall, 32.7% (n=316) of AYA had conspicuous screening, of whom 30.4% (n=96) reported suicidal or self-harm thoughts (nearly 12% of all screened participants). One in three conspicuously screened patients was receiving psychotherapeutic (25.9%) and/or psychopharmacological (13.6%) treatment. Adolescents with conspicuous screening were older (15.8 vs. 15.2 years; p<.0001), more often female (81% vs. 64%; p<.0001) and overweight (25% vs. 17%; p=0.006). They had higher disease activity (physician global assessment on NRS 0-10; 1.7 vs. 1.2; p<.0001), more functional limitations (CHAQ; 0.44 vs. 0.14; <.0001), and rated their health status worse (NRS 0-10; 3.5 vs. 1.8; p<.0001) than inconspicuous screened patients. Females (OR 2.33 [CI 1.53-3.56]; p<.0001), older patients (OR 1.09 [CI 1.01-1.18]; p=0.026), patients with more functional limitations (OR 3.36 [CI 1.98-5.72]; p<.0001), and patients with worse subjective health status (OR 1.17 [CI 1.07-1.27]; p<.0001) were more likely to be conspicuously screened. Regular sports participation was associated with a lower likelihood of conspicuous screening (OR 0.69 [CI 0.49-0.98]; p=0.039).

**Conclusion:** A large-scale outpatient screening of adolescents and young adults with juvenile idiopathic arthritis in Germany uncovered a high prevalence of anxiety and depression symptoms., as well as psychological undertreatment. The need for routine screening to identify this issue and provide optional support to affected patients has become evident.

The COACH project was funded by the BMBF (01GL1740F) and the NPRD was financially supported by the German Children's Rheumatism Foundation, Abbvie, Chugai, GSK, Novartis and Pfizer to date.

**Patient Consent**

Not applicable (there are no patient data)

**Disclosure of Interest**

None declared

**Reference**

1. Fair DC, et al. Depression and anxiety in patients with juvenile idiopathic arthritis: current insights and impact on quality of life, a systematic review. Open Access Rheumatol Res Rev 2019;11:237–52.

## PT016 JADAS10- and CJADAS10-based disease activity states for psoriatic arthritis, enthesitis-related arthritis, and RF+ polyarthritis

### S. M. Orsi^1^, M. Burrone^1^, A. I. Rebollo Gimenez^2^, F. Ridella^1^, S. Rosina^2^, L. Carlini^3^, I. Rumba-Rozenfelde^4^, N. Shafaie^5^, T. Avcin^6^, P. Quartier^7,8^, N. Ruperto^9^, A. Ravelli^10^, M. Gattorno^2^, A. Consolaro^1,2^ on behalf of the Paediatric Rheumatology International Trials Organisation (PRINTO)

#### ^1^Dipartimento di Neuroscienze, Riabilitazione, Oftalmologia, Genetica e Scienze Materno-Infantili (DiNOGMI), Università degli Studi di Genova; ^2^UOC Reumatologia e Malattie Autoinfiammatorie; ^3^UOC Servizio di Sperimentazioni Cliniche Pediatriche PRINTO, IRCCS Istituto Giannina Gaslini, Genoa, Italy; ^4^Pediatric Rheumatology, University of Latvia and University Children Hospital, Riga, Latvia; ^5^, Department of Pediatrics and Rheumatology, Shariati Hospital, Rheumatology Research Center, Teheran, Iran, Islamic Republic Of; ^6^Department of Allergology, Rheumatology and Clinical Immunology, University Children's Hospital, University Medical Center Ljubljana, Ljubljana, Slovenia; ^7^Assistance Publique-Hopitaux de Paris, Necker-Enfants Malades University Hospital; ^8^Université Paris-Cité, Paris, France; ^9^UOC Servizio di Sperimentazioni Cliniche Pediatriche, PRINTO, ^10^Direzione Scientifica, IRCCS Istituto Giannina Gaslini, Genoa, Italy

##### **Correspondence:** S. M. Orsi

*Pediatric Rheumatology 2023*, **21(Suppl 2):**PT016

**Introduction:** The measurement of disease activity level is of central importance in the evaluation of the patient with juvenile idiopathic arthritis (JIA). The Juvenile Arthritis Disease Activity Score (JADAS) and its clinical version excluding the acute phase reactant (cJADAS) were validated and are increasingly used in clinical trials and routine practice. To allow score interpretation, cutoffs have been developed and subsequently validated for JADAS10 and cJADAS10 in RF- polyarthritis and oligoarthritis. The need to have cutoffs for other arthritis categories is increasingly evident.

**Objectives:** To validate the JADAS10 and cJADAS10 disease activity state cutoffs to separate the states of inactive disease (ID), minimal disease activity (MiDA), moderate disease activity (MoDA), and high disease activity (HDA) in children with RF+ polyarthritis, PsA and ERA.

**Methods:** JIA children from 49 countries included in the EPidemiology, treatment and Outcome of Childhood Arthritis (EPOCA) study were considered. For PsA and ERA, the decision on whether to use oligoarthritis or polyarthritis cutoffs was based on the most frequent pattern of joint involvement at visit. Discriminative ability was assessed by calculating and comparing in each disease activity state the level of pain (0-10 VAS) and functional ability impairment (measured with the Juvenile Arthritis Functional Ability Score, JAFS, 0-45) and the frequency of patients satisfied with current disease state, starting a new medication, and having morning stiffness. Comparisons of quantitative variables among groups were made by Kruskal-Wallis test; Dunn’s test was used to assess differences between pairs of patient groups. Percentage data were compared by chi-squared test or Fisher’s exact test. Bonferroni’s adjustment was applied to explore post-hoc differences between pairs of patient groups.

**Results:** 309 children with PsA, 959 with ERA, and 382 with RF+ polyarthritis were included. 88% children with PsA and 91% with ERA had oligoarticular disease, at study visit; therefore, oligoarthritis cutoffs were used for these categories.

The level of pain and functional ability was significantly different among the JADAS-based disease states, with pain and JAFS scores increasing progressively from ID to HDA (Kruskal-Wallis test p <0.001). The percentage of patients who prescribed a new medication, with morning stiffness < 15 minutes, and who were satisfied with current disease state were different in the JADAS-based disease states. Paired comparison showed significant discrimination for most comparisons.

**Conclusion:** Both the JADAS10 and cJADAS10 cutoffs to define disease activity states validated for oligoarthritis and polyarthritis showed good discriminative validity in RF+ polyarthritis, PsA and ERA. These results preliminarily indicate that available cutoffs might be used for these categories of JIA.

**Patient Consent**

Not applicable (there are no patient data)

**Disclosure of Interest**

None declared

## PT017 Determining moderate and high disease activity cut-off values for Parent Juvenile Arthritis Disease Activity Score (PARJADAS)

### F. Ridella^1^, R. Naddei^2^, M. Burrone^1^, S. M. Orsi^1^, V. Panaviene^3,4,5^, C. Pruunsild^5,6^, G. Chedeville^5,7^, S. Vilaiyuk^5,8^, N. Ruperto^5,9^, M. Gattorno^10^, A. Ravelli^11^, A. Consolaro^1,10^

#### ^1^Department of Neurosciences, Rehabilitation, Ophthalmology, Genetics and Maternal-Infantile Sciences, University of Genova , Genova; ^2^Rheumatology Department, Federico II University Hospital, Naples, Italy; ^3^Children’s Hospital, Affiliate of Vilnius University Hospital Santaros Clinic; ^4^Vilnius University, Clinic of Children’s Diseases, Vilnius, Lithuania; ^5^Paediatric Rheumatology International Trials Organisation (PRINTO), Genova, Italy; ^6^Tartu University Hospital, Children' s Clinic, Department of General Pediatrics and Neurology, Tartu, Estonia; ^7^The Montreal Children's Hospital, Rheumatology Division, Montréal (QC), Canada, Montréal, Canada; ^8^Mahidol University Faculty of Medicine, Ramathibodi Hospital Department of Pediatrics, Bangkok, Thailand; ^9^Istituto Giannina Gaslini, Clinical Trial Department; ^10^Istituto Giannina Gaslini, Rheumatology and Autoinflammatory diseases Unit; ^11^Istituto Giannina Gaslini, Scientific Direction, Istituto Giannina Gaslini, Genova, Italy

##### **Correspondence:** F. Ridella

*Pediatric Rheumatology 2023*, **21(Suppl 2):**PT017

**Introduction:** The parent Juvenile Arthritis Disease Activity Score (parJADAS)^1^ is a disease activity tool developed specifically for remote monitoring of patients with Juvenile Idiopathic Arthritis (JIA). It relies solely on the patient or parent perception of disease activity. Previous studies have demonstrated the excellent discriminant ability^1^, reliability^2^ and good criterion validity^2^ of parJADAS.

**Objectives:** This study aimed to establish the cut-off values of moderate disease activity (MoDA) and high disease activity (HDA) for the parJADAS in patients with JIA.

**Methods:** The parJADAS (score range 0-40) is derived by summing four values: 1) parent’s assessment of disease activity on a 21-numbered circle 0-10 Visual Analogue Scale (VAS); 2) assessment of pain intensity on a 21-numbered circle 0-10 VAS; 3) proxy assessment of active joints, up to a maximum of 10 joints; 4) assessment of morning stiffness (MS) on a Likert scale, ranging from no MS (0 points) to > 2 hours of MS (10 points). The study dataset is composed of 8772 patients with JIA, which were enrolled in the the multinational study “Epidemiology, Treatment and Outcome of Childhood Arthritis” (the EPOCA study). At each visit, the attending physician subjectively categorized the patients into one of the following disease activity states: inactive disease (ID), minimal disease activity (MDA), moderate disease activity (MoDA), or high disease activity (HDA). The following methods were implemented to establish the cut-off values for parJADAS: 1) Mapping: the 25^th^ percentile value of the parJADAS in patients classified as MoDA or HDA, respectively; 2) Youden Index: Youden Index (J) identifies the maximum potential effectiveness of the biomarker through the Receiver Operating Characteristic (ROC) curve analysis. The proposed cut-offs for both MoDA and HDA were subsequently calculated as the mean of the two obtained values with the aforementioned methods, for each disease activity state.

**Results:** Data from a total of 2,146 patients with moderate disease activity (MoDA) and 343 patients with high disease activity (HDA) were analyzed. For MoDA, the obtained cut-off value using the mapping approach was 6.5. The ROC curve analysis demonstrated an area under the curve (AUC) of 0.829 (95% CI 0.821 to 0.837). The ROC analysis further yielded a Youden Index of 5, indicating a sensitivity of 82.5% and specificity of 68.9% for the identified cut-off value. Regarding HDA, the mapping approach yielded a cut-off value of 13.25. The ROC curve analysis showed an AUC of 0.870 (95% CI 0.863 to 0.877). The Youden Index was calculated as 9.5, indicating a sensitivity of 85.7% and specificity of 71.4% for the determined cut-off value. The proposed cut-off values for moderate disease activity (MoDA) and high disease activity (HDA) were determined as the mean of the values obtained from both the mapping approach and the Youden index approach for each disease state. For MoDA, the mean value was calculated to be 5.75, while for HDA, the mean value was determined as 11.375. These values were rounded to ≥5.5 for MoDA and ≥11 for HDA, representing the clinically relevant cut-offs for distinguishing between different disease activity states in Juvenile Idiopathic Arthritis (JIA) patients.

**Conclusion:** Tentative cut-off values for classifying the states of MDA and HDA using parJADAS were calculated. The obtained values will be tested in the validation analysis. Once validated, the cut-offs are ideally suited to identify subjects at risk of disease flare when remotely monitored with the parJADAS; subsequently, these are the patients to be soon referred to their Pediatric Rheumatology Center for a comprehensive clinical examination.

**Patient Consent**

Not applicable (there are no patient data)

**Disclosure of Interest**

None declared

**References**

1. Ridella et al. Annals of the Rheumatic Diseases, volume 78, Issue Suppl 2. Abstract “Discriminant ability of the parent version of the Juvenile Arthritis Disease Activity Score in a large multination cohort of patients with Juvenile Idiopathic Arthritis“
2. van Dijkhuizen et al. Arthritis Care Res (Hoboken) 2023 Feb;75(2):391-400. doi: 10.1002/acr.24855. „Validity and Reliability of Four Parent/Patient–Reported Outcome Measures for Juvenile Idiopathic Arthritis Remote Monitoring“

## PT018 Medical care for children with Juvenile idiopathic arthritis in Ukraine at the present stage

### L. F. Bohmat^1^, Y. Y. Boyko ^2^, O. A. Oshlianska^3^, O. B. Synoverska^4^, N. S. Shevchenko^1^

#### ^1^Association of Pediatric Rheumatologists of Ukraine, Kharkiv; ^2^Association of Pediatric Rheumatologists of Ukraine, Lviv; ^3^Association of Pediatric Rheumatologists of Ukraine, Kyiv; ^4^Association of Pediatric Rheumatologists of Ukraine, Ivano-Frankivsk, Ukraine

##### **Correspondence:** N. S. Shevchenko

*Pediatric Rheumatology 2023*, **21(Suppl 2):**PT018

**Introduction:** Since September 2014, a medical reform has been initiated in Ukraine. At the beginning of 2022, the primary and secondary stages were reformed. Against this background, there are no clear data on the prevalence and incidence of rheumatic diseases in children, including juvenile idiopathic arthritis (JIA).The main feature of 2022 in Ukraine was the massive migration of the population. More than 10 million people became internally displaced persons: about 6.5 million became internally displaced persons, about 4 million more left the territory of Ukraine. The huge migration of displaced persons has placed a high strain on existing medical facilities in the western regions of the country and created additional barriers to accessing medical care.

**Objectives:** The purpose of the study was to clarify the state of specialized care for children with JIA.

**Methods:** An analysis was made of the work of specialists - pediatric rheumatologists of Ukraine on changes in the contingent of children with JIA for 2022.

**Results:** According to the data of 2017-2019, the number of children with JIA in Ukraine reached 2800 people. The current Ukrainian protocol for the treatment of JIA was introduced on July 22, 2012 ^1^, and was based on the American Protocol for the Treatment of JIA - ACR 2011. In Ukraine, the concept of treat to target ^2^ has been introduced, according to which basic biological anti-inflammatory therapy (BBT) is prescribed in case of ineffectiveness of traditional treatment for 3-6 months. Unresolved issues include an insufficient range of biological agents registered for pediatric patients. The second problem is the lack of recommendations on the appointment of BBT as a starting or monotherapy for certain types of JIA (systemic arthritis, polyarthritis, spondylarthritis with active enthesitis).

A sufficiently large number of children with JIA remain under the supervision of Ukrainian pediatric rheumatologists, despite the massive departure of patients from Ukraine to other countries. Among the total number of patients in Ukraine, children who fell ill in 2022 range from 15 to 20%. State funding for the treatment of children with JIA continues. Children receive BBT, including about 40% of patients who fall ill in 2022. Average number of patients with JIA who receive BBT in Ukraine is 30%. The main problem was the interruption of BBT for more than three months due to the closure of the medical institutions or the child's move to another place of residence. Activation of the inflammatory process occurred in 83.3% of such cases.

**Conclusion:** Despite the difficult situation in the health care system of Ukraine, which was the result of incomplete reform and military aggression, the work of pediatric rheumatologists in Ukraine continued, and the provision of biological therapy to patients with JIA is maintained. At the same time, it is necessary to improve the work of pediatric rheumatologists with a wider implementation of international recommendations.

**Patient Consent**

Not applicable (there are no patient data)

**Disclosure of Interest**

None declared

**References**

1. https://zakon.rada.gov.ua/rada/show/v0832282-12#top
2. Ravelli A, et al.,doi: 10.1136/annrheumdis-2018-213030.

## PT019 Two clusters of juvenile psoriatic arthritis identified at initial presentation to paediatric rheumatology in a nationwide UK cohort

### S. J. W. Shoop-Worrall^1^, C. Ciurtin^2^, G. Cleary^3^, F. McErlane^4^, L. Coates^5^, N. Geifman^6^, K. L. Hyrich^1^ on behalf of CAPS PIs

#### ^1^Centre for Epidemiology Versus Arthritis, The University of Manchester, Manchester; ^2^Centre for Adolescent Rheumatology, Division of Medicine, University College London, London; ^3^Alder Hey Children’s Hospital, Liverpool; ^4^Royal Victoria Infirmary, Newcastle upon Tyne; ^5^Oxford Psoriatic Arthritis Centre, University of Oxford, Oxford; ^6^School of Health Sciences, Faculty of Health and Medical Sciences, University of Surrey, Surrey, United Kingdom

##### **Correspondence:** S. J. W. Shoop-Worrall

*Pediatric Rheumatology 2023*, **21(Suppl 2):**PT019

**Introduction:** The heterogenous presentation and variable clinical response of juvenile psoriatic arthritis (JPsA) to disease-modifying therapies suggests undiscovered subgroups within this disease. Nevertheless, JPsA is often studied under the umbrella of juvenile idiopathic arthritis, with few studies interrogating JPsA separately. To improve stratified treatment of this rare disease, such subgroups must be uncovered.

**Objectives:** To identify novel, phenotypically consistent subgroups of children and young people (CYP) with JPsA at the point of first contact with paediatric rheumatology.

**Methods:** CYP were initially selected if enrolled between January 2001 and December 2019 to the Childhood Arthritis Prospective Study, a UK, multicentre, prospective inception cohort of JIA. Those who had a physician’s diagnosis of JPsA at any time point through the 10-year follow-up were included, to allow for onset of psoriatic signs after initial diagnosis. At initial presentation to paediatric rheumatology, clinical features within the ILAR classification criteria for JPsA were collected: an active joint count and the presence or absence of psoriasis, dactylitis and nail abnormalities. Latent class analysis used these features to identify clusters of disease. Between one and ten clusters were tested and an optimal model selected based on statistical fit.

**Results:** Of 1,753 CYP with JIA recruited to CAPS within the study period, a total of 161 CYP had ever had a diagnosis of JPsA (n=97 diagnosed as JPsA at initial presentation to paediatric rheumatology). The majority were female (61%), of white ethnicity (94%) and the median age at initial presentation was 10 years (IQR 6, 13).

The optimal latent class model identified two clusters of JPsA. An oligoarticular cluster (90%, median active joint count (IQR): 2 (1,5)) had a higher proportion of CYP affected by psoriasis (Cluster 1: 29%, Cluster 2: 14%). A polyarticular cluster (10%, median active joint count (IQR): 20 (16, 27)) had a higher proportion with nail abnormalities (Cluster 1: 8%, Cluster 2: 27%). There were similar proportions of dactylitis among the clusters (Cluster 1: 18%, Cluster 2: 15%).

**Conclusion:** This study identifies two clusters of JPsA at initial presentation to paediatric rheumatology with differences in key features used to classify this disease. Such subgroups may have different experiences of disease, and future analysis will explore characteristics, alongside disease impact and response to therapy for these groups.

**Patient Consent**

Yes, I received consent

**Disclosure of Interest**

None declared

## PT020 Hyperproduction of immunoglobulin G in patients with Oligo-/Poly-Juvenile idiopathic arthritis

### M. Trevisan, I. Caiello, P. Palomba, S. Cascioli, A. Aquilani, G. Tarantino, R. Nicolai, M. Pardeo, C. Bracaglia, S. Magni Manzoni, R. Carsetti, E. Marasco, F. De Benedetti

#### Bambino Gesu Children Hospital, Rome, Italy

##### **Correspondence:** M. Trevisan

*Pediatric Rheumatology 2023*, **21(Suppl 2):**PT020

**Introduction:** Juvenile idiopathic arthritis (JIA) is an umbrella term encompassing different forms of arthritis that may have different pathological mechanisms. Hypergammaglobulinemia, reflecting B cell hyperactivity, has been described in JIA. Genetic studies of associations with HLA allele, immunologic studies focusing on B lymphocytes, and the presence of specific clinical features support the hypothesis that early-onset JIA (presenting before age 6 years) is a distinct clinical entity, regardless of the number of joints involved.

**Objectives:** We aim to study serum levels of immunoglobulins and in vitro production of immunoglobulins following B cell activation in patients with oligo-/poly-JIA (o-/p-JIA) diagnosed before the age of 6 years.

**Methods:** We enrolled patients with o-/p-JIA (n=34), aged matched healthy donors (HD) (n=19) and patients with systemic JIA (sJIA) (n=10), all younger than 6 years. Serum levels of IgG, IgM and IgA were retrieved from medical records of OPBG. B cells of o-/p-JIA patients and HD were activated in vitro for 7 days with CpG, concentration of secreted immunoglobulins was measured by ELISA.

**Results:** We focused on patients age 0 to 6 years, as B cell subsets and immunoglobulins levels of children younger than 6 years are still immature and differ significantly from children older than 6 years and young adults. Immunoglobulins levels were obtained at disease onset and before the initiation of any treatment for JIA. The median age in years was: HD 1.99 [IQR 1.37, 3.38], sJIA 2.76 [2.07, 3.64], o-/p-JIA 2.54 [1.66, 4.03] (ANOVA, p=0.28). Of the o-/p-JIA patients, 25 (73.5%) had oligo-JIA and 9 (26.5%) had poly-JIA; 28 (82.4%) were ANA positive and 14 (41.2%) developed uveitis. Levels of serum IgG were significantly higher in o-/p-JIA compared to HD and sJIA (median [IQR] mg/dL: HD 8.16 [6.67, 9.50], sJIA 7.40 [7.12, 12.27], o-/p-JIA 11.49 [10.10, 14.70], ANOVA p<0.001). We observed no differences between the 3 groups for IgM and IgA. When we divided o-/p-JIA patients according to number of joints involved and ANA positivity we did not observed significant differences for levels of IgG, IgM or IgA. PBMCs of 20 o-/p-JIA patients and 92 HD were stimulated in vitro for 7 days with the TLR9 agonist CpG, that induces B cell differentiation into plasmablasts. We observed significantly higher levels of IgG in the supernatant of stimulated cells of JIA patients than of HD (median [IQR]: HD 0.42 [0.12, 3.24], o-/p-JIA 2.75 [0.52, 9.05], t-test p=0.024). We observed no differences in the production of IgM and IgA.

**Conclusion:** Our data support the hypothesis of a hyperactive B cell compartment in patients with o-/p-JIA younger than 6 years. We observed higher levels of serum IgG and higher in vitro production of IgG by stimulated B cells in patients with o-/p-JIA than HD and sJIA patients. Further work is required to dissect the molecular pathways leading to B cell activation in o-/p-JIA.

**Patient Consent**

Yes, I received consent

**Disclosure of Interest**

None declared

**References**

1. Marasco, A&R, 2018
2. Marasco, EJI, 2017
3. Wouters, Clin Exp Rheumatol, 2002

## PT021 Defining predictive transcriptomic signatures for T cell subsets involved in Juvenile Idiopathic Arthritis (JIA)

### V. Alexiou^1,2^, A. Callaghan^3^, M. Kartawinata^1,2^, E. Ralph^1,2,4^, B. Jebson^1,2^, M. G. L. Wilkinson^1,2^, A. Radziszewska^2,5^, H. Peckham^2,5^, E. C. Rosser^2,5^, C. Curtin^2,5^, E. Vigorito^3^, L. R. Wedderburn^1,2,4^ on behalf of the CLUSTER Consortium

#### ^1^Infection, Immunity, and Inflammation Programme, UCL Great Ormond Street Institute of Child Health; ^2^Centre for Adolescent Rheumatology Versus Arthritis at UCL University College London Hospital (UCLH) and Great Ormond Street Hospital (GOSH), London; ^3^MRC Biostatistics Unit, University of Cambridge, Cambridge; ^4^NIHR Great Ormond Street Hospital Biomedical Research Centre; ^5^Division of Medicine, University College London, London, United Kingdom

##### **Correspondence:** V. Alexiou

*Pediatric Rheumatology 2023*, **21(Suppl 2):**PT021

**Introduction:** The role of regulatory T cells (Tregs) is well established in juvenile idiopathic arthritis (JIA) pathogenesis. JIA studies have also shown that a pro-inflammatory subset of Th17 cells, expressing the marker CD161, is enriched in the synovial fluid (SF) of JIA patients and correlates with disease activity. Establishing transcriptomic signatures which faithfully infer the proportions of potentially pathogenic cells in JIA may be a valuable prognostic tool.

**Objectives:** To explore whether RNA sequencing (RNAseq) data can be used to predict the cellular proportions of Treg and CD161+ CD4+ T cell populations measured by flow cytometry in healthy and JIA peripheral blood mononuclear cells (PBMC) and SFMC of JIA patients.

**Methods:** Matched flow-cytometry and RNAseq data from PBMC (n = 158) and SFMC (n = 43) of JIA patients and PBMC (n = 39) from healthy controls were used for the modelling analysis. Specifically, Treg (CD4+CD25hiCD127lo) and CD161+ CD4+ proportions (% of CD4+ T cells) were measured by flow cytometry and CD4+ sorted RNAseq data of the same samples were used to predict the flow cytometry measured proportions. To provide biologically informed predictors, recent literature on Treg and CD161+ CD4+ T cell from heathy and disease states was reviewed to generate the cell-specific lists of genes which are differentially expressed compared to non-Treg and CD161- CD4+ subsets respectively. RNAseq data from sorted Tregs (CD4+CD25hiCD127lo) and conventional T cells (Tconvs) (CD4+CD25loCD127+) were generated from SFMC of JIA patients (n = 8) and PBMC from healthy controls (n = 3) and analysed to validate the literature-based Treg gene list across tissue and disease state. For the modelling analysis, the elastic net method was used for variable selection from the cell-specific gene lists to predict the flow-cytometry-measured proportions using the RNAseq data separately in SFMC and PBMC samples. The performance of the model was assessed by repeated cross-validation.

**Results:** A 42 Treg gene list generated from the literature review was able to cluster the sorted Tregs separately from the Tconvs regardless of tissue and disease origin. Preliminary analysis showed that the model is more predictive in SFMC samples (R = 0.77) than in PBMC samples (R = 0.37).

**Conclusion:** Biologically informed signatures are valuable in discriminating cell populations of interest at the transcriptional level regardless of cell origin and disease state. The preliminary data show that the predictive capacity of conventional modelling methods is limited, so alternative algorithms are being explored.

**Patient Consent**

Yes, I received consent

**Disclosure of Interest**

V. Alexiou: None declared, A. Callaghan: None declared, M. Kartawinata: None declared, E. Ralph: None declared, B. Jebson: None declared, M. Wilkinson: None declared, A. Radziszewska: None declared, H. Peckham: None declared, E. Rosser: None declared, C. Curtin: None declared, E. Vigorito: None declared, L. Wedderburn Grant / Research Support with: LRW Declares in kind contributions to CLUSTER by AbbVie, GSK, UCB, Sobi and Pfizer inc and non renumerated collaborations with Lilly and Novartis.

## PT022 Characterisation of cellular adaptations to the inflammatory environment in active Juvenile idiopathic arthritis using spectral flow cytometry

### M. H. Attrill^1^, D. Shinko^1^, V. Alexiou^2,3^, M. Kartawinata^2,3^, C. study group^2,3,4^, J. study group ^2,3,4^, L. R. Wedderburn^2,3,4^, A. M. Pesenacker^1^

#### ^1^Institute of Immunity and Transplantation, University College London; ^2^UCL Great Ormond Street Institute of Child Health; ^3^Centre for Adolescent Rheumatology versus Arthritis at UCL UCLH and GOSH; ^4^NIHR Biomedical research centre at GOSH, London, United Kingdom

##### **Correspondence:** M. H. Attrill

*Pediatric Rheumatology 2023*, **21(Suppl 2):**PT022

**Introduction:** Children and young people with Juvenile Idiopathic Arthritis (JIA) experience repeated inflammatory flares of their joints leading to pain, fatigue, reduced quality of life and ultimately joint destruction and disability. While therapeutics have improved, treatment still fails for 30-50% of patients.

**Objectives:** To unlock potential insight into JIA disease pathogenesis and novel treatment targets, we need to understand the cellular composition and specialised phenotype of synovial fluid mononuclear cells (SFMC) from inflamed joints, so that ultimately targeted treatment can restore the immunoregulatory balance.

**Methods:** Using 5-laser full spectrum flow cytometry, we designed and verified a 37-parameter panel to assess the cellular composition and phenotype between JIA SFMCs (n=18), JIA peripheral blood (PBMCs, n=52), as well as healthy control PBMCs (n=18). The panel identified monocyte, B, NK, and dendritic cell subsets in addition to CD4- effector (Teff), CD4+ conventional (Tconv) and regulatory T cell (Treg) phenotype. Unbiased high dimensional analysis was performed after pre-processing in FlowJo using the R package Spectre, allowing raw data integration, clustering through FlowSOM/Phenograph, dimensionality reduction via UMAP and quantitative statistical analysis and visualisation.

**Results:** We identified highly upregulated and JIA SF exclusive phenotypes across cell types by unbiased clustering. The Fc receptor CD16 was absent from SF monocyte and NK cell subsets, suggesting a lack of antibody-mediated action, which could help explain failure of antibody-mediated treatments. Two unusual subsets of dendritic cells were exclusively found in the inflamed joint, highly expressing 4-1BB, CD71, CD39 and Ki67, revealing maturation, proliferation, and adaptation to the inflammatory environment. SF T cells, including Tregs, and B cells highly expressed multiple activation markers and unique cell surface receptor combination exclusive to cells from the inflamed joint. A significant cluster of CD4- Teff expressed the transcription factor FoxP3, which has been indicated to promote changes in metabolic behaviour of CD8+ Teff within tumours to allow survival in an environment of restricted metabolic resources. Moreover, the transferrin receptor CD71, which is critical to increase iron metabolism and enables cellular survival and proliferation, was upregulated on the majority of cells from the inflamed joint.

**Conclusion:** High dimensional flow cytometry revealed phenotypic and metabolic adaptations of SF cell subsets and SF cells had unique cell surface receptor combinations, which may be used to target cells in the inflammatory environment in the future while sparing circulating cells. These findings may facilitate more targeted therapies to achieve treatment success for more children and young people with JIA and minimalizing systemic side effects.

**Patient Consent**

Not applicable (there are no patient data)

**Disclosure of Interest**

None declared

## PT023 Identification of variables associated with poor prognosis in patients with COVID-related multisystem inflammatory syndrome (MIS-C). Results from thehyperped-COVID registry

### R. Caorsi^1^, F. Bovis^2^, C. Speziani^1^, A. Consolaro^1,3^, C. Bracaglia^4^, F. Minoia^5^, M. Cattalini^6^, P. Brogan^7^, C. Wouters^8^, A. Taddio^9^, F. Candotti^10^, I. Meyts^11^, F. De Benedetti^4^, N. Ruperto^12^, M. Gattorno^1^ on behalf of the Steering Committee for HyperPED-COVID registry for RITA-ERN, ISSAID, PRES, ESID, PRINTO

#### ^1^Reumatologia e malattie auotinfiammatorie, IRCCS Istituto Giannina Gaslini; ^2^Department of health sciences (DISSAL); ^3^Dipartimento di Neuroscienze, Riabilitazione, Oftalmologia, Genetica e Scienze Materno-Infantili (DiNOGMI), University of Genova, Genova; ^4^Division of Rheumatology, IRCCS Ospedale Pediatrico Bambino Gesù, Rome; ^5^Pediatric Rheumatology, Fondazione IRCCS Ca' Granda-Ospedale Maggiore Policlinico, Milano; ^6^Pediatric clinic, University of Brescia and ASST Spedali Civili di Brescia, Brescia, Italy; ^7^UCL GOSH Institute of Child Health, London, United Kingdom; ^8^Pediatric Rheumatology and Immune-inflammatory diseases, Leuven - Universitair ziekenhuis, Leuven, Belgium; ^9^Institute for Maternal and Child Health IRCCS "Burlo Garofolo" and Univeristy of Trieste, trieste, Italy; ^10^Division of Immunology and Allergy, Laboratory of Inherited Immune Disorders, CHUV, Lausanne, Switzerland; ^11^Department of Microbiology and Immunology, University of Leuven, Leuven, Belgium; ^12^trial center, IRCCS Istituto Giannina Gaslini, Genova, Italy

##### **Correspondence:** R. Caorsi

*Pediatric Rheumatology 2023*, **21(Suppl 2):**PT023

**Introduction:** COVID-related Multisystem Inflammatory Syndrome in children (MIS-C) is a serious inflammatory condition characterized by systemic inflammation with multiorgan failure, that can occur in children and young adults after COVID-19 infection. By now, different publications have provided evidence about the clinical manifestations, possible treatment and outcome of this condition, without analyzing the parameters associated to a increased risk of sequelae or death.

**Objectives:** To evaluate the presence of variables associated with a poor prognosis (sequelae or death) in patients with MIS-C, taking into consideration the age at disease presentation and the geographical origin of the patients.

**Methods:** Data regarding clinical manifestations, laboratory features, response to treatment and outcome of patients with MIS-C patients were collected by the Hyper-Ped registry, available online on PRINTO and ESID websites. Univariable and multivariable logistic regression analyses were assessed to identify possible factors associated with a poor prognosis in patients with MIS-C.

**Results:** 1009 patients, were analysed.

432 patients were form Western Europe (Belgium, Denmark, Italy, Portugal, Spain), 334 were from Eastern Europe (Bulgaria, Croatia, Czech Republic, Greece, Latvia, Russian Federation, Serbia, Slovenia, Turkey, Ukraine), 168 from Latin America (Argentina, Brazil, Mexico) and 75 from Other countries (Bangladesh, Egypt, India, Singapore).

The final model identified 9 variables associated to the outcome: patients coming from Other countries (OR 7.06) presented a higher risk for the outcome rather those from Western Europe. The presence of increased neutrophil count (OR 1.04), lymphopenia (OR 3.38), myocarditis (OR 3.74), coronary aneurism (OR 14.1), renal hypertension (OR 10.07) and palpable purpura (OR 7.85) is correlated with the outcome, as the need of respiratory support (OR 2.4). Prophylactic treatment with heparin (OR 0.22) is protective for the outcome.

**Conclusion:** From the analysis of the data of the registry, it appears that patients coming from less resourced country presented a poorer prognosis than the others. It’s reasonable that these results depends on the reduced possibility of access to intensive cares and to biological drugs. The presence of cardiac involvement, hypertension and hematological abnormalities are associated to a poorer outcome.

**Patient Consent**

Not applicable (there are no patient data)

**Disclosure of Interest**

None declared

## PT024 Humoral and cellular immunogenicity, efficacy, and safety of COVID-19 MRNA vaccination in patients with pediatric rheumatic diseases: a prospective cohort study

### M. Hamad Saied^1,2^, J. W van Straalen^1^, M. jansen^1^, N. Wulffraat^1^, J. swart^1^, S. Roock^1^, G. Joode-Smink^1^, E. Van Nieuwenhove^1^, B. Vastert^3^

#### ^1^Pediatric Immunology and Rheumatology, University Medical Center Utrecht, Utrecht, Netherlands; ^2^Pediatric and Rheumatology, Technion Faculty of MedicineTechnion Faculty of Medicine, haifa, Israel; ^3^University Medical Center Utrecht, Utrecht, Netherlands

##### **Correspondence:** M. Hamad Saied

*Pediatric Rheumatology 2023*, **21(Suppl 2):**PT024

**Introduction:** Children with pediatric autoimmune inflammatory rheumatic disease are at higher risk for infections due to both their underlying disease and immunosuppressive treatments. At the same time, vaccinations pose a great challenge in this patient group due to lower immunogenicity secondary to their underlying disease, their immunosuppressive treatment and the concern about disease flares following vaccination

Severe COVID-19 illness can occur in these patients , especially among those with underlying comorbidities

**Objectives:** To evaluate immunogenicity, efficacy and safety of COVID-19 vaccination in patients with pediatric autoimmune inflammatory rheumatic disease (pedAIIRD).

**Methods:** A prospective cohort study was performed at the pediatric rheumatology department of the Wilhelmina Children’s Hospital in Utrecht. Vaccination dates, COVID-19 cases and vaccine-related adverse events (AEs) were registered for all pedAIIRD patients during regular control visits from March, 2021 – August, 2022. SARS-CoV-2 IgG antibody levels and T-cell responses were measured and clinical and drug therapy data were collected. Rate of COVID-19 disease was compared between vaccinated and unvaccinated patients.

**Results:** A total of 157 patients were included, of whom the majority had JIA (88%) and were vaccinated against COVID-19 (87%). Geometric mean concentrations (GMCs) of post-vaccine antibody levels against SARS-CoV-2 were above the threshold for positivity in patients who did or did not use biological agents at the time of vaccination, although the biological users demonstrated significantly lower antibody levels (adjusted GMC ratio: 0.38, 95% CI: 0.21 – 0.70). T-cell responses were adequate in all but two patients (8%), who received combination therapy of methotrexate with tocilizumab or methotrexate with adalimumab at the time of vaccination. The adjusted rate of reported COVID-19 was significantly lower for fully vaccinated patients compared to non-vaccinated patients (HR: 0.53, 95% CI: 0.29 – 0.97). JIA disease activity scores were not significantly different after vaccination, no serious AEs were reported.

**Conclusion:** COVID-19 mRNA vaccines were immunogenic (both cellular and humoral), effective and safe in a large cohort of pedAIIRD patients despite their use of immunosuppressive medication.

**Trial registration identifying number:** This study was approved by the UMCU Medical Ethical Committee (METC number 22-643). T-cell responses were measured in patients who gave informed consent in the Pharmachild study (METC number 11-499c).

**Patient Consent**

Yes, I received consent

**Disclosure of Interest**

None declared

## PT025 The comprehensive assessment OF SARS-COV-2 immunity in children with Juvenile idiopathic arthritis utilizing T-cell response measured by interferon-gamma release assay

### K. Kapten^1^, K. Orczyk^2^, E. Smolewska^1^

#### ^1^Department of Pediatric Cardiology and Rheumatology; ^2^Department of Pediatric Infectious Diseases, Medical University of Lodz, Łódź, Poland

##### **Correspondence:** K. Kapten

*Pediatric Rheumatology 2023*, **21(Suppl 2):**PT025

**Introduction:** The SARS-CoV-2 pandemic revealed numerous limitations to the widely known and accessible methods of evaluating individual immunity, necessitating new diagnostic tools, such as a novel T-cell-based Interferon-γ Release Assay (IGRA), that is proposed to assess cellular immunity, in addition to standard serological testing.

**Objectives:** This study aimed to evaluate and compare humoral and cellular responses to the recent coronavirus infection in the cohort of children suffering from juvenile idiopathic arthritis (JIA). In addition, the authors aimed to assess the immunity provided by the SARS-CoV-2 vaccine, particularly, in individuals undergoing different treatment regimes.

**Methods:** This prospective study included 55 pediatric patients between the ages 2-16 with different types and various stages of JIA. The cohort group included children with both positive and negative history of Covid-19 infection, as well as vaccinated and unvaccinated individuals. The cellular response to the virus was measured using a specific quantitative IGRA in whole blood, while subsequently, the anti-SARS-CoV-2 ELISA test was performed, quantifying the levels of IgA, IgM, and IgG antibodies in serum.

**Results:** The research found a significant correlation between the cellular response to SARS-CoV-2 measured by the IGRA test and the levels of IgA (p<0.00003, R=0.537) and IgG (p<0.0001, R=0.668) antibodies, along with the detection of antibodies with the nucleocapsid protein (IgG NCP) (p<0,003, R=0,0399). Notably, no correlation with IgM antibodies titers in serum was observed. Regardless of the number of boosters administered, all vaccinated individuals developed a T-cell response to the mRNA vaccine (p=0.0016). The humoral response, specifically the levels of IgG antibodies in patients receiving biological treatment (adalimumab, baricitinib, etanercept, or tocilizumab) were significantly lower compared to the group not undergoing such therapy (p=0.0369).

**Conclusion:** The strong affinity of SARS-CoV-2 with mucosal membranes can be associated with a correlation between cellular response and IgA antibody levels. Hence, the sole testing of humoral immunity, by measuring IgG and IgM antibodies may no longer remain the most precise diagnostic method. IGRA proves to be a highly accurate tool in the assessment of individual immunity both after viral infection and vaccination. Additionally, the suppression of humoral response observed in biological treatment protocols may impact individual susceptibility to the virus, possibly proving to be a protective factor against the SARS-CoV-2 infection.

**Patient Consent**

Not applicable (there are no patient data)

**Disclosure of Interest**

None declared

## PT026 Generation of high affinity memory B cells against spike protein of SARS-COV2 virus in patients with Juvenile idiopathic arthritis

### A. Aquilani, E. Piano Mortari, I. Caiello, G. Tarantino, R. Nicolai, S. Magni Manzoni, F. De Benedetti, R. Carsetti, E. Marasco

#### Bambino Gesu Children Hospital, Rome, Italy

##### **Correspondence:** E. Marasco

*Pediatric Rheumatology 2023*, **21(Suppl 2):**PT026

**Introduction:** Vaccination-induced protection against infections relies on the capacity of generating specific antibodies and memory B cells (MBCs), which can rapidly respond to successive infections. TNF inhibitors (TNFi), used to treat patients with juvenile idiopathic arthritis (JIA), do not only dampen inflammation, but they have been shown to also inhibit the germinal center response and, thus, to decrease the frequency of MBCs.

**Objectives:** We aim to study the frequency of SARS-COV2 spike-specific MBCs following vaccination or infection in patients with JIA.

**Methods:** B cell phenotype and spike specific B cells was assessed on PBMCs by flow cytometry as previously described (Terreri, 2022).

**Results:** We enrolled 36 patients with a diagnosis of JIA according to the ILAR criteria. Twenty-two (61%) patients had a diagnosis of oligoarticular JIA, 12 (33%) patients had polyarticular JIA, 1 psoriatic JIA, and 1 ERA. Nine patients were on treatment with MTX, 27 were on TNFi (13 adalimumab, 11 etanercept, 1 golimumab, 2 infliximab). Twenty-seven patients (75%) were vaccinated with 3 doses of SARS-CoV2 mRNA vaccine, 5 patients (14%) received 2 doses of SARS-CoV2 mRNA, 4 patients (11%) refused vaccination and contracted COVD19. All vaccinated patients received a vaccine against the original strain (BNT162b2 or mRNA-1273). In total, 23 patients contracted COVID19, all patients showed only mild symptoms. We enrolled 11 healthy healthcare workers without history of autoimmunity(HD) who underwent mRNA vaccination for SARS-CoV2.

The frequency of high affinity MBCs specific for SARS-CoV2 was higher in HD than in JIA patients (mean (SD) of MBCs: HD 0.46 (0.21), JIA 0.30 (0.22), p=0.03). All high affinity MBCs specific for SARS-CoV2 were switched (mean frequency of high affinity IgM- MBCs were 91.4% for HD and 89.3% for JIA). A large fraction of high affinity MBCs specific for SARS-CoV2 reacted against the omicron strain, although the frequency was lower in JIA patients than HD (mean (SD) of MBCs: HD 63.37% (21.52), JIA 44.61% (25.42), p= 0.032). The frequency of low affinity MBCs specific for SARS-CoV2 was similar between the two groups (mean (SD) of MBCs: HD 0.63% (0.53), JIA 0.84% (0.91), p=0.458). Patients on TNFi showed a lower frequency of high affinity spike-specific MBCs than patients who were on MTX only (mean (SD) of MBCs: MTX 0.42% (0.25), TNFi 0.25% (0.20), p=0.04). Interestingly, patients who refused to get vaccinated and contracted COVID19, did not produce high affinity MBCs specific for SARS-CoV2 compared to patients who were vaccinated (mean (SD) of MBCs: JIA non vaccinated 0.05% (0.05), JIA vaccinated 0.33% (0.21), p=0.017).

**Conclusion:** Our data showed that mRNA vaccination was able to induce the generation of high affinity MBCs against SARS-CoV2 spike protein, although at a lower frequency than HD. COVID19 infection alone was not able to induce high affinity MBCs against SARS-CoV2, highlighting the importance of vaccination for patients with JIA.

**Patient Consent**

Yes, I received consent

**Disclosure of Interest**

None declared

**Reference**

1. Terreri, Cell Host Microbe, 2022

## PT027 Analysis of B cell subsets and B cell cytokines in pediatric sjogren’s syndrome

### C. Bracaglia, R. Nicolai, A. Boni, I. Caiello, F. De Benedetti, E. Marasco

#### IRCCS Bambino Gesù Children's Hospital, Rome, Italy

##### **Correspondence:** E. Marasco

*Pediatric Rheumatology 2023*, **21(Suppl 2):**PT027

**Introduction:** Pediatric Sjögren's syndrome (pSS) is a rare disorder that is often diagnosed late due to the lack of validated diagnostic criteria and validated biomarkers. The pathogenesis is largely unknown, but there is evidence of involvement of both the innate and adaptive branch of the immune system. Immunological overactivity is central in the pathogenesis of pSS. Several studies showed the presence of B cells abnormalities in patients with SS with an expansion of naïve B cells and a decrease in the frequency of memory B cells.

**Objectives:** We set out to investigate the distribution of B cell subsets and B cell cytokines in patients with pSS at disease onset and at follow up visits.

**Methods:** A monocentric retrospective cohort study was conducted on 17 patients with pSS enrolled at the department of Rheumatology of Bambino Gesù Children's Hospital. Serum levels of BAFF were analyzed by ELISA. B-cell phenotype was assessed on peripheral blood mononuclear cells (PBMCs) by flow cytometry.

Systemic disease activity was evaluated by ESSDAI (EULAR Sjogren's syndrome disease activity index) and Clinical-ESSDAI score (Clin-ESSDAI), according to 2020 EULAR recommendations; active disease was defined by ClinESSDAI ≥1 and remission by ClinESSDAI=0. As controls we selected age-matched people with no diagnosis of pSS or any other systemic autoimmune disease.

**Results:** Serum levels of BAFF were significantly higher in patients with pSS than the control group (p<0,05). We correlated levels of biomarkers with clinical and laboratory parameters: we observed a positive correlation between hypergammaglobulinemia and BAFF (rho=+0,80).

Analysis of B-cell subsets at disease onset revealed the expansion of a population of atypical memory B cells (p=0,00049) and reduction in lgM memory B cells (p=0,0034) compared to the control group. We, then, compared the distribution of B cell subpopulations at disease onset with samples obtained at follow-up for each patient: no significant differences were observed. We also investigated the distribution of Tfh cells in pateints with pSS and we observed a significant expansion of CXCR5-PD1hi Tfh.

**Conclusion:** Patients with pSS showed high levels of BAFF at disease onset. Alteration in B cell subsets are present in patients with pSS compared to controls, with an expansion of atypical memory B cells and Tfh. The B cell abnormalities are not affected by current treatment. Our data confirm an hyperactivation of B cells and B cell-derived chemokines and in patients with pSS and provide evidence for their development as biomarkers and to develop new therapeutic strategies amied at controlling B cell hyperactivation in pediatric patients with SS.

**Patient Consent**

Yes, I received consent

**Disclosure of Interest**

None declared

## PT028 Screening multisystem inflammatory syndrome in children: accuracy of SickKids screening pathway compared to ACR algorithm

### G. Mastrangelo^1^, P. Tsoukas ^1,2^, T. Mizzi ^3^, B. D. Gamulka^4^, A. Xu^1,2^, A. H. H. Cheng^1,2^, R. S. M. Yeung^1,2,5^ on behalf of SickKids MIS-C Working Group

#### ^1^Division of Rheumatology, Department of Paediatrics; ^2^Cell Biology Program; ^3^Department of Emergency Medicine; ^4^Department of Paediatrics, The Hospital for Sick Children; ^5^Department of Immunology and Institute of Medical Science, University of Toronto, Toronto, Canada

##### **Correspondence:** G. Mastrangelo

*Pediatric Rheumatology 2023*, **21(Suppl 2):**PT028

**Introduction:** Multisystem Inflammatory Syndrome in Children (MIS-C), also known as Pediatric Inflammatory Multisystem Syndrome temporally associated with COVID-19 (PIMS), is one of the most serious complications associated with COVID-19. The clinical features of MIS-C are not unique and are commonly seen in childhood febrile conditions. Thus, there is a need to identify those febrile children with MIS-C to enable early diagnosis and treatment. In response to the health care emergency, a multidisciplinary team from The Hospital for Sick Children (SickKids) of Toronto developed a preliminary screening pathway for the evaluation of possible MIS-C.

**Objectives:** The primary objective is to determine if the SickKids screening pathway is sensitive and specific to identify patients with MIS-C from other febrile children with suspected but not confirmed diagnosis of MIS-C. The secondary objective of this study is to determine how the ACR MIS-C algorithm performs compared to the SickKids screening pathway in differentiating children with MIS-C from the febrile controls.

**Methods:** Retrospective case-controlled, cross-sectional study including children who have been assessed at SickKids with suspected or confirmed MIS-C from March 2020 to March 2022. The MIS-C group included all children meeting the most permissive case definition as per international MIS-C and PIMS definitions and adjudicated by our multi-disciplinary MIS-C working group; the febrile control group consisted of all patients with a history of three or more days of fever who were suspected of MIS-C and qualified for the SickKids MIS-C screening pathway, but did not fulfill criteria for MIS-C after adjudication by our multi-disciplinary group. SickKids and ACR pathways were retrospectively applied to both groups. The diagnosis result obtained using the pathways was compared with the final clinical diagnosis made using the WHO definition criteria, used as the gold standard. From the contingency table, sensitivity and specificity have been calculated along with a 95% confidence interval.

**Results:** 402 children (241 with MIS-C and 161 febrile controls) were included in the study. The median age was 4.18 years (IQR: 1.9 to 9.0) and 237 (60%) were male. For the SickKids screening pathway, the sensitivity was 62% (95%CI, 54.2% to 70.4%), and specificity was 91% (95%CI, 86.9% to 94.2%). The positive predictive value (PPV) was 79% and the negative predictive value (NPV) was 81%. Overall, the balanced accuracy was equal to 76%. The ACR screening pathway had 74% sensitivity (95%CI, 67.3% to 81.4%), and 99% specificity (95%CI, 98.1% to 100%), with PPV of 98% and NPV of 87%. The balanced accuracy was 87%.

**Conclusion:** The SickKids MIS-C screening pathway has a high specificity, but a low sensitivity and accuracy in capturing children with MIS-C at the onset of the disease. Overall, the ACR algorithm performs better at differentiating children with MIS-C from febrile controls.

**Patient Consent**

Yes, I received consent

**Disclosure of Interest**

None declared

## PT029 Convergence and divergence in KD and MIS-C: the results of the covasaky survey

### M. V. Mastrolia^1^, M. Martini^2^, G. Memmini^3^, G. Ferrara^4^, R. Bernardini^5^, D. Peroni^6^, R. Consolini^7^, R. Agostiniani^8^, S. Falorni^9^, C. Azzari^10^, G. B. Calabri^11^, G. Indolfi^12^, M. L'Erario^13^, S. Trapani^14^, G. Simonini^1^

#### ^1^Rheumatology Unit, ERN ReCONNET center, Meyer Children's Hospital IRCCS, NEUROFARBA Department, University of Florence, Firenze; ^2^Paediatric Unit, San Donato Hospital, Arezzo; ^3^Division of Neonatology and Paediatrics, Apuane Hospital, Massa Carrara, AUSL Toscana Nord Ovest, Pisa; ^4^Paediatric Unit, Santa Maria Annunziata Hospital, Bagno a Ripoli, AUSL Toscana Centro, Firenze; ^5^Paediatric Unit, San Giuseppe Hospital, Empoli; ^6^Department of Clinical and Experimental Medicine, Section of Paediatrics, University of Pisa; ^7^Section of Clinical and Laboratory Immunology, Department of Clinical and Experimental Medicine, University of Pisa, Pisa; ^8^Paediatric Unit, San Jacopo Hospital, Pistoia; ^9^Paediatric Unit, Misericordia Hospital, Grosseto; ^10^Paediatric Immunology Unit, Department of Health Sciences, Meyer Children's University Hospital IRCCS, Firenze, Italy; ^11^Cardiologic Unit, Meyer Children's University Hospital IRCCS; ^12^Paediatric Unit, Meyer Children's Hospital IRCCS, NEUROFARBA Department, University of Florence; ^13^Paediatric Intensive Care Unit, Meyer Children's University Hospital IRCCS; ^14^Paediatric Unit, Meyer Children's University Hospital IRCCS, Department of Health Sciences, University of Florence, Firenze, Italy

##### **Correspondence:** M. V. Mastrolia

*Pediatric Rheumatology 2023*, **21(Suppl 2):**PT029

**Introduction:** MIS-C and KD share many clinical features although these conditions may diverge in terms of epidemiologic and biochemical parameters.

**Objectives:** To compare two cohorts of KD and MIS-C patients.

**Methods:** A prospective collection of demographics, clinical findings, treatment and outcome data of KD and MIS-C patients between November 1^st^ 2020 to February 28^th^ 2023 belonging to 8 Pediatric units was conducted. Additionally, for each patient a blood sample at disease onset (T0), and at recovery (T1) was collected to assess type 1 interferon score (IFN score) and serum cytochines: CXCL9, CXCL10, IL18, IFN gamma, IL6, IL1b.

**Results:** 87 patients (43 KD, 44 MIS-C) were included in the study. Age at onset was significantly higher in MIS-C compared to KD patients (mean 31±23 vs 94±50 months, p<0.001). No differences in gender and ethnicity were observed. Considering clinical manifestations, extremities abnormalities and mucosal involvement were more often recorded in KD patients (p=0.027; p<0.001). Neurological findings, in particular headache (p=0.002) and meningism (p=0.035), were significantly more frequent in MIS-C patients (p=0.002). Irritability was a typical clinical feature in the KD cohort (p<0.001).Gastrointestinal symptoms were more frequently observed in MIS-C group (p=0.013) as well as respiratory involvement (p=0.019) and splenomegaly (p=0.026). Conversely, gallbladder hydrops (p=0.01) and lymphadenopathy (p=0.07) more often occurred in KD patients.The frequency of cardiac manifestations overall was significantly higher in MIS-C group (p<0.001), although coronary arteries aneurisms were more frequently observed in KD patients (p=0.012). The length of stay was longer in MIS-C patients (mean 8.6±4.1 vs 14.5±6.9 days, p=0.002) and PICU admission was significantly higher (p<0.001) together with the need for inotropes (p=0.048). Steroids and intravenous anakinra were more frequently administered in MIS-C cohort (p<0.001). ASA antiplatelet treatment was more often adopted in KD (p<0.001) while anticoagulant prophylaxis with heparin in MIS-C patients (p<0.001). The lymphocyte count was significantly lower in MIS-C group (p<0.001), while the CRP value was higher compared to those of KD patients (p=0.001). IFN score and IL18 values at T0 were higher in KD patients (p=0.019; p=0.016), CXCL10 at T0 was more elevated in the MIS-C cohort (p=0.007). No other significant differences in the biomarkers values were detected at the different timepoints (T0-T1 interval median 6 days IQR 8.25) between the two groups. A significant decrease of CXCL9 and CXCL10 values from T0 to T1 in both KD (p=0.02; p=0.015) and MIS-C patients (p=0.004; p<0.001) was reported.

**Conclusion:** The epidemiological, clinical, and biochemical differences of our KD e MIS-C cohorts confirm previous reported data. The role of the tested biomarkers in distinguishing KD from MIS-C is not completely clarified even if a larger prospectively assessment could be useful to monitor the response to treatment throughout the disease course.

**Patient Consent**

Yes, I received consent

**Disclosure of Interest**

None declared

## PT030 Co-expression and interaction of Co-inhibitory tigit and co-stimulatory CD226 altering treg fitness in active JIA

### A. M. Pesenacker^1^, D. Shinko^1^, M. H. Attrill^1^, C.-S. W. Huang^1^, S. Narendra Babu^1^, V. Alexiou^2,3^, M. Kartawinata^2,3^, L. R. Wedderburn^2,3,4^, C. study group^2,3,4^, J. study group^2,3,4^, C. Hinze^1^

#### ^1^Div Infection and Immunity, UCL Institute of Immunity and Transplantation; ^2^UCL Great Ormond Street Institute of Child Health; ^3^Centre for Adolescent Rheumatology Versus Arthritis at UCL UCLH and GOSH; ^4^NIHR Biomedical Research Centre at GOSH, London, United Kingdom

##### **Correspondence:** A. M. Pesenacker

*Pediatric Rheumatology 2023*, **21(Suppl 2):**PT030

**Introduction:** FOXP3+CD4+ regulatory T cells (Tregs) are crucial to maintain tolerance, however, in Juvenile Idiopathic Arthritis (JIA) Tregs are not fit for service, and thus fail to control inflammation leading to repeated inflammatory flares of joints, pain, loss of mobility, and ultimately joint destruction and disability.

**Objectives:** To understand why Tregs from the site of inflammation and blood of children and young people with active JIA fail to suppress inflammation, we investigated the co-inhibitory co-receptor TIGIT and co-stimulatory CD226, which may be utilised by Tregs to interpret cues from the microenvironment in inflamed joints to change action accordingly.

**Methods:** We assessed co-receptor and their ligand expression in JIA synovial fluid mononuclear cells (SFMCs) and peripheral blood MC (PBMCs) from active and inactive JIA patients and healthy controls at protein level utilising full spectrum flow cytometry, and mRNA level by nanoString. Moreover, TIGIT-CD226 interaction and ligand-dependency was assessed by luciferase-based NanoBiT® system and confocal microscopy in stably transduced cell lines.

**Results:** We showed by flow cytometry and mRNA expression that TIGIT and CD226 are mostly co-expressed in SF Tregs (>60% double positive). This double positive Treg subset could also be detected in PB of children with clinically active JIA but was near absent in healthy control and children with clinically inactive JIA. Interestingly, overall TIGIT and CD226 expression was increased in SF CD4- effector and CD4+ conventional T cells compared with blood, however only a small proportion of effector cells co-expressed both receptors. The co-receptor ligands CD155 (PVR) and CD112 (Nectin2) were also increased by mRNA (total SFMC) and protein on SF antigen presenting cell subsets. Since TIGIT and CD226 have proposed opposite cellular effects, we investigated co-receptor interaction, and established that TIGIT and CD226 interact in the absence of ligand, but this interaction increased 4.7-fold with CD155 availability. This ligand-dependent enhancement could be inhibited when using blocking antibodies for TIGIT-CD155 or CD226-CD155 binding (3.5/3.8-fold decrease from ligand alone). Moreover, we saw clear co-localisation of TIGIT and CD155, CD226 and CD155, as well as TIGIT, CD226 and CD155 triple co-localisation using confocal microscopy.

**Conclusion:** We have demonstrated that TIGIT and CD226 directly interact in cis when co-expressed. With TIGIT-CD226 co-expression mainly seen in JIA SF Tregs, this receptor-receptor interaction will likely alter Treg signalling, behaviour and functional outcome at the site of inflammation, while in healthy control blood Tregs the direct effects of TIGIT or CD226 predominate. Breaking this interaction could serve as a novel treatment target in JIA without interfering with TIGIT- or CD226-dependent functions when expressed as single co-receptor.

**Patient Consent**

Not applicable (there are no patient data)

**Disclosure of Interest**

None declared

## PT031 Artificial intelligence model 'morgaf' for diagnosing paediatric systemic lupus erythematosus: do we need US?

### E. Aliyev^1,2^, Y. Uğur^3^, Y. Bilginer^1^, S. Ozen^1^

#### ^1^Pediatric Rheumatology, Hacettepe University School of Medicine; ^2^SEMBA Science, Education & Informatics Ltd. Co.; ^3^Keytech Electronic & Software Ltd. Co., Ankara, Türkiye

##### **Correspondence:** E. Aliyev

*Pediatric Rheumatology 2023*, **21(Suppl 2):**PT031

**Introduction:** Systemic Lupus Erythematosus (SLE) is a chronic, autoimmune-based disease characterized by multiple organ involvement and autoantibodies. Although it generally has typical findings, it is a complex disease to diagnose, and early diagnosis and treatment are essential in the disease process. The diagnosis of the disease is based on SLICC 2012 criteria. The artificial Intelligence (AI) model was tested in diagnosing the condition. Our study aims to develop a computer-based AI model to assist clinicians in diagnosis.

**Objectives:** Fifty SLE patients followed up in Hacettepe University Pediatric Rheumatology Outpatient Clinics, and 50 healthy individuals similar to them in terms of age and gender were included in the study. Data sets, including clinical and laboratory findings of the individuals at the time of diagnosis, were given as input to the AI model. Python® software language and Tensorflow® AI library were preferred for model development.

**Methods:** The concept of 'Neural Networks' (NN) is becoming increasingly widespread in AI studies. Morgaf used the 'Recurrent Neural Network' (RNN) model preferred. The most important difference of the RNN from other networks is that while training the model while processing the inputs at the moment Tn, it also remembers the inputs at the moment Tn-1 (Tn is any time unit). With this, error rates on the network are lower than in other models. The laboratory and clinical results of the patients were digitized as 1, -1 (abnormal), and 0 (normal range). One hundred data were increased 15 times to 1500 with repetitions, and 80% were separated as training data and 20% as validation data. Morgaf is designed as a model that builds an NN with 1024 neurons. The homogeneity of the variables was tested by covariance analysis (p=0.792). Regression analysis was performed for both homogeneously distributed groups and tested with Levene (p=0,147). During the model training, the error rate in the early training phase of the model was approximately 0.5. In the later generations of the model, the error decreased to 0.028. This error rate was considered sufficient, and the training phase was terminated. Training continued autonomously in the latent phase. The study was planned to be single blind. Thirty case data (data utterly foreign to the model) were given to Morgaf and simultaneously to expert pediatric rheumatologists, and their interpretations were compared. Prediction success was evaluated by performing regression analyses between both groups.

**Results:** Morgaf accepted 70% and above as a definitive diagnosis of SLE, averaging 93% (78-98%). It accepted 10% and below for a completely healthy case; the average was 1% (0-3%). To recognize diseases requiring follow-up, he set himself a range of 10%-70% and estimated the mean to be 33% (15-47%). There was no difference between Morgaf's estimates and actual diagnoses (p=0.297). He was 100% successful in recognizing lupus disease. This rate was the same as the diagnostic acuity of clinicians. Morgaf (93.3%) gave clearer recommendations for non-lupus cases than clinicians (70%) (p=0.034). Regression analysis showed that Morgaf (y=0.9264xi) was more successful in non-selective prediction than clinicians (y=0.7322xi).

**Conclusion:** Our study is the first in the literature to develop and test an AI model as a diagnostic tool for pediatric SLE. The developed AI model was at least as successful as pediatric rheumatologists in differentiating SLE patients from healthy controls and non-SLE patients. With this study, we have shown that Morgaf can help clinicians in the diagnostic and differential diagnosis. The model can also be a diagnostic tool for non-specialist clinicians. The plan to further develop Morgaf to reach a clinician-like diagnostic acuity (with visual and auditory data processing integration) is one of the project's future goals.

**Patient Consent**

Not applicable (there are no patient data)

**Disclosure of Interest**

None declared

**References**

1. Yones, S. A., Annett, A., Stoll, P., Diamanti, K., Holmfeldt, L., Barrenäs, C. F., Meadows, J. R. S., & Komorowski, J. (2022). Interpretable machine learning identifies paediatric Systemic Lupus Erythematosus subtypes based on gene expression data. *Scientific reports*, *12*(1), 7433.
2. Wu, J., Yang, W., & Li, H. (2022). An artificial neural network model based on autophagy-related genes in childhood systemic lupus erythematosus. *Hereditas*, *159*(1), 34.

## PT032 Association of type I interferon subtypes and disease activity in Juvenile dermatomyositis

### T. R. Moreau^1,2^, V. Bondet^2^, J. Ramos^3^, C. Albert-Vega^2^, F. Rahal^2^, N. Ouldali^3^, M.-L. Frémond^4^, P. Quartier^4^, C. Bodemer^4^, A. Isapof^5^, A. Welfringer^4^, C. Vinit^4^, C. Dumaine^3^, B. Fournier^4^, C. Gitiaux^4^, I. Melki^3^, B. Bader-Meunier^4^, D. Duffy^2^, M. Rodero^1^

#### ^1^Université Paris Cité, CNRS; ^2^Institut PAsteur; ^3^Hôpital Robert Debré; ^4^Hôpital Necker; ^5^Hôpital Trousseau, Paris, France

##### **Correspondence:** B. Bader-Meunier

*Pediatric Rheumatology 2023*, **21(Suppl 2):**PT032

**Introduction:** Juvenile Dermatomyositis (JDM) is a rare inflammatory autoimmune paediatric disease associated with a type I interferon (IFN) signature. Questions remain about the relative contribution of interferon alpha subtypes or interferon beta to the pathophysiology of the disease. Here, we addressed this question by quantifying IFN beta, IFNalpha 2c, and all 13 IFNalpha subtypes (Pan-A) in a longitudinal cohort of JDM patients.

**Methods:** This longitudinal study included 187 samples from 52 JDM patients. Autoantibody expression and clinical measures of disease activity were evaluated: Childhood Myositis Assessment Scale (CMAS), Manual Muscle Testing in 8 muscles (MMT-8) for muscle testing; skin score of Disease Activity Score (DAS) for the skin. Pan-A, IFN alpha2c and IFN beta were measured in the plasma of patients by digital ELISA (Simoa) using homebrew assays. Correlation between IFN-I titers and clinical scores was evaluated using Spearman’s test followed by a Bonferroni correction.

**Results:** All assays correlated with all clinical scores for the entire cohort. After stratification based on MSA expression (MDA5+: n=12, NXP2+ n=11, TIF1g+ n=10, None n=19), we observed in MDA5+ patients, a very strong association between the two IFN alpha assays and all three clinical scores. IFNbeta levels were also strongly correlated with all three scores in MDA5+ patients, but to a lesser extent compared to the two IFNbeta tests. The only significant correlation in the other 3 groups was the correlation between IFN beta titer and CMAS in a TIF1gamma positive patient.

**Conclusion:** The association between JDM disease activity and plasmatic IFN-I levels has been described in previous studies. Our results suggest that this effect was mainly driven by MDA5 positive patients challenging the role of IFN-I subtypes in the pathophysiology of MDA5 negative JDM patients. Ongoing work will try to identify the specific immune pathways that lead to dysregulated IFN-I responses in MDA5+ patients using functional immune assays.

**Trial registration identifying number:**

**Patient Consent**

Not applicable (there are no patient data)

**Disclosure of Interest**

None declared

## PT033 Presence of Anti-IFN-ALPHA2 autoantibodies in a french multicentric cohort of pediatric patients with SLE

### S. Khaldi-Plassart^1^, S. Assant^2^, P. Bastard^3^, S. Pons^2^, K. Saker^2^, I. Kone-Paut^4^, I. Melki^5^, H. Reumaux^6^, S. Decramer^7^, C. Kevorkian-Verguet ^8^, H. Flodrops^9^, P. Consortium^10^, E. Chopin^11^, B. Bader-Meunier^12^, A. Belot^1^

#### ^1^National Referee Centre for Rheumatic and AutoImmune and Systemic diseases in children, Hospices Civils de Lyon; ^2^Joint Research Unit Civils Hospices of Lyon-bioMérieux, HCL, Lyon; ^3^Laboratory of Human Genetics of Infectious Diseases, Necker Branch, INSERM U1163, Necker Hospital for Sick Children; ^4^Pediatric Rheumatology and CEREMAIA, Bicêtre Hospital APHP, University of Paris Sud Saclay, le KREMLIN Bicetre; ^5^General Paediatrics, Infectious Disease and Internal Medicine Department, Hôpital Robert Debré, AP-HP, Paris; ^6^Paediatric Rheumatology Department, Centre Hospitalier Régional Universitaire de Lille., Lille; ^7^Pediatric Nephrology and Internal Medecine, CHU de Toulouse., Toulouse; ^8^Pediatric Immunology, Albert Michallon Hospital, Grenoble; ^9^Service de Pédiatrie, Groupe Hospitalier Sud Réunion, CHU de La Réunion, Saint-Pierre, France; ^10^JIRcohorte, Lausanne, Switzerland; ^11^Centre de Biotechnologie Cellulaire Et Biothèque, Hospices Civils de Lyon, Lyon; ^12^Department for Immunology, Hematology and Pediatric Rheumatology, Necker Hospital, APHP, Institut IMAGINE,, Paris, France

##### **Correspondence:** S. Khaldi-Plassart

*Pediatric Rheumatology 2023*, **21(Suppl 2):**PT033

**Introduction:** Systemic lupus erythematosus (SLE) is a heterogeneous autoimmune disease affecting multiple organ systems, characterized by increased expression of type I interferon (IFN)- regulated genes (50%–75% of patients). The presence of anti-IFN-α autoantibodies (AAbs) has been reported in adult patients with SLE (5% to 27%). Interestingly, neutralizing Aabs anti-IFN-α has been reported and are associated with reduced IFN-αserum levels, a reduced disease activity and a history of severe infectious episodes (COVID-19 pneumonia or episodes of cutaneous herpes zoster) illustrating the clinical impact of these AAbs. To the best of our knowledge, no studies have been conducted thus far that report the presence of anti-interferon autoantibodies (Aabs) in pediatric patients with systemic lupus erythematosus (SLE).

**Objectives:** The aims of this study are to evaluate the presence of anti-IFN-alpha Aabs and their neutralizing capacities, and to identify clinico-biological manifestations associated with the presence of anti-IFN-alpha Aabs in a French multicentric cohort of pediatric patients with SLE.

**Methods:** We tested 143 biological samples from pediatric patients with SLE from the French national lupus biobank and performed IFN-α2 dosage (ELISA) and functionally tested the inhibitory capacities of each sera sample on IFN activity. We collected clinical data retrospectively from the PEDIALUP registry of the JIRcohort database.

**Results:** The presence of anti-IFN-α AAbs was detected by ELISA in 26 (18,2%) of the 143 patients. Neutralizing anti-IFN-α2 Aabs were present in 11 patients (7,7 %) and neutralizing anti-IFN-α2, anti-IFN-β or anti-IFN-ω Aabs in 18 patients (12,6%) with pediatric-onset SLE. Clinical data collected from 83 patients showed there was no signifcant difference in terms of gender or median age or disease duration between patients with or without ELISA-detectable anti-IFN-α Aabs. We compared 3 groups: ELISA-negative anti-IFN-α AAbs group (anti-IFN-αAabs <100 ng/ml)(N=64) ; ELISA-positive but non-neutralizing anti-IFN-α Aabs group (N=10) ; ELISA-positive and neutralizing anti-IFN-α Aabs group (N=9). We observed a trend of less disease activity in patients with ELISA positive and neutralizing anti-IFN-α Aabs group (no significant) and no significant difference related to infectious event. .

**Conclusion:** This is the first report on the presence of anti-IFN-α2 Aabs in patients with pediatric onset SLE (French multicentric cohort) (N=143). We observed high prevalence of neutralizing and non-neutralizing anti-IFN-αAAbs in pediatric onset SLE patients compared to pediatric controls. Further studies are required to address their place in the management and follow-up of jSLE in the future.

**Patient Consent**

Yes, I received consent

**Disclosure of Interest**

None declared

**References**

1. Zhang Q et al. *Inborn errors of type I IFN immunity in patients with life-threatening COVID-19*. Science. 23 oct 2020;370(6515):eabd4570.
2. Mathian A et al. *Lower disease activity but higher risk of severe COVID-19 and herpes zoster in patients with systemic lupus erythematosus with pre-existing autoantibodies neutralising IFN-α*. Ann Rheum Dis. 16 août 2022;annrheumdis-2022-222549.
3. Bradford HF et al. *Inactive disease in patients with lupus is linked to autoantibodies to type I interferons that normalize blood IFNα and B cell subsets*. Cell Rep Med. janv 2023;4(1):100894.

## PT034 High-dimensional interrogation of the adult-onset and childhood-onset Systemic Lupus Erythematosus (SLE) immunomes

### K. Nay Yaung^1,2^, J. G. Yeo^1,2,3^, A. Hui Nee Law^2,4^, M. Wasser^1^, T. Arkachaisri^2,3^, K. L. Teh^3^, Y. X. Book^5^, J. Thumboo^2,4^, A. Hsiu Ling Low^2,4^, S. L. Poh^1^, A. Jin Mei Lim^1^, S. Albani^1,2,3^

#### ^1^Translational Immunology Institute, SingHealth Duke-NUS Academic Medical Centre; ^2^Duke-NUS Medical School; ^3^Rheumatology and Immunology Service, KK Women’s and Children’s Hospital; ^4^Department of Rheumatology & Immunology, Singapore General Hospital; ^5^KK Women's and Children's Hospital, Singapore, Singapore

##### **Correspondence:** K. Nay Yaung

*Pediatric Rheumatology 2023*, **21(Suppl 2):**PT034

**Introduction:** Systemic lupus erythematosus (SLE) is a complex, systemic autoimmune disease with unpredictable disease course. It interferes with the balance between immunity and regulation, resulting in immune system dysfunction. Capturing its inherent heterogeneity can yield mechanistic insights to improve theragnostic strategies, especially when only two targeted biologics have been FDA-approved for SLE treatment despite numerous clinical trials. Furthermore, adult and paediatric SLE patients are usually studied in silos, with treatment for the latter group often extrapolated from adult data with no direct evidence of benefit.

**Objectives:** We aim to resolve the immunopathogenic complexity of SLE with high-dimensional mass cytometry (CyTOF) to study and dissect the adult-onset and childhood-onset SLE immunomes. We hypothesise that immune dysregulation in SLE is driven dually by impaired immunoregulation and lowered activation threshold for immune effector cells.

**Methods:** Peripheral blood mononuclear cells (PBMCs) were studied with a 43-marker CyTOF panel. The adult group comprised 26 SLE (timepoints: 40, median age: 39.5 years) and 23 age-matched healthy subjects. The paediatric group included 30 patients (53, 14 years) and 17 healthy. Quality check, batch-effect correction, cell clustering, annotation and visualisation after t-distributed Stochastic Neighbour Embedding (t-SNE) dimensional reduction were performed using our Extended Polydimensional Immunome Characterisation (EPIC) pipeline.^1^ Cell frequencies are presented as a percentage of CD45^+^PBMCs with median and interquartile range. Statistical significance was defined as p<0.05 (Kruskal-Wallis test, Dunn’s multiple comparisons).

**Results:** Multiple derangements were noted in all major immune lineages but mainly in CD4^+^ T cells. Interestingly, there were no significant differences in the naïve (CD45RA^+^) and memory (CD45RA^-^) natural regulatory T cells (T_regs_) (CD3^+^CD4^+^CD25^+^FoxP3^+^CTLA4^+^TIGIT^+^PD1^+/-^) between SLE and healthy except for an increase in memory T_regs_ in the paediatric population (SLE versus healthy: 1.16 [0.79 to 1.92]%; 0.48 [0.32 to 0.80]%, p<0.0001). Instead, memory T_reg_-like cells (CD3^+^CD4^+^CD45RA^-^CD25^-^FoxP3^+^CTLA^+^) were enriched in SLE in both adults (2.33 [1.50-3.31]; 1.16 [0.94-1.71], p<0.001) and children (2.75 [1.90-4.36]; 1.28 [0.83-1.78], p<0.0001). Concurrently, CTLA4-expressing naïve and memory non-T_reg_ CD4^+^ T effector cells were significantly reduced in adult-onset SLE compared to healthy (naïve: 1.61 [0.85-2.63]; 4.65 [3.2-7.18], p<0.0001; memory: 0.54 [0.34-0.79]; 1.46 [1.00-2.06], p<0.0001).

**Conclusion:** With a multi-parametric unbiased approach, we identified derangements in the immunoregulatory and effector axes in SLE: inadequate natural T_reg_ response in both adult- and childhood-onset SLE, and an overall tolerance reset in adult-onset SLE. Future therapeutic strategies may need to focus on these to restore immune homeostasis. The concurrent study of adult- and childhood-onset SLE offers a unique opportunity to identify mechanistic commonalities that will be amenable to similar therapeutic strategies and dissimilarities that warrant different treatment approaches.

**Patient Consent**

Yes, I received consent

**Disclosure of Interest**

None declared

**Reference**

1. Yeo JG, Wasser M, Kumar P, Pan L, Poh SL, Ally F, Arkachaisri T, Lim AJ, Leong JY, Lai L, Yeo KT, Lee ESC, Chua CJH, Larbi A, Nyunt MSZ, Ng TP, Chiesa S, Gattorno M, Martini A, Paleja BS, Duterte CA, Chen J, Nay Yaung K, Tang SP, Ng SK, Yung CF, Tan AYJ, Lee SY, Ginhoux F, Albani S. The Extended Polydimensional Immunome Characterization (EPIC) web-based reference and discovery tool for cytometry data. Nature Biotechnology. 2020 Jun 1;38(6):679-84.

## PT035 Growth differentiation factor 15 (GDF15) and fibroblast growth factor 21 (FGF21) serum levels are increased in patients with juvenile dermatomyositis (JDM) at disease onset

### M. I. Petrone^1^, E. Marasco^1^, G. Prencipe^1^, I. Caiello^1^, V. Matteo^1^, L. Farina^1^, F. Piemonte^2^, C. Torda^2^, F. De Benedetti^1^, R. Nicolai^1^

#### ^1^Division of Rheumatology; ^2^Laboratory of Muscular and Neurodegenerative Diseases, IRCCS Ospedale Pediatrico Bambino Gesù, Rome, Italy

##### **Correspondence:** M. I. Petrone

*Pediatric Rheumatology 2023*, **21(Suppl 2):**PT035

**Introduction:** JDM patients show upregulation of interferon (IFN)-stimulated genes and downregulation of several genes related to mitochondrial function, suggesting a link of mitochondrial dysfunction and muscle inflammation in idiopathic inflammatory myopathies (IIM). The mitokines GDF15 and FGF21 are induced in situations of muscle stress, particularly mitochondrial myopathies. One published study demonstrated that GDF15 is increased in serum and muscle of adult patients with IIM and unpublished data showed elevated GDF15 plasma levels in JDM patients.

**Objectives:** To investigate serum levels of mitokines GDF15 and FGF21 in JDM patients at diagnosis, before start of immunosuppressive and glucocorticoid treatment, and evaluate possible correlations with clinical and laboratory findings, as well as with IFN-related biomarkers (type I IFN score, CXCL10, CXCL9 and neopterin).

**Methods:** We collected blood samples of 16 treatment-naïve JDM patients enrolled at time of diagnosis. Serum levels of FGF21, GDF15, CXCL10, CXCL9 and neopterin were analyzed by ELISA (normal values: 0-200 pg/ml, 200-1000 pg/ml, <300 pg/ml, <150 pg/ml, <1.59 ng/ml respectively); expression of 6 IFN-induced genes (IFI27, IFI44L, IFIT1, ISG15, RSAD2, SIGLEC1) was measured by real-time PCR and used to calculate a type I IFN score. For each patient, the following clinical data were recorded: physician’s global assessment (PGA) of disease activity VAS (Visual Analogue Scale), Childhood Myositis Assessment Score (CMAS), serum levels of creatine phosphokinase (CK, IU/l), MSA (myositis specific autoantibodies) status. Correlations were determined by the Spearman’s rank correlation coefficient. Non-parametric tests were used for comparisons between 2 groups.

**Results:** Twelve out of 16 patients were female (75%). Median age at disease onset was 6.1 years [IQR 4.25, 9.82] and median disease duration at diagnosis 3.4 months [IQR 1.80, 7.12]. Eleven patients were positive for at least one MSA. Median FGF21 levels were in the range of normal values [54 pg/ml (IQR 30.75-393.5)], whereas median GDF15 levels where increased [1370 pg/ml (IQR 1022-2055)]. Median GDF15 levels tended to be higher in anti-NXP2pos patients (n=4) when compared to the anti-NXP2neg patients (n=12) (3675 vs 1264 pg/ml, p=0.069). GDF15 levels, but not FGF21, showed significant correlation with CK levels (r_S_=0.61, p=0.013), PGA (r_S_=0.55, p=0.026) and CMAS (r_S_=-0.62, p=0.013). We found no significant correlations of GDF15 or FGF21 levels with IFN-related biomarkers.

**Conclusion:** In our cohort a significant proportion of JDM patients showed increased levels of GDF15, whereas median FGF21 levels were normal. GDF15 levels were highest in anti-NXP2+ patients. GDF15 levels were correlated to global and muscular disease activity. Our data support the potential use of GDF15 as biomarker for IMM.

**Patient Consent**

Yes, I received consent

**Disclosure of Interest**

None declared

**References**

1. De Paepe B, Verhamme F, De Bleecker JL. The myokine GDF-15 is a potential biomarker for myositis and associates with the protein aggregates of sporadic inclusion body myositis. Cytokine. 2020 Mar;127:154966. doi: 10.1016/j.cyto.2019.154966. Epub 2020 Jan 2. PMID: 31901761
2. Duvvuri B, Pachman L, Morgan G, Hermanson P, Wang T, Lood C. Growth and Differentiation Factor 15, an emerging biomarker of mitochondrial dysfunction-associated myopathies: implications for Juvenile Dermatomyositis. ACR Convergence 2022. November 10-14, 2022. Philadelphia, PA, USA.

## PT036 Active jsle is associated with distinct NK CELL transcriptional and phenotypic alterations

### A. Radziszewska^1,2^, H. Peckham^1,2^, N. M. de Gruijter^1,2^, R. Restuadi^1^, M. Butt^1,2^, E. C. Jury^2^, E. C. Rosser^1,2^, C. Ciurtin^1,2^

#### ^1^Centre for Adolescent Rheumatology Versus Arthritis at UCL UCLH and GOSH; ^2^Centre for Rheumatology Research, Division of Medicine, University College London, London, United Kingdom

##### **Correspondence:** A. Radziszewska

*Pediatric Rheumatology 2023*, **21(Suppl 2):**PT036

**Introduction:** SLE (Systemic Lupus Erythematosus) is a multisystem autoimmune disease characterized by production of autoantibodies against nuclear antigens. While the adaptive immune response has been studied extensively in SLE, there is limited and at times contradictory evidence for the role of natural killer (NK) cells in SLE and, in particular, in the more severe, juvenile phenotype (JSLE).

**Objectives:** The aim of this study was to investigate the phenotype and function of NK cells in patients with JSLE compared to age/sex-matched healthy controls (HCs) using flow cytometric and transcriptional profiling.

**Methods:** Peripheral blood mononuclear cells (PBMCs) were collected from JSLE patients (n=45, 15.2-32.2 years), and age/sex-matched HCs (n=66, 15.4-29.8 years). *Ex vivo* PBMCs were stained with anti-CD3, -CD8, -CD56, -perforin, -granzyme A, -granzyme B, -granulysin antibodies and analysed by flow cytometry. Annexin V and PI staining was used to detect NK cell apoptosis with and without IFN-α stimulation. Cytolytic activity of NK cells against CFSE labelled K562 target cells was assessed in a flow cytometric 4hr co-incubation assay. Statistical significance was calculated using Mann-Whitney U tests or t-tests as appropriate. Transcriptomic analysis of RNA sequencing data from purified NK cells from a subset of JSLE patients (n=12) and HCs (n=6) was performed using R software and the DESeq2 model.

**Results:** The proportions of total NK cells (p=0.00001) as well as perforin (p=0.00001) and granzyme A (p=0.0002) expressing NK cell populations were markedly diminished in JSLE patients. Reductions in NK cells in JSLE were associated with increased disease activity (SLEDAI) (r=-0.34, p=0.031). Transcriptomic analysis of NK populations from active and inactive JSLE patients (SLEDAI score cut-off 4) and HCs confirmed that disease activity was the main driver of differential gene expression in NK cells. NK cells from active JSLE patients compared to HCs had a high number of differentially expressed genes (n=239, p.adj<0.05) while NK cells from inactive patients were transcriptionally similar to those of HCs (n=4, p.adj<0.05). Although NK cytotoxic function appeared to be preserved in JSLE, NK cells from JSLE patients exhibited increased propensity toward apoptosis when stimulated with IFN-α (p=0.029).

**Conclusion:** This is the first study to show that the cytotoxic capacity and frequencies of NK cells were diminished in JSLE patients, and more so in active disease, characterised by profound NK cell transcriptomic differences. NK cells from JSLE patients were more susceptible to apoptosis which may, at least in part, explain the reduction of NK cells in JSLE.

**Patient Consent**

Not applicable (there are no patient data)

**Disclosure of Interest**

None declared

## PT037 Immunophenotyping revealed the distinct association between lymphocyte subsets and clinical manifestations in childhood-onset systemic lupus erythematosus

### A. Shimbo^1^, S. Kaneko^1^, H. Irabu^1^, Y. Akutsu^1^, K. Imai^2^, H. Kanegane^3^, M. Shimizu^1^, T. Morio^1^

#### ^1^Department of Pediatrics and Developmental Biology, Graduate School of Medical and Dental Sciences, Tokyo Medical and Dental University (TMDU), Tokyo; ^2^Department of Pediatrics,, National Defense Medical College, Tokorozawa; ^3^Department of Child Health and Development, Graduate School of Medical and Dental Sciences, Tokyo Medical and Dental University (TMDU), Tokyo, Japan

##### **Correspondence:** A. Shimbo

*Pediatric Rheumatology 2023*, **21(Suppl 2):**PT037

**Introduction:** Childhood-onset systemic lupus erythematosus(cSLE) is an autoimmune disease characterized by multisystem involvements, breakdown of self-tolerance and overproduction of autoantibodies with significant clinical heterogeneity. Differences in clinical phenotypes are reported to be related to underlying immune dysregulation.

**Objectives:** We aimed to determine the immunological phenotypes of cSLE by lymphocyte subsets and cytokine profiles and to investigate the association between the immunological phenotypes and clinical manifestations.

**Methods:** Peripheral blood samples were obtained from 22 cSLE patients at their diagnosis. The healthy control (HC) group consisted of 7 age-matched children for lymphocyte subsets and 10 for cytokines. The clinical manifestations were collected from medical records retrospectively. Lymphocyte subsets were evaluated by multicolour flow cytometry and serum cytokine levels were determined by enzyme-linked immunosorbent assay.

**Results:** In cSLE patients, the proportions of memory B, NK, iNKT and γδT cells were significantly decreased, whereas αβT, Treg, activated T cells and plasmablasts were significantly increased. Both myeloid dendritic cells (DCs) and plasmacytoid DCs were decreased in cSLE. IL-18, CXCL9, sTNF-RII, IL-6, IFNα and BAFF were increased compared to HC. Lupus nephritis was related to increased CD8+ T cells and memory B cells, and chilblain lupus was related to decreased γδT cells. Increased IFNα was related to increased memory B cells (% of CD19+ cells), transitional B cells, plasmablasts, IgM memory B cells and NK cells. In terms of treatment response, the increased proportion of Th17 was related to a longer duration of PSL reduction.

**Conclusion:** cSLE exhibited characteristic lymphocyte subset profiles markedly different from HC. Clinical manifestations of SLE were associated with lymphocyte subsets and cytokines. Th17 proportion may be associated with treatment response.

**Patient Consent**

Yes, I received consent

**Disclosure of Interest**

None declared

## PT038 Longitudinal clinical and biomarker trajectories in Juvenile dermatomyositis

### S. R. Veldkamp^1^, E. Noppers^2^, E. J. H. Schatorjé^3^, W. Armbrust^4^, S. S. M. Kamphuis^5^, P. C. E. Hissink Muller^6^, J. M. van den Berg^7^, B. M. Feldman^8^, J. Drylewicz^1^, F. van Wijk^1^, A. van Royen-Kerkhof^2^, M. H. A. Jansen^2^

#### ^1^Center for Translational Immunology, University Medical Center Utrecht; ^2^Paediatric Rheumatology and Immunology, Wilhelmina Children's Hospital, University Medical Center Utrecht, Utrecht; ^3^Paediatric Rheumatology, Amalia Children’s Hospital, Radboud University Medical Centre Nijmegen, Nijmegen; ^4^Paediatric Rheumatology and Immunology, Beatrix Children’s Hospital, University Medical Center Groningen, Groningen; ^5^Paediatric Rheumatology, Sophia Children’s Hospital, Erasmus University Medical Centre, Rotterdam; ^6^Paediatric Rheumatology, Willem Alexander Children’s Hospital, Leiden University Medical Centre, Leiden; ^7^Paediatric Immunology, Rheumatology and Infectious Diseases, Emma Children’s Hospital, Amsterdam University Medical Centers, Amsterdam, Netherlands; ^8^Division of Rheumatology, The Hospital for Sick Children, Child Health Evaluative Sciences, The Hospital for Sick Children Research Institute, and Departments of Pediatrics and Institute of Health Policy Management & Evaluation, University of Toronto, Toronto, Canada

##### **Correspondence:** S. R. Veldkamp

*Pediatric Rheumatology 2023*, **21(Suppl 2):**PT038

**Introduction:** The clinical management of juvenile dermatomyositis (JDM) patients is complicated by the heterogeneity in disease course, as well as the uncertainty regarding which patients can safely taper or discontinue immunosuppressive medication.

**Objectives:** We aimed to identify different disease trajectories with latent class mixed-effects models (LCMM), using clinical disease activity measures and two previously validated biomarkers Galectin-9 and CXCL10.

**Methods:** Longitudinal clinical and biomarker data of the first 60 months of follow-up (FU) were used of 81 JDM patients from 7 tertiary centers. Clinical data included scores for global disease activity (PGA), skin disease activity (aCAT), and muscle disease activity (CMAS). Biomarker (Galectin-9, CXCL10, CK) measurements were collected as part of regular care. The best LCMM for each variable was based on BIC value, maximum posterior probabilities, and clinical relevance.

**Results:** LCMM analysis of PGA scores identified 3 classes. PGA scores in class 1 (69%) steadily declined and stayed low, while in class 2 (20%) they declined and increased again around 20 months of FU. PGA scores in class 3 (11%) remained high. During FU, class 3 had significantly higher aCAT scores than class 1. Calcinosis occurred in 5%, 25% and 33% of class 1, 2 and 3, respectively. LCMM analysis of aCAT scores identified 2 classes. Patients in class 1 (78%) had gradually declining scores which then remained low, while patients in class 2 (22%) showed persistent skin disease activity. LCMM analysis of CMAS scores identified 2 classes. Patients in class 1 (54%) showed a slow but steady improvement of muscle disease during FU. Patients in class 2 (46%) had more serious muscle disease at baseline, but a faster improvement than class 1. Class 2 was defined by higher PGA scores and CK levels at baseline compared to class 1. Although LCMM analyses of Galectin-9 and CXCL10 were limited by a small number of measurements, two classes of CXCL10 could be identified. In class 1 (83%) CXCL10 levels steadily declined and remained low, while in class 2 (17%) levels initially declined, but increased again around 20 months of FU. Notably, all 3 patients in class 2 with available CXCL10 measurements after 25 months FU, flared (at 29, 32 and 45 months FU, respectively). Finally, a trend towards higher Galectin-9 levels was observed in PGA class 2 at 20 months FU, compared to class 1 PGA (no measurements available in PGA class 3).

**Conclusion:** LCMM analysis identified distinct disease trajectories in JDM patients that align with disease courses typically seen in the clinic: most patients achieve persistent remission, some experience flares, and a smaller subgroup has refractory (skin) disease. Rising levels of Galectin-9 and CXCL10 could be indicative of an upcoming flare. Combining longitudinal clinical and biomarker data can provide insights in underlying phenotypes and enable personalized treatment.

**Patient Consent**

Not applicable (there are no patient data)

**Disclosure of Interest**

None declared

## PT039 Comparison of cite-seq data between patients with Juvenile dermatomyositis and healthy controls identifies differences in cell composition and gene and epitope expression

### C. Wibrand^1,2^, E. Flynn^2^, G. Rabadam^2^, G. Hartoularos^2^, Y. Sun^2^, Z. J. Gartner^2^, C. Ye^2^, S. Kim^3^, M. Sirota^2^, J. Neely^2^

#### ^1^Aarhus University, Aarhus, Denmark; ^2^University of California, San Francisco; ^3^UCSF Benioff's Children's Hospital, San Francisco, United States

##### **Correspondence:** C. Wibrand

*Pediatric Rheumatology 2023*, **21(Suppl 2):**PT039

**Introduction:** Juvenile dermatomyositis (JDM) is a rare, serious systemic autoimmune condition, and much remains unknown about the pathogenesis of and the immune cell types and cell-specific pathways associated with disease.

**Objectives:** To increase knowledge of immune dysregulation in JDM with immunophenotyping at the cellular level using CITE-seq.

**Methods:** Multiplexed single cell RNA and protein sequencing was applied to 27 peripheral blood samples to simultaneously profile the gene and cell surface antibody (ADT) expression associated with JDM (n=15, samples=22) compared to healthy pediatric controls (HC)(n=5). Data processing steps included quality control, dimensional reduction, clustering, and cell type annotation using canonical ADT and gene markers. To determine JDM-associated changes, we performed: 1) cell type proportion analysis between individual groups (treatment naïve (TN), inactive, HC) (adjusted p-value < 0.05), 2) correlation analysis between cell type proportion and disease activity measures (p < 0.05), 3) differential gene and ADT expression analysis between TN JDM and HC for each cell type using DESeq2 (FDR < 0.05), and 4) gene overrepresentation analysis (GOA) with clusterProfiler (adjusted p value < 0.05).

**Results:** Using unsupervised clustering of 107,661 immune cells, we identified 29 clusters, which compromised 21 unique immune cell populations. TN patients had statistically significantly higher levels of transitional B cells compared to inactive JDM, and cell type proportion was positively correlated with disease activity measures. TN patients had significantly fewer NK and gd T cells than both healthy controls and inactive JDM, and cell type proportion was negatively correlated with disease activity measures. Tregs were significantly increased in TN JDM compared to HC. Although most differentially expressed genes were found within naïve B cells, monocytes, and specific T-cell subsets, including Tregs, many differentially expressed genes were shared among cell types, and GOA revealed overrepresentation of genes involved in type 1 interferon and interferon gamma pathways for most cell types. However, we found a characteristic heterogeneity in the interferon signature expression among patients. Analysis of differences in cell surface protein levels revealed upregulation of SIGLEC-1 and LAMP-1 on the surface of both CD14+ and CD16+ monocytes.

**Conclusion:** Alterations in immune cell composition within the B, CD4^+^ T, gd T, and NK cell compartments are associated with disease activity in JDM. Monocytes and naive B cells experience major gene expression changes during disease, but all cell types are heavily influenced by interferon signaling, although patient-specific expression patterns are observed. Future directions of this work include characterization of JDM-associated immunophenotypes at the transcriptomic and proteomic levels and network analysis.

**Patient Consent**

Not applicable (there are no patient data)

**Disclosure of Interest**

None declared

## PT040 Dysregulated mitochondrial gene signature as a biomarker for Juvenile dermatomyositis (JDM)

### M. G. L. Wilkinson^1,2,3^, R. Restuadi^1,2,3^, H. D. Nguyen^1,2^, M. Kartawinata^1,2^, V. Alexiou^1,2^, L. R. Marshall^1,2^, B. R. Jebson^1,2^, E. Ralph^1,2,3^, H. Peckham^2^, A. Radziszewska^2^, N. De Gruijter^2^, G. W. Otto^3,4^, G. Hall^3,4^, W.-Y. Lin^5^, D. Kelberman^3,4^, S. Castellano^3,4^, C. Cuirtin^2^, S. Eaton^6^, C. T. Deakin^1,2,3^, C. Wallace^5,7^, E. C. Rosser^2,8^, L. R. Wedderburn^1,2,3^ on behalf of UK Juvenile Dermatomyositis Research Group, Centre for Adolescent Rheumatology Versus Arthritis, CLUSTER consortium

#### ^1^Infection, Immunity and Inflammation Research and Teaching Department, UCL Great Ormond Street Institute of Child Health; ^2^Centre for Adolescent Rheumatology Versus Arthritis at UCL UCLH and GOSH, UCL; ^3^NIHR Biomedical Research Centre at GOSH, Great Ormond Street Hospital; ^4^Genetics and Genomic Medicine Research & Teaching Department, UCL Great Ormond Street Institute of Child Health, London; ^5^MRC Biostatistics Unit, University of Cambridge, Cambridge; ^6^Developmental Biology and Cancer Research & Teaching Department, UCL Great Ormond Street Institute of Child Health, London; ^7^Cambridge Institute of Therapeutic Immunology and Infectious Disease (CITIID), University of Cambridge, Cambridge; ^8^Centre for Rheumatology Research, UCL, London, United Kingdom

##### **Correspondence:** M. G. L. Wilkinson

*Pediatric Rheumatology 2023*, **21(Suppl 2):**PT040

**Introduction:** JDM is a rare childhood autoimmune myositis, typically presents with proximal muscle weakness and skin manifestations. JDM is characterised by abnormal interferon (IFN) type I signalling and mitochondrial abnormalities contributing to the disease pathogenesis(1). There is a need for better treatments, with novel therapeutics targeting IFN and mitochondria pathways, being the clear candidates.

**Objectives:** This study aimed to define and validate a JDM mitochondrial gene signature, explore whether this signature is shared with juvenile idiopathic arthritis (JIA), and investigate the disease specificity of the signature.

**Methods:** Peripheral blood mononuclear cell (PBMC) samples were obtained from treatment-naïve JDM and JIA patients and age/sex-matched child healthy controls (controls). RNA sequencing (RNAseq) was performed from total tPBMC and sorted CD14^+^ cells. Dataset-1 comprised JDM pre-treatment (n=10), and controls (n=6). Dataset-2 comprised JDM pre-treatment (n=30), JIA pre-treatment (n=84) and controls (n=18). Within dataset-2 the JDM and conrtol data were included for both CD14+ monocytes and tPBMC, while for JIA only CD14+ data were analysed. Differentially expressed genes (DEG) were analysed using EdgeR. Linear regression analysis was performed by comparing the log fold change in JDM CD14+ monocytes to PBMC, and to JIA CD14+ monocytes.

**Results:** DE analysis of dataset-1 showed gene dysregulation in the mitochondrion gene ontology term (MGO) and upregulated IFN type 1 genes (n=13) in CD14+ monocytes from JDM pre-treatment compared to controls. Confirmed by investigating the MGO of 1438 expressed genes, comparing DEG from datasets 1&2, CD14+ monocytes JDM pre-treatment compared to controls. 130 DEG in dataset-1 and 92 in dataset-2 and the overlap was 37 genes (FDR≤0.05, FC≤-1.5 or ≥1.5). The combined mitochondrial and IFN type 1 signature (MIGS) consisted of 48 genes. In dataset-2 clear separation was observed on the TPM (transcript per million) of normalised gene counts for the defined MIGS from JDM pre-treatment and controls by hierarchical clustering. Regression analysis showed that CD14+ monocytes had similar MIGS expression to total PBMC when JDM pre-treatment was compared to controls (estimate=0.99,95%CI(0.89,1.09),p-value<2.2e-16). In JIA pre-treatment the MIGS expression in CD14+ monocytes was less than in JDM pre-treatment compared to the same controls (estimate=3.62,95%CI(3.22,4.02),p-value<2.2e-16).

**Conclusion:** This study identified and validated a dysregulated mitochondrial signature in treatment-naïve JDM CD14+ monocytes, further validated in PBMCs. This signature partly overlapped with the CD14+ monocyte signatures in treatment-naïve JIA, suggesting a degree of disease-specificity. This signature could have clinical implications as a biomarker of mitochondrial health in JDM, potentially useful for patient treatment stratification.

**Patient Consent**

Yes, I received consent

**Disclosure of Interest**

None declared

**Reference**

1. Wilkinson MGL, Moulding D, McDonnell TCR, et al. Role of CD14+ monocyte-derived oxidised mitochondrial DNA in the inflammatory interferon type 1 signature in juvenile dermatomyositis Annals of the Rheumatic Diseases 2023;82:658-669

## PT041 Systemic Juvenile idiopathic arthritis associated lung disease in Europe

### C. Bracaglia^1^, F. Minoia^2^, C. Kessel^3^, S. Vastert^4^, M. Pardeo^1^, A. Arduini^1^, S. Fingerhutova^5^, I. Nikishina^6^, O. Basaran^7^, N. Kiper^8^, M. Kostik^9^, M. Glerup^10^, R. Caorsi^11^, A. Horne^12^, G. Filocamo^2^, H. Wittkowski^3^, M. Jelusic^13^, J. Anton^14^, S. Khaldi-Plassart^15^, A. Belot^15^, G. Horneff^16^, S. Palmer Sarott^17^, E. Cannizzaro Schneider^17^, L. Fotis^18^, P. Dolezalova^5^, A. Ravelli^19^, S. Ozen^7^, F. De Benedetti^1^ on behalf of MAS/sJIA Working Party of PReS

#### ^1^Division of Rheumatology, IRCCS Ospedale Pediatrico Bambino Gesù, Roma; ^2^Fondazione IRCCS Ca' Grande Ospedale Maggiore Policlinico, Milano, Italy; ^3^Department of Pediatric Rheumatology & Immunology, WWU Medical Center (UKM), Münster, Germany; ^4^Pediatric Rheumatology & Immunology, University Medical Center Utrecht, Utrecht, Netherlands; ^5^Centre for Paediatric Rheumatology and Autoinflammatory Diseases, Department of Paediatrics and Inherited Metabolic Disorders, 1st Faculty of Medicine, Charles University and General University Hospital in Prague, Prague, Czech Republic; ^6^V.A. Nasonova Research Institute of Rheumatology, Moscow, Russian Federation; ^7^Department of Pediatrics, Division of Pediatric Rheumatology, Hacettepe University; ^8^Department of Pediatrics, Division of Pediatric Pulmonology, Hacettepe University, Ankara, Türkiye; ^9^Saint-Petersburg State Pediatric Medical University, Saint-Petersburg, Russian Federation; ^10^Department of Pediatrics, Aarhus University Hospital, Aarhus, Denmark; ^11^Department of Pediatrics and Rheumatology, IRRCS Istituto G. Gaslini, Genova, Italy, ^12^Department of pediatric rheumathology Karolinska University Hospital and Department of pediatrics, Karolinska UnivKarolinska Institute, Stockholm, Sweden; ^13^Department of Pediatrics, University of Zagreb School of Medicine, University Hospital Centre, Zagreb, Croatia; ^14^Pediatric Rheumatology, Hospital Sant Joan de Déu, Universitat de Barcelona, Barcelona, Spain, ^15^Pediatric Nephrology, Rheumatology, Dermatology Unit, Hôpital Femme Mère Enfant, Hospices Civils de Lyon, Lyon, France; ^16^Pediatrics, Asklepios Clinic Sankt Augustin, Sankt Augustin, Germany; ^17^Pediatric Rheumatology, University Children’s Hospital Zurich, Zurich, Switzerland; ^18^Pediatric Rheumatology Division, 3rd Department of Pediatrics, National and Kapodistrian University of Athens,”ATTIKON” General University Hospital, Athens, Greece; ^19^IRRCS Istituto Giannina Gaslini and Università degli Studi di Genova, Genova, Italy

##### **Correspondence:** C. Bracaglia

*Pediatric Rheumatology 2023*, **21(Suppl 2):**PT041

**Introduction:** Chronic parenchymal lung disease (LD) is a new emerging severe life-threatening complication of Systemic Juvenile Idiopathic Arthritis (sJIA). The number of sJIA patients with LD is apparently increasing and interestingly they are reported more frequently in North America. Data regarding frequency and features of sJIA-LD in Europe are not available.

**Objectives:** To evaluate the burden of sJIA associated LD in Europe.

**Methods:** Patients with diagnosis of sJIA with LD, including pulmonary alveolar proteinosis (PAP), interstitial lung disease (ILD) and pulmonary arterial hypertension (PAH), followed in European paediatric rheumatology centres were identified through a survey sent to the members of the MAS/SJIA Working Party.

**Results:** Data from 49 JIA-LD patients, diagnosed in 17 European paediatric rheumatology centres between 2007 and 2022, were collected. 48 patients were Caucasian and 1 was African-American; 31 were female. The median age at sJIA onset was 7 years and LD occurred after a median time of 3 years. 27 patients had a chronic persistent sJIA course, 21 had a polycyclic course and only 1 patient had a monocyclic course; 38 patients (77%) had active sJIA at time of LD diagnosis. During the disease course, 41 (84%) patients developed MAS, 18 (44%) of whom had MAS at sJIA onset and 24 (58%) had full-blown MAS at time of LD diagnosis; 31 (76%) patients had >1 MAS episode. 42 (86 %) patients were treated with at least one IL-1 or IL-6 inhibitor before LD diagnosis: 33 with anakinra, 26 with canakinumab, and 23 with tocilizumab; 20 (40%) patients experienced drug adverse reaction to a cytokine inhibitor: 14 to tocilizumab and 6 to anakinra. 39 (80%) patients developed ILD, 6 (12%) PAP and 4 (8%) PAH. 22 (45%) patients presented acute digital clubbing; 18 (37%) patients developed hypoxia and 9 (18%) developed pulmonary hypertension. A chest CT scan was performed in all patients with evidence of septal thickening, peri-bronchovascular thickening and ground glass opacities in the majority of patients (39, 25 and 28 patients respectively). In 22 patients a bronchoalveolar lavage was performed and 15 underwent a lung biopsy. The histopathological pattern was alveolar proteinosis in 5 patients, endogenous lipoid pneumonia in 5, vasculitis in 1 and fibrosis in 1. 45 out of 49 patients were treated with glucocorticoids (GCs) at time of LD diagnosis, and 39 received IL-1 and/or IL-6 inhibitor after the diagnosis (25 anakinra, 20 canakinumab, 16 tocilizumab). Around half of the patients (23, 47%) required ICU admission and 9 (18%) died.

**Conclusion:** Lung involvement is an emerging life-threatening complication of sJIA, patients are also reported in Europe. Prompt recognition is crucial and new therapeutic strategies are needed to reduce the risk and improve the outcome of this complication.

**Patient Consent**

Yes, I received consent

**Disclosure of Interest**

C. Bracaglia: None declared, F. Minoia Consultant with: Sobi, C. Kessel Grant / Research Support with: Novartis, Consultant with: Novartis, Speaker Bureau with: Sobi, S. Vastert: None declared, M. Pardeo Consultant with: Sobi, A. Arduini: None declared, S. Fingerhutova: None declared, I. Nikishina Speaker Bureau with: Pfizer, Roche, Novartis, Pfizer, Sobi, MSD, R-Pharm, Ipsen, O. Basaran: None declared, N. Kiper: None declared, M. Kostik: None declared, M. Glerup: None declared, R. Caorsi Consultant with: Sobi, Novartis, A. Horne: None declared, G. Filocamo Consultant with: Sobi, H. Wittkowski: None declared, M. Jelusic: None declared, J. Anton Grant / Research Support with: Sobi, Novimmune, Novartis, Abbvie, Pfizer, GSK, Roche, Amgen, Lilly, BMS, Sanofi, Consultant with: Sobi, Novimmune, Novartis, Pfizer, GSK, Speaker Bureau with: Sobi, Novimmune, Novartis, GSK, Pfizer, S. Khaldi-Plassart: None declared, A. Belot Consultant with: SOBI, Novartis, Roche, Pfizer, G. Horneff Grant / Research Support with: MSD, Novartis, Roche, Speaker Bureau with: Abbvie, Chugai, Lilly, Sanofi, Novartis, Pfizer, S. Palmer Sarott: None declared, E. Cannizzaro Schneider: None declared, L. Fotis: None declared, P. Dolezalova: None declared, A. Ravelli: None declared, S. Ozen Consultant with: Sobi, Novartis, Speaker Bureau with: Sobi, Novartis, F. De Benedetti Consultant with: Abbvie, Sobi, Novimmune, Novartis, Roche, Pfizer.

## PT042 Enrichment of rare variants of hemophagocytic lymphohistiocytosis genes in systemic Juvenile idiopathic arthritis

### M. Correia Marques^1,2^, D. Rubin^1^, E. Shuldiner^3^, M. Datta^1^, E. Schmitz^1^, A. Grom^4^, D. Foell^5^, M. Gattorno^6^, J. Bohnsack^7^, R. S. M. Yeung^8^, S. Prahalad^9,10^, E. Mellins^11^, J. Anton^12^, C. Len^13^, S. Oliveira^14^, P. Woo^15^, S. Ozen^16^, I. Consortium^17^, Z. Deng^18^, M. Ombrello^1^

#### ^1^Translational Genetics and Genomics Section, National Institute of Arthritis and Musculoskeletal and Skin Diseases, Bethesda; ^2^Rheumatology, Children's National Hospital, Washington; ^3^Department of Biology, Stanford University, Stanford; ^4^Rheumatology, Cincinnati Children`s Hospital , Cincinnati, United States; ^5^Department of Pediatric Rheumatology and Immunology, University Hospital Muenster, Muenster, Germany; ^6^Unit Rheumatology and Autoinflammatory Diseases, IRCCS Istituto G. Gaslini, Genoa, Italy; ^7^Department of Pediatrics, University of Utah Eccles School of Medicine, Salt Lake City, United States; ^8^The Hospital for Sick Children, University of Toronto, Toronto, Canada; ^9^Emory University school of Medicine; ^10^Children’s Healthcare of Atlanta, Atlanta; ^11^Department of Pediatrics, Program in Immunology, Stanford University, Stanford, United States; ^12^Hospital Sant Joan de Déu, Universitat de Barcelona, Barcelona, Spain; ^13^São Paulo Federal University, São Paulo, ^14^Federal University of Rio de Janeiro, Rio de Janeiro, Brazil; ^15^University College London, London, United Kingdom; ^16^Department of Pediatrics, Hacettepe University, Ankara, Türkiye; ^17^National Institute of Arthritis and Musculoskeletal and Skin Diseases, Bethesda, United States; ^18^Biodata Mining and Discovery Section, National Institute of Arthritis and Musculoskeletal and Skin Diseases, Bethesda, United States

##### **Correspondence:** M. Correia Marques

*Pediatric Rheumatology 2023*, **21(Suppl 2):**PT042

**Introduction:** Systemic juvenile idiopathic arthritis (sJIA) is a complex inflammatory condition of childhood. It can be complicated by macrophage activation syndrome (MAS), a secondary form of hemophagocytic lymphohistiocytosis (HLH). Studies have identified an enrichment of HLH variants in sJIA patients with MAS compared with those without it. However, the role of these variants in sJIA in general is not known.

**Objectives:** To determine whether rare variation in HLH genes contributes to the risk of developing sJIA.

**Methods:** Targeted sequencing of HLH genes (*LYST, PRF1, RAB27A, STX11, STXBP2, UNC13D*) was performed in an established sJIA cohort using Illumina Nextera Custom Capture Assays and Illumina sequencers. Data processing and quality control were performed using a Genome Analysis Toolkit-based pipeline. Variants were filtered to retain high-quality, rare, protein-altering variation on Ensembl canonical transcripts. Healthy control exomes were extracted from dbGaP (phs000280.v8.p2). Rare variant association testing (RVT) was done with sequence kernel association test (SKAT). A burden test was also performed with the SKAT package. Significance was defined as p<0.05 after 100,000 permutations.

**Results:** Sequencing data from 524 sJIA cases were jointly called and harmonized with exome-derived target data from 2952 controls. Subjects were excluded if they had a monogenic disease, dissimilar genetic ancestry, or >10% missingness (total 45 cases, 28 controls). Analysis of the remaining 479 sJIA cases revealed 71 rare HLH gene variants, including recurrent ultra-rare (MAF<0.001) variants in *LYST* and *UNC13D* and a recurrent *STXBP2* variant that has been linked to severe COVID-19. RVT comparing the variant distributions of sJIA cases and controls revealed a significant association with rare (MAF<0.01) variants of *STXBP2* (p=0.019), and ultra-rare variants of *STXBP2* (p=0.006) and *UNC13D* (p=0.045). Sub-analysis of patients with known MAS status showed that the distribution of rare variants of *UNC13D* was different in 32 sJIA patients with MAS than in 90 without it (p=0.0047) or 2930 controls (p=0.03). There was an increased frequency of individuals with ≥1 rare HLH variants in all sJIA groups (MAS positive 28%, MAS negative 21%, full cohort 20%) compared to the controls (17%). Similarly, the full cohort (p=0.009) and those with MAS (p=0.025) had a higher burden of HLH variants than controls, whereas those without MAS did not (p=0.12).

**Conclusion:** We found that the association of HLH variants with sJIA is not purely linked to MAS. *STXBP2* was significantly associated with sJIA, while showing no association with MAS. Moreover, the frequency of HLH gene variants trended higher in MAS-negative sJIA than in control subjects. As seen in prior studies, *UNC13D* was associated with MAS. Taken together, this suggests that HLH variants may play a role in the pathophysiology of sJIA in general, and not only in MAS.

**Patient Consent**

Not applicable (there are no patient data)

**Disclosure of Interest**

None declared

## PT043 Changes of peripheral blood gene expression signatures in systemic Juvenile idiopathic arthritis upon treatment with canakinumab and their relationship to tapering success

### T. Hinze^1^, M. Saers^1^, C. Hinze^1^, C. Kessel^1^, C. Farady^2^, S. McCreddin^3^, M. Trautmann^4^, W. Hartmann^4^, D. Foell^1^

#### ^1^Department of Paediatric Rheumatology and Immunology, University of Münster, Münster, Germany; ^2^Novartis Pharma AG, Basel, Switzerland; ^3^Novartis Ireland Limited, Dublin, Ireland; ^4^Gerhard-Domagk-Institute of Pathology, University Hospital Münster, Münster, Germany

##### **Correspondence:** T. Hinze

*Pediatric Rheumatology 2023*, **21(Suppl 2):**PT043

**Introduction:** Systemic juvenile idiopathic arthritis (SJIA) is characterized by a pronounced dysregulation of immune-related peripheral blood gene expression. Canakinumab, an interleukin (IL)-1β blocking antibody, is clinically highly efficacious in most patients and can be successfully tapered in many. It is unclear how the presence of different gene expression signatures in patients with SJIA on canakinumab therapy relate to tapering success.

**Objectives:** The objective of this study was to identify differences in peripheral blood gene expression signatures in patients with SJIA who underwent successful canakinumab tapering and withdrawal versus those who did not.

**Methods:** In exploratory RNA seq analyses of 83 patient samples from an open-label randomized canakinumab trial (NCT02296424) or from a long-term extension trial of canakinumab (NCT00891046), and 10 paediatric healthy controls we identified candidate genes of interest associated with treatment outcomes. Subsequently, we studied 212 longitudinal whole blood RNA samples from the same cohorts via a custom 67 gene NanoString panel that was designed to represent identified candidate genes and various pathways (including neutrophil/innate immunity, Interferon (IFN) and/or erythropoiesis signatures). Correlation analyses and group comparisons were performed, considering various treatment outcomes, including success of canakinumab tapering and withdrawal.

**Results:** RNA seq analysis demonstrated that neutrophil/innate immunity signatures were reversible upon treatment with canakinumab. Using the NanoString dataset, correlation analysis across all samples showed clear neutrophil/innate immunity, IFN and erythropoiesis signatures. Treatment of active SJIA patients with canakinumab resulted in a lasting reversal of neutrophil/innate immunity and erythropoiesis signatures. When analysing 54 samples of patients with subsequent successful taper and discontinuation of canakinumab with 94 samples of patients with non-successful taper or discontinuation, there was significant differential expression of multiple genes with higher expression of erythropoiesis- and neutrophil/innate immunity-related genes in the non-successful group, whereas interferon-stimulated genes were not significantly different.

**Conclusion:** Canakinumab therapy seems to reverse prominent neutrophil/innate immunity-related and erythropoiesis gene expression signatures in patients with SJIA. Relatively higher expression of these signatures may signal a higher risk of non-successful canakinumab taper or discontinuation. This would need to be studied further.

**Patient Consent**

Not applicable (there are no patient data)

**Disclosure of Interest**

T. Hinze Grant / Research Support with: Research Support by Novartis Pharma AG, M. Saers: None declared, C. Hinze: None declared, C. Kessel Grant / Research Support with: Research Support by Novartis, C. Farady Employee with: Employee of Novartis, S. McCreddin Employee with: Employee of Novartis, M. Trautmann: None declared, W. Hartmann: None declared, D. Foell Grant / Research Support with: Research Support by Novartis

## PT044 Still’s disease patients with high interferon-stimulated gene expression have enrichment of rare, de novo and recessive variants in innate immune pathways

### N. Chowdhury^1^, Z. Deng^2^, M. Correia Marques^1^, E. G. Schmitz^1^, A. Platukus^1^, S. Brooks^2^, C. Lake^3^, L.-L. Bergeron^3^, M. Millwood^3^, M. J. Ombrello^1^

#### ^1^Translational Genetics and Genomics Section; ^2^Biodata Mining Section; ^3^Offie of the Clinical Director, National Institute of Arthritis and Musculoskeletal and Skin Diseases, Bethesda, United States

##### **Correspondence:** J. Ombrello

*Pediatric Rheumatology 2023*, **21(Suppl 2):**PT044

**Introduction:** Still’s disease (systemic juvenile idiopathic arthritis in children and adult-onset Still’s disease in adults) is an enigmatic inflammatory condition that affects people of all ages. It is characterized by inflammasome activation and pyroptosis with elevation of interleukin (IL)-1 and gasdermin D, and it is often punctuated by striking elevation of IL-18. Increased interferon (IFN) signaling, a known driver of pyroptosis, has been described in Still's disease complicated by either macrophage activation syndrome (MAS) or an emergent form of lung disease with hypersensitivity (LD/DHR), but it is unclear whether IFN activation is an active participant or an epiphenomenon.

**Objectives:** Using pathway analysis of candidate causative genes identified by trio whole exome-sequencing (WES), we sought to explore the relationship between genetic variation and increased IFN signaling in Still’s disease.

**Methods:** We examined consecutive people with Still’s disease enrolled in an IRB approved research study at the NIH Clinical Center. Whole blood expression of 28 IFN-stimulated genes (ISG) was quantified with custom NanoString arrays and expressed as a normalized score. Trio WES was performed in probands and a list of candidate causative variants (protein-altering *de novo,* recessive or X-linked variants; popfreqmax frequency<0.01) was generated. Candidate gene lists were compiled for individuals in the top or bottom quartiles of ISG scores and subjected to pathway over-representation analysis (ORA) and network topology analysis (NTA) using the Web-based Gene Set Analysis Toolkit (webgestalt.org).

**Results:** ISG scores among Still’s patients were negatively skewed and the upper quartile included 15 people with high ISG scores. LD/DHR was more common in the top quartile than in the bottom quartile (0.6, 0.13; p=0.02), and it was strongly associated with HLA-DRB1*15 alleles. The rates of active disease, IL-18 above 15K, use of IL-1 directed therapy, HLA-DRB1*15 carriage and history of MAS did not differ significantly between the groups. Trio sequencing identified 83 candidate genes among subjects in the top ISG quartile. ORA of candidate genes from individuals in the top ISG quartile revealed enrichment in the macrophage activation (ratio=21.7, p=3.4E-7, FDR=0.0055), type I IFN biosynthetic process (ratio=77.9, p=6.7E-6, FDR=0.03) and myeloid leukocyte activation (ratio=5.0, p=1.9E-5, FDR=0.039) pathways. Most people in the top ISG quartile (11/15) had candidate causative variation within these 3 pathways. Several other relevant pathways, including chemokine production, regulation of IL-18 production, and nuclear factor-kappa beta (NF-KB) signaling, also had evidence of over-representation (p<0.0005, FDR>0.05). NTA demonstrated significant enrichment of several pathways, including the MyD88-dependent TLR signaling pathway (corrected p=0.0045). ORA and NTA of 81 candidate genes from 15 subjects with the lowest ISG scores did not reveal enrichment of any pathway.

**Conclusion:** In our cohort, high ISG score was associated with the presence of LD/DHR but not MAS. Using unbiased analysis of trio WES in a Still’s cohort, we observed that people with high ISG scores had specific enrichment of potentially causative variation in innate immune pathways, most notably type I IFN production, MyD88/TLR signaling, and macrophage/myeloid leukocyte activation. Together this suggests that causative or high effect genetic variants are operative in the pathophysiology of Still's disease, where they can promote enhanced IFN signaling and may predispose to LD/DHR.

**Patient Consent**

Not applicable (there are no patient data)

**Disclosure of Interest**

None declared

## PT045 Safety of drugs for the treatment of systemic Juvenile idiopathic arthritis during the first 6 months since disease onset

### A. I. Rebollo-Giménez^1,2^, L. Carlini^1^, P. Miettunen^3,4^, E. Alexeeva ^5^, C. Myrup^6^, R. Nicolai ^7^, M. Trachana^8^, V. Stanevicha^9^, C. Ailioaie^10^, E. Tsitsami^11^, A.-V. Cochino^12^, C. Pallotti^1^, S. Scala^1^, A. Pistorio^13^, S. Vastert^14^, J. F. Swart^14^, N. Ruperto^15^ on behalf of Pediatric Rheumatology International Trials Organization

#### ^1^Unit of Rheumatology and Autoinflammatory Diseases, IRCCS Istituto Giannina Gaslini, Genoa, Italy; ^2^Universidad Autónoma de Madrid, Madrid, Spain; ^3^Alberta Children’s Hospital; ^4^University of Calgary, Calgary,AB, Canada; ^5^Federal State Autonomous Institution “National Medical Research Center of Children's Health” , Ministry of Health of the Russian Federation, Moscow, Russian Federation; ^6^Pediatric rheumatology unit 4272, Rigshospitalet, Copenhagen, Denmark; ^7^Division of Rheumatology, IRCCS Ospedale Pediatrico Bambino Gesù, Rome, Italy; ^8^First Department of pediatrics, Pediatric Immunology and Rheumatology Referral Center, Hippokration General Hospital, Thessaloniki University School of Medicine, Thessaloniki, Greece; ^9^Department of Paediatrics, Riga Stradins University, Children University Hospital, Riga, Latvia; ^10^Pediatric Rheumatology , Alexandru Ioan Cuza University of Iasi, Iasi, Romania; ^11^First Department of Pediatrics, University of Athens Medical School, Aghia Sophia Childrens Hospital, Athens, Greece; ^12^Pediatrics, Institute for Mother and Child Care, Bucharest, Romania; ^13^ Scientific Direction , IRCCS Istituto Giannina Gaslini, Genoa, Italy; ^14^Department of Pediatric Immunology and Rheumatology, Wilhelmina Children’s Hospital, Utrecht, Netherlands; ^15^UOSID Centro Trial,PRINTO, IRCCS Istituto Giannina Gaslini, Genoa, Italy

##### **Correspondence:** A. I. Rebollo-Giménez

*Pediatric Rheumatology 2023*, **21(Suppl 2):**PT045

**Introduction:** Treatment of systemic juvenile idiopathic arthritis (sJIA) includes conventional synthetic (cs) and biologic (b) disease modifying anti-rheumatic drugs (DMARDs) in addition to glucorticorticoids as needed.However,evidence on safety data regarding these treatments in sJIA is scarce.

**Objectives:** To report the adverse events (AE) of at least moderate intensity, serious AE (SAE) and events of special interest (ESI) in sJIA patients in the initial 6-months of treatment from Pharmachild registry.

**Methods:** Inclusion criteria were children with sJIA as per ILAR criteria with at least 6 month of follow-up visits.Data was available from 1987 until December 2021.Patients were classified into 4 mutually exclusive groups according to their treatment in the initial 6 months from disease onset:1)glucocorticoids, 2)bDMARDs, 3)csDMARDS and 4)combinations of these drugs. AE were classified as the latest version of MedDRA dictionary(Version 23.1). Statistical analysis included descriptive statistics,incidence rates(95% CI),time to occurrence of AE with Kaplan-Meier survival curve, the log rank test and Cox multivariate regression model.

**Results:** A total of 701/992 (71%) children with sJIA were classified into 4 different treatment arms as follows: 1)glucocorticoids(N=161), 2)bDMARDS(N=69); 3)csDMARDs(N=65) and 4)combo(N=406). The demographic characteristics of the patients included in the 4 groups were comparable. bDMARDs patients had the shortest duration of disease and older age at diagnosis compared to the other groups. With regards to the treatment, glucocorticoids exclusively were used more commonly before 2011.

A total of 125 AE in 701 patients,39 serious AE (SAE) and 48 ESI were observed in the initial 6 months of treatment.The two groups with the highest rate of AEs were a)bDMARDs with 32 AE/65 patients and b)combined therapy with 86 AE/406 patients. Infections/infestations were the most common AEs (varicella, viral gastroenteritis, impetigo, otitis media, pharyngitis, salmonellosis, herpes zoster,influenza, mycoplasma/Coxiella/viral/Epstein-Bar infection, pneumonia, pneumonia cytomegaloviral, subcutaneous abscess, respiratory tract/upper respiratory tract infection and vascular device infection),with the following rate of AEs per patient per year (N, IR*100PY-95%CI): a)bDMARDs group (**7**, 32.94 [15.70 - 69.10]) b)combination therapy group (**10**, 5.72 [3.08 - 10.63]), and c)glucocorticoids group (**2,** 4.42 [1.11 - 17.69 ])with no infectious events reported in the csDMARDs group.Gastrointestinal disorders were most frequent in the bDMARDs group(N, IR*100PY-95%CI) (**6**, 28.24 [12.69 - 62.85]), followed by the combo group(**4,** 2.29 [0.86 - 6.10]). General disorders and administrations site conditions (drug intolerance, fatigue, injection site rash, injection site abscess sterile) were more common in the bDMARDs group, followed by combination therapy group. No relevant differences have been observed for other SOCs. Kaplan Meier's showed the bDMARDs group experienced more adverse events and sooner in time when compared to the other groups (log rank test p<0.).The risk of any AE was significantly higher in patients with bDMARDs, hazard ratio 8.8 (95% CI: 3.5 to 22.1).

**Conclusion:** Treatment of children with sJIA with bDMARDs or combination therapy was associated with a higher prevalence of AE, SAE and ESI when compared to treatment with csDMARDS or glucocorticoids only in the first six months of therapy. Infections were the most common AE, principally consisting of respiratory infections and injection site reactions.

**Patient Consent**

Not applicable (there are no patient data)

**Disclosure of Interest**

None declared

## PT046 Defining criteria for disease activity states in systemic Juvenile idiopathic arthritis based on the systemic Juvenile arthritis disease activity score

### S. Rosina^1^, A. I. Rebollo Giménez^1^, L. Tarantola^2^, Y. Vyzhga^1^, L. Carlini^1^, E. Patrone^1^, M. Katsikas^3^, C. Saad Magalhães^4^, D. El-Ghoneimy^5^, Y. El Miedany^6^, R. Khubchandani^7^, P. Pal^8^, G. Simonini ^9^, G. Filocamo^10^, M. Gattinara ^11^, F. De Benedetti ^12^, D. Montin ^13^, A. Civino^14^, M. Alsuweiti^15^, V. Stanevicha^16^, V. Chasnyk^17^, E. Alexeeva^18^, S. M. Al-Mayouf^19^, S. Vilaiyuk^20^, A. Pistorio^21^, A. Ravelli^21^

#### ^1^UOC Reumatologia e Malattie Autoinfiammatorie, IRCCS Istituto Giannina Gaslini; ^2^Dipartimento di Neuroscienze, Riabilitazione, Oftalmologia, Genetica e Scienze Materno-Infantili (DiNOGMI), Università degli Studi di Genova, Genoa, Italy; ^3^Servicio de Inmunologia/Reumatologia, Hospital de Pediatria Juan P. Garrahan, Buenos Aires, Argentina; ^4^São Paulo State University (UNESP),, Botucatu, Brazil; ^5^PAIR Unit, Children's Hospital Ain Shams University; ^6^Ain Shams University, Italian Hospital St Abbassia, Cairo, Egypt; ^7^SRCC Childrens Hospital, Mumbai; ^8^Institute of Child Health, Kolkata, India; ^9^IRCCS Meyer Children’s Hospital, Florence; ^10^Fondazione IRCCS Ca' Granda-Ospedale Maggiore Policlinico; ^11^Istituto Gaetano Pini, Milan; ^12^IRCCS Ospedale Pediatrico Bambino Gesù, Rome; ^13^Regina Margherita Children Hospital, Turin; ^14^P.O. "Vito Fazzi", Lecce, Italy; ^15^King Hussein Medical Center, Amman, Jordan; ^16^University Children Hospital, Riga, Latvia; ^17^Saint-Petersburg State Pediatric Medical University, Saint-Petersburg; ^18^Federal State Autonomous Institution “National Medical Research Center of Children's Health” of the Ministry of Health of the Russian Federation, Moscow, Russian Federation; ^19^King Faisal Specialist Hospital & Research Center, Alfaisal University, Riyadh, Saudi Arabia; ^20^Mahidol University Faculty of Medicine, Bangkok, Thailand; ^21^Direzione Scientifica, IRCCS Istituto Giannina Gaslini , Genoa, Italy

##### **Correspondence:** S. Rosina

*Pediatric Rheumatology 2023*, **21(Suppl 2):**PT046

**Introduction:** The systemic Juvenile Arthritis Disease Activity Score (sJADAS) is a composite disease activity (DA) score specifically validated for use in systemic juvenile idiopathic arthritis (sJIA). The sJADAS10 is calculated as the simple linear sum of the scores of the following 5 items: 1) physician global assessment of overall DA, measured on a 10-cm visual analog scale (VAS), where 0 = no activity and 10 = maximum activity; 2) parent/patient global assessment of child’s well-being, measured on a 10-cm VAS, where 0 = very well and 10 = very poor; 3) count of joints with active disease in a maximum of 10 joints; 4) ESR or CRP level, normalized to a 0-10 scale; and 5) modified Systemic Manifestation Score (mSMS), ranging from 0 to 10, where 0 = absence of systemic manifestations and 10 = maximum activity of systemic manifestations. The range of the sJADAS10 is 0-50.

**Objectives:** To develop and validate the cutoffs in the sJADAS10 that define the states of inactive disease (ID), low (or minimal) DA (LDA), moderate DA (MDA) and high DA (HDA) in sJIA.

**Methods:** A multinational cross-sectional sample of 378 patients with definite or probable sJIA was enrolled. At study visit, each patient was categorized subjectively by the caring physician as being in the state of ID, LDA, MDA, or HDA. A total of 400 visits were collected, that were then randomly split into two samples: a developmental sample (n=240), and a validation sample (n=160). Optimal cutoff values were determined in the developmental sample against the subjective physician rating of DA state, that was used as external criterion, by multiple methods (calculation of percentiles of cumulative score distribution, ROC curve analysis, Youden index, 90% fixed specificity and kappa agreement). The choice of final cutoffs was based on clinical and statistical grounds. Cutoff validation was conducted in the validation sample by assessing discriminative ability.

**Results:** The selected sJADAS10 cutoffs were <=2.9 for ID, <=10 for LDA, 10-20.6 for MDA, and >20.6 for HDA. In validation analyses, the cutoffs showed strong ability to discriminate among DA states defined subjectively by the parents, parents’ satisfaction/dissatisfaction with illness outcome, level of child’s pain, presence/absence of functional impairment, presence/absence of morning stiffness, and normal/reduced quality of life.

**Conclusion:** We developed the cutoffs in the sJADAS10 that define the main DA states in sJIA. The cutoffs revealed good metrologic properties in the validation sample, and are therefore suitable for application in clinical practice and research.

**Patient Consent**

Not applicable (there are no patient data)

**Disclosure of Interest**

None declared

**Reference**

1. Tibaldi J, Pistorio A, Aldera E, Puzone L, El Miedany Y, Pal P, Giri PP, De H, Khubchandani R, Chavan PP, Vilaiyuk S, Lerkvaleekul B, Yamsuwan J, Sabui TK, Datta P, Pardeo M, Bracaglia C, Sawhney S, Mittal S, Hassan WA, Elderiny GF, Abu-Zaid MH, Eissa M, Sztajnbok F, das Neves Sztajnbok FC, Russo R, Katsicas MM, Cimaz R, Marrani E, Alexeeva E, Dvoryakovskaya TM, Alsuweiti MO, Alzyoud RM, Kostik M, Chikova I, Minoia F, Filocamo G, Farag Y, Lotfy H, Nasef SI, Al-Mayouf SM, Maggio MC, Magalhaes CS, Gallizzi R, Conti G, Shimizu M, Civino A, Felici E, Giancane G, Ruperto N, Consolaro A, Ravelli A. Development and initial validation of a composite disease activity score for systemic juvenile idiopathic arthritis. Rheumatology (Oxford). 2020 Nov 1;59(11):3505-3514. doi: 10.1093/rheumatology/keaa240. PMID: 32829413.

## PT047 Baseline clinical features and biomarker analysis of the Childhood Arthritis and Rheumatology Research Alliance (CARRA) Systemic Juvenile Idiopathic Arthritis-Associated Lung Disease (SJIA-LD) cohort

### E. Eloseily^1^, A. Clark^1^, M.-L. Chang^2^, M. E. Riordan^3^, A. Russell^4^, M. Natter^2^, S. Thornton^1,5^, Y. Kimura^3^, G. S. Schulert^1,5^; on behalf of CARRA Registry SJIA-LD Cohort Investigators

#### ^1^Rheumatology, Cincinnati Children's Hospital Medical Center, Cincinnati; ^2^Boston Children's Hospital, Boston; ^3^Hackensack University Medical Center, Hackensack; ^4^Duke Clinical Research Institute, Durham; ^5^Pediatrics, University of Cincinnati College of Medicine, Cincinnati, United States

##### **Correspondence:** G. S. Schulert

*Pediatric Rheumatology 2023*, **21(Suppl 2):**PT047

**Introduction:** Systemic juvenile idiopathic arthritis (SJIA) associated lung disease (SJIA-LD) is an emerging and life-threatening clinical problem with urgent unmet needs including prevalence, pathogenesis, disease biomarkers, influence of biologics, and outcomes.

**Objectives:** To define baseline clinical features and biomarker profiles of patients in the CARRA Registry SJIA-LD cohort.

**Methods:** Existing or newly enrolled CARRA Registry patients with SJIA and suspected, probable, or definite SJIA-LD were included in the cohort. In addition to standard Registry data, lung disease specific data was obtained using a standardized case report form through REDCap Cloud, and biosamples collected when available. Biomarker profiles were determined from plasma using a custom Luminex panel. This study was approved by the DCRI Reliant IRB and/or IRB of all Registry sites.

**Results:** 37 patients were enrolled in the SJIA-LD cohort, from 16 CARRA Registry sites in the US. 46% had definite (biopsy-proven), 36% probable, and 18% suspected SJIA-LD. Most common CT findings were ground glass opacities (55%) and peribronchovascular or septal thickening (41%). Of those who underwent lung biopsy, all had pulmonary alveolar proteinosis (PAP) and interstitial inflammation, and 40% had collagenous fibrosis. 77% had at least one definite episode of macrophage activation syndrome (MAS) (including 64% that met the 2016-SJIA-MAS criteria), 73% had more than one MAS episode, and 32% had subclinical MAS. MAS occurred prior to SJIA-LD diagnosis in 68% and coincided with it in 18%. More than 80% of patients remained on oral glucocorticoids. Physician global assessment of lung disease (PGALD) ranged from 0-8 (median 3.5) on a scale of 0-10 with 10 being most severe. 36% had PGALD of 0-2, while 27% had PGALD ≥5 reflecting severe disease. Overall health-related quality of life at enrollment was excellent in 26%, very good in 22%, good in 35%, and fiar in 17%.

Median serum IL-18 was markedly elevated at 24,336 ng/mL (IQR 4,147-49,275). Across all patient samples, SJIA-LD patients showed significantly increased plasma levels of IL-6, IL-12, IL-18, CXCL9, CD25, CCL11, CCL17, MCP-1, and MCP-3, compared to healthy control children. Cluster analysis defined 3 distinct groups of SJIA-LD patients. Group 1 (n=7) showed high levels of TNF, IL-6, IL-17, MCP-1 and 3, CCL11, and CCL17; group 2 (n=8) showed high IL-10, IL-12, IL-18, CXCL9, CXCL10, CD25, and CD163; and group 3 (n=10) showed high CCL15 and CCL25.

**Conclusion:** Patients in the CARRA SJIA-LD cohort represent a broad spectrum of clinical and radiographic features, disease activity, and treatment approaches. Recurrent MAS was common. Patients with SJIA-LD showed multiple distinct plasma biomarker patterns. This cohort will serve as an ongoing prospective cohort study of this emerging disease, to assess longitudinal disease progression and trajectories, as well as associated immune biomarkers and cellular populations.

**Patient Consent**

Yes, I received consent

**Disclosure of Interest**

E. Eloseily: None declared, A. Clark: None declared, M.-L. Chang: None declared, M. E. Riordan: None declared, A. Russell: None declared, M. Natter: None declared, S. Thornton: None declared, Y. Kimura: None declared, G. Schulert Grant / Research Support with: IpiNovyx, Consultant with: SOBI

## PT048 Serum Il-18 levels can improve the diagnostic performance of printo and ilar criteria for systemic Juvenile idiopathic arthritis

### M. Shimizu^1^, S. Kaneko^1^, A. Shimbo^1^, H. Irabu^1^, M. Mizuta^2^, Y. Nakagishi^2^, N. Iwata^3^, K. Yokoyama^4^, J. Yasumura^5^, K. Akamine^6^, K. Ueno^7^, S. Fujita^7^, K. Watanabe^8^, S. Watanabe^9^, H. Nishikawa^10^, J. Fujimura^11^, M. Mori^12^

#### ^1^Department of Pediatrics and Developmental Biology, Tokyo Medical and Dental University, Tokyo; ^2^Department of Pediatric Rheumatology, Hyogo Prefectural Kobe Children's Hospital, Kobe; ^3^Department of Immunology and Infectious Diseases, Aichi Children's Health and Medical Center, Obu; ^4^Department of Pediatrics, Japanese Red Cross Wakayama Medical Center, Wakayama; ^5^Department of Pediatrics, JR Hiroshima Hospital, Hiroshima; ^6^Department of Nephrology and Rheumatology, Tokyo Metropolitan Children's Medical Center, Tokyo; ^7^Department of Pediatrics, Toyama Prefectural Central Hospital, Toyama; ^8^Department of Pediatrics, Japanese Red Cross Nagaoka Hospital, Nagaoka; ^9^Department of Pediatrics, Ehime University, Toon; ^10^Department of Pediatrics, Nara Prefecture General Medical Center, Nara; ^11^Department of Pediatrics, Kakogawa Central City Hospital, Kakogawa; ^12^Department of Lifetime Clinical Immunology, Tokyo Medical and Dental University, Tokyo, Japan

##### **Correspondence:** M. Shimizu

*Pediatric Rheumatology 2023*, **21(Suppl 2):**PT048

**Introduction:** Pediatric Rheumatology International Trials Organization (PRINTO) has recently proposed revision of the current International League of Associations for Rheumatology (ILAR) criteria for systemic juvenile idiopathic arthritis (s-JIA). The requirement of arthritis has been removed from being a mandatory criterion because arthritis may not be observed as the initial manifestation of s-JIA. In contrast, leukocytosis has been added as a laboratory criterion among the minor criteria.

**Objectives:** This study was aimed to evaluate the performances of PRINTO criteria compared to ILAR criteria for s-JIA, and also to evaluate whether serum IL-18 levels improve the diagnostic performance of classification criteria for s-JIA.

**Methods:** This multicenter, retrospective study was conducted at 11 pediatric rheumatology institutes in Japan. This study included 65 patients with s-JIA and 13 patients with inflammatory diseases presenting prolonged fever over 14 days and arthritis and/or evanescent erythematous rash including refractory Kawasaki disease, reactive arthritis, Takayasu arteritis, Tubulointerstitial nephritis, Yersinia pseudotuberculosis infection, cryopyrin associated periodic syndrome and ANCA associated vasculitis. ILAR and PRINTO classification criteria were applied to all patients and checked with experts’ diagnosis. Serum IL-18 levels were determined by enzyme-linked immunosorbent assay.

**Results:** PRINTO criteria demonstrated higher sensitivity (100% vs 58%) but lower specificity (15.4% vs 84.6%) compared to ILAR criteria. Sensitivity of ILAR criteria and specificity of PRINTO criteria were improved with the addition of serum IL-18 levels ≥ 4800pg/mL to 97% and 100%, respectively.

**Conclusion:** Serum IL-18 levels could improve the diagnostic performance of PRINTO and ILAR criteria for systemic juvenile idiopathic arthritis. PRINTO criteria with the addition of serum IL-18 levels ≥ 4800pg/mL might be the best diagnostic performance for s-JIA.

**Patient Consent**

Not applicable (there are no patient data)

**Disclosure of Interest**

None declared

## PT049 Cytotoxic t cell phenotyping reveals overactivation and exhaustion as potentially inherent features in systemic JIA

### A. Swoboda, S. Schleifenbaum, C. Hinze, H. Wittkowski, D. Foell^1^, C. Kessel

#### Pediatric Rheumatology & Immunology, University Hospital Muenster, Muenster, Germany

##### **Correspondence:** A. Swoboda

*Pediatric Rheumatology 2023*, **21(Suppl 2):**PT049

**Introduction:** Systemic juvenile idiopathic arthritis (sJIA) is a rare childhood chronic inflammatory disorder with risk for life-threatening complications such as macrophage activation syndrome (MAS) or lung disease (LD). An early study on cellular phenotypes in sJIA demonstrated defects in the lytic machinery of sJIA NK- as well as CD8^pos^T cells.

**Objectives:** To date there are still little data on CD8^pos^T cell phenotypes and function outside of MAS, and therefore we aimed at an *ex vivo* whole blood phenotyping approach of CD8^pos^ T cells in non-complicated sJIA. Beyond, we set out to investigate their interplay with neutrophils as a highly abundant inflammatory cell population in sJIA, which is also known to interfere with CD8^pos^T cell function in other context.

**Methods:** Heparinized whole-blood of sJIA patients' samples (n=33) and healthy controls (n=20) were analyzed for effector molecule (IFNγ, TNFα, perforin, granzyme B) and cell activation (CD25, CD38, CD69, HLA-DR) or exhaustion (CD152 (CTLA-4), CD160, CD218a (IL-18R), CD233 (LAG-3), CD279 (PD-1), CD366 (TIM-3)). Autologous CD8^pos^T cells and neutrophils (sJIA n=7, HC n=11) were co-cultured with a range of inflammatory stimuli for 48 and 72h and analyzed by flow cytometry as described above.

**Results:** *Ex vivo*, we initially observed sJIA CD8^pos^T cells to reveal reduced cytokine (IFNγ, TNFα) and lytic molecule (granzyme B, perforin) expression compared to healthy controls, while perforin expressing cells were increased in frequency among a subset of CD69^neg/lo^CD8^pos^T cells. Compared to HC, several of the observed *ex vivo* phenotypes were recapitulated upon co-culture of sJIA CD8^pos^T cells with autologous neutrophils in ratios reflecting neutrophil excess in disease. Among HCs, effector molecule expressing CD25^pos^ or CD69^pos^CD8^pos^T cells expanded upon co-culture with neutrophils and with (neutrophil-specific) inflammatory load, which was not observed with sJIA T cells. Instead, in co-cultures of sJIA cells, perforin and granzyme expressing CD8^pos^T cells increased in frequency and with (neutrophil) stimulation, particularly among CD25^neg/lo^- and CD69^neg/lo^CD8^pos^T cells. These experiments suggest aberrant activation trajectories of sJIA CD8^pos^T cells, combined with sub-set specific increased cytotoxicity compared to healthy controls. Analyzing *ex vivo* cells for further activation and exhaustion markers reveals prominent overexpression of CD38, decreased expression of IL-18R and increased frequencies of PD-1^pos^CTLA-4^pos^ or PD-1^pos^Tim-3^pos^ cells as prominent features of sJIA CD8^pos^T cells, which were even present among (long-standing) remission patients.

**Conclusion:** Our still preliminary data point towards overactivation and exhaustion as potentially inherent features of sJIA CD8^pos^T cells, which may impact cellular effector function in context of hyperinflammatory events.

**Patient Consent**

Yes, I received consent

**Disclosure of Interest**

A. Swoboda: None declared, S. Schleifenbaum: None declared, C. Hinze Consultant with: Novartis, H. Wittkowski Consultant with: Novartis, Takeda, Octapharma and CSL-Behring, D. Foell Grant / Research Support with: Novartis, Pfizer and SOBI, Consultant with: Chugai-Roche, Novartis and SOBI, C. Kessel Grant / Research Support with: Novartis, Consultant with: Novartis and SOBI

## PT050 Unexpected lung disease in a cohort of systemic Juvenile idiophatic arthritis

### M. G. Villarreal, L. Vasconcellos, M. E. Puentes, J. P. Portigliatti, J. Manrique, M. C. Bertinotti, M. M. Katsicas

#### Hospital de Pediatria J.P. Garrahan, Buenos Aires, Argentina

##### **Correspondence:** M. G. Villarreal

*Pediatric Rheumatology 2023*, **21(Suppl 2):**PT050

**Introduction:** Systemic Juvenile Idiopathic Arthritis (sJIA) is conceptualized as an autoinflammatory disease. Lung disease (LD) is increasingly recognized in these patients.

**Objectives:** To describe frequency of LD in patients with sJIA. To compare sJIA patients with LD to those without it. To evaluate predictor variables for developing LD.

**Methods:** Patients with sJIA according to ILAR criteria were included, who had undergone at least one pulmonary HRCT (high resolution computed tomography). We reviewed their clinical charts and prospectively collected databases, recording demographic, clinical and biochemical features. Data were collected at baseline (time of diagnosis) and follow-up (at the time of HRCT). Outcome measures included: sJADAS (Systemic Juvenile Arthritis Disease Activity) and Juvenile Arthritis Damage Index (JADI). Presence of MAS (macrophage activation syndrome). Biological agents (anti-IL-1/anti-IL-6) and related adverse events also were recorded. LD was defined as suspected sJIA-LD based on objective findings on clinical examination (tachypnea, cough, or clubbing) or diffuse abnormalities on chest imaging according to Erkens et al. We compared variables between patients with and without LD using descriptive statistics, association tests and linear models for analysis.

**Results:** From a cohort of 32 sJIA patients, 18 (12F) met inclusion criteria, with median age at diagnosis was 5.2 (range 2.2-15.6) years. Median follow-up 4.7 years. At baseline, arthritis was present in 100%, fever 89%, rash 55%, lymphoproliferation 55%, enlarged lymph nodes 33%, hepatomegaly 11%, splenomegaly 17%, serositis 5%. Median sJADAS was 25 (16.5-37). At follow-up, arthritis was present in 66%, fever 22%, rash 11%, and enlarged lymph nodes and serositis 5%. Median sJADAS was 19 (0-37), JADI 0 (0-47). Thirteen (72%) patients received biological agents, of those three presented adverse events including anaphylaxis, hepatitis and local injection. Six patients had MAS. Ten patients (55%) had LD, but none of them presented with pulmonary symptoms. HRCT findings were: micronodules/nodules 54%, parenchymal thickening 45%, septal thickening 18%, bronchial wall thickening 9%, ground-glass opacities 9%, tree in bud opacities 9%. Enlarged lymph nodes (5 vs 1, p=0.04), lymphoproliferation (hepatosplenomegaly and enlarged lymph nodes) (9 vs 1, p=0.0037), and hemoglobin levels (9.2 vs 10.1 mg/dL, p=0.045) showed significant differences between patients with sJIA-LD and those without LD. Age at onset (4.9 vs 7.03 years, p=0.05) was a predictor variable to develop LD.

**Conclusion:** The frequency of sJIA-LD was high in our cohort of sJIA patients. There was a dissociation between clinical respiratory features and imaging findings. Patients with sJIA-LD presented more inflammatory features (lymphoproliferation and low hemoglobin levels) that mimicked autoinflammatory diseases.

**Patient Consent**

Yes, I received consent

**Disclosure of Interest**

None declared

## PT051 Treatment patterns and outcomes in patients with macrophage activation syndrome secondary to still’s disease treated with emapalumab: the real-HLH study

### C. E. Allen^1^, S. Chandrakasan^2^, M. B. Jordan^3,4^, J. W. Leiding^5,6^, A. Oladapo^7^, P. Pednekar^8^, K. J. Walkovich^9^, J. Yee^7^ on behalf of REAL-HLH Study Group

#### ^1^Division of Pediatric Hematology and Oncology, Baylor College of Medicine, Houston; ^2^Division of Bone and Marrow Transplant Research, Aflac Cancer and Blood Disorders Center, Children's Healthcare of Atlanta, Emory University, Atlanta; ^3^Division of Bone Marrow Transplantation and Immune Deficiency, Cincinnati Children's Hospital Medical Center; ^4^Department of Pediatrics, University of Cincinnati College of Medicine, Cincinnati; ^5^Division of Allergy and Immunology, Department of Pediatrics, Johns Hopkins University, Baltimore; ^6^bluebird bio, Cambridge; ^7^Sobi, Inc., Waltham; ^8^PRECISIONheor, Los Angeles; ^9^Division of Pediatric Hematology Oncology, Department of Pediatrics, University of Michigan Medical School, Ann Arbor, United States

##### **Correspondence:** A. Oladapo

*Pediatric Rheumatology 2023*, **21(Suppl 2):**PT051

**Introduction:** Macrophage activating syndrome (MAS) is a rare, potentially fatal complication of systemic juvenile idiopathic arthritis (sJIA) and adult-onset Still's disease (AOSD). MAS (a form of secondary HLH) is associated with overproduction of proinflammatory cytokines, such as interferon gamma (IFNγ). The REAL-HLH study assessed the real-world utilization of emapalumab, an anti-IFNγ antibody, among patients in the US.

**Objectives:** To understand treatment patterns and outcomes in patients with MAS secondary to Still's disease treated with emapalumab in the real-world setting.

**Methods:** A retrospective medical chart review conducted across 33 US hospitals identified patients treated with ≥1 dose of emapalumab between Nov. 20, 2018, and Oct. 31, 2021. Data extracted on the subset of patients with MAS secondary to Still's disease from the time of emapalumab initiation to end of data availability, death, or study end (Dec. 31, 2021) were analyzed.

**Results:** Ten of the 105 enrolled patients presented with Still’s disease (sJIA, n=9; AOSD, n=1). Most patients were female (8/10; 80%) and white (6/10; 60.0%). At diagnosis, mean (SD) age was 5.6 (6.4) years, and 70% (7/10) of patients met the 2016 ACR MAS diagnostic criteria. At diagnosis, the patient with AOSD was 22 years. At time of emapalumab initiation, all patients had received or were receiving other HLH-related therapies, including corticosteriods and anakinra; 60% of patients were in the intensive care unit. Emapalumab was mainly initiated to treat refractory (4/10; 40%), recurrent (3/10; 30%), or progressive (2/10; 20%) disease. Median (range) time from diagnosis to emapalumab initiation and treatment duration were 13.0 (1, 101) and 65.5 (25, 367) days, respectively. Median (range) emapalumab starting, maximum single, and cumulative administered dose were 3.7 (0.9-5.9), 5.8 (0.9-6.6), and 53.2 (6.7-171.3) mg/kg, respectively. Median (range) number of emapalumab doses was 15.5 (2, 35). Majority of patients achieved normal levels of ferritin (5/10; 50%), fibrinogen (6/10; 60%), platelets (8/10; 80%), alanine transaminase (8/10; 80%), absolute neutrophil count (9/10; 90%), and absolute lymphocyte count (9/10; 90%) during treatment. Median time to 1^st^ normalization of these laboratory parameters ranged from 7 to 46 days. Overall survival and 12-month survival probability following emapalumab initiation was 90% (9/10). One patient died due to uncontrolled viremia, unrelated to the clinical condition for which emapalumab was used (investigator determined).

**Conclusion:** This is the first study to report real-world treatment patterns and outcomes among patients with MAS secondary to Still’s disease treated with emapalumab. A phase 3 clinical trial of emapalumab in patients with sHLH/MAS and underlying rheumatologic disease is ongoing (NCT05001737).

**Patient Consent**

Not applicable (there are no patient data)

**Disclosure of Interest**

C. Allen Consultant with: Sobi, Inc., S. Chandrakasan Consultant with: Sobi, Inc., M. Jordan Consultant with: Sobi, Inc., J. Leiding Consultant with: Sobi, Inc., A. Oladapo Shareholder with: Sobi, Inc., Employee with: Sobi, Inc., P. Pednekar Consultant with: Sobi, Inc., K. Walkovich Consultant with: Sobi, Inc., J. Yee Employee with: Employed at Sobi, Inc. at the time the work was done

## PT052 Are there biomarkers to distinguish between PFAPA, surf and infections? Results of the pilot study

### A. Doležalová^1^, Š. Fingerhutová^1^, B. Stibůrková^2^, M. Tesařová^1^, J. Mašínová^2^, H. Hulejová^2^, N. Ondrušková^1^, N. Vinšová^1^, M. Pavlíková^3^, P. Doležalová^1^

#### ^1^Centre for Paediatric Rheumatology and Autoinflammatory Diseases ERN-RITA, Department of Paediatrics and Inherited Metabolic Disorders, General University Hospital and 1st Faculty of Medicine, Charles University; ^2^Institute of Rheumatology and Department of Rheumatology, 1st Faculty of Medicine, Charles University, Prague, Czech Republic; ^3^Department of Probability and Mathematical Statistics, Faculty of Mathematics and Physics, Charles University, Prague, Czech Republic

##### **Correspondence:** A. Doležalová

*Pediatric Rheumatology 2023*, **21(Suppl 2):**PT052

**Introduction:** Features of periodic fever, aphthae, pharyngitis, adenitis syndrome (PFAPA) are common in our patients. Although majority fulfil PFAPA criteria, some can be classified as a syndrome of undifferentiated recurrent fever (SURF). Its precise characteristics as well as distinction from PFAPA are yet to be defined.

**Objectives:** To characterize clinical, biochemical and immune phenotype of children with PFAPA and SURF during febrile and afebrile interval in comparison with controls.

**Methods:** Patients with PFAPA and SURF were prospectively recruited within an autoinflammatory disease (AID) project. Blood was taken at defined points: febrile – within 24h from recorded fever >38.0^o^C, afebrile – at least 2 weeks from the last fever. Otherwise healthy children with acute infections and patients with non-inflammatory conditions served as febrile and healthy controls (FC, HC). Sera were analysed for standard parameters (CRP, SAA, procalcitonin (PCT), ferritin, S100 proteins) and a 48-cytokine Bio-Plex panel. Patient sera were also analysed for glycosylation status of the individual glycoforms of bikunin (Bkn), a proteinase inhibitor often considered to be an acute phase protein.

**Results:** From the total of 287 AID patients 54 (18.8%) have one of hereditary periodic fevers, 179 (62.4%) have PFAPA, 54 (18.8%) undefined AID incl. SURF. In 9 PFAPA and 7 SURF patients (9/16 boys, mean age 8.9yrs) paired samples were available. In control groups (FC=7, HC=28) only one sample was analysed. In patients differences were found between febrile and afebrile samples at p<0.001 in the following: CRP, SAA, 100A8/9, ferritin, small and large Bkn glycoforms, IP-10, HGF, MIG, at p<0.01 in IL-1ra, IL-2ra, IL-18, M-CSF, at p<0.05 in IL-6, G-CSF, IL-12p40 (all higher at fever). PFAPA and SURF differed in neither febrile or afebrile samples. Febrile patients had significantly lower IL-13 than FC (p=0.0037). Afebrile patients had higher levels than HC at p<0.01 in IL-8, IL-18, GROa, TNF-b, TRAIL, at p<0.05 in IL-13, IL-17, G-CSF, IL-2ra. All CRP, SAA and PCT significantly correlated with S100A8/9, ferritin, IL-1ra, IL-6, G-CSF,IP-10, MIP-1a, IL-2ra, IL-12p40, IL-18. Both Bkn glycoforms inversely correlated with S100A8/9, ferritin, IL-2ra and M-CSF. Small and large glycoforms inversely correlated also with IP-10, IL-12p40, HGF, MIG and MIP-1a,IL-18, respectively.

**Conclusion:** We have shown that febrile PFAPA and SURF patients had increased amounts of multiple proinflammatory molecules, although none distinguished PFAPA from SURF in this small pilot cohort. Suppression of Th-2 associated cytokine IL-13 in febrile patients as well as changes in cytokines that have not been reported so far will have to be confirmed in larger cohorts and by single ELISA. For the first time we investigated glycosylation profiles in periodic fever patients and reported significant reduction of both Bkn glycoforms in febrile sera.

**Acknowledgements**

Supported by the Czech Health Research Council (AZV CR) grant NU21-05-00522 and SVV 260523.

**Patient Consent**

Yes, I received consent

**Disclosure of Interest**

None declared

## PT053 Disease control in patients with monogenetic autoinflammatory diseases under canakinumab treatment – comparison of 30 months interim data from the reliance registry

### T. Kallinich^1,2^, N. Blank^3^, J. Henes^4^, B. Kortus-Goetze^5^, P. T. Oommen^6^, A. Pankow^7^, T. Krickau^8,9,10^, C. Schuetz^11,11^, G. Horneff^12,13^, I. Foeldvari^14^, J. Rech^8,9,15^, F. Weller-Heinemann^16^, A. Janda^17^, M. Hufnagel^18^, F. M. Meier^19,20^, F. Dressler^21^, M. Borte^22^, I. Andreica^23^, P. Wasiliew^24^, M. Fiene^25^, D. Windschall^26^, J. Weber-Arden^27^, J. B. Kuemmerle-Deschner^24^

#### ^1^Department of Pediatric Respiratory Medicine, Immunology and Critical Care Medicine, Charité Universitätsmedizin Berlin; ^2^Deutsches Rheuma-Forschungszentrum (DRFZ), Berlin; ^3^Division of Rheumatology, Department of Internal Medicine, Heidelberg University Hospital, Heidelberg; ^4^Center of Interdisciplinary Rheumatology, Immunology and autoimmune diseases (INDIRA), University Hospital Tuebingen, Tuebingen; ^5^Department of Internal Medicine, Division of Nephrology, University Hospital of Giessen and Marburg, Marburg, Germany, Marburg; ^6^Department of Pediatric Oncology, Hematology and Clinical Immunology, Center for Child and Adolescent Health, Medical Faculty Heinrich-Heine-University Duesseldorf, Duesseldorf; ^7^Department of Rheumatology and Clinical Immunology, Charité-Universitätsmedizin Berlin, Berlin; ^8^Centre for rare diseases Erlangen (ZSEER), ^9^DZI (Deutsches Zentrum für Immuntherapie); ^10^Department of Pediatrics, Friedrich-Alexander University Erlangen-Nuernberg (FAU), Erlangen; ^11^Department of Pediatrics, Medizinische Fakultät Carl Gustav Carus, Technische Universität Dresden, Dresden; ^12^Department of Pediatrics, Asklepios Kinderklinik Sankt Augustin, Sankt Augustin; ^13^Department of Pediatric and Adolescent Medicine, Medical Faculty, University Hospital of Cologne, Cologne; ^14^Hamburg Centre for Pediatric and Adolescence Rheumatology, Hamburg; ^15^Department of Rheumatology and Immunology, University Hospital Erlangen, Erlangen; ^16^Division of Pediatric Rheumatology, Prof. Hess Children's Hospital, Bremen; ^17^Department of Pediatrics and Adolescent Medicine, University Medical Center Ulm, Ulm; ^18^Division of Pediatric Infectious Diseases and Rheumatology, Department of Pediatrics and Adolescent Medicine, University Medical Center, Medical Faculty, University of Freiburg, Freiburg; ^19^Fraunhofer Institute for Translational Medicine and Pharmacology ITMP; ^20^Department of General Pharmacology and Toxicology, Goethe University Hospital and Goethe University Frankfurt, Frankfurt am Main; ^21^Department of Paediatric Pneumology, Allergology and Neonatology, Children's Hospital, Hannover Medical School, Hannover; ^22^Academic Teaching Hospital of the University of Leipzig, Hospital for Children & Adolescents, St. Georg Hospital, Leipzig; ^23^Rheumazentrum Ruhrgebiet Herne, Ruhr-Universität Bochum, Herne; ^24^Division of Pediatric Rheumatology and autoinflammation reference center Tuebingen, Department of Pediatrics, University Hospital Tuebingen, Tuebingen; ^25^Rheumatology Center Greifswald, Greifswald; ^26^Clinic of Paediatric and Adolescent Rheumatology, St. Josef-Stift Sendenhorst, Northwest German Center for Rheumatology, Sendenhorst; ^27^Immunology, Novartis Pharma GmbH, Nuernberg, Germany

##### **Correspondence:** T. Kallinich

*Pediatric Rheumatology 2023*, **21(Suppl 2):**PT053

**Introduction:** Treatment of autoinflammatory periodic diseases (AID) with the interleukin-1β inhibitor canakinumab (CAN) has been shown to be safe and effective in controlled trials and real-world setting.

**Objectives:** In the RELIANCE registry, long-term safety and efficacy of CAN in patients with cryopyrin-associated periodic syndromes (CAPS), familial Mediterranean fever (FMF), hyper-IgD syndrome/mevalonate kinase deficiency (HIDS/MKD) and tumor necrosis factor receptor-associated periodic syndrome (TRAPS) on CAN therapy were investigated in routine clinical practice.

**Methods:** The RELIANCE registry is a prospective, non-interventional, observational study in Germany enrolling pediatric (age ≥2 years) and adult patients with a clinically confirmed diagnosis of AID who routinely receive CAN. Efficacy and safety parameters are recorded at baseline and assessed at 6-month intervals. To compare disease control between indications, parameters of 30 months visits were analyzed.

**Results:** In the present interim analysis, data were included from a total of n=232 patients with a diagnosis of CAPS, FMF, TRAPS and HIDS/MKD enrolled in the RELIANCE registry between October 2017 and December 2022. The median age of the total study cohort was 20.0 years (2−80 years). Most patients (n=198, 85 %) were CAN pre-treated when entering the study and the median duration of total CAN treatment before and during the study was 4 years (0−15 years).

Of 58/28/10/5 CAPS/FMF/TRAPS/HIDS patients with month 30 visits documented, 68/82/63/100% were in disease remission according to physician assessment. Patient-reported median disease activity and fatigue were low (1.5/1.5/1.5/0 and 3/2/4/0 on a 0−10 VAS scale). Inflammation markers (median) were within the limits of normal including neutrophil counts (2975/3420/3262/2897 n/μL). Statistical analysis confirmed similar efficacy across diseases in most parameters.

Even though these outcomes suggest an adequate disease control, an impact of the disease on patient´s social life was reported in 38/22/50/50% and negative influence on mood in 26/6/0/50% of patients.

**Conclusion:** The interim data of the RELIANCE study confirm sustained disease control of long-term treatment with CAN across all study indications. Even though disease activity measures suggest adequate disease control, patients´ social life and mood were negatively impacted by the disease.

**Patient Consent**

Yes, I received consent

**Disclosure of Interest**

T. Kallinich Speaker Bureau with: Roche, N. Blank Grant / Research Support with: Novartis, Sobi, Consultant with: Novartis, Sobi, Lilly, Pfizer, Abbvie, BMS, MSD, Actelion, UCB, Boehringer-Ingelheim, Roche, J. Henes Grant / Research Support with: Novartis, Roche, Consultant with: Novartis, AbbVie, Sobi, Roche, Janssen, Boehringer-Ingelheim, B. Kortus-Goetze Consultant with: Novartis, P. Oommen Grant / Research Support with: Novartis, A. Pankow: None declared, T. Krickau Grant / Research Support with: Novartis, Consultant with: Novartis, Speaker Bureau with: Novartis, C. Schuetz Grant / Research Support with: Novartis, G. Horneff Grant / Research Support with: AbbVie, Chugai, Merck Sharp & Dohme, Novartis, Pfizer, Roche, Speaker Bureau with: AbbVie, Chugai, Merck Sharp & Dohme, Novartis, Pfizer, Roche, I. Foeldvari Consultant with: Novartis, J. Rech Grant / Research Support with: Novartis, Sobi, Consultant with: AbbVie, Biogen, BMS, Chugai, GSK, Janssen, Lilly, MSD, Mylan, Novartis, Roche, Sanofi, Sobi, UCB, Speaker Bureau with: AbbVie, Biogen, BMS, Chugai, GSK, Janssen, Lilly, MSD; Mylan, Novartis, Roche, Sanofi, Sobi, UCB, F. Weller-Heinemann: None declared, A. Janda: None declared, M. Hufnagel Grant / Research Support with: Novartis, F. Meier Speaker Bureau with: Novartis, F. Dressler Grant / Research Support with: Novartis, Consultant with: Abbvie, Mylan, Novartis, Pfizer, M. Borte Grant / Research Support with: Pfizer, Shire, I. Andreica Consultant with: Abbvie, Chugai, Novartis, UCB, Galapagos, Takeda, Astrazeneca, Lilly, Boehringer Ingelheim, Amgen, Sobi, Paid Instructor with: Astrazeneca, UCB, Speaker Bureau with: Abbvie, Chugai, Novartis, UCB, MSD, Lilly, Sobi, Astrazeneca, Amgen, Pfizer, Gilead, P. Wasiliew: None declared, M. Fiene: None declared, D. Windschall: None declared, J. Weber-Arden Employee with: Novartis, J. Kuemmerle-Deschner Grant / Research Support with: Novartis, AbbVie, Sobi, Consultant with: Novartis, AbbVie, Sobi

## PT054 Prevalence, clinical features, treatment and outcome of macrophage activation syndrome in Multisystem Inflammatory Syndrome in Children (MIS-C): data from the hyperped-covid registry

### F. Lucioni^1^, R. Caorsi^2^, A. Consolaro^2,3^, C. Speziani^3^, F. Bovis^3^, C. Bracaglia^4^, M. Cattalini^5^, P. Brogan^6^, C. Wouters^7^, A. Taddio^8^, F. Candotti^9^, I. Meyts^10^, F. De Benedetti^4^, N. Ruperto^2,11^, A. Ravelli^2,3^, M. Gattorno^2^, F. Minoia^1^ on behalf of on behalf of the Steering Committee for Hyperped-COVID registry for Rita-ERN, Issaid, PRES, ESID, PRINTO

#### ^1^Fondazione IRCCS Ca' Granda Ospedale Maggiore Policlinico, Milan; ^2^IRCCS Istituto Giannina Gaslini; ^3^Università degli Studi di Genova, Genoa; ^4^IRCCS Ospedale Pediatrico Bambino Gesù, Rome; ^5^University of Brescia and ASST Spedali Civili di Brescia, Brescia, Italy; ^6^Great Ormond St Hospital, London, United Kingdom; ^7^UZ and KU Leuven, Leuven, Belgium; ^8^Institute for Maternal and Child Health IRCCS "Burlo Garofolo" and University of Trieste, Trieste, Italy; ^9^CHUV, Lausanne, Switzerland; ^10^University of Leuven, Leuven, Belgium, Leuven, Belgium; ^11^IRCCS Istituto Giannina Gaslini, Pediatric Rheumatology International Trial Organization, Genoa, Italy

##### **Correspondence:** F. Lucioni

*Pediatric Rheumatology 2023*, **21(Suppl 2):**PT054

**Introduction:** Macrophage activation syndrome **(**MAS) has been reported in up to 20-50% of patients diagnosed with Multisystem Inflammatory Syndrome (MIS-C). As clinical and laboratory features of MIS-C partially overlap with MAS, diagnosis may be challenging. The 2016 classification criteria for MAS in systemic juvenile idiopathic arthritis (sJIA) have been largely used to identify MAS in MIS-C; however, the diagnostic performance of existing MAS criteria has never been evaluated in MIS-C and no specific diagnostic criteria for MAS in MIS-C exist

**Objectives:** To evaluate prevalence, clinical and laboratory features, therapeutic approaches and outcome of MAS in patients with MIS-C

**Methods:** The HyperPED-COVID is an international registry coordinated by PRINTO, aimed to collect data on patients with MIS-C worldwide. In the case reported form, clinicians were asked to specify if MIS-C patients developed MAS. Demographic, clinical, laboratory data together with therapeutic choices and outcome were compared between patients with and without MAS. Chi-square, Fisher and Mann-Whitney test were used to analyze data, as appropriate.

**Results:** Currently, data regarding 1009 patients with MIS-C are available in the HyperPED-COVID registry; in 59 cases (5.8%) a diagnosis of MAS was made by the caring physician. Patients with MAS were more frequently collected from East-Central Europe centres (63% vs 31%, p <.0001); they were older (9.7 vs 7.9 years, p 0.003) and with a longer disease duration at MIS-C onset (10 vs 5 days, p <.001). As expected, in patients with MAS hepatomegaly and splenomegaly were more frequently reported than in non-MAS, albeit in low percentages (25% and 20%, respectively). Notably, MAS was characterized by a higher rate of myocardial dysfunction (34% vs 19%, p 0.008) and less mucocutaneous involvement (76% vs 89%, p 0.005). MAS patients had higher levels of ferritin (1085 vs 407 ng/ml, p <.0001) and lower platelet counts (137 vs 197 x 10^9^/l, p<.0001) compared with MIS-C without MAS. At MIS-C diagnosis 205 patients (20.3%) satisfied the 2016 MAS classification criteria, however 174 (84.8%) did not receive a MAS diagnosis from the caring physician. Patients with MAS were treated more aggressively, especially with glucocorticoids (97% vs 80%, p 0.002), and anakinra (42% vs 9%, p <.0001). Despite similar mortality and risk of sequelae, MAS required more frequently a circulatory support (44 % vs 26%, p 0.003).

**Conclusion:** Despite a prevalence lower than previously reported, MAS in MIS-C is associated with a more severe phenotype, requiring prompt diagnosis and aggressive treatment to avoid complications. Patients at higher risk are older and with a longer disease duration at MIS-C diagnosis. Further studies are needed to identify the best criteria to diagnose MAS in MIS-C

**Patient Consent**

Yes, I received consent

**Disclosure of Interest**

F. Lucioni: None declared, R. Caorsi: None declared, A. Consolaro: None declared, C. Speziani: None declared, F. Bovis: None declared, C. Bracaglia Consultant with: SOBI, Novartis, M. Cattalini: None declared, P. Brogan: None declared, C. Wouters: None declared, A. Taddio: None declared, F. Candotti: None declared, I. Meyts: None declared, F. De Benedetti: None declared, N. Ruperto: None declared, A. Ravelli: None declared, M. Gattorno: None declared, F. Minoia Consultant with: SOBI

## PT055 Genotype-phenotype correlation in a cohort of pediatric patients with autoinflammatory disease and nod2 gene variants

### M. F. Natale, C. Celani, S. Federici, F. De Benedetti, A. Insalaco

#### Rheumatology, Bambino Gesù Children's Hospital, Rome, Italy

##### **Correspondence:** M. F. Natale

*Pediatric Rheumatology 2023*, **21(Suppl 2):**PT055

**Introduction:** Systemic Autoinflammatory diseases (SAIDs) are a group of rare disorders caused by dysfunction of the innate immune system and characterized by periodic or chronic systemic inflammation. Nowadays a lot of monogenic or polygenic autoinflammatory diseases are described. Nucleotide-binding oligomerization domain containing 2 (NOD2) is a gene, associated with Blau syndrome, Crohn’s disease (CD) and most recently with a polygenic autoinflammatory disease with onset in adulthood called NAID or YAO-Syndrome.

**Objectives:** to describe the characteristics of a pediatric cohort with autoinflammatory undefined phenotype carrying NOD2 gene variants, to compare them with the NAID adult cohort described in literature and to evaluate genotype-phenotype correlation

**Methods:** 28 pediatric patients with undefined autoinflammatory disease carrying NOD2 gene variants were enrolled. NOD2 variants classified as pathogenic for BLAU syndrome or with an high prevalence in healthy population (polymorphisms) were excluded. Demographic, clinical, instrumental, laboratory characteristics and therapeutic approaches were collected. Our data were then put in comparison with the adult cohorts.

**Results:** The 28 patients enrolled were stratified into three subgroups based on the position of the variants on the NOD2 gene: N-Terminal (1 patient), Exon 4 (15 patients) and C-Terminal (12 patients). The 15 patients whose variants were localized on Exon 4 (10 males) presented homogeneous clinical characteristics with involvement of some target organs : skin (87%), joints (80%), bowel (54%), eyes (27%) and lymphatic system (54%). In 73% fever was present . Inflammation markers were increased in all patients while no circulating autoantibodies or signs of immunodysregulation were found. The most effective first-line therapy were glucocorticoids ; in refractory or glucocorticoid -dependent patients, IL-1/IL-6 inhibitors were used with good response. The characteristics of pediatric patients were shown to be completely similar to the adult patient cohorts described in literature. The only statistically significant differences were found in oral aphthosis, more frequent in children, and sicca syndrome exclusive of the adult patients.

**Conclusion:** This is the first study that evaluate genotypic/phenotypic characteristics of pediatric patients with systemic autoinflammatory phenotype carrying NOD2 gene variants. A close genotype-phenotype correlation has not been identified but the analysis showed a higher weight of variants localized on exon 4: patients carrying variants localized on this exon seems to show an homogeneous phenotype . Our data, if confirmed by larger future studies and functional tests, could identify a new pediatric autoinflammatory disease with defined characteristics and with a targeted and effective therapeutic approach.

**Patient Consent**

Yes, I received consent

**Disclosure of Interest**

None declared

## PT056 Ultrasonographic evaluation of enthesitis in patients with chronic nonbacterial osteomyelitis

### S. Sener^1^, E. Atalay^1^, A. E. Yildiz^2^, O. Basaran^1^, E. D. Batu^1^, Y. Bilginer^1^, S. Ozen^1^

#### ^1^Pediatric Rheumatology; ^2^Radiology, Hacettepe University, Ankara, Türkiye

##### **Correspondence:** S. Sener

*Pediatric Rheumatology 2023*, **21(Suppl 2):**PT056

**Introduction:** Enthesitis is a common presenting feature in juvenile-onset spondylarthritis and juvenile idiopathic arthritis, especially in enthesitis-related arthritis (ERA).

**Objectives:** Based on the overlapping features between ERA and chronic nonbacterial osteomyelitis (CNO), we aimed to evaluate the presence of enthesitis in CNO patients by ultrasonography (US).

**Methods:** Patients who were followed up with the diagnosis of CNO in Hacettepe University Pediatric Rheumatology Department between January 2022 and December 2022. All CNO patients had clinical symptoms lasting >6 weeks with unifocal or multifocal inflammatory bone lesions, in addition, infections and malignancies had been excluded in these patients. They were evaluated by a pediatric radiologist with the US. The insertion sites of seven bilateral tendons (common extensor and flexor tendons of the forearm, quadriceps tendon, proximal and distal patellar tendon, Achilles tendon, and plantar fascia) were examined in detail. US examinations were performed according to the pediatric OMERACT enthesitis scoring system.

**Results:** Forty CNO patients were included in this study. The median (IQR) age of the patients at diagnosis and at the time of US assessment were 9.4 (3.8) and 10.5 (6.1) years, respectively (M/F:1.1). Thirty-three patients (82.5%) were receiving anti-inflammatory treatment at the time of US evaluation. And also, the majority of patients were in clinical remission (82.5%), and had acute phase reactants in the normal range (90%). None of the patients had clinical signs of enthesitis on physical examination. Human leukocyte antigen B27 (HLA-B27) was positive in 25% (6/24) of the patients tested. In addition, unilateral or bilateral sacroiliitis was present in 80% of patients in whole-body magnetic resonance imaging (MRI). In the US, enthesitis was detected in three CNO patients (7.1%) and radiologically in four (0.6%) of 560 enthesis sites. Three of the involved enthesis areas were Achilles tendons and one was quadriceps tendon. All three patients had radiologically proven bilateral sacroiliitis and had positive HLA-B27. All of the patients were in clinical remission under anti-inflammatory therapy. Therefore, no treatment changes were made according to the US findings only. But these patients were followed up with both clinical examination and the US at close intervals. In the first six-month follow-up, enthesitis findings in one of these patients had disappeared, while subclinical enthesitis was still present in the other two patients in the US.

**Conclusion:** This is the first study to research enthesitis in patients with CNO. Although enthesitis is a rare finding in CNO, the US can be a helpful follow-up tool in CNO patients as it can detect enthesitis. In addition, if a patient with CNO has enthesitis, it would be more appropriate to consider this patient as having CNO and ERA overlap syndrome.

**Patient Consent**

Yes, I received consent

**Disclosure of Interest**

None declared

**Reference**

1. Zhao DY, McCann L, Hahn G, Hedrich CM. Chronic nonbacterial osteomyelitis (CNO) and chronic recurrent multifocal osteomyelitis (CRMO). J Transl Autoimmun. 2021 Mar 20;4:100095. doi: 10.1016/j.jtauto.2021.100095.

## PT057 Profile of autoimmunity and immune dysregulation in 319 patients with inborn errors of immunity at a tertiary care center in Southern India

### N. Singh^1^, J. Janardhanan^1^, C. Ginigeri^2^, H. K. H^2^, S. M. Naushad Ali^2^, S. Bhattad^1^

#### ^1^Pediatric Immunology and Rheumatology; ^2^Pediatrics, Aster CMI Hospital, Bengaluru, India

##### **Correspondence:** N. Singh

*Pediatric Rheumatology 2023*, **21(Suppl 2):**PT057

**Introduction:** Inborn Errors of Immunity (IEI) are a heterogeneous group of disorders that predispose affected individuals to infections, autoimmunity, autoinflammation, and malignancies. The literature on autoimmunity in IEI in Indian subcontinent is limited hence there is need to highlight the clinical presentation and management of these patients.

**Objectives:** To study the profile of autoimmune manifestations and immune dysregulation in patients with IEIs at presentation or during follow-up.

**Methods:** This study was carried out at a tertiary care center in Southern India from February 2017 to January 2023. Data on IEI was entered in a pre-designed excel sheet. The clinical spectrum of autoimmunity, treatment & outcome details were analyzed.

**Results:** Three hundred nineteen patients (311 kindreds) were diagnosed with IEI based on relevant immunological tests and/or genetic tests. Male-to-female ratio was 1.9:1. The mean age at onset of first clinical presentation in children (<18 yrs) was 1.24 years (range 1 day - 18 years) and 19 years (range 19-62 years) in adults (>18 years). The most common IEI was Severe Combined Immunodeficiency (SCID) (n=44,12%) in children and Common Variable Immune Deficiency (CVID) (n=25, 7.9 %) in adults. Autoimmunity was noted in 80 (25%) patients during their course of illness. The mean delay at diagnosis was 8.2 years in ‘autoimmune cohort’ . This was statistically higher as compared to the rest of the study population (p <0.001). Molecular testing was performed in 234 patients by exome sequencing and a genetic variant was reported in 220 cases.

The most common autoimmune manifestations included inflammatory colitis(n=23), followed by autoimmune cytopenia(n=19), arthritis(n=13) and autoimmune endocrinopathy (n=10). Autoimmune skin manifestations included pyoderma gangrenosum(n=7), alopecia areata (n=4), vitiligo(n=3), bullous pemphigoid(n=1), dermatitis herpetiformis(n=1) and cutaneous vasculitis(n=1). Systemic lupus erythematosus(n=6), Kawasaki Disease(n=4), lymphoproliferation(n=3), and autoimmune hepatitis(n=3), CNS vasculitis (n=2), nephrotic syndrome (n=1), oral aphthosis (n=1), sarcoidosis(n=1), and uveitis(n=1) were also reported.

Patients received immunomodulation as IVIG (n=43), steroids (n=36), methotrexate (n=10), colchicine (n=6), cyclosporine (n=3), sirolimus (n=3), mesalamine (n=3), leflunomide(n=2), thalidomide(n=2), tofacitinib (n=2), mycophenolate mofetil (n=1), azathioprine (n=6) and dapsone (n=1). Biologics including rituximab(n=2), anakinra (n=2), tocilizumab (n=1), adalimumab (n=1), and infliximab(n=1) were also used. One patient received romiplostim for thrombocytopenia. The overall survival was 90% in the “autoimmune” cohort.

**Conclusion:** Autoimmunity is a frequent yet underrecognized manifestation of IEI. The inherent predisposition to develop infections makes management of autoimmunity challenging and hence immunomodulation with close monitoring is the only way forward for these patients.

**Patient Consent**

Yes, I received consent

**Disclosure of Interest**

None declared

## PT058 A novel RNF31 gene mutation causes lubac deficency leading to systemicc autoifnlammations, immunodeficecy and cardiomyopathy: long term outcome after stem cell transplantion

### W. M. Suwairi^1^, J. AL Qanatish ^1^, F. Alrogi^2^, W. Eyaid ^3^, M. Essa^4^

#### ^1^Pediatric Rheumatology; ^2^Pediatric immunology; ^3^Medical Genetics; ^4^Pediatric Hematology and Transplant, KAMC/MNGHA, Riyadh, Saudi Arabia

##### **Correspondence:** W. M. Suwairi

*Pediatric Rheumatology 2023*, **21(Suppl 2):**PT058

**Introduction:** The E3 ubiquitin-protein ligase RNF31 OR HOIL-1-interacting protein (HOIP) is a ubiquitin ubiquitin-protein ligase component of the LUBAC complex. It conjugates linear polyubiquitin chains to substrates and plays a key role in NF-kappa-B activation and regulation of inflammation. LUBAC conjugates linear polyubiquitin to IKBKG and RIPK1 and is involved in activation of the canonical NF-kappa-B. Human HOIP is essential for the assembly and function of LUBAC complex and for various processes governing inflammation and immunity in both hematopoietic and nonhematopoietic cells. Bertrand Boissonet al. and Jean-Laurent Casanova reported 3 patients with HOIL-1 mutations associated with systemic autoinflammation, sever immunodeficiency and muscle amylopectinosis. (1,2 )

**Objectives:** Describe a novel mutation in RNF31 gene (HOIP) that is associated with recurrent infection, hypogammaglobulinemia, mental subnormal and sever systemic autoinflammatory disease and macrophage activation syndrome. We describe the long-term outcome (7 years) post HSCT

**Methods:** Currently, 9 years old Saudi girl who presented since age of 2 weeks with recurrent episodes of febrile illness associated with recurrent viral and bacterial infections (pneumonia, cervical lymphadenitis, URTI, and urinary tract infections. AT the age of 14 month, she presented with sever systemic inflammation, significant colitis diarrhea and laboratory evidence of Macrophage activation syndrome ( s,ferritin >5000 up to14000) disturbed coagulation, low ESR and cytopenia. Bone marrow was reactive and no hemophagocytic cells were seen. Patient responded well to high doses of systemic steroid with partial response to anti- IL=6 (Tocilizumab) and no response to anti IL-1 (anakinra)

The initial WG was not informative. Later one WES identified a homozygous non-synonymous mutation in RNF31 gene (c.1657G>A p.Glu553Lys). Both patents are heterozygous for this mutation. It is predicted to be damaging by SIFT and other softwares. The mutation is novel and has not been reported in dbSNPs or ExAC database as well as Saudi genome database. This was later on confirmed by Sanger sequencing.

The patient went for HSCT at the age of 21 months and the doner was her elder sister. The systemic autoinflammatory disease and immunodeficiency were well controlled post HCST. She was observed to have autistic behavior and IQ assessment confirmed moderate developmental delay and need for special education program. She also has polymorphisms in thyroglobulin gene, which have been reported to be associated with autoimmune thyroiditis. She is currently on L thyroxin 50 Mic. On November 2022 she was admitted to PICU in acute heart failure post viral URTI. The poor recovery of depressed myocardial function (EF to 25%) is most probably related to amlyplectinosis.

**Conclusion:** Inherited, complete deficiency of human HOIL-1, a component of the linear ubiquitination chain assembly complex (LUBAC), underlies autoinflammation, infections, and amylopectinosis. We report the clinical description and molecular analysis of a novel inherited disorder of the human LUBAC complex. HCST can be curative for the systemic autoinflammation and sever immunodeficiency but unfortunately not to amylopectinosis and mental subormality

**Patient Consent**

Yes, I received consent

**Disclosure of Interest**

None declared

**References**

1. Boisson, B. et al. Human HOIP and LUBAC deficiency underlies autoinflammation, immunodeficiency immunodeficiency, amylopectinosis, and lymphangiectasia. J. Exp. Med. 212, 939–951 (2015).
2. Boisson, B. et al. Immunodeficiency, autoinflammation and amylopectinosis in humans with inherited HOIL-1 and LUBAC deficiency. Nat. Immunol. 13, 1178-1186 (2012)
3. Kalpana Manthiram, Qing Zhou, Ivona Aksentijevich & Daniel L Kastner. Monogenic autoinflammatory diseases define new pathways in human innate immunity and inflammation. Nature immunology 18 832-842 (2017)

## PT059 Selective viral signaling and interleukin-1 (IL-1) receptor inhibition can maximize monocytic il-18 expression and drive an interferon (IFN)α-il-18-ifny hypersecretion axis in whole blood

### E. L. Verweyen, S. Schleifenbaum, V. Kienapfel, M. Schell, C. Hinze, D. Foell, C. Kessel

#### Pediatric Rheumatology & Immunology, University Hospital Muenster, Muenster, Germany

##### **Correspondence:** E. L. Verweyen

*Pediatric Rheumatology 2023*, **21(Suppl 2):**PT059

**Introduction:** Interleukin (IL) 18 is a main driver of hyperinflammation in Macrophage Activation Syndrome (MAS), which arises as serious complication in inflammatory diseases such as systemic juvenile idiopathic arthritis (SJIA). As demonstrated by us and others, type 1 interferon (T1IFN) signaling can drive IL-18 expression in both murine models and human cells.

**Objectives:** Previous data on the role of T1IFNs in IL-18 expression have been generated in context of TLR4 (LPS) stimulations. Here, we aimed to investigate the impact of viral stimuli, which are considered possible triggers of MAS, as well as IL-1 blocking therapies, potentially distorting an IL-1-T1IFN balance, on IL-18 expression and release as well as on hemophagocytosis.

**Methods:** Primary human monocytes or heparinized whole blood were stimulated with LPS or viral-infection mimicking ligands to TLR3 (p:IC), TLR7 (R837), TLR7/8 (R848), TLR8 (ssRNA40) or TLR9 (CpG), either alone or in combination, and with or without IL-1 blocking drugs (anakinra, canakinumab) or TLR8 inhibitor (CU-CPT9a). Cytokine release and/or gene expression was analyzed using multiplex bead arrays and qRT-PCRs. Phagocytosis of erythrocytes (hemophagocytosis) was assessed in monocyte-erythrocyte cocultures by flow cytometry following stimulations as described above and intracellular CD235a staining.

**Results:** Human monocyte stimulation by R848 induced rapid and massive IL-18 expression and release, which was driven through joined NFκB and T1IFN-signaling. Selective TLR8-inhibition or stimulation with ssRNA40 underscored the importance of TLR8 signaling in this context, and we further identified TLR8 signaling as a main driver of hemophagocytosis by inflammatory monocytes. In contrast, the exclusive exposure to TLR3, -4, -7 and -9 ligands resulted in significantly less monocytic IL-18 expression and release as well as hemophagocytic events. Intriguingly, combination of LPS and selected viral signals mounted significantly higher monocytic IL-18 expression compared to LPS stimulation alone. In these cell cultures, additional IL-1R1 inhibition by anakinra already resulted in some elevation of IL-18 expression, while in human whole blood experiments we observed LPS-stimulation in combination with anakinra, but not canakinumab, to strongly exacerbate LPS-induced IFN*α*, IL-18 and IFN*γ* release.

**Conclusion:** Our data demonstrate how specific viral insults or IL-1R1 blocking therapy in the context of inflammation can set off the T1IFN-IL-18-IFNγ axis as hallmark of hyperinflammation in MAS.

**Patient Consent**

Not applicable (there are no patient data)

**Disclosure of Interest**

E. Verweyen: None declared, S. Schleifenbaum: None declared, V. Kienapfel: None declared, M. Schell: None declared, C. Hinze Speaker Bureau with: Pfizer, D. Foell Grant / Research Support with: Novartis, Pfizer, Sobi, Speaker Bureau with: Chugai-Roche, Novartis, Sobi, C. Kessel Grant / Research Support with: Novartis, Consultant with: Novartis, Sobi

## PT060 Safety events reported in a cohort of patients with autoinflammatory diseases: eurofever experience

### Y. Vyzhga^1^, J. Frenkel^2^, A. Insalaco^3^, J. Anton^4^, I. Kone-Paut^5^, G. E. Legger^6^, G. Fabio^7^, M. Cattalini^8^, S. Kamphuis^9^, E. Hachulla^10^, K. Krause^11^, Z. Ekinci^12^, J. Sanchez-Manubens^13^, J. V. D. B. Van den Berg^14^, C. Herrera Mora^15^, P. Hissink Muller^16^, E. Labrador^17^, J. Potjewijd^18^, L. Carlini^19^, M. Bustaffa^20^, R. Caorsi^20^, N. Ruperto^19^, M. Gattorno^20^

#### ^1^UOC Reumatologia e Malattie Autoinfiammatorie, IRCCS Istituto Giannina Gaslini, Genoa, Italy; ^2^Department of Pediatric Immunology and Rheumatology, Wilhelmina Kinderziekenhuis, Utrecht, Netherlands; ^3^IRCCS Ospedale Pediatrico Bambino Gesù, Rome, Italy; ^4^Hospital Sant Joan de Déu, Universitat de Barcelona, Barcelona, Spain; ^5^National Referral Centre of Auto-Inflammatory Diseases and inflammatory amyloidosis, CEREMAIA, CHU de Biĉetre, APHP, University of Paris Sud, Paris, France; ^6^University Groningen, University Medical Center Groningen, Groningen, Netherlands; ^7^Fondazione IRCCS Ca' Granda Ospedale Maggiore Policlinico, Milan; ^8^Clinica Pediatrica dell'Universita' di Brescia, Brescia, Italy; ^9^ Sophia Children`s Hospital, Erasmus University Medical Centre, Rotterdam, Netherlands; ^10^ CHRU de LilleHospital Claude Huriez, Lille, France; ^11^ Charite University Hospital Berlin,, Berlin, Germany; ^12^Başkent University İstanbul Hospital, Istanbul, Türkiye; ^13^Hospital Parc Taulí de Sabadell, Barcelona, Spain; ^14^Emma Children`s Hospital, Amsterdam University Medical Centers, University of Amsterdam, Amsterdam, Netherlands; ^15^Hospital de Ninos Roberto Gilbert Elizalde, Guayaquil, Ecuador; ^16^Academisch Ziekenhuis Leiden, Leiden, Netherlands; ^17^San Pedro Hospital, Logrono, Spain; ^18^Maastricht University Medical Center, Maastricht, Netherlands; ^19^Gaslini Trial Centre; ^20^IRCCS Istituto Giannina Gaslini, Genoa, Italy

##### **Correspondence:** Y. Vyzhga

*Pediatric Rheumatology 2023*, **21(Suppl 2):**PT060

**Introduction:** The pathophysiology of AIDs is progressively updated, with new monogenic diseases being discovered every year and multiple treatment options available. The increasing experience has allowed us to estimate the risk of developing potential complications and safety issues associated with treatment. Therefore, prolonged drug use requires safety monitoring and tolerability of therapy in children affected by these disorders.

**Objectives:** The present study is aimed to evaluate the impact of safety events in the largest international registry for Autoinflammatory diseases, called Eurofever.

**Methods:** The Eurofever project was promoted in 2008 by the working group for autoinflammatory diseases of the Paediatric Rheumatology European Society (PRES). It started enrolment in 2009, in 2015, the registry was amended with the development of a longitudinal part and the inclusion of several items for the registration of the safety events. All safety events of moderate, severe, very severe intensity are reported in Eurofever, regardless of a possible suspected causal relationship to any therapies and according to the latest release of the Medical Dictionary for Regulatory Activities (MedDRA, Version 23.1).

**Results:** Of the 4552 patients with AIDs enrolled in the registry since 2009, 2464 displayed complete safety information. In 1499 of them retrospective data (from disease onset to the enrolment in the registry) were available. The remaining, 965 patients were followed longitudinally, with a mean follow-up of 22.7 months (range 7.33 – 31.07). The group of AEs that was the most frequently reported was infections and infestations (94; 19.6 %), gastrointestinal disorders (66; 13.8 %), followed by nervous system disorders (41; 8.6 %) and general disorders and administration site conditions (35; 7.3 %). 82/479 events were reported as serious, with the highest number of infections and infestations as the most reported (25.6 %), followed by surgical and medical procedures (15.8 %), immune system disorders (14.6 %), injury, poisoning, and procedural complications, and blood and lymphatic system disorders (4.9 %). 112 (23.4 %) safety reports of special interest were reported, with the absolute prevalence of infections and infestations (76/112). Among the other events of special interest, we retrieved seven episodes (5.9 %) of MAS and six (5.0 %) cases of malignancies. The highest number (43.2 %) of safety events came from 103 patients with FMF. Meanwhile, among the patients with rarer SAIDs, such as DIRA, Blau syndrome, PAPA, and DADA2, safety events were also reported.

99 drug-related AEs were reported and described by treating physicians at the context of their severity, relation with event onset, and action taken toward the medication. The highest number of drug-related AEs were related to colchicine (31/99 reports, 31.3 %) and were reported from 15 FMF, 3 SURF, 2 MKD, and 1 PFAPA and 1 Behcet disease patients. The overall incidence of AEs associated to biologic DMARDSs, was of 40 (40.4 %) events. Among patients treated with synthetic DMARDs, the highest number of AEs reports came from those receiving methotrexate, diagnosed with CRMO, undefined AID and Behcet`s disease.

**Conclusion:** This study is the first attempt to overview available safety information on the management of patients with AIDs. Results stress the importance of long-term monitoring of treatments given to patients with AIDs. Reporting safety is mandatory due to high possibility that some potential safety events may remain undetected or unreported.

**Patient Consent**

Yes, I received consent

**Disclosure of Interest**

None declared

## PT061 Description of the characteristics of the nailfold capillary structure in healthy children

### H. Adiguzel Dundar^1^, A. Adrovic^2^, S. Demir^3^, F. Demir^4^, F. Cakmak^5^, N. Aktay Ayaz^5^, B. Sözeri^4^, Y. Bilginer^3^, Ö. Kasapcopur^2^, E. Unsal^1^

#### ^1^Pediatric Rheumatology, Dokuz Eylül University Faculty of Medicine, Izmir; ^2^Pediatric Rheumatology, Istanbul University-Cerrahpaşa, Cerrahpaşa Medical Faculty, Istanbul; ^3^Pediatric Rheumatology, Hacettepe University Faculty of Medicine, Ankara; ^4^Pediatric Rheumatology, Health Sciences University, Ümraniye Training and Research Hospital; ^5^Pediatric Rheumatology, Istanbul University Çapa Medical Faculty, Istanbul, Türkiye

##### **Correspondence:** H. Adiguzel Dundar

*Pediatric Rheumatology 2023*, **21(Suppl 2):**PT061

**Introduction:** Nailfold capillaroscopy is the best method for the early diagnosis of connective tissue diseases, especially systemic sclerosis, and evaluation of microcirculation in children and adolescents. Although there are many studies to identify normal capillaroscopic findings in healthy adults, there are limited number of studies for normal reference ranges by age and gender in the children and adolescents.

**Objectives:** The aim is to define and standardize the nail bed capillary properties in healthy Turkish children and adolescents.

**Methods:** This multicenter cross-sectional pilot study included; 564 healthy children and adolescents from 5 pediatric rheumatology centers. Using the *Dino-Lite CapillaryScope 200 Pro / MEDL4N Pro capillaroscopy* device, two images of 1mm radial and ulnar edge were obtained from the 4^th^ fingernail bed of the non-dominant hand at 200x magnification. Capillary density, capillary morphology (*i.e.,* capillary tortuosity, capillary crossing, giant capillary, capillary meandering and branched capillary), microhemorrhage and avascular area were the parameters. Also 3 consecutive capillaries from each image; arterial and venous limb diameter, loop diameter, capillary length, capillary width, and intercapillary distance were measured. The children included in the study were classified according to their age; Group 1: 5-7 years, Group 2: 8-10 years, Group 3: 11-14 years, and Group 4: 15-17 years old.

**Results:** A total of 1128 images were obtained from 564 healthy children included in the study and 3384 capillary measurements were made. A positive correlation was determined between age and capillary density (p<0.001, R=0.450, CI95% 0.398-0.503). Capillary density was significantly lower in *Group* 1 than in other *Groups.* Although there was significant difference between the age groups in terms of arterial and venous limb diameter, loop diameter, capillary length, intercapillary distance; there was no significant difference between age groups and capillary width (Table I). There was significant difference between age groups and presence of dilated capillary, capillary tortuosity, avascular area. In total 1128 image evaluations, dilated capillary in 8.7%, Capillary tortuosity in 14.4%, crossed capillary in 43.1%, micro-hemorrhage in 2.7%, avascular area in 4.8% (Table II). There is no capillary presented with meandering, giant capillary or branched capillary. There was a good level of agreement between the researchers, as 10 cases with 60 capillaries were evaluated with a good level of agreement.

**Conclusion:** This is the first study to evaluate capillary morphology in healthy Turkish children. This study also adds that some special forms such as micro-hemorrhage and avascular area, which is always named as pathological in adult age, can be seen in healthy children. These data will be guiding in capillaroscopic studies in various patient groups, particularly in children with collagen vascular diseases.

**Patient Consent**

Not applicable (there are no patient data)

**Disclosure of Interest**

None declared

## PT062 Rheumai: nailfold videocapillaroscopy classification model supported by artificial intelligence

### F. Çakmak^1^, Y. Durusoy^2^, Ö. Akgün^1^, N. Aktay Ayaz^1^

#### ^1^Pediatric Rheumatology; ^2^Istanbul Faculty of Medicine, Istanbul, Türkiye

##### **Correspondence:** F. Çakmak

*Pediatric Rheumatology 2023*, **21(Suppl 2):**PT062

**Introduction:** Nailfold videocapillaroscopy (NVC) examination is a simple and non-invasive method used to evaluate microvascular architecture. Capillary density, capillary dimensions (total capillary width, arteriyal width, venous width, apical loop, intercapillary distance, capillary length), capillary arrangement, and capillaroscopic alterations (tortiosity, increased cross-section, meandering capillaries, branched capillaries, bushy capillaries, enlarged capillaries, giant capillaries, avascular area) are analyzed from the images of NVC. In children, capillary morphology is categorized into four categories as normal capillary morphology, minor abnormality, major abnormality, and scleroderma pattern.

**Objectives:** With this study, we aimed to enable clinicians who do not have sufficient experience in the field of capillaroscopy to make NVC morphological classification in patient follow-up with an artificial intelligence supported system and to use capillaroscopy more effectively in patient follow-up.

**Methods:** The archieve of NVC images of Istanbul University Istanbul Faculty of Medicine was analyzed retrospectively. Images were evaluated according to the Ingegnoli system by two different capillaroscopists. 92 normal morphologies, 20 minor disorders, 153 major disorders, and 17 scleroderma patterns, classified by consensus, were presented to artificial neural networks (ANNs). Since the minor disorder and normal morphology classes did not differ clinically, these two classes were combined as a single class, resulting in a total of 3 classes. 80% of the data was reserved for training and 20% for testing. The images were first resized to 512x512, and then right-left rotation, rotation and zoom data augmentation methods were applied to the images in the training data. Training was carried out for 30 epochs using the Resnet 101 model. The model was then evaluated on the untrained test data. Results were evaluated using performance measures such as accuracy, precision, recall, F1 score, and area under the ROC curve (AUC).

**Results:** In this study, capillary morphology was classified as normal capillary morphology, major disorder, and scleroderma pattern using the artificial intelligence method on capillaroscopy images. The model, which was evaluated on 66 data allocated for the test had 94% accuracy. Also, for the normal class, the AUC value is 0.962, the precision value is 0.90, the recall value is 1.00, the f1-score value is 0.95. For the major abnormality class, the AUC value is 0.946, the precision value is 0.97, the recall value is 0.90, f1-score value is 0.93. For the scleroderma pattern class, the AUC value was 0.987, the precision value was 1.00, the recall value was 0.86, and the f1-score value was 0.92.

**Conclusion:** Artificial intelligence assisted capillary morphology classification was able to place capillaroscopy images in the correct classes with high sensitivity. The lack of time to provide NVC evaluation during outpatient clinic controls will be overcome by saving time with this method.

**Patient Consent**

Yes, I received consent

**Disclosure of Interest**

None declared

## PT063 A practical approach to uveitis screening in children with juvenile idiopathic arthritis

### I. Foeldvari^1^, H. Petrushkin ^2,3^, M. Bohn^2,4^, A. L. Solebo^3,5^, S. T. Angeles-Han^6^, R. Bangsgaard^7^, J. Calzada-Hernández^8^, T. Constantin ^9^, J. de Boer^10^, J. Díaz-Cascajosa^11^, C. Edelsten^3^, M. Glerup^12^, H. Ingels^13^, J. Klotsche^14^, A. Marino^15^, E. Miserocchi^16^, E. Nordal^17^, R. K. Saurenmann^18,19^, G. Simonini^20^, N. Stuebiger^21^, J. Anton ^22^

#### ^1^Schön Klinik Hamburg Eilbek, Hamburg , Germany; ^2^Moorfields Eye Hospital NHS Foundation Trust; ^3^Great Ormond Street Hospital NHS Foundation Trust; ^4^West Hertfordshire Teaching Hospitals NHS Foundation Trust; ^5^University College London Institute of Ophthalmology, London , United Kingdom; ^6^Cincinnati Children’s Hospital Medical Center, University of Cincinnati, Cincinnati, United States; ^7^Department of Ophthalmology, Copenhagen University Hospital Glostrup/Rigshospitalet, Copenhagen, Denmark; ^8^Hospital Sant Joan de Déu, Universitat de Barcelona, Barcelona, Spain; ^9^Semmelweis University, Budapest, Hungary; ^10^UMC Utrecht, Utrecht, Netherlands; ^11^Ophthalmology Department, Hospital Sant Joan de Déu, Universidad de Barcelona, Barcelona, Spain; ^12^Department of Pediatrics, Aarhus University Hospital, Aarhus; ^13^Department of Paediatrics and Adolescent Medicine, Rigshospitalet, Copenhagen University Hospital, Copenhagen, Denmark; ^14^German Rheumatism Research Centre, Leibniz Institute, Berlin, Germany; ^15^ Unit of Pediatric Rheumatology, ASST G. Pini-CTO; ^16^Department of Ophthalmology, San Raffaele Scientific Institute, University Vita-Salute, Milan, Italy; ^17^Department of Pediatrics, University Hospital of North Norway and Pediatric Research Group, Tromsø, Norway, ^18^Department of Pediatrics, Kantonsspital , Winterthur; ^19^Department of Rheumatology, University Children's Hospital, Zurich, Switzerland; ^20^Anna Meyer Children's Hospital and University of Florence, Florence , Italy; ^21^Universitätsklinikum Hamburg-Eppendorf, Augenklinik, Hamburg , Germany; ^22^Division of Pediatric Rheumatology, Hospital Sant Joan de Déu, Universitat de Barcelona, Barcelona, Spain

##### **Correspondence:** I. Foeldvari

*Pediatric Rheumatology 2023*, **21(Suppl 2):**PT063

**Introduction:** Juvenile idiopathic arthritis (JIA) associated uveitis is the most frequent cause of uveitis in the paediatric population, and often presents as a silent chronic anterior uveitis, which can lead to blindness. Adherence to current screening guidelines is halted by difficulties in assessing specialised services. Additionally, current protocols are often complex and rely on the knowledge of disease biomarkers; information which may not be available to many paediatric ophthalmologists.

**Objectives:** The Multinational Interdisciplinary Working Group for Uveitis in Childhood (MIWGUC) identified the need to simplify JIA screening so paediatric ophthalmologists with no access to detailed information on JIA subtype can carry out safe screening with confidence, while also enabling other eye care professionals to play a role if needed.

**Methods:** The group consisted of 10 rheumatologists and 8 ophthalmologists. A consensus meeting took place on January 2023 in Barcelona, Spain. A summary of the current evidence for JIA-associated uveitis screening was presented to the expert panel. Nominal group technique was used to reach consensus.

**Results:** The need for a practical but safe guideline for uveitis screening was identified by the panel. At the end of the discussion, three screening recommendations were proposed and approved by the voting members. They represent a standardised approach to JIA screening and only take into account the JIA age of diagnosis to determine the screening interval until adulthood.

**Conclusion:** By removing the need for the knowledge of the JIA subtype, ANA positivity or treatment status, the MIWGUC recommendations can be more easily implemented in the clinical practice by a paediatric ophthalmologist, or other trained eye care professionals, who may not work directly with rheumatology or uveitis specialist. This holds particular significance in areas with limited resources. However, the proposed protocol is less tailored to the individual than those it references.

**Patient Consent**

Not applicable (there are no patient data)

**Disclosure of Interest**

None declared

**Reference**s

1. Walscheid K, Klotsche J, Tappeiner C, *et al.* Adherence to ophthalmological screening recommendations and course of uveitis in children with juvenile idiopathic arthritis: data from the Inception Cohort of Newly diagnosed patients with JIA (ICON-JIA) study. *Clin Exp Rheumatol* 2020;**38**:792–8. doi:32105591
2. Heiligenhaus A, Niewerth M, Ganser G, *et al.* Prevalence and complications of uveitis in juvenile idiopathic arthritis in a population-based nation-wide study in Germany: suggested modification of the current screening guidelines. *Rheumatology* 2007;**46**:1015–9. doi:10.1093/rheumatology/kem053
3. Angeles-Han ST, Ringold S, Beukelman T, *et al.* 2019 American College of Rheumatology/Arthritis Foundation Guideline for the Screening, Monitoring, and Treatment of Juvenile Idiopathic Arthritis–Associated Uveitis. *Arthritis Care Res (Hoboken)* 2019;**71**:703–16. doi:10.1002/acr.23871
4. Leinonen S. A Nordic screening guideline for juvenile idiopathic arthritis-related uveitis. *Acta Ophthalmol* Published Online First: 2 December 2022. doi:10.1111/aos.15299

## PT064 Decoding the t-cell receptor repertoire in chronic arthritis across different ages, disease groups, and t-cell subsets

### M. Ha^1^, V. Van Deuren^2^, G. Elias^3^, M. Kuznetsova^1^, E. Bartholomeus^1^, N. de Vrij^2^, J. Dehoorne^4^, T. Renson^4^, E. Geens^5^, N. Aerts^5^, R. Wittoek^4,5^, C. Heusdens^6^, E. De Wachter^7^, S. Peeters^1^, M. Domagalska^8^, I. Maes^8^, A. Suls^9^, E. Lion^1^, S. Vanhee^10^, W. Adriaensen^8^, K. Mullan^2^, R. Joos^5^, K. Laukens^2^, P. Meysman^2^, B. Ogunjimi^1,5,6^

#### ^1^Faculty of Medicine and Health Sciences; ^2^Department of Mathematics and Computer Science, University of Antwerp, Antwerp; ^3^GSK, Rixensart, ^4^Ghent University Hospital, Ghent; ^5^Ziekenhuis Netwerk Antwerpen; ^6^Antwerp University Hospital, Antwerp; ^7^Brussels University Hospital, Brussels; ^8^Institute of Tropical Medicine; ^9^Center of Medical Genetics, University of Antwerp, Antwerp; ^10^University of Ghent, Ghent, Belgium

##### **Correspondence:** M. Ha

*Pediatric Rheumatology 2023*, **21(Suppl 2):**PT064

**Introduction:** T-cells are important actors in the pathophysiology of chronic autoimmune arthritis and are abundantly present both in synovial fluid and synovial tissue in different arthritic diseases, such as juvenile idiopathic arthritis (JIA), rheumatoid arthritis (RA), psoriatic arthritis (PsA), and HLA-B27^+^ spondyloarthritis (SpA). T-cell receptors (TCRs) are pivotal in recognizing antigenic peptides and driving T-cell response against the antigens. Current research indicates that there exists an overlap between synovial fluid TCRs in adult SpA patients against self-antigens and microbial antigens [1]. The molecular mimicry theory hypothesizes that in these HLA-B27^+^ SpA patients, T-cells elicited after bacterial infection may target self-antigens presented on synovial tissue [2]. Similarly, recent research has indicated that CD8^+^ T-cells specific to Epstein-Barr virus (EBV), cytomegalovirus (CMV), and influenza virus were present in the synovial fluid of RA patients [3].

**Objectives:** The objectives of this study are (i) investigate whether TCRs against viral epitopes are present and enriched in the synovial fluid of chronic arthritis patients; (ii) examine whether there is TCR overlap between the different T-cell subsets (e.g., between CD4^+^, CD8^+^, and regulatory T-cells) in the same patient and also between different patients; (iii) assess how synovial fluid cell phenotype distribution differs between patient groups and across ages.

**Methods:** 21 patients were recruited in rheumatology centres in Flanders: 4 patients with oligo-articular JIA, 4 HLA-B27^+^ enthesitis-related JIA patients (JIA-ERA), 2 juvenile PsA patients, 4 adult PsA patients, 1 adult HLA-B27^+^ SpA patient, 3 RA patients, and 3 Lyme arthritis patients. Their synovial fluid was collected and processed into synovial fluid mononuclear cells (SFMC). Single-cell RNA-seq and TCR-seq were performed on the SFMC. Bulk TCR-seq was performed on the CD4^+^, CD8^+^, and regulatory T-cell fractions.

**Results:** Our data indicate that JIA-ERA and Lyme arthritis patients had the least TCR diversity compared to other arthritis groups (Shannon entropy index = 0.96 and 0.92 for JIA-ERA and Lyme arthritis, respectively), which agrees with their high number of expanded TCR clonotypes (more than 600 largely and hyperexpanded clones). Among all the identified T-cell phenotypes, Th1/Th17 cells had the most clonal expansion with more than 250 moderately expanded clonotypes (i.e., frequency 5-10), more than 500 largely expanded clonotypes (i.e., frequency 10-50), and more than 100 hyperexpanded clonotypes (i.e., frequency > 50). Th1/Th17 cells were also the dominant population among all patient groups, having the highest proportion out of the total T-cell count in JIA-ERA and PsA patients (31% and 19%, respectively). Furthermore, TCRs against viruses like EBV, CMV and even SARS-CoV-2 are present in the SFMC of paediatric and adult arthritis patients.

**Conclusion:** The findings in this study provide insights into how TCR repertoires differ between arthritis groups, patients’ ages, T-cell subsets, and whether viral/bacterial infection contributes to arthritis onset and flares. Such knowledge will greatly contribute to the understanding of T-cells' and TCRs' participation in the underlying pathophysiology of arthritis.

**Patient Consent**

Yes, I received consent

**Disclosure of Interest**

None declared

**References**

1. Yang, X. et al. *Nature* **2022**, *612*, 771–777.
2. Bowness, P. *Annu Rev Immunol* **2015**, *33*, 29–48.
3. Zheng, Z. et al. *Ann Rheum Dis* **2023**, *82*, 438–440.

## PT065 Treat-to-target in Polyarticular-Onset Jia (PJIA) – first data of the prokind-rheuma project

### G. Horneff^1^, K. Minden^2,3^, K. Tenbrock^4^, D. Föll^5^, K. Vollbach^4^, A. Klein^1^, J. P. Haas^6^, D. Windschall^7^, T. Kallinich^2^, F. Weller^8^, S. Mrusek^9^, K. Mönkemöller^10^, M. Hufnagel^11^, I. Földvari^12^, A. Hospach^13^, R. Trauzeddel^14^, P. Oommen^15^, C. Schütz^16^, N. Brück^16^, J. Kümmerle-Deschner^17^, J. Brunner^18^, F. Dressler^19^, J. Klotsche^3^

#### ^1^Asklepios Kinderklinik Sankt Ausgustin, Sankt Ausgustin; ^2^Department of Pediatric Respiratory Medicine, Immunology and Critical Care Medicine, Charité Universitätsmedizin Berlin; ^3^PA Epidemiology, Deutsches Rheuma-Forschungszentrum, Berlin; ^4^Klinik für Kinder- und Jugendmedizin, Universitätsklinikum Aachen, Aachen; ^5^Klinik für Pädiatrische Rheumatologie und Immunologie, Universitätsklinik Münster, Münster; ^6^Deutsches Zentrum für Kinder- und Jugend-rheumatologie, Garmisch-Partenkirchen; ^7^Klinik für Kinder- und Jugendrheumatologie, St. Josef-Stift Sendenhorst, Sendenhorst; ^8^Eltern-Kind-Zentrum Prof.Hess, Klinikum Bremen-Mitte , Bremen; ^9^Kinderarztpraxis, Baden-Baden; ^10^Kinderkrankenhaus der Stadt Köln, Köln; ^11^Sektion Päd. Infektiologie und Rheumatologie, Universitätsklinik Freiburg, Freiburg; ^12^Hamburger Zentrum für Kinder- und Jugendrheumatologie, Hamburg; ^13^Kinderrheumatologie, Klinikum Stuttgart - Olgahospital, Stuttgart; ^14^Klinik für Kinder- und Jugendmedizin, Helios Klinkum Berlin-Buch, Berlin; ^15^Klinik für Kinder- und Jugendmedizin, Med. Einrichtungen der Heinrich-Heine-Universität Düsseldorf, Düsseldorf; ^16^Klinik für Kinder- und Jugendmedizin, Universitätsklinikum Carl Gustav-Carus, Dresden; ^17^Klinik für Kinder- und Jugendmedizin, Zentrum für Kinder- und Jugendrheumatologie, arcT, Universitätsklinik Tübingen, Tübingen, Germany; ^18^Kinder- und Jugendheilkunde, Medizinische Universität Innsbruck, Innsbruck, Austria; ^19^Kinderklinik - Rheumaambulanz, Medizinische Hochschule Hannover, Hannover, Germany

##### **Correspondence:** G. Horneff

*Pediatric Rheumatology 2023*, **21(Suppl 2):**PT065

**Introduction:** There is growing evidence that effective therapy with early achievement of inactive disease improves the outcome of JIA. Therefore, a treat-to-target approach with the goal of achieving inactive disease within the first 12 months of treatment is recommended [1]. To support the implementation of such an approach in clinical practice, four consensus-based treatment pathways based on current clinical practice for pJIA were proposed [2].

**Objectives:** To investigate whether the recommended therapy pathways for pJIA are followed in daily routine and what the outcomes are.

**Methods:** ProKind-Rheuma is a multicentre, prospective, non-interventional observational study. Patients with newly diagnosed pJIA were enrolled from January 2020 to June 2022 and followed prospectively for up to 15 months. Physician- and parent-reported data were collected in a standardized way up to five times (e.g., disease activity with the cJADAS-10, functional limitations with the Childhood Health Assessment Questionnaire (CHAQ), quality of life with the PedsQL 4.0); data from patients with a follow-up time (FU) of more than 9 months were included in this analysis.

**Results:** A total of 170 pJIA patients (RF- polyarthritis: n=128, RF+ polyarthritis: n=23, RF unknown: n=19) were recruited 1.0 months (SD 1.8) after diagnosis from 17 paediatric rheumatology centres. Data on 12-month-FU were available for 83 patients. Patients improved significantly over time: mean cJADAS10 decreased from 16.4±6.1 to 2.8±3.6, CHAQ from 0.9±0.8 to 0.3±0.5 and PedsQL increased from 65.9±21.8 to 85.9±13.8. At the 6- and 12-month-FU, 51% and 59%, respectively, had inactive disease and 70% and 77%, respectively, had at least minimally active disease according to the 2021 cJADAS-10 cutoffs [3].

Approximately two-thirds of patients had been treated according to the proposed treatment pathways, with n=33 (40%) treated with MTX and, if required, an additional biologic (pathway or group I) or biologic monotherapy (group II) from month 3 at the earliest. The pathway with additional high-dose iv methylprednisolone pulse therapy (group III) and the pathway with additional intra-articular glucocorticoids in >4 joints (group IV) were followed in n=14 (17%) and n=6 (7%) patients, respectively. In group I/II, 24 (73%) achieved minimally active disease and 18 (55%) achieved cJADAS remission, in group III 8 (57%) and 6 (43%), and in group IV 3 (50%) and 1 (17%). At last FU, 15 patients (48%) in group I/II were taking biologics, 3 (60%) in group III and 6 (46%) in group IV. The patients, n=30 (36%), for whom no clear allocation to a treatment pathway was possible, had inactive disease in 52% and at least minimally active disease in 71% at the 12-month FU. About half (53%) were receiving biologics at this time.

**Conclusion:** A treat-to-target approach is currently being used for most patients with polyarticular JIA. In the majority of those, the disease was clinically inactive or minimally active after about 12 months of treatment. Further analysis is needed to confirm the results, to determine who is not achieving the treatment goal and why, and to determine which treatment pathways are most effective.

**Patient Consent**

Yes, I received consent

**Disclosure of Interest**

G. Horneff Speaker Bureau with: Novartis, MSD, Pfizer, Roche, Sanofi, Sobi, Biogen, K. Minden Speaker Bureau with: Novartis, Amgen, K. Tenbrock: None declared, D. Föll: None declared, K. Vollbach: None declared, A. Klein: None declared, J. Haas: None declared, D. Windschall: None declared, T. Kallinich: None declared, F. Weller: None declared, S. Mrusek: None declared, K. Mönkemöller: None declared, M. Hufnagel: None declared, I. Földvari: None declared, A. Hospach: None declared, R. Trauzeddel: None declared, P. Oommen: None declared, C. Schütz: None declared, N. Brück: None declared, J. Kümmerle-Deschner: None declared, J. Brunner: None declared, F. Dressler: None declared, J. Klotsche: None declared

**Reference**s

1. Ravelli A, Consolaro A, Horneff G, et al. Ann Rheum Dis 2018;77:819-28.
2. Horneff G, Klein A, Ganser G, et al. Pediatr Rheumatol Online J 2017;15:78.
3. Trincianti C, Van Dijkhuizen EHP, Alongi A, et al.; Arthritis Rheumatol 2021;73:1966-75.

## PT066 Psoriasis spectrum arthritis in children: a multicenter observational series

### S. Pastore^1^, A. Tommasini^1,2^, A. Pin^1^, A. Taddio^1,2^, F. Corona^2^, G. Martini^3^, A. Meneghel^3^, F. Tirelli^3^, F. dell’Apa^3^, M. Fastiggi^4^, M. Cappella^4^, N. Possemato^5^, F. Zulian^3^

#### ^1^Institute for Maternal and Child Health IRCCS "Burlo Garofolo"; ^2^University of Trieste, Trieste; ^3^Department of Woman and Child Health, Pediatric Rheumatology Unit, University of Padova, Padua; ^4^Pediatric Rheumatology, Ospedale di Reggio Emilia; ^5^UO Reumatologia, ASMN-IRCCS Reggio Emilia, Reggio Emilia, Italy

##### **Correspondence:** S. Pastore

*Pediatric Rheumatology 2023*, **21(Suppl 2):**PT066

**Introduction:** The definition of psoriatic arthritis in pediatric patients (JPsA) is controversial.

**Objectives:** to describe clinical and laboratory characteristics and response to therapy in patients with JPsA.

**Methods:** retrospective multicentre observational study of pediatric patients meeting the ILAR criteria for psoriatic arthritis or criteria for enthesitis related arthritis (ERA) with psoriasis and/or family history of psoriasis in a first-degree relative. Clinical and laboratory data were collected in a structured anonymized database. Disease remission was defined according to the Wallace criteria.

**Results:** We included 71 patients (44 F, 27 M) followed at 3 Italian pediatric rheumatology centers. Disease onset occurred at a median age of 10 years (two peaks, 8 and 14 years). At the disease onset , Erythrocyte Sedimentation Rate (ESR) and/or C Reactive Protein (CRP) were elevated in 61% of patients (ESR in 58%, CRP in 34%). Antinuclear Antibodies (ANA) were positive in 31 of 66 tested patients. HLA B-27 was present in 10 of 50 tested patients. Disease remission was obtained in 69/71 patients, whilst two patients had only partial response due to intractable uveitis. In 20/71 (28.1%) disease remission was obtained with intraarticular steroid injections. A conventional disease modifying antirheumatic drugs (DMARDs) was effective in 23/71 patients (32.4%). Biological agents were needed to obtain disease remission in 36/71 patients (50.7%)

55 patients (77.5%) met the ILAR criteria for JPsA diagnosis, whilst 16 were undifferentiated (8 HLA B27 positive males with disease onset over 6 years of age and 8 with ERA). Dactylitis and/or tenosynovitis were more common in JPsA than in undifferentiated cases but the difference was not significant (64 vs 55%).

Unsupervised principal component analysis based on clinical features allowed identifying two cluster of disease respectively composed of 45 and 26 patients. The first cluster was characterized by early onset (before 12 years), predominant oligoarticular pattern with uveitis, familial history of psoriasis or psoriatic arthritis. The second group was characterized by predominant onset in adolescence, polyarticular pattern and psoriasis appearance often before the onset of arthritis. Tenosynovitis, enthesitis and axial involvement were also more common in the second cluster (50 vs 22; 31 vs 9%; 15% vs 0).

**Conclusion:** Many pediatric patients with arthropathies associated with psoriasis or family history of psoriasis don’t meet classification criteria for JPsA but display clinical overlap with it. Two major clusters have been identified among psoriasis-related arthropathies, based on the age at onset, number of involved joints and presence of psoriasis. Further studies will clarify whether the definition of these clusters may have any impact on future classifications of JPsA.

**Patient Consent**

Not applicable (there are no patient data)

**Disclosure of Interest**

None declared

## PT067 Why is it time to include ultrasound-detected tenosynovitis in the definition of JIA?

### A. I. Rebollo-Giménez^1,2^, L. Carlini^1^, E. van Dijkhuizen^3^, S. Magni-Manzoni ^4^, S. Lanni^5^, F. Casabona ^6^, A. Feliciello^6^, M. Dellepiane^1^, C. Malattia^1^

#### ^1^Unit of Rheumatology and Autoinflammatory Diseases, IRCCS Istituto Giannina Gaslini, Genoa, Italy; ^2^Universidad Autónoma de Madrid, Madrid, Spain; ^3^Department of Pediatric Immunology and Rheumatology, Wilhelmina Children's Hospital, Utrecht, Netherlands; ^4^Rheumatology Division, IRCCS bambino Gesù Children's Hospital, Rome; ^5^Pediatric Rheumatology, Fondazione IRCCS Cà Granda Ospedale Maggiore Policlinico, Milan; ^6^Dipartimento Di Neuroscienze, Riabilitazione, Oftalmologia, Genetica E Scienze Materno-Infantili (Dinogmi), Università Degli Studi Di Genova, Genoa, Italy

##### **Correspondence:** A. I. Rebollo-Giménez

*Pediatric Rheumatology 2023*, **21(Suppl 2):**PT067

**Introduction:** Recent studies have shown that ultrasound (US) detected tenosynovitis (TS) is frequent in patients with Juvenile Idiopathic Arthritis (JIA) and may be a very early feature of JIA at the disease onset. Currently, the diagnosis of JIA is based on the finding of arthritis and does not include inflammation of periarticular structures such as tendons. To date, the diagnostic significance of TS in patients with JIA is still unknown.

**Objectives:** To determine the diagnostic significance of ultrasound findings of TS in patients with JIA.

**Methods:** We conducted an observational multicentric cross-sectional study including JIA patients with disease onset between 2013 and 2016. Clinical characteristics, US scan, laboratory test results and Juvenile Arthritis Disease Activity Score for 10 joint counts (JADAS10) were collected in an encrypted database. Patients performed a musculoskeletal ultrasound scan of the wrists, fingers, and ankles for the assessment of tenosynovitis according to the OMERACT (Outcome Measures in Rheumatology) US definitions. We ran a logistic model with TS as response variable to identify clinical and demographic features associated to TS.

**Results:** Musculoskeletal US was performed in 167 new-onset JIA patients. US revealed the presence of synovitis in 101/167 patients (60.5%) while 72/167 patients had TS (43.1%) in the scanned joints. Nine out of 72 (12.5%) patients presented with isolated TS without joint involvement.

Ankle TS was detected in 81% (58/72) of the patients that presented TS whereas the wrist/hand’s tendons were affected in 19.4 % (14/72) of these patients.

At the bivariate analysis, the predictors of having TS at disease onset were: increased acute phase reactants, high baseline active joint counts, high disease activity based on JADAS score, and RF- polyarticular and oligoarticular ANA negative JIA ILAR category.

In the final multivariate analysis, RF- polyarticular JIA category was an independent predictor of TS (OR: 3.01; 1.11-8.43 p-value 0.031). In addition, higher values of JADAS corresponded to a higher risk of having TS (an increase of 1 in the JADAS-10 score corresponded to an increase of 11% in the risk of having TS, p-value 0.0019).

**Conclusion:** A significant percentage of JIA patients showed TS at disease onset. TS may be present as an isolated manifestation without joint involvement. Patients with polyarticular JIA and high disease activity had a significant higher rate of tendons involvement. Our results suggest a potential role of TS as a marker of severity at the disease onset. Longitudinal studies are needed to verify the prognostic value of TS on the disease course.

**Patient Consent**

Not applicable (there are no patient data)

**Disclosure of Interest**

None declared

**Reference**

1. Rooney ME, McAllister C, Burns JF. Ankle disease in juvenile idiopathic arthritis: ultrasound findings in clinically swollen ankles. J Rheumatol. 2009 Aug;36(8):1725-9. doi: 10.3899/jrheum.080508. Epub 2009 May 1. PMID: 19411390.

## PT068 Efficacy and safety of secukinumab in Juvenile idiopathic arthritis: interim results from the extension of the junipera trial

### N. Ruperto^1^, I. Foeldvari^2^, E. Alexeeva^3^, N. A. Ayaz^4^, G. Schulert^5^, S. Ozen^6^, A. Popov^7^, A. V. Ramanan^8^, C. Scott^9^, B. Sozeri^10^, E. Zholobova^11^, S. Chakraborty^12^, X. Zhu^13^, R. Martin^13^, S. Whelan^14^, S. Kaur^15^, L. Pricop^13^, D. J. Lovell^5^, A. Martini^16^, H. Brunner^5^ on behalf of PRINTO and PRCSG investigative sites

#### ^1^IRCCS Istituto Giannina Gaslini, UOSID Centro Trial, Genova, Italy; ^2^Hamburger Zentrum fuer Kinder und Jugendrheumatologie, Hamburg, Germany; ^3^National Scientific and Practical Center of Children's Health, Moscow, Russian Federation; ^4^Department of Pediatric Rheumatology, Faculty of Medicine, Istanbul University-Cerrahpasa, Istanbul, Türkiye; ^5^UC Department of Pediatrics, Cincinnati Children's Hospital, University of Cincinnati, Cincinnati, OH, United States; ^6^Hacettepe University Medical Faculty, Ankara, Türkiye; ^7^Urals State Medical University Ekaterinburg, Sverdlovsk, Russian Federation; ^8^Bristol Royal Hospital for Children & Translational Health Sciences, University of Bristol, Bristol, United Kingdom; ^9^Department of Paediatric Rheumatology, Red Cross War Memorial Children’s Hospital, University of Cape Town, Cape Town, South Africa; ^10^Division of Pediatric Rheumatology, University of Health Sciences, Ümraniye Training and Research Hospital, Istanbul, Türkiye; ^11^First Moscow State Medical University n.a. I.M.Sechenov, Moscow, Russian Federation; ^12^IQVIA, Durham, North Carolina; ^13^Novartis Pharmaceutical Corporation, East Hanover, NJ, United States; ^14^Novartis Ireland Ltd, Dublin, Ireland; ^15^Novartis Pharma AG, Basel, Switzerland; ^16^Università di Genova, Genova, Italy

##### **Correspondence:** N. Ruperto

*Pediatric Rheumatology 2023*, **21(Suppl 2):**PT068

**Introduction:** Secukinumab has demonstrated efficacy and safety in patients with enthesitis-related arthritis (ERA) and juvenile psoriatic arthritis (JPsA) categories of juvenile idiopathic arthritis (JIA) for up to 2 years.^1^ After completion of a 2-year primary study (JUNIPERA), a long-term extension (LTE) study was conducted to evaluate the continued efficacy and safety of secukinumab in patients with ERA and JPsA.

**Objectives:** Here we report the interim efficacy and safety results of the LTE study.

**Methods:** In the primary study, a total of 86 patients (2 to <18 years of age) received secukinumab up to week 12 in the open-label (OL) period.^1^ JIA American College of Rheumatology (ACR)30 responders at week 12 (n=75) were subsequently randomised to secukinumab (n=37) or placebo (n=38) up to week 100 in study period 2. Those who flared after randomisation (secukinumab, n=10; placebo, n=21) received OL secukinumab in study period 3 up to week 100.^1^ A total of 55 out of 61 patients who had completed the primary study consented to enter the LTE study, among which 54 patients received secukinumab (s.c.) (75/150 mg in patients <50/ ≥50 kg) every 4 weeks up to 4 years. Patients whose signs and symptoms were not fully controlled, as judged by the investigator in the LTE study, could have dose escalation of their secukinumab dose from 75 mg to 150 mg or 150 mg to 300 mg. Median Juvenile Arthritis Disease Activity Scores (JADAS)-27 were presented up to week 156 for efficacy, and adverse events (AEs) and serious AEs were presented for the entire treatment period up to the cut-off date (02-Feb-2022).

**Results:** The median (Q1, Q3) JADAS-27 improved from 14 (9.3, 19.0) at baseline to 0.7 (0.0, 1.5) at week 104 for patients receiving secukinumab in the primary study, which was maintained in the LTE study till week 156 (0.7 [0.2, 2.8]). There was a notable reduction in median JADAS-27 from high disease activity at baseline to minimal and inactive disease activity at week 12 and week 104, respectively. The inactive disease status achieved in the primary study was sustained till week 156 in the LTE study. A total of 19 patients had dose escalation in the LTE study: 8 patients from 75 mg to 150 mg and 11 patients from 150 mg to 300 mg. The overall exposure-adjusted incidence rate per 100 patient-years (PY) of treatment-emergent AEs was 98.4 PY in the entire JIA population. The most commonly reported AEs were nasopharyngitis (n=9, 16.7%) and arthralgia (n=8, 14.8%). One major adverse cardiovascular event, not related to the study drug, and 2 cases of uveitis were reported. No cases of Crohn’s disease or deaths were reported, and no patient discontinued treatment due to an AE.

**Conclusion:** With secukinumab treatment, the JADAS-27 inactive disease status was sustained from week 104 to week 156 in patients with JIA who had completed the 2-year primary study and enrolled in the LTE study. Safety data were consistent with adult and paediatric indications, with no new or unexpected safety signals.

**Trial registration identifying number:** NCT03769168

**Patient Consent**

Not applicable (there are no patient data)

**Disclosure of Interest**

N. Ruperto Grant / Research Support with: BMS, Eli-Lilly, GlaxoSmithKline, F Hoffmann-La Roche, Janssen, Novartis, Pfizer, Sobi, Consultant with: Ablynx, AstraZeneca-Medimmune, Bayer, Biogen, Boehringer, Bristol Myers and Squibb, Celgene, Eli-Lilly, EMD Serono, Glaxo Smith and Kline, Hoffmann-La Roche, Janssen, Merck, Novartis, Pfizer, R-Pharma, Sinergie, Sobi and UCB, Speaker Bureau with: Ablynx, AstraZeneca-Medimmune, Bayer, Biogen, Boehringer, Bristol Myers and Squibb, Celgene, Eli-Lilly, EMD Serono, Glaxo Smith and Kline, Hoffmann-La Roche, Janssen, Merck, Novartis, Pfizer, R-Pharma, Sinergie, Sobi and UCB, I. Foeldvari Consultant with: Novartis, BMF, Bayer, Genentech, Sanofi, Abbvie, Chugai, Medac, BMS, Pfizer, E. Alexeeva Grant / Research Support with: Novartis, Pfizer, Sanofi, MSD, AMGEN, Eli Lilly, Roche, Speaker Bureau with: Novartis, Pfizer, Sanofi, MSD, AMGEN, Eli Lilly, Roche, N. A. Ayaz: None declared, G. Schulert Grant / Research Support with: Novartis, Consultant with: Novartis, S. Ozen: None declared, A. Popov: None declared, A. V. Ramanan Speaker Bureau with: Roche, Sobi, Eli Lilly, UCB, Novartis, C. Scott: None declared, B. Sozeri: None declared, E. Zholobova Grant / Research Support with: Pfizer, Novartis, Speaker Bureau with: Abbvie, Pfizer, Roche, Novartis, S. Chakraborty Shareholder with: IQVIA, Employee with: IQVIA, X. Zhu Shareholder with: Novartis, Employee with: Novartis, R. Martin Shareholder with: Novartis, Employee with: Novartis, S. Whelan Shareholder with: Novartis, Employee with: Novartis, S. Kaur Shareholder with: Novartis, Employee with: Novartis, L. Pricop Shareholder with: Novartis, Employee with: Novartis, D. Lovell Consultant with: AstraZeneca, Wyeth, Amgen, Abbott, Pfizer, Hoffmann-La Roche, Novartis, UBC, Janssen, GlaxoSmithKline, Boehringer Ingelheim, Celgene, Bristol Myers Squibb, AbbVie, Paid Instructor with: DSMB member: Forest Research, NIH-NIAMS, Canadian Arthritis Society, Speaker Bureau with: Abbott, Novartis, A. Martini Consultant with: Eli Lilly, EMD Serono, Janssen, Novartis, Pfizer, Abbvie, Speaker Bureau with: Eli Lilly, EMD Serono, Janssen, Novartis, Pfizer, Abbvie, H. Brunner Grant / Research Support with: Bristol-Myers Squibb, Eli Lilly, GlaxoSmithKline, F. Hoffmann-La Roche, Janssen, Novartis, and Pfizer, Consultant with: Aurinia, Abbvie, AstraZeneca-Medimmune, Biogen, Boehringer, Bristol-Myers Squibb, Celgene, Eli Lilly, EMD Serono, GlaxoSmithKline, F. Hoffmann-La Roche, Merck, Novartis, R-Pharm, Sanofi, Pfizer, Speaker Bureau with: Pfizer, Roche and GlaxoSmithKline

**Reference**

1. Brunner HI, et al. *Ann Rheum Dis*. 2023;82(1):154-160.

## PT069 Synovial fluid potentiates local fibroblasts to drive inflammatory monocytes in oligoarticular juvenile idiopathic arthritis

### T. Schmidt^1,2^, A. Mossberg^1,2^, E. Berthold^1,2^, P. Król^1,2^, A. A. Bengtsson^3^, F. Kahn^4^, B. Månsson^1^, R. Kahn^1,2^

#### ^1^Department of Pediatrics, Clinical Sciences Lund; ^2^Wallenberg Center for Molecular Medicine; ^3^Department of Rheumatology, Clinical Sciences Lund; ^4^Department of Infection Medicine, Clinical Sciences Lund, Lund University, Lund, Sweden

##### **Correspondence:** T. Schmidt

*Pediatric Rheumatology 2023*, **21(Suppl 2):**PT069

**Introduc**t**ion:** Little is known of the mechanisms driving monocyte and neutrophil activation in oligoarticular juvenile idiopathic arthritis (oJIA). Synovial fibroblasts (S-Fib) are recognized as key drivers of inflammation in adult arthritis, but little is known of their role in oJIA.

**Objectives:** To explore if S-Fib from oJIA patients induce activation in healthy monocytes and neutrophils.

**Methods:** S-Fib were isolated from the synovial fluid (SF) of n=10 patients with oJIA. Isolated S-Fib were primed or not with pooled cell-free SF (n=8-10) to mimic a disease relapse. They were subsequently analyzed by mass spectrometry, cytokine production and their ability to induce migration of monocytes and neutrophils. The influence of S-Fib on the healthy monocyte- and neutrophil phenotypes and function was studied in co-culture systems. These cells were subsequently analyzed using various assays believed to reflect key pathological events, such as cytokine production, T-cell activation, and reactive oxygen species (ROS) production.

**Results:** Compared to S-Fib alone, priming of S-Fib with SF induced production of IL-6 (p=0.0402), IL-8 (p=0.0344), and enhanced immune cell migration to supernatants (p<0.0346). Furthermore, S-Fib induced an inflammatory phenotype in healthy monocytes, such as production of inflammatory cytokines (p<0.0209) and an increased ability to induce T-cell proliferation (p=0.0351). Importantly, these effects were further enhanced by priming of the S-Fib prior to co-culture. Moreover, the effect of S-Fib co-culture on neutrophils were minor, although co-culture with SF-primed S-Fib prevented apoptosis (p=0.0069). Finally, S-Fib supernatants did not induce the observed monocyte phenotype, but monocytes had increased adhesion to SF-primed S-Fib (p<0.0001), suggesting a role of cell-cell contact.

**Conclusion:** Our data show a role for S-Fib in driving inflammation in childhood-onset arthritis by inducing pro-inflammatory monocytes and pro-longing neutrophil survival, processes potentiated by inflamed SF. These data further support that targeting cell-cell interactions could be a viable option to explore for novel treatment strategies in arthritis.

**Patient Consent**

Not applicable (there are no patient data)

**Disclosure of Interest**

None declared

## PT070 Validation of a novel ultrasound scoring system for the evaluation of pediatric finger arthritis

### P. Vega-Fernandez^1,2^, K. Rogers^2^, M. Quinlan-Waters^2^, A. Cassedy^3^, A. Meyers^2,4^, T. V. Ting^1,2^

#### ^1^Pediatric, University of Cincinnati; ^2^Cincinnati Children's Hospital Medical Center, Cincinnati, United States; ^3^Biostatistics, Cincinnati Children's Hospital Medical Center; ^4^Radiology, University of Cincinnati, Cincinnati, United States

##### **Correspondence:** P. Vega-Fernandez

*Pediatric Rheumatology 2023*, **21(Suppl 2):**PT070

**Introduction:** Juvenile Idiopathic Arthritis (JIA) is the most common chronic rheumatic disease in children. Currently, clinical evaluation of arthritis is subjective and provider dependent. Musculoskeletal ultrasound (MSUS) is an objective imaging technique that can be used to assess joint inflammation.

**Objectives:** This study aims to validate a novel pediatric-specific MSUS scoring system[1] for the assessment of finger arthritis.

**Methods:** Children with a diagnosis of JIA who received a MSUS of the finger joints were eligible for this study. Clinical data collected included presence/absence of MCP arthritis on physical examination (PE), and physician- and patient- reported outcomes. A comprehensive finger MSUS examination, including B-mode and Power Doppler (PD) mode of the metacarpophalangeal (MCP) and proximal interphalangeal (PIP) joints was performed on all participants by an American College of Rheumatology MSUS certified pediatric rheumatologist. MSUS images were scored by pediatric MSUS experts, who were blinded to clinical and imaging information, as per recently published semiquantitative MSUS scoring system (0-normal to 3-severe)[1]. For the current report, MSUS was recorded abnormal if any of the B-mode images had a score equal or greater than 2 or if PD images had a score of 1-3. B-mode images with a MSUS scores of 0 and 1 and PD-mode scores of 0 were interpreted as normal. A subset of participants received an MRI with and without contrast of the fingers (2^nd^ through 5^th^ finger) immediately after MSUS performance. MRI of the MCP joint was scored as abnormal if there was presence of increased synovial thickening (ST) and joint effusion (JE) as per previously proposed MRI scoring systems [2, 3]. Spearman’s Correlations were used to calculate the associations between variables.

**Results:** Twenty-three children (mean age of 12.5 years) with JIA were enrolled in this study. In some instances, bilateral MCP’s examination was performed and recorded. For the current report 139 MCPs were analyzed. At the time of MSUS collection, 23 (16.6%) of the MCPs had arthritis by physical examination. A weak correlation between physical examination and MSUS was found (r=0.23, p=0.006). Of these 139 MCPs, 24 MCPs had MRI in addition to MSUS and physical examination. There was a weak correlation between MRI and physical examination (r=0.26, p=0.2). A strong moderate correlation between MRI and MSUS for the presence of MCP arthritis (r=0.62, p=0.002).

**Conclusion:** The weak correlation of MSUS synovitis with finger arthritis by physical examination and the moderate strong correlation of MSUS synovitis with contrast-enhanced MRI, suggest that MSUS provides an objective bedside assessment of MCP arthritis. MSUS has the potential to effectively inform JIA medical decision making real-time. Further analysis is underway.

**Patient Consent**

Yes, I received consent

**Disclosure of Interest**

P. Vega-Fernandez: None declared, K. Rogers: None declared, M. Quinlan-Waters: None declared, A. Cassedy: None declared, A. Meyers Consultant with: paid consultant for Pfizer, T. Ting: None declared

**References**

1. Vega-Fernandez, P., et al., *Musculoskeletal Ultrasound in Childhood Arthritis Limited Examination: A Comprehensive, Reliable, Time-Efficient Assessment of Synovitis.* Arthritis Care Res (Hoboken), 2023.**75**(2): p. 401-409.
2. Damasio, M.B., et al., *MRI of the wrist in juvenile idiopathic arthritis: proposal of a paediatric synovitis score by a consensus of an international working group. Results of a multicentre reliability study.* Pediatr Radiol, 2012. **42**(9): p. 1047-55.
3. van Dijkhuizen, E.H.P., et al., *Effect of the Inclusion of the Metacarpophalangeal Joints on the Wrist Magnetic Resonance Imaging Scoring System in Juvenile Idiopathic Arthritis.* J Rheumatol, 2018.**45**(11): p. 1581-1587.

## PT071 Assessment of cardiorespiratory fitness using the progressive aerobic cardiovascular endurance run in patients with Juvenile idiopathic arthritis and familial mediterranean fever: a healthy control study

### A. Albayrak^1,2^, N. Arman^3^, A. Yekdaneh^1,4^, F. Demirkan^5^, N. Aktay Ayaz^5^

#### ^1^Institute of Graduate Studies, Physiotherapy and Rehabilitation Doctorate Program, Istanbul University-Cerrahpasa; ^2^Faculty of Health Sciences, Department of Physiotherapy and Rehabilitation, Istanbul Kent University; ^3^ Faculty of Health Sciences, Department of Physiotherapy and Rehabilitation , Istanbul University-Cerrahpasa; ^4^Vocational School of Health Services, Physiotherapy English Program, Fenerbahce University; ^5^Department of Pediatric Rheumatology, Istanbul University, Istanbul Faculty of Medicine, Istanbul, Türkiye

##### **Correspondence:** A. Albayrak

*Pediatric Rheumatology 2023*, **21(Suppl 2):**PT071

**Introduction:** Juvenile Idiopathic Arthritis (JIA) and Familial Mediterranean Fever (FMF) are the most common autoimmune and autoinflammatory rheumatic diseases in childhood. Cardiorespiratory fitness, which is an important indicator of physical function, plays a critical role in health-related outcomes in children and adolescents with rheumatic disease. The need for extensive equipment and trained personnel, accompanied with the inability to assess large numbers of children at one-time makes the objective assessment of cardiorespiratory fitness in a clinic setting unmanageable. The Progressive Aerobic Cardiovascular Endurance Run (PACER) has become a routine test for predicting cardiorespiratory fitness in children with chronic disease.

**Objectives:** The aim of this study was to evaluate the cardiorespiratory fitness with PACER, to investigate feasibility of the PACER in patients with JIA and FMF and compare the results with healthy controls.

**Methods:** Sixty-seven (22 JIA, 20 FMF, 22 healthy) children and adolescents aged between 11 to 17 years old were included in the study. Cardiorespiratory fitness was evaluated with the PACER, which is a cardiorespiratory fitness test in FitnessGram Physical Activity Test Battery. Participants were instructed to run back and forth across a marked 20-m course in a straight line, pivot and turn on completing a lap, and pace themselves in accordance with an audio recording. Participants were instructed to continue running until the pace could no longer be maintained. Strong verbal encouragement was provided by research personnel to continue running as long as possible. Participants completed the PACER test individually. By using the FitnessGram “VO2 calculator” application, VO2 peak was calculated for each patient based on the number of laps that patients completed. Patients were then classified, according to the age- and gender-specific cut-off points of FitnessGram as Needs Improvement (NI)-Health Risk, NI and healthy fitness zone (HFZ). The SPSS Version 24.0 program was used for statistical analysis.

**Results:** The mean age of the children and adolescents diagnosed with JIA and FMF and healthy controls included in the study was 13.77±1.82, 13.75±2.02 and 14.96±0.73 years, respectively. The PACER results of patients with JIA and FMF and their healthy peers were shown in Table 1. Compared with their healthy peers, the PACER test results of children and adolescents with JIA and FMF were significantly lower (p<0.05). The PACER test results of children with JIA and FMF were statistically similar (p>0.05) and the scores of nearly all participants were significantly lower than the age-appropriate normal scores. When the FitnessGram Standards were applied, %85.7 of them and %72.7 of healthy controls were categorized in the “NI-Health Risk”, %11.9 of them and %22.7 healthy controls in the NI and only %2.4 of them and %4.5 healthy controls in the HFZ for VO2 health category.

**Conclusion:** Cardiorespiratory fitness was significantly affected in children and adolescents diagnosed with both JIA and FMF. Reduced cardiorespiratory fitness can be related to many factors such as pain, fatigue and low physical activity level. The PACER can be an alternative tool for assessing cardiovascular fitness in patients with JIA and FMF. Future studies are needed to determine the factors influencing cardiorespiratory fitness in patients diagnosed with JIA and FMF for understanding barriers and to clarify the fitness facilitators. We believe that focusing on physical activity and exercise programs for improving cardiorespiratory fitness may be beneficial in minimizing health risks in patients with JIA and FMF.

This study was supported within the scope of the Scientific and Technological Research Council of Turkey (TUBITAK) 1001-Scientific and Technological Research Projects Support Program (Project number: 121E690).

**Patient Consent**

Yes, I received consent

**Disclosure of Interest**

None declared

## PT072 Blood transcriptomics to facilitate diagnosis and stratification in paediatric rheumatic diseases

### M. Ha^1^, J. Schippers^2^, P. Maes^3^, E. Bartholomeus^1^, L. Van Os^3^, J. Dandelooy^3^, J. Leysen^3^, O. Aerts^3^, E. De Smet^3^, K. Guerti^3^, M. De Maeseneer^4^, N. Aerts^5^, V. Sabato^3^, A. Suls^2^, J. Van der Werff ten Bosch^4^, J. Dehoorne^6^, R. Joos^5^, K. Laukens^7^, P. Meysman^7^, B. Ogunjimi^1,3,5^

#### ^1^Faculty of Medicine and Health Sciences; ^2^Center of Medical Genetics, University of Antwerp; ^3^Antwerp University Hospital, Antwerp; ^4^Brussels University Hospital, Brussels; ^5^Ziekenhuis Netwerk Antwerpen, Antwerp; ^6^Ghent University Hospital, Ghent; ^7^Department of Mathematics and Computer Science, University of Antwerp, Antwerp, Belgium

##### **Correspondence:** M. Ha

*Pediatric Rheumatology 2023*, **21(Suppl 2):**PT072

**Introduction:** Paediatric rheumatology covers a broad range of local and systemic inflammatory diseases that are caused by autoimmune and/or autoinflammatory mechanisms. Children who present with rheumatic symptoms often pose several challenges to their physicians due to the diagnostic challenges caused by the heterogeneity of many rheumatic diseases, variable clinical presentations, and complex pathophysiology.

**Objectives:** The objective of this study is to improve the early diagnosis of paediatric rheumatic diseases via whole blood transcriptomics combined with machine learning. We aim to investigate the gene expression of whole blood from children with rheumatic diseases and apply machine learning on the transcriptome data to develop classification models for identifying different disease groups [1].

**Methods:** The cohort included 41 control cases (i.e., children without viral infection or rheumatic diseases), 47 children with viral infection, and 147 children having different rheumatic diseases: chronic recurrent multifocal osteomyelitis (CRMO), deficiency of IL-1 receptor antagonist (DIRA), juvenile idiopathic arthritis (JIA), periodic fever, aphthous stomatitis, pharyngitis, adenitis (PFAPA), systemic JIA (sJIA), undifferentiated systemic autoinflammatory disorders (uSAID), acrocyanosis-vasculopathy, chilblains, interferonopathy, linear scleroderma, Lyme arthritis, raynaud, sarcoid, uveitis, and vasculitis. RNA sequencing was performed on whole blood collected from all participants. Analyses of differentially expressed genes, gene ontology enrichment, KEGG pathways, and Random Forest classifier development were conducted based on the transcriptomic data.

**Results:** Chemokine signalling was observed in acrocyanosis-vasculopathy and PFAPA patients. AMPK signalling (which mediates the cellular energy level) was found in CRMO and DIRA patients, while mTOR signalling (which regulates cell proliferation and differentiation) was exhibited in Lyme, PFAPA, and sJIA. Patients with acrocyanosis-vasculopathy, JIA, Lyme, PFAPA, raynaud, sJIA, and uSAID could be distinguished well from other rheumatic groups by Random Forest classifiers with high area-under-the-curve (AUC) values (AUC = 0.76 ± 0.18, 0.69 ± 0.16, 0.92 ± 0.13, 0.76 ± 0.22, 0.86 ± 0.25, 0.79 ± 0.22, and 0.94 ± 0.13, respectively). When comparing HLA-B27^+^ enthesitis-related JIA with other juvenile arthritis groups (i.e., psoriatic, oligoarticular, polyarticular, undifferentiated, and Lyme arthritis), cytokine receptor interaction, JAK-STAT, IL-17, and TNF signalling pathways were enriched. Random Forest classification between these groups showed AUC values in the range 0.73 – 0.81 in leave-one-out cross-validation.

**Conclusion:** Overall, our study indicates that blood transcriptomics combined with machine learning is a promising tool for paediatric rheumatic disease classification and diagnosis. Application of machine learning on other clinical and molecular data has potential to assist paediatric rheumatologists in predicting the course of diseases, identifying important risk factors, and estimating treatment responses.

**Patient Consent**

Yes, I received consent

**Disclosure of Interest**

None declared

**Reference**

1. Ha, M.K. et al. Pediatr. Rheumatol. 2022, 20, 91.

## PT073 Treatment escalation in polyarticular juvenile idiopathic arthritis: a population in Germany in 2014 and 2015—a retrospective observational health claims data study

### G. Horneff^1^, J. Borchert^2^, J. Diesing^2^, P. Klaus^3^, R. Heinrich^2^, H. Dally^3^, C. Hagemann^3^, S. Kock^4^, T. Schönfelder^2,5^

#### ^1^Department of General Paediatrics, Asklepios Clinic Sankt Augustin, Sankt Augustin; ^2^WIG2 GmbH, Leipzig; ^3^Pfizer Pharma GmbH; ^4^Institute for Applied Health Research (InGef), Berlin; ^5^Lehrstuhl Gesundheitswissenschaften/Public Health, Technische Universität Dresden, Dresden, Germany

##### **Correspondence:** G. Horneff

*Pediatric Rheumatology 2023*, **21(Suppl 2):**PT073

**Introduction:** Current polyarticular juvenile idiopathic arthritis (polyJIA) treatment options are not always sufficient in easing symptoms and reducing further damage.

**Objectives:** We evaluated treatment patterns in newly diagnosed patients with polyJIA, with a focus on changes possibly indicating inadequate achievement of treatment goals.

**Methods:** Using a retrospective observational cohort study on two non-overlapping longitudinal health claims databases (WIG2, Scientific Institute for Health Economics and Health System Research GmbH, and InGef, Institute for Applied Health Research Berlin GmbH), a representative sample of 3.5 and 4 million patients, respectively, was analysed. Incident polyJIA patients were identified with inpatient or outpatient ICD-10 GM (German modification) diagnosis codes M08.0 or M08.3. The prescription date was assigned to a given half-year (HY) period relative to the index (first diagnosis) quarter. For each HY period, the highest drug escalation treatment was categorized, based on the following hierarchy (highest to lowest escalation: bDMARDs, csDMARDs, GCs, NSAIDs, none). Results from baseline period and 3-year follow-up from both cohorts and data sets were pooled

**Results:** We identified 121 and 58 newly diagnosed polyJIA patients in the pooled data set (2014/2015) in the InGef and WIG2 databases, respectively. At one year before the first diagnosis, most patients were not being treated with any of the pre-defined medications (66% WIG2 and 71% InGef), but of those who were, most were being treated with NSAIDs (21% and 12%), with little change in the -1HY. In the first HY period after index, most patients in the population were being treated with a csDMARD (not a bDMARD), however there were still some patients (24% and 12%) receiving none of the investigated treatments at this time.

CsDMARD as the highest escalation drug used peaked in the first HY period after index, and (52% and 33%) while bDMARD use continued to increase throughout the 3-year follow-up. At 3HY following diagnosis, 40% and 26% of patients were treated (at highest escalation) with a csDMARD, and 24% and 19% with a bDMARD.

After about 3 years of follow-up, many patients were receiving none of the treatments (31% and 45%), however some were still taking csDMARDs (17% in both databases) and even more taking bDMARDs (36% and 18%). The proportion of patients taking bDMARDs peaked at the end of the 3-year follow-up in one dataset (WIG2, 36%) and at the 3HY mark in the other (InGef, 19%), however in the latter, remained high by the end of the follow-up (at 3 years, 18%).

**Conclusion:** Throughout follow-up over three years from diagnosis, there was an increasing use of bDMARDs, and use of csDMARDs (as highest escalation) remained over 17%, indicating a need for further advanced treatment options for a subset of the population.

**Patient Consent**

Not applicable (there are no patient data)

**Disclosure of Interest**

G. Horneff Grant / Research Support with: Novartis, Roche, MSD, Speaker Bureau with: Lilly, Pfizer, GSK, Sanofi, J. Borchert Grant / Research Support with: Research activities of WIG2 GmbH commissioned and financed by Pfizer Pharma GmbH, Employee with: WIG2 GmbH, J. Diesing Grant / Research Support with: Research activities of WIG2 GmbH commissioned and financed by Pfizer Pharma GmbH, Employee with: WIG2 GmbH, P. Klaus Shareholder with: Pfizer Pharma GmbH, Employee with: Pfizer Pharma GmbH, R. Heinrich Grant / Research Support with: Research activities of WIG2 GmbH commissioned and financed by Pfizer Pharma GmbH, Employee with: WIG2 GmbH, H. Dally Shareholder with: Pfizer Pharma GmbH, Employee with: Pfizer Pharma GmbH, C. Hagemann Employee with: During project phase; now former employee of Pfizer Pharma GmbH, S. Kock Employee with: InGef GmbH, T. Schönfelder Grant / Research Support with: Research activities of WIG2 GmbH commissioned and financed by Pfizer Pharma GmbH, Employee with: WIG2 GmbH

## PT074 Safety of intravenous use of anakinra in pediatric inflammatory conditions

### M. Klanjscek^1^, M. Trevisan^2^, S. Pastore^3^, M. Pardeo^2^, F. De Benedetti^2^, A. Tommasini^1,3^, A. Taddio^1,3^, C. Bracaglia^2^

#### ^1^University of Trieste, Trieste; ^2^Division of Rheumatology, IRCCS Ospedale Pediatrico Bambino Gesù, Rome; ^3^Institute for Maternal and Child Health, IRCCS Burlo Garofolo, Trieste, Italy

##### **Correspondence:** M. Klanjscek

*Pediatric Rheumatology 2023*, **21(Suppl 2):**PT074

**Introduction:** Anakinra is a recombinant human interleukin-1 (IL-1) receptor antagonist; it blocks the activity of IL-1, a pro-inflammatory cytokine involved in the immune response. It is primarily used by subcutaneous injection in the treatment of several autoinflammatory conditions such as: Still's disease, familial mediterranean fever and cryopyrin-associated periodic syndromes (CAPS). Intravenous anakinra is used in clinical practice, especially for Macrophage Activation Syndrome (MAS)/Hemophagocytic Lymphohistiocytosis (HLH), despite this being an off-label route of administration. In acute and life-threatening diseases, the subcutaneous route is often problematic, absorption may be unreliable in patients with critical illness, peripheral oedema or anasarca and multiple painful injections are needed to achieve the high doses required. Intravenous administration of anakinra enables a higher and faster maximal plasma concentration to be achieved, compared with subcutaneous delivery. Some authors have already reported its effectiveness via intravenous administration in some specific pediatric clinical settings such as MAS/HLH.

**Objectives:** To collect the patients treated by intravenous anakinra in order to demonstrate the safety of intravenous administration of anakinra in a cohort of children affected by different inflammatory conditions.

**Methods:** This is a bicentric retrospective study. All pediatric patients treated with intravenous anakinra from January 1^st^, 2017, to December 31^st^, 2022, in IRCCS Maternal and Child Health Institute Burlo Garofolo in Trieste (Italy) and in the IRCCS Bambino Gesù Children’s Hospital in Roma (Italy) were enrolled. Data about quantitative characteristics related to drug administration (dosage administered, treatment duration), information regarding the hospital setting (admission or non-admission to the ICU), findings about the clinical response, presence or absence of side effects, their nature and the patient’s outcome were collected.

**Results:** Our case series includes 48 patients with different underlying clinical conditions; the most represented are MAS/HLH 40% and MIS-C 19%.

Side effects were observed in 7 out of 48 treated children (14%), in most cases they were represented by transient elevation of hepatic or pancreatic enzymes (5/48, 10%). One patient (1/48, 2%) exhibited a maculopapular rash and fever 20 minutes after the infusion, which resolved upon discontinuation of the medication. In another case (1/48, 2%) there was an immediate reaction with hypotension and vomiting, which resolved after administration of plasma-derived medicinal products (PDMPs) and antihistamine. Seven patients (14%) experienced an unfavorable outcome due to the progressive and untreatable nature of their underlying clinical condition, specifically sepsis (2/7), acute respiratory failure, post-transplant complications, cardiac arrest, DRESS and intractable HLH.

**Conclusion:** The intravenous use of anakinra in pediatrics could represent a more manageable and effective alternative in the treatment of some life-threatening acute clinical conditions, ensuring the possibility to administer a high dosage of the drug and its absorption even in critical settings. The intravenous administration of anakinra at a dosage of 2-20mg/kg/day has proven to be safe in our case series. Side effects demonstrate to be transient or pharmacologically manageable. It is necessary to expand this research involving other Centers in order to increase the number of cases and the statistical significance.

**Patient Consent**

Yes, I received consent

**Disclosure of Interest**

None declared

## PT075 Functional gastrointestinal disorders in patients with juvenile fibromyalgia syndrome

### C. Lavarello^1,2^, L. Carlini^3^, A. Nahim^1,2^, M. Mori^1^, A. Ronchetti^4^, F. Casabona^1,2^, A. Feliciello^1,2^, S. Arrigo^5^, E. Pescio^6^, M. Gattorno^1^, C. Malattia^1,2^

#### ^1^Rheumatology and autoinflammatory diseases Unit, IRCCS Istituto Giannina Gaslini; ^2^Department of Neurosciences, Rehabilitation, Ophthalmology, Genetic and Maternal Infantile Sciences (DINOGMI), University of Genoa; ^3^Trial center, Rheumatology and autoinflammatory diseases Unit; ^4^Physical Medicine and Rehabilitation Unit; ^5^Gastroenterology and digestive endoscopy Unit; ^6^Psychology Unit, IRCCS Istituto Giannina Gaslini, Genoa, Italy

##### **Correspondence:** C. Lavarello

*Pediatric Rheumatology 2023*, **21(Suppl 2):**PT075

**Introduction:** Juvenile Fibromyalgia Syndrome (JFS) is a disabling condition characterized by widespread musculoskeletal pain, fatigue, sleep, cognitive and mood disturbances. A considerable proportion of patients with JFS experience gastrointestinal (GI) symptoms such as abdominal pain, bloating, and constipation **(1)**. Although the association between fibromyalgia and Functional Gastro-Intestinal disorders (FGID) is well established in adults with fibromyalgia, so far no study has explored this association in JFS.

**Objectives:** To investigate the frequency of FGID in JFS patients and its impact of the disease burden.

**Methods:** JFS patients followed at our center between 2021 and 2023 were included in the present study. Patients underwent a multidisciplinary evaluation. We applied Rome IV criteria **(2)** to investigate type and frequency of FGID. The Children Depression Inventory (CDI) and the Multidimensional Anxiety Scale for Children (MASC) were used to evaluate presence and severity of mood disorders. Participants were asked to rate their average level of pain in the previous two weeks using the standard 100-mm pain Numerical Rating Scale (NRS) that ranges from 0 (no pain) to 100 mm (pain as bad as it can be). Self-report measures (100 mm NRS) were also used to rate the severity of symptoms in the following domains: fatigue, headache, symptoms severity upon awakening, and global assessment (PGA) of disease severity.

**Results:** We included 47 JFS patients (F 42) with median age at onset of 13.7 (IQR 11.3-15.2) and median age at JFS diagnosis of 15.6 (IQR 14.1-16.5). Thirty-nine out of 47 (87%) patients referred at least one gastrointestinal symptom. Abdominal pain (85.1%), nausea (44.7%), constipation (34%), pyrosis (31.9%), bloating (27.7%), postprandial nausea (27.7), and dyspepsia (25.5%) were more common Thirteen out of 39 (33.3%) reported a frequency of symptoms > 4 days a week and 20 out of 39 (51.3%) reported that these symptoms are limiting daily activities. 14 patients (29.8%) underwent invasive investigations (e.g. endoscopy). 28 patients (59.6%) met Rome IV criteria for at least one FGID: dyspepsia in association with IBS (18.5%), IBS (14.8%), functional dyspepsia (14.8%), functional constipation (3.7%), and functional abdominal pain not otherwise specified (48.2%). We found a trend of higher PGA in patients with JFS and FGID even if with a difference not statistically significant (p 0.06).

**Conclusion:** FGID were diagnosed in a significant percentage of JFS patients and have a relevant impact on disease severity. Most common FGID were IBS and functional abdominal pain not otherwise specified. Our results highlight the need for a multidisciplinary approach to the assessment of JFS patients. Elucidation of the common pathophysiologic mechanisms underlying these disorders could provide insights for the development of more comprehensive and targeted therapeutic approaches.

**Patient Consent**

Yes, I received consent

**Disclosure of Interest**

None declared

**References**

1. Erdrich S, Hawrelak JA, Myers SP, Harnett JE. A systematic review of the association between fibromyalgia and functional gastrointestinal disorders. Therap Adv Gastroenterol. 2020 Dec 8:13:1756284820977402. doi: 10.1177/1756284820977402. PMID: 33343707; PMCID: PMC7727037.
2. Thapar N, Benninga MA, Crowell MD, Di Lorenzo C, Mack I, Nurko S, Saps M, Shulman RJ, Szajewska H, van Tilburg MAL, Enck P. Paediatric functional abdominal pain disorders. Nat Rev Dis Primers. 2020 Nov 5;6(1):89. doi: 10.1038/s41572-020-00222-5. PMID: 33154368.

## PT076 White matter abnormalities in brain MRI in children with non-infectious uveitis

### I. Maccora^1,2^, J. Hendrikse^3^, C. de Libero^4^, V. Koopman-Kalinina Ayuso^3^, L. Corbelli^1^, R. Brandsma^5^, L. Gatti^1^, M. Jansen^6^, R. Nievelstein^7^, J. Kuiper^3,8^, R. Caputo^4^, J. H. de Boer^3^, G. Simonini^1,2^

#### ^1^Rheumatology Unit, ERN ReConnet Center, Meyer Children's Hospital IRCCS, Florence, Italy; ^2^NeuroFARBA Department, University of Florence, Florence, Italy; ^3^Department of Ophthalmology, University Medical Center Utrecht, Utrecht, Netherlands; ^4^Ophthalmology Unit, Meyer Children's Hospital IRCCS, Florence, Italy; ^5^Department of Pediatric Neurology, University Medical Center Utrecht; ^6^Department of Immunology, Wilhelmina Children’s Hospital; ^7^Department of Radiology and Nuclear Medicine, University Medical Center Utrecht; ^8^Center for Translational Immunology, University Medical Center Utrecht, Utrecht, Netherlands

##### **Correspondence:** I. Maccora

*Pediatric Rheumatology 2023*, **21(Suppl 2):**PT076

**Introduction:** Childhood non-infectious uveitis (cNIU) is a rare disease whose differential diagnosis is extremely challenging and need to take into account several systemic diseases including demyelinating disease. Therefore, a thorough assessment should be considered, because of the presence of White Matter Abnormalities (WMA) in these children is important as they are often treated with anti-TNFα therapy which could worsen these lesions.

**Objectives:** The aim of this study is to report the prevalence WMA in children with non-infectious uveitis (NIU).

**Methods:** We performed a retrospective chart medical review at Meyer Children’s Hospital IRCCS (Florence) and at the University Medical Center Utrecht, involving children with less than 18 years old, with cNIU that underwent a cerebral MRI before starting a systemic treatment. Clinical, laboratory and radiological data were collected. As main outcome we considered the brain MRI abnormality.

**Results:** From two tertiary centers, the UMC Utrecht and the Meyer’s Children’s Hospital, a total of 112 children (53 female), 35 (31.3%) with anterior uveitis, 30 (26.8%) with intermediate uveitis, one with posterior uveitis (0.89%) and 46 (41.1%) with panuveitis, were included. 91 have bilateral uveitis (81.25%). Among the 112 children, 96 (85.7%) have idiopathic uveitis, 8 Tubular interstitial associated uveitis (TINU) (7.14%), and 8 others (7.14%). The median age at onset of uveitis was 109 months (range 98-139). A total of 29 children (25.9%) showed WMA on cerebral MRI. Among these children with WMA, 8 have anterior uveitis (27.5%), 10 intermediate uveitis (34.4%), 11 panuveitis (37.9%). In addition, incidental findings included one patient with a glandule pineal lesion, five patients with cysts, two patients with gliosis, one patient with optic neuritis, one patient with venous angioma, one patient with optic nerve atrophy and one patient with plexus papilloma. None of the patients with WMA showed neurological symptoms when the brain MRI was performed. In total, 72.34% of all MRI abnormal findings were seen in non-anterior uveitis patients.

**Conclusion:** In both tertiary centers, white matter abnormalities were frequently found on cerebral MRI, especially in non-anterior uveitis patients. Typically, these abnormalities were asymptomatic, however it is critical to early identify possible brain involvement in order to choose the better treatment.

**Patient Consent**

Yes, I received consent

**Disclosure of Interest**

None declared

## PT077 Diagnostic value of minor salivary gland biopsy in children: a monocentric retrospective study over 10 years

### F. Adeline^1,2^, A. Hittinger^1^, L. Bolko^1^, C. Guettier^3,4^, I. Kone Paut^4,5^, A. Schvartz^5,6^

#### ^1^Department of Rheumatology, Maison Blanche Hospital, Reims University Hospital; ^2^University of Reims Champagne-Ardennes, Reims; ^3^Department of anatomopathology, Bicêtre University Hospital, Assistance Publique Hôpitaux de Paris; ^4^University of Paris Saclay; ^5^Department of Paediatric Rheumatology, Reference Center for Autoinflammatory diseases and amyloidosis, Bicêtre University Hospital, Assistance Publique Hôpitaux de Paris, Le Kremlin-Bicêtre; ^6^University of Paris Saclay, Le Kremlin Bicêtre, France

##### **Correspondence:** A. Schvartz

*Pediatric Rheumatology 2023*, **21(Suppl 2):**PT077

**Introduction:** Minor salivary gland biopsy (MSGB) is a common test in medicine, easily prescribed by both pediatric and adult specialists(1). Despite its simplicity, MSGB is not without risk. A prospective study about MSGB complications revealed 10% of it (7% for local pain and 3% for paresthesia) are persistent(2). Although the MSGB is a major criterion for the diagnosis of Sjogren's Syndrome (SS), multiple studies have outlined difficulties in standardization(3). SS is a rare disease in children and can differs from the adult counterpart. MSGB is also used for the diagnosis of sarcoidosis where it allows visualization of granulomas. Several studies have conflicting results for this indication(4,5). To our knowledge, no study has investigated the accuracy of MSGB in children in SS and sarcoidosis suspicions.

**Objectives:** The aim of our study was to analyze diagnostic performance of MSGB in children suspected of Sjogren’s syndrome or sarcoidosis.

**Methods:** We did a retrospective monocentric study on patients under 18 years old who had a MSGB between October 2011 and December 2021 at Bicêtre University Hospital tertiary center. Clinical and biological data were collected digitally. Indication was made by the clinician in charge of the patient. Histological analysis was done by a dedicated expert physician. Biopsy was considered positive if Chisholm-Mason score was superior or equal to 3, or focus score was superior or equal to 1 for suspicion of SS, according to the ACR/EULAR criteria, and if non caseified granulomas were present in cases of sarcoidosis.

**Results:** One hundred sixty-two MSGB were analyzed, 47 were pathological and 34 confirmed the initial suspicion. MSGB were divided into 2 groups according to indications. Among the 66 MSGB performed for suspected SS, 23 were positive and 13 were associated with a confirmed final diagnosis. Twenty patients (31%) had positive anti-SSA antibodies. MSGB was sensitive and specific (Se=81%, Sp=80). Anti-SSA antibodies improved specificity (98%), but no other parameters.

For sarcoidosis suspicion, 114 biopsies were performed, 21 (18%) were associated with the final confirmed diagnosis and 7/21 (33%) of them were positive. On 63 patients who had ophtalmological examination, 40 had granulomatous uveitis. MSGB was not sensitive but very specific (Se=33%, Sp=98%). The presence of granulomatous uveitis improved the sensitivity and specificity of the test and the 7 patients who had positive biopsies and granulomatous uveitis had confirmed sarcoidosis.

**Conclusion:** Minor salivary gland biopsy is a simple and moderate invasive test in children, which is a good confirmation diagnostic tool but should not be used as a screening tool in sarcoidosis. It does not provide added value in the event of suspicion of SS compared to other non-invasive parameters, especially anti-SSA antibodies.

**Patient Consent**

Not applicable (there are no patient data)

**Disclosure of Interest**

None declared

**References**

1. Pellegrini M, et al. Current Salivary Glands Biopsy Techniques: A Comprehensive Review. *Healthcare* 2022.
2. Lida Santiago M, et al. Frecuencia de complicaciones y rédito de la biopsia de glándula salival menor. *Reumatología Clínica* 2012
3. Wicheta S, et al. Discrepancies in Interpretation of the Minor Salivary Gland Biopsy in the Diagnosis of Sjögren Syndrome. *Journal of Oral and Maxillofacial Surgery* 2019
4. Blaise P, et al. Minor salivary gland biopsy in diagnosing ocular sarcoidosis. *British Journal of Ophthalmology* 2011
5. Bernard C, et al. Ocular sarcoidosis: when should labial salivary gland biopsy be performed? *Graefes Arch Clin Exp Ophthalmol* 2013

## PT078 Assesment of ovarian dysfunction in adolescent female patients with severe rheumatic disease receiving cyclophoshamide

### C. A. Tatar^1^, G. Ozer^2^, M. Kasap Cüceoglu^3^, Y. Bayındır^3^, O. Basaran^3^, E. D. Batu^3^, B. Oguz^2^, S. Ozen^3^, A. Ozon^4^, Y. Bilginer^3^

#### ^1^Department of Pediatrics; ^2^Department of Radiology; ^3^Department of Pediatrics, Division of Rheumatology; ^4^Department of Pediatrics, Division of Endocrinology, Hacettepe University, Ankara, Türkiye

##### **Correspondence:** C. A. Tatar

*Pediatric Rheumatology 2023*, **21(Suppl 2):**PT078

**Introduction:** Cyclophosphamide (CYC) with its immunosuppressive properties continues to be the drug of choice in patients with severe rheumatic disease, such as vasculitis and systemic lupus erythematosus (SLE). However, ovarian failure due to cytotoxicity of CYC remains to be a major concern in adolescent female patients with high probability of survival.^1^

**Objectives:** To analyze the risk of ovarian failure in women, diagnosed with rheumatic diseases and treated with CYC during adolescence.

**Methods:** This is a single-center cross-sectional cohort study involving 21 female patients (age 12-29 years) diagnosed with rheumatic disease (15 with SLE and the remaining with systemic vasculitides) between 2000 and 2020 who received CYC therapy. History of illness, details of menstrual irregularities, serum levels of FSH, LH, estradiol and AMH was recorded in 21 patients, and transabdominal ultrasonographic assessment of ovarian and uterine volume, antral follicular count, endometrium thickness and corpus to cervix ratio were carried out in 18 patients on days 3-5 of the menstrual cycle. Analyses were stratified according to treatment with low and high cumulative CYC doses (< or = 3g and > 3g, respectively) as well as the presence of amenorrhea which was defined as the absence of menstruations for three or more consecutive cycles.

**Results:** Median age at diagnosis was 12 (4-17) years and at the onset of CYC initiation it was 13 (9-18) years. The median age at the time of current evaluation was 16 (12-29) years with a median duration of 4 (1-17) years between treatment onset (CYC) and evaluation. The median of cumulative CYC dosage was 3 (0,5-7) grams and treatment duration was 6 (1-22) months. Eight women (38,1%) developed transient amenorrhea during follow-up, however none had sustained amenorrhea. The distribution of cumulative CYC dose was the same across categories of amenorrhea. There were no statistically significant differences between serum FSH, LH, estradiol levels and sonographic findings of low and high cumulative CYC doses. Notably, the AMH was comparably lower in the group with history of amenorrhea (p=0,068) and with high cumulative CYC doses.

**Conclusion:** Our findings support that children’s ovaries are much more resistant to the gonadotoxic effects of CYC, which is inconsistent with adult data. However, low AMH levels may indicate a future risk of ovarian dysfunction necessitating watchful monitoring.

**Patient Consent**

Yes, I received consent

**Disclosure of Interest**

None declared

**Reference**

1. Ejaz, K., Abid, D., Juneau, P., Chu, J., Hasni, S. Use of gonadotropin-releasing hormone agonists for ovarian preservation in patients receiving cyclophosphamide for systemic lupus erythematosus: A meta-analysis. Lupus. 2022; 31(14), 1706-1713.

## PT079 Mapping clinical characteristics in children and adolescents with symptomatic hypermobility in tertiary paediatric rheumatology settings. An Irish perspective

### S. U. Ward^1^, S. Dockrell^2^, J. Deane^3^, J. Simmonds^4^, K. Robinson ^5^, E. Carberry ^6^, C. Lowry ^7^, O. G. Killeen ^8^, N. Ambrose ^8^, E. J. MacDermott^8^

#### ^1^Discipline of Physiotherapy, School of Medicine, Trinity College University of Dublin; ^2^Discipline of Physiotherapy, School of Medicine, Trinity College, University of Dublin, Dublin, Ireland; ^3^Exercise and Rehabilitation Sciences, University of Birmingham, Birmingham; ^4^Great Ormond Street Institute of Child Health, University College London, London , United Kingdom; ^5^Physiotherapy , Children’s Health Ireland at Temple Street; ^6^Physiotherapy , Children’s Health Ireland at Crumlin; ^7^Children’s Health Ireland at Temple Street; ^8^Children’s Health Ireland at Crumlin, Dublin, Ireland

##### **Correspondence:** S. U. Ward

*Pediatric Rheumatology 2023*, **21(Suppl 2):**PT079

**Introduction:** Determining clinically significant joint hypermobility in childhood presents a challenge to clinicians. Previous research has demonstrated an evolving phenotype whereby hypermobility and symptoms may further develop or resolve over time. This is the first study to map clinical characteristics of children and adolescents presenting with symptomatic hypermobility in tertiary rheumatology settings from an Irish perspective.

**Objectives:** Define the clinical characteristics of children and adolescents presenting with symptomatic hypermobility to tertiary paediatric rheumatology services in Ireland.

**Methods:** A prospective cross-sectional study was carried out on children and adolescents aged 6-16 years recognised as having symptomatic hypermobility determined by a paediatric rheumatologist. A structured interview and clinical measurements were conducted. Standardised assessments included screening for generalised hypermobility (Beighton score and Lower Limb Assessment Score (LLAS)), Foot Posture Index, the 6-minute walk test, Y-balance test, measurement of strength and endurance and motor skills screening (BOT-2 Brief). Parent and child questionnaires assessing pain, multidimensional fatigue and quality of life were also completed following strict protocols.

**Results:** Eighty participants (26 males, 54 females) were included. Mean age was 11.6±3 years (range 6.0-16.92). Generalised joint hypermobility (Beighton score of ≥6/9) was identified in 79% of participants, and a further 9% classified using the LLAS. The mean time from onset of symptoms to recognition of symptomatic hypermobility was 32±23 months (range 3-108 months). All participants reported pain in more than one joint for >3 months; most affected were the knee (73%) and ankle (55%). Comorbidities were common with 49% reporting three or more. The most common were neurodevelopmental (48.8%) and chronic pain (25%). Below average motor skills were identified in 44% of participants. Muscle strength and endurance were poor with only 24% and 32% respectively achieving within the normative range. Feeling tired ‘often’ or ‘almost always’ was reported by 46% of participants and finding it hard to keep attention ‘often’ or ‘almost always’ was reported by 30%. PedsQL generic core scores (60 ±18.22) were reduced compared to normative data (83±14.79).

**Conclusion:** This study identified long delays to recognition of symptomatic hypermobility and the significant burden of symptoms and comorbidities in children and adolescents. The high levels of neurodevelopmental diagnoses, poor motor skills and multidimensional fatigue are of particular concern in this population. Earlier recognition and standardised assessments within a biopsychosocial framework are needed to develop better care pathways to improve outcomes.

**Patient Consent**

Not applicable (there are no patient data)

**Disclosure of Interest**

None declared

## PT080 Thirty-second sit-to-stand test as an alternative tool for the assessment of functional capacity in patients with Juvenile idiopathic arthritis: a pilot study

### A. Yekdaneh^1^, N. Arman^2^, A. Albayrak^3,4^, O. Akgun^5^, F. G. Demirkan^5^, N. Aktay Ayaz^5^

#### ^1^Vocational School of Health Services Physiotherapy English Program, Fenerbahçe University; ^2^Faculty of Health Sciences Department of Physiotherapy and Rehabilitation; ^3^Institute of Graduate Studies Physiotherapy and Rehabilitation Doctorate Program, Istanbul University-Cerrahpaşa; ^4^Faculty of Health Sciences, Department of Physiotherapy and Rehabilitation, Istanbul Kent University; ^5^Istanbul Faculty of Medicine, Department of Pediatric Rheumatology, Istanbul University, Istanbul, Türkiye

##### **Correspondence:** A. Yekdaneh

*Pediatric Rheumatology 2023*, **21(Suppl 2):**PT080

**Introduction:** Juvenile Idiopathic Arthritis (JIA) is a disease of unknown etiology that affects mostly the knee and ankle joints, causing inflammation, malposition, and deterioration in functional performance (1). Children with JIA are less physically active than their healthy peers, and even children with mild involvement may exhibit decreased functional capacity and delayed development of complex motor skills, which may lead to physical inactivity and reduced quality of life (2, 3). The 6-minute walk test (6MWT) is frequently used to evaluate functional performance in patients with JIA. The 6MWT is a continuous walking test that maintains a constant speed and is administered at the child's own pace, and therefore may create a certain monotony on the functional performance of children. This may affect the functional performance and prevent the correct interpretation of the test. At the same time, it is emphasized that alternative tests are needed because the evaluation procedures of the test are not practical enough (4).

**Objectives:** The aim of this study was to investigate whether the 30-second Sit to Stand Test (30STST) is an alternative tool for assessing functional capacity in patients with JIA.

**Methods:** 28 patients with JIA (13 girls, 15 boys) aged 11-16 years were included in the study. Functional capacity of the participants was evaluated with the 30STST, 6MWT, and 10 Stair Climb Test (10SCT). SPSS Version 24.0 program was used for statistical analysis.

**Results:** The mean age of patients with JIA was 13.64±1.78 years, and the mean of the 6MWT, 30STST and 10SCT was 495.82±105.41 meters, 12.57±2.44 seconds, and 9.73±2.83 seconds, respectively. Between 30STST and 6MWT a high (r=0.73 p<0.001) and between 30STST and 10SCT a moderately significant correlation was found (r=-0.39, p=0.03).

**Conclusion:** As a results of this study, a significant relationship was found between the 30STST and 6MWT and 10SCT in patients with JIA. We believe that 30STST may have the potential to be a valid and valuable alternative tool for evaluating functional capacity in clinical routine, as it offers a quick and easy assessment in a small area compared to other functional capacity tests for patients with JIA.

This study was supported within the scope of the Scientific and Technological Research Council of Turkey (TUBITAK) 1001-Scientific and Technological Research Projects Support Program (Project number: 121E690).

**Patient Consent**

Yes, I received consent

**Disclosure of Interest**

None declared

**References**

1. Merker J, et al. Pathophysiology of Juvenile Idiopathic Arthritis induced pes planovalgus in static and walking condition: a functional view using 3D gait analysis. Pediatr. Rheumatol. Online J.13(1):21, 2015.
2. Hulsegge GH, et al. Fundamental movement skills, physical fitness and physical activity among Australian children with juvenile idiopathic arthritis. J Paediatr Child Health. 2015;51(4):425-32.
3. Pritchard L, et al. Reproducibility of the Six-Minute Walk Test in Children and Youth With Juvenile Idiopathic Arthritis. Arthritis Care Res. 2022;74(4):686-690.
4. Scalco JC, et al. PSYCHOMETRIC PROPERTIES OF FUNCTIONAL CAPACITY TESTS IN CHILDREN AND ADOLESCENTS: SYSTEMATIC REVIEW. Rev Paul Pediatr. 2018;36(4):500-510.

## PT081 Learning from longitudinal data in childhood onset systemic lupus erythematosus: which biomarkers have predictive value for endothelial involvement?

### S. C. Bergkamp^1^, N. D. Bergkamp^2^, M. J. Wahadat^3,4^, M. P. Gruppen^1^, A. Nassar - Sheikh Rashid^1,5^, T. W. Kuijpers^1^, S. W. Tas^6^, M. J. Smit^2^, M. A. Versnel^4^, J. M. van den Berg^1^, S. Kamphuis^3^, D. Schonenberg - Meinema^1^

#### ^1^Department of Paediatric Immunology, Rheumatology and Infectious Diseases, Emma Children’s Hospital, Amsterdam University Medical Centres (AUMC), University of Amsterdam; ^2^Division of Medicinal Chemistry, Faculty of Science, VU University, Amsterdam Institute for Molecular and Life Sciences (AIMMS),, Amsterdam; ^3^Department of Paediatric Rheumatology, Sophia Children’s Hospital, Erasmus University Medical Centre; ^4^Department of Immunology, Erasmus University Medical Centre, Rotterdam; ^5^Department of Paediatrics, Zaans Medisch Centrum, Zaandam; ^6^Rheumatology and Clinical Immunology, and Laboratory for Experimental Immunology, Amsterdam Rheumatology and Immunology Centre, Amsterdam University Medical Centres (AUMC), University of Amsterdam, Amsterdam, Netherlands

##### **Correspondence:** S. C. Bergkamp

*Pediatric Rheumatology 2023*, **21(Suppl 2):**PT081

**Introduction:** The pathophysiological mechanisms for premature atherosclerosis in (childhood-onset) systemic lupus erythematosus ((c)SLE) are not completely understood (1). Besides traditional risk factors, the endothelium plays a major role (2). Recently we hypothesized that the endothelium stays in a dysregulated state in SLE, even with low disease activity (3). However, previous studies were mostly cross-sectional and only performed in adult SLE.

**Objectives:** To determine serum biomarkers of endothelial cell (EC) activation in longitudinal samples of (treatment-naïve) cSLE patients (active vs. low SLE Disease Activity Index (SLEDAI)) and to compare them with healthy controls. Second objective was to assess the correlation of these EC markers with disease activity overtime.

**Methods:** Patient data and blood samples were used from a multicentre longitudinal cSLE cohort. Disease activity was evaluated by SLEDAI-2K, with cut-offs for active (>4) versus low (≤4) activity. Levels of CXCL12 (SDF-1), TWEAK, VEGF, CXCL10 (IP-10), ADAMTS13, Angiopoietin-2, Pentraxin-3, E-Selectin, Thrombomodulin, P-selectin, CCL2 (MCP-1), VCAM-1, ICAM-1, vWF-A2 and Gas6 were measured in cSLE (t=1 and t=2) and in HC (1 sample). Patient groups and healthy controls were compared by t-tests and ANOVA, with significance for p < 0.05. Correlations between EC biomarkers and SLEDAI were calculated with Pearson correlations.

**Results:** 47 cSLE patients (n=30 treatment naive patients at t=1) and 42 HC were included. Mean age at diagnosis was 14 (± 2.3) years. Median time between t=1 and t=2 was 14.5 months (IQR 9-24 months). Median SLEDAI at t=1 was 12 (IQR 6-18), median SLEDAI at t=2 was 2.5 (IQR 2-6). Serum levels of Angiopoietin-2, CCL2 and VCAM-1 were higher in cSLE (at t=1, compared to HC), but did not correlate with SLEDAI. At t=1, serum levels of CXCL10 (p=0.01), Thrombomodulin (p=0.03) and VCAM-1 (p=0.01) were higher in the group with active cSLE, compared to those with low disease activity. At t=2, Angiopoietin-2, CCL2, CXCL10, GAS6, Thrombomodulin and VCAM-1 were significantly higher in cSLE compared to HC, despite low median disease activity at that time point.

**Conclusion:** Our results suggest that in cSLE, the endothelium maintains in an active state over time, even in state of low disease activity. These markers represent activation of ECs with vascular inflammation, EC activation and a pro-angiogenic state. This study could aid in unravelling a part of the pathophysiology of premature atherosclerosis in cSLE patients.

**Patient Consent**

Yes, I received consent

**Disclosure of Interest**

None declared

**Reference**s

1. Ardoin SP, Schanberg LE, et al. Laboratory markers of cardiovascular risk in pSLE: the APPLE baseline cohort. Lupus. 2010 PMID: 20861207
2. Westerweel PE, Luyten RK, et al. Premature atherosclerotic cardiovascular disease in SLE. Arthritis Rheum. 2007 May; 1384-96. PMID: 17469095
3. Bergkamp SC, Wahadat MJ et al., J Inflamm (Lond). 2023 May 16;20(1):18. PMID: 37194071;

## PT082 Physician global assessment of disease activity in Childhood-onset Systemic Lupus Erythematosus (CSLE) – does the approach matter?

### H. I. Brunner^1^, E. A. Ogbu^1,2^, J. L. Huggins^1^, A. Merritt^1^, M. Quinlan-Waters^1^, C. Robben^1^, C. Chen^3^, D. J. Lovell^1^, B. Huang^3^

#### ^1^Rheumatology, Cincinnati Children's Hospital , Cincinnati; ^2^Pediatrics, Johns Hopkins University, Baltimore; ^3^Biostatistics & Epidemiology, Cincinnati Children's Hospital , Cincinnati, United States

##### **Correspondence:** H. I. Brunner

*Pediatric Rheumatology 2023*, **21(Suppl 2):**PT082

**Introduction:** Physician Global Assessment of Disease Activity (PhGA) are commonly used as outcome measures in pediatrics rheumatology. For cSLE, the traditional visual analog scale (range: 0 – 10; 0=inactive; 10= very active; PhGA_0-10_) but also the SELENA-SLEDAI (range: 0-3; 0= none, 1=mild,2=moderate, 3=severe; PhGA_0-3_) are used to measure treatment response, flare, and Lupus Low Disease Activity Status with PhGA_0-3_ <1.

**Objectives:** To compare the measurement properties of the PhGA_0-10_ and the PhGA_0-3_in cSLE and with scores of the SLEDAI-2k, and the SELENA-SLEDAI.

**Methods:** Secondary data analysis from a convenience sample of 100 cSLE followed every 3 months for up to 7 visits (1). Ratings of PhGA_0-10_, PhGA_0-_ , parent assessment of patient well-being (ParGA ; range:0= very poorly, 10=very well), SLEDAI-2k and SELENA-SLEDAI were compared. After linear transformation of PhGA_0-10_ to a 0-3 range (tPhGA_0-10_) frequency of PhGA_0-3_<1 were compared.

**Fig. 1 (abstract P463).**
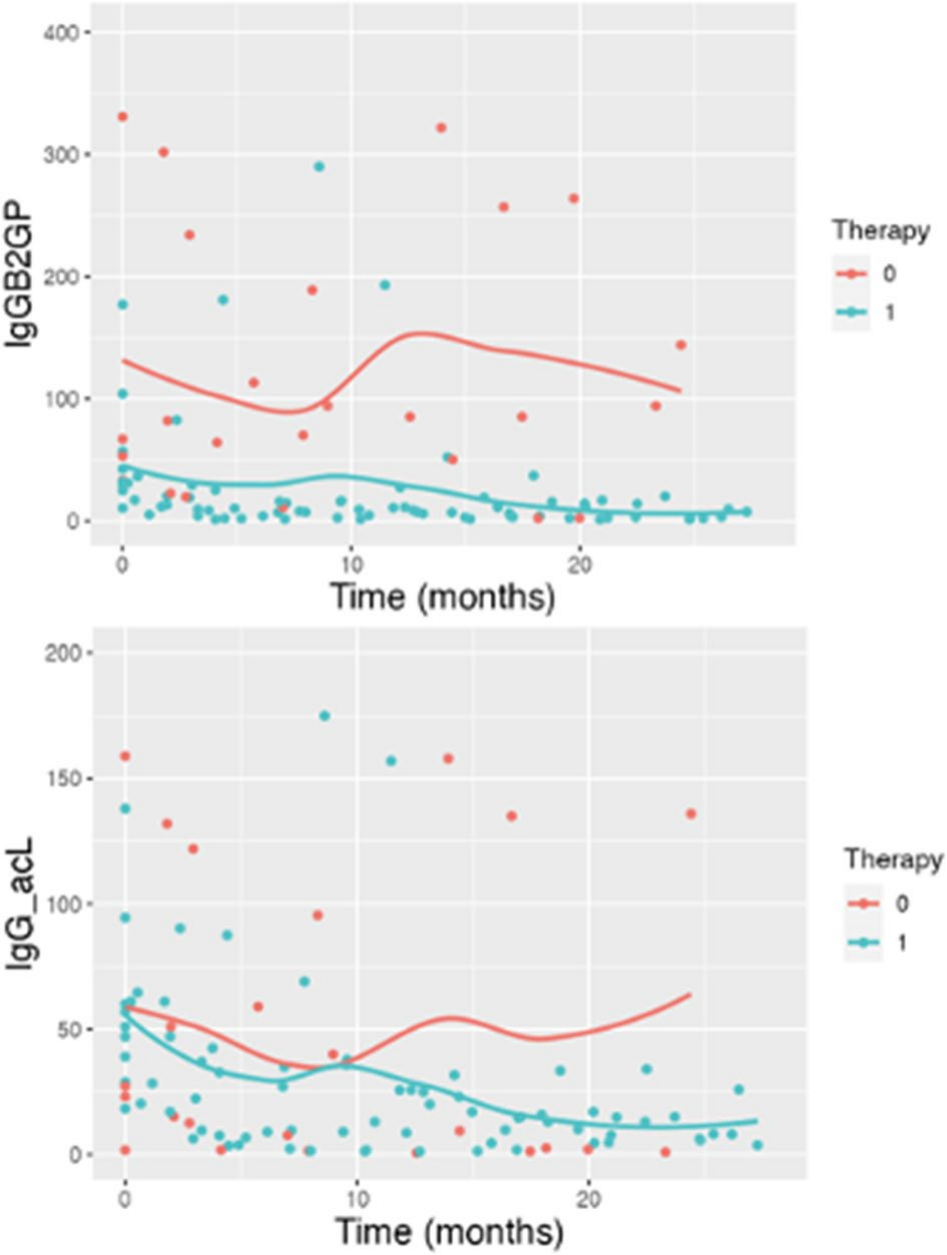
See text for description.

**Results:** In 601 visits, mean (SD)/median (range) of PhGA_0-10_, PhGA_0-3_, SLEDAI-2K, SELENA-SLEDAI were 2.13 (1.87)/2 (0-10), 0.79 (0.64)/1(0-3),4.63 (4.14)/ 4 (0-28) and 4.51 (4.1) / 4 (0-32). PhGA_0-10_ were moderately correlated with PhGA_0-3_ (r=0.73; p<0.0001; Figure 1) with more variability for PhGA_0-3_ >2. ParGA was weakly correlated with PhGA_0-10_, PhGA_0-3 ,_ SLEDAI-2k and SELENA-SLEDAI scores (r = -0.34, -0.30, -0.19 and -0.20). SELENA-SLEDAI and SLEDAI-2k scores were highly (r=0.98) correlated with each other. However, SLEDAI-2K/SELENA-SLEDAI scores were weakly correlated with PhGA_0-3_ (r=0.28/0.28; p <.001) and moderately correlated with PhGA_0-10_ (r= 0.56/0.54; p <.0001). There were 490/497 of 601 visits with PhGA_0-3_ <1 / tPhGA_0-10_ < 1 [Kappa (SE)=0.59 (0.04), McNemar p=0.4].

**Conclusion:** Using the traditional PhGA_0-10_ in cSLE yields almost identical LLDAS rates compared to the PhGA_0-3_ . Given its closer association with the scores of disease activity indices in cSLE, use of the PhGA_0-10_ may be preferable in pediatric populations.

**Patient Consent**

Yes, I received consent

**Disclosure of Interest**

None declared

**Reference**

1. Mina R, Klein-Gitelman MS, Nelson S, Eberhard BA, Higgins G, Singer NG, Onel K, Tucker L, O'Neil KM, Punaro M, Levy DM, Haines K, Martini A, Ruperto N, Lovell D, Brunner HI. Validation of the systemic lupus erythematosus responder index for use in juvenile-onset systemic lupus erythematosus. Ann Rheum Dis. 2014 Feb;73(2):401-6. PMID: 23345596.

## PT083 Indications, efficacy, and tolerance for rituximab in childhood-onset systemic lupus erythematosus: a retrospective study of the JIR cohort

### J. Cognard^1^, H. Reumaux^2^, D. Leguevaques^2^, M. Hofer^3^, M. Pha^4^, I. Kone-Paut^5^, L. Rossi^5^, F. Aeschlimann^6^, I. Melki^6^, P. Quartier^6^, C. Pietrement^1^, D. Urbina^7^, C. Rebelle^7^, P. Pillet^8^, Y. Hatchuel^9^, A. Felix^9^, B. Bader-Meunier^6^, A. Belot^10^

#### ^1^General Pediatrics Department, American Memorial Hospital, CHU Reims, Reims Champagne-Ardenne University, Reims; ^2^Pediatric Rheumatology Department, Jeanne de Flandre University Hospital, Lille, France; ^3^Department of Paediatrics, Centre Hospitalier Universitaire Vaudois (CHUV), Geneva, Switzerland; ^4^Sorbonne Université, Assistance Publique-Hôpitaux de Paris, Groupement Hospitalier Pitié-Salpêtrière, French National Referral Center for Systemic Lupus Erythematosus, Antiphospholipid Antibody Syndrome and Other Autoimmune Disorders; ^5^Department of Paediatric Rheumatology, Bicêtre University Hospital; ^6^Department of Paediatric Hematology-Immunology and Rheumatology, Necker-Enfants Malades Hospital, Assistance Publique-Hôpitaux de Paris, Reference Centre for Rheumatic, Autoimmune and Systemic Diseases in Children, Paris; ^7^Paediatric Department, Marseille University Hospital Saint Joseph, Marseille; ^8^Paediatric Department, Bordeaux University Hospital Pellegrin, Bordeaux; ^9^General Pediatrics Department, Martinique, Fort-De-France; ^10^University of Lyon, CIRI, INSERM U1111, National Referee Centre RAISE, Pediatric Rheumatology, HFME, Lyon, France

##### **Correspondence:** J. Cognard

*Pediatric Rheumatology 2023*, **21(Suppl 2):**PT083

**Introduction:** Childhood-onset systemic lupus erythematosus (cSLE) is a rare autoimmune disease that leads to significant morbidity. There is a lack of studies examining the use of Rituximab (RTX), a B-cell depleting agent, in the pediatric lupus population.

**Objectives:** The aim of this study was to retrospectively evaluate the current indications, efficacy, and adverse effects of Rituximab in the treatment of cSLE in within the Juvenile Inflammatory Rheumatism (JIR) cohort (Pedialup module).

**Methods:** We conducted a national retrospective study of medical records and data collected within the JIR cohort on patients treated by RTX at a their pediatric age, over a period from July 2009 to February 2022.

**Results:** A total of 41 patients received 132 courses of Rituximab over a 12 year period. 35 (85,4%) were female, with a mean age at diagnosis of 11,7 years. The median administration of RTX occured 16 months after diagnosis. At the initiation of Rituximab treatment, 87% of children had received or were receiving corticosteroids, 21% were on NSAIDs, 82% were on immunosuppressants (MMF, MTX, AZA, CYC, TAC) and 95% were on hydroxychloroquine. The primary indications were Lupus nephritis (51,2%), aggressive polyarthritis with steroid dependence (19,5%) and refractory cytopenia (12,2%). Additionally, 3 children (7,3%) were treated for neuropsychiatric disorders. SLEDAI clinico-biological disease activity score demonstrated statistically significant improvements at 3 and 6 months after RTX perfusion (*p<* 0,001 ; one-way ANOVA followed by Tukey’s multiple comparisons test), along with a cortisone-sparing effect (from 0,93 mg/kg to 0,39 mg/kg ; *p=*0,001) and improvements of relevant biomarkers. Treatment efficacy appeared to be more prononced in children with extra-membranous glomerulonephritis, although this subgroup was small (*p*=0,03). Adverse effects occurred in 17 % of patients, including 7,3% with anaphylactic reactions that resolved upon discontinuation of treatment. No cases of severe infection have been reported during the first 12 months follow-up. However, one year after the last known infusion, 48% of the children required an intensification of their background immunosuppressive treatment or initiation of a new therapeutic approach.

**Conclusion:** The use of RTX in cSLE appears to be safe and effective, particularly in the treatment of lupus nephritis, polyarthritis and refractory cytopenia leading to a reduction in disease activity and in steroid usage. Further studies and international collaboration are required to confirm and expend these data.

**Patient Consent**

Yes, I received consent

**Disclosure of Interest**

None declared

**References**

1. Sawhney S, Agarwal M. Rituximab use in pediatric systemic lupus erythematosus: Indications, efficacy and safety in an Indian cohort. Lupus. 2021 Oct
2. Tambralli A, Beukelman T, Cron RQ, Stoll ML. Safety and Efficacy of Rituximab in Childhood-onset Systemic Lupus Erythematosus and Other Rheumatic Diseases. J Rheumatol 2015
3. Watson L, Beresford MW, Maynes C, Pilkington C, Marks SD et al. The indications, efficacy and adverse events of rituximab in a large cohort of patients with juvenile-onset SLE. Lupus 2015

## PT084 Application of criss score, revised criss score and RCID score in patients with diffuse cutaneous Juvenile systemic sclerosis

### J. Klotsche^1^, I. Foeldvari^2^, K. Torok^3^, F. Del Galdo^4^, D. Furst^5^, O. Kasapcopur^6^, A. Adrovic^6^, B. Feldman^6^, M. T. Terreri^6^, A. P. Sakamoto^6^, F. Sztajnbok^6^, J. Anton^6^, M. Katsicas ^6^, V. Stanevicha^6^, S. Appenzeller^6^, T. Avcin ^6^, S. Johnson^6^, M. Kostik^6^, H. Malcova^6^, E. Marrani^6^, W.-A. Sifuentes-Giraldo^6^, R. Khubchandani^6^, D. Nemcova ^6^, M. J. Santos^6^, D. Schonenberg-Meinema^6^, C. Battagliotti^6^, L. Berntson^6^, B. Bica^6^, J. Brunner^6^, D. Eleftheriou^6^, L. Harel^6^, G. Horneff^6^, T. Kallinich^6^, T. Lehman^6^, K. Minden^6^, M. Moll^6^, S. Nielsen^6^, A. Patwardhan^6^, V. Smith^6^, N. Helmus^2^

#### ^1^German Rheumatism Research Center, Berlin; ^2^Hamburg Centre for Pediatric and Adolescence Rheumatology, Hamburg, Germany; ^3^University of Pittsburgh, Children’s Hospital of Pittsburgh, Pittsburgh, United States; ^4^University of Leeds, Leeds, United Kingdom; ^5^University of California, University of Florence, Florence, Italy; ^6^jSSc Collaborative Group, Hamburg, Germany

##### **Correspondence:** I. Foeldvari

*Pediatric Rheumatology 2023*, **21(Suppl 2):**PT084

**Introduction:** Juvenile systemic sclerosis (jSSc) is a rare disease in childhood. To date, no composite response index exists to assess treatment effect in jSSc patients. ACR CRISS score (probability of improvement ranging from 0 to 1 based on mRSS, FVC%, PtGA, MDGA and HAQ-DI) and revised ACR CRISS (rCRISS, proportion of patients who improve in ≥ 3/5 ACR CRISS core items by a certain percentage, e.g. 30%, except 5% for FVC) were developed by experts in the field as outcome measures in adult patients with SSc. In addition, the Ranked Composite Important Difference (RCID) score was recently introduced as anchor to the ACR CRISS.

**Objectives:** We aimed to study the applicability and performance of the ACR CRISS, rCRISS and RCID in a prospectively followed cohort of patients with diffuse cutaneous jSSc.

**Methods:** Data from the international jSSc inceptions cohort were used for this analysis. The ACR CRISS, rCRISS and RCID were calculated between baseline and 12-months follow-up according to the scoring algorithms. Missing values in the core items were estimated by multiple imputation by chained equations. Here we aimed to determine the value of the response measures to detect clinically change defined by the anchor questions about change (much better or little better versus almost the same, little worse or much worse) in patients overall health due to scleroderma since the last visit provided by the treating physicians and parents or patients (aged > 12 years).

**Results:** We included 95 jSSc patients with diffuse cutaneous subtype with available baseline and 12-months visit. Seventy-nine percent were female, the mean age at enrollment was 13.0 (3.8) and the mean disease duration was 3.1 (2.8) years. Among 95 patients, 57% were treated with steroids, 47% with methotrexate, 27% with MMF and 3% with a biological at baseline. ACR CRISS showed a ceiling effect (>.998) in 51% and a floor effect (<0.005) in 26% of patients. Patients who reported at least moderate improvement had a median ACR CRISS of 0.99 and in mean 2.6 (1.3) core items that improved by ≥20% from baseline to 12-months follow-up. The rCRISS 20/30/50 responses were 59%/49%/33% in patients who reported improvement and 25%/25%/8% in patients with worsening. The RCID was approximately normal distributed (mean 20.7, SD 43.4). Mean (SD) RCID for patients who reported worsening was -10.5 (38.6) vs RCID of 20.7 (45.2) for patients who reported improvement. RCID scores for physician reported anchors of worsening or improvement were 6.5 (44.2) and 18 (45.4) , respectively. The concordance between a positive RCID score and rCRISS 20/30 was moderate (rCRISS 20 and RCID, 43%, kappa=0.43; rCRISS 30 and RCID, 38%, kappa=0.36).

**Conclusion:** Our data confirmed the presence of a ceiling and floor effect of ACR CRISS as shown in studies of adult SSc patients. The CRISS, rCRISS and RCID response distinguished between patients who rated their disease course since last visit as worsened or improved. Future studies should focus on the determination of specific pediatric weights for the CRISS and RCID components rather than extrapolation from adult SSc. In general, the RCID offers a meaningful tool in order to determine response to therapy in future clinical trials in jSSc patients.

**Patient Consent**

Not applicable (there are no patient data)

**Disclosure of Interest**

None declared

## PT085 Together we are stronger: the lupus café, reflections on building peer support groups and collaborative partnerships between patients with rheumatic diseases, healthcare professionals and charities

### E. Moraitis^1,2^, Y. Glackin^3^, A. Affendi^3^, K. Corden^4^, S. Ruane^5^, P. Howard^6^

#### ^1^Rheumatology, Great Ormond Street Hospital for Children NHS Foundation Trust; ^2^Infection, Immunity and Inflammation, GOS UCL Institute of Child Health; ^3^Great Ormond Street Hospital for Children NHS Foundation Trust; ^4^Clinical Psychology, Oxford University Hospitals NHS Foundation Trust; ^5^Clinical Psychology, Great Ormond Street Hospital for Children NHS Foundation Trust; ^6^Chief Executive Officer , Lupus UK, London, United Kingdom

##### **Correspondence:** E. Moraitis

*Pediatric Rheumatology 2023*, **21(Suppl 2):**PT085

**Introduction:** In the recent years, healthcare is moving away from the traditional view of the patient as passive recipient of services. Organisations around the world have increased their efforts to involve patients and make their participation in healthcare active using various forms of engagement.

**Objectives:** The aim of this project was to describe the development of a peer support and partnership group for patients and families living with lupus, healthcare professionals and charity.

**Methods:** Lupus Café was an idea generated from feedback in the lupus clinics at Great Ormond Street Hospital (GOSH).Young people and families reported that they were feeling isolated living with lupus. At the appointments we offer multidisciplinary advice, support and information but young people were clear that they also want to hear from other young people about their experience of living with lupus.

GOSH is a LUPUS UK Centre of Excellence and due to our large cohort of patients with lupus, we were ideally situated to facilitate this support in partnership with LUPUS UK and allow families and young people to meet each other.

Lupus Café started in 2021 as a virtual meeting for patients above the age of 10 years and the name was chosen to capture the informal nature of the meeting. We subsequently organised a face-to-face meeting and extended the invites to patients and families from other UK centres. In 2022, with support from PReS Lupus Working Party, we organised the first ever international event for children and families living with lupus. The meetings were facilitated by a team of lupus nurse, psychologist, doctor and charity representatives. Detailed feedback was collected at each meeting and the agenda of the meetings was developed based on the suggestions provided in the feedback forms.

**Results:** We have facilitated 4 patient and families group events between 2021-2023, of which 3 virtual (one with international patient and clinicians participation from 3 continents) and one face to face event with national participation. The feedback was positive and 93% participants indicated that they wish to attend further meetings and 7% that they will maybe attend. The patients indicated as the most positive aspects “meeting others with lupus and getting advice from each other”, “sharing experiences”, “having questions answered by professionals and other parents”, “a lot of useful information and resources”, “hearing about research”. Clinical teams’ feedback revealed that listening to patients’ and families’ lived experience of the disease and healthcare journeys identified areas for service improvement and also increased their awareness of research priority areas.

**Conclusion:** The patient support groups improve the quality of life of young people. In addition, patient and charities partners can transform the clinical development process and should be embedded in the care of patients with rheumatic disease. They are mutually beneficial, educational, and the unique perspectives provided by patients’ lived experiences can inform clinicians’ and researchers’ approaches.

**Patient Consent**

Not applicable (there are no patient data)

**Disclosure of Interest**

None declared

## PT086 Comparison of outcome in 101 childhood lupus nephritis with diverse immunosuppressives in two different centers in metropolitan cities - New Delhi , India and London, UK

### D. B. Pandya^1,2^, M. Aggarwal ^2^, M. Marlais^3^, E. Moraitis^1^, S. Sawhney^2^, C. Papadopoulou^1,4^, M. Al Obaidi^1,4^

#### ^1^Pediatric Rheumatology, Great Ormond Street Hospital for Children, London, United Kingdom; ^2^Pediatric & Adolescent Rheumatology , Sir Gangaram Hospital , Delhi , India; ^3^Pediatric Nephrology, Great Ormond Street Hospital for Children; ^4^Institute of Child Health, GOSH UCL, London, United Kingdom

##### **Correspondence:** D. B. Pandya

*Pediatric Rheumatology 2023*, **21(Suppl 2):**PT086

**Introduction:** Lupus nephritis (LN) is more common and aggressive in paediatrics compared to adults^**1**^**.** There are several studies in adults showing better outcomes with various immunosuppressive combinations in LN^**2**^**.** There is scarcity of such data in paediatric LN^**3**^.

**Objectives:** To compare the outcome of childhood-LN with different immunosuppressives in two tertiary Paediatric Rheumatology Centres, Sir Gangaram Hospital (SGRH) and Great Ormond Street Hospital for Children(GOSH) at 12-months follow-up.

**Methods:** This is a retrospective study including 101(SGRH=61, GOSH=40) children with biopsy proven class III-IV-V-LN, treated between January-2011 to December-2021. Renal outcomes were assessed using the CARRA definitions of substantial response, moderate response and renal flare^**4**^. Systemic Lupus Erythematosus Disease Activity Index (SLEDAI) and dose of steroids at onset were calculated. Statistical analysis was performed by T tests and Chi Square static with yates' correction.

**Results:** Median age of onset was 12.6years in SGRH and 13 years in GOSH. M:F ratio was 1:2.8 in SGRH and 1:4 in GOSH. Average SLEDAI at onset was significantly higher in GOSH-24.5 compared to SGRH-17(p<0.05). Intravenous (IV) pulse glucocorticoids (GC) were given in 49/61(80%) and 31/40(78%) in SGRH & GOSH respectively. Average dose of IV GC was significantly higher in GOSH-28.5 vs SGRH-8.4 mg/kg/day(p<0.05). Average starting dose of oral steroids was higher in SGRH-1 vs GOSH-0.8mg/kg/day(p<0.05). For Induction(0-6months), SGRH patients received six doses IV cyclophosphamide (CYC, N=18/61), Mycophenolate mofetil (MMF,N=39/61) and Rituximab(RTX,N=4/61) while GOSH patients received RTX+MMF(N=19/40), RTX+ two doses CYC(N=8/40), RTX+Azathioprin(AZA,N=4/40) and MMF(N=9/40). For maintenance(6-12months), SGRH patients received MMF(N=38/61), MMF+Tacrolimus(N=14/61), RTX(N=6/61) and AZA(N=3/61) while GOSH patients received MMF(N=24/40), RTX(N=8/40) and AZA(N=8/40). Overall, there is no significant difference in substantial response (p 0.38), moderate response (p 0.77) and off GC (p 0.85) between the two centres. There is no significant difference between CYC vs RTX+MMF in substantial response (p 0.88), moderate response (p 0.64), renal flares (p 0.37) and being off GC (p0.79).Similarly, no significant difference between CYC vs RTX+CYC in substantial response (p 0.92), moderate response (p 0.38), renal flares (p 0.44) and being off GC (p 0.97).

**Conclusion:** LN outcomes are comparable between the two centres, despite the significant differences in disease severity, dose of GC and choice of immunosuppressives at onset. Larger multicentre studies are needed to look into long-term efficacy and safety of the different immunosuppressive agents in paediatric LN.

**Patient Consent**

Yes, I received consent

**Disclosure of Interest**

D. B. Pandya Grant / Research Support with: I have been awarded by Asia Pacific League of Associations for Rheumatology (APLAR) and PReS EMERGE grants to work as an International Fellow at Great Ormond Street Hospital , London, UK. This project is an integral part of this fellowship. , M. Aggarwal : None declared, M. Marlais: None declared, E. Moraitis: None declared, S. Sawhney: None declared, C. Papadopoulou: None declared, M. Al Obaidi: None declared

**References**

1. VAH Sato et al , Lupus 2012 Aug;21
2. Jin Deng et al , Turk J Med Sci 2018 Oct 31
3. Aeagon et al , Lupus 2016 Apr 25
4. Rina Mina et al , Arthritis Care Res 2013 March 1

## PT087 Correlation of nailfold capillaroscopy changes with disease activity and damage scores in juvenile dermatomyositis: an observational study from North India

### S. Reddy^1^ on behalf of Pandiarajan Vignesh, Suprit Basu, Prabal Barman, Vinay Keshavamurthy, Deepti Suri, Surjit Singh, V. Pandiarajan^1^, S. Basu^1^, P. Barman^1^, V. Keshavamurthy^2^, D. Suri^1^, S. Singh ^1^

#### ^1^Pediatrics, ^2^Dermatology and Venereology, PGIMER, Chandigarh, India

##### **Correspondence:** S. Reddy

*Pediatric Rheumatology 2023*, **21(Suppl 2):**PT087

**Introduction:** Assessment of capillary abnormalities in Juvenile Dermatomyositis (JDMS) may provide us a sensitive diagnostic and also a valid indicator of disease activity. However, association of NFC changes with myositis specific autoantibodies (MSA) and myositis damage index are not known.

**Objectives:** To study the NFC findings of JDMS and to correlate it with disease activity scores like Childhood myositis assessment scale (CMAS), Manual muscle testing-8 (MMT-8), Muscle disease activity score (MDAS), Skin disease activity score (SDAS) and Myositis Damage Index (MDI).

**Methods:** We enrolled 44 children with JDMS and 25 healthy controls. Of the 44 cases, 10 children were newly diagnosed with JDMS and NFC was done at presentation and 2 monthly for duration of 6 months. NFC was done during follow-up for the remaining 34 patients. We performed NFC using Digital Capillaroscope with OptiPix^TM^ Capillaroscopy and assessed quantitative parameters like length, width and inter-capillary distance and qualitatively for abnormal capillary morphology. MMT8, CMAS, MDAS, SDAS and MDI were assessed at each visit.

**Results:** Capillary tortuosity, oedema, enlarged loops and ramification was seen in 88.6%, 15.9%, 72.7% and 56.8% in cases as compared to 68%, 4%, 8% and 8% in controls. Other parameters like avascularity (81.6%), micro-bleeding (54.5%), giant capillaries (29.5%), bushy capillaries (40.9%) were seen in only in cases. Of the 10 children with JDMS enrolled for prospective follow-up, we noted significant improvement only in capillary density at 6 months duration (p=0.002). Of the MSA subgroups (10 NXP2, 7 TIF-gamma, 5 MDA5, 3 SAE-1, 3 Mi2-beta), we noted higher proportions of microbleeding in the TIF-gamma subgroup. Of the total 61 instances in which the NFC is performed, we noted that capillary density, microbleeding, and giant capillaries correlated with both skin and muscle disease activity measures. Bushy capillaries correlated only with skin disease activity (p=0.009). We also noted that avascularity index (p=0.025) and capillary density (p=0.025) correlated best with myositis damage index

**Conclusion:** Our study reiterates that capillaropathy plays a significant role in pathogenesis of JDMS. Only capillary density and avascularity corelated with disease damage score (MDI). Longitudinal follow-up revealed capillary density as a marker of improvement of disease activity

**Patient Consent**

Not applicable (there are no patient data)

**Disclosure of Interest**

None declared

## PT088 Type-I interferon signatures in patients with Juvenile-onset sle are heterogeneous and associated with inflammation and cardiometabolic risk irrespective of disease activity

### S. Atif, J. Peng, E. C. Jury, C. Ciurtin, G. A. Robinson

#### Rheumatology, University College London, London, United Kingdom

##### **Correspondence:** G. A. Robinson

*Pediatric Rheumatology 2023*, **21(Suppl 2):**PT088

**Introduction:** Patients with juvenile-onset systemic lupus erythematosus (JSLE, onset <18 years) typically have more severe disease and relatively higher cardiovascular and mortality risk compared to adult-onset patients. This could be associated with more predominant type-I interferon (IFN) signalling.

**Objectives:** We investigated the heterogeneity of type-I IFN transcriptomic signatures in JSLE patients and their relationship with inflammatory pathways and co-morbidities using multi-omic analysis.

**Methods:** RNA sequencing (UCL Genomics) was used to assess differentially expressed genes (DEGs, p<0.01) in peripheral blood mononuclear cells (PBMCs) between JSLE patients with low disease activity (n=29, mean age=19, SLEDAI<4) and healthy controls (HCs, n=8, mean age=18). Data was analysed by gene ontology pathway enrichment and network analysis, hierarchical clustering, receiver operating characteristic (ROC) analysis, and comparison of normalised gene counts. Proteomics (Olink) and Metabolomics (Nightingale) assessed serum proteins and metabolites, respectively, associated with inflammation and cardiovascular disease.

**Results:** JSLE patients had significantly enriched type-I IFN signalling pathways compared to HCs (p<0.0001) associated with a vast network of pro-inflammatory pathways. IFN scores correlated positively with proteomic (such as ICAM1 and VCAM1) and metabolic (such as glycoprotein acetyls and the Apolipoprotein(Apo)B:A1 ratio) biomarkers known to reflect inflammation and cardiovascular disease risk. Interestingly, IL-10 signalling was the most significantly upregulated pathway in JSLE patients (vs HCs) independent from IFN-associated DEGs. Despite low disease activity, patients clustered into a high (H-IFN, 66%) and low (L-IFN, 34%) IFN signature group using normalised gene counts and validated by IFN score (p<0.0001). There was no difference in IFN score between the L-IFN group vs HCs (ROC: p=0.53, AUC=0.59), in contrast to a significantly higher IFN score in the H-IFN group vs HCs (ROC: p<0.0001, AUC=1.00). 281 DEGs were upregulated in the H-IFN (vs L-IFN) group, where the most enriched pathways (aside from IFN signalling) were cell cycle associated, with top contributing genes REC8 and PSME2. Finally, there was no significant difference in 5-year average SLEDAI or serological measures of disease between IFN groups, supporting the pro-inflammatory role of type-I IFN signatures independent of standard measures of disease activity.

**Conclusion:** JSLE patients can be stratified based on type-I IFN signatures associated with proinflammatory mechanisms, cardiovascular disease risk, and upregulated cell cycle pathways even in low disease activity states. This suggests that targeted biomarker patient stratification or therapeutic interventions may prevent long-term consequences of molecular dysregulation underlying low/moderate disease activity in JSLE.

**Patient Consent**

Yes, I received consent

**Disclosure of Interest**

None declared

## PT089 Primary antiphosfolipid syndrome in pediatrics. report of 51 cases

### A. R. Torres-Jimenez^1^, V. RAMIREZ-NOVA^1^, A. I. CESPEDES-CRUZ^1^, B. SANCHEZ-JARA^2^, A. VELAZQUEZ-CRUZ^1^, V. C. BEKKER-MENDEZ^3^, F. X. GUERRA-CASTILLO^3^

#### ^1^Department of Pediatric Rheumatology; ^2^Department of Pediatric Hematology, ^3^IMSS National Medical Center La Raza, CDMX, Mexico

##### **Correspondence:** A. R. Torres-Jimenez

*Pediatric Rheumatology 2023*, **21(Suppl 2):**PT089

**Introduction:** The antiphospholipid syndrome is not well defined in pediatric population and there are no validated criteria at this age. The criteria for adults are specific but lack sensitivity when applied to children, so the incorporation of non-criteria clinical manifestations is important in the pediatric population.The preliminary classification criteria for antiphospholipid syndrome were recently shown in ACR convergence 2022. Its usefulness in the pediatric population is not yet known.

**Objectives:** Describe the frequency of thrombotic and non-thrombotic clinical manifestations, laboratory and treatment in patients with pediatric primary antiphospholipid syndrome. To test the classification criteria recently shown in ACR convergence 2022 in the pediatric population.

**Methods:** A retrospective study was carried out in patients with a diagnosis of primary antiphospholipid antibody syndrome, under 16 years of age, under follow-up by the pediatric rheumatology service of the General Hospital, National Medical Center, La Raza, from January 2013 to April 2023. The antiphospholipid syndrome was defined when it met the laboratory criteria of the Sidney criteria and the presence of thrombosis or non-criteria manifestations of the disease (hematological, neurological, cutaneous, renal, cardiac or pulmonary). Demographic, clinical, laboratory, treatment, and prognosis data were collected. Patients were classified according to the preliminary antiphospholipid syndrome criteria presented at ACR convergence 2022 to determine their usefulness in pediatric population.

**Results:** We report 51 patients, 35 female (69%) and 16 male (31%), mean age 12.24 years, evolution time 19 weeks. Thrombosis 11 patients (22%), 3 arterial and 8 venous. Only thrombotic manifestations in 2 (4%), only non-thrombotic manifestations in 40 (78%) and both in 9 (18%). Non-thrombotic manifestations; Hematologic: thrombocytopenia 35 patients (69%), autoimmune hemolytic anemia 24 (47%), Fisher-Evans syndrome 14 (28%), lupus anticoagulant with hypoprothrombinemia syndrome 2 (4%). Dermatological: livedo reticularis 28 (55%), skin ulcers 3 (6%), Raynaud's phenomenon 11 (22%). Neurological: epilepsy 1 (2%), migraine 4 (8%), chorea 1 (2%) and cognitive impairment 3 (6%). Renal in 8 (16%). Laboratory: prolonged aPTT 47 (92%), lupus anticoagulant 51 (100%), positive IgG anticardiolipin 36 (71%), positive IgM anticardiolipin 31 (61%). AntiB2GPI was performed in only 7 patients, being positive in all. Treatment: anticoagulation in patients with thrombosis, antiplatelet in 41 (80%), steroid 47 (92%), immunosuppressant 46 (90%) and rituximab 6 (12%). According to the preliminary criteria of antiphospholipid syndrome presented at ACR convergence 2022, it was possible to classify 26 patients (51%) with primary antiphospholipid syndrome, with the Sydney criteria only 11 patients (22%). If other hematological manifestations are added, giving the same value as thrombocytopenia to hemolytic anemia, as well as 3 points to the combination of hemolytic anemia and thrombocytopenia or to the lupus anticoagulant with hypoprothrombinemia syndrome, 13 more patients were added, classifying a total of 39 patients as primary antiphospholipid syndrome (76%).

**Conclusion:** The clinical characteristics of patients with pediatric primary antiphospholipid syndrome differ from those presented in adults, since non-thrombotic manifestations are more frequent in children. The criteria recently shown in ACR Convergence 2022 improve the classification of pediatric patients with primary antiphospholipid syndrome, but they still fail to classify half of the patients, so it is suggested to add other hematological manifestations to these in order to improve their usefulness in pediatric population.

**Trial registration identifying number:** 1

**Patient Consent**

Not applicable (there are no patient data)

**Disclosure of Interest**

None declared

## PT090 The efficacy and safety of jak-inhibitors in patients with refractory Juvenile dermatomyositis: single center experience

### I. Tsulukiya^1^, E. Alexeeva^1,2^, T. Dvoryakovskaya^1,2^, O. Lomakina^1^, A. Fetisova^1^, K. Isaeva^1^, A. Chomakhidze^1^, K. Chibisova^1^, I. Kriulin^1,2^, E. Krekhova^1^, M. Shingarova^1,2^, M. Botova^1^, N. Kondrateva^1^, T. Kriulina^1,2^, M. Kokina^1,2^

#### ^1^Rheumatology, National Medical Research Center of Children's Health; ^2^Pediatric, Sechenov First Moscow State Medical University, Moscow, Russian Federation

##### **Correspondence:** I. Tsulukiya

*Pediatric Rheumatology 2023*, **21(Suppl 2):**PT090

**Introduction:** Some patients with juvenile dermatomyositis (JDM) demonstrate incomplete responses to conventional therapy and some experience disease recurrences. Patients with JDM who are refractory to corticosteroids or other immunosuppressive medications, including biologics such as rituximab and abatacept, face poor outcome and suffer from various sequelae of the disease. Therefore, it is important to find new treatments for refractory JDM. Studies have reported that type I interferon pathways are upregulated in patients with dermatomyositis, and it has been reported that JAK-inhibitors (JAKi) can inhibit interferon signaling and showed an overall good efficacy for refractory dermatomyositis patients.

**Objectives:** To evaluate the efficacy and safety of JAK-inhibitors in children with refractory dermatomyositis.

**Methods:** This was a single-center retrospective study, including 17 (7—male, 10—female) children with refractory JDM. Refractory JDM was defined as patients who failed two or more steroid sparing agents, including biologic agents, or high-dose steroids. They were all treated using JAKi combined with steroids and other immunosuppressive agents**.** A mean age of JAK-inhibitors initiation was 8,4 years (range 2,1 –16,7 years).

**Results:** Seventeen refractory patients with JDM treated with tofacitinib (n = 9) or upadacitinib (n = 8) were included. Median of duration of disease prior to initiation JAKi was 8,2 (IQR 5,0 – 13,8) months. At the baseline, patients received CS (n = 17), methotrexate (MTX, n = 2), hydroxychloroquine (HCQ, n = 2), mycophenolate mofetil (MMF, n = 6), cyclosporine A (CsA, n = 2). 8 (47%) patients had received previous bDMARDs; 9 (53%) were bDMARD naïve. The main indications for treatment were refractory muscle involvement (n = 7) and skin rash (n = 10). The skin rash and muscle weakness improved in 14/17 (82%) patients within 3 months of JAKi introduction. All responders could decrease the dose of steroid. The mean daily steroid dose decreased from 1,3 mg/kg/d (range 0,35-2 mg/kg/d) to 0,4 (range, 0,3-0,8).

All patients responded well to JAK-inhibitors with significant improvement in clinical and inflammatory indices without occurrence of severe adverse events.

**Conclusion:** This study showed improvement of muscle strength, resolution of cutaneous lesions, increased daily quality of life and successful tapering of steroids when JAK-inhibitors used. Tofacitinib and upadacitinib can be considered when treating refractory JDM cases. Further randomized controlled trials are warranted to assess its efficacy in JDM.

**Patient Consent**

Not applicable (there are no patient data)

**Disclosure of Interest**

None declared

## PT091 Safety and efficacy of biologic therapies in refractory/severe pediatric behçet’s disease: an international cohort

### Ö. Akgün^1^, F. G. Demirkan^1^, T. Coşkuner^2^, V. Çam^3^, M. Polat^4^, E. Esen^5^, N. Şahin^6^, Ö. Baba^7^, G. Kılbaş^8^, N. G. Kocamaz^9^, K. Öztürk^10^, D. Rigante^11^, M. Jelusic^12^, S. Özdel^9^, S. Yüksel^8^, M. Kalyoncu^7^, H. E. Sönmez^6^, A. Paç Kısaarslan^5^, E. Çelikel^4^, M. V. Mastrolia^13^, E. D. Batu^3^, S. Özen^3^, B. Sözeri^2^, N. Aktay Ayaz^1^

#### ^1^Pediatric Rheumatology, İstanbul Faculty of Medicine; ^2^Pediatric Rheumatology, Ümraniye Training and Research Hospital, İstanbul; ^3^Pediatric Rheumatology, Hacettepe University Faculty of Medicine; ^4^Pediatric Rheumatology, Ankara Bilkent City Hospital, Ankara; ^5^Pediatric Rheumatology, Erciyes universty Faculty of Medicine, Kayseri; ^6^Pediatric Rheumatology, Kocaeli Universty Faculty of Medicine, Kocaeli; ^7^Pediatric Rheumatology, Karadeniz tecnical universty Faculty of Medicine, Trabzon; ^8^Pediatric Rheumatology, Pamukkale Universty Faculty of Medicine, Denizli; ^9^Pediatric Rheumatology, Ankara Etlik City Hospital, Ankara; ^10^Pediatric Rheumatology, Goztepe Prof. Dr. Süleyman Yalçın City Hospital, İstanbul, Türkiye; ^11^Department of Life Sciences and Public Health, Fondazione Policlinico Universitario A. Gemelli IRCCS Università Cattolica Sacro Cuore, Rome, Italy; ^12^Pediatric Rheumatology, University of Zagreb School of Medicine, Zagreb, Croatia; ^13^Pediatric Rheumatology, Meyer Children Hospital IRCCS, Firenze, Italy

##### **Correspondence:** Ö. Akgün

*Pediatric Rheumatology 2023*, **21(Suppl 2):**PT091

**Introduction:** Behçet's disease (BD) is a chronic and recurrent multisystem inflammatory disease that can involve vessels of all sizes and types, classified as variable vascular vasculitis. Recurrent oral and/or genital aphthae, uveitis, ocular findings, and skin lesions are the main symptoms of BD.

**Objectives:** It is aimed to collect international data on the safety and efficacy of biologic therapies in patients with resistant/severe pediatric BD.

**Methods:** This study was designed to be retrospective, observational, multicenter, and international. Patients who received biologic therapy and met the "International Criteria for Behçet's Disease" and/or "Pediatric Behçet's Disease" and were diagnosed with BD before the age of 18 were included in the cohort.

**Results:** In the preliminary report, 69 patients who met the criteria of the study from 13 centers were included in the cohort. The mean age of the patients was 16±4.5 years, and 31.9% (22) of them were female. Ocular, mucocutaneous, and neurologic involvement were the most common reasons for starting biologic therapy (52.2%, 18.8%, and 14.5%, respectively). Anti-TNF-alpha agents were preferred first-line biological therapy in 66 (95.6%) patients, and interferon and IL-6, and IL-1 blockers were preferred in three patients. The median time to achieve remission of 59 patients with remission data was 3 months (IQR:2-6). A biological switch was performed in 8 (11.5%) patients for various reasons. Four patients using infliximab as the first biologic agent were switched to adalimumab treatment for resistant uveitis. Adalimumab treatment was started in one patient who was diagnosed with uveitis while receiving etanercept treatment. Etanercept treatment was started in one patient who developed a hypersensitivity reaction during adalimumab treatment. When uveitis developed in a patient using anakinra, adalimumab was switched. One (1.4%) of patients in the cohort were in remission without the drug, 58 (84.1%) were in remission with the drug, and 6 (8.7%) were in partial remission. 4 (5.8%) patients were resistant despite biological therapy.

**Conclusion:** Biological drugs are increasingly used in the treatment of pediatric BD. We observed that tumor necrosis factor-alpha inhibitors are the most commonly preferred biological agents. These treatments had an acceptable safety profile and high remission rates.

**Trial registration identifying number:** Ethics committee number:1653276

**Patient Consent**

Yes, I received consent

**Disclosure of Interest**

None declared

**References**

1. Jennette, J. C., Falk, R. J., Bacon, P. A., Basu, N., Cid, M. C., Ferrario, F., ... & Watts, R. A. (2013). 2012
  revised international chapel hill consensus conference nomenclature of vasculitides.
2. Koné-Paut, I. (2016). Behçet’s disease in children, an overview. Pediatric Rheumatology, 14(1), 1-8.
3. Davatchi, F. (2012). Diagnosis/classification criteria for Behcet's disease. Pathology research international, 2012.
4. Batu, E. D., Sönmez, H. E., Sözeri, B., Aviel, Y. B., Bilginer, Y., & Özen, S. (2017). Paediatric rheumatology The performance of different classification criteria in paediatric Behçet’s disease. Clin Exp Rheumatol, 35(108), S119-S123.

## PT092 Expression of inflammasomes (NLRP3, Aim2, NLRC4, NLRP1, NLRP12) and underlying cytokines (Caspase 1, Il-18, and Il-1β) in pre and post patients with kawasaki disease

### K. Arora, R. Rikhi, D. Suri, A. Rawat, S. Singh

#### Advanced Pediatrics centre, Post graduate institute of Medical Education and Research, Chandigarh, India

##### **Correspondence:** K. Arora

*Pediatric Rheumatology 2023*, **21(Suppl 2):**PT092

**Introduction:** Kawasaki disease (KD) is an acute systemic vasculitis of childhood. Both innate and adaptive immune pathways are involved in pathogenesis. Inflammasomes are innate immune system receptors that regulate the activation of caspase-1 and induce inflammation. There is a paucity of information on the role of inflammasomes in KD

**Objectives:** To examine the expression of inflammasomes (NLRP3, NLRC4 and AIM2, NLRP1, NLRP12), caspase- 1, PYCARD, Interleukin- 18 and Interleukin-1β (IL-1β) in treatment naive (pre intravenous immunoglobulin) and post IVIg treatment in children with KD.

**Methods:** 20 patients with KD were enrolled in the study. Diagnosis of KD was based on American Heart Association 2017 criteria. All patients were treated with intravenous immunoglobulin (IVIg) and aspirin. Pre IVIg (n=20) and, 4-6 weeks after treatment, post IVIg (n=20) blood samples were collected. Expression of inflammasome (NLRP3, NLRC4, AIM2, NLRP12, NLRP1), caspase 1, IL-18 and IL-1β were assessed by real time PCR in patients with KD and compared with normal controls.

**Results:** Real time PCR analysis was performed in 20 patients with KD (20 in acute stage; 20 post IVIg) and compared with 20 controls. There was a significant increase in AIM2 expression in patients as compared to controls. There was also increase in IL-1β and IL-18 expression in pre IVIg patients as compared to post IVIg patients. Significant decrease in NLRP12 expression in pre IVIg patients as compared to post IVIg patients. Based on previous data and keeping NLRP3 as key molecule we also grouped patients based on NLRP3 expression. Group 1 patients had reduced NLRP3 in Pre IVIg condition as compared to post IVIg and controls. AIM2 expression was also increased in Group 1 patients. Group 2 patients had significantly increased NLRP3 expression as compared to post patients leading to increased IL-1β expression.

**Conclusion:** Increased expression of NLRP3, caspase 1, IL-18 and IL-1β in patients with acute KD suggests activation of inflammasome pathway in pathogenesis of KD. Previous studies (Wang *et.al*) showed increase in AIM2 in serum of KD patients. There was found to be increase in AIM2 in our set of patients. Blocking of this pathway may provide another therapeutic target for KD.

**Patient Consent**

Yes, I received consent

**Disclosure of Interest**

None declared

**Reference**

1. Wang Z, Wang Q, Jin J, Rong X, Wu T, Qiu H, Wu R. The diagnostic role of AIM2 in Kawasaki disease. Clin Exp Med. 2021 Feb;21(1):41-47.

## PT093 Predicting ivig resistance in kawasaki disease : proposal for an Indian scoring system

### J. N. Bathia^1^, D. Pal^2^, N. Ahmed^3^, H. De^3^, S. Azad^3^, P. Pal^1^

#### ^1^Pediatric Rheumatology; ^2^Institute of Child Health, Kolkata, India; ^3^Pediatric Medicine, Institute of Child Health, Kolkata, India

##### **Correspondence:** J. N. Bathia

*Pediatric Rheumatology 2023*, **21(Suppl 2):**PT093

**Introduction:** Various risk scoring systems have been developed to predict Intravenous immunoglobulin (IVIG) resistance in Kawasaki disease (KD), however, they have not been found to be useful in other ethnicities.

**Objectives:** The aim of the study is to produce a set of cutoff values for the parameters that would best predict IVIG resistance in Kawasaki Disease in the Indian cohort of patients.

**Methods:** The analysis involves predicting the IVIG resistance label of patients for different cutoff values of the parameters and calculating the accuracy of the predictions based on the ground truth labels.

The analysis tried to keep the basic framework of the 3 Japanese scoring methods in the new method proposed.

Dataset was divided into training and test datasets, with the training dataset having more data, and being used to produce the scoring mechanism, and testing dataset being used to compare accuracy of proposed new model to the established methods. The training dataset comprised of 70 patients, 22 being IVIG resistant. The testing dataset had 45,15 were IVIG resistant. Both datasets combined is called Full dataset. For each predictor used in all of the scoring methods, a list of possible points to be tested for being the potential new cutoffs in the scoring mechanism were generated.4 different scoring mechanisms were tested:

- Scoring based on original Kobayashi, where the cutoff (=/>5), all the original Kobayashi predictors (but new cutoff value predicted for each), and the greater/lower than signage corresponding to each predictor was re-used.
- On Sano and Egami, in a similar way.
- A new scoring method, using a structure similar to the established scoring methods, but selecting predictors based on Logistic Regression and testing different values of the cutoff for best performer.

For each mechanism, the corresponding predictors and their list of values on which to test them were used to generate a grid.

For each proposed mechanism, the set of predicted labels for IVIG Resistance was calculated iteratively for each set of points on the grid, and the sensitivity and specificity were calculated. The set of values of the predictors that resulted in the best prediction accuracy were noted.To allow the proposed cutoff values of predictors in the scoring mechanism to generalize well to unseen future data, a K-fold cross validation (with K=5) approach was used. The choice of predictor values that give the highest mean accuracy is chosen as optimal for the problem. This allows the proposed scoring mechanism to have high accuracy.For comparison purposes, on test dataset the original Kobayashi values performed as follows: On test dataset: Sensitivity= 0.66, Specificity=0.5 On full dataset: Sensitivity=0.75 Specificity=0.56

**Results:** The above analysis was done for all 4 of the proposed scoring mechanisms; the Kobayashi based approach produced best results. Using the same variables used in Kobayashi, and running an analysis on the training dataset, we came up with the following new values of the variables:

**Sodium=133, fever=4 days, AST=100, Neutrophils=84%, CRP=17 mg/dl, age=21months, platelet=6 lacs.**

The signs are what was used in the original Kobayashi study for each of the variables (eg. for Sodium it was <=). We had the following results on datasets: On test dataset: Sensitivity=0.8 , Specificity=0.43 On full dataset: Sensitivity= 0.9, Specificity=0.52

**Conclusion:** The proposed values can be seen to have better sensitivity than the original values on the dataset, and thus will act as a better guide for screening patients for possible IVIG resistance.

**Patient Consent**

Yes, I received consent

**Disclosure of Interest**

None declared

## PT094 Levels of soluble receptors for tumor necrosis factor types i and ii in the blood of patients with systemic juvenile arthritis and monogenic autoinflammatory diseases (FMF, CAPS, TRAPS)

### E. Fedorov^1^, S. Salugina^1^, M. Cherkasova^2^

#### ^1^Pediatric; ^2^Immunology and Molecular Biology of Rheumatic Diseases, V.A.Nasonova Research Institute of Rheumatology, Moscow, Russian Federation

##### **Correspondence:** E. Fedorov

*Pediatric Rheumatology 2023*, **21(Suppl 2):**PT094

**Introduction:** The leading component in the pathogenesis of autoinflammatory diseases is, which includes systemic juvenile arthritis (sJA), an increase in interleukin 1ß.At the same time, cytokines form an interdependent network and an increase in one of the pro-inflammatory cytokines leads to hyperproduction of other pro-inflammatory cytokines. One of the important cytokines is the tumor necrosis factor(TNF). The determination of the levels of its soluble receptors in the blood allows us to indirectly assess the overexpression of TNF.

**Objectives:** to evaluate the levels of sTNF-RI and sTNF-RII in the blood sera of patients with sJA and monogenic autoinflammatory diseses(mAIDs) and their correlation with serum levels of commonly accepted markers of inflammation (C-reactive protein, Serum Amyloid A, Ferritin)

**Methods:** 114 patients were included in the study, 43 of them with sJA(38%): male/female 20/23; age of inclusion in the study 3-19 years, FMF 33 (29%) patients; male/female 15/18; age 3-37 years, CAPS 23(20%)patients; male/female 15/8; age 1-51 years,, TRAPS 15(13%)patients; male/female 8/7, age 4-51 years. The control group consisted of 5 children and adolescents with orthopedic pathology (scoliosis, pes planus) The diagnosis in all patients with mAIDs was confirmed by the detection of pathogenic alleles of the corresponding genes. sTNF-RI and sTNF-RII were determined in blood serum by ELISA using Invitrogen kits (Bender MedSystems GmbH, Austria): Normal values of sTNF-RI 1.47-4.16 ng/ml, sTNF-RII 3.4-10.8 ng/ml. CRP and SAA were determined by nephelometric method using Siemens reagents on the BN ProSpec Siemens analyzer, Germany. Ferritin was determined by the ELISA method using kits manufactured by Orgentec Diagnostika GmbH, Germany. Statistical processing was performed using the SPSS program. The reliability of the differences between the groups was assessed by the Kruskal-Wallis test. Correlation was estimated by Spearman Rank Order Correlations.

**Results:** The levels of sTNF-RI Me[Q1; Q3] in patients with sJA were 2.43ng/ml [1.95; 3.43], in patients with FMF 2.47 ng/ml [1.89; 2.71], CAPS - 2.24ng/ml [1.84; 2.93], TRAPS - 1.6 ng/ml [1.06; 3.11]. In the control group 1.63 ng/ml [1.38; 1.65]. According to the Kruskal-Wallis criterion, the differences in values between here and the control group (p=0,04), TRAPS and sJA (p=0,01) were statistically significant.

The levels of sTNF-RII Me[Q1, Q3] in patients with sJA were 7.15 ng/ml [3,28; 23,2], in patients with FMF 28,82 ng/ml [3,98; 82,2], CAPS - 8.55 ng/ml [4.32, 110.0], TRAPS - 26.81 ng/ml [8.72, 110.0]. In the control group 0,577ng/ml [0,46; 1,05]. According to the Kruskal-Wallis criterion, the differences in values between CAPS and the control group (p=0,04), TRAPS and the control group (p=0.004), FMF and the control group (p=0,007) were statistically significant.

In patients with sJA, there was a statistically significant (p<0.05) correlation of the level of sTNF-RI with CRP (r=0.73), with SA (r=0.64) and ferritin (r=0.66). In patients with CAPS, there was a statistically significant (p<0.05) correlation of the level of sTNF-RI with CRP(r=0.52), with SATA (r=0.51) and ferritin (0.45). A statistically significant negative correlation (r=- 0.8) was found between the level of sTNF-RII and CRP in patients with TRAPS.

**Conclusion:** the maximum concentrations of sTNF-RI were found in patients with sJA and FMF, the minimum in patients with TRAPS. The maximum serum concentrations of sTNF-RII were detected in patients with FMF and TRAPS, the minimum in patients with sJA. Statistically significant positive correlations of sTNF-RI concentration with generally accepted laboratory markers of inflammatory activity (CRP, SAA, ferritin) were found in patients with sJA and CAPS. In patients with TRAPS, a significant negative correlation was found between the concentrations of sTNF-RII and CRP. These data may indicate a different role of TNF in different AIDs

**Patient Consent**

Not applicable (there are no patient data)

**Disclosure of Interest**

None declared

## PT095 Systemic Juvenile Idiopathic Arthritis (SJIA): the great ormond street hospital experience (2005-2021)

### C. Foley, D. McKenna, K. Gallagher, K. McLellan, H. Alkhdher, M. Al Obaidi, D. Eleftheriou, S. Lacassagne, E. Moraitis, C. Papadopoulou, C. Pilkington, P. Brogan

#### Great Ormond Street Hospital, London, United Kingdom

##### **Correspondence:** C. Foley

*Pediatric Rheumatology 2023*, **21(Suppl 2):**PT095

**Introduction:** Systemic juvenile idiopathic arthritis (sJIA) is a complex, systemic inflammatory disorder, driven by both innate and adaptive immunity. Improved understanding of sJIA pathophysiology has led to recent therapeutic advances including a growing evidence base for the earlier use of IL-1 or IL-6 blockade as first line treatment.

**Objectives:** To describe the clinical presentation, therapeutic interventions, complications, and remission rates at different timepoints over the disease course of patients with sJIA.

To identify potential therapeutic signals in patients who received biologic treatment early in the disease course compared to those who did not.

**Methods:** We used electronic institutional clinical record coding to identify all patients with a diagnosis of sJIA seen at GOSH over a 16-year period. Using the search terms sJIA, Still’s disease, systemic onset JCR (juvenile chronic arthritis) and AOSD, we identified patients diagnosed with sJIA, between 2^nd^ October 2005 – 21^st^ October 2021 inclusive. Medical notes were reviewed retrospectively. All patients with a final diagnosis of sJIA made by a consultant paediatric rheumatologist were included.

Patient demographics including age at diagnosis and sex were recorded. Symptoms and signs present at diagnosis informing the ILAR sJIA classification criteria were documented. Data on the active joint count, presence of early morning stiffness, presence of uveitis as defined by the standardisation of uveitis nomenclature working group, physician’s global assessment (PGA) of overall disease activity on a visual analogue scale 0 – 100 mm (0 = no activity; 100mm = maximum activity), erythrocyte sedimentation rate (ESR, mm/hour) and C-reactive protein (CRP, mg/litre) were also captured.

Time-points for data collection were: at diagnosis, 3-months post diagnosis, 1-year post diagnosis and last review. Clinical and laboratory data as outlined above were recorded for each time-point.

Clinically inactive disease (CID) was defined using the modified Wallace Criteria

Complications of particular interest were captured. These were frequency of macrophage activation syndrome (MAS), sJIA-associated lung disease (sJIA-LD), requirement for haematopoietic stem cell transplantation (HSCT), and deaths.

Descriptive statistics were reported as median and range or inter-quartile range (IQR) for continuous variables, and as absolute frequencies and percentages for categorical variables unless otherwise specified. Comparisons of quantitative variables between two groups were made by Mann-Whitney U test. Categorical data were compared using Fisher’s exact test. All statistical tests were 2-tailed; p values <0.05 were considered significant.

**Results:** A total of 76-children (female n=40, 53%) were diagnosed with sJIA, median age 4.5 years (range 0.6–14.1); 36% (27/76) presented with suspected or confirmed MAS. A biologic disease modifying anti-rheumatic drug (bDMARD) alone was commenced as first-line treatment in 28% (n=21/76) of the cohort; however, at last review, 84% (n=64/76) had received treatment with a bDMARD. Clinically inactive disease (CID) was achieved by 88% (n=67/76) of the cohort at last review, however only 32% (24/76) achieved treatment-free CID. At 1-year follow-up, CID was achieved in a significantly greater proportion of children who received treatment with a bDMARD within 3-months of diagnosis compared to those who did not (90% versus 53%, p=0.002).

**Conclusion:** Based on an ever-increasing evidence base for the earlier use of bDMARD in sJIA and our experience of the largest UK single centre case series described to date, we now propose a new therapeutic pathway for children diagnosed with sJIA in the UK based on early use of bDMARD. Reappraisal of the current National Health Service commissioning pathway for sJIA is now urgently required.

**Patient Consent**

Not applicable (there are no patient data)

**Disclosure of Interest**

None declared

## PT096 Association of Glutathione S-Transferase (GST) gene polymorphisms with clinical features of IGA vasculitis

### M. Held^1^, A. Juras^2^, M. Sestan^1^, M. Sapina^3^, N. Kifer^1^, S. Srsen^4^, S. Huljev Frkovic^1^, M. Frkovic^1^, A. Gagro^5^, K. Crkvenac Gornik^2^, M. Jelusic^1^

#### ^1^Department of Pediatrics, University Hospital Centre Zagreb; ^2^Department of laboratory diagnostics, University Hospital Centre Zagreb, University of Zagreb School of Medicine, Zagreb; ^3^Department of Pediatrics, University Hospital Centre Osijek, Josip Juraj Strossmayer University of Osijek, Faculty of Medicine, Osijek; ^4^Department of Pediatrics, University Hospital Centre Split, University of Split School of Medicine, Split; ^5^Children's Hospital Zagreb, Josip Juraj Strossmayer University of Osijek, Faculty of Medicine, Zagreb, Croatia

##### **Correspondence:** M. Held

*Pediatric Rheumatology 2023*, **21(Suppl 2):**PT096

**Introduction:** Non-HLA gene variants may play an important role in the pathogenesis of IgA vasculitis (IgAV). Glutathione S-transferases (GSTs) are metabolic enzymes involved in cellular detoxification processes of potentially toxic and carcinogenic compounds. Deletions in GST reduce detoxification activity, and thus increase susceptibility to various diseases.

**Objectives:** To investigate the influence of gene polymorphisms *GSTA1, GSTM1, GSTP1 and GSTT1* on IgAV susceptibility and clinical heterogeneity of the disease.

**Methods:** Clinical data were collected from database of IgAV patients from three Croatian centers for pediatric rheumatology. The Flexigene DNA set (Qiagen) was used to isolate DNA from whole blood. The presence of GSTM1 and GSTT1 polymorphisms was determined by polymerase chain reaction in patients and controls, while GSTA1 and GSTP1 genotyping was performed using the PCR-RFLP method (eng. Polymerase Chain Reaction – Restriction Fragment Length Polymorphism).

**Results:** The study enrolled 124 IgAV patients (67 girls and 57 boys) with median age at the time of diagnosis 6.3 (4.3-8.2) years and 168 age- and sex-matched controls without any history of autoimmune diseases. All patients had purpuric rash, 85.5% had joint involvement, 36.3% had gastrointestinal (GI) manifestations, while 29.8% developed nephritis. A statistically significant difference between patients and controls was observed in genotypes GSTP1 Ile/Ile (48.33% vs. 22.62%, p<0,001) and Leu/Ile (4.17% vs. 28.57%, p<0,001). A higher frequency of null GSTM1 genotype was observed in IgAV patients with GI system involvement in comparison to patients without GI system involvement (52.2% vs. 28.6%, p=0.014). The GSTP1 Val/Val genotype appeared significantly more often in patients who developed urological complications (acute scrotum) within disease course (60% vs. 40%, p=0,037).

**Conclusion:** The studied GSTP1 polymorphisms showed a possible association with a higher individual susceptibility to IgAV. GSTM1 genotype variants seem to be involved in the pathogenesis of GI manifestations of disease.

SUPPORT: Croatian Science Foundation Project IP-2019-04-8822.

**Patient Consent**

Yes, I received consent

**Disclosure of Interest**

None declared

## PT097 Pediatric takayasu arteritis: a multicenter retrospective cohort study

### M. Kasap Cuceoglu^1^, E. D. Batu^1^, P. N. Akpınar Tekgöz^2^, E. Arslanoglu Aydın^3^, C. Arslanoglu^4^, R. M. Kısla Ekinci^5^, H. Kose^6^, S. S. Kılıç^7^, A. Pac Kısaarslan^4^, S. Özdel^3^, B. Çelikel Acar^2^, S. Ozen^1^

#### ^1^Pediatric Rheumatology, Hacettepe University; ^2^Pediatric Rheumatology, Ankara Bilkent City Hospital; ^3^Pediatric Rheumatology, Ankara Etlik City Hospital, Ankara; ^4^Pediatric Rheumatology, Erciyes University, Kayseri; ^5^Pediatric Rheumatology, Adana City Training & Research Hospital, Adana; ^6^Pediatric Rheumatology; ^7^Pediatric Immunology and Rheumatology, Uludag University, Bursa, Türkiye

##### **Correspondence:** M. Kasap Cuceoglu

*Pediatric Rheumatology 2023*, **21(Suppl 2):**PT097

**Introduction:** Takayasu arteritis (TA) is a chronic, inflammatory, granulomatous vasculitis that commonly affects the aorta and its major branches.^1^ Diagnosis is difficult due to nonspecific symptoms at onset. Effective treatment is essential due to the high morbidity and mortality rates in follow-up.

**Objectives:** We aimed to identify demographic, clinical characteristics, and outcomes of pediatric TAK in a national cohort.

**Methods:** We conducted a multicenter retrospective cohort study in Turkey. The clinical data were collected from patients’ charts in six rheumatology centers. Patients who were diagnosed with TAK before 18 years of age were included in the study. All patients met the Ankara 2008 classification criteria.^2^ ITAS 2010 score was used to evaluate disease activity.^3^

**Results:** Overall, 43 pediatric TAK (p-TAK) patients were included (86% female). The median age of symptom onset was 13.2 (1-14.4) years, with a diagnostic delay of 5 (1-57) months, and a median follow-up time of 42 (6-146) months. The most common symptoms at presentation were neck, back, or abdominal pain (n=17,39.5%), fever (n=11,25.5%), and hypertension (n=7,16.2%). 39 patients had elevated acute phase reactants. The most common angiographic type at diagnosis was type IIa (13/43) and the least frequent types were IV (n=4) and IIb (n=1). Median ITAS2010 was 12 (6-18) and 3 (0-12) at admission and at last control, respectively. At the last visit, ITAS 2010 was <3 in 13 patients.Treatment included corticosteroids (n=43,100%), conventional (n=28,65.1%) and biological disease-modifying anti-rheumatic drugs (n=35, 81.5%), and other immunosuppressive therapies (cyclophosphamide (n=19,44.1%)). The median duration of corticosteroid use was 31 (6-96) months. Antihypertensive and anticoagulant drugs were used by 27 and 23 patients, respectively.There was a switch between biologic drugs in 17 patients. The first biologic drugs were tocilizumab (n=16), adalimumab (n=10), and infliximab (n=7), and the median time for the first biological drug use was 18 (2-70) months. The most frequent second biologic drugs were tocilizumab (n=8) and adalimumab (n=6). The median duration of use was 24 (1-72) months. Surgical procedures were required in nine patients with severe disease refractory to medications. In follow-up angiographic imaging, there was deterioration in 14 patients, improvement in 7, and stable findings in 21 patients. 24 (55.8) patients had refractory disease. Only, 4 patients had drug-free remission. Two patients died.

**Conclusion:** Despite aggressive immunosuppressive therapy and use of biologic agents, complete disease control was achieved in a small portion of p-TAK patients.

**Patient Consent**

Yes, I received consent

**References**

1. Jennette JC, et al. 2012 revised International Chapel Hill Consensus Conference Nomenclature of Vasculitides. *Arthritis Rheum*. Jan 2013;65(1):1-11.
2. Ozen S. et. 2010. EULAR/PRINTO/PRES criteria for Henoch-Schonlein purpura, childhood polyarteritis nodosa, childhood Wegener granulomatosis and childhood Takayasu arteritis: Ankara 2008. Part II: Final classification criteria. *Ann Rheum Dis*.;69(5):798-806.
3. Misra R. et al. 2013. Development and initial validation of the Indian Takayasu Clinical Activity Score (ITAS2010). Rheumatology (Oxford).52(10):1795-801.

## PT098 Validation of the pediatric behçet’s disease classification criteria

### C. Matucci-Cerinic^1,2^, H. Palluy^3^, S. Al-Mayouf ^4^, P. Brogan^5^, L. Cantarini^6^, A. Gul^7^, O. Kasapcopur^8^, J. Kuemmerle-Deschner ^9^, S. Ozen^10^, D. Saadoun^11^, F. Shahram^12^, F. Bovis^13^, E. Mosci^14^, N. Ruperto^14^, M. Gattorno^2^, I. Kone-Paut^3^ on behalf of Eurofever Registry and PRINTO network

#### ^1^DINOGMI, University of Genoa; ^2^UOC Rheumatology and Autoinflammatory Diseases, IRCCS Istituto Giannina Gaslini, Genoa, Italy; ^3^Pediatric rheumatology and CEREMAIA, Bicêtre Hospital, APHP, University Paris Saclay, Paris, France; ^4^Department of Pediatrics, King Faisal Specialist Hospital and Research Center College of Medicine, Alfaisal University, Riyadh, Saudi Arabia; ^5^University College London Great Ormond Inst of Child Health, and Great Ormond Street Hospital NHS , London, United Kingdom; ^6^Rheumatology Unit, Department of Medical Sciences, Surgery and Neurosciences,, University of Siena, Siena, Italy; ^7^Division of Rheumatology, Department of Internal Medicine, Istanbul Faculty of Medicine, Istanbul University; ^8^Cerrahpasa Medical School, Istanbul University-Cerrahpasa Turkey, Istanbul, Türkiye; ^9^Division of Pediatric Rheumatology, Department of Pediatrics and Autoinflammation Reference Center, University Hospital Tuebingen, Tuebingen, Germany; ^10^Department of Pediatric Rheumatology, Hacettepe University, Ankara, Türkiye; ^11^Department of Internal Medicine and Clinical Immunology, CEREMAIA, Sorbonne Universités, APHP Groupe Hospitalier Pitié-Salpêtrière, Paris, France, ^12^Rheumatology research center, Shariati Hospital, Tehran Univ of Medical Sciences, Tehran, Iran, Islamic Republic Of; ^13^Dept of Health Sciences (DISSAL), University of Genoa; ^14^UOC Servizio di Sperimentazioni Cliniche Pediatriche, PRINTO, IRCCS Istituto Giannina Gaslini, Genoa, Italy

##### **Correspondence:** C. Matucci-Cerinic

*Pediatric Rheumatology 2023*, **21(Suppl 2):**PT098

**Introduction:** Behçet’s disease (BD) is an autoinflammatory disease characterized by a variable vessel vasculitis. In the past, several criteria have been created for adult BD classification. In 2015, the first PEDiatric Behçet’s Disease classification criteria, the PEDBD, were proposed by an international Expert consensus (1).

**Objectives:** to perform a validation of the PEDBD classification criteria in a cohort of internationally validated pediatric BD, through an international Expert-based consensus process.

**Methods:** 210 patients (70BD, 40 PFAPA, 35 FMF, 26 MKD, 22 TRAPS, 17 Undefined/SURF) were randomly selected from the Eurofever Registry. A set of 11 Experts evaluated the patients to assign a diagnosis: in the 1^st^ round, clinical and serological data were evaluated; in the 2^nd^ round genetic data were added; in the 3^rd^ round the other Experts’ votes and comments were shown. Using the expert consensus as gold standard (agreement>80%), the PEDBD, the ISG and the ICBD criteria were then applied to BD patients and to the confounding diseases in order to define their sensitivity, specificity and accuracy.

**Results:** An Expert agreement was found for 66.2% of patients. BD patients with an agreement (24) were considered as “confirmed-BD”, and those with a partial agreement (60-70%) as probable-BD (10). When comparing confirmed-BD patients with the confounding diseases, the presence of an older age at disease onset, oral ulcers (100%), genital ulcers (77%), skin manifestations (50%), a positive pathergy reaction (39%), posterior uveitis (27%), cranic nerve palsy (17%), retinal vasculitis and papillary oedema (8%), venous thrombosis (8%) and anal/perianal ulcers (8%) resulted BD distinctive elements. Fever was present in 50% of patients. HLA-B51 was positive in 69% of patients.

The ISG, ICBD and PEDBD criteria were applied to confirmed and probable-BD, and to the confounding disease group, showing a sensitivity of 0.50, 0.79 and 0.58, a specificity of 1.00, 0.97, 0.99, and an accuracy of 0.91, 0.94 and 0.92, respectively.

**Conclusion:** the PEDBD were extremely specific in classifying BD patients, while the ICBD had a better sensitivity, especially for patients with only bipolar aphtosis. One limitation is that specific monogenetic BS mimics were not included as disease controls, thus the true accuracy of all these criteria may be lower in practice. The complexity of childhood BD suggests larger prospective international cohorts to foster the performance of the criteria, and to understand if BD clusters and ethnic variables should be added to the criteria.

**Patient Consent**

Yes, I received consent

**Disclosure of Interest**

None declared

**Reference**

1. Koné-Paut I et al. Consensus classification criteria for paediatric Behçet's disease from a prospective observational cohort: PEDBD. *Ann Rheum Dis.* (2016) 75:958–64.

## PT099 Calculating the fraction of kawasaki disease potentially attributable to seasonal pathogens: a time series analysis

### Z. Valtuille^1^, A. Lefevre-Utile^2^, N. Ouldali^3,4^, C. Beyler^4^, P. Boizeau^5^, C. Dumaine^4,6^, A. Felix^4^, Z. Assad^4^, A. Faye^2^, I. Melki^4,6^, F. Kaguelidou^4^, U. Meinzer^4,6,7^

#### ^1^Hôpital Robert Debré, APHP; ^2^Université Paris-Cité, APHP; ^3^Université Paris Cité; ^4^Hôpital Robert-Debré, APHP; ^5^Robert-Debré University Hospital, Assistance Publique-Hôpitaux de Paris; ^6^National Reference Centre for Rare Pediatric Inflammatory Rheumatisms and Systemic Autoimmune diseases (RAISE); ^7^Université Paris-Cité, Paris, France

##### **Correspondence:** U. Meinzer

*Pediatric Rheumatology 2023*, **21(Suppl 2):**PT099

**Introduction:** Kawasaki disease (KD) is an acute, febrile, systemic vasculitis of children that primarily affects medium-sized blood vessels with a tropism for the coronary arteries. Although the etiological factors remain unknown, infections have been suggested as the trigger of KD. Using correlation analysis, a recent study showed that in South Korea, outbreaks of several viral infections precede KD outbreaks. Studies on the temporal association between seasonal infections and KD in the epidemiological context outside of Asia are currently lacking, and data to assess the fraction of KD potentially attributable to seasonal infections are not available.

**Objectives:** We sought to calculate the fraction of KD potentially attributable to seasonal infections.

**Methods:** This cohort study used a population-based time series analysis from the French hospitalization database. We included all children aged 0 to 17 years hospitalized for KD in France over 13 years. The monthly incidence of KD per 10,000 children over time was analyzed by a quasi-Poisson regression model. The circulation of eight common seasonal pathogens over the same period was included in the model to analyze the fraction of KD potentially attributable to each pathogen.

**Results:** From January 1, 2007, to December 31, 2019, we included 10,337 children with KD and 442,762 children with the selected infectious diseases. In the KD cohort, the median age [IQR] was 2 [0-4] years, 6164 [59.6%] were boys. Adenovirus infection was potentially responsible for 24.4% [21.5-27.8] (p<0.001) of KD, Norovirus for 6.7% [1.3-11.2] (p=0.002), and RSV 4.6% [1.2-7.8] (p=0.022). Sensitivity analyses found similar results. Subgroup analyses revealed that these significant associations prevailed among children younger than 5 years.

**Conclusion:** This cohort study of data from a comprehensive national hospitalization database indicated that approximately 35% of KD was potentially attributable to seasonal infections.

**Patient Consent**

Yes, I received consent

**Disclosure of Interest**

None declared

## PT100 Predictive factors of long-lasting remission following anakinra discontinuation in patients with systemic juvenile idiopathic arthritis after achievement of clinical inactive disease

### G. Nardini^1^, D. Pires Marafon^1^, C. Bracaglia^1^, E. Sacco^1^, A. De Matteis^1^, I. Caiello^2^, G. Prencipe^2^, F. De Benedetti^1^, M. Pardeo^1^

#### ^1^Division of Rheumatology; ^2^Laboratory of Immuno-Rheumatology, Bambino Gesù Children's Hospital, IRCSS, Rome, Italy

##### **Correspondence:** G. Nardini

*Pediatric Rheumatology 2023*, **21(Suppl 2):**PT100

**Introduct**i**on:** Systemic juvenile idiopathic arthritis (sJIA) is a rare inflammatory disease of unknown etiology. Several uncontrolled studies showed that early treatment with anakinra is associated with a better outcome, according to the “window of opportunity” hypothesis. However, very limited scientific evidence is available on withdrawal strategy. So far, anakinra withdrawal modalities are heterogeneous among the different rheumatology centres.

**Objectives:** The aim of this study was to identify predictive factors of disease flare, as suggestive of persistent course of the disease, after anakinra discontinuation, in patients with systemic juvenile idiopathic arthritis (sJIA) who reached clinical inactive disease (CID) off glucocorticoids (GCs).

**Methods:** We retrospectively analyzed data of 39 consecutive sJIA patients followed in our center who withdrew anakinra after achieving CID off GCs for at least 6 months. All patients underwent a 24-month follow-up after discontinuation. They were subsequently divided into two groups based to the presence or the absence of disease flare during the follow-up. Demographic, clinical and laboratory data of the patients were evaluated in univariate and multivariate analysis as predictors of flare.

**Results:** Ten out of 39 patients (25.6%) flared after a median time of 7.9 months from anakinra discontinuation. In univariate analysis, disease duration ≥ 3 months at anakinra initiation (p=0.001), anakinra dose < 2 mg/kg/day (p=0.065) and abrupt withdrawal instead of tapering of anakinra (p=0.016) were associated with occurrence of flare after discontinuation. In the multivariate analysis, disease duration ≥ 3 months at baseline was the only variable significantly associated with flare after anakinra discontinuation (Odds Ratio 15.16, CI 95% 1.7-131.9; p = 0.014).

**Conclusion:** Our data show that early treatment with anakinra (3 months) is associated with lower risk of flare following discontinuation suggesting that early treatment may prevent development of a chronic persistent sJIA course.

**Patient Consent**

Not applicable (there are no patient data)

**Disclosure of Interest**

G. Nardini: None declared, D. Pires Marafon: None declared, C. Bracaglia Consultant with: Sobi, Novartis, E. Sacco: None declared, A. De Matteis: None declared, I. Caiello: None declared, G. Prencipe: None declared, F. De Benedetti Grant / Research Support with: Sobi, Abbvie, Novimmune, Novartis, Roche, Sanofi, Pfizer, Consultant with: Sobi, Novartis, M. Pardeo Consultant with: Sobi

**References**

1. Nigrovic PA. Review: is there a window of opportunity for treatment of systemic juvenile idiopathic arthritis? Arthritis Rheumatol 2014; 66:1405-13.
2. Pardeo M, Rossi MN, Pires Marafon D, Sacco E, Bracaglia C, Passarelli C, et al. Early Treatment and IL1RN Single-Nucleotide Polymorphisms Affect Response to Anakinra in Systemic Juvenile Idiopathic Arthritis. Arthritis Rheumatol 2021; 73:1053-1061.

## PT101 Comparison of physical activity level and motivation for physical activity participation of patients with Juvenile idiopathic arthritis with their healthy peers

### A. Albayrak^1,2^, N. Arman^3^, A. Yekdaneh^1,4^, O. Akgun^5^, N. Aktay Ayaz^5^

#### ^1^Institute of Graduate Studies, Physiotherapy and Rehabilitation Doctorate Program, Istanbul University-Cerrahpasa; ^2^Faculty of Health Sciences, Department of Physiotherapy and Rehabilitation, Istanbul Kent University; ^3^Faculty of Health Sciences, Department of Physiotherapy and Rehabilitation, Istanbul University-Cerrahpasa; ^4^Vocational School of Health Services, Physiotherapy English Program, Fenerbahce University; ^5^Department of Pediatrics, Department of Pediatric Rheumatology, Istanbul University, Istanbul Faculty of Medicine, Istanbul, Türkiye

##### **Correspondence:** A. Albayrak

*Pediatric Rheumatology 2023*, **21(Suppl 2):**PT101

**Introduction:** Juvenile Idiopathic Arthritis (JIA) is the most common childhood rheumatic disease and is characterized by joint swelling, pain, stiffness, muscle weakness and muscle atrophy. In children and adolescents with JIA, participation in physical activity plays an important role in managing functional, structural, and activity-related limitations caused by the disease (1). Despite this, it has been reported that patients with JIA cannot be able to reach the recommended level of physical activity, and lack of motivation is thought to be one of the main obstacles to participation in physical activity (2,3).

**Objectives:** The aim of this study was to compare the physical activity level and motivation for physical activity participation of JIA patients with their healthy peers.

**Methods:** Twenty-two patients (11 girls, 11 boys) diagnosed with JIA and 21 healthy peers (14 girls, 7 boys) aged 11-18 years were included in the study. The physical activity level of children and adolescents was evaluated with the Physical Activity Question Form and Physical Activity Questionnaire for Adolescents, and the motivation to participate in physical activity was evaluated with the Physical Activity Motivation Scale. SPSS Version 24.0 program was used for statistical analysis.

**Results:** The mean ages of patients with JIA and their healthy peers were 14.32±2.16 and 13.90±0.76 years, respectively. In the comparison between the groups, In patients with JIA, the physical activity level and motivation to participate in physical activity were statistically significantly lower compared to their healthy peers (p<0.05). In addition, 81.8% of the patients with JIA had "insufficient" physical activity levels.

**Conclusion:** Compared to their healthy peers, patients with JIA were found to have lower physical activity levels and motivation to physical activity participation. In patients with JIA, we believe that when evaluating participation in physical activity, motivational factors should be evaluated comprehensively and motivational facilitators should be taken into account when planning a physical activity program.

This study was supported within the scope of the Scientific and Technological Research Council of Turkey (TUBITAK) 1001-Scientific and Technological Research Projects Support Program (Project number: 121E690).

**Patient Consent**

Yes, I received consent

**Disclosure of Interest**

None declared

**References**

1. Kuntze, G., Nesbitt, C., Whittaker, J. L., Nettel-Aguirre, A., Toomey, C., Esau, S. et al. Exercise therapy in juvenile idiopathic arthritis: a systematic review and meta-analysis. Archives of Physical Medicine and Rehabilitation, 2018;99(1):178-193.
2. Lelieveld, O. T., Armbrust, W., Van Leeuwen, M. A., Duppen, N., Geertzen, J. H., Sauer, P. J. et al. Physical activity in adolescents with juvenile idiopathic arthritis. Arthritis Care & Research: Official Journal of the American College of Rheumatology, 2008;59(10):1379-1384.
3. Nørgaard, M., & Herlin, T. Specific sports habits, Leisure-Time physical activity, and School-Educational physical activity in children with juvenile idiopathic arthritis: Patterns and barriers. Arthritis Care & Research, 2019;71(2):271-280.

## PT102 Juvenile idiopathic arthritis: transition from pediatric to adult rheumatology

### S. A. Germe^1^, Z. Balik^2^, Z. Ozsoy^1^, Y. E. Dalkilic^3^, L. Kilic^1^, E. D. Batu^2^, O. Basaran^2^, Y. Bilginer^2^, S. Apras Bilgen^1^, S. Ozen^2^

#### ^1^Division of Rheumatology, Department of Internal Medicine; ^2^Division of Rheumatology, Department of Pediatrics; ^3^Department of Internal Medicine, Hacettepe University, Ankara, Türkiye

##### **Correspondence:** Z. Balik

*Pediatric Rheumatology 2023*, **21(Suppl 2):**PT102

**Introduction:** Juvenile idiopathic arthritis (JIA) is the most common chronic idiopathic inflammatory arthritis of childhood, and it often persists in adulthood. Disease exacerbations, joint damage, and extra-articular manifestations may lead to morbidities. A well-prepared transition to adult rheumatology is crucial to improve the ongoing care and well-being of patients.

**Objectives:** The aim of this study is to review the clinical findings, treatments, and follow-up processes of JIA patients who were transferred to adult rheumatology.

**Methods:** The medical records of the JIA patients transferred from the Department of Pediatric Rheumatology to the Department of Adult Rheumatology between January 2015 and May 2022 were retrospectively reviewed.

**Results:** A total of 107 patients (45 girls, 62 boys) were included in the study (Table 1). The most common diagnosis was enthesitis-related arthritis, followed by oligoarticular JIA. Comorbidity was present in 38 (35.5%) patients. The most common comorbidity was familial Mediterranean fever (n=17, 15.9%) followed by inflammatory bowel disease (n=3, 2.8%) and scoliosis (n=3, 2.8%). One hundred (93.5%) patients had peripheral and 60 (56.1%) patients had axial symptoms. Twenty-eight (26%) patients had extra-articular findings. In adult rheumatology, 46 (43%) patients were followed without any complaints. Thirty-four (31.8%) patients had axial and 39 (36.4%) patients had peripheral symptoms. Extra-articular findings were observed in 10 (9.3%) patients. In the pediatric rheumatology department, all patients received non-steroidal anti-inflammatory drugs (NSAIDs) as first-line therapy (Table 2). The most frequently used disease-modifying anti-rheumatic drug (DMARD) was methotrexate. Biological drugs were given to 72 (67.3%) patients. During the transfer, 53 (49.5%) patients were on biological drugs, 9 (8.4%) conventional DMARD, and two (1.9%) patients were on both. Forty-three (40.2%) patients were transferred without medication. In adult rheumatology, 31 (29.9%) patients were followed without medication. NSAIDs were used in 40 (37.4%) patients. The most frequently used DMARD was methotrexate. Biological drugs were prescribed to 57 (53.3%) patients. In adult rheumatology, 27 patients who were transferred without taking any medication did not require it. But 16 of them were needed for drugs. Seven of 16 patients required biologics. In the last appointment, 55 (51.4%) patients were using biological drugs.

**Conclusion:** In adult rheumatology, more than half of JIA patients had recurrent complaints. And at the last control, half of the transferred patients still required biological drugs. Thus, an uninterrupted follow-up is vital for the JIA patients transferred to adult rheumatology. Knowing the distinct phenotypes and managing the transition without interruption will contribute to the improvement of JIA prognosis.

**Patient Consent**

Yes, I received consent

**Disclosure of Interest**

None declared

**Reference**

1. Debrach AC, Rougelot A, Beaumel A, Cabrera N, Belot A, Duquesne A, et al. Comparison of paediatric and adult classification criteria in juvenile idiopathic arthritis during the transition from paediatric to adult care. Joint Bone Spine. 2021;88(1):105047.

## PT103 Awareness of childhood arthritis: results from a representative online survey in the UK

### R. P. Beesley^1,2^, R. M. Beesley^1^

#### ^1^Juvenile Arthritis Research, Tonbridge, United Kingdom; ^2^ENCA, Geneva, Switzerland

##### **Correspondence:** R. P. Beesley

*Pediatric Rheumatology 2023*, **21(Suppl 2):**PT103

**Introduction:** Low awareness that Children and Young People (CYP) may develop arthritis (Juvenile Idiopathic Arthritis, JIA) has been identified as a risk factor for delayed diagnosis and worse clinical outcomes. The level of community awareness in the UK has not previously been reported in scientific literature.

**Objectives:** To measure awareness that CYP may develop arthritis, providing a baseline against which to monitor improvements.

**Methods:** Ipsos UK, on behalf of UK charity Juvenile Arthritis Research, conducted an online survey about awareness of childhood arthritis among a representative quota sample of adults aged 16-75. Fieldwork was between 10-13 February 2023. Data were weighted to the known population proportions for adults aged 16-75 in the UK. This work was carried out in accordance with the requirements of the international quality standard for market research, the MRS Code of conduct, ISO 20252, and participants gave informed consent prior to taking part.

**Results:** A total of 2044 adults aged 16-75 in the UK completed the survey online. When asked about the earliest age ranges someone could get arthritis, overall 40% indicated age ranges under the age of 16. This was higher amongst female respondents (47% vs 33% of males), older respondents (52% of those aged 45-75 vs 29% of those aged 16-44), and those from a White ethnic group (43%, compared to 23% among ethnic minority groups). Respondents were more likely to be aware the earliest someone could get arthritis is under the age of 16 if they had arthritis themselves (60%) or knew someone with arthritis (43%). However, only 19% of respondents were aware that children under the age of 5 can get arthritis.

Overall, 29% of respondents were aware that some types of arthritis can affect your eyesight; awareness of this extra-articular complication was also higher amongst those with arthritis (34%).

A total of 18% of respondents reported having arthritis themselves, and 55% claimed to know an adult with arthritis. In contrast, only 3% claimed to know of a child aged 15 or under with arthritis, although this was higher amongst parents of children aged 17 or under living in their household (7%).

Significant differences between ethnic groups were identified, with respondents from ethnic minority groups less likely to be aware the earliest that someone could get arthritis is under the age of 16 (23%, compared to 43% of those from White ethnic groups). Respondents from ethnic minority groups were also more likely to believe incorrect assumptions about arthritis to be true – such as ‘arthritis can be cured’ (25% compared to 6% of those from White ethnic groups), ‘blood tests can always confirm a diagnosis of arthritis (38% compared to 24% of those from white ethnic groups), and ‘X-rays can always confirm a diagnosis of arthritis’ (44% compared to 30% of those from white ethnic groups).

**Conclusion:** Awareness that children and young people under the age of 16 can get arthritis is low amongst the general population. Low awareness can lead to delays in diagnosis. Increasing awareness is therefore vital, and the #ThinkJIA campaign, school-based toolkits, and community-led initiatives developed by Juvenile Arthritis Research can be used to increase awareness of JIA. Targeted resources to increase awareness amongst ethnic minority groups should be considered. Ongoing review and monitoring, using these 2023 data as a baseline will be important to assess the efficacy of future awareness activities.

**Patient Consent**

Not applicable (there are no patient data)

**Disclosure of Interest**

None declared

## PT104 The experience of juvenile idiopathic arthritis in family life

### M. M. Delliou^1,2^, F. Galani^1^, E. Repa^2^, K. Spanidou^2^, C. Papachristou^1^

#### ^1^Psychology, Aristotle University of Thessaloniki; ^2^Parents' and Caregivers' Association of Children with Chronic Rheumatic Diseases, Thessaloniki, Greece

##### **Correspondence:** M. M. Delliou

*Pediatric Rheumatology 2023*, **21(Suppl 2):**PT104

**Introduction:** Previous data on Juvenile Idiopathic Arthritis (JIA) -the predominant type of Juvenile Rheumatic Diseases- report that this chronic disease affects both patient and family life. Relevant Greek data focusing on the family perspective are limited.

**Objectives:** To investigate the experience of parenting a child diagnosed with JIA, the interplay between JIA, family relations and everyday life, and coping behaviors regarding potential life changes.

**Methods:** This is a qualitative research study based on semi-structured in-depth interviews with parents of an offspring with JIA. The interview domains were: a. the experience of JIA diagnosis over time, b. the relationship between parents, the JIA patient and siblings, c. the impact of JIA on everyday life, d. their coping mechanisms. Interviews were transcribed, coded and analyzed anonymously in compliance with ethics research guidelines and following the principles of the interpretative phenomenological analysis.

**Results:** Nine parents (M:F, 3:6) aged 39-51 years (mean= 47), were included in the study. Their educational level differed: 3/9 had a high-school diploma, 5/9 a Bachelor’s degree and 1/9 a Master’s one. Interviews lasted 25 to 60 minutes. Our findings show that JIA led to the creation of strong bonds between parents, siblings, and the patient (9/9). JIA created confusion within families in early stages of the disease and frequently raised disagreements on issues related to medication (4/9). The parental focus on JIA minors occasionally fired jealousy of the healthy siblings (3/9). Additionally, JIA altered the family members’ daily life by shifting their focus on disease management (9/9) and frequently forced affected minors to modify their activities (4/9). The upcoming stress led parents to various coping mechanisms (9/9). 3/9 parents sought reliable information from Health Professionals, 6/9 shared their experience with other JIA affected families and 7/9 searched psychological support. Alternatively, 2/9 found intrafamilial discussions equally effective. 2/9 parents tried to maintain a positive attitude towards minor daily issues and disease management by developing easily understood and less frightening ways to present JIA impact to their minors. Impressively, 5/9 reported that JIA eventually had a positive impact by teaching the family to re-evaluate and re-prioritize problems and to appreciate moments of happiness. Finally, parents presented minors with JIA as fighters who developed discipline, empathy and diversity acceptance (4/9).

**Conclusion:** JIA post-diagnosis presents a challenge for parents introducing changes in relations and everyday life. However, most families gradually adjusted and accepted JIA acknowledging some beneficial effects. These findings can assist Health Professionals in supporting families with JIA and parents to develop constructive scenarios for the co-living with the chronic disease.

**Patient Consent**

Yes, I received consent

**Disclosure of Interest**

None declared

## PT105 Therapeutic benefits of gardening in paediatric rheumatology

### S. Francis^1^, P. Livermore^2^

#### ^1^Occupational Therapy; ^2^NIHR BRC Clinical Academic Lead, Great Ormond Street Hospital, London, United Kingdom

##### **Correspondence:** S. Francis

*Pediatric Rheumatology 2023*, **21(Suppl 2):**PT105

**Introduction:** In recent years, there has been a shift of focus in health, from illness to well-being. How we feel physically and mentally is one of the most important factors for our overall wellbeing and how we can manage and deal with diagnosis and changes in health makes a difference. A gardening group was piloted for children and young people attending a 2-week rheumatology rehabilitation programme. The aim of the group was to promote physical and mental wellbeing for children with a chronic rheumatological condition. Gardening is an activity which can be graded and adapted based on the child or young person’s abilities. It can be done seated or standing, using fine, gross motor and cognitive skills. It is also a sustainable activity.

**Objectives:** - Recent well-being research suggests that building the following 5 actions into our day to day lives is important for wellbeing: connect, be active, take notice, keep learning, giving. Gardening is an activity which encompasses all of these.

- Better physical health through exercise and learning how to use or strengthen muscles to improve mobility and upper limb strength.

- Improved mental health through a sense of purpose and achievement. Reduction of stress and anxiety.

- The opportunity to connect with others, reducing feelings of isolation or exclusion.

- Acquiring new skills and learning about growing food and nature.

- Just feeling better for being outside, connecting to nature, reducing time in hospital accommodation and time on electronic devices.

**Methods:** The pilot group ran over a 13-week period, located at a local community garden next to the hospital, for 90 minutes/session. It was facilitated by 2 occupational therapists/student and the head gardener. Activities varied depending on the season, sowing seeds, pricking out, watering, sweeping leaves, seed bombs, arts and craft related activities. The practice of self-management strategies related to rheumatology conditions was also included; warm up exercises, breathing exercises, upper limb stretches. Practice of Pacing and joint protection.

**Results:** We evaluated the pilot through looking at attainment and engagement and asked children to rate their experience. Overall the pilot was a success with many children keen to return. Thirteen sessions were carried out between July and November 2022, with 32 patients attending. Average attendance 2.5 children per group. Children attended 1-2 sessions. The 14 older children who completed an evaluation, all reported a sense of achievement.

**Conclusion:** To our knowledge this is the first piece of work considering the therapeutic benefit of gardening in paediatric rheumatology. Gardening offers many therapeutic benefits; promoting well-being, the opportunity to leave the hospital environment and is a sustainable activity. We aim to embed it in the rehabilitation programme permanently and open it up to other patients, such as rheumatology inpatients.

**Patient Consent**

Not applicable (there are no patient data)

**Disclosure of Interest**

None declared

**References**

1. Aked et al (2018) Five ways to well-being: Communicating the evidence.NEF economics as if people and the planet mattered. SDU Strategy https://networks.sustainablehealthcare.org.uk/occupational-therapy-sustainable-practice-network-ot-susnet/blog/2014/12/house-agrees
2. Soga et al (2016) Gardening is beneficial for health: A Meta-analysis. Preventative medicine reports 5 (2017) 92-99.
3. Thrive: Using gardening to change Lives: www.thrive.org.uk
4. Van Den Berg AE (2011) Gardening promotes neuroendocrine and affective restoration from stress. Journal of health psychology. Jan; Vol. 16 (1), pp. 3-11.

## PT106 “My condition is worth researching” co-producing research study recruitment strategies with and for adolescents and young adults

### L. E. Lunt^1,2^, A. Bridges^3^, L. Gahr^3^, A. M. Hood^4^, D. Ghio^4^, Y. R. A National Advisory Group^3^

#### ^1^Centre for Musculoskeletal Research, Versus Arthritis Centre for Epidemiology, The University of Manchester; ^2^Manchester Academic Health Science Centre, National Institute of Health Research Manchester Biomedical Research Centre, Manchester University NHS Foundation Trust, Manchester; ^3^ A National Advisory Group of the Barbara Ansell National Network for Adolescent Rheumatology, Your Rheum, Manchester; ^4^School of Health Science, Division of Psychology and Mental Health, Faculty of Biology, Medicine and Health, The University of Manchester, United Kingdom

##### **Correspondence:** D. Ghio

*Pediatric Rheumatology 2023*, **21(Suppl 2):**PT106

**Introduction:** A key part of the research process is the invitation and recruitment to studies. However, little is known about how and why young people decide to take part. Your Rheum is a UK young person’s advisory group, providing young people aged 11-24 and diagnosed with rheumatic condition(s), the opportunity to input into rheumatology research.

**Objectives:** To identify what information young people need to know when deciding to take part in research studies. Also, to co-produce strategies for future studies, in reaching young people to invite them to take part in research.

**Methods:** At a virtual Your Rheum meeting eight young people, (F=7, M=1, age range 12-24) took part in group discussions, sharing their experiences of taking part in research and their decision process. Online tools Mentimeter and Miro were used to aid conversations and share ideas.

**Results:** The majority of young people had experience of taking part in research as a study participant (n=5). Deciding to participate in research included the following considerations: benefit/impact (will the research help them/others); connecting with others (meeting other young people); research topic (important/relevant to them, passionate about); which is then balanced against convenience (was the research opportunity easy to find, will taking part be easy and quick), and reimbursement (incentives for participation, will I be valued). The clinic environment was highlighted as a good and trustworthy recruitment strategy – being approached by a member of the research team was considered ideal, even if it was someone they had not met previously. However, whilst the young people discussed being open to hearing about research opportunities, they reflected that they are rarely exposed to these invitations or hearing about current research. Many recalled little discussions of research at their clinical appointments, particularly when ‘young’ in a paediatric or adult clinic.

**Conclusion:** It is essential to understand the perspectives of intended study participants, to plan successful recruitment strategies. This not only includes *how* they take part but *why* they take part. Young people consider multiple factors about themselves and others before deciding to participate in research. Also, if young people are not aware of research, they can not take part in it. Ensuring we consider these factors when designing our studies and recruitment strategies is beneficial to all involved. Co-produced recruitment strategies would aid inclusive (and increased) research participation.

**Patient Consent**

Not applicable (there are no patient data)

**Disclosure of Interest**

None declared

## PT107 Exploring themes of vocational development in real-world UK paediatric and adolescent rheumatology clinical consultations

### L. E. Lunt^1,2^, S. Verstappen^1,2^, R. Lee^1,2^

#### ^1^Versus Arthritis Centre for Epidemiology, Centre for Musculoskeletal Research, The University of Manchester; ^2^National Institute of Health Research Manchester Biomedical Research Centre, Manchester Academic Health Science Centre, Manchester University NHS Foundation Trust, Manchester, United Kingdom

##### **Correspondence:** R. Lee

*Pediatric Rheumatology 2023*, **21(Suppl 2):**PT107

**Introduction:** Education, vocational development and early employment experiences are important stages of adolescence and young adulthood (AYA). They are key developmental milestones which inform and shape self-identity and impact outcomes in adulthood, such as employment participation. In addition, these non-medical factors are well recognised social determinants of health. Healthcare professionals are considered well positioned to support the vocational needs/issues of AYAs with long-term health conditions (LTHCs). However, it is important to understand more about how a healthcare professional currently support the vocational needs of AYAs with LTHCs, in a healthcare setting to inform practice and interventions.

**Objectives:** To explore how communication about education, vocational readiness and employment feature in real-world paediatric rheumatology clinical consultations, between healthcare professionals, children/young people and parents.

**Methods:** In this qualitative study real-world clinical consultations were audio-recorded across three paediatric and adolescent rheumatology departments in the UK and transcribed verbatim for analysis. Consultations took place in-person or virtually between June-December 2021. A thematic analysis approach was taken to identify themes and sub-themes within consultation communication, using NVivo software to manage the analysis process.

**Results:** 30 clinical consultations were analysed. Healthcare professional’s predominantly raised topics of conversation related to school/careers/employment (n=18/30). Five key themes were identified; 1. Mechanisms to start communication about vocational development 2. Direct and indirect support from healthcare professional in vocational development issues, 3. Career specific guidance, 4. Transitioning healthcare alongside vocational development, 5. Disclosure about past positive and negative experiences related to vocational issues.

**Conclusion:** Healthcare professionals are in a key position to support the vocational development needs of AYAs with LTHCs, although to date, it has not been clear in the literature how vocational development communication occurs in real-world clinical settings. It is evident from this novel data, that a range of components of communication pertaining to vocational development are raised in the healthcare setting. Further work is needed to explore how these components relate to existing interventions such as a transitional care plan.

**Patient Consent**

Not applicable (there are no patient data)

**Disclosure of Interest**

None declared

## PT108 Intervention framework to improve pediatric rheumatology clinical care: a qualitative inquiry among non-specialist healthcare workers in Kenya

### A. Migowa^1,2^, S. Bernatsky^3^, A. Ngugi^4,5^, H. Foster^6^, P. Muriuki^7^, R. Rianga^8^, S. Luchters^2,9,10^

#### ^1^Paediatrics and Child Health, Aga Khan University Medical College East Africa, Nairobi, Kenya; ^2^Population Health, Ghent University, Ghent, Belgium; ^3^Rheumatology, McGill University Health Centre, Montreal, Canada; ^4^Population Health, Brain and Mind Institute, Aga Khan University Medical College East Africa, Nairobi, Kenya; ^5^Centre for Global Health Equity, University of Michigan, Michigan, United States; ^6^Population and Health Institute, Newcastle University, Newcastle, United Kingdom; ^7^African Population and Health Research Centre; ^8^Population Health, Aga Khan University Medical College East Africa, Nairobi, Kenya; ^9^Centre for Sexual Health and HIV AIDS Research, Harare, Zimbabwe; ^10^Liverpool School of Tropical Medicine, Liverpool, United Kingdom

##### **Correspondence:** A. Migowa

*Pediatric Rheumatology 2023*, **21(Suppl 2):**PT108

**Introduction:** Delay in diagnosis and access to specialist care is a major problem for many children and young people with rheumatic disease in sub-Saharan Africa. Most children with symptoms of rheumatic disease present to non-specialists for care. There is an urgent need to understand and scale-up paediatric rheumatology knowledge and skills amongst non-specialist healthcare workers to promote early diagnosis, prompt referral, and management.

**Objectives:** We explored strategies from the perspective of healthcare workers in Kenya to improve the clinical care offered to pediatric rheumatology patients.

**Methods:** We conducted 12 focus group discussions with clinical officers (third-tier community health workers) nurses, general practitioners and paediatricians across 6 regions in Kenya. Interviews were conducted on zoom, audio-recorded, transcribed, and analyzed using MAXQDA software.

**Results:** A total of 68 individuals participated; 11 clinical officers, 12 nurses, 10 general practitioners, 27 paediatricians and 7 others. Most (*n* = 53) were female, and the median age was 36 years (range 31–40 years). Fifty per cent of the participants (34 of 68) worked in public health facilities across 6 regions of Kenya. Our study revealed the need for patient-centred interventions, health worker interventions and health system interventions.

Patient interventions proposed included individual patient education and psychosocial support. Proposed broader community interventions included outreach advocacy campaigns, financial support, prompt identification and referral of cases.

Health worker interventions recommended include clinical interventions aimed at availing diagnostic, management, referral and follow up guidelines. Educational interventions for the health workers should focus on symptom identification, management strategies and communication skills.

Health system interventions include availing diagnostic tools, improving access to care and promoting integrated holistic clinical care.

It was proposed that the interventions be delivered through a blend of in-person session, virtual platforms and social media forums.

**Conclusion:** Improving outcomes for pediatric rheumatology patients would require an integrated approach which is context specific for the various regions that still lack access to clinical care.

**Patient Consent**

Not applicable (there are no patient data)

**Disclosure of Interest**

None declared

## PT109 Children’s and parent’s experiences after completing the one-year Juvenile arthritis support program

### K. Mördrup^1,2^, E. Broström^2,3^, K. Palmblad^1^, J. G. Jungner^2^, C. Bartholdson^1,2^

#### ^1^Highly Specialized Pediatric Orthopedics and Medicine, Paediatric Rheumatology Unit; ^2^Womens and Childrens Health, Karolinska Institutet, ^3^Womens and Childrens Health, Center for Paediatric Clinical Trials, Stockholm, Sweden

##### **Correspondence:** K. Mördrup

*Pediatric Rheumatology 2023*, **21(Suppl 2):**PT109

**Introduction:** In Sweden, approximately 2000 children are living with Juvenile Idiopathic Arthritis (JIA)^1^. About 200 children are diagnosed every year. When the child is diagnosed, the families often experience loneliness and lack of information, with few resources available to help them^2^. However, information provided at the time of diagnosis can be difficult to remember^3^. Therefore, families require repeated information and supportive care^4^. Against the background, a one-year Juvenile Arthritis support program (JASP-1) was developed, consisting of 7 patient-and family-centered visits planned during the first year after JIA diagnosis.

**Objectives:** To describe the levels of children’s and parent’s satisfaction after completing the Juvenile arthritis support program (JASP-1) and compare outcomes with children and parents who received standard care.

**Methods:** Children diagnosed with JIA and their parents were offered the opportunity to participate in the JASP-1 from the time of diagnosis and the following year. One year after JIA diagnosis, the children and/or their parents were invited to answer a study-specific questionnaire comprising 16 questions. The questionnaire assessed their experiences with the information, communication, participation, and emotional support they had received during the first year with JIA. In order to compare outcomes, the questionnaire was answered by both participants in JASP-1 and patients and parents receiving standard care.

**Results:** Totally 56 children, along with their parents, participated in the JASP-1, while 24 received standard care. In all 16 questions the participants in JASP-1 reported higher levels of satisfaction with their care compared to those receiving standard care. In 9 of the 16 questions, the results showed significant differences. Some examples on questions with significant differences include the assessment of the child’s health condition, whether they received information about how the health condition could affect everyday life, and if they have had the opportunity to receive supportive care if needed.

**Conclusion:** The children and parents who participated in the JASP-1 expressed higher satisfaction levels with the care they received compared to those who received standard care. Based on these findings, we conclude that the JASP-1 is likely an effective way to support children and parents after diagnosis and has the potential to improve quality of care within pediatric rheumatology.

**Patient Consent**

Not applicable (there are no patient data)

**Disclosure of Interest**

None declared

**References**

1. https://barnreumaregistret.se/for-patienter/information-om-barnreumatism/
2. Yuwen, W., Lewis, F. M., Walker, A. J., & Ward, T. M. (2017). Struggling in the Dark to Help My Child: Parents' Experience in Caring for a Young Child with Juvenile Idiopathic Arthritis. *J Pediatr Nurs*, *37*, e23-e29. 10.1016/j.pedn.2017.07.007
3. Heath-Watson S and Sule S. Living with Juvenile Idiopathic Arthritis: Parent and Physician Perspectives. *Rheumatology and therapy*. 2018; 5: 1-4.
4. van Dijkhuizen EHP, Egert T, Egert Y, et al. Patient's experiences with the care for juvenile idiopathic arthritis across Europe. *Pediatr Rheumatol Online J*. 2018; 16: 10.

## PT110 Aan eight week physiotherapy intervention in ten patients with juvenile idiopathic arthritis and temporomandibular joint involvement

### M. Nørgaard^1^, P. B. Stoustrup^2^

#### ^1^Department of Physiotherapy, Aarhus University Hospital, Palle Juul-Jensens Boulevard 123, DK-8200 Aarhus N; ^2^Section of Orthodontics, Department of Dentistry, Aarhus University, Vennelyst Boulevard 9-11, DK-8000 Aarhus C, Aarhus, Denmark

##### **Correspondence:** M. Nørgaard

*Pediatric Rheumatology 2023*, **21(Suppl 2):**PT110

**Introduction:** Incidence of Temporomandibular joint (TMJ) involvement in patients with Juvenile Idiopathic Arthritis (JIA) is high and frequently impair joint/muscle function, leading to joint degeneration, dentofacial deformities and mandibular growth disturbances. Orofacial symptoms and functional impairment of TMJ and surrounding tissues are seen in 26-74% of cases. No treatment consensus exists and modalities varies from counselling to surgery. Improving aesthetics, function and pain reduction seems effective. However, intervention studies are sparse and heterogeneous, leading to low evidence, and little is known about effectiveness of physiotherapy (PT) in children with TMJ-arthritis.

**Objectives:** To assess effects of orofacial PT and self-managed exercise programs in JIA-patients with TMJ-involvement, and degree of patient compliance to this treatment.

**Methods:** Individual PT-treatments were performed once weekly for eight weeks with PT-modalities according to clinical features, using intra-/extra-oral manipulation, orofacial massage, muscle strengthening, muscle/tissue stretching, relaxation techniques etc. An individualized self-managed exercise program was performed daily.

**Results:** Ten JIA-patients (all females) aged 12-18 years with acute/chronic arthritis in one/both TMJ(s) participated. Nine of 10 patients had bilateral TMJ-involvement. TMJ-symptoms were pain, dysfunction, clicking/crepitation, decreased joint range of motion (ROM), muscle disturbances etc. Two patients reported more pain in non-/least affected TMJ (overuse), and two patients had excessive ROM in affected TMJ(s) (less use/hypermobility). All patients reported orofacial muscle/tissue pain during treatment of specific muscles, predominantly mm. pterygoids (external/internal), supra-/infra-hyoids and masseters. More patients had decreased ROM of cervical joints and/or pain in neck muscles (mm. scalene, sternocleidomastoids etc.).

Pain intensity (VAS 0-10) decreased in 6/10 patients (mean decrease 1,89 (range 0.7-3.9)). In 3 patients, intensity increased 0.5, and in one patient 4.0 (disease flare). Pain frequency (NRS 0-4) decreased in 8/10 patients (mean decrease 1.25 (range 1-3)), whereas 2/10 had steady levels (3).

In all patients, interventions were well tolerated, and all reported diminishing PT-related symptoms during intervention period. Compliance to PT-interventions/home exercises were 100%/95-100%, respectively. All patients reported improvements of overall symptoms during intervention period, especially own competence in obtaining improvements with specific PT-tools.

**Conclusion:** The eight-week intervention with physiotherapy and self-managed exercises improved patients´ symptoms of TMJ-arthritis, notably their competence in managing them. Thus, physiotherapy should be considered part of standard care in TMJ-arthritis, though more studies are warranted.

**Patient Consent**

Not applicable (there are no patient data)

**Disclosure of Interest**

None declared

## PT111 Juvenile idiopathic arthritis does not affect school performance – a national register-based study

### M. J. Pedersen^1^, C. Høst^2^, S. N. Hansen^1^, J. Klotsche^3^, K. Minden^3^, B. Deleuran^4,5^, B. H. Bech^1^

#### ^1^Department of Public Health, Aarhus University; ^2^Department of Paediatric and Adolescent Medicine, Aarhus University Hospital, Aarhus, Denmark; ^3^Deutsche Reuma-Forschungszentrum Berlin, Berlin, Germany; ^4^Department of Biomedicine, Aarhus University; ^5^Department of Rheumatology, Aarhus University Hospital, Aarhus, Denmark

##### **Correspondence:** M. J. Pedersen

*Pediatric Rheumatology 2023*, **21(Suppl 2):**PT111

**Introduction:** Previous studies have shown conflicting results on school performance and academic achievements among children with juvenile idiopathic arthritis (JIA). No study has to our knowledge investigated school performance of children with JIA compared to healthy peers on a national level.

**Objectives:** We aimed to compare the results of the National Danish School Testing and final 9^th^ grade exams in the subjects Danish and mathematics between children with JIA and their peers. Further to study possible differences across parental socioeconomic status (SES).

**Methods:** A population-, register-based cross-sectional study was performed. The study population included all children participating in at least one National School Testing in reading or mathematics between 2^nd^ and 8^th^ grade from 2011 to 2019 (n = 812,461). A limit of at least five hospital contacts with a diagnosis code of JIA (ICD-10 codes DM08 and DM09) before the date of the test was used to define JIA patients in our study. Linear regression was used to estimate differences in mean test scores between children with JIA and their peers on the different school grade levels and subjects. Analyses of the final exam grades were stratified on SES variables to test for interaction.

**Results:** Fifteen hundred forty-one children with JIA participated in at least one National School testing. The results of the National Danish School Testing showed no significant difference in scores between children with JIA and children without JIA in reading (mandatory in grade 2, 4, 6, and 8). In mathematics there was no statistically significant difference in the school test results in 3^rd^ and 8^th^ grade but children with JIA scored almost 2 points lower (coefficient -1.73 95% CI [-3.33; -0.13]) than their peers in mathematics in 6^th^ grade. We found no statistically significant differences in the mean grades of the final 9^th^ grade exams in neither Danish nor mathematics between children with JIA and children without JIA. Family income was the only SES factor affecting the association between JIA and final exam grade scores. For children living in high income families children with JIA had higher average exam scores than peers but for children living in middle income families children with JIA had lower average exam scores than peers. Children with JIA of low income families did not differ statistically significant from their peers.

**Conclusion:** Children with JIA perform as good as their peers in school. Family income was the only SES factor associated with the final exam results.

**Patient Consent**

Not applicable (there are no patient data)

**Disclosure of Interest**

None declared

## P001 Could early presentation and diagnostics predict JIA: an analysis of pediatric patients presenting to ped with non-traumatic joint pain

### D. Karakaitė^1^, E. Ambrozaite^2^, A. Sanipaitiene^3,4^, L. Jankauskaite^3,4^

#### ^1^Lithuanian University of Health Sciences, Kaunas, Lithuania; ^2^Medical Academy; ^3^Department of Pediatrics, Lithuanian University of Health Sciences; ^4^Department of Pediatrics, Hospital of Lithuanian University of Health Sciences Kauno Klinikos, Kaunas, Lithuania

##### **Correspondence:** A. Sanipaitiene

*Pediatric Rheumatology 2023*, **21(Suppl 2):**P001

**Introduction:** Juvenile idiopathic arthritis (JIA) is chronic autoimmune disorder characterized by inflammation of one or more joints leading to stiffness, swelling, and pain. Early JIA diagnosis and treatment can improve outcomes. Many other diseases can mimic initial episode of JIA; thus, it is crucial to identify early signs of chronic disease.

**Objectives:** We aimed to determine distribution of symptoms and treatment as well as most frequent diagnostic tests in patients presenting with joint pain to a pediatric emergency department (PED).

**Methods:** Retrospective data analysis from electronic healthcare record system was conducted. Data of all children presenting to PED complaining of joint pain January 2018-February 2022 were analysed. Cases were divided into two groups: children progressing to chronic form of the disease (JIA), and those who did not (nJIA). Statistical data analysis was performed using SPSS 29.0. P value <0.05 was considered significant.

**Results:** Data of 110 children (55 JIA, 55 nJIA) were analysed; 63.6% were female. Median age was 12 (6-15) and did not differ in both groups. Median days from the onset of symptoms did not differ (61d as for JIA vs 31d as for nJIA, p>0.05). Pain was predominant symptom in both groups (92.73%-JIA; 89.1%-nJIA). Children complained of joint swelling and joint stiffness more frequently in JIA group compared to nJIA (74.5% vs 47.3% and 41.8% vs 29.1% respectively).

In PED, CBC was performed in 99.09% (n=109) cases, ESR in 93.64% (n=103). However, only eosinophil count was higher in JIA compared to nJIA (0.2x10*9 (0.1;0.32) vs 0.13x10*9 (0.08;0.2)). All were referred to pediatric rheumatologist. During follow-up, ANA was tested in 84.5% (n=94). 54.5% (n=61) had positive result of which 62.3% were from JIA group. In PED, 105 children (95.5%) received joint ultrasound. Effusion was found in 75 patients (71.4%), of which 56% belonged to the JIA group. In the same group, one joint involvement prevailed (n=30, 60%), polyarthritis was diagnosed only in 3 patients (6%). Less than half of nJIA patients (n=22, 40%) presented with no joint pathology in ultrasound. All patients were prescribed NSAIDs in PED.

**Conclusion:** Our research uncovered that the pain symptom predominated in both patients with subsequent JIA and without at the visit to PED. None of investigations in PED was able to identify chronic disease. Therefore, all patients suspected of nontraumatic joint pain should be referred to pediatric rheumatologist.

**Patient Consent**

Not applicable (there are no patient data)

**Disclosure of Interest**

None declared

## P002 Etiology and characteristics of arthritis in children

### A. Demir^1^, N. Çakar^2^, Z. B. Özçakar^2^, F. Aydin^2^, G. Vatansever^1^, T. Uçar^3^, E. Çiftçi^4^, E. Ünal^5^, F. Yalçinkaya^2^

#### ^1^Department of Pediatrics; ^2^Pediatric Rheumatology; ^3^Pediatric Cardiology; ^4^Pediatric Infectious Diseases; ^5^Pediatric Oncology, Ankara University School of Medicine, Ankara, Türkiye

##### **Correspondence:** F. Aydin

*Pediatric Rheumatology 2023*, **21(Suppl 2):**P002

**Introduction:** Arthritis is described as an inflammation of the joint. Arthritis in children could be one of the clinical signs of many different diseases.

**Objectives:** The aim of this study is to evaluate the etiology of arthritis in children.

**Methods:** Files of patients who were diagnosed with arthritis between "January 2016 - December 2019" in Ankara University Faculty of Medicine were retrospectively evaluated.

**Results:** The mean age of 424 patients included in the study was 8.68±4.30 (0.07-17.92) years, and 218 (51.4%) were girls. Fifty-two percent of the patients had rheumatic disease (19.6% juvenile idiopathic arthritis [JIA], 16.3% autoinflammatory disease, 14.2% vasculitis, 1.7% autoimmune connective tissue disease), 23.6% had reactive arthritis (RA), 12% had acute rheumatic fever (ARF), 5.2% had malignant disease, 4.7% had septic arthritis, and 2.8% had orthopedic disease. The most commonly involved joint was the knee (55%). Monoarticular involvement was more common in septic arthritis (85%), malignant diseases (77%), reactive arthritis (71%), and orthopedic diseases (66.7%). Forty-five percent of the patients with ARF had migratory arthritis, and 29.4% had monoarthritis. The majority of the patients (68.9%) presented with acute arthritis, and these rates were 92% in RA, 98% in vasculitis, 90% in septic arthritis, and 82.4% in familial Mediterranean fever (FMF). Chronic arthritis was more common in patients with JIA, chronic recurrent multifocal osteomyelitis (CRMO), and non-leukemia malignant diseases than the others (p<0.001). The history of recurrent arthritis was high in patients with Behçet's disease (BD), FMF, CRMO, JIA, and autoimmune connective tissue diseases (100%, 86.3%, 81.8%, 73.5%, and 71.4%, respectively). Among nine patients with leukemia, 5 of them were misdiagnosed as having other diseases (3 RA, 1 ARF, and 1 traumatic arthritis).

**Conclusion:** Arthritis in children could be one of the clinical signs of many different diseases. Rheumatic and infectious causes were detected as the major etiologic factors of arthritis in childhood in our center. It should also be kept in mind that arthritis may be a manifestation of malignant diseases.

**Patient Consent**

Not applicable (there are no patient data)

**Disclosure of Interest**

None declared

## P003 The incidence and prevalence of juvenile idiopathic arthritis differs by ethnic group in England

### R. P. Beesley^1^, K. Hyrich^1,2,3^, J. H. Humphreys^1,2^

#### ^1^Centre for Epidemiology, University of Manchester; ^2^Kellgren Centre for Rheumatology, Manchester Royal Infirmary; ^3^National Institute for Health Research Manchester Biomedical Research Centre, Manchester, United Kingdom

##### **Correspondence:** R. P. Beesley

*Pediatric Rheumatology 2023*, **21(Suppl 2):**P003

**Introduction:** Juvenile Idiopathic Arthritis (JIA) is a heterogenous group of autoimmune disorders characterised by chronic joint inflammation, affecting children and young people (CYP) under the age of 16. The association between ethnicity and JIA has not been investigated previously in the UK context. Differential rates between ethnic groups could be indicative of underlying biological differences and/or health inequities.

Health inequities (avoidable systemic differences in health outcomes for different population groups) may lead to specific ethnic groups being less likely to be referred resulting in delays in diagnosis and apparent differences in incidence rates.

**Objectives:** To calculate and compare the incidence and prevalence rates of JIA in different ethnic groups in England.

**Methods:** CYP with JIA were identified in anonymised electronic primary care records (the Clinical Practice Research Datalink (CPRD) Aurum database) using pre-defined read code lists between 1 January 2003 and 31 December 2018. Cases were further validated through linked Hospital Episode Statistics (HES) data with either >=3 outpatient specialist care (rheumatology/paediatric rheumatology) appointments or a HES inpatient admission coded with JIA, prior to age 16. Ethnic group was extracted from CPRD/HES records and aggregated to broad ethnic groups. Incidence and prevalence rates by broad ethnic group were calculated using CYP under the age of 16 in CPRD, as of December 2018. Indirect standardisation was performed by age and region using ONS Census 2021 data, to account for varying ethnic make-up across different age groups and regions of England. The distribution of observed JIA cases across ethnic groups was subsequently compared with the expected distribution based on population statistics using Chi2.

**Results:** A total of 424 incident cases were identified in CYP <16y between January 2003 and December 2018 using code lists. The overall age and region indirectly standardised incidence rate was 5.4 per 100,000 population age <16y, varying from 6.2 for White CYP to 2.7 for CYP with Asian ethnic group.

A total of 389 HES-validated cases were identified, giving an indirectly standardised incident rate of 5.4 per 100,000 population age <16y, varying from 6.3 for White CYP to 2.9 for CYP with Asian ethnic group.

In December 2018 there were 795 prevalent cases from code lists (742 validated using HES). The indirectly standardised prevalence rate was 59 per 100,000 CYP under the age of 16, varying from 68 for White CYP to 29 for CYP with Mixed ethnic group

Incidence and prevalence of JIA were statistically significantly lower amongst all non-White ethnic groups compared to White ethnic group.

**Conclusion:** The incidence and prevalence of JIA amongst CYP in England differs by ethnic group, being highest amongst ‘White’ CYP and lower amongst other ethnic groups, and is not in keeping with the known distribution of ethnic groups in the <16y England population. Understanding whether this reflects a health inequity or differences in the underlying biology of JIA needs further investigation.

**Patient Consent**

Not applicable (there are no patient data)

**Disclosure of Interest**

None declared

## P004 Anti-adalimumab in jia, a practical french experience on a cohort of 47 patients

### M. Blin^1,2^, P. Quartier^3,4^, B. Bader Meunier^3^, S. Chhun^5^, D. Ternant^6^, F. Uettwiller^1,2^

#### ^1^Paediatric Rheumatology Department, Tours University Hospital; ^2^Department of Clinical Immunology and Allergology, Tours; ^3^Pediatric Immunology-Hematology and Rheumatology Unit; ^4^University Paris-Descartes; ^5^Laboratory of Immunology, Necker Hospital, Assistance Publique-Hôpitaux de Paris, Centre-Université de Paris, Paris, France, Paris; ^6^Tours University Hospital, Tours, France; Medical Pharmacology Department, Tours, France, Tours, France

##### **Correspondence:** M. Blin

*Pediatric Rheumatology 2023*, **21(Suppl 2):**P004

**Introduction:** Anti-TNF antibodies represent a major therapeutic advance in the treatment of chronic inflammatory diseases, including JIA. Despite the fully humanized nature of adalimumab, numerous patients experienced an incomplete or loss of response to adalimumab by the formation of anti-drug antibodies [1][2]. For JIA, there are no guidelines regarding adalimumab dosing and AAA (anti-adalimumab antibodies) detection in France.

**Objectives:** The aim of this work was to report clinical practices within two french centers (of reference and competence) regarding the indications for the dosage of adalimumab and to describe therapeutic attitude resulting from it.

**Methods:** Data were collected in 47 patients from two french centers AP-HP (Greater Paris University Hospitals) and University Hospitals of Tours, from January 2015 to January 2020. Patients under 18 years old and with juvenile idiopathic arthritis according to ILAR criteria had to receiving adalimumab for more than 3 months and have at least one adalimumabemia assay during the period of interest. Adalimumabemia assay is performed using the enzyme-linked immunosorbent assay, dosing AAA were performed for residual adalimumab levels < 1μg/mL at the reference center laboratory and for levels < 0.1μg/mL at the center of competence.

**Results:** Fourty-seven patients followed for JIA were included (sex ratio 0.42), 61 assays were performed. The mean adalimumabemia was 7.91 μg/mL (+/- 8.01). AAA were positive for 28% of assays, i.e. 1 out of 4 patients, with an average comparative adalimumab level of 0.48 μg/mL (+/- 0.3).

Almost 75% of the assays were performed for joint and/or ophthalmologic activity, the remaining 25% for routine dosing during follow-up. There were signs of immunization at the consultation for 6.6% of the dosages. Assays performed in the context of joint and ophthalmologic activity were significantly associated with the presence of AAA (p=0.01).

When AAA was detected, 82.4% (14/17 assays, p=0.0034) of patient’s therapeutics were modified. In the presence of AAA, the clinician opted to discontinue adalimumab in 64.7% of cases (11/17 assays; p<0.0001), mostly replacing it with another biotherapy as infliximab (anti-TNF) or tocilizumab (anti-IL6).

**Conclusion:** In the presence of AAA with low levels of adalimumabemia, the clinician adjusted the treatment in a large proportion of patients, discontinuing it in more than 2 out of 3 children. For the remaining patients, treatment was extended with an adjustment of therapeutics.An additional study to determine the clinical and biological factors guiding the practitioner in his therapeutic attitude and therapeutic thresholds in JIA remains to be carried out.

**Patient Consent**

Yes, I received consent

**Disclosure of Interest**

None declared

**Reference**

1. S. Masegosa, S. Copete, R. Jimenez, E. Collantes, et R. Roldan, « AB0881 Anti-Adalimumab Antibodies in Juvenil Idiopathic Arthritis and Loss of Response. Preliminary Study », *Ann. Rheum. Dis.*, vol. 75, n^o^ Suppl 2, p. 1203-1204, juin 2016, doi: 10.1136/annrheumdis-2016-eular.5838.
2. A. Skrabl-Baumgartner, W. Erwa, W. Muntean, et J. Jahnel, « Anti-adalimumab antibodies in juvenile idiopathic arthritis: frequent association with loss of response », *Scand. J. Rheumatol.*, vol. 44, n^o^ 5, p. 359-362, sept. 2015, doi: 10.3109/03009742.2015.1022213.

## P005 The effect of disease activity on physical fitness, quality of life and functionality in patients with Juvenil idiopatic arthritis

### S. Bozcuk^1^, B. Başakçı Çalık^1^, E. Gür Kabul^2^, S. Yüksel^1^

#### ^1^Pamukkale University, Denizli; ^2^Uşak University, Uşak, Türkiye

##### **Correspondence:** S. Bozcuk

*Pediatric Rheumatology 2023*, **21(Suppl 2):**P005

**Introduction:** Juvenile Idiopathic Arthritis (JIA), is the most common rheumatic disease of childhood. The activity level of these children is low from an early age and important problems are observed in the realization of daily living activities (1). While children with JIA show similar basic motor skills compared to their healthy peers; these children participate in physical activities less than their peers from a young age (2).

**Objectives:** This study was conducted to examine the effect of disease activity on physical fitness, quality of life and functionality in children with JIA.

**Methods:** 32 children with JIA (mean age: 13.62±2.25 years) with an age range of 10-17 years were included in the study. Children with JIA were divided into two groups as low (n=19) and moderate (n=13) disease activity levels. After recording demographic data, disease activity was determined by JADAS-27 (Juvenile Arthritis Disease Activity Score), physical fitness level by Brockport physical fitness test battery, quality of life by PedsQl 3.0 arthritis module (Pediatric Quality of Life Inventory), and functionality by CHAQ (Childhood Health Assessment Questionnaire) was evaluated.

**Results:** As a result of the comparative analysis; In terms of physical fitness levels, the push-up test (p=0.027) was significant in favor of low disease activity, while the difference was not significant in other subtests. In terms of quality of life; some PedsQL child form which is pain (p=0.002), total score (p=0.040), activities of daily living (p=0.013) of children with JIA; there was significant in favor of low disease activity. Some PedsQL parent form which is pain (p=0.002), total score (p=0.033), activity of daily living (p=0.039), of children with JIA; there was significant in favor of low disease activity. In terms of functionality which is CHAQ eating (p=0.022), reaching (p=0.000), rising (p=0.006), walking (p=0.013), hygiene (p=0.001), activity (p=0.047), total score (p=0.003) ), pain (p=0.000) and general well-being (p=0.000), the difference was significant in favor of low disease activity.

**Conclusion:** According to the results of our study, we found that disease activity had a similar effect on the physical fitness level of children with JIA and that the disease had a bad effect on physical fitness even at low disease activity level. For this reason, we believe that children with JIA regardless of disease activity levels should be guided to recreational sports and they should be trained in terms of increasing physical activity In addition, it was observed that disease activity negatively affected the quality of life of both children with JIA and their parents, and the functionality of the children. We suggest that it should not be forgotten that the quality of life can be increased and functionality can be improved by controlling the disease activity.

**Patient Consent**

Not applicable (there are no patient data)

**Disclosure of Interest**

None declared

**References**

1. Bohr, AH; Nielsen, S.; Müller, K.; Karup Pedersen, F.; Andersen, LB Juvenil İdiyopatik Artritli çocuk ve ergenlerde tatmin edici inflamasyon kontrolüne rağmen fiziksel aktivitede azalma. ***Pediatr Rheumatol Online J*** 2015, 13, 57.
2. Hulsegge G, Henschke N, McKay D, Chaitow J, West K, Broderick C, Singh-Grewal D. Jüvenil idiyopatik artritli Avustralyalı çocuklar arasında temel hareket becerileri, fiziksel uygunluk ve fiziksel aktivite. ***J Pediatr Çocuk Sağlığı*** 2015; 51(4): 425-32.

## P006 Investigation of the validity and reliability of the turkish version of the child and adolescent participation scale (CASP) questionnaire in individuals with Juvenile idiopathic arthritis: a pilot study

### S. Buran^1^, M. O. Tüfekçi^2^, N. B. Karaca^2^, Y. Bayındır^3^, V. Yıldız Kabak^4^, S. Atasavun Uysal^4^, E. Aliyev^3^, Y. Bilginer^3^, E. Ünal^1^, S. Özen^3^

#### ^1^Department of Heart and Respiratory Physiotherapy and Rehabilitation, Hacettepe University Faculty of Physical Therapy and Rehabilitation; ^2^Department of Basic Physiotherapy and Rehabilitation, Hacettepe University Institute of Health Sciences; ^3^Department of Pediatrics, Division of Rheumatology, Hacettepe University Faculty of Medicine; ^4^Department of Basic Physiotherapy and Rehabilitation, Hacettepe University Faculty of Physical Therapy and Rehabilitation, Ankara, Türkiye

##### **Correspondence:** S. Buran

*Pediatric Rheumatology 2023*, **21(Suppl 2):**P006

**Introduction:** “Participation” is an important parameter from the perspectives of both the Outcome Measures in Rheumatology (OMERACT) 2018 JIA Core Set and the ICF-CY (International Classification of Functioning Disability and Health - Children and Adolescents) (1-2) however, there is a need for valid and reliable measurement tools in Turkish to assess the participation status of individuals with JIA.

**Objectives:** The aim of our study was to investigate the validity and reliability of the Turkish version of the Child and Adolescent Participation Scale (CASP) in individuals with JIA.

**Methods:** Our study included 60 people who were followed up with the diagnosis of JIA in Hacettepe University Pediatric Rheumatology clinic between March 2022 and September 2022. Participation (CASP), functionality (Childhood Health Assessment Questionaire (CHAQ)) and biopsychosocial status (Juvenile Arthritis Biopsychosocial and Clinical Questionnaire (JAB-Q)) of individuals with a diagnosis of JIA whose demographic information was obtained were recorded. For reliability, CASP was applied a second time to randomly selected 20 children seven days after the assessments. Internal consistency was determined by Cronbach's alpha and test-retest reliability was determined by ICC (Intraclass Correlation Coefficient).

**Results:** The mean age of individuals with JIA included in the study was 13.61 ± 3.22 years. The participants' disease activity scores (median (IQR)) were JADAS-71 2.0 (0.0/8.0) for individuals with JIA (n=51) and BASDAI 0.6 (0.1/4.2) for individuals with ERA (n=9). Test-retest was applied to 20 individuals. According to the results of the correlation analysis performed for validity, CASP total score and CHAQ disability index score (rho=-0.429, p=0.001) showed moderate; CHAQ pain, CHAQ general well-being, and JAB-Q total score (rho=-0.272, p=0.036; rho= -0.386, p= 0.002; rho=-0.317, p=0.014, respectively) showed weak correlation. Cronbach's alpha coefficient for internal consistency was found to be 0.924 and ICC coefficient 0.948 (p<0.001) with very high reliability.

**Conclusion:** Data from this pilot study, in which we examined the validity (correlation with other scales) and reliability (test-retest method) of CASP in individuals with JIA, showed that CASP is potentially a valid and reliable scale for assessing participation.The moderate and low correlations with other scales can be explained by the absence of a scale that fully meets the participation parameter, as explained in the German version study of CASP (3). Considering the potential of our preliminary results, it was concluded that the study should continue until the number of cases compatible with the number of scale items is reached.

**Patient Consent**

Not applicable (there are no patient data)

**Disclosure of Interest**

None declared

**References**

1. Tofani M, Mustari M, Tiozzo E, Dall'Oglio I, Morelli D, Gawronski O, et al. The development of the International Classification of Functioning, Disability and Health for Child and Youth (ICF-CY) Core Sets: a systematic review. Disabil Rehabil. 2022;1-10.
2. Morgan EM, Munro JE, Horonjeff J, Horgan B, Shea B, Feldman BM, et al. Establishing an Updated Core Domain Set for Studies in Juvenile Idiopathic Arthritis: A Report from the OMERACT 2018 JIA Workshop. J Rheumatol. 2019; 46(8), 1006–1013.
3. De Bock F, Bosle C, Graef C, Oepen J, Philippi H, Urschitz MS. Measuring social participation in children with chronic health conditions: validation and reference values of the child and adolescent scale of participation (CASP) in the German context. BMC Pediatr. 2019;19(1):125.

## P007 The right side is more frequently involved in Juvenile idiopathic arthritis children

### M. Burrone^1^, S. M. Orsi^1^, A. I. Rebollo-Gimenez^2^, F. Ridella^1^, L. Carlini^3^, M. Gattorno^2^, Y. Uziel^4^, M. Trachana^5^, P. Lahdenne^6^, P. Dolezalova^7^, A. Ravelli^8^, A. Consolaro^1,2^, on behalf of the Paediatric Rheumatology International Trials Organisation (PRINTO)

#### ^1^Dipartimento di Neuroscienze, Riabilitazione, Oftalmologia, Genetica e Scienze Materno-Infantili (DiNOGMI); ^2^UOC Reumatologia e Malattie Autoinfiammatorie; ^3^UOC Servizio di Sperimentazioni Cliniche Pediatriche PRINTO, IRCCS Istituto Giannina Gaslini, Genova, Italy; ^4^Pediatric Rheumatology Unit, Meir Medical Center, Kfar Saba, Tel Aviv, Israel; ^5^Pediatric Immunology and Rheumatology Referral Center, Hippokration General Hospital, Thessaloniki, Greece; ^6^Pediatric Rheumatology, Children's Hospital, Helsinki, Finland; ^7^Department od Paediatrics and Inherited Metabolic Disorders, 1st Faculty of Medicine, Charles University and General University Hospital, Prague, Czech Republic; ^8^Direzione Scientifica, IRCCS Istituto Giannina Gaslini, Genova, Italy

##### **Correspondence:** M. Burrone

*Pediatric Rheumatology 2023*, **21(Suppl 2):**P007

**Introduction:** Juvenile Idiopathic Arthritis (JIA) is the most common form of chronic rheumatic disease in children, as well as an important cause of long and medium-term disability. Oligoarthritis accounts for up to 50% of all children with chronic arthritis in Western countries and it predominantly asymmetric affects the joints of the lower extremities, with the knee being most frequently involved, followed by the ankle. Multiple previous studies tried to suggest that intra-articular mechanical stress is an important factor contributing to joint inflammation. A recent study showed that joints on the dominant side in the right-handed are clinically and radiologically more affected than the non-dominant side in patients with rheumatoid arthritis. It is still unclear whether right-side joints get more affected in patients with JIA.

**Objectives:** To evaluate the pattern of joint involvement in JIA children and the laterality of joint involvement in a large multinational dataset.

**Methods:** Children with JIA enrolled in the EPidemiology, treatment and Outcome of Childhood Arthritis (EPOCA)study were considered for this analysis. The EPOCA dataset is made of patients seen consecutively for a period of 6 months in 118 pediatric rheumatology centers in 49 countries. Each patient underwent retrospective and cross-sectional assessments, including measures of disease activity and damage and questionnaires on the well-being and quality of life of the children. We assessed the frequency of single joint involvement and we compared the difference between right and left side. The Exact Poisson Method has been used to test the ratio of the two rates. We considered only the swollen joint count and not tender and limited joints because the latter are affected by perception of patient and can be expected to be overestimated in the dominant side.

**Results:** We included a total of 9,081 patients with JIA. In the right side, we observed a total of 6459 swollen joints, compared to the 6174 present in the left side. We observed a significantly higher (p-value = 0.01) incidence rate in terms of swollen joints in the right side (IR, [95% CI]: 0.71 [0.69, 0.73]) than in the left side (0.68 [0.66, 0.69]). When we separately analyzed the upper and lower extremities, we saw the same difference only for the upper extremities (p < 0.01 for upper arms, p = 0.3 for lower arms). In addition, we separately evaluated each joint to analyze whether there were joint-specific tendencies, via a chi-square test for each joint. Of the 34 joints evaluated, 21 (61.7%) showed a higher proportion of swollen joints in the right side and 10 (29.4%) higher proportion of swollen joints in the left side. Of the 17 joints in the upper arm, 15 (88.2%) showed a higher proportion of swollen joints in the right side. However, just the knee and the 1st MCP showed a statistically significant prevalence in the right side (p < 0.05).

**Conclusion:** The current study indicates that the right side is clinically more affected compared to the left side in patients with JIA. Although the absolute difference was small, the same tendency was observed in most individual joints (61.7%). This difference is more relevant when considering only the upper arm, which use can be considered to be more affected by dominancy. Our results could be potentially underestimated because the patients’ side dominancy was not available in the dataset. These findings could potentially reflect aspects of the disease pathophysiology not yet unveiled.

**Patient Consent**

Not applicable (there are no patient data)

**Disclosure of Interest**

None declared

## P008 The efficiency of rheumatologic physical exam done via zoom video communication vs. face-to-face physical exam of children with Juvenile Idiopathic Arthritis (JIA)

### Y. Butbul Aviel^1^, Y. Azulay^1^, R. Tal ^2^, G. Amarylio^2^

#### ^1^e Department of Pediatrics B, Ruth Rappaport Children's Hospital, Rambam Medical Center, Haifa, Israel, Haifa; ^2^Pediatric Rheumatology Unit, Schneider Children's Medical Center, Petach Tikva, Israel

##### **Correspondence:** Y. Butbul Aviel

*Pediatric Rheumatology 2023*, **21(Suppl 2):**P008

**Introduction:** Juvenile Idiopathic Arthritis (JIA) is the most common chronic inflammatory rheumatic condition of childhood. This condition requires regular medical monitoring to prevent complications. This follow up can be done virtually. With the emergence of the COVID pandemic and lockdowns virtual assessment become crucial. Yet, the efficiency of remote assessment of children with JIA remained unclear.

**Objectives:** To assess the efficiency of rheumatologic physical exam and treatment decisions made virtually Vs. face-to-face (f2f) in children with JIA in two tertiary Israeli hospitals, Rambam and Schneider medical center. Additional goal was to evaluate the parents satisfaction with virtual assessment.

**Methods:** First, the patients filled out two self-questionnaires regarding their disease severity and demographic data. Then, the patients were examined frontally by one doctor and virtually by the other through a video chat application ("Zoom"). After each assessment- both frontally and virtually, the assessing doctor filled out a questionnaire about the severity of the disease, which joints were involved in the disease in his opinion, and his medical recommendations. The doctors were blinded to each other's assessment. Finally, the patients filled out their level of satisfaction with the virtual evaluation.

**Results:** The average score of the patients' satisfaction with the virtual evaluation was 7.63±2.871[0-10] with a median score of 8.5. The mean score of their willingness to participate in the future in a combined medical follow-up done both f2f and virtually was 5.47±3.835 [0-10], with a median of 6. By average, the diseased joints diagnosed by the two doctors had a 68% match, with a standard deviation of 41.68%. The average score for the disease severity on a scale of 0- 10 given during the f2f and virtual assessment were 0.953±1.297[0-5] and 2.66±2.98[0-8] respectively p-0.64. The medical recommendations that were given by the frontal and virtual doctors had a 68.8% (22 patients) match between the two assessments. No statistical difference was found in the decision to start any kind of medical treatment between the two doctors, with p-value of 1. The decisions to increase dosage of current treatment, to start biological therapy and to start DMARD was correlated f2f and virtually (P-1 for all ). The recommendations of injection to the joint and decrease dosage of current treatment were each partially correspondent with p value of 0.47 and 0.43, respectively. The correlation of the total recommendation between the two doctors was measured with a kappa score of 0.56- "moderate agreement".

**Conclusion:** Our research demonstrates a high satisfaction rate with the virtual assessment among patients, with moderate agreement about joint assessment between virtual to F2F assessment.

**Patient Consent**

Yes, I received consent

**Disclosure of Interest**

None declared

## P009 Nutritional status and its relationship between disease activity, functional status, fatigue, quality of life and pain in Juvenile idiopathic arthritis

### B. C. Caglayan^1^, B. Basakcı Calık^2^, E. Gur Kabul^3^, G. Kılbas^4^, S. Yuksel^4^

#### ^1^Physiotherapy and Rehabilitation, Istanbul Okan University, Istanbul; ^2^Physiotherapy and Rehabilitation, Pamukkale University, Denizli; ^3^Physiotherapy and Rehabilitation, Usak University, Usak; ^4^Pediatric Rheumatology, Medical Faculty of Pamukkale University, Denizli, Türkiye

##### **Correspondence:** B. C. Caglayan

*Pediatric Rheumatology 2023*, **21(Suppl 2):**P009

**Introduction:** Nutritional impairment in Juvenile Idiopathic Arthritis (JIA) is common recognized problem which affects general well-being, disease control and growth (1,2).

**Objectives:** The aim of this study was to investigate the nutritional status and its relationship between disease activity, functional status, fatigue, quality of life and pain in JIA.

**Methods:** A total of 37 children and adolescents (22 female, 15 male), with a mean age of 13.05±2.63 years and diagnosed with JIA were included in the cross-sectional study. After collecting demographic data, the nutritional status was assessed using a questionnaire including the presence of daily milk and white/red meat consumption, weekly egg, legumes and daily liquid intake. Disease activity with Juvenile Artritis Disease Activity Score (JADAS),functional status with Childhood Health Assessment Questionnaire (CHAQ), quality of life with with Pediatric Quality of Life Inventory 3.0 Arthritis Module (PedsQL), fatigue with Pediatric Quality of Life Inventory Multidimensinal Fatigue (PedsQL-MF), pain with Numeric Pain Scale were evaluated. Independent Sample T test and Mann-Whitney U test were used to analyze the data, and bivariate analyses (Spearman or Pearson correlation) were performed.

**Results:** The mean of body mass index was 20.83 ± 4.38 kg/m^2^. Egg intake (weekly) had a low negative correlation with CHAQ-dressing (p=0.025; r=-0.369), CHAQ-eating (p=0.022; r=-0.379), and CHAQ-reach (p=0.008; r=-0.429) while a low positive correlation with PedsQL-daily activities (p=0.031; r=0.355) and PedsQL-MF-cognitive (p=0.036; r=0.346). Legume intake (weekly, portion) had a low negative correlation with PedsQL-MF-sleep (p=0.029; r=0.359), PedsQL-MF-cognitive (p=0.020; r=-0.381), and PedsQL-total score (p=0.026; r=-0.367) while a low positive correlation with CHAQ-overall well-being score (p=0.040; r=0.339). 73% (n=27) of participants consumed daily milk, and 51.4% (n=19) consumed white/red meat. JADAS and CHAQ-pain scores were significantly worse patients consuming milk compared to not (p=0.027, p=0.035, respectively). While PedsQL-treatment (p=0.020) was better patients consuming meat, PedsQL-MF-cognitive was worse (p=0.024) compared to not.

**Conclusion:** Nutrition status may have effects on disease activity, functional status in terms of pain and well-being, cognitive and sleep parameters of fatigue, and quality of life in children and adolescents with JIA.

**Patient Consent**

Yes, I received consent

**Disclosure of Interest**

None declared

**References**

1. Zare N, Mansoubi M, Coe S, Najafi AA, Bailey K, Harrison K, Sheehan J, Dawes H, Barker K. An investigation into the relationship between nutritional status, dietary intake, symptoms and health-related quality of life in children and young people with juvenile idiopathic arthritis: a systematic review and meta-analysis. BMC Pediatr. 2023 Jan 2;23(1):3. doi: 10.1186/s12887-022-03810-4.
2. Cleary AG, Lancaster GA, Annan F, Sills JA, Davidson JE. Nutritional impairment in juvenile idiopathic arthritis. Rheumatology (Oxford). 2004 Dec;43(12):1569-73. doi: 10.1093/rheumatology/keh387. Epub 2004 Oct 5. PMID: 15466896.

## P010 Multifactorial aspects of iga nephropathy in oligoarticular juvenile idiopathic arthritis: role of coeliac disease onset in a patient with ongoing adalimumab treatment

### S. Cataldi^1^, A. Omenetti^2^, B. Lattanzi^2^, L. Caponi^1^, G. Simona^1^, A. Ranghino^3^, S. Cazzato^2^

#### ^1^Department of Pediatrics, Marche Polytechnic University; ^2^Pediatric Unit, Department of Mother and Child Health; ^3^Nephrology, Dialysis and Renal Transplantation Unit, AOU Marche, Salesi Children’s Hospital, Ancona, Italy

##### **Correspondence:** S. Cataldi

*Pediatric Rheumatology 2023*, **21(Suppl 2):**P010

**Introduction:** Renal involvement was anecdotally reported in oligoarticular juvenile idiopathic arthritis (oligo-JIA). IgA nephropathy (IgAN) may occur during adalimumab (ADA) but it usually resolves following withdraw. JIA patients may present autoimmune comorbidity including coeliac disease (CD) which may be associated with IgAN

**Objectives:** To highlight potential aetiology of IgAN in oligo-JIA

**Methods:** We report a 13 years old boy affected by ANA+ oligo-JIA, who developed IgAN during ADA treatment

**Results:** The patient was diagnosed with oligo-JIA at the age of 3. He was initially treated with intra-articular steroids and s.c. methotrexate (MTX), followed by introduction of etanercept (ETN). Due to onset of relapsing uveitis, ETN was switched to ADA. Despite persistent JIA remission, he abruptly developed proteinuria and haematuria. Drug-induced renal damage was considered and ADA promptly discontinued. However, proteinuria and haematuria worsening occurred regardless ADA discontinuation. Renal biopsy was performed and unveiled mesangial IgAN. Unfortunately, ADA discontinuation resulted in ocular and joint relapses. In order to target kidney and JIA, systemic steroid regimen was started with prompt improvement of IgAN and JIA. However, at steroid tapering, both articular and ocular relapses occurred. Treatment was implemented (MTX+ tocilizumab) with JIA remission also at steroid withdraw. Autoimmune profiling was unremarkable except for unforeseen IgA-class tissue transglutaminase antibodies (tTGA) positivity with slight elevation of IgA in the presence of HLA-DQ2/DQ8. Elevation of tTGA was not confirmed at following assessments, not allowing a definitive serological CD diagnosis. Interestingly, following steroid suspension, CD indices gradually increased leading to indication of endoscopic examination. CD diagnosis was confirmed in March 2023 and the exclusion diet was started

**Conclusion:** We herein address the multifactorial putative causes underlying IgAN onset in JIA: 1) a relapsing disease course with required long-term biological regimen, suggests underlying disease severity; 2) the patient developed IgAN during ADA therapy, with proteinuria/haematuria worsening despite drug discontinuation, which weakens but not rules out the drug-induced hypothesis; 3) several evidence indicate a role for gut-renal connection in IgAN, and CD is part of the autoimmune clinical spectrum potentially associated with JIA. At present, is it not clear whether or not the ADA-induced IgAN reported is consequent to the drug itself or it may be due to the undergoing autoimmune disease for which the drug has been started. A strict surveillance of autoimmune comorbidity and proteinuria should be considered in JIA patient in need of ADA treatment, especially in presence of HLA-DQ2/DQ8 haplotype.

**Patient Consent**

Yes, I received consent

**Disclosure of Interest**

None declared

## P011 Predictors of adding biologic disease modifying antirheumatic drugs to the treatment of oligoarticular JIA patients on methotrexate

### M. C. Polat^1^, E. Çelikel^1^, Z. Ekici Tekin^1^, V. Güngörer^1^, M. Sezer^1^, T. Kurt^1^, M. M. Kaplan^1^, N. Tekgöz^1^, C. Karagöl^1^, S. Coşkun^1^, N. Öner^1^, S. Sezer^2^, B. Çelikel Acar^1^

#### ^1^Pediatric Rheumatology, Ankara Bilkent City Hospital; ^2^Rheumatology, Ankara Training and Research Hospital, Ankara, Türkiye

##### **Correspondence:** Çelikel Acar

*Pediatric Rheumatology 2023*, **21(Suppl 2):**P011

**Introduction:** Oligoarticular juvenile idiopathic arthritis (JIA) is the one of the most common chronic musculoskeletal childhood disorders. According to the International League of Associations for Rheumatology (ILAR) criteria, oligoarticular JIA is defined as chronic inflammatory arthritis of unknown etiology that begins before age 16 and lasts for more than six weeks. In general, patients with oligoarticular JIA have the best outcome among the JIA subtypes. However, untreated patients can also suffer a great burden of disease such as bone erosion, joint dislocation, ankylosis, disability, loss of function, and growth disturbances.Some researches have investigated into the predictive factors of the prognosis in JIA, but few have focused into the predictors of adding bDMARDs to the treatment.

**Objectives:** To compare the demographic and clinical characteristics of the groups with and without biological disease modifying antirheumatic drugs (bDMARDs) added to the treatment of oligoarticular juvenile idiopathic arthritis (JIA) patients using methotrexate (MTX) and also to determine the predictors of adding bDMARDs to treatment.

**Methods:** The patients with oligoarticular JIA were divided into two groups receiving MTX (n=77) and MTX plus bDMARDs (n=29). Predictors of adding bDMARDs were investigated by comparing demographic, clinical features and laboratory findings.

**Results:** A total of 106 children with oligoarticular JIA using MTX were included in this study. Gender, age at first diagnosis, duration of disease at the first visit, and disease duration were similar in both groups. The ankle (*p*=0.02), wrist (*p*=0.01) and proximal interphalangeal (*p*=0.02) involvement was higher in the MTX plus bDMARD group. There was no significant difference in erythrocyte sedimentation rate (ESR) and C-reactive protein level at the time of diagnosis (*p*=0.13, *p*=0.81, respectively). Multivariate analysis showed that predictive factors of adding of bDMARDs were extended oligoarticular JIA subtype (*p*=0.02), increased ESR (*p*=0.04) and presence of uveitis (*p*=0.004).

**Conclusion:** Extended oligoarticular JIA subtype, increased ESR, and uveitis were determined as predictors of adding bDMARDs.

**Patient Consent**

Not applicable (there are no patient data)

**Disclosure of Interest**

None declared

**Reference**s

1. Kahn PJ (2013) Juvenile idiopathic arthritis: what the clinician needs to know. Bulletin of the NYU Hospital for Joint Diseases 71(3):194-199.
2. Petty RE, Southwood TR, Manners P, Baum J, Glass DN, Goldenberg J, et al (2004) International League of Associations for Rheumatology classification of juvenile idiopathic arthritis: second revision, Edmonton, 2001. J Rheumatol 31(2):390-392.
3. Okamoto N, Yokota S, Takei S, OkuraY, Kubota T, Shimizu M, et al (2018) Clinical practice guidance for juvenile idiopathic arthritis (JIA) 2018.
4. Modern Rheumatology 29(1): 41-59. 10.1080/14397595.2018.1514724.Epub 2018 Oct 29. Prakken B, Albani S, Martini A (2011) Juvenile idiopathic arthritis. Lancet 377(9783):2138-2149.
5. Zaripova LN, Midgley A, Christmas SE, Beresford MW, Baildam EM, Oldershaw RA (2021) Juvenile idiopathic arthritis: from aetiopathogenesis to therapeutic approaches. Pediatric Rheumatology 19(1):135.

## P012 Psychological state in children with Juvenile idiopathic arthritis

### I. Chyzheuskaya^1^, L. Belyaeva^1^, A. Chyzhevskaya^2^, T. Matsushko^1^, A. Vishnevskaya^1^

#### ^1^4th City Children's Clinical Hospital; ^2^National Academy of Sciences of Belarus, Minsk, Belarus

##### **Correspondence:** I. Chyzheuskaya

*Pediatric Rheumatology 2023*, **21(Suppl 2):**P012

**Introduction:** Growing mental health problems are of great importance both for the pediatric rheumatological population and for society as a whole. The chronic course of the disease, pain syndrome, restriction of movements, frequent long-term hospitalizations are a powerful maladaptive factor for a child. These problems lead to a narrowing of the sphere of self-expression, self-realization, difficulties in the formation of social ties that are significant for the functioning and development of the individual, disruption of normal family life with a developing sense of dependence. Such circumstances accompany a range of psychological, emotional and social consequences of the disease.

**Objectives:** The purpose of the study is to assess the psychological state of children with juvenile idiopathic arthritis.

**Methods:** The study included 128 patients with various variants of juvenile idiopathic arthritis aged 5 to 17 years who were treated in the rheumatology department of the 4th city children's clinical hospital in Minsk. The following methods were used in the psychological examination: To diagnose the emotional state and the level of mental performance, M. Luscher's color test was used. Ch.D. Spielberger's questionnaire was used to assess personal and situational anxiety. To identify the individual psychological properties of the personality of the patient used the G.J. Eysenck questionnaire for adolescents, consisting of 60 questions including a scale of extra-, introversion, neuroticism and "lie scale". Testing of patients and interpretation of the results were carried out jointly with a psychologist.

**Results:** All patients of the clinical group according to the ILAR classification were distributed as follows: systemic variant occurred in 23 (17.9%) patients, oligoarticular variant in 80 (62.5%) patients, polyarticular variant in 11 (8.5%) patients, enthesitic arthritis in 15 (11.7%). The mean age of the patients was 11.8±3.2 years, with a mean disease duration of 4.7±2.1 years. In the course of the study of the emotional state of children, high values of the index of anxiety were revealed - 3.4±2.1. It should be noted that in children with incomplete clinical remission, the anxiety index is almost 2 times higher than in children with complete clinical remission. The average indicators of reactive and personal anxiety were significantly higher (Р<0.001) in children with JIA in comparison with indicators of reactive and personal anxiety in children of the control group (43.77±1.37 points for JIA and 27.6±0.62 points for the control group, respectively, reactive anxiety;37.84±1.27 points for JIA and 28.7±0.51 points for control group according to personal anxiety). According to the results of testing according to G.J. Eysenck, the majority of adolescents with JIA surveyed showed emotional instability (78%) and features characteristic of introversion (68%). According to G.J. Eysenck, high rates of introversion in combined with emotional instability correspond to a state of anxiety.

**Conclusion:** As a result of psychometric studies, it was found that children with JIA are characterized by emotional imbalance, anxiety, isolation, most of them suffer from interpersonal communication problems.

**Patient Consent**

Yes, I received consent

**Disclosure of Interest**

None declared

## P013 State of the intestinal microbiota in children with Juvenile idiopathic arthritis

### I. Chyzheuskaya^1^, L. Belyaeva^1^, T. Matsushko^1^, A. Vishnevskaya^1^, A. Chyzhevskaya^2^

#### ^1^4th City Children's Clinical Hospital; ^2^National Academy of Sciences of Belarus, Minsk, Belarus

##### **Correspondence:** I. Chyzheuskaya

*Pediatric Rheumatology 2023*, **21(Suppl 2):**P013

**Introduction:** Intestinal microbiota is crucial for the development of lymphoid tissues, as well as for the maintenance and regulation of intestinal immunity. It determines a person's health, his immune response to various unfavorable factors and the formation of a mechanism for the primary prevention of diseases. When the composition or function of the microbiota changes, dysbiosis develops. Dysbiotic conditions alter intestinal motility and permeability, as well as distort the immune response, thereby creating the prerequisites for the development of a pro-inflammatory state.

**Objectives:** The aim of the study was to assess the state of intestinal microflora in children with juvenile idiopathic arthritis.

**Methods:** 147 children aged from 2 to 17 years with juvenile idiopathic arthritis were examined in the rheumatology department of the 4th city children's clinical hospital in Minsk. The qualitative and quantitative composition of microflora in biotopes was determined in all patients and the results obtained were compared with the established norm.

**Results:** The analysis of the obtained results showed that disorders of the intestinal microflora were found in 109 (74.1%) of the examined patients. Dysbiotic shifts in most cases affected both aerobic and anaerobic components of the intestinal biocenosis. The most frequently revealed decrease in the total number of E. coli. In 47 (31.9%) patients with JIA, dysbiotic changes were accompanied by the release of opportunistic bacteria. Severe dysbiotic changes in the intestinal microflora with the presence of an association of several opportunistic bacteria were found in 6 (4.1%) patients with JIA. In 19 (12.9%) children with JIA, the amount of Staphylococcus aureus exceeded the allowable limit. In 22 (20.2%) children with JIA, fungi of the genus Candida were isolated in pathological amounts. Other opportunistic enterobacteria were found in 11 (10.1%) children with JIA. Dysbiotic changes in the intestinal microflora were not always accompanied by clinical signs of intestinal dysfunction. So, out of 128 children with JIA and normal stool, 76 of them had changes in the microflora of varying degrees.

**Conclusion:** Dysbiotic changes in children with JIA were characterized by disorders in the content of obligate microflora, among which, in most cases, there was a decrease in the total amount of E. coli, the presence of hemolytically active and lactose-negative strains of enterobacteria, an increase in the content of yeast-like fungi and Staphylococcus aureus. The severity of changes in the composition of the intestinal microflora in the examined patients did not depend on gender, age, and the presence of intestinal dysfunction. T he revealed dysbiotic states of the intestine indicate a systemic violation of the colonization resistance of the mucous membranes of the gastrointestinal tract in children with JIA, requiring their complex treatment with an effect on the body's immunoreactivity.

**Patient Consent**

Yes, I received consent

**Disclosure of Interest**

None declared

## P014 Long-term effectiveness of etanercept and adalimumab in Juvenile idiopathic arthritis

### S. Costi^1^, A. Amati^2^, S. Germinario^2^, C. Iannone^2^, M. R. Pellico^2^, P. Marco^2^, A. Marino^1^, R. F. Caporali^3,4^, C. B. Chighizola^1,3^

#### ^1^Pediatric Rheumatology, ASST-PINI-CTO; ^2^Rheumatology; ^3^Department of Clinical Sciences and Community Health, University of Milan, ^4^Rheumatology, ASST-PINI-CTO, Milan, Italy

##### **Correspondence:** S. Costi

*Pediatric Rheumatology 2023*, **21(Suppl 2):**P014

**Introduction:** Biological agents have dramatically changed the disease course in juvenile idiopathic arthritis (JIA). However, few long-term data are available.

**Objectives:** To investigate the long-term efficacy and safety of first-line therapy with etanercept (ETN) and adalimumab (ADA) as first-line biological therapy in JIA.

**Methods:** Clinical data of patients with longstanding JIA treated with ETN or ADA as first-line biotherapy were retrospectively collected. The retention rate of the two biological agents was estimated using the Kaplan-Meier method.

**Results:** Among the 100 patients analyzed (74% female), 51 received ETN and 49 ADA. 90% were oligo and polyarticular subcategories. Mean age at disease onset was 6 years (IQR 15.8), with a median follow-up time of 12 years (IQR 8). The median duration of treatment was 58 months (IRQ 45) for ETA and 46 (IQR 44) months for ADA (*p*= 0.224). Biological agents were associated with methotrexate in 92% (n=47) of patients receiving ENT and in 87% (n=43) of patients treated with ADA (*p*= 0.521). At the last follow-up, 85 (85.9%) patients were in remission on medication.

The overall retention rates of the two agents at 2, 5, and 10 years were 77%, 60%, and 34% respectively. Survival at 7 years was higher in ADA population (54% vs 31% in ETN; *p*= 0.24) with a lower rate of discontinuation after that time point. The hazard ratio for discontinuation was greater with ETN than ADA (2.026; 95% CI [1.086 to 3.781], *p*= 0.024). The median retention duration for ETN was 64 months (95% CI [68.0 to 108.0]). The estimated median survival was not reached for the ADA group. In 64 cases (64%), treatment courses were discontinued due to: uveitis activity (8%; 3 in ADA group and 5 in ETN group), secondary non-response (20%; 7 in ADA and 13 in ETN), adverse events (17%; 5 in ADA group and 12 in ETN), pregnancy wish (1%; 1 in ETN group) and sustained remission (15%; 5 in ADA and 10 in ETN).

Patients in the ETN group were more frequently treated with intraarticular joint injections compared to ADA group (12 vs. 8; *p*= 0.055).

**Conclusion:** Long-term treatment with ETN and ADA is effective for children with JIA. In this real-life cohort, the retention rate is high, with retention for ADA superior to that of ETN. Most patients keep their treatment over the years to maintain remission.

**Patient Consent**

Not applicable (there are no patient data)

**Disclosure of Interest**

None declared

## P015 C-reactive protein and erythrocyte sedimentation rate are not predictive of treatment response in Juvenile idiopathic arthritis

### J. B. de Jonge^1,2^, S. de Roock^1,2^, R. S. Yeung^3^, J. van Loosdregt^2^, S. J. Vastert^1,2^, S. M. Benseler^4,5^, J. F. Swart^1^ on behalf of on behalf of UCAN CAN-DU consortia

#### ^1^Department of Pediatric Rheumatology, Division of Paediatrics, University Medical Center Utrecht, Wilhelmina Children's Hospital; ^2^Center for Translational Immunology, University Medical Center Utrecht, Utrecht University, Utrecht, Netherlands; ^3^Division of Rheumatology, Department of Paediatrics, Immunology and Institute of Medical Science, The Hospital for Sick Children, University of Toronto, Toronto, Ontario; ^4^Alberta Children's Hospital Research Institute, University of Calgary; ^5^Division of Rheumatolgoy, Department of Pediatrics, Alberta Children's Hospital, Cumming School of Medicine, Univserity of Calgary, Calgary, Alberta, Canada

##### **Correspondence:** J. B. de Jonge

*Pediatric Rheumatology 2023*, **21(Suppl 2):**P015

**Introduction:** Juvenile Idiopathic Arthritis (JIA) is the most common chronic rheumatic disease during childhood. The clinical outcomes of JIA vary from patients reaching complete remission of medication to severe long-lasting symptoms and long-term use of anti-rheumatic drugs. Accurate prediction of the response to therapy may help avoid therapy failure and might prevent damage by optimally utilizing the window of opportunity to treat patients.

**Objectives:** To assess the efficiency of C-reactive protein (CRP) and erythrocyte sedimentation rate (ESR) as predictors of the treatment response in JIA patients six months following the start of various treatment strategies.

**Methods:** Consecutive children were identified from the prospective UCAN CAN-DU study, consisting of JIA patients (according to ILAR criteria) from Canada and the Netherlands. Selection criteria were: the presence of a baseline and follow-up (at six months ± 60 days) visit and the availability of CRP and/or ESR measurements at baseline. Systemic JIA patients were excluded because of the aberrant treatment strategy. Categorizing patients into being treatment naïve and starting JIA treatment for the first time (n = 131) and/or starting biological treatment for the first time regardless of previous medication (n = 140), resulted in a total of 245 inclusions. Successful treatment was defined as reaching an active joint count of zero at the 6-month follow-up without treatment intensification.

**Results:** Elevated baseline CRP (> 1mg/dl) and ESR (>20 mm/hr) levels in treatment naïve patients were not associated with an increased risk of treatment failure (risk ratio (RR) 1.16, 95% confidence interval (CI) 0.82-1.63; RR 1.13, CI 0.80-1.59, respectively). No association was found between treatment failure and elevated CRP and ESR levels determined in patients starting with biologicals either (RR 1.18, CI 0.76-1.84; RR 0.82, CI 0.48-1.40, respectively). A significant association between elevated baseline ESR levels and an increased risk for treatment failure was found when ESR levels were established within six months after symptom onset in treatment naïve patients (n = 66; RR 2.42, CI 1.27-4.59).

**Conclusion:** CRP and ESR are insufficient predictors of therapy response in JIA patients to act as a guide in therapeutic strategies and prevent therapy failure. Our findings underline the urgent need for biomarkers with a better prognostic value for treatment response in JIA.

**Patient Consent**

Not applicable (there are no patient data)

**Disclosure of Interest**

J. de Jonge: None declared, S. de Roock: None declared, R. Yeung Consultant with: Consulting fees from Novartis and Lily outside the submitted work, J. van Loosdregt: None declared, S. Vastert: None declared, S. Benseler: None declared, J. Swart: None declared

## P016 Proposal for a successful pediatric to adult rheumatologic transitional model

### L. De Nardi^1^, S. Pastore^2^, A. Taddio^2^, A. Tommasini^1,2^

#### ^1^University of Trieste; ^2^IRCCS Burlo Garofolo Trieste, Trieste, Italy

##### **Correspondence:** L. De Nardi

*Pediatric Rheumatology 2023*, **21(Suppl 2):**P016

**Introduction:** Transition from pediatric to adult care is an important process for patients with chronic diseases. Juvenile Idiopathic Arthritis (JIA) does not make exception and assuring a successful transition process is one of the main goal in JIA long-term management.

**Objectives:** This study aims to examine transition outcomes of a cohort of patients with JIA transitioned from pediatric to adult healthcare services at a single centre. We aim to report our transition experience, comparing our results with those existing in literature, and exploring possible correlations between disease relapses after transition and disease characteristics in pediatric age.

**Methods:** Pediatric patients with JIA who underwent a healthcare transition process from the Rheumatology Department of “Burlo Garofolo” Pediatric Institute, Trieste, to the adult Rheumatology Clinic of “Santa Maria della misericordia” Hospital, Udine, between 2017 and 2022, were consecutively recruited. For each patient the following variables were collected: age at transition, sex, age at onset, family history, number and type of involved joints, JIA type according to ILAR criteria, presence of enthesitis, tenosynovitis, uveitis, anti-nuclear antibody status and rheumatoid factor levels. Information about JADAS-27 score and therapies before and after transition process were also collected. A semi-structured survey exploring satisfaction rate of patients was distributed through email. Categorical variables were expressed as numbers (%) and compared by the χ^2^ test or Fisher’s exact test.

**Results:** 36 patients were recruited (26 F, 10 M): 9 polyarticular, 13 oligoarticular, 7 oligoarticular-extended, 8 psoriatic, 3 systemic JIA type and 3 enthesitis-related arthritis. Medium age at the transition was 18.6 years old (SD 1.03). JADAS-27 score values significantly decreased after transition process, with a mean difference of 2.6 (p= 0.014). No patients were lost to follow-up and in 8 out of 36 (22.2%) a step-up therapy was needed within the first 12 months. Among these 8 patients no correlation was found with JIA subtype, age at onset, type of involved joints and other variables explored. Finally, the 81.3% of patients who answered the online survey about transition experienced were satisfied, while the 18.7% declare they were not (response rate 50%).

**Conclusion:** This study proposes a transition model of care which showed good results in terms of outcome measures. However, further validated studies involving a larger cohort of patients are needed to improve the transition experience for patients with JIA.

**Trial registration identifying number:** Not available

**Patient Consent**

Not applicable (there are no patient data)

**Disclosure of Interest**

None declared

## P017 Study of a prevalent JIA cohort from a single centre in North India

### P. Dekate, M. Agarwal, S. Sawhney

#### Paediatric Rheumatology, Sir Ganga Ram Hospital, Delhi, India

##### **Correspondence:** P. Dekate

*Pediatric Rheumatology 2023*, **21(Suppl 2):**P017

**Introduction:** Juvenile Idiopathic arthritis (JIA) encompasses a heterogenous group of chronic inflammatory arthritides with a huge burden on healthcare. Data from the Indian subcontinent on this condition is scarce.

**Objectives:** To study clinical profile and disease outcome of JIA patients at a tertiary-level Paediatric Rheumatology centre.

**Methods:** Medical records of consecutive JIA patients from January 2006 who had visited the clinic at least twice, were studied. Demographics, JIA ILAR 2004^1^ subcategories and autoantibody profiles were analysed. Disease outcome was studied at the last follow-up using the Wallace criteria^2^.

**Results:** Of 1495 JIA patients, data from 1021 patients were analysed.

*Demographics*: 570 (56%) were males. The median age at symptom onset was 6 years, the youngest in OJIA subcategory at 2 years and the oldest with ERA at 10 years. The median age at diagnosis was 8 years with a median delay to diagnosis of 11 months.

*Subcategories*: ERA and SJIA were predominant subcategories with 35.4% and 29.6% patients respectively. PJA, OJIA and PsA were seen in 15.86%, 16.5% and 0.4% of patients respectively. 23 children (2.2%) had UJIA.

*Autoantibodies and HLA B 27:* 84 patients were RF or ACPA positive (8.2%). ANA positive in 20% of patients of the whole cohort. Maximum ANA positivity was seen in OJIA (65.5%) and PJIA (38.3%).HLA-B27 was positive in 280 (86.7%) patients with ERA (HLA B27 data missing for 39 patients).

*Uveitis:* Chronic anterior uveitis was observed in 87 (8.5%) patients. Acute anterior uveitis was seen in 34 (9.4%) ERA children.

*Outcome:* At the last follow-up (at a median of 2 years), 547 children (53.6%) had no active disease. Amongst these, 257 (25.2%) had inactive disease,265 (26%) attained clinical remission on treatment and 25 children (2.4%) were in clinical remission off treatment.SJIA (31.1%), PsJIA (50%) and ERA (28.7%) were the most common subcategories of JIA to have achieved remission on or off treatment. 474 (46.4%) patients had active disease, most commonly seen in OJIA (53.6%) and PJIA (49.4%) subcategories.

*Follow-up*: Of the total cohort of 1021 patients, 506 (49.5%) were lost to follow-up (not seen at this unit for > 18 months). Of the patients who currently followed at the unit, at a median follow-up of 3 years, 323 (62.7%) had no active disease, while 192 (37.3%) children continued to have ongoing disease activity.

**Conclusion:** To the best of our knowledge, this is the largest cohort of JIA from a single centre in India. This study showed a male preponderance with a median age of onset of 6 years. The median delay to diagnosis of the cohort was almost a year. Enthesitis-related arthritis is the commonest subcategory seen. Uveitis was predominant in the OJIA subcategory with ANA positivity. At a median follow-up of 2 years, more than half of our patients had no active disease. Half of the patients in our cohort were lost to follow-up.

**Patient Consent**

Yes, I received consent

**Disclosure of Interest**

None declared

**References**

1. Petty RE et al, The Journal of rheumatology. 2004 Feb;31(2):390-2
2. Wallace CA et al, The Journal of rheumatology. 2004 Nov 1;31(11):2290-4.

## P018 Should all patients with Juvenile Idiopathic Arthritis (JIA) undergo routine screening for Inflammatory Bowel Disease (IBD) at JIA onset?

### F. Di Domenico, G. Tarantino, A. Aquilani, E. Marasco, R. Nicolai, F. De Benedetti, S. Magni Manzoni

#### Rheumatology Division, IRCCS Bambino Gesù Children’s Hospital, Rome, Italy

##### **Correspondence:** F. Di Domenico

*Pediatric Rheumatology 2023*, **21(Suppl 2):**P018

**Introduction:** Some patients with Juvenile Idiopathic Arthritis (JIA) may develop Inflammatory Bowel Disease (IBD) along the disease course. It is unknown whether a systematic screening at JIA onset may allow early detection of subclinical IBD for prompt investigation and treatment.

**Objectives:** To describe demographic and clinical features in a single-center cohort of patient at JIA onset, grouped according to underwent IBD screening; to assess usefulness of routine IBD screening at JIA onset.

**Methods:** We performed a retrospective cohort study by reviewing the clinical charts of patients at JIA onset seen at the study center in a 4-years-period who were routinely screened for IBD through sequential fecal calprotectin determination and abdominal ultrasound. Demographical and clinical features, including IBD occurrence up to the last follow-up visit, were registered. The comparison group was represented by patients at JIA onset that, for any reason, did not undergo to the screening in the same period. Descriptive statistics was used for the analysis (STATA 15).

**Results:** Among a total of 148 patients (73.6% females) at JIA onset enrolled from March 2019 to January 2023, 91 had persistent oligoarthritis, 6 extended oligoarthritis, 40 RF-negative polyarthritis, 6 systemic JIA and 3 psoriatic. Antinuclear antibodies (ANA) were positive in half of the study cohort. Of them, 37 patients at JIA onset (27 females), with a median age of 3.7 years (IQR 2.2-6.9) underwent to routine IBD screening. The control group included 111 JIA patients (82 females), with a median age of 4.6 (IQR 1.9-9.5) at disease onset. In the study cohort, 8 (21.6%), 4 (10.8%), 0 (0%) patients presented pathologic fecal calprotectin at the 1^st^, 2^nd^ and 3^rd^ sampling, respectively; abdominal ultrasound revealed pathologic features (thickness of bowel loops) only in 2 (5.5%) children. None of them in both groups (screening and control), regardless from starting conventional and/or biological DMARDs treatment, developed clinically relevant IBD after a median follow up of 1.9 (IQR 1.1-3.1) years.

**Conclusion:** Evolving IBD is still a matter of interest in JIA patients. In a single center cohort of patients at JIA onset routine IBD screening could not provide added benefit in the early detection of subclinical IBD compared to patients without screening, due to the absence of IBD at the last follow up in both groups. Further studies with also screening of symptoms over time would potentially reveal more appropriate and applicable.

**Patient Consent**

Not applicable (there are no patient data)

**Disclosure of Interest**

None declared

**References**

1. Ferrara G. et al. “Fecal Calprotectin to Detect Inflammatory Bowel Disease in Juvenile Idiopathic Arthritis” J Rheumatol 2018; 45:1418-1421.
2. Barthel D. et al. “Inflammatory Bowel Disease in Juvenile Idiopathic Arthritis Patients Treated with Biologics” J Rheumatol 2015; 42:2160-5.

## P019 Association between c reactive protein to albumin ratio, neutrophil to lymphocyte ratio, platelet to lymphocyte ratio and disease activity in patients with Juvenile idiopathic arthritis

### G. Di Donato^1,2^, M. Attanasi^2^, D. M. d'Angelo^2^, S. La Bella^2^, C. Gentile^2^, A. Di Ludovico^2^, F. Lauriola^2^, P. L. Marulli^2^, F. Chiarelli^2^, L. Breda^1,2^

#### ^1^Pediatric Rheumatology Unit, SS Annunziata Hospital; ^2^Department of Pediatrics, University G. D'Annunzio, Chieti, Italy

##### **Correspondence:** G. Di Donato

*Pediatric Rheumatology 2023*, **21(Suppl 2):**P019

**Introduction:** Recent researches pointed out the role of new derivative indices (C reactive protein to albumin ratio-CAR, neutrophil to lymphocyte ratio-NLR, platelet to lymphocyte ratio-PLR) as biomarkers of disease activity in patients with rheumatic diseases, including rheumatoid arthritis (RA) [1-5].

**Objectives:** The first aim of our study was to investigate the role of CAR, PLR and NLR as potential markers of disease activity in patients with non-systemic JIA (nsJIA) and to investigate the relationship between CAR, PLR and NLR and the risk of flare or persistent disease activity during 18 months follow up.

**Methods:** We performed a prospective, cross-sectional study involving 130 nsJIA patients who referred to the Rheumatology Unit of the Department of Pediatrics, Chieti, Italy, from December 2019 to June 2022. Exclusion criteria were steroid therapy and/or infectious disease at enrollment time or in the previous 2 weeks and associated autoimmune diseases. Of the total 130 JIA patients, 74 had clinically active disease, while 56 had inactive disease according to Wallace criteria. Also, 62 healthy controls were included in the study. Clinical, demographic and laboratory data were collected for each patient at baseline (T0) and at 3 months (T1), 6 months (T2), 12 months (T3) and 18 months (T4) during follow up. Disease activity was evaluated through Juvenile Arthritis Disease Activity Score (JADAS-27).

**Results:** At baseline, CRP values showed a statistically significant difference between patients and controls (p=0.046), as did CAR (p=0.046), with higher levels in the study group, while no differences were found for NLR and PLR. However, CAR, NLR and PLR were not different between active patients, patients in remission and controls and they din not correlate with JADAS-27 in JIA patients. A GEE Model was used to investigate the association between CAR, NLR and PLR and relapse risk in inactive patients during 18 months follow up: CAR values at baseline resulted significant in predicting disease flare at 6 months (p=0.022). Besides, when considering the whole study group (both active and inactive patients), we found that CAR and NLR baseline levels were predictive of permanent disease activity at 6 months follow up (p<0.001).

**Conclusion:** CAR and NLR could indicate persistent disease activity in the brief term in nsJIA, thus influencing therapeutic decisions. Their predictive value might be increased by their combined use and by the evaluation of their trend over time during follow up.

**Trial registration identifying number:** Protocol MGB AIG N. 254 14/03/2017. Review Board of Chieti-Pescara University.

**Patient Consent**

Yes, I received consent

**Disclosure of Interest**

None declared

**References**

1. Sunar İ, Ataman Ş. Serum C-Reactive Protein/Albumin Ratio in Rheumatoid Arthritis and its Relationship With Disease Activity, Physical Function, and Quality of Life. Arch Rheumatol. 2020 Feb 7;35(2):247-253.
2. He Y, Tang J, Wu B, et al. Correlation between albumin to fibrinogen ratio, C-reactive protein to albumin ratio and Th17 cells in patients with rheumatoid arthritis. Clin Chim Acta. 2020 Jan;500:149-154.
3. Jin Z, Cai G, Zhang P, et al. The value of the neutrophil-to-lymphocyte ratio and platelet-to-lymphocyte ratio as complementary diagnostic tools in the diagnosis of rheumatoid arthritis: A multicenter retrospective study. J Clin Lab Anal. 2021 Jan;35(1):e23569.
4. Erre GL, Paliogiannis P, Castagna F, et al. Meta-analysis of neutrophil-to-lymphocyte and platelet-to-lymphocyte ratio in rheumatoid arthritis. Eur J Clin Invest. 2019 Jan;49(1):e13037.
5. Li W, Liu S, Chen C, et al. Neutrophil-to-lymphocyte ratios and platelet-to-lymphocyte ratios in juvenile systemic lupus erythematosus: correlation with disease manifestations. Ann Palliat Med. 2021 Sep;10(9):9406-9414.

## P020 Safety and efficacy of intra-articular corticosteroid injections in the hip joint in juvenile idiopathic arthritis

### F. Di Stasio^1,2^, A. Petaccia^2,3^, N. Tuzger^2,3^, C. Eboli^2,3^, G. Filocamo^2,3^, S. Lanni^2,3^

#### ^1^Università degli studi Milano Bicocca; ^2^Pediatric Immunorheumatology Unit, Fondazione IRCCS Ca’ Granda Ospedale Maggiore Policlinico; ^3^Università degli studi Milano, Milan, Italy

##### **Correspondence:** F. Di Stasio

*Pediatric Rheumatology 2023*, **21(Suppl 2):**P020

**Introduction:** Intra-articular corticosteroid injections (IACIs) are used in the management of juvenile idiopathic arthritis (JIA) to obtain rapid relief of symptoms through resolution of synovitis. This procedure is less frequently used for the hip compared to the other joints for the concern about a potential risk of inducing femoral head necrosis, particularly in children.

**Objectives:** This study aimed to report our experience on the efficacy and safety of IACIs of the hip joints performed in JIA patients.

**Methods:** This is an observational, retrospective, single-center study which includes JIA patients who underwent IACI of the hip joint between 2018 and 2023, followed at our center. Clinical data of patients were collected from medical records. All procedures were evaluated for efficacy and occurrence of post-procedural complications. The outcome was evaluatedby assessing the specific section of lower limbs of the Juvenile Arthritis Functionality Scale (JAFS-LL) reported on the Italian version of the Juvenile Arthritis Multidimensional Assessment Report (JAMAR). A specific questionnaire concerning resolution of pain and function of the hips was sent to the parents of children injected. When available, the radiographic assessment through Magnetic Resonance Imagine (MRI) was considered before and after the procedures.

**Results:** Six patients were enrolled for a total of 20 IACIs. All the IACIs were performed under ultrasound guidance. The majority of patients were males (n=5). Median age at disease onset and at the time of IACIs was 5,6 (3,3-7,4) years and 8,7 (8,3-19,1) years, respectively.

One patient had rheumatoid factor (RF) negative polyarticular JIA, two had RF positive polyarticular JIA, one had enthesitis-related arthritis and two had systemic JIA.

Patients with systemic JIA underwent multiple IACIs: one patient was injected seven times (three in the right hip and four in the left hip), and the other patient was injected eight times (four in the right hip and four in the left hip). The median time interval between the injections was 10 (8-11) months. Patients had ongoing treatment with DMARDs and biologic drugs. After IACIs all patients obtained rapid relief of symptoms. Only in one patient pain was documented after IACI of the hip.

The median score of PF-LL was 1 (0 – 2). Median follow-up time was 2,9 (1,7-3,4) years.

At present MRI was performed after the IACIs in three patients and showed the absence of new damage or progression of previous lesions. The MRI has been planned for the remaining patients in the next 3 months.

**Conclusion:** In our cohort of JIA patients, IACI of the hip joint resulted a safe procedure. This treatment was able to induce rapidly relief of pain in the treated joints and improved the physical function of lower limbs in patients with JIA.

**Patient Consent**

Yes, I received consent

**Disclosure of Interest**

None declared

## P022 Hormonal predictors in Juvenile idiopathic arthritis

### R. Eremciuc, N. Revenco, O. Gaidarji, A. Cracea, E. Nedealcova

#### Pediatric department, State University of Medicine and Pharmacy "Nicolae Testemitanu" from Republic of Moldova, Chisinau, Moldova, Republic of

##### **Correspondence:** R. Eremciuc

*Pediatric Rheumatology 2023*, **21(Suppl 2):**P022

**Introduction:** Chronic inflammatory conditions are often associated with growth failure, ranging from mildly decreased growth velocimetry to severe forms of short stature. Little is known about the association between juvenile idiopathic arthritis and hormonal dysfunction or autoimmune endocrine disease.

**Objectives:** The aim of this study was to identify corelations between hormonal profile and/or endocrine glands assessment with juvenile idiopathic arthritis characteristics in children.

**Methods:** 97 patients with a diagnosis of JIA according to the criteria of ILAR were included. Patients’ evaluation included baseline assessment and follow up on 6, 12 and 18 months. At baseline, the hypothalamic-pituitary-peripherical axis and autoimmune activity were assessed. The statistical analysis of the data was carried out descriptive analysis of variables, application of *t* and *z* test, Pearson, univariate ANOVA test. The confidence interval was 95%, and P values <0.05 were accepted as statistically significant.

**Results:** The average ageage of the investigated children was 10.66 years ± 4.53 years. The most frequent subtype of onset of JIA was the oligoarticular form in 44.33% of cases. Children with short stature, with z-score values <-1.5 SD were detected in 15.46% of cases. Although, in 41.24% low serum values of IGF1 were detected. On reverse, the serum values of IGF-BP3 were included in 43.30% cases higher than the 90th percentile. The Pearson test, indicates an intensely positive correlation between these 2 variables (r=0.84). Depending on disease activity, moderate negative correlation was established between DAS28 and IGF1 and IGF-BP3, respectively, only in the subgroup of children on long-term glucocorticosteroids (r=-0.23 for IGF1 and r=-0.36 for IGF -BP3). We found also correlation between oestrogens, testosteron, thyroid functional tests, thryroid estimated volumed and JIA characteristics like - disease duration, age at onset, global assessment by physician and/or parent, DAS28 and JADAS71 score of activity.

**Conclusion:** Children with juvenile idiopathic arthritis may develop hormonal dysfunction. Pediatric peculiarities, by applying age- and sex-specific reference ranges (percentiles or SDS), are important for the interpretation of hormone test measurements in children and adolescents. Screening of autoimmune pathology at the level of the endocrine glands, through laboratory tests, but also ultrasonographic evaluation in possible cases is necessary to be applied to children with JIA.

**Patient Consent**

Yes, I received consent

**Disclosure of Interest**

None declared

**References**

1. d’Angelo, D. M., Di Donato, G., Breda, L., & Chiarelli, F. (2021). Growth and puberty in children with juvenile idiopathic arthritis. In *Pediatric Rheumatology* (Vol. 19, Issue 1). BioMed Central Ltd. 10.1186/s12969-021-00521-5
2. Holmes, D. T., van der Gugten, J. G., Jung, B., & McCudden, C. R. (2021). Continuous reference intervals for pediatric testosterone, sex hormone binding globulin and free testosterone using quantile regression. *Journal of Mass Spectrometry and Advances in the Clinical Lab*, *22*, 64–70. 10.1016/j.jmsacl.2021.10.005
3. Yamada, S., Horiguchi, K., Akuzawa, M., Sakamaki, K., Yamada, E., Ozawa, A., Kobayashi, I., Shimomura, Y., Okamoto, Y., Andou, T., Andou, Y., & Yamada, M. (2023). The Impact of Age- and Sex-specific Reference Ranges for Serum TSH and FT4 on the Diagnosis of Subclinical Thyroid Dysfunction: A Multi-center Study from Japan. *Thyroid*. 10.1089/thy.2022.0567

## P023 Immunoprofiling of synovial fluid and plasma samples from oligo jia patients reveals distinct biomarker patterns during disease trajectory

### H. Erlandsson Harris^1,2^, H. Qu^2^, M. Neog^2^, K. Palmblad^3^, E. Sundberg^4^, E. Melén^5,6^, C. Aulin^2^

#### ^1^Clinical Science, University of Bergen, Bergen, Norway; ^2^Medicine, Karolinska Institutet; ^3^Pediatric Rheumatology, Karolinska Hospital; ^4^Women’s and Children’s Health; ^5^Clinical Sciences and Education, Karolinska Institutet; ^6^Sachs Children’s Hospital, Stockholm, Sweden

##### **Correspondence:** H. Erlandsson Harris

*Pediatric Rheumatology 2023*, **21(Suppl 2):**P023

**Introduction:** Immune profiling of clinically well-characterized samples is key to allow precision-based treatment of juvenile idiopathic arthritis (JIA); who, when and how to treat.

**Objectives:** To reveal immune mechanisms involved in the disease pathogenesis and their persistence over time we set out to define the immune profiles in synovial fluid and plasma samples from clinically well-characterized oligo JIA children.

**Methods:** Plasma and SF samples from 14 clinically well-described oligo JIA patients and twenty-eight age- and sex-matched healthy controls, were analysed by proximity extension assay (PEA), a highly sensitive proteomic immunoassay determining the presence of 92 inflammation-related biomarkers. Biomarkers being significantly up- or down-regulated in cross-sectional and paired analysis were defined and related gene ontology (GO) biological processes and Kyoto Encyclopedia of Genes and Genomes (KEGG) pathways were explored by STRING.

**Results:** The immune profiles of oligo JIA plasma samples and healthy control plasma samples were indistinguishable in a cross-sectional analysis, only MMP-1 was significantly upregulated in oligo JIA plasma. In a paired analysis of SF and plasma samples obtained two years apart from five different patients, in SF 19 biomarkers were significantly higher in the early phase of the disease course while in plasma only 4 biomarkers differed with disease duration. Pathway analysis indicated that chemotaxis was the main character defining the early phase of oligo JIA with a decreased strength in the persistent phase. Longitudinal analysis of 20 SF and 10 plasma samples from an individual patient revealed that immunosuppressive effects of methotrexate (MTX) treatment were evident in both plasma and SF with different kinetics.

**Conclusion:** Analysis of synovial fluid samples are more suited for disease immunoprofiling than plasma samples. Treatment efficiency of MTX could be followed in both synovial fluid and in plasma samples. Longitudinal analysis revealed that cell chemotaxis was significantly enriched in the early phase of the disease. Targeting chemokines could be a potential therapeutic option for achieving disease remission in the early phase.

**Patient Consent**

Yes, I received consent

**Disclosure of Interest**

None declared

## P024 New onset heart failure in adolescents with Juvenile idiopathic arthritis treated with anti-tnf-alpha biologic dmards

### K. Kourtesi^1^, S. Mavrogeni^2^, L. Fotis^1^

#### ^1^Department of Pediatrics, Division of Pediatric Rheumatology, National and Kapodistrian University of Athens, Attikon General University Hospital; ^2^Cardiology, Onasseio Cardiac Surgery Center, Athens, Greece

##### **Correspondence:** L. Fotis

*Pediatric Rheumatology 2023*, **21(Suppl 2):**P024

**Introduction:** TNF-alpha inhibitors safety profile regarding worsening or new onset of heart failure remains controversial with various publications presenting the risk of cardiovascular side effects and rapid improvement after drug discontinuation (1).

**Objectives:** Very limited data is available for children and adolescents regarding worsening or new onset heart failure following TNF-alpha inhibitor administration. Cardiac Echo has low sensitivity in detecting myocardial inflammation and cardiovascular magnetic resonance (CMR) could prove a more sensitive and reliable imaging method.

**Methods:** We report two cases of adolescents diagnosed with juvenile idiopathic arthritis (JIA), and treated with TNF-alpha inhibitors, who developed myocardial inflammation, confirmed by CMR.

**Results:** Patient 1, a 14-year-old female, was diagnosed with Juvenile Ankylosing Spondylitis and started on adalimumab (ADA) monotherapy. Three months later, she complained of new onset of intermittent chest pain, fatigue, and difficulty breathing during regular exercise. Cardiac enzymes were within normal limits, cardiac ECHO was normal. CMR provided evidence of myocardial inflammation. ADA was discontinued and secukinumab (SCN) 150 mg/week for 4 weeks followed by 150 mg/q4weeks was initiated along with losartan and bisoprolol, and a prednisolone taper for 3 months. Symptoms resolved and CMR 6 months later revealed myocardial inflammation resolution. Patient 2, a 15-year-old female, was diagnosed with RF (+) polyarticular JIA and was initially treated with methotrexate and a 3 month prednisolone taper. Etanercept (ETN) was added 6 weeks after stopping prednisolone. Nine months after starting (ETN) she mentioned fatigue and difficulty breathing during exercise. Cardiology evaluation with ultrasound revealed a reduced ejection fraction (30-35%) of the left ventricle. CMR showed mild pericardial effusion and evidence of inflammation. ETN was discontinued and she started on subcutaneous tocilizumab (TCZ) 162 mg/q2weeks, losartan, carvedilol and a 3 month prednisolone taper. Symptoms resolved and CMR 6 months later revealed myocardial inflammation resolution and improvement of cardiac function.

**Conclusion:** Switching treatment from TNF-alpha-inhibitors to other biologic DMARDs proved beneficial for the repair of myocardial inflammation in adolescent patients with JIA. These two cases are the first reported on the beneficial effect of SCN and TCZ on myocardial inflammation and heart failure, induced by ADA and ETN treatment respectively in adolescents. CMR is a reliable method to identify early myocardial involvement and perform accurate cardiac disease monitoring, as cardiac echo has low sensitivity in detecting myocardial inflammation.

**Patient Consent**

Yes, I received consent

**Disclosure of Interest**

None declared

**Reference**

1. Page RL, O’Bryant CL, Cheng D, MD, Dow TJ, Ky B, Michael Stein CM, Spencer AP, PharmD, Trupp RJ, Lindenfeld JA. Clinical Statements and Guidelines - AHA Scientific Statement. Drugs That May Cause or Exacerbate Heart Failure A Scientific Statement From the American Heart Association. Circulation 2016; 134(6):e32-e69

## P025 Treatment and outcome of patients with Juvenile idiopathic arthritis – results from a tertiary care center in Freiburg, Germany 2008 – 2018

### M. Freudenhammer^1^, P. Drees^2^, M. Hufnagel^1^

#### ^1^Center for Pediatrics and Adolescent Medicine; ^2^Medical Center University of Freiburg, Freiburg, Germany

##### **Correspondence:** M. Freudenhammer

*Pediatric Rheumatology 2023*, **21(Suppl 2):**P025

**Introduction:** In recent years, the concept of a "window of opportunity", i.e., early and aggressive of juvenile idiopathic arthritis (JIA) has become a matter of scientific interest.

**Objectives:** Analysis of a real-life JIA cohort from a single tertiary care center in Germany to identify factors potentially influencing the outcome

**Methods:** Retrospective analysis of demographic and clinical data from patients with a definite diagnosis of JIA (according to ICD-10 code) who were taken care for in the outpatient clinic for pediatric rheumatology in Freiburg, Germany, between 2008 and 2018. Data were collected from the medical records using a standardized questionnaire.

**Results:** 334 Patients with different subtypes of JIA were included in the study (sJIA 11,1%, oJIA 49,2%, pJIA 19,8%, EAA 13,8%, PsA 5,7%, undifferentiated JIA 1,5%). Mean time to diagnosis was 2,0 months (IQR 1,0-6,0). 68% of patients were treated with MTX, 34% received biologic DMARDS. The treatment target of a controlled disease activity (i.e., inactivity or remission) was reached in 63,5% of patients at the final visit of the study period (mean time of treatment of 5,2 years [IQR 1,7-10,1]). Clinical inactivity was at least transiently reached in 81,4% patients after a mean treatment duration of 9,6 months (IQR 4,8-21,6). In patients with a diagnosis before 2008, median duration from diagnosis to first clinical inactive disease was longer compared to patients diagnosed after 2008 (16,0 vs 9,1 months, p=0,0001), although use of oral glucocorticoids declined (40,3% vs. 22,8%, p=0,0001). In patients with JIA-associated uveitis who had a relapse during treatment with adalimumab, more than half had evidence of anti-drug antibodies, of which 82% did not take co-medication with MTX.

**Conclusion:** An early diagnosis and a prompt initiation of targeted treatment allows reaching inactive disease in high percentage of patients within nine months. Patients on adalimumab should be encouraged to take co-medication with MTX to prevent development of anti-drug antibodies and subsequent relapses.

**Patient Consent**

Not applicable (there are no patient data)

**Disclosure of Interest**

None declared

## P026 Screening for temporomandibular joint involvement and uveitis in a newly diagnosed JIA cohort

### A. Gamba^1^, A. I. Rebollo-Giménez^2^, S. M. Orsi^1^, M. Burrone^1^, P. Camicione ^3^, L. Anfigeno^4,5^, M. B. Damasio^4^, C. Malattia ^1,2^, S. Viola^2^, M. Gattorno^2^, A. Consolaro^1,2^

#### ^1^Dipartimento di Neuroscienze, Riabilitazione, Oftalmologia, Genetica e Scienze Materno-Infantili (DiNOGMI), Università degli Studi di Genova; ^2^UOC Reumatologia e Malattie Autoinfiammatorie; ^3^UOC Oculistica; ^4^UOC Radiologia, IRCCS Istituto Giannina Gaslini; ^5^Dipartimento di Scienze della Salute (DISSAL), Università degli Studi di Genova, Genoa, Italy

##### **Correspondence:** A. Gamba

*Pediatric Rheumatology 2023*, **21(Suppl 2):**P026

**Introduction:** In Juvenile Idiopathic Arthritis (JIA), temporomandibular joint (TMJ) involvement and chronic iridocyclitis still represent a major source of long-term damage and reduced life quality health.

**Objectives:** To study the frequency of TMJ involvement and chronic anterior uveitis among patients affected by JIA, together with the investigation of clinical characteristics and impact of the early JIA management on patient prognosis.

**Methods:** We included in the study the clinical charts of consecutive patients with a non-systemic JIA diagnosis, visited in our center in the first six months from disease onset between January 2018 and April 2019 with at least 4 years of follow-up. Only patients who received the first treatment for arthritis at the study Unit were included. TMJ involvement was assessed by magnetic resonance imaging (MRI). JIA-associated uveitis was recorded by the ophthalmologist at regular follow up visits.

**Results:** A total of 49 patients (73.5 % females, median age at JIA onset 4.6 years) with a median disease duration of 4.3 years were included in the study. The JIA category distribution was: 53.1% oligoarticular arthritis, 42.9% polyarticular arthritis and 4.1% enthesitis-related arthritis. In the first six months of disease course, 85.7 % of children received intraarticular corticosteroid injections (IACIs), 57.1% methotrexate, 14.2% biologic DMARDs. TMJ involvement was radiologically identified in 15/49 patients (30.6%) at a median age of 8 years and it was more frequent in the polyarthritis category (66.7 %). No difference was observed in the frequency of TMJ involvement between children receiving any systemic treatment in the first 6 months (37.5%) and those receiving only IACIs (17.6%) (p = 0.12). Out of 7 patients receiving anti-TNF treatment in the first 6 months of the disease (57.1%). Uveitis was found in 12.2% (6/49) of the patients, 83.3% of whom had oligoarthritis. No difference was observed in the frequency of uveitis between children receiving any systemic treatment in the first 6 months (12.5%) and those receiving only IACIs (11.8%) (p = 0.96).

**Conclusion:** TMJ involvement was common in the first 5 years of disease course in this small cohort of JIA children, whereas the frequency of uveitis was surprisingly low. Early treatment with systemic medication and early biologic treatment initiation did not seem to protect JIA patients from these potentially severe complications.

**Patient Consent**

Not applicable (there are no patient data)

**Disclosure of Interest**

A. Gamba: None declared, A. Rebollo-Giménez: None declared, S. Orsi: None declared, M. Burrone: None declared, P. Camicione : None declared, L. Anfigeno: None declared, M. Damasio: None declared, C. Malattia : None declared, S. Viola: None declared, M. Gattorno: None declared, A. Consolaro Grant / Research Support with: Prof. A. Consolaro received honoraria for lectures, presentations, speakers bureaus, manuscript writing or educational events from Pfizer and Abbvie. Prof. A. Consolaro also received grants for investigator-initiated research projects from Pfizer and Alfa Sigma.

## P027 Additive biologic treatment for temporomandibular arthritis in patients with Juvenile Idiopathic Arthritis (JIA)

### M. Glerup^1^, C. J. Kellenberger^2^, A. Küseler^3^, C. Høst^1^, T. K. Pedersen^3,4^, T. Herlin^1^, P. Stoustrup^3^

#### ^1^Department of Paediatric and Adolescent Medicine, Aarhus University Hospital, Aarhus, Denmark; ^2^Department of Diagnostic Imaging, and the Children's Research Center , University Children's Hospital Zürich, Zürich, Switzerland; ^3^Section of Orthodontics, Aarhus University; ^4^Department of Oral and Maxillofacial Surgery, Aarhus University Hospital, Aarhus, Denmark

##### **Correspondence:** M. Glerup

*Pediatric Rheumatology 2023*, **21(Suppl 2):**P027

**Introduction:** The Temporomandibular Joint (TMJ) is one of the most commonly involved joints in JIA affecting around 30-40% of patients and still there are no randomized controlled studies to support the clinicians in their clinical decision making. Hence, much uncertainty still exists about the effect of disease modifying anti-rheumatic (DMARD) treatment of TMJ arthritis and dentofacial growth disturbances.

**Objectives:** The aim of this study is to investigate the efficacy of biologics in combination with methotrexate or leflunomide on TMJ arthritis measured by: 1) Orofacial symptoms and dysfunctions during the two years of systemic treatment, 2) MRI-verified additive inflammation score, 3) Additive deformity score.

**Methods:** This prospective, longitudinal single center cohort study was based on data from 16 consecutive patients diagnosed with MRI verified TMJ arthritis, that were included at the Department of Pediatric and Adolescent Medicine, Aarhus University Hospital, Denmark between September 2018 and May 2020. Alongside, all patients had standardized, longitudinal orofacial examinations performed at the Regional Specialist Craniofacial Clinic, Section of Orthodontics, Aarhus University. Arthritis level was scored by a radiologist, blinded to the clinical data.

Inclusion criteria were: 1) diagnosis of JIA according to the International League of Associations for Rheumatology (ILAR) criteria, 2) MRI verified TMJ arthritis leading to initiation of anti-TNFi (in either DMARD naïve children or as an addition to methotrexate or leflunomide already prescribed prior to the MRI), 3) an MRI 6 and 24 months after initiation of anti-TNFi 4) clinical follow-up after the MRI by a pediatric rheumatologist and an orthodontist.

Exclusion criteria: 1) Previous TMJ steroid injection or need for injection during the follow-up time 2) Previous orthognathic surgery 3) Trauma, syndromes or comorbidities that potentially could affect the dentofacial growth.

**Results:** Of the 16 patients, 89% were females, median age at first MRI was 13 years (IQR 11.4-16.1),

median disease duration was 8 years (IQR 3.4-11.1) and 7 (44%) received MTX (n=5)/leflunomide (n=2) when TMJ arthritis was diagnosed. During follow-up, the number of patients with pain on movement decreased significantly (p=0.005) and additionally, the number of patients with reduced translation of the MJ condyle decreased (p=0.046). The TMJ additive inflammation score decreased significantly from median 4(IQR 1.5-5.0) at baseline to median 1(IQR 0-2) at the 2-year follow-up (FU), p<0.001. The mandibular ramus height increased by a median of 1.8 mm from the 1^st^ to the 3^rd^ MRI (IQR 0.1-3.25mm), p=0.002. The additive deformity score increased insignificantly (p=0.1) from median 2 (IQR 1-3) at baseline to 3 (IQR 0.5-3) at the 2-year FU.

**Conclusion:** This is the first longitudinal, prospective study to show that the additive inflammation score in TMJ arthritis and the related orofacial signs and symptoms can be reduced by treatment with anti-TNFi while maintaining normal mandibular ramus growth.

**Patient Consent**

Yes, I received consent

**Disclosure of Interest**

None declared

## P028 Juvenile idiopathic arthritis in infants and toddlers

### V. Gungorer^1^, N. Öner^1^, E. Çelikel^1^, Z. Ekici Tekin^1^, N. Tekgöz^1^, M. Sezer^1^, C. Karagöl^1^, S. Coşkun^1^, M. M. Kaplan^1^, M. C. Polat^1^, Ö. Aydemir^2^, B. Çelikel Acar^1^

#### ^1^Pediatric Rheumatology, Ankara Bilkent City Hospital, Ankara; ^2^Department of Public Health, Konya Meram Directorate of Health, Konya, Türkiye

##### **Correspondence:** B. Çelikel Acar^1^

*Pediatric Rheumatology 2023*, **21(Suppl 2):**P028

**Introduction:** Juvenile idiopathic arthritis (JIA) is the most common cause of idiopathic inflammatory chronic arthritis in childhood and consists of a heterogeneous group of diseases occurring with arthritis that lasts longer than 6 weeks and is seen under 16 years of age. Depending on the number of joints involved in the first 6 months and the presence of extra-articular symptoms, it is divided into seven categories according to the International League of Associations for Rheumatology (ILAR) criteria. Although the general characteristics of the subgroups are well known, there is a lack of age-oriented studies in the literature. Since JIA is most often seen in children over 2 years of age, data are more limited regarding early-onset JIA.

**Objectives:** The aim of the study is to present the demographic, clinical features, and laboratory findings of juvenile idiopathic arthritis (JIA) patients aged <3 years, by grouping them as infant and toddler.

**Methods:** Patients diagnosed with JIA who were younger than 3 years of age were included in the study. The patients were divided into two age groups as infants and toddlers. Descriptive, clinical, and laboratory characteristics of the patients were reviewed retrospectively.

**Results:** A total of 81 patients diagnosed under <3 years of age were included in the study. Of these, 66 (76.7%) were toddlers and 15 (17.4%) were infants. Oligoarticular JIA (oJIA) was found to be higher in toddlers than infants (*p*=0.004). The rate of rheumatoid factor negative (RF-) polyarticular JIA (pJIA) was higher in infants compared to toddlers, but no significance was found. The median time from symptom to diagnosis was 2 months in infants and 3 months in toddlers, and this period was longer in infants (*p*=0.001). The rate of development of polyarticular involvement, hepatosplenomegaly and lymphadenopathy was higher in infants compared to toddlers (respectively, *p*=0.002, *p*=0.020, *p*=0.035). In laboratory examination, only erythrocyte sedimentation rate (ESR) was different between the two groups, and it was higher in infants (*p*=0.008).

**Conclusion:** Depending on age, different subtypes of JIA may be prominent. In the infantile period, polyarticular involvement and higher ESR draw attention while, in toddlers, the oJIA subtype is dominant.

**Patient Consent**

Not applicable (there are no patient data)

**Disclosure of Interest**

None declared

**Reference**s

1. Momah T, Ray L. Juvenile idiopathic arthritis: Old disease, new tactics. J Fam Pract. 2019; 68: 8-13.
2. Martini A, Lovell DJ, Albani S, et al. Juvenile idiopathic arthritis. Nat Rev Dis Primers 2022; 27: 5.
3. Savolainen E, Kaipiainen-Seppänen O, Kröger L, et al. Total incidence and distribution of inflammatory joint diseases in a defined population: results from the Kuopio 2000 arthritis survey. The Journal of rheumatology 2003; *30*: 2460–2468.
4. Thierry S, Fautrel B, Lemelle I, et al. Prevalence and incidence of juvenile idiopathic arthritis: a systematic review. Joint bone spine 2014; 81: 112–117.
5. Petty RE, Southwood TR, Manners P, et al. International League of Associations for Rheumatology classification of juvenile idiopathic arthritis: second revision, Edmonton. J Rheumatol. 2004; 31: 390-392.
6. Russo RA, Katsicas MM. Patients with very early-onset systemic juvenile idiopathic arthritis exhibitmore inflammatory features and aworse outcome. J Rheumatol 2013; 40: 329–

## P029 Long-term immunoprotection after live attenuated measles-mumps-rubella booster vaccination in children with Juvenile idiopathic arthritis

### M. Hamad Saied^1,2^, J. W van Straalen^1^, M. jansen^1^, N. Wulffraat^1^, J. swart^1^, S. Roock^1^, G. Joode-Smink^1^

#### ^1^Pediatric Immunology and Rheumatology, University Medical Center Utrecht, Utrecht, Netherlands; ^2^Pediatric and Rheumatology, Technion Faculty of MedicineTechnion Faculty of Medicine, haifa, Israel

##### **Correspondence:** M. Hamad Saied

*Pediatric Rheumatology 2023*, **21(Suppl 2):**P029

**Introduction:** Vaccines, especially live attenuated vaccines, in children with JIA pose a great challenge due to both potential lower immunogenicity and safety as a result of immunosuppressive treatment. For many years, in the Netherlands, JIA patients receive a measles-mumps-rubella (MMR) booster vaccine at the age of nine years as part of the national immunization program.

**Objectives:** To study long-term humoral immunoprotection in a large cohort of JIA patients who received the MMR booster vaccine while being treated with immunomodulatory therapies at the Wilhelmina Children’s Hospital in Utrecht, the Netherlands.

**Methods:** MMR-specific IgG antibody concentrations in stored serum samples of vaccinated JIA patients were determined with chemiluminescent microparticle immunoassays (CMIA). Samples were analyzed five years after MMR booster vaccination and at last available follow-up visit using both crude and adjusted analyses. Additional clinical data were collected from electronic medical records.

**Results:** In total, 236 samples from 182 patients were analyzed, with a median duration between vaccination and last available visit of 6.9 years (IQR: 2.8 – 8.8). Twenty-eight patients were using bDMARDS of whom 96% anti-TNF agents and 4% tocilizumab. Percentages of protective antibody levels against measles after five years were significantly lower for patients who used bDMARD therapy at vaccination compared to patients who did not: 60% versus 86% (*P* = 0.03). For mumps (80% versus 94%) and rubella (60% versus 83%) this difference did not reach statistical significance (*P* = 0.11 and *P* = 0.07, respectively). Antibody levels post-vaccination decreased over time, albeit not significantly different between bDMARD users and non-bDMARD users.

**Conclusion:** The MMR booster vaccine demonstrated long-term immunogenicity in the majority of children with JIA from a large cohort, although lower percentages of protective measles antibody levels were observed in bDMARD users. Hence, it might be indicated to measure antibody levels at least five years after MMR booster vaccination in the latter group and advice an extra booster accordingly.

**Trial registration identifying number:** Patients were included from the ongoing observational Pharmachild register. Pharmachild obtained approval from the Institutional Review Board of the University Medical Center Utrecht (11-499c) and is carried out in accordance with the Declaration of Helsinki. All patients provided written informed consent/assent.

**Patient Consent**

Yes, I received consent

**Disclosure of Interest**

None declared

## P030 High rheumatoid factor does not diminish efficacy of TNF inhibitors in seropositive JIA

### B. Hügle, J.-P. Haas

#### German Center for Pediatric and Adolescent Rheumatology, Garmisch-Partenkirchen, Germany

##### **Correspondence:** B. Hügle

*Pediatric Rheumatology 2023*, **21(Suppl 2):**P030

**Introduction:** Rheumatoid-factor positive or seropositive polyarthritis is a category of juvenile idiopathic arthritis (JIA) and considered the juvenile form of rheumatoid arthritis (RA). TNF inhibitors (TNFi) are frequently used to treat seropositive polyarthritis and RA.

Rheumatoid factor (RF) is an IgM autoantibody against the Fc portion of immunoglobulin G, which is present in the majority of TNFi. High RF titers are a poor prognostic factor for RA. Because RF can bind to the immunoglobulin Fc portion, it might bind the Fc portion of certain TNFi and influence their clinical efficacy. The latter has been shown in studies of adults with RF positive RA, but not yet in children.

**Objectives:** The aim of this study was to determine efficacy of TNFi in children with seropositive polyarthritis according to their rheumatoid factor levels.

**Methods:** The database of the German Center for Pediatric and Adolescent Rheumatology was searched for patients with JIA, category seropositive polyarthritis, admitted between Januar 2019 and March 2023. Patients were included if they started a TNFi during that time and had at least one assessment within 3 to 12 months after starting medication. Patients were excluded if they had any other disease or factor that would influence rheumatoid factor levels. Data collected was age at time of diagnosis, RF at time of diagnosis, anti-CCP-antibodies at time of diagnosis, other drugs given at the time of start of TNFi, age at the start of TNFi treatment and JADAS and cJADAS prior to and after start of TNFi treatment. Data was analyzed using descriptive statistics, and changes in JADAS and cJADAS on TNFi were compared between patients with RF < 150 U/ml and RF ≥ 150 U/ml using repeated measures ANOVA.

**Results:** 40 patients with seropositive polyarthritis were identified, of which 17 were included, 8 with RF < 150 U/ml at diagnosis, and 9 with RF ≥ 150 U/ml, median age at diagnosis 14.6 years (range 7.3 – 15.7 years). Mean RF was 84.9±40.7 U/ml for the low RF group, and 263.4±64.1 U/ml for the high RF group; anti-CCP antibodies levels were 132.0±137.3 U/ml and 239.1±169.6 U/ml, respectively. 16 patients (94%) were treated with etanercept, and one with golimumab. 15 patients were additionally treated with methotrexate. Median time between assessments was 135 days (range 95 – 364 days).

Median age at start of TNFi was 14.7 years (range 7.4 – 17.7 years). Mean JADAS (cJADAS) at treatment start was 26.0 ± 16.9 (24.4±13.7), and 5.5 ± 6.0 (5.1±5.7) at time of assessment after starting TNFi. A repeated-measures ANOVA determined that mean JADAS and cJADAS scores did not differ significantly across the two time points (JADAS: F(1, 16) = 1.901, p = .188, cJADAS: F(1, 16) = 1.050, p = .327).

**Conclusion:** Unlike in adults, efficacy of TNFi was not diminished by elevated levels of RF in this cohort of pediatric patients with seropositive polyarthritis. Further studies are necessary to confirm these findings in a larger cohort of children with seropositive JIA.

**Patient Consent**

Not applicable (there are no patient data)

**Disclosure of Interest**

None declared

## P031 Clinical outcome of methotrexate treatment in JIA

### V. Iacomi, N. Revenco

#### SUMPh "Nicolae Testemitanu", Chisinau, Moldova, Republic of

##### **Correspondence:** V. Iacomi

*Pediatric Rheumatology 2023*, **21(Suppl 2):**P031

**Introduction:** The current widely accepted criteria to appreciate an improvement in patient disease evolution in response to a therapeutic intervention for juvenile idiopathic arthritis (JIA) are the American College of Rheumatology Pediatric response criteria developed in 1997. The genetic polymorphisms of the MTHFR gene are as well considered a novel scientific background for improvement outcome difficulties.

**Objectives:** To assess the relationship between MTHFR gene polymorphisms in JIA patients using methotrexate and the ACR Pedi 30% Index.

**Methods:** An observational case-control study involved 68 patients using methotrexate for JIA treatment. The genetic polymorphism was tested through Polimerase Chain Reaction in Real Time and the appreciation of improvement was assessed after 24 weeks from treatment onset.

**Results:** There has been examined 68 children, in whom the genetic testing revealed 23 (33,8%) cases of MTHFR combined C677T/A1298C (9 (39,1%)) and T677T (14 (60,9%)), and 45 (66,2%) cases of no mutations samples. The gender distribution was 37 (54,4%) girls and 31 (45,6%) boys with a mean age of 133,8 months CI 95% [0,81-0,97]. From the sample in which genetic polymorphism was found, only 2 (8,7%) children achieved low activity or disease remission, compared to 24 (53,3%) children from the mutation free sample, according to ACR Pedi 30% (χ^2^=12,842, p=0,0001).

**Conclusion:** There has been determined a significant relationship between the MTHFR genetic background and the methotrexate response assessment over ACR Pedi 30% Index use in children with JIA.

**Patient Consent**

Yes, I received consent

**Disclosure of Interest**

None declared

## P032 Clinical features of children with juvenile idiopathic arthritis in Nicaragua

### K. V. Jirón Mendiola

#### Pediatrics, Vivian Pellas Hospital, Managua, Nicaragua

##### **Correspondence:** K. V. Jirón Mendiola

*Pediatric Rheumatology 2023*, **21(Suppl 2):**P032

**Introduction:** Juvenile idiopathic arthritis (JIA) is a term of unknown etiology persisting for at least 6 weeks with a clinical onset before 16 years of age. The disease spectrum spans from self-limited oligoarthritis to ongoing multiple joints destruction, and may involve severe systemic manifestations or sight-threatening uveitis. In order to provide a better quality on medical treatment and follow-up, updated knowledge of the epidemiology, clinical features, and course of JIA is essential. This is the first study of our country using the International League of Associations for Rheumatology (ILAR) classification criteria for childhood-onset idiopathic inflammatory arthritis and called it JIA, with new patients.

In view of this, our aim was to describe the clinical manifestations, disease, and treatment course of JIA for a start in our country Nicaragua.

**Objectives:** To describe the clinical manifestations, disease, and treatment course of JIA for a start in our country Nicaragua.

**Methods:** An observational, cross-sectional study was carried out, analyzing the clinical records of patients diagnosed with inflammatory myopathies, with follow-up in the Pediatric Rheumatology Section of the Vivian Pellas Hospital. General patient data, age at diagnosis, clinical and laboratory criteria at diagnosis, Rheumatoid Factor and other antibodies, as well as complementary studies performed, are recorded in order to describe the characteristics found.

**Results:** Out of 64 children with chronic joint pain, 54 were diagnosed as JIA: oligoarthritis (persistent 14.8%;extended 29.6%),polyarthritisrheumatoid factor-negative(37%),polyarthritisrheumatoidfactor-positive(11.1%),psoriaticarthritis (1.8%),enthesitis-relatedarthritis(ERA;7.4%),and undifferentiated arthritis (3.7%). Antinuclear antibodies(27.7%)

Uveitis was observed in 1.8% of patients. Disease-modifying anti rheumaticdrugs, including biologic medications,were usedin 100% of children during the observational period. At the last follow-up, 5.5% of patients experienced a continuously active or relapsing course.

**Conclusion:** This is the first study in our country as a start to continue increasing research in our population. The clinical presentation of patients has a defined variability in terms of antibody positivity, their identification allows the intentional search for complications for timely treatment and improvement of the patient's quality of life and achieve remission.

**Patient Consent**

Yes, I received consent

**Disclosure of Interest**

None declared

## P033 Trend of treatments for articular juvenile idiopathic arthritis compared with rheumatoid arthritis in adolescent and young adult using the epidemiological receipt database

### T. Kawabe^1^, R. Sakai^2^, E. Tanaka^3^, E. Inoue^4^, Y. Inoue^5^, Y. Inoue^5^, T. Miyamae^1^, A. Moriichi^6^

#### ^1^ Pediatric Rheumatology, Institute of Rheumatology, Tokyo Women’s Medical University School of Medicine, Tokyo; ^2^Department of Public Health and Epidemiology, Meiji Pharmaceutical University, Kiyose; ^3^Division of Rheumatology, Department of Internal Medicine, Institute of Rheumatology, Institute of Rheumatology, Tokyo Women’s Medical University School of Medicine; ^4^Showa University Research Administration Center, Showa University, Tokyo; ^5^Department of General Medical Science, Graduate School of Medicine, Chiba; ^6^Department of Specific Pediatric Chronic Disease Information, National Center for Child Health and Development, Tokyo, Japan

##### **Correspondence:** T. Kawabe

*Pediatric Rheumatology 2023*, **21(Suppl 2):**P033

**Introduction:** There exist some disparities in the medical management of articular juvenile idiopathic arthritis (aJIA) and rheumatoid arthritis (RA). Both diseases have chronic arthritis as their primary pathogenesis, yet the variations in pathogenesis are yet to be explicated. Clinical practice dictates distinct treatment guidelines and insurance drug coverage for each disease. As such, it is imperative to consider optimal medical care for aJIA, which necessitates continuous medical attention into adulthood.

**Objectives:** This study is to elucidate the present state of medical treatment for aJIA by juxtaposing its characteristics and challenges with those of RA in adolescent and young adult (aya-RA) via the database.

**Methods:** The study utilized the JMDC claims database, an epidemiological receipt database that has accumulated receipts and medical examination data received from multiple health insurance associations since 2005, from the fiscal years (FY) 2016 to 2020, focusing on subjects under the age of thirty. The data extraction process used ICD10 codes, and patients with at least two prescriptions for DMARDs were included. We compared the actual prescribing of csDMARD, b/tsDMARD, and glucocorticoids (GC), which are the main therapeutic agents, and the medication expenses.

**Results:** Of the 5,109,040 enrollees, 377 patients with aJIA and 1,496 patients with RA were included in this study. With respect to trends in therapeutics prescriptions from 2016-2020, the percentage of b/tsDMARD prescriptions for aJIA patients increased from 40% to 54%, while csDMARDs decreased from 93% to 84%, and GCs decreased from 51% to 40%. For aya-RA patients, b/tsDMARD prescriptions increased slightly from 38% to 41%, while csDMARDs decreased slowly from 93% to 86%. In the FY2020, aJIA showed a significantly lower percentage of prescriptions for csDMARDs alone and a higher percentage of prescriptions for combination of csDMARDs and b/tsDMARDs compared to aya-RA. b/tsDMARDs were prescribed, with ADA, TCZ, and ETN being the most commonly prescribed for aJIA, and TCZ, ETN, and ADA for aya-RA in descending order of frequency. The use of biosimilars accounted for 15% of bDMARD prescriptions in aya-RA patients in the FY2020, while the rate in aJIA overall was a low 3.1 %. In the same year, medication expenses for aJIA were 2.1 times higher than for aya-RA, at about 7,100 €.

**Conclusion:** The proportion of b/tsDMARDs prescribed in aJIA showed an upward transitional trend, leading to higher medication expenses relative to aya-RA. Future investigations are necessary to elucidate the suitability of aJIA management based on cost-effectiveness in terms of severity, disability, complications, quality of life, and labor productivity.

**Patient Consent**

Not applicable (there are no patient data)

**Disclosure of Interest**

None declared

## P034 Switching from adalimumab originator to biosimilar in children and young people with JIA

### L. Kearsley-Fleet^1^, E. Baildam^2^, M. W. Beresford^3,4^, S. Douglas^5^, H. E. Foster^6^, T. R. Southwood^7^, K. L. Hyrich^1,8^

#### ^1^Centre for Epidemiology Versus Arthritis, Manchester Academic Health Science Centre, The University of Manchester; ^2^The Alexandra Hospital, Manchester; ^3^Department of Paediatric Rheumatology, Alder Hey Children’s NHS Foundation Trust; ^4^Institute of Life Course and Medical Specialities, University of Liverpool, Liverpool; ^5^Scottish Network for Arthritis in Children (SNAC), Edinburgh; ^6^Population and Health Institute, Newcastle University, Newcastle; ^7^Institute of Child Health, University of Birmingham, Birmingham; ^8^National Institute of Health Research Manchester Biomedical Research Centre, Manchester Academic Health Science Centre, Manchester University NHS Foundation Trust, Manchester, United Kingdom

##### **Correspondence:** L. Kearsley-Fleet

*Pediatric Rheumatology 2023*, **21(Suppl 2):**P034

**Introduction:** The reduced cost of biosimilars has resulted in many children and young people with JIA receiving the adalimumab originator being switched onto the biosimilar in a non-medical switch (i.e. not for ineffectiveness or adverse event) due to competitive pricing. It is known that biosimilars show comparable efficacy to their originators in randomised clinical trials among biologic-naïve adults with RA received a biologic for the very first time. However, less is known about children and young people with JIA switching from an originator to a biosimilar product.

**Objectives:** This analysis aimed to describe (a) drug survival, and (b) disease activity following a switch from originator to biosimilar product, compared with a cohort of matched patients who remained on the originator.

**Methods:** Patients in the UK JIA Biologics Register switching from adalimumab originator to adalimumab biosimilar were matched 1-to-1 with those who remained on originator therapy; using gender, age (±2 years), disease duration (±2 years), originator start year (±2 years), line of therapy, and ILAR. Patient characteristics are presented at point of switch/matched date (index date). Time on adalimumab from index date onwards was calculated. Patients were censored at their final follow-up date, or any switch from originator to biosimilar in the originator comparison cohort. Change in disease activity from index date to six months (±4 months) was calculated. Multiple imputation accounted for missing data.

**Results:** As of 13-March-2023, 139 children and young people had switched from adalimumab originator to biosimilar, of which 112 had follow-up available and were matched with patients remaining on originator therapy; 61% female, median age at start of originator 11 years, median age at switch/match date 13 years, 81% polyarticular JIA, and 75% started originator as their first biologic.

The data suggest patients switching onto biosimilar were more likely to subsequently stop adalimumab treatment compared with those remaining on the originator; at 1 year 82% biosimilar patients and 90% originator patients remained on adalimumab, with 64% and 80% at 2 years respectively (did not reach statistical significance; hazard ratio for stopping 1.94, 95%CI 0.99-3.78). Of the 31 biosimilar patients who stopped treatment (median 2 years follow-up), 14 switched back to the originator (10 in first year, and 5 reported injection-related reasons for returning), 14 started a different biologic, and three remained off treatment at last follow-up.

Of the 58 matched patients with available disease activity, median change in JADAS-71 after six months was -0.2 units, with no evidence that JADAS-71 change differed between patients switching to adalimumab biosimilar compared with those remaining on originator (p=0.47).

**Conclusion:** Many JIA patients have now switched from adalimumab originator to adalimumab biosimilar, with the majority still receiving their biosimilar after one year. Disease activity remained similar between patients switching versus those on originator, although the data suggest more patients on biosimilar therapy stopped treatment; 4.5% of those on the biosimilar therapy stopped for biosimilar injection-related problems which may need to be investigated further. Switching back to originator was uncommon, 9% overall in the first year, suggesting good tolerance of non-medical switching in this patient population. This is reassuring to clinicians and patients regarding the impact of non-medical biological switching.

**Patient Consent**

Not applicable (there are no patient data)

**Disclosure of Interest**

None declared

## P035 Development of a decision support tool for withdrawal of biologic therapy in the absence of clinical evidence in non-systemic juvenile idiopathic arthritis

### M. M. Kip^1,2^, J. A. van Til^1^, R. Marinescu-Muster^3^, K. Groothuis-Oudshoorn^1^, G. Currie^4,5,6^, S. M. Benseler^7,8^, J. F. Swart^2,9^, S. J. Vastert^2,9^, N. Wulffraat^2,9^, R. S. Yeung^10^, D. A. Marshall^5,8,11^, M. J. IJzerman^1^ on behalf of on behalf of the UCAN CAN-DU and UCAN CURE consortia

#### ^1^Department Health Technology & Services Research, University of Twente, Enschede; ^2^Department of Pediatric Rheumatology, Wilhelmina Children's Hospital, Utrecht; ^3^Department of Communication Science, University of Twente, Enschede, Netherlands; ^4^Alberta Children's Hospital Research Institute; ^5^Department of Community Health Sciences, Cumming School of Medicine; ^6^Department of Paediatrics, Cumming School of Medicine; ^7^Alberta Children’s Hospital Research Institute; ^8^Division of Rheumatology, Department of Pediatrics, Alberta Children’s Hospital, Cumming School of Medicine, University of Calgary, Calgary, Canada; ^9^Faculty of Medicine, Utrecht University, Utrecht, Netherlands; ^10^Division of Rheumatology, The Hospital for Sick Children, Department of Paediatrics, Immunology and Institute of Medical Science, University of Toronto, Toronto; ^11^Department of Medicine, Cumming School of Medicine, University of Calgary, Calgary, Canada

##### **Correspondence:** M. M. Kip

*Pediatric Rheumatology 2023*, **21(Suppl 2):**P035

**Introduction:** Clinical guidance regarding biologic therapy withdrawal decisions in children with non-systemic juvenile idiopathic arthritis (JIA) is lacking, resulting in unwanted treatment variation.

**Objectives:** To develop and test a preference-based decision tool to support biologic therapy withdrawal decisions in children with non-systemic JIA.

**Methods:** An initial set of criteria used in the decision to withdraw biologic therapy was obtained from a previously conducted literature review and a focus group (Currie, Pham et al. 2021). Qualitative interviews among pediatric rheumatologists and expert discussions were used to remove redundant, potentially overlapping, and preferentially dependent criteria as well as to map the current decision process. Prior weights for the influence of each criterion on the withdrawal decision were derived from a clinical vignette study among pediatric rheumatologists. The identified criteria as well as their prior weights were incorporated in a decision support tool which was created in the interactive R-based 'Shiny' web application.

**Results:** The nine criteria that were selected involved: whether the child is rheumatoid factor positive or negative; the time between biologic therapy start and reaching inactive disease; history of spine involvement; history of temporomandibular joint involvement; history of joint damage; history of treatment failure with biologics; history of flares; history of uveitis; and whether or not the child and/or parents prefer to withdraw or continue biologic treatment. The prior weights from the clinical vignette study were used to estimate: 1) a likelihood of biologic therapy withdrawal and 2) the value of withdrawal over continuing treatment, for all combinations of scores that can be assigned to the nine criteria. The preferences of the child and/or parents whether to withdraw biologic therapy, as well as the occurrence of flares and uveitis in the current treatment period, were found to affect the withdrawal decision most strongly. Users can manually adjust the prior weights of the decision criteria in the tool to reflect their own judgement. This allows them to directly observe the impact of this adjustment on the likelihood of the withdrawal decision as well as on the value of withdrawal over continuing treatment.

**Conclusion:** In the absence of clinical guidance, this tool can support pediatric rheumatologists in deciding whether to withdraw biologic therapy in non-systemic JIA patients in clinical remission, and at which point in time. Thereby, unwanted treatment variation can be reduced. Also, this tool informs clinicians about the influence of each criterion on the decision. Future research will be used to optimize the design of the tool through conducting focus group sessions with pediatric rheumatologists to test its face validity, content validity and user friendliness. Data collected during future use of this tool in clinical practice will update and improve the tool’s underlying statistical model.

**Patient Consent**

Not applicable (there are no patient data)

**Disclosure of Interest**

M. M. Kip: None declared, J. A. van Til: None declared, R. Marinescu-Muster: None declared, K. Groothuis-Oudshoorn: None declared, G. Currie: None declared, S. M. Benseler: None declared, J. F. Swart Consultant with: Consulting fee from Amgen, outside the submitted work., S. J. Vastert Grant / Research Support with: Grants and personal fees from SOBI and Novartis during the conduct of the study, N. Wulffraat: None declared, R. S. Yeung Consultant with: Consulting fees from Novartis and Lily outside the submitted work, D. A. Marshall Grant / Research Support with: Non-financial support from ISPOR, and personal fees from Analytica, outside the submitted work., Consultant with: Non-financial support from consultancy (Illumina), outside the submitted work., M. J. IJzerman Grant / Research Support with: Institutional support from Illumina

**Reference**

1. Currie GR, Pham T, Twilt M, IJzerman MJ, Hull PM, Kip MMA, Benseler SM, Hazlewood GS, Yeung RSM, Wulffraat NM, Swart JF, Vastert SJ, Marshall DA. Perspectives of Pediatric Rheumatologists on Initiating and Tapering Biologics in Patients with Juvenile Idiopathic Arthritis: A Formative Qualitative Study. Patient. 2022 Sep;15(5):599-609. doi: 10.1007/s40271-022-00575-x. Epub 2022 Mar 24. PMID: 35322390.

## P036 Economic evaluation of alternative blood sampling strategies for patients with Juvenile Idiopathic Arthritis (JIA)

### N. H. Weghorst^1^, R. Kusters^1,2^, S. de Roock^3,4^, J. F. Swart^3,4^, M. M. Kip^1,3^

#### ^1^Health Technology & Services Research, University of Twente, Enschede; ^2^Laboratory for Clinical Chemistry and Hematology, Jeroen Bosch Ziekenhuis, 's-Hertogenbosch; ^3^Department of Pediatric Rheumatology, Wilhelmina Children's Hospital; ^4^Faculty of Medicine, Utrecht University, Utrecht, Netherlands

##### **Correspondence:** M. M. Kip

*Pediatric Rheumatology 2023*, **21(Suppl 2):**P036

**Introduction:** Many patients with chronic conditions currently undergo three-monthly phlebotomies in the hospital to monitor their health status and potential side effects of medication.

**Objectives:** This study provides insight in the cost impact of five alternative strategies for undergoing blood sampling in the treating hospital. The current study focuses on patients with JIA treated in the Wilhelmina Children’s Hospital (WCH).

**Methods:** The alternative blood sampling strategies, as alternative to blood sampling in the treating hospital, which were evaluated involved: 1) an at home finger prick blood sample which is send to the laboratory, 2) an at home finger prick point-of-care (POC) test, 3) a phlebotomist who visits the patient at home and takes the sample to the laboratory, 4) a phlebotomy plus analysis at a service phlebotomy centre, and 5) a phlebotomy plus analysis at a regional hospital. For all five strategies and for current practice the following costs were analysed: 1) costs per phlebotomy plus analysis, and 2) one-year costs assuming that patients undergo four check-ups per year in the WCH, of which one or two could be replaced by strategy 1-5. Costs were analysed both from a hospital and a societal perspective. The total costs for each strategy were calculated by adding up the associated resource use and accompanying costs for each strategy. One-way sensitivity analyses and a probabilistic sensitivity analysis were performed.

**Results:** The societal costs of blood sampling in the WCH (current practice) were €123.98 per phlebotomy, of which 85% was attributable to travel costs and productivity losses. An at home finger-prick costs €33.98, an at home POC test costs €22.40, a phlebotomist visiting the patient at home costs €52.19, the patient visiting a service phlebotomy centre costs €46.18, and the patient visiting a regional hospital costs €49.67. Over one year, the societal costs of blood sampling in the WCH are €495.67/patient. When assuming that one or two of these phlebotomies could be replaced by an alternative strategy, the annual societal costs will decrease, with the lowest costs for at home POC testing (€394.37 and €282.18 for one and two replacements, respectively), followed by an at home finger prick (€405.96 and €305.35, respectively), and the highest costs for a phlebotomist at home (€424.12 and €352.28, respectively).

**Conclusion:** An at home POC test or an at home finger prick were the most cost saving alternatives to undergoing a phlebotomy in the WCH. The majority of cost savings is attributable to a reduction in travel costs and productivity losses. Currently, the desirability of at home blood testing from the perspective of the patient and/or parent is investigated. This same study also investigates whether the implementation of at home testing can be used to skip one or two consultations with a paediatric rheumatologist on an annual basis, which may result in additional cost savings.

**Patient Consent**

Not applicable (there are no patient data)

**Disclosure of Interest**

None declared

## P037 Efficacy and safety of rituximab in patients with RF-positive juvenile idiopathic arthritis

### N. Kondrateva^1^, E. Alexeeva^1,2^, T. Dvoryakovskaya^1,2^, O. Lomakina^1^, A. Fetisova^1^, K. Isaeva^1^, A. Chomakhidze^1^, K. Chibisova^1^, I. Tsulukiya^1^, M. Botova^1^, I. Kriulin^1,2^, E. Krekhova^1^, M. Shingarova^1,2^, T. Kriulina^1,2^

#### ^1^Rheumatology, National Medical Research Center of Children's Health; ^2^Pediatric, Sechenov First Moscow State Medical University, Moscow, Russian Federation

##### **Correspondence:** N. Kondrateva

*Pediatric Rheumatology 2023*, **21(Suppl 2):**P037

**Introduction:** RF-positive polyarthritis is one of severe type of juvenile idiopathic arthritis (JIA) leading to dramatically decrease quality of life if left non-control. It closely resembles adult rheumatoid arthritis. Although DMARDs, steroids and biologic agents such as abatacept or TNF inhibitors significantly improve quality of life and prognosis for patients with RF-positive JIA there are still some patients who did not respond properly. Rituximab (RTX) is B-cell depleting agent successfully used in adult with refractory rheumatoid arthritis, so it may be a good treatment option for children with RF-positive JIA.

**Objectives:** To evaluate safety and efficacy of rituximab in children with RF-positive JIA.

**Methods:** Single-center retrospective study, including 20 patients (all – female) with RF-positive JIA who was treated with rituximab. Rituximab was used in combination with steroids or DMARDs. Efficacy was assessed by pediatric criteria of American College of Rheumatology (ACRpedi) and the index JADAS-71.

**Results:** Among 20 children 5 (25%) were biologic-naïve and rituximab was primary biologic DMARD. 15/20 (75%) patients previously receive bDMARDs: abatacept in 14/20 (70%), TNF inhibitors in 8/20 (40%), tocilizumab in 4/20 (20%). Average duration of the disease before RTX initiation was 4,99 (3,41;8,44) years. Rituximab was administered by two regimes (1000mg 2 times with 2 week interval or 375 mg/m^2^ 4 times weekly). Rituximab was used in combination with steroids - 7/20 (35%), methotrexate - 4/20 (20%), leflunomide – 12/20 (60%), sulfasalazine – 1/20 (5%), cyclosporine A - 1/20 (5%).

Four weeks after first rituximab infusion there was a statically significant (p<0,05) decrease in number of joints with active arthritis (Me 12 vs 0), decrease in JADAS71 activity score (Me 22,1 vs 3,5). One month after first infusion 20(100%)/17(85%)/7(35%)/1(5%) of patients achieved ACRpedi30/50/70/90 respectively. 6 (30%) of patients achieved JADAS71<1 score (inactive disease stage).

Among 17 patients twelve weeks after first rituximab infusion there was a statically significant (p<0,05) decrease in number of joints with active arthritis (Me 12 vs 0), decrease in JADAS71 activity score (Me 22,1 vs 0). Three month after first infusion 17 (100%)/ 15 (88%)/13 (76%)/5(29%) of patients achieved ACRpedi30/50/70/90 respectively. 12 (70%) of patients achieved JADAS71<1 score (inactive disease stage).

Twelve months after rituximab initiation 10/12 (83%) achieved ACRpedi30, 9/12 (75%) – ACRpedi50, 8/12 (67%) –ACRpedi70, 4/12 (33%) – ACRpedi90. Inactive disease identified by JADAS71 <1 score was achieved by 8/12 (67%).

During the first twelve months of observation no irreversible side effect occurred. Infusion related reactions were observed in 13/20 (65%) during first infusion, but quantity significantly decrease during next infusion. Infections – 18 episodes per 13 patient years (2- herpes zoster reactivation; 1- chicken pox; 2-bacterial pneumonia; 1- panniculitis).

**Conclusion:** Rituximab is effective in children with RF-positive JIA, leading to improving by fourth week after first administration. Rituximab has a good safety profile but patients need to be under close supervision during administration.

**Patient Consent**

Not applicable (there are no patient data)

**Disclosure of Interest**

None declared

## P038 How do the characteristics of juvenile idiopathic arthritis affect the continuation or refusal of vaccination against diphtheria: cross-sectional study data?

### M. Kostik^1,2^, N. Lyubimova^2^, O. Goleva^3^, S. Kharit^3^

#### ^1^Pediatric Department No. 3, Saint-Petersburg State Pediatric Medical University; ^2^Department of Pediatrics and Medical Rehabilitation № 1, Almazov National Medical Research Centre; ^3^Pediatric Research and Clinical Center for Infection Diseases, Saint-Petersburg, Russian Federation

##### **Correspondence:** M. Kostik

*Pediatric Rheumatology 2023*, **21(Suppl 2):**P038

**Introduction:** Patients with juvenile idiopathic arthritis (JIA) often stop being vaccinated after the onset of the disease due to fear of disease flare, although the effectiveness and safety of vaccination in immunocompromised patients has been demonstrated.

**Objectives:** The aim of our study was to evaluate the JIA characteristics associated with the refusal to continue to be vaccinated against diphtheria.

**Methods:** In a cross-sectional study, we included data about patients who continued (n=25) or refused (n=51) vaccination against diphtheria after the onset of JIA. In all patients, the levels of anti-diphtheria vaccine antibodies (IgG) were determined with the ELISA. The data are presented with a median and 25%-75%.

**Results:** The age of disease onset, JIA duration and JIA categories were similar between groups. Patients, declined the following vaccination often receive methotrexate and biologics and switched at least one biologic. Methotrexate (OR=9.5 [95%CI: 1,004; 90.3]) and biologics (OR=4.4 [95%CI: 1.6; 12.1]) were predictors of refusal of revaccination against diphtheria. Vaccination against diphtheria was effective, as evidenced by the almost two fold prevalence of patients with a protective antibody titer compared to those who refused revaccination. Serious adverse events, as well as JIA flares in three months after vaccination were not recorded.

**Conclusion:** The continuation of vaccination against diphtheria in children with JIA against diphtheria was effective and safe. The treatment with methotrexate and biologics was predictors of refusal of revaccination against diphtheria. Further studies are needed to confirm the safety and efficacy of vaccination against diphtheria in children with JIA and can increase the level of confidence of physicians in vaccination of children with rheumatic diseases.

**Trial registration identifying number:** This work was financially supported by the Ministry of Science and Higher Education of the Russian Federation (Agreement No. 075-15-2022-301).

**Patient Consent**

Yes, I received consent

**Disclosure of Interest**

None declared

## P039 The role of synovial calprotectin in identify of juvenile idiopathic arthritis vs pigmented villonodular synovitis: a pilot study

### A. Kozhevnikov^1,2^, E. Derkach^1^, S. Vissarionov^1^

#### ^1^H.Turner National Medical Research Center for Children's Orthopedics and Trauma Surgery; ^2^Saint-Petersburg State Pediatric Medical University, Saint-Petersburg, Russian Federation

##### **Correspondence:** A. Kozhevnikov

*Pediatric Rheumatology 2023*, **21(Suppl 2):**P039

**Introduction:** Juvenile idiopathic arthritis (JIA) is one of the most severe inflammatory joint diseases. Oligoarticular subtype (oligo-JA) most common form of JIA. Pigmented villonodular synovitis (PVNS) is a rare pseudoneoplastic origin of synovial membrane disease characterized by mimic oligo-JA cause. Differential diagnosis of these diseases is often difficulty. Intraarticular steroids used in JIA may negatively affect on course of PVNS. Laboratory tests neither rule in nor rule out JIA. Serum calprotectin is a heterodimer of two calcium-binding proteins present in the cytoplasm of neutrophils that is released in inflammatory processes. Synovial calprotectin has been demonstrated as a promising biomarker of JIA.

**Objectives:** The purpose of the investigation was to explore significance of synovial calprotectin in differential diagnosis JIA and PVNS.

**Methods:** A total of 30 children who were suspected of oligo-JA and 8 – PVNS were enrolled. PVNS – all diffuse form of knee diagnosed after histological futures. Oligo-JA – all with active knee arthritis, ANA positive. The synovial fluid calprotectin (synCal), level of IL6 and TNF-alpha was tested by an enzyme-linked immunosorbent assay (ELISA). Synovial levels were presented as a median [5th; 95th percentile]. Serum calprotectin was also analyzed.

**Results:** The median of synCal level was 108 μg/ml [28,2; 237] in the oligo-JA group and 1,53 μg/ml [1,26; 1,69] in children with PVNS (p < 0.01). The median level synovial IL6 was 4200 pg/ml [330; 14400] in the JIA group and 4300 pg/ml [1612; 6580] in children with PVNS (p > 0.05). Synovial level of TNF-alpha were not correlated with active oligo-JA and PVNS (2,13 vs 1,93 pg/ml, p>0,05). Level of Serum calprotectin in active oligo-JA were 2,61 μg/ml [1,25; 3,95], PVNS - 1,25 μg/ml [0,78; 2,35].

**Conclusion:** Differential diagnosis of oligo-JA and PVNS often causes difficulties. Elevated synovial levels of interleukin-6 cannot to be criteria PVNS. Synovial calprotectin showed association with JIA. Calprotectin appears to be a useful marker of joint inflammatory and could be significance like biomarker for diagnostic criteria JIA.

**Patient Consent**

Not applicable (there are no patient data)

**Disclosure of Interest**

None declared

## P040 Is the MEFV heterozygosity a predisposing factor for Pfapa syndrome phenotype?

### A. S. Donmez^1^, S. Kilic Kaya^1^, A. Cayir^1^, S. Sahin^2^, A. Adrovic^1^

#### ^1^Pediatrics, Erzurum Public Hospital, Erzurum; ^2^Pediatric Rheumatology, Istanbul University-Cerrahpasa, Istanbul, Türkiye

##### **Correspondence:** A. Adrovic

*Pediatric Rheumatology 2023*, **21(Suppl 2):**P040

**Introduction:** PFAPA syndrome represents the most common auto-inflammatory disease worldwide. Familiar Mediterranean fever is a period fever mostly seen in Mediterranean countries. Due to common clinical features, the clinical distinction in FMF endemic regions could be challenging.

We aimed to explore whether the MEFV heterozygosity influences the disease phenotype in PFAPA patients.

**Objectives:** We aimed to explore whether the MEFV heterozygosity influences the disease phenotype in PFAPA patients.

**Methods:** Patients diagnosed as FMF and /or PFAPA syndrome were randomized. All the patients were evaluatedregarding the clinical features and underlying MEFV gene mutations. The comparison analysis between patients with MEFV heterozygosity and homozygosity has been performed.

**Results:** A total of 92 patients were included in the study: 54 (58.7%)with FMF and 38 (41.3%) with PFAPA syndrome. The mean age at the diagnosis was 3.25 ±1.15 years, at the time of the study was 5.06±2.12 years. Among all, 46 (50.5%) of patients were heterozygous while 45 (49.5%) were homozygous for MEFV gene mutations. While there was no difference regarding the frequency and the duration of the fever attacks, the age at diagnosis was significant lower in homozygous patients. The family history of tonsillectomy, recurrent otitis attacks and diarrhea were significantly more common among heterozygous patients. Patients with MEFV heterozygosity were more prone to obtain diagnosis of PFAPA syndrome while those with homozygosity were more commonly diagnosed as FMF.

**Conclusion:** PFAPA syndrome could be challenging diagnosis in populations endemic for FMF. Thus, patients with recurrent fever syndromes should be evaluated for underling MEFV gene mutation, especially in regions with high frequent FMF. MEFV heterozygosity is more associated with PFAPA syndrome phenotype. Prospective studies with higher number of patients are required.

**Patient Consent**

Yes, I received consent

**Disclosure of Interest**

None declared

**References**

1. Gozen ED, Yildiz M, Kara S, Tevetoglu F, Haslak F, Adrovic A, Sahin S, Barut K, Ulkersoy İ, Gücüyener N, Gunalp A, Yener HM, Ada M, Kasapcopur O. Long-term efficacy of tonsillectomy/adenotonsillectomy in patients with periodic fever aphthous stomatitis pharyngitis adenitis syndrome with special emphasis on co-existence of familial Mediterranean fever. Rheumatol Int. 2023 Jan;43(1):137-145. doi: 10.1007/s00296-022-05210-4. Epub 2022 Sep 18. PMID: 36116090.
2. Yıldız M, Haslak F, Adrovic A, Ülkersoy İ, Gücüyener N, Şahin S, Barut K, Kasapçopur Ö. Periodic Fever, Aphthous Stomatitis, Pharyngitis, and Adenitis Syndrome: A Single-Center Experience. Turk Arch Pediatr. 2022 Jan;57(1):46-52. doi: 10.5152/TurkArchPediatr.2021.21229. PMID: 35110078; PMCID: PMC8867508.
3. Adrovic A, Sahin S, Barut K, Kasapcopur O. Familial Mediterranean fever and periodic fever, aphthous stomatitis, pharyngitis, and adenitis (PFAPA) syndrome: shared features and main differences. Rheumatol Int. 2019 Jan;39(1):29-36. doi: 10.1007/s00296-018-4105-2. Epub 2018 Jul 17. PMID: 30019226.

## P041 25-hydroxyvitamin D levels in patients with chronic recurrent multifocal osteomyelitis

### N. Afifi, S. Gardee, A. Gunaratram, B. Kaur, D. Klepárník, Z. Pytelová, K. Bouchalová

#### Paediatric Rheumatology, Department of Paediatrics, Faculty of Medicine and Dentistry, Palacky University Olomouc and University Hospital, Olomouc, Czech Republic

##### **Correspondence:** K. Bouchalová

*Pediatric Rheumatology 2023*, **21(Suppl 2):**P041

**Introduction:** Chronic recurrent multifocal osteomyelitis (**CRMO**) is a rare autoinflammatory disease. The etiology is unknown, and its clinical manifestations may include bone pain, joint swelling, and fever. There is no definitive classification of CRMO - the diagnosis is *per exclusion*. There are also no definitive guidelines for treatment. Treatment is tailored to the patient, starting with NSAID therapy with the addition of bisphosphonates and/or biologics if/when required. 25-hydroxyvitamin D (**25-OH vitamin D**) is a steroid hormone that controls calcium and phosphate metabolism and bone mineralization. In the standard population the percentage of hypovitaminosis D is between 8-30% *(Leão et al, 2021).* There has not been any investigations or publications into the levels of 25-OH vitamin D in CRMO patients.

**Objectives:** Comparison between the level of 25-OH vitamin D in patients with CRMO at the time of diagnosis and the level at the last check-up.

**Methods:** Sixteen patients from the pediatric clinic in the university hospital in Olomouc, Czech Republic were included in the study, in which four were male and twelve were female. The mean age of the patients is 12.2. The normal levels for serum 25-OH vitamin D is 75-250 nmol/L *(Charoenngam et al, 2020)*. 25-OH vitamin D was measured using the method of Chemiluminescent immunoassay. In this study, 25-OH vitamin D levels were compared at the time of diagnosis and last follow-up using the Wilcoxon paired test.

**Results:** At the time of diagnosis twelve of the sixteen patients had low levels of 25-OH vitamin D compared to the reference value 75nmol/l. Two out of the remaining four were borderline. Prevalence of a low level of 25-OH vitamin D in patients with CRMO was found to be 75%. Patients with CRMO received 25-OH vitamin D supplements. The levels of 25-OH vitamin D at the last check-up were statistically significantly higher than 75nmol/l, **(p=0.004)**. Wilcoxon paired test showed a statistically significant difference between baseline and last follow up levels **(p=0.011).**

**Conclusion:** In our series of CRMO patients, we analyzed for the first time levels of 25-OH vitamin D in CRMO patients. We revealed that 75% of patients had low levels of 25-OH vitamin D at time of diagnosis, which was successfully reversed by vitamin D supplementation and the clinical status of the patients was improved.

**Patient Consent**

Not applicable (there are no patient data)

**Disclosure of Interest**

None declared

**References**

1. Leão, L. M., Rodrigues, B. C., Dias, P. T., Gehrke, B., Souza, T. da, Hirose, C. K., & Freire, M. D. Vitamin D status and prevalence of hypovitaminosis D in different genders throughout life stages: A Brazilian cross-sectional study. *Clinics*, *2021 January;76.* doi:10.6061/clinics/2021/e2571
2. Charoenngam N, Holick MF. Immunologic Effects of Vitamin D on Human Health and Disease. *Nutrients. 2020 July15;12(7):2097*. doi: 10.3390/nu12072097. PMID: 32679784; PMCID: PMC7400911.

## P042 Novel gene mutation of tumor necrosis factor receptor-associated periodic syndrome (TRAPS) identified across three generation in an omani family

### S. Al Abrawi, F. Al Bulushi, S. Al Hashmi, R. Al Jashmi

#### Royal Hospital, Muscat, Oman

##### **Correspondence:** S. Al Abrawi

*Pediatric Rheumatology 2023*, **21(Suppl 2):**P042

**Introduction:** Tumour Necrosis Factor Receptor Associated Periodic Syndrome TRAPS is a rare genetic disease that causes auto-inflammatory symptoms. It is an autosomal dominant inheritance caused by mutation of the TNFRSF1A gene, which is characterized by recurrent episodes of fever, abdomen pain, arthralgia, periorbital oedema, conjunctivitis and erythematous skin rash. Though it was initially described in European ancestry. The mutations of TNFRSF1A have now been found in other ethnicities including those from Arab origin.

**Objectives:** The aim of this paper is to describe the novel mutation of TRAPS across three generations in an extended Omani family.

**Methods:** We retrospectively reviewed the demographic and clinical features through electronic medical record notes of patients from an extended Omani family seen at Royal hospital, a tertial hospital in Muscat, Oman between 2016-2022 whom genetic test revealed a novel heterozygous mutation (c.375T/P. Cys125Trp) of TRAPS.

**Results:** We identified 10 members of the same family in three generation with age range from (2 yr to 79 yr) with a novel heterozygous mutation (c.375T/P.Cys125Trp) of Tumour Necrosis Factor Receptor Associated Periodic Syndrome.All patients had recurrent attacks of prolonged fever more than 2 weeks associated with arthralgia, myalgia and abdominal pain with elevated inflammatory markers. Periorbital edema was observed in 30% of patients. Majority of patients have mild disease activity and on demand symptomatic therapy of non steroid anti-inflammatory drug,and steroid.Few patients are on tumour necrosis factor blocker etanercept because they refused Interleukin 1 inhibitor anakinra subcutaneous daily injection.Canakinumab IL-1B inhibitor is not available due to extremely high cost.There is no evidence of secondary amyloidosis was identified across three generation in this extended family inspite of long duration of disease, indicating that this novel heterozygous mutation (c.375T/P.Cys125Trp) of TRAPS has mild course of disease.

**Conclusion:** To the best of our knowledge, this novel heterozygous mutation (c.375T/P. Cys125Trp) has not been described from other ethnicities.TRAPS needs to be investigated in patients with recurrent attacks of prolong fever for more than a week to avoid delay in diagnosis.

**Patient Consent**

Yes, I received consent

**Disclosure of Interest**

S. Al Abrawi Consultant with: No conflict of interest , F. Al Bulushi Consultant with: No conflict of interest, S. Al Hashmi Consultant with: No conflict of interest, R. Al Jashmi Consultant with: No conflict of interest

**References**

1. Al-Mayouf SM, Almutairi A, Albrawi S, Fathalla BM, Alzyoud R, AlEnazi A, et al. Pattern and diagnostic evaluation of systemic autoinflammatory diseases other than familial Mediterranean fever among Arab children: A Multicenter Study from the Pediatric Rheumatology Arab Group (PRAG). Rheumatology International. 2019;40(1):49–56.
2. Nezos A, Argyropoulou OD, Klinaki E, Marketos N, Karagianni P, Eliopoulos E, et al. Molecular and clinical spectrum of four pedigrees of traps in Greece: Results from a national referral center. Rheumatology. 2019;59(6):1241–6.
3. Lachmann HJ, Papa R, Gerhold K, Obici L, Touitou I, Cantarini L, et al. The phenotype of TNF receptor-associated autoinflammatory syndrome (TRAPS) at presentation: A series of 158 cases from the Eurofever/EUROTRAPS International Registry. Annals of the Rheumatic Diseases. 2013;73(12):2160–7.

## P043 ADA2 deficiency mimicking behcet's disease: another face of a rare monogenic autoinflammatory disorder

### A. I. Almojali, A. Alrasheed

#### Pediatric Rheumatology, King Abdullah Specialised Children's Hospital, Riyadh, Saudi Arabia

##### **Correspondence:** A. I. Almojali

*Pediatric Rheumatology 2023*, **21(Suppl 2):**P043

**Introduction:** Deficiency of adenosine deaminase 2 (DADA2) is an autosomal recessive autoinflammatory disease with broad expanding clinical spectrum caused by pathogenic variants in ADA2 gene. On the other hand, Behçet disease is another vasculitic disorder that has a strong genetic association with the presence of human leukocyte antigen 51 (HLA-B51), which helps in distinguish this disease from other systemic vasculitides.

**Objectives:** To report a case series of three siblings with confirmed DADA2 presenting with different phenotypes, two of which resembling Behcet's disease with positive HLA-B51.

**Methods:** Whole-exome sequencing (WES) was performed in the index case, and Sanger sequencing of ADA2 gene was done in other siblings.

**Results:** We report a family with three siblings confirmed to have DADA2 disease. The index case **(II.1)** was presented at age of 4 years with bilateral painful erythema nodosum like lestions of the lower limbs, episodes of low-grade fever, arthritis, and history of recurrent oral ulcers with negative autoantibodies. HLA-B51 revealed a positive result and she was started on colchicine with impression of Behçet disease. She was followed regularly in the clinic for almost 2 years, during which she needs methotrexate and subsequently Adalimumab due to poor response. Ultimately, her symptoms have improved significantly after adding Adalimumab. During that time, her younger sister **(II.2)** presented to our clinic at age of 5 years with almost similar presentation (recurrent oral ulcers, arthritis, episodes of low-grade fever) and laboratory results (negative immunologic panel and positive HLA-B51). Additionally, she had significant livedo reticularis involving her lower limbs. She was started initially on colchicine, and WES was sent for both patients looking for a genetic cause of systemic vasculitis. Genetic testing identified an ADA2 homozygous variant c.139G>A p.(Gly47Arg) in both sisters. Sanger sequencing of ADA2 gene was done to their younger sister with age of 4 years **(II. 3)** who is asymptomatic, and the same homozygous variant was identified. After the result, II. 2 was started on Adalimumab with excellent response.

**Conclusion:** Our report expands the significant variable phenotype of DADA2 as it can mimic Behçet disease with positive HLA-B51. It also indicates the phenotypic severity may differ even in siblings with the same variants.

**Patient Consent**

Yes, I received consent

**Disclosure of Interest**

None declared

## P044 Not easy-peasy to diagnose: familial mediterranean fever unaccompanied by fever as neither always Mediterranean

### S. D. Arık^1^, G. Kavrul kayaalp^1^, V. gulıyeva^1^, F. demırkan^1^, N. aktay ayaz^1^, O. akgun^1^, S. G. karadag^1^, B. sozerı^2^, K. ulu^2^, S. turker caglayan^2^, T. coskuner^2^

#### ^1^Pediatric rheumatology, Istanbul Unıversity; ^2^Pediatric Rheumatology, Umraniye Training and Research Hospital, Istanbul, Türkiye

##### **Correspondence:** S. D. Arık

*Pediatric Rheumatology 2023*, **21(Suppl 2):**P044

**Introduction:** The diagnosis of FMF is based on the demonstration of recurrent episodes of peritonitis, pleuritis, pericarditis, and arthritis, usually accompanied by fever and supported by the presence of MEFV gene variants. Classical attacks of familial Mediterranean fever (FMF) are often accompanied by fever, but some of the patients have attacks without fever.

**Objectives:** To compare the characteristics of FMF patients with and without fever during their attacks and draw attention to the different clinical presentations of FMF in children.

**Methods:** Medical files of patients aged 0-18 years who were followed up with the diagnosis of FMF in two reference pediatric rheumatology centers were reviewed retrospectively. The patients were divided into two groups: Children who had had no fever in any of their attacks were assigned as group 1, and those who had fever during their attacks were classified as group 2.

**Results:** Out of 2003 patients evaluated, 191 (9.53%) patients had attacks not accompanied by fever. The median age at onset of symptoms afebrile group and febrile group were 7.0 and 4.0 years, respectively (p<0.001). The median age at diagnosis (8.6 vs 6.0 years, p<0.001) was significantly higher in the febrile group, but there was a delay in diagnosis in the febrile group. The annual number of attacks and abdominal attacks were more common in group 2. The frequency of symptoms was compared and arthritis, erysipelas-like rash, exercise-induced leg pain, arthralgia and myalgia were more common in group 1. The distribution of MEFV mutation variances was similar in both groups. Carrying one pathogenic mutation or biallelic pathogenic mutations of the two groups were evaluated and no significant difference was found. Colchicine resistance was observed similarly in patients without fever (2.6%) and patients with fever (5.6%) (p=0.078).

**Conclusion:** The data from the assessment of children with FMF attacks not accompanied with fever were presented for the first time. Children with late age onset of FMF and dominance of musculoskeletal features may display attacks not accompanied with fever.

**Trial registration identifying number:** (Date:21/05/2020; No:19)

**Patient Consent**

Yes, I received consent

**Disclosure of Interest**

None declared

## P045 Exertional leg pain represents a severe disease phenotype in childhood familial Mediterranean fever

### F. Aydin, Z. B. Özçakar, P. Ö. Avar Aydin, N. Çakar

#### Pediatric Rheumatology, Ankara University School of Medicine, Ankara, Türkiye

##### **Correspondence:** F. Aydin

*Pediatric Rheumatology 2023*, **21(Suppl 2):**P045

**Introduction:** Familial Mediterranean fever (FMF) is the most common monogenic autoinflammatory disease. Recurrent fever, serositis and arthritis are common findings of the disease. In addition, musculoskeletal complaints such as exertional leg pain can be overlooked, although they are common and affect patients' quality of life.

**Objectives:** The aim of this study was to evaluate the frequency of exertional leg pain in pediatric FMF patients and to analyze the association of this finding with other characteristics of FMF.

**Methods:** The files of FMF patients were retrospectively evaluated. The clinical characteristics and disease severity of the patients with exertional leg pain were compared with the patients without exertional leg pain.

**Results:** The study included 541 FMF patients (287 females), 149 (27.5%) with exertional leg pain. The median colchicine dosage was significantly higher in patients with exertional leg pain (p=0.02), arthritis (p=0.001) and arthralgia (p<0.001) were encountered more frequently in the attacks of these patients. The median disease severity scores calculated by both Mor severity scale and International FMF severity scoring system (ISSF) were significantly higher in patients with exertional leg pain compared to those without. In the group of patients with exertional leg pain the M694V mutation, either in 1 allele or in 2 alleles, was found to be significantly more common (p=0.006 and p<0.001, respectively).

**Conclusion:** Exertional leg pain in pediatric FMF patients is the component of the moderate-to-severe disease course, and this may be considerably associated with the presence of the M694V mutation.

**Patient Consent**

Not applicable (there are no patient data)

**Disclosure of Interest**

None declared

**References**

Eshed I, Kushnir T, Livneh A, et al. Exertional leg pain as a manifestation of occult spondyloarthropathy in familial Mediterranean fever: an MRI evaluation. Scand J Rheumatol. 2012;41:482–486.
Livneh A, Langevitz P, Zemer D, Zaks N, Kees S, Lidar T, et al. Criteria for the diagnosis of familial Mediterranean fever. Arthritis Rheum. 1997;40:1879–85.
Langevitz P, Livneh A, Zemer D, Shemer J, Pras M. Seronegative spondyloarthropathy in familial Mediterranean fever. Semin Arthritis Rheum. 1997;27:67–72.
Eshed I, Rosman Y, Livneh A, et al. Exertional leg pain in familial Mediterranean fever: a manifestation of an underlying enthesopathy and a marker of more severe disease. Arthritis Rheumatol. 2014;66:3221–3226.
5. Yalçınkaya F, Ozen S, Ozcakar ZB ¸ Aktay N, Çakar N, Düzova A, et al. A new set of criteria for the diagnosis of familial Mediterranean fever in childhood. Rheumatology. 2009;48:395-398.

## P046 Patient with a novel mutation in CTLA-4 gene

### E. B. Basahl^1^, A. Y. Alfarsi^2^, N. M. Aldajani^1,2^, M. A. Nashawi^1,2^

#### ^1^Faculty of Medicine, King Abdulaziz University; ^2^Pediatrics, King Abdulaziz University Hospital, Jeddah, Saudi Arabia

##### **Correspondence:** E. B. Basahl

*Pediatric Rheumatology 2023*, **21(Suppl 2):**P046

**Introduction:** A protein-coding gene called CTLA4 (Cytotoxic T-Lymphocyte Associated Protein 4) is a negative immunological regulator for the functioning of T cells by inhibiting T cell differentiation and proliferation ^(1)^. Mutation in this gene is known to cause multiple autoimmune phenotypes.

**Objectives:** We present a patient with novel mutation in CTLA 4 gene

**Methods:** Case presentation

**Results:** 11-year-old girl was referred to our clinic with a suspected autoimmune disease. She developed several clinical features since the age of 8 years. At the beginning recurrent lower respiratory tract infections and complicated with interstitial lung disease. Furthermore, she developed finger clubbing. Otherwise, she noticed to have progressive Hepatosplenomegaly and generalized lymphadenopathy. in the last months, she developed small joint arthritis and exanthem over the extremities. Regarding her labs, she found to have pancytopenia, hypogammaglobulinemia and high inflammatory markers. Whole exome sequencing was sent and showed a novel homozygous mutation in CTLA-4 gene (c.*54T>G,).

Patient started on regular IVIG as well as Steroid and Abatacept improved clinically.

**Conclusion:** We present a patent with multiple autoimmune phenotypes, which were found to have a novel mutation in CTLA-4 gene. Further workup regarding functional analysis and segregation of the variant in the family is needed to confirm the diagnosis.

**Patient Consent**

Yes, I received consent

**Disclosure of Interest**

None declared

**Reference**

1. Schwab C, Gabrysch A, Olbrich P, Patiño V, Warnatz K, Wolff D, et al. Phenotype, penetrance, and treatment of 133 cytotoxic T-lymphocyte antigen 4-insufficient subjects. J Allergy Clin Immunol [Internet]. 2018 ;142(6):1932–46. Available from: https://pubmed.ncbi.nlm.nih.gov/29729943/

## P047 Cluster analysis to identify different clinical phenotypes of chronic non-bacterial osteomyelitis patients

### Y. Bayindir^1^, O. Basaran^1^, S. Demir^2^, E. Aliyev^1^, K. Ulu^3^, E. Kayhan^4^, F. Haslak^5^, O. Akgun^6^, E. A. Aydin^7^, R. Isguder^8^, Z. E. Tekin^9^, H. Kose^10^, O. Baba^11^, N. Karacayir^12^, S. Ayduran^13^, E. G. Ay^14^, U. K. Akca^15^, H. A. Dundar^16^, M. K. Cuceoglu^1^, S. Caglayan^3^, A. Y. Bulbul^4^, S. E. Varol^5^, E. D. Batu^1^, H. E. Sonmez^17^, K. Ozturk^18^, M. K. Gurgoze^14^, S. Yuksel^13^, M. Kalyoncu^11^, S. A. Bakkaloglu^12^, S. S. Kilic^10^, E. Unsal^8^, S. Ozdel^7^, B. C. Acar^9^, N. A. Ayaz^6^, O. Kasapcopur^5^, A. P. Kisaarslan^4^, B. Sozeri^3^, Y. Bilginer^1^, S. Ozen^1^

#### ^1^Department of Pediatric Rheumatology, Hacettepe University, Ankara; ^2^Department of Pediatric Rheumatology, Eskisehir Osmangazi University, Eskisehir; ^3^Department of Pediatric Rheumatology, Umraniye Research and Training Hospital, Istanbul; ^4^Department of Pediatric Rheumatology, Erciyes University, Kayseri; ^5^Department of Pediatric Rheumatology, Istanbul University, Cerrahpasa Faculty of Medicine; ^6^Department of Pediatric Rheumatology, Istanbul University, Capa Faculty of Medicine, Istanbul; ^7^Department of Pediatric Rheumatology, Ankara Etlik City Hospital, Ankara; ^8^Department of Pediatric Rheumatology, Dokuz Eylul University, Izmir; ^9^Department of Pediatric Rheumatology, Ankara City Hospital, Ankara; ^10^Department of Pediatric Rheumatology and Immunology, Uludag University, Bursa; ^11^Department of Pediatric Rheumatology, Karadeniz Technical University, Trabzon, ^12^Department of Pediatric Rheumatology, Gazi University, Ankara; ^13^Department of Pediatric Rheumatology, Pamukkale University, Denizli; ^14^Department of Pediatric Rheumatology, Fırat University, Elazıg; ^15^Department of Pediatric Rheumatology, Aydın Maternity and Children's Hospital, Aydın; ^16^Department of Pediatric Rheumatology, Van Research and Training Hospital, Van; ^17^Department of Pediatric Rheumatology, Kocaeli University, Kocaeli; ^18^Department of Pediatric Rheumatology, Istanbul Medeniyet University, Istanbul, Türkiye

##### **Correspondence:** Y. Bayindir

*Pediatric Rheumatology 2023*, **21(Suppl 2):**P047

**Introduction:** Chronic non-bacterial osteomyelitis (CNO) is an autoinflammatory bone disease frequently seen in children and adolescents. The onset and clinical course of the disease vary among patients.

**Objectives:** We aimed to identify different clinical phenotypes among chronic non-bacterial osteomyelitis patients using cluster analysis.

**Methods:** In this multicenter cohort study, we included all patients diagnosed with CNO between the years of 2005-2022. A two-step cluster analysis was performed to identify subgroups of patients based on the affected bones including humerus, ulna, radius, hand bones, femur, tibia, fibula, foot bones, vertebra, scapula, clavicula, sternum, costa, mandibula, sacrum, ilium, ischium, head bones.

**Results:** There were 307 patients diagnosed with CNO. Patients were classified into 4 clusters using cluster analysis. Cluster 1 (n = 79) consisted predominantly of patients with vertebra, clavicula, and sternum involvement. Cluster 2 (n = 115) consisted predominantly of patients with lower extremities as femur, tibia, fibula, and foot-bones involvement. Cluster 3 (n=50) was composed mainly of patients with upper extremities as humerus, radius, and ulna involvement. Cluster 4 (n = 63) consisted predominantly of patients with the ilium, ischium, and sacrum involvement. The age at symptom onset [10.68 (0.92-17.61); p=0.021] and the age of diagnosis [13.23 (2.00-18.02); p=0.004] were higher and female gender (59.5%; p=0.009) was more prominent in Cluster 1 than other clusters. Arthritis, enthesitis and gastrointestinal system involvement were not significantly different between clusters.

**Conclusion:** We identified 4 clinical phenotypes of pediatric CNO patients. The age of symptom onset and age of the diagnosis; tended to be higher in cluster 1 which consisted of patients with more vertebra, clavicula, and sternum involvement. The frequency of the female patients and skin involvement were also found to be higher in those patients.

**Patient Consent**

Yes, I received consent

**Disclosure of Interest**

None declared

## P048 Monogenic disorders in the maiden cohort of childhood rheumatological illnesses from Nepal

### D. Bhattarai^1^, A. Z. Banday^2^, P. K. Patra^3^

#### ^1^Pediatric Immunology & Rheumatology, Advanced Centre for Immunology and Rheumatology, Kathmandu, Nepal; ^2^Pediatric Rheumatology, Government Medical College, Srinagar, Kashmir, India, Srinagar; ^3^Pediatrics, All India Institute of Medical Sciences, Patan, India

##### **Correspondence:** D. Bhattarai

*Pediatric Rheumatology 2023*, **21(Suppl 2):**P048

**Introduction:** Availability of subspecialty has opened a window for diagnosing pediatric rheumatological diseases (PRDs) in resource-limited countries. Monogenic disorders are also being detected as the cause of inborn errors of immunity and pediatric rheumatological diseases (PRDs).

**Objectives:** To describe the maiden profile of patients diagnosed with monogenic PRDs at a tertiary care centre in Kathmandu, Nepal during 2020-2023.

**Methods:** Case records of patients diagnosed with monogenic PRDs at the rheumatology clinics in Nepal during Aug 2020-April 2023 were analysed. The lead author (DB) collated data from all patients. Diagnosis and treatments were based on internationally acclaimed guidelines.

**Results:** A total of 553 patients with definite PRDs were diagnosed. Most (71%) patients were referred from various hospitals in the Himalayan range (Nepal, Northern Indian states, and Bhutan). The mean duration from initial presentation to diagnosis was 13.5 months. Juvenile idiopathic arthritis (n = 181), connective tissue disorders (n = 113), vasculitides (n = 104), autoinflammatory diseases (n = 61), immune-dysregulation with lymphoproliferation (n = 24), and arthritis/vasculitis related to infections (n = 21) constituted the significant proportions. Monogenic causes were diagnosed in 19 patients. All these disorders were reported for the first time in Nepal. Monogenic disorders (with afflicted genes) included A20 haploinsufficiency (*TNFAIP3)*, *ARPC1B* deficiency (*ARPC1B*), deficiency of adenosine deaminase 2 (*CECR1*), Blau syndrome (*NOD2*), activated phosphoinositide 3-kinase δ syndrome (*PIK3CD GOF)*, tumor necrosis factor receptor-associated periodic syndrome (*TNFRSF1A)*, Pyogenic sterile arthritis, pyoderma gangrenosum, and acne syndrome (*PSTPIP1*), X-linked inhibitor of apoptosis protein deficiency (*XIAP*), Chronic Atypical Neutrophilic Dermatosis Lipodystrophy & Elevated Temperature (*PSMB8)*, hyperimmunoglobinemia D with periodic fever syndrome (*MVK*), Familial hemophagocytic lymphohistiocytosis (*STX11*), Familial cold autoinflammatory syndrome (*NLRP3*), autoimmune lymphoproliferative syndrome (*TNFRSF6*), primary pulmonary hypertension (*BMPR2*) *C1QA* defect, and *CTLA4* defect. One child with a monogenic disorder is undergoing stem cell therapy.

**Conclusion:** We present the first ever cohort of PRDs diagnosed in Nepal which also includes monogenic autoinflammatory disorders. The availability of pediatric subspecialists and diagnostics has shifted the paradigm of diagnosing PRDs in Nepal. It is prudent to keep the monogenic disorders in mind while dealing with PRDs. Lack of awareness coupled with various socioeconomic and environmental factors accounted for a late presentation, increased morbidity, disability, and mortality in these diseases.

**Patient Consent**

Yes, I received consent

**Disclosure of Interest**

None declared

## P049 Diagnostic approach to pediatric patients with recurrent fevers: a survey on general pediatricians’ perspective

### C. V. Bronzoni, L. A. Baselli, M. Cucchetti, M. Rossano, E. Lo Iudice, C. V. Agostoni, G. Filocamo, F. Minoia

#### Fondazione IRCCS Ca' Granda Ospedale Maggiore Policlinico, Milan, Italy

##### **Correspondence:** C. V. Bronzoni

*Pediatric Rheumatology 2023*, **21(Suppl 2):**P049

**Introduction:** Fever is the leading reason for pediatric referral and must be managed with attention, as it fits into a broad differential diagnosis. Recurrent fever in particular is often a challenge involving both general pediatricians (GPs) and specialists; however, a common diagnostic approach is often lacking, especially when autoinflammatory conditions are suspected.

**Objectives:** To investigate GPs perspective and unmet needs in the management of recurrent fevers, particularly in the suspicion of autoinflammatory diseases.

**Methods:** GPs who practice in the Milan area were invited to anonymously complete a 16-question web-survey. The survey was open on May 9^th^ 2023 and is still ongoing. The questionnaire was organized in 3 sections: 1) demographic: to frame physicians’ expertise; 2) clinical: to investigate approach to a patient with recurrent fever; GPs were asked which are the most relevant elements that rise their suspicion on autoinflammatory syndromes and the most relevant variables affecting their decision-making process; 3) educational: to explore potential needs to foster a common approach to recurrent fevers.

**Results:** A total of 59 responses have been collected so far. Most participants are 30-45 years old, work in an outpatient setting and have been practicing as paediatrician for 5-20 years. Almost 70% of GPs wait at least 6 months, and at least 6 episodes, before referring a patient with recurring fever to a specialist. The most relevant items for the diagnosis of PFAPA, selected by more than 90% of participants, were regular timing of episodes, features of pharyngitis, steroidal response, stereotypical features, complete well-being between episodes and negativity of infectious tests. Monogenic autoinflammatory syndromes were suspected by more than 75% of GPs in case of Mediterranean ethnicity (86%), arthro-myalgias (81%) and positive family history (76%). Poor growth (89%), disease onset < 2 years (82%), splenomegaly (79%) and more than 8 infections/year (76%) were considered the most suggestive symptoms of primary immune deficiency. The most employed tool to rule out an infectious disease during an acute febrile episode was the quick strep-test (86%), followed by urine dipstick and acute phase reactants. Before referring a patient with recurrent fever to a specialist, more than 75% of GPs required cell blood count, immunoglobulins, acute phase reactants during episode and in well-being. The most frequently referred specialist was immunologist (86%) followed by rheumatologist (64%). All participants agreed that the educational offer about autoinflammatory disorders needs to be expanded, including useful tests to be performed, criteria for referral to specialists, diary format to track episodes and diagnostic score to identify high risk patients.

**Conclusion:** A common diagnostic approach and an active cross-talk between GPs and specialists are essential to foster a timely referral and a proper management for children with recurrent fevers.

**Patient Consent**

Not applicable (there are no patient data)

**Disclosure of Interest**

C. V. Bronzoni: None declared, L. Baselli: None declared, M. Cucchetti: None declared, M. Rossano: None declared, E. Lo Iudice: None declared, C. V. Agostoni: None declared, G. Filocamo: None declared, F. Minoia Consultant with: SOBI

## P050 Family based phenotype, genotype and functional analysis in adenosine deaminase-2 deficiency, single center study

### S. Çağlayan^1^, T. Coşkuner^1^, M. H. Yarar^2^, B. Sozeri^1^

#### ^1^Pediatric Rheumatology; ^2^Clinical Genetics, Ümraniye Research and Training Hospital, Istanbul, Türkiye

##### **Correspondence:** S. Çağlayan

*Pediatric Rheumatology 2023*, **21(Suppl 2):**P050

**Introduction:** Adenosine deaminase-2 (ADA-2) deficiency is a monogenic autoinflammatory disorder caused by autosomal recessive mutations in the ADA-2 gene. Patients present with a wide spectrum of symptoms including recurrent fever episodes, livedo reticularis/racemosa, immunodeficiency, hematologic involvement, PAN-like clinic, and early onset stroke. The aim of our study was to perform a functional evaluation with both genotypic and enzyme activity level analysis in patients with ADA-2 deficiency and asymptomatic family members.

**Objectives:** he aim of our study was to perform a functional evaluation with both genotypic and enzyme activity level analysis in patients with ADA-2 deficiency and asymptomatic family members.

**Methods:** Patients followed up with the diagnosis of ADA-2 deficiency at the Health Sciences University Umraniye Training and Research Hospital between 2016 and 2022 and their asymptomatic family members were included in the study. The ADA-2 gene and ADA-2 enzyme activity level of all individuals included in the study were analyzed.

**Results:** A total of 12 patients (F/M:5/7) and 9 asymptomatic family members (F/M:7/2) were included in the study. The median age of the patients was 12.1 (min-max: 2.5-22) years, and the median age of the asymptomatic group was 40 (min-max:7-57) years. The median age of onset of complaint, and age at diagnosis were 3.5 (min-max: 1month-14) years, and 8.5 (min-max: 1.7-16) years, respectively. Four (33.3%) patients had mild clinical phenotypes, and eight (66.6%) patients had severe clinical phenotypes. The most common clinical findings were recurrent fever (75%), vascular involvement (66.7%), arthralgia (50%), myalgia (25%), arthritis (25%), abdominal pain (25%), mental retardation (25%), immunodeficiency (25%), Diamond-Blackfan anemia (16.7%), entrapment neuropathy (8.3%) and ischemic stroke (8.3%).

The median ADA-2 enzyme activity level for patients with mild clinical phenotypes, severe clinical phenotypes, and asymptomatic individuals was 4.1 (0-12.1), 0 (0-0.02), and 38.9 (4.4-57.4) mU/g of protein, respectively.

The G47R allele was detected in 4 of 8 patients with a severe clinical phenotype, the S50L allele was detected in 2 patients, the R49fs allele was detected in 1 patient, and the Y453C allele was detected in 1 patient. In nine asymptomatic individuals, four G47R alleles, three Y453C alleles, one K34fs allele, and one R49fs allele were heterozygous. While no statistically significant difference was found between the allele and enzyme activity levels carried by the asymptomatic family members (p=0.690), the lowest enzyme activity level was found in the individual carrying the R49fs allele.

**Conclusion:** In our study, it was determined that the clinical findings in the patients were independent of the genotype and enzyme activity, which was consistent with the literature. Therefore, it was concluded that it is not possible to explain the variation in the phenotype only with the causative mutation or residual enzyme activity level. There is a need for more comprehensive studies on various epigenetic factors that can explain this situation.

**Patient Consent**

Yes, I received consent

**Disclosure of Interest**

None declared

## P051 Pteridine metabolites in patients with familial mediterranean fever

### K. Çalişgan^1^, A. Ç. Aktuğlu Zeybek^2^, K. Barut^3^, M. S. Cansever^4^, T. Zubarioglu^2^, E. Kiykim^2^, E. Isat^5^, Ö. Kasapçopur^3^

#### ^1^Istanbul University-Cerrahpaşa, Cerrahpaşa Faculty of Medicine, istanbul, Türkiye; ^2^Pediatric Nutrition and Metabolism; ^3^Pediatric Rheumatology; ^4^Department of Medical Services and Techniques, Vocational School of Health Services; ^5^Metabolic Diseases Resarch Laboratory, Istanbul University-Cerrahpaşa, Cerrahpaşa Faculty of Medicine, istanbul, Türkiye

##### **Correspondence:** K. Barut

*Pediatric Rheumatology 2023*, **21(Suppl 2):**P051

**Introduction:** Familial Mediterranean Fever (FMF) is an inherited autoinflammatory disease characterized by recurrent fever and serosal inflammation. Pteridine metabolites are formed as a result of the degradation of guanosine triphosphate and can be used as a biomarker in some autoimmune diseases, malignancies and infections. Triptophan is degraded to kynurinine by indolamine 2,3-dioxygenase (IDO) which is induced by the activation of cellular immunity. Kynurinine to triptophan ratio is used as a indicator for IDO enzyme activity, and increased ratios are associated with increased activity of cellular immunity. Dihydropterin reductase (DHPR) is an enzyme responsible for de novo regeneration of tetrahydrobiopterin and its activity is changed in some diseases where the activity of cellular immunity increases.

**Objectives:** To measure serum/urine pteridine metabolites, serum kynurinine and tryptophan levels and blood DHPR activity in FMF patients.

**Methods:** This is a single-center, prospective and cross-sectional study. The study included 81 children who were followed up with the diagnosis of FMF in the pediatric rheumatology outpatient clinic and 66 healthy children as a control group. Thirty-seven patients were evaluated at the attack period, 32 patients at the attack free period, and 12 patients at the subclinical inflammation period. Neopterin, biopterin, isoxanthopterin, pterin-6 carboxilic acid, pterin, monapterin levels were measured from the serum and urine samples of the participants. Concurrent serum kynurinine and tryptophan levels and DHPR activity were also measured. Laboratory findings of FMF patients and healthy controls subjects were compared and evaluated

**Results:** All of the pteridine metabolites measured in the urine were higher in FMF patients compared to healthy subjects. Urinary pteridine metabolites were higher in the patients at attack free period than in the patients at attack and subclinical inflammation periods. All serum pteridine metabolites were found to be higher in the healthy subjects than in the patients. There was no statistical difference for serum pteridine metabolites values between the patients. DHPR activity was found to be lower at the attack period compared to the attack free period. While the ratio of kynurinine/tryptophan did not differ between the patients and the healthy group, it was found to be higher in patients who are at the attack period than those who are attack free.

**Conclusion:** Pteridine metabolites, can be used to demonstrate the systemic inflammation in FMF patients.

**Patient Consent**

Yes, I received consent

**Disclosure of Interest**

None declared

## P052 Cognitive and psychological functioning in children and young adults with deficiency of adenosine deaminase 2

### A. Zanetti^1^, L. Primavera^2^, S. Signa^3^, S. Volpi^3^, P. Moretti^1^, M. Gattorno^3^, M. Bertamino^1^, R. Caorsi^3^

#### ^1^Physical Medicine and Rehabilitation Unit; ^2^Psychology Unit; ^3^UOC Reumatologia e Malattie Autoinfiammatorie, IRCCS Istituto Giannina Gaslini, Genoa, Italy

##### **Correspondence:** R. Caorsi

*Pediatric Rheumatology 2023*, **21(Suppl 2):**P052

**Introduction:** Deficiency of adenosine deaminase 2 (DADA2) is a monogenic autoinflammatory disorder presenting with a broad spectrum of clinical manifestations, including vasculopathy, immunodeficiency, and hematologic disease. Among the vascular manifestations, early-onset strokes play a central role. Few data are available on DADA2 patients’ cognitive and neuropsychological outcome, with or without stroke.

**Objectives:** We aimed to describe the intellectual function of children and young adults with DADA2 followed by a single Italian Rheumatology Center, confronting the outcomes between patients with and without stroke. Furthermore, the prevalence of emotional and behavioral problems was examined within the cohort.

**Methods:** In this observational study we enrolled all DADA2 patients undergoing regular follow-up at Rheumatology Center in our Institute. The general intellectual outcome was assessed by means of the age-appropriate Wechsler Scales: Wechsler Intelligence Scale for Children Fourth edition: WISC IV, and Wechsler Adult Intelligence Scale Fourth edition: WAIS IV. Emotional and behavioral problems incidence was assessed through the patients’ parents filling out the Childhood Behavior Check List (CBCL/6-18) and the Adult Behavior Check List (ABCL/18-59) questionnaires.

**Results:** Nine out of 11 patients gave their consent to the evaluation. Age at the time of evaluation was between 10 and 26 years with 5 out of 9 patients underaged (<18 years old); 5 out of 9 examined patients experienced a stroke between 6 and 17 years of age. Overall, full-scale IQ observed in DADA2 patients ranged from 50 to 116. Patients with a history of stroke (n=5) presented poorer results for visuospatial functions compared to linguistic ones, consistent with literature on pediatric stroke patients, which reports a better recovery of left-hemisphere functions, generally associated to language lateralization. Conversely, patients who did not experience a stroke (n=4) presented an opposite cognitive profile, characterized by better visuospatial skills and lower verbal abilities, not concordant with the preliminary findings on patients suffering from chronic systemic diseases (i.e. systemic juvenile idiopathic arthritis).

Regarding emotional and behavioral problems, 5/9 patients (55.5%) presented with internalizing problems and 2/9 with externalizing problems (22.2%). Among patients showing internalizing problems, two patients showed an intellectual profile within the average range; the three patients with the lowest full-scale IQ showed scores above the clinical cut-off at the internalizing problems scale, and one of them also at the externalizing problems scale.

**Conclusion:** DADA2 patients in our cohort showed different intellectual outcomes depending on the presence or absence of a previous stroke. Differently from patients suffering from other chronic systemic diseases, we found a peculiar profile characterized by lower verbal abilities in patients without stroke. Therefore, we suggest to routinely add to the follow-up of DADA2 patients the assessment of cognitive profile and of emotional and behavioral problems, even in patients who did not experience a stroke. Larger multicentric studies are needed to validate our observation.

**Patient Consent**

Not applicable (there are no patient data)

**Disclosure of Interest**

None declared

## P053 Idiopathic pericarditis: anakinra as a first line of treatment

### C. Celani, S. Federici, M. F. Natale, F. De Benedetti, A. Insalaco

#### Division of Rheumathology, IRCCS Ospedale Pediatrico Bambino Gesù , Rome, Italy

##### **Correspondence:** M. Natale

*Pediatric Rheumatology 2023*, **21(Suppl 2):**P053

**Introduction:** Acute idhiopatic pericarditis is a difficult clinical problem that can relapse in 30% of patients. According to the last guidelines of European Society of Cardiology (ESC) anti-inflammatory drugs (NSAIDs) represent the first line treatment for acute pericarditis, alone or with low doses of colchicine. Glucocorticoids are not recommended as first line treatment increasing the risk of chronicity and recurrences. The role of IL 1 blocker Anakinra in pericarditis has been described in children with recurrent pericarditis resistant to colchicine and glucocorticoid dependent in different series of paediatric and adult patients but is never described as a first line of treatment

**Objectives:** To describe 5 patients who was treated with Anakinra at first episode of idiopathic pericarditis and to analyse the response to the treatment and the number of flares after the beginning of therapy

**Methods:** We retrospective collected 5 patients (3 males) with a diagnosis of acute pericarditis that was treated with Anakinra as first line of treatment. Pericarditis was diagnosed according to the ESC guidelines. One patient with postoperative pericarditis was also included. We also compared these patients with others 5 patients who were treated with NSAIDs/colchicine or glucocorticoids alone as first line of treatment. during one year of follow-up.

**Results:** We collected 5 patients with a first episode of acute pericarditis who received anti IL1 blocking agent as first line of treatment together with NSAID. The median age at disease onset was 12.6 years (range 11-15 years). After the beginning of treatment with Anakinra all patients showed a complete response in terms of clinical manifestation, reduction of pericardial effusion and normalization of laboratory variables. They were discharged in a mean time of 8.6±4 days and during the daily treatment with anakinra no relapses were noted during 1 year of follow-up. All patients were also able to discontinue antiinflammatory drugs a few days after Anakinra was initiated. We compared these patients with other 5 patients (median age 13.4 years range 12-15 years) with acute pericarditis treated at the first episode with NSAID/colchicine or glucocorticoids.This group of patients relapsed almost 2 times or 1 time before beginning Anakinra during one year of follow-up. Because of the flares these patients were a hospitalized once or 2 times during the same year.

**Conclusion:** We describe five patients with a first episode of idiopathic acute pericarditis in which Anakinra was administrated as first line of treatment with a complete response in few days and with the ability to discontinue the other drugs. During the daily treatment with anakinra no relapses were noted for one year. The problem of the treatment of idiopathic pericarditis is known and it is important to establish an optimal therapeutic regimen to avoid recurrences, to reduce duration of hospitalization and the accesses to the emergency room. The use of anakinra as first line of treatment in idiopathic pericarditis can be helpful to control relapses and to avoid glucocorticoid dependence but longitudinal studies are necessary to establish the best possible approach to prevent relapses during anakinra tapering or withdrawal.

**Patient Consent**

Yes, I received consent

**Disclosure of Interest**

None declared

## P054 Four cases of chronic non-bacterial osteomyelitis associated with systemic vasculitis

### C. Celani, V. Messia, M. Pardeo, S. Federici, M. F. Natale, F. De Benedetti, A. Insalaco

#### Division of Rheumathology, IRCCS Ospedale Pediatrico Bambino Gesù, Rome, Italy

##### **Correspondence:** M. Natale

*Pediatric Rheumatology 2023*, **21(Suppl 2):**P054

**Introduction:** Chronic non-bacterial osteomyelitis (CNO) is an auto-inflammatory bone disorder presenting with a wide spectrum of clinical manifestations. It usually presents with recurrent bone pain in one or multiple sites and is often associated with other chronic inflammatory conditions including arthritis, inflammatory bowel disease and/or skin involvement (psoriasis, pustulosis palmaris et plantaris, pyoderma gangrenosum, severe acne). Magnetic resonance imaging (MRI) is the gold standard radiological tool for the diagnosis. We describe four patients affected by CNO and vasculitis.

**Objectives:** To describe four pediatric cases of CNO in which a diagnosis of vasculitis was made: 3 polyarteritis nodosa (PAN) and 1 Takayasu arteritis (TAK).

**Methods:** Demographic clinical, laboratory and instrumental, characteristics were collected for each patients

**Results:** We report four pediatric patients that were referred to our rheumatology department: 3 with recurrent bone pain and 1 with a systemic inflammatory clinical picture (persistent fever, gastrointestinal symptoms and rash). All patients, presented at onset multifocal lesions at MRI and elevated markers of inflammation: median C-reactive protein(CRP) was 8.74 mg/dl (2.59-14.8), erythrocyte sedimentation rate (ESR) 61 (94-35) and serum amyloid-A (SAA) 439 (662-106). Bone biopsies, performed in all patients, were consistent with the diagnosis of chronic osteomyelitis. Only in one patient vasculitis (PAN) preceded the appearance of CNO,that was diagnosed 5 years later based on clinical symptoms and MRI

**Conclusion:** We describe four patients with CNO and vasculitis, three with PAN and one with TAK. Nowaday the association between CNO and vasculitis is reported only in a few clinical cases and only one with Takayasu arteritis. Interesting to note that all three patients in which diagnosis of CNO preceded the appearance of vasculitis, presented at the onset a clinical picture characterized by systemic inflammation with fever and elevated markers of inflammation; this kind of presentation, although described, is not very common in CNO patients. All patients moreover presented at the onset of the disease multiple bone lesions. These data suggest the importance to follow with particular attention patients affected by CNO, especially that with multifocal involvement and increased markers of inflammation at the onset ,to highlight precociously any sign of systemic vasculitis. Further study would be helpful to demonstrate this association.

**Patient Consent**

Yes, I received consent

**Disclosure of Interest**

None declared

## P055 Transmission of Cryopyrin-Associated Periodic Syndromes (CAPS) from asymptomatic somatic mosaicism

### G. Côte, E. Merlin

#### Chu Estaing, Clermont-Ferrand, France

##### **Correspondence:** G. Côte

*Pediatric Rheumatology 2023*, **21(Suppl 2):**P055

**Introduction:** The cryopyrin-associated periodic syndromes (CAPS) are a set of diseases usually caused by heterozygous mutation of NLRP3 gene which leads to excessive inflammasome activation.

**Results:** A 14 years-old female patient presented episodes of arthralgia associated with urticaria-like rash, and red sore eyes, without fever. She also suffered from recurring headaches, progressive hearing loss and decreased visual acuity. Treatment with anti-interleukin 1 agent enabled to reach clinical remission and keep the disease under control. DNA analysis by next generation sequencing revealed a heterozygous mutation c.1693 A>G (Lys565Glu) in NLRP3 gene which is compatible with the diagnosis of CAPS. DNA analysis of the father showed that he carries the same mutation in somatic mosaicism (11% of cells), although he has never presented any symptom that can be linked to the CAPS.

**Conclusion:** This case shows a new variant of mutation in the NLRP3 gene inherited from asymptomatic somatic mosaicism.

**Patient Consent**

Yes, I received consent

**Disclosure of Interest**

None declared

**Reference**

1. Tatjana Welzel and Jasmin B. Kuemmerle-Deschner, Diagnosis and Management of the Cryopyrin-Associated Periodic Syndromes (CAPS): What Do We Know Today?, J. Clin. Med, 2021, 10(1), 128

## P056 Flow cytometry on the track of interferon: measure of siglec1 expression in rheumatological conditions, infections and healthy controls

### F. Burlo^1^, M. Di Rosa^2^, M. Padovan^1^, V. Boz^2^, L. De Nardi^1^, F. Vittoria^2^, P. Di Rocco^2^, S. Pastore^2^, A. Taddio^1,2^, A. Amaddeo^2^, A. Tommasini^1,2^, A. Tesser^2^, E. Valencic^2^

#### ^1^University of Trieste; ^2^Institute for Maternal and Child Health, IRCCS “Burlo Garofolo”, Trieste, Italy

##### **Correspondence:** L. De Nardi

*Pediatric Rheumatology 2023*, **21(Suppl 2):**P056

**Introduction:** Cells exposed to interferon-mediated inflammation display hyperexpression of interferon stimulated genes (ISGs), whose measure allows calculating the so-called interferon score (IS). The IS measure has gained attention to screen monogenic interferonopathies and to stratify rheumatologic diseases ^1.^ However, the availability of the assay to only a few centers hindered a full evaluation of its limitations and potential. Measurement of the expression of Siglec1 on circulating monocytes by flow cytometry may be an alternative to IS with lower cost and rapid reporting time. However, apart from few reports on inflammatory myopathies, experience comparing the two methods is limited ^2.^

**Objectives:** To compare the cytometric Siglec1 assay with IS on patients with rheumatologic diseases, infections, and healthy controls.

**Methods:** Patients with rheumatological conditions; patients with acute infections; subjects undergoing blood draw for preoperative examinations not related to immunopathologic problems and healthy adult controls. Measure of the interferon score based on six ISGs by RT-PCR and assessment of monocyte expression of Siglec1 by flow cytometry. Statistical analysis: correlation of the two examinations and comparison of Siglec1 expression in different groups. Analysis of cases with discrepant results.

**Results:** We enrolled 98 subjects (30 of whom with rheumatological disorders, median age 14.1 years, female 29; 20 with acute infections, median age 5.7 years, female 20; 27 controls, median age 14.6 years, 14 female) with a total of 104 Siglec1 measures by flow cytometry and 42 assessment of the IS by RT-PCR.There was a good positive correlation between the two assays (R square of 80%). All the subjects with a normalized mean fluorescence intensity (MFI) greater than 1 had also a positive IS (> 4.5), whilst 3 subjects (a girl with SLE, a girl with COPA and a boy with HA20) had a positive IS (respectively 7.3, 5.9 and 6.5) with normal expression of Siglec1. When analyzing single ISG expression, these three subjects tended to display a lower amount of Siglec1 expression also by RT-PCR.Subjects with acute infections tended to present higher cytometric levels of Siglec1 than subjects with rheumatological conditions (respectively average MFI 8.0 and 3.4, p=0.12).

**Conclusion:** The measure of Siglec1 by flow cytometry has a good correlation with the measure of the IS and can be a convenient assay to obtain rapid results in most laboratories. The assay may not be informative if performed during acute infectious illness. One limitation of our study is that almost all patients with rheumatologic conditions analyzed were already on treatment, usually with good control of the disease. Expanding the study on a larger number of patients both at onset of disease and during follow up may help disclosing the potential of this assay in clinical practice.

**Patient Consent**

Not applicable (there are no patient data)

**Disclosure of Interest**

None declared

**References**

1. Rice GI, et al. Assessment of Type I Interferon Signaling in Pediatric Inflammatory Disease. J Clin Immunol. 2017 Feb;37(2):123-132.
2. Graf M, et al. SIGLEC1 enables straightforward assessment of type I interferon activity in idiopathic inflammatory myopathies. RMD Open. 2022 Feb;8(1):e001934.

## P057 Preliminary results: colchicine plasma and target tissue concentrations in patients with FMF

### M. Romano^1^, B. Eser^2^, N. Zitoun^1^, D. Piskin^1^, F. Garcia-Bournissen^1^, E. Demirkaya^1^

#### ^1^Schulich School of Medicine, Western University, London, Canada; ^2^University of Health Sciences, Ankara, Türkiye

##### **Correspondence:** E. Demirkaya

*Pediatric Rheumatology 2023*, **21(Suppl 2):**P057

**Introduction:** Familial Mediterranean Fever (FMF) is the most common autoinflammatory disease, characterized by recurrent self-limited attacks of fever lasting 2-3 days. Colchicine, the mainstay of therapy, decreases the number of attacks and prevents complications such as amyloidosis. Approximately 15% of patients are considered to be colchicine resistant or intolerant.

**Objectives:** To study colchicine plasma and neutrophils concentrations in FMF patients, and to model the pharmacokinetics–pharmacodynamics relationships in colchicine responders and non-responders.

**Methods:** A cohort of FMF patients provided data for this study. Two groups were established according to colchicine response (responders and non-responders). Intracellular and serum colchicine measurements were performed using LCMS/ MS (Agilent 6420) with liquid-liquid extraction. Colchicine trough and peak (i.e. 2 hours after dose) measurements were obtained from each patient.

**Results:** A total of 142 patients were enrolled in the cohort (50% males). The median age was 19 years (IQR 14-33.5), the median weight was 57 kg (IQR 45.5–73.5). The median colchicine dose was 1.5 mg/day (IQR 1.5–2), and the median weight-corrected colchicine dose was 0.0294 mg/kg/day (IQR 0.0226–0.0385). 58% of patients took the medication BID, 32% TID, and the rest either OD or 4 times/day. There were no statistically significant differences in weight-adjusted colchicine dose between males and females. Median plasma colchicine levels were 0.76 ng/ml at trough (IQR 0.46–1.18), and 2.7 ng/ml at peak (IQR 1.55–3.7). Median neutrophil colchicine concentrations (N=21) were 0.36 (IQR 0.07–0.39) at a trough and 0.37 (IQR 0.09–0.41) at peak. Median lymphocyte concentrations (N=21) were similar to those of the neutrophils: trough 0.348 ng/ml (IQR 0.35–0.40) and peak 0.36 ng/ml (IQR 0.07–0.44). No correlations were observed between plasma and neutrophils or lymphocytes colchicine concentrations at trough or peak. Neutrophils and lymphocyte concentrations were highly correlated at both trough and peak, with Spearman rank rho=0.67, p<0.001 and rho=0.88 p<0.001, respectively. Plasma colchicine concentrations at trough did not differ significantly between responsive (N=51) and non-responsive (N=87) patients, but they were significantly higher in non-responsive patients at peak (p=0.012), with a median colchicine peak plasma concentration of 2.94 ng/ml (IQR 1.79 – 4.18) in non-responsive vs 2.08 (IQR 1.28 – 3.22) in responsive patients. Similarly, neutrophils colchicine concentrations were not statistically different between responsive and non-responsive patients at the trough, but peak intracellular neutrophil concentrations were significantly higher at peak in non-responsive patients (p=0.021), with median concentrations of 0.394 (IQR 0.2 – 0.42) vs 0.025 (IQR 0.023 – 0.173) in non-responsive vs responsive patients respectively.

**Conclusion:** We successfully quantified colchicine levels in serum and target cells. Interestingly, we observed that a subgroup of non-responders had serum colchicine levels higher than responders. These findings suggest that colchicine concentrations can be used to decide which non-responders could still benefit from higher dosages. Further analysis of our data will define clinically relevant therapeutic ranges for colchicine.

**Patient Consent**

Yes, I received consent

**Disclosure of Interest**

None declared

## P058 Phenotypic and genotypic characteristics of patients with DNASE1L3 deficiency: preliminary results from the eurofever registry

### E. Drago^1^, L. Breda^2^, R. Caorsi^3^, G. Di Donato^2^, S. La Bella^2^, F. Madia^4^, A. AlSaleem^5^, P. Fenaroli^6^, A. Vaglio^7,8^, S. M. Al-Mayouf^9^, N. Ruperto^10^, M. Gattorno^3^, S. Volpi^1,3^

#### ^1^Department of Neuroscience, Rehabilitation, Ophthalmology, Genetics, Maternal and Child Health (DINOGMI), University of Genoa, Genova; ^2^Paediatric Department, University of Chieti "G. D'Annunzio", Chieti; ^3^Center for autoinflammatory diseases and immunodeficiencies; ^4^Medical Genetics Unit, IRCCS Istituto Giannina Gaslini, Genova, Italy; ^5^Department of Pediatric Rheumatology, King Faisal Specialist Hospital and Research Center, Riyadh, Saudi Arabia; ^6^Nephrology Unit, University Hospital, Parma; ^7^Department of Biomedical, Experimental and Clinical Sciences "Mario Serio", University of Florence; ^8^Medical Genetics Unit, Meyer Children's Hospital, Florence, Italy; ^9^Department of Pediatric Rheumatology, King Faisal Specialist Hospital and Research Center, Riyadh, Saudi Arabia; ^10^UOSID Centro Trial, PRINTO, IRCCS Istituto Giannina Gaslini, Genova, Italy

##### **Correspondence:** E. Drago

*Pediatric Rheumatology 2023*, **21(Suppl 2):**P058

**Introduction:** Null mutations in DNASE1L3 cause a rare type 1 interferonopathy, typically associated with juvenile systemic lupus erythematous (jSLE) with renal involvement or hypocomplementemic urticarial vasculitis syndrome (HUVS)[1,2]. Due to the severity of the renal involvement, which often leads to a pediatric end-stage renal disease (ESRD), a better characterization of the early stages of disease and prompt therapeutic approach appears to be crucial to improve prognosis.

**Objectives:** This study was undertaken to describe the genotype, phenotype, and response to treatment in an international cohort of DNASE1L3 deficiency.

**Methods:** All patients with DNASE1L3 deficiency are being enrolled from 2009 in the Eurofever registry. Epidemiologic, demographic, biochemical and clinical data were collected by local physicians and anonymized. Independent ethics committee approval for enrolling patients was granted in accordance with local requirements.

**Results:** 13 patients with DNASE1L3 deficiency from 4 Centers are currently enrolled in Eurofever. Six out of 13 of the patients have complete and available data. The median age at disease onset was 5.5 years (IQR 3.6-8.1 years), with a median diagnostic delay of 6.0 years (IQR 4.7-18.75 years). The most frequent mutations appear to be deletions at nucleotides 289 to 291. The most frequent phenotype found was jSLE with renal involvement. All 3 patients with jSLE resulted in pediatric-ESRD with subsequent kidney transplantation. Pt3 before KT was on treatment with ruxolitinib, with good control of the inflammatory component but progression of renal damage (serum creatinine at start of therapy: 2.88 mg/dl, eGFR 20mL/min/1.73m2). 2/6 patients presented with an undefined inflammatory phenotype, characterized by urticarial rash, lymphoproliferation and recurrent fever. Serositis (Pt2) and Jaccoud arthropathy (Pt5) are features of patients with DNASE1L3 deficiency.

**Conclusion:** Preliminary results of the study confirm that the widespread use of NGS panels and whole exome sequencing allow earlier genetic diagnosis, thus broadening the phenotypic spectrum in the early stages. Increasing the cohort it will be important to define whether early use of drugs that act on the interferon pathway, such as JAK-inhibitor, could be promising for preventing progression of the renal damage.

**Patient Consent**

Yes, I received consent

**Disclosure of Interest**

None declared

**References**

1. Tusseau M, Lovšin E, Samaille C, et al. DNASE1L3 deficiency, new phenotypes, and evidence for a transient type I IFN signaling. J Clin Immunol. 2022;42(6):1310-1320.
2. Al-Mayouf SM, Sunker A, Abdwani R, et al. Loss-of-function variant in DNASE1L3 causes a familial form of systemic lupus erythematosus. Nat Genet. 2011;43(12):1186-1188. Published 2011 Oct 23.

## P059 Majeed syndrome: first description in a patient of European ancestry

### E. Drago^1^, A. Bertoni^2^, A. Grossi^3^, M. B. Damasio^4^, S. Volpi^1,2^, M. Miano^5^, I. Ceccherini^3^, M. Gattorno^2^, R. Caorsi^2^

#### ^1^Department of Neuroscience, Rehabilitation, Ophthalmology, Genetics, Maternal and Child Health (DINOGMI), University of Genoa; ^2^Center for autoinflammatory diseases and immunodeficiencies; ^3^UOSD Laboratory of Genetics and Genomics of Rare Diseases; ^4^Department of Radiology; ^5^Hematology Unit, IRCCS Istituto Giannina Gaslini, Genova, Italy

##### **Correspondence:** E. Drago

*Pediatric Rheumatology 2023*, **21(Suppl 2):**P059

**Introduction:** Majtext-breakeed syndrome is a monogenic autoinflammatory disease caused by loss-of-function mutations in LPIN2. Clinically it manifests with triad of early onset chronic recurrent multifocal osteomyelitis (CRMO), congenital dyserythropoietic anemia (CDA) and Sweet syndrome. In vitro studies revealed that lipin-2 is an innate immune regulator through a control on activation of the inflammasome NLRP3^1^. So far, only patients from Middle Eastern countries with Majeed syndrome have been described in the literature, except for one patient of African-American descent^2^.

**Objectives:** To present the first case of a Majeed syndrome in a girl of European ancestry.

**Methods:** Patient’s medical records were reviewed. A NGS panel for autoinflammatory diseases was performed and the mutation was confirmed by Sanger analysis. Freshly isolated monocytes were activated with LPS +/- ATP. The concentration of cytokines was assessed in monocytes supernatants by using R&D Systems kits specific for IL-1β, IL-1α, TNF-α and IL-1RA.

**Results:** A 2-year-old girl presented with recurrent pain in the lower limbs, increase of acute phase reactants and persistent microcytic anemia. She was the first child of non-consanguineous Italian parents. The MRI showed bilateral STIR hyper-intensity and contrast enhancement of the spongy osseous tissue of femur distal diaphysis and tibial proximal metaphysis. The whole-body MRI detected similar bilateral findings in radius, ulna, astragalus. To further investigate the clinical picture and exclude malignancy, bone marrow aspirate, trephine biopsy and bone biopsy of the proximal right tibial were performed. Bone marrow analysis showed increased trilinear cellularity with ineffective erythropoiesis and signs of CDA as many bi/trinuclear erythroblasts and inter-nuclear bridging. The bone biopsy showed chronic inflammatory infiltrate with diffuse fibrosclerosis. Microbiological investigations were negative. NGS panel detected the presence of two novel compound heterozygous mutations in LPIN2 gene, confirmed by Sanger analysis: c.2144_2145dupGT, inherited from the mother, and c.1693_1694delGA frameshift, inherited from the father. Treatment with anakinra was started with a prompt resolution of the clinical picture. Increased kinetics and concentration of IL-1β was observed in the patient's monocytes before therapy than in healthy controls. In contrast, after three months of treatment, patient’s monocytes secreted less IL-1β compared to healthy controls. About six months after the start of therapy, resolution of microcytic anemia was observed with marked improvement in dyserythropoiesis at bone marrow aspirate.

**Conclusion:** We describe the first case of Majeed syndrome of a patient with European ancestry. The functional test performed on circulating monocytes before and after therapy with anakinra confirms that the novel LPIN2 mutations detected are pathogenetic, since the results are similar to those obtain in other patients with this condition, thus confirming the role of LPIN2 in the NLRP3 inflammasome activation. As expected, anti-IL1 agents were effective, leading not only to the resolution of bone lesion but also to an improvement of dyserythropoiesis.

**Patient Consent**

Yes, I received consent

**Disclosure of Interest**

None declared

## P060 Investigation of autoinflammation-related cardiac autonomic dysfunction in patients followed up with familial mediterranean fever

### M. Ergene^1^, E. Baskın^2^, M. Demir^3^, K. S. Gülleroğlu^4^, B. Varan^3^

#### ^1^Pediatrics, Sincan State Hospital; ^2^Pediatric Rheumatology; ^3^Pediatric Cardiology; ^4^Pediatric Nephrology, Baskent University, Ankara, Türkiye

##### **Correspondence:** M. Ergene

*Pediatric Rheumatology 2023*, **21(Suppl 2):**P060

**Introduction:** In recent studies, ECG (Electrocardiography) parameters showing the presence of cardiac autonomic dysfunction in FMF patients have been investigated. For this purpose, it has been suggested that cardiac dysfunction parameters such as corrected QT dispersion (QTcd) and corrected JT dispersion (JTcd) on ECG may be useful.

**Objectives:** In our study, we evaluated patients with a diagnosis of FMF with their epidemiological, clinical and laboratory characteristics and aimed to investigate the importance of cardiac autonomic dysfunction by examining some ECG parameters, which are accepted as electrocardiographic markers for ventricular arrhythmias.

**Methods:** A group of 63 pediatric patients aged between 0-18 years, who applied to the Department of Pediatric Rheumatology, Department of Pediatrics, Başkent University Ankara Hospital, between January 2010 and December 2021, were diagnosed with FMF and then applied to the Pediatric Cardiology Polyclinic for general control purposes, as a group of 63 pediatric patients. 63 healthy children without chronic disease were included in the study as the control group. FMF patients were selected from genetically homozygous or combined heterozygous patients who were followed for at least two years, did not have amyloidosis, and were in good general condition in the attack-free period. The 12-lead ECGs of both the patient and control groups were taken and heart rate, QTcmin, QTcmax, JTcmin, JTcmax, QTc dispersion, and JTc dispersion were evaluated as ECG parameters. The relationship of ECG parameters with clinical, laboratory and genetic results was investigated.

**Results:** The mean age of the patients was calculated as 12.1±4.24 years and the mean age of the control group as 13.21±2.90 years. The mean age at diagnosis of the patients was 5.76±4.43 years, the mean follow-up period was 6.41±4.06 years and the mean duration of colchicine treatment was 6.54±3.82 years. The gender, age, body weight and height levels of the patient and control groups were found to be similar (p>0.05). CRP (mg/L) and sedimentation measurement levels in the patient group were significantly higher than in the control group (p<0.05). In addition, heart rate (/min) and corrected JT dispersion (ms) measurement levels were found to be significantly higher in the patient group than in the control group (p<0.05). When the ECG parameters of patients with at least one M694V mutation in at least one allele and the control group were compared, heart rate and JTcd measurement levels were found to be significantly higher (p<0.05).

**Conclusion:** Our study showed that JTcd, which is accepted as an ECG indicator of the deterioration in the cardiac conduction system, is higher in FMF patients compared to the control group. This result suggests that the cardiac conduction system begins to be affected in FMF patients, even though they do not show symptoms in childhood.

**Patient Consent**

Yes, I received consent

**Disclosure of Interest**

None declared

**References**

1. Tufan A., Lachmann H. Familial Mediterranean Fever from pathogenesis to treatment: a contemporary review, Turk J Med Sci, 50:1591-1610, 2020
2. Akçay A., Acar G., Sayarlıoğlu M., Sokmen A., Kaya H., Ispiroğlu M., Koroğlu S., QT dispersion and transmural dispersion of repolarisation in patients with familial Mediterranean fever, Mod Rheumatology 19:550-555, 2009
3. Tunca M, Akar S., Onen F., Familial Mediterranean fever (FMF) in Turkey: results of a nationwide multicenter study. Medicine 84:1-11, 2005
4. Kucuk A., Gezer I., Ucar R., Karahan A. Familial Mediteranean Fever, Acta Medica 57(3): 97-104, 2014

## P061 Clinical characteristics of a portuguese cohort with undefined autoinflammatory diseases

### M. S. Faria^1,2^, C. Zinterl^1,2^, R. Campanilho-Marques^1,2,3^, I. C. Esteves^4^, J. G. Marques^4^, P. C. Reis^3,4,5^, M. Conde^6^, F. O. Ramos^1,2,3^

#### ^1^Paediatric Rheumatology Unit; ^2^Rheumatology Department, Centro Hospitalar Universitário Lisboa Norte; ^3^Instituto de Medicina Molecular, Faculdade de Medicina da Universidade de Lisboa, Centro Académico de Medicina de Lisboa; ^4^Paediatric Department; ^5^Paediatric Rheumatology Unit, Paediatric Department, Centro Hospitalar Universitário Lisboa Norte; ^6^Paediatric Rheumatology Unit, Paediatric Department, Centro Hospitalar Universitário Lisboa Central, Lisbon, Portugal

##### **Correspondence:** M. S. Faria

*Pediatric Rheumatology 2023*, **21(Suppl 2):**P061

**Introduction:** Systemic autoinflammatory diseases (SAIDs) are rare disorders characterized by periodic or chronic multisystemic inflammation. A distinct diagnosis cannot be met in half of these patients, being classified as undefined SAIDs (uSAIDs).

**Objectives:** To evaluate the clinical features and treatment response of a uSAID portuguese cohort.

**Methods:** Patients with recurrent or persistent episodes of systemic inflammation associated with serum acute phase reactants elevation who do not meet the PRINTO diagnosis criteria for any well-defined SAIDs were classified as having uSAID. Patients who do not have any pathogenic gene mutation or have one variant of uncertain significance (VUS) of a gene related to an autosomal dominant SAID were included. Categorical variables are described as frequencies and percentages and continuous variables as median (IQR1, IQR3).

**Results:** This study included 22 patients (12 females and 10 males). The median age at disease onset was 3.5 (IQR: 1.5, 8.0) years old and the median time of disease duration was 6.7 (IQR: 4.3, 12.1) years. Most patients (n=12) had recurrent episodes lasting 2 to 5 days. Three patients had persistent inflammation. Regarding the 19 patients with recurrent inflammation, 8 (42.1%) had more than 12 episodes per year and the intercritical interval was regular in 10 (52.6%). Besides fever, the most commonly reported manifestations were mucocutaneous lesions (n=25) including mouth ulcers (n=11), arthralgia/arthritis (n=22), abdominal pain (n=13), and lymphadenopathy (n=9). Concerning genetic analysis, 11 were found to have a VUS in genes associated with SAIDs or mutations in unrelated genes. The most frequently used therapy was oral steroids (n=17) with favourable clinical response in 13 patients and 2 exhibited complete remission with oral steroids on demand. Colchicine led to a complete remission/decrease in the number of episodes in 9 of the 15 treated patients. Anakinra was used in 4 patients - 1 complete remission as a first line steroid-sparing agent; 1 partial response in a patient that had been treated with steroids, colchicine and sulfasalazine with no response; and ineffective as a first line steroid-sparing agent in a patient with persistent disease and as a third line after cyclophosphamide and azathioprine. The two patients treated with canakinumab (as a first line in a patient with persistent disease and as a second line in a patient that had not responded to anakinra) achieved complete remission. None of the 3 patients submitted to tonsillectomy achieved remission.

**Conclusion:** Similarly to other cohorts, mucocutaneous lesions, abdominal pain and arthralgia were frequently reported. Despite the frequency of mouth ulcers and lymphadenopathy, the preponderance of abdominal pain and the low-rate response to on-demand steroid therapy contrasts to PFAPA. Most patients had a favourable response to colchicine and IL-1 inhibitors.

**Patient Consent**

Not applicable (there are no patient data)

**Disclosure of Interest**

None declared

## P062 Association of clinical phenotype and laboratory markers with TNFAIP3 variants in patients affected by haploinsufficiency of A20 (HA20): a monocentric study

### S. Federici^1^, C. Celani^1^, M. F. Natale^1^, C. Passarelli^2^, I. Caiello^3^, G. Prencipe^3^, F. De Benedetti^1,3^, A. Insalaco^1^

#### ^1^Division of Rheumatology; ^2^Laboratory of medical genetic, ^3^Laboratory of Immuno-Rheumatology, IRCCS Ospedale pediatrico Bambino Gesù, Rome, Italy

##### **Correspondence:** S. Federici

*Pediatric Rheumatology 2023*, **21(Suppl 2):**P062

**Introduction:** HA20 is an autoinflammatory disease caused by heterozygous mutations in *TNFAIP3*, encoding A20, a negative regulator of inflammation. Several cases have been reported and the phenotype expanded to include not only autoinflammatory but also autoimmune manifestations. We previously described markedly high circulating levels of CXCL9 and CXCL10 in a family with HA20

**Objectives:** To describe a monocentric cohort of HA20 patients, to assess the pathogenicity of previously undescribed variants and to evaluate the association between the clinical phenotype, laboratory data and the disrupted domain of A20. We also evaluated if CXCL9 and 10 can be used as a marker of the disease

**Methods:** We collected 18 subjects from 8 families carrying variants in the *TNFAIP3* gene and measured the circulating levels of CXCL9 and CXCL10

**Results:** Patients of family 1-2 show a predominantly autoimmune phenotype. Pts 1-4 are consanguineous with several autoimmune disease. All had recurrent oral ulcers and 1 also recurrent fever^1^. Pts 5-6 include a patient with type I diabetes, celiac disease, tiroiditis, cerebral vasculitis, atrophic autoimmune gastritis and her clinically asymptomatic mother. Patient of family 3-4 showed a prevalent bowel involvement. Pts 7-8 include a child with a very early onset inflammatory bowel disease, oral and genital ulcers and arthralgias and her father with recurrent fever and oral and genital ulcers. Pts 9-11 include 2 children and their mother with a severe inflammatory bowel diseases, inflammatory cutaneous manifestations, arthritis and immunological alterations. Pts 12-13 include a child and her mother with recurrent fever, oral and genital ulcers and arthromyalgias. Patient 14 presented a chronic arthritis/tenosynovitis with elevation of acute phase reactants and patient 16 with late onset recurrent fever, abdominal pain and mild chronic gastritis and colitis. The parents of these patients (pts 15,17) were asymptomatic. Finally, patient 18 presented with recurrent pericarditis and celiac disease. 7 out of the 9 variant found were not previously reported. 3/9 variants causing a stop codon are classified as pathogenic (class 4-5) while the other missense mutations as VUS/benign (class 2-3). CXCL9 and CXCL10 resulted markedly elevated in 7/8 patients with stop codon variants and in 2 families carrying missense variants (except pts 11) but in normal range in the other patients

**Conclusion:** A marked clinical heterogeneity even among patients with the same variant was observed and the spectrum of clinical manifestations is broadening. We haven’t found a clear correlation between the localization of the variant and the clinical picture. CXCL9 and 10 resulted markedly elevated in the majority of more severely affected patients but not detectable in others clearly symptomatic, even in pt 16 carrying a variant previously described as pathogenic. Further studies are needed to assess the validity of these cytokines as markers of this disease

**Patient Consent**

Yes, I received consent

**Disclosure of Interest**

None declared

**Reference**

1. Front Immunol. 2022 Jan 26

## P063 MEFV gene variation R202Q is common in patients with familial mediterranean fever in Greece

### K. Kourtesi^1^, M. Economidou^2,3^, A. Papadopoulou^4^, A. Antoniou^5^, E. Atsali^1^, V. Papaevangelou^6^, P. Kollia^3^, L. Fotis^1^

#### ^1^Department of Pediatrics, Division of Pediatric Rheumatology, National and Kapodistrian University of Athens, ATTIKON General University Hospital; ^2^Laboratory of Clinical Biochemistry, National and Kapodostrian University of Athens, ATTIKON General University Hospital; ^3^Department of Genetics and Biotechnology, National and Kapodistrian University of Athens, Faculty of Biology; ^4^Laboratory of Clinical Biochemistry; ^5^2nd Department of Psychiatry; ^6^Department of Pediatrics, National and Kapodistrian University of Athens, ATTIKON General University Hospital, Athens, Greece

##### **Correspondence:** L. Fotis

*Pediatric Rheumatology 2023*, **21(Suppl 2):**P063

**Introduction:** R202Q polymorphism of the *MEFV* gene has been characterized as a benign variant rather than a causative pathogenic mutation of Familial Mediterranean Fever (FMF). Its frequency in healthy population ranges from 0.0432 to 0.3621, depending on the population studied (1). Moreover, underlying *MEFV* mutations could affect periodic fever, aphthous stomatitis, pharyngitis, and adenitis (PFAPA) syndrome’s.

**Objectives:** The objective is to examine the genotype distributions of *MEFV* variants in patients with FMF and PFAPA and particularly R202Q polymorphism.

**Methods:** The records of patients with a clinical diagnosis of FMF and PFAPA genetically tested with Sanger sequencing where retrospectively reviewed. Diagnosis was based on the Eurofever/PRINTO classification criteria.

**Results:** 132 patients were genetically tested for a periodic fever syndrome from 01/01/2018 to 31/12/2022. Twenty one (16%) where diagnosed with FMF, 72 (54.5%) with PFAPA, 27 (20.5%) with syndrome of undifferentiated recurrent fevers (SURF). FMF patients (42.9% males) had a mean age of 49.5 ± 52.2 months and a mean age at diagnosis of 64.8 ± 54.5 months. Main presenting symptoms were fever (85.7%), musculoskeletal symptoms (33.3%), and abdominal pain (38.1%). At least one *MEFV* mutation/polymorphism was identified in all FMF patients. R202Q homozygosity was identified in 5 (23.8%), R202Q heterozygosity was identified in 9 (42.9%), while 7 were compound heterozygotes: 4 were R202Q and M694Val, 1 was R202Q and M680I, 1 was R202Q, P369S, R408Q and 1 was M680I and R761H. R202Q variant was present in homozygous or heterozygous state in 20/21 (95.2%) patients with FMF. PFAPA patients were 62.5% males with a mean age of 23.9 ± 29.2 months and a mean age at diagnosis of 46.5 ± 39.2 months. The main symptoms were fever in 71 patients (98.6%), tonsillitis in 64 (88.9%), adenopathy in 49 (68.1%) and oral ulcers in 22 (31.9%). Among them 58 patients were tested for *MEFV*. Twenty-three (32%) patients were positive for *MEFV* mutations/polymorphisms. R202Q heterozygosity was identified in 13 (22.4%) patients, R202Q homozygosity was identified in 2 (3.4%), 3 were heterozygotes for E148Q, 1 for T267I, 1 for K695R, 1 for R408Q, 1 for A744S and 1 compound heterozygous for R202Q and A744S. R202Q variant was present in homozygous or heterozygous state in 16/58 (27.6%) of PFAPA patients tested for MEFV.

**Conclusion:** R202Q polymorphism although a common variant, it is present in a larger proportion of patients diagnosed with FMF in Greece in either heterozygous, compound heterozygous and homozygous state. A potential pathogenic role of this variant should be reconsidered. In PFAPA patients the frequency of the R202Q polymorphism is comparable to that of the general healthy population in Greece (2).

**Patient Consent**

Yes, I received consent

**Disclosure of Interest**

None declared

**References**

1. 16-3304463-C-T | gnomAD v2.1.1 | gnomAD [Internet]. Available from: https://gnomad.broadinstitute.org/variant/16-3304463-C-T?dataset=gnomad_r2
2. Giaglis S, Papadopoulos V, Kambas K, Doumas M, Tsironidou V, Rafail S, et al. MEFV alterations and population genetics analysis in a large cohort of Greek patients with familial Mediterranean fever. Clin Genet 2007 May 71(5):458–67.

## P064 Uric acid as a promising cardiometabolic risk marker in children with autoinflammatory diseases

### M. F. Gicchino, A. N. Olivieri, A. Barlabà, M. Luciano, E. Miraglia del Giudice, A. Di Sessa

#### Department of Woman, Child and General and Specialized Surgery, University of Campania Luigi Vanvitelli, Napoli, Italy

##### **Correspondence:** M. F. Gicchino

*Pediatric Rheumatology 2023*, **21(Suppl 2):**P064

**Introduction:** Autoinflammatory diseases (AIDs) are a heterogeneous group of multisystem disorders characterized by self-limiting recurring episodes of fever and systemic inflammation (e.g. rash, arthritis, serositis) due to innate immune system dysregulation. Autoinflammatory diseases can be divided in monogenic and polygenic types. Monogenic autoinflammatory syndromes are those with identified genetic mutations, such as familial Mediterranean fever (FMF), tumor necrosis factor receptor-associated periodic fever syndrome (TRAPS), mevalonate kinase deficiency or hyperimmunoglobulin D syndrome HIDS, cryopyrin-associated periodic fever syndromes (CAPS). Those without an identified genetic mutation are known as polygenic and include Behçet syndrome, chronic recurrent multifocal osteomyelitis (CRMO), systemic juvenile idiopathic arthritis (sJIA) and among others. Particularly, a central pathogenic role for NOD-like receptor protein 3 (NLP3) inflammasome and Interleukin 1 (IL-1) has been highlighted in this context. Of note, both factors have been implied in the development of cardiometabolic diseases such as type 2 diabetes (T2D), insulin-resistance (IR), atherosclerosis, and obesity.

**Objectives:** As the usefulness of uric acid (UA) as cardiometabolic risk marker, we aimed to explore this association in a cohort of children diagnosed with AIDs.

**Methods:** We retrospectively examined 42 children (mean age 6.78±3.92 years) diagnosed with autoinflammatory diseases (4 subjects with Iper-IgD syndrome, 1 with Behçet disease, 3 with muckle-wells, 11 with Familial Mediterranean Fever (FMF), 13 with Periodic *Fever*, Aphthous Stomatitis, Pharyngitis, cervical Adenitis (PFAPA), 5 with systemic JIA, and 6 Chronic recurrent multifocal osteomyelitis (CRMO)) attending our Rheumatology Clinic. Anthropometric, biochemical, and instrumental assessments were performed. Participants were stratified in four groups according to quartiles of serum UA. Non-alcoholic fatty liver disease (NAFLD) was defined as the presence of ultrasound detected liver steatosis and/or alanine aminotransferase levels >40IU/L. All the parameters were collected before starting treatment.

**Results:** Patients belonging to the highest UA quartile showed increased both *systolic (SBP) and diastolic (DBP)* blood pressure standard deviation score (SDS) (p=0.02 and p=0.04, respectively) than subjects belonging to the lowest quartiles. A trend for body mass index (BMI)-SDS was also observed across UA quartiles (p=0.06). Higher serum creatinine and glucose levels (p=0.016 and p=0.04, respectively) were also found in these patients than in subjects belonging to the lowest quartiles. The percentage of hypercholesterolemia significantly increased across UA quartiles (p=0.01). Children belonging to the highest UA quartile had an odds ratio (OR) to show NAFLD of 1.08 (95% CI 1.01-1.17, p=0.05).

**Conclusion:** Children with AIDs presented with a worse cardiometabolic across UA quartiles. Our findings suggest that in clinical practice UA might represent a useful marker of cardiometabolic for these patients. Given that, a careful monitoring of UA should be recommended to avoid the serious burden of cardiometabolic consequences in children with AIDs.

**Patient Consent**

Yes, I received consent

**Disclosure of Interest**

None declared

## P065 Experience with Type I interferonopathies in the pediatric rheumatology unit of a tertiary hospital

### M. I. González Fernández^1^, I. Burgos Berjillos^1^, M. Martí Masanet^1^, B. López Montesinos^1^, L. Lacruz Pérez^1^, E. Novella Maestre^2^, A. Mensa-Vilaró^3^, J. I. Aróstegui Gorospe^3^, I. Calvo Penadés^1^

#### ^1^Pediatric Rheumatology Unit, Hospital Universitario y Politecnico La Fe. La Fe Health Research Institute; ^2^Genetics Unit, Hospital Universitario y Politecnico La Fe, Valencia; ^3^Department of Immunology, Hospital Clínic-IDIBAPS, Barcelona, Spain

##### **Correspondence:** I. Calvo Penadés

*Pediatric Rheumatology 2023*, **21(Suppl 2):**P065

**Introduction:** Monogenic type I interferonopathies are a heterogeneous group of inherited autoinflammatory diseases characterized by a overproduction of type I interferons (IFNs).

**Objectives:** To describe a cohort of patients with monogenic type I interferonopathies from a single center.

**Methods:** We reviewed those patients with definitive diagnosis or suspicion of monogenic type I interferonopathy in the Pediatric Rheumatology Unit of a tertiary hospital in the period 2017-2023. Genetic studies were carried out using next-generation sequencing and/or Sanger sequencing. Interferon Signature (IS) was evaluated through the 28-IFN response genes score using NanoString, being considered as positive those scores >1.73.

**Results:** We identified 7 patients with monogenic type I interferonopathy: 3 patients with Aicardi-Goutières syndrome (AGS), 2 patients with familial chilblain lupus, one patient with COPA syndrome and one patient with suspected type I interferonopathy and negative genetics.

Median age at disease onset was 4 (IQR: 0.92-7.50) years. Clinical manifestations included skin lesions (86%; 6/7), neurological symptoms and/or neuroimaging abnormalities (57%; 4/7), hematological abnormalities (43%; 3/7), pulmonary (14%; 1/7) and renal disease (14%; 1/7).

Among patients with AGS, P1 was homozygote for p.R97H TREX1 variant and presented with cerebral vasculitis at 14 months of age. P2, homozygote for p.A675T ADAR variant, had neonatal presentation with thrombopenia, hypertransaminasemia, hypotonia and abnormalities in brain MRI. P3, homozygote for p.A177T RNASEH2B variant, had delayed psychomotor development, seizures and neuroimaging with frontal leukodystrophy and calcifications.

Both patients with familial chilblain lupus had family history with dominant pattern. P4 carried previous described p.G166E STING1 variant. P5 was genetically negative.

Patient with COPA syndrome (P6) carried p.R233H variant and presented with renal MPO-ANCA positive vasculitis-like leading to kidney transplant, livedo reticularis, arthritis and diffuse interstitial lung disease with subpleural cysts.

IS, performed before starting treatment with JAK inhibitors, was positive in 86% (6/7) patients with a median 8.71 (IQR: 5.85-13.58). The positive result of the IS in Patient P7, who was genetically negative, led to requesting a cranial CT that showed calcifications in globus pallidus strongly suggesting the diagnosis of type I interferonopathy.

Four patients are being treated with baricitinib and one patient with ruxolitinib, well tolerated in all cases. Patient P3 is planned to start soon JAK inhibitor. Patient P4, who carried the p.G166E STING1 variant, presents mild skin symptoms and no systemic treatment has been started.

**Conclusion:** Monogenic type I interferonopathies are rare and complex disorders that can present with a broad spectrum of clinical manifestations in which an early recognition is essential for appropriate management. Our findings highlight the importance of considering type I interferonopathies in patients with neurological symptoms, skin suggestive manifestations, hematological abnormalities and pulmonary disease.

In our experience, a positive IS helped to guide the diagnosis and potentially to start treatment with JAK inhibitors in patients with symptoms suggestive of type I interferonopathy and negative results on genetic tests.

**Patient Consent**

Yes, I received consent

**Disclosure of Interest**

None declared

## P066 Idiopathic recurrent pericarditis in children

### A. Gunalp^1^, E. Kilic Konte^1^, E. Aslan^1^, F. Haslak^1^, M. Yildiz^1^, N. Ulug^2^, R. Dedeoglu^2^, S. Sahin^1^, A. Adrovic^1^, K. Barut^1^, O. Kasapcopur^1^

#### ^1^Pediatric Rheumatology; ^2^Pediatric Cardiology, Istanbul University Cerrahpasa Medical School, Istanbul, Türkiye

##### **Correspondence:** A. Gunalp

*Pediatric Rheumatology 2023*, **21(Suppl 2):**P066

**Introduction:** Idiopathic Recurrent Pericarditis (IRP); defined as the appearance of the signs and symptoms of pericardial inflammation after a symptom-free period of at least 4-6 weeks following an episode of acute pericarditis. It can be a demanding condition that can affect up to 30% of the patients who have experienced acute pericarditis. In developed countries 70% of pericarditis cases in children are defined as idiopathic. Autoimmunity is thought to play an important role in this disease.

**Objectives:** The aim of this study to evaluate the course of pediatric recurrent pericarditis cases from diagnose to treatment and investigate genetic and autoimmune mechanisms in cases whose etilogy has not been clarified.

**Methods:** The clinical features, laboratory findings and outcomes of the patients with IRP were evaluated retrospectively.

**Results:** A total of 13 patients who were diagnosed with IRP were included. Female/ Male :4 (30.2 %) / 9 (69.2%). Mean age of the patients was 13.8 (±4.6) year and mean age of first attacks was 10.7 (±4.3). Mean follow up was 3.7 (2.7) year. Median of total attacks was 4 (20). Accompanying to chest pain; 8 (61.5%) of the patiens had fever, 5 (38.5%) had back pain, 3 (23.1%) had nausea, 4 (30.8%) had dyspnea and 2 (15.4%) had palpitation. Pericardiocentesis was performed in 6 (46.2 %) of the patients at first attack. 2 (15.4%) of the patients had pleural effusion at the first attack. Colchicine and corticosteroids were first line treatments. 7 (53.8%) of the patients had no mutation, 4 (30.8%) had heterozygous mutation at exon 2 of MEFV, 1 (%7.7) had TNFRS13B and 1 (7.7%) had TNFRS1A heterozygous mutation with uncertain clinical significance. Anti-IL-1 agents were added of 9 (69.2%) of the patients with recurrrent attacks during the follow-up. Current treatment of the patients; all of them are still on colchicine, 5 (38.5 %) are anakinra and 3 are on (23.1%) canakinumab, 1 (7.7%) is still on corticosteroid. Median of annual recurrences prior to anti-IL-1 was 3 (2-10) and following to anti-IL-1 mean annuel number of recurrences is 1.2 (1.7) **(p:0.07)**.

**Conclusion:** Adult studies have emerged that autoimmune and genetic mechanism may play a role in the etiopathogenesis of IRP. This is the first outcomes of a study planned to be multicenteral. Our aim is to increase and share our knowledge and experience of the IRP disease.

**Patient Consent**

Yes, I received consent

**Disclosure of Interest**

None declared

## P067 A case of candle syndrome caused by digenic PSMB8/PSMB10 heterozygotic mutations

### A. Horne^1,2^, K. Palmblad^3^, P. Marits^4^, Å. Laestadius^5^, P. Brodin^1,6^

#### ^1^Department of Women's and Children's Health, Karolinska Institute; ^2^Department of Pediatric Rheumatology, Karolinska University Hospital, Stockholm; ^3^Department of Pediatric Rheumatology, Karolinska University Hospital, Stockholm; ^4^Department of Clinical Immunology and Transfusion Medicine; ^5^Pediatric nephrology, Karolinska University Hospital, Stockholm, Sweden; ^6^Department of Immunology and Inflammation, Imperial College London, London, United Kingdom

##### **Correspondence:** A. Horne

*Pediatric Rheumatology 2023*, **21(Suppl 2):**P067

**Introduction:** Chronic atypical neutrophilic dermatosis with lipodystrophy and elevated temperature (CANDLE) syndrome, is caused by mutations in proteasome associated genes (1). CANDLE caused by digenic PSMB8/PSMB10 heterozygotic mutations is so far not reported in the litterature.

**Objectives:** To describe the clinical phenotype of a preterm infant with CANDLE caused by digenic PSMB8/PSMB10 heterozygotic mutations.

**Methods:** Case report.

**Results:** Our girl is a firstborn child of parents of Swedish ancestry. She was born by sectio premature week 32 and was small for gestational age. At birth she developed hyperbilirubinemia requiring IVIG and exchange therapy. She subsequently developed fever, severe ascites and liver calcinosis as well as skin lesions with central necrosis, failure to thrive and lipodystrophy. Kidney involvement with proteinuria, tubulopathy, nephrocalcinosis and hypertension, anemia and severe thrombocytopenia and reduced IgG levels. No infectious, metabolic or malignant cause were found and genetic analyses by whole genome sequencing with normal variants for ~400 genes associated with inborn errors of immunity. At 7 weeks of age the weight was 2,5 kg and she rapidly deteriorated. High dose steroids was initiated with general improvement, of ascites and skin lesions. A 30-gene panel mRNA analysis was performed and an elevated IFN signature found (2) and a skin biopsy showed cell infiltrates coherent with CANDLE syndrome. (3) Reanalyses of WGS found digenic heterozygote PSMB8/10 variants, 1.A rare missense variant in PSMB8 with a predicted change of amino acid 209 from glycine to arginine (c.625G>A, p.Gly209Arg)- de novo 2.A rare truncating variant in PSMB10 (c.508C>T, p.Gln170Ter). Treatment with Barcitinib was initiated and a significant amelioration of manifestations but kidney involvement and severe growth retardation was seen. We have now added anti-IFNAR1 antibody treatment with the aim to reduce or even replace the barcitinib.

**Conclusion:** The clinical phenotype of CANDLE caused by digenic PSMB8/PSMB10 heterozygotic mutations seems severe. Our preterm baby might be the youngest patient (so far reported) started on treatment for the CANDLE syndrome with a JAK-inhibitor. This early therapy has definately prevented some disabling manifestations but unfortunately not been fully effective. The dosing of JAK-inhibitors in small and growing children is challenging and new therapy agents are warranted.

**Acknowledgments**

The patient ´s family. Professor Raphaela Goldbach-Mansky, NIH and Dr Sara Alehashemi, NIH for invaluable support.

**Patient Consent**

Yes, I received consent

**Disclosure of Interest**

None declared

**References**

1. Torrelo A. CANDLE syndrome as a paradigm of proteasome-related auto- inflammation. Front Immunol
  2017;8:927.
2. de Jesus AA, et al. Distinct interferon signatures and cytokine patterns define additional systemic
  autoinflammatory diseases. J Clin Invest. 2020
3. Symmank D, Borst C, Drach M, Weninger W. Dermatologic Manifestations of Noninflammasome-Mediated
  Autoinflammatory Diseases. JID Innov. 2022

## P068 The relationship of pteridine metabolites with disease activation in childhood behçet and PFAPA patients

### B. Hotaman^1^, Ö. Kasapçopur^1^, S. Şahin^1^, M. Yıldız^1^, A. Günalp^1^, E. Kılıç Könte^1^, E. Aslan^1^, A. Ç. Aktuğlu Zeybek^2^, E. Kıykım^2^, T. Koçkaya^2^, M. S. Cansever^2^, K. Barut^1^

#### ^1^Pediatric Rheumatology; ^2^Child Nutrition and Metabolism, Istanbul University-Cerrahpasa, Cerrahpasa Medical Faculty, Istanbul, Türkiye

##### **Correspondence:** K. Barut

*Pediatric Rheumatology 2023*, **21(Suppl 2):**P068

**Introduction:** Pteridine metabolites including neopterin, biopterin, DHPR activity and Kynurenin/Tryptophan ratio are immunologic markers of cellular immune activation. In PFAPA and pediatric Behçet Disease (BD) which cellular immunity takes part in pathogenesis, there is no reliable laboratory marker to indicate disease activation and follow-up and there is a lack of data in the literature to evaluate all these parameters in patients with PFAPA and pediatric BD.

**Objectives:** The purpose of this study was to determine the serum and urine levels of pteridine metabolites, DHPR activity, and Kynurenin/Tryptophan ratio in patiens with PFAPA and pediatric BD and to evaluate their correlation with disease activation.

**Methods:** Among the patients with PFAPA and BD under 18, who were admitted for the routine control or with disease activation between September 2022 and December 2022 were included in the study. Additional blood and urine samples were collected besides their routine tests and analysed, also data were obtained from face-to-face interviews and their medical records.

**Results:** The study included 106 patients (Female %40) (BD group: 61; %31 were active, PFAPA group; 45 %64 were active) and 67 control group. The average age was 5.15 for PFAPA years, and 14.91 years for pediatric BD. We found that mean serum neopterin, serum neopterin/biopterin ratio and urine pteridine metabolites concentrations in PFAPA and BD were significantly higher than in the controls (Neopterin in PFAPA:12,09 nmol/L, in BD: 8,76, in control: 8,79; Neopterin/Biopterin in PFAPA:29,62, in Behçet: 19,65, in control: 6,23; Neopterin/creatin in PFAPA 32,35, in BD: 17,34, in control:2,45) But there was no significant difference between clinically active and inactive PFAPA and BD. Also, no difference was found in DHPR and Kynurenin/Tryptophan concentrations between patient and control groups.

**Conclusion:** This study showed that serum neopterin, neopterin/biopterin ratio and urine pteridine concentrations can be used to evaluate cellular immune activation in PFAPA and pediatric BD. This can be used for diagnosis and follow-up of the patients when routine laboratory tests are useless. On the other hand, no difference was found between active and inactive patient groups in PFAPA and BD, so furher studies are needed to understand if pteridine concentrations change with disease activaton in PFAPA and BD patients or not.

**Patient Consent**

Yes, I received consent

**Disclosure of Interest**

None declared

**References**

1. Köse O, Arca E, Akgül O, Erbil K. The levels of serum neopterin in Behçet's disease--objective marker of disease activity. J Dermatol Sci. 2006;42(2):128-30.
2. Ozkan Y, Mete G, Sepici-Dincel A, Sepici V, Simsek B. Tryptophan degradation and neopterin levels in treated rheumatoid arthritis patients. Clin Rheumatol. 2012;31(1):29-34.
3. Girgin G, Tolga Sahin T, Fuchs D, Kasuya H, Yuksel O, Tekin E, et al. Immune system modulation in patients with malignant and benign breast disorders: tryptophan degradation and serum neopterin. Int J Biol Markers. 2009;24(4):265-70.
4. Marsálek P, Svoboda M, Smutná M, Blahová J, Vecerek V. Neopterin and biopterin as biomarkers of immune system activation associated with castration in piglets. J Anim Sci. 2011;89(6):1758-62.

## P069 Clinical characteristics and outcomes of chronic nonbacterial osteomyelitis in children: a single, tertiary center case series in Abu Dhabi, UAE

### S. Iqbal^1^, K. Mahmood^2^

#### ^1^Pediatrics; ^2^Pediatric rheumatology, SKMC, Abu dhabi, United Arab Emirates

##### **Correspondence:** S. Iqbal

*Pediatric Rheumatology 2023*, **21(Suppl 2):**P069

**Introduction:** Chronic nonbacterial osteomyelitis is a primary autoinflammatory bone disease that presents more frequently in children and is characterized by inflammatory bone lesions in the absence of an infectious etiology^1^. There is minimal data available on this condition in UAE

**Objectives:** The aim of this study is to evaluate the demographic, clinical, laboratory, imaging, histopathologic characteristics, and treatment responses of children with CNO

**Methods:** We carried out a retrospective single-center case series study of pediatric and adolescent patients treated for CNO between 2022-2023 at a tertiary center in Abu Dhabi. EMR was reviewed in order to collect data. Analysis was done using excel sheet

**Results:** 6 patients were included in the study, out of which 83.3% were females. The median current age and age of disease onset was 10.5 years range: 5-14 and 5.5 years range: 4-11.All patients had a pattern of recurrent multifocal disease with bone pain and arthralgia. 66% of patients presented with leg (hip, knee) pain, 33.3% presented with limping, 16.6% had back pain, 16.6% had shoulder pain.Mean ESR was 44.6mm/hr.Median CRP was 12 mg/L with 60% of patients with CRP > 5 mg/L. 25% of patients had positive RF and all had negative antinuclear antibodies and negative HLA-B27.The most common affected sites were metaphysis, diaphysis of long bones including tibia 100%, fibula 80%, femur 60%, humerus 60%, thoracic spine 60% and lumbar spine 40%. It was represented on wbMRI as bone marrow edema and multifocal bone marrow signals—hyperintense signal on STIR and hypointense on T1W.Bone marrow biopsy was done for 2 patients and showed cellular marrow with maturing trilineage hematopoiesis.All received treatment with NSAIDs and responded well. Of the patients whose disease was not controlled with NSAIDs, 3 patients received bisphosphonates and achieved remission.

**Conclusion:** The diagnosis of CNO should include clinical history, laboratory and imaging examination, and histopathological examination. Other causes of chronic bone pain should be ruled out. For treatment, NSAIDs are used as first-line drugs followed by steroids,bisphosphonates, and TNF-α inhibitors. Combination therapy with bisphosphonates and TNF-α inhibitors may be an option for refractory CNO. The limitation of this study is its small sample size. Thus, further studies including more patients from other tertiary centers are required to formulate diagnostic and treatment strategies for CNO. ^(2)^.

**Patient Consent**

Yes, I received consent

**Disclosure of Interest**

None declared

**References**

1. Balbi Gabriela et al Chronic Nonbacterial Osteomyelitis Report of Thirteen Cases *International Journal of Clinical Rheumatology*, Open Access Journals, 28 Apr. 2021, www.openaccessjournals.com/articles/chronic-nonbacterial-osteomyelitis-report-of-thirteen-cases-14612
2. Ma L Liu Tang, H *et al* Clinical characteristics and outcomes of chronic nonbacterial osteomyelitis in children: a multicenter case series *Pediatr Rheumatol* **20**1(2022). 10.1186/s12969-021-00657-4

## P070 H syndrome treatment with tocilizumab: analysis of 14 cases and literature review

### M. Jouret^1,2^, R. Jaquot^3^, J. Donadieu^4^, B. Bader-Meunier^1^, A. Phan^5^, B. Neven^1^, P. Seve^3^, A. Belot^2^

#### ^1^Immuno-rhumatology, Hôpital Necker Enfants Malades , Paris; ^2^Pediatric Nephro-Rhumato-Dermatology, Hôpital Femme-Mère Enfants; ^3^Internal Medicine Unit, University Hospital Lyon Croix-Rousse, Lyon; ^4^Pediatric Haematology Unit, Hôpital Trousseau; ^5^Pediatric Nephro-Rhumato-Dermatology, Hôpital Femme-Mère Enfants, Paris, France

##### **Correspondence:** M. Jouret

*Pediatric Rheumatology 2023*, **21(Suppl 2):**P070

**Introduction:** H syndrome is a rare autoinflammatory disease and an autosomal recessive syndrome caused by a mutation in the *SLC29A3* gene. That gene that encodes human equilibrative nucleoside transporter 3 (hENT3) should be implicated in the differentiation between mesenchymal and hematopoietic stem cells. This pathology belongs to the non-Langerhans Cell Histiocytoses (non-LCH) R group.

The syndrome is characterized by constellations of clinical features including hematological, cutaneous, osteoarticular, neuro-sensitive, and endocrinal features. For now, this pathology doesn’t have any standard treatment yet.

**Objectives:** The objective of this study was to evaluate the efficacy of Tocilizumab to treat H syndrome yet, such a rare disease with few attested cases renders prospective studies difficult to be properly conducted.

**Methods:** A systematic literature review was made on the database MEDLINE using the following MESH keyword: (« SLC29A3 »). Thanks to the results we have retrospectively described a group of patients with *SLC29A3* mutation treated with Tocilizumab in referenced French medical research centers.

**Results:** 135 patients with *SLC29A3* mutation were found throughout the literature. The French cases referenced 4 patients diagnosed at pediatric or adult age. In total 14 patients were treated with Tocilizumab and responded partially or completely in 92% of the cases. The treatment was intensified up to 12mg/kg/2 weeks in case of partial response.

Tocilizumab seems to be effective to control the autoinflammatory (fever and chronically biological inflammation), proliferative (organomegaly, peri-organic histiocytic infiltration) hematological (erythroblastopenia) articular (arthritis), and cutaneous (hypertrichosis, hyperpigmentation) manifestations.

**Conclusion:** Tocilizumab seems to be a first-choice treatment for patients with H syndrome. Early scheduled treatment could help prevent long-term complications, keeping in mind the secondary infection risk that might come along with biotherapy.

**Patient Consent**

Yes, I received consent

**Disclosure of Interest**

None declared

**References**

1. Baldwin, Stephen A., Sylvia Y.M. Yao, Ralph J. Hyde, Amy M.L. Ng, Sophie Foppolo, Kay Barnes, Mabel W.L. Ritzel, Carol E. Cass, et James D. Young. « Functional Characterization of Novel Human and Mouse Equilibrative Nucleoside Transporters (HENT3 and MENT3) Located in Intracellular Membranes ». *Journal of Biological Chemistry* 280, no 16 (April 2005): 15880-87. 10.1074/jbc.M414337200.
2. Colmenero, Isabel, Vered Molho-Pessach, Antonio Torrelo, Abraham Zlotogorski, et Luis Requena. « Emperipolesis: An Additional Common Histopathologic Finding in H Syndrome and Rosai–Dorfman Disease ». *The American Journal of Dermatopathology* 34, no 3 (may 2012): 315-20. 10.1097/DAD.0b013e31823b99fc.
3. Nair, Sreenath, Anne M. Strohecker, Avinash K. Persaud, Bhawana Bissa, Shanmugam Muruganandan, Craig McElroy, Rakesh Pathak, et al. « Adult Stem Cell Deficits Drive Slc29a3 Disorders in Mice ». *Nature Communications* 10, no 1 (3 juillet 2019): 2943. 10.1038/s41467-019-10925-3

## P071 Grey zone in the spectrum of autoinflammatory diseases: familial mediterranean fever accompanying periodic fever, aphthous stomatitis, pharyngitis, and adenitis syndrome: single-center experience

### E. Kılıç Könte, M. Yildiz, F. Haslak, A. Adrovic, A. Günalp, N. Gucuyener, I. Ulkersoy, E. Aslan, S. Sahin, K. Barut, O. Kasapcopur

#### Pediatric Rheumatology, Istanbul University, Cerrahpasa, Istanbul, Türkiye

##### **Correspondence:** E. Kılıç Könte

*Pediatric Rheumatology 2023*, **21(Suppl 2):**P071

**Introduction:** Despite the advanced knowledge concerning autoinflammatory diseases (AID), more data regarding the optimal treatment options and outcomes of the children who met the criteria of more than one AID are required.

**Objectives:** This study aimed to measure the response rates to colchicine and tonsillectomy and to evaluate the factors affecting the colchicine response in patients with Familial Mediterranean Fever (FMF) accompanying Periodic Fever, Aphthous Stomatitis, Pharyngitis, And Adenitis Syndrome (PFAPA) syndrome.

**Methods:** A total of 131 patients who met the modified Marshall and pediatric FMF criteria were included. Clinical findings, demographic features, family history, treatment responses, results of Mediterranean fever (MEFV) gene sequencing and colchicine response were evaluated. The possible factors detected with Chi-square, Fisher exact, independent samples t-test, and Mann Whitney U were further entered into the logistic regression analysis to identify independent predictors of colchicine response.

**Results:** In our cohort 58(44,2%) patient were female. The median age of onset was 18 months (1-77 months), and the age at diagnosis was 47±21,88 months. Intervals between episodes were 21 (7-90) days. Consanguineous marriage was detected in 17 (13%) of the patients. The most common clinical finding is fever, which is observed in all the patients, followed by exudative pharyngitis (88.5%), abdominal pain (86.3%), arthralgia (61.8%), stomatitis (51.1%), adenitis (42%), myalgia (28.7%), chest pain (16%), maculopapular rash (12.2%), arthritis (8.4%), and erysipelas-like rash(4.6%). *MEFV* gene variants were identified in 106 (80.9%) patients. The most common variants were *M694V* heterozygous (29%). Patients with tonsillopharyngitis, aphthous stomatitis and a PFAPA family history were more likely to be colchicine-resistant, while those with M694V and exon 10 mutations were more prone to have a favorable response to colchicine.

**Conclusion:** PFAPA syndrome patients with the MEFV gene variant showing typical FMF symptoms should be treated with colchicine, even after tonsillectomy. It has yet to be clarified when colchicine will be discontinued in FMF co-existing patients. Tonsillectomy should be performed before the colchicine treatment in patients with a PFAPA family history and those without exon 10 mutations.

**Patient Consent**

Yes, I received consent

**Disclosure of Interest**

None declared

**References**

1. Adrovic A, Sahin S, Barut K, Kasapcopur O (2019) Familial Mediterranean fever and periodic fever, aphthous stomatitis, pharyngitis, and adenitis (PFAPA) syndrome: shared features and main differences. Rheumatol Int 39:29-36
2. Butbul Aviel Y, Harel L, Abu Rumi M, Brik R, Hezkelo N, Ohana O, Amarilyo G (2019) Familial Mediterranean Fever Is Commonly Diagnosed in Children in Israel with Periodic Fever Aphthous Stomatitis, Pharyngitis, and Adenitis Syndrome. J Pediatr 204:270-274
3. Pehlivan E, Adrovic A, Sahin S, Barut K, Kul Cınar O, Kasapcopur O (2018) PFAPA Syndrome in a Population with Endemic Familial Mediterranean Fever. J Pediatr 192:253-255
4. Gattorno M, Caorsi R, Meini A, Cattalini M, Federici S, Zulian F, Cortis E, Calcagno G, Tommasini A, Consolini R, Simonini G, Pelagatti MA, Baldi M, Ceccherini I, Plebani A, Frenkel J, Sormani MP, Martini A (2009) Differentiating PFAPA syndrome from monogenic periodic fevers. Pediatrics 124:e721-728

## P072 Clinical profile and treatment of blau syndrome in a tertiary care centre in South India

### B. Krishna^1^, A. K. Tennelli^2,3^, A. P. Rao^2,3^

#### ^1^Paediatrics, Manipal Hospital; ^2^Paediatric Rheumatology, Indira Gandhi Institute Of Child Health; ^3^Paediatric Rheumatology, Manipal Hospital, Bangalore, India

##### **Correspondence:** B. Krishna

*Pediatric Rheumatology 2023*, **21(Suppl 2):**P072

**Introduction:** Blau syndrome (BS) is a rare monogenic form of autoinflammatory disease caused by a gain of function mutation in the CARD15/NOD2 gene. It is characterized by a triad of Arthritis, Dermatitis, and Granulomatous Uveitis in early childhood.

**Objectives:** To study the clinical profile, laboratory findings, and treatment response in patients with Blau syndrome.

**Methods:** A retrospective observational study of 16 children with Blau syndrome who were seen over a period of 5 years. Data on demographic and clinical features, laboratory investigations, and treatment were recorded.

**Results:** 16 children with Blau syndrome were included in the present study. The median age of onset of symptoms was 18 months. There were 50% (8) females and 50% (8) males. The classical triad of arthritis, dermatitis, and uveitis was present in 9 patients. The frequency of arthritis, dermatitis, and uveitis was 100% (16), 81% (13), and 62% (10) respectively. Camptodactyly was observed in 31% (5). The median age of diagnosis of ocular symptoms was 3yrs. Family history was noted in 25% (4) patients. Other features like Gastrointestinal involvement 12% (2), Renal 6%(1), and Respiratory 6% (1). Laboratory parameters reported elevated CRP and ESR in all patients.7 patients were confirmed genetically. Steroid and Methotrexate were used as first-line drugs in all patients and other drugs were added in refractory cases. 6 patients were treated with Biologics. The mean duration of treatment was 3.5 years and the Mean duration of follow-up was 5 years, however, 2 patients were lost to follow-up.

**Conclusion:** The possibility of BS should always be considered in any patient with arthritis and early ocular involvement. Severe joint contractures and blindness may occur if diagnosis and appropriate treatment are delayed. Early treatment may improve the prognosis.

**Patient Consent**

Yes, I received consent

**Disclosure of Interest**

None declared

**References**

1. Matsuda T, Kambe N, Ueki Y, Kanazawa N, Izawa K, Honda Y, et al. Clinical characteristics and treatment of 50 cases of Blau syndrome in Japan confirmed by genetic analysis of the NOD2 mutation. Ann Rheum Dis. 2020;79:1492–9.
2. Kumrah R, Pilania RK, Menia NK, Rawat A, Sharma J, Gupta A, Vignesh P, Jindal AK, Rikhi R, Agarwal A, Gupta V, Singh S, Suri D. Blau syndrome: Lessons learned in a tertiary care centre at Chandigarh, North India. Front Immunol. 2022 Sep 15;13:932919. doi: 10.3389/fimmu.2022.932919. PMID: 36189202; PMCID: PMC9521334.
3. Carlos D. Rosé and others, Blau syndrome: cross-sectional data from a multicentre study of clinical, radiological and functional outcomes, *Rheumatology*, Volume 54, Issue 6, June 2015, Pages 1008–1016, 10.1093/rheumatology/keu437

## P073 Curation and expansion of human phenotype ontology for systemic autoinflammatory diseases improves phenotype-driven disease matching

### W. K. Maassen^1^, O. Kul Cinar^2^, G. E. Legger^3^, P. L. van Daele^4^, M. Gattorno^5^, B. Bader-Meunier^6,7^, C. Wouters^8^, T. Briggs^9,10^, L. F. Johansson^1^, K. van der Velde^1^, M. A. Swertz^11^, E. Omoyinmi^12^, E. Hoppenreijs^13^, Y. Crow^14^, A. Belot^15^, D. Eleftheriou^2,12^, R. Caorsi^16^, F. A. Aeschlimann^17,18^, G. Boursier^19^, P. Brogan^12,20^, M. Haimel^21^, M. E. Van Gijn^22^

#### ^1^Department of Genetics, University Medical Centre Groningen, Groningen, Netherlands; ^2^Department of Paediatric Rheumatology, Great Ormond Street Hospital for Children NHS Trust, London, United Kingdom; ^3^Department of Rheumatology and Clinical Immunology, University Medical Centre Groningen, Groningen; ^4^Department of internal medicine and Department of immunology, Erasmus Medical Centre, Rotterdam, Netherlands; ^5^UOC Reumatologia e Malattie Autoinfiammatorie, IRCCS Istituto Giannina Gaslini, Genoa, Italy; ^6^Department of Paediatric Haematology-Immunology and Rheumatology, Necker-Enfants-Malades University Hospital, AP-HP Centre Université Paris Cité; ^7^Laboratory of Immunogenetics of Paediatric Autoimmune Diseases, UMR 1163, Imagine Institute, INSERM, Université Paris Cité, Paris, France; ^8^Department of Pediatric Rheumatology, University Hospital Leuven, Leuven, Belgium; ^9^Division of Evolution and Genomic Sciences, University of Manchester; ^10^Manchester Centre for Genomic Medicine, St Mary’s Hospital, Manchester University Hospitals NHS Foundation Trust, Manchester, United Kingdom; ^11^Groningen Bioinformatics Center, University Medical Centre Groningen, Groningen, Netherlands; ^12^Inflammation and Rheumatology Section, University College London Great Ormond Street Institute of Child Health, London, United Kingdom; ^13^Department of Paediatric Rheumatology, Sint Maartenskliniek / Radboud University Medical Center, Nijmegen, Netherlands; ^14^Institute of Genetics and Cancer, MRC Human Genetics Unit, The University of Edinburgh, Edinburgh, United Kingdom; ^15^Pediatric Nephrology, Rheumatology, Dermatology Unit, RAISE, INSERM, CNRS, CIRI, Hospital of Mother and Child, Hospices Civils of Lyon, Claude Bernard University, Lyon, France; ^16^Center of Autoinflammatory Diseases and Immunodeficiencies, IRCCS Istituto Giannina Gaslini, Genoa, Italy; ^17^Department of Pediatric Immunology-Hematology and Rheumatology, Necker University Hospital - APHP, Paris, France; ^18^Division of Pediatric Rheumatology, University Children’s Hospital Basel, Basel, Switzerland; ^19^Department of molecular genetics and cytogenomics, Rare and Autoinflammatory diseases unit, CeReMAIA, CHU Montpellier, Univ Montpellier, IRMB, INSERM, Montpellier, France; ^20^Department of Paediatric Rheumatology, Great Ormond Street Hospital for Children NHS Foundation Trust, London, United Kingdom; ^21^Boehringer Ingelheim RCV GmbH & Co KG, Vienna, Austria; ^22^Department of Genetics, Genome Diagnostics, University Medical Centre Groningen, Groningen, Netherlands

##### **Correspondence:** O. Kul Cinar

*Pediatric Rheumatology 2023*, **21(Suppl 2):**P073

**Introduction:** Accurate and standardized phenotypic descriptions are essential to diagnose rare disease patients and to discover new diseases. Human Phenotype Ontology (HPO) is extensively used for this purpose. The use of HPO has not been widely implemented in the field of Systemic Auto inflammatory Disease (SAID) because the lack of proper HPO terms and SAID annotation. Therefore, in a consortium endeavour HPO for SAID has been improved and curated. However, it has not been investigated if the curation process improved diagnosing SAID patients.

**Objectives:** Here, we aimed to study if improved HPO annotation of the diseases enhanced SAID identification. In addition, we aimed to demonstrate the potential of phenotype-driven genome diagnostics using curated HPO terms for SAIDs.

**Methods:** We collected HPO terms and, if available, whole exome sequencing (WES) data, from 98 genetically confirmed SAID patients across eight different European SAID expertise centres. LIRICAL, a computational algorithm was used to estimate the effect of the HPO curation on prioritizing the correct SAID for each patient.

**Results:** The results showed that the total amount of detected diagnoses increased from 66% to 86% and that the total amount of diagnoses with the highest rank increased from 38 to 45. Additionally, a pilot study showed that curation improved detection of the correct SAID in HPO based WES analysis (from 10/12 to 12/12). The list of candidate diseases left to be interpreted, while still containing the correct diagnosis, strongly decreased from 35 to 2, indicating comparable numbers to the diagnostic pipeline MOLGENIS VIP (virtual SAID gene panel).

**Conclusion:** This study demonstrates that curation of HPO terms for SAIDs improved the ability to computationally match SAID patients to their known diagnosis, thereby indicating the high potential of HPO based genome diagnostics for SAIDs.

**Patient Consent**

Not applicable (there are no patient data)

**Disclosure of Interest**

None declared

**References**

1. Georgin-Lavialle S, Fayand A, Rodrigues F, Bachmeyer C, Savey L, Grateau G. Autoinflammatory diseases: State of the art. Presse Med. 2019;48(1 Pt 2):e25-e48.
2. Benito-Lozano J, Arias-Merino G, Gomez-Martinez M, Ancochea-Diaz A, Aparicio-Garcia A, Posada de la Paz M, et al. Diagnostic Process in Rare Diseases: Determinants Associated with Diagnostic Delay. Int J Environ Res Public Health. 2022;19(11).
3. Robinson PN, Kohler S, Bauer S, Seelow D, Horn D, Mundlos S. The Human Phenotype Ontology: a tool for annotating and analyzing human hereditary disease. Am J Hum Genet. 2008;83(5):610-5.
4. Yuan X, Wang J, Dai B, Sun Y, Zhang K, Chen F, et al. Evaluation of phenotype-driven gene prioritization methods for Mendelian diseases. Brief Bioinform. 2022;23(2).
5. Haimel M, Pazmandi J, Heredia RJ, Dmytrus J, Bal SK, Zoghi S, et al. Curation and expansion of Human Phenotype Ontology for defined groups of inborn errors of immunity. J Allergy Clin Immunol. 2022;149(1):369-78.

## P074 Evolutionary profile of pfapa syndrome

### M. Labouret^1^, I. Elhani^2^, V. Hentgen^1^

#### ^1^Department of General Pediatrics, Reference Center for Auto-inflammatory Diseases and Amyloidosis (CEREMAIA), Versailles Hospital, Versailles; ^2^ Department of Internal Medicine, Reference Center for Auto-inflammatory Diseases and Amyloidosis (CEREMAIA), Tenon Hospital, Paris, France

##### **Correspondence:** M. Labouret

*Pediatric Rheumatology 2023*, **21(Suppl 2):**P074

**Introduction:** The periodic fever, aphthous stomatitis, pharyngitis and cervical adenitis (PFAPA) syndrome is the most common cause of autoinflammatory periodic fever in children. There are few studies on the age and mode of resolution of this syndrome. The only generally accepted observation is that PFAPA is a self-limited disease that usually resolves spontaneously by adolescence at the latest.

**Objectives:** To evaluate age, time to recovery, and clinical predictive factors of disease duration.

**Methods:** Retrospective, single-center study. All patients diagnosed with PFAPA at the Versailles Hospital Center and included in the JIR cohort between January 2016 and March 2023 were identified. Recovery was defined as the absence of any febrile episode for at least 12 months. All patients were recontacted by phone, except those for whom a febrile relapse had been identified in the previous 12 months.

**Results:** 207 patients were diagnosed with PFAPA. Of these, 56 (27%) patients had a resolution of periodic fever, 115 (56%) were still active and 36 (17%) patients lost to follow-up could not be contacted. In the resolved fever group, the median time from first contact with the center to the last follow up was 6.5 years (Q1-Q3: 4.7-8.8) and 3.3 years (Q1-Q3: 2.0-4.4) from last febrile episode to last news. The median duration of symptoms was 5.0 years (Q1-Q3: 2.8-8.1) and the median age at last febrile episode was 8.3 years (Q1-Q3: 5.2-9.9). No correlation was identified between age of onset and duration of symptoms (Spearman r =-0.41, p = 0.002). Tonsillectomy was performed in 8 patients, 4 of whom had complete resolution of episodes, 3 had temporary remission and 1 experienced a short period of spacing of the episodes before resuming. Only 2 patients in this group had been treated with colchicine without reported efficacy. Among the 115 patients with persistent fever, we identified 10 patients initially stated to be recovered, who relapsed after a median remission period of 32 months (Q1-Q3: 25-39).

**Conclusion:** We present the data from a large cohort of PFAPA patients followed in a reference center for autoinflammatory diseases. In the resolved fever group, the last febrile episode occurs for the great majority before adolescence. The duration of symptoms is variable and does not seem to correlate with the age of onset. The identification of relapses after at least 12 months without a febrile episode raises questions about the definition of recovery. Further investigations and longer-term follow-up are needed to confirm these data and to identify predictive factors for recovery.

**Patient Consent**

Not applicable (there are no patient data)

**Disclosure of Interest**

None declared

## P075 Combination therapy of plasmapheresis with continuous intravenous anakinra infusion to calm the cytokine storm in macrophages activation syndrome secondary to systemic juvenile idiopathic arthritis

### R. Al Jashmi, S. Al Abrawi, A. Al Sawafi

#### Child Health, Royal Hospital, Muscat, Oman

##### **Correspondence:** R. Al Jashmi

*Pediatric Rheumatology 2023*, **21(Suppl 2):**P075

**Introduction:** Macrophage activation syndrome (MAS) is one of the most common fatal complications of inflammatory diseases in children is a subtype of secondary hemophagocytic lymphohistiocytosis (HLH). In recent studies, Interleukin-1 inhibitors have been shown to be effective in treating systemic JIA, particularly when combined with MAS. There is limited literature describing plasmapheresis as an effective and rapid method of removing inflammatory cytokines from the body of patients with MAS-associated cytokines storms.

**Objectives:** This report describes our experience with plasmapharesis and continuous intravenous anakinra infusion for treating macrophage activation syndrome.

**Methods:** We reported a six-year-old boy admitted under general paediatric care for evaluation of a one-month history of daily fever, multiple arthritis, cervical lymphadenopathy, evanescent skin rash and elevated inflammatory markers. A child was examined by multiple teams, including the Infectious Disease team, the Haemat-Oncology team, and the Rheumatology team. The patient was diagnosed with systemic onset Juvenile Idiopathic Arthritis and started on intravenous methylprednisolone pulses (IVMP) and subcutaneous (SC) anakinra. Nevertheless, the child went into status epilepticus and was intubated. He continued to deteriorate and required intensive inotropic support as well as dialysis.After two days of being afebrile, he began to spike a fever again and his labs confirmed MAS with ferritin reached 26000. Among the measures taken to escalate the treatment are the switching of anakinra to an IV as a continuous infusion, the addition of ciclosporin, and the initiation of plasmapheresis. Following three days of plasma exchange, the child showed dramatic improvement and was able to be extubated and removed from the inotropes. Despite the child's stable haemodynamics and improved MAS labs, we encountered difficulty switching anakinra to subcutaneous administration. We had two failed attempts where the child developed fever, a skin rash, and a mild increase in ferritin. The child remained in PICU receiving IV continuous anakinra for more than three weeks. Once ferritin reached 900, we initiated an IV bolus instead, and after a week with normal clinical and biochemical improvement, we changed to SC and the child was discharged home asymptomatic and with completely normal MAS lab results.

**Results:** Timely recognition of the cytokine storm led to the escalation of treatment at an early stage. This child is the first patient at our hospital to receive continuous IV Anakinra infusion, and the outcome has been favourable despite a stormy course in the intensive care unit.

**Conclusion:** Anakinra has been used off-label in critically ill patients with MAS, thrombocytopenia, subcutaneous edema, and neurological dysfunction. Tapering from intravenous Anakinra to subcutaneous was challenging. This case taught us that tapering must begin at the right time to avoid hiccups or unfavourable outcomes. As reported in a few studies, plasmapheresis combined with immunosuppressants can reduce MAS-associated mortality by rapidly removing cytokines from the body.

**Patient Consent**

Yes, I received consent

**Disclosure of Interest**

R. Al Jashmi Consultant with: no conflict of interest, S. Al Abrawi Consultant with: no conflict of interest, A. Al Sawafi Consultant with: no conflict of interest

**References**

1. Fajgenbaum DC, June CH. Cytokine Storm. *N Engl J Med*. 2020;383(23):2255-2273. doi:10.1056/NEJMra2026131
2. Phadke O, Rouster-Stevens K, Giannopoulos H, Chandrakasan S, Prahalad S. Intravenous administration of anakinra in children with macrophage activation syndrome. Pediatr Rheumatol Online J. 2021 Jun 29;19(1):98. doi: 10.1186/s12969-021-00585-3. PMID: 34187503; PMCID: PMC8240425.
3. Henderson LA, Cron RQ. Macrophage Activation Syndrome and Secondary Hemophagocytic Lymphohistiocytosis in Childhood Inflammatory Disorders: Diagnosis and Management. Paediatr Drugs. 2020 Feb;22(1):29-44. doi: 10.1007/s40272-019-00367-1. PMID: 31732958; PMCID: PMC7334831
4. Chen Y, Hu Z, Cai S, Shen G, Zhong J, Dong L. Efficacy of plasma exchange on top of standard immunosuppressive therapy in adult autoimmune inflammatory rheumatic diseases-associated macrophage activation syndrome, a single center real-world analysis. Semin Arthritis Rheum. 2022 Aug;55:152043. doi: 10.1016/j.semarthrit.2022.152043. Epub 2022 Jun 7. PMID: 35696776.
5. Martini A, Lovell DJ, Albani S, Brunner HI, Hyrich KL, Thompson SD, Ruperto N. Juvenile idiopathic arthritis. Nat Rev Dis Primers. 2022 Jan 27;8(1):5. doi: 10.1038/s41572-021-00332-8. PMID: 35087087.

## P076 Current treatment in macrophage activation syndrome worldwide: a systematic literature review to inform the metaphor project

### F. Baldo^1,2^, R. Erkens^3^, M. Mizuta^4^, C. Bracaglia^5^, D. Foell^6^, M. Gattorno^7^, M. Jelusic^8^, J. Anton^9^, P. Brogan^2^, S. Canna^10^, S. Chandrakasan^11^, R. Cron^12^, F. De Benedetti^5^, A. Grom^13^, M. Heshin-Bekenstein ^14^, A. Horne^15^, R. Khubchandani ^16^, S. Ozen^17^, P. Quartier^18^, A. Ravelli^7^, M. Shimizu ^19^, G. Schulert^13^, C. Scott^20^, R. Sinha ^21^, N. Ruperto^7^, J. Swart^3^, S. Vastert^3^, F. Minoia^1^ on behalf of the PReS MAS/sJIA Working Party and Pediatric Rheumatology International Trial Organization

#### ^1^Fondazione IRCCS Ca’ Granda Ospedale Maggiore Policlinico, Milano, Italy; ^2^Great Ormond Street Hospital, London, United Kingdom; ^3^University Medical Center, Utrecht, Netherlands; ^4^Department of Pediatric Rheumatology, Hyogo Prefectural Kobe Children's Hospital , Kobe, Japan; ^5^IRCCS Ospedale Pediatrico Bambino Gesù, Roma, Italy; ^6^University Hospital Muenster, Muenster, Germany; ^7^IRCCS Giannina Gaslini, Genova, Italy; ^8^University Hospital Centre Zagreb, University School of Medicine, Zagreb, Croatia; ^9^Hospital Sant Joan de Déu, Barcelona, Spain; ^10^Children’s Hospital of Philadelphia, Philadelphia; ^11^Children’s Healthcare of Atlanta, Atlanta; ^12^University of Alabama at Birmingham, Birmingham, ^13^Cincinnati Children’s Hospital, Cincinnati, United States; ^14^ Dana Dwek Children's Hospital, Tel Aviv, Israel; ^15^Karolinska Institute, Solna, Sweden; ^16^SRCC Childrens Hospital, Mumbai, India; ^17^Hacettepe University Children's Hospital, Ankara, Türkiye; ^18^Université de Paris, IMAGINE Institute, Necker Children’s Hospital, Paris, France; ^19^- Tokyo Medical and Dental University, Tokyo, Japan; ^20^Red Cross Children Hospital and Groote Schuur Hospital, Cape Town, South Africa; ^21^Systemic JIA Foundation , Cincinnati, United States

##### **Correspondence:** F. Baldo

*Pediatric Rheumatology 2023*, **21(Suppl 2):**P076

**Introduction:** Treatment of macrophage activation syndrome (MAS) is still largely empiric and not yet standardized across countries, mainly due to lack of robust evidence and different access to medications worldwide. The METAPHOR project is a PReS/PRINTO initiative aimed to optimize treatment in systemic juvenile idiopathic arthritis (sJIA) and MAS. In this context, a systematic literature review on MAS treatment was conducted.

**Objectives:** To assess the state of the art of current therapeutic approaches to MAS worldwide, to highlight areas at major risk of discrepancies among practitioners

**Methods:** A systematic literature search of both EMBASE and PUBMED was performed in June 2022. Paper screening was done by 2 independent teams based on agreed criteria. Data extraction was standardized following the PICOs approach. The panel of experts defined for the METAPHOR project assessed paper validity, using the Joanna Briggs Institute appraisal tools, and category of evidence following the EULAR standardized operating procedure.

**Results:** Fifty-nine papers were finally included (86% retrospective case series), describing a total of 984 patients with MAS (783 sJIA, 97 systemic lupus erythematosus (SLE), 70 Kawasaki disease (KD) and 34 with other rheumatologic conditions). Most papers received a low to moderate validity score (70%) and 3 or 4 category of evidence (90%). All papers mentioned the use of glucocorticoids (details available in 513 patients), most frequently intravenous methylprednisolone (71%); dexamethasone was used in 10% of patients, more frequently with SLE. Papers reported a 13-80% range of patients treated with cyclosporine (2-8 mg/kg/day), with data available in 389 MAS: only 10% had reported outcome, favourable in 94%. Data on anakinra were available for 180 MAS patients (84 sJIA, 8 SLE, 2 vasculitis, 1 KD), with a known favourable outcome in 41/48 and a dosing range 1.7-20 mg/kg/day. Etoposide was mentioned, alone or as part of the HLH 2004 protocol, in 23 papers, but only 11 reported patient details: in 9 papers etoposide was administered as for HLH-94/04 protocol, while 2 studies reported 11 MAS treated with low-dose etoposide (50–100 mg/m^2^ once weekly) with positive outcome. The only JAK-inhibitor reported was ruxolitinib, used in 6 MAS, without serious adverse events. Immunoglobulin use was extensively mentioned, mostly as a co-medication; however, no specific data on immunoglobulins’ efficacy were available. Haematopoietic stem cell transplant was mentioned in 5 papers, reporting 9 sJIA-MAS treated and a favourable outcome in 8.

**Conclusion:** Despite a large number of patients reported, the level of evidence regarding treatment of MAS is still poor, mainly due to the retrospective nature of most studies and the lack of reported outcomes reliably attributable to a specific medication or condition. Novel data recently published, including the first clinical controlled trial in MAS, should be taken into account in a next forthcoming update.

**Patient Consent**

Not applicable (there are no patient data)

**Disclosure of Interest**

None declared

## P077 Efficacy of MAS825, a bispecific il-1 and il-18 neutralizing antibody, in a patient with relapsing macrophage activation syndrome during refractory systemic juvenile idiopathic arthritis

### C. Bracaglia^1^, M. Pardeo^1^, A. De Matteis^1^, A. Arduini^1^, A. M. Greco^2^, I. Caiello^1^, G. Prencipe^1^, F. De Benedetti^1^

#### ^1^Division of Rheumatology; ^2^Division of Hospital Pharmacy, IRCCS Ospedale Pediatrico Bambino Gesù, Roma, Italy

##### **Correspondence:** C. Bracaglia

*Pediatric Rheumatology 2023*, **21(Suppl 2):**P077

**Introduction:** MAS is a severe, life-threatening complication of sJIA with significant morbidity and mortality. Patients frequently require long-term immunosuppressive treatment with several side effects.

**Objectives:** To report the response to MAS825, a bispecific monoclonal antibody targeting IL-1β and IL-18, in a patient with refractory-sJIA and chronic relapsing MAS.

**Methods:** Serum levels of IL-18, CXCL9 and neopterin were measured by ELISA.

**Results:** A 9-year-old Caucasian girl, presented a first HLH episode in April 2017, at 3-year-old, with persistent fever, hepatosplenomegaly, CNS involvement with seizures, marked hyperferritinemia and high CXCL9 levels (201991 pg/ml). No rash nor arthritis were noted. She met the HLH-2004 criteria and she received emapalumab in the context of the primary HLH trial. She had complete remission with normalization of CXCL9. After 10 months from emapalumab treatment, she presented recurrent episodes of relapsing/remitting fever, rash, thrombocytopenia, increased levels of inflammatory markers, of liver function tests (ALT 1333, AST 851, LDH 822 UI/l) and hyperferritinemia (>1500 ng/ml). Treatment with prednisone (PD) (0.5-1 mg/kg/die) was administered for 8 months. In February 2020, she developed arthritis and she was referred to our attention. At this time she met the ILAR criteria for sJIA and the EULAR/ACR MAS criteria (ferritin 3.460, PLT 64.000, triglyceride 220; fibrinogen 385, AST 100). A CT scan showed splenomegaly and hepatomegaly, with disseminated round lesions. Intravenous methylprednisolone (3 pulses of 30 mg/kg) followed by oral PD (0.5 mg/kg) and anakinra (5 mg/kg) were started without significant improvement. She then presented a further episode of full-blown MAS with severe marrow and liver involvement. CXCL9, neopterin and IL-18 levels were persistently elevated. Emapalumab was administered, on CU regimen, for 13 months with clinical remission and normalization of laboratory parameters after 5 months. CXCL9 and neopterin normalized, IL-18 decreased but remained persistently elevated. GCs were tapered to <0.2 mg/Kg after 4 months and discontinued after 7. Anakinra was maintained at 5 mg/kg/day. In December 2021, after a viral infection, she presented fever and cytopenia. PD (1 mg/kg/day) was started again with partial response. In June 2022, MAS825 on CU regimen was started (10 mg/kg every 2 weeks) with rapid improvement of clinical and laboratory features. GCs were completely stopped after 5 MAS825 infusions. After 10 months of treatment, she is in complete clinical remission, without any other treatment.

**Conclusion:** Simultaneous neutralization of IL-1β and IL-18 may represent an efficacious therapeutic approach in sJIA patients with chronic/relapsing MAS with liver and marrow involvement.

**Patient Consent**

Yes, I received consent

**Disclosure of Interest**

C. Bracaglia: None declared, M. Pardeo Consultant with: Sobi, A. De Matteis: None declared, A. Arduini: None declared, A. Greco: None declared, I. Caiello: None declared, G. Prencipe: None declared, F. De Benedetti Consultant with: Abbvie, Sobi, Novimmune, Novartis, Roche, Pfizer.

## P078 Emapalumab treatment followed by hematopoietic stem cell transplantation in systemic juvenile idiopathic arthritis complicated by recurrent macrophage activation syndrome

### C. Bracaglia^1^, M. Pardeo^1^, G. Marucci^1^, S. Riccio^1^, F. Quagliarella^2^, I. Caiello^1^, G. Prencipe^1^, P. Merli^2^, F. Locatelli^2^, F. De Benedetti^1^

#### ^1^Division of Rheumatology; ^2^Department of Hematology/Oncology, Cell and Gene Therapy, IRCCS Ospedale Pediatrico Bambino Gesù, Roma, Italy

##### **Correspondence:** C. Bracaglia

*Pediatric Rheumatology 2023*, **21(Suppl 2):**P078

**Introduction:** MAS is a life-threatening complication of different rheumatic diseases, particularly of sJIA.

**Objectives:** To report the case of 17-year-old girl with sJIA complicated by recurrent severe MAS episodes.

**Methods:** Patient received emapalumab (anti-IFNg antibody) in two subsequent MAS episodes and then underwent an uncomplicated hematopoietic stem cell transplantation (HSCT) while on emapalumab and anakinra granting complete control of inflammatory activity of the underlying disease.

**Results:** A 13-year-old girl presented with fever, rash and hepato-splenomegaly. Laboratory parameters were consistent with full-blown MAS. In the absence of clear evidence of an underlining condition, a diagnosis of secondary HLH was made, treatment with high dose of IV methylprednisolone (mPDN) and oral cyclosporine (CYA) was started with progressive improvement. After one year, still on CYA, she presented with fever, rash and arthritis with laboratory parameters consistent with MAS. Diagnosis of sJIA complicated by MAS was made. In 24 hours, she rapidly worsened and was admitted in ICU. High dose of IV mPDN (7 pulses of 30 mg/kg/day) as well as IV CYA (5 mg/kg/day) did not yield a response. Emapalumab was started, in the NI-0501-06 trial, (6 mg/kg initial dose followed by 3 mg/kg every 3 days) for 11 infusions. Conditions progressively improved. To prevent flares of the underlining sJIA, anakinra (2 mg/kg/day) was started. After 2 years in clinical remission, while on anakinra every other day, she presented with fever, vomiting and diarrhea. Anakinra was immediately increased to daily dosing. Stool analysis showed Salmonella infection and antibiotic therapy was started. Nevertheless, she rapidly worsened, laboratory parameters were again consistent with full-blown MAS. She required ICU admission for multiorgan failure. Anakinra was administered IV and the dose increased up to 12 mg/kg/day. IV MPDs (8 pulses of 30 mg/kg/day) as well as IV CYA (5 mg/kg/day) were started with partial response. Based on her previous response to emapalumab, it was started again (compassionate use) with marked and rapid improvement. Because of recurrent MAS episodes and of their rapidly evolution, the patient underwent an ex-vivo T cell-depleted haploidentical HSCT from her mother. The conditioning regimen was based on a Thiotepa-Treosulfan-Fludarabine scheme. Emapalumab was continued 1 month after HSCT together with anakinra. The patient achieved full donor engraftment with complete donor-derived immune reconstitution after 3 months. 30 months after HSCT, she is in excellent clinical condition on anakinra every other day, with complete remission of sJIA/MAS, also confirmed by persistently normal levels of IL-18 and CXCL9.

**Conclusion:** This case provides further evidence of the efficacy of emapalumab in MAS, of the potential benefit of HSCT in difficult to treat sJIA patients. Notably, full control of inflammatory activity with emapalumab and anakinra may help to obtain a successful HSCT and reduce the risk of rejection.

**Patient Consent**

Yes, I received consent

**Disclosure of Interest**

C. Bracaglia Consultant with: Sobi, Novartis, M. Pardeo Consultant with: Sobi, G. Marucci: None declared, S. Riccio: None declared, F. Quagliarella: None declared, I. Caiello: None declared, G. Prencipe: None declared, P. Merli Consultant with: Sobi, F. Locatelli Consultant with: Sobi, F. De Benedetti Consultant with: Abbvie, Sobi, Novimmune, Novartis, Roche, Pfizer.

## P079 Relapsing macrophage activation syndrome rescued by treatment with MAS825, an anti-il1 and Il-18 monoclonal antibody, in two patients with systemic onset juvenile idiopathic arthritis

### R. Caorsi^1^, S. Rosina^1^, A. Bertoni^1^, F. Penco^1^, V. Natoli^1^, C. Matucci Cerinic^1^, R. Papa^1^, A. Corcione^1^, S. Volpi^1^, A. Ravelli^2^, M. Gattorno^1^

#### ^1^Reumatologia e Malattie Autoinfiammatorie; ^2^Direzione Scientifica, IRCCS Istituto Giannina Gaslini, Genova, Italy

##### **Correspondence:** R. Caorsi

*Pediatric Rheumatology 2023*, **21(Suppl 2):**P079

**Introduction:** Macrophage activation syndrome (MAS) is a life-threating condition, even in the biologic era, being some patients resistant to multiple drugs, immunosuppressant and biological.

**Objectives:** to describe the clinical course of two patients with severe and relapsing MAS, complicating SoJIA, not controlled by different associations of steroids, immunosuppressant and biological drugs, who displayed a quick and complete response to IL-1 and IL18 blockade with MAS825.

**Methods:** Data regarding the clinical course of the patients before and after treatment with MAS825 were collected.

**Results:** Patient 1 is a 20 years old male, with disease onset at the age of 2 years; the patient experienced multiple disease flares, with steroid-dependence and incomplete response to both synthetic (MTX, thalodiomide, cyclosporine) and biological (anakinra, etanercept, adalimumab, tocilizumab, abatacept) disease modifying anti-rheumatic drugs (DMARDS). While in treatment with oral baricitinib (4 mg/day), subcutaneous methotrexate (MTX, 15 mg weekcly) and a minimal dose of oral steroid (prednisone 0,15 mg/kg/day) the patient experienced, following COVID-19 infection, a relapse of the disease with laboratory signs of MAS, treated with high doses of iv steroids, intravenous anakinra (300 mg/day) and cyclosporine (100 mg twice a day), associated to the ongoing treatment with baricitinib, without amelioration; due to the appearance of laboratory signs of thrombotic microangiopathy, the patient was treated with plasmapheresis and eculizumab, with amelioration. In light of the persistence of laboratory signs of MAS, i.v. MAS825 700 mg every 2 weeks was started, with quick amelioration, allowing steroids tapering. After 8 months the patient is still on treatment with MAS825 every 2 weeks, associated with MTX,cyclosporine and low doses of steroids.

Patient 2 is a 10 years old girl suffering from SoJIA from the age of 1 year with relapsing episodes of MAS, steroid dependence and incomplete response to both synthetic (MTX, cyclosporine) and biological (anakinra, tocilizumab, canakinumab, baricitinib) DMARDS. While on treatment with canakinumab, baricitinib (4 mg/day), cyclosporine and low doses of steroids (0,3 mg/kg/day), the girl experience an MAS, following an infectious otomastoiditis, not completely controlled by treatment with iv high doses steroids, anakinra and cyclosporine. In light of that, the patient was treated with i.v. MAS825 10 mg/kg every 2 weeks, with prompt amelioration of MAS parameters. The girl has received two doses of the drug, so far.

High levels of plasmatic IL-18 were detected before the initiation of MAS825 treatment, in both patients.

**Conclusion:** The two clinical case enlighten the effectiveness of blocking both IL-18 and Il-1 in patients with severe MAS complicating SoJIA, confirming the crucial role of IL-18 in the pathogenesis of this condition.

**Patient Consent**

Yes, I received consent

**Disclosure of Interest**

None declared

## P080 Kawasaki disease complicating with macrophage activation syndrome: 3 case reports

### E. Corinaldesi

#### Ramazzini Hospital, Carpi, Italy

##### **Correspondence:** E. Corinaldesi

*Pediatric Rheumatology 2023*, **21(Suppl 2):**P080

**Introduction:** Kawasaki Disease ( KD) is an acute vasculitis affecting children mainly under 5 years of age, leading to coronary artery aneurysms with an incidence rate of up to 25% if untreated . Macrophage Activation Syndrome ( MAS), a rare life threatening complication, can occur during the .acute, subacute, and chronic phases of KD. The incidence of MAS in patients with KD has been estimated to be 1.9% Early recognition and immediate therapeutic intervention is the key to avoid these complications. Since Up-to-date patient reports for KD complicated with MAS are limited , we report three cases of KD complicating with MAS.

**Objectives:** To describe three case of KD complicating with MAS and shown the importance of a prompt diagnosis and treatment to avoid the severe complications of MAS.

To highlight the efficacy of anti IL-1 blocker, anakinra, to treat unresponsive cases of KD comp0licating with to IVIG and steroids

**Methods:** We reviewed retrospectively clinical charts of three cases of children affected by KD complicating with MAS hospitalized in two Pediatric . Unit of two Hospitals in Emilia Romagna, a northern region of Italy

**Results:** Case 1 : a previously healthy 23 month-old girl with full clinical criteria of KD and a haemorrhagic rash who developed MAS during the acute phase of illness; she responded promptly to an infusion of high dose of Immune Globulin intravenous (IVIG) and high dose of pulses of methylprednisolone ( MPD) with improvement of laboratory tests without development of coronary artery alterations (CAA) at any phase of illness..

Case 2: a previously healthy 10 month-old girl presenting with fever and maculopapular rash, IVIG- non responder, complicating in the subacute phase with MAS manifesting persistent fever, hypertransaminasemia, hyperferritinemia and hypofibrinogenemia after two high doses of IVIG and boluses of MPD; she responded to addiction of IL-1 blocker , anakinra. She did not present CAA alterations at any phase of illness. .

Case 3 : a previously healthy 26 month-old boy with incomplete KD ( fever, maculopapular rash, cheilitis and hyperemic conjunctivitis) with gallbladder hydrops and IVIG-non responder to 2 doses and high dose of boluses of MPD . In subacute phase he complicated with MAS and responded to anakinra iv. During subacute phase he developed transient aneurysms that regressed during the chronic phase

**Conclusion:** An early diagnosis is crucial to start a prompt and aggressive therapy to halt the severe MAS-related inflammation, in order to limit severe complications, morbidity and mortality. Diagnosis of the occurrence of MAS can be challenging due to overlapping features of the severe forms of KD, but the typical clinical manifestations along with the laboratory tests can help.

Despite the small number of cases, in our experience IL-1 blocker is a valid therapy after ineffectiveness of first -line treatment of the KD complicating with MAS. Further evidences are needed to support our findings.

**Patient Consent**

Yes, I received consent

**Disclosure of Interest**

None declared

## P081 Characteristics of macrophage activation syndrome in systemic JIA patients receiving anakinra as first-line treatment

### R. Erkens^1,2^, L. Huber^2^, A. Verwoerd^1^, G. Rogani^1,2^, D. Schonenberg^3^, M. Van den Berg^3^, W. Armbrust^4^, E. Legger^4^, S. Kamphuis^5^, E. Schatorjé^6^, E. Hoppenreijs^6^, J. Swart^2^, M. Jansen^2^, J. Van Loosdregt^1^, B. Vastert^1,2^

#### ^1^Center for Translational Immunology, University Medical Center Utrecht; ^2^Department of Pediatric Rheumatology and immunology, Wilhelmina Children's Hospital, Utrecht; ^3^Division of Pediatric Rheumatology and Immunology, Amsterdam University Medical Center, Amsterdam; ^4^Division of Pediatric Rheumatology and Immunology, University Medical Center Groningen, Groningen; ^5^Division of Pediatric Rheumatology and Immunology, Erasmus Medical Center, Rotterdam; ^6^Division of Pediatric Rheumatology and Immunology, University Medical Center Radboud, Nijmegen, Netherlands

##### **Correspondence:** R. Erkens

*Pediatric Rheumatology 2023*, **21(Suppl 2):**P081

**Introduction:** Systemic Juvenile Idiopathic Arthritis (sJIA) is a severe inflammatory disease with auto-inflammatory characteristics. The introduction of targeted biologic therapies has revolutionized the treatment and as such improved outcomes for children with sJIA. The IL-1 receptor antagonist, anakinra, is used as first-line treatment for sJIA in the Netherlands since 2008 and has been shown to induce and sustain inactive disease with approximately 50% of patients being able to taper and stop within the first year of disease (1). However, also with this strategy, around 25-30% of patients experience a more refractory disease course, necessitating maintenance therapy. Multiple eposodes of Macrophage Activation Syndrome (MAS), a severe complication of sJIA, has been suggested to be one of the refractory sJIA phenotypes (2). The EULAR/ACR/PRINTO 2016 MAS classification criteria (3) have been developed to facilitate the diagnosis of MAS in sJIA. However, there is still a scarcity of data on their performance in sJIA patients treated with biologicals. Recent studies indicate that treatment of sJIA with biologicals might change some clinical and laboratory features of MAS (4).

**Objectives:** We therefore aimed to describe the (clinical and laboratory) characteristics of sJIA patients who developed MAS while treated with first-line anakinra and to evaluate whether the 2016 MAS classification criteria are still applicable in these patients.

**Methods:** In this cohort study, we used both retrospective data (2008-2016) as well as data from ESTIS a nationwide, prospective multicenter cohort study (2017 onwards) selecting patients who developed MAS during treatment with first-line anakinra. In total, 15 patients were included with at least 1 year of follow up. We described the demographic, clinical, laboratory and immunologic features of MAS in patients started on recombinant IL-1RA therapy as first-line therapy for sJIA.

**Results:** We included fifteen patients, with a total of sixteen episodes of MAS. The estimated incidence of MAS was 15.6% in the first year after start of anakinra (7/45 in the prospective nationwide, multicenter cohort study ESTIS in the period 2017-2021). Eleven out of the fifteen patients were female. A significant share (11/16 episodes) of the MAS episodes occurred within 2 months after the onset of sJIA and four episodes (4/16, 25%) were triggered by a primo EBV infection at the onset of MAS. All episodes of MAS met the 2016 classification criteria for MAS in sJIA.

**Conclusion:** While first-line treatment with anakinra in sJIA results in high response rates and minimal corticosteroid use, this strategy does not seem to lower the risk or incidence of MAS in SJIA in the first year of disease. The EULAR/ACR/PRINTO 2016 classification criteria for MAS are applicable to patients treated with first-line anakinra. We recommend to closely monitor the laboratory features from the classification criteria in (female) patients within the first months after the onset of sJIA and suggest to test all patients at the onset of sJIA on EBV status.

**Patient Consent**

Yes, I received consent

**Disclosure of Interest**

R. Erkens: None declared, L. Huber: None declared, A. Verwoerd: None declared, G. Rogani: None declared, D. Schonenberg: None declared, M. Van den Berg: None declared, W. Armbrust: None declared, E. Legger: None declared, S. Kamphuis: None declared, E. Schatorjé: None declared, E. Hoppenreijs: None declared, J. Swart: None declared, M. Jansen: None declared, J. Van Loosdregt: None declared, B. Vastert Grant / Research Support with: Research grant from SOBI in a public-private grant opportunity (2017-2024), Speaker Bureau with: SOBI and Novartis

**References**

1. Ter Haar *et al.* Arthritis Rheumatol. 2019 PMID: 30848528
2. Erkens *et al.* Rheum Dis Clin North Am. 2021 PMID: 34635293
3. Ravelli *et al.* Arthritis Rheumatol. 2016 PMID: 26314788
4. Schulert *et al.* Arthritis Care Res (Hoboken). 2018 PMID: 28499329

## P082 Macrophage activation syndrome as a rare initial symptoms of systemic lupus erythematosus in a child

### Z. Hikmah

#### Child Heath Department, Universitas Airlangga, Surabaya, Indonesia

##### **Correspondence:** Z. Hikmah

*Pediatric Rheumatology 2023*, **21(Suppl 2):**P082

**Introduction:** Macrophage Activation Syndrome (MAS) is a rare but serious complication of juvenile systemic lupus erythematosus (jSLE) that can occur in children. It is characterized by an uncontrolled activation and proliferation of immune cells called macrophages, leading to cytokine storm and multiorgan dysfunction. D-dimer is a blood test that measures the level of a protein fragment produced when a blood clot dissolves. High levels of D-dimer may indicate the presence of a blood clot or a hypercoagulable state, but it can also be elevated in certain inflammatory conditions, including MAS. MAS is rarely found in children, especially as an initial symptom.

**Objectives:** To present a case with MAS as an initial symptoms in jSLE

**Methods:** A 7-year-old boy came with complaints of persistent high fever for 12 days. Patients complain of severe stomach pain and vomiting. There is joint pain in the area of the right knee, a painful sensation and severe dizziness. From physical examination obtained high fever, hepatosplenomegaly, and limited movement of the right and left joints. Laboratory tests showed elevated triglycerides, increased ferritin, thrombocytopenia, leukopenia, elevated transaminases, and very high levels of D-dimer (88,600 ng/mL). Autoantibody tests show positive ANA and anti-ds-DNA tests.

**Results:** Patients were diagnosed with MAS and SLE and were given methylprednisolone pulse and oral methotrexate therapy. The results of normal echocardiography, CT scan of the head are also normal. Because there is no improvement even the patient is unconscious then patients are treated with intravenous immunoglobulin (IVIG) and significant improvement is obtained. The patient returned home on the 15th day in improved condition, therapy continued in the clinic

**Conclusion:** MAS is a very rare as an initial symptoms of jSLE. Early detection of MAS and finding the cause is very important and can give a better prognosis.

**Patient Consent**

Yes, I received consent

**Disclosure of Interest**

None declared

## P083 Use of rituximab in the treatment of refractory macrophage activation syndrome in patients with systemic juvenile idiopathic arthritis

### I. Kriulin^1,2^, E. Alexeeva^1,2^, T. Dvoryakovskaya^1,2^, K. Isaeva^1^, A. Chomakhidze^1^, O. Lomakina^1^, A. Fetisova^1^, K. Chibisova^1^, E. Krekhova^1^, I. Tsulukiya^1^, M. Botova^1^, N. Kondrateva^1^, M. Shingarova^1,2^, M. Kokina^1,2^, T. Kriulina^1,2^

#### ^1^Rheumatology, National Medical Research Center for Children’s Health; ^2^Pediatric, Sechenov First Moscow State Medical University (Sechenov University), Moscow, Russian Federation

##### **Correspondence:** I. Kriulin

*Pediatric Rheumatology 2023*, **21(Suppl 2):**P083

**Introduction:** Macrophage activation syndrome (MAS) is a complication of systemic juvenile idiopathic arthritis (sJIA), which is a consequence of uncontrolled proliferation of T–lymphocytes and macrophages in combination with cytolytic dysfunction of natural killers and CD8+ T-lymphocytes. For the treatment of primary MAS, the HLH-2004 protocol is used, which includes multi-stage pathogenetic therapy. The use of a number of drugs from this protocol (etoposide, high doses of methotrexate intrathecally) in patients with MAS with sJIA is ineffective and entails many infectious complications. Currently, little information has been accumulated in the world about the effectiveness of MAS therapy in patients with sJIA, including with the use of genetically engineered biological drugs.

**Objectives:** To evaluate the effectiveness of rituximab in the treatment of refractory MAS in patients with sJIA.

**Methods:** The retrospective study included 100 patients with sJIA and MAS, of which 51 (51%) were girls and 49 (49%) boys. All patients were treated in the rheumatology department of national Medical Research Center for Children's Health (Moscow). To assess the effectiveness of therapy, the criteria for achieving a response to therapy and the criteria for the inactive stage of MAS described in the consensus treatment of patients with hemophagocytic lymphohistiocytosis (HLH-2004) were used.

**Results:** Rituximab for the treatment of MAS was used in 5 patients: 1 patient was biologic naive and in 4 patients with MAS that developed against the background of biologic therapy: in 2 patients – kanakinumab, in 1 – adalimumab and in 1 – tocilizumab. All patients were treated with intravenous and oral glucocorticoids (GC), cyclosporine and IVIG. Therapy was ineffective in all children. When trying to reduce the dose of intravenous GC, a recurrence of MAS developed. In 3 patients, the Epstein-Barr virus (detected in the blood by PCR) was the trigger factor for the development of MAS. Against the background of rituximab therapy, the criteria for response to therapy were achieved in all patients, the criteria for the inactive stage of MAS – in 3. In 2 patients, the criteria for the inactive stage of MAS were not achieved. They needed to escalate the dose of oral corticosteroids. The outcomes in this group are distributed as follows: remission was detected in 3 patients, failure to meet the response criteria in 1 patient, and death in 1 patient.

**Conclusion:** A possible therapeutic option for treatment of refractory MAS to standard immunosuppressive therapy may be the use of monoclonal antibodies to CD20+ B lymphocytes of rituximab in combination with GC, cyclosporine and IVIG, especially in the complicated case of Ebstein-Barr virus infection.

**Patient Consent**

Not applicable (there are no patient data)

**Disclosure of Interest**

None declared

## P084 Optimizing treatment in systemic juvenile idiopathic arthritis and macrophage activation syndrome: the metaphor project, a pres/printo initiative

### F. Minoia^1^, F. Baldo^1,2^, R. Erkens^3^, C. Bracaglia^4^, D. Foell^5^, M. Gattorno^6^, M. Jelusic^7^, J. Anton^8^, P. Brogan^2^, S. Canna^9^, S. Chandrakasan^10^, R. Q. Cron^11^, F. De Benedetti^4^, A. Grom^12^, M. Heshin-Beckenstein^13^, A. Horne^14^, R. Khubchandani^15^, M. Mizuta^16^, S. Ozen^17^, P. Quartier^18^, A. Ravelli^6^, M. Shimizu^19^, G. Schulert^12^, C. Scott^20^, R. Sinha^21^, N. Ruperto^6^, J. Swart^3^, S. Vastert^3^ on behalf of on behalf of the PReS MAS/sJIA Working Party and Pediatric Rheumatology International Trial Organization

#### ^1^Fondazione IRCCS Ca' Granda Ospedale Maggiore Policlinico, Milan, Italy; ^2^Great Ormond St Hospital, London, United Kingdom; ^3^University Medical Center Utrecht, Utrecht, Netherlands; ^4^IRCCS Ospedale Pediatrico Bambino Gesù, Rome, Italy; ^5^University Hospital Muenster, Muenster, Germany; ^6^IRCCS Giannina Gaslini, Genoa, Italy; ^7^University Hospital Centre Zagreb, Zagreb, Croatia, ^8^Hospital Sant Joan de Déu, Barcelona, Spain; ^9^Children’s Hospital of Philadelphia, Philadelphia; ^10^Children’s Healthcare of Atlanta, Atlanta; ^11^University of Alabama at Birmingham, Birmingham; ^12^Cincinnati Children’s Hospital, Cincinnati, United States; ^13^Dana Dwek Children's Hospital, Tel Aviv Medical Center, Tel Aviv University, Tel Aviv, Israel; ^14^Karolinska Institute, Solna, Sweden; ^15^SRCC Childrens Hospital, Mumbai, India; ^16^Department of Pediatric Rheumatology, Hyogo Prefectural Kobe Children's Hospital, Kobe, Japan; ^17^Department of Pediatrics, Hacettepe University, Ankara, Türkiye; ^18^Université Paris-Cite, IMAGINE Institute, Necker Children’s Hospital, Paris, France; ^19^Tokyo Medical and Dental University, Tokyo, Japan; ^20^University of Cape Town, Cape Town, South Africa, ^21^Systemic JIA Foundation, Cincinnati, United States

##### **Correspondence:** F. Minoia

*Pediatric Rheumatology 2023*, **21(Suppl 2):**P084

**Introduction:** Despite increasing therapeutic options which are globally improving the care of patients with systemic juvenile idiopathic arthritis (sJIA) and macrophage activation syndrome (MAS), a wide heterogeneity in the current management of these two conditions worldwide still exists, potentially due to differences in treatment strategies and access to medications, lack of evidence regarding therapeutic approaches to refractory patients, and involvement of different specialists.

**Objectives:** To capture the current real-life experience in sJIA and MAS treatment worldwide and to evaluate the major unmet needs from physician and patient perspectives.

**Methods:** The project is conducted through the following steps: 1) systematic literature review on current therapeutic options; 2) development of surveys on a web platform, set up by PRINTO. The main topics are identified by a panel of experts and 2 surveys are developed: one addressed to physicians and a second to patients, both exploring unmet needs to compare perspectives; 3) forwarding physician survey to all the centers in the PRINTO network. MAS survey is forwarded also to Histiocyte Society members, while patient survey is sent through the sJIA Foundation network; 4) data collection; 5) data analysis; 6) final consensus meeting.

**Results:** The project was started in 2022. In the first year, the Steering Committee identified the panel of experts which included: 18 pediatric rheumatologists with high expertise in sJIA and MAS representative of different clinical settings worldwide, 2 pediatric hematologists, 1 young pediatric rheumatologist from EMERGE, 1 patient representative and 1 methodologist. A systematic literature review on MAS treatment was performed from June 2022 to December 2022: the validity of papers was evaluated and scored by experts. Data were extracted in February 2023 and presented to experts, who voted and selected items to be included in the clinical section of the MAS survey. Regarding patient involvement, a first open-ended survey within sJIA Foundation members defined the most relevant issues that may affect treatment by patient opinion; those items will be discussed by a representative core group of international patients and parents and the most relevant aspects will be included in the final surveys. The first survey on MAS treatment will be forwarded by August 2023.

**Conclusion:** The METAPHOR project will improve the knowledge of the pediatric rheumatology community on the current challenges in sJIA and MAS treatment worldwide, to foster the achievement of a uniform approach and to guide future research.

**Patient Consent**

Not applicable (there are no patient data)

**Disclosure of Interest**

F. Minoia Consultant with: SOBI, F. Baldo: None declared, R. Erkens: None declared, C. Bracaglia Consultant with: SOBI, Novartis, D. Foell: None declared, M. Gattorno: None declared, M. Jelusic: None declared, J. Anton: None declared, P. Brogan: None declared, S. Canna: None declared, S. Chandrakasan: None declared, R. Cron Grant / Research Support with: SOBI, Consultant with: SOBI, Sironax, F. De Benedetti: None declared, A. Grom: None declared, M. Heshin-Beckenstein: None declared, A. Horne: None declared, R. Khubchandani: None declared, M. Mizuta: None declared, S. Ozen: None declared, P. Quartier: None declared, A. Ravelli: None declared, M. Shimizu: None declared, G. Schulert Grant / Research Support with: IpiNovyx, Consultant with: SOBI, C. Scott: None declared, R. Sinha: None declared, N. Ruperto: None declared, J. Swart: None declared, S. Vastert: None declared

## P085 The early stages of diagnosis and management of suspected hemophagocytic lymphohistiocytosis/macrophage activation syndrome (HLH/MAS): a systematic literature review

### B. Shakoory^1^, D. Poddigne^2^, O. Kul Cinar^3^, J. Park^4^, M. Romano^4^, A. Geerlinks^4^, M. Wilejto^4^, K. Kernan^5^, M. Hines^6^, D. Piskin^4^, A. Ravelli^7^, R. Goldbach-Mansky^1^, F. de Benedetti^8^, R. Marsh^9^, S. Canna^10^, E. Demirkaya^4^

#### ^1^National Institute of Health, Bethesda, United States; ^2^Department of Medicine, Nazarbayev University School of Medicine (Nur-Sultan) , Nur-Sultan, Kazakhstan; ^3^Department of Paediatric Rheumatology, Great Ormond Street Hospital for Children NHS Foundation Trust, London, United Kingdom; ^4^Schulich School of Medicine, Western University, London, Canada; ^5^University of Pittsburgh/UPMC Children’s Hospital of Pittsburgh, Pittsburgh; ^6^St. Jude Children’s Research Hospital, Memphis, United States; ^7^Istituto Giannina Gaslini; ^8^Università degli Studi di Genova, Genova, Italy; ^9^Cincinnati Children’s Hospital Medical Center, Cincinnati; ^10^The Children’s Hospital of Philadelphia, Philadelphia, United States

##### **Correspondence:** M. Romano

*Pediatric Rheumatology 2023*, **21(Suppl 2):**P085

**Introduction:** Hemophagocytic Lymphohistiocytosis (HLH) and Macrophage Activation Syndrome (MAS) represents a spectrum of life-threatening systemic hyperinflammatory syndromes that can complicate inflammatory contexts. They can progress rapidly, and early identification and management are critical for preventing organ failure and mortality. Practice patterns in recognizing and managing these conditions vary widely.

**Objectives:** A systematic literature review (SLR) focusing on early diagnosis, trigger identification, monitoring procedures and treatment for HLH/MAS was undertaken to inform in pediatric and adult populations.

**Methods:** The PRISMA guidelines were followed for this systematic review. An electronic search of the databases PubMed, EMBASE and Cochrane Library were conducted and all relevant literature up to November 2020 was searched. Studies were included when full-text articles were available, published in the English language and if they reported more than 6 different cases. Information on the study characteristics, and details about diagnosis, triggers, treatment and monitoring were recorded.

**Results:** Of the 18020 articles related to diagnosis, treatment, and monitoring of HLH/MAS from the review of the database, 258 articles were selected for full-text review. Of these, 167 articles were included for data extraction. A total of 17 studies were reviewed to determine which routine laboratory abnormalities were associated with HLH/MAS. CBC abnormalities like leukopenia (especially neutropenia), anemia, thrombocytopenia, elevated acute phase reactants (CRP, ESR, LDH, and/or ferritin), coagulopathy and abnormal CSF findings at the onset of HLH/MAS were characterized through studies. HLH/MAS can be triggered by different factors and could be divided into primary (genetic/familial) and secondary (acquired) subgroups. The list of underlying triggers was broad and varied between pediatric and adult patients. There were 24 articles selected for a treatment review. Overall, four main categories of drugs are used in the treatment of HLH-MAS: systemic glucocorticoids, Intravenous immunoglobulin, etoposide, and anakinra. The use of other drugs is much less consolidated and should be considered for individual indications. Discussion with an expert consultant is highly encouraged due to the absence of firm evidence.

**Conclusion:** The articles in this systematic review included a spectrum of MAS-HLH syndromes associated with various predisposing/underlying conditions and/or triggering infections. In the absence of randomized studies, the only outcomes that would be extracted from mostly uncontrolled studies were survival. While there is an unequivocal benefit of early treatment resulting in improved survival, the SLR also points to the need to conduct better-controlled studies in HLH/MAS with the ultimate goal to improve management and outcome predictions.

**Patient Consent**

Not applicable (there are no patient data)

**Disclosure of Interest**

None declared

**References**

1. Ravelli A, et al. 2016 Classification Criteria for Macrophage Activation Syndrome Complicating Systemic Juvenile Idiopathic Arthritis: A European League Against Rheumatism/American College of Rheumatology/Paediatric Rheumatology International Trials Organisation Collaborative Initiative. Arthritis Rheumatol. 2016;68(3):566-76.
2. Canna SW, Marsh RA. Pediatric hemophagocytic lymphohistiocytosis. Blood. 2020;135(16):1332-43.

## P087 High-dose intravenous anakinra in children with hyperinflammatory sepsis

### M. Trevisan^1^, F. Tortora^2^, A. De Matteis^1^, M. Pardeo^1^, R. Bianchi^2^, F. De Benedetti^1^, C. Bracaglia^1^

#### ^1^Rheumatology Division; ^2^Department of Anesthesia and Critical Care, IRCCS Ospedale Pediatrico Bambino Gesù, Rome, Italy

##### **Correspondence:** M. Trevisan

*Pediatric Rheumatology 2023*, **21(Suppl 2):**P087

**Introduction:** Despite advances in care, sepsis is still a life-threatening condition. In recent years, increasing evidence reported a subgroup of septic patients who develops a “cytokine storm” with features of an HLH/MAS phenotype. Therefore, some authors suggested to treat those patients with the IL-1 inhibitor anakinra ^1^. Indeed, in a post-hoc analysis of the anakinra sepsis trial patients with with disseminated intravascular coagulopathy (DIC) and hepatobiliary dysfunction (HBD) were shown to benefit from treatment with anakinra ^2^.

**Objectives:** To evaluate efficacy and safety of IL-1 blockade treatment in pediatric patients with hyperinflammation related to sepsis.

**Methods:** We performed a retrospective analysis of patients with confirmed sepsis and hyperinflammatory features (ferritin > 800 ng/mL) treated with anakinra. We evaluated mortality rate, DIC according to the ISTH score (DIC if ≥5 points), HBD criteria (bilirubin > 2,5 mg/dL, INR > 1,5 and AST/ALT >2 UNV) and safety events.

**Results:** We retrospectively collected data from 10 patients with proven bacterial sepsis and treated with anakinra in our hospital, all of them requiring ICU admission and intensive care support. Eight patients (80%) were female with a median age of 7 years (0-24). Anakinra was administered at high dose (mean dose 10 mg/kg/day, range 5-20) within 8 days from symptom onset and continued for a median time of 34 days. Most of the patients required concomitant intravenous steroids (80%), antibiotics, renal replacement therapy and inotropic treatment. The mortality rate of the entire cohort was 30% (3/10), the median hospitalization time was 70 days (9-205) and ICU stay 44 days (3-130). At the disease onset, no statistical differences were noted in terms of leukocytes, ferritin level (median 10.049 ng/mL), platelets count (median 71.000/mm3) and other main inflammatory parameters between patients who survived and those who died, except for a higher aspartate aminotransferase (AST) (p<0.03) and prothrombin (PT) time (p=0,07) in those who died. According to Shakoory’s criteria, only three patients presented DIC and HBD criteria, two of them died. No serious or mild adverse events were recorded during treatment with anakinra, even using the intravenous route. Deaths were all related to the preexisting background disease or to sepsis.

**Conclusion:** As previously reported in adult patients with sepsis^1,2^, anakinra seems to be efficacious and safe in a cohort of pediatric septic patients with hyperinflammatory features in the context of sepsis. Although our cohort is small, the overall mortality was 30%, lower than a 60-70% previously reported in hyperinflammatory sepsis^1^. We observed an increased AST level and PT in patients who died. Lastly, anakinra seems to be safe in pediatric sepsis, even at high doses.

**Patient Consent**

Not applicable (there are no patient data)

**Disclosure of Interest**

None declared

**References**

1. Eloseily EM et al. Benefit of Anakinra in Treating Pediatric Secondary Hemophagocytic Lymphohistiocytosis. *Arthritis Rheumatol*. 2020.
2. Shakoory B et al. Interleukin-1 Receptor Blockade Is Associated With Reduced Mortality in Sepsis Patients With Features of Macrophage Activation Syndrome: Reanalysis of a Prior Phase III Trial. *Crit Care Med*. 2016.

## P088 Referral to the paediatric rheumatologist – what could be this cause of macrophage activation syndrome?

### Q. Wu^1^, I. Turtsevich^1^, A. Naheed^1^, S. Eisen^2^, L. Nabarro^3^, A. Bamford^1^, D. Eleftheriou^1^, M. Al Obaidi^1^, E. Moraitis^1^

#### ^1^Great Ormond Street Hospital; ^2^University College Hospital; ^3^Hospital for Tropical Diseases, London, United Kingdom

##### **Correspondence:** Q. Wu

*Pediatric Rheumatology 2023*, **21(Suppl 2):**P088

**Introduction:** Macrophage Activation Syndrome (MAS) is a potentially life-threatening systemic inflammatory condition characterised by cytopenias, liver dysfunction and coagulopathy. It can be secondary to active rheumatological diseases, malignancy and a number of infections.

**Objectives:** Highlight to busy Paediatric Rheumatology teams the importance of ruling out important differentials which can masquerade rheumatic conditions with MAS.

**Methods:** Case report

**Results:** 6-year female was referred to our Paediatric Rheumatology Service for possible systemic onset juvenile idiopathic arthritis (sJIA). She presented with 3-weeks fever, intermittent abdominal pain, mild cough, with travel history to Italy 6-months prior. Positive examination findings were palpable liver edge and shotty cervical lymphadenopathy. Laboratory markers demonstrated worsening Hb 86, falling WCC 4.43, falling platelets 165, low CRP 11, low ESR 3, rising ferritin 1069, rising LDH 1340, worsening transaminitis (AST 740, ALT 586, ALP 307, GGT 121, bilirubin 16), normal fibrinogen, mildly elevated triglyceride 1.98. sCD25 was 3902. Abdominal ultrasound showed splenomegaly with hypoechoic nodules. Echo was unremarkable with normal coronary vessels. Infectious Diseases work up included negative results for Hepatitis A, B and C, CMV, EBV, adenovirus and parvovirus, blood culture, urine culture, and normal CXR.

Our impression was: 1) not typical of sJIA in view of lack of arthritis, rash and quotidian fever, with normal inflammatory markers, 2) cytopenia, transaminitis, hyperferritinaemia and high LDH might represent MAS, which could also be complication of infection or malignancy.

Infectious work-up subsequently showed positive leishmaniasis serology, demonstrating past exposure but not necessarily confirming cause of presenting symptoms. Bone marrow aspirate was performed to confirm positive microscopy and PCR, after which she was started on empiric treatment with liposomal amphotericin B for visceral leishmaniasis under Infectious Diseases team. PCR for *Leishmania donovani* was positive on bone marrow aspirate. A few days after starting treatment, she remained pyrexial with rising transaminitis and LDH, falling albumin and Hb. Treatment with 2mg/kg daily prednisolone was initiated for MAS secondary to leishmaniasis, she improved with complete resolution of fever and normalisation of laboratory parameters.

**Conclusion:** sJIA with MAS is diagnosis of exclusion after ruling out malignancy, infection and other autoinflammatory/autoimmune processes, where multidisciplinary team input across Rheumatology, Infectious Diseases, Immunology and Haematology are valuable. In certain cases, bone marrow aspiration can be critical, when excluding unusual infection as well as malignancy, and where safe and feasible, should be considered before treatment is initiated, especially where clinical features are atypical for underlying Rheumatological causes.

**Patient Consent**

Yes, I received consent

**Disclosure of Interest**

None declared

## P089 tofacitinib experience in pediatric rheumatology patients single tertiary center in Saudia Arabia

### A. Asiri, M. Almarri, A. AlRowais

#### Pediatric , Prince Sultan Medical Military City, Riyadh, Saudi Arabia

##### **Correspondence:** A. Asiri

*Pediatric Rheumatology 2023*, **21(Suppl 2):**P089

**Introduction:** The JAK-STAT signaling pathway plays a crucial role in cell functions, and its dysregulation has been linked to various diseases such as infections, chronic arthritis, and cancer. Targeting this pathway has shown promise in treating immunological and inflammatory conditions, particularly in rheumatoid arthritis. Tofacitinib has been approved by the FDA for the treatment of several diseases, including rheumatoid arthritis and polyarticular juvenile idiopathic arthritis. There are ongoing clinical trials for using JAK-inhibitors in other diseases such as lupus erythematosus and Crohn's disease. However, recommendations for using JAK-inhibitors in pediatric rheumatic diseases and its optimal dosage and safety are still not fully evaluated.

**Objectives:** To assess effectiveness and possible side effects of tofacitinib in several pediatric rheumatology with different indications

**Methods:** Retrospective medical chart review of total 6 patients in pediatric rheumatology clinics

2 with poly Articular JIA and 2 systemic JIA with poly Articular patterns and 3 with interpheronopathy (monogenic lupus with confirmed genetic defect)

**Results:** Complete remission was achieved in 3/7 patients; 3 among JIA patients, 1/3 among monogenic lupus have partial response two non-responders with c1q deficiency monogenic lupus discontinued tofacitinib. Corticosteroids were successfully tapered off in 4/7 patients and discontinued in 2/7patients. 3 patients had side effects not requiring treatment discontinuation: liver enzyme elevation (n = 2), mild skin allergy (n = 1).1 patient developed mild varicella infection and pneumonia required stopping medication

**Conclusion:** Tofacitinb are effective new therapies for the treatment of several immune-mediated diseases. In our center jak inhibitors has shown the best results in patients with JIA and and reasonable effectiveness in type I interferonopathies .randomized controlled clinical trials are needed to use JAK-inhibitors safely in pediatric rheumatic diseases and explore new indication

**Patient Consent**

Not applicable (there are no patient data)

**Disclosure of Interest**

None declared

## P090 Leflunomide as an add on drug to methotrexate in the treatment of juvenile idiopathic arthritis

### J. N. Bathia^1^, S. Sam^2^, P. Pal^1^

#### ^1^Pediatric Rheumatology; ^2^Pediatric Medicine, Institute Of Child Health, Kolkata, India

##### **Correspondence:** J. N. Bathia

*Pediatric Rheumatology 2023*, **21(Suppl 2):**P090

**Introduction:** Juvenile Idiopathic Arthritis (JIA) is the most common chronic rheumatic disease in children. Non biologic disease modifying anti-rheumatic agents (DMARDS) are the first line of therapy, commonly methotrexate (Mtx). Recent data suggests early use of biologic DMARDS. However, in resource limited countries, use of biologics remains a financial barrier. Leflunomide has been used as a cheaper additive DMARD in those with inadequate response to Mtx.

**Objectives:** To note the response to leflunomide (Lef) as a second add on drug after atleast three months of methotrexate therapy.

**Methods:** This is a retrospective data analysis of children with JIA at Institute of Child Health, Kolkata. JIA was defined as per ILAR criteria. Patients who had received Lef as an additive drug, atleast three months after initiation of Mtx, were included. Those receiving therapy apart from Mtx and Lef combination were excluded. Study period was 18 months. Response was noted as per Wallace criteria.

**Results:** 52 patients received Leflunomide due to suboptimal response to three months of Mtx. Most common JIA subtype who received Lef following Mtx was Systemic JIA 46.2% (24/52) followed by Oligoarticular JIA 25% (13/52), Rheumatoid factor (RF) negative polyarticular JIA 19.2% (10/52), RF positive polyarticular JIA 7.7% (4/52) and enthesitis related arthritis (ERA) 1.9% (1/52).

67.3% (35/52) achieved remission as defined by the Wallace criteria. The remaining 17 patients required additive therapy. RF negative polyarticular JIA had the highest remission rate post Lef 80% (8/10), followed by oligoarticular JIA 69.2 %(9/13), Systemic JIA 62.5 % (15/24) and RF positive polyarticular JIA 50% (2/4). Median time to achieve remission as per Wallace criteria was 3 months. 95% of patients who achieved remission continued to be in remission on combination of Mtx and Lef. 11/52 (20%) patients had transient transaminitis (upto 2-3 times the upper normal) which normalized within 15 days of stopping Lef. Reintroduction of Lef at lower doses with gradual escalation to the required dose did not result in any further elevation of the liver enzymes.

**Conclusion:** Addition of Lef in JIA patients having suboptimal response to Mtx has a favorable outcome with 67% achieving remission without increase in toxicity and should be considered in developing countries with poor affordability for biologics.

**Patient Consent**

Yes, I received consent

**Disclosure of Interest**

None declared

## P091 Change of biologic unmasks orofacial granulomatosis in a patient with juvenile idiopathic arthritis

### S. Coyne, N. Keenan, J. MacMahon, E. J. MacDermott

#### National Centre for Paediatric Rheumatology, CHI at Crumlin hospital, Dublin, Ireland

##### **Correspondence:** S. Coyne

*Pediatric Rheumatology 2023*, **21(Suppl 2):**P091

**Introduction:** Rates of Inflammatory Bowel disease (IBD) are higher in children with Juvenile idiopathic arthritis (JIA) than the general population (1). Orofacial Granulomatosis (OFG) is a rare disorder and is increasingly recognised as a subtype of Crohn’s disease (2).

**Objectives:** To describe an unusual presentation of IBD related arthropathy

**Methods:** A 14-year-old boy with polyarticular JIA, presented with lip swelling and blistering two hours post first tocilizumab infusion. Lip swelling was again noted with subsequent tocilizumab infusions, raising concern for drug hypersensitivity. The patient described onset of swelling several hours post infusion, worsening over days and notably, without full resolution. No other symptoms of IgE-mediated drug allergy, (e.g. respiratory compromise, urticaria, cardiovascular collapse) were observed. His symptoms did not resolve when tocilizumab infusions were stopped. On further questioning, the patient reported new onset diarrhoea and subjective weight loss, which began at the same time. Of note, the patient had been receiving Adalimumab prior to starting Tocilizumab. No new connective tissue symptoms such as rash, fever or hair loss were recorded. No other joints were active on rheumatological exam.

**Results:** On examination, the lip swelling was felt to be in keeping with OFG. His inflammatory markers were within normal limits, ESR 11mm/hr (1-13), Platelets 243 x10^9/L (150-400), CRP <5mg/l (<10 ). His connective tissue screen was slighty above normal range at 0.9 (<0.69). His faecal calprotectin was elevated at 98ug/g (<50). In view of his other new symptoms and temporal association with cessation of anti-TNF therapy, an urgent GI review was arranged. OGD with biopsy was organised. Pathology revealed an increase in lamina propria cellularity with focal active cyptitis and well form epithelioid granulomas. This supported a diagnosis of Crohn’s disease.

**Conclusion:** As inflammatory bowel disease is more common in children with JIA, patients should always be asked regarding gastrointestinal symptoms. This case also highlights the importance of thorough history taking in the context of a potential drug reaction. Tocilizumab hypersensitivity reactions are rare, and the description was not in keeping with IgE-mediated or delayed drug reaction. A key finding here was that the lip swelling did not resolve between doses and had associated cheilitis. Gastrointestinal symptoms began in the ‘wash out’ period changing from adalimumab, an anti-TNF inhibitor that is used to treat IBD, to tocilizumab, an IL-6 inhibitor which has been reported to aggravate underlying or undiagnosed IBD (3). OFG is a known manifestation of Crohn’s disease and 40% of paediatric patients with OFG have intestinal involvement (2).

**Patient Consent**

Yes, I received consent

**Disclosure of Interest**

None declared

**References**

Barthel D et al. The Journal of Rheumatology, 2015;42(11):2160-2165.
Lazzerini M et al. Association between orofacial granulomatosis and Crohn's disease in children: systematic review. World J Gastroenterol. 2014;20(23):7497-504.
3. Borghini R et al. Onset of Suspected Ulcerative Colitis After Treatment with Tocilizumab in Patient with Celiac Disease and Juvenile Idiopathic Arthritis, Inflammatory Bowel Diseases. 2021;27(6)76-78.

## P092 A rare case of delayed hypersensitivity after anakinra administration in a patient with systemic juvenile idiopathic arthritis

### C. De Simone^1^, F. Fedele^1^, M. Tardi^2^, E. Sammarco^3^, F. Barbato^3^, L. Martemucci^2^, F. Orlando^2^

#### ^1^Department of Translational Medical Science, University of Naples “Federico II”; ^2^Department of General and Emergency Pediatrics, Santobono-Pausilipon Children’s Hospital, Pediatric Rheumatology Unit; ^3^Department of General and Emergency Pediatrics, Santobono-Pausilipon Children’s Hospital, Pediatric Dermatology Unit, Naples, Italy

##### **Correspondence:** C. De Simone

*Pediatric Rheumatology 2023*, **21(Suppl 2):**P092

**Introduction:** Anakinra is an IL-1 receptor antagonist that inhibits the activity of both IL-1α and IL-1β. It is widely used for the treatment of autoinflammatory diseases in pediatric age. According to Medical Dictionary for Regulatory Activities (MedDRA) skin and subcutaneous tissue disorders related to Anakinra are uncommon (≥ 1/1,000 to < 1/100). Drug-induced hypersensitivity syndrome (DIHS) is an acute autoimmune reaction thought to be mediated by T cells and involving a variety of regulatory mechanisms, although not specifically understood.

**Objectives:** To describe a case of DIHS due to Anakinra.

**Methods:** The patient is a female, ten years old, affected by systemic Juvenile Idiopathic Arthritis (sJIA) complicated by macrophage activation syndrome (MAS) diagnosed in August 2022. She was initially treated with steroids associated to subcutaneous Anakinra at the dosage of 2 mg/kg. Due to the worsening of laboratory parameters Anakinra was incremented to 3 mg/kg without response, then it was shifted to parental administration at the dosage 4 mg/Kg/die associated to endovenous Metilprednisolone, finally obtaining clinically and laboratory response. Patient was discharged with oral Prednisone (1 mg/kg) and subcutaneous Anakinra (5 mg/kg/die). Approximately forty days after starting Anakinra, the patient presented diffuse maculo-erythematous rash. Given the apyrexia and the normal results of laboratory parameters, excepted for LDH 530 U/L, an adverse reaction to Anakinra was suspected and the dosage of the drug was decreased to 3 mg/kg without benefits. During next 7 days, the rash worsened and ecchymotic area with exfoliative reaction at the drug injection site was evidenced. Due to the persistence of apyrexia and negativity of inflammatory indexes during decalage of steroids (0.5 mg/Kg/die), Anakinra was stopped. The patient presented diffuse exfoliative dermatitis with restitutio ad integrum in ten days after discontinuation of Anakinra.

**Results:** According to European Registry of Severe Cutaneous Adverse Reactions to Drugs and Collection of Biological Samples (RegiSCAR) Score DIHS was probable. Diagnostic confirmation of DIHS to Anakinra was provided by the gradual resolution of the rash after drug discontinuation without relapse of sJIA.

**Conclusion:** Clinical experience indicates that Injection site reactions (ISRs) are the most common skin adverse reaction due to Anakinra, generally within the first weeks of initiating therapy. ISRs are defined as a constellation of symptoms including swelling, erythema, pruritus, and pain around the injection site. On the other hand, DIHS usually occurs 1-3 weeks after the first administration of the causative medication and it can cause mild to severe mucosal and cutaneous reactions. Among DIHS ,the drug reaction with eosinophilia and systemic symptoms (DRESS), has rarely been reported in patients treated with Anakinra, predominantly in patients with sJIA, however our patient did not show eosinophilia. To our knowledge, it has been reported only one case^1^ of DIHS due to Anakinra in a 2-year-old male with sJIA and MAS.

**Patient Consent**

Yes, I received consent

**Disclosure of Interest**

None declared

**Reference**

1. Villacis-Nunez DS, Bilcha K, Spraker M, Rouster-Stevens K, Cooley A. Severe Immediate and Delayed Hypersensitivity Reactions to Biologics in a Toddler With Systemic Juvenile Idiopathic Arthritis. J Investig Med High Impact Case Rep. 2022 Jan-Dec;10:23247096221077836. doi: 10.1177/23247096221077836. PMID: 35225032; PMCID: PMC8891877.

## P093 A real-world experience on the efficacy of biosimilar adoption in children with juvenile idiopathic arthritis

### M. F. Gicchino, A. N. Olivieri, A. Amodio, G. Capasso, E. Miraglia del Giudice, A. Di Sessa

#### Department of Woman, Child and General and Specialized Surgery, University of Campania Luigi Vanvitelli, Napoli, Italy

##### **Correspondence:** M. F. Gicchino

*Pediatric Rheumatology 2023*, **21(Suppl 2):**P093

**Introduction:** Juvenile Idiopathic Arthritis (JIA) represents the most common chronic rheumatic disease in childhood affecting joints and other structures. According to International League of Association for Rheumatology (ILAR), seven subtypes of arthritis can be defined in relation with the number of joints and the extra-articular involvement occurring in the first six months of disease. Non steroidal antinflammatory drugs (NSAIDs) and intra-articular steroids represents the first line treatment for JIA. Systemic steroids, disease modifying anti-rheumatic drugs (DMARDs) and biologic drugs are used in children with severe disease. In the last 20 years, the use of biologic drugs such as tumour necrosis factor a inhibitors (TNF-a inhibitors) has dramatically improved JIA outcomes. While many biologics are still covered by patents, some others, such as Etanercept (ETA) and Adalimumab (ADA) are now available as biosimilars (BIOs) because their patents have expired. To date, there is limited real-world evidence about the use of biosimilars (BIOs) in children with Juvenile Idiopathic Arthritis (JIA).

**Objectives:** To evaluate the efficacy profile of BIOs compared to their Etanercept (ETA) and Adalimumab (ADA) originators in children with JIA.

**Methods:** We retrospectively examined 59 children (mean age 7.88±4.44) with JIA treated with BIOs of ETA or ADA in follow up at our Department. 18 patients were in treatment with ETA originator; 19 patients with ADA originator; 18 with ADA BIOs, and 5 with ETA BIOs. The efficacy profile of TNF-inhibitors therapy was evaluated at baseline, at 1-(T1), 3- (T2), and 6- (T3) months. Inflammation markers (C-reactive protein and erythrocyte sedimentation rate), number of active joints, Juvenile Arthritis Disease Activity Score (JADAS 10), were assessed at every observation point to define disease activity. Relapses were defined according to Wallace criteria. Pain at injection site was evaluated through visual analogic scale (VAS). Both anti-Adalimumab and anti Etanercept antibodies were also assessed at T1, T2 and T3.

**Results:** VAS at injection site was significantly lower in JIA children treated with BIOs compared to those with originators (p= 0.001 and p=0.01, respectively). More, higher JADAS -10 scores were reported at baseline, at T1 and T2 months in JIA children treated with BIOs than in those receiving originators (p=0.001, p=0.01, and p=0.005, respectively). No differences were found for JADAS-10 at T3 between the two groups (p=0.29). Relapses were significantly lower for BIOs compared to originators (p=0.01). At T6, patients treated with ADA or ETA originator showed significant higher anti-drug autoantibodies levels than those treated with BIOs (p=0.04, p=0.03 respectively) . None of patients experienced side effects.

**Conclusion:** Although preliminary, our findings supported a long-term efficacy and safety profile of BIOs in children with JIA.

**Patient Consent**

Yes, I received consent

**Disclosure of Interest**

None declared

## P094 Navigating the specific carbohydrate diet for juvenile idiopathic arthritis: child and parental experiences

### N. Hagström^1^, E. Lövestam^2^, A. Koochek^2^, L. Berntson^1^

#### ^1^Women's and Children's Health; ^2^Food Studies, Nutrition and Dietetics, Uppsala University, Uppsala, Sweden

##### **Correspondence:** N. Hagström

*Pediatric Rheumatology 2023*, **21(Suppl 2):**P094

**Introduction:** Insights into the immunological role of the gastrointestinal tract in autoimmune conditions, such as juvenile idiopathic arthritis (JIA), have opened the door to diet as a potential adjunctive treatment option. The only diet studied thus far is the specific carbohydrate diet (SCD) showing promising results. However, prior to further investigation, it is essential to gain insights into the participants' perspectives regarding the SCD and its feasibility, acceptability, and perceived efficacy as a therapeutic approach for JIA.

**Objectives:** to conduct a qualitative evaluation focused on the experiences of children and parents regarding the intervention, how they navigated challenges, and their support needs.

**Methods:** Semi-structured interviews were carried out with 12 children and young adults with JIA and 15 parents from 13 families. The interviews consisted of both individual and shared parts and the transcripts were analyzed using systematic text condensation.

**Results:** A majority of the interviewees perceived the intervention beneficial, and 12 out of 13 reported numerous positive effects while adhering to the diet. These effects included reduced pain, morning stiffness and normalized gastrointestinal function. Most were willing to redo the intervention in its current form. While the challenges were many, all continued the diet for one to three months, and some adhered to a modified version for up to 24 months. For parents, the main challenge was *managing practical issues,* while the children and young adults struggled with *dealing with socioemotional consequences*. To manage practical aspects, parents utilized preparation and planning, and some involved the children in the kitchen. Socioemotional consequences were addressed by choosing to focus on the positive and relying on a supportive and encouraging environment. Despite implementing these adaptations, areas requiring additional support included finding simple, quick, and child-friendly solutions, strengthening organizational food skills such as meal planning and socioemotional communication, and preparation.

**Conclusion:** Considering the overall positive experiences and attitudes of the interviewees, dietary interventions like the SCD may be considered a suitable target for further research. However, based on the reported challenges and participants’ suggestions, the intervention could be optimized by: 1) revising the recipe booklet, adding more quick, simple, and child-friendly recipes; 2) adding an educational part on meal planning; and 3) preparing families to handle socioemotional challenges and providing professional socioemotional support. Our study may offer valuable guidance to other researchers considering the feasibility of similar interventions or trials.

**Patient Consent**

Yes, I received consent

**Disclosure of Interest**

None declared

## P095 Evaluation of the clinical practice strategies (CLIPS) in juvenile inflammatory rheumatism in four regions of the world

### J. Yan^1^, C. Lanna^2^, D. Dey^3^, M. Hofer^4^; on behalf of JIR-CliPS network

#### ^1^Paediatric-Rheumatology, Starship Child Health and University of Auckland, Auckland, New Zealand; ^2^Rheumatologist, Medical School, Federal University of Minas Gerais State (UFMG), Belo Horizonte, Brazil; ^3^Rheumatology Unit, Department of Medicine and Therapeutics, University of Ghana, Accra, Ghana; ^4^Pediatric immune-Rheumatology, CHUV, University of Lausanne , Lausanne, Switzerland

##### **Correspondence:** M. Hofer

*Pediatric Rheumatology 2023*, **21(Suppl 2):**P095

**Introduction:** Juvenile Inflammatory Rheumatism (JIR) is a group of rare inflammatory diseases with an onset during childhood or adulthood. Many affected patients will require lifelong medication. Although evidence or consensus-based recommendations for diagnosis and treatment exist, they are difficult to implement in a real-life setting due to the wide variation of (country-specific) medical systems and financial capabilities. Furthermore, the variable training of physicians in these rare diseases may also influence the treatment plans.

**Objectives:** The main objective is to collect real-life clinical practice strategies (CliPS) from physicians all over the world who care for these patients with the selected inflammatory conditions.

Our secondary objectives are to assess the reasons for discrepancy with existing guidelines and to compare, in a prospective manner, the different CliPS regarding indications, treatment adjustments and long-term patient outcome evaluation.

**Methods:** We selected five areas of interest and developed questionnaires: Lupus nephritis / IgA vasculitis and Kawasaki Disease / Use of bDMARDs in genetic AID / PFAPA and SURF / sJIA and AOSD. Questionnaires were finalized in 2022 and a pilot study started with a WUN grant in Brazil, Ghana, New-Zealand and Switzerland.

Since June 2022, the online questionnaires have been distributed to all physicians caring for patients with diseases within our five areas of interest. The questionnaires are still available online.

The medical systems and the local constraints of the four countries have been described. Data collected until the 7th of November 2022 has been analyzed.

**Results:** Brazil is the largest country with the most people. Switzerland is the most dense. Ghana’s youth make up 46% of the population. The Swiss specialist to rheumatology patient ratio is 1:356 compared to that in Ghana 1:94666. Families in Ghana have to pay for the majority of health expenses and medication, with limited access to biologics. All countries share the lack of recognition of symptoms and signs of these rare conditions by caregivers and referrers, and inequity in the care that young patients closer to large cities and university/tertiary hospitals receive compared with those from more distant areas.

During a six month period, 80 physicians from these four countries answered the questionnaires.

**Conclusion:** The four countries differ vastly in location, size, geography, population size and density, availability and funding of specialist care and essential medications, all factors that influence physician's clinical practice and delivery of healthcare. Although the challenges affecting patients and physicians are similar, different solutions are necessary because of the local specificities. Most international treatment guidelines, developed in North America and Western Europe, are difficult to apply in other countries. There is a need to offer a range of solutions which could be adapted to every local setting. The JIR-CliPS project aims to recommend different strategies to give the best possible care to the patients around the world.

**Patient Consent**

Not applicable (there are no patient data)

**Disclosure of Interest**

None declared

## P096 Treatment of JIA subsequent to intraarticular steroid injection – a survey of german pediatric rheumatologists

### S. Meister^1^, G. Mathias^1^, B. Hügle^2^, H. Johannes-Peter^2^

#### ^1^Physiotherapy; ^2^Rheumatology, German Center for Pediatric and Adolescent Rheumatology, Garmisch-Partenkirchen, Germany

##### **Correspondence:** B. Hügle

*Pediatric Rheumatology 2023*, **21(Suppl 2):**P096

**Introduction:** No evidence-based recommendations exist for the treatment of JIA subsequent to intraarticular steroid injections (IACSI) in patients with juvenile idiopathic arthritis (JIA). No current data is available on the specific treatment algorithms in various pediatric rheumatologic institutions treating these cases.

**Objectives:** The aim of this survey was to determine the current standard of treatment following IACSI in JIA in pediatric rheumatologic institutions.

**Methods:** An online survey regarding the treatment subsequent to IACSI of the lower extremity in patients with JIA was developed. Questions were designed by physicians and physiotherapists; topics included: target joint of the IACSI, drugs used in injection, mode of sedation, treatment as in- or outpatient. Also included in the survey were questions regarding physiotherapy, bed rest, reduction of strain/weight, including direct and out-patient recommendations.103 institutions that are listed by the German Society of Pediatric and Adolescent Rheumatology (GKJR) were contacted via SurveyMonkey. Survey responses were analyzed using descriptive statistics.

**Results:** 51 responses (49 complete responses) from 49 centers were analyzed (response rate 48%). Median number of IACSI annually per institution was 30 (range 0 – 2200), average number was 109 ±324. Institutions injected the following joints: knee 100%, ankle 91%, hands and finger joints 85%, elbow 85%, hips 50%, shoulder 44%. IACSI were performed as out-patient in 24%, partly in-patent in 21% and as an in-patient in 56%. 11% of institutions did not prescribe bed rest, 37% for the day of the IACSI, 37% for one day and 15% for three days after IACSI. 61% of institutions prescribed physiotherapy following IACSI. Recommendations regarding reduction of strain (full, partly, none) varied according to the injected joint: hip joint 9%/32%/43%, knee 17%/47%/38%, ankle 17%/47%/36. 62% of institutions support evidence-based criteria for recommendations regarding reduction of strain.

**Conclusion:** Due to the necessary sedation and analgesia in children most IACSI are performed in a (partly) in-patient setting. Experiences, recommendations and strategies differ significantly between institutions, but evidence-based criteria for post-IACSI treatment of JIA patients would be welcomed by most institutions. Based on this data a multicentric prospective study regarding post-IACSI treatment is suggested.

**Patient Consent**

Not applicable (there are no patient data)

**Disclosure of Interest**

None declared

**Reference**

1. Wallen MM, Gillies D. Intra-articular steroids and splints/rest for children with juvenile idiopathic arthritis and adults with rheumatoid arthritis. Cochrane Database of Systematic Reviews 2006, Issue 1. Art. No.: CD002824.

## P097 Rituximab usage in sjögren's syndrome in children: literature review and clinical cases

### V. Melnikova, K. Belozerov, R. Raupov, E. Kalashnikova, V. Masalova, E. Isupova, E. Gaidar, M. Kaneva, T. Kornishina, I. Chikova, O. Kalashnikova, V. Chasnyk, M. Kostik

#### Hospital Pediatric, Saint Petersburg State Pediatric Medical University, Saint-Petersburg, Russian Federation

##### **Correspondence:** M. Kostik

*Pediatric Rheumatology 2023*, **21(Suppl 2):**P097

**Introduction:** Sjögren's syndrome (SS) is an autoimmune disease characterized by the involvement of exocrine glands. The diagnosis is based on swelling of salivary, lacrimar and other exocrine glands and some systemic features: arthralgia, chronic muscle fatigue, neuropathy, hematological abnormalities, and specific laboratory (SSA antibodies (anti-Ro)) and instrumental tests (Schirmer test). The most common manifestation of SS is recurrent parotitis in childhood. The treatment of SS includes steroid therapy, hydroxychloqine and non-biologic DMARDs. In some refractory cases rituximab (RTX) might be use, which is rare in paediatrics.

**Objectives:** to analyse cases of SS in children treated with RTX.

**Methods:** retrospective analysis of 3 cases, literature review.

**Results:** *Patient 1* (girl, 15 yo). The onset of SS was at 7 yo and it was manifested with dermatitis, fever (39 °С) and cytopenia. Five years later the right inguinal started being painful, also were revealed the increase of gamma-globulins (36.6%), rhematoid factor (RF, 128.8 IU/ ml) levels and bilateral sialadenitis and parenchymal parotitis by ultrasound (US). She had positive SSa/Ro-52 and antinuclear antibodies (ANA, 1:10240). Four weekly infusions of RTX 500 mg was administered due to high laboratory activity and cytopenia. After 2 years no signs of active disease have been found in the follow-up, ANA was 1:2560, RF 67.3 IU/ ml.

*Patient 2* (girl, 15 yo). At the age of 13 she had facial asymmetry, dry mouth, arthralgia and myalgia. There were high levels of RF (823 IU/ml), ESR (60 mm/h). Two years after she had hemorrhagic rash, cytopenia and hypergammaglobulinemia (HGG), ANA 1:1280, and positive SSA/Ro-52, SS-B, SS-A. US of the salivary glands showed parotitis. RTX was administered due cytopenia, high autoimmune activity and fascial asymmetry. After 1 year positive dynamic has been noted, but facial asymmetry is present.

*Patient 3* (girl, 12 yo). The onset of the disease was at 11 yo. It was characterised by weakness and dyspnea. She was hospitalised and high levels of RF (132 IU/ml), HGG and hypoxemia were detected. Also she was diagnosed by pulmonary arterial hypertension (PAH) by echocardiogram (90 mm Hg). The patient was positive for ANA, SS-A, SS-B and Ro-52. Because of PAH she was treated with RTX. At the last check-up (6 month later) her activity of the disease were under control but without direct influence on PAH, which was controled with combination of bosentan and sildenafil.

**Conclusion:** SS is a rare disease in childhood. RTX might be a treatment option for refractory SS.

**Patient Consent**

Yes, I received consent

**Disclosure of Interest**

None declared

**References**

1. Luppi, Fabrizio et al. “Lung complications of Sjogren syndrome.” *European respiratory review : an official journal of the European Respiratory Society* vol. 29,157 200021. 18 Aug. 2020
2. Lieberman, Scott M. “Childhood Sjögren syndrome: insights from adults and animal models.” *Current opinion in rheumatology* vol. 25,5 (2013): 651-7.

## P099 Safety and efficacy of anti-tnf -a biosimilars in children: preliminary results from the Bio_Junior (Biosimilar Versus Originator in Juvenile Idiopathic Arthritis) study

### G. Martini, G. Minca, M. Fiorito, F. Vittadello, A. Meneghel, F. Tirelli, M. Mazzarolo, M. E. Zannin, F. Zulian

#### Department of Woman and Child Health, University of Padova, Padova, Italy

##### **Correspondence:** G. Martini

*Pediatric Rheumatology 2023*, **21(Suppl 2):**P099

**Introduction:** Biosimilars (BIO) drugs present similar, but not completely equivalent, efficacy and immunogenicity to an approved biologic agent. In recent years the lower price of BIO allowed a wide use of these drugs but large-scale studies on their use in children with JIA are lacking.

**Objectives:** To evaluate safety and efficacy of anti-TNF-a biosimilars in a cohort of children with JIA naïve to biological disease modifying drugs (bDMARDs)

**Methods:** Retrospective comparative cohort study including patients with JIA followed at our department from 2008 to now. Patients treated with adalimumab BIO and originator (ORI) for >9 months were included, demographical data, disease course and side effects were analysed.

**Results:** One hundred-thirteen patients naïve to bDMARDs were included (102 JIA, 6 psoriatic arthritis, 5 enthesitis-related arthritis). The BIO (34 patients) and ORI (79 patients) groups were homogeneous for clinical subtypes, age at onset, positivity for Antinuclear antibodies, HLA-B27 and Rheumatoid Factor, conventional DMARDs (cDMARDs) treatment and ESR and CRP values. No statistically significant difference between the 2 groups were observed in: time to achieve disease remission (median 8 months in both), rate of patients able to reduce cDMARDs and incidence of infections (11.8% in ORI, 6.1% in BIO). Side effects were mild (pain at injection site and headache) and reported by 14.2% and 14.7% in the ORI and BIO group, respectively. At anti-TNF-a start patients in the ORI group presented significantly longer duration of cDMARDs treatment, greater rate of uveitis history, higher JADAS scores and uveitis activity. Disease remission, defined as JADAS score £1.5 for >6 months, was achieved in 50% and 80% patients in ORI and BIO group, respectively (*p<0.05*).

**Conclusion:** Our results show that in patients naïve to bDMARDs BIO are effective and safe. In recent years, an earlier use of bDMARDs due to the treat to target (T2T) strategy and to availability of more affordable BIOs seems to favourably influence the clinical outcome, but we need prospective studies to confirm it.

**Patient Consent**

Not applicable (there are no patient data)

**Disclosure of Interest**

None declared

## P100 Pilocytic astrocytoma in adolescent under the TNF-inhibitors therapy: a clinical case of unexpected finding

### I. Nikishina, V. Matkava, A. Arefeva, S. Arsenyeva, L. Medyntzeva, T. Pachkoria, M. Shalygina

#### Paediatric, V.A. Nasonova Scientific Research Institute of Rheumatology, Moscow, Russian Federation

##### **Correspondence:** V. Matkava

*Pediatric Rheumatology 2023*, **21(Suppl 2):**P100

**Introduction:** Patients (pts) receiving Biologics especially TNF-α inhibitors (TNF) require for monitoring because of potential risk of cancer development. In pediatric practice all insignificant manifestation, e.g., neurologic symptoms, requires detailed examination. Pilocytic astrocytoma (PA) is the most common benign brain tumor among children which is characterized by slow growth rate in the cerebellum and chiasmatic/hypothalamic region.

**Objectives:** To describe a clinical case of newly diagnosed PA which was detected by routine radiological assessment in patient with Juvenile Idiopathic Arthritis who was treated by TNF-inhibitors.

**Methods: Case report:** Boy, 16 y.o. was admitted to our clinic with diagnosed polyarticular course of JIA. Since Jule 2020 he had suffered from arthritis of knees, wrists after trauma and viral infection. He was treated by methotrexate 15 mg/week, NSAIDs, short course of oral corticosteroids 4 mg/day and repeated intraarticular steroid injections with insufficient effect. TNF-a inhibitor golimumab subcutaneously 50 mg/month was initiated in May 2021 due to persistent arthritis of metacarpophalangeal and phalangeal hand joints, knees, hip joints, manifestation of temporomandibular joint (TMJ) arthritis, elevated inflammatory markers (ESR, CRP), high rate of JADAS-10 (22 points).

**Results:** Patient showed a good clinical response to therapy with decreased JADAS-10 score (6 points). During dynamic observation in December 2022 MRI of TMJ was performed due to progressive pain syndrome and limitation of motion. There were MRI signs of instability of the left TMJ with disc subluxation. Imaging area included solitary-cystic structure changes in the brain substance on the left side at the level of the midbrain and subcortical nuclei. Detailed examination observed mild abnormalities in neurologic status: mild paresthesias and weakness in the right lower extremity, syndrome of distal symmetric sensory polyneuropathy was verified by neurologist. Considering MRI findings of newgrowth in brain, biologic therapy was withdrawn immediately. Patient was admitted to neurosurgical department. Brain MRI with contrast agent confirmed solitary-cystic neoplasm with size 50.5 mm x 40.2 mm x 41.6 mm in the left basal nuclei, in the left thalamus and left midbrain projection with possible glial genesis. Newgrowth finding combined with clinical manifestation of right-sided hemiparesis were indication for microsurgical removal in Neurosurgery Research Center. Histology verified a low malignancy pilocytic astrocytoma. Because of good result of surgeon treatment chemotherapy didn`t administrate. Methotrexate was restarted. Further indication of Biologics requires more time of observation.

**Conclusion:** This clinical case should increase awareness among specialists about newgrowth under the biologic therapy. There is no confidence about genesis of PA: as a consequence of GOL treatment or self-growth condition. Detailed radiological assessment including visualization of TMJ detected unexpected finding of brain tumor despite the negligible neurological symptoms. Early surgical removal of tumor mass with histological confirmation allow to avoid chemotherapy prescription.

**Patient Consent**

Yes, I received consent

**Disclosure of Interest**

None declared

## P101 Janus kinase inhibition in an infant with the type 1 interferonopathy Aicardi-Goutières-Syndrome 7

### J. B. Reichelt^1^, J. Gburek-Augustat^1^, M. K. Bernhard^1^, D. Gräfe^2^, M. A. Lee-Kirsch^3^, A. Merkenschlager^1^, C. Klemann^4^

#### ^1^Hospital for Children and Adolescents, Department of Pediatric Neurology; ^2^Hospital for Children and Adolescents, Department of Pediatric Radiology, University Hospital Leipzig, Leipzig; ^3^Department of Pediatrics, Medical Faculty Carl Gustav Carus, Technical University Dresden, Dresden; ^4^Hospital for Children and Adolescents, Department of Pediatric Immunology, Rheumatology and Infectiology , University Hospital Leipzig, Leipzig, Germany

##### **Correspondence:** J. B. Reichelt

*Pediatric Rheumatology 2023*, **21(Suppl 2):**P101

**Introduction:** The Aicardi-Goutières-Syndrome type 7 (AGS7) is a rare monogenic type 1 interferonopathy caused by pathogenic variants in the *IFIH1*-gene. Janus kinase inhibition (JAKi) with Ruxolitinib (RXL) may inhibit the interferon response and thus pose a promising therapeutic option.

**Objectives:** To report the use of RXL in an infant with AGS7.

**Methods:** Review of patient charts, imaging, and laboratory data.

**Results:** A male hypotrophic neonate presented with cholestasis, acute liver failure, splenomegaly, and convulsive seizures at 12 days of age. Cranial magnetic resonance imaging (MRI) showed bilateral hyperintense supratentorial lesions at the basal ganglia. Trio-Exome sequencing revealed an autosomal-dominant heterozygous de-novo mutation in *IFIH1*. JAKi treatment with 14 mg/m^2^ of RXL was initiated at four months of age. Subsequently, the patient improved clinically, started to gain weight, and continuously gained developmental skills. After initiating RXL, liver function tests (LFTs) decreased from > 5 times above the upper limit to almost normal levels. Likewise, sIL-2R markedly dropped upon initiating treatment. During the 19 months of RXL treatment, improvement in multiple developmental domains without developmental regression or clinical signs of disease activity was observed. Upon initiation of RXL, the initial IFN-signature score of 1272 (normal value <12) decreased to a minimum of 20. However, it once relapsed to 1273 (without a clinical correlate), prompting an increase in RXL dose to 30 mg/m^2^, leading to a decrease of the IFN-signature score to 20. The patient received steroids only once for three days during a febrile episode, as then no infection could be diagnosed. At the last follow-up (FU), at the age of 2 years, he presented in clinically excellent condition with only mildly impaired development, but he could speak about 15 single words, sit freely, crawl, and stand with assistance. Brain MRI showed neither new hyperintensities nor calcifications. Cerebrospinal fluid (CSF) analysis demonstrated no abnormalities, including neurofilament light chains below the detection limit. Laboratory findings at last FU showed mildly increased liver enzymes, CK-MB, and thrombocytes and a mild leukopenia and anemia, possibly attributed to RXL treatment.

**Conclusion:** Early initiation of JAKi with RXL in a severely affected infant with AGS7 was associated with an clinically mitigated disease course with no signs of active neuroinflammation and achievement of developmental milestones without loss of previously gained skills. Strikingly, CSF analysis, including neurofilament light chains, was normal under continuous RXL treatment. However, despite very early JAKi, an overall delay in motor and verbal development was observed. This raises the question of whether even earlier initiation of JAKi (e.g., via identification of patients via newborn screening) could have resulted in a better functional outcome.

**Patient Consent**

Yes, I received consent

**Disclosure of Interest**

None declared

## P102 Safety of antirheumatic drugs in patients with Juvenile Idiopathic Arthritis (JIA)

### I. Rukavina, M. Sestan, M. Held, D. Grguric, M. Frkovic, M. Jelusic

#### Division of Paediatric Immunology, Rheumatology and Allergology, Department of Paediatrics, University Hospital Centre Zagreb, University of Zagreb School of Medicine, Zagreb, Croatia

##### **Correspondence:** I. Rukavina

*Pediatric Rheumatology 2023*, **21(Suppl 2):**P102

**Introduction:** Juvenile idiopathic arthritis (JIA) is the most common chronic rheumatologic disease in children and it is characterized by arthritis of unknown origin. During treatment with antirheumatic drugs, numerous adverse events have been reported.

**Objectives:** To determine and compare the incidence of adverse events (AEs) during treatment of JIA with synthetic and biologic disease modifying antirheumatic drugs (s/b DMARD).

**Methods:** We retrospectively reviewed the medical data of children aged 1-18 years who were diagnosed with JIA and had received synthetic DMARDs (methotrexate, sulfasalazine, leflunomide) and biologics (adalimumab, etanercept, infliximab, tocilizumab, anakinra, abatacept, golimumab, secucinumab or tofacitinib) between January 2014 and December 2022 in Pediatric Clinic at University Hospital Centre Zagreb. Patients who were followed up regularly for at least 6 months were included.

**Results:** 359 patients had received sDMARDs and bDMARDs were given to 152 patients. The highest number of AEs was in the sDMARD group (55 patients, 15.3%): 36 patients who received methotrexate did not tolerate the drug due to nausea and vomiting (10%) and 12 of them had developed transitory elevated liver enzymes (3.3%). Sulfasalazine was ceased in 6 patients (1.67%): at 2 patients due to allergic rash, in 2 due to excessive hair loss and in 2 of them due to stomach pain and vomiting. There is also one patient that had received leflunomide who developed chest pain and fatigue. In bDMARD group there was less side effects but serious AEs were reported in 4 patients (1.1%) (adalimumab - miliary tuberculosis (1) and elbow septic arthritis (1), tocilizumab - chronic disseminated intravascular coagulation (1), infliximab - one patient developed anaphylactic reaction). Skin allergic reaction was noticed among biologics as well - anakinra (2), etanercept (1), adalimumab (1) and 2 patients developed psoriasis like rash (adalimumab). One patient in this group experienced headache while he had received adalimumab.

**Conclusion:** Immunosuppressive therapy for JIA is generally well tolerated and not toxic. AEs are more often in sDMARD group while in bDMARD group are more serious. Since side effects, particularly serious one, can occur, caution and regular check-ups are needed.

**Patient Consent**

Yes, I received consent

**Disclosure of Interest**

None declared

## P103 Interleukin-1 inhibition in Cryopyrin-Associated Periodic Syndromes (CAPS) – a real-life personalized treatment approach

### Ö. Satirer^1^, B. Zapf ^1^, P. Wasiliew^1^, J. Kümmerle-Deschner^1^, T. Welzel ^2^

#### ^1^Rheumatology, Department of Pediatrics , University Hospital Tuebingen, Tübingen, Germany; ^2^Pediatric Rheumatology, University of Basel, Basel, Switzerland

##### **Correspondence:** Ö. Satirer

*Pediatric Rheumatology 2023*, **21(Suppl 2):**P103

**Introduction:** Cryopyrin-associated periodic syndromes (CAPS) are a spectrum of phenotypes mostly caused by autosomal dominant variants in the *NLRP3*-gene (1). Treatment focuses on IL-1 inhibition to achieve no/minimal disease activity (DA) to prevent/reduce hearing loss (HL), organ damage, morbidity and mortality. Therefore, regular DA monitoring with DA-targeted treatment adjustments (treat-to-target: T2T) is recommended over the disease course (2,3). However, longitudinal data on T2T in CAPS is limited.

**Objectives:** To analyze treatment adjustment over the disease course in patients with CAPS.

**Methods:** A single-center longitudinal study in patients with ≥3 visits who met the CAPS classification (4) or diagnostic (5) criteria treated with IL-1 inhibitor ≥1 year between January 2007 and December 2022 was performed. Demographic characteristics, geno- and phenotype, c-reactive protein (CRP), serum Amyloid A (*SAA)*, DA, organ damage and treatment adjustments were recorded.

**Results:** 54 patients, 50% female, were included. Pathogenic/likely pathogenic variants were identified in 57%, mostly A439V and E311Kheterozygous. Variants of unknown significance (VUS) were detected in 33%, mostly Q703K and V198M heterozygous. 8% of patients met clinical criteria only. The median age at the first study visit was 13 years (IQR 5;42). At the first visit, HL was detected in 31% (n=17) of patients, with 23.5% (n=4) having pathogenic and 47.1% (n=8) likely pathogenic variants. The median follow-up was 759 visits and 103 months (IQR 63;145). During follow-up, 65% of patients had treatment adjustments. Of these, 46% received an increased dose/interval due to increased DA. Despite treatment and normal CRP/SAA at visits, HL worsened in 5 patients, necessitating dose increase with subsequent recovery of HL in 2/5 patients. Of those, receiving treatment increase , 58% had pathogenic, 41% had likely pathogenic, and 33% had VUS. Treatment reduction (dose/frequency) due to clinical long-term remission was possible in 10 patients (18%; 1x pathogenic, 4x likely pathogenic, 5x VUS). There was a significant correlation between treatment increase and PGA/PPGA at the initial visit, the visit at which the dose and/or frequency was increased, and the final visit (p<0.01, paired samples t-test). No significant correlation was found for CRP/SAA level changes.

**Conclusion:** DA can increase over the disease course in CAPS irrespective of treatment with risk of organ damage. Regular DA targeted treatment adjustments (T2T) can prevent under/-overtreatment due to guided treatment increase or tapering. During treatment particularly, the clinical DA monitoring by using the PGA, PPGA, and validated instruments (autoinflammatory disease activity index, autoinflammatory disease damage index ) seem to be sensitive instruments to guide personalized treatment in CAPS.

**Patient Consent**

Not applicable (there are no patient data)

**Disclosure of Interest**

None declared

**Reference**s

1. Kone-Paut and Galeotti, 2015
2. Romano et al. 2021
3. Hansmann, Lainka et al., 2020
4. Gattorno et al. 2019
5. Kuemmerle-Deschner et al. 2017

## P104 Clinical characteristics, family history and treatment response in girl with card14 MET119THR mutation

### V. Selmanovic^1^, A. Cengic^1^, I. Aksentijevich^2^, A. Kapetanovic^3^

#### ^1^Allergology, Rheumatology and Clinical Immunology, Children's Hospital University Clinical Center Sarajevo, Sarajevo, Bosnia and Herzegovina; ^2^2Inflammatory Disease Section/Clinical Genetics Service, IDS/CGS, NIH, National Human Genome Research Institute (NHGRI), USA, Bethesda, United States; ^3^Department for Dermatopatology, University Clinical Center Sarajevo, Sarajevo, Bosnia and Herzegovina

##### **Correspondence:** V. Selmanovic

*Pediatric Rheumatology 2023*, **21(Suppl 2):**P104

**Introduction:** Caspase recruitment domain family member 14 gene (CARD14) mutations have been associated with papulosquamous eruption including psoriasis and pityriasis rubra pilaris. Caracteristic features of subjects with CARD14 mutation include early onset, prominent facial involvement, minimal or absent response to conventional therapy including corticosteroids, methotrexate, adalimumab. CARD14 Met119Thr mutation was not linked with specific treatment so far. We describe excellent treatment response to secukinumab.

**Objectives:** to describe the clinical caracteristics, family history and treatment response in 9y old girl with CARD14 gene mutation

**Methods:** case report

**Results:** girl 8,5y; disease onset was at age 1 month with erythodermia evolving to papulosquamous eruption. First child of nonconsanguinous parents. Father was previously diagnosed as ichtiosis congenita, mother as psoriasis vulgaris. Child skin biopsy was not diagnostic, differential included ichtiosis lamelaris and psoriasis. Topical treatement had no major improvement. Girl’s clinical presentation included : generalized papulosquamous eruption; palmoplantar keratoderma; thick, abnormally shaped, yellow, dystrophic nails; skin photosensitivity, an increased sensitivity to heat, polyarthritis (developed at age 2y10mo, accompanied with fever and high inflammatory markers); ectropion; enlarged tonsilles, reccurent respiratory infections. No major skin response to systemic steroids, MTX, adalimumab but successfully controled joint disease. Father has milders clinical presentation : erythodermia with papulosquamous eruption, involved face, cheeks, ears, islands of follicular papules on the trunk and extremities; palmoplantar keratoderma, dystrophic nails, ectropion. Family genetic analysis revealed that girl and her father has same mutation of CARD14 Met119Thr. Mother has heterozygous variant c.676-6G>A (30% of population are carriers). Secukinumab was introduced in February 2023 and had excellent treatment response: no ectropion, generalized improvement of skin disease, significant improvement of quality of life. Same therapy is planned for the father.

**Conclusion:** We described CARD14 Met119Thr mutation in father and child that was not reported in none of the database of human variants, but was found in two patients with psoriasis, so is likely pathogenic. Subjects with suggestive clinical features should undergo genetic testing, given the high probability of response to secukinumab if CARD14 mutation is present.

**Patient Consent**

Yes, I received consent

**Disclosure of Interest**

None declared

**References**

1. Israel L and Mellett M (2018) Clinical and Genetic Heterogeneity of CARD14 Mutations in Psoriatic Skin Disease. Front. Immunol. 9:2239. doi: 10.3389/fimmu.2018.02239
2. Craiglow et al. CARD14-associated papulosquamous eruption: A spectrum including features of psoriasis and pityriasis rubra pilaris. J AM ACAD DERMATOL, SEPTEMBER 2018

## P105 Toxic epidermal necrolysis as a complication to the treatment of sjia – a case report

### M. J. Søndergaard, H. A. S. Ingels, C. Myrup

#### Pediatric rheumatology, Rigshospitalet, Copenhagen, Denmark

##### **Correspondence:** M. J. Søndergaard

*Pediatric Rheumatology 2023*, **21(Suppl 2):**P105

**Introduction:** We present a case of severe toxic epidermal necrolysis (TEN) following treatment for systemic juvenile idiopathic arthritis (sJIA) in an eight-year-old girl (1). Naproxen was suspected as the triggering medication.

**Objectives:** To present this case of a rare cutaneous adverse drug reaction (cADR) to a commonly used treatment in pediatric rheumatology. A review of the literature does not reveal a similar case.

**Methods:** The data was collected retrospectively from the electronic patient record.

**Results:** An eight-year-old girl, previously healthy, was admitted to a pediatric ward with five days of intermittent fever peaking late evening at 39°C, and a transient erythematous rash. The patient was unwilling to walk while febrile. Blood-samples showed: CRP 250 mg/l, WBC 43-25x10^9^/l, Ferritin 4000 mg/l. The patient was treated with standard IV antibiotics for a total of seven days. Microbiological analyses were all negative. On day nine, after onset of fever, she was transferred to a tertiary center for further evaluation. Due to suspicion of sJIA, naproxen (10 mg/kg twice daily) was initiated. Reaching 14 days of fever and a rising Ferritin (5200 mg/l) treatment with pulse-steroids (15 mg/kg/day for 3 days) was initiated with a good clinical and paraclinical response and the patient was discharged on tapering doses of Prednisone. On day 27 (after onset of fever) the rash reappeared, a flair in sJIA was suspected and she was started onsubcutaneous tocilizumab (RoActemra, 162 mg). The rash continued to evolve, without fever, into a standing, more elevated, darker red and itchy rash. After five days the patient was readmitted with a fulminant eruption with bullae. The skin, starting on the back and nates, was rolling off in large areas, provoked by only light handling. There was no mucosal involvement. A skin biopsy confirmed the diagnosis of TEN. Naproxen and tocilizumab were discontinued when a cADR was considered. The patient was treated in the intensive-care unit for 17 days with open exposure, antibiotics and oral Prednisone. The body surface affected was estimated to be more than 50%. The patient was discharged after 31 days in hospital, with only a minimal Prednisone dose of 5 mg. Interestingly, at the clinical examination 4 months after disease onset, the patient was still in remission without any form of treatment.

The continued use of Naproxen, from day 10 to day 33, in conjunction with the corticosteroids was considered the most likely drug to cause the reaction. Other drugs considered were esomeprazole, which were given prophylactic during the same period as naproxen and tocilizumab, but it was started after the cADR erupted making it unlikely to be the causal factor.

**Conclusion:** TEN is a rare but serious complication. In this case we suspect it to be a side-effect to Naproxen, a drug commonly used in pediatric rheumatology.

**Patient Consent**

Yes, I received consent

**Disclosure of Interest**

None declared

**Reference**

1. Ramien M, Goldman JL. Pediatric SJS-TEN: Where are we now? F1000Research. 2020 Aug 13;9:982.

## P106 Adalimumab exposure with and without methotrexate in pediatric inflammatory rheumatic diseases: a pilot-study

### T. Welzel^1,2,3^, K. Gohlen^2^, A. Atkinson^2,3^, V. Gotta^2^, J. B. Kuemmerle-Deschner^4^, C. Michler^4^, G. Koch^2^, J. N. van den Anker^2^, M. Pfister^2^, A. Woerner^1^

#### ^1^Pediatric Rheumatology; ^2^Pediatric Pharmacology; ^3^Pediatric Research Center, University Children’s Hospital Basel (UKBB), University of Basel, Basel, Switzerland; ^4^Division of Pediatric Rheumatology, Department of Pediatrics and Autoinflammation Reference Center Tuebingen (ARCT), University Hospital Tuebingen, Tuebingen, Germany

##### **Correspondence:** T. Welzel

*Pediatric Rheumatology 2023*, **21(Suppl 2):**P106

**Introduction:** Adalimumab is commonly used in pediatric inflammatory rheumatic diseases (PiRD). Up to now, adalimumab is dosed in fixed weight-based bands, irrespective of co-medication/potential factors influencing adalimumab pharmacokinetics (PK).

**Objectives:** To analyze adalimumab concentrations in PiRD patients with and without methotrexate to better understand adalimumab PK.

**Methods:** This is a two-center prospective trial in PiRD patients aged 2 to18 years treated with adalimumab and methotrexate (group A-M) or adalimumab alone (group A) for ≥12 weeks. Adalimumab concentrations were measured at 1 to 9 (C_max_) and 10 to 14 (C_min_) days post-dose in patients with stable adalimumab dosing regimen during 3 to 6 months. Furthermore, adalimumab concentrations were collected in treatment naïve PiRD patients 3 to 7 and 10 to 14 days after their first adalimumab dose. Concentrations were analyzed with enzyme-linked immunosorbent assay (lower limit of quantification: 0.5 mg/L). Analyses comparing C_min_ levels in group A-M and group A (t-test) and pharmacometric (PMX) pharmacokinetic analysis utilizing MONOLIX (V.2020 R1) for quantification of a potential co-medication effect of methotrexate on adalimumab apparent clearance (CL/F) were performed.

**Results:** A total of 28 patients (N=14 each group) with a median age of 11.3 years (IQR 8.92-13.2) completed the study. Of these, 20 (71%) had juvenile idiopathic arthritis, 7 (25%) non-infectious uveitis and 1 (4%) chronic recurrent multifocal osteomyelitis. Group A-M consisted of more girls (71.4%) and children were diagnosed younger (6.3 years [IQR 2.4, 9.0]) compared to group A (35.7% girls, 8.8 years [IQR 5.7, 10.1]). At baseline, groups A-M and A has been treated with adalimumab during 17.8 months [9.6, 21.6] and 15.8 months [8.5, 30.8], respectively. Mean adalimumab C_min_ was slightly but non-statistically significant higher in group A-M (13.5±7.8) compared to group A (10.4±6.0, p=0.2). Observed C_min_ values showed a high inter-subject heterogenecity (range: 0.5 to 26 mg/L). In the PMX analysis, all available 72 adalimumab concentration were included. The co-medication with methotrexate was associated in this study with a slight decrease (15%) in adalimumab CL/F (p=0.5).

**Conclusion:** Given available data, in this study a small statistically non-significant effect of methotrexate co-medication on adalimumab exposure was detected. Further PK investigation is warranted to better understand high inter-subject heterogenecity. The high inter-subject heterogenecity and reported adalimumab exposure changes over time [1] indicate a benefit of personalized dosing by model based simulations in children with PRD.

TW and KG contributed equally and should be considered as co-first authors. MP and AW contributed equally should be considered as co-last authors

**Trial registration identifying number:** Ethical approval was obtained (EKNZ, 2019-0091, Eberhard Karls University Tuebingen (321/2019B01). NCT04042792.

**Patient Consent**

Not applicable (there are no patient data)

**Disclosure of Interest**

None declared

**Reference**

1. Marquez et al. Pharmaceutics 2021

## P107 Comparison of the efficacy, safety and immunogenicity of a proposed biosimilar MSB11456 with tocilizumab reference product in moderate-to-severe rheumatoid arthritis: results of a randomized double-blind study (APTURA I)

### A. Zubrzycka-Sienkiewicz^1^, M. Misterska-Skora^2^, M. Socik Pojawa^3^, K. Klama^4^, M. Ullmann^5^, C. Petit-Frere^5^, A. Illes^5^, P. Baker^5^, J. Monnet^5^, J. Brzezicki^6^ on behalf of APTURA I Working Group

#### ^1^"Reumatika - Centrum Reumatologil", NZOZ, Warsaw, ^2^Centrum Medyczne Oporów, 4a/1Ulica Solskiego, Wrocław, ^3^MICS Centrum Medyczne Warszawa, 53 Ulica Wronia lokal B10, Warsaw, ^4^Solumed Centrum Medyczne, 77a Ulica J.H. Dąbrowskiego, Poznań, Poland, ^5^Fresenius Kabi SwissBioSim, Biosimilars, Eysins, Switzerland, ^6^Centrum Kliniczno Badawcze, 35-36/A Ulica Studzienna, Elbląg, Poland

##### **Correspondence:** A. Zubrzycka-Sienkiewicz

*Pediatric Rheumatology 2023*, **21(Suppl 2):**P107

**Introduction:** MSB11456, a proposed biosimilar to US-licensed and EU-approved tocilizumab has shown equivalent pharmacokinetic (PK), pharmacodynamic (PD), safety, tolerability, and immunogenicity profiles when given as a single subcutaneous (SC) dose in healthy volunteers.

**Objectives:** This multicenter, randomized, double-blind, multiple fixed-dose, parallel group study compared the efficacy, safety, and immunogenicity of MSB11456 and EU-approved tocilizumab for the treatment of adults with moderate-to-severe RA (NCT04512001).

**Methods:** Patients were randomized to weekly SC 162mg injections of either MSB11456 (n=302) or EU-approved tocilizumab (n=302) for 24 weeks (W). At W24, patients in the EU-approved tocilizumab group were re-randomized to continued treatment or switched to MSB11456 up to W52. Patients receiving MSB11456 continued treatment until W52. A safety evaluation was conducted up to W63. At W24, Disease Activity Score-28 Joint Count (DAS28)-erythrocyte sedimentation rate (ESR) change from baseline (primary endpoint) was analyzed to determine the least squares mean (LSM) difference between MSB11456 and EU-approved tocilizumab; MSB11456 was considered equivalent to EU-approved tocilizumab if the 95% confidence interval (CI) was within the equivalence interval -0.6–0.6. Secondary endpoints were 20% improvement in American College of Rheumatology core set measures (ACR20) at W24 and DAS28-ESR at W12. Additional endpoints included ACR50/70 (50% and 70% improvement), change in DAS28-C-reactive protein, Simplified Disease Activity Index, Clinical Disease Activity Index, evaluation of immunogenicity at various time points up to W55 and safety up to W63.

**Results:** At W24, the LSM difference in the change from baseline in DAS28-ESR between treatments was 0.01 (95% CI -0.19, 0.22). The 95% CI for the LSM difference in the change from baseline in DAS28-ESR between treatments was fully included within the predefined equivalence interval, indicating therapeutic equivalence of MSB11456 and EU-approved tocilizumab. Equivalence was further supported with the analyses of the other efficacy endpoints. Treatment-emergent adverse events (TEAEs) were usually mild or moderate and occurred at similar frequency in both treatment groups. There were no discernible patterns in terms of the nature, frequency, or other characteristics of serious or treatment related TEAEs to suggest a difference between treatments. Immunogenicity results were similar in both treatment groups. The switch from EU-approved tocilizumab to MSB11456 at W24 had no clinically relevant impact on efficacy or safety, including immunogenicity.

**Conclusion:** Equivalent efficacy and similar immunogenicity and safety profiles of MSB11456 and EU-approved tocilizumab were demonstrated in patients with moderate-to-severe RA. PK, immunogenicity, and safety of EU-approved tocilizumab was found to be similar across the approved indications, including pediatrics. Thus, the present data on MSB11456 are relevant for pediatric patients.

**Trial registration identifying number:** NCT04512001

**Patient Consent**

Not applicable (there are no patient data)

**Disclosure of Interest**

A. Zubrzycka-Sienkiewicz: None declared, M. Misterska-Skora: None declared, M. Socik Pojawa: None declared, K. Klama: None declared, M. Ullmann Employee with: Fresenius Kabi SwissBioSim, C. Petit-Frere Employee with: Fresenius Kabi SwissBioSim, A. Illes Employee with: Fresenius Kabi SwissBioSim, P. Baker Employee with: Fresenius Kabi SwissBioSim, J. Monnet Employee with: Fresenius Kabi SwissBioSim, J. Brzezicki: None declared

## P108 Juvenile-Onset Systemic Lupus Erythematosus (JSLE): a study at a tertiary medical center in Abu Dhabi

### A. Y. M. Abuhamad^1^, Ikram Qaisar Shaalan^2^, Shima Kamal Eldin Yasin (skyasin@ssmc.ae)^2^, A. Almarzooqi^1^, K. W. Khawaja^3^

#### ^1^Paediatrics Rheumatology; ^2^Rheumatology Department Sheikh Shakhbout Medical City, Abu Dhabi, UAE; ^3^Chair of Paediatrics Rheumatology Department, Shaikh Shakhbout Medical City(SSMC) in partnership with Mayo Clinic, Abu Dhabi, United Arab Emirates

##### **Correspondence:** Ikram Qaisar Shaalan (iqshaalan@seha.ae)

*Pediatric Rheumatology 2023*, **21(Suppl 2):**P108

**Introduction:** There are significant variations in the way lupus presents in children[1][2]. There is no data from the United Arab Emirates.

**Objectives:** Review our experience with jSLE over the last eight years by looking at the demographics, clinical features, blood parameters, treatments and outcome.

**Methods:** Retrospective chart review of electronic medical record in our tertiary centre in Abu Dhabi from January 2015 to January 2023.

**Results:** 57 patients in total with the diagnosis of JSLE. 51 female (89.5%) and 6 male individuals (10.5%) with female to male ratio of 9:1. Mean age at disease onset was 10.2 years (range: 0.5 - 16 years). The average age at diagnosis was 10.5 years (range: 0.5 - 16 years).

The most frequent presenting symptoms were skin rash and arthralgia 36 out of 57 patients (63%). Arthritis in 10 out of 57 patients (18%). Raised urine protein: creatinine ratio in 20 out of 57 patients (35 %). 17 out of 57 patients (29.8%) were diagnosed with lupus nephritis, and renal biopsy done in 15 out of 17 patients (88%).

Among those who underwent biopsy, 10 out of 15 patients (67%) had grade IV-V renal disease, while 3 out of 15 patients (20%) had grade III renal disease.

Anemia was the most frequent hematologic abnormality at onset, affecting 45% of patients, Lymphopenia in 13 out of 57 patients (22.8%). Thrombocytopenia in 11 out of 57 cases (19%). All 57 patients in the study had positive ANA result. Anti-ds DNA level was high in 52/57 patients (91.2%).

All patients in the study received hydroxychloroquine. 24.5% (14/57) received Methotrexate, 44% (25/57) received Mycophenolate, and 35% (20/57) received Azathioprine. Steroids were used as the primary therapeutic approach in most cases (94.5%; 54/57). In severe disease Rituximab (17.5%; 10/57), cyclophosphamide (19%; 11/57), and IVIG (23%; 13/57) were used.

Maximum total pBILAG 2004 at last follow up of 13 (range 8-20). Maximum SLEDAI score of 13 (8-20).

**Conclusion:** The commonest features at presentation were rash and arthralgia (63%) followed by anemia (45%) then lupus nephritis (29%) then arthritis (18%). All ANA positive at presentation. DsDNA positive in 91%.

For treatment methotrexate in 24.5%, Mycophenolate in 44%, Azathioprine in 35%. Rituximab 17.5% and cyclophosphamide in 19%. Maximum total pBILAG at last follow up of 13(8-20) and maximum SLEDAI of 13 (8-20). More ethnicity focused studies needed for more targeted treatment and better outcome.

**Patient Consent**

Not applicable (there are no patient data)

**Disclosure of Interest**

None declared

**References**

1. Hiraki LT, Benseler SM, Tyrrell PN, Hebert D, Harvey E, Silverman ED. Clinical and Laboratory Characteristics and Long-Term Outcome of Pediatric Systemic Lupus Erythematosus: A Longitudinal Study. The Journal of Pediatrics. 2008b Apr;152(4):550–6.
2. Smith EMD, Lythgoe H, Midgley A, Beresford MW, Hedrich CM. Juvenile-onset systemic lupus erythematosus: Update on clinical presentation, pathophysiology and treatment options. Clinical Immunology. 2019 Dec;209:108274.

## P109 Neuropsychiatric manifestations and lupus nephritis in a juvenile libyan male systemic lupus erythematosus: as lupus challenge

### A. A. Abushhaiwia^1^, H. A. Ahmed^2^, J. R. Mohammed^2^

#### ^1^Pediatric Rheumatology , university of Tripoli , Faculty of Medicine , Tripoli Children's Hospital; ^2^General Pediatric , Tripoli Children's Hospital , Tripoli , Libya

##### **Correspondence:** A. A. Abushhaiwia

*Pediatric Rheumatology 2023*, **21(Suppl 2):**P109

**Introduction:** Neuropsychiatric (NP) lupus and lupus nephritis are one of the most profound manifestations of the Systemic lupus erythematosus (SLE), with wide variety ofclinical manifestations, and the most difficult therapeutic problem. Here we present acase of JSLE, with major organ involvement including neuropsychiatric systemiclupus erythematosus (NPSLE) and lupus nephritis (LN).

**Objectives:** to illustrate the diagnostic and therapeutic challenges in juvenile neuropsychiatric and renal lupus

**Methods:** A case report

**Results:** A 12 years old boy without relevant medical or medication history with an unremarkable family. a 2-month history of fevers, arthralgia, malar rash,alopecia, poor appetite and malaise. Over the course of 2 weeks, he had a history of severe headache, twice vomiting, cognitive dysfunction and psychiatric symptoms,such as tinnitus; Poor sleeping mood and aggressive behaviou.he never had seizures.Mouth ulcer,nasal ulcer. On clinical examination, he had fever reached 41c, no meningeal sign, power, tone, and reflexes were normal. But was unable to walk due to restricted movement of knees and ankles as well as both elbows. Laboratory studies showed anaemia with haemoglobin10.4 g/l; low leukocyte count 2.2 X10 ^3^ ; normal PLT 378X10 ^3^ , a raised (ESR) of 127 mm/hr, and (CRP);9mg\dl , a brain MRI showed multiple deep white matter small foci of signal alteration (related to vasculitis), MRI spine was normal, MRI brain angio was normal cerebral vessels. No pathology was detected in the abdominal U\S. Immunological investigation showed both (ANA) 1:5120 and (anti-ds DNA) antibodies 1;800 iu\ml were positive with high titter, anti-SSB (La), anti-SSA (Ro) was positive with high titter 1;200 IU\ml, complement levels. Rheumatoid factor, anti RNP abs, anti-Smith (Sm), anti-cardiolipin and anti-b2 glycoprotein antibodie, were all negative. Raised urine Pr: cr ratio and microscopic heamaturia (urine RBCs up to 70-73 Showing 92% dysmorphism i.e. RBC cast) . Despite the above mentioned, his blood pressure and renal function test, are always normal, renal ultrasound was normal. Eyes examination were normal no optic neuritis.To avoid cyclophosphamide-related gonad toxicity. He was treated with methypredinsolone infusion 1gm once per day for 5 days then followed by oral steroid 1.5mg\kg\day, 2gm IVIG once, enalopril 5mg once per day &MMF 1g twice per day.Consequently his neuropsychiatric symptoms and MUSK, in addition skin and ulcers symptoms subsided, but still Persistence of microscopic heamaturia and raised urine pr: cr ratio, renal biopsy was performed &showed Diffuse proliferative glomerulonephritis IV, after the second month of treatment brought to complete remission of nephrotic syndrome and nephritis.

**Conclusion:** Our clinical case suggests that the IVIG, pulse steroid and MMF combination therapeutic scheme appears to be safe and successful in achieving a clinically significant response.

**Patient Consent**

Yes, I received consent

**Disclosure of Interest**

None declared

## P110 Adolescent lupus with bilateral jugular vein thrombosis and rowell syndrome : a case report

### D. Alwakeel, M. Al Marri

#### Pediatric Rheumatology , Prince sultan military medical city , Riyadh , Saudi Arabia

##### **Correspondence:** D. Alwakeel

*Pediatric Rheumatology 2023*, **21(Suppl 2):**P110

**Introduction:** SLE is a chronic autoimmune disease ,and may result in significant morbidity and mortality.antiphospholipid antibodies are found in 30-40% of patients with SLE and are associated with hypercoagulability.There is a relatively low incidence of isolated internal jugular vein thrombosis.Only four cases of IJV thrombosis as a main manifestation of APS have previously been documented in the literature.Rowell syndrome (RS) is an uncommon condition that causes EM-like lesions in people with LE.The majority of cases have been documented in middle-aged women.Up to our extensive research, this is the first case of an adolescent patient who presented with SLE and APS that was complicated by bilateral internal jugular vein thrombosis and Rowel syndrome.

**Objectives:** Case presentation:

13 years old girl,presented with left leg DVT,Her labs showed leuckopenia,thrombocytopenia,hemolytic anemia,prolonged aPTT, positive autoantibodies,and low complement.Patient was started on LMWH, then shifted to direct oral anticoagulant and hydroxychloroquine.But she was readmitted with bilateral internal jugular vein thrombosis.aPLA were resulted as triple positive,and her condition was worsening.she underwent thrombectomy and Rituximab was added .then she developed itchy skin rash.Skin biopsy confirmed the diagnosis of Rowell syndrome.SLE treatment was optimized, and during follow up she improved clinically and radiologicaly.

**Conclusion:** Direct oral anticoagulant s adding another risk of thrombosis for SLE patients with triple positivity of antiphospholipid antibodies, and caution should be taken when treating this group of patients.Furthermore, Rowell syndrome is uncommon, and a positive anti-SSA test is one of the minor criteria for its diagnosis; therefore, physicians should be aware of these rare manifestation.

**Patient Consent**

Yes, I received consent

**Disclosure of Interest**

None declared

**References**

1. Levy DM, Kamphuis S. Systemic lupus erythematosus in children and adolescents. Pediatr Clin North Am. 2012 Apr;59(2):345-64. doi: 10.1016/j.pcl.2012.03.007. PMID: 22560574; PMCID: PMC3348509.
2. Al-Zoubi NA. Spontaneous internal jugular vein thrombosis as primary presentation of antiphospholipid syndrome: case report. Vasc Health Risk Manag. 2018 Jul 16;14:153-155. doi: 10.2147/VHRM.S170140. PMID: 30038499; PMCID: PMC6052923.
3. Bhat RY, Varma C, Bhatt S, Balachandran C. Rowell syndrome. Indian Dermatol Online J. 2014 Nov;5(Suppl 1):S33-5. doi: 10.4103/2229-5178.144526. PMID: 25506561; PMCID: PMC4252948.
4. Pengo V, Denas G, Zoppellaro G, Jose SP, Hoxha A, Ruffatti A, Andreoli L, Tincani A, Cenci C, Prisco D, Fierro T, Gresele P, Cafolla A, De Micheli V, Ghirarduzzi A, Tosetto A, Falanga A, Martinelli I, Testa S, Barcellona D, Gerosa M, Banzato A. Rivaroxaban vs warfarin in high-risk patients with antiphospholipid syndrome. Blood. 2018 Sep 27;132(13):1365-1371. doi: 10.1182/blood-2018-04-848333. Epub 2018 Jul 12. PMID: 30002145

## P111 Mucocutaneous manifestations in systemic lupus erythematosus with juvenile onset: single center retrospective study

### M. Kaleda, A. Arefieva, I. Nikishina, T. Pachkoria, A. Shapovalenko, S. Salugina

#### Pediatric, V. A. Nasonova Research Institute of Rheumatology, Moscow, Russian Federation

##### **Correspondence:** A. Arefieva

*Pediatric Rheumatology 2023*, **21(Suppl 2):**P111

**Introduction:** Patients diagnosed with juvenile-onset systemic lupus erythematosus (jSLE) often have lesions of the skin and mucous as part of their presentation.

**Objectives:** to analyze mucocutaneous manifestations of jSLE.

**Methods:** We have assessed skin and mucous signs in 182 pts with jSLE who were observed in the pediatric department of our institute. Diagnosis of SLE was reviewed based on 2012 SLICC criteria. Their clinical and laboratory data were analyzed. The skin lesions were classified as specific (acute, subacute and chronic) or nonspecific LE-related, e.g. photosensitivity, urticaria, Raynaud's phenomenon, livedo reticularis or skin vasculitis.

**Results:** 149 pts (81.9%) had cutaneous signs, 127 girls/22 boys. Acute cutaneous LE lesions were in 129 pts (86.6% of all pts with skin involvement), including 99 pts (66.4%) with classic malar rash. 46 pts (30.9%) had subacute cutaneous LE. 2 pts (1.3%) had bullous skin eruptions. Chronic cutaneous LE included: chronic discoid lesions (DLE) in 8 pts (5.4%), lupus panniculitis – in 1 patient (0.7%), chilblain lupus erythematosus – in 2 pts (1.34%). 40 pts (26.8%) observed photosensitivity. Vascular phenomena were common: Raynaud's phenomenon occurred in 33 pts (22%), 56 (37.6%) had cutaneous vasculitis and 15 (10%) – livedo reticularis, chronic urticaria was noted by 3 pts (2%). 37 pts (24.8%) had a combined skin lesion. A non-scarring alopecia occurred in 54 pts (36.2%). 40 pts (26.8%) had oral or nasopharyngeal ulcers. 45 pts (30.2%) had erythema of the hard palate. Cheilitis was seen in 43 pts (28.9%). Pts with skin involvement were statistically significant more likely to have mucous involvement (p=0.05), leukopenia (p=0.006), thrombocytopenia (p=0.048). We didn’t observe a statistically significant difference in skin and mucous involvement between girls and boys. Median disease activity by SLEDAI at the time of jSLE verification was 13 [9;17] in pts with skin manifestations and 10 [7;13] in pts without skin manifestations.

**Conclusion:** The majority of pts with SLE have skin lesions, most often within the framework of an acute cutaneous LE lesions. The presence of skin manifestations correlated with mucous involvement, leukopenia, thrombocytopenia and higher activity of the disease by SLEDAI. There was no statistically significant difference in skin lesions in boys and girls, as well as no relationship between the type of skin lesions and involvement of other organs.

**Patient Consent**

Yes, I received consent

**Disclosure of Interest**

None declared

## P112 Simultaneous manifestation of systemic lupus erythematosus and type 1 diabetes in girl with mutation in gene RPP38: case report

### M. Kaleda^1^, A. Arefieva^1^, I. Nikishina^1^, T. Pachkoria^1^, G. Svetlova^2^, D. Sorokin^2^

#### ^1^Pediatric, V. A. Nasonova Research Institute of Rheumatology; ^2^Pediatric, Endocrinology Research Centre, Moscow, Russian Federation

##### **Correspondence:** A. Arefieva

*Pediatric Rheumatology 2023*, **21(Suppl 2):**P112

**Introduction:** Currently, there are a large number of studies that demonstrate common pathogenetic mechanisms and genetic associations in many autoimmune diseases, including systemic lupus erythematosus (SLE), Type 1 Diabetes (T1D) and autoimmune thyroiditis (AIT).

**Objectives:** to present a rare case of simultaneous manifestation of SLE and T1D in girl with mutation in gene RPP38.

**Methods:** Case report.

**Results:** An 8-yo girl fell ill in November 2018. On 1st admission in local hospital she had fever, lymphadenopathy, hepatosplenomegaly, hemorrhagic rash, Hb 70 g/l, WBC 2.27x10^9^/L, platelets 24×10^9^/L, fibrinogen 1.5 g/l, ALT 114 U/L, АSТ 697 U/L, ferritin 900 ng/ml, hyperglycemia 14.7 mmol/l. An infectious process, solid tumors and blood diseases was ruled out. 5 weeks after she developed arthritis, malar rash, diffuse alopecia, aphthous stomatitis. ANA 1/2560 h+cytopl, anti-dsDNA 10N, hypocomplementemia. Patient was diagnosed as SLE with Macrophage Activation Syndrome at onset and T1D. She was started insulin sc, GC iv 15 mg/kg №3, GC per os 0.8 mg/kg, intravenous immunoglobulin (IVIG) 0.5 g/kg, mycophenolate mofetil (MMF) 500 mg/day with good efficacy. After 2 months, when trying to reduce the dose of GC per os to 0.5 mg/kg, flare developed. She received repeat course of IVIG, GC iv, dose of GC was increase to 1.0 mg/kg. On 1^st^ admission in our clinic in March of 2019 she had general fatigue, hyperpigmentation after rash on the face and lower legs, enanthema, diffuse alopecia, Hb 104g/l, ANA 1/1280sp+cytopl, antiDNA - 1.5N, antiRNP 6N, antiSm 5N, direct Coombs test+, hyperglycemia 16.4 mmol/l. Instrumental modalities (US, sialography, sialometry) identified Sjogren's syndrome (SS). MMF was increased to 750 mg/day, hydroxychloroquine 200 mg/day was started. Inactive status of SLE was achieved after 6 months of therapy. In February 2022 patient was hospitalized to Endocrinology Research Centre. Based on laboratory findings of elevated antithyroid peroxidase (TPO) antibodies (687 U/ml (<5.6)) AIT was diagnosed. Thyroid-stimulating hormone (TSH) and free thyroxine (fT4) levels was normal so there was no need of treatment. Increased levels of GADa was found (1217U/ml) so T1D was confirmed. Due to low daily dose of insulin and HbA1c levels 6.3-7.0% during disease the whole exome sequencing was performed. In RPP38 (NM_183005.5) gene the heterozygous variant c.709G>T, p.Glu237X was found. In November 2022 clinical symptoms of SLE absent, ANA 1/320h+sp+cytopl, antiDNA - 1.5N. Now girl receives GC per os 2.5 mg/day, hydroxychloroquine 150 mg/day, MMF 750 mg/day, insulin (Glargine 300, Glulisine) 25-28 U/day (0.55 U/kg/day).

**Conclusion:** Our patient has multiple autoimmune syndrome, including SLE, SS, T1D, AIT with simultaneous occurrence of rheumatological and endocrine pathology, the significance of the identified mutation for the pathogenesis of which is still unclear.

**Patient Consent**

Yes, I received consent

**Disclosure of Interest**

None declared

## P113 Subclinical microvascular alterations in patients with juvenile systemic lupus erythematosus: the role of optical coherence tomography angiography

### G. B. Beretta^1^, C. Mapelli^2^, G. Leone^2^, M. Nassisi^2^, A. Mastrangelo^3^, M. Rossano^1^, S. Lanni^1^, A. Petaccia^1^, G. Filocamo^1^, F. Minoia^1^

#### ^1^Pediatric Immunorheumatology; ^2^Ophtalmology Unit, ^3^Pediatric Nephrology Dialysis and Transplant, Fondazione IRCCS Ca’ Granda Ospedale Maggiore Policlinico, Milan, Italy

##### **Correspondence:** G. B. Beretta

*Pediatric Rheumatology 2023*, **21(Suppl 2):**P113

**Introduction:** Retinal manifestations are a potentially sight-threatening complication of systemic lupus erythematosus (SLE), affecting 7-26% of adult patients. Optical coherence tomography angiography (OCTA) detected subclinical macular microvascular alterations in adult SLE without overt clinical manifestations. Evidence regarding retinal microvascular changes in juvenile SLE (jSLE) is still lacking.

**Objectives:** To evaluate subclinical alterations in retinal microvascularization through OCTA analysis in a monocentric cohort of jSLE patients without clinical ocular involvement.

**Methods:** Consecutive patients with juvenile SLE without clinical ophthalmic manifestations were enrolled at our Institution over a 1-year period. Both eyes from each patient were scanned with 3x3 mm OCTA acquisitions centered on the fovea. Superficial capillary plexus (SCP) and deep capillary plexus (DCP) angiograms were analyzed and perfusion density (PD) and vessel density (VD) were calculated. The foveal avascular zone (FAZ) was also measured. All data were compared with a cohort of age-matched healthy subjects using generalized estimating equation. Medical records were reviewed regarding clinical and laboratory parameters, histopathological data and treatment. The Systemic Lupus Erythematosus Disease Activity Index (SLEDAI) was used to assess disease activity.

**Results:** Both eyes of 12 patients (9 females) were evaluated so far. At OCTA, median age was 17.8 years and median disease duration was 4.6 years. All patients had lupus nephritis (LN), and 7 of them were classified as stage IV. Hematological, mucocutaneous and musculoskeletal symptoms were reported in 9, 5 and 5 patients, respectively. Median SLEDAI was 13 at disease onset and 19,5 at maximum disease activity. Eleven patients were treated with hydroxychloroquine (HCQ), but OCT scans didn’t reveal any alteration suggestive for toxic retinopathy. AT OCTA all patients were on low-dose corticosteroids (5-7.5 mg/day) while 8 patients were on mycophenolate mofetil. Patients with jSLE had significantly lower PD (30.13 ± 3.23 %) and VD (10.77 ± 1.48 mm^-1^) in the SCP compared to 16 age-matched heathy subjects (34.069 ± 2.22 %, p<0.01 and 13.7 ± 1 mm^-1^, p<0.01, respectively). No differences between jSLE and controls were detected in retinal thickness or volume, DCP parameters and in FAZ area. Patients with stage IV LN had a significantly larger FAZ area (0.28 mm^2^ vs 0.20 mm^2^, p=0.01) compared to patients with lower stage LN. Additionally, PD and VD in both SCP and DCP were slightly lower, but the differences were not statistically significant. No correlation was found between OCTA findings and disease duration, SLEDAI or autoantibody titers.

**Conclusion:** Patients with jSLE present retinal microvascular abnormalities, even without any clinical symptoms. OCTA is a non-invasive technique which may detect early systemic vascular involvement and prevent further visual deterioration. Further prospective studies on larger cohorts are needed to better understand the prognostic role of OCTA abnormalities and their correlation with clinical phenotype.

**Patient Consent**

Yes, I received consent

**Disclosure of Interest**

None declared

## P114 The use of childhood lldas: first results from a real-life longitudinal childhood lupus cohort show good feasibility but difficult attainment

### S. Bergkamp, T. Kanagasabapathy, M. Gruppen, T. Kuijpers, A. Nassar-Sheikh Rashid, J. M. van den Berg, D. Schonenberg-Meinema

#### Emma Childrens Hospital, Department of Pediatric Immunology, Rheumatology and Infectious Diseases, University of Amsterdam, Amsterdam University Medical Centers, Amsterdam, Netherlands

##### **Correspondence:** S. Bergkamp

*Pediatric Rheumatology 2023*, **21(Suppl 2):**P114

**Introduction:** For Treat-to-Target (T2T), the concept of Lupus-Low-Disease-Activity-State (LLDAS) as a specific target in systemic lupus erythematosus (SLE) has been defined (1). This adult LLDAS (aLLDAS) has been shown to be a feasible target in cSLE patients and attainment leads to lowering risk for severe flare and cumulative damage (2). Recently, an international cSLE T2T Task Force has adapted the LLDAS definition specifically for childhood-onset SLE (cSLE) patients (cLLDAS) (3).

**Objectives:** The first objective was to investigate if the time to reach first aLLDAS and cLLDAS differs in cSLE. The second objective was to observe if cSLE patients were able maintain cLLDAS for 50% of follow-up time (cLLDAS-50) in a real-life cohort.

**Methods:** Data from a prospective longitudinal cSLE cohort were used, with patient/parent consent. Patients were classified as cSLE by SLICC 2012 criteria with a disease onset < 18 years old. Definitions were: aLLDAS according to Franklyn (1), disease activity score by Systemic Lupus Erythematosus Activity Index -2K (SLEDAI-2K) and cLLDAS by cLSE T2T Task Force (3). cLLDAS-50 was defined as attainment of cLLDAS in more than 50% of follow-up. Binary regression analysis was used for testing predictors for (not) attaining cLLDAS-50 with significance for p<0.05.

**Results:** A total of 48 cSLE patients were studied, with a median age at diagnosis of 14 years, median diagnostic delay of 4.5 months and median follow-up of 51 months (range 14-163 months). Median SLEDAI at diagnosis was 12.5 (IQR 9-18). Mean number of visits was 11 per patient. Use of mycophenolate mofetil (MMF) was in 60.4%, azathioprine (AZA) in 39.6%, and use of hydroxychloroquine (HCQ) in 100% of patients. 41.7% of patients used a biologic (rituximab or belimumab) at any time point. Each patient reached aLLDAS and cLLDAS at least once during follow-up. Mean time to reach first aLLDAS/cLLDAS was 8.4 months and 9.3 months respectively. Only 58.3% (28/48) of patients were able to maintain cLLDAS-50. By univariate binary regression analysis, higher SLEDAI at diagnosis (OR 0.92, p=0.023), renal involvement (OR 0.12, p=0.002), ever use of biologics (OR 0.11, p=0.001) and compliance problems (OR 0.22, p=0.022) were negative predictors for attaining cLLDAS-50. Timing of starting biologic treatment was not taken into account. A longer time from diagnosis to first cLLDAS was also a negative predictor for attaining cLLDAS-50 (OR 0.85, p=0.005). By multivariate analysis, none of these variables was associated with (not) attaining cLLDAS-50.

**Conclusion:** In a longitudinal cSLE cohort, reaching cLLDAS occurred later than aLLDAS, emphasizing the need for an adapted LLDAS definition for cSLE. This study shows that reaching cLLDAS in cSLE is feasible, but attaining cLLDAS-50 is difficult.

**Patient Consent**

Yes, I received consent

**Disclosure of Interest**

None declared

**References**

1. Franklyn K, Lau CS, Navarra SV, et al. Definition and initial validation of a lupus low disease activity state (LLDAS). Ann Rheum Dis 2016;75:1615–21.
2. Smith et al. Attainment of low disease activity and remission targets reduces the risk of severe flare and new damage in childhood lupus. Rheumatology 2022;61: 3378-3389.
3. Smith et al. PReS-endorsed international childhood-onset lupus T2T task force definition of childhood lupus low disease activity state (cLLDAS). Clin Immunol. 2023 Mar 17;250:109296. doi: 10.1016/j.clim.2023.109296. Online ahead of print.

## P115 Worldwide evaluation of Clinical Practice Strategies (CLIPS) in childhood-onset lupus nephritis through the Juvenile Inflammatory Rheumatism (JIR)-clips network

### R. Carlomagno^1^, L. Lamot^2^, V. Natoli^3^, S. Sener^4^, J. Calzada-Hernandez^5^, J. Cognard^6^, D. Dey^7^, S. Ganhao^8^, N. Jordan^9^, B. Kasap Demir^10^, C. Lanna^11^, S. Marks^12^, A. Migowa^13^, A. Mubarak^14^, J. Pauvonic Pantic^15^, K. Pateras^16^, C. Pruunsild^17^, D. Schonenberg-Meinema^18^, P. Riley^19^, M. Verkaaik^14^, S. Sahin^20^, A. Belot^6^, E. M. Smith^3^, S. Kamphuis^14^ on behalf of the PReS Lupus Working Party and the JIR-CLiPS Network

#### ^1^Pediatric Rheumatology and Immunology Unit, Lausanne University Hospital, Lausanne, Switzerland; ^2^Department of Pediatrics, University of Zagreb School of Medicine, University Hospital Centre Zagreb, Zagreb, Croatia; ^3^Department of Pediatric Rheumatology, Alder Hey Children’s NHS Foundation Trust, University of Liverpool, Liverpool, United Kingdom; ^4^Department of Pediatric Rheumatology, Hacettepe University Faculty of Medicine, Ankara, Türkiye; ^5^Department of Rheumatology, Hospital Sant Joan De Deu, Barcelona, Spain; ^6^Pediatric Nephrology, Rheumatology, Dermatology Unit, Hospital of Mother and Child, Hospices Civils of Lyon, Lyon, France; ^7^Department of Medicine and Therapeutics, College of Health Sciences, University of Ghana School of Medicine and Dentistry, Accra, Ghana; ^8^Department of Rheumatology, Centro Hospitalar e Universitario de Sao Joao, Porto, Portugal; ^9^Department of Rheumatology, St James Hospital, Dublin, Ireland; ^10^Department of Pediatrics, Division of Nephrology and Rheumatology, İzmir Katip Çelebi University, Izmir, Türkiye; ^11^Department of Locomotor System, Faculdade de Medicina, Universidade Federal de Minas Gerais, Belo Horizonte, Brazil; ^12^Department of Pediatric Nephrology, Great Ormond Street NHS Foundation Trust Hospital, London, United Kingdom; ^13^Department of Paediatrics and Child Health, Aga Khan University Medical College East Africa, Nairobi, Kenya; ^14^Department of Pediatric Rheumatology, Erasmus University Medical Center, Rotterdam, Netherlands; ^15^Institute of Pathophysiology, University of Belgrade, Belgrade, Serbia; ^16^Laboratory of Epidemiology & Artificial Intelligence, Faculty of Public Health, University of Thessaly, Volos, Greece; ^17^Children’s Clinic, Tartu University Hospital, Tartu, Estonia; ^18^Department of Pediatric Rheumatology, Amsterdam UMC, Amsterdam, Netherlands; ^19^Department of Pediatric Rheumatology, Royal Manchester Children's Hospital, Manchester, United Kingdom; ^20^Department of Pediatric Rheumatology, Cerrahpasa Medical Faculty, Istanbul University-Cerrahpasa, Istanbul, Türkiye

##### **Correspondence:** R. Carlomagno

*Pediatric Rheumatology 2023*, **21(Suppl 2):**P115

**Introduction:** Childhood-onset systemic lupus erythematosus (cSLE) presents with renal disease as lupus nephritis (LN) in up to 60% of patients, occurring earlier and with increased severity than adult-onset SLE. This is a severe and potentially life-threatening disease, requiring early aggressive treatment with immunosuppressive medication. “European evidence-based SHARE recommendations for LN in cSLE”[1] support clinicians in the diagnosis and treatment of cSLE patients with LN. Country-specific access and reimbursement policies for specific disease management-strategies may limit the application of these recommendations in a real-life setting in many countries.

**Objectives:** We aimed to collect real-life clinical practice strategies (CliPS) for LN from physicians all over the world taking care of selected JIR diseases in order to produce appropriate, world practical disease management strategies.

**Methods:** We developed questionnaires to explore strategies used by physicians’ worldwide relating to the diagnosis, treatment and follow-up of cSLE patients with LN. Questionnaires were finalized in 2022, and since September 2022 distributed through channels as JIRcohort network, national rheumatology and nephrology societies and networks. We performed an interim analysis on the data extracted on January 27, 2023, focusing on induction treatment of LN.

**Results:** Ninety-seven physicians from 29 countries among Europe, South America, Africa, Oceania and Middle East responded to the LN questionnaire, 74 (76%) pediatric rheumatologists, 10 (10%) pediatric nephrologists, 2 (2%) pediatricians, and 11 (11%) adult physicians. Forty-four (45%) respondents had been caring for LN patients for ≥10 years, and 64 (66%) worked in university hospitals. Overall, the proportion of respondents selecting medications for LN were: prednisone (PDN) 88%, mycophenolate mofetil (MMF) 82%, cyclophosphamide (CYC) 75%, azathioprine 47%, cyclosporine (CSA) 31%, tacrolimus (TAC) 17%, rituximab (RTX) 39% and belimumab (BLM) 16%. Physicians predominantly treated class I LN with PDN (63%) and AZA (24%); class II LN with PDN (82%), AZA (36%) and MMF (21%). Comparable frequencies of PDN, MMF, TAC and BLM were used for class III (PDN 83%, MMF 69%, TAC 7%, BLM 10%) and IV (PDN 81%, MMF 71%, TAC 10%, BLM 12%) LN. AZA was preferred more in class III (18%) than IV (3%). CYC and RTX treatment were selected more frequently in class IV (CYC 72%, RTX 35%) than in class III (CYC 49%, RTX 21%). For class V, physicians preferred mostly PDN (80%) and MMF (72%); other drugs were CYC 41%, CSA 30%, RTX 26%, TAC 16%, and BLM 9%. Only 9% of physicians used voclosporin in class III, IV and V.

**Conclusion:** The majority of the respondents were pediatric rheumatologists and experienced physicians, working in university hospitals. Selected treatments were mostly based on recommendations, as the preferred medications for induction treatment in LN were PDN, MMF and CYC, particularly for class III, IV and V. Further analyses on treat-to-target and prednisone tapering schedules are ongoing.

**Patient Consent**

Not applicable (there are no patient data)

**Disclosure of Interest**

None declared

**Reference**

1. Groot et al., Ann Rheum Dis. 2017 Nov;76(11):1788-1796.

## P116 A life-threatening pulmonary complication in systemic lupus erythematosus: diffuse alveolar hemorrhage

### N. Öner, E. Çelikel, Z. Ekici Tekin, V. Güngörer, M. Sezer, N. Tekgöz, C. Karagöl, S. Coşkun, M. M. Kaplan, M. Polat, B. Çelikel Acar

#### Ankara Bilkent City Hospital, Ankara, Türkiye

##### **Correspondence:** Çelikel Acar

*Pediatric Rheumatology 2023*, **21(Suppl 2):**P116

**Introduction:** Systemic lupus erythematosus (SLE) is a connective tissue disease affecting many systems. Here, two patients with diffuse alveolar hemorrhage, a rare involvement of SLE, are reported.

**Results:** Case 1: A 9-year-old girl presented with malaise, fever and dyspnea. Physical examination revealed fever, bilateral diffuse rales and rhonchi. Laboratory findings included leukocyte count 5070/mm^3^, absolute lymphocyte count (ALC) 670/mm^3^, hemoglobin 11.3 g/dL, platelets 32,000/mm^3^, erythrocyte sedimentation rate (ESR) 68 mm/h, C-reactive protein (CRP) 20 mg/dL. Antinuclear antibody (ANA) titer was 1:3200, anti-double stranded DNA antibodies (antidsDNA) 510 RU/ml, anti-RNP antibody and anti-sm antibody were positive. Direct Coombs was +2, C3 level was 0.2 g/L, C4 level was 0 g/L. 24-hour urine proteinuria was 13 mg/m^2^/h.

Pulse methyl prednisolone (PMP) 30mg/kg/day was started with a diagnosis of SLE. On the 2nd day of treatment, hemoglobin decreased from 11.3 g/dL to 9.7 g/dL. Diffuse alveolar hemorrhage was observed on chest radiography and thoracic computed tomography (CT) (Figure 1). She was intubated. Plasmapheresis was performed for 7 days. Cyclophosphamide (CYC) was given. However, it was switched to rituximab (RTX) because of persistent respiratory failure and refractory thrombocytopenia. Clinical and imaging findings improved.

Case 2: A 10-year-old girl presented with complaints of malaise and headache. Physical examination revealed malar rash, pretibial edema and hypertension. Laboratory findings were as follows: leukocyte count 3560/mm^3^, ALC 841/mm^3^, hemoglobin 8.1 g/dL, platelets 146,000/mm^3^, ESR 80 mm/h, CRP 6.8 mg/dL, creatinine 1.23 mg/dL, albumin 2.6 mg/dL. ANA titer was 1:1000, antidsDNA >800 RU/ml, direct Coombs +2, c3 level was 0.3 g/L and c4 level was 0 g/L. 24-hour urine protein was 435 mg/m^2^/h.

A diagnosis of SLE was made and PMP 30 mg/kg/day (3 days) was started. Hemoptysis developed during follow-up. Hemoglobin decreased from 8.1 g/dL to 6.8 g/dL. Diffuse alveolar hemorrhage was observed on chest radiography and CT (Figure 2). Plasmapheresis was performed and CYC was added. Renal biopsy revealed class 4 lupus nephritis. Control CT showed regression in pulmonary findings. However, treatment was intensified with plasmapheresis (2 days), IVIG (1 g/kg/day-2 days) and RTX because of refractory anemia and thrombocytopenia.

**Conclusion:** Diffuse alveolar hemorrhage is a life-threatening complication of SLE. A favorable outcome can be achieved with plasmapheresis and intensive immunosuppressive therapy.

**Patient Consent**

Yes, I received consent

**Disclosure of Interest**

None declared

## P117 Outcomes of juvenile onset systemic lupus erythematosus in young adulthood

### A. Radziszewska, M. Niwa, J. Peng, Y. Gao, H. Peckham, M. Butt, C. Ciurtin

#### Centre for Adolescent Rheumatology, University College London, London, United Kingdom

##### **Correspondence:** C. Ciurtin

*Pediatric Rheumatology 2023*, **21(Suppl 2):**P117

**Introduction:** Juvenile systemic lupus erythematosus (JSLE) is characterised by a more severe clinical presentation than the adult phenotype, as well as increased risk for damage accrual and comorbidities. Most of the published clinical research in JSLE is focused on the first few years of the disease course, and therefore there is an unmet need to investigate JSLE outcomes in adulthood.

**Objectives:** To characterise the medium-term outcomes of young adults (YA) with JSLE followed up in our centre to identify disease outcomes and predictors of damage.

**Methods:** Detailed patient and disease characteristics, including demographics, clinical and serological parameters, type of organ involvement and medication, were collected from medical records. Disease activity and damage were assessed using validated scores (SLEDAI-2000 and pBILAG, and PedSDI, respectively). Results are presented using descriptive statistics and regression analysis.

**Results:** Complete datasets were available for 89 JSLE patients (75 females and 14 males) with a mean age of 26 ± 4.18 years and disease duration of 13.5± 4.71 years (31.4% White, 24.7% Black and 22.4% Asian and 21.5% Other). There were no sex differences between patients who accrued damage (27%) vs. not (73%), but patients with damage were older (27±3 vs. 25±4 years, p=0.026), had more (50% vs. 27.6%, p=0.048) and neurological involvement (33.3% vs. 13.8%, p= 0.038), and higher median global BILAG (1±1.7 vs. 0.9±3.3, p= 0.048) but not SLEDAI (2±2.21 vs. 2±2.9, p=0.09) at the last assessment. Patients without damage were more frequently treated with azathioprine (73.8% vs. 50%, p=0.033), while there were no significant differences in the use of other medications, including steroids. Overall, non-White vs. White patients had higher SLEDAI scores at last assessment (p=0.005). Neurological manifestations and presence of anti-La antibodies were predictors of damage in the multivariate logistic regression analysis, when accounting for age, sex, ethnicity, renal disease, disease duration, and disease activity at last assessment (p=0.04, p=0.01, respectively). YA with JSLE were 4.541 times more likely to accrue damage, if they had neurological involvement and 6.67 times more likely if they tested positive for anti-La antibodies. Anti-La antibody (found in 15.7% YA with JSLE in this cohort) correctly classified 36.36% of YA with damage (if positive) and 90.32% of YA without JSLE- associated damage (if negative) (ROC=0.79).

**Conclusion:** This analysis, of the largest YA JSLE cohort in the UK, highlights ethnic disparities in JSLE outcomes in young adulthood. Neurological manifestations, LN, older age, and more active disease at the last assessment were the main predictors of damage, providing evidence for both disease-severity determinants, as well as increased age, as main drivers of JSLE-related damage, despite paediatric studies previously highlighting that JSLE was more severe in younger children. The relevance of anti La seropositivity in predicting damage accrual requires further investigation. Future research in JSLE in young adulthood is needed to validate these preliminary findings and translate them into optimised management strategies for improved outcomes in this under-researched patient population.

**Patient Consent**

Not applicable (there are no patient data)

**Disclosure of Interest**

None declared

## P118 The predictive value of autoantibody profiles and risk of (late-onset) lupus nephritis in CSLE

### M. de Jong^1^, S. Bergkamp^1^, M. Oosterveld^2^, E. van Leeuwen^3^, M. Gruppen^1^, T. Kuijpers^1^, J. M. van den Berg^1^, D. Schonenberg-Meinema^1^

#### ^1^Emma Childrens Hospital, Department of Pediatric Immunology, Rheumatology and Infectious Diseases; ^2^Emma Childrens Hospital, Department of Pediatric Nephrology; ^3^Department of Laboratory Medicine, Laboratory of Medical Immunology, University of Amsterdam, Amsterdam University Medical Centers, Amsterdam, Netherlands

##### **Correspondence:** M. de Jong

*Pediatric Rheumatology 2023*, **21(Suppl 2):**P118

**Introduction:** Specific autoantibody profiles and associations with (new) organ involvement in Systemic Lupus Erythematosus (SLE) have been studied before (1), but the exact influence of autoantibodies on the disease course, specifically in children, remains unclear. Lupus nephritis (LN) occurs in around 50-82% of cases of childhood-onset SLE (cSLE) patients with most cases presenting early on in the disease (2,3). However, the amount of longitudinal studies concerning cSLE is limited and there is a lack of data on the development of new organ involvement over time.

**Objectives:** To study the autoantibody profiles in a longitudinal cSLE-cohort and determine whether specific autoantibodies can be associated with the occurrence of lupus nephritis (LN) and Systemic Lupus Erythematosus Disease Activity Index (SLEDAI) score at diagnosis. Second objective was to identify if there is still risk of new renal involvement after two years of diagnosis.

**Methods:** The study population consisted of patients with the diagnosis cSLE, as defined by the SLICC 2012 criteria as SLE with disease onset before the age of 18 years. We prospectively collected clinical features, laboratory results and SLEDAI score. Binary logistic regression analysis was performed to relate independent variables to the occurrence of lupus nephritis.

**Results:** N=51 cSLE patients were studied of which forty-three (43/51, 84.3%) were female. Median age at disease onset was 14.8 years (IQR 13.6-16.4) and median follow-up time was 4.6 years (IQR 2.8-8.2). Logistic regression analysis showed an association between anti-RNP 70kD presence and the occurrence of LN (OR 9.128, 95% CI 1.268-65.713, p=0.028). No statistical association was found between specific autoantibodies and SLEDAI score at diagnosis. Three patients (3/21, 13.4%) developed LN after two years of diagnosis and all three were not therapy compliant.

**Conclusion:** Our study showed an association between anti-70-kD positivity and the occurrence of LN in cSLE. No specific autoantibody was associated with SLEDAI score at diagnosis. cSLE patients can still develop LN after two years of diagnosis which is important for screening protocols in daily practice.

**Patient Consent**

Yes, I received consent

**Disclosure of Interest**

None declared

**References**

1. Benito-Garcia E, Schur PH, Lahita R. Guidelines for immunologic laboratory testing in the rheumatic diseases: anti-Sm and anti-RNP antibody tests. Arthritis Rheum. 2004;51(6):1030-44.
2. Samanta M, Nandi M, Mondal R, Hazra A, Sarkar S, Sabui T, et al. Childhood lupus nephritis: 12 years of experience from a developing country's perspective. Eur J Rheumatol. 2017;4(3):178-83.
3. Parikh SV, Almaani S, Brodsky S, Rovin BH. Update on Lupus Nephritis: Core Curriculum 2020. Am J Kidney Dis. 2020;76(2):265-81.

## P119 Comparisons of different anti-dsdna detection methods (ELISA vs ELIA) in CSLE

### M. de Jong^1^, S. Bergkamp^1^, E. van Leeuwen^2^, M. Gruppen^1^, T. Kuijpers^1^, J. M. van den Berg^1^, D. Schonenberg-Meinema^1^

#### ^1^Emma Childrens Hospital, Department of Pediatric Immunology, Rheumatology and Infectious Diseases; ^2^Department of Laboratory Medicine, Laboratory of Medical Immunology, University of Amsterdam, Amsterdam University Medical Centers, Amsterdam, Netherlands

##### **Correspondence:** M. de Jong

*Pediatric Rheumatology 2023*, **21(Suppl 2):**P119

**Introduction:** The presence of anti-dsDNA antibodies in above threshold titres is one of the established criteria for the diagnosis of Systemic Lupus Erythematosus (SLE), however there is a variety of detection methods that are being utilized. This may lead to a lack of uniformity in results and therefore hypothetically to misdiagnose patients.

**Objectives:** The objective of this study was to compare two different anti-dsDNA detection methods (ELISA vs EliA) in childhood-onset SLE (cSLE) and to assess whether there is uniformity in the results.

**Methods:** The study population consisted of cSLE patients, classified by the 2012 SLICC criteria with a disease onset <18 years old. The enzyme-linked immunosorbent assay (ELISA) test and the enzyme-labelled anti-isotype assay (EliA) (ThermoFisher Scientific) test for anti-dsDNA presence were performed simultaneously.

**Results:** In total, twenty-four cSLE patients were tested simultaneously of which twenty-two (22/24, 91.7%) were female. Median age at disease onset was 14.4 years (IQR 12.6-16.3) and median disease duration was 4.1 years (IQR 2.4-7.4). Six patients (6/24, 25%) had a positive anti-dsDNA test with the ELISA method, but tested negative with the EliA method. One patient (1/24, 4.2%) tested borderline-negative with the ELISA method, and was positive with the EliA method. Thus in total, seven patients (7/24, 29.2%) did not have synchronous results.

**Conclusion:** We demonstrated a 25-30% discrepancy in results between two different anti-dsDNA detection methods, mainly due to positive outcomes with the ELISA method, while the EliA method tested negative. This emphasizes the importance of considering the differences in sensitivity and specificity between anti-dsDNA detection methods to avoid missing potential SLE patients.

**Patient Consent**

Yes, I received consent

**Disclosure of Interest**

None declared

## P120 Assessment of skin findings in patients with childhood onset systemic lupus erythematosus

### E. Esen, S. Ö. Çiçek, A. P. Kısaarslan, M. H. Poyrazoğlu

#### Pediatric Rheumatology, Erciyes University Faculty of Medicine, Kayseri, Türkiye

##### **Correspondence:** E. Esen

*Pediatric Rheumatology 2023*, **21(Suppl 2):**P120

**Introduction:** Systemic lupus erythematosus (SLE) is an autoimmune disease with multiorgan involvement. The skin is one of the most frequently involved organs. Skin manifestations in SLE were defined as specific and non-specific lupus findings according to histopathological features. Specific involvement is classified as acute cutaneous lupus erythematosus (CLE), subacute CLE and chronic CLE. The importance of cutaneous findings in terms of systemic involvement is not clear.

**Objectives:** We aimed to analyze the cutaneous manifestations of SLE. Also, we aimed to investigate the relationship between skin findings and other systemic manifestations of disease.

**Methods:** Patients who were diagnosed with SLE under the age of 18 and followed up in the pediatric rheumatology clinic of Erciyes University were included in the study. Demographic characteristics, laboratory and skin findings of the patients were analyzed retrospectively.

**Results:** A total of 118 patients included to the study.Five patients were followed up with the diagnosis of cutaneous lupus, and no additional systemic findings were observed in addition to the skin findings in these patients. Another 113 patients had other systemic manifestations except skin findings. Four patients were diagnosed with monogenic lupus under the age of 5 years.Eighty point five percent of the patients were female, and the median age at diagnosis was 155 (15-213) months.Acute cutaneous lupus was observed in 86 patients, subacute cutaneous lupus in four, and chronic cutaneous lupus in 28 patients.The distribution of lupus-specific and non-specific skin findings of the patients are given in Table 1.When system involvement was evaluated according to skin findings, musculoskeletal system involvement was higher only in patients with nonspecific skin findings.Pleuritis/pericarditis was seen more frequent in patients with only specific skin findings.None of the patients with pleuritis/pericarditis had nonspecific skin findings.Laboratory findings did not differ between groups. System involvement and laboratory findings data according to skin findings are shown in Table 2.

**Conclusion:** Skin manifestations are very common in SLE and may be a clue in terms of other organ involvement.With further analysis, we are planning to examine the relationship between the subtype of the skin findings and the system involvements.

**Patient Consent**

Yes, I received consent

**Disclosure of Interest**

None declared

**References**

1. Chiewchengchol D, Murphy R, Morgan T, Edwards SW, Leone V, et al. Mucocutaneous manifestations in a UK national cohort of juvenile-onset systemic lupus erythematosus patients *Rheumatology*, Volume 53, Issue 8, August 2014, Pages 1504–1512,10.1093/rheumatology/keu137
2. Chiewchengchol D,Murphy R, Edwards SW, Beresford MW, Mucocutaneous manifestations in juvenile-onset systemic lupus erythematosus: a review of literatüre, Pediatr Rheumatol Online J. 2015 Jan 5;13:1. doi:10.1186/1546-0096-13-1.
3. Harry O. Yasin S.,BrunnerH. Childhood-Onset Systemic Lupus Erythematosus: A Review and Update, J Pediatr 2018 May;196:22-30.e2. doi: 10.1016/j.jpeds.2018.01.045.

## P121 Rituximab induction and maintenance therapy (RTX-IMT) can achieve sustained remission in severe courses of juvenile-onset systemic lupus erythematosus

### F. Speth^1,2^, A. Fröhlich^1,2^, S. Farmand^2^, A. Matskevych^1^, S. Loos^1^, K. Lehmberg^2^, A. C. Muntau^1^, I. Müller^2^, J. Pagel^1,2^

#### ^1^Department of Pediatrics; ^2^Division of Pediatric Stem Cell Transplantation and Immunology, Department of Pediatric Hematology and Oncology, University Medical Center Hamburg-Eppendorf, Hamburg, Germany

##### **Correspondence:** S. Farmand

*Pediatric Rheumatology 2023*, **21(Suppl 2):**P121

**Introduction:** B cells play an important role in the pathogenesis of juvenile-onset systemic lupus erythematosus (JSLE). While use of rituximab (RTX) for B-cell depletion in adult SLE patients shows conflicting results^1,2^, there is growing evidence that RTX is a valuable option in JSLE^3^. Notably, the efficacy of RTX on the clinical response in adult SLE patients is associated to complete peripheral B-cell depletion^2^, and a sustained response may require repetitive RTX administrations^4,5^.

**Objectives:** To achieve sustained remission in patients with severe juvenile-onset systemic lupus erythematosus by means of rituximab induction and maintenance therapy (RTX-IMT).

**Methods:** 9 patients (age 10-17 years) with SLEDAI value ≥12 and positive anti-dsDNA antibodies and/or severe autoimmune cytopenia or nephritis class III or IV (+/-V), were treated with RTX-IMT over a time period of 12-66 months. Induction therapy consisted of 4x375mg/m^2^ RTX within the first month and an initial methylprednisolone pulse (10mg/kg/d for 3 days). Maintenance therapy varied between 2-11 cycles of RTX (1x750mg/m^2^) to achieve the following combined “treat to target” endpoints: B-cell (CD19) count <5/mm^3^ and IgM <0.4 g/l and SLEDAI <6, negative anti-dsDNA antibodies, C3/C4 normalization (2/3 required). All patients received additive therapy with hydroxychloroquine, mycophenolate and low-dose prednisone. Supportive IgG-replacement and pneumocystis-jirovecii-prophylaxis were also administered during RTX-IMT.

**Results:** RTX-IMT was safe and highly effective in all patients. Mean SLEDAI dropped from 34.2 to 1.3. All patients showed C3/C4 normalization and 6/9 patients became negative for anti-dsDNA antibodies. There were no flares or persistent organ damage during the observation time. The treatment was well tolerated and no adverse events or major infections occurred. We suspect that the regular use of immunoglobulins contributed to the observed low infection rate and thus possibly eliminated an important trigger for flares.

**Conclusion:** Our data show the efficacy of RTX-IMT in severe JSLE under predefined treat-to-target conditions. We propose the combination of IgM <0.4g/l and CD19 <5/mm^3^ as immunological key parameters reflecting a profound depletion of B cells in blood and lymphatic tissue. Furthermore, we advocate accompanying IgG replacement to reduce infectious complications during B-cell depletion. RTX should be evaluated in randomized controlled trials in JSLE under continuous documentation of immunological parameters to achieve a high level of safety and effectiveness.

**Trial registration identifying number:** Not applicable.

**Patient Consent**

Yes, I received consent

**Disclosure of Interest**

None declared

**References**

1. Wise LM, Stohl W. Belimumab and Rituximab in Systemic Lupus Erythematosus: A Tale of Two B Cell-Targeting Agents. Front Med (Lausanne). 2020 Jun 30;7:303
2. Gomez Mendez LM, Cascino MD et al. Peripheral Blood B Cell Depletion after Rituximab and Complete Response in Lupus Nephritis. Clin J Am Soc Nephrol. 2018 Oct 8;13(10):1502-1509.
3. Peterknecht E, Keasey MP, Beresford MW. The effectiveness and safety of biological therapeutics in juvenile-onset systemic lupus erythematosus (JSLE): a systematic review. 2018 Nov;27(13):2135-2145.
4. Cassia MA, Alberici F et al. Rituximab as Maintenance Treatment for Systemic Lupus Erythematosus: A Multicenter Observational Study of 147 Patients. Arthritis Rheumatol. 2019 Oct;71(10):1670-1680.
5. Chen X, Shi X et al. Rituximab as maintenance therapy following remission induction in relapsing or refractory systemic lupus erythematosus. Rheumatology (Oxford). 2023 Mar 1;62(3):1145-1152

## P122 Systemic lupus of pediatric onset in Afro-caribbean children: a cohort study in the french west Indies and French Guiana

### A. Felix^1^, F. Delion^2^, Y. Hatchuel^1^

#### ^1^Martinique University Hospital, Fort-De-France, Martinique; ^2^Guadeloupe University Hospital, Pointe-a-Pitre, Guadeloupe

##### **Correspondence:** A. Felix

*Pediatric Rheumatology 2023*, **21(Suppl 2):**P122

**Introduction:** Systemic diseases of pediatric onset are more frequent in the Afro-Caribbean population. We performed a study of patients followed in the French overseas departments of America (FOAD) for pediatric systemic lupus erythematosus (pSLE).

**Objectives:** The aims were to describe the clinical and biological specificities during childhood in this population.

**Methods:** A retrospective study was conducted between January 2000 and September 2021. Patients with pSLE were identified from multiple sources: computerized hospital archives, registry of referring pediatricians, adult specialists in internal medicine and the French National Registry for rare diseases. We studied SLE with pediatric onset defined by international criteria.

**Results:** Overall, 2148 patients were identified, of whom 54 were included. The average follow-up was 8.3 years (range: 0.3—25 years). We observed an increase in new diagnoses over time. At onset, pSLE patients had a median of 10 SLICC criteria (range: 4–12), and the median EULAR/ACR 2019 score was 38 (12—54). At onset, one third of patients had renal involvement, 15% had neurolupus and 41% cardiac involvement. During childhood, 54% had renal involvement, and 26% suffered from neurolupus. Patients suffered a median of 3 flares during childhood, and 26% had more than 5 flares. Patients with younger age at onset ( under 12 years old ) had worse outcomes, with an average of 5.6 flares during childhood (vs 2.6 for children diagnosed after 12 years old, *p* = 0.02). They required an average of four lines of background therapy during childhood (vs 2 for children diagnosed after 12 years old, *p* = 0.04). Half of them required the addition of rituximab during childhood (*p* = 0.05). Sex was not associated with severity of pSLE during childhood (boys vs girls: average number of flares, respectively 3.1 vs 3.4; *p* = 0.42; average steroid dose during childhood, respectively, 9.5 mg vs 10.8 mg; *p* = 0.21). However, at the time of transition to adult care, boys seemed to have better disease control than girls, with median SLEDAI of 3 vs 11 (*p* = 0.12), median ESR after one hour of 5 vs 30 (*p* = 0.03), and median steroid dose at 4 mg vs 10 mg (*p* = 0.04) respectively.

**Conclusion:** This retrospective study from the FOAD between 2000 and 2021 describes a cohort of 54 patients suffering from pSLE. This retrospective cohort of patients with pSLE followed in the FOAD is the largest cohort of Afro-Caribbean children treated in a developed country’s healthcare system. The outcomes of Afro-Caribbean patients were similar to those in Western population, but with worse disease activity at onset. Since our study highlighted a worrisome trend towards a persistent increase in the prevalence of pediatric lupus over time, environmental factors specific to these regions need to be investigated in future prospective studies.

**Patient Consent**

Yes, I received consent

**Disclosure of Interest**

None declared

## P123 Protein-losing enteropathy: early manifestation of childhood-onset systemic lupus erythematosus

### F. Flora^1^, C. De Simone^1^, M. Tardi^2^, L. Martemucci^2^, F. Orlando^2^

#### ^1^Department of Translational Medical Science, Section of Pediatrics, University of Naples Federico II; ^2^Pediatric Rheumatology Unit, Department of General and Emergency Pediatrics, Santobono-Pausilipon Children's Hospital, Naples, Italy

##### **Correspondence:** F. Flora

*Pediatric Rheumatology 2023*, **21(Suppl 2):**P123

**Introduction:** Protein-losing enteropathy (PLE) is a rare manifestation of systemic lupus erythematosus (SLE) in children. It is characterized by leakage of serum protein into the gastrointestinal tract, which results in hypoproteinemia and generalized edema without significant proteinuria, severe liver disease or malabsorption^1^. PLE is often described as a complication of SLE, but in some cases, it is the presenting manifestation.

**Objectives:** We report a case of SLE that initially presented as PLE.

**Methods:** A 14-year-old girl was admitted to our department for eyelid and pretibial edema and chest pain. She had a story of weight gain and periorbital edema upon awakening in the last 3 months. There was no history of medication or diarrhea or chronic illness that could explain these symptoms. Laboratory tests showed hypoalbuminemia (Albumin 1.9 g/dl), ANA and Anti dsDNA positivity, C3 at the lower limits (0.9 g/l). Principal hepatologic, gastrointestinal and endocrinologic causes were excluded. Proteinuria was normal. Gastroscopy and colonoscopy provided no evidence of lesions, and biopsies showed non-specific changes. Fecal a1-antitrypsin showed an intestinal protein loss (>3000 mg/g stools), consistent with the diagnosis of PLE; however, she did not have a diagnosis of SLE according to 2019 EULAR/ACR criteria^2^. She started steroid therapy with symptoms resolution and no relapse after stopping steroids. After 19 months off-therapy, she had a clinical and laboratory relapse with fever, eyelid edema and hypoalbuminemia (1.7 g/dl). Laboratory and radiological investigations revealed low C3 and C4 levels and serositis. There was no significant proteinuria. ANA and Anti dsDNA were confirmed positive. SLE was diagnosed based on the 2019 EULAR/ACR criteria^2^. She underwent methylprednisolone in intravenous pulses of 1 g/day for 3 days. Hydroxychloroquine and methotrexate were started for maintenance therapy.

**Results:** We reanalyzed the 12 pediatric cases described in the literature of SLE with PLE^3^ on the basis of the most recent EULAR/ACR criteria. The median age was 12 years (0.9-17). Five of those (41.7%) presented PLE as initial manifestation of SLE which was subsequently diagnosed. The median time between the onset of PLE and the diagnosis of SLE was 36 months (range 4-67), while in our patient was 21 months. Only three patients (25%) presented diarrhea as symptomatology of PLE. In all cases, steroid therapy was practiced, in 4/12 (33.3%) azathioprine, in 3/12 (25%) cyclosporine and in a single case cyclosporine, azathioprine and rituximab. To our knowledge, this is the first case of LUPLE in childhood treated with methotrexate.

**Conclusion:** Although PLE does not meet the diagnostic criteria for SLE, the diagnosis of SLE should be considered in children with hypoalbuminemia without other explanation, even in the absence of gastrointestinal symptoms. Therefore, children with PLE should be monitored over time to detect symptoms and laboratory alterations suggestive of SLE.

**Patient Consent**

Yes, I received consent

**Disclosure of Interest**

None declared

**References**

1. Trapani S, Rubino C, Simonini G, et al. Gastrointestinal and hepatic involvement in paediatric systemic lupus erythematosus. *Clin. Exp. Rheumatol.* 2021;39:899–906.
2. Aringer M, Costenbader K, Daikh D, et al. 2019 European League Against Rheumatism/American College of Rheumatology Classification Criteria for Systemic Lupus Erythematosus. *Arthritis Rheumatol. (Hoboken, N.J.)*. 2019;71:1400–1412.
3. Naddei R, Orlando F, Aloj G, et al. Differential diagnosis of hypoalbuminemia in childhood: protein losing enteropathy associated to systemic lupus erythematosus in a young boy. *Eur. J. Gastroenterol. Hepatol.* 2020;32:127–129

## P124 Linear cutaneous lupus erythematous in a twenty months old child

### M. F. Gicchino^1^, V. Piccolo^2^, E. Miraglia del Giudice^1^, A. N. Olivieri^1^

#### ^1^Department of woman, child and general and specialized surgery; ^2^Dermatology, University of Campania Luigi Vanvitelli, Napoli, Italy

##### **Correspondence:** M. F. Gicchino

*Pediatric Rheumatology 2023*, **21(Suppl 2):**P124

**Introduction:** Linear cutaneous lupus erythematosus (LCLE) is an unusual presentation of cutaneous lupus following Blaschko’s lines. It is described mostly in children and young adults and is usually not associated with systemic involvement.

**Objectives:** to report a case of linear cutaneous lupus erythematosus in a young child

**Methods:** a twenty months old child presented to our paediatric rheumatology department with an itchy erythematous linear lesion on her right lower limb since six months. Skin lesion appeared after a Sars-Cov-2 infection. After a dermatological evaluation treatment with topical steroids was started, but the lesion did not improve. Patient’s parents did not refer systemic symptoms such as fever, weight loss, arthralgia, oral ulcers, photosensitivity, or cardiopulmonary symptoms. Physical examination revealed linear cutaneous erythematosus lesion with erythemato-violaceous papules on her posterior right thigh and leg. Patient did not present neither joint pain nor limp. The rest of clinical examination was unremarkable. Complete blood count, inflammatory parameters, liver, kidney function, iron levels was in the norm. Both Antinuclear antibodies and ENA were negative. Sars-Cov-2 serology showed a previous infection (IgG 1417 BAU/ml), the rest of virological screening was negative. Quantiferon test was negative. Urinalysis was in the norm. Faecal calprotectin was negative. Both heart and abdomen ultrasound were in the norm. Thanks to a skin ultrasound, performed with a high frequency probe a subcutaneous involvement was ruled out. In order to perform a correct diagnosis and start an adequate treatment a skin biopsy was performed.

**Results:** Skin biopsy revealed vacuolar interface dermatitis with superficial lymphohistiocytic perivascular and periadnexal infiltrates. According to biopsy result linear cutaneous lupus erythematous was diagnosed. After an ophthalmologic evaluation hydroxychloroquine treatment (5mg/kg/day) was started with improvement of symptoms.

**Conclusion:** LCLE represents a rare subtype of CLE mostly described among children and young adults. The term “linear” refers to its peculiar clinical presentation in which lesions follow Blaschko’s lines. LCLE in childhood presents less probability of evolving to systemic disease. In childhood median age of onset was 9 years old (1–17 years old), with a female predominance of 3F:1M. The head and neck area were most frequently involved (57%), followed by extremities (46%) and trunk (14%). LCLE lesions were usually erythematous and/or violaceous and may be atrophic (46%) and/or squamous (40%). Patients were mostly asymptomatic but itch was occasionally noted, as in the case reported. Skin biopsy is very important to confirm diagnosis and start an adequate treatment. Hydroxychloroquine, a first-line drug, promotes remission of lesions in most cases along with a favourable safety profile. Dapsone, methotrexate, moderate to high potency topical corticosteroids and topical calcineurin inhibitors can also be employed in patients that are unresponsive to hydroxychloroquine or when this drug is contraindicated.

**Patient Consent**

Yes, I received consent

**Disclosure of Interest**

None declared

## P125 Monogenic lupus patient with a novel DNASE1L3 gene variant and anca associated vasculitis

### S. S. Hashad, I. A. Almsellati1, H. M. Etayari on behalf of Iman A Almsellati, Hala M. Etayari, Majda N. Etfil

#### Rheumatology , Tripoli Children Hospital , Tripoli , Libya

##### **Correspondence:** S. S. Hashad

*Pediatric Rheumatology 2023*, **21(Suppl 2):**P125

**Introduction:** SLE and AAV are rare diseases that share clinical symptoms, and possibly severe renal involvement. They can be easily distinguished by demographic characteristics, an autoantibody profile and a renal pathology.

**Objectives:** A Case report of monogenic Lupus patient with a novel mutation variant in the DNASE1L3 gene presented as ANCA associated vasculitis.

**Methods:** Case report:

**Results:** Case report:

A 9-year-old boy, product of non-consanguineous parent, from a closely related tribe. His brother was diagnosed as microscopic Polyangitis proven with renal biopsy and serology. His sister is a case of lupus nephritis. He presented with one year history of intermittent low fever, generalized maculopapular rash, arthritis of large joints and recurrent abdominal pain. The results of investigation showed that HGB was 7.1, ESR was 80 and Urine routine examination was normal. Rheumatologic workup revealed: ANA, Anti dsDNA and ENA were negative and normal complement study. He was diagnosed as "systemic onset juvenile idiopathic arthritis". He was Received IV and oral steroid. His symptoms improved and his laboratory finding normalized. 6 months later while on low 5 mg prednisolone, he had disease flare with rash, arthritis, recurrent episodes of haemoptysis &recurrent abdominal pain. Re-evaluated: Para nasal maxillary sinusitis, CT of chest showed bilateral granulation tissues. C-ANCA was positive. He fulfilled the Criteria for Diagnosing Granulomatosis with Polyangitis. Remission induced by Methylprednisolone and Rituximab, maintained by oral prednisolone and methotrexate for 3 years. Due to poor drug compliance, relapsed with sever nephritis. Remission induced by Methylprednisolone and maintained by mycophenolate mofetil. Due to the recurrent severe relapses and the family history of other sibling with a diverse rheumatoogical disease a Whole Exome Sequencing was requested and the analysis identified the homozygous mutation variants c, 418c>t in the DNASE1L3 gene, he confirmed as a Case of Monogenic lupus/AAV overlap syndrome.

**Conclusion:** A loss of function variant in DNASE1L3 gene has been identified in familial SLE cases with the association of AAS. We identified a novel DNASE1L3 gene variant leading to monogenic lupus and. Secondary screening of family members is advised. This is the first time this homozygous variant c, 418c>t in the DNASE1L3 gene reported as pathogenic in the literatures.

**Patient Consent**

Yes, I received consent

**Disclosure of Interest**

None declared

## P126 Role of nigella sativa as an immunomodulator in reducing the Anti-U1-RNP antibody

### Z. Hikmah, M. Masturina

#### Child Health Department, Universitas Airlangga, Surabaya, Indonesia

##### **Correspondence:** Z. Hikmah

*Pediatric Rheumatology 2023*, **21(Suppl 2):**P126

**Introduction:** Systemic lupus erythematosus (SLE) is an autoimmune disease with various manifestations and varying degrees of severity. Recent studies suggest the therapeutic potential of Nigella sativa in SLE. Nigella sativa (NS) has anti-inflammatory and immunomodulatory effects by reducing symptoms of SLE. Anti-U1-RNP antibody is associated with various clinical and prognostic manifestations in SLE

**Objectives:** The aim of this study is to determine the difference of anti-U1-RNP in SLE mice that administered Nigella sativa

**Methods:** The research design was an experimental study. The study consisted of 10 healthy mice and 30 mice injected with intraperitoneal pristane to become a lupus model after 4 months. Thirty mice model lupus were randomly divided into 3 treatment groups, placebo, steroids and nigella sativa. The dose of NS was 4.8g/kg/day for 28 days. Evaluation of the results was performed on the 28th day by taking blood samples and evaluating the kidney histopathological.

**Results:** The mean of anti-U1-RNP antibody in the four groups were 2.95 ± 1.06 in non SLE mice, and 9.47 ± 0.43 , 3.28 ± 0.81, 3.66 ± 1.20 for placebo, steroid dan NS mice, respectively). There was significant differences of anti-U1-RNP antibody between the placebo and steroid groups, and between the placebo and nigella sativa groups (p<0.05). There was no significant difference in Anti-U1-RNP antibody (p>0.05) between steroid and nigella sativa group. However, in the ANOVA analysis, there was a significant difference in Anti-U1-RNP antibody in the four treatment of groups (p<0.05).

**Conclusion:** Nigella sativa had potential therapy in SLE which acts as an immunomodulator by significantly reducing the value of Anti-U1-RNP antibody. Further exploration regarding the efficacy of therapy, effective dosage, and safety of therapy needs to be performed to implement as therapy in SLE patients.

**Patient Consent**

Not applicable (there are no patient data)

**Disclosure of Interest**

None declared

## P127 Cardiovascular involvement in pediatric systemic lupus erythematosus: seven years of experience of university children’s hospital, sofia, Bulgaria

### D. Hristova, A. Dimitrova, M. Ganeva, K. Temelkova, T. Vasilev, S. Stefanov

#### Pediatric rheumatology and cardiology, University Children's Hospital , Sofia, Bulgaria

##### **Correspondence:** D. Hristova

*Pediatric Rheumatology 2023*, **21(Suppl 2):**P127

**Introduction:** Pediatric-onset SLE (15-20% of all cases) is associated with a higher incidence of cardiovascular involvement and a more aggressive clinical course.

**Objectives:** To describe the cardiac manifestations in patients diagnosed with SLE between December 2015 and April 2023.

**Methods:** Demographic, clinical and instrumental parameters of children with SLE were recorded. We performed electrocardiography and echocardiography initially and then regularly during follow-ups, along with X-ray scans and markers of cardiac myocytolysis in cases of suspected myocardial involvement.

**Results:** Twenty-six patients with SLE (16 girls and 10 boys; ratio 1.6:1) with a mean age of 12.5 (5-16) years were admitted for a period of 7 years. Myocardial involvement was found in 7.7%, pericarditis in 30.7% (two patients underwent surgical drainage). Mitral valve prolapse was registered in 23.1%, mitral regurgitation in 57.7%, aortic regurgitation in 23.1%, coronary injury in 11.5%, arterial hypertension in 11.5%. The clinical course was favorable in most of the children, two patients (7.7%) died (severe hypertension, cardiorespiratory failure).

**Conclusion:** Our data demonstrates that the most commonly observed cardiac manifestations in children with SLE are mitral regurgitation, pericarditis, mitral valve prolapse, and aortic regurgitation.

**Patient Consent**

Yes, I received consent

**Disclosure of Interest**

None declared

## P128 Pediatric systemic lupus erythematosus: single center's experience

### M. Kapranova, S. Kurbanova, E. Zholobova, A. Sologub, A. Nargizyan, E. Korobyants, M. Dzis, A. Marandzhyan, S. Valieva

#### Department of Rheumatology, Morozov Children’s City Clinical Hospital, Moscow, Russian Federation

##### **Correspondence:** S. Kurbanova

*Pediatric Rheumatology 2023*, **21(Suppl 2):**P128

**Introduction:** Systemic lupus erythematosus (SLE) is a multifactorial autoimmune rheumatic disease with uncontrollable immune response, characterized by autoantibody production and protean organ manifestations. Clinical appearance varies from isolated skin damage or joint involvement to critical organ-threatening disease like nephritis.

**Objectives:** The purpose of this study was to evaluate clinical and epidemiological data based on a retrospective analysis of 42 patients with SLE from a single medical center.

**Methods:** We reviewed the medical records of patients diagnosed with SLE at Morozov Children's City Clinical Hospital between January 1st, 2020 and May 31, 2023. For each patient we collected the following data: age, sex, familiar history, clinical and laboratory parameters, inflammatory activity, extractable nuclear antibodies and treatments.

**Results:** The median age of patients was 13 years. 73.8% of patients (n=31) were girls, 26.2% (n=11) were boys. According to the clinical criteria of SLICC 2012, the most common manifestations were skin lesions - 81% (n=34), immunological criteria - (ANA and/or anti – dsDNA) 100% (n=42), hemolytic anemia - 54.8% (n=23) and leukopenia - 52.4% (n=22). The frequency of other manifestations was: synovitis - 47.6% (n=20), thrombocytopenia - 47.6% (n=20), renal disorders - 45.2% (n=19), serositis - 31% (n=13), neurological disorders - 4.76% (n=2). Disease activity was high in more than a half of the patients (54.8%, n=23), 40.4% (n=17) had moderate disease activity, 4.8% (n=2) had minimal disease activity. Induction therapy by IV pulse methylprednisolone was used in 71.4% (n=30), oral prednisolone – in all patients (n=42), mycophenolate mofetil in 57.1% (n=24), hydroxychloroquine – 88.1% (n=37), IVIG – 40.5% (n=17), IV cyclophosphamide – 14.3% (n=6), methotrexate – 7.1% (n=3). Biologic therapy was given to 31% of patients (n=13), with Rituximab being the drug of choice.

**Conclusion:** Diagnosing of systemic lupus erythematosus requires a multidisciplinary approach and is a challenge for clinicians. Being alert for systemic disease helps to make a timely diagnosis and prevent the development of complications in patients.

**Patient Consent**

Yes, I received consent

**Disclosure of Interest**

None declared

## P129 The wide spectrum of clinical manifestations of antiphospholipid syndrome in children

### H. D. Karakas, F. Aydin, Z. B. Ozcakar, N. Cakar

#### Pediatric Rheumatology, Ankara University, Ankara, Türkiye

##### **Correspondence:** H. D. Karakas

*Pediatric Rheumatology 2023*, **21(Suppl 2):**P129

**Introduction:** Antiphospholipid syndrome (APS) is a systemic autoimmune disorder characterized by an increased risk of arterial and venous thrombosis, associated with the presence of persistently positive antiphospholipid antibodies (aPL). APS may occur as a stand-alone disorder (primary APS) or in patients with other autoimmune diseases, commonly SLE (secondary APS). APS also develops life-threatening multiorgan thrombosis, called catastrophic APS (CAPS). In addition, the presence of aPL can lead to a wide spectrum of clinical findings other than thrombosis, such as thrombocytopenia and hemolytic anemia, and neurological disorders like migraine, epilepsy, and chorea may precede the thrombotic signs.

**Objectives:** The aim of this study was to highlight the broad spectrum of clinical manifestations of pediatric APS.

**Methods:** The medical records of the patients with APS and probable APS were evaluated retrospectively from January 2016 to December 2022.

**Results:** Among 6 patients (female:5, 83.3%), four patients who met the Sapporo criteria were diagnosed with pediatric APS (two primary APS), one with probable primary APS, and one with probable secondary APS. The median age at diagnosis was 14 years (8 months–17 years). Two patients were also diagnosed as having probable CAPS since three organ systems were involved. One of them was diagnosed concurrently, and the other was diagnosed with SLE later in the follow-up. The antiphospholipid antibody positivity was confirmed after 12 weeks in five patients (one patient died in the pediatric intensive care unit within a month). The triple aPL test positivity was detected in five patients. Four patients presented with vascular involvement, three with venous thrombosis, and one with both arterial and venous thrombosis. Three patients had thrombocytopenia, one had psychiatric complaints (SLE), and one had valvular heart disease at the time of the APS diagnosis. Four patients received low molecular weight heparin as long-term anticoagulation therapy and for thrombosis prophylaxis. Corticosteroids were given to 3 patients with SLE. SLE patients, who presented with severe manifestations, were treated with immunosuppressive drugs, such as rituximab (n = 1), cyclophosphamide (n = 1), or azathioprine (n = 1), along with corticosteroids. Four patients were treated with hydroxychloroquine. Two patients had recurrent thrombosis at follow-up, and two died during follow-up due to CAPS complications.

**Conclusion:** This study demonstrates that children with APS may present with a wide range of symptoms, from thrombocytopenia to CAPS.

**Patient Consent**

Yes, I received consent

**Disclosure of Interest**

None declared

**References**

1. Rumsey, D. G., et al. (2017). Diagnosis and treatment of antiphospholipid syndrome in childhood: A review. *Blood cells, molecules & diseases*, *67*, 34–40. 2- Nageswara Rao, A. A., et al. (2017). A retrospective review of pediatric antiphospholipid syndrome and thrombosis outcomes. *Blood coagulation & fibrinolysis:an international journal in haemostasis and thrombosis*, *28*(3), 205–210.

## P130 The frequency of hashimoto thyroiditis in patients with juvenile systemic lupus erythematosus

### E. Kılıç Könte^1^, H. Karakas^2^, K. Ucak^1^, A. Günalp^1^, F. Haslak^1^, E. Aslan^1^, G. Tarcin^2^, M. Yildiz^1^, H. Erdogan^2^, A. Adrovic^1^, K. Barut^1^, O. Evliyaoglu^2^, S. Sahin^1^, O. Kasapcopur^1^

#### ^1^Pediatric Rheumatology; ^2^Pediatric Endocrinology, Istanbul University, Cerrahpasa, Istanbul, Türkiye

##### **Correspondence:** E. Kılıç Könte

*Pediatric Rheumatology 2023*, **21(Suppl 2):**P130

**Introduction:** Juvenile onset systemic lupus erythematosus (jSLE) is a systemic autoimmune disease characterized by multi-organ involvement. Hashimoto's thyroiditis (HT) is a chronic inflammatory autoimmune disease. Higher rates of coexisting autoimmune diseases, including immune thrombocytopenia, autoimmune hepatitis and HT have been reported in patients with adult-onset SLE. Data from jSLE patients are limited.

**Objectives:** We aimed to compare the frequency of HT between patients with jSLE and healthy subjects. In addition familial aggregation of HT were assessed in both groups.

**Methods:** Demographic and clinical data of the jSLE patients including, age at disease onset and diagnosis, clinical manifestations, systemic lupus erythematosus disease activity index (SLEDAI-2K) scores (at diagnosis, every 6 months after diagnosis and at last visit) were recorded in a retrospective manner. Anti-thyroid peroxidase antibody (anti-TPO), anti-thyroglobulin (anti-TG), thyroid stimulating hormone(TSH), thyroxine(sT4) levels and family history of Hashimoto's thyroiditis were screened at the study visit both in patients with jSLE and in healthy subjects. Increased anti-TPO and/or anti-TG levels with impaired thyroid function tests were diagnosed as HT. Patients with increased TSH level without any autoantibody positivity was classified as subclinical hypothyroidism.

**Results:** Sixty-four jSLE patients and 56 healthy and age-matched controls have been enrolled to the study. Female subjects constituted the 81.2% (n=52) of the jSLE population. The age of the jSLE group at disease onset, diagnosis and at study visit were 11,6(±2,8), 12,1(±2,8) and 16,6(±4,4), respectively. The most common manifestation in patient group was malar rash (67,1%), followed by constitutional manifestations (50%), arthritis (48,4%), photosensitivity (35,9%), oral/nasal ulcers (31,2%), alopecia (20,3%), serositis (15,6%), neurological disease (9,3%) and autoimmune hepatitis (6,25%). The frequency of anti-dsDNA positivity and hypocomplementemia (C3 and/or C4) were 85,9% and 67,1%. The mean SLEDAI-2K score recorded cumulatively was 4,3 (±3,1), whereas the SLEADI-2K at last visit was 3,59(±3,8). There was no statistical significance for disease symptoms, laboratory parameters and SLEDAI-2K scores between patient groups with/without HT. Although the rate of HT was higher in juvenile SLE patients (n=9/64, 14.1%) compared to healthy controls (n=2/56, 3.6%); this was not statistically significant (p=0.06). In jSLE group, 7(10,9%) had a family history of HT, whereas only 1 patient (5.4%) in control group had a family member with HT.

**Conclusion:** Higher rates of Hashimoto's thyroiditis was observed in jSLE patients comparing to healthy children. Patients with jSLE patients should be screened for autoimmune tyhroid diseases as well as for other coexisting autoimmune diseases .

**Patient Consent**

Yes, I received consent

**Disclosure of Interest**

None declared

**References**

1. Duan L, Shi Y, Feng Y. Systemic lupus erythematosus and thyroid disease: a Mendelian randomization study. Clin Rheumatol. 2023.
2. Kumar K, Kole AK, Karmakar PS, Ghosh A. The spectrum of thyroid disorders in systemic lupus erythematosus. Rheumatology International. 2012;32:73-8.
3. Wu CH, Chen CA, Lin SH, Weng CT, Kuo PL, Shieh CC. Increased risk of early-onset childhood systemic lupus erythematosus for children born to affected parents: A nationwide child-parent cohort study. Front Immunol. 2022;13:966809.

## P131 Misdiagnosis of juvenile localized scleroderma in a child girl; case report

### A. A. Abushhaiwia^1^, N. B. Abushhiwa^2^

#### ^1^Pediatric Rheumatology; ^2^Pathology department , University of Tripoli, Faculty of Medicine , Tripoli , Libya

##### **Correspondence:** A. A. Abushhaiwia

*Pediatric Rheumatology 2023*, **21(Suppl 2):**P131

**Introduction:** LS is a rare chronic inflammatory or fibrosis of skin and underlying tissue without vascular or internal organ involvement associated with a high risk for morbidity in children.

**Objectives:** This case highlights a condition which many paediatricians are not familiar with localized scleroderma to recognize it. in order to improve patient care.

**Methods:** We describe clinical, laboratory,our experience and treatment response of scleroderma child

**Results:** A 7-year-old girl who came to our attention last November 2022 with progressive difficult walking, tiptoe walking, stiff hands and difficulty in lifting her fingers, limitation movement or contracture of her back & both upper and lower extremities . Her medical history was non-specific. Her symptoms first appeared one year ago (2021) with stiff of both hands, toes of feet, back , both upper and lower extremities.Physical examination revealed the stiffness of both hands and fixed flexion deformily of her fingers (PIPJ) were confirmed, and she had difficulty move her back and contractures of cervical, spine, elbows & ankles. With atrophic epidermis were documented on the dorsal part of the both hands on the both elbow as well as on the lateral ankle regions in both extremities, and both thighs respectively with hyper-pigmented lesions (morphea like lesion ) her parents had not paid significant attention to these lesions. Other examination results were non-specific.

At neurological clinic before she came to Rheuma clinic as considered her possibly she had metabolic error or multiple sclerosis MS, she was performed BM aspiration concluded no abnormal cells , hyper-cellular bone marrow with esinophilia&megakaryocytic hyperplasia,NCV & EMG, MRI brain &spine were within normal limits.WES test was performed was negative.Haematological examination showed leukocytosis 16.12 X10^3^,HGB 11.3gm\dl ,thrombocytosis 535 X10^3,^ neutrophilia 55% , moderate eosinophilia 16 %, ESR was elevated 55ml\hr , CRP was positive 9.8mg\dl and blood biochemistry,urine analysis all were normal.The rheumatoid factor was negative.ANA, antidsDAN ab, ENA all were negative.Electrocardiographic and echocardiography.No ocular uveitis was detected, respiratory function test, and high-resolution computed tomography results were also normal. No pathology was detected in the abdominal ultrasonography.Deep skin I,II biopsies was performed,which consistent with scleroderma .Therapy with systemic prednisolone oral 1mg\kg\day and methotrexate injection 15mg\m2\wk SC was initiated. During the 4-month follow-up visit after treatment initiation, the child reported no new lesions. a mild improvement of her movement and the brownish coloration of the lesion.

**Conclusion:** Recognition of this condition and early diagnosis is of outmost importance apart from a careful full skin examination.seeking histopathological confirmation of unclear cutaneous lesions via skin biopsy.as prompt initiation of treatment may be of greatest benefit in the early stages of the disease.

**Patient Consent**

Yes, I received consent

**Disclosure of Interest**

None declared

## P132 Tocilizumab reduces disease activity in jls patients with severe or refractory disease

### A. Aquilani, E. Marasco, F. De Benedetti, R. Nicolai

#### Rheumatology, Ospedale Pediatrico Bambino Gesù, IRCCS, Rome, Italy

##### **Correspondence:** E. Marasco

*Pediatric Rheumatology 2023*, **21(Suppl 2):**P132

**Introduction:** Systemic treatment with methotrexate (MTX) is recommended for juvenile localized scleroderma (JLS) when there is a risk for disability. Unfortunately, up to 6 to 10% of JLS cases are resistant to MTX. Biologic agents have been proposed as potential therapeutic options for refractory JLS. Recently, some case series reported the effectiveness of tocilizumab (TCZ) in these patients.

**Objectives:** The objective of our retrospective study is to evaluate the efficacy of tocilizumab in JLS patients resistant or intolerant to previous therapy (as second- or third-line therapy) and as first line in patients with severe disease.

**Methods:** We enrolled 10 patients with JLS (circumscribed, linear, pansclerotic and mixed scleroderma) followed at Bambino Gesù Children Hospital, who received TCZ for at least 2 years. The modified Localized Scleroderma Activity Index (mLoSSI) and the Localized Scleroderma Skin Damage Index (LoSDI), PGA-disease activity and PGA-damage were retrieved from patients’ charts and used to assess disease activity and damage, respectively.

**Results:** All patients were treated with MTX. Four patients with extensive skin involvement were started on a combination of TCZ + MTX as first line therapy. Six patients were started on TCZ as second- or third-line therapy (4 for refractory disease and 2 for MTX-related side effects), three of these patients had received Rituximab (RTX) before starting TCZ.

In 6 patients who started TCZ as second- or third-line therapy, we did not observe statistically significant differences for mLoSSI and PGA activity between baseline (pre-MTX) and pre-TCZ. Two patients showed a reduction in both mLoSSI and PGA activity with MTX treatment but they had to discontinue MTX due to side effects (high liver enzymes). The other four patients showed stable or worsening mLoSSI and PGA activity. One year after starting TCZ, all 10 patients showed a reduction in both mLoSSI and PGA activity; the improvement further progressed after 2 years of treatment. There was a statistically significant reduction in both mLoSSI (Friedman test p= 0.00013, Kendall W coefficient = 0.814) and PGA activity (Friedman test p= 0.00013, Kendall W coefficient = 0.814).

In 6 patients who started TCZ as second- or third-line therapy, we did not observe any difference in LoSDI and PGA damage between baseline (pre-MTX) and pre-TCZ. After starting TCZ, patients did not show a significant reduction in neither LoSDI nor PGA damage after 1 or 2 years of treatment. It is interesting to note that only 2 patients showed an almost complete resolution of the skin lesions.

**Conclusion:** In our cohort of patients, we showed the efficacy of TCZ in reducing disease activity and halting damage accrual. TCZ may represent a possible therapeutic option for patients with severe or refractory JLS.

**Patient Consent**

Yes, I received consent

**Disclosure of Interest**

None declared

## P133 Juvenile en coup de sabre morphea with rasmussen’s encephalitis-like syndrome – a case report

### M. Borkowski^1^, E. Smolewska^1^, M. Biernacka-Zielińska^1^, L. Przysło^2^, J. Roszkiewicz^1^, A. Wosiak^1^

#### ^1^Department of Pediatric Cardiology and Rheumatology; ^2^Department of Developmental Neurology and Epileptology, Medical University of Lodz, Łódź, Poland

##### **Correspondence:** M. Borkowski

*Pediatric Rheumatology 2023*, **21(Suppl 2):**P133

**Introduction:** Central nervous system complications in juvenile localized scleroderma, one of which is Rasmussen’s encephalitis, are variable in presentation and severity. The condition’s pathological ties to rheumatic disease are still underrepresented in literature.

**Objectives:** To describe the case of our patient, O. - a girl with juvenile localized scleroderma, *en coup de sabre* type, who developed a constellation of neurological signs reminiscent, but not fully satisfying the diagnostic criteria of Rasmussen’s encephalitis.

**Methods:** We provide descriptive data regarding the course of O.’s rheumatic disease and the neurological complications thereof, as well as the patient’s neuroimaging studies.

**Results:** The diagnosis of O.’s juvenile localized scleroderma was made at the age of eleven, after an *en coup de sabre* lesion formed on her chin. Treatment with methotrexate and methylprednisolone was started, to good tolerance and slowing of the progression of cutaneous disease. Later, at age fourteen, O. developed myoclonic seizures involving jaw musculature, which progressed into weekly tonic-clonic fits. As the girl’s epileptic events evolved, pharmacotherapy was initiated with short-lived success. In February 2022, O. suffered an episode of *epilepsia partialis continua* with left-sided myoclonus and ipsilateral spastic hemiparesis. A five month long hospitalization followed, during which O. received varied regimens of antiepileptic drugs, plus 10 plasmapheresis courses, with an irregular pattern of remissions and relapses. In EEG studies obtained across O.’s hospitalizations, there was a right hemispheric slowing with unilateral epileptic activity. Brain MRI revealed first a presence, then a progression of right-sided white matter hyperintensities in T2 and FLAIR sequences. The clinical presentation, as well as imaging and neurophysiological abnormalities were found to mimick Rasmussen’s encephalitis, without meeting the formal diagnostic criteria.

Given the unusual course of O.’s rheumatic disease, the girl was qualified for biological therapy. As of the time of writing this report, O. has received six doses of tocilizumab (anti-interleukin-6-receptor antibody) with good tolerance and clinical improvement. The disease activity monitoring will continue.

**Conclusion:** In spite of the well-established association between craniofacial morphea and epilepsy, little attention has been given to cases where the seizures have Rasmussen’s encephalitis-like features. Awareness of this complication may set a precedent for research regarding the intertwining pathophysiology of localized scleroderma and encephalitides*,* and underline the need for evidence-based guidelines for treatment of neurological complications of rheumatic disease.

**Patient Consent**

Yes, I received consent

**Disclosure of Interest**

None declared

**References**

1. Hixon et al. *Epilepsy in Parry-Romberg syndrome and linear scleroderma en coup de sabre: Case series and systematic review including 140 patients.* Epilepsy Behav. 2021 Aug;121(Pt A):108068
2. Paprocka et al. *Difficulties in differentiation of Parry-Romberg syndrome, unilateral facial sclerodermia, and Rasmussen syndrome.* Childs Nerv Syst. 2006 Apr;22(4):409-15

## P134 Effect of the janus kinase inhibitor tofacitinib in the treatment of juvenile scleroderma

### L. Colussi^1^, A. Dagri^2^, S. Pastore^3^, A. Taddio^1,3^, A. Tommasini^1,3^

#### ^1^University of Trieste , Trieste; ^2^University of Udine , Udine, ^3^Institute for Maternal and Child Health IRCCS "Burlo Garofolo", Trieste, Italy

##### **Correspondence:** L. Colussi

*Pediatric Rheumatology 2023*, **21(Suppl 2):**P134

**Introduction:** Juvenile scleroderma (JS) is a rare connective tissue disease, with different subtypes depending on the extent of the disease (skin, visceral organs) and the depth of the lesions. No drug has been shown to be of unequivocal benefit. Recently, literature reports of the use of JAK-inhibitors for scleroderma have been promising, but data mainly concern adult patients.[1]

**Objectives:** To evaluate the efficacy of the JAK inhibitor tofacitinib in controlling and preventing disease progression in JS.

**Methods:** The data of 3 JS patients treated with tofacitinib were retrospectively reviewed.

**Results:** All patients were females. Patient 1 was diagnosed with juvenile systemic sclerosis at the age of 13, for a history of Raynaud's phenomenon, changes in capillaroscopy, lung and skin involvement. Blood tests showed an antibody profile consistent with the diagnosis (hypergammaglobulinemia, highly positive ANA and anti-SCL70), increased indices of inflammation and interferon score. She was initially treated with prednisone, mycophenolate mofetile (MMF) and rituximab. However, the first infusion of the biological drug had to be interrupted due to an anaphylactoid reaction, so tofacitinib was started with progressive clinical improvement. After two years, therapy with JAK-inhibitor was stopped, with successive benefit. Patient 2 at the age of 11 presented with important joint stiffness of left hand and wrist associated with thickened and translucent overlying skin lesions. Nailfold capillaroscopy showed a reduction in the number of capillaries and tortuosity of the remaining vessels, while blood tests, pulmonary function tests and echocardiography were normal, so juvenile localized scleroderma was diagnosed. Nevertheless, the joint involvement was suspicious of a borderline systemic form of disease. Therefore, she received immediately treatment with prednisone, methotrexate (MTX) and tofacitinib for one year with noticeable clinical and radiological improvement. Then, after 8 months without therapy, MMF was introduced because of the occurrence of new scleroderma lesions. Due to progression of disease, the drug was substituted with tofacitinib, based on the clear benefit seen in previous course of treatment. Patient 3 presented at the age of 3 to our Center with a 2-year history of linear scleroderma of the right leg without evidence of systemic involvement. She was treated with corticosteroids and MTX with initial apparent benefit. Nevertheless, after one year, she showed face involvement suggestive of Parry Romberg syndrome, so tofacitinib was started. Clinical skin manifestations improved after 3 months of treatment.

**Conclusion:** In our small series, the use of tofacitinib was effective in blocking JS progression in medium term follow-up with a reduction of disease activity and tissue damage (LoSCAT score).

**Patient Consent**

Yes, I received consent

**Disclosure of Interest**

None declared

**Reference**

1. McGaugh S et al. Janus kinase inhibitors for treatment of morphea and systemic sclerosis: A literature review. *Dermatol Ther. 2022 Jun;35(6):e15437. doi: 10.1111/dth.15437. PMID: 35278019.*

## P135 Diffuse juvenile systemic sclerosis patients show distinct organ involvement, antibody pattern and have more severe disease in the largest jssc cohort of the world. results from the juvenile scleroderma inception cohort

### I. Foeldvari^1^, J. Klotsche^2^, K. Torok^3^, O. Kasapcopur^3^, A. Adrovic^3^, B. Feldman^3^, F. Sztajnbok^3^, M. T. Terreri^3^, A. P. Sakamoto^3^, S. Johnson^3^, J. Anton^3^, V. Stanevicha^3^, R. Khubchandani^3^, D. Schonenberg-Meinema^3^, E. Al-Abadi^3^, E. Alexeeva^3^, M. Katsicas ^3^, S. Sawhney^3^, V. Smith^3^, S. Appenzeller^3^, T. Avcin ^3^, M. Kostik^3^, T. Lehman^3^, H. Malcova^3^, E. Marrani^3^, C. Pain^3^, A. Patwardhan^3^, W.-A. Sifuentes-Giraldo^3^, N. Vasquez-Canizares^3^, P. Costa Reis^3^, M. Janarthanan ^3^, M. Moll^3^, D. Nemcova ^3^, M. J. Santos^3^, S. Abu Al-Saoud^3^, C. Battagliotti^3^, L. Berntson^3^, B. Bica^3^, J. Brunner^3^, D. Eleftheriou^3^, L. Harel^3^, G. Horneff^3^, D. Kaiser^3^, T. Kallinich^3^, D. Lazarevic^3^, K. Minden^3^, S. Nielsen^3^, F. Nuruzzaman^3^, S. Opsahl Hetlevik^3^, Y. Uziel ^3^, N. Helmus^1^

#### ^1^Hamburg Centre for Pediatric and Adolescence Rheumatology, Hamburg; ^2^German Rheumatism Research Center, Berlin; ^3^jSSc collaborative group, Hamburg, Germany

##### **Correspondence:** I. Foeldvari

*Pediatric Rheumatology 2023*, **21(Suppl 2):**P135

**Introduction:** Juvenile systemic sclerosis (jSSc) is an orphan disease with a prevalence of 3 in 1,000,000 children. In adult patients there are significant differences between clinical presentation of cutaneous diffuse (djSSc) and cutaneous limited phenotypes (ljSSc).

**Objectives:** To study and compare the clinical presentation of jSSc patients with djSSc and ljSSc subtypes at the time of inclusion in the cohort.

**Methods:** We reviewed the baseline clinical characteristics of the patients, who were recruited to the jSScC prior to April 2023. jSScC is a prospective cohort of jSSc patients, who developed the first non-Raynaud´s symptom before the age of 16 years and were under the age of 18 years at the time of inclusion.

**Results:** The jSScC included 238 patients, 69% had cutaneous diffuse subtype. The median age at onset of Raynaud’s phenomenon was 10.4 years and median age at first non-Raynaud’s symptom was 10.9 years. Median disease duration was 2.5 years. The female/male ratio was not significantly lower in the djSSc subtype (3.3:1 vs 4.7:1). Antibody profile was similar, with the exception of a significantly higher number of anticentromere positive patients in the ljSSc (10% vs 2%, p=0.019). Patients with djSSc had significantly higher modified Rodnan Skin Score (17 vs 4, p=0.011), more frequently sclerodactyly (85% vs 56%, p<0.001), Gottron papules (31% vs 15%, p=0.008), a history of digital ulceration (61% vs 29%, p<0.001), active ulceration (20% vs 8%, p=0.024), telangiectasia (45% vs 22%, p=0.001), a decreased Body Mass Index z score < -2 (19% vs 6%, p=0.010) and decreased joint range of motion (64% vs 47%, p=0.017). Patients with ljSSc had significantly higher rate of cardiac involvement (11% vs 2%, p=0.006). There was no difference between the groups regarding pulmonary and renal involvement, gastrointestinal involvement beside decreased BMI < -2 z score; and muscle weakness.

Regarding patient related outcomes assessed by visual analogue scales (0 to 100), djSSc patients had more severe disease related to patient reported global disease activity (40 vs 30, p=0.041), patient reported global disease damage (40 vs 25, p=0.001), and patient reported Raynaud activity by (30 vs 18, p=0.025). Additionally, physician related outcomes assessed by visual analogue scales, the physician reported global disease activity (35 vs 20, p=0.034), and physician reported global disease damage (30 vs 20, p=0.011), were significantly higher in djSSc patients.

**Conclusion:** In the largest jSSc cohort in the world, djSSc patients have significantly more severe disease according to patient and physician related outcomes than ljSSc patients. Patients with djSSc also had more cutaneous, vascular, and musculoskeletal involvements and patients with ljSSc had more cardiac involvement. Interestingly, we found no significant differences regarding interstitial lung disease, pulmonary hypertension or gastrointestinal involvement, although the number of patients with decreased BMI ≤-2 z score was significantly higher in the djSSc patients.

This project was supported by an unrestricted grant from „Joachim Herz Stiftung“

**Patient Consent**

Not applicable (there are no patient data)

**Disclosure of Interest**

None declared

## P136 The pattern of medication use significantly changed over 36 months observation period. result from the juvenile scleroderma inception cohort

### I. Foeldvari^1^, J. Klotsche^2^, O. Kasapcopur^3^, A. Adrovic^3^, K. Torok^3^, B. Feldman^3^, M. T. Terreri^3^, A. P. Sakamoto^3^, J. Anton^3^, S. Appenzeller^3^, E. Marrani^3^, T. Avcin ^3^, D. Nemcova ^3^, M. J. Santos^3^, F. Sztajnbok^3^, L. Berntson^3^, J. Brunner^3^, G. Horneff^3^, S. Johnson^3^, T. Kallinich^3^, M. Kostik^3^, F. Nuruzzaman^3^, A. Patwardhan^3^, N. Helmus^1^

#### ^1^Hamburg Centre for Pediatric and Adolescence Rheumatology, Hamburg; ^2^German Rheumatism Research Center, Berlin; ^3^jSSc collaborative group, Hamburg, Germany

##### **Correspondence:** I. Foeldvari

*Pediatric Rheumatology 2023*, **21(Suppl 2):**P136

**Introduction:** Juvenile systemic sclerosis (jSSc) is an orphan disease with a prevalence of 3 in 1,000,000 children. Currently no medications are licensed for the treatment of jSSc. Due to its rarity, only recently the first management and treatment guidelines have been published, the jSSc SHARE (Single Hub and Access point for paediatric Rheumatology in Europe) recommendations, reflecting consensus opinion upon pediatric rheumatologists(1). We reviewed the applied medication in the treatment of the patients in the juvenile systemic scleroderma inception cohort (jSScC) up to April 2023.

**Objectives:** To better understand the international practices of treatment for jSSc, both at baseline and over 36 months observation period and to compare if real world therapies are congruent with the recent SHARE recommendations.

**Methods:** We reviewed the change of the applied medication in the treatment of jSSc patients over 36 months in the jSScC. The frequency of medications was calculated across the cohort at timepoint 0 (enrollment), and 36 months. jSScC is a prospective cohort of jSSc patients, who developed the first non-Raynaud´s symptom before the age of 16 years and are under the age of 18 years at the time of inclusion.

**Results:** We extracted data from 71 patients from the jSScC who were followed for 36 months, 75% had diffuse subtype. At the time of inclusion in the cohort the median disease duration was 2.4 years, median age of the first non-Raynaud symptom was 10.3 years. We captured the recorded medications at 0 months and 36 months. 64/71(90%) received any kind of Disease modifying drug (DMARD).

The glucocorticoid use decreased from month 0 to 36 months from 56% to 31% (p=0.004). The methotrexate use decreased from 53% to 25% (p=0.001), in opposite the mycophenolate use increased from 22% to 67% (p<0.001). The cyclophosphamide use decreased from 14% to 0% (p=0.002). Tocilizumab use increased from 0% to 17% (p=0.001). All other medication use showed no significant changes. Endothelin receptor antagonist was used in 17% patients at time point 0 and 22% at 36 months. PDE-5 blocker use increased from 3% to 9%.

**Conclusion:** At baseline half of the patients were on glucocorticoids. This is more frequent than typical adult SSc practice but coincides with jSSc SHARE treatment recommendations(#1)(1). After 36 months observation in the cohort over 90% of patients received a DMARD therapy. Methotrexate and mycophenolate mofetil were the most commonly prescribed DMARDs, which also reflects the SHARE treatment recommendations(#2, #3). At 36 months the use of glucocorticoids, methotrexate and cyclophosphamide decreased, and the use of mycophenolate and tocilizumab increased. In general, biological DMARDs are typically considered in severe or refractory disease (SHARE recommendation #7), reflecting the lower percentage compared to csDMARDs. Endothelial receptor antagonists, such as bosentan, were used over time in approximately 20% of the patients, reflecting SHARE recommendation #6 for pulmonary hypertension and/or digital tip ulcers. This is the first evaluation looking at clinical medication practice pattern in jSSc over 36 months, and its comparison to recently published consensus guidelines.

This project was supported by an unrestricted grant from „Joachim Herz Stiftung“

**Trial registration identifying number:** 1. Foeldvari I, Culpo R, Sperotto F, Anton J, Avcin T, Baildam E, et al. Consensus-based recommendations for the management of juvenile systemic sclerosis. Rheumatology (Oxford). 2021;60(4):1651-8.

**Patient Consent**

Not applicable (there are no patient data)

**Disclosure of Interest**

None declared

## P137 Anti-topoisomerase positivity is not associated with baseline organ severity in juvenile systemic sclerosis

### I. Foeldvari^1^, J. Klotsche^2^, K. Torok^3^, O. Kasapcopur^3^, A. Adrovic^3^, B. Feldman^3^, F. Sztajnbok^3^, M. T. Terreri^3^, A. P. Sakamoto^3^, S. Johnson^3^, J. Anton^3^, V. Stanevicha^3^, R. Khubchandani^3^, D. Schonenberg-Meinema^3^, E. Alexeeva^3^, M. Katsicas ^3^, S. Sawhney^3^, V. Smith^3^, E. Al-Abadi^3^, S. Appenzeller^3^, T. Avcin ^3^, M. Kostik^3^, T. Lehman^3^, H. Malcova^3^, E. Marrani^3^, A. Patwardhan^3^, W.-A. Sifuentes-Giraldo^3^, N. Vasquez-Canizares^3^, P. Costa Reis^3^, M. Janarthanan ^3^, D. Nemcova ^3^, C. Pain^3^, M. J. Santos^3^, S. Abu Al-Saoud^3^, C. Battagliotti^3^, L. Berntson^3^, B. Bica^3^, J. Brunner^3^, L. Harel^3^, G. Horneff^3^, D. Kaiser^3^, T. Kallinich^3^, D. Lazarevic^3^, K. Minden^3^, M. Moll^3^, S. Nielsen^3^, F. Nuruzzaman^3^, S. Opsahl Hetlevik^3^, Y. Uziel ^3^, N. Helmus^1^

#### ^1^Hamburg Centre for Pediatric and Adolescence Rheumatology, Hamburg; ^2^German Rheumatism Research Center, Berlin; ^3^jSSc collaborative group, Hamburg, Germany

##### **Correspondence:** I. Foeldvari

*Pediatric Rheumatology 2023*, **21(Suppl 2):**P137

**Introduction:** Juvenile systemic sclerosis (jSSc) is an orphan disease with a prevalence of 3 in 1 000 000 children. In adult patients anti-topoisomerase (anti-Scl70) positivity is a risk factor for diffuse cutaneous subtype and increased rate of interstitial lung disease (ILD), but data in children are scarce. Juvenile systemic scleroderma inception cohort (jSScC) is a prospective cohort of jSSc patients, who developed the first non-Raynaud´s symptom before the age of 16 years and were under the age of 18 years at the time of inclusion.

**Objectives:** To study and compare clinical presentation of jSSc patients, who are anti-Scl-70 positive or negative at the time of inclusion in the (jSScC).

**Methods:** We reviewed the baseline clinical characteristics of the patients, who were recruited to the jSScC prior to the 30th of April 2023 and compared their clinical characteristics based on anti-Scl 70 antibody positivity.

**Results:** 225 patients with jSSc were included in the cohort, 33% had anti-Scl70 antibodies (n=74). 68% (n=155) of patients had diffuse cutaneous subtype. There were no significant differences regarding diffuse subtype in the group with or without Scl70 antibodies (77% vs 65%; p=0.5). The median age at onset of Raynaud phenomenon was similar between two groups (10.3 years [7.6–12.8] in the anti-Scl70+ group and 10.6 years [7.2-13.1] in the anti Scl70 negative group). At the time of inclusion, median disease duration was 2.4 years and 2.5 years, respectively. The female/male ratio did not differ significantly. There were no differences between groups regarding organ involvement, including cutaneous, vascular, cardiac, renal, gastrointestinal and musculoskeletal involvement, sicca symptoms, interstitial lung disease or pulmonary hypertension. The only differences found were in the patient reported outcomes, namely in the patient global disease damage (p=0.024) and the reported Raynaud´s activity (p=0.019), which were significantly higher in the anti-Scl 70+ patients.

**Conclusion:** This is an intriguing finding that reinforces our previous published results of 80 jSSc patients, where the differences were not significant between anti Scl70 positive and negative patients^1^. In this study, there were differences only in the patient reported global disease damage and the patient reported Raynaud´s activity.

This project was supported by an unrestricted grant from „Joachim Herz Stiftung“

**Patient Consent**

Not applicable (there are no patient data)

**Disclosure of Interest**

None declared

**Reference**

1. Foeldvari I, Klotsche J, Torok KS, et al. CHARACTERISTICS OF THE FIRST 80 PATIENTS AT TIMEPOINT OF FIRST ASSESSMENT INCLUDED IN THE JUVENILE SYSTEMIC SCLEROSIS INCEPTION COHORT. WWW.JUVENILESCLERODERMA.COM. Journal of Scleroderma and Related Disorders 2018;4(1-13). DOI: 10.1177/2397198318790494.

## P138 improvement across organ system, physician and patient reported outcome measures over a 36-time period in the juvenile systemic scleroderma inception cohort

### I. Foeldvari^1^, J. Klotsche^2^, O. Kasapcopur^3^, A. Adrovic^3^, K. Torok^3^, B. Feldman^3^, M. T. Terreri^3^, A. P. Sakamoto^3^, J. Anton^3^, S. Appenzeller^3^, E. Marrani^3^, T. Avcin ^3^, M. Katsicas ^3^, D. Nemcova ^3^, M. J. Santos^3^, F. Sztajnbok^3^, L. Berntson^3^, J. Brunner^3^, G. Horneff^3^, S. Johnson^3^, T. Kallinich^3^, M. Kostik^3^, K. Minden^3^, F. Nuruzzaman^3^, A. Patwardhan^3^, N. Helmus^1^

#### ^1^Hamburg Centre for Pediatric and Adolescence Rheumatology, Hamburg; ^2^German Rheumatism Research Center, Berlin; ^3^jSSc Collaborative Group, Hamburg, Germany

##### **Correspondence:** I. Foeldvari

*Pediatric Rheumatology 2023*, **21(Suppl 2):**P138

**Introduction:** Juvenile systemic sclerosis (jSSc) is an orphan disease with a prevalence of 3 in 1, 000, 000 children. The Juvenile Systemic Scleroderma Inception cohort (jSScC) is the largest cohort of jSSc patients in the world. The jSScC collects longitudinal data prospectively in jSSc, allowing the evaluation of the development of organ involvement and patients and physician reported outcomes in jSSc over time.

**Objectives:** To review changes in clinical characteristics and patient and physician reported outcomes over the 36 months observation period since enrollment in the cohort.

**Methods:** The jSScC enrolls jSSc patients who developed the first non-Raynaud´s symptom before the age of 16 years and are under the age of 18 years at the time of inclusion. We reviewed jSScC patient clinical data and patient and physician reported outcomes of those with 36 months follow up from the time of inclusion until 1^st^ of April 2023.

**Results:** We could extract data of 74 patients, 74% with diffuse cutaneous subtype. The female/male ratio was 3.6:1. 89% of the patients were Caucasian. Median age of onset of Raynaud symptom was 9.3 years and the median age of onset of non-Raynaud symptom was 10.3 years. Median disease duration was 2.3 years at the time of inclusion in the cohort (T0). Ninety percent of the patients were treated with disease modifying anti-rheumatic drugs at T0 and 90% after 36months (T36). Four clinical parameters improved significantly over time: the median modified Rodnan skin score decreased from 10 to 7 (p=0.041), the number of patients with swollen joints decreased from 16% to 4% (p=0.014), the number of patients with elevated CK value decreased from 25% to 9% (p=0.042) and the number of patients with muscle weakness decreased from 16% to 3% (p=0.009). All other organ involvement did not show any statistically significant change from T0 to T36.

Three of the four patient reported outcomes improved significantly from T0 to T36: patient reported disease activity (VAS 0 – 100) from 40 to 20 (p=0.014), patient reported disease damage (VAS 0 – 100) from 40 to 20 (p=0.005), patient reported Raynaud activity (VAS 0 – 100) from 20 to 10 (p=0.034). One of the three physician reported outcomes improved significantly: the physician global disease activity (VAS 0 – 100) from 30 to 15 (p=0.001).

**Conclusion:** Skin and musculoskeletal clinical features improved significantly over 36 months. It is reassuring that major internal organ manifestations, such as cardiac, pulmonary and gastrointestinal were stable. No renal crisis occurred over the 36-month time period. The patient and physician-reported outcomes had the most positive impact over the 36 months period in this large international cohort.

This project was supported by an unrestricted grant from „Joachim Herz Stiftung”

**Patient Consent**

Not applicable (there are no patient data)

**Disclosure of Interest**

None declared

## P139 An updated overview of juvenile systemic scleroderma in a French cohort

### L. Jacquel^1,2^, R. Bechara^2^, J. Terzic^2^, A.-C. Rameau^3^, E. Chatelus^4^, L. Rossi^5^, I. Kone-Paut^5^, U. Meinzer^6^, I. Lemelle^7^, C. Rebelle^8^, D. Urbina^9^, P. Pillet^10^, P. Choquet^11^, J. El Maamari^12^, A. Zaloszyc^2^ on behalf of SOFREMIP

#### ^1^Department of Clinical Immunology and Internal Medicine; ^2^Department of Pediatrics, University Hospital of Strasbourg; ^3^Department of Pediatrics, Groupe Hospitalier de la région de Mulhouse et Sud Alsace; ^4^Department of Rheumatology, University Hospital of Strasbourg, Strasbourg; ^5^Department of Paediatric Rheumatology and CEREMAI, Hôpital de Bicêtre; ^6^Department of General Pediatrics, Hôpital Robert Debré, Paris; ^7^Department of Pediatrics, Nancy University Hospital, Nancy; ^8^Department of Pediatrics, Hôpital Marseille Saint Joseph; ^9^Department of Pediatrics, University Hospital of Marseille, Marseille; ^10^Department of Pediatrics, Pellegrin Hôpital des enfants, University Hospital of Bordeaux, Bordeaux; ^11^Department of Pediatrics, Hospital of Annecy Genevois, Annecy, France; ^12^Division of Pediatric Hematology / Oncology, BC Children's Hospital, Vancouver, Canada

##### **Correspondence:** L. Jacquel

*Pediatric Rheumatology 2023*, **21(Suppl 2):**P139

**Introduction:** Systemic scleroderma encompasses a range of disorders characterized by vascular and connective tissue abnormalities, leading to varying degrees of skin and internal organ inflammation and fibrosis. Although rare in pediatric patients, juvenile systemic scleroderma (JSSc) is a severe and life-threatening condition that significantly impacts children's development.

**Objectives:** This study aims to provide an overview of JSSc in children under 16 years old in France over the past decade.

**Methods:** Practitioners were contacted via email through the SOFREMIP organization (French Society for the Study of Pediatric Inflammatory Diseases) to identify potential participants based on their diagnosis of systemic sclerosis or scleromyositis. Clinical, epidemiological, and immunological data, as well as information on treatments, were collected through consultation and hospitalization records. The ACR/EULAR criteria, validated in adult systemic sclerosis, were evaluated at diagnosis for each patient to assess their sensitivity in our pediatric cohort.

**Results:** Our study included 18 patients from 8 different French centers. The median time to diagnosis after the onset of symptoms was 9 months. While our cohort exhibited a balanced distribution between localized and diffuse subsets of the disease, we observed a higher prevalence of the diffuse subset in children above the age of 10 (5 patients out of 6 included in our study). Skin induration was the most commonly reported symptom, while Raynaud's phenomenon was present in only 61% of the children at initial clinical evaluation. Digestive symptoms were encountered in 80% of cases and were typically present at the time of diagnosis. In contrast, respiratory involvement could arise during the follow-up period. Growth failure was highly prevalent in our cohort, occurring in more than half of the cases. All children tested positive for antinuclear antibodies, with anti-Scl70 being the most common specificity, even among children with limited cutaneous subsets. Interestingly, we found a high sensitivity of the ACR/EULAR criteria for diagnosing JSSc in our cohort with 83% of patients meeting these criteria, except for 3 children who presented with overlap syndromes. Despite the frequent use of corticosteroids at the onset, no deaths or renal crises were reported. Three patients received treatment with biological agents, specifically Rituximab and Tocilizumab.

**Conclusion:** JSSc is a rare yet severe disease that requires prompt and specialized multidisciplinary care. The ACR/EULAR criteria should be adopted as the standard for diagnosing systemic sclerosis in the pediatric population, as they demonstrate higher sensitivity than the pediatric criteria proposed in 2007. However, the diagnostic delay remains excessively long, which exposes children to irreversible organ damage. Further research is necessary to establish appropriate diagnostic criteria for overlap syndromes and assess the efficacy of biotherapies in children.

**Patient Consent**

Yes, I received consent

**Disclosure of Interest**

None declared

**References**

1. Martini G, Foeldvari I, Russo R, Cuttica R, Eberhard A, Ravelli A, et al. Systemic sclerosis in childhood: Clinical and immunologic features of 153 patients in an international database. Arthritis Rheum. 2006 Dec;54(12):3971–8.
2. Foeldvari I, Culpo R, Sperotto F, Anton J, Avcin T, Baildam E, et al. Consensus-based recommendations for the management of juvenile systemic sclerosis. Rheumatology. 2021 Apr 6;60(4):1651–8.

## P140 A multicentre study of clinical profile of Juvenile Localized Scleroderma (JLS) in indian children

### M. Janarthanan^1^, A. Khan^2^, A. Prabhudesai^2^, S. Balan^3^, S. Easwar^3^, A. Rao^4^, M. Agarwal^5^, M. Mamadapur^6^, S. Bhattad^7^, P. Pal^8^, S. R^9^, V. Viswanathan^10^, S. Guha^11^, J. Raghuram^12^, G. Subramaniam^13^, R. Khubchandani^2^

#### ^1^Rheumatology, Sri Ramachandra Medical College, Chennai; ^2^Pediatric Rheumatology, SRCC Children's Hospital, Mumbai; ^3^Rheumatology, Amrita Institute of Medical Sciences , Kochi; ^4^Pediatric Rheumatology, Manipal Hospital, Bengaluru; ^5^Pediatric Rheumatology, Sir Ganga Ram Hospital, Delhi; ^6^Rheumatology, Madras Medical College, Chennai; ^7^Pediatric Rheumatology, Aster CMI Hospital, Bengaluru; ^8^Pediatrics, Institute of Child Health, Kolkata; ^9^Rheumatology, JSS Medical College, Mysuru; ^10^Pediatric Rheumatology, Jupiter Hospital, Thane; ^11^Pediatrics, VIMS, Kolkata; ^12^Pediatrics, Aster Women & Children's Hospital, Bengaluru; ^13^Pediatrics, Private Clinic, Nagpur, India

##### **Correspondence:** M. Janarthanan

*Pediatric Rheumatology 2023*, **21(Suppl 2):**P140

**Introduction:** Localized scleroderma is a rare autoimmune disease characterized by skin thickening and excessive accumulation of collagen with fibrosis. Sequelae include cosmetic problems, joint contractures, limb length discrepancy, facial atrophy and extra dermal complications such as ocular, musculoskeletal or neurological.

**Objectives:** To describe the clinical profile, subtypes and management of JLS in an Indian cohort

**Methods:** The study was conducted by Pediatric Rheumatology Indian Collaborative Experience (PRICE) under the auspices of Pediatric Rheumatology Society of India. Retrospective data of JLS patients was collected online from 13 centres in a standardised questionnaire. This included sex, age of onset, current age, time to diagnosis, family history of connective tissue disorders, type of localized scleroderma, part/s of body involved, diagnostic methods, tests for autoimmunity, extra- cutaneous manifestations, treatment, follow up and complications.

**Results:** Data of 114 children from 13 centres was received. 6 patients were excluded from the study due to insufficient data. M: F ratio was 4:6. Disease onset occurred most commonly in the age range of 5 and 10 years. (55.5%). For 29/108 children the time to diagnosis was more than 2 years. There was no family history of connective tissue disorder in any of the patients. Linear scleroderma 40(37%), morphea (single small circumscribed lesion) 22(20.4%), mixed 19(17.6%), Encoup de sabre11(10.2%), Parry Romberg 9(8.3%) were the commonest types.The face 37 (34.3%), abdomen 31(28.7%), right leg 30(27.8%) were the commonest body parts involved. Clinical diagnosis of scleroderma was reached in 90(83.3%).Tests for autoimmunity were performed in 77(71.3%) and ANA was positive in 49(64.4%). Arthritis was the most common extra cutaneous manifestation in 9, followed by seizures in 3 and uveitis in 1 patient.Oral steroids were used in the treatment of 59(54.6%).Methotrexate 94(87.%) was the commonest DMARD used in treatment . Adherence to treatment was good in 87 (79.6%) and serial photos at every visit was the commonest monitoring tool 84(77.7%). Liver function abnormalities were noted in 13(12%). Surgical procedure was performed in 7(6.5%) which included tendon release in 5. Cosmetic complications were noted in 57(52.7%).Of the children who were followed up for at least a year, no further progression of lesions was noted in 49(45.4%) and reduction in size of lesions in 29(26.9%).

**Conclusion:** We present data of 108 children with localized scleroderma, the first multicentre study of this kind from India. Linear scleroderma was the commonest type with the face being the most common body part involved.Methotrexate followed by mycophenolate were the common DMARDS used in treatment. Arthritis was the most common extracutaneous manifestation.

**Patient Consent**

Yes, I received consent

**Disclosure of Interest**

None declared

## P141 Autoantibody profiling in juvenile systemic sclerosis may correlate with the clinical phenotype: our experience at chandigarh, India

### S. Machhua^1^, A. K. Jindal^1^, P. Nadig^1^, S. K. Sharma^2^, P. Barman^1^, S. Sharma^1^, M. Dhaliwal^1^, R. Pilania^1^, V. Pandiarajan^1^, D. Suri^1^, A. Rawat^1^, R. W. Minz^1^, S. Singh^1^

#### ^1^Advanced Pediatrics Center; ^2^Internal Medicine, PGIMER, Chandigarh, India

##### **Correspondence:** S. Machhua

*Pediatric Rheumatology 2023*, **21(Suppl 2):**P141

**Introduction:** Juvenile systemic sclerosis (jSSc) is a rare multisystem connective tissue disease with an approximate incidence of <1 per million children, characterised by widespread skin fibrosis and internal organ involvement. Less than 5% cases of systemic sclerosis (SSc) have their onset in childhood. In adults with SSc, a number of serum autoantibodies (autoAbs) have been described, many of which have been associated with clinical phenotypes. However, there is a paucity of literature on this aspect in jSSc.

**Objectives:** To analyse the autoAb profile and their clinical associations in patients with jSSc.

**Methods:** Patients diagnosed to have jSSc and fulfilling the Pediatric Rheumatology European Society (PReS)/ American College of Rheumatology (ACR)/ European League Against Rheumatism criteria (EULAR) classification criteria were enrolled from the Pediatric Rheumatology Clinic, Department of Pediatrics, Postgraduate Institute of Medical Education and Research, Chandigarh, India. ANA (Antinuclear antibody) was tested using indirect immunofluorescence on HEp-2 cells Nova Lite HEp-2 ANA (INOVA Diagnostics). Patients were further examined for the presence of 13 SSc associated autoAbs by a line immunoblot assay (Euroline Systemic Sclerosis (Nucleoli) Profile, (immunoglobulin G) kit, Euroimmun).

**Results:** In this study, 18 patients (9 Female, 9 Male; with mean age at disease onset 9.35 years and mean age at diagnosis: 10.5 years) were enrolled. All had positive ANA. Sixteen (88.8%) patients were positive for SSc associated autoAbs. AutoAbs to topoisomerase I (topo I), centromere A (CENP A) and centromere B (CENP B), RNA polymerase III (RP11, RP155), fibrillarin (U3RNP), nucleolus organizer region (NOR)-90, Th/To, PM-Scl75, PM-Scl100, Ku, platelet-derived growth factor receptor (PDGFR) and Ro-52, were observed in 9 (50%), 0, 1(5.5%), 1(5.5%), 1(5.5%), 2(11.1%), 1(5.5%), 2(11.1%), 1(5.5%), 2(11.1%), 2(11.1%), 0 and 4(22.2%), respectively. Topo I autoAb was the most common autoab. It was observed that Raynaud's phenomenon was significantly more common in patients with Topo I autoAb positivity (p < 0.04). No significant difference was found with interstitial lung disease, pulmonary arterial hypertension, and skin and gastrointestinal involvement. Mean ± SD modified Rodnan skin thickness score (mRSS) at the time of diagnosis was high in topo I positive jSSc patients (27.5±13.1) compared to jSSc patients without topo I positivity (18.6±10.5). Four patients were found to be positive for multiple autoAbs ranging from 2-7.

**Conclusion:** These findings suggest a potential role of topo I autoAb in the clinical manifestations of jSSc. In addition, 4 of our patients had monospecific PM-Scl100 autoAbs. These results suggest that although topo I autAb is the most clinically useful biomarker in jSSc, performing an extended SSc immunoblot will help identify those patients who are negative for the routinely tested autoAbs (topo I, CENP and RNAP autoAb), thereby potentially uncovering additional relevant disease markers (especially PM-Scl100 autoAb) and improving our understanding of the autoAb spectrum in jSSc.

**Patient Consent**

Yes, I received consent

**Disclosure of Interest**

None declared

## P142 Juvenile localized craniofacial scleroderma and central nervous system manifestations – experience of a paediatric rheumatology unit

### S. I. Almeida^1,2^, I. Madureira^1^, R. Silva^3^, C. Conceição^4^, M. P. Ramos^1^, M. Conde^1^

#### ^1^Paediatric Rheumatology Unit, Hospital Dona Estefânia, Centro Hospitalar Universitário Lisboa Central, Lisboa; ^2^Paediatric Department, Hospital Beatriz Ângelo, Loures; ^3^Paediatric Neurology Department; ^4^Neuroradiology Department, Hospital Dona Estefânia, Centro Hospitalar Universitário Lisboa Central, Lisboa, Portugal

##### **Correspondence:** I. Madureira

*Pediatric Rheumatology 2023*, **21(Suppl 2):**P142

**Introduction:** Juvenile localised craniofacial scleroderma (JLCS) is a rare chronic disease characterised by inflammation and fibrosis of the face and scalp. Linear lesions, called “*en coup de sabre*” (ECDS), affect the skin and subcutaneous tissue. Parry-Romberg syndrome (PRS) is a diffuse hemifacial atrophy involving the skin, soft tissues and bone, with minimal or absent skin fibrosis, that may occur isolated or associated with ECDS. Neurologic involvement is common (25-50%).

**Objectives:** Characterisation of patient demographics, neurological manifestations, laboratory and brain imaging findings, treatment and outcomes in children with ECDS/PRS.

**Methods:** Retrospective case series of children diagnosed with ECDS/PRS from 2008 to 2022. We identified patients with clinical manifestations or skin biopsies consistent with ECDS/PRS.

**Results:** Eight patients were identified (6 female) with a median age at diagnosis of 9 years (min 6–max 17) and a median time from disease onset of 32 months (min 5-max 37). Two patients were diagnosed with ECDS and PRS, 2 with PRS and 4 with ECDS. The median follow-up time was 7 years (min 1–max 13). Neurological manifestations were present in 4 patients (2 before the soft-tissue lesions - learning difficulties and seizures, and 2 after - migraine with aura and ischemic stroke); all these patients presented an abnormal neurological examination – paresis (4/4), hyperactive deep tendon reflexes (2/4) and language and memory disorders (1/4). EEG was abnormal in 3/4 patients, 2/4 with paroxysmal activity. MRI was performed in all patients, showing intracranial abnormalities in 3: white matter hyperintensities (2), micro-haemorrhages (1), vasculitis (1) and ischemic lesion (1). One patient with seizures displayed no MRI abnormalities. All patients with neuroradiological findings had neurological manifestations and performed a CSF examination showing oligoclonal bands in 3/3 and pleocytosis in 1/3. Laboratory results revealed elevated ESR (≥20mm/h) in 2/8, positive ANA (≥1:160) in 4/8 and other autoantibodies in 3/8. No association was found between autoimmunity and neurologic involvement (*p* = 0,48). All patients were treated with corticosteroids and methotrexate; 3 needed to repeat corticosteroids due to new neurological findings (2) and new skin lesion (1); 1 also started mycophenolate mofetil after progression of brain lesions on MRI. Two patients needed antiepileptic medications. At the last follow-up, 6 patients were in remission.

**Conclusion:** Patients with JLCS have a significant burden of CNS involvement. Neurological symptoms may precede soft-tissue manifestations, making the diagnosis exceptionally challenging. Consistent with previous studies, we also found in one patient a lack of neuroimaging abnormalities despite the presence of neurological symptoms. CSF examination can be of extreme importance for monitoring disease activity.

**Patient Consent**

Not applicable (there are no patient data)

**Disclosure of Interest**

None declared

**Reference**

1. Maloney E et al. The central nervous system manifestations of localized craniofacial scleroderma: a study of 10 cases and literature review. Pediatr Radiol. 2018 Oct;48(11):1642-1654.

## P143 Neurological involvement in pediatric localized scleroderma: a systematic review of the literature

### E. Marrani^1^, S. Soldovieri^1^, B. Castelli^1^, G. simonini^1^, I. Foeldvari^2^

#### ^1^Pediatric Rheumatology, AOU Meyer IRCCS, Firenze; ^2^Hamburger Zentrum für Kinder- und Jugendrheumatologie, hamburg, Italy

##### **Correspondence:** E. Marrani

*Pediatric Rheumatology 2023*, **21(Suppl 2):**P143

**Introduction:** Localized scleroderma (LS), also called morphea, is the most common form of scleroderma in pediatric age and it is associated to a worse prognosis, compared to the adult-onset disease. Extra-cutaneous manifestations are common in the pediatric population and associated with a more severe functional impairment.

**Objectives:** Aims of our study was to describe the neurological manifestations reported in pediatric scleroderma using a systematic review approach

**Methods:** A systematic literature review was performed in accordance with PRISMA guidelines using electronic bibliographic databases: MEDLINE via PubMed and EMBASE. Data obtained were extracted using a dedicated database containing clinical data that best categorize patient characteristics. Criteria for inclusion: studies including patients with a diagnosis of localized scleroderma with onset before 16 years of age and reporting a description of neurological manifestation

**Results:** The search returned a total of 2074 published till 30/06/2021; after selection process 359 patients with neurologic complications of JLS were found, extrapolated from 78 publications related to neurologic involvement.

Neurological involvement is significantly more frequent in patients with linear scleroderma of the face (En coup de sabre or Parry-Romberg syndrome, 216 out of 359) and is rarely observed in patients with other subtypes (8 generalized type, 11 isolated linear type of the trunk/extremities and 1 deep morphea).

Moreover, the left involvement of the face is predominant (n=35 right sided, n=47 left sided). Five patients have both right and left involvement (bilateral en coup de sabre).

The median age of disease onset was 6 years and the median age of neurological manifestations onset.

Headache was the most commonly reported symptom in 53% of patients with neurologic disease (n=190), migraine type described in n=35 children with side pain coincident with the side of cutaneous involvement.

The second neurological sign were seizures, founded in 29,5% (n=106), predominantly motor seizures, less commonly absence seizure. When stated, the age of onset of epilepsy appeared to be earlier than other neurological signs/symptoms.

Any type of neuroimaging sign was reported in 23,4% of patients (n=84), diagnosed by TC or MRI exams. They are described in patients with scleroderma of the face (n=42 ECDS, n=33 PRS, n=9 unknown), with left side cutaneous involvement 1.8 times more frequent than right one.

**Conclusion:** Neurological involvement is significantly more frequent in patients with linear scleroderma of the face, slightly more on the left side of the face compared to the right one. Neurological symptoms primarily include headache, followed by seizures. Many patients have neuroimaging abnormalities, described by TC or MRI exams, frequently related to the affected side of the face. Neuroimaging findings usually are associated to neurological symptoms, but in some cases patients with positive radiogram are asymptomatic.

A latency between onset of neurological manifestations and onset of cutaneous disease suggest the importance of a regular follow-up of these patients.

**Patient Consent**

Not applicable (there are no patient data)

**Disclosure of Interest**

None declared

## P144 Ocular manifestations of pediatric localized scleroderma: a systematic review of the literature

### E. Marrani^1^, S. Soldovieri^1^, B. Castelli^1^, G. simonini^1^, I. Foeldvari^2^

#### ^1^Pediatric Rheumatology, AOU Meyer IRCCS, Firenze; ^2^Hamburger Zentrum für Kinder- und Jugendrheumatologie, hamburg, Italy

##### **Correspondence:** E. Marrani

*Pediatric Rheumatology 2023*, **21(Suppl 2):**P144

**Introduction:** Localized scleroderma (LS), also called morphea, is the most common form of scleroderma in pediatric age and it is associated to a worse prognosis, compared to the adult-onset disease. If not promptly recognized, ocular manifestations might results in severe functional impairment

**Objectives:** Aims of our study was to describe the ocular manifestations reported in pediatric scleroderma using a systematic review approach

**Methods:** A systematic literature review was performed in accordance with PRISMA guidelines using electronic bibliographic databases: MEDLINE via PubMed and EMBASE. Data obtained were extracted using a dedicated database containing clinical data that best categorize patient characteristics. Criteria for inclusion: studies including patients with a diagnosis of localized scleroderma with onset before 18 years of age and reporting a description of extracutaneous manifestations.

**Results:** The search returned a total of 2074 published till 30/06/2021; after selection process 80 articles were included in the analysis. Any type of ocular sign was reported in 208 patients. Mean age at onset of cutaneous disease was 8 years, with almost all patients having concomitant diagnosis of ocular involvement.

The main manifestation included enophthalmos, present in 21% of children with ocular disease (n = 44), equally distributed between right and left eye; only 2 patients had bilateral involvement. All patients had JLS of the face (n = 32 patients with PRS, the others with ECDS). Twenty patients with enophthalmos had a central nervous system (CNS) involvement. Uveitis was the second most common ocular manifestation, described in 18% of patients with ocular disease (n = 37). Uveitis was more frequently anterior and unilateral The median age of disease onset among these patients was 5,5 years ANA positivity was described in 7 patients. N

Keratitis was diagnosticated in 6,7% of this group of patients (n = 14), more often band keratopathy. One patient had a specific diagnosis of Coats disease. In 8 patients it was surely associated with uveitis. Keratitis was usually associated to scleroderma of the face, more frequently to Parry Romberg subtype. Fifteen patients presented strabismus, more often from restricted eye motility, 3 from resorption of orbital bone, 1 from paralytic origin, Two patients complained of subsequent diplopia.

Other ocular manifestations less common were adnexal anomalies in 7,2% (n = 15), decreased reactivity to light in 4,8% (n = 10, 9 of which with CNS involvement), refractive errors in 4,3% (n = 9), iris anomalies in 3,3% (n = 7), orbital myositis in 2,4% (n = 5), lens anomalies (cataract) in 1,9% (n = 4).

**Conclusion:** Enophthalmos was the most frequent ocular alteration, followed by uveitis. Ocular findings were associated to JLS of the face in most of the cases, with females affected twice than males. Strabismus was a common finding, usually from restricted eye motility. Decreased pupillary reactivity to light was often observed. Many patients complained of reduced visual acuity. Adnexal anomalies were frequently described as well as refractive errors.

**Patient Consent**

Not applicable (there are no patient data)

**Disclosure of Interest**

None declared

## P145 Linear scleroderma of the head, face and rasmusen syndrome-case report

### B. Kołodziejczyk^1^, A. Gazda^1^, I. Naishtetik^1^, P. Gietka^1^, T. Kmieć^2^

#### ^1^Department of Rheumatology of the Developmental Age , Nationale Institute of Geriatrics, Rheumatology and Rehabilitation; ^2^Department of Neurology and Epileptology/Department of Neurology and Epileptology, The Children’s Memorial Health Institute, Warsaw, Poland

##### **Correspondence:** I. Naishtetik

*Pediatric Rheumatology 2023*, **21(Suppl 2):**P145

**Introduction:** In the course of saber-cut scleroderma ( ECDS) and Rarry-Romberg syndrome - subtypes of linear scleroderma of the head and face, neurological symptoms may occur, including epileptic seizures, and in Parry-Romberg Syndrome, progressive atrophy of the cerebral hemisphere on the side of facial atrophy, hemiparesis.

Rasmussen's encephalitis, chronic focal encephalitis (CFE) is a chronic, focal encephalitis, leading to progressive atrophy of one hemisphere of the brain. The clinical picture of the disease consists of epileptic seizures, cognitive decline and progressive hemiparesis.

**Objectives:** A 12-year-old boy diagnosed with linear scleroderma of the head and face and CFE.

The diagnosis of ECDS was established at the age of 6 (2016). Glucocorticosteroid (GCs) therapy - pulsed methylprednisolone and oral, methotrexate ( MTX) was used. The progression of scleroderma changes was observed during the reduction of GCs doses, in craniofacial MRI ( 2018) - features of hemifacial atrophy. Treatment was modified - cyclosporine A (CsA) instead of MTX and IVIG. Clinical improvement in scleroderma was obtained. In performed until 09. 2019. MRI of the brain - no abnormalities, EEG - normal.

At the age of 9 (11.2019), on CysA therapy, generalized epileptic seizures, concentration disorders, involuntary movements occurred, left-sided paresis, aphasia. In brain MRI- a focal lesion in the right frontal lobe, EEG - changes located in the right temporo-parietal area. Anti-epileptic treatment was applied, he also received IVIG, GCs pulses. Scleroderma therapy was modified - mycophenolate mofetil, tocilizumab, oral GCs, IVIG were used.

In the course of the diagnosis a number of diseases of the central nervous system were excluded.

**Methods:** Analysis of the patient's medical history. Literature review in pubmed, medline, embase on the co-occurrence of localized scleroderma and Rasmusen syndrome.

**Results:** At the age of 10 (2021), CFE was suspected, in brain MRI - focal lesion and cortico-subcortical atrophy of the right hemisphere of the brain. In 2021 right hemispherectomy was performed. In histopathological examination - lymphoid cell infiltration, the whole picture may correspond to CFE. Since the surgery- no epileptic seizures, left-sided paresis persists. Oral GCs and IVIG were continued in the treatment.

**Conclusion:** In the course of ECDS and Parry-Romberg syndrome there are neurological symptoms, these forms always require imaging studies of the central nervous system. However, the coexistence of local scleroderma with other neurological diseases should be taken into account, especially if the patient does not respond to scleroderma treatment. Differential diagnosis is difficult, both diagnosis and treatment require the cooperation of specialists in neurology, rheumatology, radiology.

**Patient Consent**

Yes, I received consent

**Disclosure of Interest**

None declared

## P146 Usefulness of machine learning classification algorithms for classification of raynaud's phenomenon in children

### M. Sestan^1,2^, D. Turudic^1,2^, M. Held^1^, N. Skreb^1^, M. Baresic^1,3^, N. Kifer^1,2^, M. Frkovic^1,2^, J. Stipic^4^, D. Milosevic^1^, M. Jelusic^1,2^

#### ^1^University of Zagreb School of Medicine; ^2^Department of Paediatrics; ^3^Department of Internal Medicine; ^4^Department of Neurology, University Hospital Centre Zagreb, Zagreb, Croatia

##### **Correspondence:** M. Sestan

*Pediatric Rheumatology 2023*, **21(Suppl 2):**P146

**Introduction:** Raynaud's phenomenon (RP) is often encountered in pediatric clinical practice. The most important question in patients with RP is whether it is primary or secondary. Currently, the only proposed imaging method for RP is nailfold capillaroscopy (NC). However, other additional imaging methods, such as computerized color telethermography (CCTT) may improve the correct classification of the patients with RP.

**Objectives:** Our goal was to establish the diagnostic accuracy of both imaging methods, NC and CCTT, in the early detection of RP.

**Methods:** We performed a cross-sectional retrospective study using unsupervised machine learning classification algorithms for the early diagnosis of RP.

**Results:** A total number of 232 children with symptoms of RP in the period from 2010 to 2021 were included in this study. The median (interquartile range) age of the patients was 15.21 (13.58-16.83) years, with a female-to-male ratio of 3.8:1. Using the data from medical history, clinical examination, laboratory parameters, NC, CCTT, 42.2% of the children were diagnosed with PRP, 21.6% with SRP, while in 36.2% we have no observed characteristic pattern of RP (symptomatic RP, SyRP). We used three JCHAIDStar algorithms, which generate decision trees, to classify with CCTT/NC values into PRP, SRP, and SyRP subgroups. The first algorithm uses CCTT in the first node. If CCTT was consistent with PRP, the patient is diagnosed as PRP. If CCTT was consistent with SRP, antinuclear antibodies (ANA) (2nd node) and extractable nuclear antigen (ENA) screen (3rd node) are used to distinguish PRP from SRP children. If CCTT was normal, the algorithm uses beta-2 glycoprotein antibodies (β2-GPI) and ENA to distinguish SRP from SyRP. Precision of JCHAIDStar algorithm by using LOOCV cross-validation after recall is high for all groups (PRP : SRP: SyRP = 95.7% : 89.4% : 89.1%) with ROC areas for all groups > 0.86. The second algorithm begins with NC. If NC findings were normal, the algorithm uses β2-GPI (2nd node) to distinguish PRP, SRP and SyRP. If NC was not normal, ANA (2nd node) and ENA (3rd node) are used to distinguish SRP from PRP. Precision of this algorithm was PRP : SRP: SyRP = 81.7% : 95.8% : 85.0% by using LOOCV cross-validation with ROC areas for all groups > 0.82. Finally, we have constructed the third algorithm that combines CCTT and NC. The CCTT findings have the highest priority in this algorithm. ENA (1st node), NC (2nd node) and IgG values (3rd node) are used to distinguish PRP from SRP, while NC was used to to distinguish SRP from SyRP. Precision was PRP : SRP: SyRP = 90.8% : 85.5% : 94.6%, with ROC areas > 0.9.

**Conclusion:** Both NC and CCTT have their place in the diagnosis of RP and are complementary to each other. Using unsupervised machine learning we have constructed simple algorithms that are applicable in classification of RP in routine clinical practice.

**Patient Consent**

Not applicable (there are no patient data)

**Disclosure of Interest**

None declared

## P147 Histopathological and molecular analysis in dermis and epidermis of patients withsystemic and localized scleroderma

### B. Sozeri, S. Turkmen

#### Pediatric Rheumatology, Health Sciences University, Istanbul, Türkiye

##### **Correspondence:** B. Sozeri

*Pediatric Rheumatology 2023*, **21(Suppl 2):**P147

**Introduction:** Scleroderma is a highly complex disorder in its clinical manifestations and pathogenesis. It has a wide range of clinical manifestations due to varying degrees of vasculopathy, autoimmunity, altered endothelium function, and abnormal fibrosis which are accused in the pathogenesis of the disease.

**Objectives:** The aim of this study is to shed light on the pathogenesis of the disease in the childhood via dermal immunohistochemical analysis of the cases.

**Methods:** A single-blind clinical trial is conducted with evaluation of the tissue samples obtained from patients. The samples are stained with PAS, hematoxylin and eosin, E-Cadherin, CTGF, Tunnel, and staining for TGF-β1 and evaluated by light microscopy. In addition both TGFB1 level and mRNA expression analyses in plasma and tissue samples from patients are performed. A total of 15 patients (systemic, n = 8 or localized; n = 7) were enrolled in the study. None of these patients has any medical condition other than skin disease.

**Results:** The mean age of the disease onset was 9.2 ± 1.2 years and the mean age of the time of the diagnosis was 15.3 ± 3.2 years. All patients with systemic sclerosis have antinuclear antibody (ANA) titer between 1/160-1/640. There is no ANA positivity in patients with localized scleroderma. A total of 22 tissue samples (15 diseased tissue, healthy tissue and 7) were examined. Clinically with different characteristics in the two subgroups of the histopathology examination was shown to be distinct from the tissue level.

**Conclusion:** TGF-B levels, which is playing a fundamental role in the pathogenesis of the disease are found in both plasma and skin have been shown to be locally high. This elevation was found particularly in patients with systemic scleroderma to be more pronounced. Also in patients with localized scleroderma,skin fibroblasts has been shown to limit the pathologic response. Treatment for this disease is still questionable and results obtained from studies will throw light on new treatment possibilities

**Patient Consent**

Yes, I received consent

**Disclosure of Interest**

None declared

## P148 Profile of children and adolescents diagnosed with juvenile localized scleroderma in a single university center of pediatric rheumatology

### F. Sztajnbok, P. R. Souza, F. C. Zonis, M. F. Rodrigues, A. R. Fonseca, J. L. Monteiro, I. M. Paz, M. R. Vasti, R. G. Almeida

#### ^1^Pediatric Rheumatology, Universidade Federal do Rio de Janeiro, Rio de Janeiro, Brazil

##### **Correspondence:** F. Sztajnbok

*Pediatric Rheumatology 2023*, **21(Suppl 2):**P148

**Introduction:** Localized scleroderma (LS) is more frequent in pediatric age than systemic scleroderma (SS). Although SS may be an important cause of morbimortality, especially when lungs are involved, LS is not life threatening, but may cause severe deformities and interfere with self esteem more often in adolescent patients.

**Objectives:** To describe clinical, epidemiological and therapeutic profile of children and adolescents diagnosed at a university hospital in Rio de Janeiro, Brazil.

**Methods:** A cross-sectional, descriptive and retrospective study including children and adolescents between 2 and 16 years diagnosed with localized scleroderma and followed up at a pediatric rheumatology outpatient clinic of a university hospital during 2022-2023. Diagnosis was based on typical skin thickening or skin biopsy.

**Results:** A total of seventeen patients were included. The female/male ratio was about 1,5:1. The time between the onset of symptoms and diagnosis was 2.8 years and the mean age at diagnosis was 7 years. The most frequent form of localized scleroderma was linear scleroderma, affecting 41% of patients, followed by mixed morphea (29.5%), circumscribed morphea (23.5%) and pansclerotic morphea (6%). The most affected anatomical sites were the lower limbs (88%), followed by the trunk (53%), upper limbs (35%), abdomen (23%) and head-neck (19%). Skin biopsy was performed in 75% of patients, showing scleroderma active inflammation in 83%. Laboratory alterations were observed in 25% of the patients, such as eosinophilia (12.5%), high acute phase reactants (12.5%) and antinuclear antibodies (25%). The mean treatment time was 32 months. Most patients received subcutaneous or oral methotrexate (75%). Topical Tacrolimus 0,03% were used in 43.7% of patients and methylprednisolone pulse therapy was prescribed for 25% of the patients. Remission was achieved in 81,8% of patients who received methotrexate with or without tacrolimus, and 75% of patients who received methotrexate combined with pulse therapy. Only 18.75% of patients had disease reactivation during treatment. Functional disability due to joint contractures was found in a patient with pansclerotic morphea.

**Conclusion:** Localized scleroderma is a disease with a limited course and good prognosis. Early diagnosis and treatment are important to reduce the risk of disabilities and deformities.

**Patient Consent**

Yes, I received consent

**Disclosure of Interest**

None declared

## P149 Systemic sclerosis sine scleroderma in children is more aggressive than in adults

### F. Tirelli^1^, E. Zanatta^2^, G. Martini^1^, M. Binda^2^, B. Moccaldi^2^, C. Giraudo^3^, A. Meneghel^1^, F. Zulian^1^

#### ^1^Rheumatology Unit, Department of Woman and Child Health, University Hospital of Padova; ^2^Division of Rheumatology; ^3^Unit of Advanced Clinical and Translational Imaging, Department of Medicine DIMED, University Hospital of Padova, Padova, Italy

##### **Correspondence:** F. Tirelli

*Pediatric Rheumatology 2023*, **21(Suppl 2):**P149

**Introduction:** Juvenile Systemic Sclerosis (JSSc) is a rare condition in childhood and its variety with no skin involvement, named *sine scleroderma* (ssSSc), is rare^1^.

**Objectives:** To compare clinical and laboratory features of pediatric ssSSc with adult patients.

**Methods:** Clinical and laboratory data of pediatric ssSSc, retrospectively retrieved from the Padua University Hospital electronic medical records (EMR), case reports from the literature as well as from a previous international registry on JSSc, were compared with a cohort of adult patients with ssSSc. Patients were defined as having ssSSc if they never had skin involvement but all the following features: (I) Raynaud’s Phenomenon (RP) or vascular equivalent (II) positive antinuclear antibodies (ANA) (III) intestinal dysmotility and/or interstitial lung disease (ILD) or pulmonary arterial hypertension (PAH) and/or cardiac or renal involvement, typical of scleroderma, (IV) no other defined connective tissue diseases^2.^

**Results:** Eighteen juvenile and 38 adult-onset ssSSc patients, with a mean disease duration of respectively 5.8 and 9.7 years, entered the study. The proportion of females was significantly lower in juvenile compared to adult ssSSc (38.9% vs 89.5%, p<0.0001). When compared to adults, juvenile ssSSc patients displayed less SSc – specific capillaroscopy abnormalities (68.8% vs 94.6%, p=0.02) while significantly higher respiratory (55.6% vs 23.7%, p=0.02) and cardiac involvement (50.0% vs 2.6%, p<0.0001). No difference between the two groups were noted in the frequency of RP (juvenile 88.9% vs adult 97.4%), pulmonary arterial hypertension, (16.7% vs 7.9%), gastrointestinal (61.1% vs 68.4%) and musculoskeletal manifestations (16.7% vs 18.4%). Digital ulcers (DU) were more frequent in juvenile ssSSc (35.3% vs 10.5%, p=0.054). The delay in diagnosis was high both in children (19.6 mo.) and in adults (13.9 mo.). The outcome was significantly worse in pediatric ssSSc as 6 patients (33%) deceased (no.3) or reached an end-stage organ failure (no.3) in comparison to no patient in the adult cohort. Anti-centromere (ACA) were significantly lower in children (20.0% vs 68.4%, p=0.001) while no difference was noted for anti-Scl70.

**Conclusion:** The higher cardiac and pulmonary involvement in juvenile ssSSc, which are known leading cause of mortality in SSc, suggest a more aggressive course than in adults. The high ACA positivity might suggest that adult ssSSc can evolve in limited cutaneous SSc, which has a more indolent course. The absence of skin changes explains the delay in diagnosis and underlines the need for a prompt internal organs evaluation in patients with RP, SSc-specific autoantibodies and typical capillaroscopy changes.

**Patient Consent**

Not applicable (there are no patient data)

**Disclosure of Interest**

None declared

**Reference**

1. Zulian F. et al Systemic sclerosis sine scleroderma in children. Rheumatology 2022. ^2^Poormoghim H. et al., Systemic sclerosis sine scleroderma: demographic, clinical, and serologic features and survival in forty-eight patients. Arthritis Rheum 2000.

## P150 Ultrasonographic evaluation of localized scleroderma in children

### S. Türkmen, B. Sözeri

#### Clinic of Pediatric Rheumatology, Health Sciences University, Ümraniye Training and Research Hospital, İstanbul, Türkiye

##### **Correspondence:** S. Türkmen

*Pediatric Rheumatology 2023*, **21(Suppl 2):**P150

**Introduction:** Localized scleroderma (LS) is a chronic, autoimmune disease characterized by inflammation and fibrosis in the dermis and subcutaneous tissue. In the inflammatory phase, skin lesions are usually seen as erythematous purple plaques, while in the late phase, white-yellow, waxy hard lesions are seen. However, there are often delays in diagnosis.

**Objectives:** In this study, the skin and subcutaneous tissues of patients with LS were evaluated ultrasonographically and compared with the morphological structure of intact skin.

**Methods:** In this study, children with LS who were followed up in the Health Sciences University Ümraniye Training and Research Hospital Pediatric Rheumatology clinic between June 2016 and February 2023 were evaluated. 21 patients were included in the study. The lesion area and the morphology of the intact dermis and subcutaneous tissue adjacent to the lesion of the patients were evaluated by gray scale (GS) ultrasonography with a high frequency (12-13 MHz) linear probe.

**Results:** This study was performed with 21 LS patients, including 4 plaque morphea, 16 linear scleroderma, 1 generalized morphea. 71.4% of the patients were girls (n=15). The median age at diagnosis was 8 years (IQR 3-15). The physical examination of the patients was repeated before the ultrasonographic evaluation. The lesions of the patients were classified as early and late stages according to the physical examination and ultrasonographic findings. When ultrasonographic findings of early-stage lesions were compared with intact tissue, dermal thickness was decreased in morphea tissue, while an increase was observed in subcutaneous tissue thickness and echogenicity. A decrease of thickness was found in dermal and subcutaneous tissue of morphea lesion when it was compared to the intact tissue in late-stage lesions.

**Conclusion:** Ultrasonographic evaluation of the skin and subcutaneous tissue may be useful in cases where there is doubt in the diagnosis of LS and in the evaluation of morphea activity. According to the studies, it is clear that there are some morphological changes in the lesion area compared to the intact tissue in the ultrasonographic evaluation of morphea lesions. While the lesion tissue layer appears thicker than the normal layer due to dermal edema in early-stage lesions, a decrease in dermal and subcutaneous tissue thickness is detected in late-stage atrophic lesions. However, in some late-stage lesions, an increase in thickness may also be observed due to advanced fibrosis. The advantages of ultrasonography are that it is non-invasive, safe, easily accessible and gives fast results. However, further studies with larger populations are needed to prove that changes in dermal and subcutaneous tissue thickness in morphea can be used in lesion staging.

**Patient Consent**

Yes, I received consent

**Disclosure of Interest**

None declared

**References**

1. Laverde-Saad A, Lopez-Negrete E, Roustan G, Alfagemeb F. Dermatologic ultrasound in the management of childhood linear morphea. *Dermatol Online J*. 2021;27(7):10.5070/D327754364. Published 2021 Jul 15. doi:10.5070/D327754364
2. Wortsman X, Wortsman J, Sazunic I, Carreño L. Activity assessment in morphea using color Doppler ultrasound. *J Am Acad Dermatol*. 2011;65(5):942-948. doi:10.1016/j.jaad.2010.08.027
3. Li SC, Liebling MS, Ramji FG, et al. Sonographic evaluation of pediatric localized scleroderma: preliminary disease assessment measures. *Pediatr Rheumatol Online J*. 2010;8:14. Published 2010 Apr 27. doi:10.1186/1546-0096-8-14

## P151 Kawasaki disease: clinical and biochemical characteristics and outcomes at tertiary hospital in the emirate of Abu Dhabi-10 year experience in a single tertiary centre

### M. Abdulrahman, J. Al Mistraihi, S. Al Jneibi, S. Al Remeithi, K. Mahmood

#### Sheikh Khalifa Medical City, Abu dhabi, United Arab Emirates

##### **Correspondence:** M. Abdulrahman

*Pediatric Rheumatology 2023*, **21(Suppl 2):**P151

**Introduction:** Kawasaki disease (KD) is an acute, self-limiting autoimmune vasculitis. It affects mainly children below 5 years , with higher incidence between 6 to 11 month. It has non-specific physical and lab findings and is diagnosed based on specific criteria. There is limited knowledge about KD demographic and biochemical characteristics in our region

**Objectives:** The aim of this study was to evaluate clinical and bio-chemical characteristics and outcomes of KD among children at SKMC Abu Dhabi, UAE. Also we investigated predictors for failure to standard therapy.

**Methods:** Retrospective chart review of all pediatric patients admitted with KD at our center during the period of January 2011 to January 2020

**Results:** Total of 102 patients identified, 41 (40%) were female and 61(60%) are male. The mean age at diagnosis was 3 years , Mean duration of illness at presentation was 6 days. Of all patients, 41 % (n= 42)had complete KD and 59% (n-60) were incomplete. Most common clinical presentations were oral mucous membrane changes 80.4%, bilateral non exudative conjunctivitis 79.4 % and polymorphous rash 68.6%. Average hemoglobin level was 96 g/dL (+/-32), WBC count was 15,000 and platelet count of 425,00 at presentation. Laboratory findings showed hypoalbuminemia (66%), elevated CRP (92%), transaminitis (56%) and only 16% of patients had sterile pyuria. Of the patients that developed complications most common cardiac complication was CAL (6%) and non-cardiac complications included hepatosplenomegaly (7%), osteomyelitis, MAS-like illness, gall bladder distention and cervical lymphadenitis (4%).

79% of the patient’s symptoms resolved with initial treatment with IVIG and aspirin while remaining 21% required a second dose of IVIG and glucocorticoid therapy.

Resistance to treatment (11%) was seen mainly among males (81%) and the age group of 6-10 years (45%)

**Conclusion:** Although majority of the results were not statistically significant due to small sample size. Our population was indifferent from those reported internationally, including our demographic data that it is more prevalent in males, had high CRP level, anemia, leukocytosis and thrombocytosis.

**Patient Consent**

Not applicable (there are no patient data)

Detailed references will be provided at a later time if required

**Disclosure of Interest**

None declared

**References**

1. Mehmet Gündüz, Yasemin Akın, Esra Çelik Kuzaytepe, Ayşe Karaaslan, Esra Çetinkaya Polatoğlu, Hüseyin Kıyak (2018). Clinical Evaluation of Children with Kawasaki Disease Hospitalized in Our Clinic: A Retrospective Study. *Southern Clinics of Istanbul Eurasia*.
2. Alexopoulos, A. Vekiou L. Lycopoulou ,A. Tavena , E. Lagona ,T. Kakourou (2012). Kawasaki disease in Greek children: a retrospective study. Journal of the European academy of dermatology and dermatology and venerology.
3. Gorczyca, D., Postępski, J., Olesińska, E. *et al.*The clinical profile of Kawasaki disease of children from three Polish centers: a retrospective study.

## P152 Bilateral giant coronary artery aneurysms in a 7-month-old infant girl with kawasaki disease

### A. A. Abushhaiwia^1^, S. sasi ^2^, M. E. Addala^3^, Y. AElfawires^4^

#### ^1^Pediatrics Rheumatology , ^2^Pediatrics cardiology , university of Tripoli, faculty of Medicine , Tripoli Children's Hospital , ^3^Pediatrics cardiology , ^4^Pediatrics Rheumatology , Tripoli Children's Hospital , Tripoli , Libya

##### **Correspondence:** A. A. Abushhaiwia

*Pediatric Rheumatology 2023*, **21(Suppl 2):**P152

**Introduction:** Kawasaki disease is an acute vasculitis that affects medium-sized arteries, particularly the coronary arteries that occurs mostly in infants and young children. It is more common among boys than girls. Aneurysms are more common in those patients in whom treatment is delayed or missed.The best of our knowledge, the giant coronary aneurysms detected in our patient are the largest ones ever described in our Kawasaki disease cases.

**Objectives:** Highlights the importance of timing to prevent coronary artery complication in treating KD

**Methods:** case report

**Results:** A 10th month -old Libyan infant girl born to non-consanguineous, healthy parents. Presented to paediatric Rheumatology clinic at age 7 months as Complete KD with giant left coronary aneurysm (8mm) at 36 day of illness since September 2022. Initially she was treated for bacterial meningitis at private clinic for 17 days then transferred to local public hospital for further evaluation where she had been admitted for another 14days .she was diagnosed with complete form KD at the 22 days of illness, based on her background history that revealed she had skin rash ,bilateral conjunctivitis,edema of both hands and feet ,peeling of her extremities and diaper area. Laboratory tests showed leukocytosis WBC 31X10 ^3^,HGB 11.7,plat 795 thrombocytosis, high elevated CRP 180 mg/dl, high elevated ESR 99, echocardiography discovered giant aneurysms in her coronary artery 8mm and given intravenous IVIG single dose once and aspirin 5 mg /kg/day thendischarged .At our paediatric Rheumatology clinic review 5 weeks after discharge, she was clinically well, a febrile and gaining weight.The echocardiogram was repeated and showed a progressive enlargement of both coronary aneurysms (bilateral dilated coronaries LCA 4mm with aneurysm 7mm,and RCA 3mm with aneurysm 5mm) on the day 36 days and her ESR was 40mm/HR but CRP negative. Treat her with an oral steroid 1 mg/kg plus kineret L1 Inhibitor SC 3 mg/kg SC daily,in addition to aspirin 5 mg\kg\day. She was to be treated for about a month without any changes to her echocardiography, but her CRP was negative. ESR was normal 8mm\hr, HGB 11,MCV, 78.5,Plat 540. CT angiography for coronary showed LC fusiform aneurysm measuring about 27mm in length and 9mm in diameter, no mural thrombosis, RCA shows ostio proximal aneurysmal dilatation measuring about 6mm in length and 3 mm in diameter, keeps her on aspirin oral 5 mg\kg daily,echocardiogram and ECG were made every a month, 2 months, 3 months interval periodically with the same previous findings without any progressive or regressive findings.

**Conclusion:** For prevention of CAA, current treatment guidelines advise to use IVIG before the 10th day of the illness. Unfortunately, IVIG was administered to our case on the 23rd day and between weeks of illness, due to delayed diagnosis and treatment. Early detection and treatment of KD could prevent cardiac consequences of Kawasaki disease.

**Patient Consent**

Yes, I received consent

**Disclosure of Interest**

None declared

## P153 A challenging case of polyarteritis nodosa presenting as stroke and severe myalgia in a young female child; first case report in Libya

### A. A. Abushhaiwia

#### Pediatric Rheumatology , University of Tripoli, Faculty of Medicine ,Tripoli Children's Hospital, Tripoli , Libya

##### **Correspondence:** A. A. Abushhaiwia

*Pediatric Rheumatology 2023*, **21(Suppl 2):**P153

**Introduction:** polyarteritis nodosa is a rare and systemic necrotising vasculitis in children of medium-sized vessels in children.We herein report the case of a child girl with PAN

**Objectives:** To describe a child girl with PAN and emphysis the importance of early diagnosis and timely treratment

**Methods:** A Case report

**Results:** A 10 year-old female child with history of long recurrent episodes of fever, myalgia ,arthralgia for 3 weeks without diagnosis, she had admissions in different institutions with the suspicion of malignancy or JIA .in September 2022 was referred to and admitted to the pediatric rheumatology 2 weeks later. with history of fever up to 38.5 ° c for 3 weeks, severe myalgia, arthritis both elbows,wrists, abdominal pain and neurological manifestations ( convulsion and headache ) .On physical examination, her had blood pressure was 122/92mmHg,HR(85 beats/min), and temperature 38.7°C.her heart sounds were regular without any cardiac murmurs or rubs, and her lungs were also clear on auscultation bilaterally. Her abdomen was flat and soft. she had tenderness all over her muscle and arthritis both elbows,wrists, and The laboratory values were as follows: WBC 36 X10^3^; Hb 12 g/dL; hematocrit 35.6%; platelets 787X10^3^;blood film leukocytosis with severe neutrophilia,CRP 235mg/dL,ESR 80mm\hr. CPK 28 U/L, LDH 216 U\L, S.ferritin was normal 118 ng/ml.Covid IgG was positive with high titer 1.62 (negative < 0.9AU\ml) but IgM was negative <0.03, D-dimer was slightly elevated 0.59 mg\l ( normal 0.00-0.50) .ASOTwas positive with high titer 1430 IU\ml. Urinalysis &urine protein/creatinine ratio results were normal.results for rheumatoid factor, ANA, (PR3-ANCA) &(MPO) ANCA were all negative.MRI brain imaging revealed bilateral parietoocciptal cortical ischemic changes, CT scan brain angiography was normal.EMG was normal.Nerve conduction study revealed compression neuropathy right ulnar nerve beyond the elbow|(cubital tunnel syndrome) of sever degree .CTscan chest abdomen revealed mild hepatomegaly.CT angiography of both upper &lower limbs were normal ,ECG,Echo both were were normal.During her admission she had purpuric erythematous was over her left foot .In accordance with previous findings, skin, joint , muscle symptoms and neurological involvement we found a diagnostic possibility of vasculitis (PAN) was suggested.she was treated with IVIG 2gm\kg once and MPD pulse therapies( 3 doses) followed by oral prednisolone 1mg\kg\day &MMF 500mg twice \day,both the clinical symptoms and laboratory values improved dramatically. She currently continues to take low-dose prednisolone and MMF.

**Conclusion:** The most appropriate treatments for PAN still remain to be determined.Treatment with mycophenolate mofetil (MMF) combined with glucocorticoids treatments were found to be very effective, and her symptoms improved dramatically within 3 months.

**Patient Consent**

Yes, I received consent

**Disclosure of Interest**

None declared

## P154 Isolated gastrointestinal manifestation in a child with microspcopic polyangiitis

### L. Alfaqih^1^, H. Aljehafy^2^, M. Nashawi^3^

#### ^1^Faculty of medicine, Taif universtiy, ^2^Pediatric , Taif children hospital, Taif, ^3^Pediatric , Faculty of medicine,king abdulaziz universty, Jeddah, Saudi Arabia

##### **Correspondence:** L. Alfaqih

*Pediatric Rheumatology 2023*, **21(Suppl 2):**P154

**Introduction:** Microscopic polyangiitis (MPA) is a necrotizing vasculitis with few or no immune deposits, predominantly affecting small vessels. Necrotizing arteritis involving small and medium arteries may be present. Necrotizing glomerulonephritis is very common. (1).

**Objectives:** We report a rare pediatric case of isolated gastrointestinal manifestations of MPA-associated vasculitis in a 10-year-old Saudi boy.

**Methods:** Case presentation.

**Results:** A 10-year-old boy was admitted to our hospital with a history of fever, nausea, vomiting, and severe right lower abdominal pain for five weeks. He is known to have bronchial asthma, diagnosed at the age of one year, and a long time in remission on medication.

On physical examination, the patient was febrile with normal blood pressure. Abdominal examination revealed generalized tenderness, more specifically, on the right lower abdomen. He had no rashes, no arthritis, or palpable lymph nodes.

Laboratory results revealed microcytic anemia, normal leukocytes, platelets, eosinophil count, and high inflammatory markers. He received multiple courses of antibiotics with no significant improvement. Tests for possible viral and bacterial infections were negative.

Abdominal ultrasound and chest x-ray revealed no abnormality. Abdominal CT with contrast was also normal without vascular changes. After exclusions of malignancy via clinical features and blood film, autoimmune disease was suspected. During the admission, he tested positive for COVID-19 rapid antigen test. Although he did not have upper airway symptoms, during this time, his liver enzyme and CRP increased.

ANA and anti-dsDNA results were negative. Perinuclear anti-neutrophil cytoplasmic antibody (pANCA) came positive. Furthermore, microscopic urinalysis revealed no microscopic hematuria or proteinuria.

Based on the clinical presentation and laboratory findings and after excluding possible causes. A diagnosis of Microscopic polyangiitis was considered. The patient was started on oral prednisolone and azathioprine and noticed an improvement in his condition.

**Conclusion:** According to our knowledge, this is the first pediatric case with isolated gastrointestinal associated with ANCA vasculitis. Due to the clinical features, anemia, high inflammatory markers, and the positivity of pANCA,The diagnosis of MPA vasculitis was considered. Regarding COVID-19 infection, we believe this to be an incidental finding, as the patient developed the fever and abdominal pain before the infection.

**Patient Consent**

Yes, I received consent

**Disclosure of Interest**

None declared

**Reference**

1. Jennette JC, Falk RJ, Bacon PA, Basu N, Cid MC, Ferrario F, et al. 2012 revised International Chapel Hill Consensus Conference Nomenclature of vasculitides. Arthritis Rheumatism. 2013;65(1):1-11. doi: 10.1002/art.37715.

## P155 Kawasaki disease presenting with gastrointestinal symptoms: a diagnostic challenge

### M. Arora, R. K. Pilania, A. Gummadi, A. Singh, S. K. Logananthan, A. K. Jindal, V. Pandiarajan, D. Suri, S. Singh

#### Pediatrics, Pgimer, Chandigarh, India

##### **Correspondence:** M. Arora

*Pediatric Rheumatology 2023*, **21(Suppl 2):**P155

**Introduction:** Kawasaki disease (KD) is the commonest childhood vasculitis. Gastrointestinal(GI) symptoms can occasionally be the forerunner of KD and may pose a diagnostic challenge to treating physicians. There is paucity of literature on GI presentations of KD.

**Objectives:** To evaluate children with KD who had predominant GI presentations

**Methods:** We analysed case records of all children with KD with GI presentations during the period January 1994-April 2021, who had presented to our pediatric Rheumatology Clinic. Diagnosis of KD was based on American Heart Association criteria

**Results:** From January 1994 to April 2021, we diagnosed 1078 children with KD. Of these, 24 (19 boys; 5 girls) had a GI presentation. All had GI symptoms during acute phase of disease. Median age at diagnosis was 3.5 years (range 4 weeks-13 years). Manifestations included acute gastroenteritis(n=8); blood in stools(n=3); upper gastrointestinal bleed due to duodenal ulcer(n=1); abdominal distension, vomiting, features suggestive of subacute intestinal obstruction, intussusception(n=2); colitis (n =3); mesenteric mass(n=1), ulcers in colon (n=1); gall bladder perforation(n=1); jaundice (n=7) and acute fulminant liver failure (n=1). Delays in diagnosis ranged from 7 days-4 weeks. Twenty children responded to single dose of IVIg (2g/Kg); 3 children required a second dose of IVIg, infliximab was given in one case. 2-D echocardiography examination revealed normal sized coronary arteries in 21 patients. One patient with acute fulminant liver failure had left main coronary artery(LMCA) aneurysm (2.8mm;+2.7z) and macrophage activation syndrome and succumbed to illness. Two patients with jaundice had multiple aneurysms (LMCA= 6.17 mm (+7.42); LAD=4.68mm(+6z); RCA= 7.5mm (+10.63Z)) and dilated right coronary artery (4.2 mm) respectively

**Conclusion:** None of the GI symptoms are part of the AHA criteria. GI presentation of KD is uncommon and may create diagnostic confusion for the treating physician. One must not overlook the common characteristic features and laboratory criteria in presence of such unusual findings because they can help in early diagnosis and management of KD, ultimately reducing morbidity.

**Patient Consent**

Not applicable (there are no patient data)

**Disclosure of Interest**

None declared

## P156 Childhood cogan syndrome: clinical manifestations, treatment, and outcome a multicenter study among pres members

### R. A. Bakry^1^, E. D. Batu^2^, S. Özen^3^, N. Martin^4^, M. Jelusic^5^, T. Hospach^6^, S. Hashed^7^, D. Rigante^8,9^, J. Sánchez-Manubens ^10,11,12^, S. M. Al-Mayouf^13,14^

#### ^1^Maternity and Children Specialized Hospital , Jeddah, Saudi Arabia; ^2^Department of Pediatrics, Division of Rheumatology, Hacettepe University; ^3^Department of Pediatric Rheumatology, Hacettepe Üniversitesi, Ankara, Türkiye; ^4^Department of Pediatric Rheumatology, Royal Hospital for Children, Glasgow, United Kingdom; ^5^Department of Paediatrics, University of Zagreb School of Medicine, Zagreb, Croatia; ^6^Department of Pediatric Rheumatology, Leiter Zentrum für Pädiatrische Rheumatologie am Klinikum Stuttgart, Stuttgart, Germany; ^7^Department of Pediatric Rheumatology, Tripoli Children Hospital, Tripoli, Libya; ^8^Fondazione Policlinico Universitario A. Gemelli IRCCS; ^9^Università Cattolica Sacro Cuore, Rome, Italy; ^10^Hospital Universitari Parc Taulí Sabadell; ^11^Institut d'Investigació i Innovació I3PT; ^12^Universitat Autònoma de Barcelona, Barcelona, Spain; ^13^King Faisal Specialist Hospital and Research Center; ^14^College of Medicine, Alfaisal University, Riyadh, Saudi Arabia

##### **Correspondence:** R. A. Bakry

*Pediatric Rheumatology 2023*, **21(Suppl 2):**P156

**Introduction:** Cogan syndrome is a rare multisystem autoimmune inflammatory disease that manifests as occular inflammation classically described as interstitial keratitis and vestibulo-auditory dysfunction. It is classified as a variable- vessel vasculitis with no predominant-sized vessel involved. Based on the presenting clinical manifestation, CS may or may not be typical.

**Objectives:** To report the spectrum and clinical manifestations, highlight the current treatment approaches, and highlight the long term of childhood CS.

**Methods:** *This is a multicenter retrospective cross-sectional study conducted on patients younger than 18 years of age who were diagnosed with or suspected of having CS*. An invitation for participation was sent to all PReS vasculitis working party members. We received clinical data from 9 different countries. The collected data comprised demographic and clinical findings and long-term outcome using the Pediatric Vasculitis Damage Index (PVDI).

**Results:** A total of 14 patients (57% male) with median age of onset of 11.2 (IQR 8.2-14) years. Two patients were suspected to have CS. The initial diagnosis was inaccurate in more than 70% with interval time untiill diagnosis 0.7 (IQR 0-1) years. There was no family history of CS or any history of vasculitis. The most frequent clinical manifestations were sensorineural hearing loss in 13 patients (78.5% with tinnitus and 57% with vertigo), followed by ocular signs which were reported in 12 patients (85%), including uveitis (85%), iritis (28%), interstitial keratitis (43%), and retinitis (14%). The most common systemic symptom was headache (9/14) followed by MSK manifestations in form of arthralgia in 50% and arthritis in 29% of patients. Osteitis was noted in 2 patients. Only 28% of the enrolled patient noted to have fever. One patient had pericarditis, myocarditis and aortic insufficiency. Abdominal pain and vomiting were noted in 36%. 50% of patients had elevated inflammatory markers. Four patients were found to have proteinuria. ANA was positive in 3 patients, while C-P ANCA were noted in one patient. 28.5% had an abnormal echocardiography findings and 14% had evidence of vasculitis in angiography. 93% of CS patients were treated with glucocorticoids as first-line with excellent response in 78.5%. All 14 patients received different types of immunosuppressants. Methotrexate was initiated in 11 patients with more than sixty percent improvement rate. Infliximab was used to treat 6 patients with >65% response. Combination of tocilizumab with mycophenolate mofetile or methotrexate succeeded to treat two CS patients. 3 patient received Cochlear implant. The PVDI showed sensorineural hearing loss in 71%, visual impairment in 14% and valvular heart disease in 21.5%. However, only one patient died due to disease or treatment complication.

**Conclusion:** This study presents the first international data on childhood CS. It shows a heterogeneous spectrum of clinical manifestations and the higher percentage of the atypical form of CS at the pediatric age group. This report intended to increase awareness of this disease among health care providers. Hopefully, this work will be the first step to propose a treatment strategy for childhood CS.

**Patient Consent**

Not applicable (there are no patient data)

**Disclosure of Interest**

None declared

## P157 Effect of treatment intensification with infliximab on moderate to giant coronary artery aneurysms in kawasaki disease with respect to it’s time of administration : a retrospective analysis

### J. N. Bathia, P. Pal

#### Pediatric Rheumatology, Institute of Child Health, Kolkata, India

##### **Correspondence:** J. N. Bathia

*Pediatric Rheumatology 2023*, **21(Suppl 2):**P157

**Introduction:** In Kawasaki Disease(KD) treatment intensification with corticosteroids or infliximab (IFX) has been proposed for children presenting with Coronary artery aneurysm (CAA) at diagnosis.There is paucity of data on the effect of IFX, after IVIG,on medium to large CAAs with respect to the time of administration of IFX

**Objectives:** To determine the duration to diminution in size of medium and large CAAs with respect to time of administration of IFX after Intravenous immunoglobulin (IVIG).

**Methods:** This is a retrospective analysis of data of children with KD with medium to large CAAs, who received IFX (after IVIG) between January 2016 to September 2022 at Institute of Child Health, Kolkata. Medium to large CAAs were present at diagnosis or developed on follow-up echoes post IVIG.The timing of IFX administration was classified as ≤3 days, 4 to 7 days, ≥8 days after the first dose of IVIG. The duration to response of CAA was noted in three categories reduction of CAA by atleast 1z score, reduction by 50% from baseline and complete reduction (CAA z score ≤2z)

**Results:** 219 children were diagnosed as Kawasaki disease. 28 (10.9%) patients had CAA at diagnosis or developed CAA after IVIG. 20 of these received IFX after IVIG. Other 8 received additional therapy, hence excluded.13 had medium CAA. All had complete regression of CAA with a median duration of 180 days.7 had large CAA of which 3 had complete regression of CAA over 2 years while 4 others had persistent CAA.

**Medium CAA**

**IFX ≤3 DAYS AFTER IVIG Total 7 patients,** Days to reduction by 1z from baseline (median days) 28 days; Days to reduction by 50% from baseline (median days) 42 days; Days to complete reduction (median days) 168 days

**IFX 4 TO 7 DAYS AFTER IVIG Total 4 patients,** Days to reduction by 1z from baseline (median days) 42 days; Days to reduction by 50% from baseline (median days) 42 days; Days to complete reduction (median days) 180 days

**IFX ≥8DAYS AFTER IVIG Total 2 patients,** Days to reduction by 1z from baseline (median days) 56 days; Days to reduction by 50% from baseline 90, 180 days respectively; Days to complete reduction (median days) 180 and 240 days respectively

**Large CAA**

**IFX ≤3 DAYS AFTER IVIG Total 2 patients,** Days to reduction by 1z from baseline 14 and 16 days respectively; Days to reduction by 50% from baseline 45 and 365 days; Days to complete reduction: In first patient complete reduction was seen by 365 days,second patient has medium CAA at 2 years

**IFX 4 TO 7 DAYS AFTER IVIG Total 3 patients,** Days to reduction by 1z from baseline 120, 45, 190 days respectively; Days to reduction by 50% from baseline 240, 180 and 270 days; Days to complete reduction first patient regressed by 730 days (2 yrs),second patient has 2.3z CAA at 4 years and third patient has medium CAA at 2 years

**IFX ≥8DAYS AFTER IVIG Total 2 patients,** Days to reduction by 1z from baseline 42 days in both’; Days to reduction by 50% from baseline 365 and 730 days respectively; Days to complete reduction: In first patient complete reduction was seen by 730 days,second patient has medium CAA at 4 years

**Conclusion:** From our cohort we noted therapy intensification with Infliximab results in regression of CAAs. Medium coronary artery aneurysms completely regressed even when Infliximab is administered late. Even few giant CAAs completely regressed with Infliximab following IVIG

**Patient Consent**

Yes, I received consent

**Disclosure of Interest**

None declared

## P158 Comparing apples to algorithms: application of adult Acr/Eular 2022 classification criteria for Anca-Associated Vasculitis (AAV) in pediatric patients

### E. S. Bosman, K. A. Morishita, D. A. Cabral on behalf of PedVas Investigators Network

#### Department of Pediatrics, University of British Columbia and BC Children’s Hospital, Vancouver, Canada

##### **Correspondence:** E. S. Bosman

*Pediatric Rheumatology 2023*, **21(Suppl 2):**P158

**Introduction:** The classification of rare childhood onset ANCA-associated vasculitis (AAV) continues to evolve. Granulomatosis with polyangiitis (GPA) or eosinophilic granulomatosis with polyangiitis (EGPA) were classified using the 1990 ACR criteria until 2008 when the pediatric specific EULAR/PRINTO/PRES (ped-EPP) criteria were developed for GPA. There have been no formal classification criteria for diagnosing microscopic polyangiitis (MPA) but patients have been classified using the European medical agency (EMA) algorithm, which was adapted for pediatrics (ped-EMA)^1^. In 2022, ACR/EULAR published new criteria for classifying GPA, MPA, and EGPA for adults. These criteria have not been validated for pediatric vasculitis patients.

**Objectives:** To assess the applicability of the ACR/EULAR criteria in children with small-medium vessel vasculitis using clinical data collected in A Registry for Childhood Vasculitis (ARChiVe).

**Methods:** All ARChiVe patients with a physician (MD) diagnosis of EGPA, GPA, MPA, unclassified AAV, or unclassified vasculitis were included in this study (n=448). Eligible patients were sequentially classified using time-of-diagnosis data according to the ped-EMA classification as follows: EGPA patients were first classified using either ACR 1990 or Lanham’s Criteria. Then, GPA patients were identified with the ped-EPP and ACR-1990 criteria; patients fulfilling only adult ACR-1990 criteria (n=15) were excluded from comparison analysis. Remaining patients who were ANCA positive or had ped-EMA defined surrogate markers for GPA were considered ‘unclassified AAV’. Finally, patients were classified as MPA using ped-EMA surrogate markers, or were unclassifiable vasculitis. In parallel, the ACR/EULAR criteria were applied to the same patient cohort to define EGPA, GPA, MPA or ‘no classification’. The outcome of classification using the ped-EMA versus the new ACR/EULAR classification criteria were compared against the MD diagnosis, and with each other.

**Results:** Out of 448 patients, 1.1%, 53.3%, and 25.7% were classified with the ECR/EULAR criteria as EGPA, GPA, and MPA, respectively. Although the ACR/EULAR criteria aim to be mutually exclusive, 25 pediatric patients simultaneously met classification criteria for both GPA and MPA (considered unclassifiable AAV). In only 52% (n=232) of the patients was MD diagnosis concordant with the classification made with both classification systems. Of the remaining patients, 51 (24%) had a MD diagnosis concordant with ped-EMA classification and 85 (40%) with the ACR/EULAR classification. The ACR/EULAR criteria, classified more patients to MPA (n=115) than either MD-diagnosis (n=70) or ped-EMA (n=68).

**Conclusion:** Comparing the new adult-derived ACR/EULAR classification to existing pediatric classification in a large cohort of pediatric AAV patients resulted in a 64% increase of MPA diagnoses; more diagnoses were concordant with MD diagnosis (72 vs 64 % respectively); and fewer patients were considered as unclassifiable AAV. The addition of formal weighted criteria for MPA favours the use of the new ACR/EULAR criteria in pediatrics. Which classification better defines underlying biology or clinical prognosis remains unclear.

**Patient Consent**

Yes, I received consent

**Disclosure of Interest**

None declared

**Reference**

1. Cabral DA, et al. Comparing Presenting Clinical Features in 48 Children With Microscopic Polyangiitis to 183 Children Who Have Granulomatosis With Polyangiitis (Wegener's): An ARChiVe Cohort Study. *Arthritis Rheumatol*. 2016;68(10):2514-2526. doi:10.1002/art.39729

## P159 Atypical cutaneous phenotype in a patient with late-diagnosed Kawasaki disease

### I. Burgos Berjillos^1^, M. Martí Masanet^1,2^, M. González Fernández^1,2^, L. Lacruz Pérez^1^, B. López Montesinos^1^, I. Calvo Penadés^1^

#### ^1^Pediatric Rheumatology, Hospital Universitari i Politècnic La Fe; ^2^Pediatric Rheumatology, La Fe Health Research Institute, Valencia, Spain

##### **Correspondence:** I. Burgos Berjillos

*Pediatric Rheumatology 2023*, **21(Suppl 2):**P159

**Introduction:** Kawasaki Disease (KD) patients may present with several types of skin lesions which can mimic a different disorder and that could make us move away from the diagnostic suspicion of KD.

**Objectives:** Describe the association of atypical skin lesions and delayed diagnosis in a patient with KD.

**Methods:** Case report

**Results:** We present a 3 month infant with intermittent 25-day fever, irritability, orange-pinkish crusted papules and vesicles on the skin (cheeks, upper and lower limbs), swollen hands and feet and pincer nails with hyperkeratosis. Blood tests showed thrombocytosis, neutrophilic leukocytosis and acute-phase reactants elevation. After antibiotic treatment, pulse methylprednisolone (2mg/kg/day) was started, with analytical and clinical improvement. The first diagnostic suspicion in her origin hospital was histiocytosis, so she was referred to our center, where a skin biopsy ruled out this entity.

The patient was readmitted due to fever recurrence and worsening of the general condition after steroid discontinuation and in the context of SARS-CoV-2 infection. She received another pulse methylprednisolone and was referred to our hospital. Given the atypical manifestations and evolution, we ruled out infectious diseases, hereditary autoinflammatory syndromes, hypovitaminosis, zinc deficiency and inborn errors of metabolism. We performed an echocardiography, showing medium coronary artery aneurisms (z-score: left coronary artery: 5,3; left anterior descending: 7; right coronary artery: 6,9). At that time the patient met incomplete KD criteria, so we started intravenous immunoglobulin (IVIG) (2g/kg), combined antiplatelet therapy and intravenous methylprednisolone (2 mg/kg/day). Given patient’s age, delayed diagnosis, presence of medium coronary aneurysms, persistence of clinical inflammatory signs and steroid therapy prior to the time the diagnosis was made, infliximab was added to the therapy. 6 days from the IVIG infusion, aneurysms reduced to small aneurysms, and before patient’s discharge the coronary aneurysms disappeared. Systemic inflammatory symptoms improved until their resolution. Skin lesions persisted, reappearing and changing from vesicles and papules to crusts, progressing independently to the systemic disease or the treatment. We found in literature that psoriasiform lesions and pincer nails are associated with KD, but dermatologist evaluation and a new biopsy dismissed psoriasiform lesions.

Skin lesions improved after 2 months of topical tacrolimus, and 7 months from the onset, only residual erythema remains, with no recurrence of active lesions nor systemic inflammation.

**Conclusion:** Atypical skin lesions in KD force to expand its differential diagnosis without losing focus of the importance of early diagnosis and treatment in the cardiologic KD prognosis.

**Patient Consent**

Yes, I received consent

**Disclosure of Interest**

None declared

## P160 Localised orbital granulomatosis with polyangiitis manifesting as an orbital pseudotumor in an adolescent girl

### S. Coyne, K. Gallagher, E. J. MacDermott, O. Killeen

#### National Centre for Paediatric Rheumatology, CHI at Crumlin Hospital, Dublin, Ireland

##### **Correspondence:** S. Coyne

*Pediatric Rheumatology 2023*, **21(Suppl 2):**P160

**Introduction:** Granulomatosis with polyangiitis (GPA) is an autoimmune vasculitis affecting small- to medium-sized blood vessels. Localised GPA has a female predominance and adolescence onset. Although GPA is very rare in children, it can be aggressive and needs urgent treatment (1).

**Objectives:** To describe a rare presentation of localised GPA in an adolescent girl.

**Methods:** An 11 year old Irish girl presented to paediatric ophthalmology with a 4 week history of left orbital swelling and proptosis. The swelling was acute in onset with no associated pain. There was no history of trauma. Initially she was treated with oral antibiotics by her GP for a presumed orbital cellulitis but with no improvement. The patient was otherwise well with no systemic symptoms. On examination, periorbital swelling and purple/red discolouration of eyelid was noted. She had no cranial nerve palsies and her systemic examination was otherwise unremarkable. The patient was started on oral prednisolone pending further investigations.

**Results:** A CT of the orbit confirmed an ill-defined soft tissue mass superior to the left orbit. An orbital biopsy was subsequently performed. No neoplastic features were present. She was subsequently referred to paediatric rheumatology. Blood tests showed mildly elevated ESR of 19mm/hr with a normal CRP of <5mg/dL. Anti-Myeloperoxidase antibodies (MPO) were positive (pANCA+, MPO 7.7U/ml (0 - 3.4U/ml) with a negative anti-proteinsase-3 (PR3) 0.7U (0 - 1.9U). Following discussion with pathology, examination of deeper levels revealed a loose granulomatous inflammatory pattern with scattered giant cells and no necrosis. Staining was not suggestive of IgG4 related disease. A full systemic workup including urine protein/creatinine ratio, abdominal ultrasound, spirometry, chest x-ray and echocardiogram were all normal.

**Conclusion:** On the basis of the clinical and pathological findings, a diagnosis of localised orbital granulomatosis with polyangiitis (GPA) was made. The patient is currently on mycophenolate mofetil. In childhood onset GPA, ENT is the most common site involved however ocular disease is not uncommon (2). Although PR3 antibodies are more often positive (69%), MPO antibodies can also be positive (21%) (2). Positive MPO antibodies prompted examination of deeper levels of biopsy revealing features that are most likely consistent with GPA. This case highlights prompt multi-specialty collaboration is key in achieving a favourable outcomes in GPA.

**Patient Consent**

Yes, I received consent

**Disclosure of Interest**

None declared

**References**

1. Peters JE, Gupta V, Saeed IT, Offiah C, Jawad ASM. Severe localised granulomatosis with polyangiitis (Wegener's granulomatosis) manifesting with extensive cranial nerve palsies and cranial diabetes insipidus: a case report and literature review. BMC Neurol. 2018;18(1):59.
2. Iudici M, Quartier P, Terrier B, Mouthon L, Guillevin L, Puéchal X. Childhood-onset granulomatosis with polyangiitis and microscopic polyangiitis: systematic review and meta-analysis. Orphanet J Rare Dis. 2016;11(1):141.

## P161 Recurrent or corticosteroid dependent leukocytoclastic vasculitis: which therapy?

### C. de Simone ^1^, F. Fedele ^1^, F. Orlando ^2^, L. Martemucci ^2^, M. Tardi^2^

#### ^1^Department of Translational Medical Science, University of Naples “Federico II” , Section of Pediatrics; ^2^Department of General and Emergency Pediatrics, Santobono-Pausilipon Children Hospital, Pediatric Rheumatology Unit, Naples , Italy

##### **Correspondence:** C. de Simone

*Pediatric Rheumatology 2023*, **21(Suppl 2):**P161

**Introduction**

Leukocytoclastic vasculitis (LCV) is the histopathological description of a small vessel vasculitis (SVV) affecting the skin and internal organs, characterized by neutrophilic infiltration and fibrinoid necrosis within and around the vessel wall with signs of leukocytoclasia.

According to the revised International Chapel Hill Consensus Conference (CHCC), histological LCV can be found in several conditions, such as ANCA-associated vasculitis, immune complex vasculitis, vasculitis associated with systemic diseases and vasculitis secondary to other causes, such as drugs, sepsis or cancer. The skin biopsy should be performed to confirm the diagnosis.

**Objectives**

To report the use of colchicine for treatment of children with recurrent or corticosteroid dependent LCV.

**Methods**

We report five patients with LCV diagnosed from 2021 to 2023, including three males and two females. The median age was 11 years (2.4-13.7). Four patients presented persistent vasculitis (over 12 weeks), one patient chronic steroid-dependent Henoch-Schonlein purpura (HSP). Clinical examination showed palpable purpura mainly involves the lower legs and the back, sometimes with a tendency to confluence, covering large areas of the skin. The HSP patient presented complications as rectal bleeding and orchitis. The other four patients presented only skin manifestations. In all 5 patients, laboratory tests did not show significant alterations: platelet count, coagulation screening, renal function and urinalysis, autoantibodies, complement fractions were all normal. All the patients underwent skin biopsy, showing neutrophilic infltration with disintegration of neutrophil nuclei into fragments or nuclear dust, fibrin deposition, within and around the vessel walls, and extravasated red blood cells, damaged endothelial cells. Only in one patient the biopsy showed also scattered eosinophils in the dermis without eosinophilia.

**Results**

Given the persistence and the recurrence of the symptoms, all five patients were treated with corticosteroids with response. Four patients presented recurrence of lesions after stopping steroids, the one HSP patient presented persistence of lesions during steroids treatment. Therefore, Colchicine (0.5–1 mg /day, according to patient’s age) was started, with resolution of vasculitis in maximum 2-3 weeks, without adverse events. One patient stopped Colchicine due to the will of the parents, with recurrence of lesions four days after the last administration. Then, the treatment was resumed with clinical response.

**Conclusion**

The use of Colchicine in LCV is reported in literature. Our patients presented resolution of skin lesions without adverse reaction. Therefore, Colchicine could be considered in early phase of recurrent vasculitis avoinding chronic use of steroids.

**Patient Consent**

Yes, I received consent

**Disclosure of Interest**

None declared

## P162 Idiopathic intracranial hypotension occurring in a 12 year old girl treated for behcet disease: case report and brief report of the literature

### C. Dumaine^1^, C. Lafay^1^, R. Tohme^2^, M. Elmaleh^3^, C. Titah^4^, A. M. Felix^1^

#### ^1^Rheumatology and Infectious Diseases, National Reference Centre for Rare Pediatric Inflammatory Rheumatisms and Systemic Autoimmune diseases (RAISE); ^2^Robert-Debré University Hospital, Assistance Publique-Hôpitaux de Paris; ^3^Radiology, Robert-Debré University Hospital, Assistance Publique-Hôpitaux de Paris; ^4^Ophtalmology, Adolph de Rothschild hospital, Paris, France

##### **Correspondence:** C. Dumaine

*Pediatric Rheumatology 2023*, **21(Suppl 2):**P162

**Introduction:** Behcet disease (BD) is a systemic inflammatory disease, with very rare pediatric onset, that can lead to severe ophthalmologic and neurologic complications.

**Objectives:** Our aim was to describe the clinical course and radiological findings of a 12 year old girl treated for Behcet disease presenting with intracranial hypotension, and to do a brief review of the literature.

**Methods:** Medical record were reviewed and we collected clinical, radiological, biological data and disease course under treatment. Imaging was interpreted by a specialist in pediatric brain radiology.

**Results:** This 12 year old child was first addressed to tertiary center Robert Debré University Hospital for bilateral intermediate uveitis in January 2018. She first presented with a history of recurrent oral aphtosis, genital ulcerations, arthritis and arthralgias, leading to the diagnosis of BD. While treated with Colchicine and NSAIDs, she later developed fatigue, headaches and posterior active uveitis with typical vasculitis. Neurological explorations (brain MRI and lumbar punction) at diagnosis and at the onset of posterior ocular manifestation in february 2021 were normal.

As recommended for severe ocular involvment, after steroid pulses, we introduced a antiTNF alpha treatment (Adalimumab 40mg/14dys), in addition to Azathioprine and Colchicine. She rapidly improved allowing progressive decrease of oral steroids until 3 mg per day, and withdraw of the azathioprine (due tu abdominal pains) with no relapse. On her last evaluation mid march 2023, she had inactive disease.

One week after, she presented with orthostatic headaches, dizziness, difficulties in concentrating and fatigue. Clinical and neurological examination was normal, and routine laboratory tests (NFS, ESR, liver and hepatic functions) were normal. Emergency brain and medullar imaging leaded to the diagnosis of intra-cranial hypotension, with a typical «Dinosaur tail sign», and a lightly thickened of supra, subtentorial and spinal dura mater, with gadolinium-enhanced. No sign of thrombophlebitis and no CSF leak was visualized.

She rapidly improved with a symptomatic treatment combining NSAIDs and cafeine, and no need to increase her anti-inflammatory baseline treatment.

**Conclusion:** Here we reported the first pediatric case of intracranial hypotension, a rare cause of headache, occurring in a patient followed for BD (the only other found in the literature was in an adult patient). This manifestation was isolated, considered as idiopathic, with no sign of BD activity, and resolved with symptomatic treatment only.

**Patient Consent**

Yes, I received consent

**Disclosure of Interest**

None declared

**References**

1. Schievink WI. Spontaneous Intracranial Hypotension. N Engl J Med. 2 déc 2021;385(23):2173-8.
2. Schievink WI, Maya MM, Louy C, Moser FG, Sloninsky L. Spontaneous intracranial hypotension in childhood and adolescence. J Pediatr. août 2013;163(2):504-10.
3. Albayram S, Gunduz A, Saip S, Ozer H, Gulsen F, Kocer N, et al. Intrathecal gadolinium-enhanced MR-cisternography in spontaneous intracranial hypotension associated with Behcet’s syndrome. Headache. avr 2007;47(4):613-6

## P163 Pediatric eosinophilic granulomatosis with polyangiitis responding to mepolizumab: case report

### R. Escamilla^1^, A. Arellano^1^, A. Tlacuilo^1^, A. Delgado^2^

#### ^1^Pediatric Rheumatology; ^2^Pathology, UMAE Hospital de Pediatría CMNO, Instituto Mexicano del Seguro Social, Guadalajara, Jalisco, Mexico

##### **Correspondence:** R. Escamilla

*Pediatric Rheumatology 2023*, **21(Suppl 2):**P163

**Introduction:** Eosinophilic granulomatosis with polyangiitis (EGPA) is an eosinophilic vasculitis, characterized by history of asthma and allergies affecting ears, nose and airway, associated with positivity to antineutrophil cytoplasmic autoantibodies (ANCA) in 30-40%. EGPA treatment in pediatrics is based on the use of corticosteroids and disease-modifying antirheumatic drugs. Mepolizumab, an anti-interleukin-5 monoclonal antibody, reduces blood eosinophil counts and may have value in the treatment of EGPA.

**Objectives:** To report a case of pediatric EGPA, on treatment with Mepolizumab.

**Methods:** The patient's data and relevant photographs of studies were collected of the clinical record. A review of the literature was made with articles from indexed medical journals.

**Results:** A 10-year-old female presented to the Pediatric Rheumatology department with chronic cough and multiple hospitalizations due to pneumonic infections since 2 years old. Afterwards she developed noisy breathing and was diagnosed with allergic rhinitis and hard control asthma by the age of 5 years, the patient was referred due to persisting lung infiltrates on chest radiograph and eosinophilia. Laboratory testing corroborated the presence of eosinophilia >1000/L in multiple determinations and elevated acute phase reactants, however ANCA were negative. A high resolution computed tomography scan of the lungs showed dense infiltrates; therefore the patient underwent bronchoscopy with tracheal biopsy, showing necrosis and perivascular inflammatory infiltrate within the endothelium, integrating an EGPA diagnosis.

The patient was started on Prednisone and Cyclophosphamide as induction therapy followed by Methotrexate as maintenance during two years, without significant improvement, persisting with exacerbations of asthma, requiring multiple hospitalizations and use of supplementary oxygen, since 7 years-old.

Because of the protracted course to the established treatment and after written informed consent, we initiated Mepolizumab, on a SC 100 mg scheme each four weeks. By the month six of treatment the supplementary oxygen was suspended with no further hospitalizations. The follow-up CT lung scan showed lesser infiltrates and no adverse events were reported.

**Conclusion:** EGPA is a rare systemic vasculitis with very limited cases reported in the pediatric population, while Mepolizumab, an anti-interleukin-5 monoclonal antibody, reduces blood eosinophil counts and improve refractory cases, which also allows dose reduction of high doses of systemic and inhaled steroids. No specific pediatric treatment recommendations exist due to rare nature of the disease in pediatric population.

**Patient Consent**

Yes, I received consent

**Disclosure of Interest**

None declared

**Reference**s

1. Pavord ID, et al. Mepolizumab for severe eosinophilic asthma (DREAM): a multicentre, double-blind, placebo-controlled trial. 2012;380:651–9.
2. Pavord ID, et al. From DREAM to REALITI-A and beyond: Mepolizumab for the treatment of eosinophil-driven diseases. Allergy. 2022;77:778–97.
3. Bettiol A, et al. Mepolizumab for eosinophilic granulomatosis with polyangiitis: A European multicenter observational study. Arthritis Rheumatol. 2022;74:295–306.

## P164 Pulmonary bleeding and thrombosis

### R. M. Alcobendas, A. R. Camba, C. U. Gascon, C. Millan, B. Díaz, M. Garrido

#### Hospital La Paz, Madrid, Spain

##### **Correspondence:** C. U. Gascon

*Pediatric Rheumatology 2023*, **21(Suppl 2):**P164

**Introduction:** Anti-neutrophil cytoplasmic antibody-associated vasculitides (AAV) are rare systemic diseases. The literature in children is scarce and frequently refers to renal involvement. Treatment is usually guided by guidelines and recommendations for adult patients.

**Objectives:** To describe a clinical case of ANCA-positive vasculitis and associated antiphospholipid syndrome.

**Methods:** Clinical chart review

**Results:** 14-year-old female patient was admitted to the Intensive Care Unit due to a massive pulmonary hemorrhage requiring assisted mechanical ventilation. She presented a one-month previous history of asthenia, febrile episodes, and joint pain. Laboratory tests showed a significant elevation in C-reactive protein levels, while the complete blood count, renal function, and liver function were within normal range. Chest X-ray revealed diffuse opacities consistent with pulmonary bleeding and bilateral cavitated lesions. Immunology report confirmed the presence of ANCA PR3 antibodies, raising a strong suspicion of Granulomatosis with Polyangiitis. Treatment with methylprednisolone boluses, immunoglobulins, cyclophosphamide, and plasmapheresis was started. However, the patient did not show improvement in the following days. As a result, rituximab therapy was initiated showing a slow but consistent improvement and leading to extubation and complete discontinuation of oxygen therapy.

In parallel, an extension study performed revealed extensive bilateral thrombosis in the jugular, subclavian, and axillary veins. Further thrombosis studies revealed positive results for lupus anticoagulant, anti-β2 glycoprotein I, and anti-cardiolipin antibodies. Due to the high risk of pulmonary rebleeding, systemic anticoagulant therapy could not be initiated and a mechanical thrombectomy was performed. However, one week later, thrombi reappeared. Following the administration of three doses of rituximab, the patient started subcutaneous bemiparin, resulting in rapid resolution of the thrombi

**Conclusion:** The association of AAV and Antiphospholipid syndrome is not frequent. Its association with pulmonary bleeding represents a therapeutic challenge.

**Patient Consent**

Yes, I received consent

**Disclosure of Interest**

None declared

**References**

1. Yates M, Watts RA, Bajema IM, Cid MC, Crestani B, Hauser T et al. **EULAR**/ERA-EDTA recommendations for the management of ANCA-associated vasculitis.Ann Rheum Dis. 2016 Sep;75(9):1583-94.
2. Calatroni M, Oliva E, Gianfreda D, Gregorini G, Allinovi M, Ramirez GA et al. **ANCA**-associated **vasculitis** in childhood: recent advances. .Ital J Pediatr. 2017 May 5;43(1):46

## P165 Distribution of HLA-A, HLA-B and HLA-DRB1 genes in Croatian population with IGA vasculitis

### M. Held^1^, K. Stingl Jankovic^2^, M. Sestan^1^, M. Sapina^3^, N. Kifer^1^, S. Srsen^4^, M. Frkovic^1^, A. Gagro^5^, Z. Grubic^2^, M. Jelusic^1^

#### ^1^Department of Pediatrics, University Hospital Centre Zagreb, University of Zagreb School of Medicine; ^2^Tissue Typing Centre, Clinical Department for Transfusion Medicine and Transplantation Biology, University Hospital Centre Zagreb, Zagreb; ^3^Department of Pediatrics, University Hospital Centre Osijek, Josip Juraj Strossmayer University of Osijek, Medical Faculty Osijek, Osijek; ^4^Department of Pediatrics, University Hospital Centre Split, University of Split School of Medicine, Split; ^5^Children's Hospital Zagreb, Josip Juraj Strossmayer University of Osijek, Medical Faculty Osijek, Zagreb, Croatia

##### **Correspondence:** M. Held

*Pediatric Rheumatology 2023*, **21(Suppl 2):**P165

**Introduction:** IgA vasculitis (IgAV) is a small vessel vasculitis occurring predominantly in childhood. Studies concerning the genetic background of IgAV have confirmed that susceptibility to the disease may be influenced by Human Leukocyte Antigens (HLA), with HLA-DRB1 gene showing a strong association with the disease.

**Objectives:** To investigate HLA-A, B, DRB1, DQA1 and DQB1 polymorphisms among Croatian patients with IgAV and their influence to disease susceptibility and clinical heterogeneity.

**Methods:** 130 children from three pediatric rheumatology centers fulfilling the diagnostic EULAR/PRINTO/PRES criteria for IgAV and 202 unrelated healthy individuals were enrolled. Genomic DNA was extracted from whole peripheral blood. The HLA-DRB1 genes were analysed using the Next Generation Sequencing (NGS) method, while HLA-A, B, DQA1, and DQB1 polymorphisms were determined by polymerase chain reaction methods in combination with oligonucleotides specific for HLA allelic groups (PCR-SSO).

**Results:** Among IgAV patients, 71 were girls and 59 were boys with a median age 6.3 (4.4-8.1) years at the time of diagnosis. All patients had purpuric rash, 108 (83.1%) had arthralgia or arthritis, 39 (30%) had gastrointestinal (GI) involvement, while 30 (23.1%) patients developed IgA vasculitis nephritis (IgAVN). HLA-A*03 (21.6% vs. 12.4%, p=0.012), HLA-B*37 (2.9% vs. 0.2%, p=0.017) and HLA-DRB1*12 (3.1% vs. 0.7%, p=0.029) genes were significantly more frequent in IgAV than in controls. HLA-A*26 (12.5% vs. 1.0%, p=0.008), HLA-B*55 (10% vs. 0%, p=0.006), HLA-DRB1*10 (5.1% vs. 0.6%, p=0.03), HLA-DQA1*01 (38.6% vs. 57.5%, p=0.037) and HLA-DQB1*05 (18.2% vs. 36.2%, p=0.027) genes were associated with an increased risk for GI involvement among IgAV patients. A significantly more frequent occurence of HLA-A*32 (11.8% vs. 1.0%, p=0.013), HLA-DRB1*04:04 (5.0% vs. 0%, p=0.012) and HLA-DQB1*05 (47.6% vs. 27.1%, p=0.015) genes was observed in patients with IgAVN.

**Conclusion:** Our results demonstrated that there is an association of HLA-A*03, HLA-B*37 and HLA-DRB1*12 genes with susceptibility to IgAV in Croatian children. On the other side, different genes of HLA-A, B, DRB1, DQA1 and DQB1 loci showed an association with the clinical manifestations of the disease itself.

**Patient Consent**

Yes, I received consent

**Disclosure of Interest**

None declared

## P166 Comparison of Eular/Printo/Pres Endorsed Ankara 2008 And 2022 ACR/EULAR classification criteria in childhood granulomatosis with polyangiitis

### U. Kaya Akca^1^, E. D. Batu^1^, M. Jelusic^2^, M. Calatroni^3^, R. Bakry^4^, M. Frkovic^2^, N. Vinšová^5^, R. T. Campos^6^, A. Horne^7^, S. Caglayan^8^, A. Vaglio^9^, G. Moroni^3^, G. Emmi^10^, G. M. Ghiggeri^11^, O. Koker^12^, R. A. Sinico^3^, S. Kim^13^, A. Gagro^14^, C. Matucci-Cerinic^15,16^, E. Çomak^17^, Z. E. Tekin^18^, E. Arslanoglu Aydin^19^, M. Heshin-Bekenstein^20^, B. Celikel Acar^18^, M. Gattorno^16^, S. Akman^17^, B. Sozeri^8^, K. Palmblad^7^, S. M. Al-Mayouf^21^, C. A. Silva^6^, P. Doležalová^5^, S. Ozen^1^ on behalf of on behalf of the Vasculitis Working Party of the Pediatric Rheumatology European Society (PReS)

#### ^1^Hacettepe University Faculty of Medicine, Ankara, Türkiye; ^2^UHC Zagreb, University of Zagreb School of Medicine, Zagreb, Croatia; ^3^IRCCS Humanitas Research Hospital, Milan, Italy; ^4^East Jeddah Hospital, Jeddah, Saudi Arabia; ^5^General University Hospital and 1st Faculty of Medicine, Charles University, Prague, Czech Republic; ^6^Hospital das Clínicas da Faculdade de Medicina, Universidade de São Paulo (HCFMUSP), São Paulo, Brazil; ^7^Astrid Lindgren Children's Hospital, Stockholm, Sweden; ^8^Umraniye Training and Research Hospital, Istanbul, Türkiye; ^9^Meyer Children's Hospital, Florence, Italy, Florence; ^10^University of Florence, Florence, Italy, ^11^IRCCS Istituto Giannina Gaslini, Genoa, Italy; ^12^Marmara University Medical School, Istanbul, Türkiye; ^13^University of California, San Francisco, United States; ^14^Children's Hospital Zagreb, Zagreb, Croatia; ^15^DINOGMI, Università degli Studi di Genova, Genoa, Italy; ^16^UOC Rheumatology and autoinflammatory diseases, IRCCS Istituto Giannina Gaslini, Genoa, Italy; ^17^Akdeniz University, Antalya; ^18^Ankara City Hospital; ^19^Dr. Sami Ulus Maternity and Child Health and Diseases Training and Research Hospital, Ankara, Türkiye; ^20^Dana-Dwek Children's Hospital, Tel Aviv Sourasky Medical Center, Tel Aviv University, Tel Aviv, Israel; ^21^King Faisal Specialist Hospital and Research Center, Alfaisal University, Riyadh, Saudi Arabia

##### **Correspondence:** U. Kaya Akca

*Pediatric Rheumatology 2023*, **21(Suppl 2):**P166

**Introduction:** Granulomatosis with polyangiitis (GPA) is an antineutrophil cytoplasmic antibody (ANCA) associated vasculitis. The 2022 American College of Rheumatology/ European Alliance of Associations for Rheumatology (ACR/EULAR) GPA classification criteria for adult patients have been recently published.

**Objectives:** We aimed to assess the performance of the ACR/EULAR GPA classification criteria in pediatric patients and compare it with the EULAR/Pediatric Rheumatology International Trials Organization (PRINTO)/Pediatric Rheumatology European Society (PReS) endorsed Ankara 2008 criteria.

**Methods:** Retrospective data of pediatric GPA patients in 20 centers from 9 countries were evaluated. The diagnosis of GPA was made according to the expert opinion. The control group consisted of patients with other primary systemic vasculitis or diseases that mimic vasculitis. All patients were younger than 18 years at diagnosis.

**Results:** The study included 77 GPA and 102 control patients. Of the GPA patients, the median age at diagnosis was 13.6 years (Q1-Q3: 11.4-15.9) and the median follow-up time was 3.7 years (Q1-Q3: 1.3-5.9). The control group consisted of patients with Takayasu arteritis (n=20), immunoglobulin A vasculitis (n=19), sarcoidosis (n=16), microscopic polyangiitis (MPA) (n=16), polyarteritis nodosa (n=14), Behçet's disease (n=12), vasculitis associated with connective tissue diseases (n=2), eosinophilic granulomatosis with polyangiitis (n=1), juvenile dermatomyositis (n=1), and Cogan's syndrome (n=1).

Of the GPA patients, constitutional symptoms (85.7%) and upper airway involvement (76.6%) were the most common presentations followed by renal (72.7%), musculoskeletal (64.9%), and pulmonary involvement (61.0%). Granulomatous inflammation in biopsy was reported in 50.6% of GPA patients. Overall, 81.8% of GPA patients were proteinase 3-ANCA (PR3-ANCA) and/or myeloperoxidase-ANCA (MPO-ANCA) positive (72.7% for PR3-ANCA).

The sensitivity of the Ankara 2008 criteria and the 2022 ACR/EULAR GPA classification criteria was 94.8% and 89.6%, while the specificity was 92.1% and 96.0%, respectively. In the GPA group, 73 patients fulfilled the Ankara 2008 criteria and 69 the 2022 ACR/EULAR classification criteria.

**Conclusion:** In the pediatric population, both the 2022 ACR/EULAR criteria and the Ankara 2008 criteria had a good performance, with the former having a lower sensitivity and higher specificity than the latter. Pediatricians may favor the Ankara 2008 criteria in the pediatric population because of its practical use.

**Patient Consent**

Yes, I received consent

**Disclosure of Interest**

None declared

## P167 Severe pulmonary haemorrhage secondary to granulomatosis with polyangiitis

### N. Keenan, S. Coyne, J. MacMahon, E. J. MacDermott

#### Rheumatology, Children’s Health Ireland at Crumlin, Dublin, Ireland

##### **Correspondence:** N. Keenan

*Pediatric Rheumatology 2023*, **21(Suppl 2):**P167

**Introduction:** Granulomatosis with Polyangiits (GPA) is an ANCA-associated vasculitis of unknown aetiology, that is rare in the paediatric population.(1) Lung involvement, at presentation or with flares, is a common manifestation. It has a variable severity, the most significant of which is diffuse alveolar haemorrhage (2).

**Objectives:** Presentation:

A previously well 15-year-old boy presented with a 3-week history of fatigue, 4 days of intermittent joint pain/stiffness; papular rash to elbows and a 1 day history of fever. He was treated for presumed reactive arthritis however remained febrile and subsequently developed watery ‘black-coloured’ stool, macroscopic haematuria and productive cough with haemoptysis. Bloodwork showed ongoing rise in inflammatory markers with new onset significant anaemia.

He became persistently tachycardic and developed reduced O2 saturations. Examination revealed reduced air entry bilaterally with coarse crackles; diffuse arthritis; erythematous papules on elbows, shins and dorsal aspect of feet; oral ulceration and unilateral septal ulceration.

**Methods:** Investigations:

CT Thorax demonstrated a 2cm cavity in left mid zone, in keeping with extensive intra-alveolar haemorrhage.

Due to the constellation of clinical findings, the main differential was vasculitis and urgent immunological bloodwork was sent. This showed a highly positive PR3-ANCA, in keeping with a diagnosis of GPA.

**Results:** Treatment:

Given the concern for diffuse active pulmonary haemorrhage, a 3-daycourse of pulse IV methylprednisolone (30mg/kg) was commenced.

Despite initial improvement, on day 3 of therapy the patient was noted to be pale, lethargic with increased cough and drop in haemoglobin, raising concern for ongoing active pulmonary haemorrhage. Second line therapy with IV rituximab was commenced with good effect and the patient was discharged 2 weeks later.

**Conclusion:** Pulmonary haemorrhage in children is a medical emergency requiring prompt management. Although GPA is rare in childhood, it should be considered in the context of unexplained haemoptysis and haemorrhage and we would strongly recommend early testing for ANCA in these patients.

**Patient Consent**

Yes, I received consent

**Disclosure of Interest**

None declared

**References**

1. Saenz Rios F, Devaraj S, Movva G, Movva H, Nguyen QD. Granulomatosis With Polyangiitis in a Pediatric Male. Cureus. 2020 Dec 13;12(12):e12055. doi: 10.7759/cureus.12055. PMID: 33447484; PMCID: PMC7802401.
2. Filocamo G, Torreggiani S, Agostoni C, Esposito S. Lung involvement in childhood onset granulomatosis with polyangiitis. Pediatr Rheumatol. 2017;15:28

## P168 Clinical characteristics and risk factors for renal involvement in children with henoch-schonlein purpura

### P. Khaosut^1^, B. Chatpaitoon^2^

#### ^1^Department of Pediatrics, Center of Excellence for Allergy and Clinical Immunology, Division of Allergy, Immunology and Rheumatology, Faculty of Medicine, Chulalongkorn University, King Chulalongkorn Memorial Hospital; ^2^Department of Pediatrics, Faculty of Medicine, Chulalongkorn University, King Chulalongkorn Memorial Hospital, Bangkok, Thailand

##### **Correspondence:** P. Khaosut

*Pediatric Rheumatology 2023*, **21(Suppl 2):**P168

**Introduction:** Henoch-Schoenlein purpura (HSP; newly known as IgA vasculitis) is a self-limiting systemic vasculitis in children. However, renal involvement is associated with a long-term unfavorable outcome and can lead to significant morbidity, such as end-stage renal disease. Consequently, it is important to assess the risk factors for developing renal involvement in HSP.

**Objectives:** To describe the clinical and laboratory characteristics of childhood HSP with renal involvement and identify risk factors associated with HSP nephritis.

**Methods:** This was an ambidirectional descriptive study of 77 children with HSP. All demographic data, clinical features, and laboratory tests were collected from electronic medical records from January 2010 to December 2022 at King Chulalongkorn Memorial Hospital, Thailand. Risk factors for renal involvement in HSP were assessed using multivariate logistic regression. Kaplan–Meier survival analysis was used to define the time to develop renal involvement.

**Results:** Twenty-five children (32.4% of HSP patients) developed HSP nephritis. The common findings in HSP with renal involvement were microscopic hematuria (100%), nephrotic range proteinuria (44%) and non-nephrotic range proteinuria (40%). Multivariate logistic regression showed that overweight (BMI ≥ 23 kg/m^2^) (OR 4.53; 95% CI 1.17-17.54; p= 0.029), hypoalbuminemia (OR 4.12; 95% CI 1.11-15.31; p= 0.034) and recurrence (OR 3.83; 95% CI 1.02-14.31; p= 0.045) were significantly associated with renal involvement. During one year of follow-up, the mean time to develop renal involvement was 9 months (SD=0.60).

**Conclusion:** Overweight, hypoalbuminemia, and recurrence were predictive factors of HSP nephritis. Our study emphasizes that HSP patients with risk factors should be closely and adequately monitored for at least one year after the onset of the disease.

**Patient Consent**

Yes, I received consent

**Disclosure of Interest**

None declared

## P169 Kawasaki disease: clinical manifestations and therapy

### B. Koren, V. Berce

#### University Medical Centre , Maribor, Slovenia

##### **Correspondence:** B. Koren

*Pediatric Rheumatology 2023*, **21(Suppl 2):**P169

**Introduction:** Kawasaki disease, also known as mucocutaneous lymph node syndrome, is an acute febrile illness of unknown cause that mainly affects children younger than 5 years of age. It is a vasculitis of the medium- sized vessels that has a predilection for the coronary arteries and is the leading cause of acquired heart disease in developed countries.

**Objectives:** To evaluate the clinical manifestations and therapy in pediatric patients with Kawasaki disease.

**Methods:** A single small center study was performed. Patients with Kawasaki disease treated as inpatients in our pediatric clinic from January 2018 till December 2022. Diagnostic criteria according to the 2002 Diagnostic Guidelines of the Japan Kawasaki Disease Research Committee were used. Patients with characteristics similar to Kawasaki disease who were diagnosed with MIS-C/PIMS-TS were excluded from the study. Data were collected retrospectively from the patients’ medical records.

**Results:** 27 patients were included. The mean age of all patients was 2.9 years (range 4 months – 9 years). Female and male patients were almost equally affected (female 14 and male 13 patients).

Typical (complete) disease was diagnosed in 18 patients and atypical (incomplete) disease in 9 patients.

All patients presented with fever of at least 5 days. 24 patients (88.9%) had sore throat and/or red, cracked lips and/or erythema of palms and soles, 17 patients (62.9%) had conjunctivitis, 12 patients (44.4%) had edema of hands and feet and/or erythema of palms and soles, 19 patients (70.4%) had enlarged neck lymph node and 17 patients (62.9%) had rash.

The mean duration of fever was 8.1 days (range 5- 17 days). 2 patients developed hepatopathy, 2 renal disease (proteinuria and hematuria) and 1 serous meningitis.

Coronary artery dilatations and/or aneurysms developed in 4 patients, diagnosed with atypical (incomplete) disease.

All our patients were treated with intravenous immune globulin (IVIG) and Aspirin. Three patients with atypical (incomplete) disease received glucocorticoids. 2 patients with persistent coronary artery aneurysms and elevated inflammatory parameters were referred to another tertiary care center where they were treated with biological therapy. In 1 patient mild coronary artery resolved after 1 week of treatment.

**Conclusion:** Our patients responded well to treatment. They had relatively few complications and no deaths. Our study demonstrated that coronary artery aneurysms can develop especially in patients with atypical (incomplete) Kawasaki disease, which is consistent with literature data. Therefore, early diagnosis and prompt treatment are particularly important.

**Patient Consent**

Not applicable (there are no patient data)

**Disclosure of Interest**

None declared

**References**

1. McCrindle BW, Rowley AH, Newburger JW, et al. Diagnosis, treatment and long-term management of Kawasaki disease: A scientific statement for health professionals from the American heart association. Circulation 2017; 135:e927.
2. Owens AM, Plewa MC. Kawasaki disease. StatPearls (Internet) 2022.
3. Cattalini m, Della Paolera S, Zunica f, et al. Defining Kawasaki disease and pediatric inflammatory multisystem syndrome- temporally associated to SARS-CoV-2 infection during SARS-CoV-2 epidemic in Italy: results from a national, multicenter survey. Pediatr Rheumatol Online J. 2021;19(1):29.

## P170 Detection of missed left circumflex coronary artery abnormalities in children with Kawasaki disease: insights from ct coronary angiography at Chandigarh, North India

### R. Kumar^1^, M. Singhal^2^, A. Thangaraj^1^, A. Jindal ^1^, P. Vignesh^1^, D. Suri^1^, S. Singh^1^

#### ^1^Paediatric Allergy Immunology Unit, Department of Pediatrics, Advanced Paediatrics Centre; ^2^Department of Radiodiagnosis and Imaging, Postgraduate Institute of Medical education and Research, Chandigarh, India

##### **Correspondence:** R. Kumar

*Pediatric Rheumatology 2023*, **21(Suppl 2):**P170

**Introduction:** Kawasaki disease (KD) is a systemic vasculitis with a predilection for coronary arteries. Two-dimensional echocardiography (2DE) has hitherto been the preferred imaging modality for assessing coronaries in children with KD. 2DE, however, has several limitations. For instance, coronary artery abnormalities (CAAs) involving the left circumflex artery (LCx) can be missed on 2DE.

**Objectives:** In this study, we have evaluated the involvement of LCx in children with KD on computed tomography coronary angiography (CTCA) performed on a 128-slice dual source platform.

**Methods:** Diagnosis of KD was based on American Heart Association (AHA) guidelines. Radiation-optimized CTCA was performed in 225 children with KD over nine years (2013 – 2022). 2DE was performed on the same day, before or after CTCA. Patients were managed using AHA-based treatment protocols. CTCA was performed on 128 slice- dual-source CT scanner (Somatom definition flash, Siemens Healthineers, Erlangen, Germany) using non-ionic contrast (Omnipaque 350, GE Healthcare, Ireland).

**Results:** On CTCA, involvement ofLCx was seen in 41/225 (18.2%) patients with KD. However, 2DE detected LCx abnormalities in only 16/41 patients. Four patients(9.75%) had isolated LCx involvement. CTCA showed 47 CAAs in LCx in 41 patients. Aneurysms (40 fusiform; 2 saccular) were seen in 39 patients; stenoses in 3; thrombosis in 2. One patient had an aneurysm and a stenosis, while two had only stenoses. Thromboses and stenoses had both been missed on 2DE. Giant aneurysms in LCx were seen in 6patients. Proximal LCx aneurysms were seen in 39 patients - of these, 12 had distal extension as well. Distal LCx aneurysms, in the absence of proximal involvement, were seen in 6 patients. Two patients had non-contiguous multiple aneurysms. Based on the findings of the CTCA, treatment protocols had to be modified in 3/41 (7.3%)patients.

**Conclusion:** Abnormalities of LCx were seen in 18.2% (41/225) of patients on CTCA. The majority of these had been missed on 2DE. Isolated LCx CAAs were seen in 4 patients. CTCA should be the preferred imaging modality for the assessment of LCx. Our findings have important implications for treatment planning and follow-up of children with KD. 2DE alone is inadequate for an assessment of LCx in children with KD.

**Patient Consent**

Yes, I received consent

**Disclosure of Interest**

None declared

**References**

1. Singhal M, Pilania RK, Jindal AK, Gupta A, Sharma A, Guleria S, et al. Distal coronary artery abnormalities in Kawasaki disease: experience on CT coronary angiography in 176 children. Rheumatology. 2023 Feb 1;62(2):815–23.
2. Singhal M, Singh S, Gupta P, Sharma A, Khandelwal N, Burns JC. Computed Tomography Coronary Angiography for Evaluation of Children With Kawasaki Disease. Curr Probl Diagn Radiol. 2018 Aug;47(4):238–44.

## P171 Cardiovascular outcomes in kawasaki disease: single center's experience

### S. Kurbanova^1^, M. Kantemirova^1,2^, S. Valieva^1^, A. Glazyrina^1^, E. Zholobova^1,3^

#### ^1^Department of rheumatology, Morozov Children’s City Clinical Hospital, Moscow; ^2^Department of Pediatrics, RUDN University, Moscow; ^3^Department of pediatrics, First Moscow State Medical University, Moscow, Russian Federation

##### **Correspondence:** S. Kurbanova

*Pediatric Rheumatology 2023*, **21(Suppl 2):**P171

**Introduction:** Kawasaki disease (KD) is an inflammatory disorder of young children, associated with vasculitis of the coronary arteries (CA) with subsequent aneurysm formation in up to one-third of untreated patients. Those who develop aneurysms are at life-long risk of coronary thrombosis or the development of stenotic lesions, which may lead to myocardial ischaemia, infarction or death. The coronary sequelae of KD have significant morbidity and mortality and are the second most common cause of acquired cardiac disease in children.

**Objectives:** We aimed to study the risk factors for cardiovascular outcomes in children with KD

**Methods:** We examined 188 patients (boys to girls ratio – 2:1, median age 24 months [11;38]) with KD hospitalised in Morozov Children’s City Clinical Hospital in 2014-2019. 19 (10.1%) of them were younger than6 months, 29 (15.4%) - 6-12 months, 117 (62.3%) - 12 months-5 years, 23 (12.2%) - 5 years and older. In all patients, we evaluated coronary lesions by echocardiography and coronary angiography immediately after the acute stage. Adverse outcomes included coronary artery disease and mortality. Univariate and multivariate logistic regression analyses were conducted to explore the relationships between adverse outcome and gender, age, clinical diagnosis, laboratory biomarkers, initiation time of IVIG administration.

**Results:** In the acute period of KD, cardiovascular systems lesions were detected in 48.9% of children and included coronary (aneurysms of CA in 49 children (26%)) and non-coronary (myocarditis in 47 patients (25%), pericarditis in 18 patients (9.6%), mitral valve insufficiency in 49 (26.1%)). Systemic artery aneurysms developed in 2 patients (1.06%). The majority (90.4%) of children with KD had favourable outcome. Adverse outcomes included the formation of distant aneurysms of the CA throughout 8 weeks from the onset of the disease in 13 patients (6.9%) and death in acute/subacute period of illness (2.7%) due to sudden acute occlusive thrombosis of the CA against the background of active necrotizing coronaritis with the formation of aneurysms of the CA against the background of carditis.

Markers of adverse outcome of KD, according to our data, were high levels proANP (95% CI 2.325–205.776) and NT-proBNP (95% CI 2.610–108.118). Threshold levels of natriuretic peptides in the acute period of the KD werestatistically significant for determining the risk of developing adverse outcomes:more than 984 pg/ml (with sensitivity 79%, specificity 84.8% and overall predictive value 82.9%) for NT-proBNP, andmore than 1.015 nmol/L (with sensitivity 87.5%, specificity 75.8%, and overall predictive value 78.0%) for proANP.

**Conclusion:** The incidence of coronary aneurysm in acute KD was 26%, 75% of which subsequently regressed. Death occurred in 2.7%. Current evidence suggests that NT-proBNP and proANP may be used as a diagnostic tool for KD complicated with adverse outcomes.

**Patient Consent**

Yes, I received consent

**Disclosure of Interest**

None declared

## P172 Resolution of giant coronary artery aneurysm in kawasaki disease with long-term anakinra therapy: a case report

### J.-E. Lo^1^, A. Zeft^2^, S. Panupattanapong^2^

#### ^1^National Taiwan University, Taipei, Taiwan, Province of China, ^2^Center for Pediatric Rheumatology and Immunology, Cleveland Clinic Children's, Cleveland, United States

##### **Correspondence:** J.-E. Lo

*Pediatric Rheumatology 2023*, **21(Suppl 2):**P172

**Introduction:** Giant coronary artery aneurysms in Kawasaki disease (KD) are less likely to regress to normal size, and therefore expose patients to life-long risk of major cardiac events, such as acute coronary thrombosis. As IL-1β plays a pivotal role in KD inflammatory pathogenesis and aneurysm formation, anakinra (an IL-1 receptor antagonist) was proposed as an effective treatment for a pediatric patient with KD who developed a giant coronary artery aneurysm.

**Objectives:** To describe the efficacy of anakinra in a KD patient who developed multiple extensive coronary artery aneurysms.

**Methods:** case report

**Results:** A 3-year-old male with incomplete KD presented with 3 days of fever, cervical lymphadenopathy, non-exudative bilateral conjunctivitis, and erythematous cracked lips. Supportive laboratory criteria included leukocytosis, thrombocytosis, elevated inflammatory markers, transaminitis, hypoalbuminemia and sterile pyuria. On day 4 of illness, his initial echocardiogram was normal. He received aspirin and IVIG 2 g/kg but became hypotensive with alteration of consciousness during IVIG infusion. The infusion was stopped and he was transferred to pediatric intensive care unit. Repeat echocardiogram revealed globally decreased systolic function (ejection fraction 41%) and mild right coronary artery ectasia (z-score 2.4). He received high dose methylprednisolone and daily anakinra 4 mg/kg/day subcutaneous injection. Two days later, anakinra was escalated to 10 mg/kg/day due to recrudescence of fever and rising inflammatory markers. His fever subsided, however subsequent echocardiogram study on day 10 of illness identified progressive dilatation of right main coronary artery with medium size aneurysms (z-score 5.6). The patient was given a single dose of infliximab 10 mg/kg. The next day, further images were obtained due to a concern for systemic vasculitis, which showed normal brain MRA and abdomen CTA, however chest CTA revealed multiple coronary artery aneurysms as follows: a giant aneurysm in left anterior descending (z-score 12) and moderate aneurysms in the right main (z-score 5.6) left main (z-score 4.2), and left circumflex coronary (z-score 6.1) arteries. He received ongoing treatment with anakinra 100 mg daily (7.7 mg/kg/day), 6-week taper of corticosteroids (starting dose 2 mg/kg/day), enoxaparin and aspirin.

After 3 months of treatment, the aneurysms regressed to normal size, including the giant aneurysm, except for a small residual aneurysm of the right main coronary artery (z-score 3.5). Given no further reduction of this small aneurysm after 6 months of therapy, anakinra was discontinued.

**Conclusion:** This report describes a patient with incomplete KD who developed multiple medium aneurysms and one giant aneurysm, despite timely IVIG administration and aggressive immunotherapy. After receiving anakinra for six months, the aneurysms reduced in size, and the giant aneurysm completely regressed.

**Patient Consent**

Yes, I received consent

**Disclosure of Interest**

None declared

## P173 Ocular involvement in paediatric behçet disease: a multicentric Italian experience

### I. Maccora^1,2^, S. I. Orsini^1^, R. Gallizzi^3^, D. Montin^4^, M. Cattalini^5^, F. La Torre^6^, A. Spagnolo^7^, F. Diomeda^8^, G. Simonini^1,2^

#### ^1^Rheumatology Unit, ERN ReConnet Center, Meyer Children's Hospital IRCCS; ^2^NeuroFARBA Department, University of Florence, Florence; ^3^Department of Health Science Magna Graecia University of Catanzaro, Catanzaro; ^4^Department of Pediatrics and Public Health, University of Turin, Turin; ^5^Pediatrics Clinic, ASST Spedali Civili di Brescia, University of Brescia, Brescia; ^6^Department of Pediatrics, Giovanni XXIII Pediatric Hospital, University of Bari, Bari; ^7^Department of Human Pathology of Adulthood and Childhood Gaetano Barresi, Unit of Pediatrics, University of Messina, Messina; ^8^Pediatric Rheumatology and Immunology Unit, “Vito Fazzi” Hospital, Lecce, Italy

##### **Correspondence:** I. Maccora

*Pediatric Rheumatology 2023*, **21(Suppl 2):**P173

**Introduction:** Behçet disease (BD) is a rare disease in childhood and ocular involvement may lead to several complications including blindness if not properly recognized and treated.

**Objectives:** To describe a cohort of paediatric BD patients with ocular involvement and evaluate the prescribed treatment.

**Methods:** This is a multicentric retrospective study involving 6 paediatric rheumatology units in Italy. To be eligible patients should have a diagnosis of paediatric Behçet syndrome according to International Criteria for Behçet’s Disease Criteria and/or to the International Study Group Criteria for BS, or Paediatric BD classification criteria. Demographic, laboratory, and clinical data were collected and followed up to March 2023. Ocular characteristics, including different performed treatments, were assessed and registered according to SUN.

**Results:** 33 children with a diagnosis of BD with associated uveitis (22 male 66.7%, 28 Caucasian 84.8%) resulted eligible. The median age at onset of BD and uveitis were respectively 137.5 months (range 30-206) and 132 months (36-208). Uveitis diagnosis preceded the BD diagnosis in 18 children (54.5%). Among the 33 children, 5 had ANA positivity (15.2%) and 17 HLA B51 positivity (51.5%). Twenty-five patients showed bilateral uveitis (75.8%), 16 showed panuveitis (48.5%), 8 anterior uveitis (24.2%), 5 posterior uveitis (15.2%), and 4 intermediate uveitis (12.1%). Twenty-five children (75.8%) had symptomatic uveitis: 20 hyperaemia, 13 photophobia, 9 ocular pain, 18 blurred vision. As far as systemic manifestations is concerned, 3 children showed recurrent fevers (9.1%), 27 recurrent oral ulcers (81.8%), 6 recurrent genital ulcers (18.2%), 4 gastrointestinal symptoms (12.1%), 11 cutaneous rash (33.3%), 4 CNS involvement (12.1%), and 2 thrombosis (6%).

Before the specific ocular treatment, 18 patients received at least one systemic, non steroid-treatment for the underlying disease. All the patients received at least one systemic treatment for the uveitis,: 25 received adalimumab, 2 tocilizumab, 1 abatacept, 3 infliximab, 4 azathioprine, 1 methotrexate. The systemic treatment other than corticosteroids was started after a median time from diagnosis of 15 months (range 0-141) and the median duration was 25 months (range 4-73). The remission was achieved with 30/35 treatments (85.7%) after a median time of 8 months (6-24). Six children had a relapse in therapy after the achievement of remission (20%). Fourteen patients stopped the therapy for persistent remission, but 5 relapsed (35.7%) after a median time of 9 months (range 1-48).

**Conclusion:** Uveitis in BD is a sight-threatening condition, and it presents as a panuveitis in up to the 50% of cases. Biologic treatments are often required to control ocular inflammation in BD.

**Patient Consent**

Yes, I received consent

**Disclosure of Interest**

None declared

## P174 Analysis of worldwide differences of Clinical Practice Strategies (CLIPS) in the management of Kawasaki disease through the JIR-clips network: a cost action

### Daiva Gorczyca^1^, Margarita Ganeva^2^, Jurgita Marčiulynaitė^3^, Zeynep Balik (dr.zeynepbalik@gmail.com)^4^, Cristina Lanna^5^, Jacqueline Yan^6^, Dzifa Dey^7^, Teresa Giani^8^, Aušra Šnipaitienė^3^, Arūnė Ramanauskienė^9^, Seza Ozen^10^, Tilmann Kallinich for the JIR-CLIPS network^11^

#### ^1^Charité Universitätsmedizin Berlin, Berlin, Germany; ^2^Department of Pediatric Rheumatology, Medical University Sofia, Sofia, Bulgaria; ^3^Peaditric Clinic, Lithuanian University of Health Sciences, Kaunas, Lithuania; ^4^Department of Pediatrics, Division of Rheumatology, Hacettepe University Faculty of Medicine, 06100 Ankara, Turkey; ^5^Department of Locomotor System, Universidade Federal de Minas Gerais, Belo Horizonte, Brazil; ^6^Faculty of Medical and Health Sciences, Paediatrics, Child and Youth Health, The University of Auckland, Auckland, New Zealand; ^7^Rheumatology Unit, Department of Medicine and Therapeutics, University of Ghana Medical School, College of Health Sciences, Accra, Ghana; ^8^AOU Meyer, Florence, Italy; ^9^Gijos Klinikos, Kaunas, Lithuania; ^10^Hacettepe University, Ankara, Türkiye; ^11^Department of Paediatric Pulmonology, Immunology, and Intensive Care Medicine, Charité Universitätsmedizin Berlin, Berlin, Germany

##### **Correspondence:** J. Marčiulynaitė

*Pediatric Rheumatology 2023*, **21(Suppl 2):**P174

**Introduction:** Kawasaki disease (KD) is the predominant cause of childhood-acquired heart disease. Despite great improvement in the management of KD, some challenges remain. Due to the wide array of country-specific medical systems and financial capacities implementation of existing recommendations or guidelines in a real-life setting have some difficulties.

**Objectives:** The main objective of our network is to collect real-life clinical practice strategies (CliPS) from physicians all over the world taking care of KD patients into a library and compare the management strategies to future implementation in differential medical systems.

**Methods:** We performed an interim analysis of the online survey assessing treatment practices during the acute phase of KD and the management of patients with resistance to initial treatment. The project is funded as a COST (European Cooperation for Science and Technology) action CA21168. The questionnaire is distributed in English using different channels since September 2022.

**Results:** Between September 2022 and April 2023 137 questionnaires were sent. Responses from 113 physicians from 24 countries and five continents were collected. Most physicians were from Brazil (20%), Turkey (16%), and New Zealand (15%). Physicians from 9 subspecialties answered the survey, most frequently paediatric rheumatologists (48%). All physicians were involved in both inpatient and outpatient care and most of them (85%) were children professionals. Forty-seven percent of the respondents reported an experience with KD patients longer than 10 years and 96% of them agreed that all children with KD should be initially treated with IVIG. In initial therapy, 96% of responders used IVIG 2 g/kg and the majority (66%) prefer an aspirin dose of 30-50 mg/kg/day. Corticosteroids are mostly used as part of initial therapy with features of macrophage activation syndrome (MAS) (65%), with features of shock (60%), and in the presence of evolving coronary artery aneurysms (42%). Forty-two percent of physicians defined IVIG-resistant or refractory KD as persistent or recrudescent fever 48 hours after the end of the first IVIG infusion. A second dose of 2 g/kg IVIG without corticosteroids was used by 39% of responders.

**Conclusion:** Variations regarding the treatment and some aspects of KD diagnosis exist in real-life clinical practices. Future studies are warranted to define the best strategies to be applied to different medical systems.

**Patient Consent**

Not applicable (there are no patient data)

**Disclosure of Interest**

None declared

## P175 Thrombotic manifestations in pediatric behcet disease patients: a multicenter comparative study from Eurofever registry

### M. V. Mastrolia^1,2^, C. Matucci-Cerinic^3^, S. Ozen^4^, O. Kasapcopur^5^, C. Gaggiano^6^, I. Koné-Paut^7^, L. Cantarini^6^, P. Dusser^7^, Ü. Kaya-Akça^4^, M. Yildiz^5^, J. Brunner^8^, G. Filocamo^9^, R. Gallizzi^10^, A. Insalaco^11^, S. Pastore^12^, D. Rigante^13^, J. Sanchez-Manubens^14^, E. Tsitsami^15^, M. Gattorno^2,3^, G. Simonini^1,2^ on behalf of Eurofever Registry

#### ^1^Rheumatology Unit, ERN ReCONNET center, Meyer Children's Hospital IRCCS, Firenze; ^2^These authors equally contributed; ^3^Center of Autoinflammatory Diseases and Immunodeficiencies, Department of Pediatrics and Rheumatology, IRCCS Istituto G. Gaslini, Genova, Italy; ^4^Department of Pediatrics, Hacettepe University Faculty of Medicine, Ankara; ^5^Department of Pediatric Rheumatology, Cerrahpasa Medical School, Istanbul University-Cerrahpasa, Instanbul, Türkiye; ^6^Department of Medical Sciences, Surgery and Neurosciences, Rheumatology Unit, University of Siena and Azienda Ospedaliero-Universitaria Senese [European Reference Network (ERN) for Rare Immunodeficiency, Autoinflammatory and Autoimmune Diseases (RITA) Center], Siena, Italy; ^7^Department of Pediatric Rheumatology, Reference Centre for Autoinflammatory Disorders CEREMAI, Bicêtre Hospital, University of Paris SUD, Paris, France; ^8^Department of Pediatrics, Innsbruck Medical University, Innsbruck, Austria; ^9^Pediatric Immunoreumatology Unit, Fondazione IRCCS Ca’ Granda Ospedale Maggiore Policlinico, Milano; ^10^Department of Medical of Health Sciences, Magna Graecia University, Catanzaro; ^11^Division of Rheumatology, IRCCS Ospedale Pediatrico Bambino Gesù, Rome; ^12^Institute for Maternal and Child Health, RCCS “Burlo Garofolo” , Trieste; ^13^Department of Life Sciences and Public Health, Fondazione Policlinico Universitario "A. Gemelli" IRCCS, Rome, Italy; ^14^Pediatric Rheumatology, Section Pediatric Service, Parc Taulí Sabadell University Hospital, Institute for Research and Innovation I3PT, Sant Joan de Deu Hospital, Autonomous University of Barcelona, Barcelona, Spain; ^15^Pediatric Rheumatology Unit, 1st Department of Pediatrics, Children's Hospital "Aghia Sophia", University of Athens, Athens, Greece

##### **Correspondence:** M. V. Mastrolia

*Pediatric Rheumatology 2023*, **21(Suppl 2):**P175

**Introduction:** Arterial and venous thrombosis occurs in 6.6 to 38.4% of pediatric Behçet disease (BD) and cerebral sinus is one of the most involved districts.

**Objectives:** To report clinical features and outcomes of a pediatric BD multicenter cohort with thrombosis, to identify possible predictive factors of thrombosis.

**Methods:** A retrospective data collection of pediatric BD patients with thrombosis (T+) included in the EUROFEVER registry was conducted. Clinical data of BD patients without thrombosis (T-), belonging to the same contributing rheumatology units have been retrieved from EUROFEVER registry. T+ and T- groups were matched in a 2:1 ratio.

**Results:** 37 T+ patients were compared to 74 T- patients across 13 European pediatric rheumatology centers. BD onset occurred at a median age of 153 months (IQR±57) and 156 months (IQR±51) in the T+ and T- group, respectively. At onset, ICBD criteria fulfillment was significantly higher in the T- group compared to the T+ (p=0.015), whereas no differences were detected in ISG and PEDBD criteria frequencies. No gender differences were observed, Caucasian ethnicity significantly recurred in T- group while Middle Eastern in T+ group (p=0.002). HLA-B51 haplotype positivity was significantly reported in T- patients (p=0.04). At onset, pustolosis was most frequently observed in the T- group (p<0.001) as well as gastrointestinal symptoms (p<0.001) and ocular involvement (p=0.022). Conversely, neurological symptoms were more often described in T+ patients (p=0.034). As for T+ group, thrombosis was reported at BD presentation in 8/37 patients (29.6%). For the remaining patients, who developed thrombosis later in the disease course, the comparison of symptoms revealed that oral aphtosis (p=0.003), genital aphtosis (p=0.014) and posterior uveitis (p=0.050) were more frequently observed at BD onset than at thrombosis presentation, while pustolosis (p=0.02) and fever (p=0.019) significantly coexisted with thrombosis. Thrombosis type was mainly venous (26/37,70.3%), predominantly involving the cerebral sinuses (21/37, 56.8%). 16/29 (55.2%) T+ patients were on treatment before thrombosis onset with at least one systemic, non-steroid treatment. After thrombosis occurence, 35/37 (94.6%) T+ patients underwent or added an immunomodulatory treatment. 26/32 (81.3%) and 26/33 (21.2%) started anticoagulant and antiplatelet therapy, respectively. At a median follow up of 4 months (IQR±10), 9/28 (32.1%) T+ patients resolved thrombosis, 8/28 (28.6%) had a partial regression and 11/28 (39.3%) a persistence. A recurrence was reported in 6/31(19.4%) as venous thrombosis.

**Conclusion:** Middle Eastern ethnicity significantly recurred in T+ group, pustolosis and fever were more frequently concomitant to thrombosis. Neurological symptoms at onset could be related to thrombosis development in the BD course. Sinus veins were confirmed as the most frequent thrombosis site. Larger studies are required to better define predictive risk factors of thrombosis in pediatric BD.

**Patient Consent**

Yes, I received consent

**Disclosure of Interest**

None declared

## P176 Gender differences in clinical manifestations of pediatric behçet’s syndrome: cross-sectional analysis in a Portuguese cohort

### R. Nicolau^1,2^, T. Beirão^3^, F. Guimarães^4^, F. Aguiar^2,5^, S. Ganhão^5^, M. Rodrigues^2,5^, I. Brito^2,5^

#### ^1^Rheumatology, Centro Hospitalar Tondela-Viseu, Viseu; ^2^Medicine, Faculty of Medicine, University of Porto, Porto; ^3^Rheumatology, Centro Hospitalar Vila Nova de Gaia/Espinho, Gaia; ^4^Pediatric, Centro Hospitalar Entre Douro e Vouga, Santa Maria da Feira; ^5^Pediatric and Young Adult Rheumatology Unit, Centro Hospitalar e Universitário São João, Porto, Portugal

##### **Correspondence:** R. Nicolau

*Pediatric Rheumatology 2023*, **21(Suppl 2):**P176

**Introduction:** Behçet’s syndrome (BS) is a relapsing systemic disease with highest prevalence seen along the Silk Road. Similar to adult onset BS, pediatric onset is also seen equally in both genders. Gender differences in clinical manifestations have been reported, but scarce data are available.

**Objectives:** To characterize demographic and clinical features of a single-center cohort of portuguese BS patients with pediatric onset.

**Methods:** Longitudinal, retrospective, and descriptive study that included pediatric BS patients followed at our Pediatric and Young Adult Rheumatology Unit. Demographic and clinical data from medical records were collected retrospectively and the gender differences were evaluated.

**Results:** Twenty-four patients were included, mainly female patients (60.9%). Mean±SD age at symptom-onset and at diagnosis was 10.48±4.5 and 13.74±4, respectively. Those ages did not differ between two genders. All of the patients fulfilled International Criteria for BS, 18 (78.3%)

Pediatric BS group criteria and 16 (69.6%) fulfilled International Behçet’s Study Group criteria. No cases of familial history of BD were recorded. During disease course, oral aphtous ulcers (100%), genital ulcers (91%) and pseudofolliculitis lesions (47.8%) prevailed among the mucocutaneous signs, while the most common major organ-related manifestations were vascular and ocular involvement, with 7 (29.2%) cases uveitis including 3 (12.5%) cases of retinal vasculitis. Panuveitis (75%) was the predominant type. Vascular, ocular and joint involvement were significantly higher in males (p<0.001, p=0.021 and p=0.034, respectively). Although erythema nodosum (8.7%) and cardiac involvement (4.3%) were more common in males, the difference was not significant. Genital ulcers, pseudofolliculitis and gastrointestinal involvement were similar in two groups. Concerning the therapeutic approaches, conventional disease modifying anti-rheumatic drugs (cDMARDs) were the most frequently prescribed medication (91.3%), followed by colchicine (60.9%) and azathioprine (52.3%).

**Conclusion:** Our results are in line with what has been described in the literature. Ocular, vascular and joint manifestations had a higher frequency in pediatric males. This highlights importance of multidisciplinary monitoring to achieve better outcomes and avoid complications.

**Patient Consent**

Not applicable (there are no patient data)

**Disclosure of Interest**

None declared

## P177 Hypocomplementemic urticarial vasculitis: a pediatric case

### M. Peluso^1^, F. Orlando^2^, M. Tardi^3^, L. Martemucci^3^

#### ^1^Department of Translational Medical Science,, University of Naples “Federico II"; ^2^Department of General and Emergency Pediatrics, Santobono-Pausilipon Children’s Hospital, Pediatric Rheumatology Unit; ^3^Department of General and Emergency Pediatrics, Santobono-Pausilipon Children’s Hospital, Pediatric Rheumatology Unit, Naples, Italy

##### **Correspondence:** M. Peluso

*Pediatric Rheumatology 2023*, **21(Suppl 2):**P177

**Introduction:** Urticarial vasculitis (UV) is a rare cutaneous vasculitis of small vessels (SVV) characterized by wheal-like lesions that tend to last more than 24 hours, healing with a residual ecchymotic postinflammatory hyperpigmentation. The histopathologic pattern is definide as leukocytoclastic vasculitis. UV can be limited to the skin or may be associated with constitutional symptoms, including fever and arthralgias, and involvement of other organs such as kidneys, lungs, gastrointestinal tract, and eyes. The Chapel Hill Consensus Conference (CHCC) 2012 included the hypocomplementemic urticarial vasculitis (HUV) (anti-C1q vasculitis) in the group of ‘immune complex SVV’. HUV was defined as ‘a form of vasculitis accompanied by urticarial and hypocomplementemia affecting small vessels and associated anti-C1q antibodies’. HUV is a rare disease in children.

**Objectives:** To describe a case of pediatric HUV.

**Methods:** A 2 years old male infant was admitted to our hospital with urticarial erythematous rash lasting 9 days, bilateral non-secreting conjunctivitis, erythema and cracking of lips. His recent medical history was remarkable for fever 5 days before (lasting 6 days). Blood tests showed elevation of inflammatory indexes and hypocomplementemia (reduced C3 and undosable C4). The clinic and blood tests were suggestive of hypocomplementemic ANCA-negative urticarial vasculitis. Urine tests, abdominal ultrasound, cardiac examination and ocular fundus excluded complications of vasculitis. Secondary forms were excluded with negative ANA, anti dsDNA, anti-cardiolipin, antiphospholipid antibodies, normal expression of immune system, absence of signs of acute Sars-COV-2 infection. Unfortunately, it was not possible to detect anti-C1q antibodies.

**Results:** Patient underwent prednisone 1 mg/kg/die orally for one week, discontinuated in three weeks. The rash disappeared and C3 and C4 levels were progressively normalized in one month.

**Conclusion:** Urticaria-like lesions, palpable purpura, petechiae and angioedema are characteristic features of HUV. Systemic involvement includes arthralgias and myalgias, arthritis, kidney involvement (proteinuria or hematuria), ocular involvement (episcleritis, uveitis, and conjunctivitis) and gastrointestinal symptoms, including abdominal pain, nausea, vomiting, and diarrhea. Laboratory marker of HUV is hypocomplementemia, with a profile indicative of classical pathway activation (reduced C1, C2,C4, C3). CHCC addendum distinguished HUV from the normocomplementemic urticarial vasculitis, a skin-limited vasculitis, not accompanied by systemic involvement. This form of vasculitis is associated with normocomplementemia and absence of anti-C1q antibodies. Even if our patient did not perform the skin biopsy and dosage of anti-C1q antibodies, the cutaneous picture, the systemic involment and the hypocomplementemia were suggestive of HUV.

**Patient Consent**

Yes, I received consent

**Disclosure of Interest**

None declared

## P178 Central Nervous System (cns) vasculitis, an unreported feature in activated phosphoinositide 3-kinase delta syndrome 1 (APDS 1) treated with Haematopoietic Stem Cell Transplant (HSCT)

### A. Prabhudesai^1^, N. P. Maldar^1^, A. Khan^1^, H. Panwala^2^, R. Khubchandani^1^

#### ^1^Pediatric Rheumatology; ^2^Pediatric Radiology, SRCC Childrens Hospital, Mumbai, India

##### **Correspondence:** A. Prabhudesai

*Pediatric Rheumatology 2023*, **21(Suppl 2):**P178

**Introduction:** APDS (PASLI disease) (**p**110d-**A**ctivating mutation causing **S**enescent T cells, **L**ymphadenopathy, **I**mmunodeficiency) is a primary immunodeficiency due to heterozygous GOF mutations in PI3Kδ catalytic p110δ or PIK3R1 genes causing APDS1 and APDS2 respectively.Digital vasculitis with C1q deficiency is reported in APDS2.Vasculitis not been reported in APDS1.

**Objectives:** To highlight a unique case of APDS1 with CNS vasculitis successfully treated with HSCT

**Results:** A 4-year-old girl (14 kg / 91 cm) born to a non-consanguineous marriage, presented with acute onset weakness of the left upper limb and lower left half of face. MRI brain showed large right sided fronto-parieto-temporal and smaller chronic gliotic infarcts in the right MCA and MCA-ACA watershed territories. There was focal segmental stenosis of multiple intracranial arteries suggesting CNS vasculitis. History also revealed recurrent respiratory infections, severe dental caries and an episode of severe hand foot mouth disease during infancy. The leucocyte count was 6090 with absolute lymphocytes count of 518. Absolute B lymphocytes (197) T lymphocytes (311) and NK cells (10) were all reduced. Immunoglobulins levels were normal. ANCA antibodies were negative and ADA2 activity was normal. On whole exome sequencing a pathogenic missense mutation, **E**1021K c.3061G>A (p. Glu1021Lys**)** in PIK3CD gene was identified. Infection screen for viruses and blood cultures were negative. Antimicrobial prophylaxis, monthly IVIG and anticoagulation were commenced. With a 100% HLA match in the sibling, HSCT was offered and performed as a curative option with sirolimus used in the interim. Fourteen months after HSCT, she has achieved 100 % chimerism and has had no infections or vasculitis features. There is improved anthropometry (19 kg/98 cms), with residual weakness in the left upper limb. A repeat MRI brain after 14 months shows no fresh lesions.

**Conclusion:** Our case adds CNS vasculitis to the wide phenotype of the ultra rare APDS 1 and to the list of monogenic vasculitides. We report success with HSCT.

**Patient Consent**

Yes, I received consent

**Disclosure of Interest**

None declared

## P179 Unravelling the environmental etiology of Kawasaki disease in Japan: insights from spatiotemporal patterns

### A. Fontal^1^, S. Pozdniakova^1^, S. Borràs^1^, J. C. Burns^2^, Y. Nakamura^3^, X. Rodó^1^

#### ^1^Climate and Health, ISGlobal, Barcelona, Spain, ^2^Pediatrics, UCSD School of Medicine, La Jolla, United States, ^3^Pediatrics, Jichi Medical Hosp., U. Tokyo, Tokyo, Japan

##### **Correspondence:** X. Rodó

*Pediatric Rheumatology 2023*, **21(Suppl 2):**P179

**Introduction:** Kawasaki disease (KD), a systemic vasculitis primarily affecting children under five years old, has remained an enigma despite over half a century of extensive research. The aetiology of KD is thought to be multifactorial, involving various environmental, biological, and chemical factors. The prevailing theory suggests an unidentified agent, entering via the upper respiratory tract, triggers a severe immunological response in genetically susceptible children. Building on previous research that links tropospheric winds to KD incidence (Rodó et al, 2014), this study delves into the consistency of spatial distribution in KD incidence changes at the prefecture level.

**Objectives:** This study aims to discern whether temporal shifts in KD incidence at the prefectural level in Japan exhibit spatial coherence. The focus is twofold: (1) the nationwide epidemic events of 1979, 1982, and 1986, and (2) the pronounced seasonal patterns observed from 2000 onwards.

**Methods:** Utilising 26 Japanese nationwide surveys to date (Ae et al, 2022), we compiled daily case counts and estimated daily incidence at the prefectural level from 1979 to 2020, encompassing a total of 404,960 cases. These data were then used to assess temporal and spatial correlations through pairwise Pearson Correlation, Global Moran's I, and hierarchical clustering.

**Results:** Our analysis reveals significant spatial autocorrelation at the prefectural level for KD incidence, albeit inconsistent across the entire temporal domain. The spatiotemporal dynamics of the 1982 and 1986 epidemics present distinct profiles: the former exhibits a single wave across numerous prefectures, while the latter displays two separate waves in two distinct clusters of prefectures.

**Conclusion:** The results of this study align with the hypothesis that a common agent, prevalent in similar environments, could be a significant driver of KD incidence. Further investigation is required to pinpoint the specific environmental agents involved and elucidate their role in KD etiology. This study offers a fresh perspective on potential KD aetiology and may inform future research and public health strategies.

**Patient Consent**

Not applicable (there are no patient data)

**Disclosure of Interest**

None declared

**References**

1. Rodó, X., Curcoll, R., Robinson, M., Ballester, J., Burns, J. C., Cayan, D. R., ... & Morguí, J. A. (2014). Tropospheric winds from northeastern China carry the etiologic agent of Kawasaki disease from its source to Japan. Proceedings of the National Academy of Sciences, 111(22), 7952-7957.
2. Ae, R., Makino, N., Kuwabara, M., Matsubara, Y., Kosami, K., Sasahara, T., & Nakamura, Y. (2022). Incidence of Kawasaki Disease Before and After the COVID-19 Pandemic in Japan: Results of the 26th Nationwide Survey, 2019 to 2020. JAMA pediatrics, 176(12), 1217-1224.

## P180 HLA-B51 and clinical manifestations in Juvenile behçet´s syndrome

### T. Beirão^1^, R. Nicolau^2^, F. Guimarães^3^, F. Aguiar^4^, S. Ganhão^4^, M. Rodrigues^4^, I. Brito^4^

#### ^1^Rheumatology, Centro Hospitalar Vila Nova de Gaia/Espinho, Vila Nova de Gaia; ^2^Rheumatology, Centro Hospitalar Tondela-Viseu, Viseu; ^3^Pediatrics, Centro Hospitalar Entre Douro e Vouga, Santa Maria da Feira; ^4^Pediatric and Young Adult Rheumatology Unit, Centro Hospitalar Universitário São João, Porto, Portugal

##### **Correspondence:** M. Rodrigues

*Pediatric Rheumatology 2023*, **21(Suppl 2):**P180

**Introduction:** Behçet's syndrome (BS) is a rare, chronic, multisystem inflammatory disorder of unknown etiology. The age of onset is typically between 20 and 30 years, but 15-20% of patients have onset before the age of 16 (juvenile BS). The human leukocyte antigen (HLA) B51 has been linked to BS in several populations [1]. However, the association between HLA-B51 and clinical manifestations in JBD is not well established.

**Objectives:** The objective of this study is to investigate the association between HLA-B51 and clinical manifestations in JBS.

**Methods:** We conducted a retrospective cohort study of 15 JBD patients followed in a tertiary hospital. HLA-B51 genotyping was performed using a PCR-based method. We compared the clinical manifestations and treatments between HLA-B51 positive and negative patients.

**Results:** Of the 15 patients, 10 (66.67%) were HLA-B51 positive. The HLA-B51 positive group had a later disease onset compared to the HLA-B51 negative group (mean age: 11.70 vs. 7.00 years, p=0.036). The HLA-B51 positive group also had a lower frequency of GI manifestations compared to the HLA-B51 negative group (0.00% vs. 40.00%, p=0.032) and a higher frequency of use of cDMARDs (90.00% vs. 20.00%, p=0.007).

**Conclusion:** Our study suggests that HLA-B51 is associated with a later disease onset, fewer GI manifestations, and increased use of cDMARDs in JBD patients. These findings may have important clinical implications for the diagnosis and management of JBD, especially in populations with a high prevalence of HLA-B51. The study with highest number of patients linked HLA-B51 with genital ulcers, ocular involvement and skin manifestations [2]. Further studies with larger sample sizes and more diverse populations are needed to confirm these results and investigate the underlying mechanisms of the observed associations.

**Patient Consent**

Not applicable (there are no patient data)

**Disclosure of Interest**

None declared

**References**

1. Bettencourt, A., et al., *New insights of HLA class I association to Behcet's disease in Portuguese patients.* Tissue Antigens, 2008. 72(4): p. 379-82.
2. Yildiz, M., et al., *Pediatric Behcet's Disease.* Front Med (Lausanne), 2021. 8: p. 627192.

## P181 Paediatric cogan´s syndrome. Literature review and practical approach to diagnosis and management

### K. Rücklová^1^, T. von Kalle^1^, A. Koitschev^1^, K. Gekeler^1^, M. Scheltdorf^1^, A. Heinkele^1^, F. Blankenburg^1^, I. Kötter^2^, A. Hospach^1^

#### ^1^Klinikum Stuttgart, Stuttgart; ^2^University Hospital Hamburg-Eppendorf and Clinic for Rheumatology and Immunology Bad Bramstedt, Hamburg, Germany

##### **Correspondence:** K. Rücklová

*Pediatric Rheumatology 2023*, **21(Suppl 2):**P181

**Introduction:** Cogan syndrome is a rare vasculitis characterized by interstitial keratitis and vestibular impairment accompanied by sensorineural hearing loss.

**Objectives:** To collect all published paediatric Cogan´s syndrome cases with their clinical characteristics, disease course, treatment and treatment outcome in order to design a practical diagnostic work-up and the best therapeutical strategy for these patients.

**Methods:** All published case reports of paediatric Cogan´s syndrome were identified in PubMed with the keywords „Cogan syndrome“ and „children“ or „childhood“. The cohort was supplemented with our own patient. Data on clinical manifestations, treatment modalities and outcome were extracted from the case reports, analyzed and presented as absolute numbers and percentage.

**Results:** Altogether, 55 paediatric cases of Cogan´s syndrome were identified. All patients suffered from inflammatory ocular and vestibulo-auditory symptoms. In addition, 32/55 (58%) manifested systemic symptoms with musculoskeletal involvement being the most common with a prevalence of 25/55 (45%), followed by neurological and skin manifestations. Aortitis was detected in 9/55 (16%). Most patients (44/54, 81%) were treated with systemic corticosteroids. Methotrexate was used in 14/54 (26%) and only 2 patients obtained biologics. Regarding prognosis, remission in ocular symptoms was attained in 33/48 (69%), whereas only 16/52 (32%) achieved a significant improvement in auditory function. Mortality was 2/55 (4%).

**Conclusion:** This study presents an analysis of the largest cohort of paediatric Cogan´s syndrome patients. Based on the collected data, the first practical guide to a diagnostic work-up, including laboratory tests, imaging such as echocardiography and gadolinium- enhanced MRI of the brain and inner ear is provided. Given the relatively bad outcome of the auditory function, early use of biologics in patients with sensorineural hearing loss should be considered.

**Patient Consent**

Yes, I received consent

**Disclosure of Interest**

None declared

## P182 An unusual presentation of vasculitis

### E. Sundberg

#### Pediatric Rheumatology, Karolinska, Stockholm, Sweden

##### **Correspondence:** E. Sundberg

*Pediatric Rheumatology 2023*, **21(Suppl 2):**P182

**Introduction:** This 14 years old boy of Caucasian origin presented at our pediatric emergency room with abdominal pain since three weeks and suspicion of constipation. Occational vomiting had been noted and supposed weight loss without knowledge of prior weight.

Medical history included allergic rhinitis since age of 8, and more recent right sided hearing disability and tinnitus with otosaphingitis diagnosis by Ear Nose and Throat specalist 3 months before admittance to emergency room.

**Results:** At the emergency room visit blood sampling revealed markedly elevated plasma pancreatic amylas levels of 43 microkat/L (ref.1,15-1,1) och plasma Lipase 46 microkat/L (ref. 022-1,0). CRP 69. Blood cell count, liver enzymes were normal. INR1.4 slightly raised. Kreatinin was normal and urinalyses showed lowest amount of red blood cells, +1. Working diagnosis was pancreatitis and the boy were admitted for in-patient investigations. The following day Ultrasound indicated a rounded expansive 5 cm tumor later verified by MRI. Images were consistant with a pseudo papillary pancreatic tumor or less likely a pancreatoblastoma.

Decision for exstirpation of the tumor was made and after 3 weeks in the hospital the tumor was removed.

PAD of the tumor showed inflammatory infiltrates, granulomatous inflammation and necrotising vasculitis. IgG4 staining indicated IgG4 related disease. No signs of malignancy were noted i any parts of the speciments. Patient were diagnosed with Granulomatous Poly Arthritis (GPA) with IgG4 overlap disease based on above findings, presence of intense rinitis/sinuitis with bloody discharge och minor lung infiltrate on CT scan together with markely raised PR 3 antibodies level > 800 E/L (ref. <1).

Anti inflammatory treatment first with cyclophosphamide and high dose methylprednisone later per oral prednisone and rituximab in mono therapy has been successful with no sign of disease relapse after 2 years.

**Conclusion:** Vasculidities can be hard to diagnose for a number of reasons including unusual presentation like this round shaped tumor causing abdominal pain. A broad differential diagnostic approch is of importance when signs of inflammatory disease and in case of inflammatory small or even bigger tumors IgG4 related disease should be suspected and investigated.

**Patient Consent**

Yes, I received consent

**Disclosure of Interest**

None declared

## P183 Disseminated tuberculosis versus granulomatosis with polyangiitis: a challenging diagnosis

### S. Tangcheewinsirikul, T. Kanjanaphan

#### Paediatrics, Navamindradhiraj University, Bangkok, Thailand

##### **Correspondence:** S. Tangcheewinsirikul

*Pediatric Rheumatology 2023*, **21(Suppl 2):**P183

**Introduction:** Granulomatosis with polyangiitis (GPA) is a rare systemic vasculitides affecting small-vessel predominant that involve multiple organ systems. Tuberculosis (TB) which caused by *Mycobacterium tuberculosis*, is a significant cause of morbidity and mortality worldwide, particularly in endemic area. Pulmonary TB is the most common form of the diseases; however, it can also involve other parts of the body – disseminated tuberculosis (DTB), leading to diagnostic challenges and might delayed treatment.

**Objectives:** We present a case of DTB mimicking GPA in paediatric patient.

**Methods:** Diagnostic investigation including basic and immunological blood tests, tissue immunology and pathology were conducted.

**Results:** A 15-year-old girl presented with a 1-month history of joint swelling, epistaxis, haemoptysis, vasculitis rashes and weight loss. On examination, she revealed marked swelling at her left ankle and localized vasculitis lesions along both legs (Fig 1A). Her chest x-ray was done and demonstrated patchy infiltration at right upper lung (Fig 1B). GPA was one of the possible diagnoses according to pulmonary infiltration, arthritis, and vasculitis rashes. Laboratory investigation revealed an elevated inflammatory response. Anti-neutrophil cytoplastic antibodies were negative: MPA-ANCA 0.1 U/ml (0-3.5), PR3-ANCA 0.2 U/ml (0-2.0). Several consolidations with an air bronchogram together with reticulonodular opacities with tree-in-bud pattern scattering throughout both lungs were reported on her chest CT (Fig 1C) and accidentally finding a hypodense lesion at the right lobe of the liver was detected (1.7 x 2.0 x 2.4 cm). The bronchoalveolar lavage was done and revealed a negative acid-fast stain. Immunological profiles returned with positive TB-DNA PCR which confirmed the diagnosis of DTB. An anti-TB regimen including isoniazid, rifampicin, ethambutol, and pyrazinamide was prescribed. Her symptoms have gradually improved.

**Conclusion:** DTB can presented with a wide range of clinical features which can overlap those of GPA and should be considered in the differential diagnosis of patients with granulomatous disease, particularly in endemic areas. The diagnosis of DTB can be challenging, particularly in patients with extrapulmonary involvement. Diagnostic tests such as tissue biopsy, culture, and molecular testing could aid in the diagnosis. Clinicians should maintain a high index of suspicion for TB and perform thorough diagnostic investigations to ensure timely and appropriate treatment. Prompt diagnosis and management are encouraged for improving outcomes in patients with DTB.

**Patient Consent**

Yes, I received consent

**Disclosure of Interest**

None declared

## P184 Presentation of a teenage girl with persistent headache with the diagnosis of Primary Angiitis of the Central Nervous System (PACNS)

### S. Taximi^1^, S. Mouskou^1^, A. Korona^1^, V. Ziaka^1^, G. Vartzelis^2^, A. Stamati^2^, G. Velonakis^3^, S. Vassilopoulou^4^, K. Voudris^1^, O. Vougiouka^5^

#### ^1^Pediatric Neurology Department; ^2^Second Department of Pediatrics of the National and Kapodistrian University of Athens, Children's Hospital "P. & A. Kyriakou"; ^3^Second Radiology Department of the National and Kapodistrian University of Athens, Aiginitio Hospital; ^4^First Neurological Department, Eginitio Hospital, National and Kapodistrian University of Athens; ^5^Rheumatology Outpatient Clinic, Second Department of Pediatrics of the National and Kapodistrian University of Athens, Children's Hospital "P. & A. Kyriakou", Athens, Greece

##### **Correspondence:** O. Vougiouka

*Pediatric Rheumatology 2023*, **21(Suppl 2):**P184

**Introduction:** The diagnosis of primary central nervous system (CNS) vasculitis is often difficult. There are neither specific clinical features nor a classical clinical course, and no blood or imaging investigations that can confirm the diagnosis. Cerebral vasculitis is a descriptive term rather than a specific disease, referring to inflammation within the wall of central nervous system (CNS) blood vessels associated with destructive changes, occlusion and infarction.(1)

**Objectives:** To present a case of PACNS presenting with only headaches and non specific symptoms and to show how challenging a PACNS diagnosis can be due to its non-specific symptoms and lack of universally accepted diagnostic criteria.

**Methods:** A 15 year old female with unremarkable medical background presented with frontal headaches and episodes of mild dizziness and weakness, 2-3 times per week, starting six months ago. Her physical and neurologic examination was normal. The brain MRI revealed multiple lesions in white matter (high intensity in T2 and Flair) in both hemispheres, ganglia and brainstem, reminiscent of demyelinating lesions, while the MRA and spine MRI were normal.

**Results:** The patient underwent an extensive laboratory investigation. The blood tests were normal but the CSF presented cells and high protein, as well as oligoclonal band type 4, consistent with systemic immune reaction. She was also tested for systemic immune diseases and the results came back negative. Due to the worsening of the symptoms, a second MRI was performed three weeks later including vessel wall imaging (Black Blood MRI) which revealed an increase of the brain lesions and vessel wall inflammation. Since primary systemic vasculitic syndrome and other alternative possible diagnoses were excluded, the MRI evidence of vasculitis led to PACNS diagnosis. The teenager was treated with high doses of methylprednisolone (30 mg/kg) for 5 days followed by a 12-week tapering, aspirin and vitamin D and showed immediate clinical improvement. At the 3-month follow-up she had no symptoms and a smaller number of lesions in the MRI scan. However, in the six-month follow-up the brain MRI showed deterioration (without concomitant clinical deterioration) and, as a result, she received a second round of methylprednisolone pulses and rituximab as adjunctive therapy.

**Conclusion:** Primary CNS vasculitis remains a difficult-to-reach diagnosis. According to the latest proposed criteria (1), it is important to exclude other possible diagnoses and to have histopathologic evidence of vasculitis. Alternatively, the diagnosis of PACNS is possible with CSF pleocytosis and high protein levels with imaging evidence of vessel wall inflammation. Early recognition is crucial for patients’ prognosis.

**Patient Consent**

Yes, I received consent

**Disclosure of Interest**

None declared

**References**

1. Rice CM, Scolding NJThe diagnosis of primary central nervous system vasculitis, Practical Neurology 2020;20:109-114.

## P185 Pulmonary presentation of kawasaki disease: a diagnostic challenge

### S. Thakur^1^, A. Sharma^2^, A. MS^2^

#### ^1^Dr. RPGMC Tanda, Kangra, India; ^2^Department of Paediatrics, Dr. RPGMC Tanda, Kangra, India

##### **Correspondence:** S. Thakur

*Pediatric Rheumatology 2023*, **21(Suppl 2):**P185

**Introduction:** Kawasaki Disease (KD) is a medium vessel vasculitis predominantly affecting children. It has a predilection for coronary artery and is hence the leading cause of heart disease in children. Although its cause remains unknown, a well-defined diagnostic criteria has been established by American Heart Association. Cases with involvement of respiratory, gastrointestinal, central nervous, genitourinary and musculoskeletal systems are also seen, although they are rare.

The following is a case report of Kawasaki disease with empyema, an unusual involvement of the pulmonary system.

**Methods: Case Report**

A 7 year old male child presented to hospital with fever, cough and difficulty in breathing since 1 week. On examination, chest on right side was dull on percussion with reduced breath sounds and ultrasonography suggestive of pleural effusion. Pus was drained and its gram staining showed scanty gram positive cocci. He was started on meropenem, ceftriaxone and vancomycin, continued for 20 days without any significant improvement. On 20^th^ day, patient started developing maculopapular rash over the whole body, palmoplantar desquamation of skin, edema of hands and feet and was then referred to our hospital.

At the time of presentation, fever was documented at 102^o^F. Patient had pallor, generalized lymphadenopathy, chest was dull on percussion with edema on hands and feet. The desquamation of skin had progressed to other parts of the body. Subsequent pus culture came out sterile. Inflammatory markers were raised. The patient was given Intravenous Immunoglobulins (IVIG) which led to clinical improvement and no fever spikes were recorded after its administration. Echocardiography showed small aneurysm with a Z-score of LCA=3.9, RCA=4.43. ceftriaxone and meropenem were substituted by linezolid.

**Conclusion:** Although Kawasaki disease predominantly presents with mucocutaneous manifestations, the involvement of other systems is always a possibility. This can lead to misdiagnosis and mistreatment, leading to worsening of the patient’s symptoms over time.

The given case emphasizes the importance of considering KD as a diagnosis in children with persistent fever who have not responded to antibiotics and/or in cases where the disease does not follow an expected clinical course. Another inference that can be drawn is that KD might be triggered by a preceding infection thus hinting towards the role of superantigens in the etiology. The case report also highlights the importance of Echocardiography as a diagnostic tool in such obscure cases.

**Patient Consent**

Yes, I received consent

**Disclosure of Interest**

None declared

**References**

1. McCrindle BW, Rowley AH, Newburger JW, et al.; American Heart Association Rheumatic Fever, Endocarditis, and Kawasaki Disease Committee of the Council on Cardiovascular Disease in the Young; Council on Cardiovascular and Stroke Nursing; Council on Cardiovascular Surgery and Anesthesia; Council on Epidemiology and Prevention. Diagnosis, treatment, and long-term management of Kawasaki disease: a scientific statement for health professionals from the American Heart Association [published correction appears in *Circulation*. 2019;140(5):e181–e184]. 2017;135(17):e927-e999.
2. Singh S, Gupta A, Jindal AK, Gupta A, Suri D, Rawat A, Vaidya PC, Singh M. Pulmonary presentation of Kawasaki disease-A diagnostic challenge. Pediatr Pulmonol. 2018 Jan;53(1):103-107. doi: 10.1002/ppul.23885. Epub 2017 Sep 26. PMID: 28950425; PMCID: PMC7167766.
3. Arslanoglu Aydin E, Demir S, Aydin O, Bilginer Y, Ozen S. Pleural effusion as an atypical presentation of Kawasaki disease: a case report and review of the literature. J Med Case Rep. 2019 Nov 25;13(1):344. doi: 10.1186/s13256-019-2284-4. PMID: 31760956; PMCID: PMC6876070.
4. Ramamoorthy JG, Anantharaj A. Pulmonary Presentation of Atypical Kawasaki Disease. Indian J Pediatr. 2021 Apr;88(4):413-414. doi: 10.1007/s12098-020-03591-7. Epub 2021 Jan 8. PMID: 33415549; PMCID: PMC7790719.
5. Leahy TR, Cohen E, Allen UD. Incomplete Kawasaki disease associated with complicated Streptococcus pyogenes pneumonia: A case report. Can J Infect Dis Med Microbiol. 2012 Fall;23(3):137-9. doi: 10.1155/2012/638357. PMID: 23997782; PMCID: PMC3476559.

## P186 Kawasaki disease and severe skeletal infection by MSSA

### A. Koloi^1^, M. Tsolia^1^, I. Eleftheriou^1^, S. Oikonomou^1^, A. Chatzikyriakos^2^, A. Catsouli^2^, C. Zampakides^2^, A. Doudoulakakis^3^, E. M. Dimitrakopoulou^3^, E. Bozavoutoglou^3^, O. Vougiouka^1^

#### ^1^Second Department of Pediatrics, National and Kapodistrian University of Athens; ^2^First Department of Orthopaedics; ^3^Microbiology Department, “P.& A. Kyriakou” Childrens’ Hospital, Athens, Greece

##### **Correspondence:** O. Vougiouka

*Pediatric Rheumatology 2023*, **21(Suppl 2):**P186

**Introduction:** Although its causal factors and entire pathogenesis remain elusive, accumulating evidence indicates that the pathogenesis of Kawasaki Disease could be associated with dysregulated innate immune response to various infectious agents in genetically predisposed children. A possible pathogen proposed as the trigger is *Staphylococcus aureus*, some strains of which produce superantigens, such as Toxic Shock Syndrome Toxin-1 (TSST-1).

**Objectives:** We present a toddler with severe skeletal infection by methicillin-susceptible *S.aureus* (MSSA), associated with bacteremia, which manifested features of Staphylococcal scalded skin syndrome (SSSS) and Kawasaki disease (KD).

**Methods:** Case report of Kawasaki Disease triggered by Staphylococcus

**Results:** A previously healthy 3-year-old male, with a recent history of leg injury, presented with high-grade fever for five days and rash. On admission, physical examination was remarkable for polymorphous rash, erythema and edema of palms and soles, erythematous lips and strawberry tongue. The patient had elevated CRP, anemia, hypoalbuminemia and hyponatremia. The echocardiogram revealed Z-score of the coronary arteries >2 (LM ~3,3mm, LAD ~2,5-2,8mm, RCA ~2,2mm). On high clinical suspicion of Incomplete KD, the patient was treated with intravenous immune-globulin (IVIG) of 2g/kg.Meanwhile, blood cultures developed MSSA and the patient presented progressive, cutaneous erythema and desquamation, considered to be a toxin-mediated manifestation; thus, he was treated with intravenous cloxacillin and clindamycin. Clinical examination 24 hours after initiation of antibiotics revealed antalgic position of the left lower limb and restriction of motion of the hip. Ultrasonography and MRI demonstrated septic arthritis of the hip joint with femur pandiaphysitis. The patient underwent multiple surgical drainages. On 15^th^ day of fever, the diagnosis of KD was established as he developed periungual desquamation of the hands and feet, alongside with thrombocytosis (platelet count: 918000/μl). A second IgG infusion of 2 gr/Kg was administered along with antiplatelet aspirin dose to which he immediately responded. The patient was discharged 40 days after hospitalization with cardiologic, rheumatologic and orthopedic follow-up. Coronal and large vessels ultrasound were normal. Staph-genes clfA, hla, sea and sec were identified. Staphylococcal enterotoxins A & C (transcripted by sea & C respectively) have been described as superantigens.

**Conclusion:** This case could be an example supporting the infectious theories of the pathogenesis of KD described. Furthermore, it shows the complexity of differential diagnosis and the consequent necessity of constant vigilance and review in clinical practice.

**Patient Consent**

Yes, I received consent

**Disclosure of Interest**

None declared

**References**

[i] Nakamura A, Ikeda K, Hamaoka K. Aetiological Significance of Infectious Stimuli in Kawasaki Disease. Front Pediatr. 2019 Jun 28;7:244
[ii] Nagata S. Causes of Kawasaki Disease-From Past to Present. Front Pediatr. 2019 Feb 5;7:18.

## P187 Scurvy: a forgotten differential diagnosis of musculoskeletal pain in children

### R. Amalia, D. Muktiarti, N. Kurniati

#### Allergy Immunology Division, Child Health Department, Cipto Mangunkusumo Hospital-Faculty of Medicine, Universitas Indonesia, Jakarta, Indonesia

##### **Correspondence:** R. Amalia

*Pediatric Rheumatology 2023*, **21(Suppl 2):**P187

**Introduction:** Vitamin C deficiency or scurvy is a disease with prominent musculoskeletal symptoms that could mimic rheumatologic diseases. Even though scurvy is rare in developed countries, it is still found quite often in less resourceful countries.^1^

**Objectives:** To describe clinical characteristics, management, and outcome of children with scurvy at Cipto Mangunkusumo Hospital, Indonesia.

**Methods:** Retrospective study was performed from February 2022 to April 2023. All children who were diagnosed with scurvy enrolled into this study.

**Results:** From February 2022 to April 2023, twelve children were diagnosed with scurvy. The majority are boys (2/12) with median age is 5.8 years and time to diagnosis range is 3.5 months (1-12 months). All children were referred to our hospital with other diagnosis. Six children were diagnosed with JIA, 2 children with SLE, and 2 children with transverse myelitis. The last two children were diagnosed with Tuberculous spondylitis and osteomyelitis. Nine children had moderate or severe malnutrition. Neurodevelopmental conditions were found in four children.

Lower limb pain, limping, and subsequently refusal to walk occurred in all children. Arthritis was only found in three children. Four children experienced back pain. Almost all children had malaise (11/12) and loss of appetite (10/12). Mucosal and cutaneous symptoms were less common. Seven children had gingival hypertrophy with spontaneous gum bleeding. Petechiae or ecchymosis was only found in 2 children.

Most common laboratory abnormalities were anemia. Six children had severe anemia with initial hemoglobin varied from 4.3-8.3 g/dL. Ascorbic acid level only examined in 2 children with the results were both <0.1 mg/dL. Plain X-rays of femur and genu were obtained from all children. Calcification at metaphysis (Frenkel line) happened in all children. Ten of them had scorbutic zones. Pelkan spur and periosteal hemorrhage were found in 4 and 2 children.

Oral ascorbic acid (100 mg three times daily) was given to all children. One child was lost to follow up. From the rest gum bleeding, gum hypertrophy, and musculoskeletal pain were resolved in the first 2 weeks. Limping was gradually resolved in 2-4 months.

**Conclusion:** Scurvy should be considered as a differential diagnosis in children presenting with musculoskeletal pain, gum hypertrophy and bleeding. Especially if children had malnutrition or neurodevelopmental problems.

**Patient Consent**

Not applicable (there are no patient data)

**Disclosure of Interest**

None declared

**References**

1. Trapani S, Rubino C, Indolfi G, Lionetti P. A narrative review on pediatric scurvy: the last twenty years. Nutrients. 2022;14(3):684.

## P188 Mixed connective tissue disease in children: observational study from one center

### M. Kaleda, A. Arefieva, I. Nikishina, N. Yudkina, Z. Verizhnikova

#### Pediatric, V. A. Nasonova Research Institute of Rheumatology, Moscow, Russian Federation

##### **Correspondence:** A. Arefieva

*Pediatric Rheumatology 2023*, **21(Suppl 2):**P188

**Introduction:** Mixed connective tissue disease (MCTD) is a rare systemic connective tissue disease in children. In the international literature, we have found not so many studies of MCTD with juvenile onset.

**Objectives:** to describe the clinical and immunological characteristics, treatment of children with MCTD, hospitalized to our pediatric rheumatology center.

**Methods:** The study included all patients (pts) with MCTD in the period between 2019 and 2023 years. We tested pts with anti-RNP+ against 4 different sets of criteria for MCTD: Kasukawa’s, Alarcon-Segovia, Kahn and Sharp criteria. The patient was included in the analysis if he met at least one set of criteria.

**Results:** 18 pts (17 girls) fulfilled of criteria for MCTD (56,25% of all pts with anti-RNP+): Kahn's criteria - 15 pts, Sharp's criteria - 9, Kasukawa's criteria - 10, Alarcon-Segovia criteria – 8. 4 pts met only one set of criteria, 6 pts – 2 sets of criteria, 6 pts – 3 sets, 2 pts – all 4 sets. The most common combination of criteria – Sharp’s + Kahn’s (8 pts) or Alarcon-Segovia + Kahn’s (8 pts). The median age at onset of MCTD was 12.25 years [IQR 9.7; 13.9]. Clinical symptoms included arthritis (100%), various skin lesions (94.4%), Raynaud's phenomenon (88.9%), lymphadenopathy (72.2%), fatigue (50%), lung involvement (38.8%), myopathy (27.8%), nephritis and myocarditis (11.1% each). Skin involvement included sclerodactyly (7 pts), telangiectasias (2), malar rash (5), heliotrope rash and Gottron's papules (4), erythema nodosum (1), livedo reticularis (2). Sjögren's syndrome (SS) was diagnosed in 17 pts (94.4%), 15 had isolated involvement of salivary glands, 2 – combined with lacrimal glands. Sicca syndrome was occurred in 12 pts. All pts had anti-RNP+ in titre of >200 U/l. All pts had antinuclear antibodies (ANA) in titer ≥1/1280. Speckled (sp) type of ANA was isolated in 77.8% of pts, mixed type (h+sp+cytoplasmic) – 22.2%. Other autoantibodies included anti-DNA+ (4 pts), anti-Ro+ (4), anti-La+ (1), anti-Sm+ (4). RF+ was in 6 pts. Hypergammaglobulinemia was verified in 10 pts. Capillaroscopic changes in the nailfold were noted in 77.8%: 5 pts – nonspecific abnormalities, 5 pts – an early scleroderma pattern, 3 – a late scleroderma pattern with a myopathic component and 1 – a changes characteristic of DM. The most common combination of features of MCTD included Raynaud's phenomenon, arthritis, SS, lymphadenopathy and hypergammaglobulinemia (9pts, 50%). All pts received GC per os in low dose, 9 pts – hydroxychloroquine, 7 – methotrexate, 3 – MMF, 12 pts (66.7%) received biologics: 3 – rituximab, 8 – abatacept, 1 – belimumab with good efficacy and safety.

**Conclusion:** Most of pts with MCTD in our study met the criteria of Kahn. Our results demonstrate possible different combinations of sets of criteria. Only 2 patients met all sets of criteria, which indicates the need to use several sets of criteria when verifying MCTD. The combination of Raynaud's phenomenon, arthritis, SS, lymphadenopathy and hypergammaglobulinemia had a half of pts with MCTD. According our data biologics is efficacy and safety option in children with MCTD.

**Patient Consent**

Yes, I received consent

**Disclosure of Interest**

None declared

## P189 Juvenile recurrent parotitis and response to hydroxychloroquine

### S. Balan^1^, A. Sasidharan^1^, P. M. Krishna^1^, B. Faisal^2^

#### ^1^Rheumatology & Clinical Immunology; ^2^ENT, Amrita Institute of Medical Sciences, Kochi, India

##### **Correspondence:** S. Balan

*Pediatric Rheumatology 2023*, **21(Suppl 2):**P189

**Introduction:** Juvenile Recurrent Parotitis (JRP) is the recurrent inflammatory parotitis in children of unknown etiology, commonly beginning between 3 and 6 years of age. JRP could be the first presenting symptom of an underlying immunodeficiency, HIV infection and Sjogren syndrome. The presentation of Sjogren Syndrome in children is very different from adults such that the exisiting American European Classification Criteria for Sjogren Syndrome are rarely fulfilled by pediatric patients. The need to study the clinical profile of these children and work towards criteria for diagnosis is necessary for understanding this disorder.

**Objectives:** The objective of our study was to assess clinical ,laboratory ,imaging and lip biopsy status of children presenting with juvenile recurrent parotitis, their response to therapeutic sialendoscopy and lavage followed by immunomodulation with hydroxychloroquine.​

**Methods:** A retrospective and prospective review of medical records of all paediatric patients diagnosed with JRP presenting to our ENT department from Jan 2016 to present was conducted. ​

18 children with Juvenile recurrent parotitis were evaluated. Mean age was 5 years. ​

Out of these 6(33.3%) were boys and 12(66.6%) were girls. ​

Demographic, clinical and history data were assessed including age of onset, number of episodes, sicca symptoms ENA profile, USG report, Focus score and Ophthalmological assessment including Schirmer test.

**Results:** Out of all 18 patients assessed, along parotid swelling 1. 4(22.5%) of them had recurrent fever spikes as the presenting symptom​

1. 7(38.8%) had dry eyes with positiveschrimer,​

1 had dry mouth and 1 had active arthritis. ​

4.ANA was positive in 9(50 %) out of the 18, ENA profile was negative in all. ​

5.All of them underwent lip biopsy with two having focus score more than 1. ​

6.Sialadenoscopy and lavage was performed in all, once in 9 , twice in seven and thrice in two .​

7.After commencement of Hydroxychloroquine there was no further recurrence in any child.​

**Conclusion:** Our cohort of patients with JRP, following clinical and laboratory evaluation, diagnostic sialadenoscopy with lip biopsy and lavage were then commenced on Hydroxycholoroquine. More than 50% had recurrence of symptoms following the first lavage, but had no further recurrence after the initiation of HCQ. Regular follow up and larger studies are required to understand this condition in children better as well as the effectiveness of HCQ in these patients. Creation of registries will contribute to the same.​

**Patient Consent**

Not applicable (there are no patient data)

**Disclosure of Interest**

None declared

**References**

1. Katz P.,et al. Treatment of juvenile recurrent parotitis. *Otolaryngol Clin North Am.* 2009;42:1087–1091
2. Coziana Ciurtin, Cho et al. Barriers to translational research in Sjögren's syndrome with childhood onset: challenges of recognising and diagnosing an orphan rheumatic disease, The Lancet Rheumatology,Volume 3, Issue 2,2021
3. Basiaga ML, Stern SM, et al; Childhood Arthritis and Rheumatology Research Alliance and the International Childhood Sjögren Syndrome Workgroup. Childhood Sjögren syndrome: features of an international cohort and application of the 2016 ACR/EULAR classification criteria. Rheumatology (Oxford). 2021 Jul 1;60(7):3144-3155.
4. Breanne L. Schiffer, et al, Sjögren's syndrome in children with recurrent parotitis, International Journal of Pediatric Otorhinolaryngology,Volume 129,2020

## P190 A study on cytokine release syndrome in children with viral infections at a tertiary care center in Eastern India

### J. N. Bathia^1^, M. Arjumand^2^, P. Khatua^3^, P. Pal^1^

#### ^1^Pediatric Rheumatology; ^2^Pediatric Medicine; ^3^Pediatric Infectious Diseases, Institute of Child Health, Kolkata, India

##### **Correspondence:** J. N. Bathia

*Pediatric Rheumatology 2023*, **21(Suppl 2):**P190

**Introduction:** Infection as a cause of Cytokine release syndrome (CRS) is now increasingly seen. In the recent upsurge of viral lower respiratory tract infections (LRTI) in the city during winter and spring of 2023, many patients were noted to suffer from CRS. Not all satisfied the HLH 2004 criteria especially the cut off for cytopenias but required specific anti-inflammatory therapies. With no available consensus guidelines on hypercytokinemic states we intend to describe the clinical features, laboratory findings and response to therapy in such patients.

**Objectives:** To note the demographic profile, clinical features, laboratory investigations, therapy and outcome of cytokine release syndrome due to viral lower respiratory tract infections

**Methods:** Retrospective analysis of data of all children who had cytokine release syndrome due to viral LRTI admitted from January 2023 to March 2023, at Institute of Child Health, Kolkata. CRS was diagnosed on basis of persistent continuous fever, multisystem involvement, falling platelets hemoglobin and absolute neutrophil count, transaminitis, and elevated ferritin in the background of a viral infection and negative cultures.

**Results:** Twenty patients had viral LRTI induced CRS during the study period. The median age was 36 months, 13 were males, 8 were females. 14 were positive for Adeno virus, 1 for human metapneumovirus and 1 Corona 229e. 13 were males, 8 were females. All had continuous fever, 17 had tachypnoea, 17 CNS involvement in the form of irritability, lethargy and one had seizures, 15 had oedema, 19 had hepatomegaly and 9 had splenomegaly. Median day of fever at admission was 4 days and median day of fever at diagnosis of CRS was 13 days. Median investigations at diagnosis was Hemoglobin 9.15 mg/dl, Total leukocyte count 4490/cmm, Absolute neutrophil count 2088, Platelet 1.15 lakhs/cmm, C-Reactive Protein (CRP) 27.6 mg/L, Aspartate aminotransferase (AST) 116.5 U/l, Albumin 3.05 g/dl, Ferritin 2954 ng/ml, Triglyceride 162mg/dl. CRP was noted to be normal at diagnosis in 7 patients.

18 patients received intravenous dexamethasone, one received hydrocortisone and one required no intervention. One child was non responsive to dexamathasone and had to be initiated on Intravenous methyl prednisolone. Dexamethasone was given at 10 mg/m2 for 3 to 4 days followed by tapering over next 9 to 10 days. Of all the symptoms fever was the first to respond to therapy. After initiation of steroids, in 3 patients there was no further fever, amongst the rest defervescence was noted within 12 hours in 4, within 24 hrs in 8, within 48 hrs in 1, >48 hrs in 3 patients. Encephalopathy and oedema subsided later at 4 to 5 days after initiation of therapy. All patients were successfully discharged. On follow up, only 1 child had recurrence of fever and cough after 10 days that was diagnosed as secondary bacterial pneumonia and responded to antibiotics.

**Conclusion:** High degree of suspicion is required to diagnose CRS. Criteria of HLH-2004 maybe not fulfilled. Multisystem involvement, trends in cytopenia with rising ferritin, transaminitis and fall in albumin helps in early identification. CRP may not always be raised. Early identification and initiation of immunosuppression is the key to a successful outcome

**Patient Consent**

Yes, I received consent

**Disclosure of Interest**

None declared

## P191 Sjogren syndrome: single disease different presentations

### J. N. Bathia^1^, S. Poddar^2^, R. Sinha^2^, P. Pal^1^

#### ^1^Pediatric Rheumatology; ^2^Pediatric Nephrology, Institute of Child Health, Kolkata, India

##### **Correspondence:** J. N. Bathia

*Pediatric Rheumatology 2023*, **21(Suppl 2):**P191

**Introduction:** Juvenile Sjogren Syndrome is a rare entity with only a few hundred reported cases. Due to varied clinical presentation diagnosis is challenging and often delayed or undiagnosed. We report 5 cases of Juvenile onset Sjogren Syndrome with different presentations including 2 with distal renal tubular acidosis (dRTA)

**Results: Case 1** 5yr8mo old girl, presented with recurrent bilateral parotitis for 2 years. No other systemic features, no ocular or oral symptoms. ANA, Anti Ro and Anti La negative. Schirmer test was positive, minor salivary gland biopsy was suggestive of lymphoplasmocytic infiltration with focus score 1. Methotrexate (Mtx) and Hydroxychloroquin sulfate (HCQs) was started. Subsequently, due to inadequate response mycophenolate mofetil was added (MMF). Three months later the patient started responding.

**Case 2** 7 yrs old girl, presented with intermittent fever and arthritis of bilateral wrist, PIP and DIP of fingers. No other systemic features, no ocular or oral symptoms. ANA 1:1000 homogeneous, Anti Ro Positive, RF positive. Schirmer test was positive and minor salivary gland biopsy was suggestive of Chronic plasmocytic sialoadenitis. Mtx and HCQs was started. She responded within 3 month of therapy. After 9 months she developed Raynaud’s like symptoms requiring nifedipine.

**Case 3**: 9 yrs male, presented with intermittent fever, facial erythematous rashes and bilateral parotid enlargement for 1 month. No other symptoms. Eye evaluation was normal. ANA 1:1000 speckled, Anti Ro Positive, Anti La Positive, RF positive. Skin biopsy showed Inflammatory dermatosis. Mtx was started. Responded within 1 month of therapy.

**Case 4**: 2yr7mo old girl, presented with inability to walk, listlessness for 6 months. Weight and height were below 3^rd^ centile and she was noted to have bilateral genu valgum with features of rickets. Investigations were suggestive of distal renal tubular acidosis (dRTA) with autoimmune hemolytic anemia with proteinuria. Kidney biopsy showed minimal change disease. SLE was ruled out. No other systemic features, no ocular or oral symptoms. ANA 1:160 speckled, Anti Ro Positive, Anti La Positive, RF positive. Eye evaluation was normal. MMF and HCQs started along with management for dRTA. Three months later the patient started responding.

**Case 5**: 10 old girl with a past history of recurrent immune thrombocytopenia presented with fever, polyarthralgia and limb pain. Investigations were suggestive of dRTA. Kidney biopsy showed interstitial nephritis. SLE was ruled out. No other systemic features, no ocular or oral symptoms. ANA 1:1000,Anti Ro Positive. Eye evaluation was normal. MMF and HCQs started along management for dRTA. Three months later the patient started responding

**Conclusion:** Juvenile Sjogren Syndrome is a rare heterogenous disease. Presenting features are varied including Distal renal tubular acidosis. With no validated criteria clinical diagnosis remains pivotal.

**Patient Consent**

Yes, I received consent

**Disclosure of Interest**

None declared

## P192 Endothelial function in children with a history of Paediatric Inflammatory Multisystem Syndrome (PIMS) post-COVID-19 – case/control study

### Y. Butbul Aviel, N. M. Merkulocv, A. Blinzovski

#### Pediatric Rheumatology Service Department of Pediatrics B, Ruth Rappaport Children's Hospital, Rambam Medical Center, Haifa, Israel

##### **Correspondence:** Y. Butbul Aviel

*Pediatric Rheumatology 2023*, **21(Suppl 2):**P192

**Introduction:** The acute infection with SARS-CoV-2 is generally mild in children, whilst the post-infectious manifestations, including paediatric inflammatory multisystem syndrome (PIMS) is more complex.

The pathogenesis of PIMS is still not clear but there were some evidence for endothelial injury during the acute phase.

The long term effect of PIMS is still not clear.

**Objectives:** The aim of our study was to evaluate the endothelial function in children that were recovered from PIMS.

**Methods:** This research was an observational prospective study. The study group comprised of 17 children diagnosed with PIMS. The minimum interval between the diagnosis with PIMS and endothelial testing was 3 months. Endothelial function evaluation was assessed by endothelin level and by a noninvasive technology named peripheral arterial tonometry, using an EndoPAT™ device. This method measures blood flow in the limb, in response to arterial occlusion, and calculates a Reactive Hyperemic Index (RHI) as an index of endothelial function. RHI values of the study group were compared to those of a known control group.

Data regarding clinical features treatment and outcome of patients with PIMS were retrospectively collected.

**Results:** Seventeen children and adolescents with PIMS underwent endothelial function studies at average 393±260 days post PIMS. Endothelial function was compared to that of a known control group comprising of 18 healthy children and adolescents. The two groups had similar characteristics, , male to female ratio, and BMI but were differ in age (11.8±1.04 vs 14.8±2.2 p-0.049). The mean LNRHI was similar 0.44±0.34 in the study group and 0.45±0.23 in the control group no correlation was found between LNRHI and laboratory or clinical features.

Endothelin levels were abnormal in 6/15 (40%) and was correlated with total days of hospitalization (r-0.685 p-0.002), platelet count on admission (r-0.685 p-0.002), maximal ferritin levels (r-0.494 p-0.044), ALT and albumin levels (r-0.658 p-0.004 for both).

**Conclusion:** Our data suggest that patients with PIMS may have endothelial dysfunction that may last long time following the acute phase larger studies are need to confirm our findings.

**Patient Consent**

Yes, I received consent

**Disclosure of Interest**

None declared

## P193 Clinical, laboratory and imaging manifestation of Juvenile Sjögren Syndrome: a case series from a single medical center in Detroit, Michigan

### B. Fathalla^1,2^

#### ^1^Allergy, immunology and rheumatology, Children's Hospital of Michigan, Detroit; ^2^College of Medicine , Central Michigan University, Mount Pleasant, United States

##### **Correspondence:** B. Fathalla

*Pediatric Rheumatology 2023*, **21(Suppl 2):**P193

**Introduction:** Juvenile Sjögren's syndrome is a rare and possibly underdiagnosed condition in children. The exact incidence and prevalence are unknown and spectrum of clinical manifestations is variable and defers from adult-onset disease.

**Objectives:** To report spectrum of clinical, laboratory and imaging manifestations of patients with juvenile Sjögren's syndrome following in a single medical center.

**Methods:** A retrospective review of medical records of children with Sjögren's syndrome following since year 2020. Data collection was approved by IRB committee.

**Results:** Patients are five females and one male. Four patients are African Americans and two are Caucasians. Median age at onset was 10.5 years (range 8 – 12) and median current age is 14 years (range 9 – 17). Most common clinical manifestations were recurrent painful parotid swelling in five patients, sicca symptoms and dental carries in two patients, rashes in two patients (discoid lupus and urticarial rash), cervical lymphadenopathy in two patients, and arthralgia and arthritis in two patients. Renal biopsy in one patient with mild high creatinine showed mild plasma cell infiltrate with no evidence for acute tubular injury or tubulointerstitial nephritis. Evidence of lung disease included asymptomatic changes on pulmonary function test in one patient, and abnormal chest CT findings in one patient with an episode of acute hemoptysis and difficulty breathing. Work up included ANA positivity in all patients, anti-SSA and anti-RNP positivity in five patients, anti-Smith and rheumatoid factor positivity in three patients, anti-DsDNA positivity in two patients, low C3 and C4 in one patient, double positive antiphospholipid antibodies positivity in one patient, and negative ANCA and anti-SSB in all patients. Also, high IgG and soluble IL-2 receptor were noted in four patients, borderline high ACE in three patients and normal IgG4 in five patients. TBNK flowcytometry was unremarkable. All had chest x-ray, echocardiography, pulmonary function tests, and abdominal ultrasound as part of their evaluation. Head ultrasound study in all patients showed parotid glands enlargement with heterogenicity, calcifications and/or cystic changes. These were also noted on head MRI done in three patients. Four patients had minor salivary gland biopsy showing lymphocytic infiltrates consistent with Sjögren's syndrome. The clinical course was primary Sjögren in four patients and possible secondary Sjögren in two patients with features of evolving SLE. Treatment included systemic steroids, plaquenil, methotrexate and/or mycophenolate mofetil with significant improvement in four patients and less success in two patients due to refusal of medications.

**Conclusion:** Diagnosis of juvenile Sjögren's Syndrome is challenging. There is no widely accepted classification criteria and differential diagnose is extensive requiring several investigations to rule out other diseases. Head ultrasound studies are proving to be a reliable diagnostic tool, are readily available, and practical for follow up.

**Patient Consent**

Not applicable (there are no patient data)

**Disclosure of Interest**

None declared

## P194 Chest CT scan as a screening tool to detect lung involvement in pediatric Rheumatic diseases

### S. Prountzos^1^, K. Kourtesi^2^, D. Moriki^3^, O. Sardeli^3^, A. Mazioti^1^, E. Alexopoulou^1^, K. Douros^3^, L. Fotis^2^

#### ^1^Department of Radiology; ^2^Department of Pediatrics, Division of Pediatric Rheumatology; ^3^Department of Pediatrics, Division of Pediatric Pulmonology, National and Kapodistrian University of Athens, ATTIKON General University Hospital, Athens, Greece

##### **Correspondence:** L. Fotis

*Pediatric Rheumatology 2023*, **21(Suppl 2):**P194

**Introduction:** Pulmonary involvement in rheumatic diseases may affect more than one component of the respiratory system, such as the airways, vessels, parenchyma, pleura, and respiratory muscles, and can be related to the illness itself, medications used, or may manifest secondary to infections amongst others (1-4).

**Objectives:** The study aimed to evaluate the association between respiratory symptoms, spirometry results, and chest computed tomography (CT) findings in children with rheumatic diseases and pulmonary involvement.

**Methods:** Chest CT scans of children with rheumatic diseases performed in our tertiary center between 01/01/2021 and 31/12/2022 were retrospectively reviewed. All patients underwent chest CT examination using paired end-inspiratory/ forced-expiratory scan protocol using 1-mm collimation and slice thickness reconstruction algorithm. Parenchymal opacities, ground-glass opacities, reticular pattern, honeycombing, parenchymal bands, bronchiectasis, and peribronchial wall thickening were examined. Air trapping was assessed during expiratory scans. Symptoms and spirometry results were reviewed. Correlations between clinical findings and spirometric parameters (FEV1% and FVC%) were checked with the Spearman test. All children had no history of chronic lung disease or recent lower respiratory tract infections.

**Results:** Fifteen children (13 females and 2 males) with a median age of 14 years had radiologic evidence of lung involvement. Four children were diagnosed with systemic lupus erythematosus, 2 with systemic sclerosis, 2 with juvenile dermatomyositis, 2 with juvenile idiopathic arthritis, 2 with mixed connective tissue disorder, 1 with microscopic polyangiitis, 1 with granulomatosis with polyangiitis and 1 with non-specific vasculitis. Five (33%) patients had parenchymal opacities, reticular pattern, and parenchymal bands, 9 (60%) had ground-glass opacities, 13 (86%) had peribronchial wall thickening of varying degrees, 7 (47%) had bronchiectasis, 8 (53%) had air-trapping in the form of mosaic attenuation during expiratory scans, and 1 (6%) had honeycombing. Most patients were free of respiratory symptoms. Five (33.3%) patients reported early fatigue during exercise and 3 (20%) reported a dry cough. None of them reported dyspnea. Spirometry revealed a restrictive pattern in 3 (20%) patients while only 1 (6%) patient had an obstructive pattern. We found no association between spirometric indices and the presence of cough or fatigue.

**Conclusion:** Mild symptoms such as dry cough, or early fatigue during exercise may be associated with extensive findings of pulmonary alterations. It is common to detect lung involvement even in asymptomatic patients. Clinical assessment and spirometry may not detect lung involvement and chest CT may be warranted to prove lung disease.

**Patient Consent**

Yes, I received consent

**Disclosure of Interest**

None declared

**References**

1. Richardson AE, Warrier K, Vyas H. Respiratory complications of the rheumatological diseases in childhood. Arch Dis Child. 2016 Aug;101(8):752–8.
2. Tarantino G, Esposito S, Andreozzi L, Bracci B, D’Errico F, Rigante D. Lung Involvement in Children with Hereditary Autoinflammatory Disorders. Int J Mol Sci. 2016 Dec;17(12): 2111.
3. Ramphul M, Gallagher K, Warrier K, Jagani S, Bhatt JM. Why is a paediatric respiratory specialist integral to the paediatric rheumatology clinic? Breathe (Sheffield, England). 2020 Dec;16(4):200212
4. Ruano CA, Lucas RN, Leal CI, Lourenço J, Pinheiro S, Fernandes O, et al. Thoracic manifestations of connective tissue diseases. Curr Probl Diagn Radiol. 2015 Jan;44(1):47–59.

## P195 Imaging characteristics and presentation patterns of chronic non-bacterial osteomyelitis in a cohort of greek pediatric patients using whole-body magnetic resonance imaging

### E. Kostoula^1^, S. Prountzos^2^, K. Kourtesi^3^, A. Kourti^4^, V. Bizimi^2^, E. Atsali^3^, V. Papaevangelou^1^, S. Fessatou^4^, O. Papakonstantinou^2^, L. Fotis^3^

#### ^1^Department of Pediatrics; ^2^Department of Radiology; ^3^Department of Pediatrics, Division of Pediatric Rheumatology; ^4^Department of Pediatrics, Division of Pediatric Gastroenterology, National and Kapodistrian University of Athens, ATTIKON General University Hospital, Athens, Greece

##### **Correspondence:** L. Fotis

*Pediatric Rheumatology 2023*, **21(Suppl 2):**P195

**Introduction:** Chronic nonbacterial osteomyelitis (CNO) is a rare inflammatory bone disorder affecting children and adolescents. Whole-body magnetic resonance imaging (WB-MRI) has emerged as a useful tool for the evaluation of CNO due to its ability to provide detailed imaging of the entire skeletal system (1).

**Objectives:** The primary objective of this study was to describe the WB-MRI findings at the time of diagnosis in a series of Greek children with CNO. The secondary objective was to evaluate the diagnostic utility of WB-MRI in detecting the extent and severity of bone involvement in these patients.

**Methods:** The whole-body MR imaging (WBMRI) studies of all children with a final diagnosis of CNO from September 2018 to April 2023 were retrospectively reviewed. The working diagnosis of CNO for all children had been based on the “Bristol criteria” (2). WBMRI included coronal T1 and short-tau inversion recovery (STIR), whole spine sagittal STIR, and ankle/foot sagittal STIR images. High signal intensity lesions on STIR images corresponding to bone marrow edema were recorded. The WB-MRI images were reviewed by two experienced radiologists, and the findings were analyzed for the distribution, extent, and severity of bone involvement.

**Results:** Thirty seven Greek children (19 females and 18 males, mean age; 11 years, range; 6-16years) participated in the study. The WB-MRI findings showed that all but 1 patient had multiple bone lesions (mean number; 11 lesions), 5 children had paucifocal (2-4 lesions) disease, while the rest of the patients (n=31, 83.8%) had multifocal bone involvement. Only one patient was submitted to a bone biopsy. The most affected sites were tibia (64.9%), femur and calcaneus (51.4% each), tarsal bones (48.6%), metatarsals (45.9%), talus (43.2%) and fibula (40.5%), while carpal bones and ulna were rarely affected (2.7% each). Spine lesions localized in the cervical, thoracic, lumbar and sacral vertebrae, were documented in 9 patients (24%), with sacral lesions in 6 of them. Bilateral metaphyseal and epiphyseal involvement with transphyseal extension were common, but the periosteal reaction and well-defined lesion margins were rare.

**Conclusion:** Frequent involvement of the foot and ankle and substantial spinal involvement in almost a quarter of the patient population were seen in Greek pediatric patients with CNO. Furthermore, the widespread bone involvement detected by WB-MRI highlights the importance of a whole-body imaging approach in the evaluation of CNO sparing the need for other diagnostic tests like bone biopsy that is a necessity only in the case of monofocal disease.

**Patient Consent**

Not applicable (there are no patient data)

**Disclosure of Interest**

None declared

**References**

1. Andronikou S, Kraft JK, Offiah AC, Jones J, Douis H, Thyagarajan M, et al. Whole-body MRI in the diagnosis of paediatric CNO/CRMO. Rheumatology (Oxford). 2020 Oct 1;59(10):2671–80.
2. Roderick MR, Sen ES, Ramanan A V. Chronic recurrent multifocal osteomyelitis in children and adults: current understanding and areas for development. Rheumatology (Oxford). 2018;57(1):41–8.

## P196 Arthritis as the presenting manifestation of childhood leukemia

### V. Fraga^1^, C. Abreu^1^, S. Sousa^1^, M. J. Santos^1,2^

#### ^1^Rheumatology, Hospital Garcia de Orta, Almada; ^2^Unidade de Investigação em Reumatologia, Instituto de Medicina Molecular, Faculdade de Medicina, Universidade de Lisboa, Centro Académico de Medicina de Lisboa , Lisboa, Portugal

##### **Correspondence:** V. Fraga

*Pediatric Rheumatology 2023*, **21(Suppl 2):**P196

**Introduction:** Childhood leukemia can present with musculoskeletal manifestations, such as joint pain, swelling and limping, mimicking Juvenile Idiopathic Arthritis (JIA)^1^. Distinguishing between the two conditions can be challenging and requires high clinical suspicion.

**Objectives:** The objective of this study was to characterize children who presented with arthritis as the first manifestation of acute leukemia.

**Methods:** We performed a retrospective study of children referred to the pediatric rheumatology outpatient clinic over the past 15 years with arthritis and who were subsequently diagnosed with leukemia. Demographic characteristics, clinical manifestations, initial laboratory findings and outcomes are reported.

**Results:** A total of 3 patients were identified, two girls and one boy. The mean age at clinical presentation was 6± 4.3 years and the mean interval between joint symptoms and leukemia diagnosis was of 5 ± 1 months.

All patients presented with limb pain, limping and arthritis, with a polyarticular pattern in 2 of them. The most common joints involved were the knee, wrist and the ankle.

Other symptoms such as weight loss, anorexia or skin rash were present in 2 patients.

Regarding laboratory results, cytopenias were observed in 2 patients and only 1 presented peripheral blast cells. One patient had no hematologic abnormalities in the initial investigation. Elevated inflammatory markers were present in all patients and the mean ESR was 107±16 mm/h. The patients’ demographic characteristics, clinical features, laboratory results and outcomes will be presented in **table 1.**

The most frequent diagnosis was B-cell Acute Lymphocytic Leukemia (B-ALL) (n=2) and one patient was diagnosed with Acute Myeloid Leukemia (AML) through skin lesion biopsy. Regarding clinical outcomes, one patient is in remission, one is currently receiving chemotherapy and one died.

**Conclusion:** Arthritis and limb claudication can be the initial manifestations of childhood leukemia. Large joints were mostly affected and more frequently in a polyarticular pattern. Hematologic abnormalities are not always present, especially peripheral blast cells.

To conclude, the presence of persistent constitutional symptoms, sudden onset of bone or joint pain, hepatosplenomegaly and abnormal laboratory results should raise suspicion for childhood leukemia over JIA, particularly B-ALL, which has been more frequently associated with musculoskeletal involvement^1^

**Patient Consent**

Yes, I received consent

**Disclosure of Interest**

None declared

**References**

1. Kittivisuit, S., Sripornsawan, P., Songthawee, N. *et al.* Musculoskeletal involvement in childhood leukemia: Characteristics and survival outcomes. *Pediatr Rheumatol* **20**, 34 (2022). 10.1186/s12969-022-00692-9

## P197 Objective structured clinical examination to certify pediatric rheumatologists

### F. García-Rodríguez^1^, S. Muñoz López^1^, C. Hernández Díaz^1^, B. A. Mota Mondragón^1^, H. E. Fragoso Loyo^1^, M. Vazquez del Mercado Espinosa^1^, A. Alpuche Hernandez^2^, S. Mendieta Zerón^1^, A. R. Torres Jiménez^1^, E. Faugier Fuentes^1^, A. Vargas Guerrero^1^, M. Pérez Cristóbal^1^, C. I. Meléndez Mercado^1^, N. Rubio^1^, P. B. Lara Herrera^1^

#### ^1^Mexican Board of Rheumatology; ^2^Universidad Nacional Autónoma de México, Mexico, Mexico

##### **Correspondence:** F. García-Rodríguez

*Pediatric Rheumatology 2023*, **21(Suppl 2):**P197

**Introduction:** Objective structured clinical examination (OSCE) is an evaluation method based on the simulation of representative clinical scenarios. The OSCE assesses individual performance, considering knowledge, skills, and attitudes during practice, to determine if a physician is competent to serve as a pediatric rheumatologist. Since 2003, the Mexican Board of Rheumatology certifies pediatric rheumatologists as a legal requirement to practice their profession in Mexico, and since 2013 the OSCE has been part of the evaluation for this purpose.

**Objectives:** To describe the process of design, validation, and results of the implementation of the new version of the OSCE to certify pediatric rheumatologists in Mexico.

**Methods:** Between 2020 and 2022, the Mexican Board of Rheumatology members worked to design an OSCE with 5 case scenarios to be implemented during the certification process. For each case, several standardized documents were made, such as a table of specifications, rating forms (rubric), examiner guide, scripts for standardized patients, and candidate instructions. Validation was performed using face validity within experts, and statistically using classic test theory methods (internal consistency, difficulty index, and discrimination index). Scenarios were piloted in two separate moments (December 2021 and December 2022) and the OSCE was taken in January 2023.

**Results:** The five scenarios for the OSCE were cases with spondyloarthropathy, polyarthritis, inflammatory myopathy (IM), and two connective tissue disorders (CTD), which were evaluated using 54 items, and the grade was later expressed on a 0 – 100 scale. This was first piloted with one pediatric rheumatologist as a candidate and 9 examiners, then, adequations were made and a second pilot was performed with 5 candidates (2 general practitioners, 1 pediatrician, and 2 pediatric rheumatologists) and 12 examiners.

Finally, the OSCE was taken place in January 2023, with 6 candidates, all female, aged between 30 and 34. Internal consistency was acceptable (Cronbach alpha 0.78) and most of the items showed adequate discrimination indexes. The overall grade was 71.1 (62.9 – 79.8), with higher notes in the polyarthritis case (77.3 [60.6 – 86.3]) and lower in IM (65.1 [50 – 80.3]). Those competencies related to communication, professionalism, and ethics showed higher scores compared with clinical abilities and treatment decisions (83.3 [52 - 100] vs 66.7 [33 – 89]). Skills related to past medical history were those with the lower scores in the candidates. Feedback was given to the candidates and program directors.

**Conclusion:** The OSCE is a powerful tool to determine the competency of professionals who aimed to practice pediatric rheumatology, showing detailed results about the performance of each candidate. The information collected will help the programs improve their training methods and future pediatric rheumatologists fill the gaps in knowledge and clinical skills.

**Patient Consent**

Not applicable (there are no patient data)

**Disclosure of Interest**

None declared

**References**

1. Curran et al. The pediatric rheumatology objective structured clinical examination: progressing from a homegrown effort toward a reliable and valid national formative assessment. Pediatric Rheumatology (2019) 17:5.
2. Pascual Ramos et al. Performance of an objective structured clinical examination in a national certification process of trainees in rheumatology. Reumatol Clin. 2015;11(4):215–220.
3. Martínez González, Trejo Mejía. ¿Cómo realizar un ECOE? Inv Ed Med;7(28):99-107.

## P198 A 3-year old child with persistent lameness

### M. F. Gicchino^1^, A. N. Olivieri^1^, G. Di Mauro^1^, G. Mondillo^1^, E. Miraglia del Giudice^1^, L. A. Nasto^2^

#### ^1^Department of Woman, Child and General and Specialized Surgery; ^2^Department of Orthopaedics, University of Campania Luigi Vanvitelli, Napoli, Italy

##### **Correspondence:** M. F. Gicchino

*Pediatric Rheumatology 2023*, **21(Suppl 2):**P198

**Introduction:** Limp is a symptom very common in childhood, it could depend on inflammatory, infective, orthopaedic, neoplastic diseases.

**Objectives:** To report a case of persistent lameness in a 3 years old child.

**Methods:** A 3 years old child, was admitted to our Pediatric Department because of lameness. The symptom began one week previously following an upper respiratory tract infection. When patient came to our observation the articular examination revealed pain, functional limitation of the left hip and lameness. Left hip ultrasound revealed joint effusion. Hip transitory synovitis was diagnosed and therapy with NSAIDs was prescribed. During NSAIDs treatment pain improved, but lameness persisted, so patient was hospitalized to perform further investigations. Left hip X ray revealed radiolucency and fragmentation of the left femoral neck. Blood examinations showed an increasing in C-reactive protein (5 mg/dl; normal value< 1mg/dL) and erythrocyte sedimentation rate (25mm/h; normal value <20); complete blood count liver, kidney function, iron levels were in the norm. Both Antinuclear antibodies (ANA) and extractsble nuclear antibodies (ENA) were negative. Virological screening (including Sars-Cov2) was negative. Quantiferon test was negative. Urinalysis was in the norm. Faecal calprotectin was negative. Both heart and abdomen ultrasound were in the norm. Suspecting ahip septic arthritis/ osteomyelitis were performed both a blood colture and a colture of synovial fluid. Meanwhile colture results treatment with Ceftriaxone and Clyndamicin was started. Both blood and synovial coltures were negative, and despite antibiotic treatment lameness persisted so pelvis MRI was performed.

**Results:** Pelvis MRI revealed at the left femur: coxa vara characterized by a homogenous signal intensity at the proximal epiphysis, not showing structural alterations or volumetric reduction. An enlargement of the space between the capital femoral epiphysis and the proximal femur was detected. A migration of femoral epiphysis and a superior dislocation of the proximal femur were also detected. MRI findings were compatible with "developmental coxa vara".

**Conclusion:** Developmental coxa vara is a rare condition, with an incidence of 1 in 25000 births. Developmental coxa vara is characterized by a primary cartilaginous defect in the femoral neck with an abnormal neck-shaft angle, shortening of the femoral neck and shortening of the affected lower limb. Typically, the deformity does not manifest until after birth and usually not until walking age. Clinically the child presents with a painless limp resulting from a combination of true Trendelenburg gaited relatively minor limb length inequality in unilateral cases. Easy fatigability or pain around the gluteal muscles may be a complaint. Our patient is in follow up at orthopaedic clinic of our Department in order to established the adequate surgical treatment.

**Patient Consent**

Yes, I received consent

**Disclosure of Interest**

None declared

## P199 Remitting seronegative symmetrical synovitis with pitting oedema in a young adolescent

### J. B. Lima^1^, C. Zilhão^2^, S. Alves^2^

#### ^1^Paediatrics; ^2^Rheumatology Paediatric Unit, Centro Materno-Infantil do Norte (CMIN) do Centro Hospitalar Universitário de Santo António (CHUdSA), Porto, Portugal

##### **Correspondence:** J. B. Lima

*Pediatric Rheumatology 2023*, **21(Suppl 2):**P199

**Introduction:** Remitting seronegative symmetrical synovitis with pitting oedema (RS3PE) is a rare clinical entity characterized by symmetrical tenosynovitis of both hands and ankles with pitting oedema, negative rheumatoid factor (RF), absence of radiographic erosions and excellent response to low dose steroids. It is usually associated to elderly patients, but it may occur in younger patients.

**Objectives:** To highlight the importance of recognizing RS3PE in pediatric patients through a case report.

**Methods:** Clinical details were retrospectively collected using routine clinical records.

**Results:** A healthy 17-year-old female presented with a 3-month history of pain and swelling of both hands (involving wrist and proximal interphalangeal joints), feet (involving ankle) and knees, with morning stiffness. She denied any constitutional symptoms, chronic cough, rash, ophthalmological complaints, Raynaud’s phenomenon, diarrhoea, genitourinary symptoms, recent infection, or taking any drugs. There was no relevant family history. On examination, oedema of the dorsal surface of the hand extending up to the wrist was observed, as well as, pitting oedema of the feet extending up to the knees. Joint’s examination revealed symmetrical tenderness and swelling, with limited range of motion, in the wrists, proximal interphalangeal joints, ankles and knees, with bilateral positive patellar tap test and a heel to buttock distance of 9 cm. There was no axial involvement nor features of enthesitis. Radiologic evaluation of the hands did not reveal any erosions. Ultrasound showed features of extensor tenosynovitis of the hand, bilateral ankle and knee effusion, with synovial hypertrophy. Further investigation revealed negative anti-nuclear antibodies, anti-double-strand DNA, antiphospholipid antibodies, anti-SSA and SSB, RF, anti-cyclic citrullinated peptide antibody and anti-neutrophil cytoplasmic antibodies. Complement, immunoglobulin, faecal calprotectin, and Interferon-Gamma Release Assay were normal. Human leukocyte antigen B27 was negative. The patient was initially treated with naproxen with no improvement. Low-dose prednisolone was started (15mg - 0.3mg/kg/day), with a dramatic resolution within 3 days and a weight loss of 8 kg. However, since symptoms partially returned while tapering off prednisolone, it was decided to start methotrexate, which enabled prednisolone weaning and achieving remission.

**Conclusion:** The association of pitting oedema, hand tenosynovitis and dramatic response to low dose steroids make the diagnosis of RS3PE syndrome very likely. Although RS3PE is mainly seen in elderly patients, it can occur in paediatric patients. As it is an uncommon syndrome, it may be overlooked in paediatric rheumatology and should be recognized in a typical clinical scenario.

**Patient Consent**

Yes, I received consent

**Disclosure of Interest**

None declared

## P200 Assestment of pediatric patients with undifferentiated connective tissue disease

### E. Marchettini^1^, S. Pastore^2^, A. Taddio^1,2^, A. Tommasini^1,2^

#### ^1^Department of Medical, Surgical and Health Sciences, University of Trieste; ^2^Institute for Maternal and Child Health IRCCS “Burlo Garofolo", Trieste, Italy

##### Correspondence: E. Marchettini

*Pediatric Rheumatology 2023*, **21(Suppl 2):**P200

**Introduction:** UCTD is a clinical entity used to identify clinical and serological manifestations of systemic autoimmune disease that do not meet common criteria for classification of rheumatic diseases. Data in children are completely absent, but first signs and symptoms may occur at this age.

**Objectives:** To characterize clinical, laboratory, imaging, and genetic features in a pediatric case series of UCTD.

**Methods:** This is a retrospective observational study. We analyzed clinical records of patients referred to our center with symptoms and serological signs of inflammation who didn’t receive a definite diagnosis.

**Results:** 8 patients have been included in the study (figure 1). Main reasons for referral included constitutional symptoms and positive autoantibody finding. The most common symptom was asthenia. More than 50% of patients presented neurological complaints. All but one had positive ANA associated with one or more among: leukopenia, positive Ro antibodies, and hyper IgG. All presented mild to moderate increase of ESR, in some cases together with a mild elevation of CRP. Positive IFN signature score was detected in all but one patient. In five cases we performed salivary glands ultrasonography: case #2 presented “SS like changes” in parotid glands with a Focus Score of 8 at biopsy, despite absence of any sicca syndrome symptom. Other patients presented non-specific alterations of submandibular glands. Genetic analysis for autoinflammatory diseases revealed pathogenic or potentially predisposing variants in over 50% of them. In case #7 it revealed a STAT1-gain of function mutation. The patient had recurrent oral candidiasis and was treated with baricitinib and antimalarials, with complete benefits. In Case #8 emerged a heterozygosity for two ADA2 mutations. He developed hypogammaglobulinemia and underwent treatment with etanercept. Case #6, considering a SLE-like dominated by severe headaches and mood disorder, was treated with corticosteroids and mycophenolate, with partial benefit, whereby presented a dramatical response to JAK-inhibitors. The other 5 patients are followed by clinical and laboratory follow-up, with no treatment so far.

**Conclusion:** UCTD are not rare in children. The detection of specific autoantibodies, alterations of parotid glands and elevated inflammatory indices could be predictive of evolution in SS, but only a longer follow up could confirm this hypothesis. Genetic evaluation is essential to exclude monogenic conditions that may mimic an undefined rheumatic disease. Treatments are not mandatory and have to be chosen weighing up potential risks and benefits. Biological characterization may offer stratification criteria which could suggest tailored treatment choices in complex cases, e.g. JAK-inhibitors, antimalarials or biologics.

**Patient Consent**

Yes, I received consent

**Disclosure of Interest**

None declared

## P201 Juvenile mixed connective tissue disease: experience of a paediatric rheumatology unit

### M. I. N. Marques, I. Madureira, C. Henriques, M. Conde, M. P. Ramos

#### Pediatric Rheumatology Department, Hospital Dona Estefânia - Centro Hospitalar Lisboa Central, Lisbon, Portugal

##### **Correspondence:** I. Madureira

*Pediatric Rheumatology 2023*, **21(Suppl 2):**P201

**Introduction:** Mixed Connective Tissue Disease (MCTD) is a rare immune-mediated disorder characterized by overlapping features of juvenile idiopathic arthritis, systemic lupus erythematosus (SLE), systemic sclerosis, and dermatomyositis (DM)/polymyositis, associated with high titers of anti-U1 ribonucleoprotein antibodies (U1RNP). The heterogeneity of clinical manifestations, both at presentation and over time, present a diagnostic challenge. Furthermore, no classification criteria for MCDT have been validated for children.

**Objectives:** Characterization of patient demographics, clinical and complementary findings, and treatment in children with MCTD.

**Methods:** A single centre retrospective case series of children diagnosed with MCTD from 2008 to 2022. We identified children fulfilling both classification criteria for MCTD proposed by Alarcón-Segovia and Kasukawa.

**Results:** Eight patients were identified (7 female), median age at diagnosis of 10 years (5-15 years) and median time to diagnosis of 12 months (m) (4-36m). At onset, all had Raynaud phenomenon (RP) and arthritis, 6/8 had systemic symptoms, 6/8 had skin manifestations (puffy fingers 3/8, DM rash 2/8, SLE rash 1/8 and one with digital erythema), 3/8 had myositis (one with muscle weakness), 2/8 presented with serositis, and 1/8 with aseptic meningitis. All presented an abnormal nailfold capillaroscopy.

During follow-up, two additional patients developed puffy fingers and digital erythema; of the others, two evolved with digital ulcers, one with sclerodactyly, and two with SLE rash. 4/8 patients showed reduced Diffusing Capacity for Carbon Monoxide; none had interstitial lung disease on CT. One had gastroesophageal reflux disease.

All patients showed elevated ESR and hypergammaglobulinemia as well as positive ANA (≥1:160) and anti-U1RNP antibodies. One had hypocomplementemia. 4/8 had positive RF (2 with polyarticular arthritis; 2 with oligoarthritis); other positive antibodies were: anti-SSA (1/8), anti-Ro52 (2/8), anti-dsDNA (1/8), and anti-RP155 (1/8). During disease course, 6/8 patients had anaemia, 5/8 leukopenia, and 5/8 lymphopenia.

At diagnosis, all patients were treated with hydroxychloroquine, corticosteroids, and calcium channel blockers for RP management; 7/8 started methotrexate but 2 switched to azathioprine. 1/8 patient was treated with cyclophosphamide followed by maintenance therapy with mycophenolate mofetil.

**Conclusion:** Our findings are in accordance with MCTD literature, regarding the age of onset and time until diagnosis, female predominance and clinical manifestations, with RP, arthritis and skin disease associated with positive anti-U1RNP being the most common presenting features. Inflammatory symptoms decreased over time with scleroderma-like features predominating later in the disease course. Pulmonary function abnormalities were frequent, despite the absence of respiratory symptoms and lung disease on imaging exams in these patients.

**Patient Consent**

Not applicable (there are no patient data)

**Disclosure of Interest**

None declared

## P202 Clinical manifestations at the onset of Pediatric Mixed Connective Tissue Disease (PMCTD): a systematic review

### E. Marrani^1^, A. Terminiello^2^, M. V. Mastrolia^1^, I. Maccora^1^, I. pagnini^1^, V. maniscalco^1^, S. abu rumeileh^1^, G. simonini^1^

#### ^1^Pediatric Rheumatology; ^2^Pediatrics, AOU Meyer IRCCS, firenze, Italy

##### **Correspondence:** E. Marrani

*Pediatric Rheumatology 2023*, **21(Suppl 2):**P202

**Introduction:** pMCTD is a rare disorder that includes features of systemic lupus erythematosus, polymyositis/dermatomyositis, juvenile idiopathic arthritis, and systemic sclerosis. Fifty years have passed since Sharp identified MCTD in 1972, and diagnosis of this disorder remains challenging

**Objectives:** The aim of this review is to identify any clinical features at the diagnosis of pMCTD and manifestations that are not currently part of the available diagnostic criteria.

**Methods:** A systematic literature review was performed in accordance with PRISMA guidelines using electronic bibliographic databases. Data obtained were extracted using a dedicated database containing clinical data that best categorize patient characteristics. Criteria for inclusion: studies including patients with a pMCTD diagnosis with onset before 18 years of age and reporting a description of initial clinical features.

**Results:** The search returned a total of 41 articles for subsequent data extraction, with a total number of 218 patients. They were predominantly female (81.56%, n=167), and the mean age at onset was 147 months (median 126 months, 10.5 years). When indicated, the most commonly used criteria for diagnosis were Kasukawa criteria (50%, 11 studies), then Alarcon-Segovia criteria (31%, 7 studies), then Sharp criteria (23%, 5 studies); no Khan criteria were used.

Joint involvement, Raynaud's phenomenon, myositis, and swollen fingers/hands are the most common clinical features at diagnosis according to the data reported in the literature, although with slightly lower percentages than in other reviews. Dermatologic signs are very heterogeneous, but were found to be a very present feature at disease onset, affecting 1/3 of patients. Fever, not covered by any of the diagnostic criteria, was noted in 1/4 of cases. Pulmonary and esophageal involvement are reported in a lower percentage at the onset of the disease, indicating a more developmental nature of these conditions.

**Conclusion:** The data from this systematic review suggest greater clinical heterogeneity of the disease in the pediatric population, for which there are no validated diagnostic criteria. Typical features appear to be less common when case reports are included, suggesting a less characteristic initial presentation than an advanced stage; therefore, the absence of typical features at baseline should not preclude a diagnosis of pMCTD. Fever often occurs early in the disease and is not included in the diagnostic criteria. This systematic review may provide useful insights for future research to better assess the clinical features of pMCTD and the potential development of scores/algorithms for diagnosis in the pediatric population.

**Patient Consent**

Not applicable (there are no patient data)

**Disclosure of Interest**

None declared

**References**

1. Sharp GC, Irvin WS, Tan EM, Gould RG, Holman HR (1972) Mixed connective tissue disease–an apparently distinct rheumatic disease syndrome associated with a specific antibody to an extractable nuclear antigen (ENA). Am J Med 52(2):148–159

## P203 Knowledge and practice of Kenyan Child Health practitioners on immunikzation of children with immune mediated inflammatory disorders: a cross-sectional survey

### L. O. Okong'o^1^, O. O. Malande^2^, C. Karanja-Chege^3^, M. Nzoka^4^

#### ^1^Paediatrics and Child Health, University of Nairobi, Nairobi, Kenya, ^2^Director, Esat Africa Centre for Vaccines and Immunization, Kampala, Uganda, ^3^Paediatrics and Child Health, Kenyatta University, ^4^board member, East Africa Centre for Vaccines and Immunization, Nairobi, Kenya

##### **Correspondence:** L. O. Okong'o

*Pediatric Rheumatology 2023*, **21(Suppl 2):**P203

**Introduction:** Immune mediated inflammatory diseases (IMIDs) encompass a wide array od disorders including autoimmune and autoinflammatory conditions. These diseases are associated with inflammation and immune suppression; and are often treated with immunosuppressive agents making the affected patients severely immunosuppressed. Thus, these patients are likely to derive optimum benefit from immunization against infectious agents. Many patients depend on healthcare workers (HCW) to recommend appropriate vaccines and it is thus important that the HCW have the correct knowledge and attitudes towards vaccines to ensure that the patients receive correct treatment. We conducted a survey among Kenyan child health care providers to determine their knowledge and practices concerning immunization of children with IMIDs.

**Objectives:** To determine the knowledge and practices of HCW on immunization for children with IMIDs in Kenya

**Methods:** This was a cross-sectional descriptive study conducted among Kenyan child health practitioners attending the annual Kenya Paediatric Association scientific conference in April 2022. Data was collected using a self-administered paper questionnaire during the first day of the conference. A total of 150 questionnaires were distributed. The completed questionnaires were collected and the data transcribed onto an MS Excel for analysis

**Results:** Seventy-six responses were received from the HCWs: 50 Female, 34 Male, and 2 did not indicate their gender. The majority (60/76 or 78.9%) were practicing Pediatricians. Majority (90.7%) knew there were additional vaccines or boosters that should be given to children with IMIDs and 84.6% were in support of children with IMIDs receiving the additional vaccines. The other 15.4% were non-committal (not sure). The vaccines by the participants were Meningococcal 11, Influenza 5, pneumococcal 4, chicken pox 2 while Hib was not mentioned. The rest mentioned other vaccines or did not know the specific vaccine boosters or antigens. Further, only 10.4% reported to have ever recommended any of these vaccines for a child with IMIDs despite having managed such children.

**Conclusion:** Most child health providers in Kenya know about IMIDs and the need for booster immunizations or additional vaccines. However, this knowledge has not been turned into practice and majority are not aware of the specific vaccines that should be given and the recommended schedule. Training on specific vaccines and their schedule is recommended while there is also need to include recommendations on immunization of children with IMIDs in the national immunization guidelines for greater uptake of needed vaccines by children with IMIDs.

**Patient Consent**

Not applicable (there are no patient data)

**Disclosure of Interest**

None declared

**References**

1. Vaccination schedule for Kenya (who.int)
2. Furer V, Rondaan C, Heijstek MW, Agmon-Levin N, van Assen S, Bijl M, Breedveld FC, et al. 2019 update of EULAR recommendations for vaccination in adult patients with autoimmune inflammatory rheumatic diseases. Ann Rheum Dis. 2020 Jan;79(1):39-52. doi: 10.1136/annrheumdis-2019-215882. Epub 2019 Aug 14. PMID: 31413005.
3. Dell' Era L, Esposito S, Corona F, Principi N. Vaccination of children and adolescents with rheumatic diseases. Rheumatology (Oxford). 2011 Aug;50(8):1358-65. doi: 10.1093/rheumatology/ker102. Epub 2011 Apr 10. PMID: 21482543.

## P204 Scurvy mimicking rheumatologic disease

### J. M. Ourique, M. B. Camargo, T. D. S. Scheid, S. H. Machado

#### Hospital de Clínicas De Porto Alegre, Porto Alegre, Brazil

##### **Correspondence:** J. M. Ourique

*Pediatric Rheumatology 2023*, **21(Suppl 2):**P204

**Introduction:** Vitamin C is a water-soluble compound that plays an important role in human physiology. Humans are unable to produce it and rely on diet to obtain it. A diet poor in foods containing vitamin C can lead to its deficiency and possibly scurvy.

**CASE REPORT**: Male, 11 years old, previously healthy, started with hip pain, showing bilateral synovitis. He had mild anemia, without leukocytosis and without alteration of inflammatory tests. He treated transient synovitis with non-steroidal anti-inflammatory drugs (NSAIDs). After 40 days, he had pain and difficulty walking again. MRI of the pelvis was performed, showing sacroiliitis, and he also presented worsening anemia, with anisocytosis and microcytosis, elevation of inflammatory tests, starting then prednisolone 1mg/kg. He did not have arthritis, but pain that was incompatible with physical examination, in addition to mild gingival hyperplasia. Lumbosacral spine MRI requested, with small central disc herniation at L4-L5. Electroneuromyography was performed on lower limbs, with report of polyradiculitis, with current denervatory activity. He came to the hospital for skin lesions (hematomas and purpuric lesions) and disabling pain.

He was always an anxious child, with food selectivity. Decreased red meat intake gradually, in the last 6 months without eating meat and eggs. Little acceptance of greens, fruits and vegetables. Large consumption of cow's milk. He had a Hb of 5.7, elevated CRP and ESR, other tests were normal. Objectively: pain when performing small movements, discomfort in bed in the "frog position", in addition to arthritis of the wrists, knees and ankles. He had gingival hyperplasia and skin lesions - dry skin with bleeding perifollicular. Laboratory tests ruled out recent viral infections by hepatitis B, hepatitis C, HIV, syphilis, cytomegalovirus, herpes simplex, epstein barr, toxoplasmosis and parvovirus; autoimmune diseases and vasculitis as well (ANA, ANCA, FR, C3, C4, normal IgA). Diagnosis of vitamin C deficiency was suggested, Vit C=0.05 (RR 0.5-1.5). Treatment with vitamin C was initiated and showed excellent response with improvement of gingivitis, skin lesions and diffuse pains.

**Conclusion:** Although scurvy is generally considered a disease of the past, it is still present. It needs to be remembered about the differential diagnosis with rheumatological diseases that can cause painful hemarthrosis and subperiosteal hemorrhage. Complications are due to vascular fragility following inappropriate collagen synthesis, which respond quickly within 24 hours and improve over days and weeks with vitamin C replacement.

**Patient Consent**

Yes, I received consent

**Disclosure of Interest**

None declared

**References**

1. Magiorkinis E,Beloukas A, Diamantis A. Scurvy: past, present and future. Eur J Intern Med. 2011;22(2):147- 152.
2. Montalto, M.,Porceddu,E., Pero,E., Lupascu, A.,Gallo, A.,De Simone,C.,Nucera, E.,Aruanno, A., Giarretta, I.,Pola,R. and Landolfi, R. (2021),Scurvy: A Disease not to be Forgotten. Nutrition in Clinical Practice,36:1063-1067

## P206 Children with JIA and HLA B27: are they more susceptible to allergies?

### T. Pungertnik^1^, N. Toplak^1,2^

#### ^1^Department of Allergology, Rheumatology and Clinical Immunology, University Children's Hospital, University Medical Centre Ljubljana, Slovenia; ^2^ Faculty of Medicine, University of Ljubljana, Slovenia, Ljubljana, Slovenia

##### **Correspondence:** T. Pungertnik

*Pediatric Rheumatology 2023*, **21(Suppl 2):**P206

**Introduction:** Juvenile idiopathic arthritis (JIA) is characterized by Th1 type of immune system dysregulation in children. We aimed to investigate whether these children are also more prone to Th2 immune-related conditions such as allergies or atopic dermatitis (AD).

**Objectives:** The aim of this study was to determine a possible association between JIA and Th2 mediated diseases, including food and inhalant allergies, and AD, and whether being HLA B27 positive increases the likelihood of developing them.

**Methods:** All patients diagnosed with JIA and followed at the University Children’s Hospital Ljubljana between January 2017 and April 2023 were included. The cohort was then analyzed using chi-squared test to determine the incidence of allergies and HLA B27 presence.

**Results:** A total of 835 patients diagnosed with JIA were included, with 310 (37.1%) being male and 525 (62.9%) being female. Among them, 177 (21.2%) were found to have allergies, with 81 (26.1%) male and 96 (18.3%) female. A statistically significant difference was found between sexes (*p* = .007). Out of the 134 (21.2%) patients who tested positive for HLA B27, 33 (24.6%) had allergies, while out of the 701 who tested negative, 144 (20.5%) had allergies. Our analysis did not find a statistically significant association (*p* = .28). 180 allergies among 154 patients were included, with 18 individuals having multiple types of allergies. Allergy to inhalant allergens was found in 130 patients (72.2%), 61 male (69.3%) and 69 female (75.0%); food allergies in 23 patients (12.8%), 10 male (11.4%) and 13 female (14.1%); 14 patients (7.8%) had insect venom allergies, 9 male (9.1%) and 5 female (2.2%); and 13 patients (5.4%) reported AD, including 8 male (9.4%) and 5 female (5.6%).

**Conclusion:** Our study found observed a similair prevalence of allergies in children with JIA in Slovenia than in the general population (1). We observed a higher incidence of allergies in male patients, as is true in the general population (2). Furthermore, our analysis did not reveal any significant association with HLA B27 presence. Interestingly, previous research conducted in Taiwan found that children with allergies were more likely to develop JIA (3). Additionally, a large population-based Finnish study showed a positive association between JIA and AD (4). Larger studies are needed to better evaluate the relationship between JIA and allergy prevalence.

**Patient Consent**

Not applicable (there are no patient data)

**Disclosure of Interest**

None declared

**References**

1. Mazur M, Czarnobilska M, Dyga W, Czarnobilska E. Trends in the Epidemiology of Allergic Diseases of the Airways in Children Growing Up in an Urban Agglomeration. *J Clin Med*. 2022;11(8):2188. Published 2022 Apr 14.
2. Kurukulaaratchy RJ, Karmaus W, Arshad SH. Sex and atopy influences on the natural history of rhinitis. Curr Opin Allergy Clin Immunol. 2012;12(1):7-12.
3. Lin CH, Lin CL, Shen TC, Wei CC. Epidemiology and risk of juvenile idiopathic arthritis among children with allergic diseases: a nationwide population-based study. Pediatr Rheumatol Online J. 2016;14(1):15. Published 2016 Mar 10.
4. Keskitalo PL, Jokelainen J, Tasanen K, Sinikumpu SP, Huilaja L. Juvenile idiopathic arthritis in children and adolescents with atopic dermatitis: A Finnish nationwide registry study. J Am Acad Dermatol. 2023;88(5):1187-1189.

## P207 Rhupus syndrome in children: a multi-center retrospective cohort study

### S. Sener^1^, E. D. Batu^1^, S. Sahin^2^, D. Gezgin Yildirim^3^, M. Kisla Ekinci^4^, H. Kisaoglu^5^, Y. Karali^6^, S. Demir^7^, U. Kaya Akca^8^, A. Gunalp^2^, S. Turkmen^9^, G. Kayaalp^10^, C. Arslanoglu^11^, R. Torun^12^, O. Basaran^1^, A. Pac Kisaarslan^11^, B. Sozeri^9^, N. Aktay Ayaz^10^, S. A. Bakkaloglu^3^, S. S. Kilic^6^, M. Kalyoncu^5^, Y. Bilginer^1^, E. Unsal^12^, O. Kasapcopur^2^, S. Ozen^1^

#### ^1^Pediatric Rheumatology, Hacettepe University, Ankara; ^2^Pediatric Rheumatology, Istanbul Cerrahpasa University, Istanbul; ^3^Pediatric Rheumatology, Gazi University, Ankara; ^4^Pediatric Rheumatology, Adana City Hospital, Adana; ^5^Pediatric Rheumatology, Karadeniz Technical University, Trabzon; ^6^Immunology and Rheumatology, Uludag University, Bursa; ^7^Pediatric Rheumatology, Eskisehir Osmangazi University, Eskisehir; ^8^Pediatric Rheumatology, Aydin Gynecology and Children's Hospital, Aydin; ^9^Pediatric Rheumatology, Umraniye Training and Research Hospital; ^10^Pediatric Rheumatology, Istanbul University, Istanbul; ^11^Pediatric Rheumatology, Erciyes University, Kayseri; ^12^Pediatric Rheumatology, Dokuz Eylul University , Izmir, Türkiye

##### **Correspondence:** S. Sener

*Pediatric Rheumatology 2023*, **21(Suppl 2):**P207

**Introduction:** Rhupus syndrome is a rare overlap syndrome that combines features of both juvenile idiopathic arthritis (JIA) and systemic lupus erythematosus (SLE). Rhupus syndrome does not have a clear definition and a proven therapeutic strategy, especially in children.

**Objectives:** In this study, we aimed to evaluate children with Rhupus syndrome.

**Methods:** All patients with onset under the age of 18, diagnosed with JIA according to ILAR classification criteria, meeting SLICC criteria for SLE, and additionally rheumatoid factor (RF) positivity were included in the study. Patients diagnosed with mixed connective tissue disease according to the Alarcón-Segovia and Kasukawa criteria were excluded from the study.

**Results:** Thirty pediatric patients with Rhupus syndrome were included in the study (F/M=28/2). The median (IQR) age at SLE diagnosis was 11.5 (5.9) years, while the median (IQR) age at JIA diagnosis was 12.3 (5.5) years. The most prominent phenotype was JIA (60%) at the disease onset, and SLE (73.3%) at the last visit. All patients had chronic arthritis, 66.7% had constitutional symptoms, 80% had skin findings, 50% had oral ulcers, 16.7% had non-scarring alopecia, and 10% had serositis. Among the major organ involvements; 53.3% had hematological, 23.3% renal involvement, 10% gastrointestinal, and 6.7% neuropsychiatric involvement. Arthritis was polyarticular (73.3%), asymmetric (66.7%), and erosive (53.3%) in most patients. Proximal interphalangeal (PIP, 23.1%), knee (17.6%), and metacarpophalangeal (MCP, 16.5%) joints were the most commonly involved (Figure 1). None of the patients had rheumatoid nodules. Antinuclear antibody (ANA) and RF were positive in all patients. For the treatment of SLE, hydroxychloroquine was used in all patients, corticosteroid in 26 (86.7%), mycophenolate mofetil in 14 (46.7%), azathioprine in seven (23.3%), cyclophosphamide in six (20%), rituximab in five (16.7%), intravenous immunoglobulin in five (16.7%) and cyclosporine A in two (6.7%) patients. For JIA symptoms, corticosteroids were used in 23 patients (76.6%), methotrexate in 22 (73.3%) patients, nonsteroidal anti-inflammatory drugs (NSAIDs) in 17 (57.6%) patients, tumor necrosis factor (TNF) inhibitors were used in three (10%) and Janus kinase (JAK) inhibitors were used in one (3.3%) patient. After a median (IQR) 3.7 (7.2) years follow-up period, 20 patients (66.7%) had complete remission, seven patients (23.3%) had partial remission, and three (10%) had resistant disease.

**Conclusion:** Our study is the largest in the literature evaluating pediatric Rhupus cases. It is noteworthy that chronic arthritis was found in all Rhupus cases, with erosive arthritis in about 53.3% of patients. With this study, we hope to raise the awareness of clinicians and improve their disease management by addressing the symptoms and signs, and treatment options of pediatric Rhupus patients.

**Patient Consent**

Yes, I received consent

**Disclosure of Interest**

None declared

**References**

1. Ziaee V, Moradinejad MH, Bayat R. RHUPUS Syndrome in Children: A Case Series and Literature Review. Case Rep Rheumatol. 2013;2013:819629. doi: 10.1155/2013/819629.
2. Antonini L, Le Mauff B, Marcelli C, Aouba A, de Boysson H. Rhupus: a systematic literature review. Autoimmun Rev. 2020 Sep;19(9):102612. doi: 10.1016/j.autrev.2020.102612.

## P208 IGG4 related disease- a case series of four pediatric patients

### T. Singla^1^, N. K. Bagri^1,2,3,4,5^ on behalf of Rachana Meel, Seema Kashayp, Manisha Jana, Rakesh Kumar

#### ^1^Pediatrics; ^2^Ophthalmology; ^3^Pathology; ^4^Radiodiagnosis; ^5^Nuclear Medicine, All India Institute of Medical Sciences (AIIMS), New Delhi, Delhi, India

##### **Correspondence:** T. Singla

*Pediatric Rheumatology 2023*, **21(Suppl 2):**P208

**Introduction:** Immunoglobulin G4–related disease (IgG4- RD) is a rare systemic autoimmune disease characterized by fibroinflammatory infiltration that can affect nearly every organ system. Orbital proptosis is one of the presentation of IgG4RD, and is often diagnosed late, which may lead to visual impairment. Diagnosis is often challenging in our settings where the background rate of infections such as tuberculosis is high. Herein, we report 4 children with IgG4RD presenting with orbital proptosis from our center.

**Objectives:** To describe the clinical spectrum and outcome of children with orbital IgG4-RD in children.

**Methods:** A chart review of children with IgG4RD (diagnosed as per 2019 ACR/EULAR classification criteria) followed in pediatric rheumatology clinic, department of Pediatrics and Dr Rajendra Prasad Ophthalmic center at All India Institute of Medical Sciences, New Delhi was carried out. The clinical, radiological and pathological details, treatment and outcome were noted in a predesigned proforma.

**Results:** Four children (2 girls) with a mean (SD) age of 87 (±27) months, diagnosed with IgG4-RD (from March 2022 to February 2023) were enrolled for this chart review. The mean (SD) duration of symptoms was 16 (±8) months. All children presented with orbital proptosis (unilateral n=3, bilateral n=1). Other features were redness of eye, visual disturbances, headache and vomiting. IgG4 levels were available in 3 children; all three had elevated IgG4 levels [mean (SD) =266 (±42) mg/dl ]. The MRI of the orbit showed ill-defined infiltrative lesion in orbit with involvement of extraocular muscles with lacrimal gland involvement in two subjects. Two out of four children, who were amenable to tissue biopsy showed histopathological confirmation of IgG4-RD with lymphoplasmacytic infiltrate and IgG4 immunostaining positivity (>10/hpf). PET scan performed in two children revealed metabolically active disease in bilateral orbits with additional uptake in bilateral cervical lymph node and spleen in one and orbital with lacrimal gland involvement in other. All children were managed with steroids (oral, n=3/ intralesional, n=1) ± DMARDs ( azathioprine, n=2 and mycophenolate mofetil, n=1). The steroids were tapered and stopped by 6 months of follow-up in all subjects. At last follow-up [mean (SD) duration 9 (±3) months] all children were in remission with reduction in the proptosis; 2 were off all medications whilst two maintained remission on DMARDs (azathioprine, n=1, mycophenolate mofetil, n=1).

**Conclusion:** IgG4-RD is an infiltrative disease, and orbital proptosis is one of the common presentations. Although extraocular involvement is rare but use of imaging modalities like PET scan can show other sites, such as splenic infiltrate in one of our cases and may help in assessing the overall burden of the disease. A close of follow-up with ophthalmology and pediatric rheumatology team is pivotal for timely diagnosis and management.

**Patient Consent**

Yes, I received consent

**Disclosure of Interest**

None declared

**References**

1. Kamisawa T, Zen Y, Pillai S, Stone JH. IgG4-related disease. The Lancet. 2015 Apr 11;385(9976):1460-71.
2. Karim F, Loeffen J, Bramer W, Westenberg L, Verdijk R, van Hagen M, van Laar J. IgG4-related disease: a systematic review of this unrecognized disease in pediatrics. Pediatric Rheumatology. 2016 Dec;14(1):1-9.

## P209 Gene expression profiling of South African children with Kawasaki disease

### T. F. Spracklen^1,2,3^, S. C. Mendelsohn^4^, C. Butters^2,4^, H. Facey-Thomas^2^, M. Erasmus^4^, H. van der Ross^2^, C. Scott^2,5^, L. Zühlke^1,2,3^, T. J. Scriba^4^, K. Webb^2,6^

#### ^1^South African Medical Research Council; ^2^Department of Paediatrics and Child Health; ^3^Cape Heart Institute; ^4^Institute of Infectious Disease and Molecular Medicine; ^5^Clinical Research Centre, University of Cape Town, Cape Town, South Africa; ^6^Crick African Network, The Francis Crick Institute, London, United Kingdom

##### **Correspondence:** T. F. Spracklen

*Pediatric Rheumatology 2023*, **21(Suppl 2):**P209

**Introduction:** Kawasaki Disease (KD) is an acute paediatric vasculitis of unknown aetiology, possibly linked to infectious causes. Symptoms such as rash, fever, inflammation and oedema typically resolve spontaneously, but many children may suffer permanent coronary artery damage. Gene expression profiling can improve our understanding of disease, but no such study has been conducted in KD patients from Africa.

**Objectives:** To investigate gene expression profiles of KD in South African children compared to healthy controls.

**Methods:** This investigation included 8 children with acute KD pre-treatment (baseline), 54 healthy non-inflammatory paediatric controls, and 19 controls with other inflammatory conditions. Longitudinal post-treatment samples were available for 4 children with KD (12 specimens) at various timepoints after intravenous immunoglobulin (IVIG) alone or IVIG with methylprednisolone. Expression of 80 broadly representative immune-related genes was determined by real-time quantitative PCR. Differentially expressed genes (DEGs) were identified through nonparametric pairwise comparisons between cases and controls, adjusted by Holms correction.

**Results:** We identified 4 DEGs in cases compared to controls: *BPI*, *CASP5*, *FCGR1B* and *MMP8* were all significantly upregulated; no downregulated genes were identified. No significant differences were observed between KD and inflammatory controls. Expression of *CASP5* in KD patients remained elevated during the study period while the other three genes appeared to return to normal levels. All four genes were in the 27-gene expression signature for South African multi-system inflammatory syndrome in children (MIS-C) which we recently described. One patient (P4) was resistant to IVIG treatment; all 4 DEGs remained elevated in this patient up to 24 days after initial treatment. Further features of patient P4’s gene expression profile included marked elevation of *GZMA* (10-fold) and *GBP2* (67-fold) at baseline, followed by excessive fluctuations of these genes during treatment. *DDX58* and *DEFA4* were aberrantly expressed towards the end of the follow-up period.

**Conclusion:** These findings expand upon the inflammatory phenotype of KD in our setting, as the identified DEGs function in immune pathways such as neutrophil degranulation, interferon signalling and pyroptosis. Although our results are in accordance with other populations, we were unable to identify any genes that would distinguish KD from MIS-C in our cohort. The increased *GZMA* and *GBP2* expression in patient P4 suggests, for the first time, the potential of these genes as biomarkers of IVIG-resistance in KD.

**Trial registration identifying number:** Not applicable

**Patient Consent**

Not applicable (there are no patient data)

**Disclosure of Interest**

None declared

## P210 Salivary gland ultrasound features in childhood Sjögren’s disease and recurrent sialadenitis in a multidisciplinary clinic

### S. M. Stern^1^, A. Park^2^, A. Holley^3^

#### ^1^Pediatrics; ^2^Head and Neck Surgery; ^3^School of Medicine, University of Utah, Salt Lake City, United States

##### **Correspondence:** S. M. Stern

*Pediatric Rheumatology 2023*, **21(Suppl 2):**P210

**Introduction:** Differentiating between Childhood Sjögren’s Disease (cSjD) and other forms of sialadenitis (OFS), is challenging, but important due to different approaches to long-term management.

**Objectives:** The aim of this study is to analyze differences in salivary gland ultrasounds in patients with cSjD compared to OFS.

**Methods:** This is a retrospective analysis of patients seen in a pediatric multidisciplinary otolaryngology/rheumatology clinic (PMORC) between 12/2019 and 4/2022 presenting with chronic sialadenitis. Patients were categorized as cSjD vs OFS by expert rheumatology and otolaryngologist opinion. Patterns of salivary gland ultrasound involvement and clinical signs of salivary and lacrimal gland swelling were compared between the two groups.

**Results:** Thirty patients presented to PMORC, 25 with complete evaluations were included; 12 (48%) and 13 (52%) were categorized as cSjD and OFS respectively. Fourteen (56%) patients were female with a total median age of symptom onset of 6 years (4-9 years 25^th^-75^th^). The median age of the cSjD group was 7.5 years (4.5-9 years 25^th^-75^th^) vs the median age of the OFS group of 6 years (4-9 years 25^th^-75^th^).

All patients with cSjD vs 12/13 (92%) with OFS had clinical evidence of parotitis at their initial evaluation. Clinical evidence of bilateral parotitis was seen in 9/12 (75%) with cSjD compared with 4/13 (31%) in the OFS group (p = 0.033). Fifty percent (6/12 patients) with cSjD vs 23% patients (3/13) with OFS had clinical evidence of submandibular involvement (p=0.063), of whom 5/6 with cSjD vs 2/3 with OFS had bilateral submandibular involvement (p=0.069). Four (33%) patients with cSjD had lacrimal gland inflammation vs 1 (8%) patient with OFS (p=0.049); all 5 patients had bilateral involvement.

Parotid glands were visualized on ultrasound for all patients; however, 3/12 (25%) cSjD and 9/13 (69.2%) OFS patients did not complete a submandibular ultrasound (p=0.013). All cSjD vs 8/13 (62.5%) OFS patients had evidence of parotid gland inflammation on ultrasound (p= 0.0075). Eighty-three percent of cSjD (10/12) patients vs 30.7% of OFS patients (4/13) had bilateral evidence of parotid gland inflammation on ultrasound (p = 0.0033). 8/9 (88.9%) cSjD vs 2/4 (50%) OFS patients had submandibular involvement on ultrasound (p = 0.073). 7/9 (77.8%) vs 2/4 (50%) had bilateral submandibular involvement on ultrasound (p = 0.18). Of the patients with parotid and submandibular ultrasounds, the cSjD groups had an average of 3.7 glands involved compared to 1.5 in the OSF group (p = 0.0052).

**Conclusion:** There appears to be a statistically significant difference in the ultrasound and clinical examination pattern of salivary gland inflammation seen in patients with cSjD compared to OFS in children. Patients with cSjD appear to have more glandular involvement compared to those with OFS.

**Patient Consent**

Not applicable (there are no patient data)

**Disclosure of Interest**

None declared

## P211 Homocystinuria: a differential diagnosis of Marfan syndrome and a cause of thrombosis

### F. Sztajnbok, I. M. Paz, F. C. Zonis, A. R. Fonseca, J. L. Monteiro, M. F. Rodrigues, P. R. Souza, M. R. Vasti, R. G. Almeida

#### Pediatric Rheumatology, Universidade Federal do Rio de Janeiro, Rio de Janeiro, Brazil

##### **Correspondence:** G. Almeida

*Pediatric Rheumatology 2023*, **21(Suppl 2):**P211

**Introduction:** Homocystinuria is a recessive hereditary disease caused by cystathionine β-synthase deficiency, which makes the differential diagnosis with Marfan syndrome. There is presence of hypotonia, joint stiffness, lens displacement, in addition to thrombosis and mental retardation. Early intervention through targeted dietary restriction (methionine) and/or drug intervention (pyridoxine) are important to improve the patient's symptoms.

**Objectives:** To report the case of a patient with visual alteration and marfanoid habits, with the main hypothesis of Marfan Syndrome, but who had thrombosis and, in diagnostic investigation, final diagnosis proved to be homocystinuria.

**Methods:** The information was obtained by reviewing the patient's medical record.

**Results:** We report the case of an 11-year-old child, admitted to our service with a history of hospitalization three months before for deep venous thrombosis in the left iliac and femoral artery. After hospital discharge, she developed asthenia, pain in the left lower limb and dizziness. She had a previous report one year before admission of an episode of convulsive crisis, and magnetic resonance imaging showed encephalomalacia in the left frontal lobe. In the admission exam, we verified marfanoid habits. She was referred to the ophthalmology service, which identified mild lens subluxation and myopia (grade 5) in both eyes. Given the clinical picture, some laboratory tests were requested, including plasma aminogram, which showed an altered (increased) profile, with emphasis on group 3 (regions of glutamine, taurine, citrulline, serine, OH-proline and homocysteine). Thus, there was a diagnosis of homocystinuria.

**Conclusion:** The reported case highlights the importance of considerations regarding differential diagnoses, so that the patient is properly diagnosed and treated, obtaining an improvement in the clinical condition

**Patient Consent**

Yes, I received consent

**Disclosure of Interest**

None declared

## P212 Childhood Sjogren’s syndrome: a nationwide multicenter study

### G. Kılbaş^1^, S. Yüksel^1^, S. Ayduran^1^, S. Şener^2^, T. Coşkuner^3^, K. Ulu^3^, H. Kısaoğlu^4^, E. Aslan^5^, E. K. Könte^5^, C. Arslanoğlu^6^, T. Aydın^7^, Y. S. Oğuzkaya^8^, F. Çakmak^9^, D. G. Yıldırım^10^, M. M. Kaplan^11^, H. E. Sönmez^12^, M. K. Ekinci^13^, K. Öztürk^14^, F. Demir^15^, E. Bağlan^16^, B. Bozkaya^17^, S. N. Taşkın^18^, S. Demir^19^, E. Sağ^20^, E. D. Batu^2^, S. Şahin^5^, S. Bakkaloğlu^10^, S. S. Kılıç^8^, A. P. Kısaarslan^6^, B. Ç. Acar^11^, N. A. Ayaz^21^, M. Kalyoncu^4^, B. Sözeri^3^, E. S. Ünsal^7^, Ö. Kasapçopur^5^, S. Özen^2^

#### ^1^Pediatric Rheumatology, Pamukkale University Faculty of Medicine, Denizli; ^2^Pediatric Rheumatology, Hacettepe University Faculty of Medicine, Ankara; ^3^Pediatric Rheumatology, Ümraniye Training and Research Hospital, İstanbul; ^4^Pediatric Rheumatology, Karadeniz Technical University Faculty of Medicine, Trabzon; ^5^Pediatric Rheumatology, Cerrahpaşa University Faculty of Medicine, İstanbul; ^6^Pediatric Rheumatology, Erciyes University Faculty of Medicine, Kayseri; ^7^Pediatric Rheumatology, Dokuz Eylül University Faculty of Medicine, İzmir; ^8^Pediatric Rheumatology, Uludağ University Faculty of Medicine, Bursa; ^9^Pediatric Rheumatology, Başakşehir Çam ve Sakura City Hospital, İstanbul; ^10^Pediatric Rheumatology, Gazi University Faculty of Medicine; ^11^Pediatric Rheumatology, Ankara City Hospital, Ankara; ^12^Pediatric Rheumatology, Kocaeli University Faculty of Medicine, Kocaeli; ^13^Pediatric Rheumatology, Adana City Training and Research Hospital, Adana; ^14^Pediatric Rheumatology, Göztepe Prof. Dr. Süleyman Yalçın City Hospital; ^15^Pediatric Rheumatology, Acıbadem Health Group, İstanbul; ^16^Pediatric Rheumatology, Ankara Etlik City Hospital, Ankara; ^17^Pediatric Rheumatology, Samsun Training and Research Hospital, Samsun; ^18^Pediatric Rheumatology, Diyarbakır Childeren's Hospital, Diyarbakır; ^19^Pediatric Rheumatology, Eskişehir Osmangazi University Faculty of Medicine, Eskişehir; ^20^Pediatric Rheumatology, Ankara Training and Research Hospital, Ankara; ^21^Pediatric Rheumatology, İstanbul University İstanbul Faculty of Medicine, İstanbul, Türkiye

##### **Correspondence:** G. Kılbaş

*Pediatric Rheumatology 2023*, **21(Suppl 2):**P212

**Introduction:** Sjögren's syndrome is a rare autoimmune disease in childhood and is characterized by a heterogeneous presentation. Evidence-based treatment guidelines are lacking, especially in the pediatric in children.

**Objectives:** The aim of this nationwide multicenter study is to define the demographic, clinical and laboratory characteristics of childhood Sjögren's Syndrome and to guide clinicians in this direction. The secondary aim is to identify differences (gender, clinical and laboratory findings) according to the age groups (preadolescent and adolescent).

**Methods:** In this retrospective study, 87 patients (under the age of 19 who met the 2016 ACR/EULAR criteria or diagnosed with expert opinion) from 20 pediatric rheumatology centers in Turkey were included. Demographic, clinical and laboratory characteristics, disease activity and treatment modalities were analyzed. The patients were divided into two groups as preadolescents (age ≤120 months) and adolescents (age >120 months) according to age at onset of symptoms. Disease activity was evaluated with ESSDAI (EULAR Sjögren's syndrome disease activity index).

**Results:** Of 87 patients, 75 (86.2%) were female and 12 (13.8%) were male. The median age of symptoms and age at diagnosis were 138 (29-208) and 150 (70-217) months, respectively. The median follow-up period was 24 months (min-max, 2-128). The most common clinical findings dry mouth (~75%), dry eyes (65.5%), arthralgia (~61%), parotitis (57.5%), fatigue (~49%), lymphadenopathy (~37%), arthritis (34.5%), rash (31%), weight loss (~24%), Raynoud's phenomenon (19.5%) and fever (19.5%), respectively. The clinical domains containing the highest frequencies of active patients included the articular (~47%), glandular (~44%), lymphadenopathy (~40%), and constitutional (~39%) domains, respectively. There were 52 (~60%) patients who met the 2016 ACR/EULAR classification criteria. Minor salivary gland biopsy showed Chisholm & Mason grades 3-4 in 37 out of 54 patients biopsied. Ten (83%) of 12 male patients were in the preadolescent group (83% vs 17%). Parotitis and lymphadenopathy (51.4%) were most common in preadolescents (77% vs 19% p=0.001 and 51% vs 27%, p=0.020, respectively). The frequencies of arthralgia, arthritis, rash, myalgia and Raynoud phenomenon were significantly higher in adolescent. Almost all of the patients (~96%) had a complete or partial response to the treatment.

**Conclusion:** The study showed that the clinical presentation and gender difference of preadolescent and adolescent Sjögren's syndrome were obvious. Glandular involvement and male gender were more intense in the preadolescent patients with Sjögren's syndrome. Our study may contribute to the literature in order to define the presentation of the disease and to establish specific classification criteria in childhood Sjögren's syndrome.

**Patient Consent**

Yes, I received consent

**Disclosure of Interest**

None declared

## P213 Globalization and real-world implementation of a pediatric rheumatology learning resource

### M. Alessi^1^, on behalf of yes , M. Chan^2^, T. Tanner^3^, D. O’Leary^4^, D. Abraham^5^

#### ^1^Imam Abdulrahman Bin Faisal University and King Fahad hospital of University, Pediatric Department, AlKhobar, Saudi Arabia; ^2^BC Children’s Hospital and University of British Columbia, Vancover, Canada; ^3^Children’s Hospital at Montefiore, Bronx, NY, United States; ^4^UCD Centre for Arthritis Research, Dublin, Ireland; ^5^Paediatric Rheumatology and Immunology Unit, Department of Paediatrics and Child Health, Faculty of Medicine and Health Sciences, Stellenbosch University, Cape Town, South Africa

##### **Correspondence:** M. Alessi

*Pediatric Rheumatology 2023*, **21(Suppl 2):**P213

**Introduction:** The Pediatric Rheumatology Learning Modules (PRML) were developed in 2015 as an academic curriculum for pediatric rheumatology fellowship training at a single center. With new discoveries and demand for use in different global contexts.

**Objectives:** We formed a virtual working group (WG) to update and modify content. We describe our process in adapting a learning resource, and our early experiences and feedback from piloting our work.

**Methods:** The WG (n=5) includes patients, trainees, and faculty from 5 countries and 4 continents. Literature reviews; the Textbook of Pediatric Rheumatology, 8th Edition; and ongoing user feedback on clarity and relevance, guide included content. Global adaptation draws on frameworks for internationalizing medical school curricula: plain language use, removal of cultural identifiers, generic drug names, and practice variability due to resource inequity, e.g., biologics. We created faculty guides by request to address challenging questions and to include prompts for discussion.

Strategies were discussed and refined after a pilot review of 3/37 modules including initial review, user feedback, learning objectives, references, literature review, and quizzes. Proposed changes are independently reviewed by group members; if universally agreed upon, they are accepted prior to a bi-weekly meeting where discrepancies are discussed. Final accepted changes require 75% consensus. 10-item multiple choice quizzes assess baseline knowledge and application of clinical knowledge.

All work is facilitated online (virtual meetings, cloud-based file sharing, real-time group file editing).

The updated PRLM are piloted as available at 2 pediatric rheumatology (PR) centers. Quiz results assess knowledge change and feedback from trainees and faculty are collated (email, comments on electronic documents) are used to refine the PRLM.

**Results:** The WG met 26 times over 18 months with 31 of the original 37 modules and quizzes revised. Two new modules were developed in response to gaps identified (transition, therapeutics).

At the pilot centers, modules are reviewed weekly. Trainees (PGY4-6) complete quizzes then attempt module questions. Module answers are prepared for discussion with faculty, with the same module quiz repeated afterwards. Quiz answers are then discussed.

Mean baseline quiz scores (n=240) from 13 trainees show pre- and post-modules scores of 71.6% and 91.6%, and 63.2% and 80.7% at centers 1 and 2. PGY-4 trainees gain more knowledge from training than PGY-6 trainees (23% vs 9%). Feedback from trainees and faculty includes ensuring quiz and module content align with learning objectives and ways to improve question clarity. Trainees prefer group discussion with PRLM to didactic teaching and feel modules help prepare them for board examinations.

**Conclusion:** The PRLM is the first evidence-based educational resource developed for PR trainees for teaching and learning in a group setting designed for any global context. Developing the PRLM requires time, learning, knowledge-sharing, and stable internet connections. Real-world user feedback was limited to 2 centers. More widespread use and assessment of PRLM as a tool are needed to make meaningful conclusions for future application.

**Patient Consent**

Not applicable (there are no patient data)

**Disclosure of Interest**

None declared

## P214 Gait analysis in children and adolescents with juvenile idiopathic arthritis using smart insole system

### N. Arman^1^, A. Albayrak^2,3^, A. Yekdaneh^4,5^, F. G. Demirkan^6^, N. Aktay Ayaz^6^

#### ^1^Faculty of Health Sciences Department of Physiotherapy and Rehabilitation, Istanbul University-Cerrahpaşa; ^2^Institute of Graduate Studies Physiotherapy and Rehabilitation Doctorate Program, Istanbul University-Cerrahpasa; ^3^Faculty of Health Sciences, Department of Physiotherapy and Rehabilitation, Istanbul Kent University; ^4^Institute of Graduate Studies Physiotherapy and Rehabilitation Doctorate Program, Istanbul University-Cerrahpaşa; ^5^Vocational School of Health Services Physiotherapy English Program, Fenerbahçe University; ^6^Istanbul Faculty of Medicine, Department of Pediatric Rheumatology, Istanbul University, Istanbul, Türkiye

##### **Correspondence:** N. Arman

*Pediatric Rheumatology 2023*, **21(Suppl 2):**P214

**Introduction:** Problems such as joint swelling, effusion, tenderness, pain and decrease in joint range of motion, which are common especially in load-bearing joints, affect gait function in patients with Juvenile Idiopathic Arthritis (JIA). Problems that usually occur in the lower extremities affect the time-distance parameters, kinematic and kinetic properties of walking and cause functional problems (1,2). The aim of this study was to examine the gait characteristics using the smart insoles system in children and adolescents with JIA and compare the results with their healthy peers.

**Objectives:** The aim of this study was to examine the gait characteristics using the smart insoles system in children and adolescents with JIA and compare the results with their healthy peers.

**Methods:** Thirty-four patients with JIA between the ages of 11-18 and 29 healthy peers were included in the study. DigitSole Pro® Smart Insoles System were used to evaluate monopedal (angles of the foot during heel strike, heel and toe separation from the ground, stride length, step time, stance phase time, swing phase time, foot advance angle) and bipedal (cadence, walking speed (km/ s) and double contact time) gait parameters. SPSS Version 24.0 program was used for statistical analysis.

**Results:** The mean age of patients with JIA and their healthy peers included in the study was 13.79±2.26 and 13.97±2.48 years, respectively. When children and adolescents with JIA were compared with their healthy peers, a statistically significant increase in cadence and statistically significant decrease were found in right-left swing phase duration, and push-off phase duration, right-left foot separation angle from the ground, and right-left step duration (p< 0.05). Moreover, a significant negative correlation was found between the angle of departure of the foot from the ground, cadence and the duration of the swing phase for both the right and left sides in patients with JIA (p<0.05).

**Conclusion:** Children and adolescents with JIA have different gait characteristics compared to their healthy peers. According to our results, most prominent gait characteristics are an increase in cadence, a decrease in stance, swing and pre-swing phase duration. We believe that these results may be associated with problems such as lower extremity muscle strength, balance, fatigue and pain. In future studies, we suggest that revealing the relationship between gait parameters and lower extremity functions will make it easier to decide on the exercise program for patients with JIA.

This study was supported within the scope of TUBITAK 1001-Scientific and Technological Research Projects Support Program 121E690.

**Patient Consent**

Yes, I received consent

**Disclosure of Interest**

None declared

**References**

1. Montefiori E, Modenese L, Di Marco R, Magni-Manzoni S, Malattia C, Petrarca M, et al. Linking Joint Impairment and Gait Biomechanics in Patients with Juvenile Idiopathic Arthritis. Ann Biomed Eng. 2019;47(11):2155–67.
2. Woolnough L, Pomputius A, Vincent HK. Juvenile idiopathic arthritis, gait characteristics and relation to function. Gait Posture. 2021;85:38–54.

## P215 The effect of self-management supported telerehabilitation-based exercise in juvenile idiopathic arthritis

### B. C. Caglayan^1^, B. Basakcı Calık^2^, E. Gur Kabul^3^, G. Kılbas^4^, S. Yuksel^4^

#### ^1^Physiotherapy and Rehabilitation, Istanbul Okan University, Istanbul; ^2^Physiotherapy and Rehabilitation, Pamukkale University, Denizli; ^3^Physiotherapy and Rehabilitation, Usak University, Usak; ^4^Pediatric Rheumatology, Medical Faculty of Pamukkale University, Denizli, Türkiye

##### **Correspondence:** B. C. Caglayan

*Pediatric Rheumatology 2023*, **21(Suppl 2):**P215

**Introduction:** Children with Juvenile Idiopathic Arthritis (JIA) experience pain, stiffness, fatigue poor quality of life and have impaired exercise capacity. They spend less time on physical activities compared to their healthy peers. (1,2) Due to the chronic nature of the disease, it is important that children learn to manage their health and cope with the problems (3).

**Objectives:** This study aimed to examine the effect of self-management education supported telerehabilitation-based exercise on pain, functional status, lower extremity muscle strength and performance, quality of life, and self-efficacy in children and adolescents with JIA.

**Methods:** The study included 13 participants (8 female, 5 male) diagnosed with JIA with a mean age of 13.23±2.58. Before exercise training all participants received a self management education session, including the definition of the immune system and JIA, symptoms and chronic pain and their management, relaxation and breathing, joint protection principles and energy conservation techniques, the importance of physical activity and motivational support. They also attended telerehabilitation-based exercise training (functional exercises focused on trunk stabilization, including warm-up and cool-down) three times a week for 8 week. Self management education continued throughtout the exercise training. After demographic and disease-related data were recorded, pain (numeric rating scale), functional status (Childhood Health Assessment Questionnariaes-CHAQ), lower extremity muscle strength and performance (five times sit-to-stand test), quality of life (Pediatric Quality of Life Inventory 3.0 Arthritis Module-PedsQL) and self-efficacy (Self-Efficacy For Managing Chronic Disease 6-item Scale-SE) were assessed at baseline and after the intervention. The Paired Sample T-test and Wilcoxon test were used to analyze the data.

**Results:** After intervention, there were significant improvement in PedsQL-pain and hurt (p=0.05), PedsQL- worry (p=0.018), self-efficacy (p=0.04) and five times sit-to-stand test (p=0.001).

**Conclusion:** Self management education supported telerehabilitation-based exercise program has positive efffects on pain, hurt and worry perception in quality of life, self-efficacy and lower extremity muscle strength and performance in children and adolesants with JIA.

**Patient Consent**

Yes, I received consent

**Disclosure of Interest**

None declared

**References**

1. Iversen MD, Andre M, von Heideken J. Physical Activity Interventions in Children with Juvenile Idiopathic Arthritis: A Systematic Review of Randomized Controlled Trials. Pediatric Health Med Ther. 2022 Apr 14;13:115-143. doi: 10.2147/PHMT.S282611.
2. Tarakcı E, Kısa EP, Arman N, Albayrak A. Physical activity and exercise in patients with pediatric rheumatic disease: A systematic search and review. Turk Arch Pediatr. 2021 May 1;56(3):179-186.
3. Armbrust W, Bos JJ, Cappon J, van Rossum MA, Sauer PJ, Wulffraat N, van Wijnen VK, Lelieveld OT. Design and acceptance of Rheumates@Work, a combined internet-based and in person instruction model, an interactive, educational, and cognitive behavioral program for children with juvenile idiopathic arthritis. Pediatr Rheumatol Online J. 2015 Jul 23;13:31. doi: 10.1186/s12969-015-0029-5.

## P216 Does patient reported absence of swollen and painful joints really mean that the active joint count is zero?

### A. D. W. de Vos, B. Vastert, E. van Nieuwenhove, M. Jansen, B. Prakken, N. Wulffraat, A. van Royen, S. de Roock, J. Swart

#### Pediatric Immunology and Rheumatology, Wilhelmina Children's Hospital, Utrecht, Netherlands

##### **Correspondence:** A. D. W. de Vos

*Pediatric Rheumatology 2023*, **21(Suppl 2):**P216

**Introduction:** Due to increasing health costs and as learned during the recent COVID-19 pandemic, ehealth is becoming more popular in replacing a hospital visit. We wondered if patient reported outcomes (PRO) could be used to replace live visits in the hospital.

**Objectives:** To investigate the association between a patient reported absence of swollen and painful joints (SPJC) within 2 weeks prior to the active joint count assessment by the physician (AJC) in patients with Juvenile Idiopathic Arthritis (JIA).

**Methods:** This single-centre retrospective study was executed on 5464 available JAMAR questionnaires completed by patients between 2011 and 2023. Only the first available JAMAR of each patient was used. Assessment by a pediatric rheumatologist was performed between 0 and 14 days after completing JAMAR. Details about age, gender, therapy alterations or additional examinations during or after the hospital visit were collected. χ^2^-test was used for categorical variables, T-tests for normally distributed variables and Kruskal-Wallis Rank Sum test for non-normally distributed variables.

**Results:** A total of 778 participants were included, each with one questionnaire. 488 were completed by parents and 290 by children. 286/778 (36.8%) participants reported 0 SPJC of whom 236 (82.5%) had an AJC of 0. The association between the SPJC of 0 and an AJC of 0 was statistically significant (χ^2^ = 187.8, *p-value= <0.01).*

Of the 50 participants stating a SPJC of 0, but with an AJC>0, only 4 (8%) had an active joint that cannot be reported in the JAMAR. The AJC of these 50 patients varied between 1 and 9. Participants with polyarticular JIA were more often incorrect about having no active joints (20 participants (40%), *p-value= <0.01*).

Participants with active joints did however report a significantly higher VAS disease activity (median 1.0 [IQR: 0.0-8.3], *p-value= 0.03*) and VAS pain (median 2.0 [IQR 0.0-10.0], *p-value= 0.01*) in the JAMAR compared to those with an AJC of 0 (median: 0.0 [0.0-2.0] and (median 0.0 [IQR: 0.0-3.3] for disease activity and pain respectively). Furthermore, stating in the JAMAR that the current disease state was “remission” was indeed associated with having an AJC of 0 (88.3% in the AJC of 0 group vs 73.9% in the AJC≥1 group, *p-value= 0.02*).

Of the 50 participants with an AJC≥1 20 (40%) had therapy adjustments, 4 (8%) underwent further imaging, and 1 (2%) got advice about improving therapy adherence.

**Conclusion:** The patient reported absence of SPJC is associated with the AJC. Of the people who report an SPJC of 0 83% indeed had an AJC of 0. However, if only the SPJC is considered, 7% of patients who are in need of therapy adjustments will be missed.

**Patient Consent**

Not applicable (there are no patient data)

**Disclosure of Interest**

None declared

## P217 Data visualization over time of the juvenile arthritis multidimensional assessment report using an electronic web-based dashboard

### M. Doeleman, T. Rutjes, N. Wulffraat, J. Swart, S. De Roock

#### Paediatric Immunology & Rheumatology, UMC Utrecht, Utrecht, Netherlands

##### **Correspondence:** M. Doeleman

*Pediatric Rheumatology 2023*, **21(Suppl 2):**P217

**Introduction:** The Juvenile Arthritis Multidimensional Assessment Report (JAMAR) is a questionnaire consisting of 15 patient-centered measures on important dimensions of health and disease regarding patients with JIA^1^. Despite its value for clinical practice, a single JAMAR provides only a cross-sectional view of the patient and is impractical to view in its entirety during outpatient clinics. In result, useful information and trends of disease-related parameters may be overlooked.

**Objectives:** Development of an electronic dashboard to visualize JAMAR questionnaires over time based on requirements of paediatric rheumatologists and patients with JIA.

**Methods:** Paediatric rheumatology outpatient clinics were observed to gather information on the use of the JAMAR. One paediatric rheumatologist and four patients/parents were interviewed using semi-structured interviews to gather requirements for the visualizations and user experience. The dashboard was developed using the Shiny package and R programming language^2^, and evaluated by giving members of the paediatric rheumatology team (4 physicians and 1 nurse) specific tasks while using the dashboard (i.e. task-based analysis).

**Results:** During outpatient clinics, a lack of usage of the information provided by the JAMAR was observed. Patients/parents noted during interviews that clinicians often ask redundant questions that are already answered via the JAMAR. Although the JAMAR is accessible in our electronic health records, several clicks are required to reach important information and only one JAMAR can be viewed at the same time. Both clinicians and patients expressed interest in JAMAR outcomes and described them as valuable. Primary requirements of the dashboard were that 1) all answers of the JAMAR would be accessible; and that 2) JAMAR data could be viewed over time for each individual patient. During evaluation of the dashboard, members of the paediatric rheumatology team were tasked with finding patients and identifying trends in JAMAR responses over time. All participants completed the tasks without any problems and stated that the dashboard was intuitive to use. An interactive demo of the JAMAR dashboard can be found at: https://mjhdoeleman.shinyapps.io/jamar-dashboard-demo.

**Conclusion:** The JAMAR dashboard enables to view questionnaire responses over time, identify improvement or worsening of disease course, and easily discuss the course of JAMAR dimensions with patients and their parents (e.g. discussing “targets” for treat-to-target strategies). Data visualization of important information could add value and efficiency to outpatient clinics for both clinicians and JIA patients. Due to the flexibility of the Shiny framework, the developed dashboard can be easily adapted to other types of data, clinical parameters, or patient-reported outcomes. Clinical implementation of the JAMAR dashboard and development of additional features is currently ongoing.

**Patient Consent**

Not applicable (there are no patient data)

**Disclosure of Interest**

None declared

**References**

1. Filocamo G, Consolaro A, Schiappapietra B, et al. A new approach to clinical care of juvenile idiopathic arthritis: the juvenile arthritis multidimensional assessment report. *J Rheumatol.* 2011;38(5):938–53. 10.3899/jrheum.100930
2. R Core Team (2022). R: A language and environment for statistical computing. R Foundation for Statistical Computing, Vienna, Austria. https://www.R-project.org/

## P218 Comparison of functional isometric muscle strength in the upper limb between patients with juvenile idiopathic arthritis and familial Mediterranean fever using a novel device

### I. Donmez^1^, N. Arman^1^, A. Yekdaneh^2,3^, Y. Acıkgoz^1^, A. Albayrak^3^, K. Ucar^1^, N. Aktay Ayaz^4^

#### ^1^Faculty of Health Sciences Department of Physiotherapy and Rehabilitation, Istanbul University-Cerrahpasa; ^2^Vocational School of Health Services Physiotherapy English Program, Fenerbahce University; ^3^Institute of Graduate Studies Physiotherapy and Rehabilitation Doctorate Program, Istanbul University-Cerrahpasa; ^4^Istanbul Faculty of Medicine, Department of Pediatrics, Department of Pediatric Rheumatology, Istanbul University, Istanbul, Türkiye

##### **Correspondence:** I. Donmez

*Pediatric Rheumatology 2023*, **21(Suppl 2):**P218

**Introduction:** Juvenile Idiopathic Arthritis (JIA) and Familial Mediterranean Fever (FMF) are chronic, inflammatory, and autoimmune disorders that are prevalent in the pediatric population, with substantial impact on affected individuals’ muscle strength and daily functioning (1,2). Upper extremity muscle strength affects performance in activities of daily living (ADL). Therefore, assessing muscle strength is important for setting an exercise program as a treat to target for improving ADL (3). However, manual assessment of muscle strength in children is too hard, novel functional methods that are easy to apply are needed.

**Objectives:** The aim of the study was to examine muscle strength by conducting an objective evaluation of the isometric contraction forces of the shoulder girdle muscles in children and compare the results between children with JIA and FMF.

**Methods:** This study included 33 children with JIA and 22 children with FMF. The isometric muscle strength of the shoulder girdle muscles was objectively evaluated using a novel device called the K-Push, a hand-held dynamometer (Kinvent Physio, France). The device was presented to the participants and measurements were conducted in standing positions in 0° and 90° shoulder flexion for clamping force, 90° elbow flexion for push-down force, 90° shoulder flexion while standing for push-front wall force, and 90° shoulder abduction while standing for push-side wall force. Participants performed three repetitions of maximum isometric contractions for 5 seconds in each position, and the values were recorded using the K-Push software on a smartphone. A rest of 15 seconds was allowed between repetitions. No verbal encouragement was given during measurement. Values were not shared with the participants, in order for the results and motivation not to be affected. The peak force values, time to reach maximum force, mean force values, fatigue, and rate of force development (RFD) were recorded for every measurement. SPSS Version 24.0 program was used for statistical analysis.

**Results:** The mean age of patients with JIA and FMF was 13.39±1.74 and 13.45±1.79 years, respectively. Significant differences were observed between the two groups in the following parameters: peak and mean force for 0° clamping flexion, peak force, mean force and RFD for 90° clamping, peak and mean force for push down, maximum, peak and mean forces for push front wall and mean force for push side wall (p<0.05). Moreover, patients with JIA had significantly lower scores than patients with FMF in all measures.

**Conclusion:** Functional upper extremity muscle strength was significantly lower in patients with JIA compared to patients with FMF. This condition may be associated with multiple joint involvement, systemic factors, severe muscle inflammation and fatigue. The decrease in muscle strength should be considered and evaluated when setting an exercise program for both diseases. Testing muscle strength using a handheld dynamometer with a smartphone application is user-friendly because of ease of use and quick access to results and provides comprehensive information of muscle strength in patients with JIA and FMF.

This study was supported within the scope of the Scientific and Technological Research Council of Turkey (TUBITAK) 1001-Scientific and Technological Research Projects Support Program (Project number: 121E690).

**Patient Consent**

Yes, I received consent

**Disclosure of Interest**

None declared

**References**

1. Martini A, Lovell DJ, Albani S, Brunner HI, Hyrich KL, Thompson SD, Ruperto N. Juvenile idiopathic arthritis. Nat Rev Dis Primers. 2022 Jan 27;8(1):5. 10.1038/s41572/021/00332/8
2. Tufan A, Lachmann HJ. Familial Mediterranean fever, from pathogenesis to treatment: a contemporary review. Turk J Med Sci. 2020 Nov 3;50(SI-2):1591-1610. 10.3906/sag/2008/11
3. Hoeksma AF, van Rossum MA, Zinger WG, et al.: High prevalence of hand-and wrist-related symptoms, impairments, activity limitations and participation restrictions in children with juvenile idiopathic arthritis. J Rehabil Med 2014;46:991–6. 10.2340/16501977-1879

## P219 Virtually the same, but remotely different: experiences of video and telephone appointments for juvenile idiopathic arthritis

### C. Pain^1,2^, H. Saron^3^, B. Carter^3^, J. Sandars^3^, J. Ainsworth^1^, L. Whitty^1^, I. Sinha^1,4^, S. Singhal^2^, G. Cleary^2^, J. Downing^5^, T. Marson^5,6^

#### ^1^Women’s and Children’s Health, University of Liverpool; ^2^Paediatric Rheumatology, Alder Hey Children's NHS Foundation Trust; ^3^Faculty of Health, Social Care and Medicine, Edge Hill University; ^4^Respiratory, Alder Hey Children's NHS Foundation Trust; ^5^Applied Research Collaborative, North West Coast, National Institute for Health and Social Care Research; ^6^Pharmacology and Therapeutics, University of Liverpool, Liverpool, United Kingdom

##### **Correspondence:** C. Pain

*Pediatric Rheumatology 2023*, **21(Suppl 2):**P219

**Introduction:** The COVID-19 pandemic required a rapid adaptation to virtual (telephone/video) appointments, previously uncommon in paediatric practice. There are no experience-based or qualitative studies exploring the perspectives of clinicians, parents and/or children and young people (CYP).

**Objectives:** To explore the experiences of CYP, parents and clinicians of remote (telephone/video) appointments for juvenile idiopathic arthritis (JIA) and co-create health information resources to improve virtual appointments.

**Methods:** Observations of virtual appointments and semi-structured interviews with clinicians, parents and CYP.

**Results:** CYP (7-18 yrs), parents and clinicians were observed during telephone (n=5) and video (n=11) appointments. CYP (n=8), parents (n=5 mothers, n=1 father) participated in interviews. Ten interviews with 7 clinicians (n=4 doctors, n=2 nurse specialists, n=1 physio) were conducted.

**Virtually the same:** Virtual appointments were conducted in a consistent way, following a similar frame-work to face-to-face (F2F). CYP and parents perceived this as a benefit. Overall, virtual appointments were experienced positively by all. Participants were confident the appointment covered everything it needed to and that the outcomes were the same as F2F. Clinicians were generally positive about virtual appointments if it was *‘right for everyone, at the right stage of a child’s disease’*.

**Remotely different:** Differences between virtual and F2F appointments influence the overall experience. Virtual appointments save time and money but change the ‘whole’ hospital experience for families: *‘I quite like the video because it means that I don’t have to go on a long drive. So, it means that I miss less school (Girl, 16yrs).* Some raised concerns about clinical examination as this was the most notable difference: *I think it should be a mix [hospital and remote]. Because with the doctors, they fully examine you [at hospital] (Girl, 14yrs).*

An animation, and information leaflets to improve experiences of remote appointments were co-created in a workshop with CYP and their families.

**Conclusion:** Clinicians have adapted to new ways of working that they feel is safe when patients are stable and when all stakeholders are able to use the technology. Despite positive experiences and the hope that virtual consultations remain an option, most of those interviewed wanted a choice on whether the next appointment would be F2F or remote and that the decision on the type of appointment should be through shared decision-making with CYP, parents and clinicians. Concerns stem from a lack of understanding about what will happen during a virtual appointment and sometimes a disconnect between expectation and reality.

**Trial registration identifying number:** N/A

**Patient Consent**

Not applicable (there are no patient data)

**Disclosure of Interest**

None declared

## P220 Development of a web app to evaluate hypermobility with the beighton score at all ages

### B. H. León^1^, M. Vélez Arteaga^1^, B. I. Nicolalde^1^, J. Ibarra-Fiallo^2^, J. Ibarra Delgado^2^, G. Carrera ^1^

#### ^1^Ciencias de la Salud, ^2^Ciencias e Ingenierias, Universidad San Francisco de Quito, Quito, Ecuador

##### **Correspondence:** B. Leon

*Pediatric Rheumatology 2023*, **21(Suppl 2):**P220

**Introduction:** People with hypermobility are characterized by an increased passive and/or active range of movement in their joints. Hypermobility could be a typical characteristic of an individual, part of a benign syndrome, or, in rare cases,one constituent of a Heritable Connective Tissue Disorder. This can present as Generalized Joint Hypermobility (GHJ) with a prevalence of 1 in 500. Hypermobility is a genetic trait, but age, sex, exercise, nutrition, trauma, and ethnicity seem to contribute to its natural history, with few studies describing its incidence through infancy and adolescence. GJH, in turn, can frequently affect children and adolescents presenting with symptoms like chronic articular, muscle, or widespread pain; fatigue, and joint dysfunction that require identification by general practitioners and specialized physicians, but its evaluation is frequently omitted during physical examination. The Beighton Score (BS) is a method used to assess hypermobility during physical examination, is part of the 2017 International Classification of Ehlers-Danlos Syndrome and is used in research studies of GJH. Unfortunately, it is commonly forgotten during physical examination, so hypermobility and its possible associations go undiagnosed. A user-friendly and accessible digital tool to evaluate hypermobility across different age groups may address this oversight.

**Objectives:** To create a Web App that uses the Beighton Score to evaluate hypermobility across all ages.

**Methods:** A questionnaire was administered to 80 medical students of different levels and universities to assess their knowledge of hypermobility. Less than 40% remembered the BS and less than 20% recall the nine required maneuvers or the scoring system. After looking at the BS in a paper, students promptly remember the scale. We teamed up with the IT department to develop an App to (re) educate health professionals and to make the BS available for consultation. The prototype is currently at: www.hypermov.com (for best results, use Firefox browser); once live, future iterations will include a https secure SSl certificate.. Data-wise Hypermov collects the data inputted directly from the user and calculates the BS. These results are then sent to a server and classified by the user's location, gender, ethnicity, and age. We added to the regular positive and negative questions to score each of the 9 maneuvers of the BS, one of “doubt” in order to document how the BS has a subjectivity level that has been addressed by several authors and needs to be quantified in future studies.

Data collection is used for epidemiological and demographic purposes only and given an anonymity layer. No personal information is required nor is there any way to identify or track users.

The App was tested from a user experience standpoint to ensure its accessibility and user friendly capability.To gather insights about the main flow of the App, an interaction prototype of HyperMov was sent to 65 testers of various medical and not health related backgrounds. IT then corrected difficulties and errors that users of any sector might have experienced.

**Results:** Using the system usability scale, we created a survey about the Hypermov Website App's preliminary version answered by 65 subjects: 46.3% were attending physicians in university settings, 9% residents, 22.4% medical students, and 22.3% non-medicine-related individuals. The majority of these subjects, that is 93.5%, agreed that HyperMov was easy to use, 84% stated that they would recommend the App, 50.7% considered it a valuable assessment of initial evaluation of hypermobility and 63.7% answered that it is a fine educational tool. The time registered for completing the BS app was: 22.7% people used less than a 1 min, 39.4% had the result of their score in 1-2 min, 31.8% in 3-4 min, and 6.1% took more than 4 min.

**Conclusion:** HiperMov.com is a user- friendly Web App that helps assess hypermobility across different age groups. It was recommended as an educational tool by most medical attending physicians that participated in our survey and may aid users to easily identify hypermobility in order to seek further medical attention if needed.

**Patient Consent**

Not applicable (there are no patient data)

**Disclosure of Interest**

None declared

## P221 Analysis of postural stability during half-squat in patients with juvenile idiopathic arthritis: a pilot study

### A. Yekdaneh^1^, N. Arman^2^, A. Albayrak^3,4^, V. Guliyeva^5^, N. Aktay Ayaz^5^

#### ^1^Vocational School of Health Services Physiotherapy English Program, Fenerbahce University; ^2^ Faculty of Health Sciences Department of Physiotherapy and Rehabilitation, Istanbul University-Cerrahpaşa; ^3^Institute of Graduate Studies Physiotherapy and Rehabilitation Doctorate Program, Istanbul University-Cerrahpasa; ^4^Faculty of Health Sciences, Department of Physiotherapy and Rehabilitation, Istanbul Kent University; ^5^Istanbul Faculty of Medicine, Department of Pediatric Rheumatology, Istanbul University, Istanbul, Türkiye

##### **Correspondence:** A. Yekdaneh

*Pediatric Rheumatology 2023*, **21(Suppl 2):**P221

**Introduction:** Juvenile Idiopathic Arthritis (JIA) is a chronic disease characterized by joint swelling, pain and limitation (1). Decreased lower extremity performance and joint range of motion, and associated low physical function and impaired postural stability are observed in patients with JIA (2). The squat, which is frequently used to evaluate functional status and balance performance, is associated with lower extremity neuromuscular control. During the squat, especially the eccentric strength and endurance of the quadriceps muscle is important for stability and performance (3,4). Due to the impaired neuromuscular control seen in patients with JIA, it is important to evaluate body weight-bearing tasks such as squats and to evaluate the performance, dynamic postural stability and movement speed of patients during these tasks.

**Objectives:** The aim of this study was to evaluate the dynamic postural stability of patients with JIA during half-squat and comparing with healthy children.

**Methods:** 29 children and adolescents aged 10-18 years who have at least one lower extremity joint involvement (15 Girls,14 Boys) with JIA and 10 healthy (9 Girls,1 Boys) peers were included. The postural stability of the participants during the half-squat was evaluated with the Kinvent Physio K-FORCE Plates (Kinvent Physio, France) that enables measurement of static and dynamic balance in a wide range of movements. Sway surface areas, displacements from the center of gravity and displacement velocities were recorded. SPSS Version 24.0 program was used for statistical analysis.

**Results:** The mean age of children and adolescents with JIA and healthy was 13.79±1.78 and 14.5±1.26 years, respectively. The mean score of left and total surface areas was statistically higher in patients with JIA while the amount of displacement from the center of gravity was lower, compared to their healthy peers (p<0.05).

**Conclusion:** It is seen that the dynamic postural stability during the half-squat is negatively affected in patients with JIA with lower extremity involvement, compared to their healthy peers. These results suggest that the fact that patients with JIA with lower extremity involvement have more sway surface areas and less displacement during half-squat may be related to the need for more corrective movements and effort expenditure in order to provide postural stability during squat movement. Therefore, we believe that it may be important to include function-oriented objectively assessments and exercises such as squats to improve neuromuscular control and postural stability in the lower extremities for children and adolescents with JIA, when setting an exercise program.

This study was supported within the scope of the Scientific and Technological Research Council of Turkey (TUBITAK) 1001-Scientific and Technological Research Projects Support Program (Project number: 121E690).

**Patient Consent**

Yes, I received consent

**Disclosure of Interest**

None declared

**References**

1. Woolnough L et al. Juvenile idiopathic arthritis, gait characteristics and relation to function. Gait Posture. 2021;85:38–54.
2. TAKKEN T et al. Physical activity and health related physical fitness in children with juvenile idiopathic arthritis. Ann Rheum Dis 2003; 62:885-9.
3. Talarico, Maria et al. (2019). Dynamic Postural Control Is Influenced by Single-Leg Squat Speed and Depth Under Single-Task and Dual-Task Paradigms. Journal of Applied Biomechanics.
4. Endo Y et al. The relationship between the deep squat movement and the hip, knee and ankle range of motion and muscle strength. J Phys Ther Sci. 2020;32(6):391-394.

## P222 A case report of Henoch-Schonlein purpura secondary to COVID-19 infection improved by administration of colchicine

### A. M. A. Abushhaiwia^1^, N. Elajnef^1^, A. Ateeq^2^

#### ^1^Pediatric Rheumatology, University of Tripoli, faculty of Medicine, Tripoli Children's Hospital; ^2^Pediatric Rheumatology , Tripoli Children's Hospital, Tripoli, Libya

##### **Correspondence:** A. M. A. Abushhaiwia

*Pediatric Rheumatology 2023*, **21(Suppl 2):**P222

**Introduction:** HSP is a vasculitis of small vessels involving multiple organs. Viral infections are known triggers of leucocytoclastic vasculitis. There have been multiple reports in literature about different presentations of COVID-19 in children. There is a spectrum of dermatological and vascular presentations which may range from mild skin rashes to severe arterial strokes.

**Objectives:** To describes a 3-year-old boy who presented with purpuric rashes following a completely asymptomatic COVID-19 infection,

**Methods: case report**

**Results:** 3-year-old Libyan boy child , previously healthy, presented to pediatric Rheumatology with a history of skin rashes on both lower &upper extremities of one month duration. His family medical history was positive for autoimmune disease and renal disease (one sibling has nephrotic syndrome). The rash started on his lower legs which spread to his upper limbs, his both ears and face over time. The rash was petechial and purpuric in nature. It was palpable and non-pruritic. There was history of abdominal pain, fever, joint pain. A significant medical history included fever ,URTI infection around 4 weeks prior to the onset of rashes . On physical examination, growth parameters were normal. He was afebrile and his blood pressure was 80/45 mm Hg. His respiratory rate was 16/min .His skin examination revealed rashes involving the dorsal feet and lower extremities. There were scattered non-blanching round dark red palpable petechiae and macular skin rashes, Initial examination revealed a purpuric rash more prominent on the lower extremities than the upper extremities with mild tenderness to palpation of the abdomen. Laboratory values revealed CRP was negative <1 mg/L, (WBC) 10,2 x 103\mm^3^ (neutrophils 38.4%, lymphocytes 52.3%), hemoglobin 13 g/dL, platelet count 340x103\mm^3,^ESR 20mm\hr, blood urea 10 mg/dL, creatinine 0.29 mg/dL. Normal UPC >0.2 mg/mg. Urine microscopy was nile. Serum complement levels demonstrated normal level of C3 (123 mg/dL, normal range 80–160 mg/dL) and low level of C4 (14.7mg/dL, normal range 16–48 mg/dL),His ANA screen was negative, ANCAp,c were negative. COVID-19 IgG was positive with high titter 79.52, COVID-19 IgM was negative. Chest X-ray ,ECG and echo were normal, skin biopsy was not undertaken, but based on the features of his clinical presentation, Diagnosis of HSP was made it .The child was given a course of oral prednisolone (1 mg/kg per day) for 2 weeks followed by gradual tapering over the next 4 weeks. The patient’s persistent lesions regardless of oral prednisone. Therefore, we suggest that our patient developed a hypersensitivity reaction and vasculitis secondary to COVID-19.His symptoms improved significantly with colchicine 0.5mg once per day treatment and the rash continued to fade and finally healed with hyperpigmentation within few days.

**Conclusion:** HSP following a SARS-CoV-2 infection. Can be treated by colchicine

**Patient Consent**

Yes, I received consent

**Disclosure of Interest**

None declared

## P223 Impact of COVID-19 on children with rheumatological disorders in Ireland

### A. Alkandari, E. Rowe, K. Peate, D. Deely, K. Gallagher, E. J. MacDermott, O. G. Killeen

#### National Centre for Paediatric Rheumatology, Our Lady's Children's Hospital, Crumlin, Dublin , Ireland

##### **Correspondence:** A. Alkandari

*Pediatric Rheumatology 2023*, **21(Suppl 2):**P223

**Introduction:** In early 2020, coronavirus disease 2019 (Covid-19) spread quickly to become a global pandemic. We now know that children tend to experience a less severe form of the disease than the adult population and that patients with decreased immunity and chronic diseases are at a gretaer risk of severe illness and morbidity.

**Objectives:** This study describes how Covid-19 has affected paediatric rheumatology patients since the pandemic began in Ireland.

**Methods:** This study was an online survey, a paper form was available if the parents preferred. The patients were given information leaflets about the study at all hospital visits, including clinic and inpatient stays. Patients were also informed of the survey if they contacted the rheumatology nurse helpline, and they received information by mail. To make the survey easier for families to access, a QR code was provided so that parents could scan it and go directly to the survey. The survey included 19 primary questions, some of which led to additional inquiries based on participant responses. The participants could access the online survey between August and December 2022.

**Results:** A total of 77 participants took the online survey, 61 of whom completed it. Juvenile idiopathic arthritis (JIA) was the most common diagnosis, accounting for 58.3% of cases, followed by chronic nonbacterial osteomyelitis (CNO) with 9.7% and Down syndrome associated arthritis (DA) 6.4%. Girls accounted for 64.7% of the patient population, while boys accounted for 35.2%. Prior to contracting Covid-19, 55.7% of participants were in clinical remission either on or off medication at their last clinic visit, while 44.2% were aware that their disease was still active as per their last clinical assessment. Only 1 patient had confirmed COVID in 2020. Most patients (63%) contracted COVID 19 in 2022. Additionally, 15% of the participants experienced 2 confirmed episodes, and one participant was infected on 3 separate occasions. The most common method for diagnosing Covid-19 infection (50.7%) was antigen testing, with PCR confirmation in 26% of cases, and 23% of individuals had positive results with both tests. Diarrhoea was the least-reported symptom at 6%, while fatigue was the most reported symptom at 66%. Interestingly, one patient reported hearing loss during active infection. Of the patients, 68.7% withheld their medication during the infective episode, and 55.5% contacted our team when they did confirm infection. Additionally, 12.7% of participants required general practitioner or hospital visits at the time of infection. Hospital admission was required for four patients, but none required oxygen therapy or intensive care support. Before contracting the disease, 53.9% had received a Covid-19 vaccine; of those, 52% had received two doses and 26.4% had received three. A total of 80% had not received the booster shot although they were approved for the same.

**Conclusion:** Despite being on immunosuppression therapy, Covid-19 infection did not result in fatal disease in our patients, as earlier studies had reported. Due to lockdown constraints and social distancing including high adherence with facial masking in our country, it seems that most patients acquired their infection later in the pandemic, rather than at the beginning. There was a high rate of vaccine uptake in this cohort – half of the participants were vaccinated before infection – which may have contributed to milder infections.

**Patient Consent**

Not applicable (there are no patient data)

**Disclosure of Interest**

None declared

## P224 Covid-19 in children with rheumatic diseases, the largest tertiary center experience from Saudi Arabia

### A. I. Almojali, on behalf of Jubran Alqanatish, Amal Ahmed, Abdulmajeed Alfadhel, Areej Albelali, Suliman Alghnam

#### Pediatric Rheumatology, King Abdullah Specialised Children's Hospital, Riyadh, Saudi Arabia

##### **Correspondence:** A. I. Almojali

*Pediatric Rheumatology 2023*, **21(Suppl 2):**P224

**Introduction:** Coronavirus disease 2019 (COVID-19) pandemic is a relatively new global health issue affecting multimillions individuals around the world. The literature still deficient for pediatric studies in this filed, especially when it comes to those with rheumatic diseases.

**Objectives:** To identify the rate, clinical manifestations, risk factors, and outcome of COVID19 infection in children and adolescents with rheumatic diseases.

**Methods:** Retrospective cohort study of pediatric rheumatology patients (< 19 years old) who presented to King Abdullah Specialized Children's Hospital, Riyadh, Saudi Arabia, as outpatients, inpatients, day care and/or emergency department visits during the period of March 2020 to March 2022.

**Results:** Among 482 pediatric patients with rheumatic diseases, 126 (26.1%, 95 CI: 21.8%–31.1%) contracted COVID-19 infection. Fever was the most frequent clinical manifestation (55.6%, n= 70), followed by respiratory symptoms (50.8%, n= 64), and almost 30% of patients (37 patients) were asymptomatic. Most of the patients recovered without complications (84.9%, n= 107), and mortality was reported in 3 patients (2.38%). A total of 19 patients were hospitalized, representing 15.1% of all positive cases. There was no statistically significant association between gender, age groups, pre-existing comorbidities, COVID-19 vaccine doses, nor using traditional or biological DMARDs with contracting COVID-19 infection. However, hospitalization was significantly associated with male gender (p=0.009), young age (p<0.001), vaccination status (p<0.001), b-DMARDs (p<0.001) and glucocorticoids (p<0.001). The risk of hospitalization was almost 6 times higher in males (OR=5.97, 95%CI: [1.44-24.71]), and was higher in patients receiving b-DMARDs (OR=17.53, 95%CI: [1.8-165.9]) or glucocorticoids (OR=6.69, 95%CI: [1.46-30.64]). Vaccinated children were at lower risk to be admitted due to COVID19 in comparison to those who didn’t receive the vaccine (OR=0.09, 95% CI: [0.02-0.654]).

**Conclusion:** In our cohort of patients (<19 years) with rheumatic diseases, one quarter of them had COVID-19 infection during the last 2 years. Most of the time, the course of illness is mild and self-limiting with no complications. Results suggest that exposure to b-DMARDs or glucocorticoids increase the risk of hospital admission. As expected, receiving COVID19 vaccine lower the risk of hospitalization during the infection.

**Patient Consent**

Not applicable (there are no patient data)

**Disclosure of Interest**

None declared

## P225 A case of a severe cutaneous vasculitis associated with SARS-COV-2 virus

### P. R. Ambaram, B. J. Mistry

#### ^1^Department of Paediatrics and Child Health, University of the Witwatersrand, Johannesburg, South Africa

##### **Correspondence:** P. R. Ambaram

*Pediatric Rheumatology 2023*, **21(Suppl 2):**P225

**Introduction:** Very few cases of Coronavirus disease 2019 (COVID-19) associated paediatric vasculitis have been reported. Most cases in the literature have presented with phenotypic features of IgA vasculitis followed by chilblains and ANCA associated vasculitis. To our knowledge, no case of severe COVID 19 associated cutaneous vasculitis that have either a DADA2 (Deficiency of adenosine deaminase 2), SAVI (STING-associated vasculopathy with onset in infancy) or PAN (Polyarteritis Nodosa) phenotype have been described.

**Objectives:** We describe a case of an infant with positive SARS-CoV-2 antibodies that presented with severe progressive cutaneous vasculitis refractory to conventional vasculitis treatment but eventually responded well to biologic therapy.

**Methods:** Our patient, a South African female, presented in January 2021 at 6 months of age when South Africa was experiencing a second wave of Covid 19 cases. The history was that of a 3-week duration of cold peripheries, blue fingertips, and ulcers on her feet. Her skin lesions involved peripheral sites including digits of her hands and feet, as well as her nose and cheeks. In addition, she presented with a large atrial septal defect and a pulmonary embolism. SARS-CoV-2 PCR was negative on admission, but the antibodies were positive. A CT angiogram showed features of pulmonary hypertension with no aortitis, central or peripheral vasculitis, however a skin biopsy confirmed a vasculitis.

**Results:** She was initially treated as a Multisystem Inflammatory Syndrome in Children (MIS-C) and given intravenous corticosteroids, immunoglobulin, and anticoagulation therapies. Subsequently, she was given intravenous Iloprost and six cycles of cyclophosphamide but continued to have worsening cutaneous vasculopathy with necrosis and ischemia involving mainly her hands and feet. Considering her age and the nature of presentation, genetic testing was done to exclude a hereditary systemic autoinflammatory disease, which was negative for suspected DADA2 and SAVI. Finally, she has showed a favourable response to Etanercept with resolution of her cutaneous lesions despite autoamputation of her distal phalanges.

**Conclusion:** Our patient presented with features of a severe small to medium vessel vasculitis in the presence of SARS-CoV2 antibodies that responded only to Etanercept which then raised the possibility of an underlying hereditary systemic autoinflammatory disease. Similar to other studies, it remains unclear whether SARS-CoV-2 is the cause or the trigger of the vasculitis.

**Patient Consent**

Yes, I received consent

**Disclosure of Interest**

P. Ambaram Grant / Research Support with: Sponsorship accepted from Pfizer to attend the PReS 2023 congress, B. Mistry Grant / Research Support with: Sponsorship accepted from Pfizer to attend the PReS 2023 congress

**References**

1. Batu ED, Sener S, Ozen S. COVID-19 associated pediatric vasculitis: A systematic review and detailed analysis of the pathogenesis. Semin Arthritis Rheum.
2. Ozen S, Batu ED, Taşkıran EZ, et al. A Monogenic Disease with a Variety of Phenotypes: Deficiency of Adenosine Deaminase 2. J Rheumatol. 2020;47(1):117-125.
3. Romita P, Maronese CA, DE Marco A, et al. COVID 19-associated chilblain-like acral lesions among children and adolescents: an Italian retrospective, multicenter study. Ital J Dermatol Venerol. 2023;158(2):117-123.
4. Patel, S., Jin, L. TMEM173 variants and potential importance to human biology and disease. Genes Immun 2019, 82–89.
5. Akhade K, Ganguly S, Nanda R, Mohapatra E, Goel AK. Cutaneous manifestations associated with COVID-19 in children: A systematic review. J Family Med Prim Care. 2021 Jan;10(1):93-101.
6. Singh H, Kaur H, Singh K, Sen CK. Cutaneous Manifestations of COVID-19: A Systematic Review. Adv Wound Care (New Rochelle). 2021;10(2):51-80.

## P226 Evaluation of COVID-19 and COVID-19 vaccination in pediatric rheumatic diseases

### G. Aytac, B. R. Taskın, I. Aydin, S. Imamoglu, G. Aksu, A. Berdeli, N. Kutukculer

#### Pediatric Rheumatology, Ege University, Izmir, Türkiye

##### **Correspondence:** G. Aytac

*Pediatric Rheumatology 2023*, **21(Suppl 2):**P226

**Introduction:** The World Health Organization (WHO) officially declared the SARS-CoV-2 outbreak a global pandemic on March 2020. Since the outset of the COVID-19 pandemic, risk factors for severe disease have been identified. It is not clear whether patients with rheumatic disease are at increased risk of serious COVID-19 disease. The relationship between infections and rheumatic diseases has been known for a long time. Infectious agents may play a role in the etiology, and may trigger rheumatic diseases.

**Objectives:** Patients with systemic autoimmune rheumatic diseases are considered at risk of more serious infections, both because of the immune dysregulation that characterizes these diseases and/or the use of immunosuppressive therapy, but it is unclear whether they are more susceptible to developing COVID-19. We aimed to evaluate the course of COVID-19 disease in patients with pediatric rheumatic disease, to examine the relationship between rheumatic disease activation and transmission of COVID-19 disease, and to evaluate the relationship of COVID-19 vaccines with side effects and disease activation in patients with pediatric rheumatic disease

**Methods:** We examined the patients who applied to the pediatric rheumatology department. The patients were informed about the study and received written informed consent. Through a standard pre-designed questionnaire, participants were asked whether they had COVID-19, their covid symptoms, and their immunization status. Questionnaires were asked face to face and by telephone

**Results:** We included 471 pediatric patients with primary rheumatic diseases. 142 (89.9%) patients had at least one COVID-related symptom, while 16 (10.1%) were asymptomatic when diagnosed with COVID. The most common symptom was fever and fatigue, reported in 61.8% of, followed by headache (%44.6), sore throat (%41.4), myalgia (%39.5) cough (38.2%), and 16 (10.1%) cases asymptomatic. Thirty of the patients (21.4%) reported an increase in the frequency of disease flares after COVID-19, and 110 of the patients (%78.6) didn't report disease flare or occurrence of any additional late complication.

**Conclusion:** We did not have any patients who needed hospitalization or intensive care admission. In our study, 10.1% were asymptomatic. We found that chronic rheumatic diseases were not associated with poor outcomes in children with COVID-19. Vaccine-related side effects were found to be 23.6%, and the frequency of disease activation after vaccination was 4.6%.Safety issues of Covid- 19 vaccines in rheumatic patients are unknown, as most clinical trials of vaccines exclude immunosuppressant patients.

**Patient Consent**

Yes, I received consent

**Disclosure of Interest**

None declared

**References**

Ferri C, Giuggioli D, Raimondo V;Andolina M, et al. COVID-19 and rheumatic autoimmune
  systemic diseases: report of a large Italian patients series Clin Rheumatol 2020 Nov;39(11):3195-3204
2. Arachchillage DJ, Rajakaruna I, Pericleous C, et al. Autoimmune disease and COVID-19: a multicentre observational study in the United Kingdom Rheumatology (Oxford). 2022 Dec; 61(12)
Furer V, Rondaan C, Levin A. N, et al. Point of view on the vaccination against COVID-19 in patients with autoimmune inflammatory rheumatic diseases, RMD Open2021 Feb;7(1

## P227 Differences and similarities of pediatric inflammatory multisystemic syndrome and Kawasaki disease: a comparative mono-centric Moroccan cohort

### K. Bouayed^1,2^, M. Jalal^1^, G. Benbrahim Ansari^1,2^, H. Aboufaris^1^, A. Sakhi^1,2^ on behalf of Kawarabi

#### ^1^Pediatric Rheumatology, Hôpital Mère-Enfant A. Harouchi, CHU Ibn Rochd; ^2^Faculté de Médecine et de pharmacie, Université Hassan II, Casablanca, Morocco

##### **Correspondence:** K. Bouayed

*Pediatric Rheumatology 2023*, **21(Suppl 2):**P227

**Introduction:** Kawasaki disease KD is a multisystemic vasculitis that affects median and small caliber vessels with a predominance for coronary arteries. Il is related to the new syndrome called Pediatric Inflammatory Multisystemic Syndrome PIMS which is a post infectious inflammatory disease occurring after SARS COV2 infection. Although these two entities share many similarities, there are a marked differences.

**Objectives:** To compare the two diseases to highlight specific criteria for each group and to describe demographic, clinical, biological and therapeutic features.

**Methods:** Retrospective prospective observational study: Pre-pandemic "retrospective period" January 2019 - February 2020. During and after the pandemic "prospective period" March 2020 - February 2023. Comparison of KD patients with a diagnosis based on American Heart Association criteria with PIMS patients diagnosed on sustained fever, inflammatory syndrom and positive COVID 19 serology.

**Results:** Among 92 patients, 63 KD and 29 PIMS, with a male predominance. The median age was 2.7 years for KD patients and 4 years for PIMS. All patients had sustained fever with a median duration about 7 days. 80% of KD patients had conjunctivitis compared to 68% of PIMS patients. Rash, extremity involvement, and cervical adenopathy were reported in both diseases with percentages of 60, 54.5 and 23 for KD versus 62, 31, and 25 for PIMS, respectively. Abdominal pain was reported in 3.7% of KD cases versus 54.2% of PIMS cases.

All patients had marked inflammatory syndrome. Erythrocyte sedimentation rate ESR averaged about 82 mm the first hour for KD and 77 for PIMS. The mean C-reactive protein CRP was 110 mg/l for KD and 144 for PIMS. Lymphopenia was highly marked in PIMS compared to KD with a percentage of 33% versus 5.6%. Cardiac enzymes were higher in PIMS patients, with 29% myocarditis and 14.2% coronary dilatation, compared with 27% coronary involvement in KD patients. All KD patients received IV-IG and acetyl salicylic acid ASA in anti-inflammatory doses, whereas PIMS patients received IV-IG infusion, corticosteroid therapy, and ASA in antiplatelet-aggregating doses. Apyrexia was achieved at D1 of treatment in the majority of patients, but 4.7% of KD had a persistent aneurysm versus 3.5% of PIMS patients.

**Conclusion:** In view of many similarities, to differentiate KD from PIMS may be a hard task. Our series is characterized -as described in the literature- by a significant frequency of abdominal pain and lymphopenia among PIMS while coronary involvement is much higher in KD.

**Patient Consent**

Not applicable (there are no patient data)

**Disclosure of Interest**

None declared

**References**

1. Xu S, Zhou C, Fang H, Zhu W, Shi J, Liu G. Laboratory parameters between multisystem inflammatory syndrome in children and Kawasaki disease. Environ Res. 2023 May 5:116070.
2. Cattalini M, and collaborators; Rheumatology Study Group of the Italian Pediatric Society. Defining Kawasaki disease and pediatric inflammatory multisystem syndrome-temporally associated to SARS-CoV-2 infection during SARS-CoV-2 epidemic in Italy: results from a national, multicenter survey. Pediatr Rheumatol Online J. 2021 Mar 16;19(1):29
3. Folga BA, Karpenko CJ, Grygiel-Górniak B. SARS-CoV-2 infection in the context of Kawasaki disease and multisystem inflammatory syndrome in children. Med Microbiol Immunol. 2023 Feb;212(1):3-12.

## P228 Differential cytokine expression in MIS-C as compared to other inflammatory conditions in Cape Town, South Africa

### C. Butters^1,2^, U. Rohlwink^3^, M. Shey^4^, J. Day^5^, C. Scott^1^, L. Zuhlke^1,6^, H. Facey-Thomas^1^, T. Spracklen^7^, H. Van Der Ross^1^, K. Webb^1^

#### ^1^Paediatrics and Child Health, University of Cape Town; ^2^Pathology, Institute of Infectious Diseases and Molecular Medicine; ^3^Neuroscience Institute, ^4^Medicine, University of Cape Town; ^5^Dr Serena Cordoso; ^6^South African Medical Research Council; ^7^Cape Heart Institute & Children's Heart Disease Research Unit, University of Cape Town, Cape Town, South Africa

##### **Correspondence:** C. Butters

*Pediatric Rheumatology 2023*, **21(Suppl 2):**P228

**Introduction:** Multisystem inflammatory syndrome in children (MIS-C) is a hyperinflammatory disease that presents in children after exposure to severe acute respiratory syndrome coronavirus 2 (SARS-CoV-2). It is unclear whether MIS-C has a distinct cytokine expression compared to similar, severe inflammatory diseases in children.

**Objectives:** To compare and contrast the expression of seven cytokines in the serum of children with MIS-C, children with similar severe diseases and healthy children.

**Methods:** From 26 June 2020 to 9 February 2022, children with suspected MIS-C were recruited at the Red Cross War Memorial Children’s Hospital in Cape Town. Those who met the World Health Organization criteria were diagnosed with MIS-C and those who had alternate diagnoses were included as inflammatory controls (e.g. sepsis, dysentery, etc.). Serum samples were taken throughout admission. In the same time period, healthy children undergoing non-inflammatory elective surgeries were recruited with consent. Serum samples were analysed using a Luminex panel of seven cytokines. C-reactive protein (CRP) was clinically performed at the local clinical laboratory.

**Results:** Twenty-three children with MIS-C, 13 inflammatory controls and 41 healthy participants were recruited. Before treatment, children with MIS-C had elevated serum concentrations of interleukin (IL)-10 (p=0.033), CRP (p=0.002) and IL-1 receptor antagonist (RA) (p=0.054) compared to children with other inflammatory conditions. Compared to healthy children, patients with MIS-C had elevated serum concentrations of IL-1RA (p<0.001), IL-1β (p=0.024), IL-10 (p<0.001), IL-6 (p<0.001), monocyte chemoattractant protein (MCP)-1 (p<0.001), tumour necrosis factor (TNF) α (p=0.002) and IL-27 (p<0.001). In children with MIS-C, the concentrations of all eight inflammatory markers rapidly decreased after treatment with intravenous immunoglobulins and/or steroids.

**Conclusion:** These data show that MIS-C is a markedly hyperinflammatory condition with a distinct profile of inflammatory marker expression as compared to clinically similar inflammatory diseases in children.

**Patient Consent**

Not applicable (there are no patient data)

**Disclosure of Interest**

None declared

## P229 SARS-COV-2-specific T-cell responses in MIS-C are distinct compared to healthy children

### N. Benede^1,2^, C. Butters^3,4^, H. Facey-Thomas^3^, T. Spracklen^5^, C. Scott^3^, L. Zuhlke^3,6^, R. Keeton^1,2^, W. Burgers^1,2,7^, K. Webb^3^

#### ^1^Medical Virology; ^2^Institute of Infectious Diseases and Molecular Medicine; ^3^Paediatrics and Child Health, University of Cape Town; ^4^Pathology, Institute of Infectious Diseases and Molecular Medicine; ^5^Cape Heart Institute & Children's Heart Disease Research Unit, University of Cape Town; ^6^South African Medical Research Council; ^7^Wellcome Centre for Infectious Diseases Research in Africa, University of Cape Town, Cape Town, South Africa

##### **Correspondence:** C. Butters

*Pediatric Rheumatology 2023*, **21(Suppl 2):**P229

**Introduction:** Multisystem inflammatory syndrome in children (MIS-C) is a severe, hyperinflammatory disease that occurs after exposure to severe acute respiratory syndrome coronavirus 2 (SARS-CoV-2). The immunological cause of MIS-C is not clear. It is not known whether SARS-CoV-2-specific T cell responses in children with MIS-C are distinct from healthy children or children with other, non-SARS-CoV-2-related inflammatory diseases.

**Objectives:** To compare the SARS-CoV-2-specific T cell responses in children with MIS-C to seropositive children with similar, severe inflammatory conditions previously exposed to SARS-CoV-2 and convalescent healthy children.

**Methods:** From 5 January 2021 to 9 February 2022, children with suspected MIS-C were recruited at the Red Cross War Memorial Children’s Hospital in Cape Town. Those who met the World Health Organization criteria were diagnosed with MIS-C and those who had alternate diagnoses were included as inflammatory controls. Healthy children were also recruited in the same period. Samples from children with MIS-C were included prior to therapy with intravenous immunoglobulins (IVIG) and/or methylprednisolone (MP). SARS-CoV-2 exposure was confirmed with serum ELISA testing for spike-specific antibodies. Whole blood samples were stimulated with SARS-CoV-2 spike, nucleocapsid and membrane peptide pools for 24 hours. T cell production of cytokines interleukin (IL)-2, tumour necrosis factor alpha (TNFα) and interferon gamma (IFNγ) was measured by intracellular cytokine staining and flow cytometry. Net responses were calculated by subtraction of background cytokine production in unstimulated blood. The proportions of T cells producing one cytokine (monofunctional T cells), two cytokines (dual functional T cells) and three cytokines (polyfunctional T cells) was measured and compared between groups.

**Results:** Eight children with MIS-C, 6 inflammatory controls and 24 healthy children were recruited, all seropositive for SARS-CoV-2. There was no difference in the frequency of SARS-CoV-2-specific CD4+ T cells between the three groups (median: 0.06% vs 0.09% vs 0.05% respectively).

The functional profile of SARS-CoV-2-specific CD4+ T cells was significantly different in children with MIS-C compared to healthy children (p<0.0001). Children with MIS-C had fewer SARS-CoV-2-specific CD4+ T cells that were polyfunctional (i.e. expressing IL-2, IFNγ and TNFα simultaneously; p=0.0147) or dual functional (p=0.0039), while having more monofunctional CD4+ T cells compared to healthy controls (p=0.0022). There was no difference in SARS-CoV-2-specific CD4+ T cell cytokine production between children with MIS-C and inflammatory controls.

Five of the eight children with MIS-C had follow up samples after treatment. Treatment with IVIG and/or MP did not affect the frequency or cytokine production profiles of SARS-CoV-2-specific CD4+ T cells.

**Conclusion:** SARS-CoV-2-specific CD4+ T cell functional responses in children with MIS-C are from exposed healthy children, but not children with other inflammatory diseases. Therefore, the cytokine profile observed from CD4+ T cells from children with MIS-C is not unique to the disease, but more generalizable across inflammatory conditions.

**Patient Consent**

Not applicable (there are no patient data)

**Disclosure of Interest**

None declared

## P230 A systematic review and meta-analysis of the impact of immunocompromise on outcomes of COVID-19 in children and young people

### C. Ciurtin^1,2^, J. Greenan-Barrett^3^, S. Aston^4^, C. Deakin^1^

#### ^1^Centre for Adolescent Rheumatology, University College London; ^2^Department of Adolescent Rheumatology; ^3^Department of Infectious Diseases, University College London Hospital; ^4^Medical School, University College London, London, United Kingdom

##### **Correspondence:** C. Ciurtin

*Pediatric Rheumatology 2023*, **21(Suppl 2):**P230

**Introduction:** Despite children and young people (CYP) having a low risk for severe Coronavirus disease 2019 (COVID-19) outcomes, there is still a degree of uncertainty related to their risk in the context of immunodeficiency or immunosuppression, primarily due to significant reporting bias in most studies, as CYP characteristically experience milder or asymptomatic COVID-19 infection and the severe outcomes tend to be overestimated.

**Objectives:** We aimed to assess comparatively the rates of admission to intensive therapy unit (ITU) and mortality due to COVID-19 infection in immunosupressed CYP compared to CYP in the general population, as well as rates for hospitalisation and need for mechanical ventilation where data were available.

**Methods:** A comprehensive systematic review to identify globally relevant studies in immunosuppressed CYP and CYP in general population (defined as younger than 25 years of age) up to 31st October 2021 (to exclude vaccinated populations), was performed. Studies were included if they reported the two primary outcomes of our study, admission to ITU and mortality, while data on other outcomes, such as hospitalisation and need for mechanical ventilation were also collected. A meta-analysis estimated the pooled proportion for each severe COVID-19 outcome, using the inverse variance method. Random effects models were used to account for interstudy heterogeneity.

**Results:** The systematic review identified 30 eligible studies for each of the two populations investigated: immunosuppressed CYP (n=793) and CYP in general population (n=102,022). In addition to the mandatory reported outcomes (ITU admission and death), 26 studies (86.7%) reported hospitalisation and invasive ventilation related outcomes in immunosuppressed CYP, while 25 studies (83.3%) reported hospitalisation and 23 studies (76.7%) reported invasive ventilation in CYP in the general population. The quality of the studies was poor-moderate (Newcastle-Otawa Scale scores 4-6/9). Our meta-analysis found higher estimated prevalence for hospitalization 46% (95% CI 37 - 56%) vs. 16% (95% CI 11 - 23%), ITU admission 12% (95% CI 9-17%) vs. 2% (95% CI 1 - 2%), mechanical ventilation 8% (95% CI 6 - 10%) vs. 1% (95% CI 0 - 1%) and increased mortality due to severe COVID-19 infection 6.5% (4.2 - 9.9%); vs. 0.2% (95% CI 0.2 - 0.4%) in immunocompromised CYP compared to CYP in general population. Funnel plots and sensitivity analyses in the immunosuppressed CYP indicated that the estimated proportion of patients admitted to ITU or requiring invasive ventilation and their mortality rate may be significantly affected by bias, while in the reports from general population only the mortality rate may have been affected by bias. Overall, this analysis shows more severe outcomes in immunocompromised CYP, despite COVID-19 infection being considered less severe in CYP.

**Conclusion:** This is the only up to date meta-analysis in immunocompromised CYP with high global relevance, which excluded reports from hospitalised cohorts alone to minimise reporting bias and included 35% studies from low- and medium-income countries to support a wider relevance of findings. Future research is required to characterise individual subgroups of immunocompromised patients, as well as impact of vaccination; however there is clear evidence for the negative consequences of immunosuppression (irrespective of its type) on COVID-19 related outcomes in CYP, which have significant clinical implications and should guide management strategies in the context of similar pandemics.

**Trial registration identifying number**

Systematic review registered on PROSPERO - CRD42021278598

**Patient Consent**

Not applicable (there are no patient data)

**Disclosure of Interest**

None declared

## P231 Multisystem inflammatory syndrome in children as a spy of inborn error of WAS

### E. Drago^1^, F. Fioredda^2^, F. Penco^3^, I. Prigione^3^, E. Massaccesi^2^, M. Lanciotti^2^, D. Moratto^4^, R. Caorsi^3^, M. Gattorno^3^, S. Volpi^1,3^

#### ^1^Department of Neuroscience, Rehabilitation, Ophthalmology, Genetics, Maternal and Child Health (DINOGMI), University of Genoa; ^2^Hematology Unit; ^3^Center for Autoinflammatory Diseases and Immunodeficiencies, IRCCS Istituto Giannina Gaslini, Genova; ^4^Flow Cytometry Unit, Clinical Chemistry Laboratory, ASST Spedali Civili di Brescia, Brescia, Italy

##### **Correspondence:** E. Drago

*Pediatric Rheumatology 2023*, **21(Suppl 2):**P231

**Introduction:** Multisystem inflammatory syndrome in children (MIS-C) is a severe, delayed complication of SARS-CoV- infection. It belongs to the post-infectious hyperinflammatory syndromes, with a clinical and immunological profile that although peculiar is reminiscent of Kawasaki disease, macrophage activation syndromes, and toxic shock syndrome. Inborn errors of immunity (IEI) could explain the susceptibility in some children to develop this condition, resulting in a potential insight into its immunological pathogenesis.

**Objectives:** To present the first case of MIS-C with an underlying Wiskott-Aldrich syndrome (WAS) gene mutation.

**Methods:** Patient’s medical records were reviewed. The expression of WAS protein (WASp) was assessed by cytofluorimetry and compared with healthy controls. A large NGS panel for inherited thrombocytopenias was performed and the mutation was subsequently confirmed by Sanger analysis. We analyzed NLRP3 inflammasome pathway activation by means of plasma cytokine measurement, cytokine secretion and ASC speck formation following in vitro stimulation of frozen PBMCs during the acute phase and at follow-up. IFN signature was performed quantifying the expression of selected IFN-stimulated genes.

**Results:** A 2-year-old boy presented with persistent fever, perioral rash, cheilitis, and edema of the extremities about 3 months after a paucisymptomatic COVID-19. In the first year of life the child presented an easy bruising; no recurrent or invasive infections. Familiarity for mild thrombocytopenia was reported The blood tests showed leukocytosis, mild thrombocytopenia, increased acute phase reactants and pro-BNP. Echocardiogram showed coronary ectasia (Z-score +2.5) and mild pericardial effusion. MIS-C has been diagnosed, with prompt response to high-dose immunoglobulins and i.v steroids. However, following the tapering of steroidal treatment, mild-to-moderate thrombocytopenia (MPV 6.5 fL) reappeared and persisted in the following months, poorly responsive to first-line therapies. Mild facial eczema appeared during steroid and eltrombopag administration. Upon further investigation, CD8+ T-cell deficiency, increased NK-cells and IL-18 levels were found. Analysis of WASp expression was found to be significantly reduced. The NGS panel confirmed the presence of a nonsense variant (c.1001dup) with X-linked recessive inheritance resulting in the formation of a premature stop codon of the WASp protein. We found that IL-1β secretion by LPS-stimulated PBMCs significantly decreased during the acute phase compared with controls, in contrast to an increase during follow-up, already described in patients with WAS. In contrast, the percentage of ASC speck in the patient's circulating monocytes was slightly increased during the acute phase compared with controls. Interestingly, the IFN-score in the acute phase was in the normal range, compared to the elevated value away from the acute event, typical of patients with WAS.

**Conclusion:** This children with WAS gene mutations, currently resulting in an X-linked thrombocytopenia phenotype, confirms that IEI may underlie a delayed altered immune response to SARS-CoV-2. The expression of WASp results in straddling between mild and severe forms, reflected in persistent CD8+ T cell deficiency. Functional tests showed mild spontaneous activation of the NLRP3 inflammasome, which was not supported by increased IL-1β secretion after stimulation, a possible sign of exhaustion following hyper-activation.

**Patient Consent**

Yes, I received consent

**Disclosure of Interest**

None declared

## P232 Description and outcomes of Afro-Caribbean children treated for multisystem inflammatory syndrome in the French West Indies

### A. Felix, C. Grabot

#### Martinique University Hospital, Fort-De-France, Martinique

##### **Correspondence:** A. Felix

*Pediatric Rheumatology 2023*, **21(Suppl 2):**P232

**Introduction:** Several studies have reported a higher frequency and greater morbidity and mortality of multisystem inflammatory syndrome in children (MIS-C) of black African descent

**Objectives:** We aimed to describe the clinical, laboratory and echocardiographic characteristics as well as outcomes of children with MIS-C requiring admission to a pediatric intensive care unit (PICU) in the French West Indies (FWI), where the majority of the population is Afro-Caribbean.

**Methods:** Ambidirectional observational cohort study between April 1, 2020 and August 31, 2022. Children (age £18 years) with MIS-C and organ failure were included. Every patient was monitored and treated following the same protocol, with repeated biological tests, echocardiography, intravenous steroids and polyvalent immunoglobulins. The primary outcomes were clinical, laboratory and echocardiography characteristics.

**Results:** Forty children (median age 7 years, range: 5-11) were included. The majority (77%) were included prospectively. Thirty-five (87%) had gastrointestinal symptoms, 30 (75%) presented initial heart failure (with persisting diastolic dysfunction at day 7) and 18 (45%) had pericarditis. Sixteen (40%) were in cardiogenic shock and required inotropic support. Median duration of inotropic support and hospitalization in PICU were respectively 4 and 5 days. The evolution curves of the inflammatory variables matched after treatment. The clinical outcomes were favorable. The Delta variant was associated with the highest incidence of MIS-C.

**Conclusion:** The outcomes of Afro-Caribbean children with MIS-C were good, without any death or cardiac sequelae, similar to Western countries. Our work does not support an ethnic susceptibility for incidence or severity of MIS-C in an Afro-Caribbean population**.**

**Patient Consent**

Yes, I received consent

**Disclosure of Interest**

None declared

## P233 A case of multisystem inflammatory syndrome in a young female with juvenile idiopathic arthritis

### L. Gago^1,2^, M. H. L. Lourenço^1,2^, M. L. Santos^1,2^, A. F. Mourão^1,2^, J. Branco^2,3^

#### ^1^Rheumatology, Hospital Egas Moniz; ^2^Comprehensive Health Research Centre (CHRC); ^3^Hospital Egas Moniz, Lisbon, Portugal

##### **Correspondence:** L. Gago

*Pediatric Rheumatology 2023*, **21(Suppl 2):**P233

**Introduction:** COVID-19 can cause a rare multisystemic post-infectious syndrome in children, called multisystem inflammatory syndrome (MIS-C). Treatment with intravenous immunoglobulin (IVIg) and high dose corticotherapy is currently recommended, and in refractory cases, it may be necessary to initiate biological therapy. Mortality in these cases is low.^(1)^

**Objectives:** We describe a case of a 12-year-old girl with polyarticular RF+ juvenile idiopathic arthritis since she was 4,currently medicated with methotrexate 5mg/week (in remission). She presented with 6days of fever(38.5°C) associated with cervical spine pain, hands, wrists and knees arthralgias, fatigue, diffuse myalgias, dry cough, thoracalgia and vomit. She had contacted a patient with COVID-19 infection two weeks before the onset of symptoms. At physical exam she had swelling of the rightankle, pain on palpation of the thighs and arms and on mobilization of the cervical spine. Cardiopulmonary auscultation was normal and there were no muscle strength deficits. The chest radiogram was normal. Abdominal ultrasound revealed mild hepatosplenomegaly. Blood tests showed elevated C-reactive protein (CRP) (24.1) mg/dL, erythrocyte sedimentation rate(ESR) of 56 mm/h and ferritin 7426 ng/mL.PCR test to COVID- 19, blood and urine cultures were negative. However, due to suspicion ofelevation of inflammatory parameters in an infectious context, therapy with IV ceftriaxone 1 g was initiated, without improvement of symptoms. Echocardiogram revealed slight mitral regurgitation, without pericardial effusion or images suggestive of vegetations. Analytically she presented troponin T(TnT) 102 ng/mL and NT-proBNP 5372 pg/mL.The electrocardiogram showed ST segment elevation in DI, DII and DIII, aVF, V2 and V3 derivations. Acute pericarditis was assumed and treatment with methylprednisolone 15 mg/kg/day IV(3 days) was initiated, yet the patient maintained fever(38°C-39°C) and an exanthematous rash appeared on the limbs and torso. Viral serologies were ordered, and the anti-SARS-CoV-2 nucleopaside antibody was positive. Taking into account the analytical changes, the previous contactwith a COVID-19patient and the exclusion of other pathogenic microorganisms, the diagnosis of MIS-C was made and therapy was started with a single IVIg 50 grams. There was a marked improvement in blood tests (CRP 7.9 mg/dL, ESR 15 mm/h, ferritin 563, TnT 17, NT-proBNP 510 mg/dL) however, due to maintenance of diffuse pain complaints therapy with IL-1 inhibitor was started, with clinical improvement.The patient is now asymptomatic.

**Conclusion:** Diagnosis of MIS-C can be challenging due to the variability of symptoms and often the PCR COVID-19 test is negative. We pretend to alert to MIS-C associated to COVID19 in children with fever, a well-defined epidemiological history and evidence of multi-organ involvement. Early treatment can be crucial to prevent shock in these patients.

**Patient Consent**

Yes, I received consent

**Disclosure of Interest**

None declared

## P234 SARS-COV-2 and leukemia in pediatric patients: casualty or causality?

### J. Garcia-Silva^1^, A. V. Villarreal-Treviño^2^, F. García-Rodríguez^2^, N. Rubio^2^

#### ^1^Facultad de Medicina; ^2^Departamento de Pediatría, Universidad Autonoma de Nuevo León, Monterrey, Mexico

##### **Correspondence:** J. Garcia-Silva

*Pediatric Rheumatology 2023*, **21(Suppl 2):**P234

**Introduction:** Many aspects of Multisystemic Inflammatory Syndrome in Children (MIS-C) remain unknown, including short-term and long-term complications. There have been several reports describing the hematological disorders that children with MIS-C can develop, but to date there is no report of the relationship between MIS-C and hematological malignancies [1].

**Objectives:** We aim to present three cases of acute lymphoblastic leukemia in pediatric patients with a previous recent history of MIS-C.

**Methods:** We retrospectively studied the cases of three patients who were diagnosed with MIS-C and acute lymphoblastic leukemia.

**Results:** The three patients were admitted with an initial diagnosis of MIS-C based on the CDC case definition of May 2020 [2]. All the patients were treated with a combination of steroids and intravenous gamma globulin. The first patient was admitted with fever, abdominal pain, nausea, vomiting, anemia, neutropenia, lymphocytosis, elevated C-reactive protein, d-dimer, ferritin, and a positive SARS-CoV-2 PCR. Shortly after receiving his treatment, he had a promptly resolution of his symptoms and was discharged. Four months later he came back with a tumor in his left eye, after a thorough work-up, a diagnosis of ALL was concluded. The second patient arrived at our unit presenting fever, cervical lymphadenopathy, abdominal pain, hepatomegaly, pericardial effusion, anemia, neutropenia, lymphocytosis, elevated C-reactive protein, d-dimer, ferritin, and positive IgG SARS-CoV-2 antibodies. When the MIS-C symptoms of this patient didn´t resolve even after receiving treatment for 10 days, further studies were made reaching a diagnosis of ALL. The third patient presented fever, cervical lymphadenopathy, abdominal pain, hepatomegaly, pericardial effusion, anemia, neutropenia, lymphocytosis, elevated C-reactive protein, fibrinogen, lactic acid dehydrogenase and positive SARS-CoV-2 PCR. A bone marrow aspiration was performed when the patient symptoms didn´t improve after 9 days of treatment, confirming an ALL diagnosis.

**Conclusion:** Whether these cases represent SARS-CoV-2-related leukemia or a product of serendipity, remains uncertain. We present these cases as a concerning possible association, but further studies are needed to reach a conclusion.

**Patient Consent**

Yes, I received consent

**Disclosure of Interest**

None declared

**References**

1. Feldstein, L.R., et al., Multisystem Inflammatory Syndrome in U.S. Children and Adolescents. N Engl J Med, 2020. 383(4): p. 334-346.
2. Centers for Disease Control and Prevention. Multisystem Inflammatory Syndrome in Children (MIS-C) Associated with Coronavirus Disease 2019 (COVID-19). Available online: https://emergency.cdc.gov/han/2020/han00432.asp

## P235 Comprehensive study of clinical and biological manifestations of MIS-C patients with expanded VΒ21.3 positive T cells

### E. German^1^, P. Samira^2^, C. Malcus^3^, E. Javouhey^4^, A. Belot^2^ on behalf of MIS-C Lyon taskforce,

#### ^1^Chu de Rouen, Rouen; ^2^HFME Service Néphrologie, Rhumatologie, Dermatologie Pediatrique; ^3^HFME HCL; ^4^HFME urgence et réanimation pédiatrique, Lyon, France

##### **Correspondence:** E. German

*Pediatric Rheumatology 2023*, **21(Suppl 2):**P235

**Introduction:** Multisystem inflammatory syndrome in children (MIS-C) is a severe complication of COVID19. The pathophysiology of MIS-C is still unknown, but the expansion of Vβ21.3 positive T cells has been identified as a specific biomarker of the disease. However, the clinical and biological features of MIS-C patients with expanded Vβ21.3 positive T cells, as well as the longitudinal assessment of this new biomarker have not been extensively studied.

**Objectives:** Comprehensive study of clinical and biological manifestations of MIS-C patients with expanded Vβ21.3 T cells

**Methods:** We conducted a retrospective monocentric study to investigate the clinical and biological evolution of patients with MIS-C and an increase of Vβ21.3+ T cells. The study was conducted in Lyon from January 2021 to February 2022.

**Results:** Thirty-six patients were included. The majority of patients were male (67%), with a median age of 9 years. Ethnicity analysis revealed an overrepresentation of patients from African ancestry (39% of the cohort). The main comorbidity observed was overweight (36%). SARS-CoV-2 was detected in all cases with half of the cases occurring during the Delta variant period.

Patients with MIS-C and expanded Vβ21.3+ T cells exhibited a high frequency of gastrointestinal symptoms (97%) and cardiac involvement (89%), skin/mucocutaneous symptoms were observed in 75%, while conjunctivitis was present in 42%. Respiratory and neurological symptoms were reported in 42% and 19% respectively.

The median duration of fever was 5 days, with a maximum time of 10 days, and it subsided within 1 day after treatment. The severity of illness was high with 97% of cases requiring management in the intensive care unit (ICU). The median duration of ICU stay was 3 days. One-third of the children required inotropic support, and the treatment strategies included intravenous immunoglobulin and intravenous steroids (97%).

Inflammatory biomarkers were significantly elevated in all patients with high level of C-reactive protein (median 164mg/L), procalcitonin (median 11.2 ng/L) and marked lymphopenia (median lymphocyte count 0,9 G/L). Ferritin levels were also markedly elevated (>500μg/L). Notably, CRP decreased rapidly within 10 days of hospitalization for all patients. Regarding Vβ21.3+ T cells, the initial assessment showed an increase in both CD4 and CD8 T cells reaching the highest expansion at day 5 from the onset of fever. However, there was a sharp decrease until day 14 in all patient except for one.

**Conclusion:** We have characterized clinical and biological evolution of patients with an increase of Vβ21.3+ T cells which serves as a specific marker for MIS-C. MIS-C patients with expanded Vβ21.3+ T cells demonstrated a higher incidence of gastrointestinal and cardiac symptoms compared to the existing literature. Moreover, MIS-C patients with Vβ21.3+ T cells had a condition of higher severity with 97% of cases requiring intensive care support. The longitudinal assessment of Vβ21.3+ T cells highlighted the transient nature of MIS-C with a normalization of this marker within 10 days.

**Patient Consent**

Yes, I received consent

**Disclosure of Interest**

None declared

## P236 TGFΒ-induced immune anergy is a hallmark of multisystem inflammatory syndrome in children

### C. C. Goetzke^1,2,3,4^, M. Massoud^1^, S. Frischbutter^5,6^, G. M. Guerra^1^, F. Heinrich^1^, A. S.-L. von Stuckrad^2,4^, L. Ehlers^7^, S. Trouillet-Assant^8,9^, B. Sahin^2^, M. Verboom^10^, M. Hallensleben^10^, A. Heuhsen^1^, N. Unterwalder^11^, M. Ferreira-Gomes^1^, K. Lehmann^1^, H. von Bernuth^2,12^, L. Peter^13^, M. Schmück-Henneresse^13^, M. Maurer^5,6^, M. Witkowski^1,14,15^, M. A. Mall^2,3^, A. Belot^16,17^, P. Durek^1^, A. Radbruch^1^, T. Kallinich^1,2,3,4^, M.-F. Mashreghi^1^

#### ^1^German Rheumatism Research Centre Berlin (DRFZ), a Leibniz Institute; ^2^Department of Pediatric Respiratory Medicine, Immunology and Critical Care Medicine , Charité - Universitätsmedizin Berlin; ^3^Berlin Institue of Health at Charité; ^4^Center for chronically sick children; ^5^Dermatological Allergology, Allergie-Centrum-Charité, Department of Dermatology and Allergy, Charité - Universitätsmedizin Berlin; ^6^Fraunhofer Institute for Translational Medicine and Pharmacology (ITMP) Allergology and Immunology, Berlin, Germany; ^7^Laboratory for Inborn Errors of Immunity, Department of Microbiology, Immunology and Transplantation, KU Leuven, Leuven, Belgium; ^8^CIRI—Centre International de Recherche en Infectiologie, Université Claude Bernard Lyon 1, Inserm, U1111, CNRS, UMR5308, ENS Lyon, Université Jean Monnet de Saint-Etienne; ^9^Joint Research Unit Civils Hospices of Lyon-bioMérieux, Hospices Civils de Lyon, Hôpital Lyon Sud, Pierre-Bénite, Lyon, France; ^10^Institute of Transfusion Medicine and Transplant Engineering, Hanover Medical School, Hannover; ^11^Department of Microbiology and Hygiene; ^12^Department of Immunology, Labor Berlin, Charité–Vivantes; ^13^Berlin Institute of Health (BIH)-Center for Regenerative Therapies (B-CRT); ^14^Institute of Microbiology, Infectious Diseases and Immunology, Charité - Universitätsmedizin Berlin; ^15^Department of Allergy Diagnostics, Labor Berlin, Charité–Vivantes, Berlin, Germany; ^16^Centre International de Recherche en Infectiologie, University of Lyon, Institut National de la Santé et de la Recherche Médicale, U1111, Université Claude Bernard; ^17^National Reference Center for Rheumatic, Autoimmune and Systemic Diseases in Children (RAISE), Pediatric Nephrology, Rheumatology, Dermatology Unit, Hôpital Femme Mère Enfant, Hospices Civils de Lyon, Lyon, France

##### **Correspondence:** C. C. Goetzke

*Pediatric Rheumatology 2023*, **21(Suppl 2):**P236

**Introduction:** In a subset of children and young adults, a usually mild infection with SARS-CoV-2 leads to severe acute hyperinflammatory shock, termed Multisystem Inflammatory Syndrome in children (MIS-C), within 6-8 weeks post infection. The inflammation is characterized by an oligoclonal T-cell expansion and upregulation of many proinflammatory cytokines. However, the underlying pathophysiology remains unclear.

**Objectives:** The aim of this study is to elucidate the underlying pathomechanisms of MIS-C.

**Methods:** Patients were recruited at the Charité Berlin. Peripheral blood mononuclear cells and serum were used for cytokine quantification, single cell transcriptomics analysis, flow cytometry and in vitro studies. Additionally, serum and PBMCs from patients and healthy donors were used for T cell reactivation tests. This study was approved by the Charité ethics committee. All patients or their legal representatives gave written and informed consent.

**Results:** A multiplex cytokine assay was used to determine the cytokine signature in the blood of these patients. Similar to previous studies, we found high levels of many type 1, type 2 and type 3 proinflammatory cytokines as well as chemokines. Analogous to patients suffering from severe COVID19 infections, children hospitalized with MIS-C also show very high serum TGFβ levels. Using single-cell sequencing, we identified activated T cells and memory B cells as well as monocytes that exhibit a TGFβ response signature. In addition, monocytes show a decreased capacity for antigen presentation. TGFβ in the patients' serum prevented a targeted T-cell response against various viral epitopes. This T-cell anergy was accompanied by the detection of latent viruses in the patients. Moreover, we detected similar mechanisms in patients hospitalized for severe COVID19.

**Conclusion:** Our data demonstrate the mechanistic role of TGFβ in inducing hyperinflammation in PIMS by inducing immune cell anergy that promotes reactivation of latent viral infections. Impaired reactivation of T cells and decreased antigen presentation on monocytes could imaffect a balanced immune response. Similar mechanisms appear to play a role in patients suffering from severe COVID19. Thus, we conclude that hyperinflammatory consequences of SARS-CoV2 infections may be triggered by spike protein-induced TGFβ upregulation.

**Patient Consent**

Yes, I received consent

**Disclosure of Interest**

None declared

## P237 MIS-C in Sweden during the pandemic: a nationwide population-based study on clinical manifestations, treatment, ICU admissions and mortality

### A. Fasth^1,2^, C. Nordenhäll^3^, K. Palmblad^4^, P. Brodin^5,6^, S. Berg^1,2^, A. Horne^5,7^ on behalf of The Swedish Pediatric MIS-C Consortium

#### ^1^Department of Pediatrics, University of Gothenburg; ^2^Dept of Rheumatology and Immunology, Queen Silvia Children’s Hospital, Gothenburg; ^3^Department of Pediatrics, Sachs’ Children’s Hospital, Stockholm; ^4^Department of Pediatric Rheumatology, Karolinska University Hospital, Stockholm; ^5^Department of Women's and Children's Health, Karolinska Institute, Stockholm, Sweden; ^6^Department of Immunology and Inflammation, Imperial College London, London, United Kingdom; ^7^Department of Pediatric Rheumatology, Karolinska University Hospital, Stockholm, Sweden

##### **Correspondence:** A. Horne

*Pediatric Rheumatology 2023*, **21(Suppl 2):**P237

**Introduction:** During the COVID-19 pandemic, Sweden chose a more open public health approach than most parts of the world. The incidence and development of Multisystem Inflammatory Syndrome in Children (MIS-C) in this setting is intriguing, and a topic of international interest. In the beginning of 2022, the MIS-C incidence rate of 6.8 per 100 000 person-years was reported from Sweden (Rhedin et. Al. Lancet). In this study we describe our national cohort of MIS-C.

**Objectives:** To evaluate the number of patients with MIS-C and to determine the need of intensive care treatment as well as to identify demographic and clinical differences between those who needed ICU care and those who did not. Another aim was to describe mortality and severe adverse events related to immunomodulatory treatment.

**Methods:** A nationwide population-based cross-sectional study**.** The study population consisted of all children and adolescents from 0 to 19 years of age with MIS-C, as reported in the Swedish National Pediatric Rheumatology Quality Register. The study period spanned from March 2020 to July 2022. The Swedish Pediatric Rheumatology Association established a national task force and implemented mandatory reporting of MIS-C cases to ensure population-based inclusion of patients into the registry. All patients included in this study met the World Health Organization criteria for MIS-C. Univariable logistic regression with ICU admission as outcome variable was used to evaluate risk factors for ICU admission as well as for chock.

**Results:** A total of 338 MIS-C cases were identified (in Sweden) during the study period. 69 patients (20%) percent required intensive care, and of those 3 were treated in ECMO. 38/338 patients (11%) presented with chock, defined as receiving inotropic support. Clinical findings, besides chock, associated with ICU admission were: overweight/ obesity, lethargy, irritability, breathing difficulties, less features of mucocutaneous involvement, diarrhea, anemia, thrombocytopenia, lower levels of albumin and higher levels of CRP. Clinical findings associated with chock were; older age (6-19 vs 0-5 years), obesity, less features of mucocutaneous involvement, diarrhea, anemia, and thrombocytopenia. 135 (40%) received triple therapy with IVIG, corticosteroids and IL-1 blockade. 16 (5%) recovered without any immunomodulatory treatment. No deaths occurred and no serious adverse events of the immunomodulatory treatment were reported.

**Conclusion:** This study provides a comprehensive overview of MIS-C cases in Sweden during the COVID-19 pandemic. The Swedish treatment strategy for MIS-C was different from most countries with a top-down approach for severly sick patients and more frequent use of short acting IL-1 iblockade. This in contrast to a step-up approach used in many countries. The rate of ICU admission in Sweden for MIS-C is relatively low. Identification of risk populations for ICU admission and for chock, might facilitate earlier identification of these children and potentially guide targeted therapies.

**Patient Consent**

Not applicable (there are no patient data)

**Disclosure of Interest**

None declared

**References**

1. Rhedin S, Lundholm C, Horne A, Smew AI, Osvald EC, Haddadi A, Alfvén T, Kahn R, Król P; Swedish Pediatric MIS-C Consortium; Brew BH, Almqvist C. Risk factors for multisystem inflammatory syndrome in children - A population-based cohort study of over 2 million children. Lancet Reg Health Eur. 2022 Aug;19:100443. doi: 10.1016/j.lanepe.2022.100443. Epub 2022 Jun 22. PMID: 35945929; PMCID: PMC9353212.

## P238 Similarities and differences between COVID-19-associated multisystem inflammatory syndrome in children (MIS-C) and Kawasaki disease shock syndrome: case reports

### S.-Y. Lee, S. Lee, J. W. Rhim, D. C. Jeong

#### Department of Pediatrics, The Catholic University of Korea, Seoul, Korea, Republic Of

##### **Correspondence:** S. Lee

*Pediatric Rheumatology 2023*, **21(Suppl 2):**P238

**Introduction:** Multisystem inflammatory syndrome in children (MIS-C) is a serious post-infectious complication of COVID-19 characterized by hyperinflammation and multi-organ dysfunction including shock. Shock is also seen in a severe form of Kawasaki disease (KD), called KD shock syndrome (KDSS). Although MIS-C and KDSS share important characterstics, few studies have compared MIS-C and KDSS.

**Objectives:** To discuss the similarities and differences between MIS-C and KDSS.

**Methods:** We report our own experiences with MIS-C and KDSS and review the literature.

**Results: [MIS-C case]** A 9-year-old boy was hospitalized with fever and abdominal pain. He had a history of SARS-CoV-2 infection 3 weeks earlier. He presented four principal features of KD, hypotension (74/40 mmHg), coagulopathy, hepatitis. Blood tests showed severe inflammation, including CPR elevation and thrombocytopenia. Echocardiography showed pericardial effusion without coronary artery abnormalities (CAAs). He was diagnosed with MIS-C and IVIG (2g/kg/dose) was administered. Additional therapy (methylprednisolone 30 mg/kg/day) was required for hyperinflammation and multi-organ dysfunction. **[KDSS case]** A 5-year-old boy was hospitalized with fever and abdominal pain. At the local clinic, he was given antibiotics to treat cervical lymphadenopathy. However, his symptoms worsened and he developed gastrointestinal problems. He presented five KD features, coagulopathy, hepatitis, and acute kidney injury. He was diagnosed with typical KD and was administered IVIG. However, he showed uncontrolled fever and shock (68/30 mmHg) and was transferred to the ICU. Inotropics, empirical antibiotics, methylprednisolone, and antithrombin III were added for treatment. After 6 days of treatment in the ICU, fever and shock were no longer observed. No pathogens were identified in microbiological studies during hospitalization. Early echocardiography did not show CAAs but outpatient echocardiography revealed a 4.1 mm coronary aneurysm. There was no development or exacerbation of CAAs and no recurrence of MIS-C or KDSS in both patients with MIS-C and KDSS.

**Conclusion:** Both MIS-C and KDSS showed hyperinflammation, KD-features, gastrointestinal problems, hypotension, and coagulopathy. However, the extent of systemic inflammation and organ dysfunction was more severe in KDSS than in MIS-C. Another important difference between MIS-C and KDSS was whether SARS-CoV-2 was identified as an infectious trigger. Because organ dysfunction is a hallmark of MIS-C and KDSS, but not KD, MIS-C shares more clinical phenotypes with KDSS than KD. A comparison of similarities and differences between MIS-C and KDSS will provide useful clues for investigating the pathophysiology and therapeutic strategies of these two diseases.

**Patient Consent**

Yes, I received consent

**Disclosure of Interest**

None declared

**References**

This study was reviewed and approved by the Institutional Review Board of Bucheon St. Mary’s Hospital of the Catholic University of Korea (approval number: HC23ZISI0022).

## P239 Severe manifestations associated with multisystem inflammatory syndrome (MIS-C)

### M. D. L. Aldana Galvan^1^, A. K. Leos Leija^1^, N. E. Rubio Pérez^1^, A. V. Villarreal Treviño^1^, F. García Rodríguez ^1^, K. R. Bruni Guerrero^2^, R. I. Perez Gonzalez^2^

#### ^1^Pediatric Rheumatology, Hospital Universitario José Eleuterio González - Universidad Autonoma de Nuevo Leon; ^2^Pediatrics, Hospital Regional ISSSTE de Monterrey - Universidad Autonoma de Nuevo Leon, Monterrey, Nuevo Leon, Mexico

##### **Correspondence:** A. K. Leos Leija

*Pediatric Rheumatology 2023*, **21(Suppl 2):**P239

**Introduction:** MIS-C is a serious and life-threatening complication of COVID-19. Since the detection of the first cases in 2020 to date, efforts have been made to characterize the clinical presentation of the disease. The involvements of the digestive and cardiac systems are the most frequently reported.^1^

**Objectives:** To make known through this case the possibility of neurological and renal damages in the spectrum of this disease, since they are not frequent or specific manifestations. This implies a diagnostic challenge for the clinician.

**Methods:** An indirect questioning and review of the clinical file was carried out to document the case report.

**Results:** This is about a 9-year-old male patient, previously healthy, with a history of vaccination against covid-19, with 1 dose that presented seizures and was taken to the emergency room with status epilepticus; during the physical examination he had persistent fever that was difficult to control, and conjunctival hyperemia and erythematous macular lesions on both legs. During his evolution, he presented hemodynamic compromise requiring aminergic support, elevated cardiac enzymes, pericarditis, acute kidney injury that required replacement therapy with PRISMA, hepatomegaly, elevated transaminase levels, ventilatory support, and pleural effusion. Hyperferritinemic syndrome is integrated with ferritin at 11918, D-dimer 5499, and elevation of acute phase reactants. Once the infectious causes were ruled out, MIS-C was considered as a diagnostic possibility, total anti-SARS-COV-2 antibodies positive for spicula-1 (992 U/mL) and nucleocapsid (8,500 COI) were documented.

Treatment started with methylprednisolone pulses for five (5) days followed by maintaining a dose at 1 mg/kg/day, as well as fractionated immunoglobulin (2 g/kg) for two (2) days after completing renal replacement therapy, improvement in inflammatory markers was obtained, increased renal function and adequate clinical response.

**Conclusion:** Complementing the approach with inflammatory markers should be considered in all febrile patients with multi-organ failure, as well as considering Pediatric Multisystem Inflammatory Syndrome (MIS-C) as one of the diagnostic possibilities, as it is an entity that, despite a reduction in COVID-19 cases, We do not have complete immunization in our pediatric patients, so they are still at risk. As more is known about its pathophysiology and the spectrum of manifestations that could be found is evidenced, we will achieve early detection, improve treatment options and reduce morbidity and mortality in these cases.

**Patient Consent**

Yes, I received consent

**Disclosure of Interest**

None declared

**References**

1. Radia T, Williams N, Agrawal P, Harman K, Weale J, Cook J, et al. Multi-system inflammatory syndrome in children & adolescents (MIS-C): A systematic review of clinical features and presentation. Paediatr Respir Rev [Internet]. 2021;38:51–7. Available from: 10.1016/j.prrv.2020.08.001

## P240 MIS-C and methylmalonic acidaemia. The role of anakinra to abate the cytokine storm

### G. Corsello^1,2^, C. Castana^2^, M. Caserta^2^, A. Di Fiore^2^, V. Siciliano^3^, M. C. Maggio^1,2^

#### ^1^University Department PROMISE “G. D’Alessandro”, University of Palermo; ^2^ARNAS, Palermo, Paediatric Clinic, Children Hospital "G. Di Cristina"; ^3^ARNAS, Palermo, Paediatric Anesthesia and Resuscitation, Children Hospital “G. Di Cristina”, Palermo, Italy

##### **Correspondence:** M. C. Maggio

*Pediatric Rheumatology 2023*, **21(Suppl 2):**P240

**Introduction:** Multisystem Inflammatory Syndrome in Children (MIS-C) is a condition characterized by fever, inflammation, multiorgan impairment that manifests late in the course of SARS-CoV-2 infection.

Methylmalonic acidemia (MMA) secondary to mutase deficiency, *mut0,* is an inborn error of metabolism causing complete enzyme deficiency. The disease is characterized by fever, recurrent ketoacidotic crises or transient vomiting, dehydration, fatigue, vomiting, dehydration, hypotonia, frequent infections, developmental delay, intellectual disability, hepatomegaly, chronic kidney disease, pancreatitis cardiomyopathy, metabolic stroke, coma and death. Despite dietary treatment, patients undergo to life-threatening metabolic imbalance. Other long-term complications include progressive renal failure, metabolic stroke, and other neurological symptoms.

**Objectives:** We describe the clinical case of a 2-year-old child with MMA secondary to mutase deficiency, *with the documented homozygous mutation* c.2179 C>T of MMUT gene, associated to mut0 phenotype. Although it was treated from the first days of life with the diet, carnitine, hydroxocobalamin, he showed a poor metabolic control, with the program to be subjected to liver transplantation.

**Methods:** One month after SARS-CoV-2 infection, he presented fever, rash, significant increase of CRP, ferritin, triglycerides, IL-6, PRO-BNP, compatible with the diagnosis of MIS-C. He was treated with intravenous immunoglobulins (2gr/Kg), methylprednisolone (2 mg/Kg/day), with rapid clinical improvement. Ten days later, he showed the worsening of clinical conditions, with the recurrence of fever, vasculitic rash with palmoplantar extension, further increase of ferritin (1033 ug/l), IL-6 (146 pg/ml), PRO-BNP (5117 pg/ml), triglycerides, anemia, thrombocytopenia, metabolic acidosis with hyperlactatemia (180 mg/dl), increased urinary methylmalonic acid (200 mmol/mCreat), multiorgan failure. He was treated with sodium bicarbonate, thiamine, coenzyme Q, vitamin C, methylprednisolone and anakinra (2 mg/Kg/day).

**Results:** Three days after the start of anakinra, he showed a significant improvement of clinical and biochemical parameters, with the resolution of fever, vasculitis, rash, and the reduction of CRP, triglycerides, PRO-BNP (50 pg/ml), IL-6 (8 pg/ml), ferritin (579 ug/l). Unfortunately, 20 days later, a sepsis from Staphylococcus Aureus and Candida Albicans required the interruption of anakinra, with the worsening of the clinical and haematological parameters and the exitus.

**Conclusion:** We describe this case, to highlight the role of anakinra in a child with a severe form of MMA and MIS-C, with the significant clinical and biochemical improvement and the resolution of MOF, secondary to with MIS-C and metabolic imbalance. At our knowledge, this is the first case of MIS-C in MMA, treated with anakinra, described in the literature.

Further studies are needed to define the appropriateness and safety of therapy with anakinra in sepsis from Candida Albicans, a lethal complication to which the child went through prolonged venous catheterization and the high risk of infections, typical of MMA.

**Patient Consent**

Yes, I received consent

**Disclosure of Interest**

None declared

**References**

1. Maggio MC, Giordano S, Failla MC, Campione MG, Alaimo A, Corsello G. Ten-month follow-up of patients with covid-19 temporally related multi-system inflammatory syndrome in children: the experience of the children hospital of Palermo. Ital J Pediatr. 2023;49(1):37.

## P241 A case report of post-COVID polymyositis in a child

### J. Marčiulynaitė^1,2^, A. Šnipaitienė^1,2^, R. Šileikienė^1,2^

#### ^1^Department of Pediatrics, Medical Academy, Lithuanian University of Health Sciences; ^2^Pediatric Clinic, Hospital of Lithuanian University of Health Sciences Kaunas Clinics, Kaunas, Lithuania

##### **Correspondence:** J. Marčiulynaitė

*Pediatric Rheumatology 2023*, **21(Suppl 2):**P241

**Introduction:** Over the past few years, several post-COVID-19 autoinflammatory conditions have been reported. One of them, polymyositis has been described in adults [1,2], but there are no cases described in children so far. We present a severe pediatric polymyositis with possible relation to previous SARS-CoV-2 infection.

**Results:** Following unspecified respiratory viral infection, 7-year-old boy complained of increasing leg muscle pain for about a month and was worsening: limping, inable to climb stairs, NSAIDs were ineffective. Signs of tonsillitis, leg and face edema, painful leg muscles on palpation and pain during passive movements were seen on admission. A patient had no fever and no rashes. Laboratory studies showed thrombocytopenia (60x10^9^/l), normal CRP, high ESR (34mm/h) and liver enzymes (ALT 2059IU/l, AST 674IU/l, GGT 220IU/l), low albumin (30.1g/l) and increased levels of creatin-kinase (CK) (1148IU/l). The patient progressed to acute renal failure, anemia (Hgb 97g/l) and polymyositis in a few days, with increasing levels of CK to 28045IU/l and myoglobin (1297.6→8987.9μg/l). Infectious agents such as EBV, CMV, atypical bacteria were disproved. From anamnesis, the boy was not vaccinated against SARS-CoV-2 and had no PCR proven COVID-19 infection during all pandemic years. However, IgG antibodies against SARS-CoV-2 were positive (140.3BAU/ml, positive >31.5BAU/ml). Immunodeficiency, hemophagocytic lymphohistiocytosis and neurological disorders were excluded. Immunological tests (muscle-specific tyrosine kinase, ANA, anti-DNR, ANCA and myositis related autoantibodies) were negative as well as genetic acylcarnitine profile analysis. Taking into account all the exams performed, previous SARS-CoV-2 infection was the most likely cause of polymyositis. Methylprednisolone 20mg/kg pulses were given for 5 days, then switched to prednisolone 2mg/kg/d. Despite of the initiation of glucocorticoids, the condition progressed to dysphagia, leading to intubation and mechanical ventilation. Head MRI and chest CT scan showed edema of neck and chest muscles. Intravenous cyclophosphamide 750mg/m^2^ once a month has been added. After 5 days with immunosupresants CK and myoglobin levels started to decrease and the patient was extubated. In the next few weeks the patient regained ability to walk, eat and talk independently. Myoglobin and CK levels normalized in one month. During the follow up period of 2 months the patient is in remission (on prednisolone tapering and cyclophosphamide) and regains normal age-related activity.

**Conclusion:** This clinical case reveals a relatively new and poorly studied post-infectious variant of severe polymyositis in a child with previous COVID-19 infection.

**Patient Consent**

Yes, I received consent

**Disclosure of Interest**

None declared

**References**

1. Anthony S, Phrathep DD, El-Husari A, Ismaili A, Healey KD, Scott R. Post-COVID-19 Polymyositis: A Case Report. Cureus Inc.; 2022
2. Veyseh M, Koyoda S, Ayesha B. COVID-19 IgG-related autoimmune inflammatory necrotizing myositis. BMJ Case Rep. 2021

## P242 COVID-19 vaccination and PIMS-TS

### I. Morawska-Michalska^1^, E. Grywalska^2^, V. Opoka-Winiarska^3^

#### ^1^Department of Clinical Immunology; ^2^Department of Experimental Immunology; ^3^Department of Pediatric Pulmonology and Rheumatology, Medical University of Lublin, Lublin, Poland

##### **Correspondence:** I. Morawska-Michalska

*Pediatric Rheumatology 2023*, **21(Suppl 2):**P242

**Introduction:** Paediatric inflammatory multisystem syndrome temporally associated with SARS-CoV-2 infection (PIMS-TS), also known as multisystem inflammatory syndrome in children (MIS-C), is diagnosed in children who experience an inadequate inflammatory response after being exposed to the SARS-CoV-2 virus. The exact cause of this abnormal response to viral antigens is not fully understood, although it is believed to be related to various immunological mechanisms and responses to superantigens leading to hyperinflammation. Out of approximately 100 children hospitalized in our facility for PIMS-TS, only one was vaccinated against COVID-19 before developing PIMS-TS, and only three were vaccinated after the onset of PIMS-TS. The reasons for not being vaccinated included the earlier unavailability of the vaccine for younger children and the concerns of parents and doctors.

**Objectives:** The questions remain ambiguous: Can patients with a history of PIMS-TS be safely vaccinated, and can the development of PIMS-TS be triggered by the COVID-19 vaccine?

**Methods:** In our study, we present cases of patients diagnosed with PIMS-TS who were vaccinated after complete recovery, as well as the case of a boy who developed PIMS-TS temporally associated with the Pfizer BioNTech COVID-19 vaccine.

**Results:** During the prolonged follow-up (one year), we did not observe any alarming post-vaccination reactions or recurrence of the underlying disease in any of the children who had previously experienced PIMS-TS. Additionally, all children exhibited a significant increase in anti-SARS-CoV-2 antibodies in the IgG class. One of the patients developed macrophage activation syndrome (MAS) as an outcome of PIMS-TS, and in this case, vaccinations were also found to be safe and effective. In other patient who developed PIMS-TS eight weeks after receiving the second dose of the vaccine, and four weeks before admission had contact with a COVID-19 case, it is difficult to establish a definitive causal relationship.

**Conclusion:** Based on the analysis of the presented cases and literature data, we conclude that the vaccination against COVID-19 in patients with a history of PIMS-TS is safe and effective in preventing PIMS-TS recurrence and severe COVID-19. It is important to monitor the health of patients after experiencing PIMS-TS and to educate parents about the possible consequences of not vaccinating a seriously ill and often immunocompromised children. Reported cases of post-vaccination PIMS-TS might be attributed to an incomplete response to vaccination, either due to not completing a full course of vaccination or the presence of immunosuppressive factors. Cases of post-vaccination PIMS-TS, in which there is no clear evidence of exposure to the virus, should be closely monitored, and the vaccination course should be discontinued. Nevertheless, the data regarding the effectiveness of a minimum two-dose vaccination schedule in children, both in terms of disease prevention and PIMS-TS, strongly support the necessity of vaccination in this age group.

**Patient Consent**

Yes, I received consent

**Disclosure of Interest**

None declared

## P244 Levels of selected laboratory and immunological parameters during one-year observation of children diagnosed with PIMS-TS - a single-center study

### V. Opoka-Winiarska^1^, E. Grywalska^2^, I. Korona-Głowniak^3^, I. Morawska-Michalska^4^, K. Gosik^2^, A. Malm^3^, J. Roliński^4^

#### ^1^Department of Pediatric Pulmonology and Rheumatology; ^2^Department of Experimental Immunology; ^3^Department of Pharmaceutical Microbiology; ^4^Department of Clinical Immunology, Medical University of Lublin, Lublin, Poland

##### **Correspondence:** V. Opoka-Winiarska

*Pediatric Rheumatology 2023*, **21(Suppl 2):**P244

**Introduction:** Paediatric inflammatory multisystem syndrome temporally associated with SARS-CoV-2 infection (PIMS-TS) is diagnosed in children presenting with high fever (over 38.5 degrees Celsius), abdominal pain, vomits, conjunctivitis, oral mucosa inflammation, and skin rash, among other symptoms, following the exposure to the SARS-CoV-2 virus. The pathogenesis of this abnormal response to viral antigens is not entirely clear, it is however postulated that many immunological co-take part in hyperinflammation. Treatment includes intravenous infusions of immunoglobulins (IVIG), acetylsalicylic acid (ASA) and, if necessary, systemic glucocorticosteroids (GCS), as well as immunomodulating drugs.

**Objectives:** We aimed to analyze selected laboratory and immunological parameters, in patients diagnosed with PIMS-TS at the Department of Lung Diseases and Pediatric Rheumatology, Medical University of Lublin and to compare those results between selected group of patients and healthy volunteers.

**Methods:** Blood smear, CRP, ESR, D-Dimer and ferritine levels were assessed in hospital laboratory. Flow cytometry was used to assess PD-1 receptor expression on CD4(+), CD8(+), and CD19(+) lymphocytes, and ELISA test was performed to determine levels of anti-SARS-CoV-2 IgG and IgM antibodies. Tests were performed on 33 patients diagnosed with PIMS-TS and 35 healthy volunteers (HV’s) with similar age and sex distribution. Tests were conducted at the time of diagnosis prior to the therapy (33 test), as well as 6 weeks (36 test), 3 months (24 tests), 6 months (12 tests), and a year (8 tests) after the diagnosis. Statistical analysis of the results was performed using Statistica 12 with Kruskal-Wallis test and Spearman rank order correlation (p<0.05).

**Results:** We have observed statistically higher level of the acute phase parameters (ESR, CRP, D-dimer, ferrtine), and their rapid normalization (<6 weeks) after the introduction of appropriate treatment. Newly diagnosed patients had higher levels of leukocytes and lower levels of lymphocytes. No statistical differences in the serological response to the SARS-CoV-2 virus between the individual groups of patients and the control group were found. Statistically higher percentages of CD4(+)/PD-1(+) lymphocytes have been observed during the acute phase of the disease. Moreover, we have found negative correlation between ESR level and percentages of CD4(+)/PD-1(+) lymphocytes at the moment of diagnosis. There was a negative correlation between leukocyte count and percentages of CD19(+)/PD-1(+) lymphocytes as well as between lymphocyte count and percentages of CD4(+)/PD-1(+) and CD19(+)/PD-1(+) cells 6 weeks after the diagnosis. We have found negative correlation between D-dimer levels and percentages of CD19(+)/PD-1(+) cells 3 months after the diagnosis. Level of anti-SARS IgM antibodies negatively correlated with percentages of CD19(+)/PD-1(+) lymphocytes

**Conclusion:** Our study supports current findings regarding high acute phase parameters in PIMS-TS and their rapid normalization after the introduction of appropriate treatment. The study provides interesting data on the overexpression of the PD-1 receptor on T CD4+ lymphocytes during the acute phase of the disease. In the one-year follow-up, we did not notice any persistent unfavorable changes in laboratory tests.

**Patient Consent**

Yes, I received consent

**Disclosure of Interest**

None declared

## P245 Evaluation of endothelial function in COVID-19 reconvalescent children with rheumatic diseases

### O. A. Oshlianska^1,2^, A. G. Artsymovych^1^, T. B. Ignatova^2^

#### ^1^Department of Pediatrics, Pediatric Infectious Diseases, Immunology and Allergology, Shupic National Medical Academy of Postgraduate Education; ^2^Pediatric Rheumatology Center, Institute of Pediatrics, Obstetrics and Gynecology of the National Academy of Medical Sciences of Ukraine, Kyiv, Ukraine

##### **Correspondence:** O. A. Oshlianska

*Pediatric Rheumatology 2023*, **21(Suppl 2):**P245

**Introduction:** Some COVID-19 clinical features and their consequences are close to rheumatic diseases. Common pathogenetic links are recognized as vasculitis, DIC-syndrome, blood hyperviscosity. The development of endothelial dysfunction, an increase in vascular stiffness could be a predictor of adverse cardiovascular events in the future.

**Objectives:** To assess Arterial stiffness and circulatory disorders in healthy children and children with rheumatic diseases with a history of COVID-19.

**Methods:** Evaluation of the reaction to the post-occlusive reactive hyperemia test (high-resolution ultrasonography was used to analyze brachial artery responses to reactive hyperemia) and nailfold capillaroscopy in 30 healthy children (group A), 32 healthy children 3 months after COVID-19 (group B), and 34 patients with rheumatic diseases (RD) in children 3 months after COVID-19 (group C: 28 - JIA, 6 - systemic connective tissue diseases). Clinical diagnoses of RD were made according to the ILAR diagnostic criteria. Patients with exacerbation of rheumatic disease, Raynaud's syndrome were not included in the study. Examined children aged 6-18 years.

**Results:** Children after COVID-19 showed an unreliable decrease in the number of capillaries on capillaroscopy, no significant differences were found among patients with various rheumatic diseases. According to the data of the occlusion test on the brachial artery among children with rheumatic diseases who contracted COVID-19, brachial artery baseline diameter in patients with RD in remission was not significantly different from healthy children (group A = 2.72±0.30 mm, group B = 2.40±0.63 mm, group C=2.58 mm). Brachial artery maximum diameter after hyperemia in patients with RD increased insufficiently after COVID-19. Flow-mediated dilation of the brachial artery after occlusion in group A = 12.75±1.02% (8.9-12.9% depending on the type of response); group B = 12.01±15.02% (- 2.04 - 35.42), group C=8.87±6.02% (-2.9-27.4%). A hyperergic type of response was registered in 61.7% of cases with RD after COVID-19 (against 23.5% in group B, p=0.04), in 1/4 - paradoxical. Flow-mediated dilation of the brachial artery induced by reactive hyperemia in hyperergic type of response in group C exceeded the similar indicator in group B (35.42±8.58% versus 27.4±3.72%). In COVID-19 reconvalescents, a lower initial velocity of blood flow in large vessels was noted (Vps in group A = 86.70±4.88 cm/s; in group B=74.96±29.71 cm/s; in group C 70.28±9, 7 cm/s, p=0.04). However, the increase in blood flow speed in group A was equal to 22.37±6.58 cm/s; in group B 14.89±2.4 cm/s, in group C 11.09±5.35 cm/s (4.1-23.1 cm/s). No significant correlations were found between the parameters of capillaroscopy and occlusion test in patients with rheumatic diseases.

**Conclusion:** The decreased percent change in the diameter of the brachial artery induced by reactive hyperemia in patients with a history of COVID-19 compared with the healthy children indicates that systemic endothelial dysfunction exists after COVID-19. In patients with rheumatic diseases, greater violations of speed and quantitative indicators of blood flow were found.

**Patient Consent**

Not applicable (there are no patient data)

**Disclosure of Interest**

None declared

## P246 Herpes Zoster virus infection in the pediatric rheumatology unit of a tertiary hospital in the POSTCOVIDA-19 era

### E. Pardo Campo^1^, P. Gonzalez del Pozo^1^, S. Burger^1^, S. Murias Loza^2^, J. Rodriguez Suárez^2^, I. Braña Abascal^1^, M. Loredo^1^, S. Alonso Castro^1^, R. Queiro Silva^1^, M. Alperi López^1^

#### ^1^Rheumatology; ^2^Pediatrics, Hospital Universitario Central De Asturias, Oviedo, Spain

##### **Correspondence:** E. Pardo Campo

*Pediatric Rheumatology 2023*, **21(Suppl 2):**P246

**Introduction:** The herpes zoster virus infection in pediatric population with rheumatic autoimmune disease is infrequent. We know that the immunosuppression of our patients due to poor disease control as well as the use of immunosuppressive treatments can predispose to infections. However, although infection by HZV is an infrequent manifestation in patients with good disease control, the frequency of this infection, its clinical characteristics and the possible influence of immune dysregulation in rheumatic patients of pediatric age during the SARSCOV2 pandemic, is still not known.

**Objectives:** It has been described and studied as a complication in adult patients. Therefore, our aim is to describe the clinical characteristics of pediatric patients who have developed herpes zoster virus infection after the onset of the SARSCOV2 pandemic.

**Methods:** An observational study of patients belonging to the Pediatric Rheumatology Unit of the Central University Hospital of Asturias who have presented with herpes zoster virus infection since the beginning of the SARSCOV2 pandemic up to the present time was carried out. We obtained data from a total of 202 patients with follow-ups in our unit. The clinical histories were reviewed and we selected those who had presented infection by HZV.

**Results:** Number of patients (3)

Mean Age (years) 11,3

Gender 1 Male 2 Female

Diagnosis JIA type psoriatic arthritis early onset, vasculitis with polyangiitis and systemic lupus erythematosus with severe complications

Elevated ESR and/or CRP 1/3

Positive ANA 2/3

Head and neck involvement 1/3

Ophthalmic involvement 0/3

Extensive involvement 1/3

Affection of the palms and soles of the feet 1/3

Enfermedad en baja actividad/remisión 2/3

Period after pandemic onset 3/3

Corticotherapy 2/3

Methotrexate 0/3

AntiTNF 1/3

Mycophenolate mofetil 2/3

Cyclophosphamide 1/3

Subsequent antiviral 3/3

Subsequent complications 0/3

**Conclusion:** The herpes zoster virus infection is present in our pediatric rheumatology departments and can manifest itself in a variety of pathologies.

More studies are needed to be able to relate the possible immune change suffered in our patients as a result of the SARSCOV2 pandemic with the incidence of infections such as VHZ. In our practice it is necessary to actively ask about previous infections and to perform a complete examination of all patients. In the near future, the need for vaccination against HZV in our pediatric patients under immunosuppressive treatment could be considered.

**Patient Consent**

Not applicable (there are no patient data)

**Disclosure of Interest**

None declared

## P247 Long term quality of life in children with previous MIS-C in Cape town, South Africa

### F. Phoya^1^, T. Spracklen^1,2,3^, C. Butters^1,4^, H. Van der Ross^1^, K. Webb^1,5^

#### ^1^Department of Paediatrics and Child Health, University of Cape Town; ^2^South African Medical Research Council; ^3^Cape Heart Institute, University of Cape Town; ^4^Division of Immunology, Institute of Infectious Disease and Molecular Medicine, Cape Town, South Africa; ^5^The Francis Crick Institute, Crick African Network, London, United Kingdom

##### **Correspondence:** F. Phoya

*Pediatric Rheumatology 2023*, **21(Suppl 2):**P247

**Introduction:** Multisystem inflammatory syndrome in children (MIS-C) is a severe, hyperinflammatory disease that occurs after exposure to severe acute respiratory syndrome coronavirus 2 (SARS-CoV-2). Over the past 2 years, the acute effects of MIS-C have been well documented but very little data has been shown on the chronic effect of MIS-C on the quality of life of patients.

**Objectives:** To document the long-term effect on the quality of life of children with previous MIS-C, including their physical, emotional, social, and school functioning.

**Methods:** A descriptive prospective study was performed. Participants previously diagnosed with MIS-C between June 2020 and March 2022 by fulfilling the World Health Organization criteria were recalled to complete a PedsQL generic inventory score and to get a physical review between September 2022 and April 2023. This was used to evaluate the effect on their Physical, Emotional, Social, and School Functioning.

**Results:** Out of the 66 recruited patients with previous MIS-C we managed to recall 36% (24) of them to complete the PedsQL generic inventory. There was a mean duration of 22 months since admission, (minimum 8 months, maximum 32 months). Sixteen percent of our cohort were male and the average age of the group was 9,6 years old (minimum 3 years old, maximum 16 years old). During their illness, the average length of admission was 9 days and 20% needed ICU admission. At follow up, all the participants had a full recovery, no medical complaints, and a normal physical examination. The results from the PedsQL showed that 16% of the children had a deficit in their physical domain score (with the lowest score of 47% deficit) and 12% had a deficit in their psychosocial functional score which included emotional, social, and educational scores (with the lowest showing a 37% deficit).

**Conclusion:** Although children with previous MIS-C had no obvious medical sequelae, there was a prolonged effect on the quality of life in this single center cohort.

**Patient Consent**

Not applicable (there are no patient data)

**Disclosure of Interest**

None declared

**References**

## P248 Gene expression assessment and inflammatory response profiling in a paediatric population with multisystem inflammatory syndrome in children (MIS-C)

### L. Rivellino^1^, M. Z. Avramovic^2^, N. Emersic^2^, P. Elefante^3^, V. Zorzi^4^, M. Nicolin^4^, A. De Bortoli^4^, B. Jenko Bizjan^2^, S. Pastore^5^, J. Kovac^2^, M. Debeljak^2,6^, A. Tommasini^3,5^, T. Avcin^2,6^, A. Taddio^3,5^

#### ^1^University of Chieti, Chieti, Italy; ^2^University Children’s Hospital, University Medical Center Ljubljana, Ljubljana, Slovenia; ^3^University of Trieste, Trieste, ^4^Experteam s.r.l, Venezia; ^5^Institute for Maternal and Child Health IRCCS “Burlo Garofolo”, Trieste, Italy; ^6^Faculty of Medicine, University of Ljubljana, Ljubljana, Slovenia

##### **Correspondence:** L. Rivellino

*Pediatric Rheumatology 2023*, **21(Suppl 2):**P248

**Introduction:** Multisystem Inflammatory Syndrome in Children (MIS-C) is a systemic hyperinflammatory response triggered by SARS-CoV-2 infection. Despite the clinical manifestations similarities with Kawasaki disease, the underlying mechanisms leading to MIS-Cinflammatory response has not been completely revealed yet.

**Objectives:** We aimed to analyze the inflammatory gene expression profile in a paediatricpopulation with MIS-C, in order to individuate a potential gene expression signature in MIS-C patients that may correlate with the clinical presentation, organ involvement and disease outcome.

**Methods:** Twenty-two patients with MIS-C clinically diagnosed and fulfilling CDC criteria were enrolled. Twelve were admitted to Institute for Maternal and Child Health IRCCS “Burlo Garofolo” in Trieste and 10 to the University Medical Center of Ljubljana, between April 2020 and March 2022. Clinical and laboratory data were collected at onset and at the last follow-up. Samples were also collected at time of admission before any treatment to undergo gene expression profiles via 3’ RNA sequencing. Controls consisting of 8 healthy people and 3 MIS-C children during their follow up were also tested.

**Results:** A small subset of genes were especially expressed in MIS-C patients if compared to healthy individuals: CD177, S100A12, HP, ZDHHC19, ANKRD22. Furthermore, matching clinical presentation and RNA expression, these genes turned out to be more frequently overexpressed in subjects with cardiac and gastrointestinal involvement. These differentially expressed genes (DEGs) are in line with previous literature data. In particular, neutrophil involvement, CD177 and the S100A12 family are mentioned in current MIS-C literature. HP gene also plays a role in neutrophil degranulation, while ZDHHC19 is responsible for the regulation of MECP2 expression and activity. The role of the ANKRD22 gene is still unclear and not yet described in the literature: it is potentially related to COVID19 and involved in JAK-STAT pathway.

**Conclusion:** Our results confirm the presence of a hyperexpression of inflammatory genes during acute phase of MIS-C. Further studies are needed to investigate the role these genes may play in the pathophysiology of the disease, potentially providing guidance in disease severity prediction and therapeutic management and their role in other inflammatory diseases.

**Patient Consent**

Yes, I received consent

**Disclosure of Interest**

None declared

## P249 Late manifestation of familial Mediterranean fever after COVID – 19 infection

### H. Sargsyan^1,2^

#### ^1^Pediatrics N2, Yerevan State Medical University; ^2^ARABKIR JMC, Yerevan, Armenia

##### **Correspondence:** H. Sargsyan

*Pediatric Rheumatology 2023*, **21(Suppl 2):**P249

**Introduction:** Coronavirus disease 2019; COVID-19 was first identified in China in December 2019 and has spread worldwide. Familial Mediterranean Fever (FMF) is autosome-recessive inherited disorder, particularly frequent around the Mediterranean basin. In general, FMF is characterized by recurrent episodes of fever, associated with serositis

**Objectives:** We present patients with late manifestation of FMF after COVID - 19 infection. Despite the high level of FMF in Armenia, there are the first cases with late manifestation of FMF after COVID-19 infection.

**Methods:** Clinical and laboratory findings are presented.

**Results:** A 43-year-old female, 30days after the COVID-19 infection suggested by the positive PCR test. She was admitted to the ICU with complains of chest pain, breathlessness, abdominal pain. The results of investigation reveals: dilatation of left ventricule, severe changes of mitral and tricuspid valves, fluid in the pericardium, EF-15%, leukocytosis with high level neutrophils and lympopenia. Diagnosis: dileted cardiomyopathy, mitral and tricuspid regurgitation, chronic heart injury, acities, anasarca, hydrothorax from the two sides, hydropericardium, acute cholecistities. Under the treatment the situation become stable and implantation of cardioverter defibrillator after 3 months was suggested for her. However, the intestinal obstruction was started and chest pain from the left side sometimes was appear. To take into account the presence of FMF in her children those are under the follow up in our FMF Center, we send her for the testing genetic mutation for reveling MEFV. The results are: MEFV – E148Q/P369S/V726A. In the past she had not fever, thoracic pain, arthritis. By the ultrasound was discovered gallstone disease, which caused the rare abdominal pain.

A 55 year-old men 60days after the COVID -19 infection suggested by the positive PCR test, was admitted to the hospital with complains of febrile fever the last two days accompanied with severe chest pain. Investigations: CRP- 307.77, ESR-58, daily rhythm in medium - 84, EchoCG - pleural effusion in the lungs and fluid in the pericardium, MR - I°, EF-45%, hydro thorax from the left side without fresh infiltrations, troponin concentration with a gradual reduction from a peak of-1241—7.36, SAA – 52.79 The diagnosis was: myocarditis, exudative pericarditis, passed COVID -19 infection. According some FMF cases in relatives we send the patient to genetic investigation. We got the following result:V726A/N mutation. In the past, he had not any clinical signs of FMF.

Colchicine treatment with the other drugs resolve the problem.

**Conclusion:** It is not under doubt that COVID-19 infection could be a risk factor for the manifestation of FMF. Patients reporting chest pain and other features suggestive of acute myocarditis and pericarditis mostly after the Covid – 19, should also be tested for FMF genetic investigation mainly in FMF endemic origin.

**Patient Consent**

Yes, I received consent

**Disclosure of Interest**

None declared

## P250 Musculoskeletal manifestations of SARS-COV-2 in a pediatric population – a Portuguese multicentric study

### A. J. S. G. Silva^1^, B. Correia^1^, H. S. Sousa^2^, J. Dinis^3^, M. Santos^4^, V. Fraga^5^, A. F. Mourão^4^, S. Sousa^5^, F. Oliveira-Ramos^1,6^, M. Bernardo^6,7^, P. Costa-Reis^6,7^, R. Campanilho-Marques^1,6^

#### ^1^Departamento de Reumatologia, Centro Hospitalar Universitário Lisboa Norte, Lisboa; ^2^Serviço de Pediatria, Hospital de Vila Franca de Xira, Vila Franca de Xira; ^3^Serviço de Reumatologia, Centro Hospitalar Lisboa Central; ^4^Departamento de Reumatologia, Centro Hospitalar Lisboa Ocidental, Lisboa; ^5^Departamento de Reumatologia, Hospital Garcia de Orta, Almada; ^6^Unidade de Reumatologia Pediátrica; ^7^Departamento de Pediatria, Centro Hospitalar Universitário Lisboa Norte, Lisboa, Portugal

##### **Correspondence:** A. J. S. G. Silva

*Pediatric Rheumatology 2023*, **21(Suppl 2):**P250

**Introduction:** SARS-CoV-2 infections can cause severe inflammation and serve as a trigger for immune-mediated manifestations. Several case reports in adults have described auto-antibodies production (45-57%) and musculoskeletal manifestations (MSKM) after the infection (2.7-5.9%), but in children the information is scarce.

**Objectives:** To describe the MSKM, after SARS-CoV-2 infection, in a multicentric pediatric population without previously known rheumatic disease.

**Methods:** The clinical records of all new patients, between April 2020 to March 2023, from five centers, with MSKM and/or serologic findings related to rheumatic diseases (RD) after SARS-CoV-2 infection, were reviewed. Based on available data in adults, 60 days were defined as the longest period of time between exposure to the virus and the onset of MSKM or serologic findings. Patients with previous known RD were excluded. Data on demographic variables and clinical features were collected and presented as frequencies and median [interquartile range] for categorical and continuous variables, respectively.

**Results:** During the study period 1063 new patients were observed, of which 36 (3.4%) had MSKM and serologic findings related to RD after SARS-CoV-2 infection (*table 1*). Considering this 36 patients (56% female), the median age at infection was 15 [12-16] years and the median time for serological or MSKM after SARS-CoV-2 infection was 23 [15-40] days. All patients had asymptomatic (54%) or mild infection, and none required hospitalization. Following infection, the main MSKM (*figure 1*) were arthralgia (23/36, 64%), fatigue (16/36, 44%), myalgia (16/36, 44%) and acrocyanosis (8/36, 22%). The most frequent diagnosis identified (*figure 2*) were nonspecific musculoskeletal pain (NMKP: 11/36, 31%), perniosis (8/36, 22%), myositis (4/36, 11%) and connective tissue diseases (CTD: 3/36, 8%). Two patients (0.2%) had positive serologic findings (one with ANCA-PR3 and one with lupus anticoagulant), but no clinical manifestations related to these markers.

Five patients (14%) were hospitalized due to the severity of the developed RD: two with systemic lupus erythematosus (6%) and three with myositis (8%).

After a median follow-up of 15 [7.5-18] months, nearly all patients presented favorable outcomes. Regarding the patients who fulfilled diagnostic criteria of an inflammatory RD: four (11.1%) patients had complete remission, eight (22.2%) had remained with minimal disease activity and one (2.7%) with active disease. From the non-inflammatory patients, 11 (30.6%) had complete remission.

**Conclusion:** To the best of our knowledge, this is one of the few studies analyzing the MSK involvement induced by SARS-CoV-2 in a pediatric population. In this cohort, the MSKM were uncommon (3.4%), and the most frequent were NMKP, perniosis, myositis and CTD. The manifestations had a wide spectrum of severity, from mild to potentially fatal, but were early identified, treated and 41.6% reached complete remission.

**Patient Consent**

Not applicable (there are no patient data)

**Disclosure of Interest**

None declared

**References**

1. Gracia-Ramos AE, Martin-Nares E, Hernández-Molina G. New Onset of Autoimmune Diseases Following COVID-19 Diagnosis. Cells. 2021 Dec 20;10(12):3592.
2. Sacchi MC, Tamiazzo S, Stobbione P, Agatea L, De Gaspari P, Stecca A, *et al*. SARS-CoV-2 infection as a trigger of autoimmune response. Clin Transl Sci. 2021 May;14(3):898-907.
3. Pascolini S, Vannini A, Deleonardi G, Ciordinik M, Sensoli A, Carletti I, *et al*. COVID-19 and Immunological Dysregulation: Can Autoantibodies be Useful? Clin Transl Sci. 2021 Mar;14(2):502-508.

## P251 Impact of COVID-19 pandemic in patients with juvenile rheumatic diseases

### E. Kansizoglou^1^, E. Mermigka^1^, M. Trachana^2^, M. Stavrakidou^2^, P. Pratsidou-Gertsi ^2^

#### ^1^School of Medicine, Aristotle University of Thessaloniki; ^2^Pediatric Immunology and Rheumatology Referral Center, 1st Department of Pediatrics, Aristotle University of Thessaloniki, Thessaloniki, Greece

##### **Correspondence:** M. Trachana

*Pediatric Rheumatology 2023*, **21(Suppl 2):**P251

**Introduction:** Global data regarding the impact of COVID-19 pandemic in patients with Juvenile Rheumatic Diseases (JRD) are still limited.

**Objectives:** To capture COVID consequences in Greek minors with JRD.

**Methods:** A cohort of patients with JRD was developed and enrolled patients for the period 6/2022-5/2023 via a preformatted questionnaire. The main domains were: a. COVID infection(s), outcome and long-term impact b. previous history of COVID vaccination c. During the quarantine period, follow-up of their JRD, access to medication/ physiotherapy, compliance, information in respect to pandemic regarding their disease and impact of quarantine on Health Related Quality of Life (HR-QoL).

**Results:** 74 patients (M:F 23:51,13±4yrs) mostly with Juvenile Idiopathic Arthritis (93.24%), completed once the questionnaire during their scheduled visits. Regarding their treatment, 68.33% were under a combination of conventional and biologic DMARDs. COVID disease was reported by 80.82%, mainly with a mild course (77.97%), absence of COVID-related admission (93.22%) or a JRD flare (87.76%). Regarding the long-term COVID impact, 5.66% registered a loss of smell and/or taste. At least 1 dose of COVID vaccination was mentioned by 39.73% of them. Post-COVID vaccination adverse events were experienced by 17.24%, mostly mild (60%) and severe ones (40%), namely a case of IgA nephropathy and one of myocarditis, respectively. At quarantine, 55.22% were periodically evaluated by a private physician, 5.97% during their pre-scheduled IV hospital drug infusion and 35.8% by a telephone/internet communication with the pediatric rheumatologist. An uninterrupted access to medication was reported by 98%. Noteworthy, omission of either IV, sc or oral drug doses was confessed by 24.50%, primarily due to a physician recommendation (69.23%). Continuation of physiotherapy, was performed either by physical presence (59.38%), or by tele-rehabilitation (25%), or by combined sessions (18.75%). Physical activity limitation was reported by 86.96%. Information regarding JRD and the pandemic was retrieved by their private pediatrician (64.9%) and websites of either Patient Associations (49.12%) or HR ones (47.37%). The unfavorable HR impact during isolation period was an excessive internet connectivity (77.55%), food-related disturbances (34.69%), impaired school activities (36.74%), anxiety (36.74%) and depression (18.37%), respectively.

**Conclusion:** Patients with JRD mainly reported an uneventful COVID-disease course, a satisfactory compliance to medication and physiotherapy, though an impaired HR- QoL.

**Patient Consent**

Not applicable (there are no patient data)

**Disclosure of Interest**

None declared

## P252 Clinical manifestations and risk of PICU admission in children with MIS-C in Northern-Taiwan:a single center experience

### C.-T. Tseng^1^, C.-Y. Wu^1^, J.-L. Huang^2^

#### ^1^Pediatrics Division of Allergy, Asthma, and Rheumatology, Chang Gung Memorial Hospital; ^2^Pediatrics Division of Allergy, Asthma, and Rheumatology, New Taipei Municipal TuCheng Hospital, New Taipei, Taiwan, Province of China

##### **Correspondence:** C.-T. Tseng

*Pediatric Rheumatology 2023*, **21(Suppl 2):**P252

**Introduction:** Multi-system Inflammatory Syndrome in Children (MIS-C) is a systemic inflammatory disorder characterized by Kawasaki disease-like features with of multiple systems involvement in children following COVID-19 viral infection. Most studies on MIS-C are from North-American and Europe, while several studies of MIS-C in Asian have been published but data from Taiwan is scarce.

**Objectives:** This study aims to show clinical manifestations and risks of PICU admission in children with MIS-C in Northern Taiwan.

**Methods:** Patients under 18 years old who met the WHO or CDC MIS-C criteria of MIS-C and were treated at Change Gung Memorial Hospital (CGMH) between Jun. 2022– Feb. 2023 were enrolled. Clinical characteristics, laboratory datas, treatment regime and outcomes were reviewed retrospectively.

**Results:** Since June 2022, we’ve identified 28 MIS-C patients including 9 boys and 19 girls in CGMH. The average age of disease onset was 5.3 ± 3.8 years old and the average length of hospital stay is 6.8 ± 2.2 days. Most MIS-C cases presented to our hospital due to fever (100%), skin rash (64.3%), tachycardia(46.4%), vomiting(46.4%) and conjunctivitis(42.9%). Nine (32%) of these cases had been admitted to the intensive care unit (ICU) due to hypotension or neurological manifestations. None of cases resulted in systolic dysfunctions, while dilatation of coronary arteries were discovered in 2 cases (7.1%) and left main coronary artery small aneurysm was found in 1 (3.5%) case. There’s one patient had arrhythmia, and recovered after the anti-arrhythmic agent use.

The lower WBC levels (p=3.2 x 10^-2^), lowest Hb level (p <0.005) and lowest platelet counts (p = 0.8 x 10^-2^) during admission were significantly lower among those who requires PICU care, while peak D-dimer (p=0.4 x 10^-2^) and ferritin (p=0.3 x 10^-2^) levels are higher among those who had been admitted to PICU. Finally, among the MIS-C patients, no mortality nor systolic dysfunctions were found.

**Conclusion:** None of the MIS-C cases in our center resulted in severe morbidity or mortality. Those with high initial procalcitonin, ferritin, D-dimer ,prothrombin time and NT-proBNP levels are associated with ICU admission.

**Patient Consent**

Yes, I received consent

**Disclosure of Interest**

None declared

**References**

1. Dong Y, Mo X, Hu Y, et al. Epidemiology of COVID-19 Among Children in China. *Pediatrics* 2020;145.
2. Verdoni L, Mazza A, Gervasoni A, et al. An outbreak of severe Kawasaki-like disease at the Italian epicentre of the SARS-CoV-2 epidemic: an observational cohort study. *Lancet* 2020;395:1771-8.
3. Feldstein LR, Rose EB, Horwitz SM, et al. Multisystem Inflammatory Syndrome in U.S. Children and Adolescents. *N Engl J Med* 2020;383:334-46.
4. Riphagen S, Gomez X, Gonzalez-Martinez C, Wilkinson N, Theocharis P. Hyperinflammatory shock in children during COVID-19 pandemic. *Lancet* 2020;395:1607-8.
5. Choe YJ, Choi EH, Choi JW, et al. Surveillance of COVID-19-Associated Multisystem Inflammatory Syndrome in Children, South Korea. *Emerg Infect Dis* 2021;27:1196-200.
6. Putri ND, Prawira Y, Tartila T, et al. Clinical Features of Multisystem Inflammatory Syndrome in Children Associated with COVID-19 in Indonesia. *J Trop Pediatr* 2022;68.

## P253 Raynaud’s phenomenon and ischemic fingers in a 6-year-old child: coagulopathy following COVID-19 or the first presentation of rheumatic disease: case report and literature review abstract

### M. Vahedi, A. Malek

#### Department of Pediatrics, Mashhad University of Medical Sciences, Mashhad, Iran, Mashhad, Iran, Islamic Republic Of

##### **Correspondence:** M. Vahedi

*Pediatric Rheumatology 2023*, **21(Suppl 2):**P253

**Introduction:** Secondary Raynaud's generally occurs in patients with autoimmune diseases,and prolonged finger ischemia will result in ischemic ulcers and tissue necrosis(1).Post-COVID-19 vasculitis and Hypercoagulability were reported(2). Digital necrosis has been described after COVID-19 in adults. Raynaud’s Phenomenon (RP) and digital ischemia following SARS-CoV-2 infection have not been reported in children(3).

**Objectives:** The aim of our study is to highlight the possible role of post-COVID-19 hypercoagulable state and vasculitis in the development ischemic finger.

**Methods:** A case report with a literature review

**Results:** A 6-year-old girl presented with cyanosis in all fingers and toes for the past 8 days. She did not have any history of fever,rashes,or arthritis, and had no prior medical issues. The pain and cyanosis in her fingers and toes were triggered by exposure to cold.she had close contact with a person who tested positive for SARS-CoV-2 infection. Initially, we noticed discoloration in her digits, followed by evidence of necrosis, particularly on the pulp of her right middle finger and left middle toe. The results were negative for ANA,P-ANCA,C-ANCA, antiphospholipid antibodies. However, we did find leukocytosis and Eosinophilia in her peripheral blood smear. The results of the serum cryoglobulins and coagulation tests were normal. The SARS-CoV-2 RT-PCR was negative. Color Doppler ultrasonography of the extremities and an echocardiogram revealed normal findings. Despite the initial treatment with calcium channel blockers and aspirin, there was evidence of progressive finger ischemia. Therefore, sildenafil, captopril, and heparin infusion were added to her therapy after 12 hours. Due to refractory digital ischemia and suspected vasculitis, we started intravenous pulse methylprednisolone and azathioprine, which successfully stopped the progressive digital ischemia. We did not need to use prostacyclins. After 14 days,she was discharged with partial healing of the ischemic changes in her fingers and toes.

**Conclusion:** Secondary Raynaud's is often associated with rheumatologic or thromboembolic disorders. We hypothesized that RP and finger ischemia may develop after a COVID-19 infection. Timely treatment of secondary RP and digital ischemia improves Patient Outcomes. In patients with suspected vasculitis, The early initiation of corticosteroids improves prognosis and may reduce intravenous prostacyclin use.

**Patient Consent**

Yes, I received consent

**Disclosure of Interest**

None declared

**References**

1. Choi E,Henkin S.Raynaud’s phenomenon and related vasospastic disorders. Vascular Medicine.2021;26(1):56-70.
2. Wong K,Shah MUFA, Khurshid M,Ullah I, Tahir MJ,Yousaf Z.COVID-19 associated vasculitis:a systematic review of case reports and case series. Annals of medicine and surgery.2022:103249.
3. Abou-IsmailMY,Diamond A,Kapoor S,ArafahY,Nayak L. The hypercoagulable state in COVID-19:Incidence,pathophysiology,and management. Thrombosis research. 2020;194:101-15.

## P254 Immunogenicity of SARS-COV-2 vaccination in children with previous SARS-COV-2 related multi-system inflammatory syndrome in children

### H. Van Der Ross^1^, C. Butters^1^, J. Day^1^, T. Spracklen^1^, T. Moyo-Gwete^2^, P. Moore^2^, L. Zühlke^1,3^, K. Webb^1,3^

#### ^1^Faculty of Health Sciences, University of Cape Town; ^2^National Institute for Communicable Diseases (NICD); ^3^South African Medical Research Council (SAMRC), Cape Town, South Africa

##### **Correspondence:** H. Van Der Ross

*Pediatric Rheumatology 2023*, **21(Suppl 2):**P254

**Introduction:** Multi-system inflammatory syndrome in children (MIS-C) is a rare, severe hyperinflammatory condition triggered by SARS-CoV-2 infection. SARS-CoV-2 vaccination in children with previous MIS-C appears safe, but the immunogenicity of vaccination in these children is not known.

**Objectives:** To investigate the production of antibodies to spike protein and their neutralisation capacity after SARS-CoV-2 vaccination in children with previous MIS-C.

**Methods:** Children older than 12, who were diagnosed with MIS-C between 2020 and 2021 and healthy children were offered 2 doses of Pfizer COMIRNATY vaccine, 3 weeks apart, as per international guidelines. Serum was collected at baseline, 1 week after each vaccine and 6 weeks after baseline. At every time point, ELISA for wild-type spike antibody production and pseudovirus neutralization was performed.

**Results:** Three out of the eleven eligible children with previous MIS-C agreed to receive vaccination (1 girl, 2 boys, all aged 12). Four healthy children volunteered for vaccination (3 boys, 1 girl, all aged 12).

At baseline, there was no difference in the median spike OD450 in healthy children compared to the MIS-C group (2.684 vs 2.976, p=0.857). The median OD450 increased in both groups from baseline to 1 week-post 1^st^ dose (p<0.001). There was no difference between the groups at 1 week-post 1^st^ vaccine (p=0.629), 1 week-post 2^nd^ vaccine (p=0.114) or after 6 weeks (p=0.057).

Similarly, the neutralising capacity of spike antibodies in both groups increased significantly from baseline to 1 week-post 1^st^ vaccination (p=0.001). There was no difference between groups at baseline (p=0.4), 1 week-post 1^st^ vaccine (p=1.0), 1 week-post 2^nd^ vaccine (p=0.857) and after 6 weeks (p=1.0).

**Conclusion:** In this small group, the immunogenic response to SARS-CoV-2 mRNA vaccination in children with previous MIS-C is comparable to healthy controls. Further research needs to be done on the cross-immunogenicity towards of other variants.

**Patient Consent**

Yes, I received consent

**Disclosure of Interest**

None declared

## P255 Multisystem inflammatory syndrome in children (MIS-C) presented with angioedema in vaccinated child: a case report

### N. Yothakol

#### Department of Pediatrics, Somdech Phra Pinklao Hospital, Bangkok, Thailand

##### **Correspondence:** N. Yothakol

*Pediatric Rheumatology 2023*, **21(Suppl 2):**P255

**Introduction:** Multi-system inflammatory syndrome in children (MIS-C) is a novel medical condition firstly reported in COVID-19 pandemic which was difficult to diagnose because of mimicking many other diseases. Various mucocutaneous manifestations in MIS-C are common, mostly Kawasaki-like features including maculopapular eruption, conjunctivitis and cheilitis but rarely seen angioedema in literature review.

**Objectives:** To describe the rare manifestation in a case of MIS-C who presented with angioedema that was successfully treated by corticosteroids and intravenous immunoglobulin (IVIG) without further complications.

**Methods:** A case report

**Results:** A previously healthy 10-year-old boy presented with high grade fever and angioedema-like face for 3 days prior to admission. He was diagnosed as angioedema at previous hospital and treated by anti-histamine and empirical antibiotics but the clinical was not improved. He has got 2 doses of COVID-19 vaccine since last 2 months and recently had history of COVID-19 infection in the past 2 weeks which was asymptomatic. He was referred to our hospital on the third day of illness with ongoing fever and facial puffiness. His temperature was 38.8 ^0^C on admission, pulse rate was 110 bpm and blood pressure was 127/66 mmHg. He had no specific source of infection on physical examination. He also had palmoplantar erythema and swollen dorsum of both hands and feet, non-purulent bilateral conjunctival injection and generalized urticarial rash. The investigation showed high white cell count, 27.5 x 10^3^ u/L (normal 4.5-11) with neutrophils 94%, lymphocytes 4.1%, eosinophils 0.1%; ESR, 21 mm/hr (normal 0-15); CRP, 170.1 mg/L (normal 0-5); LDH, 655 U/L (normal 0-250); serum ferritin, 408 ng/mL (normal 25-350); D-dimer, > 10000 ng/mL (normal <500), and interleukin-6 level, 252.2 pg/mL (normal 0-7) which were highly suggestive of hyper-inflammatory process. There was perivascular echogenic brightness of right coronary artery on echocardiography without any aneurysm and pericardial effusion and the left ventricular function was still good. Methylprednisolone (2 mg/kg/day) and IVIG were considered as combination therapy in this case. He was also given aspirin and enoxaparin for thrombotic prophylaxis. The clinical was rapidly improved and the echocardiography returned to normal in the second week after treatment. The oral corticosteroid was tapered off within 6 weeks.

**Conclusion:** MIS-C could be characterised by a variety of different mucocutaneous manifestations, which angioedema was very uncommon. The importance of early recognition with prompt treatment will reduce morbidity and mortality. The adverse effect of COVID-19 vaccine was still also suspicious. However, the vaccinated children were reported to have less complication from MIS-C than in those who were unvaccinated.

**Patient Consent**

Yes, I received consent

**Disclosure of Interest**

None declared

## P256 Efficiency And Safety Of Upadacitinib In Patients With Juvenile Idiopathic Arthritis Without Systemic Features

### E. Krekhova^1^, E. Alexeeva^1,2^, T. Dvoryakovskaya^1,2^, I. Kriulin^1,2^, T. Kriulina^1,2^, K. Isaeva^1^, A. Chomakhidze^1^, O. Lomakina^1^, A. Fetisova^1^, K. Chibisova^1^, I. Tsulukiya^1^, M. Botova^1^, N. Kondrateva^1^, M. Shingarova^1,2^, M. Kokina^1,2^

#### ^1^Rheumatology, National Medical Research Center for Children’s Health; ^2^Pediatric, Sechenov First Moscow State Medical University (Sechenov University), Moscow, Russian Federation

##### **Correspondence:** E. Krekhova

*Pediatric Rheumatology 2023*, **21(Suppl 2):**P256

**Introduction:** Upadacitinib is a selective inhibitor of JAK-kinases. Currently, clinical trials of the use of upadacitinib in different autoimmune diseases (rheumatoid arthritis, giant cell arteritis, systemic lupus erythematosus, Crohn's disease) are continuing. Upadacitinib was registered in atopic dermatitis for children 12 years and older. There aren't any publications available now about use of upadacitinib in children with rheumatic diseases. Thus, it is relevant to present the experience of using upadacitinib in pediatric patients with juvenile idiopathic arthritis without systemic features (JIA).

**Objectives:** To evaluate the efficacy and safety of upadacitinib in children with JIA.

**Methods:** The retrospective analysis included 30 patients with JIA over 12 years old and weighing ≥ 40 kg (there was one child aged 9 years) who were prescribed upadacitinib at a daily dose of 15 mg orally. The patients were observed in the rheumatology department of National Medical Research Center for Children's Health (Moscow, Russia). The efficiency of upadacitinib was assessed by the pediatric criteria of American College of Rheumatology (ACRpedi) and the index JADAS-71.

**Results:** The study included 30 patients with JIA. 13/30 (43%) boys and 17/30 (57%) girls; 7 (23%) - with oligoarticular JIA, 10 (34%) – RF- polyarticular JIA, 6 (20%) with RF+ polyarthritis, 7 (23%) with juvenile ankylosing spondylitis.

As the first antirheumatic drug, upadacitinib was prescribed to 1 patient. 29/30 (97%) patients previously received antirheumatic treatment: GCs were used in 13/30 patients (43%), methotrexate - 12/30 (40%); cyclosporine - 8/30 (27%); leflunomide 6/30 (20%), sulfasalazine – 5/30 (16%); azathioprine – 2/30 (6.7%); mycophenolate mofetil – in 2/30 (6.7%) patients. Before the appointment of upadacitinib most of patients (29/30; 97%) received biological drugs (from 1 to 5 different biologic drugs), the median duration of the use - was 4.11 years (IQR 1.94-8.93; min 0.32; max 16.45 years).

Upadacitinib was used as monotherapy in 10/30 (34%) patients, in 5/30 (17%) - with abatacept, in 4/30 (13%) – with adalimumab, in 4/30 (13%) – with methotrexate, in 3/30 (10%) – with secukinumab, in 2/30 (7%) – with golimumab. 1 patient (3%) received upadacitinib with tocilizumab, another one (3%) with cyclosporine.

After 14 days of initiation upadacitinib therapy there were a statistically significant (p<0.001) decrease of the numbers of joints with active arthritis (Me 1.5 vs 0), decrease ESR (Me 8 vs 4.5) and CRP levels (Me 0.91 vs 0.43), decrease of the physician global assessment of disease activity on VAS (Me 40 vs 16) and decrease of JADAS-71 index (Me 10.85 vs 4.4).

Among 5 patients who received upadacitinib more than 3 months, 4 patients achieved ACRpedi-30, 3 - ACRpedi-50, 3 – ACRpedi-70 and 2 - ACRpedi-90.

We observed 1 serious adverse event (1 case of toxic hepatitis) in our cohort.

**Conclusion:** The use of upadacitinib in children with JIA without systemic features is highly effective and safe. So it can be used as an alternative antirheumatic drug in cases of inefficiency of the standard treatment with non-biologic and biologic drugs.

**Patient Consent**

Not applicable (there are no patient data)

**Disclosure of Interest**

None declared

## P257 Serum hepcidin-25 as a promising biomarker for juvenile idiopathic arthritis

### S. La Bella^1^, R. Troiani^1^, M. Muselli^2^, G. Sassano^1^, G. Di Donato^1^, C. Gentile^1^, A. Di Ludovico^1^, F. Lauriola^1^, P. L. Marulli^1^, F. Chiarelli^1^, L. Breda^1^

#### ^1^Department of Pediatrics, University "G. D'Annunzio", Chieti, Chieti; ^2^Department of Life, Health and Environmental Sciences, University of L’Aquila, Italy, L'Aquila, Italy

##### **Correspondence:** S. La Bella

*Pediatric Rheumatology 2023*, **21(Suppl 2):**P257

**Introduction:** An important area of research in juvenile idiopathic arthritis (JIA) aims to identify biomarkers of disease activity that can be more sensitive and reliable than the traditional acute-phase reactants [1, 2]. The key iron-regulatory hormone hepcidin-25 (HEP) has gained attention in adults with rheumatoid arthritis (RA) owing to the involvement of the cytokine interleukin (IL)-6 which induces the HEP production [3, 4]. Anemia in JIA may be attributable to chronic inflammation, iron deficiency (ID), or both the previous. Interestingly, HEP has been advocated as a potential biomarker to assess anemia of chronic disease and iron deficiency in adult patients with rheumatoid arthritis [4].

**Objectives:** We performed a cross-sectional study on the utility of serum HEP in a cohort of 79 JIA patients with/without anemia, determining its correlations with disease activity, anemia parameters, and conventional iron status indices.

**Methods:** 79 children with non-systemic onset JIA (14 males, 65 females), were included. For each patient, disease activity was assessed by the JIA Disease Activity Score (JADAS)-27, and laboratory tests were performed to primarily evaluate HEP levels, iron status, hemoglobin, and indices of inflammation. Parameters were tested for normality with Kolmogorov-Smirnov test and the rank-sum test or t-test were used to compare data as appropriate. Spearman’s correlation was used to assess the association between quantitative variables, and a ROC analysis was conducted on HEP levels.

**Results:** Significant positive correlations for serum HEP levels were found with ferritin (r=0.5953, p<0.0001) and CPR levels (r=0.2806, p=0.0123), as well as with JADAS-27 score (r=0.8988, p<0.0001). Significant differences were found in HEP serum levels between active and inactive patients (8.6 IQR 10.0 ng/mL vs 2.9 IQR 1.9 ng/mL; p<0.0001). Mean serum HEP concentrations were significantly greater in high disease activity group than in other groups (p<0.0001).. At the ROC curve, a HEP level > 4.35 ng/ml discriminated subjects with active disease with a sensitivity of 91.8% and a specificity of 80.0% (AUC: 0.93; 95% CI: 0.88-0.98). In addition, HEP levels in anemic, iron repleted, active disease patients were significantly higher than other patients.

**Conclusion:** HEP is significantly associated with JIA disease activity and therefore it could be useful in early detection and monitoring disease exacerbations. In addition, between anemic patients, iron repleted active disease patients showed higher HEP levels compared to iron depleted inactive ones. Further studies are needed to better elucidate the role of this biomarker in both iron metabolism and inflammation in JIA.

**Trial registration identifying number:** Protocol MGB AIG N. 254 14/03/2017. Review Board of Chieti-Pescara University.

**Patient Consent**

Yes, I received consent

**Disclosure of Interest**

None declared

**References**

1. Rosina S, Natoli V, Santaniello S, Trincianti C, Consolaro A, Ravelli A. Novel biomarkers for prediction of outcome and therapeutic response in juvenile idiopathic arthritis. *Expert Rev Clin Immunol*. 2021;17(8):853-870
2. Qu H, Sundberg E, Aulin C, et al. Immunoprofiling of active and inactive systemic juvenile idiopathic arthritis reveals distinct biomarkers: a single-center study. *Pediatr Rheumatol Online J*. 2021;19(1):173
3. Chen Y, Xu W, Yang H, et al. Serum Levels of Hepcidin in Rheumatoid Arthritis and Its Correlation with Disease Activity and Anemia: A Meta-analysis. *Immunol Invest*. 2021;50(2-3):243-258
4. Khalaf W, Al-Rubaie HA, Shihab S. Studying anemia of chronic disease and iron deficiency in patients with rheumatoid arthritis by iron status and circulating hepcidin. *Hematol Rep*. 2019;11(1):7708

## P258 Lipoma arborescens: a rare condition mimicking oligoarticular Juvenile Idiopathic Arthritis (JIA) at onset

### B. Lattanzi^1^, A. Omenetti^1^, S. Cataldi^2^, V. Galeazzi^3^, M. Marinelli^4^, S. Cazzato^1^

#### ^1^Pediatric Unit, Dept of Mother and Child Health, AOU Marche, Salesi Children's Hospital; ^2^Department of Pediatrics, Department of Pediatrics, Marche Polytechnic University; ^3^Clinical Radiology, Departments of Radiologic Science; ^4^Clinical of Adult and Paediatric Orthopedic, AOU Marche, Ancona, Italy

##### **Correspondence:** A. Omenetti

*Pediatric Rheumatology 2023*, **21(Suppl 2):**P258

**Introduction:** Mild painful knee swelling with functional limitation is one of the most common clinical picture at onset of monoarticular juvenile idiopathic arthritis (JIA)

**Objectives:** To present an unusual condition featured by overlapping clinical signs typical of monoarticular JIA.

**Methods:** Diagnostic work-up including routine and immunological blood test combined to imaging assessments (i.e. knee ultrasound and magnetic resonance imaging, MRI) and synovial biopsy was carried out. Literature revision of similar case report was performed to confirm significance of the findings

**Results:** A 12 year-old female presented with left knee swelling, mild pain and functional limitation, persisting for 8 weeks without history of recent trauma nor infections. Morning stiffness was not referred. Physical examination was unremarkable except for local mild painful swelling associated to functional limitation at squatting. General condition were good except for obesity. Eighteen months earlier, a similar episode occurred in the same joint and resolved following evacuative arthrocentesis with referred serosal synovial fluid. Persistent well-being was referred until the ongoing relapse. Given the personal and family history (i.e. psoriasis and autoimmune thyroiditis), onset of JIA was suspected. Routine tests, anti-streptococcal titer antibodies and autoimmune profiling were unremarkable while eye examination ruled out signs of uveitis. Knee ultrasound reported prominent diffuse synovial thickening with mamillated aspects, associated with mild corpuscolated joint effusion, without certain signs of hypervascularization. Pigmented villonodular hyperplasia was considered, and knees MRI performed. Surprisingly, MRI reported findings consistent with lipoma arborescens, a benign intra-articular tumor featured by villous synovial hypertrophy and lipomatous infiltration of the subsynovial tissue. Synovial biopsy confirmed the diagnosis and the patient was referred to orthopedics for therapeutical synovectomy

**Conclusion:** Lipoma arborescens usually affects the knee (mostly but not exclusively in monoarticular pattern) but every joint can be involved. Although rare in children, we revised the available literature in order to assess the significance of this finding. To date only eighteen pediatric cases have been described, affecting one knee (N=11), bilateral knees (N=6), one ankle (N=1) and one knee and one elbow (N=1). Due to its rarity, delayed diagnosis (in terms of months-years) usually occurred. Interestingly, in at least 3 cases patients had been previously diagnosed with JIA and treated accordingly, mostly for years. In one case the patient also obtained diagnosis and treatment for rheumatic fever, before receiving JIA misdiagnosis. In conclusion, although rare, lipoma arborescens should be considered in differential diagnosis of oligoarticular JIA at onset, in order to avoid misdiagnosis and overtreatment.

**Patient Consent**

Yes, I received consent

**Disclosure of Interest**

None declared

## P259 Prevalence and predictors of flares in children with juvenile idiopathic arthritis under biological therapy: a retrospective multicenter investigation

### J. Zalcman, Y. Levinsky, Y. Dizitzer, G. Amarilyo

#### Pediatric Rheumatology Unit, Schneider Children's Medical Center of Israel, Petah Tikva, Israel

##### **Correspondence:** Y. Levinsky

*Pediatric Rheumatology 2023*, **21(Suppl 2):**P259

**Introduction:** Juvenile idiopathic arthritis (JIA) is the most common chronic rheumatic disease in children. In recent years, treatment with biological medications has become a leading approach, with evidence suggesting that it may lead to clinical inactive disease (CID). Several studies have shown that 20-40% of JIA patients under various biological treatments experience joint relapse. However, there is a lack of information regarding risk factors for disease flare-ups under biological treatment.

**Objectives:** To assess the rate of patients who experienced disease flares under biological treatment and identify risk factors for disease flares under these treatments compared to patients who did not experience flares.

**Methods:** A multicenter retrospective follow-up study was conducted on children aged 0-18 years from three tertiary centers in Israel, with polyartricular disease, diagnosed with one of the relevant subtypes of JIA and treated with any form of biological therapy. Demographic and clinical data were collected. Patients were divided into groups based on the presence or absence of joint flare-ups. The groups were compared to identify risk factors.

**Results:** Seventy-six children met the study's inclusion criteria. The median age of disease onset was 4.65 years (range: 2.1-9.49 years), and the median age of achieving CID was 8.5 years (range: 5.7-11.85 years). Seventy-five percent of the children experienced flare-ups under biological treatment, with a median time from CID to flare-up of 1.2 years (range: 0.68-1.89 years). Children treated with Etanercept as the first biological therapy had four times more relapse events compared to those treated with Adalimumab (OR = 3.98, 95% CI: 1.31-12.01, p-value = 0.0142). Involvement of the wrist or ankle joint at the start of biological treatment was significantly associated with flare-ups compared to patients without involvement of these joints at treatment initiation (OR = 4.76, 95% CI: 1.35-16.79, p-value < 0.0152).

**Conclusion:** JIA patients with multiple joint involvement who initiated biological treatment are at a high risk of experiencing flare-ups (75%). Risk factors for relapse include wrist or ankle involvement at presentation and initial treatment with Etanercept compared to Adalimumab.

**Patient Consent**

Not applicable (there are no patient data)

**Disclosure of Interest**

None declared

**References**

1. Zaripova LN, Midgley A, Christmas SE, Beresford MW, Baildam EM, Oldershaw RA. Juvenile idiopathic arthritis: from aetiopathogenesis to therapeutic approaches. Pediatr Rheumatol Online J. 2021 Aug 23;19(1):135.
2. Iglesias E, Torrente-Segarra V, Bou R, Ricart S, González MI, Sánchez J, Calzada J, Antón J. Non-systemic juvenile idiopathic arthritis outcome after reaching clinical remission with anti-TNF-α therapy: a clinical practice observational study of patients who discontinued treatment. Rheumatol Int. 2014 Aug;34(8):1053-7.
3. Gieling J, van den Bemt B, Hoppenreijs E, Schatorjé E. Discontinuation of biologic DMARDs in non-systemic JIA patients: a scoping review of relapse rates and associated factors. Pediatr Rheumatol Online J. 2022 Dec 5;20(1):109.
4. Castillo-Vilella M, Giménez N, Tandaipan JL, Quintana S, Modesto C. Clinical remission and subsequent relapse in patients with juvenile idiopathic arthritis: predictive factors according to therapeutic approach. Pediatr Rheumatol Online J. 2021 Aug 21;19(1):130.

## P260 Survival of antibodies during simultaneous vaccination with PCV13 and HIB-vaccine in children with juvenile idiopathic arthritis treated by biologic drugs and/or methotrexate

### O. Lomakina^1^, E. Alexeeva^1,2^, K. Isaeva^1^, T. Dvoryakovskaya^1,2^, A. Chomakhidze^1^, A. Fetisova^1^, K. Chibisova^1^, I. Kriulin^1,2^, E. Krekhova^1^, I. Tsulukiya^1^, I. Zybkova^1^, N. Tkachenko^1^, M. Botova^1^, N. Kondrateva^1^, M. Shingarova^1,2^, M. Kokina^1,2^, T. Kriulina^1,2^

#### ^1^Rheumatology, National Medical Research Center for Children’s Health; ^2^Pediatric, Sechenov First Moscow State Medical University (Sechenov University), Moscow, Russian Federation

##### **Correspondence:** O. Lomakina

*Pediatric Rheumatology 2023*, **21(Suppl 2):**P260

**Introduction:** Juvenile idiopathic arthritis (JIA) is associated with a relatively high (higher than in the population or in children without JIA) risk of developing viral and bacterial infections, including pneumonia. This is presumably due to pneumococcal and hemophilic infections.

**Objectives:** Evaluate the effectiveness and survival of post-vaccination antibodies following vaccination of PCV13 and Hib-vaccine in children with JIA.

**Methods:** The study included 430 patients with JIA. There were 262 (60,9%) girls and 168 (49%) boys. All patients received concurrently pneumococcal conjugate (PCV13) and Hib-conjugate vaccines (0.5 ml each) subcutaneously. The antibody (IgG) titers were evaluated before vaccination, after 3 weeks and 6 months. The seroconversion rate was defined as percentages of subjects with at least a 2-fold rise in antibody titers from pre- to post-vaccination. Seroprotection was established anti-pneumococcal IgG ≥ 7 U/ml and anti-Hib IgG ≥ 1.07 ug/ml.

**Results:** In our cohort 149/430 patients (34,65%) had oligoartritis, 148/430 (34,4%) - poliRF(-), 101/430 (23,5%) - sJIA, 20 (4,7 %) - ERA, 12 (2,8 %) polyRF+.

175/340 (40,7%) patients previously vaccination received monotherapy by biologic drugs; 103/340 (24%) - biologic treatment with methotrexate; 145/340 (34 %) - only methotrexate. 7/340 (1,63%) patients didn’t receive antirheumatic drugs. Biologic treatment included anti-IL-1 (canakinumab) in 30/278 (10,8%) patients, anti- IL-6 (tocilizumab) – in 70/278 (25%), anti-TNF (adalimumab - in 64/278 (23%) and etanercept – in 114/278 (41%)) agents.

3 weeks after vaccination there was recorded a significant increase of the post-vaccination IgG titers. Anti-pneumococcal IgG level at the baseline was 40 (geometric mean; standard deviation 54) U/ml; after 3 weeks - 96 (84) U/ml (p-value <0,001); after 6 month 83 (79) U/ml (p-value <0,001). Anti-Hib IgG level at the baseline was 0,95 (1,66) ug/ml; after 3 weeks - 3,3 (1,2) ug/ml (p-value <0,001); after 6 month - 3,1 (1,04) ug/ml (p-value <0,001).

Subgroup analysis showed that there was no difference between antibody survival in patients receiving biologic monotherapy or methotrexate monotherapy or combination of biologic drugs with methotrexate. In each subgroup, 6 months after vaccination, a high titer of anti-pneumococcal and anti-Hib IgG remained.

**Conclusion:** Efficacy and survival of post-vaccination antibodies was achieved in all patients with different variants of JIA received various regiment of antirheumatic treatment.

**Patient Consent**

Not applicable (there are no patient data)

**Disclosure of Interest**

None declared

## P261 Classifying psoriatic arthritis in children and young people: comparison of ILAR, CASPAR and PRINTO criteria in a nationwide UK cohort

### T. Luo^1^, V. G. Macintyre^2^, C. Ciurtin^3^ on behalf of CAPS Principal Investigators, F. McErlane^4^ on behalf of CAPS Principal Investigators, N. Geifman^5^, L. Coates^6^, K. L. Hyrich1^1,7^, S. JW Shoop-Worrall^1^ on behalf of CAPS Principal Investigators

#### ^1^The University of Manchester; ^2^University of Manchester, Manchester; ^3^UCL, London; ^4^Royal Victoria Infirmary, Newcastle upon Tyne; ^5^Faculty of Health and Medical Sciences, University of Surrey, Guildford; ^6^University of Oxford, Oxford; ^7^NIHR Manchester BRC, Manchester University NHS Foundation Trust, Manchester Academic Health Science Centre, Manchester, United Kingdom

##### **Correspondence:** T. Luo

*Pediatric Rheumatology 2023*, **21(Suppl 2):**P261

**Introduction:** Despite an increasing recognition of corresponding clinical phenotypes in children and adults with psoriatic arthritis (PsA), a life-course approach to clinical research is precluded by differences in classification criteria, treatment guidelines and research approaches in paediatric and adult populations. Inconsistent access to biologic medications between adult and paediatric populations is one important consequence. Comparing existing classification criteria for PsA may enable the identification of homogeneous patient populations to facilitate accelerated evidence-based harmonised care.

**Objectives:** To compare the classification of PsA in an inception cohort of children and young people (CYP) from across the UK using the following validated criteria: i) paediatric International League of Associations for Rheumatology (ILAR); ii) adult CASPAR criteria; iii) preliminary Pediatric Rheumatology International Trials Organization (PRINTO) classification criteria, which do not currently include juvenile PsA.

**Methods:** CYP with a confirmed diagnosis of JIA recruited 2001-2014 were selected from the Childhood Arthritis Prospective Study (CAPS), a UK JIA multicentre inception cohort. All three classification criteria were applied to clinical data collected at baseline and at one-year follow-up, with a selected number of patient records available at six months. A descriptive analysis explored the overlap between the validated ILAR and CASPAR PsA criteria and compared their ability to classify PsA with the proposed PRINTO criteria. Features distinguishing patients classified as PsA by either ILAR or CASPAR criteria were described.

**Results:** Of 1745 children and young people, 67 were classified as PsA according to either juvenile or adult validated criteria (53 by ILAR and 67 by CASPAR criteria). The reasons for which the 14 children classified by CASPAR criteria did not fulfil ILAR criteria included: HLA-B27 positivity, symptom onset after the 6th birthday and concomitant uveitis (n=2), presence of sacroiliitis (n=3), presence of enthesitis (n=5), presence of acute anterior uveitis (n=2), a first-degree relative with ankylosing spondylitis (n=2), a first-degree relative with inflammatory bowel disease (n=3), and a first-degree relative with acute anterior uveitis (n=1). When the PRINTO criteria were applied to the 58 patients classified as PsA by CASPAR or ILAR criteria, and who had data for PRINTO classification, 48 (82.8%) were classified as Other JIA, 5 (8.6%) as Enthesitis/spondylitis-related arthritis, and 5 (8.6%) as Early-onset ANA-positive JIA.

**Conclusion:** Despite being validated in adult PsA, CASPAR criteria fully capture the spectrum of child-onset PsA classified according to ILAR criteria. This is promising for future harmonised research across age in PsA. Further refinement of the PRINTO classification criteria is currently undergoing.

**Patient Consent**

Yes, I received consent

**Disclosure of Interest**

None declared

## P262 T-cell profile in newly diagnosed JIA patients

### E. Lyngfelt^1^, M. Damgaard^2,3^, K. Thörn^3^, J. Nyström^1^, J. Lingman Framme^2,4^, K. Rydenman^2^, E. Kindgren^5,6^, J. Hätting^7^, K. Biswanger^8^, A.-C. Lundell^3^, R. Pullerits^3^, M. Bemark^9^, I. Gjertsson^3^, A. Camponeschi^3^, S. Berg^1,2^, A. Fasth^1,2^, O. Ekwall^2,3^, S. Lindgren^3,10^

#### ^1^Pediatric Rheumatology and Immunology, Queen Silvia Children's hospital; ^2^Pediatrics, Institute of Clinical Sciences; ^3^Rheumatology and Inflammation Research, Sahlgrenska Academy at University of Gothenburg, Gothenburg; ^4^Pediatrics, Halland Hospital Halmstad, Halmstad; ^5^Division of Pediatrics, Department of Biomedical and Clinical Sciences, Linköping University, Linköping; ^6^Pediatrics, Skaraborgs Hospital Skövde, Skövde; ^7^Pediatrics, Skaraborg Hospital, Lidköping; ^8^Pediatrics, Södra Älvsborg Hospital, Borås; ^9^Microbiology and Immunology, Biomedicine, University of Gothenburg; ^10^Pediatrics, Institute of Clinical Sciences, The Sahlgrenska Academy at University of Gothenburg, Gothenburg, Sweden

##### **Correspondence:** E. Lyngfelt

*Pediatric Rheumatology 2023*, **21(Suppl 2):**P262

**Introduction:** Juvenile idiopathic arthritis (JIA) is a heterogenous group of chronic arthritis in childhood with varying severity and symptomatology. The etiopathology is multifactorial and not fully understood, and it is at present hard to predict the response to treatment and the risk of complications or of more severe disease. An imbalance in the adaptive immune system, triggered by genetic and environmental factors, is believed to contribute largely to the development of JIA. Previous studies indicate that the balance between regulatory T cells and effector T cells is likely disrupted.[1-5]

**Objectives:** This study is part of a bigger cohort study that will follow newly diagnosed JIA patients over time with the aim to understand JIA better immunologically and identify factors that could predict prognosis and response to treatment. The aim of this pilot study is to compare the first blood samples of newly diagnosed and immunologically untreated JIA patients with healthy controls and to investigate whether there are differences in T cell profiles.

**Methods:** Newly diagnosed JIA patient (aged 0-15) who had not received immunomodulating treatment were consecutively recruited at diagnosis to form a population based cohort with healthy gender- and age matched controls. In this pilot study 50 children were included; 25 JIA patients and 25 healthy controls. There were 15 girls and 10 boys in each group. The mean age for the JIA group was 10,8 years (median 12,6 years) while the mean age for the HC group was 10,6 years (median 12,7 years). Peripheral blood was analyzed in both groups for immunophenotyping of fresh cells with flow cytometry. The populations were compared with multivariable analysis in SIMCA and specific cell populations were compared in GraphPad with Mann-Whitney test.

**Results:** A significant increase in activated memory regulatory T cells were seen in the JIA group (p=0,037). Furthermore, Th17 cells and naïve cytotoxic T cells were significantly increased in the JIA group (p=0,023 and p=0,026, respectively).

**Conclusion:** These first data indicate that there are differences between subgroups of T cells in peripheral blood when comparing non treated newly diagnosed JIA patients with gender and age matched controls, with increased number of activated memory regulatory T cells, Th17 cells and naïve cytotoxic T cells in the JIA group. As more patients and controls will be included in the study the consistency of these results will continue to be investigated, as well as further characterization of T cell and cytokine profiles and their potential variations for example in-between JIA subgroups.

**Patient Consent**

Not applicable (there are no patient data)

**Disclosure of Interest**

None declared

**References**

1. Martini, A., et al., *Juvenile idiopathic arthritis.* Nature Reviews Disease Primers, 2022. **8**(1): p. 5.
2. Zaripova, L.N., et al., *Juvenile idiopathic arthritis: from aetiopathogenesis to therapeutic approaches.* Pediatr Rheumatol Online J, 2021. **19**(1): p. 135.
3. Maggi, L., et al., *T cell subpopulations in juvenile idiopathic arthritis and their modifications after biotherapies.* Autoimmun Rev, 2016. **15**(12): p. 1141-1144.
4. Nistala, K., et al., *Interleukin-17-producing T cells are enriched in the joints of children with arthritis, but have a reciprocal relationship to regulatory T cell numbers.* Arthritis Rheum, 2008. **58**(3): p. 875-87.
5. Glerup, M., et al., *Long-Term Outcomes in Juvenile Idiopathic Arthritis: Eighteen Years of Follow-Up in the Population-Based Nordic Juvenile Idiopathic Arthritis Cohort.* Arthritis Care Res (Hoboken), 2020. **72**(4): p. 507-516.

## P263 Temporomandibular Joint (TMJ) Magnetic Resonance Imaging (MRI) alterations are associated with signs and symptoms of Temporomandibular Disorder (TMD) in Juvenile Idiopathic Arthritis (JIA)

### G. Vallogini^1^, P. Festa^1^, L. M. Gregori^2^, S. Giancaspro^1,3^, S. Piga^4^, A. Aquilani^5^, G. Tarantino^5^, L. Chianella^1^, R. Nicolai^5^, E. Marasco^5^, F. De Benedetti^5^, V. Quinzi^3^, A. Galeotti^1^, S. Magni-Manzoni^5^

#### ^1^Dentistry Unit; ^2^Imaging, Bambino Gesù IRCCS Children's Hospital, Rome; ^3^Department of Health, Life and Environmental Science, University of L'Aquila, L'Aquila; ^4^Clinical Epidemiology Unit; ^5^Rheumatology, Bambino Gesù IRCCS Children's Hospital, Rome, Italy

##### **Correspondence:** S. Magni-Manzoni

*Pediatric Rheumatology 2023*, **21(Suppl 2):**P263

**Introduction: Appropriate clinical screening for TMD in JIA is pivotal for the correct identification of patients who deserve MRI of TMJ.**

**Objectives: To detect clinical signs and symptoms of TMD and to investigate the association between TMJ MRI parameters and TMD in JIA patients.**

**Methods: All consecutive JIA patients at the study center in 2017-2022 were invited to participate. Patients were excluded in case of other systemic diseases, congenital or acquired craniofacial changes that may alter normal growth pattern. Study patients underwent Diagnostic Criteria/ Temporomandibular Disorders (DC/TMD) assessment; TMJ MRI was performed upon rheumatologist’s and orthodontist’s agreement. MRI synovial enhancement and thickening, joint effusion, bone marrow oedema, skeletal deformities, articular disc abnormalities were assessed with grading (0, absent-4,severe). Demographic data and rheumatologic parameters were recorded. Data were analyzed with descriptive statistics; appropriate parametric statistical tests were used for normally distributed data, non-parametric tests for the others. Associations within TMD data and between MRI parameters and TMD data were investigated (significant level: p<0.05). Statistical analyses were performed using STATA, Release 17.**

**Results:** Fifty-six JIA patients (84% females), with a median age of 13 years (IQR 9-17) and a median disease duration of 7 years (IQR 3-12) were included in the study. Patients with pain on muscle palpation of both temporal and masseter muscles (46%) showed significant reduction in both right (p=0.045) and left lateral (p=0.015) movements. Significant association was found between presence of TMJ noises (34%) and reduction of mandibular protrusion (43%) (p=0.015). Out of the 75% of patients examined with TMJ MRI, the ones with right-sided mandibular condyle deformity on MRI (96%), and those with inflammation of the synovium at the right condyle (94%), showed significant limitations in left-sided lateral movements (40%) (p=0.031 and 0.038, respectively). Patients with oedema>0 of the right condyle (93%) showed a reduction in maximum mouth opening at dental examination (p=0.018). No association was found between masticatory muscle pain and TMJ noises or pain or other MRI parameters.

**Conclusion: In a cohort of JIA patients, statistically significant associations were found between TMJ noises or lateral limitations and masticatory muscles disorders and between MRI detected TMJ deformities or condyle bone oedema and reduction in mandibular movements. Our findings support the rationale for periodic DC/ TMD assessment in JIA patients for early suspicion of TMJ involvement and prompt TMJ MRI investigation in this subset of patients.**

**Patient Consent**

Yes, I received consent

**Disclosure of Interest**

None declared

**References**

1. Stoustrup P, Twilt M, Spiegel L, et al.; euroTMjoint Research Network. Clinical Orofacial Examination in Juvenile Idiopathic Arthritis: International Consensus-based Recommendations for Monitoring Patients in Clinical Practice and Research Studies. J Rheumatol. 2017;44:326-33. doi: 10.3899/jrheum.160796. PMID: 28089967.
2. Kellenberger CJ, Junhasavasdikul T, Tolend M, Doria AS. Temporomandibular joint atlas for detection and grading of juvenile idiopathic arthritis involvement by magnetic resonance imaging. Pediatr Radiol. 2018;48:411-26. doi: 10.1007/s00247-017-4000-0. PMID: 29134239; PMCID: PMC5823950
3. Tiwari S, Nambiar S, Unnikrishnan B. Chewing side preference - Impact on facial symmetry, dentition and temporomandibular joint and its correlation with handedness. J Orofac Sci 2017;9:22-7

## P264 New medication start and association with disease activity at two consecutive registry visits for patients with JIA

### M. L. Mannion, M. S. Aswani, K. R. Hearld, E. Smitherman, L. Timmerman, J. R. Curtis on behalf of for the CARRA Registry Investigators

#### University of Alabama at Birmingham, Birmingham, United States

##### **Correspondence:** M. L. Mannion

*Pediatric Rheumatology 2023*, **21(Suppl 2):**P264

**Introduction:** Escalating medication for active disease is recommended in the treatment of patients with juvenile idiopathic arthritis (JIA).

**Objectives:** Because JIA is a chronic disease where one time point may not represent a disease course, the goal of this analysis is to assess medication change by patterns of disease activity at 2 sequential registry visits.

**Methods:** Patients with JIA enrolled in the Childhood Arthritis and Rheumatology Research Alliance Registry, a North American multicenter registry, with complete clinical Juvenile Arthritis Disease Activity Scores (cJADAS) at the 6-month and 12-month registry visits were included. Disease activity at each visit was classified according to cJADAS categories (inactive, minimal, moderate, or high) for either oligo- or polyarticular disease course, regardless of JIA categorization. Patient characteristics were determined at the 12-month Registry visit. The primary outcome was new medication start at the 12-month visit determined by a clinical site attestation field. Stratifying by the respective pairings of 6- and 12-month cJADAS categories, we examined the association between paired cJADAS values, disease activity, and medication changes.

**Results:** Our sample included 3319 patients with JIA: 72% were female, 77% were white, 91% were from the US, and two-thirds had oligoarticular (36%) or rheumatoid factor (RF)- polyarticular JIA (31%). The patterns of disease activity across paired visits were: 54% persistent inactive/low, 18% persistent moderate/high, 20% improved moderate/high then inactive/low, and 8% flaring inactive/low then moderate/high. Only 6% of all patients started a new medication after the 6-month Registry visit and 5% at the 12-month visit. A higher percentage of patients with moderate/high disease activity at the 12-month visit started a new medication (9%) compared to those with inactive/minimal disease activity at the 12-month visit (3%) (*X*^2^ p<0.0001). Of those starting a new medication at the 12-month visit, 37% had persistent inactive/minimal disease activity. Notably, 91% of patients with persistent moderate/high disease activity had no medication change at the 12-month visit.

**Conclusion:** In a large multicenter registry of patients with JIA, starting a new medication was more common for those with moderate/high disease activity at the 12-month visit, but was still uncommon overall. Interestingly, starting a new medication occurred for some patients with persistent inactive/minimal disease and did not occur for most patients with persistent moderate/high disease activity. Our findings suggest that starting a new medication is not driven by longitudinal disease activity scores. Further study is needed to identify reasons for treatment non-escalation among patients with persistent moderate or high disease activity.

**Patient Consent**

Not applicable (there are no patient data)

**Disclosure of Interest**

M. Mannion Grant / Research Support with: Rheumatology Research Foundation Norman B Gaylis, MD Clinical Research Award, M. Aswani: None declared, K. Hearld: None declared, E. Smitherman: None declared, L. Timmerman: None declared, J. Curtis Grant / Research Support with: Abbvie, Amgen, ArthritisPower, Aqtual, Bendcare, BMS, CorEvitas, FASTER, GSK, IlluminationHealth, Janssen, Labcorp, Lilly, Myriad, Novartis, Pfizer, Sanofi, Scipher, Setpoint, UCB, United Rheumatology, Consultant with: Abbvie, Amgen, ArthritisPower, Aqtual, Bendcare, BMS, CorEvitas, FASTER, GSK, IlluminationHealth, Janssen, Labcorp, Lilly, Myriad, Novartis, Pfizer, Sanofi, Scipher, Setpoint, UCB, United Rheumatology

## P265 Prescription patterns and effectiveness of the second biologic agent in juvenile idiopathic arthritis

### A. Marino^1^, S. Costi^1^, M. R. Pellico^2^, S. Germinario^2^, C. Iannone ^2^, A. Amati^2^, M. Pandolfi^2^, E. Conti^2^, M. V. Gattinara^1^, M. Cornalba^1^, C. B. Chighizola^1^, G. Filocamo^3^, F. Minoia^3^, R. F. Caporali^1^

#### ^1^Pediatric Rheumatology, ASST G.Pini-CTO; ^2^University of Milan; ^3^Pediatric Rheumatology, Fondazione IRCCS Ca' Granda Ospedale Maggiore Policlinico, Milan, Italy

##### **Correspondence:** A. Marino

*Pediatric Rheumatology 2023*, **21(Suppl 2):**P265

**Introduction:** TNF-α inhibitors (TNFi) are frequently used as first-line treatment in juvenile idiopathic arthritis (JIA), while there are no clear recommendations on the choice of the second biologic agent (bDMARD).

**Objectives:** We aim to describe the prescription pattern and effectiveness of the second bDMARD in a cohort of non-systemic JIA patients.

**Methods:** This retrospective cohort study included non-systemic JIA patients treated with at least two bDMARDs followed in two Rheumatology Pediatric tertiary centers in Milan, Italy. Analyses were performed with R commander

**Results:** The study cohort included 39 patients: 21 oligoarticular, 12 polyarticular JIA, and the remainder were psoriatic JIA and ERA (3 patients for each). The median age at disease onset and at the first bDMARD was 3 [(interquartile range (IQR) 4] and 5.9 (IQR 5.3) years, respectively. The median follow-up time was 112 (IQR 103.5) months. Etanercept and adalimumab were the most frequently prescribed first-line bDMARD (16 and 15 times, respectively). The causes of the first bDMARD discontinuation were the following: articular flare (43%), uveitis flare (33%), both articular and uveitis flare (6%), and adverse events (18%).

The median age at the second bDMARD was 9.4 (IQR 7.3) years. The majority of the patients included in the cohort (80%) received a TNFi as the second bDMARD. Adalimumab was the most prescribed second bDMARD (55%), followed by infliximab (29%); the most frequent switch was from etanercept to adalimumab (13 times) (Figure 1). To note, the concomitant use of MTX decreased significantly between the two bDMARDs courses (85% vs. 64%; p = 0.04).

The rate of clinical inactive disease (CID) at 3, 6, and 12 months did not differ significantly between the first and the second bDMARD. However, the retention time of the bDMARD decreased over the second bDMARD course (median survival time 18 [95% CI 8-135] vs. 8 [95% CI 3-73] months, respectively; *p*=0.26).

A non-TNFi was prescribed as the second bDMARD in 8 patients (20%): 4 subjects received abatacept (2 for articular disease and 2 for adverse effects), and 4 subjects were treated with tocilizumab (all for articular disease).

Uveitis activity represented the main reason for discontinuation in almost half of the patients who switched to a TNFi as the second bDMARD. No significant differences in efficacy were observed during the second course of bDMARDs between patients treated with TNFi or non-TNFi. The retention rates of TNFi and non-TNFi as second-line bDMARDs were comparable(p=0.36).

Eighteen patients discontinued the second bDMARD: 16/31 (52%) from the TNFi group and 2/8 (25%) from the non-TNFi group. The reasons for discontinuation were articular flare (60%), uveitis flare (22%), both articular and uveitis flare (6%), and adverse events (12%).

**Conclusion:** Most of the patients in our cohort received a TNFi as the second bDMARD, with a significant proportion of patients being treated without MTX. No apparent differences were seen between the non-TNFi and TNFi for the achievement of CID.

**Patient Consent**

Yes, I received consent

**Disclosure of Interest**

None declared

## P266 Physical activity, exercise environment, and health-related quality of life in juvenile idiopathic arthritis: results from the actimon study

### F. Milatz^1^, T. Kallinich^2^, R. Trauzeddel^3^, D. Windschall^4^, S. Hansmann^5^, N. Baumeister^6^, J.-P. Haas^7^, M. Klaas^8^, H. Girschick^8^, J. Peitz-Kornbrust^9^, K. Minden^1,2^

#### ^1^Epidemiology and Health Services Research, German Rheumatism Research Centre; ^2^Department of Pediatric Respiratory Medicine, Immunology and Critical Care Medicine, Charité – Universitätsmedizin Berlin; ^3^Department of Pediatrics, Helios Klinik Berlin-Buch, Berlin; ^4^Clinic of Pediatric and Adolescent Rheumatology, St. Josef-Stift Sendenhorst, Sendenhorst; ^5^Department of Pediatrics, University Hospital Tuebingen, Tuebingen; ^6^Department of Sport and Health Sciences, Technical University of Munich , Munich; ^7^German Center for Pediatric and Adolescent Rheumatology, Garmisch-Partenkirchen; ^8^Vivantes Hospital im Friedrichshain, Berlin; ^9^Department of Pediatrics, Asklepios Clinic Sankt Augustin, Sankt Augustin, Germany

##### **Correspondence:** F. Milatz

*Pediatric Rheumatology 2023*, **21(Suppl 2):**P266

**Introduction:** Regular physical activity (PA) can positively impact biopsychosocial well-being, depending on stress components (e.g., frequency, amount, intensity) and situational environmental factors.

**Objectives:** Due to the lack of valid PA-related measurements in adolescents with juvenile idiopathic arthritis (JIA), PA was objectively and subjectively assessed in everyday life, taking into account exercise environment and health-related quality of life.

**Methods:** JIA patients aged 12 to 18 years were recruited at seven German paediatric rheumatology centres within the framework of the ActiMON study. PA was objectively assessed using an accelerometer (ActiGraph wGT3X-BT) worn laterally on the right hip during all waking hours on seven consecutive days. Self-reported data were based on the MoMo PA questionnaire (PAQ), containing questions on PA frequency and exercise environment. These were linked to clinical data from the National Paediatric Rheumatological Database (NPRD) and to data on health-related quality of life assessed by the PedsQL™ 4.0. In accordance with the International Children's Accelerometry Database criteria [1], only datasets for which at least 4 valid weekdays and 1 weekend day (wearing time >8 hours) could be registered were considered for evaluation.

**Results:** Data from 94 adolescents (mean age 14.8 ± 2.1 years, female 63%, mean disease duration 7.3 ± 4.5 years, oligoarthritis 42%, cJADAS-10 1.8 ± 2.2) were analyzed. The WHO-recommended minimum level of PA, averaging 60 minutes of moderate to vigorous intensity (MVPA) per day, was achieved by 31.8%. About 85% of wearing time was identified as sedentary behavior (SB), followed by PA in light (8%), vigorous (4%), and moderate (3%) intensity. While no significant sex differences were found, early adolescents spent comparatively more time in light intensity than late adolescents (p<.0001). The time spent in SB was associated with body mass index (p=0.042). In contrast to the amount and intensity, frequency of PA correlated with PedsQL sum score (r = 0.46, p<.0001). Exercise environment was overall rated as pleasant and safe (10 items, NRS 0-4: 3.1). High traffic volume correlated negatively with the amount of time spent in MVPA (r = -0.32, p=0.032). The presence of safe sidewalks (r = 0.38, p=0.005) and pleasant conditions for bicycling (r = 0.37, p=0.006) correlated with PedsQL social subscale.

**Conclusion:** Preliminary findings suggest that the majority of adolescents with JIA fail to meet the recommended amount and intensity of PA. An exercise environment perceived as pleasant and safe could help promote PA and increase patients’ well-being.

ActiMON as part of the research network TARISMA is funded by the Federal Ministry of Education and Research (01EC1902F).

**Patient Consent**

Not applicable (there are no patient data)

**Disclosure of Interest**

None declared

**References**

1. Sherar LB et al. International children's accelerometry database (ICAD): design and methods. BMC Public Health 2011;11:485.

## P267 Laringeal stridor as presenting sign of juvenile idiopathic arthritis

### G. Minca^1^, A. Meneghel^1^, G. Martini^1^, M. L. Cagnato^1^, F. Tirelli^1^, V. A. Ferraro^2^, C. Giraudo^3^, S. Carraro^2^, D. Cecchin^3^, S. Zanconato^2^, F. Zulian^1^

#### ^1^Women’s and Children’s Health Department, University Hospital of Padova, Pediatric Rheumatology Unit; ^2^Women’s and Children’s Health Department, University Hospital of Padova, Unit of Paediatric Allergy and Respiratory Medicine; ^3^Department of Medicine (DIMED), University Hospital of Padova, Complex Unit of Nuclear Medicine, Padova, Italy

##### **Correspondence:** G. Minca

*Pediatric Rheumatology 2023*, **21(Suppl 2):**P267

**Introduction:** Juvenile idiopathic arthritis (JIA) is the most common rheumatic disease in childhood. It usually presents with peripheral joint involvement but the diagnosis can be challenging due to possible misleading presenting features mimicking infections, malignancies, or other diseases.

**Objectives:** We describe a child with a very atypical JIA onset involving the laryngeal joints.

**Methods:** Case reports

**Results:** A 4-year-old boy presented with a 6-months history of hoarse voice, recurrent barking cough with mild inspiratory stridor. Pediatric pneumologist suggested a course of inhaled and oral corticosteroids with transient improvement but relapse after steroid withdrawal. Lab tests: ESR 56 mm/h,CRP 10 mg/L,negative ANCAs,positive ANA. Neck CT and MRI showed subglottic mucosal thickening with narrowing of the larynx lumen. Laryngotracheobronchoscopy revealed edema of the subglottic region; biopsy showed non specificic subepithelial lymphocytic and granulocytic infiltrate with exclusion of a neoplastic condition. Few days after stopping a short course of corticosteroids, he reported pain and swelling on his right knee. Physical examination showed right knees arthritis and tenosynovitis of the lateral compartment of the left ankle. To further investigated the complex clinical picture, a PET-MRI was performed showing a significant increased uptake of [18F] FDG at the right knee, left ankle and peri-laryngeal region, confirming right knee arthritis, left posterior tibial tenosynovitis and cricoarytenoids inflammation. Based on clinical course, laboratory and imaging results, we ended up to a diagnosis of JIA with involvement of cricoarytenoid joints, knee and ankle. A combined treatment with weekly subcutaneous methotrexate and oral prednisone led to a good control of both articular and respiratory symptoms. Due to corticosteroid dependent course, 6 months later, Adalimumab was started. One month later corticosteroid was stopped and neck-MRI showed a marked reduction of the subglottic thickening.

**Conclusion:** Cricoarytenoid involvement is a rare life-threatening manifestation of rheumatoid arthritis in adults, responsible for acute airway obstruction ^(1)^. It has been rarely described in JIA, with only few cases reported ^(2^*)*. The most common symptoms are sore throat, hoarseness, severe inspiratory stridor, dysphonia, and dyspnea ^(2)^. Differently from what already reported in the literature, in our patient the chronic and relapsing laryngeal involvement was the presenting sign of JIA. The involvement of cricoarytenoid joints could lead to respiratory distress requiring intubation and mechanical ventilation, thus an accurate diagnosis and treatment are mandatory to avoid severe complications.

**Patient Consent**

Yes, I received consent

**Disclosure of Interest**

None declared

**References**

1. Grassi W, De Angelis R, Lamanna G et al. The clinical features of rheumatoid arthritis. Eur J Radiol,1998;27:S18-24.
2. Zulian F, Sari F, de Filippis C. Otolaryngological manifestations of rheumatic disease in children. J Ped Otorhinolaryngol,2009;73: S56-60.

## P268 Determinants of parent/patient overall well-being poor ratings in juvenile idiopathic arthritis patients with inactive disease

### R. Naddei^1^, M. Burrone^2^, F. Ridella^2^, C. Trincianti^2^, C. Herrera Mora^3^, C. Malagon^4^, A. Ibañez^5^, O. Arguedas^6^, N. Ruperto^7^, A. Ravelli^8^, A. Consolaro^7^

#### ^1^UOS Reumatologia Pediatrica, AOU Federico II di Napoli, Naples; ^2^Dipartimento di Neuroscienze, Riabilitazione, Oftalmologia, Genetica e Scienze Materno Infantili (DINOGMI), Università degli Studi di Genova, Genoa, Italy; ^3^Hospital de Niños Roberto Gilbert Elizalde, Reumatología, Guayaquil, Ecuador; ^4^Hospital Universitario Simon Bolivar, Clínica Infantil Colsubsidio, Bogota, Colombia; ^5^Rheumatology, National Institute Salud del Nino, Lima, Peru; ^6^Immunology, Hospital Nacional De Ninos Dr. Carlos Saenz Herrera, San Josè, Costa Rica; ^7^UOC Reumatologia e Malattie Autoinfiammatorie; ^8^Direzione Scientifica, IRCCS Istituto Giannina Gaslini, Genoa, Italy

##### **Correspondence:** R. Naddei

*Pediatric Rheumatology 2023*, **21(Suppl 2):**P268

**Introduction:** The parent/patient global assessment of well-being (PaGA) is one of the mostly adopted parent/child-reported outcomes measure (PCROs) in the management of juvenile idiopathic arthritis (JIA). However, its use as a strict indicator of disease activity in JIA is controversial since PaGA can be affected by several factors.

**Objectives:** To identify the determinants of poor PaGA ratings in a large multinational sample of JIA patients with inactive disease according to the physician.

**Methods:** Data were extracted from a multinational dataset of JIA subjects enrolled in the Epidemiology, treatment and Outcome of Childhood Arthritis (EPOCA) study. Only patients with a physician global assessment indicating inactive disease were included. Demographic features, JIA category and PCROs (swollen or painful joint count, morning stiffness, pain visual analogue scale [VAS], functional status, disease activity VAS, quality of life, medications’ side effects [SEs]) were compared between subjects with a PaGA<1 and >1. To identify variables independently associated with a PaGA>1, a multiple logistic regression analysis was performed, entering explanatory variables showing significant results in univariate tests (p< 0.05). To further explore the relative importance of variables, we employed a dominance analysis to rank predictors in terms of their contribution to the overall variance of the outcome.

**Results:** 675 patients out of the 3,537 (19.1%) included in the analysis had a PaGA>1. At the univariate analysis, an older age at disease onset and at the visit, an ongoing treatment, morning stiffness, higher parent/patient swollen and/or painful joint count, higher pain and disease activity level, worse functional status and quality of life, and SEs were associated with PaGA ratings >1. For the multivariable analysis, complete data were available on 3,391 patients. Independent associations with a PaGA>1 were identified for age at visit >7, parent/patient swollen or tender joint count >0, pain VAS >0, disease activity VAS >0, presence of morning stiffness, impaired functional status and quality of life, and presence of at least one SE. The dominance analysis showed that the pain VAS >0, the disease activity VAS >0 and a quality-of-life score >0 were the main determinants of PaGA scores >1, accounting for the 19.9%, the 18.6% and the 18.3% of the predicted variance.

**Conclusion:** Our study confirms that many patients mark the PaGA>1 in absence of active disease according to the physician, showing that to patients not always abrogation of inflammation means remission. Pain and impairment of quality of life appear to be the main determinants of this discordance, suggesting that PaGA reflects many aspects of the disease burden, including also non-inflammatory pain, functional ability, treatment burden and psychosocial aspects.

**Patient Consent**

Not applicable (there are no patient data)

**Disclosure of Interest**

None declared

## P269 Patients with Juvenile Idiopathic Arthritis (JIA) on intensive treatment frequently present alexithymia over fatigue, anxiety, and depression, despite low disease assessment parameters

### M. S. Nicoli^1^, E. Marasco^2^, G. Tarantino^2^, S. Riccio^3^, E. Betti^1^, M. F. Paniccia^1^, A. Aquilani^2^, R. Nicolai^2^, T. C. Grimaldi^1^, F. De Benedetti^2^, S. Magni-Manzoni^2^

#### ^1^Clinical Psycology Division; ^2^Rheumatology Division, IRCCS Bambino Gesù Children's Hospital, Rome; ^3^Paediatric Department, Campania University L. Vanvitelli, Naples, Italy

##### **Correspondence:** M. S. Nicoli

*Pediatric Rheumatology 2023*, **21(Suppl 2):**P269

**Introduction:** While anxiety and/or depression have been often documented in patients with JIA, alexithymia has been seldom investigated.

**Objectives:** To determine the frequency of depressive, anxiety and alexithymic symptoms in JIA patients and to investigate their correlation with demographic and clinical parameters.

**Methods:** Consecutive JIA patients aged 8-17 years were invited to undergo psychological standardized tests: the Patient Health Questionnaire-9 items (PHQ9), the Generalised Anxiety Disorder (GAD7) test; the Self-administered Psychiatric Scales for Children and Adolescents anxiety-related (SAFA-A), depression-related (SAFA-D) and somatic areas (SAFA-S); the PedsQL™ Multidimensional Fatigue Scale PedsQL-MFS); the Toronto Alexithymia Scale (TAS- 20); the Visual Analogue Scale (VAS) for pain intensity. Demographic data, clinical and laboratory parameters were recorded. Parents were asked to complete the proxy-reported questionnaries. Data were analyzed with descriptive statistics, with appropriate parametric statistical tests for normally distributed data, non-parametric statistical tests for the others. Comparison in the frequency of the psychological symptoms in presence/absence of active, painful/tender or limited joints was assessed. Significant level was set at p<0.05. All statistical analyses were performed using R (version R 4.0.3).

**Results:** The study included 37 JIA patients (65% females), 38% persistent oligo and 60% oligo extended or RF-negative polyarthritis, with a median disease duration of 5.5 years (IQR 3.6- 7.6). Chronic uveitis occurred in 32% ever. Respectively, 97% and 78% were receiving cs-DMARDS and b-DMARDS. Approximately 87% of patients had low levels of disease activity (c-JADAS10 <1.5 in oligo, <2.5 in poly), 78% had no limited joints. PHQ9 and GAD7 were pathologic in 22 and 18%, respectively; whereas PedsQL-MFS, SAFA-D, SAFA-A, SAFA-S in 11, 15, 13, and 10% of patients. Of note, alexithymia TAS-20 showed pathological symptoms in 24% of patients. More than 30% of children complained low/bad quality of life (<80), similarly to the parents’ answers on quality of life. No psychic, demographic or clinical features showed association with disease parameters. No significant differences were found between patients with and without pain on VAS, active or painful/tender or limited joints.

**Conclusion:** Despite low disease activity and joint limitation, alexithymia over fatigue, anxiety and depression was detected in a sizeable proportion of JIA patients on intensive treatment. Our findings support the rationale for adopting routine psychological assessment and intervention in JIA patients, independently from the disease status, and for investigating the impact of alexithymia on quality of life and adherence to treatment.

**Patient Consent**

Yes, I received consent

**Disclosure of Interest**

None declared

**References**

1. Fair D.C. et al. “Depression and Anxiety in patients with Juvenile Idiopathic Arthritis: current insights and impact on Quality of Life: a Systematic Review” Rheumatol 2019 1; 11:237-252.
2. Bano S. et al. “Prevalence of Depression in Patients with Juvenile Idiopathic Arthritis Presenting at a Tertiary Care Hospital” Cureus 2020;12: e6807.
3. Cobham V.E. et al. “Systematic Review: Anxiety in Children and Adolescents with Chronic Medical Conditions” J Am Acad Child Adolesc Psychiatry 2020; 59:595-618.
4. Badarnee M. “Psychological tendencies of children with juvenile idiopathic arthritis” Scand J Psychol 2022; 63:624-633.

## P270 Is there a corelation between the JIA subtype and the juvenile arthritis damage index?

### Z. Kolkhidova, I. Nikishina

#### Pediatric, V. A. Nasonova Research Institute of Rheumatology, Moscow, Russian Federation

##### **Correspondence:** I. Nikishina

*Pediatric Rheumatology 2023*, **21(Suppl 2):**P270

**Introduction:** There is a need for a clinical tool that can encompass all types of damage that may occur in patients (pts) with juvenile idiopathic arthritis (JIA) to effectively monitor the progression of disease and assess its various outcomes over a long period of time. The Juvenile Arthritis Damage Index (JADI) is a simple tool that can assist to predict the response to JIA treatment in everyday clinical practice.

**Objectives:** To evaluate the JADI in different subtypes of JIA in pts who received and didn`t treated by biologics (B).

**Methods:** Objective was accomplished by evaluating 410 pts admitted consecutively to our clinic. The mean age was 12.5 years (ranging from 1.8 to 18 years), and the male-to-female ratio was 1:1.6.

**Results:** The articular (JADI-A) damage was observed in 18% (76) pts and extra-articular (JADI-E) in 17.5% (72) children. The mean value of the JADI-A was 1.4 (min-0; max-60) points, the JADI-E 0.5 (min-0; max-7) points.

All pts were divided into 2 groups: 350 pts who received B (1 group) and 60, who didn`t treated by B (2 group).

The average value of JADI-A in 1 group was: in 5/10 RF/ACCP-positive (RF+) pts 3,6 (0 to 33), in 3/19 Entesit-related artritis (ERA) pts 0,3 (0 to 3), in 7/26 sJIA pts 4,5 (0 to 60), in 38/215 Polyarthritis (pJIA) pts 1,3 (0 to 40), in 2/30 Oligoarthritis (oJIA) pts 0,1 (0;1). Average JADI-E score was: in 1/10 RF+ = 2, in 2/19 ERA pts 0,2 (0 to 3), in 13/26 sJIA pts 1,5 (0 to 7), in 34/215 pJIA pts 0,3 (0 to 5), in 9/30 oJIA pts 0,8 (0;4).

The presence of articular damage was detected in the RF+ variant of JIA mostly: half of the pts had JADI-A>0. Usually it was represented by symmetrical flexion contractures and "boutonniere"-type deformities of the small joints of the hands and feet, as well as subluxations or ankyloses of the wrist joints. In sJIA, both articular and extra-articular lesions were maximal. This is probably due to the more aggressive and destructive course of this arthritis subtype, glucocorticoid therapy.

The average value of JADI-A in second group was: in 3/6 RF+ pts 6,4 (0 to 24), in 0/6 ERA pts, in 8/41 pJIA pts 1,7 (0 to 21), in 0/7 oJIA pts. Average JADI-E score was: in 1/6 RF+ = 2, in 0/6 ERA pts, in 11/41 pJIA pts 0,3 (0 to 2), in 2/7 oJIA pts 0,8 (0;4).

In second group, the highest JADI-A values were found in RF+ and pJIA. At the debut of the disease, even before B was prescribed, children already had multiple joint contractures. One-third of pts with oJIA had persistent extra-articular changes (complicated flow of uveitis with the evolution of cataracts).

**Conclusion:** The JADI is an independent, useful and practical tool for the clinical measurement of cumulative assessment of joint and extra-articular damage. The RF+, pJIA and sJIA are most susceptible to damage. It is important to analyze in detail the factors associated with the index and identify on this basis a special JIA phenotype that needs very early appointment of biological therapy.

**Patient Consent**

Yes, I received consent

**Disclosure of Interest**

None declared

## P271 Experience of biologics in seropositive juvenile idiopathic arthritis: factors influencing the choice and survival of therapy

### M. Kaleda, I. Nikishina, Z. Kolkhidova, A. Arefieva

#### Pediatric, V. A. Nasonova Research Institute of Rheumatology, Moscow, Russian Federation

##### **Correspondence:** I. Nikishina

*Pediatric Rheumatology 2023*, **21(Suppl 2):**P271

**Introduction:** Seropositive juvenile idiopathic arthritis (RF+ JIA) is one of the rare and most unfavorable subtypes of JIA, characterized by an increased frequency of inefficacy of therapy.

**Objectives:** to identify factors influencing the choice of a biologics (B) in patients (pts) with RF+ JIA and the need to replace it, to evaluate the value of the JADI for predicting the response to B.

**Methods:** We analyzed age of onset, timing of diagnosis verification and initiation of B, gender, the number of active joints at the start of B, the presence ACCP+, RF, ACCP, ESR and CRP values ​​at start of B, the presence of secondary Sjögren's syndrome (SS) in all pts with RF+ JIA who received B. Since 2021, the complex of examinations included the calculation of the Juvenile Arthritis Damage Index (JADI). The JADI was compared with the ACCP, RF, CRP, ESR and the need to prescribe and switch B.

**Results:** 82 pts (89% of all pts with RF+ JIA) received B, 11% were boys. 31.7% of them started B in 1^st^ year after onset. The median age of JIA onset was 12.0 years [7.7; 14]. The median number of active joints at start of B was 15 [10; 22], ESR – 29 [18;43] mm/h, CRP – 15.0 [5.3;31] mg/l. Extra-articular manifestations at the time of prescribing B occurred in 29% of pts, 26% - SS. 29% of pts had experience with more than 1 B. Abatacept (45.1%) and various TNF-inhibitors (40.3%) were most often used as the 1^st^ B, tocilizumab and rituximab were mainly used as 2-4 line. The reason for switching from one B to another was the development of secondary inefficacy, in 4.9% of patients - serious AE. We did not find any predictive effect on the need to switch to another B of the age of onset, the age of initiation of B, gender, the number of active joints, the presence ACCP+, the presence of SS, duration of the disease (p>0.05). Pts who received more than 1 B had a trend towards higher values ​​of RF (112 IU/ml [27,5;261] and 77,9 IU/ml [28,5;187,3]), ACCP (91,6 EU/ml [5,5;295] and 53,9 EU/ml [12;188]) and statistically significant higher CRP (28 mg/l [15;51,5] and 10 mg/l [4,63;19,9], p=0.04). We analyzed the value of the JADI in 23 pts, who received B. 28.6% of them received more than 1 of B. The mean value of JADI-A was 2.39, 50% of pts had significant JADI-A scores. We found a direct correlation of the JADI index with ACCP (p=0.021), ESR (p=0.041) and CRP (p=0.024).

**Conclusion:** JIA RF+ characterize by a high need for B, and the frequency of prescribing B is associated with significant indicators of the JADI. Pts with high surrogate measures of activity (especially CRP), given the high risk of secondary inefficacy of B, tocilizumab in the 1st line of therapy may be prefer. Attention is drawn to the trend towards higher RF and ACCP values in pts treated with more than one B. A correlation was established between the JADI and ACCP, ESR and CRP, which indirectly leads to the conclusion that it is necessary to prescribe B earlier in this category of pts.

**Patient Consent**

Yes, I received consent

**Disclosure of Interest**

None declared

## P272 The degree of cumulative damage in patients with special category of juvenile idiopathic artritis, which may recognized as a juvenile rheumatoid arthritis

### Z. Kolkhidova, I. Nikishina, M. Kaleda

#### Pediatric, V. A. Nasonova Research Institute of Rheumatology, Moscow, Russian Federation

##### **Correspondence:** I. Nikishina

*Pediatric Rheumatology 2023*, **21(Suppl 2):**P272

**Introduction:** There is no special position for the term “juvenile rheumatoid arthritis (JRA)” among categories of juvenile idiopathic arthritis (JIA) according to ILAR classification, but it presents in International Classification of Diseases-10 (ICD-10), which is using in official medical documents in many countries. JRA may be recognized as seropositive or seronegative subtype of polyarticular JIA, which is characterized by symmetrical mostly erosive polyarthritis of upper and low limbs with frequent development of articular and extra-articular damage. Despite the success in the treatment of JRA, a large number of pts receiving Biologics (B) have an active form of the disease with the presence of functional disorders and significant articular and extra-articular changes.

**Objectives:** To assess cumulative damage using JADI in pts with JRA on B, to identify variables correlating with the damage index.

**Methods:** A retrospective analysis of 50 pts with JRA subtype receiving B, consecutively admitted to our clinic. In addition to routine clinical and laboratory parameters Juvenile Arthritis Damage Index, articular (JADI-A) and extra-articular (JADI-E) were evaluated.

**Results:** Median age was 15.4 [13;17] years, 84% were girls. Disease duration was 8.3 [4.4;11.2] years. 30% (15 pts), had RF/ACCP+, Sjogren's syndrome was diagnosed in 7 pts and uveitis in 4 pts. Steady damage with JADI>0 was found in 29 pts; JADI-A in 20 pts, JADI-E in 4, and both types of damage in 5 pts. JADI-A was mainly due to contractures of proximal interphalangeal and metacarpal joints of the hands – 74 and 65 cases respectively, contractures of the elbow - 19, wrist - 18, knee - 17, ankle, metatarsal joints - 12 each, restriction of mobility of the cervical spine - 10, shoulder joints - 6 and temporomandibular/micrognathia -5. JADI-E was due to eye damage in 4 pts and avascular bone necrosis in 3 pts.

Statistical analysis revealed significantly (p<.05) higher JRA activity in pts who had and didn`t have damage: CHAQ (0,5[0;0,75] vs 0[0;0,25]), JADAS10 (19,8[13;22] vs 10[6;12]), active joint count (9[4;18] vs 3[1;5]), fewer episodes of remission (11/29 vs 17/21), and leukocytosis at the moment of B initiation (7,9[5,9;10] vs 6,7[5,5;7,4]). There was no difference between RF+ and RF- pts.

In total 82 courses of B included TNFi in 35(43%) cases, Abatacept in 26(32%), IL-6 inhibitors in 10(12%), Tofacitinib in 9 (11%), Canakinumab in 1 (1%), and Rituximab in 1 (1%). The average duration of B therapy was 5.3 years. B was needed to switch to other line in 17 pts, among which 16 had damage and only one damage was not detected.

**Conclusion:** Pts with JRA have a greater risk for developing early damage. Leukocytosis and high clinical activity of the disease may predict rapid development of damage. The JADI may become a marker of refractoriness to DMARD and Biological therapy in JRA. It seems that the administration of B in this certain JIA subtype needs much earlier than in other kinds of JIA.

**Patient Consent**

Yes, I received consent

**Disclosure of Interest**

None declared

## P273 Infantile-onset juvenile idiopathic arthritis: a multicenter pera research group study

### K. Öztürk^1^, T. Aydın^2^, G. Ö. Baykal^3^, E. Bağlan^4^, H. Kısaoğlu^5^, D. G. Yıldırım^6^, H. Köse^7^, F. G. Demirkan^8^, G. O. Yener^9^, M. Çakan^10^, B. K. Demir^11^, H. A. Dündar^12^, G. Kılbaş^13^, H. E. Sönmez^14^, S. Türkuçar^15^, B. B. Makay^2^, S. Yüksel^13^, N. A. Ayaz^16^, S. Bakkaloğlu Ezgü^6^, M. Kalyoncu^5^, S. S. Kılıç^7^, S. Özdel^4^, B. Sözeri^3^, E. Ünsal^2^ on behalf of PeRA

#### ^1^Pediatric Rheumatology, Göztepe Prof. Dr. Süleyman Yalçın City Hospital, İstanbul; ^2^Pediatric Rheumatology, Dokuz Eylul University, İzmir; ^3^Pediatric Rheumatology, University of Health Sciences, Umraniye Research and Training Hospital, İstanbul; ^4^Pediatric Rheumatology, Ankara Etlik City Hospital, Ankara; ^5^Pediatric Rheumatology, Karadeniz Technical University, Farabi Hospital, Trabzon; ^6^Pediatric Rheumatology, Gazi University, Ankara; ^7^Pediatric Rheumatology, Uludag University, Bursa; ^8^Pediatric Rheumatology, Istanbul University, İstanbul; ^9^Pediatric Rheumatology, Şanlıurfa Research and Training Hospital, Şanlıurfa; ^10^Pediatric Rheumatology, University of Health Sciences, Zeynep Kamil Women and Children's Diseases Training and Research Hospital, İstanbul; ^11^Pediatric Rheumatology, İzmir Katip Çelebi University, İzmir; ^12^Pediatric Rheumatology, Van Training and Research Hospital, Van; ^13^Pediatric Rheumatology, Pamukkale University, Denizli; ^14^Pediatric Rheumatology, Kocaeli University, Kocaeli; ^15^Pediatric Rheumatology, Konya City Hospital, Konya; ^16^Pediatric Rheumatology, Istanbul University, İstanbul, Türkiye

##### **Correspondence:** K. Öztürk

*Pediatric Rheumatology 2023*, **21(Suppl 2):**P273

**Introduction:** Juvenile idiopathic arthritis (JIA) is one of the most heterogeneous diseases of childhood that presents various clinical and laboratory characteristics. Although there is variation according to subtypes, the age of onset has been reported to be approximately 6 years. It is known that it is most common at the age of 2 years and above.

**Objectives:** A multicenter retrospective study evaluated the clinical, laboratory, and follow-up findings of patients with JIA who experienced symptoms before the age of 2 years.

**Methods:** We conducted a retrospective review of the medical records of 201 patients with JIA who experienced symptoms before 24 months of age.

**Results:** The study included 199 patients who were monitored for a minimum of 6 months after being diagnosed. Of the patients, 151 (75.1%) were female, and 48 (24.1%) were male.10.6% of the patients had a history of consanguinity. In addition, a family history of rheumatic disease was found in 30 (15.1%) patients. The mean time of onset of symptoms was 16.9±4.5 months, and the median time to diagnosis was 20 (17-23) months. The most involved joints were the knee (87.4%), ankle (42.2%), wrist (21.1%), and hand PIP (20.6%). The most common subgroups were persistent oligoarticular JIA (59.8%), systemic onset (13.6%), and RF-negative polyarticular (11.6%) type. While ANA was positive in 69.2% of the patients, the rate of uveitis at the time of diagnosis was 9.5%. Methotrexate was the first treatment in 192 (96.5%) patients. After the initial treatment, 65.8% of patients achieved remission. However, during their follow-up, 51.7% of these patients required biological DMARDs. The most used bDMARD treatments were etanercept (31.7%), adalimumab (22.1%), and tocilizumab (13.1%). Biological DMARDs were switched in 35 (17.6%) patients. The patients were divided into two groups according to the onset time (before and after 12 months). Forty (20.1%) patients with an onset time before 12 months were identified. When the two groups were compared, a significant difference was found in terms of JIA subtype, joint involvement, ANA positivity, and presence of uveitis at follow-up. Macrophage activation syndrome developed in only 9 (4.5%) of the patients. The median follow-up period of the patients was 44 (15-74) months. At the last visit, 22.1% of the patients continued to have active disease, while 59.8% were in remission on medication and 18.1% were off medication.

**Conclusion:** To the best of our knowledge evaluating infantile-onset JIAs, the largest cohort has demonstrated some clinical differences. It has been observed that the involvement of PIP in the hands and feet, as well as the development of uveitis during follow-up, is particularly notable in the group whose symptoms began before 12 months.

**Patient Consent**

Yes, I received consent

**Disclosure of Interest**

None declared

## P274 Characterization of fibroblast-like synoviocytes isolated from the synovial fluid of patients affected by juvenile idiopathic arthritis

### S. Pelassa^1^, F. Raggi^1^, C. Rossi^1^, I. Prigione^1^, J. Ferro^2^, V. Vellone^2^, M. Gattorno^1^, A. Consolaro^3^, A. Ravelli^4^, M. C. Bosco^1^

#### ^1^Unit of Autoinflammatory Diseases and Immunodeficiencies; ^2^Fetal and Perinatal Pathology Unit, U.O.C. Anatomia Patologica; ^3^Pediatric Rheumatology Clinic; ^4^Scientific Direction, IRCCS G. Gaslini, Genova, Italy

##### **Correspondence:** S. Pelassa

*Pediatric Rheumatology 2023*, **21(Suppl 2):**P274

**Introduction:** Although the role of immune cells in the pathogenesis of Juvenile Idiopathic Arthritis (JIA) is well characterized, little is known about synovial cells. Fibroblast-like synoviocytes (FLS) have been mostly isolated from the synovial membrane (SM) or fluid (SF) of adult patients affected by Rheumatoid Arthritis (RA), Osteoarthritis, (OA), or in murine models of arthritis. Two different subsets of FLS were identified, specifically the lining (LL) and sublining (SL) FLSs involved in cartilage degradation and orchestration of immune responses, respectively. Furthermore, FLS from SF are considered pluripotent cells, able to differentiate towards a chondrocyte-like phenotype. Conversely, in JIA, FLS subdivision in different subtypes, pluripotent properties, or ability to differentiate in cells with a chondrocyte-like phenotype have never been assessed.

**Objectives:** The aim of this work was to characterize FLSs isolated from the SF of JIA patients with active disease. Membrane antigen profile, gene expression, and capacity of chondrogenic differentiation were evaluated.

**Methods:** Cells from the SF of 5 JIA patients with active disease undergoing arthrocentesis were isolated by adherence [1]. Skin fibroblasts (sFBs) from 3 healthy donors were used as controls. Cytofluorimetric analysis was carried out after staining with the Abs to the leukocyte marker CD45 and to the the fibroblast markers, PDPN, THY, and CAD-11. Gene expression was performed by RT-qPCR, on cell cultured under normal condition or stimulated with pro-inflammatory stimuli as TNFα or hypoxia (1% O_2_). Chondrogenic differentiation was induced by culturing pelleted cells for 4 weeks at 37 ^o^C using a differentiation or normal (control condition) medium. Pellets were then fixed in Paraformaldehyde and stained with Alcian Blue (AB) or sliced and stained with hematoxylin-eosin (HE) and AB.

**Results:** Citofluorimetric analysis showed that the majority of adherent cells from SF of active JIA patients was CD45^-^CD90^+^PDPN^+^, a profile previously reported as typical of FLSs of the synovial SL regions of the SM of adult patients affected by arthritides. In addition, cells were negative for CAD-11, a marker of LL FLS. RT-qPCR analysis showed that the expression levels of metalloproteinases was lower or comparable to sFBs, supporting the possibility of their involvement in immune responses rather than tissue degradation. The expression of the chondrocyte genes, BMP-4 and Aggrecan, in SF-derived adherent cells was higher than in sFBs. Gene expression of IL-6, IL8, and IL1β increased in FLS after stimulation with TNF or hypoxia.

Isolated cells also demonstrated to retain the ability of differentiating into chondrocytes-like cells when cultured in differentiation compared to control medium, as shown by HE staining. Accordingly, Glycosaminoglycans (GAG) production was higher in differentiated cells than in control cells, as assessed by AB staining

**Conclusion:** Our results demonstrated that cells isolated by adherence from the SF of JIA patients with active disease exhibit a phenotype similar to that reported for SL FLSs from adult arthritis patients, may play a role in the inflammatory processes in the joint, and seem to be able to differentiate into chondrocytes . Furthermore, these data support the importance of SF from JIA patients as a source of FLSs for future investigations

**Patient Consent**

Not applicable (there are no patient data)

**Disclosure of Interest**

None declared

**References**

1. Stebulis et al., Fibroblast-like Synovial Cells Derived From Synovial Fluid, J Rheumatol. 2005 Feb;32(2):301-6

## P275 Similarities and differences between juvenile idiopathic arthritis and adult rheumatoid arthritis. data from two university centres

### E. Pelechas^1^, N. Kougkas^2^, A. A. Drosos^1^, P. V. Voulgari^1^, E. Kaltsonoudis^1^

#### ^1^Rheumatology, University of Ioannina, Ioannina; ^2^Rheumatology, University of Thessaloniki, Thessaloniki, Greece

##### **Correspondence:** E. Pelechas

*Pediatric Rheumatology 2023*, **21(Suppl 2):**P275

**Introduction:** although there are obvious and known similarities and differences in the expression of inflammatory arthritides in all age groups due to immunopathogenetic factors, clinical phenotypes, clinical manifestations and treatment options, specific similarities and differences have not been extensively studied.

**Objectives:** to investigate factors influencing the outcomes and prognosis between juvenile idiopathic arthritis (JIA) and adult rheumatoid arthritis (aRA).

**Methods:** 33 patients (26 female) with a mean age of 32 years old and a mean follow-up of 5 ±2 years were evaluated. Three comparative groups with aRA, divided by age (20-40, 40-60, and over 60 years old), were evaluated. All patients were screened for RF, ANA, ENA, and anti-CCP, were clinically assessed using the DAS28, VAS, HAC indices, and all comorbidities were recorded.

**Results:** JIA prognosis relies mainly on the immunophenotype (RF, anti-CCP, ANA seropositivity) as well as the number of the involved joints. The transition from childhood to adulthood in our cohort found 5 patients with blindness, all being ANA positive. On the other hand, ANA-positive aRA patients are often associated with Sjögren’s syndrome and thyroiditis.

In addition, extra-articular manifestations and comorbidities, such as pulmonary involvement, were more common in the ANA-positive aRA, over 60 years old population, influencing significantly treatment decisions.

**Conclusion:** inflammatory joint disease presents a significant heterogenicity and regardless of the age of onset it can be classified as oligoarticular, polyarticular or systemic disease. Recent molecular and immunological advances may lead to more selective immunotherapeutic interventions that have not been available to date. Strong HLA associations and specific T-lymphocyte populations are hallmarks of both aRA and JIA. Ideally, TCR-HLA interactions that may differ between age groups should be identified.

**Trial registration identifying number:** not applicable

**Patient Consent**

Not applicable (there are no patient data)

**Disclosure of Interest**

None declared

## P276 Nonresponsiveness to anti-tumor necrosis factor (anti-TNF) agents: another anti-TNF agents or other pathways?

### G. Pirim, B. Sozeri

#### Pediatric Rheumatology, Umraniye Training and Research Hospital , Istanbul, Türkiye

##### **Correspondence:** G. Pirim

*Pediatric Rheumatology 2023*, **21(Suppl 2):**P276

**Introduction**

The anti-tumor necrosis factor (anti-TNF) agents such as infliximab, etanercept and adalimumab, used as an alternative or in combination with synthetic disease-modifying antirheumatic drugs (DMARDs) in the treatment of juvenile idiopathic arthritis (JIA) and JIA-associated uveitis. Dose adjustment or switching of biologic agents may be required due to inadequate initial response or drug resistance to anti-TNF agents.

**Key stones**

adalimumab, etanercept, infliximab, JİA, uveitis

**Objectives**

To report the experience of treating JIA and JIA-associated uveitis with resistance to anti-TNF agents using another anti-TNF agents or other biologic agents.

**Methods**

The treatment approaches of 377 patients who received first-choice anti-TNF agent treatment for JIA and JIA-associated uveitis in Umraniye Training and Research Hospital between 2016-2023 were evaluated retrospectively.

**Results:** Drug resistance developed in 147 of 377 patients with JIA and JIA-associated uveitis who received first-choice anti-TNF agents. Dose adjustment, switching to another anti-TNF agent, and using other pathway agents (tocilizumab, secukinumab, tofaticinib) were among the treatment approaches. patients who developed drug resistance, 60.5% were diagnosed with oligoarticular JIA, 20.4% with spondyloarthritis, 10.9% with polyarticular JIA and 8.2% with psoriatic arthritis. Drug resistance developed to adalimumab in 36% (53/147) patients, etanercept in 54.4% (80/147) and infliximab in 9.5% of patients. It was found that 70.1% (103/147) of the patients used other anti-TNF agents as alternative treatment, 17.7% (26/147) preferred other pathway drugs, and 12.2% (18/147) dose increments were made. Uveitis was present in 77.7% (14/18) of the patients whose dose was increased. In the case of drug resistance, adalimumab was the most dose-increased among the anti-TNF agents, while etanercept was used to switch to other pathway agents.The success rate in switching to an anti-TNF agent was 78% for adalimumab, 61% for etanercept and 57% for infliximab.There was a higher treatment rate in the etanercept than adalinumab in choosing the other pathway (28% vs. 16%).If there is no response despite increasing the dose of adalimumab, the response is 66% with a second dose increase. No significant difference was found between age and diagnosis groups in drug preferences in anti-TNF treatment unresponsiveness (p>0.05). There was no significant difference between the treatment responses after the first drug change in patients with uveitis (p>0.05).

**Conclusion:** In this study we found that JIA patients receiving anti-TNF therapy develop drug resistance, switching to other most commonly used anti-TNF agents can lead to the inactive period.

**Patient Consent**

Not applicable (there are no patient data)

**Disclosure of Interest**

None declared

## P277 Changes to service provision for JIA in the East of England over ten years: an audit

### F. Price-Kuehne^1^, A. Sharma^2^, P. Bale^1,2^, K. Armon^1,2^

#### ^1^Cambridge University Hospitals NHS Foundation Trust, Cambridge; ^2^Norfolk and Norwich University Hospitals NHS Foundation Trust, Norwich, United Kingdom

##### **Correspondence:** F. Price-Kuehne

*Pediatric Rheumatology 2023*, **21(Suppl 2):**P277

**Introduction:** There are an estimated 500 children in receipt of treatment for JIA in the East of England. After an audit 10 years ago demonstrated inequalities and inadequate provision of care, the Child and adolescent East of England Rheumatology Service (CHEERS) network was established in 2017. This service network consists of 17 hospitals across a 100 x 125 mile predominantly rural area, and includes 2 consultant paediatric rheumatologists, 2 specialist hubs, 2 direct referral hospitals and 6 outreach clinics.

**Objectives:** We aimed to assess the provision of care for JIA in the East of England against the British Society for Paediatric and Adolescent Rheumatology/Arthritis and Musculoskeletal Alliance (BSPAR/ARMA) standards of care for JIA, 2010 (1).

**Methods:** A service provision questionnaire was sent to the Paediatric Rheumatology leads at the 17 regional hospitals, followed by a case-note review of a random sample of JIA patients from each centre. Data obtained in 2022 was compared to the original audit in 2011, prior to the establishment of the regional network.

**Results:** 15/17 regional centres returned service provision questionnaires and 10/17 participated in the case-note review, which included 105 patients.

Compared to the audit ten years previously, the provision of a child-friendly clinic setting had improved from 81% to 100% of centres, and a clinic specifically for adolescents from 19% to 53%. Access to a paediatric rheumatology clinical nurse specialist (CNS) had also improved from 38% to 67%, while access to physiotherapy had remained high (88% to 93%). Around half of centres provided access to occupational therapy and psychology, which had not changed.

Case note review confirmed improved access to: CNS (61% to 78%); physiotherapy (35% to 80%); ophthalmology screening within 6 weeks (30% to 74%); home methotrexate delivery (55% to 75%); and 92% of patients are now discussed with a specialist team.

Of note, in both 2011 and 2022, almost all hospitals were unable to offer the BSPAR/ARMA standard of 45 minutes for a new appointment (0% to 7%) and >20 minutes for a follow-up appointment (31% to 53%), and although improved over the ten-year period, only two thirds were seen within 4 weeks of receipt of referral (56% to 66%), with only 40% seen within 10 weeks of symptom onset.

**Conclusion:** This re-audit of service provision in JIA across the East of England over a ten-year period demonstrated improved access to specialist paediatric rheumatology care across the region following establishing a specialist network.

Timely access to Paediatric Rheumatology clinics remains challenging. In the current NHS climate, achieving the appointment times as recommended by the BSPAR/ARMA standard of care may be unrealistic.

Overall, these data show the value of establishing a formal clinical network, especially in an area where care is provided across a wide geographical area.

**Patient Consent**

Not applicable (there are no patient data)

**Disclosure of Interest**

None declared

**References**

1. Davies K, Cleary G, Foster H, Hutchinson E, Baildam E; British Society of Paediatric and Adolescent Rheumatology. BSPAR Standards of Care for children and young people with juvenile idiopathic arthritis. Rheumatology (Oxford). 2010;49(7):1406-8

## P278 Investigating the diversity of KLRB1-expressing T cells using single-cell rna sequencing in the synovial fluid of children with juvenile idiopathic arthritis

### E. Ralph^1,2,3^, C. Bolton^1,4,5^, C. Mahony^5^, Y. Sanchez-Corrales^3,6^, T. Xenakis^3,6^, V. Alexiou^1,2,3^, A. Croft^5^, S. Castellano^3,6^, L. Wedderburn^1,2,3^

#### ^1^Infection, Immunity and Inflammation Department, UCL Great Ormond St Institute of Child Health; ^2^Centre for Adolescent Rheumatology Versus Arthritis at UCL University College London Hospital (UCLH) and Great Ormond Street Hospital (GOSH); ^3^NIHR Great Ormond Street Hospital Biomedical Research Centre, London; ^4^The Kennedy Institute, University of Oxford, Oxford; ^5^Institute of Inflammation and Ageing, University of Birmingham, Birmingham; ^6^Genetics and Genomic Medicine Department, UCL Great Ormond St Institute of Child Health, London, United Kingdom

##### **Correspondence:** E. Ralph

*Pediatric Rheumatology 2023*, **21(Suppl 2):**P278

**Introduction:** CD161 (*KLRB1*)-expressing T cells have been shown to be highly enriched in the synovial fluid of children with Juvenile Idiopathic Arthritis (JIA), with CD161 expressed on several T cell populations including Th17, some Th1 cells (‘ex-Th17’ cells), Th17/1 cells, as well as FOXP3+ regulatory CD4 T cells (Treg). We have generated a single-cell atlas of *KLRB1*/CD161 expression in the cell populations present in synovial fluid with the aim of further understanding the role that these cells may play in the pathogenesis of JIA.

**Objectives:** To investigate the diversity of *KLRB1*/CD161-expressing T cell populations in the synovial fluid of children with JIA using single-cell RNA sequencing.

**Methods:** Single-cell RNASeq analysis was carried out on synovial fluid mononuclear cell (SFMC) samples of JIA patients (n=5), targeting 50,000 cells per patient. Gene expression and TCR libraries were prepared using the Chromium Single Cell 5' Reagent Kit (v2, Dual Index) (10X Genomics) and sequenced on a NovaSeq 6000 (Illumina). Sequencing reads were pre-processed and aligned to the GrCh38 reference genome using Cell Ranger v6 (10x Genomics). Quality control and downstream analysis, including T cell sub-clustering, was performed using Seurat v4.

**Results:** *KLRB1* was found to be expressed in a variety of cell clusters in the T cell compartment in synovial fluid. These were identified as MAIT cells, *VD2*+ gamma delta T cells, *KLRB1+* CD4 T helper cells, central memory CD4 T cells, *FOXP3*+ Tregs, an *EOMES+IL10+* cluster of CD4 T cells that resembled inducible regulatory T cells, and a cluster of *CXCL13*+ peripheral T helper cells (Tph). Peripheral T helper cells are a relatively recently described population of T cells, initially in the context of Rheumatoid Arthritis, and have been shown to provide B cell help in the periphery. Transcriptional differences between *KLRB1*-expressing and non-expressing cells were investigated and found to be particularly notable in the Treg cluster, where *KLRB1+* cells showed differential upregulation of genes characteristic of Tph cells.

**Conclusion:** This works confirms expected expression of *KLRB1* in diverse T cell populations. The observation that some of the differentially expressed genes in *KLRB1*-expressing Tregs are those that define Tph cells, suggests a previously undescribed link or shared transcriptional programme between Tregs and Tph cells. Future work will investigate a potential relationship between these cell populations using trajectory analysis and examination of T cell repertoire.

**Patient Consent**

Yes, I received consent

**Disclosure of Interest**

E. Ralph Grant / Research Support with: The CLUSTER Consortium is supported by grants from MRC, Versus Arthritis and GOSCC and has partnerships with AbbVie, GSK, UCB, Sobi and Pfizer inc. Also supported by the NIHR BRC at GOSH., C. Bolton: None declared, C. Mahony: None declared, Y. Sanchez-Corrales Grant / Research Support with: Supported by the NIHR BRC at GOSH, T. Xenakis Grant / Research Support with: Supported by the NIHR BRC at GOSH, V. Alexiou Grant / Research Support with: The CLUSTER Consortium is supported by grants from MRC, Versus Arthritis and GOSCC and has partnerships with AbbVie, GSK, UCB, Sobi and Pfizer inc. Also supported by the NIHR BRC at GOSH., A. Croft: None declared, S. Castellano Grant / Research Support with: Supported by the NIHR BRC at GOSH, L. Wedderburn Grant / Research Support with: The CLUSTER Consortium is supported by grants from MRC, Versus Arthritis and GOSCC and has partnerships with AbbVie, GSK, UCB, Sobi and Pfizer inc. Also supported by the NIHR BRC at GOSH. In kind contributions to CLUSTER by AbbVie, GSK, UCB, Sobi and Pfizer inc and non renumerated collaborations with Lilly and Novartis.

## P279 How often was remission achieved before the treat-to-target era in juvenile idiopathic arthritis?

### A. I. Rebollo-Giménez^1,2^, A. Pistorio^3^, S. M. Orsi^4^, F. Ridella^4^, E. Aldera^4^, L. Carlini^1^, V. Natoli^1,5^, M. Burrone^4^, S. Rosina^1^, A. Consolaro^1^, E. Naredo^6^, A. Ravelli^3^

#### ^1^Unit of Rheumatology and Autoinflammatory Diseases, IRCCS Istituto Giannina Gaslini, Genoa, Italy, ^2^Universidad Autónoma de Madrid, Madrid, Spain, ^3^Scientific Direction, IRCCS Istituto Giannina Gaslini, ^4^ Università Degli Studi Di Genova, Dipartimento di Neuroscienze, Riabilitazione, Oftalmologia, Genetica e Scienze Materno-Infantili, ^5^Dipartimento di Neuroscienze, Riabilitazione, Oftalmologia, Genetica e Scienze Materno-Infantili, Università Degli Studi Di Genova, Genoa, Italy, ^6^Department of Rheumatology, Bone and Joint Research Unit. Hospital Universitario Fundación Jiménez Díaz, IIS Fundación Jiménez Díaz, and Universidad Autónoma de Madrid, Madrid, Spain

##### **Correspondence:** A. I. Rebollo-Giménez

*Pediatric Rheumatology 2023*, **21(Suppl 2):**P279

**Introduction:** After the historical therapeutic approach based on the so-called “therapeutic pyramid”, the management of juvenile idiopathic arthritis (JIA) has evolved over the year towards the earlier introduction of the most effective medications and the aim of achieving disease remission as quickly as possible. In 2018, the recommendations for the treat-to-target (T2T) strategy in JIA were published. This approach is gaining increasing popularity in pediatric rheumatology practice. However, to set a benchmarking for future outcome comparison with the T2T era, there is the need to know how often clinical inactive disease was obtained before the introduction of the T2T concept.

**Objectives:** To describe the percentage of patients who attained clinical inactive disease (CID) within 2 and 5 years after treatment start before the T2T era.

**Methods:** The clinical charts of all consecutive patients who were newly diagnosed with JIA at the study center between 2007 and 2018 and had antirheumatic therapy started within 6 months after disease onset were reviewed retrospectively. The achievement of the state of CID, defined according to Wallace 2004 criteria, was assessed at 6, 12, 18, 24 and 60 months after treatment start.

**Results:** A total of 394 patients (292 females and 102 males), whose median follow up was 56.1 months, were included. Patients were subdivided into four functional JIA phenotypes: systemic (N=28, 7.1%), persistent oligoarticular (N=190, 48.2%), polyarticular (N=159,40.4%) -including extended oligoarticular, RF positive and negative polyarthritis ILAR categories- and other (N=17, 4.3%) – comprising enthesitis-related, psoriatic and undifferentiated JIA ILAR categories. During follow-up 85% of the patients received intra-articular corticosteroids, 75.4% conventional synthetic DMARDs, and 40.4% biologic DMARDs.

CID was attained by 99/394 (25.1%) patients at 6 months, 134/370 (36.2%) patients at 12 months, 126/332 (38%) patients at 18 months, 119/267 (44.6%) patients at 24 months, and 68/142 (47.9 %) patients at 60 months. The highest rate of CID among all functional JIA phenotypes at 6 months was seen in patients with systemic and oligoarticular JIA (32.1% and 29.5%, respectively); CID rates increased in all functional categories at 24 months (systemic 53.3 %, oligoarthritis 42.3%, polyarthritis 44.9% and other categories 43.8%).

**Conclusion:** The proportion of patients who attained CID before the treat-to-target era increased progressively over time but remained lower than 50% up to 5 years. Patients who reached an earlier CID had a systemic and oligoarticular phenotype. The proportion of patients who received intraarticular corticosteroids and csDMARDs during follow-up was higher comparing to that of patients who received bDMARDs.

**Patient Consent**

Not applicable (there are no patient data)

**Disclosure of Interest**

None declared

**References**

1. Ravelli A, et al. Treating juvenile idiopathic arthritis to target: recommendations of an international task force. Ann Rheum Dis. 2018 Jun;77(6):819-828. doi: 10.1136/annrheumdis-2018-213030. Epub 2018 Apr 11. PMID: 29643108.

## P280 Risk factors for arthritis relapse in mono and oligoarticular juvenile idiopathic arthritis

### C. Rigoni^1^, F. Tirelli^1^, G. Martini^1^, F. Vittadello^2^, A. Meneghel^1^, F. Zulian^1^

#### ^1^Department of Woman and Child Health, University of Padova, Padova; ^2^Fabio Vittadello, Explora - Research and Statistical Analysis, Vigodarzere, Italy

##### **Correspondence:** C. Rigoni

*Pediatric Rheumatology 2023*, **21(Suppl 2):**P280

**Introduction:** An increased number of clinical evidence shows that monoarticular juvenile idiopathic arthritis (monoJIA) is distinct from the oligoarticular form (oligoJIA) but little is known regarding the clinical course and risk factors for relapse of arthritis in these two clinical entities.

**Objectives:** To describe the possible risk factors for arthritis relapse in a cohort of patients with mono and oligoarticular JIA followed for a long time in a single Centre.

**Methods:** Patients with Mono and Oligo JIA, followed for at least two years entered the study. The absolute number of arthritis relapses and the number of relapses/patient/year were evaluated in the two groups. The possible risk factors for relapse considered were: gender, presence of uveitis, positivity of antinuclear antibodies (ANA), therapy, puberty and presence of benign joint hypermobility (BJH).

**Results:** A total of 343 patients affected by JIA, 118 monoJIA and 225 oligoJIA, followed at our center for an average of 11.8 years, were considered. The mean number of relapses/patient/years was 0.47 in oligoJIA and 0.11 in monoJIA (p 0.000). Puberty had a positive influence on the frequency of relapses as, in the pre-pubertal period, relapses averaged 0.59/patient/year versus 0.21 in the post-pubertal period. This difference was significant both in oligoJIA (0.73 versus 0.33, p 0.001) and in monoJIA (0.32 versus 0.05/year, p 0.000). As regards the relationship between relapses and therapy, one third of the relapses occurred on DMARDs treatment. Of these, 65% were on methotrexate (MTX) and 35% on biological agents (BA). We did not observe a significant difference in this aspect between oligo and monoJIA. Gender, uveitis, ANA and BJH did not influence the frequency of arthritis relapses.

**Conclusion:** MonoJIA has a more benign course than oligoJIA with a significantly lower frequency of arthritis relapses. Puberty represents a protective factor with regard to relapses in both mono and oligoJIA forms regardless of sex. One third of all relapses occurred despite DMARDs therapy.

**Patient Consent**

Not applicable (there are no patient data)

**Disclosure of Interest**

None declared

## P281 Safety of antirheumatic drugs in patients with juvenile idiopathic arthritis

### I. Rukavina, M. Šestan, M. Held, D. Grgurić, M. Frković, M. Jelušić

#### University hospital centre Zagreb, Zagreb, Croatia

##### **Correspondence:** I. Rukavina

*Pediatric Rheumatology 2023*, **21(Suppl 2):**P281

**Introduction:** Juvenile idiopathic arthritis (JIA) is the most common chronic rheumatologic disease in children and it is characterised by arthritis of unknown origin. During treatment with antirheumatic drugs, numerous adverse events have been reported.

**Objectives:** To determine and compare the incidence of adverse events (AE) during treatment of JIA with synthetic and biologic disease modifying antirheumatic drugs (s/b DMARD).

**Methods:** We retrospectively reviewed the medical files of children aged 1-18 years who were diagnosed with JIA and had received synthetic DMARDs (metotrexate, sulfasalazine, leflunomide) and biologics (adalimumab, etanercept, infliximab, tocilizumab, abatacept, golimumab, secucinumab or tofacitinib) between January 2014 and December 2022 in Pediatric Clinic at University Hospital Centre Zagreb. Patients who were followed up regularly for at least 6 months were included.

**Results:** 359 patients had received synthetic DMARDs and biologic DMARDs were given to 152 patients. The highest number of adverse events was in the synthetic DMARD group (55 patients, 15,3 % ): 36 patients who received metotrexate did not tolerate the drug due to nausea and vomiting (10 %) and 12 of them had developed transitory elevated liver enzymes (3,3 %). Sulfasalazine was ceased in 6 patients (1,67 %): at 2 patients due to allergic rash, in 2 due to excessive hair loss and in 2 of them due to stomach pain and vomiting. There is also one patient that had received leflunomide who developed chest pain and fatigue. In biologic DMARD group there was less side effects but serious adverse events were reported in 4 patients (1,1 %) (adalimumab - miliary tuberculosis (1) and elbow septic arthritis (1), tocilizumab - chronic disseminated intravascular coagulation (1), infliximab - one patient developed anaphylactic reaction). Skin allergic reaction was noticed among biologics as well - anakinra (2), etanercept (1), adalimumab (1) and 2 patients developed psoriasis like rash (adalimumab). One patient in this group experienced headache while he had received adalimumab.

**Conclusion:** Immunosuppressive therapy for JIA is generally well tolerated and not toxic. AE are more often in sDMARD group while in bDMARD group are more serious. Since side effects, particularly serious one, can occur, caution and regular check-ups are needed.

**Patient Consent**

Not applicable (there are no patient data)

**Disclosure of Interest**

None declared

## P282 Patient’s global assessment of well-being in long-term disease activity: results of 18-years follow-up in the population-based nordic JIA cohort

### V. Rypdal^1,2^, M. Glerup^3^, M. Rypdal ^4^, E. D. Arnstad^5,6^, K. Aalto ^7^, L. Berntson^8^, A. Fasth ^9^, T. Herlin^3^, C. Myrup^10^, S. Peltoniemi^11^, M. Rygg^12,13^, E. Nordal^1,2^ on behalf of the Nordic Study Group of Pediatric Rheumatology (NoSPeR)

#### ^1^Dep. of Pediatrics, University Hospital of North Norway; ^2^Dep. of Clinical Medicine, UIT the Arctic University of Norway, Tromsø, Norway; ^3^Dep. of Pediatrics, Aarhus University Hospital, Aarhus, Denmark; ^4^Dep. of Mathematics and Statistics, UIT the Arctic University of Norway, Tromsø; ^5^Dep. of Pediatrics, Levanger Hospital, Nord-Trøndelag Hospital Trust, Levanger; ^6^Dep. of Clinical Medicine, NTNU - Norwegian University of Science and Technology, Trondheim, Norway; ^7^Pediatric Research Center, New Children's Hospital, Helsinki University Hospital, Helsinki, Finland; ^8^Dep. of Women’s and Children’s Health, Uppsala University, Uppsala; ^9^Dep. of Pediatrics, Institute of Clinical Sciences, Sahlgrenska Academy, University of Gothenburg, Gothenburg, Sweden; ^10^Dep. of Pediatrics, Rigshospitalet Copenhagen University Hospital, Copenhagen, Denmark; ^11^Clinic of Rheumatology, Helsinki University Hospital, Helsinki, Finland; ^12^Dep. of Pediatrics, St. Olavs Hospital; ^13^Dep. of Clinical and Molecular Medicine, NTNU - Norwegian University of Science and Technology, Trondheim, Norway

##### **Correspondence:** V. Rypdal

*Pediatric Rheumatology 2023*, **21(Suppl 2):**P282

**Introduction:** Patients with juvenile idiopathic arthritis (JIA) may have a considerable disease burden despite no clinical signs of active inflammatory arthritis. We previously found in the Nordic JIA cohort that the patient’s global assessment (PaGA) contributed the most to a clinical Juvenile Arthritis Disease Activity Score 10 (cJADAS10) >1.

**Objectives:** The study aimed to explore factors associated with a higher PaGA score. Among the evaluated factors were the active joint count, health-related quality of Life (HRQoL) assessed with the 36-Item Short-Form Health Survey (SF-36), yielding both a mental and physical summary score. Sleep quality assessed by the validated Pittsburgh Sleep Quality Index (PSQI). Fatigue assessed by the validated Fatigue Severity Scale (FSS). Pain and the physician’s global assessment of disease activity (PhGA) denoted on a 10 cm visual analogue scale (VAS). In addition, information regarding social security benefits was evaluated.

**Methods:** In the prospective population-based multicenter Nordic JIA cohort, 427 patients fulfilled the inclusion criteria of having JIA according to the International League of Associations for Rheumatology (ILAR) criteria and a minimum of two study visits. Wilcoxon signed-rank test was used to assess the contribution of PaGA on the cJADAS10 score. Several different methods of unsupervised clustering were used to explore factors associated with PaGA.

**Results:** The PhGA and the PaGA scores increased significantly from the 8-year to the 18-year visit among patients with active disease (cJADAS10 score >1) at the last visit. The increase in the PaGA was significantly higher than the increases in the PhGA and the active joint count (p>0.0001). Clustering analyses did not show a natural splitting of the patients into distinct groups but showed a continuum where the patients with unfavorable scores on several of the assessed variables had higher PaGA scores.

**Conclusion:** In this study, the PaGA reflects valuable and complex information about the disease state 18-years after JIA onset, information that is not captured by physician-reported measurements. In a treat-to-target (T2T) approach, we need to better understand how our patients interpret the concept of inactive disease. The results also suggest a need for standardized instruction for scoring PaGA.

**Trial registration identifying number:** Not applicable

**Patient Consent**

Yes, I received consent

**Disclosure of Interest**

None declared

## P283 Unmet medical needs in patients with juvenile idiopathic arthritis in Japan: using the national database of health insurance claims and specific health checkups of Japan

### R. Sakai^1^, E. Inoue^2^, M. Shimizu^3^, K. Mitsunaga^4^, Y. Inoue^5^, T. Miyamae^6^

#### ^1^Department of Public Health and Epidemiology, Meiji Pharmaceutical University; ^2^Showa University Research Administration Center, Showa University; ^3^Department of Pediatrics and Developmental Biology,Graduate School of Medical and Dental Sciences, Tokyo Medical and Dental University, Tokyo; ^4^Department of Allergy and Rheumatology, Chiba Children’s Hospital; ^5^Department of General Medical Science, Graduate School of Medicine, Chiba University, Chiba; ^6^Division of Rheumatology, Department of Internal Medicine, Tokyo Women’s Medical University School of Medicine, Tokyo, Japan

##### **Correspondence:** R. Sakai

*Pediatric Rheumatology 2023*, **21(Suppl 2):**P283

**Introduction:** Although the treatment approved for Juvenile idiopathic arthritis (JIA) is covered by public health insurance in Japan, public health insurance covers limited medicines such as naproxen and ibuprofen as NSAIDs, conventional synthetic disease modifying antirheumatic drugs (csDMARDs), and some biological DMARDs (bDMARDs) for JIA. It is expected that unmet medical needs (UMNs) about medications and other medical practices for JIA exist, however, details have not been known yet.

**Objectives:** To assemble UMNs from rheumatologists, and investigate the trends in treatments in patients with JIA based on the UMNs using the National Database of Health Insurance Claims and Specific Health Checkups of Japan (NDB).

**Methods:** We collected possible UMNs about treatments of JIA from 16 experts in pediatric and non-pediatric rheumatology. We categorized possible UMNs into the following categories: patients’ characteristics (age, sex, severity, comorbidities), treatments (medication, clinical laboratory tests), medical costs relating with treatments for JIA, life stage (transitional care), and regional disparity in above categories. To describe trends in treatments for JIA, we used NDB, the largest health insurance database in Japan, which covers over 90% of population in Japan. No clinical and laboratory data were included in NDB. Considering the lack of data in NDB, we selected possible UMNs to investigate, and rheumatologists prioritized them considering the clinical importance. We described patients’ characteristics, actual use of the medication, and medical costs based on the priority of UMNs using the NDB. We defined patients who had at least one ICD10 code for JIA (M08.x, M090) and prescription of NSAIDs, csDMARDs, and bDMARDs approved for JIA in Japan as of October 2020 using NDB between April 2019 and March 2020.

**Results:** We collected 58 possible UMNs from 16 rheumatologists. Among these possible UMNs, 34 UMNs were selected (8 for patients’ characteristics, 19 for treatments, 3 for life stage, 3 for medical cost, 1 for regional disparity in above categories). The most highly prioritized UMNs were utilization of tocilizumab (TCZ) and abatacept (ABT). The number of patients with JIA was 19,880 (female 53.0%). The prevalence of uveitis was 2.3%. The percentage of patients with prescriptions of NSAIDs, oral corticosteroids, csDMARDs, and bDMARDs was 68.5, 34.5, 52.1, and 36.7 respectively. Among patients treated with bDMARDs, 21.3% of patients had prescriptions of TCZ and 1.4% had those of ABT. The medication expenditure per one patient per month was 384.1 euro (1 EUR=150.1 JPY).

**Conclusion:** We assembled UMNs in treatments for JIA, and showed clinical practice such as medication for the first time in Japan. Based on the information on the actual medical situation, future issues will be raised.

**Trial registration identifying number:** Not applicable

**Patient Consent**

Not applicable (there are no patient data)

**Disclosure of Interest**

R. Sakai Consultant with: Mitsubishi Tanabe Pharma Corporation, E. Inoue Grant / Research Support with: Nippontect systems Co.,Ltd. and Cyberdyne Inc., Speaker Bureau with: Bristol Myers Squibb Co., Ltd. and Eisai Co., Ltd., M. Shimizu Grant / Research Support with: MEDICAL & BIOLOGICAL LABORATORIES CO., LTD., K. Mitsunaga: None declared, Y. Inoue Grant / Research Support with: Ministry of Health, Labour and Welfare in Japan, T. Miyamae: None declared

## P284 Early diagnosis of comorbid kidney damage in children with juvenile idiopathic arthritis

### S. Samsonenko

#### Pediatrics, Dnipro State Medical University, Dnipro, Ukraine

##### **Correspondence:** S. Samsonenko

*Pediatric Rheumatology 2023*, **21(Suppl 2):**P284

**Introduction:** The problem of JIA is determined by the significant prevalence, chronic course of the disease, the severity of clinical manifestations of erosive-destructive arthritis, as well as comorbid damage to vital organs and systems, which greatly aggravates the course of the underlying disease and worsens its prognosis.

**Objectives:** Increasing the efficiency of early diagnosis and determining the risk factors of comorbid kidney damage in children with JIA, based on the study of renal biomarkers of functional status and structural damage of the kidneys.

**Methods:** Clinical, laboratory (general clinical, immunoenzymatic: cystatin C, KIM-1, TGF – β1), instrumental.

**Results:** Urinary syndrome was detected in 8.8% of children with JIA, and in 11.3% - changes in standard ultrasound of the kidneys. Meanwhile, a decrease in e-GFR according to the Counahan-Barratt formula was noted in 34% of children at the first and 26% at the second study. Estimated GFR based on serum cystatin C using the Hoek formula revealed a decrease in e-GFR in 41% of patients with JIA.

Elevated levels of KIM-1 and TGF-β1 biomarkers in urine were found in 25% of children with JIA. The increase in the level of these markers occurred when the disease activity was maintained for more than 4 years, the total duration was more than 6 years, and there was a reduced GFR and polyarthritis. A significant frequency of increased renal biomarkers in children with JIA in the presence of hypertension syndrome was noteworthy.

The results of the examination of renal markers depending on the characteristics of therapy showed the following. When using short courses of NSAIDs in children receiving methotrexate, only a tendency to increase KIM-1 in the urine was found. At the same time prolonged use of NSAIDs had a negative effect on the level of KIM-1.

The use of a combination of methotrexate and NSAIDs led to an increase in TGF-β1 levels and quadrupled the chances of increasing this marker of early renal fibrosis. In patients with a combination of methotrexate and immunobiological therapy low levels of renal biomarkers and no risk of increased TGF-β1 were observed.

**Conclusion:** Functional kidney damage is registered in 41.3% of patients with JIA.

Structural damage to the renal tubules in children with JIA is noted in 25% of patients. Risk factors for renal tubular lesions are high JIA activity (OR = 7.25; 95% CI: 1.22-43.22; p<0.04), arthritis ≥ 6 joints (OR = 5.00; 95% CI: 1.65-15.15; p<0.006), hip arthritis (OR = 10.41; 95% CI: 1.02-106.7; p<0.05), arterial hypertension (OR = 12.43 ; 95% CI: 2.26-68.27; p<0.003). An association of the development of an increased level of TGF-β1 content with the duration of the active stage of JIA ≥ 4 years (OR = 6.11; 95% CI: 2.01-18.58; p<0.01), an increase in ESR (OR = 4, 33; 95% CI: 1.35-13.88; p<0.05), polyarthritis (OR = 3.74; 95% CI: 1.12-12.51; p<0.05), arterial hypertension (OR = 6.33; 95% CI: 1.36-29.55; p<0.05).

**Patient Consent**

Not applicable (there are no patient data)

**Disclosure of Interest**

None declared

## P285 Preliminary analysis and comparison of synovial fluid leucocyte abnormalities and cytokine profile in juvenile idiopathic arthritis subtypes

### A. Scanu^1^, F. Tirelli^1^, A. Meneghel^1^, R. Luisetto^2^, C. Baggio^3^, M. L. Cagnato^1^, R. Ramonda^3^, F. Oliviero^3^, G. Martini^1^, F. Zulian^1^

#### ^1^Department of Women’s and Children’s Health-SDB; ^2^Department of Surgery, Oncology and Gastroenterology-DISCOG; ^3^Department of Medicine-DIMED, University of Padova, Padova, Italy

##### **Correspondence:** A. Scanu

*Pediatric Rheumatology 2023*, **21(Suppl 2):**P285

**Introduction:** Currently, no specific markers are available for the early stratification of patients with different subtypes of Juvenile Idiopathic Arthrits (JIA) and for prediction of disease course. It has been demonstrated that genomic instability and increased sensitivity to DNA damage can be associated with rheumatic diseases (1), but this has not been yet investigated in JIA.

**Objectives:** To evaluate differences in cell populations, cytokine concentrations, and genotoxicity signs in synovial fluid (SF) between polyarticular (poly)-JIA and oligoarticular (oligo)-JIA and according to different disease features.

**Methods:** SFs collected from the knees of 24 patients diagnosed with JIA according to ILAR criteria (2), aged 3-15 years and untreated for at least 6 months were examined under optical microscopy. White blood cell count (WBC) and the polymorphonuclear cell (PMN) percentage were determined according to standard procedures. IL-1β and IL-8 concentrations were determined by ELISA. May-Grünwald-Giemsa staining was used to evaluate the leukocyte abnormalities caused by genotoxic events (hypo- or hypersegmented PMN, binucleated monocytes, and cells with micronuclei (MN), nuclear buds, pyknosis, necrosis, and apoptosis). Cell free-DNA (cf-DNA) was isolated and quantified by spectrophotometry.

**Results:** Nine patients had poly-JIA, of which 6 were at disease onset. Of the 15 patients with oligo-JIA, 8 were at disease onset, 12 had positive antinuclear antibodies (ANA+) and 5 had uveitis (UVE+). Although not significantly, WBC count and PMN percentage were greater in SFs of patients with poly-JIA (WBC:11388.89±9176.39 cells/mm^3^, PMN:51.53±24.29%) than in oligo-JIA (WBC:7173±5159cells/mm^3^, PMN:42.4±25.5%). When SFs of children at disease onset were analyzed, WBC levels and PMN percentage were 2.16 and 1.44-fold higher, respectively, in poly-JIA than in oligo-JIA (p<0.05). IL-1β and IL-8 concentrations were greater in SFs of poly-JIA (IL-1β:58.71±90.74 pg/ml; IL-8:579.20±530.25 pg/ml) than oligo-JIA (IL-1β:38±66 pg/ml; IL-8:368.6±394.52 pg/ml), but the difference were significant only for IL-8 at disease onset (2.41-fold; p<0.01). Poly-JIA at disease onset also had a percentage of cells with MN 4.06-fold higher than that in oligo-JIA (p<0.001). SFs from oligo-JIA patients with uveitis displayed IL-1β levels and percentage of cells with MN 16.72 and 2.92-fold higher (p<0.05), respectively, than oligo-JIA patients without uveitis. Interestingly, the group with uveitis showed significantly higher cf-DNA levels (oligo-JIA-UVE+:35.92±25.65 ng/μl; oligo-JIA-UVE-:6.27±2.71 ng/μl; p<0.05). Comparison between oligo-JIA-ANA+ and oligo-JIA-ANA- patients did not show any statistical differences in the studied parameters.

**Conclusion:** This study shows that poly-JIA SFs, especially at the disease onset, have a higher inflammatory profile than oligo-JIA SFs. The high percentage of cells with MN may be related with SF genotoxic effects, and involved in cf-DNA release. All these events may induce the activation of inflammatory pathways that lead to disease complications such as uveitis.

**Patient Consent**

Not applicable (there are no patient data)

**Disclosure of Interest**

None declared

**References**

1. Baggio C, et al. Int J Mol Sci 2023;24:5450
2. Petty RE, et al. J Rheumatol 2004;31:390-2

## P286 Juvenile idiopathic arthritis (JIA) in the COVID-19 pandemic: has SARS-COV-2 infection modified the incidence of JIA in our pediatric rheumatology centre?

### F. Sciorio^1^, C. Stefani ^1^, A. Pietrobelli ^2^, C. Colombrino ^2^, L. Russo^2^, G. Orsingher^2^, S. Tagliaferri^2^, E. De Leo^2^, S. Pieropan^1^

#### ^1^Pediatric Rheumatology; ^2^University of Verona, Verona, Italy

##### **Correspondence:** F. Sciorio

*Pediatric Rheumatology 2023*, **21(Suppl 2):**P286

**Introduction:** Juvenile idiopathic arthritis is the most common chronic pediatric rheumatic disease. To date the mechanisms underlying the etiopathology are not clear, although there seems to be a genetic predisposition, influenced by various environmental factors, including infections.

**Objectives:** Provide an overview of the increased diagnoses of JIA in our pediatric rheumatology center during this COVID-19 pandemic, in order to evaluate the correlation between COVID-19 infection and the incidence of Juvenile Idiopathic Arthritis.

**Methods:** The medical history of 35 patients - diagnosed with JIA in our pediatric rheumatology center between 2018 and 2023 - was retrospectively reviewed. Current age, age at onset of symptoms, age at diagnosis, type of JIA, therapy and COVID-19 detected infection was evaluated. Two period groups according to the onset of the symptoms were evaluated: the first group included patients with symptoms that were already present between February 2018 and February 2020; in the second group the symptoms were present between February 2021 and February 2023.

**Results:** Among the 35 patients diagnosed with JIA, 13 patients (38%) experienced symptoms in the pre-pandemic period and were eventually diagnosed during the pandemic; twenty-two (62%) developed symptoms and were diagnosed during COVID-19 pandemic. In this group only one patient had had symptoms with a confirmatory positive molecular nasopharyngeal (NP) swab of COVID-19 infection. Limitations of the study is the small patient sample; molecular Sars-CoV-2 infection is difficult to rule out due to unavailability of historical NP swab testing and to subsequent anti-COVID-19 vaccination, although in Italy it is compulsory above 12 years of age and highly recommended between 6 and 12 years.

**Conclusion:** Our data showed an increase in diagnoses of JIA during this COVID-19 pandemic, revealing a suggestive possible correlation between the COVID-19 infection and incidence of JIA. Further studies are needed to identify the pathogenetic mechanisms underlying this correlation. Notwithstanding the promising results of our evaluation, it would be useful to continue the study prospectively, in order to prolong the clinical and laboratory follow up including regular NF swab testing in children with respiratory symptoms suggestive of Sars-CoV-2 infections.

**Patient Consent**

Not applicable (there are no patient data)

**Disclosure of Interest**

None declared

**References**

1. Kalinina Ayuso V, Makhotkina N, van Tent-Hoeve M, de Groot-Mijnes JD, Wulffraat NM, Rothova A, de Boer JH. Pathogenesis of juvenile idiopathic arthritis associated uveitis: the known and unknown. Surv Ophthalmol. 2014 Sep-Oct;59(5):517-31. doi: 10.1016/j.survophthal.2014.03.002. Epub 2014 Mar 29. PMID: 25130893.
2. Kaya Akca U, Atalay E, Cuceoglu MK, Balik Z, Sener S, Ozsurekci Y, Basaran O, Batu ED, Bilginer Y, Ozen S. Impact of the COVID-19 pandemic on the frequency of the pediatric rheumatic diseases. Rheumatol Int. 2022 Jan;42(1):51-57. doi: 10.1007/s00296-021-05027-7. Epub 2021 Nov 2. PMID: 34727197; PMCID: PMC8561687.
3. Berkun Y, Padeh S. Environmental factors and the geoepidemiology of juvenile idiopathic arthritis. Autoimmun Rev. 2010 Mar;9(5):A319-24. doi: 10.1016/j.autrev.2009.11.018. Epub 2009 Nov 22. PMID: 19932890.

## P287 Levels of homocysteine and vascular endothelial growth factor in adolescents with juvenile idiopathic arthritis

### N. S. Shevchenko^1,2^, T. O. Golovko^1,2^, L. F. Bohmat^2^, O. S. Pavlova^1^, Y. V. Volkova^3^, L. L. Sukhova^3^

#### ^1^Department of Pediatrics, V. N. Karazin Kharkiv National University; ^2^Department of Rheumatology and Comorbid Conditions; ^3^Laboratory of Hormonal-Metabolic and Immunological Research, S.I."Institute for Children and Adolescents Health Care of the National Academy of Medical Sciences of Ukraine", Kharkiv, Ukraine

##### **Correspondence:** N. S. Shevchenko

*Pediatric Rheumatology 2023*, **21(Suppl 2):**P287

**Introduction:** Endothelial dysfunction in the systemic inflammatory process is an important factor in the formation of irreversible changes in the vascular bed. This may be the basis of concomitant pathology of target organs, atherosclerosis, systemic fibrosis. Therefore, the study of markers of endothelial dysfunction is important for the early detection of comorbid pathology^1,2,3^.

**Objectives:** To study the level of homocysteine and VEGF in the blood of adolescents with juvenile idiopathic arthritis (JIA).

**Methods:** 74 adolescents aged 10 to 18 years old (mean age 13.51±0.33 years) were examined, 46 of them were girls, 28 were boys. 62 (83.78%) were diagnosed with JIA, polyarticular variant, 12 (16.22%) - JIA, oligoarticular variant. The duration of the disease was 66.58±5.18 months. All children received methotrexate (MTX) therapy for more than one year, the average dose was 11.10±0.46 mg/m^2^ per week. At the same time, folic acid was administered at an average dose of 9.34±0.37 mg/week. The comparison group consisted of 23 healthy peers aged 13.70±0.38 years. A study of homocysteine and VEGF in the blood was carried out by the method of competitive immunoassay using a Rayto RT-2100C analyzer, China. Statistical data processing was carried out using SPSS17 application programs (license 4-180844250981ae3dae-s/nSPSS17) on IBM PC Pentium-4.

**Results:** The level of homocysteine was 9.27±0.39 μmol/l, which was significantly lower than in the control group (11.05±1.13 μmol/l, p<0.001). In the group from 10 to 14 years, homocysteine was lower than in older patients (8.68±0.40 μmol/l vs. 10.18±0.74 μmol/l, p<0.03). Reliable dependence on the clinical manifestations of the disease, the dose of methotrexate and the duration of its administration has not been established. The serum VEGF level was 108.86±15.70 pg/ml, which was lower than in the control group (185.40±53.48 pg/ml, p<0.001). A correlation was established between VEGF and homocysteine (r = 0.47, p<0.03). There was no significant difference in VEGF depending on age (10-14 years old: 104.34±22.76 pg/ml; 15-18 years old: 112.49±21.43 pg/ml, p<0.2). The VEGF level correlated with activity indicators (JADAS-27: r = 0.42, p<0.05; ESR: r = 0.48, p<0.05; circulating immune complexes: r = 0.40, p<0.05).

**Conclusion:** In adolescents with JIA, indicators of endothelial dysfunction were found within the reference normal values and at a lower level than in healthy adolescents. The content of homocysteine mainly depended on age, VEGF depended on the severity of the inflammatory process.

**Patient Consent**

Not applicable (there are no patient data)

**Disclosure of Interest**

None declared

**References**

1. Kerekes G, Szekanecz Z, Dér H, Sándor Z, Lakos G, Muszbek L, Csipö I, Sipka S, Seres I, Paragh G, Kappelmayer J, Szomják E, Veres K, Szegedi G, Shoenfeld Y, Soltész P. Endothelial dysfunction and atherosclerosis in rheumatoid arthritis: a multiparametric analysis using imaging techniques and laboratory markers of inflammation and autoimmunity. J Rheumatol. 2008 Mar;35(3):398-406. Epub 2008 Jan 15. PMID: 18203326.
2. Ambrosino P, Lupoli R, Di Minno A, Tasso M, Peluso R, Di Minno MN. Subclinical atherosclerosis in patients with rheumatoid arthritis. A meta-analysis of literature studies. Thromb Haemost. 2015 May;113(5):916-30. doi: 10.1160/TH14-11-0921. Epub 2015 Feb 26. PMID: 25716931.
3. Yang, Zhicheng BA; Wang, Mingjie MA; Yan, Ting BA; Hu, Zhiyong MA; Zhang, Hui MA; Liu, Ruiping PhD∗. Association between vascular endothelial growth factor receptor 2 rs11941492 C/T polymorphism and Chinese Han patients in rheumatoid arthritis. Medicine 98(52):p e18606, December 2019. | DOI: 10.1097/MD.0000000000018606

## P288 Inosine-triphosphate-pyrophosphatase activity to predict remission with methotrexate in patients with juvenile idiopathic arthritis

### S. Sindici Forgiarini^1^, M. Lucafò^2^, D. Selvestrel^1^, V. Moressa^3^, S. Pagarin^1^, A. Tommasini^1,3^, M. Becker^4^, S. D. Thompson^5,6^, C. Langfeld^7^, E. Marrani^8^, G. Simonini^8^, G. Stocco^1,3^, G. Decorti^1^, A. Taddio^1,3^, S. Pastore^3^

#### ^1^Department of Medicine, Surgery and Health Sciences; ^2^Department of life Science, University of Trieste; ^3^Institute for Maternal and Child Health I.R.C.C.S. Burlo Garofolo, Trieste, Italy; ^4^Department of Pediatrics, Duke University School of Medicine, Durham; ^5^Division and Center for Autoimmune Genomics and Etiology, Cincinnati Children's Hospital Medical Center; ^6^Department of Pediatrics, University of Cincinnati College of Medicine, Cincinnati; ^7^Department of Biostatistics and Data Science, Wake Forest School of Medicine, Winston-Salem, United States; ^8^I.R.C.C.S Meyer Children’s University Hospital, Florence, Italy

##### **Correspondence:** S. Sindici Forgiarini

*Pediatric Rheumatology 2023*, **21(Suppl 2):**P288

**Introduction:** Methotrexate is the first-line therapy for juvenile idiopathic arthritis (JIA), but up to 45% of patients do not respond, leading to disease progression and joint damage. Recent studies have suggested that JIA patients with low inosine-triphosphate-pyrophosphatase (ITPA) activity do not achieve clinical remission (1).

**Objectives:** To investigate the association between ITPA activity and response to MTX therapy, assessed as clinical remission.

**Methods:** Patients with JIA from 3 major pediatric rheumatology hospitals (Trieste and Florence in Italy, Kansas City in USA) who fulfilled International League of Associations for Rheumatology criteria were enrolled and given weekly 10-15 mg/m2 of methotrexate, either orally or subcutaneously, for a minimum duration of 12 months. Demographic and clinical data of patients were collected at baseline, after 6 months of methotrexate therapy and subsequently every 3 months during the treatment period; minimum follow-up period for each patient was 12 months. Clinical remission was evaluated as 6 months remission period on methotrexate therapy only which begins within one year of starting therapy (2). ITPA activity was measured in patients’ erythrocytes with HPLC (3). Erythrocyte lysates were obtained cross-sectionally from venous blood samples collected during routine clinical care; for each patient, reported ITPA activity represents the average measurement at different time points during treatment. Statistical analysis of remission and ITPA activity association was done logistic regression analysis and non-parametric tests, adjusting for clinical center by multivariate logistic regression or meta-analysis combining results in each center.

**Results:** ITPA activity was measured in erythrocytes of 204 JIA patients (114 from Trieste, 26 from Florence, 64 from Kansas City). Patients with JIA who reach remission had higher ITPA activity than those who do not reach remission (mean±standard deviation = 53.9±19.3 nmol vs 45.8±18.0 nmol IMP/h respectively, p-value logistic regression: 0.005, p-value Wilcoxon test: 0.003). ITPA activity was different among hospitals for unclear reasons (mean±standard deviation, Trieste = 41.9 ± 19.3, Florence = 56.3 ± 16.2, Kansas city = 53.1 nmol ± 25.8 nmol IMP/h, p-value logistic regression = 0.00033, p-value Kruskal-wallis test = 4.59x10-5); however, ITPA activity was higher in patients in remission with methotrexate also adjusting for clinical center (p-value logistic regression adjusted for center = 0.00099; pvalue meta-analysis = 0.00018).

**Conclusion:** JIA patients with higher ITPA activity have increased probability of remission during therapy with methotrexate. Further studies are needed to identify a clinically useful cut-off and to identify genetic and non-genetic factors determining variability in ITPA activity.

**Patient Consent**

Not applicable (there are no patient data)

**Disclosure of Interest**

None declared

**References**

3. Pastore S, Stocco G, Moressa V, Zandonà L, Favretto D, Malusà N, et al. 5-Aminoimidazole-4-carboxamide ribonucleotide-transformylase and inosine-triphosphate-pyrophosphatase genes variants predict remission rate during methotrexate therapy in patients with juvenile idiopathic arthritis. Rheumatol Int. 2015;35(4):619-27.
4. Wallace CA, Ruperto N, Giannini E. Preliminary criteria for clinical remission for select categories of juvenile idiopathic arthritis. J Rheumatol. 2004;31(11):2290-4.
5. Shipkova M, Lorenz K, Oellerich M, Wieland E, von Ahsen N. Measurement of erythrocyte inosine triphosphate pyrophosphohydrolase (ITPA) activity by HPLC and correlation of ITPA genotype-phenotype in a Caucasian population. Clin Chem. 2006;52(2):240-7.

## P289 Urinary mirnas as non-invasive biomarkers for juvenile idiopathic arthritis

### A. Snipaitiene^1,2^, A. Slegeryte^3^, I. Juskeviciute^4^, B. Skerbaite^4^, B. Buragaite-Steponkiene^5^, K. Snipaitiene^4^, A. Baranauskaite^6^, S. Jarmalaite^4^, L. Jankauskaite^1,2^

#### ^1^Pediatric Department, Lithuanian University of Health Sciences; ^2^Pediatric Department, Hospital of Lithuanian University of Health Sciences Kauno Klinikos; ^3^Mediacal Academy, Lithuanian University of Health Sciences, Kaunas; ^4^Human Genome Research Group, Life Sciences Center, Vilnius University, Vilnius; ^5^Human Genome Research Group, Life Sciences Center, Vilnius University; ^6^Lithuanian University of Health Sciences, Kaunas, Lithuania

##### **Correspondence:** A. Snipaitiene

*Pediatric Rheumatology 2023*, **21(Suppl 2):**P289

**Introduction:** Despite known for several decades, juvenile idiopathic arthritis (JIA) is still diagnosis of exclusion. Previous studies have shown that serum and plasma non-coding RNAs, such as microRNAs (miRNAs), could serve as potential diagnostic tools of JIA inflammation. Data regarding miRNA detection in urine in JIA patients are lacking.

**Objectives:** We aimed to analyze miR-16, -146a, and -155 changes in JIA patients’ urine and serum and if they are linked to disease activity.

**Methods:** We performed prospective single center study. 31 children with JIA (excluding systemic JIA) and 24 healthy controls (HC) were enrolled. Serum and urine samples were collected every 3months for 1year. Complete blood count (CBC), erythrocyte sedimentation rate (ESR) and C-reactive protein (CRP) were analyzed. JIA disease activity was evaluated using JADAS27. All patients during first study visit (M0) were divided into active disease group (R0, n=18) and remission (R1, n=13). R0 group samples of month 12 of the study (M12) were compared to M0. miRNAs levels were evaluated using RT-qPCR. Data were analyzed with SPSS 29.0. P value <0.05 was considered significant.

**Results:** 90% (28/31) of JIA patients were female with mean age of 13.1 yrs (range 3-17 yrs). ESR levels tended to be higher in R0 compared to R1 and HC (9.18 mm/h vs 6.54 mm/h and 8.33 mm/h, respectively, p=0.326). No CBC or CRP differences were noted between the groups. Moreover, none of analyzed miRNAs were able to distinguish JIA from HC in serum. Levels of all analyzed miRNRs were significantly lower in urine of R1 and R0 group patients’ vs HC (miR-16 R0 and R1 *vs.* HC, p=0.027 and p=0.030, respectively; miR-146a R0 and R1 *vs.* HC, p=0.006 and p=0.006, respectively; miR-155 R0 and R1 *vs.* HC, p=0.010 and p=0.010, respectively). All miRNAs levels were significantly decreased in urine in Methotrexate or anti-TNF treated patients and miR-16 levels were significantly lower in serum of anti-TNF-treated group compared to HC (p=0.017). Interestingly, serum miR-155 was upregulated in M0 visit vs M12 (p=0.005), but without changes in urinary samples. However, serum miR-155 could discriminate the active disease vs remission with 77% sensitivity and 48% specificity (AUC=0.71; p=0.052). Body fluid comparison showed significantly higher levels of miR-146a in serum samples than in urine both in R0 and R1 compared to HC (p=0.025 and p=0.009, respectively), whereas higher levels of miR-155 were detected in serum (p=0.011).

**Conclusion:** Findings of the study suggest that miRNAs in urine could be useful non-invasive diagnostic markers for JIA. Further prospective studies are required to estimate miRNAs changes in different stages disease.

**Patient Consent**

Not applicable (there are no patient data)

**Disclosure of Interest**

None declared

## P290 Lipoma arborescens as a differential diagnosis of chronic monoarthritis of the knee

### F. Sztajnbok, M. R. Vasti, F. C. Zonis, M. F. Rodrigues, A. R. Fonseca, J. L. Monteiro, P. R. Souza, I. M. Paz, R. G. Almeida

#### Pediatric Rheumatology, Universidade Federal do Rio de Janeiro, Rio de Janeiro, Brazil

##### **Correspondence:** F. Sztajnbok

*Pediatric Rheumatology 2023*, **21(Suppl 2):**P290

**Introduction:** Lipoma arborescens (LA) is a rare cause of chronic monoarticular arthritis. It should be considered during investigation of oligoarticular juvenile idiopathic arthritis (JIA).

**Objectives:** To report the case of a teenage boy presenting with chronic monoarthritis of the knee undergoing investigation for JIA and tuberculous monoarthritis. The diagnosis of LA was based on magnetic resonance imaging (MRI) and histopathological study.

**Methods:** Case report and narrative literature review.

**Results:** A 12-year-old boy presented with a 2-year history of pain and progressive swelling of the left knee. No morning stiffness, nor impact in daily activities. No history of trauma or constitutional symptoms. He underwent multiple arthrocentesis for pain relief previous to pediatric rheumatologist referral.

On clinical examination, patient presented warm left knee, painless diffuse effusion and asymmetric length of legs. Laboratory tests showed no elevated inflammatory parameters, and negative antinuclear antibody (ANA), rheumatoid factor (RF) and HLA-B27. Purified protein derivative (PPD) skin test was 7 mm and normal chest x-ray. MRI showed marked effusion, synovial proliferation, post synovial fatty metaplasia, absence of bone erosions, subcortical bone edema in periphery of femoral condyles.

There was no response to treatment prescribed with nonsteroidal anti-inflammatory drugs. During follow up, needle aspiration was performed yielding 60 ml of aseptic citrine-colored fluid tested negative for Gene xpert and bacilloscopy. Histological assessment was performed after a synovial biopsy and diagnosis was consistent with LA.

**Conclusion:** Diagnostic of JIA requires ruling out other causes, including infections. Thorough investigation is mandatory in monoarthritis, including laboratory tests, imaging studies, synovial fluid analysis and biopsy in selected cases.

We describe the case aiming to raise suspicion of LA during pediatric rheumatology consultation. LA is a rare benign tumor characterized by villonodular proliferation of the synovium. There is fewer than 100 cases reported. It usually affects knee joint, in the suprapatellar pouch. Presents as slow progressive swelling, pain and effusion. Laboratory tests are normal. MRI is the imaging method of choice. Treatment is mostly by open or arthroscopic synovectomy with low risk of recurrence.

**Patient Consent**

Yes, I received consent

**Disclosure of Interest**

None declared

## P291 Adalimumab and anti-drug antibodies in a cohort of children with juvenile idiopathic arthritis: monocentric retrospective study

### G. Tarantino^1^, R. Simeoli^2^, E. Marasco^2^, A. Aquilani^1^, R. Nicolai^1^, B. M. Goffredo^2^, F. De Benedetti^1^, S. Magni Manzoni^1^

#### ^1^Rheumatology Division; ^2^Metabolic Disease Division, IRCCS, Bambino Gesù Children's Hospital, Rome, Italy

##### **Correspondence:** S. Magni Manzoni

*Pediatric Rheumatology 2023*, **21(Suppl 2):**P291

**Introduction:** Adalimumab (ADA), a fully humanized antibody against tumor necrosis factor (TNF)-α, has revolutionized the treatment of patients with juvenile idiopathic arthritis (JIA). Although most of these respond within the first weeks, a minority may show loss of response (LOR) after continued exposure. Many studies demonstrate the influence of anti-adalimumab antibodies (AAA) on serum drug concentrations and clinical outcome in adults. However, little information about AAA and LOR is available for children with JIA.

**Objectives:** To describe demographic and clinical features in a single-center cohort of JIA patients treated with ADA, grouped according to frequency and dosage of drug administration (1W vs 2W; 20 vs 40 mg); to assess ADA levels versus AAA titer and, finally, to investigate possible correlation between LOR and AAA.

**Methods:** Records of JIA patients on ADA treatment were retrospectively reviewed with focus on medical history and ELISA (enzyme-linked immunosorbent assay) ADA/AAA levels in a 3-years-period. Children with idiopathic uveitis and systemic JIA were excluded. Data were analyzed via descriptive statistics (STATA 15.1).

**Results:** From June 2019 to March 2023 we collected 416 ADA/AAA samples in 118 JIA patients treated with ADA (a median of 3.3/pt). Of them (65% females), 42 had ANA-positive oligoarthritis, 30 RF-negative polyarthritis, 11 HLA-B27 positive enthesitis-related arthritis and 8 psoriatic. The median age at disease onset was about 3.7 years. Half of study cohort was b-DMARDS naive at ADA start, while all children were on concomitant c-DMARDs (97% on Methotrexate). Chronic recurrent uveitis was the main reason for ADA starting, followed-by tenosynovitis, bowel inflammation and spine or hip active arthritis. The median disease duration at first sampling was 2.9 (IQ 1.1-5.4). Extreme variability was observed between AAA titers (median 4.2 AU/ml) and ADA levels (16.3 median micrograms/ml), regardless from 1 or 2-weekly administration and 20 or 40-mg dosage. Among both study groups, an inverse correlation between ADA and AAA was found. We identified 40 JIA patients with active disease (clinical JADAS-10 >1) or which flared on therapy (LOR), with knees or ankles as the most frequently involved. Among these in 35% we found very high AAA titers (105-1230 AU/mL). In AAA-positive patients (> 50 AU/ml) we observed higher Body Mass Index (BMI) and baseline C-reactive protein (CRP); lower albumin levels were, instead, associated with greater clearance of ADA.

**Conclusion:** Our preliminary “real life” data showed association between occurrence of AAA and lower ADA levels. A targeted risk analysis about high AAA titers and LOR incidence is pending. Monitoring of drug immunogenicity will be implemented in our daily practice and become subject of our future studies.

**Patient Consent**

Yes, I received consent

**Disclosure of Interest**

None declared

**References**

3. Skrabl-Baumgartner A., Erwa W., et al. “Anti-adalimumab antibodies in juvenile idiopathic arthritis: frequent association with loss of response.” Scand J Rheumatol 2015; 44:359–366.
4. Marino A., Real-Fernández F., et al. “Anti-adalimumab antibodies in a cohort of patients with juvenile idiopathic arthritis: incidence and clinical correlations.” Clin Rheumatol. 2018; 37:1407-1411.

## P292 Children with extended oligoarticular and polyarticular juvenile idiopathic arthritis have alterations in B and T follicular cell subsets in peripheral blood and a cytokine profile sustaining B cell activation

### C. Tomé^1^, F. Oliveira-Ramos^1,2^, R. Campanilho-Marques^1,2^, A. F. Mourão^3^, S. Sousa^4^, C. Marques^1^, A. T. Melo^1,2^, R. L. Teixeira^1,2^, A. P. Martins^5^, S. Moeda^6^, P. Costa-Reis^1,6^, R. P. Torres^3^, M. Bandeira^1,2^, H. Fonseca^6^, M. Gonçalves^5^, M. J. Santos^1,4^, L. Graca^1^, J. E. Fonseca^1,2^, R. A. Moura^1^

#### ^1^Instituto de Medicina Molecular João Lobo Antunes, Faculdade de Medicina, Universidade de Lisboa, Centro Académico de Medicina de Lisboa; ^2^Rheumatology Department, Centro Hospitalar Universitário Lisboa Norte, EPE, Hospital de Santa Maria, Centro Académico de Medicina de Lisboa; ^3^Rheumatology Department, Centro Hospitalar Lisboa Ocidental, EPE, Hospital de São Francisco Xavier, Lisbon; ^4^Rheumatology Department, Hospital Garcia de Orta, EPE, Almada; ^5^Pediatric Surgery Department; ^6^Department of Pediatrics, Centro Hospitalar Universitário Lisboa Norte, EPE, Hospital de Santa Maria, Centro Académico de Medicina de Lisboa, Lisbon, Portugal

##### **Correspondence:** F. Oliveira-Ramos

*Pediatric Rheumatology 2023*, **21(Suppl 2):**P292

**Introduction:** Juvenile idiopathic arthritis (JIA) is the most common rheumatic disease in children. Our group has recently demonstrated that extended oligoarticular and polyarticular JIA mostly evolve to a rheumatoid arthritis (RA)-like phenotype in adulthood. Disturbances in B cells, T follicular helper (Tfh) and T follicular regulatory (Tfr) cell immune responses are associated with RA pathogenesis, but their exact role in JIA development is poorly understood.

**Objectives:** The main goal of this study was to characterize the frequency and phenotype of B, Tfh and Tfr cells in peripheral blood and the cytokine environment present in circulation in children with with extended oligoarticular JIA (eoJIA) and polyarticular JIA (pJIA) when compared to healthy controls, children with persistent oligoarticular JIA (poJIA) and adult JIA patients

**Methods:** Blood samples were collected from 105 JIA patients (children and adults) and 50 age-matched healthy individuals. Peripheral blood mononuclear cells were isolated and the frequency and phenotype of B, Tfh and Tfr cells were evaluated by flow cytometry. Serum levels of APRIL, BAFF, IL-1β, IL-2, IL-4, IL-6, IL-10, IL-17A, IL-21, IL-22, IFN-γ, PD-1, PD-L1, sCD40L, CXCL13 and TNF were measured by multiplex bead-based immunoassay and/ or ELISA in all groups included.

**Results:** The frequency of B, Tfh and Tfr cells was similar between JIA patients and controls. Children with eoJIA and pJIA, but not poJIA, had significantly lower frequencies of plasmablasts, regulatory T cells and higher levels of Th17-like Tfh cells in circulation when compared to controls. Furthermore, APRIL, BAFF, IL-6 and IL-17A serum levels were significantly higher in pediatric eoJIA and pJIA patients when compared to controls. These immunological alterations were not found in adult JIA patients in comparison to controls.

**Conclusion:** Our results suggest a potential role and/or activation profile of B and Th17-like Tfh cells in the pathogenesis of eoJIA and pJIA, but not poJIA.

**Patient Consent**

Not applicable (there are no patient data)

**Disclosure of Interest**

None declared

## P293 Ultrasonographic evaluation of changes in the joint before and after intra-articular injection in children with juvenile idiopathic arthritis

### S. Türkmen, B. Sözeri

#### Clinic of Pediatric Rheumatology, Health Sciences University, Ümraniye Training and Research Hospital, İstanbul, Türkiye

##### **Correspondence:** S. Türkmen

*Pediatric Rheumatology 2023*, **21(Suppl 2):**P293

**Introduction:** Juvenile idiopathic arthritis (JIA) is the most common cause of chronic arthritis in childhood. If synovial hyperplasia is not treated in JIA, it causes to pannus formation and joint destruction. Intra-articular steroid (IAS) injection is one of the first treatment options in active oligoarticular JIA**.**

**Objectives:** The aim of this study is to compare ultrasonographic (US) synovial fluid grade change, synovial proliferation regression time and power Doppler (PD) changes at 2nd, 6th and 12th weeks in patients with oligoarticular JIA who were treated with only IAS and IAS plus systemic methotrexate.

**Methods:** In this study, 237 knee joints of 175 patients with oligoarticular JIA who were followed up in the Pediatric Rheumatology Clinic of the University of Health Sciences Ümraniye Training and Research Hospital between years of 2016 and 2022 were evaluated retrospectively. Grayscale (GS) and PD examinations were performed with a high frequency (10 – 12 megahertz) linear probe. Synovial fluid grade change, synovial proliferation in GS examination and PD change were evaluated separately for each joint before the treatment and at the 2nd, 6th and 12th weeks of the treatment. Synovitis was graded using a four-grade semi-quantitative scale. All of the patients were treated with either intra-articular administration of triamcinolone hexacetonide (1 mg/kg) alone or IAS plus systemic methotrexate. The results of patients who received only IAS treatment and those who received IAS plus systemic methotrexate therapy were compared at the 2nd, 6th and 12th weeks of treatment.

**Results:** 27.4% of 237 joints (n=65) were treated with IAS alone. The mean time to regression of synovial fluid, synovial proliferation, and PD in the IAS group was 5 ± 3,41, 5,43 ± 3,36, and 4,2 ± 2,77 weeks, respectively, while it was 6,88 ± 3,93, 7,01 ± 3,96, and 6,07 ± 3,92 weeks in the IAS plus methotrexate group, respectively (p=0.004, 0.018, 0.002, respectively**).** At the end of the 12th week in the IAS group, the synovial fluid grade regressed to 0 in 80% (n=52) of the joints, and 46% of this regression occurred in the first two weeks, whereas in the IAS plus methotrexate group, 75.5% of the joints (n=130) showed improvement. And 73.8% (n=96) of this improvement occurred after the first two weeks.

**Conclusion:** In this study, the combined use of IAS and methotrexate in the treatment of single joint at the early period in patients with oligoarticular JIA; it could not been shown to be superior to IAS treatment alone.

**Patient Consent**

Yes, I received consent

**Disclosure of Interest**

None declared

## P294 JIA-associated TMJ arthritis, idiopathic condylar resorption or anterior disc displacement – a care provider survey

### M. Twilt^1,2^, D. Sosna^1^, N. Pan^3,4^, S. Abramowicz^5,6^, M. Becker^7^, M. Lerman^8^, C. Resnick^9^, T. Ronis^10,11^, M. Stoll^12^, P. Stoustrup^13^ on behalf of CARRA Investigators and TMJaw

#### ^1^Pediatrics, Alberta Children's Hospital; ^2^Medicine, University of Calgary, Calgary, Canada; ^3^Hospital for Special Surgery; ^4^Weill Medical College of Cornell University, New York; ^5^Medicine, Emory University; ^6^Children's Healthcare of Atlanta, Atlanta; ^7^Medicine, Duke University, Durham; ^8^Children's Hospital of Philidelphia, Philadelphia; ^9^Boston Children's Hospital, Boston; ^10^Children's National Hospital; ^11^Medicine, George Washington University, Washington DC; ^12^Medicine, University of Alabama at Birmingham, Alabama, United States; ^13^Aarhus University, Aarhus, Denmark

##### **Correspondence:** M. Twilt

*Pediatric Rheumatology 2023*, **21(Suppl 2):**P294

**Introduction:** The temporomandibular joint (TMJ) can be affected in juvenile idiopathic arthritis (JIA) patients of any age or subtype. There have been reports of isolated TMJ arthritis. Idiopathic condylar resorption (ICR) and anterior disc displacement (ADD) also affect the TMJ and can be difficult to distinguish from JIA. The MRI appearance of active JIA-associated TMJ arthritis shows significant overlap with patients diagnosed with ADD. A Swiss study showed various degrees of inflammation with joint effusion, synovial thickening, and joint enhancement in both patients with JIA and ADD. Rheumatologists are increasingly asked to see patients with isolated TMJ problems. The confidence level of rheumatologists regarding differentiation between these different conditions is unclear. Interested researchers from the Temporomandibular Jaw working group (TMJaw) and the Childhood Arthritis Rheumatology Research Alliance (CARRA) TMJ interest group developed a survey addressing the confidence levels.

**Objectives:** To Survey pediatric rheumatologist (CARRA members) to assess the burden of isolated TMJ referrals and their confidence level in differentiating these conditions.

**Methods:** A survey was developed that had both closed and open-ended questions. This survey was distributed to a group of pediatric rheumatologist CARRA members. Participants were asked to identify areas of opportunity in this area including: Increasing awareness among rheumatologists on the different diagnosis, increasing awareness among radiologists, and development of algorithms to help assist to differentiate these conditions. Descriptive statistics were used.

**Results:** In total, 93 individuals participated in the survey, 94.5% (88) self-identified as pediatric rheumatologists, 5.5% (5) individuals were combined pediatric/adult rheumatologists. On average most of the participants see between 1-5 patients per year for the question of isolated JIA-associated TMJ arthritis. The majority of respondents rated themselves as somewhat confident in evaluating isolated TMJ concerns in children. Survey participants had seen both ICR and ADD patients and felt mostly minimally confident in differentiating between ICR and TMJ arthritis for both mimickers. The majority of participants stated that they have a team to discuss these cases with, most teams included a radiologist or oral and maxillofacial surgeon.

**Conclusion:** According to the results of this survey, pediatric rheumatologists have an overall level of discomfort regarding differentiation of TMJ mimickers, as many participants stated that they were minimally or somewhat confident in most circumstances at diagnosing these mimickers. However, many participants stated that they have assistance from a multidisciplinary team in aiding them with these diagnoses and mainly this includes a dedicated radiologist. These results highlight the need for additional research to help providers differentiate between these disparate TMJ conditions.

**Trial registration identifying number:** n/a

**Patient Consent**

Not applicable (there are no patient data)

**Disclosure of Interest**

None declared

## P295 Graves' disease and juvenile idiopathic arthritis, coexistence of autoimmune diseases: a case report

### G. A. Turrubiates-Hernández^1^, A. V. Villarreal-Treviño^2^

#### ^1^Pediatrics; ^2^Pediatric Rheumatology, Tecnológico de Monterrey, Monterrey, Mexico

##### **Correspondence:** A. V. Villarreal-Treviño

*Pediatric Rheumatology 2023*, **21(Suppl 2):**P295

**Introduction:** Juvenile Idiopathic arthritis (JIA) is a type of chronic arthritis that affects the synovium and tissues in joints. It develops in about 1 child in every 1000. Other autoimmune conditions such as autoimmune thyroid disease (AITD) are more common in JIA patients compared to the general population, however there are limited studies that approach this coexistence.

**Results:** A previously healthy 10 year- old girl presented to the general pediatrics consult with complaint of poor school and physical performance, palpitations and fatigue for about 6 months. Important family history included Hashimoto’s thyroiditis (mother), antiphospholipid syndrome (mother) and vitiligo (father). On physical examination goiter and tachycardia were found so she was referred to the endocrinology consult where she presented elevated free T3 (>20pg/ml), T3 (582.6 ng/dL) and decreased TSH (<0.05 μUI/mL). Thyrotropin receptor antibodies (TRAb) were positive (4.04 UI/L) and diagnosis of Graves' disease was made. About a week after, she developed joint pain in elbow, hip and knee, presenting to the rheumatology clinic with positive pullhair sign, goiter, exophthalmos and bilateral wrist, carpus and interphalangeal arthritis. Blood tests revealed normocytic normochromic anemia, elevated erythrocyte sedimentation rate and negative VDRL and direct Coombs tests; positive IgM reumathoid factor (100 U/mL) and reticular pattern antinuclear antibodies (1:320). Anti dsDNA, Sm, RNP, cardiolipin, beta2-gylcoprotein and lupus anticoagulant antibodies were all negative. Polyarticular AIJ diagnosis was made and treatment was started with methotrexate, hydroxychloroquine, folic acid and vitamin D3. She demonstrated a good clinical course presenting resolution of symptoms.

AITD have been described to be more common in JIA patients compared to the general population, specifically, female ANA positive JIA patients with positive family history are at increased risk of developing AITD. Since this relationship has not yet been fully studied, protocols that allow an adequate screening in patients with JIA have not been developed. The importance of raising awareness about the coexistence of these diseases relies in making an appropriate diagnosis to provide treatment on time.

**Conclusion:** Our case shows the importance of clinical suspicion of an autoimmune disease in a patient with one already diagnosed.

**Patient Consent**

Yes, I received consent

**Disclosure of Interest**

None declared

**References**

1. Bansal N, Pasricha C, Kumari P, Jangra S, Kaur R, Singh R. A comprehensive overview of juvenile idiopathic arthritis: From pathophysiology to management. Autoimmun Rev. 2023 Apr;22(7):103337.
2. van Straalen JW, Baas L, Giancane G, Grebenkina L, Brunner J, Vega-Cornejo G, et al. Juvenile idiopathic arthritis patients with positive family history of autoimmune thyroid disease might benefit from serological screening: analysis of the international Pharmachild registry. Pediatr Rheumatol Online J. 2023 Feb;21(1):19.

## P296 Troublesome connection. description of two patients with juvenile idiopathic arthritis (JIA) and Ataxia Telangiectasia (A-T)

### J. Wójtowicz^1,2^, A. Szczawińska-Popłonyk^3^, E. Pawlaczyk-Wróblewska^4^, I. Naishtetik^5^, P. Gietka^5^, B. Pietrucha^6^

#### ^1^Pediatric Rheumatology, REUMAPEDIC; ^2^Pediatric Rheumatology Ambulatory, National Institute of Geriatrics, Rheumatology and Rehabilitation, Warsaw; ^3^Department of Pediatric Pneumonology, Allergy and Clinical Immunology, Institute of Pediatrics; ^4^Department of Pediatric Endocrinology and Rheumatology, Institute of Pediatrics, Poznań University of Medical Sciences, Poznań; ^5^Department of Pediatric Rheumatology, National Institute of Geriatrics, Rheumatology and Rehabilitation; ^6^Department of Immunology, Children’s Memorial Health Institute, Warsaw, Poland

##### **Correspondence:** J. Wójtowicz

*Pediatric Rheumatology 2023*, **21(Suppl 2):**P296

**Introduction:** Ataxia telangictasia (A-T) belongs to primary immunodeficiency disorders with chromosomal reparation defects and therefore is associated with significant risk of infections and cancer.

**Objectives:** We describe cases of two girls, aged 4 and 8 years old, with A-T and concomitant juvenile idiopathic arthritis (JiA).

**Methods:** 1. CASE: The first patient with A-T diagnosed in the second year of life was referred to pediatric rheumatologist because of disabling swelling of the wrists. Tenosynovitis of the wrists and arthritis of both knees was found. Inflammatory parameters and complete cell count were normal. Deficit of IgA, IgG_2_ with higher concentrations of IgM were detected. Rheumatoid factor, cyclic citrullinated peptide antibodies, ANA and HLA-B27 antigen were negative. Infections were ruled-out. X-rays of the limbs and chest were not performed because of A-T. As a first line of JiA therapy injections of corticosteroids were performed and oral naproxen was recommended. Only a temporary improvement for about 3 months was achieved. Recurrence of tenosynovitis was confirmed. Due to A-T, oral hydroxychloroquine was ordered as a second-line treatment.

**Results:** 2. CASE: In the other patient, the diagnosis of JiA was established before A-T. At the age of 2, swelling and restricted mobility of the right index finger were observed. Tenosynovitis of that finger and arthritis of the metacarpophalangeal joint and both knee joints were found. ANA profile, rheumatoid factor were negative; HLA B27 was positive. Infections were ruled-out. The first-line therapy of JiA with oral naproxen, temporarily switched to ibuprofen, was recommended. Due to only partial remission, the second-line therapy was introduced with methotrexate for 18 months which led to an almost complete remission. In this patient, postural and walking instability, decreased muscle tone and dystonia, oculomotor apraxia, and dysarthria with high alpha-fetoprotein levels, lymphopenia, and hypogammaglobulinemia lead to diagnose A-T. At the age of 4, immunoglobulin replacement therapy was started. After 3 years, an infection-triggered exacerbation of JIA was observed and ibuprofen was indicated.

**Conclusion:** Only few cases of patients with A-T and JiA comorbidity were described before. Rheumatological management of these children could be difficult: ionizing imaging studies should be avoided and standard chemotherapeutic drugs, effective in JiA, raise concerns about their potential cancerogenecity and increased risk of severe infections. However, as both diseases may cause significant movement disability, treatment tailored for each A-T patient, but effective with JiA symptoms, could lead to better quality of life.

**Patient Consent**

Yes, I received consent

**Disclosure of Interest**

None declared

## P297 Long-term follow-up of juvenile oligoarthritis – what happens after 1st intra-articular corticosteroid injection?

### M. Zajc Avramovic^1,2^, N. Toplak^1,2^, S. Blazina^1,2^, G. Markelj^1,2^, N. Emersic^1,2^, T. Avcin^1,2^

#### ^1^University Children Hospital Ljubljana; ^2^University of Ljubljana, Medical faculty, Ljubljana, Slovenia

##### **Correspondence:** M. Zajc Avramovic

*Pediatric Rheumatology 2023*, **21(Suppl 2):**P297

**Introduction:** Juvenile idiopathic arthritis (JIA) combines all idiopathic arthritides in children, that last at least 6 weeks and have no other cause. In oligoarticular involvement, a common initial treatment is intra-articular corticosteroid injection.

**Objectives:** To evaluate the long-term outcomes of patients with oligoarticular JIA who received intra-articular corticosteroid injection as the first treatment for their disease. In addition, the parameters influencing different outcomes were investigated.

**Methods:** This was a retrospective study of consecutive children with idiopathic oligoarthritis lasting at least 6 weeks whose first treatment was an intra-articular injection of corticosteroids (IAC), conducted at the University Children Hospital Ljubljana, Slovenia from January 2005 to May 2023. Non-steroidal anti-inflammatory drugs were allowed before IAC. An exclusion criterion was systemic therapy at the time of or in the first 30 days after IAC. Data collected included demographics, ANA positivity, presence of HLA B27 antigen, number of affected joints, treatment data, and disease activity at the last follow-up. Log-rank (Mantel-Cox) survival analyses were performed to compare groups with different outcomes.

**Results:** 116 patients were enrolled in the study. The mean age at the time of IAC was 8.3 years (1.2-18.3) and the mean follow-up time was 4.3 years (7 months – 15.8 years). After the first IAC 40.0 % did not require further therapy. Only additional IAC was required in 15.5 %, which was performed in the mean time 2.0 years (4 months – 6 years). Systemic therapy was required in 44.0 % of patients, with 50 patients receiving methotrexate (MTX) and 1 patient receiving sulphasalazine. It was initiated on average 1.4 years (1 month - 12 years) after the first IAC. Biological therapy was initiated in 21.6 % of patients on average 2.5 years (3 months – 12 years) after the first IAC. At last follow-up, 89.7 % had inactive disease.

*ANA positivity.* Of 35.6% of patients who were ANA positive, 73 % required further therapy, 61 % required MTX and 31 % required biological therapy. Using the log-rank test (Mantel-Cox) of survival analysis ANA positivity was associated with the need for systemic therapy (P=0.040, chi square 4.22).

*HLA B27 antigen.* Of the 13.1% of patients who were HLA B27 positive, 80 % required further therapy, 67 % required MTX and 40 % required biological therapy. Using the log-rank test (Mantel-Cox) of survival analysis, HLA B27 antigen was associated with the need for systemic therapy (P=0.035, chi square 4.45).

**Conclusion:** Forty percent of children with oligoarticular arthritis requiring IAC did not need any further therapy or develop chronic rheumatic disease. ANA positivity and presence of HLA B27 were important predictors for those that required systemic therapy.

**Patient Consent**

Not applicable (there are no patient data)

**Disclosure of Interest**

None declared

## P298 Monoarticular juvenile idiopathic arthritis as a distinct clinical entity. A proof-of-concept study

### F. Zulian^1^, B. Pierobon^1^, M. E. Zannin^1^, C. Politi^2^, F. Vittadello^3^, A. Meneghel^1^, F. Tirelli^1^, G. Martini^1^

#### ^1^Rheumatology Unit, Department of Woman and Child Health, University Hospital of Padova; ^2^Forensic and Legal Medicine, University Hospital of Padova; ^3^Explora – Research and Statistical Analysis, Padova, Italy

##### **Correspondence:** F. Zulian

*Pediatric Rheumatology 2023*, **21(Suppl 2):**P298

**Introduction:** Currently, monoarticular Juvenile Idiopathic Arthritis (monoJIA) is included in the ILAR classification as oligoarticular subtype although various aspects, from clinical practice, suggest it as a separate entity.

**Objectives:** To describe the clinical characteristics of persistent monoJIA and to propose clinical criteria that may distinguish this subtype from the oligoarticular form.

**Methods:** Patients with oligoJIA and with at least two years follow-up entered the study. Those with monoarticular onset and persistent monoarticular course were compared with those with oligoJIA. Variables considered were: sex, age at onset, presence of benign joint hypermobility (BJH), ANA, uveitis, therapy and outcome. Patients who had not undergone clinical follow-up for more than 12 months were contacted by structured telephone interview.

**Results:** Of 347 oligo JIA patients followed for mean 11.4 years, 196 with monoarticular onset entered the study. A switch from monoarticular onset to oligoarticular course of 78 patients (39.8%) occurred by the first three years from onset. 118 patients (60.2%), identified as persistent monoJIA, were then compared with 229 oligoJIA. In comparison with oligoJIA, the most significant features of monoJIA were later age at onset (6.1 vs 4.7 years, p=0.002), lower female prevalence (70.3 vs 83.4%, p=0.004), higher frequency of BJH (77.7 vs 62.9%, p=0.006), lower frequency of uveitis (14.4 vs 34.1%, p=0.001) and ANA+ (68.6 vs 89.5%, p<0.001) and better long-term outcome (p<0.001).

**Conclusion:** This proof-of-concept study brings clear evidence that monoJIA, defined as persistent arthritis of unknown origin of a single joint for at least three years, presents distinctive clinical features and may be considered as a separate clinical entity from oligoJIA. Our observation may stimulate further studies and contribute to the large international debate that is attempting to develop a new classifcation system for JIA, more consistent with clinical reality.

**Patient Consent**

Yes, I received consent

**Disclosure of Interest**

None declared

**References**

1. Petty RE, et al. International League of Associations for Rheumatology classification of juvenile idiopathic arthritis: secondo revision, Edmonton, 2001*. J Rheumatol* 2004
2. Martini A. It is time to rethink juvenile idiopathic arthritis classification and nomenclature. *Ann Rheum* 2012

## P299 Worldwide assessment of clinical practice strategies (CLIPS) for systemic juvenile idiopathic arthritis and adult-onset still’s disease through the JIR-CLIPS network: a cost action

### F. Aguiar^1,2^, C. Girard-Guyonvarc’h^3^, Y. Vyzhga^4^, F. O. Ramos^5,6^, M. Jouret^7^, S. Hashad^8^, A. Lefèvre-Utile^9^, D. Hadef^10^, J. May^11^, J. P. Pantic^12^, J. M. Mosquera Angarita^13^, K. Daghor-Abbaci^14,15^, K. Pateras^16^, R. Guedri^17^, R. Faria^18^, C. Lanna^19^, S. Ozen^20^, S. Sahin^21^, S. Savic^22^, D. Foell^23^, M. Hofer^24,25^, S. Georgin-Lavialle ^26,27^, K. Theodoropoulou^28,29^ on behalf of JIR-CLIPS network

#### ^1^Pediatric Rheumatology and Young Adult Unit, Centro Hospitalar Universitário São João; ^2^Faculdade de Medicina da Universidade do Porto, Porto, Portugal; ^3^Division of Rheumatology, Department of Internal Medicine Specialties, Hôpitaux Universitaires de Genève, Genève, Switzerland; ^4^National Pirogov Memorial Medical University, Vinnytsya, Ukraine; ^5^Rheumatology Department and Pediatric Rheumatology Unit, Centro Hospitalar Universitário Lisboa Norte; ^6^Instituto de Medicina Molecular, Faculdade de Medicina da Universidade de Lisboa, Lisboa, Portugal; ^7^Reference center for Rare Auto-inflammatory Diseases and Amyloidosis, Le Chesnay-Rocquencourt, France; ^8^Tripoli Children´s Hospital, Tripoli, Libya; ^9^General Pediatrics and Pediatric Emergency Department, Jean Verdier Hospital, Bondy, France; ^10^Pediatrics Department, University of Batna 2, Batna, Algeria; ^11^Children's Hospital for Wales, Cardiff, United Kingdom; ^12^University of Belgrade, Belgrade, Serbia; ^13^Hospital Sant Joan de Déu, Esplugues de Llobregat, Spain; ^14^Internal Medicine Department, University of Algiers 1, Faculty of Medical Sciences; ^15^Biochemistry, Hemotology and Genetics Laboratory of Research, Bab El Oued University Hospital Center, Bab El Oued, Algeria; ^16^School of Medicine, Volos, Greece; ^17^Department of Pediatrics, Béchir Hamza Children’s Hospital of Tunis, Tunis, Tunisia; ^18^Centro Hospitalar e Universitário do Porto, Porto, Portugal; ^19^Locomotor Apparatus Department, Faculdade de Medicina da Universidade Federal de Minas Gerais, Belo Horizonte, Brazil; ^20^Division of Pediatric Rheumatology, Department of Pediatrics, Hacettepe University, Ankara; ^21^Pediatric Rheumatology Department, Istanbul University Cerrahpasa, Istanbul, Türkiye; ^22^Leeds Institute of Rheumatic and Muskuloskeletal Medicine, Leeds, United Kingdom; ^23^Department of Paediatric Rheumatology and Immunology, University Children's Hospital Münster, Münster, Germany; ^24^Pediatric Immuno-Rheumatology of Western Switzerland, Lausanne University Hospital, Lausanne; ^25^University Hospital, Genève, Switzerland; ^26^Internal Medicine Department, Hôpital Tenon, Sorbonne Université, Assistance Publique-Hôpitaux de Paris; ^27^French National Reference Center of Autoinflammatory Diseases and Inflammatory Amyloidosis, Paris, France; ^28^Pediatric Unit of Immunology, Allergology and Rheumatology, University Hospital of Lausanne; ^29^Department of Immunobiology, University of Lausanne, Lausanne, Switzerland

##### **Correspondence:** F. Aguiar

*Pediatric Rheumatology 2023*, **21(Suppl 2):**P299

**Introduction:** Juvenile Inflammatory Rheumatism (JIR) refers to a group of rare inflammatory diseases that present in childhood and adolescence but also sometimes later as part of a continuum from paediatric age to adulthood. Systemic juvenile idiopathic arthritis (sJIA) is a systemic inflammatory condition with joint involvement, which is classified within the spectrum of JIA. Adult-onset Still’s disease (AOSD) is considered the adult counterpart of sJIA. Despite the existence of evidence-based or consensus-based recommendations for the diagnosis and treatment of these diseases, their implementation in real-world scenarios is challenging.

**Objectives:** To gather real-life clinical practice strategies (CliPS) from physicians worldwide who treat sJIA/AOSD, with the goal of creating appropriate management plans and thereby supporting physicians in decision-making processes.

**Methods:** We selected five diseases to be the subject of the questionnaires, including sJIA/AOSD. The project was accepted for funding as a COST (European Cooperation for Science and Technology) action. Since September 2022, the online questionnaires have been distributed through different channels such as the JIRcohort network, national societies and networks and are still available online. An interim analysis of the answered questionnaires was conducted in March 2023.

**Results:** One hundred and fourteen physicians from 16 different countries answered to sJIA/AOSD questionnaire. The majority of responses were from physicians originating from Brazil (22%) and Turkey (16%). Most of the participants were paediatric physicians (71%). Approximately 45% of the respondents had more than 10 years of experience in managing these diseases, while 32.5% had less than 5 years of experience. Almost all participants declared using international criteria to support the diagnosis of sJIA/AOSD. The most commonly preferred first-line treatment were corticosteroids (CCT) (75%), followed by non-steroidal anti-inflammatory drugs (NSAIDs) (13%) and anti-interleukin (IL)-1 therapies (9%). For patients with mainly articular manifestations, the most used first-line treatments were CCT followed by methotrexate. For those presenting mainly with systemic manifestations, the first-line treatment of choice was CCT, followed by IL-1 blocking agents. About 69% of physicians considered a reduction of disease modifying anti-rheumatic drugs (DMARDs) after 6 to 12 months of disease remission.

**Conclusion:** The experience level of the participants was relatively well distributed. CCT are commonly used, whatever the disease manifestations. Conventional DMARDs and biologics are often considered. However, to better evaluate differences in real-life CLiPS, the questionnaires must be spread to more countries, and adult physicians should be encouraged as well, namely those who treat patients after transition or with adult-onset disease.

**Patient Consent**

Not applicable (there are no patient data)

**Disclosure of Interest**

None declared

## P300 Clinical usefulness of serum interleukin-6 levels as a marker of disease activity in patients with systemic juvenile idiopathic arthritis during tcz treatment

### Y. Aida, M. Mizuta, Y. Nakagishi

#### Department of Pediatric Rheumatology, Hyogo Prefectural Kobe Children’s Hospital, Kobe, Japan

##### **Correspondence:** Y. Aida

*Pediatric Rheumatology 2023*, **21(Suppl 2):**P300

**Introduction:** Systemic-onset juvenile idiopathic arthritis (s-JIA) is a refractory inflammatory disease that presents with fever, skin rash, and arthritis, and inflammatory cytokines such as interleukin (IL)-18, and IL-1β are known to be closely involved in the pathogenesis. Tocilizumab (TCZ), an anti-IL-6 receptor antibody, has been widely reported to be useful in the treatment of s-JIA. However, TCZ often masks these clinical symptoms and inflammatory response at disease flare and some infections. Therefore, it is difficult to measure the disease activity in patients with s-JIA during TCZ treatment. IL-6 is also known to be the key cytokine in the pathophysiology of s-JIA. This cytokine has been able to be measured in many hospitals all over the world after the COVID-19 pandemic. Moreover, serum IL-6 levels might be a useful tool for many inflammatory diseases in clinics because it is immediately measured.

**Objectives:** The aim of this study is to assess whether serum IL-6 levels as a useful disease marker in patients with s-JIA during TCZ treatment.

**Methods:** We measured serum IL-6 levels in 19 patients with s-JIA, who started to use TCZ in 2008-2016 in Hyogo Prefectural Kobe Children’s Hospital and investigated the clinical significance of serum IL-6 levels for differentiating active, inactive, and infection phase in s-JIA patients with TCZ treatment. Serum IL-6 levels were measured using the chemiluminescent enzyme immunoassay method.

**Results:** Serum levels of IL-6 were significantly elevated in active phase (median [range]: 283.3 [23.5–767.9] pg/mL) compared to those in inactive phase (median [range]: 13.0 [0.0–34.3] pg/mL), and infection phase (median [range]: 87.9 [11.9–526.3] pg/mL) under TCZ treatment. The optimal cut-off values were 147.0 pg/mL (AUC 0.7388) and 43.80 pg/mL (AUC 0.9948), respectively, based on ROC analysis. The kinetics of serum IL-6 levels were correlated to disease course in patients with s-JIA for a long period.

**Conclusion:** Serum IL-6 levels can be a useful disease marker in s-JIA patients during TCZ treatment.

**Patient Consent**

Yes, I received consent

**Disclosure of Interest**

None declared

## P301 "Characteristic phenotypes of JIA patients in a single tertiary hospital in Saudi Arabia and the effectiveness of the treatment"

### M. S. AlSwealh^1^, Y. O. Bahawi^1^, F. S. Aloufi^1^, M. A. Muzaffer^2^, M. A. Nashawi^2^

#### ^1^Faculty of Medicine; ^2^Pediatric Department, king Abdulaziz University, JEDDAH, Saudi Arabia

##### **Correspondence:** M. S. AlSwealh

*Pediatric Rheumatology 2023*, **21(Suppl 2):**P301

**Introduction:** Juvenile idiopathic arthritis (JIA) is a prevalent chronic rheumatological disease that affects individuals under 16 years of age [1]. The most common subtype observed in Saudi Arabian studies is systemic-onset JIA (sJIA), which is characterized by symptoms such as fever, arthritis, skin rash, and organ involvement [2,3].

**Objectives:** To describe the spectrum of phenotypes at the time of diagnosis of systemic-onset Juvenile Idiopathic Arthritis (sJIA) in our center, and to examine the various treatments received by the patients along with their outcomes after 12 months from diagnosis.

**Methods:** This study conducted a retrospective chart review to analyze the medical records of children with systemic-onset Juvenile Idiopathic Arthritis (JIA) at King Abdul Aziz University Hospital in Jeddah, Saudi Arabia, covering the period from 2013 to 2022.

**Results:** The results of our study show that 21 patients (11 females) were diagnosed with systemic-onset JIA at an average age of 6 years. Only 66% of the patients exhibited the typical triad of symptoms at diagnosis, and macrophage activating syndrome was observed in only one patient.

Regarding the initial treatment, 50% of the patients (9 out of 17) received steroids. Among them, 5 patients were treated with steroids alone, 4 patients received a combination of steroids and MTX, one patient received MTX along with Adalimumab, and 2 patients were treated with MTX in conjunction with tocilizumab. Additionally, 2 patients received only MTX, and one patient was administered Anakinra.

After 1 year, it was observed that 30% of the patients were still on steroids. Five patients were receiving methotrexate and were in remission, with 3 of them also using Anakinra, and the remaining 2 receiving tocilizumab alongside methotrexate.

**Conclusion:** Our study includes a small cohort of patients from a single tertiary center, demonstrating similar clinical presentation features to those observed in other countries. However, our findings indicate a suboptimal treatment response compared to other countries. It is important to consider the influence of patient ethnicity on treatment response, alongside factors such as treatment adherence and delayed adjustments, given the retrospective nature of the data.

**Patient Consent**

Not applicable (there are no patient data)

**Disclosure of Interest**

None declared

**References**

1. Ravelli A, Schiappapietra B, Verazza S, Martini A. Juvenile idiopathic arthritis. In: The Heart in Rheumatic, Autoimmune and Inflammatory Diseases. 2017 Jan 1;167-187. Academic Press.
2. Al Marri M, Qari A, Al-Mayouf SM. Juvenile idiopathic arthritis in multiplex families: longitudinal follow-up. Int J Rheum Dis. 2017 Jul;20(7):898-902.
3. Lee JJ, Schneider R. Systemic juvenile idiopathic arthritis. Pediatr Clin. 2018 Aug;65(4):691-709.

## P302 Subclinical lung involvement in systemic juvenile idiopathic arthritis: a tertiary care, single-center experience

### F. Anselmi^1^, F. Annunziata^1^, M. Borrelli^2^, F. Imoletti^1^, C. Cimbalo^2^, R. Naddei^1^, M. Alessio^1^

#### ^1^Department of Translational Medical Sciences- Pediatric Rheumatology unit; ^2^Department of Translational Medical Sciences- Pediatric Pneumology unit, "Federico II"University, Naples, Italy

##### **Correspondence:** F. Anselmi

*Pediatric Rheumatology 2023*, **21(Suppl 2):**P302

**Introduction:** Systemic Juvenile Idiopathic Arthritis-Lung Disease (sJIA-LD) is an emerging complication of sJIA characterised by chronic pulmonary disorders, such as pulmonary hypertension,interstitial lung disease and alveolar proteinosis. So far several risk factors (RF) have been described for sJIA-LD,including early disease onset(<2 years),macrophage activation syndrome (MAS) and a history of adverse reactions to biological drugs(ARBD).Given the increasing incidence of sJIA-LD,many Authors suggest to assess pulmonary function in children with sJIA as well as to identify and monitor those who present RF.

**Objectives:** Primary aim was to assess pulmonary involvement (PI) through non-invasive tests and to investigate RF for sJIA-LD in our cohort of patients with sJIA. Secondary aim was to evaluate the effectiveness of these tests in the initial assessment of PI in sJIA.

**Methods:** We included patients followed for sJIA(>6 years old) from March,2003 to March,2023.Exclusion criteria were a positive history of cardiopulmonary disease of other aetiology, a preliously diagnosed sJIA-LD and the presence of musculoskeletal conditions impairing the test performances. We collect demographic and clinical data, including the presence of RF for sJIA-LD..We assess cardio-pulmonary function using spirometry, transthoracic echocardiography(TE), the Six Minute Walking Test(6MWT) and the Saint George's respiratory questionnaire (SGRQ) for respiratory symptoms.

**Results:** 20/39 of our patients with sJIA fulfilled the inclusion/exclusion criteria. Analysis of RF revealed a early disease onset in 30% of patients,history of MAS in 35% and ARBD in 15%.10%of patients showed a pathological SGRQ final score;30% showed an impaired 6MWT performance (<25°percentile);3patients showed an impaired spirometry (FEV1/FVC ratio<83%in 2patients and a reduction of both FEV1and FVC<80%in1patient).Right ventricular systolic pressure at TE was normal in all patients. The average value of FEV1 resulted significantly lower in patientswith a history of MAS (94.2±9.3vs107.6±11,p<0.05) and in those with ARDB (92.2±4.2vs104.4±12,p<0.05).

**Conclusion:** None of our patients showed features of sJIA-LD on the non-invasive cardio-pulmonary assessment,although spirometry showed a significant reduction of FEV1 in patients with history of MAS and ARBD. This results may be due to the small sample size,as well to the possible inadequacy of the tests in the early LD assessment. Further studies are needed to identify the effective tools in subclinical sJIA-LD detection,as well as to assess individual RF for sJIA-LD.

**Patient Consent**

Yes, I received consent

**Disclosure of Interest**

None declared

## P303 What kind of features of systemic juvenile idiopathic arthritis are associated with lung involvement: the preliminary data of retrospective cohort study

### K. Belozerov^1^, E. Isupova^1^, N. Abramova^2^, V. Masalova^1^, E. Gaidar^1^, M. Kaneva^1^, T. Kornishina^1^, I. Chikova^1^, O. Kalashnikova^1^, L. Sorokina^1^, V. Chasnyk^1^, M. Kostik^1^

#### ^1^Hospital Pediatric; ^2^of Anesthesiology-Resuscitation, Saint Petersburg State Pediatric Medical University, Saint-Petersburg, Russian Federation

##### **Correspondence:** K. Belozerov

*Pediatric Rheumatology 2023*, **21(Suppl 2):**P303

**Introduction:** Patients with systemic juvenile idiopathic arthritis has the spectrum of respiratory symptoms: cough, dyspnoe, pleurisy, interstitial lung disease - ILD (acute and reversible and chronic progressive) and pulmonary arthery hypertension. Previous studies have shown the association of chronic ILD with macrophage activation syndrome (MAS)

**Objectives:** to find the predictors, associated with respiratory involvement (RI) in sJIA

**Methods:** In the retrospective cohort study the onset characteristics of 207 sJIA patients were included. Patients were divided in two groups with and without RI (see above mentioned variants)

**Results:** RI was in 51 cases and strongly associated with features of MAS:

SJIA with RI (n=51) // without RI (n=156)

- Hemoglobin, g/l : 90 (80; 106) // 103 (91; 113) - p 0.0006
- WBC, х10^9^/l: 17.0 (6.1; 26.5) // 14.6 (9.3; 22.0) - p 0.807
- Platelets, х10^9^/l: 211 (87; 530) // 438 (312; 587) - p 0.0004
- CRP, mg/l: 102 (33; 154) // 62 (24; 118) - p 0.097
- LDH, U/l: 725 (317; 2007) // 449 (255; 626) - p 0.001
- Ferritin, ng/ml: 1848 (748; 10948) // 380 (135; 1500) - p 0.00001
- Prothrombin, %: 75 (63; 93) // 89 (78; 103) - p 0.006
- Fibrinogen, g/l: 2.0 (1.0; 4.5) // 5.0 (3.0; 6.0) - p 0.000002
- HScore, points: 155 (98; 222) // 91 (56; 130) - p 0.000001
- HScore, %: 26 (1; 97) // 1 (1; 7) - p 0.000002

RI in sJIA was associated more often with MAS (OR=7.4 [3.3-16.6]; p=0.000002), heart involvement (OR=7.4 [3.6-15.2]; p=0.0000001), concomitant sepsis (OR=26.3 [3.1-219.6]; p=0.00002).

**Conclusion:** The identified predictors of RI in soJIA have to check to evaluation of prognosis and choosing treatment plans.

**Patient Consent**

Yes, I received consent

**Disclosure of Interest**

None declared

**References**

1. Schulert GS, Yasin S, Carey B, et al. Systemic Juvenile Idiopathic Arthritis-Associated Lung Disease: Characterization and Risk Factors. *Arthritis Rheumatol*. 2019;71(11):1943-1954. doi:10.1002/art.41073
2. Saper VE, Chen G, Deutsch GH, et al. Emergent high fatality lung disease in systemic juvenile arthritis [published correction appears in Ann Rheum Dis. 2022 Feb;81(2):e35]. *Ann Rheum Dis*. 2019;78(12):1722-1731. doi:10.1136/annrheumdis-2019-216040

## P304 Portrait of a patient with systemic juvenile idiopathic arthritis receiving biological therapy in the russian federation: federal register data

### M. Botova^1^, E. Alexeeva^1,2^, T. Dvoryakovskaya^1,2^, O. Lomakina^1^, A. Fetisova^1^, K. Isaeva^1^, A. Chomakhidze^1^, K. Chibisova^1^, I. Tsulukiya^1^, I. Kriulin^1,2^, E. Krekhova^1^, M. Shingarova^1,2^, N. Kondrateva^1^, T. Kriulina^1,2^, M. Kokina^1,2^

#### ^1^Rheumatology, National Medical Research Center of Children's Health; ^2^Pediatric, Sechenov First Moscow State Medical University, Moscow, Russian Federation

##### **Correspondence:** M. Botova

*Pediatric Rheumatology 2023*, **21(Suppl 2):**P304

**Introduction:** Systemic juvenile idiopathic arthritis (sJIA) is the rarest variant of juvenile idiopathic arthritis, characterized by severe course, frequent exacerbations, the development of life-threatening extra-articular manifestations and complications, which requires the use of expensive medications and frequent hospitalizations of patients. In the Russian Federation (RF), the provision of medicines to patients with sJIA is carried out at the expense of the federal budget, in this regard: the Federal Register (FR) of sJIA was created in 2018.

**Objectives:** To analyze clinical features, type of biologic therapy in patients with sJIA in RF according to the data from the FR.

**Methods:** Retrospective analysis of medical data of patients receiving biologic therapy with sJIA included in the FR of the RF. The analysis included patients receiving biologic agents: tocilizumab, canakinumab, etanercept, adalimumab.

**Results:** The total number of patients in the FR is 2002, among them – 928 receiving biological therapy. The study included 928 patients: females 504 (54,3%) and males 424 (45,7 %). The FR contains data of children 684 (73,7%) and adults 244 (26,3%), who were diagnosed sJIA in childhood. The analysis of the age of the onset of the disease showed that 472 (50,8 %) the patient became ill at the age of 5 years, of which 150 (16,1 %) - up to 2 years. In 263 (28,3 %) of patients onset of the disease were from 5 to 10 years, in 193 (20,7%) - at the age of over 10 years. The median age of the onset of the disease was 4,88 (IQR 2,5;9,0; min 0,17; max 17,3) years. All children had febrile fever, 789 (85,02%) –rash; 398 (42,8 %) - lymphadenopathy, 627 (67,5 %) – hepatomegaly, 518 (55,8 %) – splenomegaly, 244(26,2 %) – serositis. Active arthritis at the onset of the disease developed in 840 (90,5%) children. The Macrophage activation syndrome (MAS) was observed in 273 (29,4%). Among them MAS was observed at the onset of the disease in 158 (57,8%) patients. The average duration of the period between the onset of the disease and the verification of the diagnosis was 2,93 (IQR 1,33;7,23; min 0,2; max 229,16) months . The largest number of patients is observed in Moscow – 75 (8%).We analyzed drug therapy among of them. The 48 (63,1%) patients receive monotherapy of biological agents; 6 (7,8%) - combination of biologic drug with glucocorticosteroids (GCs); 19 (25,3%) - with DMARDs; 4 (5,2%) - with GCs and DMARDs. Among biologic therapy: 58 (76,3%) tocilizumab, 13 (17,3%) canakinumab, 4 (5,2%) etanercept. All patients now are at the stage of remission according C. Wallace criterias.

**Conclusion:** Due to to the Federal Register, all patients are provided with medicines. The creation of the Federal Register makes it possible to evaluate clinical data, the effectiveness and safety of the therapy provided in real clinical practice.

**Patient Consent**

Not applicable (there are no patient data)

**Disclosure of Interest**

None declared

## P305 Familial systemic juvenile idiopathic arthritis: think of a monogenic form

### K. Bouayed^1,2^, Y. Benchakroun^1^, A. Sakhi^1,2^

#### ^1^Pediatric Rheumatology Department, Hôpital Mère Enfant A. Harouchi, CHU Ibn Rochd; ^2^Faculté de médecine et de pharmacie, Université Hassan 2, Casablanca, Morocco

##### **Correspondence:** K. Bouayed

*Pediatric Rheumatology 2023*, **21(Suppl 2):**P305

**Introduction:** Systemic Juvenile Idiopathic Arthritis sJIA is one of the most severe childhood inflammatory diseases. It is a subtype of JIA that differs from others subtypes by the predominance of systemic features and by the treatment based on Interleukine 1 and 6 blockers labelling this form an auto-inflammatory condition by some authors. Auto-inflammatory diseases AIDs are well know to have a genetic cause, but the origin of sJIA remains a subject of controversies with arguments for both auto-immune and auto-inflammatory diseases AIDs. Nevertheless, A genetic background has been reported with homozygous mutation of LACC1 gene occurring with familial forms.

**Objectives:** To emphasize the hypothesis of an autosomal recessive form in familial forms of sJIA involving more than one sibling.

**Methods:** Retrospective and prospective case report about 6 siblings from 3 consanguineous families. with sJIA diagnosed according to International League Against Rheumatism « ILAR » criteria. All families gave the authorization for publication. The pedigree was done for all patients.

**Results:** 6 children from consanguineous families were identified., without any history of auto-immune or Autoinflammatory disease in the ascendents. All have the same geographic origin with sex ratio of 1. All patients started their disease before the age of 2 years with a marked inflammatory syndrome. 4/6 required an agressive treatment with biotherapy but only the more recent patients (N=3) could benefit from the adjonction of Tocilizumab. The remaining patients were treated with corticosteroids, non steroidal anti-inflammatory drugs and methotrexate. All patients achieved a complete remission leading to stop medication except one who was the last patient enrolled with an outcome of 18 months and is still being treated with methotrexate and NSAIDs on demand. Our patients could not benefit from genetic analysis because its unavailability in our country.

**Conclusion:** Our cases contribute to highlight the hypothesis of a genetic background as seen in other conditions of autoinflammatory diseases. On behalf these observations, we propose factors suggesting a genetic background of SJIA including consanguinity and early onset of the disease. Furthermore, our patients had a wide phenotype, ranging from mild to very severe disease. The severity of the disease would therefore be independent of the association with a potential mutation. Our goal is to perform genetic testing in these families in order to search for the LACC 1 mutation, which is the only one described so far associated with sJIA, or may be to discover a new mutation.

**Patient Consent**

Yes, I received consent

**Disclosure of Interest**

None declared

**References**

1. Wakil SM, Monies DM, Abouelhoda M, Al-Tassan N, Al-Dusery H, Naim EA, Al-Younes B, Shinwari J, Al-Mohanna FA, Meyer BF, Al-Mayouf S. Association of a mutation in LACC1 with a monogenic form of systemic juvenile idiopathic arthritis. Arthritis Rheumatol. 2015 Jan;67(1):288-95. doi: 10.1002/art.38877
2. Kallinich T, Thorwarth A, von Stuckrad SL, Rösen-Wolff A, Luksch H, Hundsdoerfer P, Minden K, Krawitz P. Juvenile arthritis caused by a novel FAMIN (LACC1) mutation in two children with systemic and extended oligoarticular course. Pediatr Rheumatol Online J. 2016 Nov 24;14(1):63.

## P306 HLA-DRB1*15 alleles in systemic juvenile idiopathic arthritis with lung disease and macrophage activation syndrome in Italy

### C. Bracaglia^1^, M. Pardeo^1^, M. Troiano^2^, G. Testa^2^, I. Caiello^1^, A. De Matteis^1^, M. Trevisan^1^, F. Locatelli^3^, M. Andreani^2^, F. De Benedetti^1^

#### ^1^Division of Rheumatology; ^2^Laboratory of Transplant Immunogenetics, Department of Haematology/Oncology, Cell and Gene Therapy; ^3^Department of Hematology/Oncology, Cell and Gene Therapy, IRCCS Ospedale Pediatrico Bambino Gesù, Roma, Italy

##### **Correspondence:** C. Bracaglia

*Pediatric Rheumatology 2023*, **21(Suppl 2):**P306

**Introduction: Systemic Juvenile Idiopathic Arthritis (**sJIA) is characterized by unique clinical features and it is considered as a polygenic inflammatory disease. **Macrophage Activation Syndrome (**MAS) and lung disease (LD) are two life threatening complications of sJIA. An association of HLA-DRB1*15 alleles with sJIA complicated by LD has been recently reported.

**Objectives:** To evaluate the HLA-DRB1*15 allele frequency in a cohort of sJIA patients.

**Methods:** DNA was extracted by automatic system Qiagen EZ1 Advanced XL from blood samples collected from sJIA patients followed in a single Pediatric Rheumatology tertiary center. The AllType NGS 11-Loci Amplification Kit (Thermo Fisher, One Lambda, Canoga Park, CA) was used to amplify target DNA regions. The PCR cycling conditions were followed as suggested by the manufacturer. The kit uses one multiplexed PCR amplifying the full HLA-A/B/C/DQA1/DPA1 genes and exon 2 to 3’UTR for HLA-DRB1/3/4/5/DQB/ DPB1 genes. Reads were analyzed with the HLA TypeStream VisualTM Software (Thermo Fisher, One Lambda, Canoga Park, CA), version 2.0.0. The IPD-IMGT/HLA Database release used was the 3.41. The strength of association between HLA alleles was estimated by 99% confidence intervals.

**Results:** Samples were collected from 98 sJIA patients, 52 females, all White Caucasian, except of 2 of African origin (1 from North-Africa and 1 from Sub-Saharan Africa) and 1 of mixed origin (Caribbean and Caucasian), with median age at disease onset of 6.9 years. The HLA-DRB1*15 allele was found in 20 (20%) out of 98 sJIA patients with a median age at disease onset of 5.4 years. These results were compared with a reference group of 1017 healthy Italian individuals, previously typed in our laboratory and representative of HLA allele frequency distribution in Italy. Eighteen, out of the 196 different HLA haplotypes observed in the patients studied, were positive for HLA-DRB1*15:01 (9.1%) compared to 94, out of 2034 (4.6%) in the healthy group with a *p* value of 0.006. However, using the Bonferroni’s correction for multiple tests, the significant association was lost (*p*=0.14), probably due to the small sample size. Of those 20 HLA-DRB1*15 positive patients, 7 (35%) had refractory sJIA, as defined by Erkens (1), compared to 14 (17%) of the HLA-DRB1*15 negative patients. Five out of 20 (25%) HLA-DRB1*15 positive patients had LD compared to 2 (3%) of HLA-DRB1*15 negative patients. Furthermore, 12 out 20 (60%) HLA-DRB1*15 positive patients had one or more MAS episode compared to 41 (52%) HLA-DRB1*15 negative patients. Only 4 patients experienced a drug adverse reaction, 3 to tocilizumab and 1 to anakinra. The 3 patients who had reaction to tocilizumab carried the HLA-DRB1*15 allele and had LD. The lung involvement in these 3 patients developed before the drug reaction. The patient who had a reaction to anakinra was negative for HLA-DRB1*15 and he did not develop lung disease (3 years follow-up).

**Conclusion:** The HLA-DRB1*15 allele seems to be more frequently carried by sJIA patients compared to healthy Italian population and therefore might be identified as a potential marker of susceptibility to the disease. This allele appears to be more frequent in a subgroup of sJIA patients with early disease onset, refractory course and with lung involvement. These data are of course limited to a small population and need to be confirmed in a larger international and multiracial cohort.

**Patient Consent**

Yes, I received consent

*This project has been funded by the systemic JIA Foundation*

**Disclosure of Interest**

C. Bracaglia Consultant with: Sobi, Novartis, M. Pardeo Consultant with: Sobi, M. Troiano: None declared, G. Testa: None declared, I. Caiello: None declared, A. De Matteis: None declared, M. Trevisan: None declared, F. Locatelli Consultant with: Sobi, M. Andreani: None declared, F. De Benedetti Consultant with: Abbvie, Sobi, Novimmune, Novartis, Roche, Pfizer.

**References**

1. Erkens R. et al, Rheum Dis Clin North Am. 2021 Nov; 47(4):585-606.

## P307 Safety and efficacy of concomitant use of anti IL17 and anti IL6 agents in a case of severe refractory systemic JIA: report of the first 18 months of treatment

### E. A. Conti, F. Chironi, A. Petaccia, S. Lanni, F. Minoia, G. Filocamo

#### IRCCS Ospedale Maggiore - Policlinico di Milano, Milan, Italy

##### **Correspondence:** E. A. Conti

*Pediatric Rheumatology 2023*, **21(Suppl 2):**P307

**Introduction:** In the last decade therapeutic options for systemic juvenile idiopathic arthritis (sJIA) increased and new treatment strategies based on early treatment and treat-to target approach were developed, leading to an impressive improvement in the care of this condition. However, a sizable number of sJIA patients still face a refractory course with a severe impact on their quality of life.

**Objectives:** To describe a refractory case of sJIA with persistent arthritis and severe cortico-dependence, successfully treated with a combination of tocilizumab and ixekizumab.

**Methods:** Case report.

**Results:** We present the case of a girl with sJIA onset at 4 years, complicated in the first month by macrophage activation syndrome (MAS). The patient has a positive family history for spondylarthritis and she is HLA B-27 and DRB1*15 positive. At onset, the child was successfully treated with glucocorticoids and IL-6 inhibitor (tocilizumab) with complete response of systemic manifestations. Four months later, she presented a flare with polyarthritis well controlled by adding methotrexate (MTX). At 1 year she was in remission and tocilizumab was gradually tapered, but a systemic and polyarticular relapse occurred, only partially responsive to tocilizumab and MTX, with persistent severe polyarthritis and cortico-dependence. A trial with IL-1 blockers, first canakinumab and then anakinra, was made without any success on the articular involvement, which required several intrarticular corticosteroid injections and long-term steroidal treatment. Given the refractory articular course, tocilizumab was switched to etanercept associated with cyclosporine and MTX; however, a new MAS episode occurred, requiring further high doses of steroids. A new attempt in sparing steroids was made with baricitinib, but the child flared with both systemic and articular symptoms. Considering the severe systemic steroidal toxicity with Cushing syndrome and growth failure, a combination of biologics was considered. Firstly, etanercept was added to tocilizumab, without success. Conversely, the combination of tocilizumab with the anti-IL17 ixekinumab led to a positive control of the disease with a significant steroid tapering. After 18 months of treatment, prednisone was tapered to 0.1 mg/kg/day, no serious adverse event was registered and an initial catch up growth was noticed.

**Conclusion:** Anti IL17 treatment can be included in the armamentarium for sJIA patients with refractory articular course. In severe refractory sJIA patients a combination of biologics may be carefully considered, balancing infectious risk with long-term steroidal toxicity.

**Patient Consent**

Yes, I received consent

**Disclosure of Interest**

None declared

## P308 A case of systemic JIA with lung disease

### A. Dagri^1^, E. Marchettini^2^, S. Pastore^3^, A. Taddio^2,3^, A. Tommasini^2,3^

#### ^1^Department of Medicine DAME-Division of Pediatrics, University of Udine, Udine; ^2^Department of Medical, Surgical and Health Sciences, University of Trieste; ^3^Institute for Maternal and Child Health IRCCS “Burlo Garofolo”, Trieste, Italy

##### **Correspondence:** A. Dagri

*Pediatric Rheumatology 2023*, **21(Suppl 2):**P308

**Introduction:** Systemic juvenile idiopathic arthritis associated with lung disease (sJIA-LD) is a recently recognized subtype of sJIA characterized by the presence of chronic pulmonary manifestations, such as pulmonary hypertension, interstitial lung disease, pulmonary alveolar proteinosis and/or endogenous lipoid pneumonia.

**Objectives:** To describe a case of sJIA-LD.

**Methods:** Case report.

**Results:** A 7-year-old girl was admitted to our PED for a 10-days history of fever associated with severe diffuse arthralgias. Clinical examination revealed active arthritis of the left hip. Blood tests showed increased ESR (88 mm/h) and CRP (156.3 mg/L), anemia (Hb 10.6 g/dL) and neutrophilic leukocytosis (WBC 24530/μl, N 20710/μl). An extremely increased IFN score (103, normal value < 2.6) was also recorded, supporting a possible cytokine storm syndrome. However, normal count of platelets and normal levels of ferritin, fibrinogen, cholesterol and triglycerides were not consistent with a possible macrophage activation syndrome (MAS). Abdominal ultrasound and echocardiography were normal. For a better diagnostic definition, we performed a chest X-ray that showed diffuse interstitial thickening, confirmed by CT scan which also revealed the presence of small marginal bronchiolectasis and bronchiectasis. This presentation was suggestive of sJA-LD, whereby therapy with an interleukin (IL)-1 inhibitor was started (IV anakinra 100 mg/day), with rapid fever and joint pain resolution. However, because of the detection of an extremely increased IFN signature associated with lung involvement, genetic analysis was performed in order to exclude rare autoinflammatory diseases (interferonopathies like COPA syndrome and periodic fevers), later found to be negative.

**Conclusion:** We described a case of an emerging sJIA-LD. In literature, reports of this clinical entity were extremely rare, but recently an increasing number of such complication was observed. Despite mild clinical respiratory presentation, these children present severe pulmonary finding on chest CT, including pleural and septal thickening, “tree-in-bud”, “ground-glass” opacities and peripheral consolidation, expression of a severe inflammation confirmed by lung biopsy. Risk factors include early onset of sJIA (<2 years of age), history of MAS and high IL-18 circulating levels. Often they have a predisposition to adverse reactions to biological drugs (especially tocilizumab). The dramatic increase in incidence after exposure to biological drugs led some authors to consider IL-1 and IL-6 blockers implicated in its pathogenesis, but this relationship still remains to be established. In fact not all patients with sJIA-LD, as in our case, were exposed to these biologic therapies. On the contrary, scientific literature, together with our case, highlighted a dramatical response of most patients to anti IL-1 and IL-6 drugs.

**Patient Consent**

Yes, I received consent

**Disclosure of Interest**

None declared

## P309 Ruxolitinib for macrophage activation syndrome complicated with systemic juvenile idiopathic arthritis: Yang and Ying?

### H. Irabu^1^, M. Shimizu^1^, F. Miyaoka^1^, S. Kaneko^1^, A. Shimbo^1^, M. Murakoshi^1^, A. Nishimura^1^, T. Udagawa^1^, T. Isoda^1^, H. Kanegane^2^, T. Morio^1^

#### ^1^Department of Pediatrics and Developmental Biology, Graduate School of Medical and Dental Sciences; ^2^Department of Child Health and Development, Graduate School of Medical and Dental Sciences, Tokyo Medical and Dental University, Tokyo, Japan

##### **Correspondence:** H. Irabu

*Pediatric Rheumatology 2023*, **21(Suppl 2):**P309

**Introduction:** JAK inhibitors are expected as one of the promising therapeutic drugs for hemophagocytic lymphohistiocytosis (HLH).

**Objectives:** Ruxolitinib, a JAK1/2 inhibitor, has been used for HLH. However, there were few reports to show the efficacy and safety of ruxolitinib for macrophage activation syndrome complicated with systemic juvenile idiopathic arthritis (s-JIA MAS).

**Methods:** We report on a case of s-JIA MAS treated with ruxolitinib.

**Results:** A three-year-old Japanese girl presented with fever and rash. Physical examination showed hepatosplenomegaly. Laboratory and radiological examination revealed extraordinary hyperferritinemia (43,539 ng/mL) and bilateral pleural effusion. Thus she was diagnosed with s-JIA MAS. Methylprednisolone pulse therapy in combination with cyclosporine A (CsA) leads to remission in MAS. However, her s-JIA symptom was difficult to maintain remission. Additional biologics therapies, including tocilizumab and canakinumab, were not fully effective. Accordingly, ruxolitinib (10mg/day) was initiated, and she achieved disease remission. However, leukopenia (<2,000/μL) was observed five days after ruxolitinib administration. The dose of ruxoritinib was reduced to 7.5 mg/day. Furthermore, three weeks after ruxolitinib treatment, She developed Epstein-Barr virus (EBV) associated HLH. Treatment with plasma exchange improved HLH. However, leukopenia and neutropenia persisted. Bone marrow examination revealed the maturation arrest of granulocytes at the promyelocyte stage. Therefore, ruxolitinib was discontinued. Leukopenia was improved two weeks after stopping ruxolitinib. She has been successfully treated with a combination of prednisolone (PSL) and CsA. Now, she has been in remission for 12 months with PSL (1mg/day). Genetic analysis for familial HLH showed no pathogenic variants.

**Conclusion:** Ruxolitinib was effective in controlling s-JIA MAS. However, we should take care of severe adverse events, including leukopenia and infection. Further studies, including appropriate therapeutic dosage settings, would be required.

**Patient Consent**

Yes, I received consent

**Disclosure of Interest**

None declared

## P310 Anakinra for systemic juvenile idiopathic arthritis: experience from a single center in Saudi Arabia

### S. Lootah^1,2,3^, A. AlRasheed^1,2,3^, W. Alsuwairi^1,2,3^, J. Alqanatish^1,2,3^

#### ^1^King Saud bin Abdulaziz University for Health Sciences; ^2^King Abdullah International Medical Research Center (KAIMRC); ^3^Pediatric Rheumatology, King Abdullah Specialized Children’s Hospital, King Abdulaziz Medical City (National Guard Health Affairs), Riyadh, Saudi Arabia

##### **Correspondence:** S. Lootah

*Pediatric Rheumatology 2023*, **21(Suppl 2):**P310

**Introduction:** Anakinra, a recombinant interleukin-1 receptor antagonist, demonstrated efficacy in treating systemic juvenile idiopathic arthritis (sJIA).

**Objectives:** The aim of this abstract is to present our center's experience with using anakinra for the treatment of sJIA.

**Methods:** We conducted a retrospective study of 21 patients with sJIA, aged 1 to 14 years, who were treated with anakinra, as first line biologic, between January 2017 and December 2023. Demographics, treatment response and outcome were analyzed.

**Results:** 75% of our cohort were below the age of 5 years at onset of sJIA. Anakinra was initiated within 2 weeks to 1 year of disease onset. Anakinra was used as monotherapy in 61.9% of patients, and given in higher doses to patients with macrophage activation syndrome. Complete remission was achieved in 77% of patients on anakinra and 86% were able to discontinue it. Anakinra was discontinued in 33%; two patients due to injection-related challenges, one patient due to transaminitis, and four patients due to poor response. The poor response to anakinra was primarily due to delayed treatment and chronic polyarticular arthritis. No serious side effects were observed.

**Conclusion:** Our results suggest that early initiation of anakinra in sJIA (within three months of disease onset) is safe and effective, with high rate of complete remission and an ability to discontinue treatment. Further research with larger sample sizes and longer follow-up periods is warranted.

**Patient Consent**

Not applicable (there are no patient data)

**Disclosure of Interest**

None declared

## P312 The juvenile arthritis damage index as a potential tool for monitoring and predicting the course of systemic juvenile arthritis

### Z. Kolkhidova, I. Nikishina

#### Pediatric, V. A. Nasonova Research Institute of Rheumatology, Moscow, Russian Federation

##### **Correspondence:** I. Nikishina

*Pediatric Rheumatology 2023*, **21(Suppl 2):**P312

**Introduction:** There is a number of biologics (B) successfully used in the treatment of Juvenile Idiopathic Arthritis (JIA). However, some patients are resistant to B therapy. One of indirect factor of therapy resistance may be extended structural damage measured by The Juvenile Arthritis Damage Index (JADI).

**Objectives:** To evaluate JADI in patients with different subtypes of JIA receiving B. To identify correlations of JADI with features of clinical manifestations in patients with systemic JIA (sJIA).

**Methods:** Single-stage evaluation of the JADI index in patients with JIA receiving B. A subgroup of patients with sJIA was formed from the total JIA group for detailed analysis.

**Results:** The study included 408 children consecutively admitted to our clinic. The mean age of the group was 12.5 years, male to female ratio was 1:1.6. There were pts with different kinds of JIA: sJIA-26, Polyarthritis-314; oligoarthritis – 42; enthesitis related arthritis (ERA)-26.

Articular damage (JADI-A) was detected in 23% of patients and extra-articular (JADI-E) in 21%. The maximum average value of JADI was revealed for the sJIA group: JADI-A m 3.6 points, JADI-E m 1.0 point.

The group of sJIA was analyzed separately. Our study showed that there was a direct correlation (p<.05) between JADI-A and current activity (JADAS10, Children Health Assessment Questionnaire (CHAQ), active joint counts, VAS, duration of disease, multiple B failures, high activity in the debut with use of glucocorticoids, and family history. A direct positive correlation was found between JADI-E and the current dose of glucocorticoids. Patients who had no remission periods had higher JADI score.

**Conclusion:** The JADI is an independent tool for measuring the cumulative assessment of joint and extra-articular damage caused by JIA, with the most unfavorable rates for sJIA. High disease activity at onset, long disease duration may predict of the development of unrecoverable disorders in sJIA. Further study of correlations between JADI and clinical features, including inflammatory biomarkers, initial localization of arthritis, and systemic count may provide an justification for the very early administration of "targeted" therapy.

**Patient Consent**

Yes, I received consent

**Disclosure of Interest**

None declared

## P313 Comparative analysis of disease course and clinical outcomes in systemic juvenile idiopathic arthritis: lessons from a 5-year follow-up study

### J. Raghuram^1^, A. K. Tennelli^2,3^, A. B^4^, A. P. Rao^2,3^

#### ^1^Paediatric Rheumatology, Aster Women & Children Hospital; ^2^Paediatric Rheumatology, Manipal Hospital; ^3^Paediatric Rheumatology; ^4^Paediatrics, Indira Gandhi Institute Of Child Health, Bangalore, India

##### **Correspondence:** J. Raghuram

*Pediatric Rheumatology 2023*, **21(Suppl 2):**P313

**Introduction:** Systemic juvenile idiopathic arthritis (SJIA) is characterized by presence of arthritis with systemic features such as fever, rash, lymphadenopathy, hepatosplenomegaly, serositis and laboratory features suggestive of inflammation. SJIA might follow monocyclic, polycyclic or persistent disease course (Wallace criteria).

**Objectives:** This study was undertaken to analyze the outcome at 5-year follow-up and remission rates of our systemic juvenile idiopathic arthritis patient cohort seen in pediatric rheumatology clinics in two tertiary care hospitals.

**Methods:** Children diagnosed with SJIA between the period 2010-2015 (as per ILAR criteria) and had been followed up (physically/telephonically) for at least 5 years were included (n=100). Retrospective data collected in standardized protocol and analyzed. Statistical analysis done using SPSS 20.0 and Datatab online statistical packages. Continuous variables were analyzed using independent sample t test and ANOVA where applicable. Nominal variables were analyzed using chi-square test and Fisher’s extract. Univariate analysis and multivariate analysis used for prediction model. Logistic regression was used. p value < 0.05 was considered significant.

**Results:** The average age of disease onset was 6.75 years (ranging from 7 months to 15 years), and the median follow-up duration was 86.59 months (ranging from 54 to 174 months).

Clinical features observed at presentation included fever in 100% of cases, arthritis in 96%, a classic evanescent rash in 55%, hepatosplenomegaly in 33%, lymphadenopathy in 13%, and serositis in 8%.

Among the patients, 46% followed a monocyclic disease course, 27% had a polycyclic course, and 27% experienced persistent disease activity at the 5-year follow-up.

Notably, a significant diagnostic delay was present, with an average duration of 9.15 months, and the differences between the sub-groups were statistically significant (p value <0.05).

Children with persistent disease activity exhibited a higher number of affected joints at disease onset (p value <0.05) and a greater occurrence of symmetric arthritis (p value <0.05).

Consistent with expectations, the group with persistent disease activity displayed more extensive joint damage.

**Conclusion:** 1."Delayed diagnosis, beyond 6 months, contributes to unfavourable outcomes in Systemic Juvenile Idiopathic Arthritis."

2."Increased initial joint involvement (mean 14.24+/-15.25) heightens the risk of persistent disease in SJIA patients." 3."Symmetric arthritis at the onset indicates a higher likelihood of persistent disease progression in SJIA."

4."The longer the disease persists, the greater the probability of joint damage in Systemic Juvenile Idiopathic Arthritis."

5."Despite a lower utilization of biologics, our study demonstrates comparable outcomes to other cohorts, potentially due to a higher proportion of children in the monocyclic disease group."

**Patient Consent**

Yes, I received consent

**Disclosure of Interest**

None declared

**References**

1. Aoust L, Rossi-Semerano L, Koné-Paut I, Dusser P. Time to diagnosis in juvenile idiopathic arthritis: a french perspective. Orphanet J Rare Dis. 2017 Feb 28;12(1):43. doi: 10.1186/s13023-017-0586-4. PMID: 28241879; PMCID: PMC5329952-
2. Peter van Dijkhuizen EH, Wulffraat NM. Early predictors of prognosis in juvenile idiopathic arthritis: a systematic literature review. Ann Rheum Dis. 2015 Nov;74(11):1996-2005. doi: 10.1136/annrheumdis-2014-205265. Epub 2014 Jun 24. PMID: 24962873

## P314 Combination of biological agents in a patient with Systemic Juvenile Idiopathic Arthritis (SJIA) and Ulcerative Colitis (UC)

### M. T. Riccio, R. Naddei, M. T. Fioretti, F. Fedele, M. Alessio

#### Traslational Medical Science, Section of Pediatrics, University of Naples Federico II, Naples, Italy

##### **Correspondence:** M. T. Riccio

*Pediatric Rheumatology 2023*, **21(Suppl 2):**P314

**Introduction:** Inflammatory bowel diseaseas and rheumatological diseases are characterised by immune dysfunction leading to an aberrant inflammatory response.

**Objectives:** We report the use of combination biological therapy in a patients with ulcerative colitis (UC) and systemic juvenile idiopathic arthritis (sJIA).

**Methods:** Case report

**Results:** L. is a 17 year-old boy. At the age of 6, he was diagnosed with sJIA due to the occurrence of fever, maculo-papular skin rash, arthralgias, laterocervical lymphadenopathy and increased inflammatory indices. He was treated with a course of systemic glucocorticoids (GCs). During the GCs discontinuation, the patient presented an episode of macrophage activation syndrome (MAS), thus intravenous methylprednisolone was started, followed by oral prednisone, and cyclosporine was added with clinical resolution. To decrease the dosage of prednisone and cyclosporine, Canakinumab was started. Two years later, the patient presented a new episode of MAS, successfully treated with intravenous GCs and an increase of cyclosporine dosage. Canakinumab was withdrawn and tocilizumab was started, then discontinued due to an allergic reaction shortly after. At the age of 10, he presented recurrent diarrhea and abdominal pain. Celiac disease was diagnosed due to positive serology and duodenal biopsy and the patient started a gluten-free diet, with clinical resolution. 5 years later, he presented wrist, elbow and fingers arthritis, so methotrexate was started, later discontinued due to intolerance. Meanwhile, bloody diarrhea and abdominal pain occurred, associated with febrile episodes and weight loss. Fecal calprotectin resulted elevated, and a diagnosis of UC was established after an ileocolonoscopy. Therefore, he started a new course of GCs, discontinued cyclosporine and started azathioprine. Shortly after, he presented two episodes of MAS with arthritis relapse, and an intestinal flare associated to an endoscopic pattern of severe pancolitis. Genetic tests for familial hemophagocytic lymphohistiocytosis and autoinflammatory disease showed no significant mutations, while the levels of CXCL-9, CXCL-10 and IL-18 resulted elevated. Anakinra (100 mg/day) was started leading to sJIA remission, and vedolizumab (300 mg every 8 weeks) was began with clinical resolution of UC symptoms, while azathioprine was withdrawn. At the 3-month follow-up, the patient was doing well, and no adverse event was present.

**Conclusion:** The sJIA/UC association is rare, as is the combined use of biological drugs. To our knowledge, this is the first described case of anakinra/vedolizumab combination, while only a few cases of infliximab/vedolizumab combination have been reported. In our patient, the combined treatment with anakinra and vedolizumab was successful in controlling both the diseases and no adverse event was present at the 3-month follow-up.

**Patient Consent**

Yes, I received consent.

**Disclosure of Interest**

None declared

## P315 Detection of ASC specks with imaging flow cytometry: a promising approach for rapid and accurate assessment of inflammasome activation in pediatric rheumatic disease

### G. Rogani^1,2,3^, R. Erkens^2,3^, F. Minoia^1^, B. Vastert^2,3^, J. Van Loosdregt^2^

#### ^1^Pediatric Immuno-Rheumatology Unit, Fondazione IRCCS Ca'Granda Ospedale Maggiore Policlinico, Milan, Italy; ^2^Center for Translational Immunology, University Medical Center; ^3^Department of Pediatric Rheumatology and Immunology, Wilhelmina Children's Hospital, Utrecht, Netherlands

##### **Correspondence:** G. Rogani

*Pediatric Rheumatology 2023*, **21(Suppl 2):**P315

**Introduction:** Autoinflammatory diseases are hallmarked by increased inflammasome activation. Quantification of inflammasome activation could potentially be used as a biomarker for disease activity and guide treatment. Inflammasome activation is hallmarked by polymerization of the diffuse cytoplasmic ASC (Apoptosis-associated Speck-like protein containing a Caspase recruitment domain) molecules into a complex called “speck”.

**Objectives:** Our study aims to develop a valid and reproducible strategy to quantify inflammasome activation by assessing ASC specks in immune cells using Image Flow Cytometry. This technique enables high-throughput quantification of ASC-speck containing cells and provides morphological measurements of individual cells.

**Methods:** We used ASC-fluorescently-tagged cell lines (GFP-ASC cells) and peripheral blood mononuclear cells (PBMCs) of healthy donors. The cells were primed with lipopolysaccharide (LPS) and subsequently activated with Nigericin to induce the canonical NLRP3-mediated inflammasome activation. Fixed and permeabilized primary cells were stained with a fluorescent anti-ASC antibody, and assessed by image flow cytometry using ImageStream X. ASC speck quantification and the cell morphology were analyzed using ImageStream Data Exploration and Analysis Software (IDEAS) and defined by 8 feature categories (size, shape, location, signal strength, texture, comparison, system and combined).

**Results:** We trained the software to discriminate between diffuse ASC signal and clustered ASC-speck phenotypes and used the Fisher’s discriminant ratio (Rd) to determine the accuracy of separation. Using GFP-ASC cells we obtained 6 features with Rd above 1.5, generally recognized as a good indicator, in each condition. These 6 features were related to the size and texture of the image, two parameters that changes characteristically after ASC redistribution in the cytoplasm. Chi-squared test revealed a significant difference in the percentage of ASC-speck-containing cells among stimulated, primed, and unstimulated conditions. Similar results were obtained with PBMCs stained for ACS.

**Conclusion:** Our study presents a fast and accurate strategy for assessing inflammasome activation. Next, we aim to expand these findings to PBMCs from patients with Systemic Juvenile Idiopathic Arthritis and Macrophage Activated Syndrome, both of which involve dysregulation of the inflammasome. Validation of this method in patient cells would provide a powerful tool to better understand the pathophysiology of the disease and to improve current diagnostic and therapeutic strategies.

**Patient Consent**

Not applicable (there are no patient data)

**Disclosure of Interest**

G. Rogani: None declared, R. Erkens: None declared, F. Minoia Consultant with: SOBI, B. Vastert Grant / Research Support with: Research grant from SOBI in a public-private grant opportunity (2017- 2024), Speaker Bureau with: SOBI and Novartis, J. Van Loosdregt: None declared

## P316 Characterization of the time-response to IL-1 receptor inhibitor and treatment retention in patients with systemic juvenile idiopathic arthritis

### S. C. Scheffler-Mendoza^1^, R. Caorsi^1^, E. Longoni^2^, E. Drago^1^, A. Consolaro^1^, C. Malattia^1^, S. Rosina^1^, R. Papa^1^, S. Viola^1^, S. Volpi^1^, A. Ravelli^1^, M. Gattorno ^1^

#### ^1^UOC Reumatologia e Malattie Autoinfiammatorie, IRCCS Istituto Giannina Gaslini, Genova; ^2^Department of Pediatrics, Vittore Buzzi Children's Hospital, Istitution: Università degli Studi di Milano, Milano, Italy

##### **Correspondence:** S. C. Scheffler-Mendoza

*Pediatric Rheumatology 2023*, **21(Suppl 2):**P316

**Introduction:** Systemic-onset juvenile idiopathic arthritis (sJIA) is associated with a severe and refractory systemic illness and is the most severe and difficult to treat of all the form of childhood arthritis (1). The use of IL-1 blockade could offer a therapeutic option in these patients (2). There have been described some predictors of the effectiveness of Anakinra. The decision of when and which treatment offer is challenging. (3)

**Objectives:** To analyze the single center experience in our patients with sJIA treated with anakinra associated with the time of the start of the intervention.

**Methods:** We conducted a retrospective review of the clinical and laboratory features of all the patients with sJIA who were treated with anakinra between 2004 and 2022, followed by our Unit. Clinical inactive disease (CID) was defined as clinical remission and normalization of inflammatory markers and calculated at 6 and 12 months from treatment initiation and at the last follow-up.

Descriptive statistics were reported as medians for continuous variables and as absolute frequencies and percentages for categorical variables.

**Results:** We analyzed 82 patients with sIJA treated with anakinra from 2004 to 2022. We divided the population in two groups according the early (<6 months) or late therapy intervention (ETI/LTI). The main clinical characteristics at the moment of treatment were fever (100%), rash (93%), arthritis (83%), hepatomegaly and lymphadenopathy (30%). The ETI group had lower frequency of fever, rash and arthritis compared with the LTI group. As the treatment started, all patients had previously received therapy with steroid (100%), DMARD (66%) and 29% with biologic DMARD. As baseline treatment, 93% of the patients received steroid and the median initial dosage of anakinra was 1.6 mg/kg daily.

At 6 months of follow-up, 58 patients were still in treatment with anakinra; 22 patients (12 in the ETI group, 10 in LTI) were in CID. At month 12, 49 patients were still on anakinra treatment; 33 patients (15 in the ETI group, 18 in LTI) displayed a CID. The mean follow-up was 8 years (6 months-19.5 years). In the last visit, 14 patients were still on anakinra; 44 patients (19 in the ETI group, 25 in the LTI) were in CID. The main reasons for anakinra discontinuation were: inefficacy (35 patients, 8 ETI, 27 LTI), complete remission (22 patients, 11 ETI, 11 LTI), side effect/adverse events (8 patients, 5 ETI, 3 LTI), switch to other IL-1 inhibitor in responder patient (6 patients, 3 ETI, 3 LTI). Independently from the time of initiation of the treatment, higher levels of WBC, higher level of ferritin and lower number of active joints correlated to CID.

**Conclusion:** In our population the early therapy intervention reduced the probability of relapsing disease. Integration of clinical and immunological data studies are necessary to identify a severe disease and treat them earlier.

**Patient Consent**

Not applicable (there are no patient data)

**Disclosure of Interest**

None declared

**References**

1. Boyarchuk O, Kovalchuk T, Kovalchuk N, Chubata O. Clinical variability of the systemic juvenile idiopathic arthritis course: literature review based on case series. Reumatologia. 2020;58(6):436-443.
2. Toplak N, Blazina Š, Avčin T. The role of IL-1 inhibition in systemic juvenile idiopathic arthritis: current status and future perspectives. Drug Des Devel Ther. 2018 Jun 8;12:1633-1643
3. Saccomanno B, Tibaldi J, Minoia F, Bagnasco F, Pistorio A, Guariento A, Caorsi R, Consolaro A, Gattorno M, Ravelli A. Predictors of Effectiveness of Anakinra in Systemic Juvenile Idiopathic Arthritis. J Rheumatol. 2019 Apr;46(4):416-421.

## P317 Safety and efficacy evaluation of Perkinra (Anakinra Manufactured by Persis Gene) in comparison with KINERET® (Anakinra manufactured by SOBI) in systemic juvenile idiopathic arthritis patients

### A. Z. Mirzaee^1^, S. Sadeghi^2^, R. Shiari^1^, R. Sinaei ^3^, F. Tahghighi ^4^, A. Karagah^2^, V. Ziaee^4^, S. H. Ghotbabadi^5^, K. Rahmani^1^, M. R. Fathi^6^, L. Ghasemi^7^, A. Miremarati^8^

#### ^1^Pediatrics, Shahid Beheshti University of Medical Sciences; ^2^Department of Clinical Pharmacy, Tehran University of Medical Sciences, Tehran; ^3^Pediatrics, Kerman University of Medical sciences, Kerman; ^4^Pediatrics, Tehran University of Medical Sciences, Tehran; ^5^Pediatrics, Shiraz University of Medical Sciences, Shiraz; ^6^Pediatrics, Chamran University of Medical Sciences, Ahvaz; ^7^Pediatrics, Qazvin University of Medical Sciences, Tehran; ^8^Pediatrics, Guilan University of Medical Sciences, Rasht, Iran, Islamic Republic Of

##### **Correspondence:** R. Shiari

*Pediatric Rheumatology 2023*, **21(Suppl 2):**P317

**Introduction:** Systemic juvenile idiopathic arthritis (sJIA) is marked with arthritis and several features of systemic inflammation, including fever, rashes, hepatosplenomegaly, lymphadenopathy, and serositis.

**Objectives:** Il-1 receptor blockers have been used as one of the most effective biologics for the treatment of resistant type of disease. The main purpose of this study was to evaluate the safety and efficacy of PerkinRA (manufactured by Persis Gene) in comparison with SOBI Kineret® (Manufactured by SOBI) in patients with sJIA.

**Methods:** This double-blind clinical trial study was performed on 72 patients with sJIA based on the 2018 ILAR criteria that were treated in the outpatient clinic of Mofid Chidren’s Hospital and Pediatric Medical Center and four other centers between Feb 2020 to March 2021. Patients were randomly assigned to two groups: one group was treated with PerkinRA (N=36), and the other group was treated with Kineret (N=36). Treatment response was based on ACR 30, ACR 50, and ACR 70. Also, the patient's vital signs, drug side effects, clinical laboratory tests for systemic evaluation, and changes in findings related to physical examinations were recorded after 1, 2, 4, 8, 12, 16, 20, and 24 weeks of the first injection, and the two groups were compared.

**Results:** 35 patients in PerkinRA and 32 patients in Kineret group were analyzed. The results showed with no clinically significant differences, patients in both groups achieve ACR 30, ACR 50 and ACR 70 during 6 month evaluation.

**Conclusion:** According to our findings, the two medicines; PerkinRA and Kineret are equal in terms of primary and secondary outcomes, and it can be concluded that the two groups were non inferior.

**Trial registration identifying number:** IR.SBMU.REC.1398.161

**Patient Consent**

Yes, I received consent

**Disclosure of Interest**

None declared

**References**

1. Ghavidel A, Farivar S, Aghamiri S, Shiari R. Association between forkhead box P3 expression level and gender of Iranian juvenile idiopathic arthritis patients. Reumatologia/Rheumatology. 2022;60(1):26-34.
2. Kumar S. Systemic juvenile idiopathic arthritis: diagnosis and management. The Indian Journal of Pediatrics. 2016;83(4):322-7.
3. Russo RA, Katsicas MM. Patients with very early-onset systemic juvenile idiopathic arthritis exhibit more inflammatory features and a worse outcome. The Journal of rheumatology. 2013;40(3):329-34.
4. Mellins ED, Macaubas C, Grom AA. Pathogenesis of systemic juvenile idiopathic arthritis: some answers, more questions. Nature Reviews Rheumatology. 2011;7(7):416-26.
5. Grevich S, Shenoi S. Update on the management of systemic juvenile idiopathic arthritis and role of IL-1 and IL-6 inhibition. Adolescent health, medicine and therapeutics. 2017;8:125.

## P318 Profile of patients with systemic onset juvenile idiopathic arthritis at a children's university hospital

### F. Sztajnbok, J. L. Monteiro, F. C. Zonis, M. F. Rodrigues, A. R. Fonseca, P. R. Souza, I. M. Paz, M. R. Vasti, R. G. Almeida

#### Pediatric Rheumatology, Universidade Federal do Rio de Janeiro, Rio de Janeiro, Brazil

##### **Correspondence:** F. Sztajnbok

*Pediatric Rheumatology 2023*, **21(Suppl 2):**P318

**Introduction:** Systemic Onset Juvenile Idiopathic Arthritis (SoJIA) is a rare childhood inflammatory disease associated with significant morbidity. The course of the disease is variable, monocyclic, polycyclic, or relapsing. It can lead to sequelae with serious impairment of quality of life.

**Objectives:** The aim of this study is to describe the clinical, epidemiological, and therapeutic profile of patients diagnosed with SoJIA in a single university center.

**Methods:** A cross-sectional, descriptive, retrospective study was carried out, analyzing medical records of patients with the diagnosis of SoJIA, between 0-17 years of age. Pediatric rheumatology follow-up took place between 2011 and 2022. The diagnosis was made according to the ILAR criteria.

**Results:** Data were collected from 31 patients (14 male and 17 female). The mean age at onset of symptoms was 6.5 years (1-11) and at diagnosis 7 years (2-11). The mean time to diagnosis was 4.7 months (1-14). Regarding the presenting manifestations, all patients presented with arthritis and prolonged fever and 29 (93.5%) had rash. Other clinical manifestations occurred less frequently: hepatomegaly (10 – 32%), splenomegaly (5 – 16%), serositis (5 – 16%), and lymph node enlargement (4 – 12.9%). Only 2 patients (6.4%) presented with macrophage activation syndrome (MAS) as a manifestation at the onset of SJIA. The most common laboratory findings were: increased erythrocyte sedimentation rate (ESR) (31 – 100%), high C-reactive protein (CRP) (24 – 77.4%), anemia (16 – 51.6%), elevation of liver enzymes (14 – 45%) and hyperferritinemia (13 – 41.9%). Concerning drug treatment, 29 patients (93.5%) received systemic corticosteroids, 26 (83.8%) methotrexate, 25 (80%) tocilizumabe, 7 (22.5%) anti-TNF agents, 6 cyclosporine (19.3%), 4 abatacept (12.9%) and 3 anakinra (9.6%). The mean time to treatment response was 5.7 months (2-14) and 15 patients (48%) remained with inactive disease for a mean time period of 27.8 months and 7 of these patients relapsed. Furthermore, 18 patients (58%) developed complications of erosive arthritis and 5 patients (16%) developed MAS during the course. There were no pulmonary complications or death.

**Conclusion:** Despite the advances in SoJIA treatment over the past decade, more than half of our patients evolved to a chronic erosive course of arthritis, and almost half still persist in disease activity, even with the use of biological drugs. Besides, 7 (22.5%) developed MAS, mostly during the disease course. This study highlights the importance of an early diagnosis and treatment and of the need for faster access to biological treatment, especially in less-resourced countries, to avoid acute complications such as MAS and chronic complications that may affect the quality of life of our patients.

**Patient Consent**

Yes, I received consent

**Disclosure of Interest**

None declared

## P319 Systemic juvenile idiopathic arthritis and adult-onset still's disease: same spectrum distinct features

### N. Tekgoz^1^, B. Acar^1^, S. Yılmaz^2^

#### ^1^Pediatric Rheumatology, Ankara City Hospital; ^2^Rheumatology, Gülhane Training Hospital, Ankara, Türkiye

##### **Correspondence:** N. Tekgoz

*Pediatric Rheumatology 2023*, **21(Suppl 2):**P319

**Introduction: Introduction**

Systemic-onset juvenile idiopathic arthritis (sJIA) and adult-onset Still’s (AOSD) disease are systemic inflammatory diseases characterized by spiking fever, arthritis and skin rash. Organ involvement such as hepatomegaly, splenomegaly and pleuritis can be seen and may be complicated with macrophage activation syndrome (MAS).

**Objectives:** The aim of this study was to evaluate the differences and similarities between sJIA and AOSD in clinical findings, laboratory parameters and treatment options.

**Methods: Material and Method**

Patient data were collected from two tertiary hospital rheumatology centers. Pediatric patients diagnosed with sJIA according to the ILAR criteria between 2002-2022 and adult patients diagnosed with AOSD according to the to the Yamaguchi criteria between 2016-2022 were included in the study . Demographic data, presenting symptoms, clinical features, laboratory parameters, medications, disease course, complications and morbidity were recorded.

**Results: Results**

The study included 39 patients with AOSD and 63 patients with sJIA. Arthritis and hepatosplenomegaly were more common in SJIA (p<0.001) and sore throat was detected more frequently in AOSD (p=0.041). Duration of fever, frequency of lymphadenopathy, skin rash and serositis were similar in two entities. Ferritin and CRP levels were significantly higher in AOSD (p=0.021 and p<0.001, respectively). Monocyclic pattern was observed more frequently in sJIA and chronic pattern was more common in AOSD (p=0.005). MAS was more developed in sJIA patients (p=0.002). Duration of treatment with oral steroid and synthetic DMARDs was significantly longer in AOSD (p<0.001 and p=0.017, respectively).

**Conclusion: Conclusion**

Systemic JIA and AOSD both develop on the background of inflammation, but there are many differences between the two diseases. This study highlights there may be a tendency in articular and monocyclic disease in sJIA. Chronic disease patterns and more extended treatment periods were seen in AOSD. Hepatosplenomegaly and sore throat were essential and common clinical findings in sJIA and AOSD, respectively. CRP and ferritin, which are important markers of inflammation were significantly higher in AOSD. Additionally, MAS which is a hyperinflammatory condition was found to be more frequent in sJIA.

**Patient Consent**

Not applicable (there are no patient data)

**Disclosure of Interest**

None declared

## P320 Macrophage activation syndrome in systemic juvenile idiopathic arthritis patients on biologic therapy: unraveling diagnostic challenges and clinical implications

### K. Ulu^1^, E. Aliyev^2^, E. Kılıç Könte^3^, A. Tanatar^4^, S. Türkmen^1^, S. Doğantan^5^, Z. Kızıldağ^6^, B. Kasap Demir^7^, D. Gezgin Yıldırım^8^, G. Otar Yener^9^, K. Öztürk^10^, Ö. Baba^11^, C. Açarı^12^, G. Kılbaş^13^, S. N. Taşkın^5^, F. Haşlak^3^, S. Çağlayan^1^, E. Bağlan^14^, H. Adıgüzel^15^, Ö. Başaran^2^, K. Barut^3^, S. G. Karadağ^4^, T. Coşkuner^1^, H. E. Sönmez^16^, S. Yüksel^13^, M. Kalyoncu^11^, S. Bakkaloğlu^8^, S. E. Yüksel^6^, A. Paç Kısaarslan^5^, Y. Bilginer^2^, N. Aktay Ayaz^4^, Ö. Kasapçopur^3^, S. Özen^2^, B. Sözeri^1^

#### ^1^Pediatric Rheumatology, Ümraniye Training and Research Hospital, Istanbul; ^2^Pediatric Rheumatology, Faculty of Medicine, Hacettepe University, Ankara; ^3^Pediatric Rheumatology, Cerrahpaşa Faculty of Medicine, İstanbul University; ^4^Pediatric Rheumatology, İstanbul Faculty of Medicine, İstanbul University, Istanbul; ^5^Pediatric Rheumatology, Faculty of Medicine, Erciyes University, Kayseri; ^6^Pediatric Rheumatology, Faculty of Medicine, Dokuz Eylül University; ^7^Pediatric Rheumatology, Tepecik Training and Research Hospital, Izmir; ^8^Pediatric Rheumatology, Faculty of Medicine, Gazi University, Ankara; ^9^Pediatric Rheumatology, Gaziantep Medical Point Hospital, Gaziantep; ^10^Pediatric Rheumatology, Göztepe Prof. Dr. Süleyman Yalçın City Hospital, Istanbul; ^11^Pediatric Rheumatology, Faculty of Medicine, Karadeniz Technical University, Trabzon; ^12^Pediatric Rheumatology, Faculty of Medicine, İnonu University, Malatya; ^13^Pediatric Rheumatology, Faculty of Medicine, Pamukkale University, Denizli; ^14^Pediatric Rheumatology, Ankara Etlik City Hospital, Ankara; ^15^Pediatric Rheumatology, Van Training and Research Hospital, Van; ^16^Pediatric Rheumatology, Faculty of Medicine, Kocaeli University, Kocaeli, Türkiye

##### **Correspondence:** K. Ulu

*Pediatric Rheumatology 2023*, **21(Suppl 2):**P320

**Introduction:** Biological drugs have revolutionized the treatment of juvenile idiopathic arthritis (sJIA) and macrophage activation syndrome (MAS). MAS remains a potential and potentially life-threatening complication in patients diagnosed with sJIA. Biological drugs specifically target immune pathways that are involved in the development of these conditions.

**Objectives:** The aim of this study is to investigate the effect of biological therapy on sJIA associated MAS.

**Methods:** Sixteen pediatric rheumatology centers across the country demographic, clinical and laboratory data of patients who were being followed up with a diagnosis of sJIA associated MAS were evaluated. The clinical and laboratory features of MAS developing under the impact of biological drugs treatment and MAS developing without this treatment were compared.

**Results:** One hundred and sixty-two patients were included in the study. 45 of the MAS events were detected under the effect of biological treatment, while the remaining 155 events have not received biological treatment in the last three months. Platelet [128 (72-232) vs 199 (130-371) 10^9^/L), ferritin on admission [1107 (676-2050) vs 2863 (1193-9562) ng/ml], C-reactive protein [15.4 (2.9-56) vs 90 (32-160) mg/L], erythrocyte sedimentation rate [13 (3-36) vs 43.5 (13-77) mm/h] and fever duration [5 (4-7.5) vs 10 (7-14.3) days] were found to be lower in the group under the impact of biological drugs. When the biologic drug-treated groups were compared with in the group not receiving biologic therapy in terms of ferritin at admission, ferritin tended to be lower in the anakinra-treated group, while it was significantly lower in the canakinumab and tocilizumab-treated groups (p=0.062, p=0.020, p=0.003). At the time of presentation, 12 (26.6%) patients in biologic drug-treated groups and 20 (12.9%) patients in the group not receiving biologic therapy did not fulfill the ferritin>684 ng/ml requirement according to MAS classification criteria (p=0.049). The rates of hepatomegaly and splenomegaly were relatively lower in the canakinumab treated group.

**Conclusion:** Biological drugs affect the clinical and laboratory features of MAS, and proposed guidelines for MAS could not be appropriate in the biological treatment setting.

**Patient Consent**

Yes, I received consent

**Disclosure of Interest**

None declared

## P321 Interleukin profile in children with systemic juvenile idiopathic arthritis

### V. Yussupova^1^, S. Valieva^2^, S. Kurbanova^3^, E. Afonina^4^, E. Zholobova^4^

#### ^1^rheumatology, I.M. Sechenov First Moscow State Medical University (Sechenov University); ^2^ Department of Rheumatology, Morozov Children’s City Clinical Hospital, Moscow, Russian Federation; ^3^Morozov Children’s City Clinical Hospital; ^4^Rheumatology, Department of pediatrics, I.M. Sechenov First Moscow State Medical University, Moscow, Russian Federation

##### **Correspondence:** V. Yussupova

*Pediatric Rheumatology 2023*, **21(Suppl 2):**P321

**Introduction:** Systemic idiopathic juvenile arthritis (sJIA) is an acute and severe autoinflammatory disorder characterized by fever, rash, generalized lymphadenopathy, hepato- and/or splenomegaly, serositis, arthritis, or arthralgias. Onset of sJIA is characterised by prevalence of autoinflammatory features rather than autoimmune. Currently, the role and diagnostic value of pro-inflammatory cytokines such as IL-1, IL-6 and IL-18, TNF, usually increased in sJIA, is being studied.

**Objectives:** the purpose of this study was to determine the level of IL-1, IL-6 and IL-18, TNF in patients with sJIA or with suspected debut of sJIA.

**Methods:**

In 10 patients with sJIA or with suspected debut of sJIA, the levels of IL-1, IL-6 and IL-1, TNF were examined. Age group from 1 year to 18 years. Patients with the following symptoms were included in the study: fever, rash, lymphadenopathy, hepato- and/or splenomegaly.

**Results:** In all children an increase in C-reactive protein, an acceleration of ESR, leucocytosis, neutrophilia, some had anaemia and thrombocytosis were noted.

The diagnosis of sJIA was confirmed in 9 children, the diagnosis of Kawasaki disease with the threat of developing macrophage activation syndrome was verified in one patient. All examined patients had elevated levels of IL-18, some of them also had elevated levels of IL-1, IL-6. 8 out of 9 patients with sJIA, had the variant of the manifestation with a predominance of systemic manifestations, in 2 patients damage to the joints was detected. In the study of the interleukin profile 7 patients out of 10 had an increased level of IL-1 in the blood serum [10,71; 1023] (reference values 0-5 pg/ml), in 9 out of 10 patients - an increased level of IL-6 [18,4; 379] (reference values < 7 pg/ml), 5 patients had elevated TNF-α levels [30,6; 59] (reference values 0-6 pg/ml). Patients with a predominance of systemic manifestations of the disease in the blood serum showed higher levels of IL-18 [1567,53: 3191] (reference values 104-650 pg/ml), in comparison with patients who had articular syndrome predominated. Also, the level of TNF-alpha did not show significant results, its increase in all patients was either insignificant, or its level in the blood was within the reference values.

**Conclusion:** Serum levels of IL-1, IL-6 and IL-18 could be a helpful tool in differential diagnosis of sJIA. In addition, some of them could be useful for prediction the variant of sJIA disease.

**Patient Consent**

Yes, I received consent

**Disclosure of Interest**

None declared

## P322 TNF inhibitors and pregnancy: a retrospective multicentric study in spondylarthritis patients

### Tiago Beirão^1^, Rafaela Nicolau^2,5^, Inês Santos^2^, Francisca Guimarães^3^, Francisca Aguiar^4,5^, Sara Ganhão^4,5^, Mariana Rodrigues^4,5^, Nádia Filipa^2^, Anabela Rocha^6^, Sofia Monteiro^6^, Iva Brito^4,5^

#### ^1^Rheumatology department, Centro Hospitalar Vila Nova de Gaia/Espinho, Porto, Portugal; ^2^Rheumatology department, Centro Hospitalar Tondela-Viseu, Viseu, Portugal; ^3^Pediatric department, Centro Hospitalar Entre Douro e Vouga, Santa Maria da Feira, Portugal; ^4^Pediatric and young adult Rheumatology unit, Centro Hospitalar Universitário de São João, Porto, Portugal; ^5^Faculty of Medicine, University of Porto, Porto, Portugal; ^6^Obstetrics department, Centro Hospitalar Universitário de São João, Porto, Portugal

##### **Correspondence:** F. Aguiar

*Pediatric Rheumatology 2023*, **21(Suppl 2):**P322

**Introduction:** Tumor necrosis factor (TNF) inhibitors are effective treatments in spondylarthritis patients. In the last years, their use during pregnancy is increasing due to higher experience in the area. However, there is still limited data on the effects of these medications on neonatal outcomes.

**Objectives:** The aim of this study is to determine neonatal outcomes in patients with spondylarthritis treated with TNF inhibitors

**Methods:** This multicentric Portuguese retrospective study between 2020 and 2022, analysed 50 pregnancies of spondylarthritis patients and their exposure to TNF inhibitors. Statistical analysis was performed to compare the outcomes between the two groups.

**Results:** In total, 16 out of 50 patients were treated with TNF inhibitor throughout pregnancy (1 with adalimumab and 15 with certolizumab). Regarding treatment, the TNF inhibitor group had a lower percentage of prednisolone prescription, although not statistically significant (9.10% vs 29.40%, p=0.065). Whilst no preterm births were recorded in this group (0.0% vs 13.3%), the only case of foetal malformation and intrauterine growth restriction was in a patient treated with TNF inhibitor (certolizumab). 1 case was admitted to the neonatal intensive care unit. No statistically significant difference was found on the gestational age at birth and birth weight.

**Conclusion:** There was no statistically significant association between the use of TNF inhibitors during pregnancy and adverse neonatal outcomes in spondyloarthritis patients. However, this study was limited by its retrospective design and small sample size. Further research is needed to confirm these results and investigate potential long-term effects of TNF inhibitor treatment during pregnancy.

**Patient Consent**

Not applicable (there are no patient data)

**Disclosure of Interest**

None declared

## P323 Enthesitis-related arthritis in children across two continents: a comparative study from Turkey and Tunisia

### F. G. Demirkan^1^, V. Guliyeva^1^, Ö. Akgün^1^, H. L. Ferjani^2^, D. B. Nessib^2^, K. Maatallah^2^, D. Kaffel^2^, W. Hamdi^2^, N. Aktay Ayaz^1^

#### ^1^Pediatric Rheumatology, İstanbul School of Medicine, İstanbul, Türkiye; ^2^Rheumatology, Kassab Orthopedics Institute, Ksar Saïd, Tunisia

##### **Correspondence:** N. Aktay Ayaz

*Pediatric Rheumatology 2023*, **21(Suppl 2):**P323

**Introduction:** Spondyloarthritides (SpA) may exhibit distinct disease spectra based on ethnic origin.

**Objectives:** The pediatric rheumatology teams from Istanbul Medical Faculty and Tunisia Kassab Institute collaborated through the 2 nd Sister Hospital initiative of European Society of Pediatric Rheumatology (PReS). They congregated for a duration of 4 months on the digital platform to share their expertise, knowledge and updates on diseases and treatments.

**Methods:** As the output of the consecutive sessions, a study examining the clinical features of children with enthesitis related arthritis (ERA) followed in these two reference centers was scheduled. The medical records of patients were retrospectively reviewed and analyzed through comparison.

**Results:** Overall 94 children were included (45 Tunisian, 49 Turkish patients). Upon presentation, sacroiliac joint tenderness and uveitis were significantly more prevalent among Tunisian patients (91% vs. 46, 26% vs.1, respectively, all p < 0.05), whereas enthesitis was significantly more prevalent among Turkish patients (46.2% vs. 8.9 in Turkish patients vs. Tunisian patients, p < 0.05). In addition, 50% of the Tunisian children versus 43.8% of the Turkish patients demonstrated sacroiliitis on magnetic resonance imaging (p p=0.7, N = 36). Less than one-third of the Turkish patients (32%) were HLA-B27 positive vs. 81.8% of Tunisian patients (p < 0.001). Although the median Juvenile spondyloarthritis disease activity (JSpADA) scores at the onset of the disease were similar between the children from the two countries (p=0.69), the score was significantly lower in Turkish children at the final visit of them (1±1.1 and 0±0.3 in Tunisian patients vs. Turkish patients, p=0.03). Conventional disease modifying anti-rheumatic drugs (cDMARDs) were the common prescribed drug in Tunisia and only 8.9% of patients were treated with biologic drugs. However, nearly one-third of the Turkish children required biologic drugs during the disease course.

**Conclusion:** It is important to emphasize that ethnic background and geographic environment are associated with distinct clinical aspects and treatment outcomes of SpA in children.

**Patient Consent**

Yes, I received consent

**Disclosure of Interest**

None declared

**References**

1. Ghantous, Nassem et al. “Do geography and ethnicity play a role in juvenile Spondyloarthritis? A multi-center binational retrospective study.” *Pediatric rheumatology online journal* vol. 19,1 4. 6 Jan. 2021, doi:10.1186/s12969-020-00489-8
2. Gulati, Reena et al. “Association of HLA-G, HLA-E and HLA-B*27 with susceptibility and clinical phenotype of enthesitis related arthritis (ERA).” *Human immunology* vol. 82,9 (2021): 615-620. doi:10.1016/j.humimm.2021.06.006
3. Skare, Thelma L et al. “Ethnic influence in clinical and functional measures of Brazilian patients with spondyloarthritis.” *The Journal of rheumatology* vol. 39,1 (2012): 141-7. doi:10.3899/jrheum.110372

## P324 Sacroiliac pain: beware of streptococcal pelvic pyomyositis

### F. Anselmi^1^, A. Lo Vecchio^2^, E. Bruzzese^2^, I. Landi^2^, R. Carpio^3^, R. Naddei^1^, M. Alessio^1^

#### ^1^Department of Translational Medical Sciences- Pediatric Rheumatology unit; ^2^Department of Translational Medical Sciences- Pediatric Infectious Diseases Unit; ^3^Department of advanced biomedical sciences, "Federico II"University, Naples, Italy

##### **Correspondence:** F. Anselmi

*Pediatric Rheumatology 2023*, **21(Suppl 2):**P324

**Introduction:** Differential diagnosis in sacroiliac pain and limping in children is challenging,ranging from infectious to inflammatory etiologies. In the past years,especially in the postCOVID-19 era,there has been a resurgence of severe GroupA beta-hemolytic Streptococcus (GABHS)infections. Nonetheless GABHS pyomyositis remains uncommon in children. Here we report the case of a limping girl with sacroiliac pain who later revealed a streptococcal myositis of iliopsoas and piriformis muscles.

A previously healthy 10-year-old girl was admitted to our Unit with a history of mild fever,limping and back pain. One week before she fell off the bike,scratching her knee,and two days after she reported a mild lumbosacral trauma felling off the chair. Plain radiographs of hips were negative. Within24h she was admitted to the hospital because of worsening of back pain and persistent fever. On admission she had fever(40°C). She had an ill-general appearance,moderate to severe pain on right sacroiliac joint palpation,painful limitation on external rotation of left thigh,without skin erythema/edema,nor any other joint abnormalities. Laboratory investigations revealed leukocytosis with neutrophilia and elevated inflammatory markers (ESR98mm/h;CRP127.7mg/l;PCT15.48ng/ml),normal CPK and aldolase levels. AntistreptolysinO titer (ASO) increased from 32-prior to admission-to 5500after 10days.The serological tests for principal viral agents and the immunological studies (ANA,ENA,RF,HLAb27) resulted negative. Magnetic resonance image of pelvis showed T2-hyperintensity within the posterior iliopsoas and piriformis muscle with a small amount of fluid tracking underneath the fascia,suggesting a diagnosis of myositis. There was no evidence of arthritis,fasciitis,osteomyelitis or abscesses. Blood,urine and throat cultures were obtained before starting empiric antibiotic therapy with clindamycin and ceftriaxone. GABHS was isolated either from multiplexPCR testing on whole blood samples or blood cultures. The patient was discharged 18days after,showing negative blood culture and inflammatory markers and recovering normal painless range of motion of legs and back.

**Conclusion:** Primary pelvic pyomyositis is a rare but increasing condition in children. Although Staphylococcus aureus remains the principal causative agent, GABHS has been proved to cause a rare and serious form of myositis,which differs from the staphylococcal pyomyositis because it may not be associated to abscess formation,it often requires a previous muscle trauma and it has a worse prognosis. Early diagnosis is therefore crucial. MRI and bacteriological tests are the most valuable diagnostic tools. This is the first report of primary streptococcal pyomyositis in iliopsoas and piriformis muscles. In children complaining of sacroiliac pain and limping,pyomyositis should be considered in the differential diagnosis in order to start the prompt and appropriate antibiotic treatment.

**Patient Consent**

Yes, I received consent

**Disclosure of Interest**

None declared

## P325 An evaluation of the efficiency of methotrexate in the treatment of juvenile enthesitis related arthritis

### L. Krämer^1^, I. Foeldvari^2^

#### ^1^Asklepios Campus Hamburg of the Semmelweis University Budapest; ^2^Hamburger Centre for Paediatric and Adolescent Rheumatology, Hamburg, Germany

##### **Correspondence:** I. Foeldvari

*Pediatric Rheumatology 2023*, **21(Suppl 2):**P325

**Introduction:** Methotrexate (MTX) is one of the suggested DMARD according to the latest German treatment guideline for the treatment of juvenile enthesitis related arthritis (ERA). Despite that, no prospective study reviewed its efficacy.

**Objectives:** This study aims to evaluate the efficacy of methotrexate used in treating children with ERA.

**Methods:** We retrospectively reviewed consecutive patients’ data from 2017 to 2021, who were treated with MTX for at least 3 months. The clinical manifestation of the ERA was determined by using JADAS and the JSpADA. Good therapeutic response was defined by a reduction of the JSpADA by 0,8 points or more. A JADAS < 2 was considered minimal disease activity, whereas a JADAS < 1 was interpreted as inactive disease. Follow-ups were held at three, six and twelve months after the start of the medical treatment.

**Results:** 188 patients with intention to treat were extracted from which 117 were treated with MTX for at least three months. At all follow-ups, the patients showed a significant reduction in both scores compared to the start. JADAS changed 9.2/6.5/5.6/3.6 and JSpADA 2.67/1.88/1.71/1.13 by mean at 0/3/6/12 months. At M03 49,57 % of the patients were considered good responders, at M12 it was 70 %. Half of the patients achieved remission at M12, another 12,82 % showed only minimal disease activity.

**Conclusion:** This study shows the efficacy of MTX as first-line therapy for the treatment of children with ERA. Comparative prospective studies on MTX and Sulfasalazine are needed to show which drug is more effective.

**Patient Consent**

Not applicable (there are no patient data)

**Disclosure of Interest**

None declared

**References**

1. Weiss PF, Colbert RA, Xiao R, Feudtner C, Beukelman T, DeWitt EM, et al. Development and retrospective validation of the juvenile spondyloarthritis disease activity index. Arthritis Care Res (Hoboken). 2014;66(12):1775-82.
2. Consolaro A, Ruperto N, Bazso A, Pistorio A, Magni-Manzoni S, Filocamo G, et al. Development and validation of a composite disease activity score for juvenile idiopathic arthritis. Arthritis Rheum. 2009;61(5):658-66.
3. Consolaro A, Bracciolini G, Ruperto N, Pistorio A, Magni-Manzoni S, Malattia C, et al. Remission, minimal disease activity, and acceptable symptom state in juvenile idiopathic arthritis: defining criteria based on the juvenile arthritis disease activity score. Arthritis Rheum. 2012;64(7):2366-74.

## P326 Systemic autoinflammatory manifestations in patients with spondyloarthritis

### C. Gaggiano^1^, M. Zajc Avramovič^2^, A. Vitale^1^, N. Emeršič^2^, J. Sota^1^, N. Toplak^2^, S. Gentileschi^1^, Š. Blazina^2^, M. Tarsia^3^, G. Markelj^2^, S. Telesca^1^, V. Parretti^1^, T. Vesel Tajnšek^2^, C. Fabiani^4^, A. Koren Jeverica^2^, B. Frediani^1^, L. Cantarini^1^, T. Avčin^2^

#### ^1^Department of Medical Sciences, Surgery and Neuroscience, Rheumatology Unit, University of Siena and Azienda Ospedaliero-Universitaria Senese (ERN RITA Center), Siena, Italy; ^2^University Children's Hospital, Department of Allergology, Rheumatology and Clinical Immunology, University of Ljubljana and University Medical Centre Ljubljana (ERN RITA center), Ljubljana, Slovenia; ^3^Department of Molecular Medicine and Development, Clinical Pediatrics; ^4^Department of Medical Sciences, Surgery and Neuroscience, Ophthalmology Unit, University of Siena and Azienda Ospedaliero-Universitaria Senese (ERN RITA Center), Siena, Italy

##### **Correspondence:** C. Gaggiano

*Pediatric Rheumatology 2023*, **21(Suppl 2):**P326

**Introduction:** Spondyloarthritis (SpA) has been associated with several autoinflammatory conditions, including FMF, Behçet’s syndrome and, more recently, undifferentiated systemic autoinflammatory disease (USAID) and Still disease (SD)[1,2].

**Objectives:** (1) to describe a cohort of patients with SpA and systemic autoinflammatory manifestations (*S-SpA cohort*) against (a) a control cohort of SD patients without SpA (*SD cohort*) and (b) a control cohort of SpA patients without systemic manifestations (*SpA cohort*); (2) to identify predictors of the development of SpA in patients classified as SD according to the ILAR, PRINTO or Yamaguchi criteria.

**Methods:** Demographic, clinical, biological, radiological, and therapeutic data of patients affected by S-SpA, SD or SpA were collected retrospectively and statistically analyzed.

**Results:** Forty-one subjects were enrolled in the *S-SpA cohort* [21F/20M; mean±SD age 39.0±15.8 years (8.9 – 69.1)]*,* 39 in the *SD cohort* and 42 in the *SpA cohort*. Mean±SD latency between systemic and articular manifestations in *S-SpA* was 8.0±10.6 years (0 – 46.2).

Compared to the *SD cohort*, *S-SpA* patients had lower mean body temperature (*p*<0.001), less frequent rash (*p*<0.001), serositis (*p*=0.049) and weight loss (*p*=0.029), while showing more frequent pharyngitis (*p*=0.002), gastrointestinal symptoms (*p*=0.004) and chest pain (*p*=0.042). In the systemic phase, ESR, CRP, WBC, ANC, and LDH tested higher in *SD* than *S-SpA* (*p*<0.01). The median treatment delay was 17.0(IQR 28.0) months in *S-SpA* and 1.5(IQR 9.8) in *SD* (*p*<0.001). Complete resolution of systemic symptoms was reported less frequently in *S-SpA* than in *SD* patients according to corticosteroid (*p*<0.001), methotrexate (*p*=0.031) or biologic drug (*p*=0.047) treatment.

MRI signs of sacroiliac inflammation and sacroiliac and spine structural damage were detected with similar frequency in *S-SpA* and *SpA* (*p*>0.05)*.* S-SpA patients had less frequent corner inflammatory lesions (*p*=0.03) and inflammation at the facet joints (*p*<0.001) while showing more interspinous enthesitis (*p*=0.01) and inter-apophyseal capsulitis (*p*<0.001). *S-SpA* and *SpA* had similar frequencies of peripheral arthritis and enthesitis (*p*>0.05), while tenosynovitis was more frequent (*p*=0.03) and uveitis less frequent (*p*=0.002) in *S-SpA* than *SpA*. Articular manifestations in *S-SpA* and *SpA* patients showed similar therapeutic responses to corticosteroids, cDMARDs and TNFα-inhibitors (*p*>0.05).

When considering the 45 subjects classified as SD (*SD cohort* n=36; *S-SpA cohort* n=9), a partial response to corticosteroids in the systemic phase could significantly predict the development of SpA (*p*=0.001; OR=18.2).

**Conclusion:** SpA should be ruled out in patients with unexplained systemic autoinflammatory manifestations, not only in the context of USAID but also in difficult-to-treat SD. Sacroiliac MRI scans may be more valuable than spine scans in detecting the typical signs of inflammation in S-SpA.

**Patient Consent**

Not applicable (there are no patient data)

**Disclosure of Interest**

C. Gaggiano Grant / Research Support with: This project received funding from the EU Horizon 2020 research and innovation programme under the EJP RD COFUND-EJP N° 825575., M. Zajc Avramovič: None declared, A. Vitale: None declared, N. Emeršič: None declared, J. Sota: None declared, N. Toplak: None declared, S. Gentileschi: None declared, Š. Blazina: None declared, M. Tarsia: None declared, G. Markelj: None declared, S. Telesca: None declared, V. Parretti: None declared, T. Vesel Tajnšek: None declared, C. Fabiani: None declared, A. Koren Jeverica: None declared, B. Frediani: None declared, L. Cantarini: None declared, T. Avčin: None declared

**References**

1. Vitale A, et al. Front Med (Rheumatology). Accepted on 9.5.2023. DOI: 10.3389/fmed.2023.1195995
2. Mitrovic S, et al. Rheumatology (Oxford). 2022;61(6):2535-2547. DOI: 10.1093/rheumatology/keab726

## P327 Subclinical enthesitis in enthesitis-related arthritis and sacroiliitis associated with familial mediterranean fever

### S. Sener^1^, E. Atalay^1^, A. E. Yildiz^2^, O. Basaran^1^, E. D. Batu^1^, Y. Bilginer^1^, S. Ozen^1^

#### ^1^Pediatric Rheumatology; ^2^Radiology, Hacettepe University, Ankara, Türkiye

##### **Correspondence:** S. Sener

*Pediatric Rheumatology 2023*, **21(Suppl 2):**P327

**Introduction:** Depending on the involved region, the most common enthesitis-related symptoms are localized pain, tenderness, and swelling. However, enthesitis can sometimes be asymptomatic and may be overlooked in routine physical assessments.

**Objectives:** In this study, we investigated the presence of subclinical enthesitis by ultrasonography (US) in asymptomatic patients with enthesitis-related arthritis (ERA) and sacroiliitis associated with familial Mediterranean fever (FMF).

**Methods:** A total of 50 patients, including 30 patients with ERA and 15 with sacroiliitis associated with FMF were included in the study. All patients were evaluated with the US by a pediatric radiologist. Enthesis of seven tendons (common extensor and flexor tendons, quadriceps tendon, proximal and distal patellar tendon, Achilles tendon, and plantar fascia) were examined on both sides.

**Results:** The median (IQR) age of the patients at diagnosis and at the time of US assessment were 10.2 (5.6) and 14.2 (9.2) years, respectively (M/F:1.8). Thirty-nine patients (78%) were receiving anti-inflammatory treatment and all patients were in clinical remission at the time of US assessment. Subclinical enthesitis was detected in 10 ERA (33.3%) and three FMF (20%) patients. Enthesitis was radiologically diagnosed in 16 (2.3%) out of 700 evaluated entheseal sites. The most frequent sites of enthesitis were Achilles (37.5%) and quadriceps (31.3%) tendons. These patients had no active complaints and acute phase reactants were within normal limits. Therefore, the patients were followed up without treatment change. However, a disease flare-up was observed in three of these patients (23.1%) during the follow-up, and their treatments were intensified.

**Conclusion:** Our results showed that the US can be particularly helpful in detecting subclinical enthesitis and predicting disease flare-ups in ERA and sacroiliitis associated with FMF patients.

**Patient Consent**

Yes, I received consent

**Disclosure of Interest**

None declared

**References**

1. Jousse-Joulin S, Breton S, Cangemi C, Fenoll B, Bressolette L, De Parscau L, et al. Ultrasonography for detecting enthesitis in juvenile idiopathic arthritis. Arthritis Care Res (Hoboken) 2011;63(6):849-55. doi: 10.1002/acr.20444.
2. Shenoy S, Aggarwal A. Sonologic enthesitis in children with enthesitis-related arthritis. Clin Exp Rheumatol 2016;34(1):143-7.

## P328 Investigating the role of biopsychosocial features in disease management of individuals diagnosed with era

### M. O. Tüfekçi^1^, S. Buran^2^, N. B. Karaca^1^, E. Aliyev^3^, Y. Bayındır^3^, Y. Bilginer^3^, E. Ünal^2^, S. Özen^3^

#### ^1^Department of Basic Physiotherapy and Rehabilitation, Hacettepe University Institute of Health Sciences; ^2^Department of Heart and Respiratory Physiotherapy and Rehabilitation, Hacettepe University Faculty of Physical Therapy and Rehabilitation; ^3^Department of Pediatrics, Division of Rheumatology, Hacettepe University Faculty of Medicine, Ankara, Türkiye

##### **Correspondence:** M. O. Tüfekçi

*Pediatric Rheumatology 2023*, **21(Suppl 2):**P328

**Introduction:** Considering the symptoms of pain, poor physical function and quality of life in individuals with enthesitis-related arthritis (ERA), physical and psychosocial evaluation of these individuals are necessary for disease management (1-3).

**Objectives:** The aim of our study was to examine the interactions of biopsychosocial features of individuals diagnosed with ERA.

**Methods:** The study included 20 individuals (7 girls, 13 boys) diagnosed with ERA who were followed up with routine controls in Hacettepe University Pediatric Rheumatology Department and demographic and clinical information were obtained. The Juvenile Arthritis Biopsychosocial and Clinical Questionnaire (JAB-Q), developed with the Delphi technique, assesses the biopsychosocial features of individuals with juvenile arthritis holistically through family, child, and clinician forms. The JAB-Q-child (patient) form includes self-reported features such as pain severity, disease activity, joint status, functionality, psychosocial status, performance in school, and fatigue. In our study, the JAB-Q-child (patient) form was used to evaluate the biopsychosocial features of individuals (2).

**Results:** The mean age of the individuals was 15.80 ± 2.57 years and the median (min-max) disease activity score BASDAI values were 0.35 (0-4.3). JAB-Q-child form median (min-max) value 21.5 (0-57) values for subheadings: pain severity 2 (0-5), disease activity 1.5 (0-6), joint status 0.5 (0-4), functionality 0 (0-8), psychosocial status 11 (0-31), performance in school 0 (0-1), fatigue 3.5 (0-14). The correlations between JAB-Q-child form total score and pain severity, disease activity and psychosocial status (rho=0.873, p<0.001; rho=0.821, p<0.001; rho=0.884, p<0.001, respectively) were very high; the correlations between functional status and fatigue (rho=0.650, p=0.002; rho=0.766, p<0.001, respectively) were high, while no relationship was found between joint status and performance in school (rho=0.387, p=0.092; rho=0.221, p=0.348, respectively) and there was no correlation between self-reported disease activity and BASDAI scores (rho=-0.020, p=0.938). In addition, high and very high correlations were observed between the subheadings of the scale (p<0.05).

**Conclusion:** Many significant relationships were found between the biopsychosocial features of individuals diagnosed with ERA. The total score of the JAB-Q-child form was most associated with psychosocial status and pain severity and least with disease activity and performance in school. Children's functionality was affected by pain severity, disease activity, and joint status, while their psychosocial status was related to pain severity and self-reported disease activity. These interactions were associated with ERA disease and emphasized the importance of biopsychosocial features of individuals in their management. The results obtained from the study were interpreted as the need for holistic approaches that take these features into account.

**Patient Consent**

Not applicable (there are no patient data)

**Disclosure of Interest**

None declared

**References**

1. Taxter AJ, Wileyto EP, Behrens EM, Weiss PF. Patient-reported Outcomes across Categories of Juvenile Idiopathic Arthritis. The Journal of Rheumatology. 2015;42(10):1914-21.
2. Unal E, Batu ED, Sonmez HE, Arici ZS, Arin G, Karaca NB, et al. A new biopsychosocial and clinical questionnaire to assess juvenile idiopathic arthritis: JAB-Q. Rheumatology International. 2018;38(8):1557-64.
3. Weiss PF, Beukelman T, Schanberg LE, Kimura Y, Colbert RA. Enthesitis-related Arthritis Is Associated with Higher Pain Intensity and Poorer Health Status in Comparison with Other Categories of Juvenile Idiopathic Arthritis: The Childhood Arthritis and Rheumatology Research Alliance Registry. The Journal of Rheumatology. 2012;39(12):2341-51.

## P329 Are coronary artery abnormalities pathognomonic of Kawasaki disease? – sepsis mouse model and literature review

### S.-Y. Lee, S. Lee, J. W. Rhim, D. C. Jeong

#### Department of Pediatrics, The Catholic University of Korea, Seoul, Korea, Republic Of

##### **Correspondence:** S. Lee

*Pediatric Rheumatology 2023*, **21(Suppl 2):**P329

**Introduction:** We hypothesize that coronary artery abnormalities (CAAs) are not pathognomonic of Kawasaki disease (KD), but may be observed in children with various diseases that cause severe systemic inflammation.

**Objectives:** To observe CAAs in a murine model of sepsis and to review the literature supporting our hypothesis.

**Methods:** To induce sepsis, 6-week-old C57BL/6 mice were intraperitoneally injected with endotoxin on days 0, 2, 5, 7, and 9. Histological findings of the major organs (i.e., heart, liver, and kidney) were compared between the sepsis and the control. The hearts of the septic mice were further examined to observe CAAs. A PubMed search was performed for literature review.

**Results:** Infiltrating inflammatory cells were relatively increased in the heart, liver, and kidneys of the sepsis group, compared with those of the control group. Lymphocytic infiltration was identified in pericardial soft tissue and myocardium (myocarditis) of septic mice. Coronary arteries were found, but no CAAs were observed in septic mice. A literature review has demonstrated the presence of CAAs in a variety of childhood diseases: Epstein-Barr virus or cytomegalovirus infection, rabies, Escherichia coli sepsis, toxic shock syndrome, viral myocarditis, Takayasu arteritis, juvenile idiopathic arthritis, rheumatic fever, leukemia, and hemophagocytic lymphohistiocytosis.

**Conclusion:** We did not observe CAAs in a murine model of sepsis. However, we found many studies of CAAs development in childhood diseases other than KD. The presence of CAAs may indicate the severity of the inflammation rather than the cause of the inflammation. Subsequent studies are needed to evaluate the clinical significance of CAAs in children.

**Patient Consent**

Not applicable (there are no patient data)

**Disclosure of Interest**

None declared

**References**

1. This study was approved by the Animal Research Ethics Committee, College of Medicine, The Catholic University of Korea (approval number: CIMH-2013-007).

## P330 Comparison of an "On-Demand" Canakinumab regimen with fixed-frequency Canakinumab in treating colchicine-resistant familial Mediterranean fever in children: a multicenter study

### K. Shehade^1^, Y. Levinsky^2^, T. Zuabi^3^, S. Kagan^3^, G. Amarilyo^2^

#### ^1^Tel Aviv University, Tel Aviv; ^2^Pediatric Rheumatology Unit; ^3^Pediatrics B, Schneider children's Medical Center of Israel, Petah Tikva, Israel

##### **Correspondence:** Y. Levinsky

*Pediatric Rheumatology 2023*, **21(Suppl 2):**P330

**Introduction:** Canakinumab, an inhibitor of interleukin-1 (IL-1b), has shown to be safe and effective in preventing attacks of familial Mediterranean fever (FMF) in individuals with colchicine-resistant (crFMF). The manufacturer recommends monthly subcutaneous injections as the standard prescription. Nevertheless, a specific group of our patients receives treatment through an "on-demand canakinumab" (COD) strategy, which involves longer intervals between drug administrations.

**Objectives:** This multicenter study aimed to compare the disease activity and drug safety between an "on-demand canakinumab" (COD) strategy and a "fixed-frequency canakinumab" (CFF) strategy for the treatment of children with colchicine-resistant familial Mediterranean fever (crFMF).

**Methods:** A retrospective analysis was conducted using data collected from three Israeli pediatric rheumatology centers. The study included children under 18 years of age with crFMF who received canakinumab treatment. Demographic parameters, clinical characteristics, cumulative drug dosages, and adverse events were compared between children treated with COD and CFF strategies.

**Results:** Out of the 51 children included in the study, 25 (49%) were treated with the COD strategy, while 26 received the CFF strategy. The demographic parameters and most disease features did not significantly differ between the two groups. Both strategies demonstrated a significant reduction in FMF attacks after the introduction of canakinumab. The median number of attacks per month did not significantly differ between the COD and CFF groups (0.33 (0.08, 0.58) vs. 0.13 (0, 0.5), respectively, P=0.485). However, the mean monthly dose was lower in the COD group compared to the CFF group (1.13±1.13 vs. 3.16±1.46 mg/kg, p < 0.001). Adverse events were similar between the two groups.

**Conclusion:** The COD strategy for individuals with crFMF achieved comparable efficacy and safety to the CFF strategy, while requiring a lower accumulated dose of canakinumab. This suggests that the COD regimen may be a less immunosuppressive and more cost-effective approach for treating children with colchicine-resistant familial Mediterranean fever.

**Patient Consent**

Not applicable (there are no patient data)

**Disclosure of Interest**

None declared

**References**

1. Arnold DD, Yalamanoglu A, Boyman O. Systematic Review of Safety and Efficacy of IL-1-Targeted Biologics in Treating Immune-Mediated Disorders. Front Immunol. 2022 Jul 6;13:888392.
2. Ozdogan H, Ugurlu S. Canakinumab for the treatment of familial Mediterranean fever. Expert Rev Clin Immunol. 2017 May;13(5):393-404. doi: 10.1080/1744666X.2017.1313116. Epub 2017 Apr 10.
3. Yücel BB, Aydog O, Nalcacioglu H, Yılmaz A. Effectiveness of Canakinumab Treatment in Colchicine Resistant Familial Mediterranean Fever Cases. Front Pediatr. 2021 Sep 10;9:710501.

## P331 Haploinsufficiency A20: a novel mutation with unusual presentation

### L. Salazar^1^, S. S. S. Rodrigues^2^, J. B. Lima^1^, R. Almeida^3^, S. Alves^4^, C. Zilhão^4^

#### ^1^Pediatrics, Centro Hospitalar e Universitário de Santo António, Porto; ^2^Pediatrics, Centro Hospitalar Entre Douro e Vouga, Santa Maria Feira; ^3^Pediatrics, Unidade Local de Saúde de Matosinhos, Matosinhos; ^4^Pediatric Rheumathology, Centro Hospitalar e Universitário de Santo António, Porto, Portugal

##### **Correspondence:** J. B. Lima

*Pediatric Rheumatology 2023*, **21(Suppl 2):**P331

**Introduction:** A20 is a protein encoded by the tumor necrosis factor alpha-induced protein-3 gene (TNFAIP3), that plays a key role in the inhibition of pro-inflammatory molecules. Loss of function mutations in this gene, causing A20 haploinsufficiency, lead to Behçet-like phenotypes, although disease manifestations may vary widely.

**Results:** A 15-year-old female with history of “PFAPA-like” episodes from 3 to 5 years of age, presented with a 12-month history of recurrent mensal episodes of fever, asthenia, anorexia, odynophagia, cervical lymphadenopathies and abdominal pain, lasting up to 3 days. She also reported genital aphthous lesions in 2 events. Additionally, she mentioned a 2-month history of right omalgia, with periods of ipsilateral intermittent clavicular tumefaction. Familiar history was irrelevant. Complementary investigation revealed a moderate increase in inflammatory markers (maximum CRP 121mg/L and serum amyloid A 8 mg/dL), with normalization between episodes. Bone MRI and scintigraphy showed signs of sternoclavicular inflammatory arthropathy. Arthrocentesis was performed, showing negative cultural exams and no neoplastic cells on anatomopathological evaluation. Considering the possibility of non-bacterial chronic osteomyelitis (CNO), treatment with naproxen was started.

In the following months, although partial improvement in sternoclavicular arthritis, recurrent fever persisted with reported oral ulcers in one episode. Immunogenetic study revealed HLA-B15 positivity. Considering a possible overlap of CNO with Behçet’s disease, colchicine was initiated.

As episodes kept occurring monthly, the patient underwent treatment with azathioprine, up to a maximum dose of 100 mg/day (~2 mg/kg/day). After 6 months of disease improvement (less intense and frequent attacks), crisis reemerged with the prior periodicity, with sternoclavicular arthritis worsening in episodes. Next generation sequencing panel for autoinflammatory diseases revealed a novel heterozygotic variant c.1632T>C in the TNFAIP3 gene. Parents genetic testing is currently pending. Treatment with adalimumab was initiated with complete resolution of inflammatory episodes and sternoclavicular arthritis - currently with 4 months follow-up.

**Conclusion:** A20 haploinsufficiency is a diagnosis with growing recognition as an important monogenic mimic of Behçet’s disease. Genetic evaluation should therefore be considered in atypical cases. Although we report a novel mutation, and while sternoclavicular arthritis is not a described feature of this disease, we believe it may be connected with the inflammatory bursts. Parents genetic evaluation will bring us new insight regarding this matter.

**Patient Consent**

Yes, I received consent

**Disclosure of Interest**

None declared

## P332 Familial Behçet-like autoinflammatory disease-3 (AIFBL3), caused by heterozygous mutation in the rela gene: a case report

### M. C. Maggio^1,2^, C. Castana^2^, C. Maltese^1^, F. Sferlazza^1^, F. Munna^1^, G. La Cagnina^1^, G. Corsello^1,2^

#### ^1^University Department PROMISE “G. D’Alessandro”, University of Palermo; ^2^ARNAS, Palermo, Paediatric Clinic, Children Hospital "G. Di Cristina", Palermo, Italy

##### **Correspondence:** M. C. Maggio

*Pediatric Rheumatology 2023*, **21(Suppl 2):**P332

**Introduction:** The autoinflammatory features of Behçet's disease (BD) and the role of innate immunity dysregulation have been highlighted and BD can be considered as the crossroad of autoinflammatory and autoimmune diseases.

**Objectives:** We describe the case of a 9-year-old caucasic male, who presented at age 6 y with recurrent episodes of fever, oral ulcers and pain at the limbs, hands, wrists. At the physical examination the child showed functional limitation of flexion and extension movements of the wrists (left > right) and a bilateral mild joint stiffness of the shoulders. He showed a mild delay in the stages of psychomotor development, and a mild hypotrophy of the muscles of the lower limbs.

**Methods:** The metabolic disease expert excluded metabolic diseases, based on the metabolic diagnostic investigations. Ultrasound documented knees joint effusion in the lateral supra-patellar seat with synovial membrane's thickening and evident right knee synovial phlogosis, minimal on the left. A Whole body MRI, reported intra joint fluid effusion in external lateral seat and in sub patellar seat of the left knee. Intraspongious edema of the cuboid of the right foot. The eye examination with slit lamp was normal; HLA-B27, Anti-streptolysin O titer, pharyngeal swab and specific serologies for infectious diseases were negative. Fecal calprotectin was normal. Antinuclear antibodies (ANA) were positive 1:320 with a granular pattern.

**Results:**

The genetic study in NGS for autoinflammatory diseases revealed a heterozygous mutation, defined as VUS, of the RELA gene: c.1537C>G (p.Pro513Ala). Mutations of the RELA gene are associated with a familiar autoinflammatory disease Behçet's disease (BD)-like type 3, with an autosomal dominant transmission. The Familial Behçet-like autoinflammatory disease-3 (AIFBL3), caused by heterozygous mutation in the RELA gene on chromosome 11q13, is characterized predominantly by chronic mucocutaneous ulceration.

**Conclusion:** The patient did not yet fulfil the paediatric BD (PEDBD) nor ICBD criteria for the diagnosis of paediatric BD, however it is well described that BD is an evolutionary disease, and clinical manifestations may appear over the years (1-3).

Monogenic BD-like conditions are increasingly recognized and to date have been found to predominantly involve loss-of-function variants in TNFAIP3. This case describes a child carrying the RELA gene mutation, with clinical symptoms evoking BD. The RELA gene mutations are conditions related to dysregulated NF-κB activation and need a strict follow-up and a prompt start of treatment, also in patients who do not fulfil the diagnostic criteria for BD.

**Patient Consent**

Yes, I received consent

**Disclosure of Interest**

None declared

**References**

1. Gaggiano C, et al. Intern Emerg Med. 2023 Apr;18(3):743-754.
2. Gallizzi R, et al. Pediatr Rheumatol Online J. 2017 Dec 21;15(1):84.
3. Vitale A, et al. Intern Emerg Med. 2022 Oct;17(7):1977-1986.

## P333 BD-like disease associated with TNFAIP3 gene mutation: a case report

### M. C. Maggio^1,2^, M. M. D'Alessandro^3^, C. Corrado^3^, F. Sferlazza^1^, G. La Cagnina^1^, F. Munna^1^, C. Maltese^1^, G. Corsello^1,2^

#### ^1^University Department PROMISE “G. D’Alessandro”, University of Palermo; ^2^ARNAS, Palermo, Paediatric Clinic, Children Hospital "G. Di Cristina"; ^3^ARNAS Palermo, O.U. of Paediatric Nephrology and Dialysis, Children Hospital “G. di Cristina”, Palermo, Italy

##### **Correspondence:** M. C. Maggio

*Pediatric Rheumatology 2023*, **21(Suppl 2):**P333

**Introduction:** Behçet's disease (BD) is a chronic, multifactorial systemic vasculitis with a strict link with autoinflammatory and autoimmune diseases. It can affect various organs and tissues, with recurrent oral and genital ulcers, skin lesions, joint pain and swelling (1), eye inflammation, gastrointestinal disease (2-3). Monogenic BD is a rare subtype of the disease, shows an earlier age of onset and a more severe disease course. One gene associated with monogenic BD is TNFAIP3, encoding A20, a protein regulating inflammation and immune response. Loss-of-function mutation in TNFAIP3 triggers a new autoinflammatory disease: HA20, characterized by a wide range of clinical pictures, caused by chronic inflammation, as BD.

**Objectives:** We describe the case of a 11-year-old boy firstly diagnosed at the age of 9 years, with hypertension, secondary to renal arteries stenosis, ascending aortic ectasia, celiac trunk ectasia, superior mesenteric artery stenosis, documented by ecocolordoppler, angio-MRI and angio-TC. The first suspected diagnosis was Takayasu arteritis.

The mother received the diagnosis of SLE, the maternal grandmother had Moschowitz disease.

**Methods:** For the start of limbs pain, periodic attacks of fever, oral aphthae he was referred to the Pediatric Rheumatology unit. He did not present swelling of knees, ankles, wrists, fingers, conformed by echography.

**Results:** MRI documented bilateral sacroiliitis, confirmed the preexisting vascular lesions, showed slightly thickened walls of sigma, with signs of inflammation. The eye examination with slit lamp was normal.

He showed positive ENA, anti-C1q, anti-cardiolipin, anti-thyroglobulin, anti-thyroid peroxidase antibodies. SAA 22 (nv<6). HLA-B51 is absent.

The genetic molecular analysis of autoinflammatory diseases, showed a heterozygous variant of TNFAIP3 classified as VUS (p.Ala545Val) with maternal segregation. Pathogenetic variants of TNFAIP3 gene are related to a dominant inherited familial autoinflammatory syndrome BD-like.

However, despite the severity of the clinical picture, the patient did not yet meet neither ICBD, nor ISG, nor PEDBD diagnostic criteria. However, the genetic background, the family history and the severe vasculitis guide the diagnosis in this challenging case.

**Conclusion:** The correlation between TNFAIP3 and Takayasu or SLE was recently proposed, however, our patient developed clinical signs, as bowel inflammation and sacroiliitis, supporting to the diagnosis of BD. Genetic counseling may be recommended for patients with monogenic BD. The diagnosis of monogenic BD should be made by a qualified healthcare team, based on a comprehensive evaluation of the patient's symptoms, medical history, physical examination, and genetic testing.

**Trial registration identifying number:**

**Patient Consent**

Yes, I received consent

**Disclosure of Interest**

None declared

**References**

1. Gaggiano C, et al. Intern Emerg Med. 2023 Apr;18(3):743-754.
2. Gallizzi R, et al. Pediatr Rheumatol Online J. 2017 Dec 21;15(1):84.
3. Vitale A, et al. Intern Emerg Med. 2022 Oct;17(7):1977-1986.

## P334 The thousand faces of mevalonate kinase deficiency: a challenging case

### G. Corsello^1,2^, C. Castana^2^, M. Caserta^2^, R. Bonadia^1^, B. Gramaglia^1^, D. Bacile^1^, S. Cancila^1^, E. Catania^1^, M. C. Maggio^1,2^

#### ^1^University Department PROMISE “G. D’Alessandro”, University of Palermo; ^2^ARNAS, Palermo, Paediatric Clinic, Children Hospital "G. Di Cristina", Palermo, Italy

##### **Correspondence:** M. C. Maggio

*Pediatric Rheumatology 2023*, **21(Suppl 2):**P334

**Introduction:** Mevalonate Kinase Deficiency is a rare inborn error of metabolism with an autosomal recessive inheritance due to mutations in the *MVK* gene. Residual enzymatic activity between 1 and 10% is associated to hyperimmunoglobulinemia D syndrome (HIDS) (characterized by an auto-inflammatory phenotype), while, if enzymatical activity is about 0%, it can cause neurological involvement (Mevalonic aciduria: MVA) (1-2).

**Objectives:** The diagnosis is based on the detection of elevated mevalonic acid levels in urine, even in the attack-free interval. This biochemical determination differentiates patients with MVA from patients with HIDS. The genetic study of the *MVK* gene confirm the diagnosis. Therapeutic options include non-steroidal anti-inflammatory drugs while more severe cases require therapy with IL-1beta or IL-1 receptor antagonists.

**Methods:** We describe the long-term follow-up of an adolescent with MVA, followed by our Metabolic Diseases Unit.

**Results:** The patient was born at 30 weeks with a birth weight of 2100g. He was admitted to neonatal intensive care unit for maladaptation to extrauterine life and respiratory distress. He showed frequent febrile episodes associated with diarrhea, bowel occlusion and hepato-splenomegaly. Laboratory findings showed anemia, leukocytosis with neutrophilia, thrombocytopenia, elevated levels of CRP, ALT, AST and ESR. After discharge, the patient showed reduced growth velocity, recurrent febrile episodes, arthritis, and vasculitis skin manifestations. After excluding infectious enteritis, auto-immune diseases, etc, at 17 months of age, the child underwent a metabolic disease specialist evaluation at our hospital. He showed elevated urinary levels of mevalonic acid and mevalonolactone; thus, a diagnosis of MVA was considered, confirmed by the genetic study, showing the homozygous c.709A>T substitution in the *MVK* gene, with a consequent p.7237S aminoacidic substitution.

Therefore, we started a therapy with NSAIDs, ubiquinone, anti-leukotriene, vitamin C and E. Moreover, we suggested systemic corticosteroid therapy for possible acute crises (methylprednisolone 2mg/kg). Given the poor clinical conditions, the neurologic impairment and the frequent febrile episodes, corticosteroid therapy was needed with an increased frequency and with higher dosage, developing steroid-dependence. He switched to anakinra, with a prompt improvement of clinical conditions. Unfortunately, a month after starting daily administration of anakinra, the patient had an adverse event which required discontinuing the therapy, which was then switched to canakinumab.

**Conclusion:** The patient is currently 18 years old and is still treated with canakinumab, with a good disease control, a satisfactory growth and neuropsychologic development. The long-term follow-up of these rare cases is useful to highlight the effectiveness of anti-IL1 treatment not only to control the attacks of the disease, but also to ensure psychomotor development, height velocity and optimize prognosis and quality of life.

**Patient Consent**

Yes, I received consent

**Disclosure of Interest**

None declared

## P335 Mevalonate kinase deficiency: early presentation caused by a rare homozygous mutation in the MVK gene

### C. Martin^1^, C. Boulanger^2,3^

#### ^1^Université Catholique de Louvain, ^2^Department of Pediatric Hematology and Oncology, Cliniques Universitaires Saint-Luc, ^3^Institut de Recherches Expérimentales et Cliniques, Université Catholique de Louvain, Brussels, Belgium

##### **Correspondence:** C. Martin

*Pediatric Rheumatology 2023*, **21(Suppl 2):**P335

**Introduction:** Mevalonate Kinase Deficiency (MKD) is a rare autosomal recessive autoinflammatory disease due to a *MVK* gene mutation. The phenotype is variable, from Periodic Fever Syndrome (PFS) to Mevalonic Aciduria (MA) and treatment remains a challenge depending on the clinical features.

**Methods:** We describe a case of a female neonate, full term from non-consanguineous African healthy parents. Fever, with ascites, transient tachypnea started the first days of life and lasted intermittently for several weeks. Despite receiving broad-spectrum antibiotics, the neonate continued to have flares. Further investigations revealed mevalonic aciduria and genetic tests confirmed the homozygous *MVK* c.346T>C, p.Tyr116His pathogenic variant. Anakinra was started with adequate response except for persistent high mevalonic aciduria. From her 6 months, she developed a very early onset inflammatory bowel disease (VEO-IBD) with recurrent life-threatening hemorrhagic ulcerative colitis. Association of immunosuppressive drugs with corticosteroids, anakinra/canakinumab, adalimumab, and methotrexate offered long-term remission of VEO-IBD and inflammation, again except mevalonic aciduria. However, no neurologic disorder or development delay were found apart from mild delay gait acquisition.

**Results:** The pattern for the PFS is characterized by fever attacks associated with systemic inflammatory reaction. Mevalonic aciduria, a severe metabolic disease, manifests as systemic inflammation and is usually accompanied by dysmorphic features, retinopathy, enteropathy and neurologic disorders. Biological tests reveal inflammatory markers, and constant elevated urinary mevalonic acid levels, even in the absence of a flare-up in MA.

To our knowledge, our patient is the second homozygote *MVK* c.346T>C. The first case showed hepatitis, developmental delay and inflammatory flares. Our patient’s auto-inflammatory manifestations associated to the persistent mevalonic aciduria, in the absence of dysmorphic features and neurologic disorders, argue for a continuum between PFD and MA. Furthermore, as described in a small serie, MVK deficiency can also mimics VEO-IBD with generally an insufficient response to the anti-TNF treatment. Our patient had an inadequate response to the anti-Il1b agent and successful results were obtained with combination of immunosuppressive therapies including corticosteroids, high doses of adalimumab and anakinra, and methotrexate. No severe side effects, such as infection, were noted and catch-up was observed.

**Conclusion:** Our case suggests that the c.346T>C homozygous mutation in *MVK* could lead to a severe early phenotype an suggests a continuum between PFS and MA. MKD should be considered in feverish patient with VEO-IBD to reduce diagnostic delay and improve outcome with early initiation of tailored treatments.

**Patient Consent**

Yes, I received consent

**Disclosure of Interest**

None declared

## P336 Chronic recurrent multifocal osteomyelitis associated to inflammatory bowel diseases: a neglected association

### C. Matucci-Cerinic^1,2^, C. Montobbio^1^, C. Olcese^1^, C. Longo^1^, S. Arrigo^3^, M. Gattorno^2^, C. Malattia^1,2^

#### ^1^DINOGMI, University of Genoa; ^2^UOC Rheumatology and autoinflammatory diseases; ^3^Pediatric Gastroenterology and Endoscopy, IRCCS Istituto Giannina Gaslini, Genoa, Italy

##### **Correspondence:** C. Matucci-Cerinic

*Pediatric Rheumatology 2023*, **21(Suppl 2):**P336

**Introduction:** Chronic recurrent multifocal osteomyelitis (CRMO) is a rare inflammatory disease characterized by multiple sterile bone lesions that typically involve the metaphysis of the long bones and the axial skeleton. Association of CRMO with Inflammatory Bowel Diseases (IBD), has been reported in some cases. However, still little is known about the topic.

**Objectives:** to evaluate the prevalence of IBD in a cohort of CRMO patients and to describe their clinical, serological and radiological characteristics and response to treatments.

**Methods:** the clinical, serological and radiological characteristics of the CRMO patients with an IBD (Crohn’s disease (CD), ulcerative colitis (UC), U-IBD (undifferentiated IBD) followed at the Istituto Giannina Gaslini were retrospectively reviewed.

**Results:** 12 patients with CRMO and associated IBD were found. 83.3% of patients were male, and in 91.6% of cases, the disease onset was characterized by CRMO symptoms, while in one case osteoarticular and GI symptoms were simultaneous. The mean age at CRMO onset and diagnosis was of 10.38 years and 11.78 years respectively, while the age at IBD onset and diagnosis was of 10.9 and 11.7 respectively. All patients presented a metaphyseal involvement of the long bones of the lower limbs, while upper limbs were involved in 33.3% of patients. Pelvic involvement was present in 66% of patients (n=8), sacroiliitis in 41.6 % (n=5) and vertebral involvement in 25% (n=3) of patients. IBD symptoms were present in 58.3 % of patients (n=7) and were characterized by abdominal pain (n=3), diarrhoea (n=5), haematochezia and weight loss (n=2). 8 patients presented CD, 1 patient UC and 3 IBD-U. 75% of patients (n=9) presented a microcytic anemia. All patients presented an elevation of the fecal calprotectine, while hemoccult was positive in 8 patients. Abdominal US showed a bowel thickening in 58.3% of patients (n=7). All patients presented a persistent elevation of CRP and ESR after CRMO treatment was started. At CRMO diagnosis all patients underwent a therapy with NSAIDS, associated in 2 cases to steroids, while one patient was started on methotrexate and one on etanercept. After IBD was diagnosed, the majority of patients underwent an oral steroid course. 6 patients were started on salazopirin, which resulted efficacious in half of patients. 4 patients responded to Adalimumab alone, while 5 achieved remission under the combination adalimumab-methotrexate.

**Conclusion:** This represents the larger cohort of IBD associated to CRMO reported in the literature, highlighting a possible underdiagnosis of this condition. Seen the frequent absence of intestinal symptoms, we suggest a screening with fecal calprotectin in all CRMO patients at disease onset and during follow-up, especially in those patients that present a persistent elevation of inflammatory parameters.

**Patient Consent**

Yes, I received consent

**Disclosure of Interest**

None declared

## P337 A case of bilateral chronic granulomatous uveitis and arthritis associated with genetic variant in the otulin gene

### A. Mauro^1^, A. Visioli^2^, V. Ansuini^1^, L. Giordano^3^, G. L. Chiaffoni^2^, R. Stefania^2^, F. Nosenzo^2^, R. Bertelli^4^, F. Zicarelli^5^, A. Invernizzi^5,6^, L. Bernardo^3^

#### ^1^Pediatric Rheumatology unit, Department of Pediatrics, Fatebenefratelli Hospital; ^2^Department of Pediatrics, Vittore Buzzi Children’s Hospital; ^3^Department of Pediatrics, Fatebenefratelli hospital, Milan; ^4^Centre for Autoinflammatory Disease and Primary Immunodeficiencies, Gaslini children's Hospital, Genoa; ^5^Eye Clinic- Department of Biomedical and Clinical Science, Sacco Hospital-University of Milan, Milan, Italy; ^6^Discipline Oftalmology, University of Sidney-Save the Sight Institute, Sidney, Australia

##### **Correspondence:** A. Mauro

*Pediatric Rheumatology 2023*, **21(Suppl 2):**P337

**Introduction:** Uveitis is caused by infections, trauma or autoimmune diseases. Symptoms can vary, ranging from asymptomatic to severe and it can cause irreversible blindness.

**Objectives:** We describe a case of chronic granulomatous uveitis associated with arthritis.

**Methods:** Y is a healthy Egyptian boy, at the age of 6 he presented the first episode of bilateral conjunctival hyperaemia and photophobia and was diagnosed with severe bilateral granulomatous uveitis, complicated with synechiae. Family history was negative for autoimmune diseases. Treatment with Dexamethasone (1 drop/eye every 2 hours) and Tropicamide (1 drops/eye 3/day) was administered, with benefit. Rheumatological consultation did not detect rash, ulcers or arthritis.

**Results:** Y was admitted to our department. Blood exams showed increased inflammatory markers (CRP 54.6 mg/L, ESR 120 mm/h), positive ANA autoantibodies (1:320, dotted pattern), negative ANCA, ENA. Negative HLA-B51 and HLA-B27. He tested negative for tuberculosis (quantiferon and Mantoux test, chest X-ray). Urine tests showed mild proteinuria but normal tubular excretion, letting us rule out the TINU diagnosis.

One year later he had the first flare of uveitis with conjunctival hyperaemia, photophobia and pain, treated with topical therapy. He quickly developed papillar and cystoid macular oedema: therapy with Prednisone (1 mg/kg/die) was started.

Concomitantly, the first episode of arthritis in the left ankle occurred, treated with Naproxen (15 mg/kg/day). Given the association of uveitis and arthritis, Methotrexate (15 mg/m2/ week) was started. After 3 months of poor response to Methotrexate, therapy with Adalimumab (20 mg/2 weeks) was started, with benefit.

**Conclusion:** Due to simultaneous bilateral granulomatous uveitis and arthritis, Y. underwent genetic investigation for mutation of the NOD2 gene, responsible for Blau Syndrome, that was negative. It revealed a variant of uncertain significance (OTULIN Gin115His), which has only been described once in literature1 in a patient affected by chronic intestinal inflammation. Study of genetic mosaicism in NOD2 gene is now ongoing.

**Patient Consent**

Yes, I received consent

**Disclosure of Interest**

None declared.

**References**

1. Marzano AV, Genovese G, Moltrasio C, Tricarico PM, Gratton R, Piaserico S, Garcovich S, Boniotto M, Brandão L, Moura R, Crovella S. Whole-Exome Sequencing in 10 Unrelated Patients with Syndromic Hidradenitis Suppurativa: A Preliminary Step for a Genotype-Phenotype Correlation. Dermatology. 2022;238:860-869. doi: 10.1159/000521263.

## P338 Use of Canakinumab for the treatment of auto-inflammatory disorders: experience from a UK tertiary paediatric rheumatology centre

### D. McLaughlin^1^, S. Hambleton ^2^, L. Craig^1^, J. Hutchinson^1^, K. Seed^1^, S. Jandial^1^

#### ^1^Paediatric Rheumatology; ^2^Immunology, Great North Children's Hospital, Newcastle Upon Tyne, United Kingdom

##### **Correspondence:** D. McLaughlin

*Pediatric Rheumatology 2023*, **21(Suppl 2):**P338

**Introduction:** NHS England clinical commissioning policies have now allowed canakinumab, an IL-1 inhibitor, to be administered by specialist teams outside of the national auto-inflammatory centre, for patients with Tumour necrosis factor Receptor Associated Periodic Syndrome (TRAPS), Familial Mediterranean Fever (FMF) and Cryopyrin Associated Periodic Syndrome (CAPS).^1-3^

**Objectives:** To determine the efficacy, safety and patient-parental satisfaction of canakinumab use in paediatric patients with auto-inflammatory disorders.

**Methods:** Patients with an underlying auto-inflammatory condition who received canakinumab, under NHS England clinical commissioning policy, were identified from electronic care records at a UK paediatric rheumatology service. Clinical information including genetic diagnosis, serum Amyloid A levels, treatments and clinical responses were extracted from patient records. A questionnaire was devised to gather the patient-parent perspective on canakinumab use and how this impacted on disease control and quality of life (QOL).

**Results:** 13 patients received canakinumab (2-4mg/kg 4-8 weekly) for treatment of CAPS (n=7), TRAPS (n=4) and FMF (n=2). 9 patients initially received anakinra (1-4mg/kg daily) and later switched to canakinumab; patients switched to canakinumab due to changes in NHS policy and not due to failure of anakinra. 1 patient had increased disease activity following change to canakinumab, despite increased dosing up to 4mg/kg 8 weekly, the patient was then switched back to anakinra after 10 months. The remaining 12 patients have continued on canakinumab; 10 without significant clinical flare or elevation in Amyloid A levels and 2 who have had a flare and rise in Amyloid A both triggered by lower respiratory tract infection. No side effect or other patient safety concerns related to canakinumab were found in any patient.

Patient-parent feedback on canakinumab use was overwhelmingly positive. No longer having to administer daily anakinra injections, less travelling time to national specialist centre for canakinumab administration (a 12 hour round trip journey for many patients), less disease flares and overall improved QOL were key feedback findings received.

**Conclusion:** In our cohort of patients who received canakinumab, the majority have had adequate disease control and importantly has had a positive impact on patient & family QOL. It was an important reminder in the 1 case where canakinumab failed that anakinra still remains a valuable therapeutic option. Future consideration to delivering canakinumab in the community setting outside of hospital would further improve patient-family satisfaction and reduce hospital clinic attendances.

**Patient Consent**

Not applicable (there are no patient data)

**Disclosure of Interest**

None declared

**References**

1. DeBenedetti F, Gattorno M, Anton J, Ben-Chetrit E, Frenkel J, Hoffman HM, et al. Canakinumab for the Treatment of Autoinflammatory Recurrent Fever Syndromes. New Eng j med. 2018;378(20):1908–19
2. NHS England: 2013/2014 Standard Contract for CAPS (All ages). [Internet] 2013 [cited 2023 May 5] Available: https://www.england.nhs.uk/wp-content/uploads/2013/06/e13-cryopyrin-ass-periodic-syn.pdf
3. NHS England Clinical Commissioning Policy: Canakinumab for treating periodic fever syndromes: TRAPS, HIDS/MKD and FMF (ages 2 years and older). [Internet] 2020 [cited 2023 May 5] Available: https://www.england.nhs.uk/wp-content/uploads/2020/03/Canakinumab-for-treating-periodic-fever-syndromes-TRAPS-HIDSMKD-and-FMF-ages-2-years-and-older.pdf

## P339 Sapho syndrome: are there differences between adults and children?

### A. T. Melo^1,2,3^, C. Matucci-Cerinic^4,5^, C. Zinterl^1,2,3^, E. Sousa^2,3^, N. Khmelinskii^2,3^, S. Barreira^2,3^, J. E. Fonseca^1,2,3^, F. O. Ramos^1,2,3^, M. Gattorno^5^

#### ^1^Pediatric Rheumatology Unit; ^2^Rheumatology Department, Hospital de Santa Maria, Centro Hospitalar Universitário Lisboa Norte, Centro Académico Médico de Lisboa; ^3^Rheumatology Investigation Unit, Instituto de Medicina Molecular, Faculdade de Medicina, Universidade de Lisboa, Centro Académico Médico de Lisboa, Lisbon, Portugal; ^4^DINOGMI, University of Genoa; ^5^UOC Rheumatology and Autoinflammatory diseases, IRCCS Istituto Giannina Gaslini, Genoa, Italy

##### **Correspondence:** A. T. Melo

*Pediatric Rheumatology 2023*, **21(Suppl 2):**P339

**Introduction:** SAPHO syndrome (synovitis, acne, pustulosis, hyperostosis, and osteitis) is a rare autoinflammatory disease characterized by osteoarticular and dermatological features. It can affect both adults and children and diagnosis and treatment are still a challenge. In children, it is characterized by chronic recurrent multifocal osteomyelitis (CRMO).

**Objectives:** To evaluate the clinical features and therapy options of SAPHO patients, while comparing the disease between children and adults.

**Methods:** Retrospective international observational study. Data on demographic variables, clinical features and treatment options were collected on patients with SAPHO diagnosis and currently followed at two tertiary European hospitals (Portugal and Italy). Patients were divided into 2 groups: a paediatric age onset group and an adult age onset group. Statistical analysis was done using SPSS 26.0, with a significance of p<0.05. Univariate analysis was performed using Fisher’s exact test and Mann-Whitney U test.

**Results:** Twenty-four patients were included, 17 children and 7 adults. Male gender was predominant in the paediatric group (n=10, 59%), with a median age at diagnosis of 14 [11-15.5] years and a median diagnostic delay of 1 [0-2.5] year. The female gender was more common in the adult group (n=4, 57%), with a median age at diagnosis of 49 [36-52] years and a median diagnostic delay of 5 [1-12] years.

Both in adults and children axial involvement was predominant [57% in adults (n=4), 71% in children (n=12], while there was a significant more frequent long bone involvement in children (88% vs 29%, p=0.009)

All patients presented with skin lesions that were the 1^st^ symptom in 3 adults (43%) and 8 children (47%). The most frequent cutaneous manifestation in adults was palmo-plantar pustulosis (PPP) (n=5, 71%), followed by hidradenitis suppurativa (HS) (n=2, 29%) and pyoderma gangrenosum (PG) (n=1, 14%). In children, acne was present in 59% of patients (n=10), PPP in 35% (n=6), HS in 24% (n=4) and 1 patient had also PG (2%).

Fever was only detected in paediatric patients (n=3, 18%). All patients had high acute phase reactants at the beginning of the disease.

Ten patients (of whom 9 were children) had a bone biopsy suggestive of SAPHO, while all others were diagnosed based only on clinical and radiological features.

The most frequent treatment in the adult group were non-steroidal anti-inflammatory drugs (NSAIDs) (n=6, 86%) and conventional disease modifying drugs (cDMARDs) (n=6, 86%). TNF inhibitors (iTNF) were used in 2 patients (29%) and bisphosphonates in 1 patient (14%). In the paediatric group, 15 patients (88%) were treated with NSAIDs and 11 (65%) with cDMARDs. Three patients (18%) were treated with bisphosphonates and 1 patient with small-molecule inhibitor of phosphodiesterase-4 (6%). Biological therapies (iTNF, IL-1 inhibitors and IL17/IL-23 inhibitors) were more commonly used in paediatric patients (n=9, 53%), with variable responses.

**Conclusion:** Long bone involvement represents the main feature of paediatric age onset SAPHO. Therapeutic options were similar in adults and children, with NSAIDs and cDMARDs being the most common treatment in both groups. This study confirms that childhood CRMO and adult SAPHO represent a continuum of the same disease.

**Patient Consent**

Yes, I received consent

**Disclosure of Interest**

None declared

## P340 Pediatric patients with chronic nonbacterial osteitis requiring transition to adult care: a longitudinal cohort study

### P. M. Miettunen^1,2^, L. Carlini^3^, A. Insalaco^4^, J. Anton^5^, J. Brunner^6^, G. E. Legger^7^, M. Cattalini^8^, E. Hoppenreijs^9^, M. Alessio^10^, A. Pistorio^3^, N. Ruperto^3^, R. Caorsi^3^, M. Gattorno^3^ on behalf of EUROFEVER and Pediatric Rheumatology International Trials Organization (PRINTO)

#### ^1^Pediatrics and Rheumatology, Alberta Children's Hospital; ^2^Pediatrics, University of Calgary, Calgary, Canada; ^3^Pediatric Rheumatology, Second Division of Pediatrics, Giannina Gaslini Institution, Genoa; ^4^Rheumatology, IRCCS Ospedale Pediatrico Bambino Gesù, Rome, Italy; ^5^Pediatric Rheumatology, Hospital Sant Joan de Déu, Universitat de Barcelona, Barcelona, Spain; ^6^Department of Pediatrics, Pediatric Rheumatology, Medical University Innsbruck, Innsbruck, Austria; ^7^Pediatrics, University of Groningen, University Medical Center Groningen, Groningen, Netherlands; ^8^Division of Immunology and Pediatric Rheumatology, Pediatric Clinic, University of Brescia, Spedali Civili, Brescia, Italy; ^9^Pediatric Rheumatology, Radboud UMC, Nijmegen, Netherlands; ^10^Pediatrics, Napoli Federico II University, Naples, Italy

##### **Correspondence:** P. M. Miettunen

*Pediatric Rheumatology 2023*, **21(Suppl 2):**P340

**Introduction:** Chronic non-bacterial osteomyelitis (CNO), also known as Chronic Recurrent Multifocal Osteomyelitis (CRMO) is an autoinflammatory disorder characterized by sterile bone osteolytic lesions with a remitting and relapsing course.

**Objectives:** To evaluate the prevalence of active disease over time in a multinational large series of CNO/CRMO patients enrolled in the Eurofever registry to help understand the need for transition care into adulthood.

**Methods:** Children from 19 countries who were < 18 years and diagnosed with CNO/CRMO were studied, and analysis of active disease was conducted in those who had follow-up visits and for whom treatment data was available. Remission was defined by the treating physician(s), and required discontinuation of therapy. Achievement of remission, number of flares, ongoing active disease and medications at final follow-up, were analyzed for patients > 10 years in 4 cohorts: a) 10- <14 years; b) 14-< 16 years; c) 16- <18 years and > 18 years.

**Results:** Complete disease activity information was available for 237 of 573 (41.4%) CNO/CRMO patients (138/237 [58.2 %] female). The median [IQ range] age at disease onset for this cohort was 10.07 [7.94-11.81] years. By imaging, (175/237 (73.8%)), monofocal disease was present in 37/175 (21.1%) and multifocal in 138/175 (78.9%) patients at baseline. At disease diagnosis, elevated ESR was present in 166/232 (71.5%) of patients, and elevated CRP in 136/233 (58.4%).The median [IQ range] duration of follow-up was 25.4 [12.4-44.1] months. Eighty seven out of 237 (37%) of patients **reached remission** at least once during follow-up. The mean (standard deviation [SD]) of **flares** per patient was 2.3 (1.7) and did not differ between the age cohorts.

The number of patients who were > 10 years at final follow-up was 189/237 (79.7%). Sixty nine out of these 189 (36.5%) children reached **remission** at least once. **Active disease** was present in 159/189 (84.1%) patients during the last 3 months before the final follow-up visit, distributed as follows: a) 10- <14 years 75/87 (86.2%); b) 14-< 16 years 27/32 (84.4%); c) 16- <18 years 32/39 (82.1%) and d) > 18 years 25/31 (80.6%). The **treatment** for active CNO/CRMO at the final follow-up visit included NSAIDs (115/189, 60.8%), conventional DMARDs (30/189, 15.9%), biologics (25/189, 13.2%), and bisphosphonates (10/189, 5.3%) and did not differ significantly between the above age groups.

**Conclusion:** Over 80 % of pediatric patients in this cohort continued to have intermittently active disease, including those patients between the ages 16-18 years, highlighting the need for transition care into adulthood. The data was similar for patients who were > 18 years, suggesting that the pattern of remitting and relapsing disease continues into young adulthood.

We highlight two observations. First, elevated inflammatory markers were seen in a high proportion of patients at baseline, suggesting a possible selection bias of more severely affected CNO/CRMO patients in the EUROFEVER cohort. Second, bisphosphonates or biologics, medications that we now know can be effective in controlling CNO/CRMO, were used relative seldom in this cohort, potentially explaining some of the high proportion of patients with active disease.

**Patient Consent**

Not applicable (there are no patient data)

**Disclosure of Interest**

None declared

## P341 Growth in children with chronic noninfectios osteitis: a longitudinal cohort study

### P. Miettunen^1,2^, L. Carlini^3^, A. Pistorio^3^, A. Insalaco^4^, J. Anton^5^, J. Brunner^6^, G. E. Legger^7^, M. Cattalini^8^, E. Hoppenreijs^9^, M. Alessio^10^, N. Ruperto^3^, R. Caorsi^3^, M. Gattorno^3^ on behalf of EUROFEVER and Pediatric Rheumatology International Trials Organization (PRINTO)

#### ^1^Pediatrics and Rheumatology, Alberta Children's Hospital; ^2^Pediatrics, University of Calgary, Calgary, Canada; ^3^Pediatric Rheumatology, Second Division of Pediatrics, Giannina Gaslini Institution, Genoa; ^4^Rheumatology, IRCCS Ospedale Pediatrico Bambino Gesù, Rome, Italy; ^5^Pediatric Rheumatology, Hospital Sant Joan de Déu, Universitat de Barcelona, Barcelona, Spain; ^6^Department of Pediatrics, Pediatric Rheumatology, Medical University Innsbruck, Innsbruck, Austria; ^7^Pediatrics, University of Groningen, University Medical Center Groningen, Groningen, Netherlands; ^8^Division of Immunology and Pediatric Rheumatology, Pediatric Clinic, University of Brescia, Spedali Civili, Brescia, Italy; ^9^Pediatric Rheumatology, Radboud UMC, Nijmegen, Netherlands; ^10^Pediatrics, Napoli Federico II University, Naples, Italy

##### **Correspondence:** P. Miettunen

*Pediatric Rheumatology 2023*, **21(Suppl 2):**P341

**Introduction:** Chronic non-bacterial osteomyelitis (CNO) is a recurring autoinflammatory disorder characterized by sterile bone osteolytic lesions. Knowledge of growth parameters at CNO diagnosis and over time is limited.

**Objectives:** To obtain longitudinal data on growth in a multinational large series of CNO patients enrolled in the Eurofever registry.

**Methods:** Children with CNO who were < 18 years old and had follow-up visits during at least 1-year period and for whom anthropometric data was available, were studied. Gender, extra-osseous manifestations, achievement of remission, active disease during the last 3 months of follow-up and therapy with corticosteroids were analyzed.

**Results:** Baseline anthropometric data was available for 237/573 (41.4%) patients (138/237 [58.2%] female), with 125/237 (52.7%) patients having both baseline and longitudinal growth data. The median (Interquartile range [IQR]) age at diagnosis was 10.07 [7.94;11.8] and duration of follow-up was 25.4 [12.4; 44.1] months. ***Extra-articular manifestation*s** developed in 39/125 (31%) of patients, with arthritis (21%), psoriasis (5%) and acne (5%) being the most common features. ***Remission*** was reached at least once by 58/125 (46.4%) of the patients. ***Active disease*** was present during the last 3 months of the follow-up in 107/125 (85.6%) patients. ***Corticosteroids*** were used in 34/125 (27%) of children, with 26/34 (77%) children receiving them continuously throughout the study period.

**Growth measures:** At baseline, (N=237), the median [IQR] height Z-score was -0.08 [-0.72; 0.83] and the median [IQR] body mass index (BMI) Z-score 0.80 [-0.43; 2.10]. For the ***longitudinal cohort*** of 125 patients, the median [IQR] **height** Z-score was -0.10 [-0.72; 0.83] at baseline and -0.02 [-0.78; 0.75] at final follow-up, with median [IQR] **BMI** Z-score at baseline 0.83 [-0.27; 2.13] and at final follow-up 0.79 [-0.08;1.96]. **Growth failure,** as measured by height deflection (=change in height Z-score less than -0.25/year), was seen in 31/125 (25%) patients, including 21/74 females and 10/51 males. There were no differences regarding the height Z-scores or growth failure with respect to the specified disease related characteristics or corticosteroids. Only corticosteroid therapy affected the BMI. The mean (standard deviation) BMI Z-score at final follow-up was 0.4 (1.5) for the patients who received ***corticosteroids*** *vs.* 1.1 (1.5) for those who did not (p-value 0.03).

**Conclusion:** CNO in this cohort had no significant impact on height or BMI in affected children at baseline.

Extra-osseous manifestations did not have an adverse effect on the growth measures studied.

Surprisingly, patients who received corticosteroids had lower BMI at the final visit, implying that ongoing active disease, even in the presence of chronic corticosteroid use, can decrease the rate of weight gain.

**Most importantly, this longitudinal study revealed that growth failure, as measured by height deflection, was present at final follow-up in 25% of CNO patients,** with females being more affected than males. We postulate that ongoing episodes of disease flares with recurring inflammation can adversely affect linear growth.

**Patient Consent**

Not applicable (there are no patient data)

**Disclosure of Interest**

None declared

## P342 Unifocal periostitis as the clinical presentation of chronic non bacterial osteitis

### C. Millán-Longo^1^, C. Udaondo^1^, R. Alcobendas^2^, B. Diaz-Delgado^1^, S. Alcolea^3^, M. Garrido^1^, J. Cabello^4^, A. Remesal^1^

#### ^1^Paediatric rheumatology; ^2^Hospital Universitario La Paz, Madrid, Spain; ^3^Paediatric infectious diaseases; ^4^Traumatology, Hospital Universitario La Paz, Madrid, Spain

##### **Correspondence:** C. Millán-Longo

*Pediatric Rheumatology 2023*, **21(Suppl 2):**P342

**Introduction:** Periostitis in childhood is frequently secondary to overuse, tipically bilateral and improves with decreased weight-bearing physical activity. However, in rare cases it can be an atypical form of chronic non-bacterial osteomyelitis (CNBO).

**Objectives:** We present a clinical case its management and outcome.

**Methods:** Medical chart review

**Results:** A 14-year-old boy was referred to the paediatric rheumatology department for severe pain in he right thigh for 3 months, limitation of knee flexion-extension and limping. The pain worsened with standing and physical activity and was refractary to NSAIDs. He reported initial swelling of the right knee at onset. He remained afebrile and without other symptoms. Previously healthy child, with no family history of interest. He played high performance football.

MRI of the right lower limb showed diffuse hypersignal of the right femoral periosteum, without fractures or focal lesions, with normal knee and hip and no muscle alterations. Blood tests showed hypertransaminasemia (GOT 63UI/L, GPT 152UI/L), normal CPK, ESR 10mm/h, C reactive protein <0.5mg/L and elevated serum amyloid A (SAA) (131mg/dL). In view of the findings, a bone biopsy was performed which confirmed chronic inflammation of the compact bone with normal bone marrow and no signs of tumour infiltration.

On suspicion of unifocal periostitis of the right femur of autoinflammatory aetiology, three intravenous pulses of 1 g methylprednisolone were administered and subcutaneous adalimumab 40 mg was started on an intensified regimen every 7 days and oral prednisone. He showed a rapid improvement with pain relief, normalisation of analytical alterations (GOT 30UI/L, GPT 56UI/L, SAA 0.35mg/dL) and gradual functional recovery until he could return to his usual physical activity.

Given the good evolution, corticotherapy was suspended in the following months and adalimumab was spaced out. He is currently asymptomatic and on treatment with adalimumab at the usual dose every 3 weeks.

**Conclusion:** Unifocal periostitis is a rare entity that could be considered in the autoinflammatory spectrum. It requires a high index of suspicion for early diagnosis and treatment to avoid the long-term consequences of associated persistent inflammation, chronic pain and functional disability. Definitive diagnosis requires bone biopsy to screen for malignancy. Autoinflammatory periostitis may be the sole manifestation of the disease, or be associated with other symptoms (SAPHO: synovitis, acne, pustulosis, hyperostosis, osteitis; DIRA: sterile multifocal osteomyelitis, periostitis and pustulosis of neonatal onset). Corticosteroids and anti-TNF drugs may be useful in its management, as in other forms of OCNB.

**Patient Consent**

Yes, I received consent

**Disclosure of Interest**

None declared

## P343 Undefined autoinflammatory diseases with diffuse lymphatic involvement: a monocentric experience

### M. F. Natale, C. Celani, S. Federici, F. De Benedetti, A. Insalaco

#### Rheumatology, Bambino Gesù Children's Hospital, Rome, Italy

##### **Correspondence:** M. F. Natale

*Pediatric Rheumatology 2023*, **21(Suppl 2):**P343

**Introduction:** Systemic autoinflammatory diseases (SAIDs) are a group of disorders characterized by periodic or persistent activation of the innate immune system. About 50% of patients present with an undefined picture and represent a real diagnostic and therapeutic challenge.

**Objectives:** To describe the clinical characteristics, laboratory assessment, genetic findings and treatment response in a cohort of patients with undefined systemic autoinflammatory disease (SAIDs) characterized by a diffuse lymphatic involvement.

**Methods:** Patients from our Pediatric Rheumatology unit with undefined SAIDs and diffuse lymphatic involvement were included. Demographic, clinical, instrumental and laboratory characteristics and therapeutic approaches were collected

**Results:** We described four patients with recurrent, unexplained, episodes of systemic inflammation. All patients presented with recurrent episodes of fever, with irregular pattern and duration, in the absence of triggering factors. Skin rash was frequent and in two out of 4 patients gastrointestinal involvement with diarrhea was detected. All cases presented with a significant involvement of the lymphatic system (hepatosplenomegaly and lymphadenopathy) radiologically detected (ultrasound, CT or MRI). Lymph-node biopsy was performed in three cases (75%) showing reactive histologic features. Blood exams revealed in all cases an increase of inflammatory markers during febrile episodes with a persistent hypergammaglobulinemia (IgG mean value 16.57). Immunological studies did not reveal abnormal values in lymphocyte subpopulations. Double negative T lymphocytes were normal and Oliveira's criteria for the diagnosis of ALPS were not satisfied in none of the patients. Interferon-gamma induced chemokines dosage showed a mild increase of values (CXCL9 mean value 545; CXCL10 mean value 786) suggesting a marginal involvement of this inflammatory pathway. Genetic tests, performed in all patients, did not revealed pathogenic variants associated with monogenic diseases. Glucocorticoid systemic therapy was effective in all cases but a quickly relapse of the disease was observed during tapering phase. We tried first line non-biological (Sirolimus) and biological treatments (Il-1 Inhibitors) with poor or incomplete response. In three out of 4 patients Il-6 Inhibitor (Tocilizumab) was started with good response, complete control of fever and partial regression of lymphatic involvement demonstrating a clear involvement of this inflammatory cytokine in this subgroup of “undefined” autoinflammatory diseases.

**Conclusion:** This study analyzes clinical and laboratory characteristics and response to treatment of a cohort of patients with undefined autoinflammatory phenotype with diffuse lymphatic involvement. Our patients presented with a peculiar phenotype in which lymphatic organ involvement has a central role showing a brilliant and homogeneous response to interleukin 6 inhibitors. This study emphasizes the importance to identify subgroups of undefined SAIDs with peculiar and homogeneous phenotypes that could help pediatric rheumatologist to choose the most effective target therapies.

**Patient Consent**

Not applicable (there are no patient data)

**Disclosure of Interest**

None declared

## P344 Clinical disease course of family members with unknown SAVI-like vasculopathy

### A. Omenetti^1^, B. Lattanzi^1^, A. Tommasini^2,3^, A. Tesser^2^, F. Faletra^2^, S. Cazzato^1^

#### ^1^Pediatric Unit, Department of Mother and Child Health, AOU Marche, Salesi Children’s Hospital, Ancona; ^2^Institute for Maternal and Child Health, IRCCS Burlo Garofolo; ^3^Università di Trieste, Trieste, Italy

##### **Correspondence:** A. Omenetti

*Pediatric Rheumatology 2023*, **21(Suppl 2):**P344

**Introduction:** We describe an Italian family with SAVI-like vasculopathy lacking genetic confirmation in STING

**Objectives:** To increase awareness of an undefined autoinflammatory disease by describing the clinical course, response to treatments and potential putative genotype in patients with unsolved SAVI-like vasculopathy

**Methods:** ADA2 genetic test was assessed in the affected pediatric patient along with strict clinical follow-up. IFN signature and Whole exome sequencing (WES) were performed in patients and healthy family members. Identified mutations were analyzed by Sanger sequencing. Fibroblasts were isolated by skin punch biopsy in the affected father

**Results:** A 6.8 years old girl (pt 1) displays livedo reticularis and palpable SAVI-like rash on cheeks and body since 2 months of age, sometimes aggravated by cold. Urticarial features were present at onset but subsided by the age of 2. No other constitutional symptoms, nor neurological involvement occurred. Her 46 years old father (pt 2) is affected by a milder disease characterized by recurrent urticarial rash since infancy, well controlled by prednisone on demand. On the contrary, her 48 years old uncle (pt 3), suffers since childhood of a severe clinical picture, featured by polyarthritis and urticarial rash. Steroids have been attempted with partial control, not preventing severe disease progression into deforming polyarthritis of small joints, erosion of the nasal bridge, cutaneous ulcers and autoamputation of toes and fingers. All affected patients have evidence of systemic inflammation. A trial with anakinra was initially attempted in pt 1 without benefit. ADA2 was investigated and ruled out. IFN-signature was slightly increased in pt 1 (score 3, normal value<2.5) and pt 2 (score 3.8) whereas it was significantly elevated in pt 3 (score 7) consistent with his more severe phenotype. WES was performed, identifying mutations in two potential candidate genes. Namely, symptomatic patients carry a novel heterozygous c.C564A variation in the *ZC3H12D* gene (stop-codon mutation), coding for a Zn-finger protein together with heterozygous c.A948G:p.I316M variant of *MB21D1* gene, which encodes the cytosolic DNA sensor cGAS. The latter is already predicted as damaging by bioinformatic tools, suggesting a potential role of Type I IFN. Off-label treatment with hydroxychloroquine (4 mg/Kg/day) was started in pt 1 leading to complete control of inflammatory markers and cutaneous features, but recurrence of flares at tapering attempts. Lung function has been regularly assessed by 6MWT, LFTs, and polygraphic monitoring without current signs of pulmonary involvement

**Conclusion:** Affected family members display a clinical picture of variable degree, which resembles a SAVI-like vasculopathy in its most severe expression. Further investigations are required to clarify pathogenesis, in order to identify a tailored treatment and follow-up strategy to avoid disease progression and complications.

**Patient Consent**

Yes, I received consent

**Disclosure of Interest**

None declared

## P345 A case of sting-associated pathology

### O. A. Oshlianska^1,2^

#### ^1^Department of Pediatrics, Pediatric Infectious Diseases, Immunology and Allergology, Shupic National Medical Academy of Postgraduate Education, Department of Pediatrics, Pediatric Infectious Diseases, Immunology and Allergology; ^2^pediatric Rheumatology Center, Institute of Pediatrics, Obstetrics and Gynecology of the National Academy of Medical Sciences of Ukraine, Kyiv, Ukraine

##### **Correspondence:** O. A. Oshlianska

*Pediatric Rheumatology 2023*, **21(Suppl 2):**P345

**Introduction:** A new class of autoinflammatory diseases clinically similar to rheumatic diseases has been singled out in recent decades. Among them, a special place belongs to interferonopathy. However, there are some difficulties remaining in the timely detection and treatment of these diseases in Ukraine.

**Objectives:** To analyze the case of early diagnosis of interferonopathy and features of the differential diagnosis.

**Methods:** Analysis of medical records data.

**Results:** A boy from the 1st pregnancy, 1st urgent physiological delivery. COVID-19 at 2 months, received antibiotic therapy. At the age of 4 mo - pulmonary lesion, diagnosed as acute bronchitis. The first elements of a rash appeared on the face and feet at 4.5 months, atopic dermatitis was considered, the patient received treatment without sufficient effect. Then the child was re-consulted, Gianotti-Crosti syndrome was suspected, topical corticosteroids were used. Skin changes progressed, at 9 months there was widespread cyanotic edematous erythema with elements of destruction on the cheeks, tip of the nose, fingers and toes, soles, over the elbow joints. Punch-biopsy of the skin was performed epidermal hyperkeratosis, neutrophil infiltration, basal vacuolization, spongiosis, interfibrous mucinosis of the dermis and lymphocytic perivascular infiltration. These changes were regarded by the dermatologist as subacute cutaneous lupus. The child was reexamined at 10 months. Blood test: Hb 108 g/l, WBC 12.1x10^9^ per l, PLT 664x10^9^ per l, ESR 39 mm/h. RF+, ANA+, dDNA+, the absolute number of T-lymphocytes was reduced, NK were not detected. IG levels were elevated: IgG 22.13 IgM -1.19, IgA - 0.86 g/l. IL1 level was also elevated. IgM, IgG to SARS-cov2, antibodies to S-protein were negative. Chest X-ray showed bilateral polysegmental opacities, thickening of the pleura in the absence of lymphadenopathy. Saturation=99%. No other changes in visceral organs and central nervous system were found. The ophthalmologist, otorhinolaryngologist, and neurologist noted no abnormalities in the child. Storage diseases have been ruled out by biochemical tests. Received antiplatelet therapy, systemic corticosteroids. This led to deterioration of the skin condition by the age of 1 year. The presence of chillblain lead to thinking about of developing a post-COVID-syndrome, paraneoplastic syndrome and autoinflammatory diseases in a child. MRI of whole patients body showed no changes in the brain, but multiple areas of hypoventilation in his lungs persisted. Genetic testing with identification of variants of 574 genes (INVITAE) was performed on the patient. Pathogenic mutation TMEM173 (exon 6, c.616T>G p.Cys206Gly) was identified. Tofacitinib therapy was initiated.

**Conclusion:** SAVI was diagnosed in a child with lung and skin lesions at 1 year of age before the development of neurological disorders. It is subject to further discussion whether or not COVID-19 in patients with rheumatic-like skin lesions could be a marker for the detection innate previous immune disease.

**Patient Consent**

Yes, I received consent

**Disclosure of Interest**

None declared

## P346 Worldwide evaluation of Clinical Practice StrategieS (CLIPS) in autoinflammatory recurrent fever syndromes through the JIR-CLIPS network: a cost action

### M. Rodrigues^1^, R. Bourguiba^2^, H. Wittkowski^3^, R. Caorsi^4^, T. Hinze^5^, I. Elhani^6,7^, M. Gattorno^4^, N. Toplak^8^, C. Vinit^9^, M. Mejbri^10,11^, M. Hofer^12^, V. Hentgen^13^ on behalf of JIR-CLIPS network

#### ^1^Pediatric and Young Adult Rheumatology Unit, Centro Hospitalar Universitário São João, Porto, Portugal; ^2^Internal Medicine, Hopital des Forces de sécurité de l’intérieur La Marsa, La Marsa, Tunisia; ^3^Pediatric Rheumatology and Immunology, University Hospital Münster, Muenster, Germany; ^4^Pediatric Rheumatology and Autoinflammatory Diseases Unit, IRCCS G. Gaslini, Genova, Italy; ^5^Clinic for Pediatric and Adolescent Rheumatology, St. Josef-Stift Sendenhorst, Sendenhorst, Germany; ^6^Internal Medicine Tenon Hospital, Paris, France, Tenon Hospital, Paris; ^7^Pediatrics, Hospital of Versailles, Versailles, France; ^8^Allergology, Rheumatology and Clinical Immunology, UCH, UMC Ljubljana, Ljubljana, Slovenia; ^9^Immunology, Haematology and Rheumatology Department, Necker Hospital, Paris, France; ^10^Unité Romande d'Immuno-Rhumatologie Pédiatrique, Hôpital des Enfants - Hôpitaux Universitaires de Genève HUG, Geneve; ^11^Unité Romande d'Immuno-Rhumatologie Pédiatrique, Centre Hospitalier Universitaire Vaudois CHUV; ^12^Pediatric Rheumatology Unit of Western Switzerland, Lausanne University Hospital (CHUV), Lausanne, Switzerland; ^13^Pediatrics - CeReMAI, Centre Hospitalier de Versailles - Hôpital André Mignot, Versailles, France

##### **Correspondence:** M. Rodrigues

*Pediatric Rheumatology 2023*, **21(Suppl 2):**P346

**Introduction:** Autoinflammatory diseases (AID) are a rare subgroup of Juvenile Inflammatory Rheumatisms (JIR) with a significant morbidity and mortality risk; many patients require lifelong medication. The implementation of evidence or consensus-based recommendations for diagnosis and treatment can be challenging due to the wide array of country-specific medical systems, financial capacities, and variable training of physicians in these rare diseases.

**Objectives:** The main objective of the JIR-CliPS network is to collect real-life clinical practice strategies (CliPS) from physicians worldwide to produce adequate treatment plans for selected JIR.

Secondary objectives are to assess the reasons for discrepancies with existing guidelines and prospectively compare different clinical strategies regarding indications, treatment adjustments, and long-term outcomes.

**Methods:** Five areas of interest were selected, of which two concern autoinflammatory recurrent fever syndromes: the use of biological drug modifying disease (bDMARDs) in genetic AID and diagnosis and treatment of PFAPA/recurrent fever of unknown significance (SURF). The questionnaires were finalized in 2022, and the project was submitted as a COST (European cooperation for science and technology) action, which was accepted (CA21168).

Since September 2022, the online questionnaires have been distributed through different channels such as the JIRcohort network, national societies, and networks, and are still available. An interim analysis of these questionnaires was performed in April 2023.

**Results:** Regarding AID questionnaires, 103 physicians from 28 different countries responded, the majority originating from Brazil and Turkey. More pediatricians than adult physicians responded (75% vs 8%), and the experience of the responding physicians was quite high. Even for the most common disease, FMF, 65% of the respondents were not aware of existing national guidelines for the management of the disease.

For PFAPA/SURF questionnaires, 123 responses were collected, with most responders being women (68.3%). The main countries were Brazil (25%) and Turkey (14.6%), and most doctors were from university hospitals (59%) and were pediatric-oriented (83.61%). 51% of the participants cared for PFAPA patients for more than 10 years.

**Conclusion:** The interim analysis of the two questionnaires showed that the first answers came mainly from pediatricians and physicians with a solid experience in autoinflammatory syndromes. These data suggest that adult physicians are less involved or less solicited to answer the questionnaires. Young physicians involved with AIDs also did not respond enough to these questionnaires, either because of lack of communication or lack of information. Adult and young physicians should be encouraged to answer these questionnaires to better evaluate real-life clinical practice strategies.

**Patient Consent**

Not applicable (there are no patient data)

**Disclosure of Interest**

None declared

## P347 Mikulicz disease - a Sjögren's mimicker

### S. S. S. Rodrigues^1^, L. Salazar^2^, J. B. Lima^2^, J. Fraga^3^, S. Alves^4^, C. Zilhão^4^

#### ^1^Pediatrics, Centro Hospitalar Entre Douro e Vouga, Santa Maria da Feira; ^2^Pediatrics, Centro Hospitalar e Universitário de Santo António, Porto; ^3^Pediatrics, Unidade Local de Saúde do Nordeste, Bragança, ^4^Pediatric Rheumathology, Centro Hospitalar e Universitário de Santo António, Porto, Portugal

##### **Correspondence:** J. B. Lima

*Pediatric Rheumatology 2023*, **21(Suppl 2):**P347

**Introduction:** Mikulicz disease is an Immunoglobulin G4-related disease (IgG4-RD) characterized by abnormal enlargement of lacrimal and salivary glands. Both Sjögren syndrome (SS) and IgG4-related sialadenitis and dacryoadenitis can exhibit swelling of lacrimal, parotid, and submandibular glands. The absence of autoantibodies, IgG4 levels and histopathologic findings are key for the diagnosis.

**Results: Case report:**

An 11-year-old boy, with previous history of asthma, allergic rhinitis, and vitiligo, presented with a 2-year history of persistent right eyelid and bilateral neck swelling, associated with periodic complains of dry eyes and mouth. Physical examination showed lacrimal, parotid, and submandibular glands enlargement.

A previous minor salivary gland biopsy revealed lymphocytic infiltrates with positive focus score (3), consistent with SS, although no immunofluorescence assay was performed. Further evaluation revealed inflammatory markers within normal range, slight IgG elevation with a high IgG4 subpopulation (1460mg/dL), normal C3 and C4, negative antinuclear antibodies, anti-SSA, anti-SSB and rheumatoid factor. Schirmer test result was 5 mm/3 minutes.

The diagnosis of IgG4-related Mikulicz disease was assumed and treatment with oral prednisolone (1 mg/kg) was started with rapid clinical improvement of both eyelid and submandibular swelling. He also reported resolution of the sicca syndrome. Total IgG4 value decreased (414 mg/dL). Abdominal, pelvic, and thoracic CT were normal as were pulmonary function tests with diffusing capacity of the lungs for carbon monoxide. Fecal elastase and calprotectin were also within the normal range.

He underwent a 6-month prednisolone course and relapsed after a 2-years remission period. IgG4 values rose to 2040 mg/dL and salivary gland biopsy was repeated. The biopsy showed an accentuated lymphoid infiltration, with acinar atrophy and slight fibrosis. Immunohistochemical analysis showed infiltration by IgG4-positive plasma cells in numbers greater than 100 per high-power field and an IgG4/IgG ratio greater than 40. Oral prednisolone was re-started together with azathioprine, with good clinical and analytic response. He is currently under corticosteroid tapering with no worsening of the symptoms.

**Conclusion:** IgG4-RD occurs predominantly in adults, but it also affects children.

When untreated, the disease can lead to irreversible organ damage caused by fibrosis, therefore prompt recognition and treatment is critical. Steroids are the first line treatment but cDMARDs, such as azathioprine or mycophenolate, or bDMARD rituximab are used as combination therapy in relapses as in some more aggressive phenotypes. Early combination therapy remains controversial.

**Patient Consent**

Yes, I received consent

**Disclosure of Interest**

None declared

## P348 Case series: phenotypic variability in Aicardi-Goutières (AGS) and AGS mimic/overlap

### M. Romano^1^, R. Berard^1^, A. Geerlinks^2^, M. Nouri^1^, J. Park^1^, T. Balci^2^, S. Park^3^, A. de Jesus^3^, E. Demirkaya^1^

#### ^1^Pediatrics; ^2^Western University, London, Ontario, Canada; ^3^NIAID, NIH, Bethesda, United States

##### **Correspondence:** M. Romano

*Pediatric Rheumatology 2023*, **21(Suppl 2):**P348

**Introduction:** Aicardi-Goutières syndrome (AGS) and AGS mimic/overlap are genetically defined as immunodysregulatory disorders characterized by the presence of a type I interferon (IFN) signature in peripheral blood and variable systemic inflammation. Patients can exhibit variable phenotypes beyond the central nervous system, such as chilblains, autoimmune hepatitis, and hematological disturbances, which may impact the prognosis and overall quality of life.

**Objectives:** To explore clinical spectrum, treatment approach, and interferon signature in AGS and AGS mimic/overlap.

**Methods:** Six patients with genetically confirmed diagnoses were included: 2 patients with classic AGS (due to TREX1 and SAMHD1 variants), 2 patients with AGS/overlap Singleton-Merten (IFIH1-related), and 2 patients with ACP5-related spondyloenchondrodysplasia (SPENCD). Peripheral blood IFN signature (28-gene IFN score) was measured using NanoString technology.

**Results:** The mean time to diagnosis from disease onset was 5.8 years (0-12 years). The most common features were developmental delay, spasticity and cutaneous lesions such an acrocyanosis. One patient (IFIH1) presented with severe psoriasis. Other features included short stature, bone dysplasia, hepatitis, abnormal dentition, idiopathic thrombolytic purpura, autoimmune hemolytic anemia, and neutropenia.

Three patients were treated with JAK inhibitors (Baricitinib 0.29 and 0.5 mg/kg/day or Tofacitinib 0.33mg/kg/day). The choice of JAK inhibitors depended on the availability of drugs and the affordability of the patients. Tofacitinib was replaced with Anifrolumab in patient with IFIH1 mutation due to the unsatisfied control. Other treatments included MMF, glucocorticoids, IVIG, Tocilizumab and Ustekinumab.

IFN signature was available for five patients and was found to be elevated in all of them at the time of diagnosis (mean IFN score: 221.71, range 89.4- 354, cutoff 25.2). Type 1 IFN signature was monitored in four patients. The patient who was treated with Anifrolumab, IFN signature significantly reduced after four dosage of infusion (25.2).

**Conclusion:** AGS and AGS mimic/overlap patients demonstrate variable phenotypes and lead to severe organ-specific inflammation and intellectual and physical disability. An elevated Type 1-IFN signature provided a clue to the diagnosis and the rationale for treatment. The IFN signature in our patients (two on JAK inhibitors, one on MMF, and one without any treatment) remained elevated regardless of treatment response and clinical course of the disease during their follow-up period. The fact that the IFN signature was not normalized in our patients (except P3 with Anifrolumab) points that better treatments are needed. Furthermore, multi-center studies with more and longer-term data would be preferable in establishing the efficacy and safety of these treatments in AGS.

**Patient Consent**

Yes, I received consent

**Disclosure of Interest**

None declared

## P349 What are the factors that contribute to the time to diagnosis of chronic nonbacterial osteomyelitis in a paediatric tertiary setting in Ireland?

### E. Rowe^1,2^, D. O'Leary^3^, O. Killeen^1^

#### ^1^Rheumatology, CHI at Crumlin; ^2^School of Nursing and Midwifery, TCD; ^3^Centre for Arthritis Research, UCD, Dublin, Ireland

##### **Correspondence:** E. Rowe

*Pediatric Rheumatology 2023*, **21(Suppl 2):**P349

**Introduction:** Chronic nonbacterial osteomyelitis (CNO) is a heterogenesous disorder that causes sterile bone lesions anywhere along the skeleton. It is an auto-inflammatory condition that can be diagnosed at any age age but primarily is seen in children and adolescents. It can be associated with other auto-inflammatory conditions and extraosseous manifestations such as IBD and psoriasis. Although the awareness of CNO is increasing, it still remains a diagnosis of exculsion and amongst the literature to date, the average time from symptom onset to a confirmed diagnosis of CNO is 2 years but can take longer. Due to its insidious onset and broad clinical features, other possible diagnosis must be outruled, primarily infection and malignancy. As a result, children and adolescents undergo a multitude of investigations, attend various healthcare physicians (HCPs) and may receive ineffective/unneccessary treatment prior to a confirmed diagnosis of CNO. Furthermore, initiation of treamtent may be delayed and complications can occur.

**Objectives**

To identify various factors that contribute/influence the timeframe from onset of symptoms to a confirmed diagnosis of chronic non-bacterial osteomyelitis.

Objectives:

1. To examine the demographics of children and adolescents with CNO in Ireland.
2. To investigate current practices in diagnosing CNO in Ireland.
3. To examine the referral pathway to a paediatric rheumatologist in receiving a confirmed diagnosis of CNO in Ireland.
4. To Identify the treatments intialtated prior to the suspected/confirmed diagnosis of CNO.
5. To explore the time it takes from symptom onset to confirmed diagnosis of CNO.
6. To identify the variability in factors that contribute to the time taken from symptom onset to confirmed diagnosis of CNO in Ireland.
7. To identify any potential barriers to early diagnosis that could be improved along the referral pathway from symptom onset to confirmed diagnosis of CNO.

**Methods:** A quantitative retrospective chart review was conducted in a tertiary hospital in Ireland with the target population being children and adolescents who have a confirmed diagnosis of CNO. It included children and adolescents up to the age of 18 years who received a confirmed diagnosis of CNO between 2011 to 2021 inclusive. Although the rheumatology service was estabilsed in 2006, this 10 year period was chosen in order to best reflect current practices as the awareness of CNO has steadily increased. Those excluded from the study was anyone with a diagnosis of Majeed syndrome, DIRA or PAPA . The rational being that this study was excuslively examining the various factors that contribute to time taken from symptom onset to confirmed diagnosis of CNO and not of those with a diagnosis in whereby CNO is a clinical feature.

80 participants were initially identified but this was reduced after the inclusion and exclusion criteria was applied. The final sample size was 57 participants after a further reduction of participants due to missing/unclear data along with charts that could not be retrieved during the data collection period.

In order to extract the data, a tool was developed. To ensure reliability and validity, the first extraction tool was reviewed by 3 clinical experts; a consultant rheumatologist, a research fellow and an advanced nurse practioner. Additional questions were added to the tool following the expert review. Ethical approval was achieved and a pilot study was then carried out in whereby data was extracted from the charts of 5 of the 57 participants identified. Following the completion of the pilot study, additional questions deemed necessary to answer the research question were added to the extraction tool.

The data from the final sample size (n=52) was manually extracted using the tool, reduced and inputted into the Statistical Package for the Social Sciences (SPSS) software resulting in descriptive and correlation statistics.

**Results:** In Ireland the average time to a confirmed diagnosis is 9.43 months but can take uo to 48 months. An average of 3-4 HCPs are visited prior to seeing a rheumatologist. It appears the time to diagnosis is primarily influenced by the time it takes to first attend a HCP and the completion of a FB-MRI.

**Conclusion:** In comparison to the literature review, this study demonstrates that overall, Ireland is excelling in the timely diagnosis of CNO however improvements can still be achieved. Further research on the referral pathway is required along with continued education for HCPs. Particularly those in referring hospitals and not only education about CNO but emphasisng the need of timely access to FB-MRIs. Further research in the validation of the current propsed diagnostic criteria, is also required.

**Patient Consent**

Not applicable (there are no patient data)

**Disclosure of Interest**

None declared

## P350 High antibiotic prescription rates for children with PFAPA

### K. Rydenman^1,2^, A. Fasth^1,3^, S. Berg^1,3^, P. Wekell^1,2,3^

#### ^1^Department of Pediatrics, Institute of Clinical Sciences, University of Gothenburg, Gothenburg; ^2^Department of Pediatrics, NU Hospital Group, Uddevalla; ^3^Queen Silvia Children’s Hospital, Department of Pediatric Rheumatology and Immunology, Gothenburg, Sweden

##### **Correspondence:** K. Rydenman

*Pediatric Rheumatology 2023*, **21(Suppl 2):**P350

**Introduction:** Periodic fever, aphthous stomatitis, pharyngitis and cervical adenitis (PFAPA) is an autoinflammatory syndrome that causes regularly recurring febrile episodes with elevated inflammatory markers. Individual episodes are hard to distinguish from bacterial infections and PFAPA episodes are thus often misdiagnosed as streptococcal tonsillitis, pneumonia and pyelonephritis, entailing a risk that children with PFAPA unduly receive repeated courses of antibiotics.

**Objectives:** To investigate the prescription rate of antibiotics for children with PFAPA and compare this with the rate for children in the general population in defined age groups and time periods.

**Methods:** Data on drug prescriptions during 2006-2017 for 333/336 children with PFAPA from a previously described cohort [1] was obtained from the Swedish National Prescribed Drug Register. The register contains all drug prescriptions dispensed at pharmacies in Sweden, linked to individuals by the Swedish personal identity numbers. The incidence rates of antibiotic prescriptions in terms of number of prescriptions/1,000 person years were calculated in children with PFAPA and children in the general population in the age groups 0–4, 5–9 and 10– 14 years during the periods 2006–2009, 2010 –2013 and 2014–2017 and compared using incidence rate ratio (IRR).

**Results:** In the age group 0-4 years, antibiotic prescription rates/1,000 person years in the PFAPA cohort were 1369 in 2006-2009, 1128 in 2010-2013 and 1218 in 2014-2017. The corresponding rates in the general population were 663, 506 and 345 respectively, resulting in an IRR of 2,1 (95% CI 1.8-2.4) in 2006-2009, 2.2 (1.9-2.6) in 2010-2013 and 3.5 (2.8-4.4) in 2014-2017.

In the age group 5-9 years, rates in the PFAPA cohort were 1093 in 2006-2009, 680 in 2010-2013 and 467 in 2014-2017. The corresponding rates in the general population were 462, 384 and 266, resulting in an IRR of 2.4 (95% CI 1.8-3.1) in 2006-2009, 1.8 (1.4-2.2) in 2010-2013 and 1.8 (1.4-2.3) in 2014-2017.

In the age-group 10-14 years, rates in the PFAPA cohort were 340 in 2006-2009, 460 in 2010-2013 and 205 in 2014-2017. Rates in the general population were 225, 204 and 153, resulting in an IRR of 1.5 (95% CI 0.6-4.0) in 2006-2009, 2.3 (1.4-3.6) in 2010-2013 and 1.3 (0.9-2.1) in 2014-2017.

**Conclusion:** Children with PFAPA received significantly more antibiotic prescriptions than children in the general population, especially in the youngest age groups. While antibiotic prescriptions have decreased substantially in Sweden in recent years, this trend is not as strong among children with PFAPA. Increased awareness of PFAPA syndrome may contribute to the diagnostic accuracy of PFAPA episodes and less antibiotic prescriptions.

**Patient Consent**

Not applicable (there are no patient data)

**Disclosure of Interest**

None declared

**References**

1. Rydenman K, et al. Epidemiology and clinical features of PFAPA: a retrospective cohort study of 336 patients in western Sweden. Pediatr Rheumatol Online J. 2022;20(1):82.

## P351 Tonsil proteomics profile of patients with periodic fever, aphthous stomatitis, pharyngitis, and adenitis syndrome (PFAPA)

### F. Mutlu^1^, M. Sarıhan^2^, N. Sahin^3^, A. Önal^1^, G. Akpınar^2^, Y. E. Bayrak^3^, H. E. Sönmez^3^, M. Kasap^2^

#### ^1^Otorhinolaryngology; ^2^Basic Medical Sciences; ^3^Pediatric Rheumatology, Kocaeli University, Kocaeli, Türkiye

##### **Correspondence:** N. Sahin

*Pediatric Rheumatology 2023*, **21(Suppl 2):**P351

**Introduction:** Periodic fever, aphthous stomatitis, pharyngitis, and cervical adenitis (PFAPA) syndrome is a recurrent fever syndrome of unknown etiology characterized by regular episodes of fever, pharyngitis, oral aphthosis, and cervical lymphadenopathy. However, PFAPA syndrome is considered as the most common periodic fever syndrome, the exact etiopathogenesis of PFAPA syndrome remains unknown. Biological fluids or tissues may provide disease-specific biomarkers that may help clinicians to find new pathogenic pathways or potential drug candidates.

**Objectives:** We aim to help explain pathogenesis by detecting biomarkers via proteomics analysis in PFAPA.

**Methods:** Tonsil tissues of seven patients with PFAPA were collected during the tonsillectomy. Seven patients with chronic tonsillitis enrolled as a control group. All patients were inactive and treatment-free. The nHPLC LC-MS/MS system was used for protein identification and label-free quantification. Bioinformatics analysis was carried out using the UniProt accession numbers of the identified proteins.

**Results:** Label-free quantification of protein extracts revealed to identity of more than 400 proteins of which at least 9 were up and 29 were downregulated. Bioinformatics analysis of differentially regulated proteins by STRING indicated that protein folding and clearance machinery were interrupted in PFAPA patients compared to the controls. The affected pathways underlined the importance of proteasomal degradation that implied the presence of a disease-forming mechanism similar to some other autoinflammatory diseases. The analysis also indicated that many biological processes were affected by the disease indicating that the pathogenesis of PFAPA is multifactorial, and might be derived from environmental factors acting on genetically predisposed individuals.

**Conclusion:** Although it is not clear that changes in tonsil protein expression whether directly related to pathogenesis or simply result of chronic inflammation, the identification of tonsil biomarkers for PFAPA may provide clinicians an opportunity to understand disease pathogenesis or develop new molecular targets for treatments.

**Patient Consent**

Yes, I received consent

**Disclosure of Interest**

None declared

## P352 IL-1 inhibitors in the treatment of patients with CINCA/NOMID - experience of the Russian federal center

### S. Salugina^1^, E. Fedorov^1^, A. Torgashina^2^

#### ^1^Pediatric; ^2^Rare rheumatic diseases, V.A.Nasonova Research Institute of Rheumatology, Moscow, Russian Federation

##### **Correspondence:** S. Salugina

*Pediatric Rheumatology 2023*, **21(Suppl 2):**P352

**Introduction:** CINCA/NOMID is a rare (orphan) monogenic autoinflammatory disease associated with a genetic mutation in the NLRP3 gene. About 100 patients have been described in the world. CINCA/NOMID is one of the more severe forms of Cryopyrin-Associated Periodic Syndromes (CAPS). It is characterized by early onset, persistent chronic course, systemic manifestations (fever, rash, general constitutional symptoms) and organ disorders (central nervous system (CNS) manifestations, arthropathy, sensorineural hearing loss, inflammatory eye disease), as well as an increase in the level of acute phase markers. In the past, many patients with CINCA/NOMID had increased morbidity and mortality due to organ damage or adverse effects from ineffective therapies The development of amyloidosis and early death at a young age cause an unfavorable prognosis. The main targeted therapy includes early and active use of IL-1 inhibitors (iIL-1).

**Objectives:** to present the experience of using iIL-1 in pts with CINCA/NOMID according to the Russian Federal Rheumatology Center.

**Methods:** The study included 8 pts (7 males) aged 10 months to 33 years, among them 3 with a disease duration of more than 10 years (13,17,33). All pts were genetically tested, mutations in the NLRP3 gene were not detected in 2. Аll pts received iIL-1 (anakinra (ANA) or canakinumab (CAN)

**Results:** The age of the onset varied from 0 to 6 months. The delay in diagnosis and in the appointment of therapy ranged from 10 months to 33 years. All pts had classic manifestations of CINCA/NOMID: fever, rash, CNS lesion, arthropathy in 6 pts, ocular Involvement in 7 pts, sensorineural hearing loss in 6 pts, increased ESR, CRP in all pts. Amyloidosis was diagnosed in 1. iIL-1 were prescribed to all pts: ANA- 6 (5 as the 1st line of therapy, 1 - as the 2nd line after CAN). ANA was replaced by CAN in 2 pts (1 showed deterioration and return to ANA). CAN was received by 5 (3 as the 1st line of therapy, 2 as the 2nd line). In 1 patient CAN was replaced with ANA due to insufficient effect from the CNS lesion and persisting acute - phase markers. The response to IL-1 inhibitor therapy was good in all pts, but it was incomplete due to the severity of manifestations and the presence of organ disorders.

**Conclusion:** Patients with CINCA/NOMID have a severe course of the disease and a poor prognosis. In this regard, they need the early appointment of iIL-1. When the CNS is affected, it is preferable to prescribe ANA due to its greater effectiveness and penetration through the blood-brain barrier. In the future it is possible to transfer to CAN, however, to obtain a complete response, it is sometimes necessary to increase the dose and shorten the interval between injections to 4 weeks.

**Patient Consent**

Yes, I received consent

**Disclosure of Interest**

None declared

## P353 Higher rates of disease control during the coronavirus pandemic in pediatric patients with autoinflammatory periodic diseases on Canakinumab treatment – interim data from the reliance registry

### C. Schuetz^1^, N. Blank^3^, J. B. Kuemmerle-Deschner^2^, J. Henes^4^, B. Kortus-Goetze^5^, P. T. Oommen^6^, A. Pankow^7^, T. Krickau^8,9,10^, G. Horneff^11,12^, I. Foeldvari^13^, J. Rech^8,9,14^, F. Weller-Heinemann^15^, A. Janda^16^, M. Hufnagel^17^, F. M. Meier^18,19^, F. Dressler^20^, M. Borte^21^, I. Andreica^22^, P. Wasiliew^2^, M. Fiene^23^, D. Windschall^24^, J. Weber-Arden^25^, T. Kallinich^26,27^

#### ^1^Department of Pediatrics, Medizinische Fakultät Carl Gustav Carus, 8Technische Universität Dresden, Dresden; ^2^Division of Pediatric Rheumatology and autoinflammation reference center Tuebingen, Department of Pediatrics, University Hospital Tuebingen, Tuebingen; ^3^Division of Rheumatology, Department of Internal Medicine, Heidelberg University Hospital, Heidelberg; ^4^Center of Interdisciplinary Rheumatology, Immunology and autoimmune diseases (INDIRA), University Hospital Tuebingen, Tuebingen; ^5^Department of Internal Medicine, Division of Nephrology, University Hospital of Giessen and Marburg, Marburg, Germany, Marburg; ^6^Department of Pediatric Oncology, Hematology and Clinical Immunology, Center for Child and Adolescent Health, Medical Faculty Heinrich-Heine-University Duesseldorf, Duesseldorf; ^7^Department of Rheumatology and Clinical Immunology, Charité-Universitätsmedizin Berlin, Berlin; ^8^Centre for rare diseases Erlangen (ZSEER); ^9^DZI (Deutsches Zentrum für Immuntherapie); ^10^Department of Pediatrics, Friedrich-Alexander University Erlangen-Nuernberg (FAU), Erlangen; ^11^Department of Pediatrics, Asklepios Kinderklinik Sankt Augustin, Sankt Augustin; ^12^Department of Pediatric and Adolescent Medicine, Medical Faculty, University Hospital of Cologne, Cologne; ^13^Hamburg Centre for Pediatric and Adolescence Rheumatology, Hamburg; ^14^Department of Rheumatology and Immunology, University Hospital Erlangen, Erlangen; ^15^Division of Pediatric Rheumatology, Prof. Hess Children's Hospital, Bremen; ^16^Department of Pediatrics and Adolescent Medicine, University Medical Center Ulm, Ulm; ^17^Division of Pediatric Infectious Diseases and Rheumatology, Department of Pediatrics and Adolescent Medicine, University Medical Center, Medical Faculty, University of Freiburg, Freiburg; ^18^Fraunhofer Institute for Translational Medicine and Pharmacology ITMP; ^19^Department of General Pharmacology and Toxicology, Goethe University Hospital and Goethe University Frankfurt, Frankfurt am Main; ^20^Department of Paediatric Pneumology, Allergology and Neonatology, Children's Hospital, Hannover Medical School, Hannover; ^21^Academic Teaching Hospital of the University of Leipzig, Hospital for Children & Adolescents, St. Georg Hospital, Leipzig; ^22^Rheumazentrum Ruhrgebiet Herne, Ruhr-Universität Bochum, Herne; ^23^Rheumatology Center Greifswald, Greifswald; ^24^Clinic of Paediatric and Adolescent Rheumatology, St. Josef-Stift Sendenhorst, Northwest German Center for Rheumatology, Sendenhorst; ^25^Immunology, Novartis Pharma GmbH, Nuernberg; ^26^Department of Pediatric Respiratory Medicine, Immunology and Critical Care Medicine, Charité Universitätsmedizin Berlin; ^27^Deutsches Rheuma-Forschungszentrum (DRFZ), Berlin, Germany

##### **Correspondence:** C. Schuetz

*Pediatric Rheumatology 2023*, **21(Suppl 2):**P353

**Introduction:** Pediatric patients with autoinflammatory diseases (AID) on Canakinumab (CAN) therapy have been affected by the coronavirus pandemic including SARS-CoV-2 infection, SARS-CoV-2 vaccination, and AID disease management.

**Objectives:** In the RELIANCE registry, severity of SARS-CoV-2 infection, safety of SARS-CoV-2 vaccination and comparison of disease management before and during pandemic in pediatric patients with cryopyrin-associated periodic syndromes (CAPS), familial Mediterranean fever (FMF), hyper-IgD syndrome/mevalonate kinase deficiency (HIDS/MKD) and tumor necrosis factor receptor-associated periodic syndrome (TRAPS) on CAN therapy was investigated in clinical practice.

**Methods:** The RELIANCE registry is a prospective, non-interventional, observational study in Germany enrolling pediatric (age ≥2 years) and adult patients with a clinically confirmed diagnosis of AID who routinely receive CAN. Efficacy and safety parameters are recorded at baseline and assessed at 6-month intervals.

**Results:** The present interim analysis includes data from n=101 pediatric patients with AID enrolled in the RELIANCE registry between October 2017 and December 2022. The median duration of CAN treatment before and during the study was 3.7 years (0-13.5 years).

During the study, 29 SARS-CoV-2 infections were reported for n=27 pediatric patients. 22 infections caused mild symptoms and 7 infections caused moderate symptoms. N=25 (24.8 %) of pediatric patients received at least one SARS-CoV-2 vaccination (15 Comirnaty, 13 not specified). Vaccination reactions (not further specified) were reported in n=3 patients. No reactions were classified as serious.

During the COVID-19 pandemic in 2020 and 2021, only 83 % of regular patient visits were performed. In addition, patients received slightly lower CAN dosing* (20% standard dose CAN [SD], 17% lower than SD, and 63% higher than SD compared to 24% SD, 10% lower than SD, and 66% higher than SD before pandemic). However, disease activity by patient rating (VAS score 1-10) improved from 3 before to 2 during the pandemic. By physician global assessment, the proportion of pediatric patients with no disease activity arose from 20% before to 45% during the pandemic (in contrast: no major changes in disease activity were documented in adult patients).

* Body weight > 40kg: Standard dose is 150 mg per 8 weeks for CAPS and 150 mg per 4 weeks for any other indication. Body weight ≤40 kg: Standard dose is 2mg per kg per 8 weeks (CAPS) or per 4 weeks (any other indication)

**Conclusion:** Pediatric AID patients under long-term Canakinumab treatment experienced improved disease control during the Coronavirus pandemic.

**Patient Consent**

Yes, I received consent

**Disclosure of Interest**

C. Schuetz Grant / Research Support with: Novartis, N. Blank Grant / Research Support with: Novartis, Sobi, Consultant with: Novartis, Sobi, Lilly, Pfizer, Abbvie, BMS, MSD, Actelion, UCB, Boehringer-Ingelheim, Roche, J. Kuemmerle-Deschner Grant / Research Support with: Novartis, AbbVie, Sobi, Consultant with: Novartis, AbbVie, Sobi, J. Henes Grant / Research Support with: Novartis, Roche, Consultant with: Novartis, AbbVie, Sobi, Roche, Janssen, Boehringer-Ingelheim, B. Kortus-Goetze Consultant with: Novartis, P. Oommen Grant / Research Support with: Novartis, A. Pankow: None declared, T. Krickau Grant / Research Support with: Novartis, Consultant with: Novartis, Speaker Bureau with: Novartis, G. Horneff Grant / Research Support with: AbbVie, Chugai, Merck Sharp & Dohme, Novartis, Pfizer, Roche, Speaker Bureau with: AbbVie, Chugai, Merck Sharp & Dohme, Novartis, Pfizer, Roche, I. Foeldvari Consultant with: Novartis, J. Rech Grant / Research Support with: Novartis, Sobi, Consultant with: AbbVie, Biogen, BMS, Chugai, GSK, Janssen, Lilly, MSD, Mylan, Novartis, Roche, Sanofi, Sobi, UCB, Speaker Bureau with: AbbVie, Biogen, BMS, Chugai, GSK, Janssen, Lilly, MSD; Mylan, Novartis, Roche, Sanofi, Sobi, UCB, F. Weller-Heinemann: None declared, A. Janda: None declared, M. Hufnagel Grant / Research Support with: Novartis, F. Meier Speaker Bureau with: Novartis, F. Dressler Grant / Research Support with: Novartis, Consultant with: Abbvie, Mylan, Novartis, Pfizer, M. Borte Grant / Research Support with: Pfizer, Shire, I. Andreica Consultant with: Abbvie, Chugai, Novartis, UCB, Galapagos, Takeda, Astrazeneca, Lilly, Boehringer Ingelheim, Amgen, Sobi, Paid Instructor with: Astrazeneca, UCB, Speaker Bureau with: Abbvie, Chugai, Novartis, UCB, MSD, Lilly, Sobi, Astrazeneca, Amgen, Pfizer, Gilead, P. Wasiliew: None declared, M. Fiene: None declared, D. Windschall: None declared, J. Weber-Arden Employee with: Novartis, T. Kallinich Speaker Bureau with: Roche

## P354 The safety and efficacy of Canakinumab treatment for non-differentiated autoinflammatory diseases: the preliminary results of retrospective cohort two-centered study

### M. Shingarova^1,2^, E. Alexeeva^1,2^, T. Dvoryakovskaya^1,2^, O. Lomakina^1^, A. Fetisova^1^, K. Isaeva^1^, A. Chomakhidze^1^, K. Chibisova^1^, I. Tsulukiya^1^, I. Kriulin^1,2^, E. Krekhova^1^, M. Botova^1^, N. Kondrateva^1^, T. Kriulina^1,2^, M. Kokina^1,2^, A. Kozodaeva^2^, K. Belozerov^3^, M. Kostik3^3^

#### ^1^Rheumatology, National Medical Research Center of Children's Health; ^2^Pediatric, Sechenov First Moscow State Medical University, Moscow; ^3^Pediatric, Saint-Petersburg State pediatric Medical University, Saint-Petersburg, Russian Federation

##### **Correspondence:** M. Shingarova

*Pediatric Rheumatology 2023*, **21(Suppl 2):**P354

**Introduction:** the blockade of interleukine-1 (anakinra and canakinumab) is a known high effective tool for monogenic autoinflammatory diseases (AID), such as FMF, TRAPS, HIDS and CAPS, but this treatment was note assessed for patients with other autoinflammatory diseases.

**Objectives:** The aim of our study was to assess safety and efficacy of canakinumab for patients with non-differentiated autoinflammatory disease (ndAID).

**Methods:** in the retrospective cohort study the information about 33 patients (13 boys and 20 girls) with ndAID from two tertial centers. The term ndAID includes children with clear features of AID, e.g. relapsed periodic fever, accompanied with increased CRP whom other reasons (infections, rheumatic, malignancy) of inflammation were excluded. Patients might have rashes, lymphadenopathia, arthralgia/arthritis, hepatosplenomegaly, oral aphthae. NGS (at least PID and AID panel) underwent in all patients and patients with known monogenic AID were excluded. Patients might be included in the study if their phenotype was not matched to previously patients have variants in known genes, they were included in the study if their clinical phenotype was not matched to previously described.

**Results:** The age of the first known episode was 2.5 (0.1-16.6) years, the age of disease diagnosis was 5.7 (0.3-23) years. The diagnostic delay was 1.0 (0.1-20) years. Patients have variants in the following genes: *IL10, NLRP12, STAT2, C8B, LPIN2, NLRC4, PSMB8, PRF1, CARD14, IFIH1, LYST, NFAT5, PLCG2, COPA, IL23, STXBP2, IL36RN, JAK1, DDX58, LACC1, LRBA, TNFRSF11A, PTHR1, STAT4, TNFRSF1B, TNFAIP3, TREX1, SLC7A7.*The main clinical features were fever (100%), rash (91%; maculo-papular predominantly), joint involvememnt (72.8%), splenomegaly (63.7%), hepatomegaly (57.6%), lymphadenopathy (48.5%), myalgia (27.2%), heart involvememnt (30.3%), intestinal involvement (18,1%); eye involvement 11.8%), pleuritis (15.1%), ascitis (3%), deafness, hydrocephalia (3%), delayed development (23.6). The signs of macrophage activation syndrome had 39.3% of the patients.

Initial non-biologic treatment before genetics was NSAID (91.2%), corticosteroids (85.3%), methotrexate (38.2%), IVIG (32.3%), cyclosporine A (23.6%), colchicines (5.8%) and biologics: canakinumab (38.2%), tocilizumab (61.8%), sarilumab, etanercept, adalimumab, rituximab, infliximab (all 3%).

The treatment after genetic was canakinumab (97%), and tocilizumab (3%). Canakinumab induced complete remission in 28 (85%), partial in 1 (3%). Two patients were primary non-responders, and two patients developed the secondary inefficacy further. All patients with partial efficacy or with inefficacy switched to tocilizumab (n=3), sarilumab (n=1), omalizumab (n=1). The total duration of canakinumb treatment was 3.6 (0.1; 8.7) years.

During the study there were no reported SAE. Patients had non-frequent mild respiratory infection with similar rate as before canakinumab.

**Conclusion:** the treatment of patients with ndAID with canakinumab was safe and effective. Further randomized clinical trials required to confirm the efficacy and safety.

**Patient Consent**

Not applicable (there are no patient data)

**Disclosure of Interest**

None declared

## P355 Genotypes and phenotypes patterns in patients with TNFRSF1А gene variants: single center experience

### M. Shingarova^1,2^, E. Alexeeva^1,2^, T. Dvoryakovskaya^1,2^, K. Isaeva^1^, A. Chomakhidze^1^, O. Lomakina^1^, A. Fetisova^1^, K. Chibisova^1^, E. Krekhova^1^, I. Kriulin^1,2^, I. Tsulukiya^1^, M. Botova^1^, N. Kondrateva^1^, M. Kokina^1,2^, T. Kriulina^1,2^, K. Savostyanov^3^, A. Pushkov^3^

#### ^1^Rheumatology, National Medical Research Center for Children’s Health; ^2^Pediatric, Sechenov First Moscow State Medical University (Sechenov University); ^3^Genetics, National Medical Research Center for Children’s Health, Moscow, Russian Federation

##### **Correspondence:** M. Shingarova

*Pediatric Rheumatology 2023*, **21(Suppl 2):**P355

**Introduction:** Tumor Necrosis Factor receptor - Associated periodic Syndrome (TRAPS) are a group of rare congenital auto-inflammatory diseases (AID) in an autosomal dominate manner and caused by variants in *TNFRSF1A* gene. The main difficulties in diagnosing TRAPS are the similarity of their clinical manifestations with other rheumatic diseases.

**Objectives:** To reveal genotype and phenotype characteristics in rheumatology department patients with *TNFRSF1A* gene variants at the National Medical Research Center of Children`s health, Moscow, Russia.

**Methods:** Retrospective study included 40 patients (26 females, 65%) with *TNFRSF1A* gene variants revealed by molecular genetic analysis and classified according ACMG criteria. Median age of debut was 5,2 (interquartile range (IQR) 0,12:15,5) years. The diagnosis of TRAPS was founded according Eurofever/PRINTO criteria.

**Results:** 5/40 (12,5%) patients had *TNFRSF1A* pathogenic variants: one each with *c.596T>C, c.374G>A, c.374G>A, c.194-14G>А,* and one had a combination of a pathogenic variant *c.242G>T* and VUS *c.362G>A*. Likely pathogenic variants had one patient - *c.184T>A* (2.5%); all heterozygous. 36/40 (90%) had VUS, the mostly *c.362G>A* variant 32 (80%), one each with *с.792delT*, *43C>T*, one had a combination two variant - *c.472+6С>Т* and *c.362G>A*; all heterozygous, and one had *c.362G>A* in homozygous. Prior to receiving the results of genetic testing, most of the patients in the study were observed with sJIA 28/40 (70%). After receiving the results the diagnoses were revised and 33/40 (82.5%) was diagnosed TRAPS. Clinical manifestations included fever in 32/40 (97%) patients, arthritis - in 31/40 (94%), rash - in 22/40 (66.7%), nervous impairment - in 19/40 (57.6%) and myalgia - in 14/40 (42.4%) patients. Intestinal changes by endoscopic examination were in 21/40 (63.7%) patients. Rarely we observed periorbital edema 3/40 (9%), sensorineural hearing loss in 3/40 (9%), affection eyes in 8/40 (24.2%) and aphthous stomatitis in one (3%). A comparative analysis clinical manifestations of patients with TRAPS 33/40 (82.5%) and patients with other diagnoses 7/40 (17.5%) revealed that fever is significantly more common with TRAPS 32/33 (97%), p=0.013, but affection eyes was more common in patients with other diagnoses 5/7 (71.4%), p=0.006. The laboratory activity was significantly higher in patients with TRAPS 33/40 (82.5%) than patients with other diagnoses 7/40 (17.5%): ESR 51.1 mm/h (IQR 36; 68) and 30 (IQR 24; 43) mm/h, p=0.02156; CRP 52 mg/l (IQR 40;60) and 10 (IQR 7,5;30,2), p= 0.001, accordingly.

**Conclusion:** 33 patients met criteria of TRAPS. The most frequent clinical manifestations were fever, arthritis, rash, myalgia and intestinal changes. Periorbital edema as criterion of diagnosis was registered only in 3/40 cases. The number of identified causal variants of the *TNFRSF1A* gene in these 33 patients showed high diversity. However, the variant *c.362G>A* was more common (80%), our results correspond the data obtained in other populations.

**Patient Consent**

Not applicable (there are no patient data)

**Disclosure of Interest**

None declared

## P356 Spectrum of systemic autoinflammatory diseases – experience from a tertiary care center in South India

### N. Singh^1^, J. Janardhanan^1^, S. Ramprakash^2^, R. C. P^2^, S. Bhattad^1^

#### ^1^Pediatric Immunology and Rheumatology; ^2^Pediatric Hematoncology and BMT, Aster CMI Hospital, Bengaluru, India

##### **Correspondence:** N. Singh

*Pediatric Rheumatology 2023*, **21(Suppl 2):**P356

**Introduction:** Systemic autoinflammatory diseases (SAIDs) are a growing group of disorders caused by a dysregulation of the innate immune system leading to episodes of systemic inflammation. They often present with recurrent episodes of fever and skin lesions, oral ulcers, and arthritis. Molecular genetics and next-generation sequencing technologies have increased our understanding of these diseases.

**Objectives:** To study the profile of autoinflammatory diseases in patients at a tertiary care centre in Bangalore, India.

**Methods:** A retrospective review of clinical records was performed, and patients with IEI were categorized according to the International Union of Immunological Societies (IUIS), Primary Immunodeficiency Diseases Committee Report on Inborn Errors of Immunity (2022). Patients with underlying IEI diagnosed with autoinflammatory disease were included in the study. A detailed analysis was performed to understand the types of autoinflammatory diseases.

**Results:** A total of 319 children with various IEI were diagnosed during the study period (February 2017 to Janaury 2023). Of these, 37 patients (6 adults, 31 children) were diagnosed to have systemic autoinflammatory diseases (SAID). Twenty out of 35 patients had non-inflammasome related diseases (54.2%), seven patients had Periodic fevers, aphthous stomatitis, pharyngitis, adenitis syndrome (PFAPA) (20 %), seven patients had diseases affecting inflammasome (20%), and two had Type I interferonopathies (5.7%) (Table 1). The male-to-female ratio was 1.1:1. The mean age at onset of symptoms and diagnosis was 4.4 years and 10.7 years respectively. The most common types of SAIDs were Chronic recurrent multifocal osteomyelitis (CRMO) (n=10), Cryopyrin associated Autoinflammatory syndromes (CAPS)(n=7), and PFAPA (n=7) followed by Blau syndrome (n=4), Otulin deficiency (n=3), Deficiency of Adenosine deaminase 2 (DADA2)(n=2), Pyogenic arthritis, pyoderma gangrenosum, acne (PAPA) syndrome(n=1), Majeed syndrome (n=1), TNF receptor associated periodic syndrome (TRAPS) (n=1), and Sideroblastic anemia, immunodeficiency, Periodic fevers, and developmental delay (SIFD) (n=1). The most common manifestations were recurrent or periodic fevers (n=15, 41%), rash (n=12, 32%), arthritis/arthralgia (n=12,32%), and recurrent oral ulcers (n=5, 13.5%). A whole exome sequencing was performed in 28 patients, and mutation were identified in 20 patients. Nonsteroidal anti-inflammatory drugs(NSAIDs) (n=11), corticosteroids(n=22), non-biologic disease–modifying anti-rheumatic drugs (DMARDS) n=13, and biologics (n=4) were used for treatment. DMARDs used were methotrexate (n=10), colchicine (n=6), leflunomide (n=2), thalidomide (n=2) and cyclosporine (n=1). Four patients (TRAPS, Otulin deficiency, DADA2, NOMID) were treated with biologics (Infliximab n=1, Tocilizumab n=1, Adalimumab n=1, Anakinra n=1) and showed a brisk response. Three CRMO patients were treated with Pamidronate and one SIFD patient received IVIg. Twenty-five patients remain on close follow, whereas a patient with SIFD died while undergoing a hematopoietic stem cell transplant.

**Conclusion:** We hereby present one of the largest single-centre cohort of SAIDs with a genetic confirmation in the majority.

**Patient Consent**

Yes, I received consent

**Disclosure of Interest**

None declared

## P357 The effectiveness of tonsillectomy in periodic fever, aphthous ulcer, pharyngitis, and adenitis syndrome in pediatric patients

### M. Twilt^1,2^, J. Storwick^1,2^

#### ^1^Pediatrics, Alberta Children's Hospital; ^2^Medicine, Univeristy of Calgary, Calgary, Canada

##### **Correspondence:** J. Storwick

*Pediatric Rheumatology 2023*, **21(Suppl 2):**P357

**Introduction:** Periodic Fevers with Aphthous Ulcers, Pharyngitis, and Adenitis Syndrome (PFAPA Syndrome) is the most common pediatric periodic fever syndrome. The most recent diagnostic criteria was described by Thomas et al. in 1999 and defines PFAPA as; regular periodic fevers lasting 3 to 7 days, occurring every 3 to 8 weeks, accompanied by at least one of the following symptoms: aphthous stomatitis, pharyngitis, and cervical adenitis. Between febrile episodes the patient is well and demonstrates normal growth and development. Treatment of PFAPA has considerable heterogenicity between practitioners, and there is no standard of care given a lack of clinical trials. Fortunately, PFAPA is relatively benign and will usually resolve spontaneously over time. In 1989, Abramson and colleagues described successful fever resolution post-tonsillectomy. Post-tonsillectomy, patients show a complete response with immediate cessation of fevers, a partial response with fever cessation followed by recurrence, or a failure with no fever cessation. The role of tonsillectomy in PFAPA treatment remains controversial.

**Objectives:** Our study aimed to determine the effectiveness of tonsillectomy as curative treatment of PFAPA compared to conservative non-surgical management.

**Methods:** We conducted a single center retrospective cohort study of children with PFAPA who were seen at the Alberta Children’s Hospital Rheumatology Clinic from January 2015 to July 2020. Inclusion criteria was based on the modified Thomas criteria for PFAPA. Exclusion criteria included patient with the Familial Mediterranean Fever gene variants, monogenic autoinflammatory diseases, and those that underwent tonsillectomy for another reason. We compared the time to fever resolution between the patients who underwent tonsillectomy and those who did not.

**Results:** We reviewed 113 charts of potential PFAPA patients and identified 34 patients who met inclusion criteria for typical PFAPA syndrome. 13 (38.2%) underwent tonsillectomy and 21 did not. In the tonsillectomy group, 10 were found to have complete response with immediate cessation of their fevers following tonsillectomy, 2 had a partial response, and 1 had no response. Patient characteristics were comparable; average age of fever onset was 2.9 and 3 years in the tonsillectomy and non-tonsillectomy groups and the average duration of PFAPA symptoms was 2.6 years and 3.2 years respectively.

**Conclusion:** Our study demonstrates that tonsillectomy may be beneficial in shorting the duration of PFAPA symptoms by approximately 6 months. Given the associated surgical risks, careful consideration should be given prior to recommending tonsillectomy as curative treatment.

**Patient Consent**

Not applicable (there are no patient data)

**Disclosure of Interest**

None declared

## P358 Reliability of genetic data for traps patients in French national rare disease database

### A. Subervie^1^ on behalf of FAI2R, S. Georgin-Lavialle^2^, H. Eric^3^, A. Belot^4^, A. Hot^5^, P. Quartier-Dit-Maire^6^, A. Aouba^7^, A. Desdoits^7^, D. Saadoun^8^, C. Richez^9^, P. Pillet^9^, I. Touitou^10^, Y. Benhamou^11^, M. Grall-Lerosey^11^, B. Granel^12^, O. Fain^13^, V. Queyrel^14^, U. Meinzer^15^, A. Lescoat^16^, I. Elhani^17^, V. Hentgen^1^

#### ^1^CEREMAIA, Centre Hospitalier André Mignot, Le Chesnay-Rocquencourt; ^2^Hôpital Tenon, Paris; ^3^CHU Lille, Lille; ^4^CHU Lyon, Bron; ^5^CHU Lyon, Lyon; ^6^Hôpital Necker APHP, Paris; ^7^CHU Caen, Caen; ^8^Hôpital Pitié Salpêtrière APHP, Paris; ^9^CHU Bordeaux, Bordeaux; ^10^CHU Montpellier, Montpellier; ^11^CHU Rouen, Rouen; ^12^Hôpital Nord APHM, Marseille; ^13^Hôpital Saint-Antoine APHP, Paris; ^14^CHU Nice, Nice; ^15^Hôpital Robert Debré APHP, Paris; ^16^CHU Rennes, Rennes; ^17^Hôpital Tenon APHP, Paris, France

##### **Correspondence:** A. Subervie

*Pediatric Rheumatology 2023*, **21(Suppl 2):**P358

**Introduction:** The “Banque de données nationales des maladies rares » (BNDMR) is a French national medical repository about rare diseases. This database is used by French Health authorities to monitor the activity of expert centers of rare diseases. Data entry in the BNDMR is compulsory for the French reference and expert centers. One of the aims of the database is to collect exhaustive data and allow epidemiological studies about rare diseases.

**Objectives:** Our goal was to test the capacity of the BNDMR to give reliable, ready to use, epidemiological data. To test this ability, we choose TNF Receptor Associated Periodic Syndrome (TRAPS), an ultra rare disease with an univocal genetic diagnosis.

**Methods:** We requested an extraction of all patients entered into the BNDMR with a diagnosis of TRAPS in January 2023. From January to March 2023, we went back to the clinical records to complete the genetic status of the *TNFRSF1A* gene of the included patients. We classified the genetic variants according to the infever classification. Data were analyzed with Excel software.

**Results:** At extraction, 132 patients in 23 different centers were eligible for our study. After exclusion of duplicates and patients in whom we were not able to retrieve the genetic status, we could perform the final analysis in 101 patients. Pathogenic and likely pathogenic variants concerned respectively 49 and 10 patients. Uncertain significant variants concerned 27 patients. For 14 patients, genetic test was not contributive and 1 patient had a not classified variant. Considering pathogenic and likely pathogenic variants, we found a prevalence of TRAPS in the French population of 1/1 315 000.

**Conclusion:** The BNDMR does not allow the performance of epidemiological studies without correction of data. Patients with a definite genetic diagnosis of TRAPS represent only 58% of patients recorded in the database. After exclusion of patients with variants of unknown origin and patients without mutations in the *TNFRSF1A* gene we found a French prevalence of TRAPS of 1/1 315 000, consistent with other European studies.

**Patient Consent**

Yes, I received consent

**Disclosure of Interest**

None declared

**References**

1. Lainka E, Neudorf U, Lohse P, Timmann C, Stojanov S, Huss K, et al. Incidence of TNFRSF1A mutations in German children: epidemiological, clinical and genetic characteristics. Rheumatology. 2009 Aug 1;48(8):987–91.

## P359 Profile of patients with chronic nonbacterial osteomyelitis from a single rheumatology pediatric center

### F. Sztajnbok, F. C. Zonis, M. F. Rodrigues, A. R. Fonseca, J. L. Monteiro, P. R. Souza, I. M. Paz, M. R. Vasti, R. G. Almeida

#### Pediatric Rheumatology, Universidade Federal do Rio de Janeiro, Rio de Janeiro, Brazil

##### **Correspondence:** F. Sztajnbok

*Pediatric Rheumatology 2023*, **21(Suppl 2):**P359

**Introduction:** Chronic nonbacterial osteomyelitis (CNO) belongs to the group of autoinflammatory diseases, is characterized by sterile bone inflammation, with or without systemic symptoms, and may be associated with other diseases.

**Objectives:** To describe clinical, laboratorial, histopathological and therapeutic aspects, and outcome, of a group of children and adolescents with CNO followed-up at a university pediatric rheumatology service, from 2000 to 2022.

**Methods:** A retrospective cross-sectional observational study was conducted through medical charts review of children and adolescents, diagnosed with CNO, at a pediatric rheumatology university center, followed-up from 2000 to 2022. Diagnosis was based on clinical evaluation and imaging and/or histopathological investigation.

**Results:** Data were collected from 16 patients (10 male, 6 female). The mean age at symptoms onset was 9.2 years. Mean time until diagnosis was 20 months (range 0-96). The mean follow-up time was 30.7 months (range 2-168). CNO was the initial diagnostic suspicion in 5 patients (31.2%). The number of bone lesions ranged from 1 to 22 (mean 9), mostly in the lower e upper limbs. Among the presented comorbidities, 9 patients (56.2%) had enthesitis and/or sacroiliitis. Other manifestations as inflammatory bowel disease, psoriasis, acne and lipodystrophy were also verified. At diagnosis, 11 patients (68%) had high acute phase reactants, 3 (18.7%) leukocytosis, 5 (31.2%) thrombocytosis, and 2 (12.5%) anemia. Among 11 patients tested, just one had HLAB27 detected, and presented with mandible CNO, fever, calcaneal enthesitis and chronic duodenitis. Whole-body MRI was performed in 12 patients (75%); and whole-body scintigraphy in 3 (18.7%). Bone biopsy was performed in 7 patients (43.7%) and suggested CNO in all of them. The medications used were nonsteroidal anti-inflammatory drugs, prednisone, methotrexate, sulfasalazine, adalimumab and etanercept. The use of 2 or more medications was necessary in all patients. Only one patient had a monocyclic course of disease, while the remainder (93.7%) had a persistent course.

**Conclusion:** Although CNO is more frequently described in females, our study had a male preponderance, involved multiple sites and was characterized by poor response to treatment. In addition, an association with enthesitis and/or sacroiliitis was present in more than half of the patients. Even though early diagnosis can be challenging, early intervention can reduce complications. Therefore CNO should be considered in cases of musculoskeletal pain in pediatrics, and full-body MRI should be requested in suspected cases.

**Patient Consent**

Yes, I received consent

**Disclosure of Interest**

None declared

## P360 А family case of autoinflammatory disease with a combination of two pathogenic mutations in the NLRP3 and MEFV genes

### A. Torgashina^1^, S. Salugina^2^, E. Fedorov^2^

#### ^1^Rare rheumatic Diseases, ^2^Pediatric Department, V.A.Nasonova Research Institute of Rheumatology, Moscow, Russian Federation

##### **Correspondence:** A. Torgashina

*Pediatric Rheumatology 2023*, **21(Suppl 2):**P360

**Introduction:** The clinical presentation of cryopyrin-associated periodic syndrome (CAPS) and familial Mediterranean fever (FMF) has been well studied, but very few studies have reported on the course of autoinflammatory disease (AID) with a combination of several pathogenic mutations.

**Objectives:** To report about family with members affected with AID with a combination of two pathogenic mutations in the NLRP3 and MEFV genes.

**Methods:** The clinical manifestation, laboratory test, treatment in patients were described.

**Results:** We report about two sisters, daughter of one of these sisters, their father who presented with symptoms of AID and mutations in genes MEFV (heterozygous p. Arg761His) and NLRP3 (heterozygous p. Val200Met).

Patient (pt) 1, AID was diagnosed at 46 years (yr) of age. First symptoms arthritis and fever, were from age 35. Further attacks were manifested by arthritis of large joints, fever up to 39, sometimes with uveitis or with urticaria rash, attacks were repeated several times a yr, their duration was up to several months (mo). During attacks, CRP was up to 35 mg/l, ESR 65 mm/h. Pt was diagnosed with AID predominance with CAPS symptoms. IL1 inh therapy normalized all parameters of AID.

Pt 2, AID was diagnosed at 41 yr of age. The first manifestations were at the age of 6 yr: bouts of abdominal pain, every 3 mo duration 1-3 days. At the age of 37, there were a fever, arthralgia and myalgia during an attack. Laboratory parameters were not controlled accurately during attacks, CRP values ​​were normal, but SAA was 32 mg/l (0-6,4 mg/l), even while taking colchicine. Colchicine stopped abdominal pain, but episodes of fever and arthralgia persisted. CRP values ​​were normal, but SAA was 30 mg/l while taking colchicine. Pt was diagnosed with FMF, colchicine resistance. IL1 inh was prescribed.

Daughter of the second pt, AID was diagnosed at 4 yr of age. From the age of 1.5 yr, attacks of febrile fever, pain in the abdomen, joints were disturbed with an interval of 1-2 mo, lasting 3-4 days. When taking colchicine, subfebrile condition and episodes of abdominal pain persist. CRP, ESR were normal, however SAA was 18 mg/l. No clear phenotype of a specific AID was observed. Pt monitoring continues.

These Pt had no hearing loss, no amyloid was found. Interestingly, the father of the pts, who had similar mutations, developed severe sensorineural hearing loss at the age of 35. No clear symptoms of AID were previously noted.

**Conclusion:** In the presented cases three generations of the same family with the same mutations were characterized by different age of onset of the AID and variable, non-obvious clinical symptoms. The development of deafness without previous clinical manifestations in an old family member necessitates active treatment of other sick family members.

**Patient Consent**

Yes, I received consent

**Disclosure of Interest**

None declared

## P361 CINCA/NOMID-cryopyrin-associated periodic syndrome diagnosed in an adult patient

### A. Torgashina^1^, S. Salugina^2^, E. Fedorov^2^

#### ^1^Rare Rheumatic Diseases; ^2^Pediatric Department, V.A.Nasonova Research Institute of Rheumatology, Moscow, Russian Federation

##### **Correspondence:** A. Torgashina

*Pediatric Rheumatology 2023*, **21(Suppl 2):**P361

**Introduction:** CINCA/NOMID (neonatal onset multisystem autoinflammatory syndrome) – one of the most severe phenotypes of CAPS (cryopyrin-associated periodic syndromes) with an early onset. The main clinical manifestations include fever, chronic urticarial rash (CU), arthropathy, CNS involvement, and elevated blood levels of inflammatory markers. Most CINCA/NOMID patients do not live to adulthood, have increased mortality compared with other CAPS phenotypes and can develop cognitive impairment, deafness and amyloidosis. About 40% CINCA/NOMID patients have no mutations.

**Objectives:** To describe our own experience in diagnosing and treating of adult patient with CINCA/NOMID.

**Methods:** The clinical manifestation, laboratory test, treatment in patient were described.

**Results:** Тhe first signs of the disease, CU, appeared at the age of several months. At the age of 1 yr 8 mo, arthritis of the knee joints, hepatomegaly, febrile FV, leukocytosis appeared. He was diagnosed with juvenile arthritis and received glucocorticoid therapy (GC), with incomplete effect. When trying to cancel GC, the FV recurred. The treatment with cyclophosphamide and methotrexate was ineffective. Hydrocephalus was diagnosed at the age of 5, and ventriculoperitoneal shunt was subsequently performed. Growth has stopped since the age of 6. From the age of 7, - hearing loss, cochlear implantation was performed at the age of 21. Mucopolysaccharidosis, osteochondrodysplasia and a number of other hereditary diseases were excluded. The patient for the first time came under observation in the federal center of rheumatology. The main clinical manifestation of the disease remains recurrent episodes of CU non-itchy rashes, episodes of FV. Laboratory data: CRP 46 mg/l, RF, ANA, ferritin and other laboratory markers were normal.

A biopsy of the duodenum was performed, amyloid masses were exposed. There are currently no signs of amyloidosis kidney disease.

In addition, there were specific clinical manifestations such as watch glass nails, spherical joint deformity, saddle-shaped deformity of the nose. The patient underwent a genetic examination. Despite the absence of mutations in the gene NLRP3, a diagnosis CINCA/NOMID was made. This diagnosis is supported by the infantile debut of the disease, episodes of FV, changes in the joints, early hearing loss with rapid progression of deafness, skull deformity and neurological symptoms, CU, acute phase inflammatory activity, insufficient response to antirheumatic therapy.

The administration of the IL1 inh led to a rapid and complete disappearance of inflammatory activity and rashes.

**Conclusion:** In the presented case, the diagnosis was established only 33 years after the onset of the disease, despite its typical course and bright phenotypic signs. It is necessary to increase the awareness of rheumatologists about diagnosis of this rare disease

**Patient Consent**

Yes, I received consent

**Disclosure of Interest**

None declared

## P362 A different way of treating TRAPS syndrome?

### M. Garrido-Martín, A. Remesal, C. Millán-Longo, B. Diaz-Delgado, C. Udaondo, R. Alcobendas

#### Hospital Universitario La Paz, Paediatric rheumatology, Madrid, Spain

##### **Correspondence:** C. Udaondo

*Pediatric Rheumatology 2023*, **21(Suppl 2):**P362

**Introduction:** Tumor necrosis factor receptor-associated periodic syndrome (TRAPS) is characterized by recurrent episodes of fever, lasting 1-3 weeks, associated with myalgia, conjunctivitis, periorbital oedema, abdominal pain, arthralgia/arthritis, and skin rash.

**Objectives:** We present a clinical case its management and outcome.

**Methods:** Medical chart review

**Results:** We describe the case of a patient first seen in 2016 for episodes of fever lasting 15 days and abdominal pain with elevated acute phase reactants, which subsequently normalized after the resolution of the symptoms.

His mother had been diagnosed in childhood with familial Mediterranean fever, without diagnostic confirmation, and treated with colchicine with a good response.

Because of the clinical manifestations and the family history, a genetic study was carried out, in which a mutation was found: c.197C>T, p.(Thr661Ile) in the TNFRSF1A gene.

Initially, the flare-ups were treated with prednisone with a good response, maintaining normal serum amyloid A between crises. However, the frequency of these flare-ups increased, so a treatment with anakinra was started, also with a good response. Given the difficulty for the patient to adhere to the daily subcutaneous treatment and given the immediate response to a single dose, we decided to administer anakinra for only 7 days during the crises.

With this regime, the patient controlled the disease adequately and had good adherence. However, in the last year, the crises have become more frequent and in the last inter-crisis analysis, an increase in serum amyloid A has been observed.

Due to the worsening of the patient and his refusal to receive the daily injection, but given the good response to IL-1 blockade, we decided to start canakinumab.

At the same time, his younger brother presented episodes suggestive of TRAPS, and a genetic study was performed, identifying the same mutation. He is currently well-controlled with anakinra during flare-ups. The mother also has the same mutation, is currently asymptomatic, untreated, and has started follow-up by Internal Medicine.

**Conclusion**

- IL-1 blockade is effective in patients with TRAPS.
- Anakinra is the first choice both to evaluate the initial response and as maintenance treatment as well as given its cost-effectiveness compared to prolonged treatment with canakinumab.
- The immediate response to a single dose of anakinra ad lib in this patient worked as a substitute for corticosteroids. However, the need to use anakinra more frequently and the increase in inter-crisis serum amyloid A have led to a switch to canakinumab.

**Patient Consent**

Yes, I received consent

**Disclosure of Interest**

None declared

## P363 Experience of on-demand Anakinra therapy in autoinflammatory diseases

### K. Ulu, B. Sözeri

#### ^1^Pediatric Rheumatology, Ümraniye Training and Research Hospital, Istanbul, Türkiye

##### **Correspondence:** K. Ulu

*Pediatric Rheumatology 2023*, **21(Suppl 2):**P363

**Introduction:** The utilization of on-demand anakinra in the management of autoinflammatory diseases has increased in recent years. Autoinflammatory diseases, characterized by dysregulated innate immune responses, often result in recurrent and debilitating inflammatory flares. Anakinra, a recombinant interleukin-1 receptor antagonist, has demonstrated remarkable efficacy in suppressing these flares and improving patients' quality of life. Several studies have investigated the use of on-demand anakinra, wherein the medication is initiated upon the onset of symptoms or flare-ups rather than as a continuous prophylactic treatment.

**Objectives:** The aim of this study was to evaluate the efficacy of on-demand anakinra therapy in the flare-ups phase of autoinflammatory diseases.

**Methods:** The patients diagnosed with autoinflammatory disease who were followed up in the pediatric rheumatology department of Ümraniye Training and Research Hospital and applied on demand anakinra due to severe acute attack were included in the study.

**Results:** A total of 13 patients (F:8 M:5) diagnosed with autoinflammatory disease were included in the study. The mean (SD) age and age of diagnosis of the cases were 13.1 (4.9) and 8.1 (4.7) years, respectively. Seven of the cases were followed up with FMF, 4 with syndrome of undifferentiated recurrent fever, 1 with mevalonate kinase deficiency and 1 with NLRP12 associated autoinflammatory disease. Of the 13 patients with autoinflammatory diseases, 11 had abdominal pain, 5 had fever, 5 had diarrhea, 1 had rash, 1 had erysipelas-like erythema, and 1 had protracted febrile myalgia. The mean duration of attack was 4.1 (1.1) days. Acute phase responses were high in all cases. Mean CRP, ESR and SAA values were 14.2 (7,2) mg/dl, 68 (37.7) mm/h, 95.9 (76.6) mg/dl, respectively. The mean number of days of anakinra administration was 3 (1.1) and the maximum dose was 2.8 (0.8) mg/kg. After anakinra treatment, CRP became negative in an average of 3.5 (0.8) days and the flare-ups ended in 1.2 (0.5) days. After exacerbations, treatment with anakinra in two patients and canakinumab in three patients was continued in addition to colchicine treatment. No anakinra-related side effects were observed in the patients included in the study.

**Conclusion:** This study highlights the potential of on-demand anakinra as an effective therapeutic strategy in managing autoinflammatory diseases flare-ups. Improving knowledge about the on-demand use of anakinra may help clinicians to guide timely and effective treatment. Larger cohort studies are needed to determine the best treatment approach and dose and to monitor possible side effects.

**Patient Consent**

Yes, I received consent

**Disclosure of Interest**

None declared

## P364 Mitochondrial derived peptides in patients with familial Mediterranean fever

### S. Üstündağ^1^, S. Yüksel^2^

#### ^1^Pediatrics, Alaşehir State Hospital, Manisa; ^2^Pediatric Rheumatology, Pamukkale University Hospital, Denizli, Türkiye

##### **Correspondence:** S. Üstündağ

*Pediatric Rheumatology 2023*, **21(Suppl 2):**P364

**Introduction:** In the pathogenesis of familial Mediterranean fever (FMF), pyrin plays a role in the regulation of inflammasomes together with proteins such as NLRP3 (1). Recently, it has been discussed that mitochondria may play a role in the pathogenesis of some autoinflammatory disorders in relation to NLRP3 and reactive oxygen species (2). The discovery of mitochondrial derived peptides (MDP), related to mitochondiral stress, suggested a more complex interaction between these relationships (3). MDPs (Humanin and MOTS-c) have been shown to have anti-inflammatory properties (4, 5).

**Objectives:** This study aims to determine how changing MDPs levels in different clinical forms of children with FMF. The best our knowledge, the study is the first in the literature.

**Methods:** This cross-sectional and prospective study was consisted of children with FMF, who applied to pediatric rheumatology and emergency divisions in Pamukkale University Hospital. According to power analysis (f=0.3, 95% confidence, 80% power), a total 148 children were included the study. The patient subgroups were described as attack, newly diagnosed and remission (37 children for each). Control group were matched according to age and sex consisted of 37 healthy children. MDP levels were analyzed by ELISA. Comparisons were done between FMF subgroups as well as the patient and healthy groups.

**Results:** MOTS-c levels were significantly higher in the patient group than the healthy group (30.37±15.1 vs 20.14±2.3 ng/L respectively, p=0.0001). Humanin levels were significantly higher in the healthy group than the patient group (555.26±253.4 vs 486.69±331.5 ng/L, respectively, p=0.024). Humanin levels were also significantly lower in the colchicine users (all of outpatients) than non-users (newly diagnosed and control), (408.3±400.0, 370.96±245.9 vs 602.20±337.2, 555.26±253.4 ng/L, respectively, p=0.0001).

**Conclusion:** This study showed that MDP levels significantly different between patients and healthy controls, also between FMF subgrups. These results are a clear example of the need for further investigation of mitochondrial stress in FMF patients. Further studies revealing the relationship between FMF and mitochondrial stress may contribute to a better understanding of the pathogenesis of autoinflammatory diseases.

**Patient Consent**

Not applicable (there are no patient data)

**Disclosure of Interest**

None declared

**References**

1. Skendros P, Papagoras C, Mitroulis I, Ritis K. Autoinflammation: Lessons from the study of familial Mediterranean fever. J Autoimmun. 2019;104(July):102305.
2. Savic S, Dickie LJ, Wittmann M, McDermott MF. Autoinflammatory syndromes and cellular responses to stress: Pathophysiology, diagnosis and new treatment perspectives. Best Pract Res Clin Rheumatol. 2012;26(4):505–33.
3. Troy L et al. “Mitochondrial-derived peptides in energy metabolism.” American journal of physiology. Endocrinology and metabolism vol. 319,4 (2020): E659-E666. doi:10.1152/ajpendo.00249.2020
4. Li W, Zhang D, Yuan W, Wang C, Huang Q, Luo J. Humanin Ameliorates Free Fatty Acid-Induced Endothelial Inflammation by Suppressing the NLRP3 Inflammasome. ACS Omega. 2020 Sep 8;5(35):22039–45.
5. Yin X, Jing Y, Chen Q, Abbas AB, Hu J, Xu H. The intraperitoneal administration of MOTS-c produces antinociceptive and anti-inflammatory effects through the activation of AMPK pathway in the mouse formalin test. Eur J Pharmacol. 2020 Mar 5;870

## P365 The role of MIR-204-3P, MIR-223-3P as epigenetic factors, its targeted cytotoxic T lymphocyte antigen 4 and deltex 1 in familial Mediterranean fever

### H. Uzun^1^, R. Hajiyeva^2^, S. Sahin^3^, S. Durmus^2,4^, A. Adrovic^5^, O. Kasapcopur^3^

#### ^1^Faculty of Medicine, Medical Biochemistry, Istanbul Atlas University; ^2^Istanbul University-Cerrahpasa, Istanbul, Türkiye; ^3^Faculty of Medicine, Pediatric Rheumatology; ^4^Department of Medical Biochemistry, Istanbul University-Cerrahpasa; ^5^Pediatric Rheumatology, KOC University, American Hospital, Istanbul, Türkiye

##### **Correspondence:** H. Uzun

*Pediatric Rheumatology 2023*, **21(Suppl 2):**P365

**Introduction:** It has been reported that miR-107, let-7d-5p and miR-148b-3p are down-regulated and miR-144-3p, miR-21-5p, miR-4454 and miR-451a levels are up-regulated in FMF patients. miR-107 and miR-148 are down-regulated in FMF patients, while miR-21 is up-regulated, showing a general pro-inflammatory profile of the innate immune system of FMF patients even in the attack-free period. Most importantly, all these miRNAs have been reported to be involved in immune processes. miR-223 levels, which may be associated with FMF disease, were found to be significantly down-regulated in patients

**Objectives:** Familial Mediterranean fever (FMF) is the most common hereditary autoinflammatory disease in the world. We investigated the efficacy of serum miR-204-3p, miR-223-3p, plasma pyrin, deltex 1 (DTX1), cytotoxic T-lymphocyte-associated antigen gene-4 (CTLA-4) levels in FMF patients and their relationship with the pathogenesis and progression of FMF.

**Methods:** Forty-eight children with FMF and 36 healthy children were included in the study. Serum miR-204-3p and miR-223-3p levels were measured by PCR method and plasma pyrin, DTX1, CTLA4 levels were measured by sandwich ELISA method.

**Results:** There was no statistical significance between the groups in plasma CTLA-4 levels. Serum miR-204-3p, miR-223-3p, plasma DTX1 levels were found to be significantly lower in FMF patients, while plasma pyrin levels (p<0.05, in all) were significantly higher. CTLA-4 levels were positively correlated with pyrin and DTX1 levels (r=0.602; p<0.001; r=0.740; p<0.001, respectively).

**Conclusion:** miR-204-3p and miR-223-3p may be effective in the pathogenesis of FMF. Increased levels of the pyrin protein encoding the *MEFV* gene may have an important role in apoptotic and inflammatory signaling pathways. A decrease in DTX1 levels and a positive correlation between DTX1 and CTLA-4 suggest that subclinical inflammation may continue in attack-free periods in FMF patients.

**Patient Consent**

Yes, I received consent

**Disclosure of Interest**

None declared

**References**

1. Tufan A, Lachmann HJ. Familial Mediterranean fever, from pathogenesis to treatment: a contemporary review. Turk J Med Sci. 2020;50(SI-2):1591-610.
2. Yıldız M, Haşlak F, Adrovic A, Barut K, Kasapçopur Ö. Autoinflammatory Diseases in Childhood. Balkan Med J. 2020;37:236-46.
3. Zadeh N, Getzug T, Grody WW. Diagnosis and management of familial Mediterranean fever: integrating medical genetics in a dedicated interdisciplinary clinic. Genet Med. 2011;13:263-69.
4. Bhatt H, Cascella M. Familial Mediterranean Fever. In: StatPearls. Treasure Island (FL): StatPearls Publishing; August 1, 2022.
5. Telatar M, Grody WW. Molecular genetic testing for familial Mediterranean fever. Mol Genet Metab. 2000;71:256-60.
6. Schnappauf O, Chae JJ, Kastner DL, Aksentijevich I. The Pyrin Inflammasome in Health and Disease. Front Immunol. 2019;10:1745.

## P366 PFAPA syndrome in twins: a case series report

### Y. Vyzhga, N. Tokarchuk

#### National Pirogov Memorial Medical University, Vinnytsya, Ukraine

##### **Correspondence:** Y. Vyzhga

*Pediatric Rheumatology 2023*, **21(Suppl 2):**P366

**Introduction:** Autoinflammatory diseases are characterized by immune system dysregulation, resulting in inflammation and systemic symptoms. PFAPA (Periodic Fever, Aphthous stomatitis, Pharyngitis, and Adenitis) syndrome is a common autoinflammatory disease in children, but its epidemiological features, especially in twins, remain incompletely understood.

**Objectives:** This case series report describes the clinical presentation and management of PFAPA syndrome in three pairs of twins.

**Methods:** Follow-up for the group of patients was conducted through regularly planned visits between the years 2022 and 2023. These visits allowed for the monitoring and assessment of the patients' condition over time. The patients' response to treatment and any changes in symptoms were evaluated during these follow-up visits.

**Results:** In the first pair of girls, born through in vitro fertilization (IVF), PFAPA symptoms appeared when they were 4.8 years old. The first girl experienced the onset of fever with a typical PFAPA pattern at the age of 4, while the second girl developed symptoms six months later. During each episode, which occurred once every 6-7 weeks, the girls exhibited symptoms of fever, pharyngitis, and cervical adenopathy. No other symptoms were observed during these episodes.

In the second pair of boys, only one child exhibited clinical signs of periodic fever. The disease onset occurred when he was 5 years old. The fever episodes were associated with symptoms such as pharyngitis, periodic oral aphthous ulcers, abdominal pain, and nausea. These episodes followed an intermittent pattern, lasting up to 5 days, and recurred every 1.5 to 2 months. Interestingly, the second child in this pair did not develop any typical symptoms of PFAPA during the period of observation. Genetic testing was conducted for both children, resulting in the detection of a variant of uncertain significance (VUS).

In the third pair of boys, the disease manifested when they were 5.9 years old. The first child experienced the initial symptoms at the age of 5, and the second child developed a similar presentation eight months later. Both children were born through IVF. A typical fever pattern emerged, occurring every 4-6 weeks and lasting for 5-6 days during each episode. Along with pharyngitis and cervical adenopathy, the boys also exhibited symptoms such as abdominal pain and vomiting. Genetic testing was performed for both children, revealing the presence of a VUS.

Longitudinal observation and appropriate treatment were provided based on the diagnoses.

**Conclusion:** While the association between IVF and autoinflammatory diseases, particularly in twins, remains limited and conflicting in current literature, this case series highlights the need for further research and collaboration to enhance understanding and optimize medical care for this specific patient population.

**Patient Consent**

Yes, I received consent

**Disclosure of Interest**

None declared

## P367 Musculoskeletal involvement in Type I interferonopathies: description of the clinical and radiological aspects among 29 patients

### T. Wauquier^1^, S. Breton^2^, B. Neven^1^, I. Melki^3^, B. Bader-Meunier^1^, M.-L. Frémond^1^

#### ^1^Pediatric Immunology Hematology Rheumatology Unit; ^2^Pediatric Radiology, Necker Hospital; ^3^Pediatric Rheumatology Unit, Robert Debré Hospital, Paris, France

##### **Correspondence:** T. Wauquier

*Pediatric Rheumatology 2023*, **21(Suppl 2):**P367

**Introduction: Type I interferonopathies**, i.e. monogenic auto-inflammatory diseases caused by constitutive activation of the type I interferon pathway, can be associated with variable phenotype. While neurological, cutaneous and pulmonary involvement are being well described in a number of these diseases, **musculoskeletal features remain poorly reported.**

**Objectives:** To describe the musculoskeletal involvement among 29 patients presenting with type I interferonopathy.

**Methods: Retrospective** cohort of patients diagnosed with Mendelian type I interferonopathy at Necker Hospital between 2014 and 2019, collecting clinical and biological findings, bone mineral density measurements. A systematic review of all radiological images was assessed by an expert radiologist.

**Results:** The cohort included 8 patients with **SAVI** (*STING1/TMEM173* mutation), 7 patients with **AGS** (Aicardi-Goutières syndrome), 3 patients with **SPENCD** (spondyloenchondrodysplasia), 3 patients with **COPA syndrome**, 2 patients with **DNAseII deficiency**, 2 patients with **RNAseTII mutation**, 1 patient with **PRAAS/Candle syndrome**, and 3 patients with presumed monogenic type I interferonopathy.

The median age at disease onset was 6 months, and median age at clinical diagnosis was 8 years (0.6 – 25 years).

71% of patients (20 of 28) presented joint involvement, encompassing arthritis (n = 9), joint **retractions apart from spasticity** (n = 7) and **radiological damage** [joint pinching or bone erosions or misalignment/luxation/subluxation or bilateral femoral head necrosis] (n = 20). 50% (9 of 18) had a positive rheumatoid factor. Three patients had typical **Jaccoud's arthropathy.**

**53.8% of patients (7 of 13) had a mean whole body bone mineral density of less than - 2 DS**. We also noted **subcutaneous calcinosis** in 4 individuals and radiological **juxta-articular calcifications** in 2 of them. Increased CPK (creatine phosphokinase) was observed in 9 patients (36%).

76% of patients (22 of 29) were treated with JAK inhibitors and improvement of joint damage was described in 7 of them: 3 patients were considered to be in complete joint remission and 4 presented partial improvement.

**Conclusion:** Overall, musculoskeletal involvement is a more common feature than expected in Mendelian interferonopathies in terms of clinically or radiologically-defined joint involvement, bone demineralisation and also musculotendinous features. The role of the type I interferon pathway in the underlying pathogenesis in these manifestations deserves further investigation.

**Patient Consent**

Yes, I received consent

**Disclosure of Interest**

None declared

## P368 Juvenile dermatomyositis the pediatric rheumatologic consultation in Albania from 2012-2022

### G. Xhelilaj, N. Kuneshka, A. Vula, A. Shkembi, E. Hysenaj

#### General Pediatrics, Mother Teresa Hospital, Tirana, Albania

##### **Correspondence:** G. Xhelilaj

*Pediatric Rheumatology 2023*, **21(Suppl 2):**P368

**Introduction:** Juvenile Dermatomyositis is a rare autoinflammatory disease of the muscles and the skin that can affect all ages and ethnic groups, characterized by a variety of symptoms which can appear suddenly or gradually. It is the most common type of juvenile idiopathic inflammatory myopathy

**Objectives:** The purpose of our study is to identify the cases of Juvenile Dermatomyositis in Albania from 2012 to 2022.

**Methods:** In our retrospective study we have reviewed the Pediatric Rheumatologic consultation database at the Mother Theresa Hospital of Tirana and identified the cases that were diagnosed with Juvenile Dermatomyositis. The information gathered was about the major symptoms at the time of the consult, sex, age, laboratory values and their follow up.

**Results:** We gathered a total number of 5200 consultations of which 19 were diagnosed and 4 were suspected as Juvenile Dermatomyositis. We have seen there was no preference based on sex but there was an age preference between 6-12 years old of which 6 out of 19 cases were diagnosed at the age of 12, the youngest was 5 years old

**Conclusion:** Juvenile Dermatomyositis remains a rare disease, 19 cases out of 5,200 consultations. Furthermore studies are needed to find incidence and prevalence in the population.

**Patient Consent**

Not applicable (there are no patient data)

**Disclosure of Interest**

None declared

**References**

1. 2020 May;30(3):411-423 Clinical practice guidance for juvenile dermatomyositis (JDM) 2018

## P369 The potential role of cell-death mechanisms in the pathogenesis of FMF attacks

### E. Yaglikara^1^, O. Boluk^1^, Y. Bayindir^2^, S. Demir^3^, Y. Bilginer^2^, M. A. Tasar^4^, S. Ozen^2,5^, E. Sag^1,5^

#### ^1^Pediatric Rheumatology, Ankara Training and Research Hospital; ^2^Pediatric Rheumatology, Hacettepe University, Ankara; ^3^Pediatric Rheumatology, Eskisehir Osmangazi University, Eskisehir; ^4^Pediatric Emergency, Ankara Training and Research Hospital; ^5^Translational Medicine Laboratory, Pediatric Rheumatology Unit, Hacettepe University, Ankara, Türkiye

##### **Correspondence:** E. Yaglikara

*Pediatric Rheumatology 2023*, **21(Suppl 2):**P369

**Introduction:** FMF is the most common autoinflammatory disease, characterized by recurrent and self-limited attacks of fever, abdominal pain, arthritis, serositis and skin features. Although the primary responsible mechanism starting the attacks is the activation of pyrin inflammasome, it is still controversial why the attacks end in 3-4 days and what is the mechanism ending these attacks.

**Objectives:** In this study we aimed to define the importance of cell-death mechanisms in the pathogenesis of FMF attacks.

**Methods:** We included 25 FMF patients admitted to our clinic and as for the control group PFAPA patients (n=10) and healthy controls (HC, n=10) were included, as well. We collected plasma samples from FMF and PFAPA patients during the attack and attack-free period. We measured the soluble plasma levels of sFas, sFasL, granzyme A, granzyme B, perforin, granulysin, IL-2, IL-4, IL-10, IL-6, IL-17A, TNF-α, IFN-γ by commercial pre-defined cytometric bead array kits.

**Results:** There were no significant difference between groups in terms sex (FMF 52% male; PFAPA 60% male; HC 60% male; p=0.664). The age of FMF patients and HC were comparable (FMF 8.5±4.5 yrs vs HC 9.9±4.2 yrs p=0.664) but PFAPA patients were younger (PFAPA 3.8±2.1 yrs) than other groups due to the nature of the disease. When compared to attack-free period, IL-6 levels were higher during attacks of both FMF (p=0.001) and PFAPA (p=0.01) patients but IL-4 (p=0.025) and IL-10 (p=0.039) levels were high only during attack period of FMF patients. We then analyse the components of apoptosis and pyroptosis. In FMF patients, the levels of sFasL (attack 102.6±58.7 pg/mL vs attack-free 70.3±45.6 pg/mL; p=0.035) and Granzyme A (attack 3840.2±3273.7 pg/mL vs attack-free 2135.5±2283.1 pg/mL; p=0.038) were significantly increased during attack period and became comparable to the levels of HC during attack-free period. This increase was not seen in PFAPA patients, where they had comparable levels with HC group both during attack period and attack-free period. Granzyme B (attack 4029.2±851.3 pg/mL vs attack-free 3002.1±1958.2 pg/mL; p=0.145) and Perforin (attack 9684.8±16180.4 pg/mL vs attack-free 5300.8±4603.5 pg/mL; p=0.203) levels were also increased during attack period of FMF patients however these differences were not statistically significant. The levels of sFasL, Granzyme A, Granzyme B and Perforin were closely correlated with each other during the attack period of FMF patients.

**Conclusion:** Our study reveals the importance of apoptosis and pyroptosis in the mechanism of FMF attacks which was different from PFAPA patients. These findings might shed light the reason for the nature of self-limited attacks but further studies are needed to prove this hypothesis.

**Patient Consent**

Not applicable (there are no patient data)

**Disclosure of Interest**

None declared

## P370 Long term follow-up of anti-IL1 treated pyrin associated auto-inflammatory disease a case report

### N. Zekri^1^, F. Magnotti^2^, G. Sarrabay^3^, Y. Jamilloux^4^, A. Rey^5^, E. Merlin^1^

#### ^1^General Pediatrics Department, University Hospital Estaing of Clermont-Ferrand, Clermont-Ferrand;^2^CIRI, Centre International de Recherche en Infectiologie, Inserm 1111, Lyon; ^3^Department of Medical Genetics, Rare Diseases and Personalized Medicine, Stem Cells, Cellular Plasticity, Regenerative Medicine and Immunotherapies, INSERM, University of Montpellier, CHU Montpellier , Montpellier; ^4^Departments of Internal Medicine, Hospices Civils de Lyon, Hôpital Universitaire de la Croix-Rousse, Lyon; ^5^General Pediatrics Department, Henri Mondor Hospital (Aurillac), Aurillac, France

##### **Correspondence:** N. zekri

*Pediatric Rheumatology 2023*, **21(Suppl 2):**P370

**Introduction:** Familial Mediterranean Fever is the most common of all described hereditary periodic fever syndromes. It has long been defined as a genetic auto-inflammatory disorder with autosomal recessive inheritance. With the increasing awareness of FMF and recent advances in the genetic testing, information concerning new patterns of inheritance, new MEFV gene mutations, genotype-phenotype relations and new treatment modalities have been accumulated. Therefore, Pyrin Associated Auto-inflammatory Disease (PAAD) was recently proposed to encompass all diseases associated with pyrin defects or MEFV mutations.

**Objectives:** Highlighting the benefits of interleukin-1 blockers in the clinico-biological management of severe and colchicine-resistant early-onset PAAD.

**Methods:** We report the follow-up of a three year-old Kurdish girl with confirmed colchicine resistant PAAD diagnosis treated by interleukin-1 blockers for 3 years.

**Results:** A three-year-old Kurdish girl with a family history of consanguineous marriage and personal history of hirsutism presented with recurrent attacks of febrile monoarthritis (mainly ankle and knee) which occurred initially twice per year and lasted for 7 days. The synovial aspiration showed culture-negative pus with very high synovial white blood cell counts. She was initially treated with antibiotics for each attack with a seemingly favorable outcome, but relapses occurred more and more early, with intervals of 1-2 weeks. Naproxen sodium, Methotrexate and Etanercept then tocilizumab didn’t prove any clinical benefit. Steroids based treatment was effective, but inflammation relapsed as soon as cessation. Genetic analyses by targeted next-generation sequencing found MEFV de novo heterozygous mutation p. Thr577Asn in exon 8. Steroids and Tocilizumab were stopped and Colchicine at 0.5mg per day was started without approved efficacy. Therefore, Anakinra was associated at 2mg/kg/day by subcutaneous injection. No attacks had been reported after 2 months of interleukin-1 inhibitors-based treatment, but arthritis recurred as soon as the treatment was administered every other day. Thus, Anakinra was pursued daily. Three years later, the patient is still in complete clinical remission with normal inflammatory work-up (CRP, SAA) and biochemistry (liver enzymes, serum creatinine and urinalysis) .

**Conclusion:** We report a 3-year-old girl with de novo heterozygous MEFV mutation in exon 8. She exhibited recurrent febrile arthritis, she was colchicine refractory, and she was maintained in clinical and biological remission with daily anakinra.

**Patient Consent**

Yes, I received consent

**Disclosure of Interest**

None declared

## P371 Potential tear-based biomarkers to differentiate JIA with and without uveitis: a pilot study

### I. Maccora^1,2,3^, M. Altaye^4^, T. Nguyen^5^, K. Greis^6^, W. Haffey^6^, A. Sproles^3^, S. Thornton^3^, V. Miraldi Utz^7^, S. Angeles-Han^3^

#### ^1^Rheumatology Unit, Meyer Children's Hospital IRCCS; ^2^NeuroFARBA Department, University of Florence, Florence, Italy; ^3^Division of Rheumatology, Cincinnati Children's Hospital, Medical Centre; ^4^Epidemiology and Biostatistics, Cincinnati Children's Hospital Medical Center; ^5^Cincinnati Eye Institute; ^6^UC Proteomics Laboratory, Department of Cancer Biology, University of Cincinnati College of Medicine; ^7^Division of ophthamology, Cincinnati Children's Hospital, Medical Centre, Cincinnati, United States

##### **Correspondence:** I. Maccora

*Pediatric Rheumatology 2023*, **21(Suppl 2):**P371

**Introduction:** Children with oligoarticular JIA are at increased risk of developing uveitis. JIA category, ANA positivity, ≤4 years JIA duration, and <6-years-old at diagnosis are known risk factors for uveitis but do not accurately predict development. Tear fluid is easily obtained and has potential for studying diagnostic biomarkers of ocular inflammation.

**Objectives:** In this pilot study, we aim to identify potential tear-based inflammatory markers associated with uveitis development in children with JIA.

**Methods:** In this comparative cohort at Cincinnati Children’s with JIA-associated uveitis (JIA-U) and JIA without uveitis (JIA-no-U), participants with high-risk factors were enrolled including: female, non-Hispanic, white and oligoarticular subtype. Medical records were reviewed for demographics, ophthalmic examination, uveitis characteristics if present, and current treatment. Tear fluid was collected using Schirmer strips. We used advanced proteomic strategies (isobaric tag for relative quantitation [iTRAQ] labeling and nanoLC-MS/MS) to quantify and compare proteins in JIA-U and JIA-no-U. A P-value ≤0.05, based on Wilcoxon rank sum exact test, was considered significant.

**Results:** Nine proteins exhibited differences in tears of 5 children with JIA-U and 5 with JIA-no-U. These proteins are associated with inflammatory arthritis and general inflammatory response pathways. Keratin type II cytoskeletal 2 epidermal, cathepsin D, Ras-related C3 botulinum toxin substrate 1, C1-tetrahydrofolate synthase cytoplasmic demonstrated notably higher expression in tears of those with JIA-U. Transitional endoplasmic reticulum ATPase, alpha-2-macroglobulin, proteasome subunit beta type-8, perlipin-3, and fibrinogen alpha chain were higher in JIA-no-U. These proteins are associated with the immune response, specifically at the level of retinal pigment epithelium (RPE) and blood retinal barrier (BRB).

**Conclusion:** JIA-associated uveitis is a vision-threatening, often asymptomatic disease where early detection and treatment improve patient outcomes. The eye is an immune-privileged organ, immunologically shielded by the BRB. We hypothesize that the immune response proteins identified within the tears of JIA-U patients are byproducts of inflammation-induced RPE and BRB breakdown that furthers the inflammatory cascade. Tears of children with JIA-U and JIA-no-U display differential expression of proteins that may be used to predict early clinical uveitis for those diagnosed with oligoarticular JIA. Further, inflammatory mediators that differentiate ocular and joint inflammation may provide insight in the pathogenesis of both JIA and uveitis. Further study in larger cohorts is needed to verify these results.

**Patient Consent**

Yes, I received consent

**Disclosure of Interest**

None declared

## P374 Development of a therapeutic range of Adalimumab serum concentrations in the management of childhood chronic non-infectious uveitis, a step towards personalised treatment

### J. Dehoorne^1^, H. Groth^2^, E. Carlé^2^, I. De Schrijver^3^, C. Sys^3^, P. Delbeke^4^, K. Elke^3^, T. Renson^1^, C. Bonroy^5^

#### ^1^Department of Paediatric Rheumatology, Ghent University Hospital; ^2^Ghent University Faculty of Medicine and Health Sciences; ^3^Department of Ophthalmology, Ghent University Hospital, Gent; ^4^Department of Ophthalmology, AZ Sint-Jan, Brugge; ^5^Department of Diagnostic Sciences, Ghent University Hospital, Gent, Belgium

##### **Correspondence:** J. Dehoorne

*Pediatric Rheumatology 2023*, **21(Suppl 2):**P374

**Introduction:** Although the use of adalimumab has significantly improved the visual outcome of children with non-infectious uveitis (CNU), some patients do not respond sufficiently or lose response over time. The use of therapeutic drug monitoring can be useful to optimise adalimumab efficacy.

**Objectives:** To identify a therapeutic range for adalimumab trough levels in the treatment of childhood CNU.

**Methods:** A retrospective, observational, cohort study of 36 children with CNU aged < 18 years, treated with adalimumab. Serum adalimumab through levels and anti-adalimumab antibodies (ADA) were analysed at least 24 weeks after start adalimumab.

**Results:** Adalimumab trough levels were significantly higher in complete responders (11.9 μg/mL [range 6.9-33.0]) compared to partial or non-responders (9.4 μg/mL [range 0-13.6]) (p = 0.003). Receiver–operator characteristics analyses with an area under the curve of 0.767 (95% CI, 0.588-0.945) defined 9.65 μg/L as the lower margin for the therapeutic range. This cut-off corresponds with a sensitivity of 88% and a specificity of 56% (positive predictive value, 85%; negative predictive value, 70%). A concentration effect curve defined 13 μg/mL as the upper margin. Approximately one-third (30.5 %) of patients had an adalimumab trough concentration exceeding 13 μg/mL. ADA were observed in only 2 patients (5.5%).

**Conclusion:** A therapeutic range of adalimumab trough levels of 9.65 μg/L to 13 μg/mL, which corresponds with an optimal clinical effect, was identified. Therapeutic drug monitoring may guide the optimisation of treatment efficacy in children with CNU in the treat-to-target era.

**Patient Consent**

Not applicable (there are no patient data)

**Disclosure of Interest**

None declared

**References**

## P375 Chronic non-bacterial osteomyelitis and uveitis

### S. Ertem^1^, F. Aydın^1^, N. Çakar^1^, F. N. Yalçındağ^2^, Z. B. Özçakar^1^

#### ^1^Pediatric Rheumatology; ^2^Opthalmology, Ankara University, Ankara, Türkiye

##### **Correspondence:** S. Ertem

*Pediatric Rheumatology 2023*, **21(Suppl 2):**P375

**Introduction:** Chronic non-bacterial osteomyelitis (CNO) is an autoinflammatory bone disease that appears from aberrant functioning of the innate immune system.

Uveitis is an inflammatory disease that deteriorates vision and childhood uveitis is usually idiopathic. Uveitis can also be associated with juvenile idiopathic arthritis (JIA), inflammatory bowel disease, sarcoidosis, Behçet's disease, autoinflammatory diseases, and infection. Association of CNO and uveitis is a rarely encountered.

**Objectives:** The aim of this study was to investigate the relationship of uveitis in pediatric patients with CNO.

**Methods:** The files of CNO patients followed in the Pediatric Rheumatology outpatient clinic between June 2016-June 2022 were reviewed retrospectively. Demographical and clinical characteristics, laboratory results and treatment modalities were recorded from the medical database of hospital, retrospectively. Patients with previous uveitis exam were included in the study; patients without ophthalmologic exams were referred for uveitis examinations.

**Results:** Twenty-one patients (71.4%, male) diagnosed with CNO were enrolled in the study. Median age at CNO onset and CNO diagnosis were 9.9 (3-13) and 10.9 (6-14) years respectively. The most common complaint was bone pain (66.7%), and the most common physical examination finding was tenderness (81%). Multifocal lesions were detected in 19 patients, vertebral involvement in 10 and sacroiliitis in 12 patients. The median clinical CNO score calculated by the Jansson criteria was 43.4. Uveitis was detected in 3 male CNO patients. Two patients developed uveitis before the diagnosis of CNO. Age at diagnosis of uveitis ranged between 5-12.5 years. All 3 patients had bilateral intermediate uveitis and received corticosteroids, methotrexate, adalimumab.

**Conclusion:** Uveitis can be seen in patients with CNO. Further studies are needed in order to show whether uveitis is associated with CNO or if it is just an incidental finding.

**Patient Consent**

Not applicable (there are no patient data)

**Disclosure of Interest**

None declared

## P376 Comparison of juvenile idiopathic arthritis-associated uveitis with idiopathic uveitis

### V. Guliyeva^1^, F. G. Demirkan^1^, N. aktay ayaz^1^, M. oray^2^

#### ^1^Department of Pediatric Rheumatology; ^2^Department of Ophthalmology, Istanbul School of Medicine, Istanbul University, istanbul, Türkiye

##### **Correspondence:** V. Guliyeva

*Pediatric Rheumatology 2023*, **21(Suppl 2):**P376

**Introduction:** Childhood uveitis is a rare condition and it is estimated that only 5-10% of all uveitis patients belong to the pediatric age group. Pediatric uveitis can be infectious or non-infectious. The most common causes of NUI are those associated with idiopathic and juvenile idiopathic arthritis (JIA).

**Objectives:** The aim of this study is to compare the demographic characteristics, follow-up and treatment protocols, management and prognosis of uveitis due to juvenile idiopathic arthritis, which is followed up jointly by the pediatric rheumatology clinic with the eye department with the diagnosis of NUI.

**Methods:** In this cross-sectional study, the uveitis type, characteristics, laboratory findings, treatment options and follow-up results of the patients followed up with the diagnosis of NUI in the Pediatric Rheumatology Clinic of Istanbul Medical Faculty between December 2012 and March 2023 were evaluated retrospectively from their medical records.

**Results:** Twenty-eight (50.9%) of 55 patients included in the study were juvenile idiopathic arthritis-associated uveitis. Mean age at presentation of patients in JİA group was 141,5 ±46 month, in İU group was 160,8 ±40 month

.Among the JIA subgroups, uveitis was most common in those with 67.8% oligoarticular JIA (73.6% persistent, 26.4% extended), 25% RF negative polyarticular and 7.2% enthesitis-associated arthritis. The median time to diagnosis of JIA following the development of uveitis was 12 months (IQR: 39); The time to develop uveitis after the diagnosis of JIA was 35 months (IQR: 36). In 73.3% of the patients, uveitis was diagnosed after JIA, at the same time in 16.7%, and after uveitis in 10%.

Compared to the patients in the JIA group, idiopathic uveitis developed at a later age. Acute onset in the JIA group and a recurrent course in the IU group were significant.

ANA positivity was found to be significantly more common in those with JIA diagnosis in laboratory findings (p=0.009). The median C-reactive protein value measured at the time of diagnosis was 3 mg/dL (IQR: 9.4), and the median sedimentation value was 12 mm/hour (IQR: 19).

Methotrexate treatment was received by 92.8% of JIA patients. 54.5% of the resistant cases received anti-TNF (Tumor necrosis factor) treatment. Ophthalmologic surgery was performed in 7.7% of the patients. At the last visit, 78.2% of the patients were in remission.

**Conclusion:** If uveitis is not treated, it can become chronic and cause damage to ocular structures. Therefore, ophthalmological evaluations of children with suspected rheumatological diseases should be done regularly. Effective communication between the rheumatologist and ophthalmologist is essential in the treatment of children with rheumatism.

**Patient Consent**

Yes, I received consent

**Disclosure of Interest**

None declared

**References**

1. E.S. Sen, A.D. Dick, A.V. Ramanan, Uveitis associated with juvenile idiopathic arthritis, Nat. Rev. Rheumatol. 11 (2015) 338–348.
2. E.S. Sen, A.V. Ramanan, Juvenile idiopathic arthritis-associated uveitis, Best Pract. Res. Clin. Rheumatol. 31 (2017) 517–534.
3. M.J. Hawkins, A.D. Dick, R.J. Lee, A.V. Ramanan, E. Carreno, C.M. Guly, A.H. Ross, Managing juvenile idiopathic arthritis-associated uveitis, Surv. Ophthalmol. 61 (2016) 197–210.

## P377 The pattern of uveitis in juvenile idiopathic arthritis forme fruste patients

### S. S. Hashad^1,2^, H. M. Etayari^1,2^, I. A. Almsillati ^1,2^, M. N. Tofil^1^

#### ^1^Rheumatology, Tripoli Children Hospital; ^2^Pediatrics, University of Tripoli, Tripoli, Libya

##### **Correspondence:** S. S. Hashad

*Pediatric Rheumatology 2023*, **21(Suppl 2):**P377

**Introduction:** Chronic uveitis may develop in a subset of children without evidence of joint or systemic involvement. They may represent a forme fruste of juvenile idiopathic arthritis and they should be monitored closely for the presence of occult inflammatory joint disease and development of arthritis or other systemic manifestations.

**Objectives:** To study the clinical and biochemical characteristics of patients with JIA froste forme who developed uveitis from 1/2002 to 9/2021.

**Methods:** Retrospective case series.

**Results:** Of the 75 patients following up because of chronic uveitis,6(8%) were identified as to have JIA forme fruste. 5(83.3%) females and 1(16.7%) males. Mean age at diagnosis 8.7±3.7years. Mean follow-up period 2.1±1.9 years.

In 3(50%) patients location arthritis was anterior, while the other 3(50%) had panuveitis. In 1(16.7%) patients uveitis was unilateral and in the other 5(83.3%) it was bilateral.

The presenting symptoms where decreased vision in 3(50%)patients, Red eye in 2(33.3%), eye pain in 1 (16.7%), and blurred vision in 1(16.7%).

4 patients presented with complications, synichae in 3(50%), cataract in 1(16.7%) and band Keratopathy in 1(16.7%)

Rheumatoid factor was negative in 100% of the patients. ANA was positive in all patients. ANA titer 1:160 in 5(83.3%), and1:640 in 1(12.5%). ANA pattern are speckled in 3(50%), Mixed in 2(33.3%), and neocleolar in 1(16.7%%). HLAB27 negative in 5(83.3%) and in 1(16.7%) it was not done. HLAB51 was negative in all the patients (100%).

Visual acuity was normal in 3 and decreased in 4 and it was hand movement in 1

Systemic steroids were used in 2(33.3%) of the patients before referral, non of the patients needed intraocular steroid injections, nor underwent operations before referral.

All the patients were treated by prednisolone and MTX, in one patient methyleprednisolone pulse was used, and in the same patient the use of biology (infliximab) was necessary.

In the last visit uveitis was inactive in 3(50%) of the patientswhile in 2(33.3%) patients the disease was still active. One patient was lost to follow-up. No patient developed new complication, synechiae, at last visit. No patient needed an intraocular injection or operations after referral.

**Conclusion:** Uveitis in JIA froste forme was not associated with high rate of complications and it was controllable by MTX and the use of biologics was necessary only in one

**Patient Consent**

Yes, I received consent

**Disclosure of Interest**

None declared

## P378 Effectiveness and safety of infliximab in the treatment of refractory uveitis in tripoli children hospital (2018-2020)

### S. S. Hashad^1,2^, H. M. Etayari^1,2^, I. A. Almisllati^1^, M. N. Tofil ^1,1^ on behalf of Soad S. Hashad*^1.2^, Hala M. Etayari ^1.2^, Iman A Almsellati^1^ Majda N. Etfil

#### ^1^Rheumatology, Tripoli Children Hospital, ^2^Pediatrics , University of Tripoli, Tripoli, Libya

##### **Correspondence:** S. S. Hashad

*Pediatric Rheumatology 2023*, **21(Suppl 2):**P378

**Introduction:** Refractory non-infectious uveitis is a sight threatening condition and finding safe and effective treatment is essential. (1)

**Objectives:** To assess the response and adverse events associated with infliximab treatment for refractory, noninfectious pediatric uveitis

**Methods:** A retrospective case series

**Results:** Nine of the 73 uveitis patients (12.3%) received infliximab (4females, 5 males), Mean age at onset of uveitis 9±5.4y. mean time between diagnosis and referral from ophthalmology 0.7±0.6Y. Diagnoses included idiopathic uveitis (n =3), juvenile idiopathic arthritis (JIA) associated uveitis (n =3), JIA frosteforme (n=1) HLAB27 assiciated uveitis (n=1), (Orbital pseudotumor n=1). Location of uveitis (pan n=5, anterior n=2 posterior n=1, intermediate=1). All patients had bilateral eye involvement. Mean duration of disease before starting infliximab 2.5±1.7y, Mean duration from starting MTX to infliximab 0.35±6.24y.

Remacade and rimsima were used. Three patients received remacade only, Three received remsima only and 3 received both. Mean duration of treatment 0.8±0.7y. In patients who had anterior uveitis the inflammation resolved in 6 and in one the inflammation improved. In the patients who had intermediate uveitis the inflammation resolved completely and in the patientswith posterior uveitis the inflammation resolved in 5 completely and improved with residual activity in 1. In one patient the treatment was discontinued after the second dose because of infusion reaction. After the 4th dose, in 4 eyes the inflammation was inactive, and after 5-9 doses the inflammation was inactive in 10 eyes.

In all the 7 patients visual acuety improved. In the patient who had low IOP before treatment and in one patient who had high IOP the pressure.

One patient had transient elevation of IOP during treatment and one patient developed high IOP after stopping the treatment. Anterior uveitis reactivated in 4 patients after stopping infliximab, intermediate uveitis in one and posterior uveitis in 4. Steroid requirements decreased from >1mg/kg to <0.5mg/kg in all the patients. Adverse reactions seen were 1 sever infusion reaction and 2 recurrent mild infections. No cases of TB activation, cytopenias or abnormal LFTs. One pateint developed new complication.

**Conclusion:** Infliximab was effective agent for the treatment of refractory pediatric uveitis without apparent serious toxicity.

Shift between biologics and biosimlar was smooth and the response was maintained.

**Patient Consent**

Yes, I received consent

**Disclosure of Interest**

None declared

## P379 Predictors of uveitis in juvenile idiopathic arthritis tripoli children hospital

### S. S. Hashad^1,2^, H. M. Etayari^1,2,3^, I. A. Almsellati^2,3^

#### ^1^Rheumatology, Tripoli Children Hospital; ^2^Pediatrics department , University of Tripoli; ^3^Tripoli Children Hospital, Tripoli, Libya

##### **Correspondence:** S. S. Hashad

*Pediatric Rheumatology 2023*, **21(Suppl 2):**P379

**Introduction:** Uveitis is a common extra-articular manifestation in Juvenile idiopathic arthritis. It is most commonly asymptomatic and needs screening to be detected. It can lead to very severe complications including loss of vision if it was not detected early and treated properly. So studying factors which can predict development of uveitis can identify patients who should have more frequent screening to prevent the morbidity associated with uveitis. (1)

**Objectives:** Here we studied the already identified risk factors and see if they can predict uveitis in our cohort of patients.

**Methods:** This is a retrospective case series from 2002 to 2021.

**Results:** A total of 271 jia patients were included, 113 (41.7%) of them were males and 158 (58.3%) of them were females. Mean age at diagnosis 8.3±3.9 years. The mean follow-up period 4.8±5 years. 15(5.5%) patients developed uveitis, 5(33.3%), and 10(66.7%) females. In 4(26.7%) patients uveitis developed before arthritis. The mean period between JIA diagnosis and development of uveitis 18.4±21 months.

Of those who developed uveitis. 3 seronegative polyarticular JIA (4.2% of all seronegative polyarticular JIA), 1 with ERA, 8 with oligoarticular JIA (13.8% of all oligoarticular patients), 3 with psJIA (17.6% of all psoriatic JIA).

Mean age at diagnosis of uveitis 5.7±2.9. 8(53.3%) of uveitispatients diagnosed at an age below 6 years, and 7 (46.7%) above 6 years (p-value 0.04).

Of those who had ESR above 20 6.9% developed uveitis, and of those who had ESR ≤20 3.9% developed uveitis.

Of all ANA positive patients, 14.7% developed uveitis and of all ANA negative patients 4% developed uveitis (pValue=0.001).

Total Number of patients who were treated by methotrexate 129, of them 8 (6.2%) developed uveitis, and the number of patients who did not use MTX 125, of them 7 (5.6%) developed uveitis.

**Conclusion**

- In this study no relation was found between sex and the development of uveitis.
- ANA positivity, age below 4 years were found to be related to the development of uveitis.
- The percentage of those who developed uveitis while using MTX and those who develop uveitis and MTX was not used us similar.

**Patient Consent**

Yes, I received consent

**Disclosure of Interest**

None declared

## P380 Characteristics of articular status in patients with juvenile idiopathic arthritis depending on the presence of uveitis

### A. A. Iakovlev, E. V. Gajdar, K. E. Belozerov, M. A. Kaneva, A. V. Kononov, L. S. Sorokina, E. A. Isupova, I. A. Chikova, V. V. Masalova, M. F. Dubko, T. N. Nikitina, O. V. Kalashnikova, V. G. Chasnyk, M. M. Kostik

#### SPbGPMU, SPb, Russian Federation

##### **Correspondence:** A. A. Iakovlev

*Pediatric Rheumatology 2023*, **21(Suppl 2):**P380

**Introduction:** It is known that uveitis influence on the course of the disease, but it is not clearly understood how intraocular inflammation influence on articular status in patients.

**Objectives:** to study the features of the course of the disease in patients with JIA, depending on the presence of uveitis.

**Methods:** A single-center retrospective study included 520 patients with uveitis. The analysis was carried out among patients who developed (n=116) and did not develop (n=404) uveitis. The minimum follow-up period was 2 years, for patients who did not develop uveitis.

**Results:** Uveitis was diagnosed in 116/520 (22.3%) children with JIA. Most often, uveitis occurred in patients with oligoarthritis and psoriatic arthritis. Our study showed that children with uveitis had an earlier age of onset of the disease. When comparing the features of the articular status, a lower frequency of involvement of the cervical spine, temporomandibular joint, shoulder, elbow, wrist joints, proximal interphalangeal joints, distal interfalangeal joints, hip joint, talus-heel joint, as well as a smaller number of active joints in children with uveitis was established. Also it was found that uveitis more often developed in children who had arthritis of the knee(86/74.1%), ankle(41/35.4%), joints of the hands and feet. Patients with uveitis received methotrexate(111/97.4% vs 293/77.5%, p = 0,000001) therapy more often, cumulative doses of corticosteroids (1000mg vs 3000mg, p = 0.051) were lower, the frequency of administration of genetically engineered biologic drugs was approximately the same in both groups. Remission of arthritis was achieved more often(86/74.1% vs 255/63.1%, p = 0.028), but the proportion of children who developed an exacerbation was higher(36/31.0% vs 60/14.9%, p = 0.00008). When calculating uveitis risk factors by binary logistic regression, it was found that the main predictors of uveitis were oligoarthritis, the number of active joints < 8, antinuclear antibody seropositivity and recurrent course of arthritis. The difference in the frequency of achieving remission may be due to more aggressive systemic therapy in the presence of uveitis.

**Conclusion:** The relationship of the occurrence of uveitis with the lesion of certain joints is shown. The prognostic role of uveitis in the severity of the course of the articular process in children with JIA has been established. The more frequent development of joint syndrome relapses in the group of patients with uveitis indicates the role of uveitis as a marker of a more severe course of JIA, which may require more active systemic therapy.

**Patient Consent**

Not applicable (there are no patient data)

**Disclosure of Interest**

None declared

## P381 Long term outcome of a juvenile idiopathic arthritis related uveitis (JIA-U) cohort: a monocentric experience

### C. Iannone^1,2^, E. Miserocchi^3^, S. Costi^4^, M. Cornalba^1^, M. Gattinara^4^, C. B. Chighizola^4,5^, R. Caporali^1,5^, A. Marino^4^

#### ^1^Division of Clinical Rheumatology, ASST-Gaetano Pini CTO; ^2^Department of Clinical Sciences and Community Health, Research Center for Adult and Pediatric Rheumatic Diseases, University of Milan; ^3^Ophthalmology, San Raffaele Hospital; ^4^Pediatric Rheumatology, ASST-Gaetano Pini CTO; ^5^University of Milan, Milan, Italy

##### **Correspondence:** C. Iannone

*Pediatric Rheumatology 2023*, **21(Suppl 2):**P381

**Introduction:** Uveitis (U) is a common extraarticular manifestation of Juvenile Idiopathic Arthritis (JIA), affecting up to 30% of patients, particularly those with oligoarticular JIA.

**Objectives:** This study aims to describe the course and management of U-JIA in a monocentric tertiary center and to investigate whether current baseline treatments can impact on ocular complications and drug escalation in the real-life settings.

**Methods:** A retrospective monocentric cohort analysis of U-JIA patients with a follow up of at least 6 months was carried out. Demographics and clinical characteristics were analyzed by standard descriptive statistical analysis. Clinical variables and drug treatments were correlated by Spearman’s rank non-parametric analysis.

**Results:** We included 60 patients affected by U-JIA with a mean follow up of 12.54 yrs (±9.09). Of these, n=43 (71.7%) were females, n= 41 (72%) were affected by oligoarticular JIA and 92.5% (n= 49/53) were ANA positive. Other diagnosis included polyarticular JIA (6.7%). The articular disease duration was 17.4 ±9 years, and the eye disease duration was 15.0± 9 yrs, while the lag between joint and eye manifestations was 7.9±31.5 months. Of these patients 93% (n= 52/56) had an anterior uveitis. Nearly half of them (45%) had a bilateral eye involvement and 89% a non-granulomatous uveitis. 6 patients had a granulomatous uveitis. 65% of patients (n=34/53) developed a uveitis sequelae defined as the presence of band-shaped keratopathy, synechiae, cataracts, glaucoma and/or macular edema, leading to a total of 21 eye surgical procedures. Interestingly, all patients with granulomatous phenotype developed an eye-related complication. MTX was administered in 91% of patients (48/53) and escalation therapy was needed in 64% of these patients. Interestingly, at the time of first uveitis diagnosis 11/49 (22.4%) were on MTX treatment, and 10/11 of them underwent treatment escalation. The correlation analysis showed MTX therapy at the time of first uveitis was not associated with development of eye complications (r=.09) and the need of a bDMARD (r=0.16).32 patients received bDMARDs with a mean of 2 bDMARD per patient, with 18 patients needing only 1bDMARD, while others requiring 1 ore more therapy switch. Monoclonal antibody against TNFa (TNFi) were prescribed as first line bDMARD in all patients, and adalimumab was the most frequently used (22/32 patients). Tocilizumab was 2^nd^ or further choice in 6 patients. Other utilized bDMARDs included anakinra (n=1), canakinumab (n=1) and abatacept (n=1). During last follow-up visit 52 patients were still receiving DMARDs, 30 of whom were on a bDMARDs. Only 1 patient developed legal blindness. The concomitant presence of ocular inflammation and active articular disease was associated with bDMARDs administration (r=0.47, p=0.01).

**Conclusion:** This long-term cohort documented the high morbidity of uveitis in JIA. Indeed, all patients needed a systemic treatment with more than half receiving one or more biological agent lines. Furthermore more than half of the patients develop an ocular complication.

**Patient Consent**

Not applicable (there are no patient data)

**Disclosure of Interest**

None declared

## P382 Pediatric uveitis continues to be a significant cause of ocular morbidity: our experience with 283 children from a tertiary care centre in North India

### A. K. Jindal^1^, P. Barman^1^, R. Aggarwal^1^, P. K. Patra^1^, S. Machhua^1^, S. Handa^2^, A. Arora^2^, M. Dogra^2^, D. Katoch^2^, R. Bansal^2^, R. Singh^2^, V. Gupta^2^, A. Agarwal^2^, P. Vignesh^1^, R. K. Pilania^1^, S. Sharma^1^, M. Dhaliwal^1^, D. Suri^1^, A. Rawat^1^, S. Singh^1^

#### ^1^Pediatrics; ^2^Ophthalmology, PGIMER, Chandigarh, Chandigarh, India

##### **Correspondence:** A. K. Jindal

*Pediatric Rheumatology 2023*, **21(Suppl 2):**P382

**Introduction:** Uveitis is a common cause of ophthalmological morbidity in children. The clinical profile, treatment protocols and long-term outcome varies depending on the etiology.

**Objectives:** To report the clinical manifestations, management and follow-up of children with uveitis from a single centre cohort of patients from North India.

**Methods:** A review of medical records of children with uveitis during the period January 1994 - April 2023 in Pediatric Rheumatology Clinic, Department of Pediatrics, Advanced Pediatrics Centre and the Retina Clinic, Department of Ophthalmology, Postgraduate Institute of Medical Education and Research, Chandigarh, India was done.

**Results:** Of 283 children with uveitis, the most common infective etiology was tubercular uveitis (n=25), followed by toxoplasmosis (n=1), toxocariasis (n=1) and staphylococcal sepsis (n=1). The non-infectious causes included juvenile idiopathic arthritis (JIA) (n=64), Blau syndrome (n=11), Behcet disease (n=6), Vogt-Koyanagi-Harada syndrome (n=4), sympathetic ophthalmitis (n=3) and miscellaneous causes (n=3). No identifiable cause was reported in 164 patients (idiopathic uveitis). Median age at diagnosis was 9 years (IQR: 6-12 years). Male: female ratio was 1.2:1. Bilateral involvement was noted in 201 patients. Most common type of uveitis was anterior (n=140), followed by panuveitis (n=73), isolated intermediate uveitis (n=41) and isolated posterior uveitis (n=29). Treatment protocols included topical corticosteroids and mydriatics in all patients and systemic corticosteroid in 220 patients [pulse methylprednisolone (n=20) and oral prednisolone (n=200)]. Other immunosuppressive drugs included methotrexate (n=195), azathioprine (n=56), mycophenolate mofetil (n=23), cyclosporin (n=7) and tacrolimus (n=3). Refractory disease was defined as no response to conventional disease modifying antirheumatic drugs. These patients were managed using adalimumab, infliximab, tocilizumab and tofacitinib (34, 6, 4 and 4 patients respectively). Of all patients, 113 (40%) developed eye complications. These complications included cataract (n=57), glaucoma (n=20), band shaped keratopathy (n=19), synechiae (n=18), corneal opacity (n=6), phthisis bulbi (n=4) and miscellaneous complications. The total duration of follow-up was 99 patient-years.

**Conclusion:** This is one of the largest single centre cohorts of paediatric uveitis in India. Idiopathic uveitis constitutes the majority of patients. Amongst the non-infectious causes, JIA is the most common while tubercular uveitis is the most common infective cause. Ophthalmological comorbidities were seen in more than 1/3^rd^ of all patients. This could be related to delays in diagnosis and possibly because biologic therapy was not frequently used for these patients.

**Patient Consent**

Not applicable (there are no patient data)

**Disclosure of Interest**

None declared

## P383 Tofacitinib is the new kid on the block for pediatric uveitis: our experience from Chandigarh, India

### A. K. Jindal^1^, P. Barman^1^, R. Aggarwal^1^, P. K. Patra^1^, S. Machhua^1^, S. Handa^2^, A. Arora^2^, M. Dogra^2^, D. Katoch^2^, R. Bansal^2^, R. Singh^2^, V. Gupta^2^, A. Agarwal^2^, P. Vignesh^1^, R. K. Pilania^1^, S. Sharma^1^, M. Dhaliwal^1^, D. Suri^1^, A. Rawat^1^, S. Singh^1^

#### ^1^Pediatrics; ^2^Ophthalmology, PGIMER, Chandigarh, Chandigarh, India

##### **Correspondence:** A. K. Jindal

*Pediatric Rheumatology 2023*, **21(Suppl 2):**P383

**Introduction:** Pediatric uveitis is a significant cause of ocular morbidity in children. A proportion of patients may experience a chronic refractory diseases course. Recently, Janus-kinase inhibitors (JAKi) such as tofacitinib have been reported useful in patients with refractory uveitis.

**Objectives:** To report the clinical profile and outcome of 4 patients with pediatric uveitis who had a refractory disease course and were managed using tofacitinib.

**Methods:** A review of medical records of children with uveitis during the period January 1994 - April 2023 in Pediatric Rheumatology Clinic, Department of Pediatrics, Advanced Pediatrics Centre and the Retina Clinic, Department of Ophthalmology, Postgraduate Institute of Medical Education and Research, Chandigarh, India was done. Clinical details and outcome of patients requiring tofacitinib were analysed in detail.

**Results:** Of the 283 patients with uveitis, 4 (2 boys) received tofacitinib. Two amongst these were genetically proven to have Blau syndrome. Indications for tofacitinib included refractory uveitis to non-biologic disease modifying anti-rheumatic drugs and failure of adalimumab (n=3), and economic reasons (n=1). Median age of onset of uveitis and median age of initiation of tofacitinib were 6 years (range: 3-14 years) and 15 years (range: 8-19 years) respectively. In all 4 patients the ocular inflammation improved following initiation of tofacitinib after a median duration of 4 months (range: 2-8 months). None of the patients reported any complications related to the use of tofacitinib.

**Conclusion:** Tofacitinib appears to be an effective therapy for children with refractory uveitis. Our preliminary results suggest that tofacitinib is safe in children with uveitis. However, we need more follow-up data on these patients to support these results.

**Patient Consent**

Not applicable (there are no patient data)

**Disclosure of Interest**

None declared

## P384 Superiority of Adalimumab in treating childhood chronic idiopathic uveitis: evidence from a multicentre experience

### I. Maccora^1,2^, C. Guly^3^, L. Sanfilippo^1^, S. Soldovieri^1^, C. de Libero^4^, R. Caputo^4^, A. V. Ramanan^5,6^, G. Simonini^1,2^

#### ^1^Rheumatology Unit, ERN ReConnet Center, Meyer Children’s Hospital IRCCS; ^2^NeuroFARBA Department, University of Florence, Florence, Italy; ^3^Ophthalmology Unit, Bristol Eye Hospital, Bristol, United Kingdom; ^4^Pediatric Ophthalmology Unit, Meyer Children’s Hospital IRCCS, Florence, Italy; ^5^Department of Paediatric Rheumatology, Bristol Royal Hospital for Children; ^6^Translational Health Sciences, University of Bristol, Bristol, United Kingdom

##### **Correspondence:** I. Maccora

*Pediatric Rheumatology 2023*, **21(Suppl 2):**P384

**Introduction:** Childhood Chronic Idiopathic Uveitis (cCIU) is a severe ocular condition that accounts for the 40% of all uveitis in children. Its timely and proper treatment is critical to prevent severe complications.

**Objectives:** To report the treatment response of a large cohort of cCIU.

**Methods:** This is a retrospective multicentre international observation study conducted at Meyer Children's Hospital IRCCS and Bristol Royal Hospital for children. The medical records of children were reviewed if they have a diagnosis of cCIU prior to 16 years old, and received a systemic treatment other than corticosteroids. We collected demographic, clinical and laboratory data. The main outcome was the achievement of response and remission on treatment according to SUN criteria.

**Results:** 146 cCIU (76 male, 42 ANA positive) with a median age at onset of 112months (range 20-199), 122 had a bilateral involvement (83.6%), 78 had anterior uveitis (53.4%), 17 anterior+intermediate (11.6%), 32 intermediate (21.9%) and 19 panuveitis (13%) were screened for inclusion criteria. 115 patients received at least one systemic therapy other than corticosteroids (78.8%), 68 two different lines (46.6%), 20 three-line treatment (13.7%), and 3 four-line treatment (2.1). The median time for the first drug administered was 6 months (range 0-73). As first line-treatment 93 children received methotrexate (63.7%), 21 adalimumab (14.4%) (of whom 4 with concomitant methotrexate), and 1 mycophenolate mofetil (MMF) (0.7%). As second line treatment 6 received methotrexate, 45 adalimumab, 15 MMF, 1 azathioprine, and 1 tacrolimus. Among the first-line therapies, we observed that patients treated with adalimumab achieved more frequently ocular response (19/21 vs 55/91 χ^2^ 8.72 p 0.01) and ocular remission (19/21 vs 42/45 χ^2^ 12.2 p <0.001), with no difference in frequency of relapse on therapy and time to first relapse on the therapy. Additionally, cCIU with ANA positivity, intermediate uveitis, and worse visual acuity (>0.4 LogMAR) were less likely to achieve disease remission with methotrexate as first-line treatment (χ^2^ 4.92, p0.026, χ^2^ 4.29 p 0.038 and χ^2^ 4.29 p 0.038 respectively). Among the second-line therapy, adalimumab achieved more frequently ocular remission (38/1 (ADA) vs 5/1 (MTX) vs 9/3 (AZA), χ^2^ 6.29 p 0.043).

**Conclusion:** Adalimumab showed better chance to achieve ocular remission, as first and second line treatment, as well as in patients with worst outcome: ANA positivity, worse visual acuity, intermediate uveitis.

**Acknowledgements**

All the patients that received adalimumab as first line treatment were followed at Meyer Children's Hospital IRCCS, and the off label prescription authorization was obtained.

**Patient Consent**

Not applicable (there are no patient data)

**Disclosure of Interest**

None declared

## P385 Outcomes of children with uveitis in Autosomal Dominant Neovascular Inflammatory Vitreoretinopathy (ADNIV)

### I. Maccora^1,2,3^, A. Sood^4^, G. Schulert^3,5^, M. Quilan-Water^3^, A. Duell^3^, J. L. Huggins^3^, T. Nguyen^4^, C. C. Sapp^4^, S. Sharma^4^, S. Sunil^4^, S. Angeles-Han^3^

#### ^1^Rheumatology Unit, Meyer Children's Hospital IRCCS; ^2^NeuroFARBA Department, University of Florence, Florence, Italy; ^3^Division of Rheumatology, Cincinnati Children's Hospital, Medical Centre; ^4^Cincinnati Eye Institute; ^5^Department of Pediatrics, University of Cincinnati College of Medicine, Cincinnati, United States

##### **Correspondence:** I. Maccora

*Pediatric Rheumatology 2023*, **21(Suppl 2):**P385

**Introduction:** Pediatric uveitis is commonly associated with rheumatic diseases and can lead to sight-threatening complications if not properly treated. Systemic immunomodulatory therapy has dramatically changed prognosis of corticosteroid refractory uveitis. Autosomal dominant neovascular inflammatory vitreoretinopathy (ADNIV) is a rare autoimmune condition caused by mutations in *CAPN5*, typically diagnosed in adults, and characterized by intermediate uveitis, retinal degeneration and neovascularization. In the early stages it is asymptomatic but inevitably leads to permanent blindness despite treatment.

**Objectives:** Our aim is to present the short-term outcomes of the first cohort of children diagnosed with ADNIV.

**Methods:** Cohort study of patients ≤18 years old at diagnosis with (+) *CAPN5* gene mutation (p.Leu244Pro), ocular findings consistent with ADNIV and a minimum follow-up of 6months (m). Treatment response was defined as a decrease in vitreous cells on clinical examination, retinal vascular leakage on ultra-widefield fluorescein angiography (UWFA), and/or macular edema on optical coherence tomography (OCT).

**Results:** 8 children (16 eyes, 5 females) with ADNIV met the inclusion criteria. The median age of ADNIV diagnosis was 14 (range 9-16) years. The median follow-up was 12 m (range [R] 6-13). Four children (50%) were asymptomatic and diagnosed by clinical examination/imaging. At diagnosis, visual acuity in the worse eye was 20/100 or better, none had anterior uveitis, while 7 had vitreous cells and 8 vascular leakage (UWFA), 2 neovascularization (UWFA), 3 macular oedema (OCT) and 1 cataract. Laboratory tests were all negative. Five of the 8 children were initially treated with oral (n=5) or local/injected corticosteroids (n=4), and anti-VEGF therapy (n=2). Due to persistent inflammation, systemic treatment was started in 7/8 patients. First line treatment was methotrexate (20 mg/weekly SQ, median duration 11 m, R6-12). Because of absent response, infliximab (10 mg/kg/dose every 4 weeks) was added in all patients after a median time from diagnosis of 3.2 m (R2.5-3.1). However, infliximab was ineffective in all patients, and 5/7switched to tocilizumab (10 mg/kg/every 2 weeks IV) after a median time from diagnosis of 9 m (R1-12) with a median duration of 3 m (R0-4).

**Conclusion:** We report on the largest series of children with ADNIV treated with systemic immunosuppression. Early testing for CAPN5 gene in at risk children, and regular scheduled screening for uveitis and vasculitis will lead to prompt intervention. Methotrexate and infliximab seem ineffective, and tocilizumab might be a promising treatment based on underling mechanism.

**Patient Consent**

Yes, I received consent

**Disclosure of Interest**

None declared

## P386 Safety and efficacy of abatacept for pediatric-onset idiopatic orbital inflammation diseases

### A. Meneghel, G. Martini, C. Camposampiero, M. Mazzarolo, N. Rocco, F. Zulian, M. E. Zannin

#### Pediatrics, University of Padova, Padova, Italy

##### **Correspondence:** A. Meneghel

*Pediatric Rheumatology 2023*, **21(Suppl 2):**P386

**Introduction:** Idiopathic Orbital Inflammation Diseases (IOIDs) are rare, severe and potentially sight-threatening conditions in children. At onset, IOIDs may include scleritis and orbital myositis as main clinical features and are characterized by a chronic-recurrent course. Step-up treatment strategy, including corticosteroids (CS), conventional Disease Modifying Drugs (cDMARDs) and biological agents, have been suggested

**Objectives:** To evaluate the efficacy of abatacept (ABA), a selective costimulation modulator, as second-line immunomodulatory agent in the treatment of IOIDs, rare but severe and potentially sight threatening conditions in children

**Methods:** A single centre-case series of consecutive pediatric patients with refractory IOIDs treated with ABA is reported. Clinical presentation at onset, disease course and treatment safety and efficacy have been evaluated

**Results:** Five consecutive patients (4 F, 1 M) entered the study: 3 with posterior scleritis and 2 with orbital myositis at onset. The mean age at disease onset was 11 years (range: 8-14). The mean follow-up was 6.2 years (range: 1-11). At onset IOID was unilateral in 4 and bilateral in 1 case. The main syntoms were orbital pain (5) and headache (3), with diplopia (2) and conjunctival hyperemia (4). All patients were initially treated with topical and i.v. corticosteroids (CS) and relapsed a mean of 3.2 months during CS discontinuation. They were treated with cDMARDs (2 mycophenolate mofetil, 3 methotrexate) but relapsed again after mean 2,5 months. ABA was introduced and a significant reduction of IOID relapses/year from mean 1.66 pre-treatment to 0.25 on-treatment was observed. During the follow-up all patients discontinued CS therapy, 4 achieved and maintained clinical remission on treatment and one full remission off-therapy. No significant adverse events were observed during ABA treatment

**Conclusion:** OIDs in children may have a severe course, often refractory to the conventional treatment. In our experience, ABA led to persistent disease remission, allowed CS discontinuation and was well tolerated. Further studies are needed to confirm these preliminary results

**Patient Consent**

Yes, I received consent

**Disclosure of Interest**

None declared

## P387 Characteristics of patients with juvenile idiopathic arthritis and uveitis in a recently created unit of pediatric rheumatology in a tertiary centre of Spain

### S. Murias^1^, I. Garcia-Moutas^2^, P. Rozas^3^, E. Pardo^1^, C. Costales^3^, J. Rodriguez^1^

#### ^1^Unit of Pediatric Rheumatology, University Hospital Central of Asturias, ^2^Faculty of Medicine, University of Oviedo, ^3^Pediatric Ophthalmology, University Hospital Central of Asturias, Oviedo, Spain

##### **Correspondence:** S. Murias

*Pediatric Rheumatology 2023*, **21(Suppl 2):**P387

**Introduction:** Juvenile Idiopathic Arthritis (JIA) is the most common rheumatic chronic disease in children, being uveitis its main extraarticular manifestation. The multidisciplinary approach of this entities improves clinical outcomes.

**Objectives:** To analyze frequency and characteristics of patients diagnosed with JIA and/or uveitis associated or not with JIA, in a cohort of patients followed in a recently created unit of Pediatric Rheumatology in a tertiary centre of Spain.

**Methods:** Retrospective, descriptive and observational study including pediatric patients diagnosed with JIA and/or uveitis (with/without JIA) followed at the formerly mentioned unit from January 2020 to March 2023. The comparative analysis of groups was performed using chi-square (c2) analysis for categorical variables, and Student's t-test or ANOVA for quantitative variables with normal distribution; or the corresponding non-parametric tests if this condition was not met. A value of p < 0.05 was considered statistically significant.

**Results:** 52 patients were included, 36 (69%) female, and being 13 diagnosed with any type of uveitis and 46 with JIA (27 oligoarthritis, 5 psoriasic, 7 polyarticular RF-, 3 enthesitis-related arthritis, 3 systemic onset JIA, 1 undifferentiated JIA). 5 patients were diagnosed with uveitis but not JIA. The median age at the last follow-up was 7 (range 2-18). The mean age of onset of JIA was 4.7 years, with a median of 2 (range 0.5-15). 24 (46.2%) of the patients had a family history of autoimmune disease. 25 (48%) patients out of the whole sample showed ANA+. Focusing on uveitis patients, 6 out of 13 were ANA+. At the last follow-up visit, 4 (7.7%) of the patients were on oral corticosteroids, 19 (36.5%) on MTX and 27 (52%) on biological drugs.

**Conclusion:** The characteristics of the patients with JIA and uveitis of any type included in this sample are consistent with those widely described across literature. Being a recently created unit, it can be concluded that multidisciplinary management of these patients, between the Pediatric Rheumatology and Ophthalmology units, is necessary for early diagnosis and optimal management

**Patient Consent**

Not applicable (there are no patient data)

**Disclosure of Interest**

None declared

## P388 Don't turn a blind eye to hearing loss: atypical Cogan's syndrome in children

### P. Nath^1^, P. Hande^2^, S. Srinivas^3^, K. Babu^4^, A. P. Rao^1,5^

#### ^1^Pediatric Rheumatology, Manipal Hospital; ^2^Department of Viteoretina, Narayana Nethralaya Eye Hospital; ^3^Department of ENT, Kauvery Hospital; ^4^Department of Uveitis and Ocular Inflammation, Vittala International Institute of Ophthalmology; ^5^Pediatric Rheumatology, Indira Gandhi Institute of child health, Bangalore, India

##### **Correspondence:** P. Nath

*Pediatric Rheumatology 2023*, **21(Suppl 2):**P388

**Introduction:** Cogan’s syndrome is a rare autoimmune vasculitis with ocular involvement and audio-vestibular symptoms. Depending on the symptoms displayed, Cogan's syndrome can be categorized as typical, featuring interstitial keratitis and vestibulo-auditory dysfunction, or atypical, which presents with severe inflammatory eye conditions like scleritis, uveitis, retinal vasculitis, retinal vein obstruction, and angle-closure glaucoma. As far as we know, there have been only a small number of reported cases of atypical Cogan's syndrome in the pediatric age group.

**Objectives:** We herein report 2 cases of atypical Cogan syndrome.

**Methods:** Retrospective data collection of the patients with Cogan syndrome over a 12-year period was done.

**Results:** Case 1: A male child aged 5 years old, initially presented with redness in the left eye which on evaluation was diagnosed as uveitis. The condition worsened gradually over the course of one year until both eyes were affected. After some months, the child began having trouble with balance while walking and, over the following year, he developed hearing loss, starting in one ear and gradually progressing to both the ears. A diagnosis of atypical Cogan syndrome with vestibulo-auditory involvement with uveitis was made and he was started off on steroids and methotrexate. He had relapses during treatment and continues to have profound hearing loss in a unilateral ear.

Case 2: 12 years old female child presented with painful redness of bilateral eyes associated photophobia. Ophthalmology examination revealed anterior uveitis. Child was started on Methotrexate with steroids. On reduction of the dose of steroids there was recurrence of anterior uveitis symptoms. Child also developed hearing loss which gradually progressed over the next 2 months. In view of intermittent uveitis and sensorineural deafness, diagnosis of atypical Cogan syndrome was made. Treatment was started with Adalimumab, methotrexate and Prednisolone. Child responded to treatment and is currently well controlled with preserved left ear hearing (hearing aid assisted). However, there is a profound hearing loss in the right ear.

**Conclusion:** These two cases highlight the fact that Cogan syndrome should be considered in any patient with uveitis with hearing loss and treatment started early when there is a window of opportunity as this might help prevent hearing loss.

**Patient Consent**

Yes, I received consent

**Disclosure of Interest**

None declared

**References**

1. Oh WH, Lee JH, Hwang JH. Atypical Cogan's Syndrome with Choroidal Effusion: A Case Report. Case Rep Ophthalmol. 2022 May 2;13(2):336-340. doi: 10.1159/000524540. PMID: 35702655; PMCID: PMC9149549.
2. Grasland A, Pouchot J, Hachulla E, Blétry O, Papo T, Vinceneux P; Study Group for Cogan's Syndrome. Typical and atypical Cogan's syndrome: 32 cases and review of the literature. Rheumatology (Oxford). 2004 Aug;43(8):1007-15. doi: 10.1093/rheumatology/keh228. Epub 2004 May 18. PMID: 15150435.

## P389 Frequency of uveitis as reported in the national registry of juvenile idiopathic arthritis in the Republic oF Moldova

### E. Nedealcova^1^, N. Revenco^1^, R. Eremciuc^1^, O. Gaidarji^1^, S. Foca^2^

#### ^1^Pediatric Department, State University of Medicine and Pharmacy "Nicolae Testemitanu"; ^2^Rheumatology, Institute of Mother and Childcare, Chisinau, Moldova, Republic of

##### **Correspondence:** R. Eremciuc

*Pediatric Rheumatology 2023*, **21(Suppl 2):**P389

**Introduction:** Juvenile idiopathic arthritis (JIA) is a prevalent rheumatic condition found in children, characterized by long-term joint inflammation. Among the various complications associated with JIA, uveitis stands out as the most common extra-articular manifestation. Uveitis, an inflammatory disorder affecting the uveal tract of the eye, can lead to substantial morbidity, including visual impairment and even vision loss. The frequency of uveitis among JIA patients exhibits regional variations, and understanding its occurrence in specific areas can be instrumental in comprehending the disease burden and implementing suitable preventive measures.

**Objectives:** This study aimed to assess the frequency of uveitis in patients with JIA registered in the National Registry of Rheumatic Diseases in Children from the Republic of Moldova.

**Methods:** A retrospective analysis of medical records was conducted for JIA patients aged ≤16 years who were admitted to a third-level hospital in the Republic of Moldova between January 2022 and March 2023. The presence of uveitis and its characteristics, including age at JIA onset and uveitis onset, gender distribution, JIA subtypes, antinuclear antibody (ANA) positivity, and ocular complications, were evaluated.

**Results:** Of the 119 patients reviewed, 19 (15.96%) presented with uveitis. The mean age at JIA onset was 6.29±4.29 years, while the average age at the onset of uveitis was 6.73±3.33 years. Among the uveitis cases, 15 (78.95%) were girls, and 4 (21.05%) were boys. Regarding JIA subtypes, 13 patients (68.42%) had persistent oligoarticular subtype, 4 (21.05%) had the negative rheumatoid factor polyarticular subtype, and 2 (10.53%) had other subtypes. Furthermore, 13 patients (68.42%) with JIA-associated uveitis tested positive for antinuclear antibodies (ANA). Ocular complications were observed in 16 (84.21%) children with uveitis.

**Conclusion:** In the Republic of Moldova, the frequency of uveitis among JIA patients was 15.96%, with a higher prevalence among girls. The persistent oligoarticular subtype was the most common JIA subtype associated with uveitis. Additionally, 68.42% of JIA patients with uveitis tested positive for antinuclear antibodies (ANA). The high rate of ocular complications (84.21%) emphasizes the importance of regular ocular screening and early detection to prevent visual impairment in this population. These findings contribute to a better understanding of the prevalence and characteristics of uveitis in JIA patients in the Republic of Moldova, providing valuable insights for clinical management and preventive measures. Further research is warranted to explore potential risk factors and optimize strategies for uveitis screening and management in this population.

**Patient Consent**

Yes, I received consent

**Disclosure of Interest**

None declared

**References**

1. Cosickic A, Halilbasic M, Selimovic A, Avdagic H. Uveitis Associated with Juvenile Idiopathic Arthritis, our Observations. *Med Arch*. 2017;71(1):52-55. doi:10.5455/medarh.2017.71.52-55
2. Sahin S, Acari C, Sonmez HE, et al. Frequency of juvenile idiopathic arthritis and associated uveitis in pediatric rheumatology clinics in Turkey: A retrospective study, JUPITER. *Pediatr Rheumatol Online J*. 2021;19(1):134. Published 2021 Aug 23. doi:10.1186/s12969-021-00613-2

## P390 Potential role of tocilizumab as the first-line biologic agent on juvenile idiopathic arthritis related uveitis

### N. Okamoto^1,2^, Y. Sugita^1^, Y. Ozeki^1^, K. Shabana^1^, N. Kawamura^2^, A. Ashida^1^

#### ^1^Department of Pediatrics, School of medicine, Osaka Medical and Pharmaceutical University, Takatsuki; ^2^Department of Pediatrics, Osaka Rosai Hospital, Sakai, Japan

##### **Correspondence:** N. Okamoto

*Pediatric Rheumatology 2023*, **21(Suppl 2):**P390

**Introduction:** We evaluated the effectiveness of tocilizumab as the first-line biologic agent on juvenile idiopathic arthritis (JIA) patients accompanied with uveitis.

**Objectives:** The effectiveness of tocilizumab (TOC) for JIA related uveitis (JIA-U) were reported in refractory cases against TNF inhibitors and TOC is proposed as one of third-line therapy in 2019 JIA uveitis guideline of ACR (recommendation 15). Intravenous (IV) TOC is the only biologic DMARD (bDMARD) that is approved for JIA under 4 years of age in Japan, therefore generally TOC-IV is administered for MTX-resistant or MTX-intolerant toddler patients with or without uveitis. The effectiveness of TOC as the first-line bDMARD on JIA-U is not established and we evaluated the clinical courses of those patients.

**Methods:** We conducted a chart-based retrospective study on JIA-U patients who were treated with TOC as an initial bDMARD and are currently attending at two regional hospitals with pediatric rheumatology units.

**Results:** 5 cases were detected and all cases are girls. Two cases are RF-negative polyarthritis and three case are persistent oligoarthritis. Three cases are ANA positive. Age of arthritis onset were 0.5, 1.75, 1.4, 3.0 and 4.9 years old, and age at diagnosis of uveitis were 1, 2.5, 11.1, 4.1 and 3.6 years old, respectively. Four cases were asymptomatic and diagnosed during a routine visit for ophthalmologists. Uveitis preceded arthritis in one case and uveitis was not active at the onset of arthritis. In all cases, TOC were administered by reason of MTX-resistant arthritis. In one case who achieved clinical remission for 2.5 years and stopped TOC, uveitis alone flared 1 month later. Uveitis became inactive with restart of TOC again. Rest four cases maintain clinical remission over a period of 0.5 to 5 years. In all cases, notable adverse event was not confirmed.

**Conclusion:** It is suspected that TOC has the potential effect of remission induction and flare suppression on JIA-U patients who are TNF inhibitor-naiive, and should be considered as the first-line bDMARD.

**Patient Consent**

Yes, I received consent

**Disclosure of Interest**

None declared

## P391 Tear proteome analyses of ANA-positive oligoarticular juvenile idiopathic arthritis patients with or without uveitis

### N. Sahin^1^, M. Sarıhan^2^, M. Kasap^2^, G. Akpınar^2^, B. Yılmaz Tuğan^3^, L. Karabaş^3^, Y. E. Bayrak^1^, H. E. Sönmez^1^

#### ^1^Pediatric Rheumatology; ^2^Medical Biology; ^3^Ophtalmology, Kocaeli University, Kocaeli, Türkiye

##### **Correspondence:** N. Sahin

*Pediatric Rheumatology 2023*, **21(Suppl 2):**P391

**Introduction:** Anti-nuclear antibody (ANA)-positivity is the best-known marker for uveitis risk in oligoarticular JIA (oJIA). But less than one-third of ANA-positive patients with oJIA develop uveitis.

**Objectives:** Biomarkers are needed to determine the risk of developing uveitis. For this purpose, we analyzed various proteins in tears by proteomic analysis.

**Methods:** Tear samples were collected using the Schirmer strips in 7 oJIA and 7 oJIA patients with uveitis (oJIA-U). All oJIA-U patients had developed bilateral anterior uveitis and were inactive and topical treatment-free. The nHPLC LC-MS/MS system was used for protein extraction and proteomics analysis. The PANTHER and STRING analyses were carried out using the UniProt accession numbers of the identified proteins.

**Results:** Patients with oJIA and oJIA-U were similar regarding age, gender, disease duration, and treatments. Twenty-two proteins were identified by the tear proteome database, 147 by the secretome database, and 258 by whole human proteome databases. Of these, 15 were identified by all three proteome databases. Ten proteins were up-regulated, and two were down-regulated, meeting the twofold regulation criteria. Upregulated proteins were cystatin-S, secretoglobin family 1D member, opiorphin prepropeptide, mammaglobin-B, lysozyme C, mesothelin, immunoglobulin kappa constant, extracellular glycoprotein lacritin, beta-2-microglobulin, immunoglobulin J chain. The highest upregulated protein was cystatin-S, with an 18-fold upregulation rate in tear samples from uveitis patients. The most highly downregulated proteins were dermcidin and prolactin-inducible-protein (Upregulation rate in tear samples: respectively, 0.01 and 0.49).

**Conclusion:** Although it is unclear whether changes in tear protein expression are directly related to pathogenesis or chronic inflammation, identifying tear biomarkers for uveitis may allow clinicians to understand pathogenesis or develop new molecular targets for treatments.

**Patient Consent**

Yes, I received consent

**Disclosure of Interest**

None declared

## P392 Efficacy and safety of biosimilars in pediatric non-infectious uveitis: real-life data from AIDA network registries

### M. Tarsia^1^, C. Gaggiano^2^, J. Sota^2^, A. Maselli^1^, C. Bellantonio^1^, S. Guerriero^3^, F. La Torre^4^, G. Ragab^5,6^, M. T. Hegazy^5,6^, A. Fonollosa Calduch^7^, M. P. Paroli^8^, E. Del Giudice^9^, M. C. Maggio^10^, M. Cattalini^11^, L. Fotis^12^, A. Marino^13^, A. Mauro^14^, A. Civino^15^, S. Grosso^1^, B. Frediani^2^, G. M. Tosi^16^, L. Cantarini^2^, C. Fabiani^16^ on behalf of AIDA Network

#### ^1^Clinical Paediatrics, Department of Molecular Medicine and Development, University of Siena; ^2^Research Center of Systemic Autoinflammatory Diseases and Behçet's Disease Clinic, ERN RITA Center, Rheumatology-Ophthalmology Collaborative Uveitis Center, Department of Medical Sciences, Surgery and Neurosciences, University of Siena, Policlinico "Le Scotte", Siena; ^3^Department of Ophthalmology and Otolaryngology, University of Bari, Bari; ^4^Department of Pediatrics, Giovanni XXIII Pediatric Hospital, University of Bari, Bari, Italy; ^5^Internal Medicine Department, Rheumatology and Clinical Immunology Unit, Faculty of Medicine, Cairo University; ^6^Faculty of Medicine, Newgiza University (NGU), Giza, Egypt; ^7^Department of Ophthalmology, Biocruces Bizkaia Health Research Institute, Cruces University Hospital, University of the Basque Country, Barakaldo, Spain; ^8^Uveitis Service, Ophthalmologic Unit, Department of Sense Organs; ^9^Pediatric Rheumatology Unit, Department of Maternal Infantile and Urological Sciences, Sapienza University of Rome, Rome; ^10^University Department PROMISE “G. D'Alessandro”, University of Palermo, Palermo; ^11^Pediatric Clinic, University of Brescia and Spedali Civili di Brescia, Brescia, Italy; ^12^Third Department of Paediatrics, National and Kapodistrian University of Athens, General University Hospital "Attikon" , Athens, Greece; ^13^Unit of Pediatric Rheumatology, Azienda Socio-Sanitaria Territoriale (ASST) Gaetano Pini-Centro Specialistico Ortopedico Traumatologico (CTO); ^14^Pediatric Rheumatology Unit, Department of Childhood and Developmental Medicine, Fatebenefratelli-Sacco Hospital, Milan; ^15^Pediatric Rheumatology and Immunology Unit, “Vito Fazzi” Hospital, Lecce; ^16^Department of Medicine, Surgery and Neurosciences, Ophthalmology Unit, ERN RITA Center, University of Siena, Siena, Italy

##### **Correspondence:** M. Tarsia

*Pediatric Rheumatology 2023*, **21(Suppl 2):**P392

**Introduction:** Biosimilar drugs (BIOs) are achieving greater visibility in the treatment of non-infectious uveitis (NIU), but there is still no comprehensive view in terms of efficacy and safety in pediatric age.

**Objectives:** This retro-prospective observational study was aimed at describing a multi-centre cohort of patients from the AutoInflammatory Disease Alliance (AIDA) International Registries with pediatric-onset uveitis treated with BIOs, examining their response to treatment and the overall drug retention rate (DRR); moreover, the safety profile was evaluated.

**Methods:** The cohort of patients was selected among 536 patients with NIU enrolled in the Uveitis AIDA Registry, with 1) onset of NIU at an age <18 years and 2) start treatment with BIOs in pediatric age. Patients with ocular involvement and receiving biosimilars at an age <18 years from the Behcet AIDA register were also included.

**Results:** Forty-seven patients (77 affected eyes) were enrolled (twenty-five female; mean age at uveitis onset 8,70 ± 3,65 years). Systemic associated disease was present in twenty-seven patients (57,4%), mostly represented by Juvenile Idiopathic Arthritis (17 patients). Regarding anatomical pattern, uveitis was classified as anterior, intermediate, posterior or panuveitis in 44, 13, 3 and 15 eyes, respectively; involvement was monolateral in 17 patient and bilateral at the onset or over the time in 30 patients; complications were retrospectively reported in 31 eyes. The BIOs employed were adalimumab (91,5% - biosimilar name molecules: ABP501 25,5%; GP2017 36,1%; SB5 25,5%), etanercept (4,3% - biosimilar name molecule: GP2015), infliximab (4,1% - biosimilar name molecules: GP1111 2,1%; SB2 2,1%). The number of relapses 12 months prior to biosimilar introduction and at last follow-up was 282,14 and 52,43 per 100 patients/year, respectively (*p* = 0.00431). Median visual acuity did not vary significantly between baseline and last follow-up visit (*p*=0.868). Median daily glucocorticoid (GC) dosage significantly decreased from 0,1 (IQR 0,675) mg/kg/day at the start of biosimilars to 0 mg/kg/day at the last assessment (*p* = 0.002). The estimated DRR at 12, 24, and 36 months of follow-up was: 92.7%, 83.3%, 70.8%, respectively. New ocular complications occurred in seven patients. Three adverse events (AE) were reported, consisting of mild severity.

**Conclusion:** Biosimilars proved an effective therapeutic role in NIU of this pediatric cohort. BIOs reduced uveitis relapses, allowing both relevant overall glucocorticoid-sparing effect and preservation of visual acuity. Safety profile is confirmed by the low number and severity of reported AE.

**Patient Consent**

Yes, I received consent

**Disclosure of Interest**

None declared

## P393 Anti-Adalimumab antibodies testing in paediatric non-infectious uveitis

### G. C. Varnier^1^, A. Chieng^1^, H. Razzouk^2^, J. Payne^2^, S. Pockar^2^, V. Sharma ^2^, J. Wong^2^, J. Ashworth^2^

#### ^1^Royal Manchester Children's Hospital; ^2^Manchester Royal Eye Hospital, Manchester, United Kingdom

##### **Correspondence:** G. C. Varnier

*Pediatric Rheumatology 2023*, **21(Suppl 2):**P393

**Introduction:** Adalimumab is a well established biologic immunosuppressive treatment for children with non-infectious uveitis. It is increasingly recognised that treatment failure can be caused by the development of anti-adalimumab antibodies (AAA) in Juvenile Idiopathic Arthrits (JIA) associated uveitis, but there is little evidence of their use in non-infectious uveitis.

**Objectives:** The aim of this study was to evaluate the use of AAA and adalimumab drug level in children with non-infectious uveitis in daily clinical practice.

**Methods:** Patients diagnosed with non-infectious uveitis and receiving treatment with adalimumab followed up in the paediatric and adolescent uveitis clinic at Manchester Royal Eye Hospital, United Kingdom, were included in this study. Demographic, clinical, treatment and laboratory data including AAA and adalimumab serum level were collected retrospectively.

**Results:** Of the 57 patients included in this study 61% were female, the most frequent diagnosis was JIA associated uveitis and chronic idiopathic uveitis, followed by granulomatous uveitis and Tubulointerstitial Nephritis and Uveitis Syndrome (TINU) representing 47.4%, 47.4%, 3.5% and 1.8% respectively. Adalimumab was given as monotherapy in 14% of patients, and in combination with methotrexate, mofetil mycophenolate, leflunomide or azathioprine in 73.7%, 7%, 3.5% and 1.8% of children respectively. 44 patients (77.2%) had no active uveitis. The remaining 13 patients (22.8%) were tested for AAA, 6/13 patients (46%) had positive AAA . The reasons for AAA testing were uveitis flare (13/13) and suspected poor treatment adherence (3/13). All patients with positive AAA had active uveitis, were almost all female (5/6) and had a shorter adalimumab treatment duration of 2.4 vs 5 years and had a significant lower median adalimumab serum level of 1.3 vs 9.7 mg/l (*p*<0.05).

Patients AAA positive and AAA negative showed no difference in terms of type of uveitis as 3/6 vs 3/7 had JIA associated uveitis; age with a median of 15.5 vs 16 years; inflammatory markers with median C reactive protein of 1 vs 1 mg/L and median erythrocyte sedimentation rate of 9 vs 5 mm/hr. The median AAA level was 200 AU/ml (IQR 132-200 AU/ml). 2/6 AAA positive patients were treated with adalimumab as monotherapy, 3/6 with adalimumab in combination with methotrexate, and 1/6 with adalimumab with mofetil mycophenolate.

**Conclusion:** Positive AAA may be a relevant factor when treatment failure occurs in paediatric non-infectious uveitis. Adalimumab serum level in combination with AAA may have a useful role to guide the clinical management of paediatric non-infectious uveitis.

**Patient Consent**

Yes, I received consent

**Disclosure of Interest**

None declared

## P394 A HLA-DRB1*11 restricted convergent T cell response underlies expansion of pathogenic peripheral t helper cells in the joints of european patients with antibiotic-refractory lyme arthritis

### J. Dirks^1^, J. Klaussner^1^, G. Haase^1^, U. Fischer^1^, A. Holl-Wieden^1^, C. Hofmann^1^, H. Girschick^2^, H. Morbach^1^

#### ^1^Department of Pediatrics, University Hospital Würzburg, Würzburg; ^2^Department of Pediatrics, Vivantes Klinikum, Berlin, Germany

##### **Correspondence:** H. Morbach

*Pediatric Rheumatology 2023*, **21(Suppl 2):**P394

**Introduction:** Antibiotic-refractory Lyme arthritis (ARLA) is thought to be mediated by an abnormal T cell response against a distinct *Borrelia burgdorferi* OspA peptide in patients with HLA-DRB1 risk alleles *01 or *04. This concept is based on the study of North American patients, but may not apply to the European context due to the different distribution of borrelia species.

**Objectives:** To dissect the cellular and molecular landscape of T helper cell responses in the inflamed joints of European patients with ARLA.

**Methods:** HLA-DRB1 genotyping. Flow cytometric analysis of synovial fluid CD4+ T cells from 11 children with ARLA. High-throughput sequencing of the T cell receptor Vβ (TCRVB) repertoire as well as single-cell RNA sequencing (scRNAseq) of sorted CD4+ T cell populations.

**Results:** Multidimensional flow-cytometric analysis revealed an oligoclonal expansion of peripheral T helper cells (T_PH_) in synovial fluid (SF) of pediatric ARLA patients. By in-depth bioinformatics analysis we identify a group of T cell receptors (TCRs) in this cell population shared between ARLA patients with a HLA-DRB1*11 background. The HLA-DRB1*11 allele frequency was 27.3% in the ARLA cohort compared to 12.5% in the common population. This TCR motive was specific for ARLA patients with a HLA-DRB1*11 background, persisted throughout disease course and was not associated to other autoimmune or infectious diseases. Furthermore, combined single cell transcriptome and paired TCRα/β sequencing linked this TCR motive to cells with an inflammatory transcriptional program driven by TCR signaling.

**Conclusion:** The shared TCR motive suggests a common antigen as trigger of the pathogenic T cell responses in the joints of the ARLA patients. The strong association to HLA-DRB1*11 – which is a protective allele in North American patients – points to a different pathogenesis of ARLA in European patients.

**Patient Consent**

Not applicable (there are no patient data)

**Disclosure of Interest**

None declared

## P395 Cytokine gene expression in two patients with coatomer subunit alpha gene variants (COPA) syndrome

### R. Rikhi, S. Basu, K. Arora, V. Joshi, A. Thangaraj, A. Rawat, S. Singh, D. Suri

#### Department of Pediatrics, Postgraduate Institute of Medical Education and Research, Chandigarh, India

##### **Correspondence:** R. Rikhi

*Pediatric Rheumatology 2023*, **21(Suppl 2):**P395

**Introduction:** Copa syndrome is autosomal dominant disease that is caused due to variants in the alpha subunit of the coatomer complex-I (COP-α). Increased Th17 cells and pro-inflammatory cytokines (IL-1β and IL-6) have been reported in patients with COPA syndrome. We estimated gene expressions of pro-inflammatory cytokines in 2 patients with variants in COPA gene.

**Objectives:** To study gene expression profile of inflammatory cytokines in whole blood samples of two patients with variants in COPA gene as well as cytokine expression levels pre and post treatment.

**Methods:** This prospective study was planned to investigate pro-inflammatory cytokine gene expressions in genetically proven patients with Copa syndrome. This cDNA converted from EDTA whole blood RNA, was utilized to analyze pro-inflammatory cytokine gene expressions carried out through SyBR Green chemistry on Real-time PCR. Serum cytokine levels (IL-2, IL4, IL6, IL10, TNF-α, and IFN-γ) were estimation in 1 patient prior to treatment of corticosteroids and JAK inhibitor using a Cytometric bead array. Patients: I: A 6-year-old boy resident of north India presented with fever, evanescent maculopapular rash, and polyarthritis (bilateral knee, bilateral wrist, left elbow, and right ankle). Examination revealed pallor, axillary and cervical lymphadenopathy, and hepatosplenomegaly. Laboratory investigations showed elevated thrombocytosis, CRP, ESR. Considering systemic juvenile idiopathic arthritis (SJIA), corticosteroids were initiated and response was noted. Subsequently, he was lost to follow-up. Two years later presented with nephrotic syndrome. Fat-pad biopsy and rectal biopsy showed deposition of amyloid. In view of amyloidosis, colchicine was started. The child however succumbed to illness. Underlying genetic defect was a novel missense variant (c.3100C>A; p.Leu1034Ile) in the COPA gene. II: A 4-year-boy born out of first-degree consanguineous marriage presented with history of recurrent pneumonia at 4 months of age. Subsequently, he had multiple chest infections and managed as case of chronic lung disease since 3 years of age. He was found to have interstitial lung disease (iLD), failure to thrive, clubbing, cyanosis and hypoxia. Genetic analysis revealed heterozygous splice-site mutation (c.841C>T, p. Arg281Trp) in exon 9 in the COPA gene.

**Results:** Elevated gene expression of TH17 cell-stimulating cytokines IL-1β, and IL-6 were elevated in these patients. IL-1β, IL6, TNF-α, and IFN-α were upregulated. Moreover, serum cytokine quantification in Case II patients revealed elevated levels of IL4, IL6, IL10, and TNF-α.

**Conclusion:** This study highlights the disturbed TH17 cell-associated immune dysregulation at molecular levels in patients with Copa syndrome.

**Patient Consent**

Yes, I received consent

**Disclosure of Interest**

None declared

## P396 Late diagnosis of primary immunodeficiency diseases occuring under the "mask" of juvenile chronic arthritis

### A. Firsa^1^, S. Salugina^1^, A. Kozlova^2^, I. Nikishina^1^

#### ^1^Children, V.A.Nasonova Research Institute of Rheumatology; ^2^Federal Research Center of Pediatric Hematology, Oncology and Immunology named after Dmitry Rogachev, Moscow, Russian Federation

##### **Correspondence:** S. Salugina

*Pediatric Rheumatology 2023*, **21(Suppl 2):**P396

**Introduction:** Primary immunodeficiency diseases (PID), genetically determined by inherited defects of the immune system, often occur under the "mask" of rheumatological diseases. Agammaglobulinemia is one of the variations of PIS, in addition to symptoms such as often recurrent and severe bacterial infections, arthritis might be detected, which may significantly complicate the verification of this disease

**Objectives:** to present a description of the clinical observation of the PIS immunodeficiency condition diagnosis of a teenage patient which occurs under the "mask" of juvenile arthritis (JA)

**Methods:** A patient born in 2005 (17 years old) was checked into the children's department of the Institute of Rheumatology with complaints of pain in the knee, ankle, wrist, shoulder joints, swelling of the second finger of the right hand, morning stiffness up to 1 hour. It is known from the anamnesis that from the age of 9 (2014) he was observed by hematologists for autoimmune thrombocytopenia, autoimmune hemolytic anemia, received therapy with cyclosporine, rituximab, glucocorticoids (GC) with gradual complete cancellation. At the age of 14 (2019), he suffered polysegmental pneumonia, focal pulmonary tuberculosis with an outcome in the form of dense foci and complete clinical cure due to preventive anti-tuberculosis therapy. At the age of 16, polyartthritis developed, morning stiffness and fever appeared. He received NSAIDs, sulfasalazine, methotrexate (MTX) in the treatment without significant effect and with the development of pancytopenia; at the same time, a persistent increase in the level of CRP was maintained. Re-prescribed GC per os at a dose of 1.5 mg / kg / day

**Results:** The examination confirmed the presence of pronounced polyarthritis, HLA-B27 "+", widespread papulo-pustular skin lesions. Blood tests showed an increase in the level of CRP to 37.3 mg/l (0-5) at normal values of ESR – 7 mm/h (2-20). Extremely low levels of immunoglobulins (Ig) IgG -0.1 g/l (7.0-16.0), IgA - 0.28 g/l (0.7-4.0), IgM – 0.18 g/l (0.4-2.3) and γ-globullin up to 1.00 (9.5-19.8) were detected, as well as a decrease in the absolute number of B cells. A hospital examination suggested the presence of agammaglobulinemia. According to the new generation sequencing (NGS), the presence of mutations was not detected. In therapy, a gradual reduction in the dose of GC was continued, MTX was canceled. Currently, the patient is being monitored in conjunction with immunologists, replacement therapy with intravenous immunoglobulin performed, in this connection an improvement in the general condition and complete relief of the joint syndrome is noted

**Conclusion:** The presented clinical observation demonstrates the importance of a comprehensive multidisciplinary approach. It is important not to narrow the differential diagnostic search to the limits of one specialty, but to look at the problem more broadly, involving in the range of suspected conditions pathologies of related sections of medicine. It is necessary to conduct a thorough analysis of the anamnesis and symptoms of the disease, to conduct a mandatory screening immunogram in all doubtful cases, since the correct diagnosis and the appointment of the correct targeted pathogenetically justified therapy depends on an in-depth examination and its competent interpretation

**Patient Consent**

Yes, I received consent

**Disclosure of Interest**

None declared

## P397 Multifocal septic arthritis causes by streptococcus group A in a pediatric patient: case report

### G. Tarantino^1^, M. Trevisan^1^, A. Ficari^2^, V. Messia^1^, R. Nicolai^1^, F. De Benedetti^1^

#### ^1^Rheumatology Division, IRCCS Ospedale Pediatrico Bambino Gesù; ^2^Pediatric Department, University of Rome Tor Vergata, IRCCS Ospedale Pediatrico Bambino Gesù, Rome, Italy

##### **Correspondence:** M. Trevisan

*Pediatric Rheumatology 2023*, **21(Suppl 2):**P397

**Introduction:** Septic arthritis is a severe infectious disease, more frequently affecting male children under 5 years old, with a mortality rate up to 10%. Invasive Group A Streptococcal (GAS) infections has recently shown increased worldwide incidence, with osteo-articular involvement in about 20% of cases. However, GAS septic arthritis is rarely reported.

**Objectives:** to describe clinical features of a GAS multifocal septic arthritis in a 5-year-old boy.

**Methods:** At the first evaluation in the emergency department (ED) patient had fever, cough and asthenia; physical examination revealed warm, painful and swollen left knee with persistent refusal to walk. No history of trauma or previous pharyngitis was reported. The blood exams showed neutrophilic leukocytosis, with increased CRP and ESR and mild hypertransaminasemia, while the musculoskeletal ultrasound (MUS) detected plentiful joint effusion and synovial thickening. Empiric intravenous antibiotic (ceftriaxone) was started without benefits; So, vital parameters worsened, fever persisted, homolateral ankle and midfoot swelled up. Early blood cultures isolated GAS, while throat swab was negative; the anti-streptolysin title (TAOSL) increased after 2 weeks. Therefore, the antibiotic therapy was switched to ampicillin (50 mg/kg/6 hours) and clindamycin (34 mg/kg/day). Normal ECG and echocardiogram excluded rheumatic fever diagnosis. Sequential magnetic resonance (MRI) confirmed unilateral oligo-arthritis without osteomyelitis. During recovery, the child condition quickly improved, the inflammatory markers gradually decreased and a 3-weeks-treatment was administered without complications.

**Results:** Septic arthritis is a rare clinical presentation of invasive GAS infection in the pediatric age. Fever with painful, swollen and limited joints are the most common symptoms: a monoarticular course is usual, although in 5-10% of cases more joints are simultaneously involved. Bacterial blood cultures should confirm diagnostic suspicion. A antibiotic combination of β-lactams and clindamycin is recommended as the best therapeutic strategy.

**Conclusion:** Our case, due to recent worldwide outbreak, would underline the importance to keep in mind this uncommon, but severe manifestation of misdiagnosed GAS infections. Prompt investigation and appropriate treatment in the presence of “red flags” ensure clinical remission with complete osteo-articular recovery and reduced long-term complications.

**Patient Consent**

Yes, I received consent

**Disclosure of Interest**

None declared

**References**

1. Boeddha N. P et al. EUCLIDS consortium et al “Group A streptococcal disease in paediatric inpatients: a European perspective. Eur J Pediatr 2023.
2. Wise J. et al. “Strep A: GPs are put on alert as cases and deaths rise. BMJ 2022.
3. Ekopimo O. et al. “Group A beta-hemolytic streptococcal osteomyelitis in children.” Pediatrics 2003.

## P398 Familial cases of CTLA4 haplodeficiency successfully treated with abatacept

### K. Yamazaki, K. Kawahata

#### Rheumatology and Allergology, St. Marianna University School of Medicine, Kawasaki, Japan

##### **Correspondence:** K. Yamazaki

*Pediatric Rheumatology 2023*, **21(Suppl 2):**P398

**Introduction:** CTLA4 haploinsufficiency is an immunodeficiency with a variety of manifestations due to reduced expression of CTLA4 protein caused by heteromutation in *CTLA4*. CTLA4 haploinsufficiency causes not only immunodeficiency but also autoimmune diseases and lymphoproliferation. We report three cases within a family successfully treated with Abatacept (ABT).

**Objectives:** To present three cases with CTLA4 haploinsufficiency within a family.

**Methods:** The cases were derived from a retrospective review of case notes and laboratory findings.

**Results:** Case 1 49-year-old male. At age 18, swollen lymph nodes were found. At age 19, he had idiopathic thrombocytopenia, and at age 32, he developed autoimmune hemolytic anemia. At age 41, he was diagnosed with CTLA4 haploinsufficiency for his daughters had the *CTLA4* mutation. They required bone marrow transplantion for lymphoproliferative disorders. At age 44, he developed granulomatous interstitial nephritis. At age 45, he started ABT because of nonspecific colitis with T-cell infiltration, arthralgia, and myalgia. After administration of ABT, pain, enteritis, renal function, and cytopenia improved.

Case 2 23-year-old male, son of Case 1. He had eczema since infancy. A recurrence of sinusitis was observed at age 9. Thrombocytopenia was observed at age 13, and hepatic dysfunction, hypogammaglobulinemia, and diarrhea at age 17. ABT was started at age 20, and eczema, diarrhea, thrombocytopenia, and hepatic dysfunction were completely resolved. Since his hypogammaglobulinemia persisted, subcutaneous gammaglobulin therapy was continued.

Case 3 22-year-old male, nephew of Case 1. He had eczema and hypogammaglobulinemia. After he started ABT at age 21, eczema markedly improved.

**Conclusion:** CTLA4 haploinsufficiency manifests a variety of conditions including immunodeficiency, autoimmune diseases and lymphoproliferation. Organ damage was caused by inflammation and the immune response due to T-cell infiltration. ABT should be used from the earliest stage of the disease to prevent organ damage.

**Patient Consent**

Yes, I received consent

**Disclosure of Interest**

None declared

## P399 The relation of femoral cartilage thickness with vitamin D and body composition in healthy adolescents: an ultrasonographic study

### P. O. Avar Aydin^1^, E. G. Koyuncu^2^

#### ^1^Pediatric Rheumatology Clinic; ^2^Physical Therapy and Rehabilitation Clinic, Tekirdag City Hospital, Tekirdag, Türkiye

##### **Correspondence:** P. O. Avar Aydin

*Pediatric Rheumatology 2023*, **21(Suppl 2):**P399

**Introduction:** Musculoskeletal ultrasonography (US) is a quick and accurate way to evaluate the thickness of the femoral cartilage (FCT). Since the knee joint is the most commonly affected joint in juvenile idiopathic arthritis, the erosion and cartilage loss are important signs of damage which can be detected earlier by US before radiographic changes. The normative data of femoral cartilage according to age, gender, and body composition is restricted in children.

**Objectives:** This study aims to provide a basic knowledge regarding the relation of FCT with vitamin D and body composition in healthy adolescents.

**Methods:** This prospective cross-sectional study involved healthy adolescents aged between 12-18 years. Adolescents with any chronic disease, knee trauma in the last 6 months, and professional sport activities were excluded. Demographic data and body measures (weight, height, waist circumference) were recorded. The medial, intercondylar, and lateral FCTs of bilateral knees were axially measured from the suprapatellar plane by 5-12 linear probes on bilateral knees while the knees were fully flexed (Figure 1). The mean of these 6 measures was referred as ‘mFCT’. Quadriceps and subcutaneous fat thicknesses (scFT) were axially evaluated on the mid-femoral area (Figure 2). Ultrasonographic evaluation was performed with at least three repeats by two physicians blinded to the laboratory tests.

**Results:** A total of 145 adolescents (101 F subjects) with a mean age of 14.9 years were included. 30.1% of the study population was found to have severe vitamin D deficiency while 59.4% had mild-moderate deficiency. Mean BMI was 61.4 and the frequency of obesity was 32.4%. mFCT was significantly lower in girls than boys (p<0.001) and normative values are provided in Table 1. It did not change according to age groups (12-15 vs. 16-18 years). A weak positive correlation was found between mFCT and quadriceps thickness (r:0.208, p:0.016). There wasn’t any correlation between mFCT and vitamin D, BMI, and scFT. The subjects with mFCT lower than mFCT of the study population had significantly lower vitamin D (p:0.036), weight SDS (p:0.034), and length SDS (p:0.036).

**Conclusion:** This study provides a basic knowledge about FCT according to vitamin D and body composition in adolescents. Boys have thicker FCT than girls. Age, BMI, and scFT do not have any relation with FCT in this age group. Despite nonsignificant correlation between FCT and vitamin D levels, subjects with thinner FCT have lower vitamin D levels.

**Patient Consent**

Yes, I received consent

**Disclosure of Interest**

None declared

**References**

1. Prakken B, et al. Lancet 2011;377:2138-49.
2. Herrera GA, et al. Rev Esp Cir Ortop Traumatol 2020;64:244-250.
3. Pamukoff, et al. Ort Res 2020;38:2685-2695.
4. Malas FU, et al. Clin Rheumatol 2014;33:1331-1334.

## P400 Effects of corticosteroids on central nervous system microvascular properties assessed by neuroimaging

### M. Difrancesco^1^, E. Ogbu^2^, C. E. Robben^2^, J. Huggins^2^, H. I. Brunner^2^

#### ^1^Radiology; ^2^Rheumatology, Cincinnati Children's, Cincinnati, United States

##### **Correspondence:** H. I. Brunner

*Pediatric Rheumatology 2023*, **21(Suppl 2):**P400

**Introduction:** Potential microvascular (MV) biomarkers for central nervous system (CNS) involvement in systemic lupus erythematosus (SLE) may be influenced by use of corticosteroids (CS).

**Objectives:** Since CS are widely used for the treatment of SLE and could also influence MV changes, CS effects need to be distinguished from MV changes secondary to CNS disease. We aimed to quantify and isolate the dose-dependent effects of oral CS on brain MV properties as assessed by diffusion-weighted imaging (DWI).

**Methods:** Using convenience sampling, 11 patients with chronic inflammatory conditions were studied at two visits: (1) the time of high dose CS use (HD-CS; prednisone >20 mg/day) and (2) after tapering to low dose (LD-CS). At each visit, cognitive ability was measured using the Pediatric Neuropsychological Assessment Metrics (PedANAM) and brain MRI was acquired, including DWI at 19 weightings (b-values) ranging from 0 to 5000 s/mm^2^. DWI signal was analyzed as a biexponential function of b-value under the intravoxel incoherent motion (IVIM) model. Besides bulk tissue diffusion (D), the IVIM model provides parameters describing microvasculature including pseudodiffusion (D*), reflecting flow through randomly-oriented microvessels, the volume fraction of blood (vbw), and their product, D* x vbw, proportional to blood perfusion. Voxel-wise change in each IVIM parameter was calculated between the HD-CS and LD-CS visits for each patient. Parameter changes were fitted across patients to a linear model including 2 terms 1) the mean parameter change and 2) the degree of CS dose reduction between HD-CS and LD-CS. Results were expressed as effect size, represented by Cohen’s f^2^, for the coefficients of the mean and dose reduction terms, with f^2^ > 0.35 considered a large effect size.

**Results:** All patients completed the HD-CS visit and 9 the LD-CS visit. Regionally, there were mean changes in all 4 IVIM parameters between HD-CS and LD-CS with f^2^ > 0.35, including vbw decreases and D* increases in medial frontal regions. Overall, the vascular parameters were mostly greater at HD-CS, with increases in the blood perfusion-related product, D* x vbw, especially widespread. Similarly, the dependence of parameter changes between HD-CS and LD-CS on the amount of dose reduction had large effect size regionally (f^2^ > 0.35), including positive dependence for vbw and negative dependence for D* in bilateral insula. Broadly, there was mostly positive dependence of vascular parameters on degree of CS dose reduction.

**Conclusion:** This pilot study provides evidence of at least moderate impact of oral CS on CNS MV as measured by DWI. Also, CS impact on brain MV varies regionally across the brain. If confirmed in a larger cohort, the parameter estimates from this study can be used to isolate the brain MV effects secondary to inflammation from CNS lupus from those caused by CS treatment.

**Patient Consent**

Not applicable (there are no patient data)

**Disclosure of Interest**

None declared

## P401 Nailfold capillaroscopy: an alternative tool for evaluating microvascular involvement and foreseeing glycemic control in children with type 1 diabetes

### F. Çakmak^1^, E. Inan Balci^2^, M. Yildiz^2^, F. G. Demirkan^1^, A. Yetim Sahin^2^, S. Poyrazoglu^2^, F. Bas^2^, F. F. Darendeliler^2^, N. Aktay Ayaz^1^

#### ^1^Pediatric Rheumatology; ^2^Pediatric Endocrinology, Istanbul Faculty of Medicine, Istanbul, Türkiye

##### **Correspondence:** F. Çakmak

*Pediatric Rheumatology 2023*, **21(Suppl 2):**P401

**Introduction:** Type 1 diabetes (T1D) is characterized by chronic hyperglycemia and microvascular changes like diabetes-specific retinopathy, nephropathy, and neuropathy. The disease is a significant global health problem with an incidence of 15 per 100,000 people worldwide and a prevalence of 9.5%. The cells of the capillary endothelium of the retina, the mesangial cells in the glomerulus, and the Schwann cells of the peripheral nerves are more vulnerable to high glucose levels, thus physicians follow-up microvascular complications by fundus examination, urinary microalbumin analysis, and electromyography (EMG). Given that high glucose levels expose all cells in the body, the nailfold capillary endothelium may appear as a significant target of microvascular involvement, and nailfold videocapillaroscopy (NVC) examination may be appropriate to gather clues about capillary microarchitecture and endothelial damage.

**Objectives:** With this study, we aimed to evaluate the microvascular structure in T1D with NVC, observe capillaroscopic alterations, and reveal the relationship between capillaroscopic abnormalities with glycemic control.

**Methods:** In this cross-sectional observational study, demographic data, clinical findings, and laboratory characteristics of 40 patients with T1D were obtained from patient files. They underwent nailfold video capillaroscopy to evaluate microvascular structure and compare findings with 40 healthy peers. The parameters evaluated from images were as follows; capillary morphology, presence of capillaroscopic changes (capillary tortuosity, capillary transition, enlarged capillary, giant capillary, capillary fold, branched/bush capillary, neoangiogenesis, avascular spaces, and microhemorrhages) and capillary width, arterial width, venous width measurements, apical ring width, intercapillary distance, and capillary length.

**Results:** Children with T1D showed significantly more capillary cross (p<0.001), enlarged capillary (p<0.001), bushy capillary (p=0.03), bizarre capillary (p<0.001), microhemorrhage (p=0.007), and neoangiogenesis (p<0.001). In addition, capillary density (p=0.01) was significantly lower in the patient group while intercapillary distance (p<0.001) was significantly longer compared with healthy volunteers. Morphologically, patients with T1D had a higher frequency of major abnormality. Lower capillary density and wider capillary loops were associated with higher HbA1c values (r:-0.32, p=0.04; r:-0.35, p=0.02).

**Conclusion:** Capillaroscopy outshines as a non-invasive, reproducible, and reliable technique to evaluate the vascular microarchitecture in patients diagnosed with juvenile T1D. Children diagnosed with T1D present with several microvascular abnormalities prevalent on NVC examination. Hyperglycemia is the main cause of endothelial damage in T1D and we can report from the NVC window that poor glycemic control can be followed by capillaroscopic alterations. Therefore, it can be considered as a simple method for monitoring the degree of glycemia to achieve therapeutic control before microangiopathy progresses.

**Patient Consent**

Yes, I received consent

**Disclosure of Interest**

None declared

## P402 Sacroiliitis: an early sign of scurvy

### C. Macucci^1^, G. Martini^2^, A. Meneghel^3^, M. Balzarin^2^, T. Giani^1^

#### ^1^Department of Pediatrics, AOU Meyer, Florence; ^2^Department of Women's and Children's Health, University of Padua; ^3^Department of Women's and Children's Health, University of Padua, Pediatric Rheumatology Unit, Padua, Italy

##### **Correspondence:** T. Giani

*Pediatric Rheumatology 2023*, **21(Suppl 2):**P402

**Introduction:** Scurvy has been the scourge of sailors for thousands of years, while nowadays it has become an uncommon and often forgotten disease. However, it can still be observed, especially in conditions associated with a restrictive diet or an inadequate absorption. vitamin C deficiency compromises collagen synthesis and iron absorption that in turn are responsible for scurvy's many clinical signs.

**Objectives:** Aim of this study is to analyze sacroiliac involvement at MRI in patients with pediatric scurvy.

**Methods:** We conducted a retrospective study including patients admitted for musculoskeletal symptoms requiring a pelvic MRI who were then diagnosed with scurvy.

Demographic, clinical, laboratory and radiological data were extracted from electronic medical records.

**Results:** We identified 6 patients, all males, admitted between 2016-2022 with a median age at the disease onset of 45 months (range 17-133 months). All patients had at least one of the following symptoms: limping, pain at lower limbs and refusal to walk.

Seventyone percent of these children had a selective diet related to a concomitant disease (multiple food allergy, autism spectrum disorder, other developmental delays).

Blood exams revealed microcytic anemia in all patients and mild increase of C reactive protein and/or erythrocyte sedimentation rate in 86% of the cases. Children were initially diagnosed with osteomyelitis (71%), spondylodiscitis (28%), transient arthritis of the hip ( 28%), CRMO (28%), cancer ( 28%), JIA ( 14%) and muscle contracture (14%). MRI showed a sacroiliac joint involvement in all 6 patients, all with a bilateral and symmetric distribution, and a patchy enhancement, while previous radiographies in the same area were negative.

In all patients a severe deficiency of vitamin C was documented. The median diagnostic delay after symptom onset was 2 months (range 1-6 months).

**Conclusion:** Bilateral, symmetric sacroiliac involvement is an early sign of scurvy detectable at MRI. Although plain radiography is the traditional modality used for evaluating patients with bone pain suspected with scurvy, radiographic changes are often late.

As the pelvis is enriched with bone marrow, the abnormal findings observed at MRI are related to the gelatinous bone marrow transformation induced by vitamin C deficiency.

**Patient Consent**

Yes, I received consent

**Disclosure of Interest**

None declared

**References**

1. Deirawan H, Fakhoury JW, Zarka M, Bluth MH, Moossavi M. Revisiting the pathobiology of scurvy: a review of the literature in the context of a challenging case. Int J Dermatol. 2020 Dec;59(12):1450-1457. doi: 10.1111/ijd.14832. Epub 2020 Mar 10. PMID: 32154584.
2. Ganske A, Kolbe AB, Thomas K, Hull N. Pediatric scurvy MRI appearance. Radiol Case Rep. 2021 Mar 10;16(5):1148

## P403 Does ‘one-stop’ clinician ultrasound scanning streamline patient management in paediatric rheumatology clinics?

### K. Llewellyn^1^, A. Leahy^2^, K. Nott^2^

#### ^1^Faculty of Medicine, University of Southampton; ^2^Paediatric Rheumatology, University Hospital Southampton, Southampton, United Kingdom

##### **Correspondence:** K. Llewellyn

*Pediatric Rheumatology 2023*, **21(Suppl 2):**P403

**Introduction:** Ultrasound, with ongoing standardization by the OMERACT group, has emerged as an indispensable tool in paediatric rheumatology.^1,2^ Increasingly, UK paediatric rheumatologists are conducting these scans in-clinic. While survey data shed light on their views, the impact of in-clinic scanning on patient management is less explored.

**Objectives:** This study aims to evaluate the effects of in-clinic ultrasound scans performed by paediatric rheumatologists, focusing on improved communication and patient management. We seek to demonstrate how musculoskeletal ultrasound can expedite diagnosis, hasten treatment decisions, and reduce the demand for external scans.

**Methods:** Following the completion of an in-clinic ultrasound scan in a tertiary paediatric rheumatology service, the attending clinicians were asked to fill out a brief, anonymous survey. Data collection was conducted over a period of seven weeks, and the responses were subsequently encoded in IBM SPSS for statistical analysis.

**Results:** A total of 38 surveys were collected, with an average of 2.8 joints scanned per patient, the knee being the most frequently assessed. It was observed that 63% of the scans eliminated the need for a radiology request. Furthermore, half of the scans facilitated immediate treatment alterations, which otherwise would have been postponed pending a departmental scan. Importantly, 32% of scans influenced a treatment decision that the parent or patient would have initially disagreed with. Clinician confidence in patient management was boosted by 82% of the scans. Moreover, 97% of scans were found to enhance communication with parents and patients.

**Conclusion:** Our study reveals substantial benefits of in-clinic ultrasound scans in paediatric rheumatology, including increased clinician confidence and improved patient communication. This approach reduced radiology requests, expedited treatment, saved NHS resources, and minimized patient education disruptions. Additionally, these scans fostered patient understanding and acceptance by educating them about their condition and treatment. Enhanced clinician confidence from in-clinic scanning positively influenced patient care, especially regarding treatment adjustments.

**Patient Consent**

Not applicable (there are no patient data)

**Disclosure of Interest**

None declared

**References**

1. Collado P, Vojinovic J, Nieto JC, et al. Toward Standardized Musculoskeletal Ultrasound in Pediatric Rheumatology: Normal Age-Related Ultrasound Findings. *Arthritis Care Res (Hoboken)* 2016;68(3):348-56.
2. Rossi-Semerano L, Breton S, Semerano L, et al. Application of the OMERACT synovitis ultrasound scoring system in juvenile idiopathic arthritis: a multicenter reliability exercise. *Rheumatology* 2020;60(8):3579-87.

## P404 The experience of low-dose whole body computed tomography in Russian patients with fibrodysplasia ossificans progressiva

### V. Matkava^1^, I. Nikishina^1^, S. Arsenyeva^1^, E. Gasymov^2^, L. Blank^2^, A. Arefeva^1^, M. Kaleda^1^

#### ^1^Paediatric; ^2^Radiology, V.A. Nasonova Scientific Research Institute of Rheumatology, Moscow, Russian Federation

##### **Correspondence:** V. Matkava

*Pediatric Rheumatology 2023*, **21(Suppl 2):**P404

**Introduction:** One of the most disabilitating disease Fibrodysplasia progressiva ossificans (FOP) characterized by formation of «second skeleton» consisted of multiple heterotopic ossification (HO) due to mutation in the ACVR1 gene. It is extremely important to use the best informative and accessible visualization methods for monitoring of the progression. Ultrasound, X-ray, MRI and CT might be useful for different stages of the disease. It seems that low-dose whole body computed tomography (WBCT) is the most preferable option of observation the HO development.

**Objectives:** To evaluate the diagnostic significance of WBCT in 10 patients (pts) with FOP. To calculate HO volume and compare it with results of Cumulative Analogue Joint Involvement Scale (CAJIS).

**Methods:** WBCT was performed without contrast in a 128-slice CT scanner. HO volume was determined by segmentation of each axial slice using semi-automatic algorithms in AW Server 3.2 with manual contouring for optimization. HO volume was calculated separately for each body region and then summarized. The CAJIS was used for functional status assessment.

**Results:** From July 2022 to May 2023 the WBCT was performed in 10 pts (3 girls/7 boys) with a verified FOP. All pts have «typical» heterozygous missense substitution c.617G>A (p.Arg206His) in the ACVR1 gene and innate malformed great toes as a classic phenotypical feature. The mean age of pts was 11.9 (2;22) years. The mean radiation exposure of WBCT was 6.8 (3.14;12.46) mSv. The mean HO volume was 196.9 (49.7;359.45) cm3. There was a tendency to increase of HO volume according to the age of pts. All pts have multiple HO in soft tissues of the cervical, thoracic areas (100%), lumbosacral - 8 (80%), upper extremities - 3 (30%), lower extremities - 4 (40%). Multiple ankylosis of the facet joints, spinous process and vertebral bodies in cervical and thoracic spine were observed in all pts. WBCT revealed many unexpected HO which were not visualized by routine radiological assessment. Peripheral osteochondromas were detected in the humerus in 6 (60%) pts, femur in 9 (90%), tibia in 8 (80%), fibula in 1 (10%). Signs of bilateral sacroiliitis were found in 6 (60%) pts. According to CAJIS the structures of the axial skeleton especially in cervical and thoracic areas were more involved then distal parts of the trunk and extremities what comparable to «natural» course of FOP progression. The mean CAJIS score was 8.9 (4;22). Structures of lower extremities were less involved, what correlates with the patients independent: all pts move without support.

**Conclusion:** WBCT seems to be the best radiological method for FOP pts for quantitative and qualitative assessment the degree of HO progression. The fist low dose WBCT successful experience in Russia was in 2 y.o. child. We obtained data about common HO volume, determined previously undetected peripheral osteochondromas and confirmed sacroiliitis. Imaging modalities, low rate of radiation exposure, correlation with CAJIS demonstrate that WBCT should be considered as a gold standard for monitoring the FOP progression.

**Patient Consent**

Not applicable (there are no patient data)

**Disclosure of Interest**

None declared

## P405 Comparison of ultrasound-detected synovitis with clinical examination and outcome measures of disease activity in children with juvenile idiopathic arthritis: a cross-sectional study

### N. K. Sande^1,2^, V. Lilleby^1^, A.-B. Aga^1^, E. Kirkhus^3^, B. Flatø^1,2^, P. Bøyesen^1^

#### ^1^Rheumatology, Oslo University Hospital; ^2^Institute of Clinical Medicine, Faculty of Medicine, University of Oslo; ^3^Radiology, Oslo University Hospital, Oslo, Norway

##### **Correspondence:** N. K. Sande

*Pediatric Rheumatology 2023*, **21(Suppl 2):**P405

**Introduction:** Detection of arthritis is important for the diagnosis, treatment and follow up of patients with juvenile idiopathic arthritis (JIA). Ultrasound is a sensitive tool in the evaluation of arthritis and is increasingly used as a supplement to clinical examination in daily practice (1). However, few studies have examined whether ultrasound reflects disease activity in patients with JIA.

**Objectives:** To assess the validity of ultrasound joint examination in patients with JIA by comparing ultrasound-detected synovitis with clinical examination and outcome measures of disease activity.

**Methods:** In a cross-sectional study, 27 patients with active JIA underwent a standardised clinical assessment including 71-joint examination. Ultrasound examinations of 26 joints, including the elbow, wrist, metacarpophalangeal 2-3, proximal interphalangeal 2-3, hip, knee, talocrural, talonavicular, subtalar and metatarsophalangeal 2-3 joints were performed by one experienced rheumatologist, blinded to clinical findings. The ultrasound images were scored according to a joint-specific semiquantitative (grade 0-3) scoring system for B-mode (BM) synovitis and power Doppler (PD) activity with age-divided reference atlas (2). For the joints assessed by ultrasound, a sum score for BM and PD scores (range 0-78), and a total synovitis score (BM + PD scores, range 0-156) was calculated. Spearman’s correlation coefficients (r_s_) were calculated between the ultrasound sum scores and the number of active joints and the 71-joint Juvenile Arthritis Disease Activity Score (JADAS71).

**Results:** A total of 24 girls and 3 boys with a median age (IQR) 12 years (3-14 years) were included. The median (IQR) number of active joints, ESR and JADAS71 was 4 joints (2-6 joints), 17 mm/hour (7-59 mm/hour) and 13.4 (9.4-28.6), respectively. The median (IQR) ultrasound sum score for BM, PD and total synovitis was 7 (4-11), 1 (0-4) and 9 (4-13), respectively. Active joints correlated moderately with ultrasound BM scores (r_s_ = 0.58), PD scores (r_s_ 0.42) and total synovitis scores (r_s_ = 0.58). The correlation between JADAS71 and ultrasound sum scores was moderate for BM scores (r_s_ = 0.65) and PD scores (r_s_ = 0.56), and strong for total synovitis scores (r_s_ = 0.70). All correlations were statistically significant (p <0.05).

**Conclusion:** Ultrasound-detected synovitis correlated moderately to strongly with active joints and JADAS71. Our findings suggest that an ultrasound synovitis score can reflect disease activity in patients with JIA and may be a valuable outcome measure in clinical practice and research.

**Patient Consent**

Not applicable (there are no patient data)

**Disclosure of Interest**

N. Sande: None declared, V. Lilleby: None declared, A.-B. Aga Consultant with: AbbVie, Eli Lilly, Novartis, Pfizer, Speaker Bureau with: AbbVie, Eli Lilly, Novartis, Pfizer, E. Kirkhus: None declared, B. Flatø: None declared, P. Bøyesen: None declared

**References**

1. Colebatch-Bourn et al. Ann Rheum Dis. 2015;74(11):1946-57.
2. Sande NK et al. RMD Open. 2021;7(2):e001581.

## P406 Musculoskeletal ultrasound (MSUS) assessment of joint involvement in immunoglobulin a vasculitis (IGAV) - a single centre study

### M. Vidović^1^, I. Radoš^1^, K. Rubelj^1^, M. Harjaček^2^

#### ^1^Pediatrics, University Clinical Hospital Center Sestre Milosrdnice, Zagreb, Croatia; ^2^College of Medicine and Health Sciences, United Arab Emirates University, Al Ain, United Arab Emirates

##### **Correspondence:** M. Vidović

*Pediatric Rheumatology 2023*, **21(Suppl 2):**P406

**Introduction:** Immunoglobulin A vasculitis (IgAV) is the most common form of vasculitis in children and is generally self-limiting disease presented predominantly with non-thrombocytopenic purpuric rash followed by joint involvement in approximately 75% of patients. Severe complications like IgA nephritis, gastrointestinal bleeding, intussusception and orchiditis occur in 20-80% of patients. Joint involvement is described as arthralgia and/or arthritis, but the extent of synovial pathology is rarely evaluated.

**Objectives:** We present a cohort of 33 patients with IgAV treated in Clinical Hospital Centre Sestre Milosrdnice, Department of Pediatrics between January 2020 and December 2022, their clinical features, musculoskeletal ultrasound findings, complications and outcome. Arthritis was defined as arthralgia with either joint swelling, tenderness and/or limited range of motion (LOM), and arthralgia was defined as pain without previously mentioned clinical features.

**Methods:** A total of 33 patients including 17 males and 16 females treated in our centre were evaluated retrospectively. The diagnosis of IgAV was based on European League Against Rheumatism/Paediatric Rheumatology International Trials Organization/Paediatric Rheumatology European Society 2008 classification criteria, and all patients presented with typical purpuric rash. The patient age ranges from 1.2-15.5 years with average of 5.5 years.

**Results:** Arthralgia was present in 23 (65.71%) patients. Signs of joint swelling, tenderness and LOM was present in 20/23 (86.9%) patients with arthralgia. MSUS showed synovitis on B mode in 6/20(30%) patients with active joints, Power Doppler (PD) signal was positive in 2/6 (33.3%) patients with synovitis. In 13/20 (65%) MSUS was indicative of subcutaneous soft tissue oedema with or without PD signal. All patents with arthralgia received ibuprofen. Two patients with severe purpura and active synovitis were treated with prednisone. Relapse was observed in 6/33 (18.18%) patients, four of them had joint involvement during first episode and during relapse. None of the patients developed gastrointestinal complications and one patient was diagnosed with IgA nephropathy.

**Conclusion:** Joint involvement in IgAV is common, non-complicating feature causing pain and discomfort. In our cohort MSUS features were mostly soft tissue edema and typical synovitis findings were rare. In conclusion, in most of our cases arthralgia although a frequent in IgAV is more often result of subcutaneous changes due to vasculitis than intraarticular synovial inflammation. MSUS proved itself once again to be a simple, cost-effective and safe diagnostic and follow-up tool in pediatric rheumatology.

**Patient Consent**

Yes, I received consent

**Disclosure of Interest**

None declared

## P407 Transition in autoinflammatory diseases

### Z. Balik^1^, G. Ayan^2^, E. D. Batu^1^, U. Kaya Akca^3^, Y. Bayindir^1^, V. Cam^1^, M. Kasap Cuceoglu^1^, S. Sener^1^, E. Aliyev^1^, L. Kilic^2^, O. Basaran^1^, Y. Bilginer^1^, S. Apras Bilgen^2^, S. Ozen^1^

#### ^1^Division of Rheumatology, Department of Pediatrics; ^2^Division of Rheumatology, Department of Internal Medicine, Hacettepe University, Ankara; ^3^Division of Rheumatology, Department of Pediatrics, Aydin Gynecology and Pediatrics Hospital, Aydın, Türkiye

##### **Correspondence:** Z. Balik

*Pediatric Rheumatology 2023*, **21(Suppl 2):**P407

**Introduction:** With the development of diagnostic and treatment methods, more children with autoinflammatory diseases are transitioning to adulthood. During the transition period, patients may experience medication compliance issues that impact their follow-up and prognosis.

**Objectives:** This study aims to evaluate the transition processes to Adult Rheumatology and medication compliance of the patients who were followed in Pediatric Rheumatology with the diagnosis of autoinflammatory disease.

**Methods:** Medical records of the patients transferred from the Pediatric Rheumatology Department to the Adult Rheumatology Department at Hacettepe University between January 2015 and May 2023 were retrospectively reviewed. Patient demographics, diagnosis, age at symptom onset and diagnosis, and pediatric and adult examination characteristics were recorded. Afterward, a telephone survey was administered. In this questionnaire, patients were asked to rate the transition process by grading between 0 (worst) and 10 (best), then answer the Morisky-4 medication compliance questions before and after the transition to evaluate medication compliance.

**Results:** Between January 2015 and January 2023, 241 patients with autoinflammatory diseases were transferred to adult rheumatology. Of 241, 145 patients were included in the study who were examined at least once in the department of adult rheumatology and filled out the questionnaire. The most common diagnosis was familial Mediterranean fever (n=136;93.8%). The median age at onset of symptom was 4.0 () years (Table 1). The median age at diagnosis was 8.27 years. The median duration from the onset of symptoms to diagnosis was 2.55 years. Comorbidities were present in 49 (33.8%) of the patients. The most common comorbidity was juvenile idiopathic arthritis (n=15; 10.3%). The median duration between the last pediatric and the first adult clinic visits. was 5.71 months. Ten patients were examined only once in adult rheumatology. The most common reason for missing an appointment was pandemia (n=25, 17.2) followed by forgetting the appointment (n=21, 14.5%) Table 2). Patients rated the transition process with a median of 9 points. After the transition, colchicine was discontinued in 3 patients. In 142 patients, medication compliance did not change in 68.3% of them, decreased in 20.4%, and increased in 11.3%.

**Conclusion:** Although most of the patients were satisfied with the transition period, a decrease in medication compliance was observed in follow-up. A good understanding of the differences between the childhood and adulthood periods and close monitoring of medication adherence during and after the transition will enhance the transition and contribute to an improved prognosis in patients with autoinflammatory diseases.

**Patient Consent**

Yes, I received consent

**Disclosure of Interest**

None declared

**References**

1. Pratsidou-Gertsi P. Transition of pediatric patients with an Auto-inflammatory Disease: an alternative version of the Daedaulus and Icarus myth. Mediterr J Rheumatol. 2018;29(3):156-62.
2. Foster HE, Minden K, Clemente D, Leon L, McDonagh JE, Kamphuis S, et al. EULAR/PReS standards and recommendations for the transitional care of young people with juvenile-onset rheumatic diseases. Ann Rheum Dis. 2017;76(4):639-46.

## P408 Investigation of the factors associated with pain and fatigue after successful transition to adult care in patients with JIA and FMF: a pilot study

### S. Buran^1^, E. Aliyev^2^, Y. Bayındır^2^, M. O. Tüfekçi^3^, M. Ekici^4^, L. Kılıç^4^, N. B. Karaca^3^, Z. Balık^2^, Y. Bilginer^2^, E. Ünal^1^, S. Özen^2^, E. D. Batu ^2^

#### ^1^Department of Heart and Respiratory Physiotherapy and Rehabilitation, Hacettepe University Faculty of Physical Therapy and Rehabilitation; ^2^Department of Pediatrics, Division of Rheumatology, Hacettepe University Faculty of Medicine; ^3^Department of Basic Physiotherapy and Rehabilitation, Hacettepe University Institute of Health Sciences; ^4^Department of Internal Medicine, Division of Rheumatology, Hacettepe University Faculty of Medicine, Ankara, Türkiye

##### **Correspondence:** S. Buran

*Pediatric Rheumatology 2023*, **21(Suppl 2):**P408

**Introduction:** Pain and fatigue are important problems that limit the quality of life in juvenile idiopathic arthritis (JIA) and familial mediterranean fever (FMF), and the risk of disability continues even if children with this disease are transferred to adult care in clinical remission.

**Objectives:** The aim of our study was to define the pain and fatigue problems and to examine the associated factors in patients with JIA or FMF who have just been transferred to adult care.

**Methods:** Our study included 17 individuals followed up with the diagnosis of JIA (n=7) or FMF (n=10), who have just been transitioned from pediatric rheumatology to adult rheumatology at Hacettepe University Faculty of Medicine between June 2022 and April 2023. Participants' descriptive information (age, gender, body mass index, inflammatory biomarkers, disease activity scores, joint problems), transition success (Transition Readiness Assessment Questionnaire (TRAQ), pain and fatigue status (Visual Analogue Scale (VAS)), sedentary behavior (International Physical Activity Questionnaire (IPAQ)), quality of life (Short Form-36 (SF-36)), biopsychosocial status (Cognitive Exercise Therapy Approach-Biopsychosocial Questionnaire (BETY-BQ)) and mood (Hospital Anxiety and Depression Scale (HADS)) were recorded.

**Results:** There was no difference between the two groups in terms of demographic data such as age, gender and BMI (p>0.05). A high level (0.70-0.89) significant correlation was found between pain and disease duration, fatigue, sedentary behavior, quality of life, and mood in JIA patients (p<0.05*). A high (0.70-0.89) and a very high level (0.90-1.00) significant correlation was found between fatigue and sedentary behavior, biopsychosocial status, and quality of life in JIA patients (p<0.05*). While there was a moderate level (0.40-0.69) but significant correlation (p<0.05*) between fatigue and quality of life in individuals with FMF, no pain-related factor could be detected.

**Conclusion:** In this study, while the bidirectional relationship between pain and fatigue was remarkable in individuals with JIA, it was observed that pain was mostly affected by emotional factors and fatigue was affected mainly by physical factors. In individuals with FMF who did not report joint involvement, it was observed that the problem of fatigue was more common than the problem of pain, and was associated with low quality of life. It would be important to analyze factors associated with pain and fatigue in larger groups of patients with pediatric rheumatic diseases in order to enhance a successful transition process and improve the quality of life in these patients.

**Patient Consent**

Not applicable (there are no patient data)

**Disclosure of Interest**

None declared

**References**

1. Tarakçı E, Arman N, Barut K, Şahin S, Adroviç A, Kasapçopur Ö. Fatigue and sleep in children and adolescents with juvenile idiopathic arthritis:a cross-sectional study. Turk J Med Sci. 2019;49(1):58-65.
2. Duruoz MT, Unal C, Bingul DK, Ulutatar F. Fatigue in familial Mediterranean fever and its relations with other clinical parameters. Rheumatol Int. 2018;38(1):75-81.
3. Foster HE, Minden K, Clemente D, Leon L, McDonagh JE, Kamphuis S, Berggren K, van Pelt P, Wouters C, Waite-Jones J, Tattersall R, Wyllie R, Stones SR, Martini A, Constantin T, Schalm S, Fidanci B, Erer B, Demirkaya E, Ozen S, Carmona L. EULAR/PReS standards and recommendations for the transitional care of young people with juvenile-onset rheumatic diseases. Ann Rheum Dis. 2017; 76(4): 639–646.

## P409 Pattern of paediatric rheumatic diseases at the lagos state university teaching hospital, Lagos, Nigeria

### A. D. Faleye

#### Paediatrics, Lagos State University Teaching Hospital, Lagos, Nigeria, Lagos, Nigeria

##### **Correspondence:** A. D. Faleye

*Pediatric Rheumatology 2023*, **21(Suppl 2):**P409

**Introduction:** Paediatric rheumatic diseases (PRDs) have been believed to be rare in children of black race and hence poorly reported in our environment**.**

**Objectives:** To compare the spectrum of PRDs in the past and the present at the Lagos state university teaching hospital (LASUTH)

**Methods:** This is a retrospective review of patients with PRDs seen over a period of one-year (May 2018 to May 2019) as a paediatric rheumatology trainee with the adult rheumatology unit of LASUTH and over a period of 15 months (February 2022 to May 2023), which has been the time the paediatric rheumatology services kicked off officially in LASUTH.

**Results: Spectrum of PRDs in my first year as a paediatric rheumatology trainee in Lagos**

We had 25 children who were newly diagnosed with rheumatic diseases. F: M of 2.6:1 with mean age of 10.0 ± 3.5 years. Twelve (48%) had juvenile systemic lupus erythematosus (JSLE), 10(40%) had juvenile idiopathic arthritis (JIA), 2(8%) had morphea and 1 (4%) had Kawasaki disease. There were four mortalities (16%) which were cases of lupus nephritis and four patients (16%) defaulted from clinic due to unknown reasons.

**Spectrum of PRDs in the past fifteen months in LASUTH**

We had 72 patients (50 new and 22 follow up cases), 43 girls and 29 boys with F: M was 1.5:1 and mean age of 11.81 ± 4.5 years. Twenty-six (36.1%) JSLE, 12 (16.7%) JIA[1 oligoarticular, 6 polyarticular and 5 cases of systemic onset JIA]**,** 2(2.8%) juvenile dermatomyositis, 1(1.4%) JDM-Scleroderma overlap, 3(4.2%) polymyositis, 3(4.2%) polyarteritis nodosa , 3(4.2%) Kawasaki disease, 1(1.4%) systemic sclerosis, 1(1.4%) granulomatosis with polyangitis, 1(1.4%) primary Sjogrens syndrome, 1(1.4%) ankylosing spondylitis, 1(1.4%) undifferentiated connective tissue disorder, 11 (15.3%) chronic musculoskeletal pain, 1(1.4%) chronic recurrent multifocal osteomyelitis, 2 (2.8%) Fibrodysplasia ossificans progressiva, 1(1.4%) aquaporin 4 antibodies optic neuritis, 1(1.4%) multiple sclerosis and 1(1.4%) hereditary multiple endochondroma.

Twenty-one (29.2%) defaulted from clinic, 1(1.4%) transitioned to adult clinic, two (2.8%) were referred to other centers due to relocation, a case of lupus nephritis with end stage renal disease discharged against medical advice (DAMA) due to caregiver burnout and two died of complications of neurolupus within few days of admission.

**Conclusion:** The diagnosis of PRDs is increasing in LASUTH. This may be due to increasing awareness among doctors/other healthcare workers and availability of paediatric rheumatology services. Sociocultural/religious beliefs, poverty, ignorance and illiteracy still remains a big challenge in our environment as these accounted for the high rate of clinic default, DAMA and mortality in this report.

**Patient Consent**

Yes, I received consent

**Disclosure of Interest**

None declared

## P410 Assessing the risk of systemic disease in children and young people with mouth ulcers: a CPRD study

### N. Goss^1^, M. Beresford^1,2^, C. Pain^2^

#### ^1^Department of Women's and Children's Health, University of Liverpool, Institute of Life Course and Medicine; ^2^Department of Paediatric Rheumatology, Alder Hey Children’s NHS Foundation Trust, Liverpool, United Kingdom

##### **Correspondence:** N. Goss

*Pediatric Rheumatology 2023*, **21(Suppl 2):**P410

**Introduction:** Healthcare professionals have made the association for very many years that some children and young people (CYP) initially presenting with mouth ulcers may later go on to develop a systemic disease. However, it is also known that the majority of CYP with mouth ulcers will not go on to develop such a serious diagnosis, since systemic disease is very rare compared to the commonality of mouth ulcers.

**Objectives:** Assess the risk of systemic diseases such as Behcet’s disease, systemic lupus erythematous (SLE) and inflammatory bowel disease (IBD) in CYP that have a code for mouth ulcers in their primary care record**.**

**Methods:** A descriptive, observational cohort study using UK primary care records from the Clinical Practice Research Datalink (CPRD). The CPRD is a resource providing access to anonymised UK primary care records and other linked databases for the purposes of clinical and public health research.

Participants included were CYP aged less than 16 years at index date, that had records available to the CPRD from 1st January 1990 to 31st December 2019. The exposed population had a code for mouth ulcers, the unexposed population did not. The unexposed participants were frequency matched to exposed participants at index date by age and sex at a maximum ratio of three to one.

The three main outcomes were: a diagnosis of Behcet’s disease, SLE or IBD. The outcome code had to occur after the index date but could be at any age. Incidence rates and ratios were calculated to assess risk, including the association with covariates such as age, sex, ethnicity and socio-economic status.

Exposures and outcomes were defined by Read Code lists formulated by the research team and validated by clinicians.

**Results:** 520,034 CYP were included in the study: 130,105 were exposed participants and 389,929 were unexposed. There was a higher incidence of systemic disease (Behcet’s, SLE and IBD cases combined) in the exposed compared to the unexposed population: 30.4 (CI:27.2-33.8) vs 0.4 (CI:0.2-0.7) cases per 100,000 person-years. The trend was particularly evident in Behcet’s cases with an incidence rate ratio of 96 (CI:23.4-394), when comparing exposed and unexposed populations. The incidence rate ratios for SLE and IBD cases were 74.7 (10.1-555.3) and 73.3 (42-128) respectively.

Adjustment for sex, age and level of deprivation did not make a significant difference to the risk. The paucity of ethnicity data was not sufficient to be used in adjustment calculations.

**Conclusion:** CYP aged less than 16 years with mouth ulcers warranting input into their primary care record, are at a significantly increased risk of subsequent systemic disease diagnosis such as Behcet’s, SLE and IBD. The absolute risk of systemic disease in any CYP is still low: of 100,000 person years of follow up after mouth ulcers there were only approximately 30 cases of systemic disease. However it is important, in some cases, for clinicians to consider rare diagnoses in the context of a common presentation. Further research is required to validate the results of this study outside of the UK primary care context.

**Patient Consent**

Not applicable (there are no patient data)

**Disclosure of Interest**

None declared

## P411 ’Mind the gap’; Transitional care 2.0 in Radboudumc Amalia children’s hospital

### L. Gossens^1^, F. Glaap-Roeven^2^, J. Fuijkschot^3^, E. Schatorjé^1^, J. P. Rake^3^

#### ^1^Pediatric Rheumatology; ^2^Pediatric nephrology; ^3^Pediatrics, Radboudumc Amalia children's Hospital, Nijmegen, Netherlands

##### **Correspondence:** L. Gossens

*Pediatric Rheumatology 2023*, **21(Suppl 2):**P411

**Introduction:** Numerous studies confirm that growing up with a chronic disease, such as rheumatism, can make adolescents vulnerable in various aspects of life. The process of acquiring insight into the disease, developing independence and self-management skills, which are generally expected of adolescents within adult care, often takes longer for adolescents with chronic diseases [1]. Despite good intentions, there remains a significant gap between child and adult care. Professionals often have their own unique approach to transition care, making it inconsistent and vulnerable. Every adolescent has the right to be well-prepared for the transition to adult care [2]. Therefore, a hospital-wide transition protocol for all adolescents, including those with rheumatic diseases, is necessary.

**Objectives:** Reduce the gap between child and adult care and enhance the understanding of disease, independence, and self-management among adolescents with chronic diseases within the Radboudumc Amalia Children’s Hospital.

**Methods:** From June to August 2022, adolescents, parents and healthcare professionals from child and adult care were interviewed about their experiences and needs for transitional care and growing up with rheumatism or another chronic disease. Based on these findings, the Dutch quality standard for transition from child to adult care [2] and the EULAR/PReS transition recommendations [3], we developed and implemented a hospital-wide transition protocol.

**Results:** To facilitate the transition process for adolescents with a chronic disease, a hospital-wide transition protocol was developed. This protocol includes recommendations for different phases of transition and specific agreements, such as structured preparation for transition starting at age 12 using the ‘Ready Steady Go’ transition questionnaires [4], appointment of a transition coordinator, organization of a multidisciplinary meeting and assure a warm transfer with a joint consultation. Additionally, tools such as a transition webpage and a dedicated location for transitional notes in the electronic patient file were established. A transition desk is also available to provide general information and support for patients, parents, and professionals.

**Conclusion:** The first experiences on the hospital-wide transition protocol and tools have been positive. However, implementation has been challenging for some departments, requiring additional assistance. The current protocol does not fully meet the needs of adolescents with intellectual disabilities, indicating the need for further refinement.

**Patient Consent**

Not applicable (there are no patient data)

**Disclosure of Interest**

None declared

**References**

1. van Staa A, van der Stege HA, Jedeloo S, Moll HA, Hilberink, SR. Readiness to Transfer to Adult Care of Adolescents with Chronic Conditions: Exploration of Associated Factors. J Adolesc Health. 2011;48;(3):295-302
2. Federatie Medisch Specialisten (2022). Kwaliteitsstandaard - Jongeren in transitie van kindzorg naar volwassenzorg.
3. Foster HE, Minden K, Clemente D, Leon L, McDonagh JE, Kamphuis S, et al. EULAR/PReS standards and recommendations for the transitional care of young people with juvenile-onset rheumatic diseases. Ann Rheum Dis. 2017;76(4):639–46
4. Nagra A, McGinnity P, Davis N. & Salmon A. (2015). Implementing transition: Ready Steady Go. Arch Dis Child Educ Pract Ed;100(6):313-20.

## P412 Serum biomarkers for prediction of disease activity status in Juvenile Idiopathic Arthritis (JIA) – single center experience

### D. S. Lazarevic^1^, C. Kessel^2^, D. Föll^2^, J. Vojinovic^1^

#### ^1^University of Nis, Faculty of Medicine, Clinic of Pediatrics, University Clinical Center , Nis, Serbia; ^2^University Hospital Muenster, Depth of Pediatric Rheumatology and Immunology, Muenster, Germany

##### **Correspondence:** D. S. Lazarevic

*Pediatric Rheumatology 2023*, **21(Suppl 2):**P412

**Introduction:** New outcome measures are needed to optimize individualized tailored treatment and to predict response in patients with juvenile idiopathic arthritis (JIA).

**Objectives:** We have tried to identify potential predictive serum biomarkers for disease outcome in JIA patients and their correlation with disease activity status.

**Methods:** We have recruited 21 consecutive JIA patients in active disease status (calculated by JADAS10/27) prior therapy escalation according to the current recommendations (1,2). JIA patients with systemic onset were ruled out from our study. All patients were simultaneously evaluated for disease activity status (JADAS10 and 27) during the 12 months follow up period every 3 months at predefined study visits. At each visit blood samples were collected for evaluation of inflammatory biomarkers (IL17A, sIL2R, IL6, CXCL9, Tweak, MMP3, S100A8/A9 and S100A12).

**Results:** A total of 21 JIA patients (7 males and 14 females; median age 7.9 years; median disease duration 4.67 years). Thirteen (61.9%) of 21 JIA patients had persistent oligoarticular, while 8 (38.1%) had polyarticular disease course. Prior the project starts up 20 JIA patients (95.2%) were on concomitant cDMARDs (Methotrexate) during 33.71±29.03 months while only one patient (4.8%) was on NSAIDs after intraarticular steroid injection. At the baseline visit, due to active disease status (JADAS10/27), 20 (95.2%) of 21 observed JIA patients commenced bDMARDs, while only one of them started treatment escalation with cDMARDs (Methotrexate). During the follow up period 14 patients (66.6%) reached inactive disease status after 3- and 6-months visit, while at the end of study twelve patients (57.15%) were in inactive disease, 2 patients (9.52%) were still active and 7 of them (33.3 %) did not succeed to finalize study protocol. S100A12 had a strong and moderate correlation with JADAS10/27 at baseline (ρ=0.746, p<0.001), after 3-monts (ρ=0.506, p=0.019) and 6-monts (ρ=0.505, p=0.039), while S100A8/9 had moderate correlation with JADAS10/27 at baseline (ρ=0.479, p=0.028) and 3-monts visit (ρ=0.550, p=0.010). Linear regression analysis has showed that serum biomarkers had statistically significant association with disease activity status (JADAS10/27) after 3- and 6-months period: IL17A (p=0.048; p=0.026); IL6 (p=0.011; p<0.001); CXCL9 (p<0.001; p=0.023) and S100А8/9 (p<0.001; p=0.013). Statistically significant difference was found in CXCL9 (p=0.041) and S100A8/9 (p=0.007) levels between patients in active disease status versus patients in inactive disease status measured at 6 months visit.

**Conclusion:** Our data demonstrated that IL17A, IL6, CXCL9, S100A12 and S100A8/9 correlate with JIA disease activity status. Larger sample of patients are needed to confirm our findings.

**Patient Consent**

Yes, I received consent

**Disclosure of Interest**

None declared

**References**

1. Consolaro A, Giancane G, Schiappapietra B, Davì S, Calandra S, Lanni S et al. Clinical outcome measures in juvenile idiopathic arthritis. Pediatr Rheumatol Online J. 2016 Apr 18;14(1):23.
2. Ringold S, Angeles-Han ST, Beukelman T, Lovell D, Cuello CA, Becker ML, et al. 2019 American College of Rheumatology/Arthritis Foundation Guideline for the Treatment of Juvenile Idiopathic Arthritis: Therapeutic Approaches for Non-Systemic Polyarthritis, Sacroiliitis, and Enthesitis. Arthritis Care Res (Hoboken). 2019 Jun;71(6):717-734.

## P413 Transition readiness in adolescents with Juvenile Idiopathic Arthritis (JIA) and their parents – two center experience

### D. S. Lazarević^1,2^, J. Vojinović^1,2^, S. Đorđević^3^, D. Novaković^4^, M. Zečević^5^, G. Sušić^3^

#### ^1^Department of Pediatric Rheumatology, Clinic of Pediatrics, University Clinical Center Nis, Serbia; ^2^Faculty of Medicine, University of Nis, Serbia, Nis; ^3^University Children`s Hospital, Belgrade, Serbia; ^4^Institute of Rheumatology, Belgrade, Serbia, Belgrade; ^5^Department of Pediatric Surgery, Clinic for Pediatric Surgery and Orthopedics, University Clinical Center, Nis, Serbia

##### **Correspondence:** D. S. Lazarević

*Pediatric Rheumatology 2023*, **21(Suppl 2):**P413

**Introduction:** The transition from pediatric to adult care is a very challenging process. To enable optimal functioning of JIA patients in adulthood, it is necessary to provide continuous adapted care and treatment through appropriate transition programs.

**Objectives:** This study aimed to evaluate which JIA patient disease characteristics might lead to self-management skills improvement in the transition readiness process. We also wanted to explore the readiness of JIA patients and their families for the transition process into the adult health care system.

**Methods:** We have recruited different JIA patient subtypes and their parents from a two study centers. Demografic data, baseline characteristics, clinical manifestations and disease status were collected and Transition Readiness Assessment Questionnaire (TRAQ) was applied to all patients and their parents at one time point.

**Results:** A total of 91 JIA patients (27 males and 64 females; median age 15.32 years, range 1.5 to 18 years; median disease duration 5.51 years, range 0.42 to 16.6 years) and their parents were enrolled. Twenty five (27.5%) of 91 JIA patients had a concomitant disease while 17 (18.7%) of them had uveitis, 6 (6.6%) cataracta and 4 (4.4%) of them Hashimoto thyroiditis. Twenty four (26.4%) of them had a family history of autoimmune diseases. In total, 51 (56%) of JIA patients were receiving biologics. There was a statistically significant association between older patient age and total TRAQ score among patients ( p<0.0001) and it was also present between older patient age and total TRAQ score among parents (p<0.0001). Total TRAQ score has a strong correlation between patients and parents (ρ=0.676, p<0.0001). Our results have showed that treatment with biologics has statistically significant impact on higher total TRAQ score and transition readiness in the group of JIA patients (p=0.038) and parents (p=0.035). Achieved success in school has statistically significant influence on total TRAQ score in JIA patients (p=0.09) and their parents (p=0.03). In JIA patients group and their parents, total TRAQ score was significantly lower in JIA patients achieving good success in school when comparing with JIA patients with very good (p=0.024; p=0.002) and excellent success (p=0.010; p=0.012). We could not find any association of JIA patient characteristics (JIA disease subtypes, disease duration, gender, concomitant diseases, uveitis, family history of autoimmune diseases, diseases status, number of hospitalizations and parents education) with TRAQ scores and JIA patients' and parents' readiness for transition.

**Conclusion:** Transition readiness of JIA patients and their parents increase with advancing age, treatment with biologics and better school success. There is no difference between transition readiness for JIA patients and their parents. This findings are of importance in order to establish and facilitate this challenging transition process.

**Patient Consent**

Yes, I received consent

**Disclosure of Interest**

None declared

## P414 Uveitis in juvenile idiopathic arhritis and the importance of the transitional care

### R. Nicolau^1,2^, T. Beirão^3^, F. Guimarães^4^, F. Aguiar^2,5^, S. Ganhão^5^, M. Rodrigues^2,5^, I. Brito^2,5^

#### ^1^Rheumatology, Centro Hospitalar Tondela-Viseu, Viseu; ^2^Medicine, Faculty of Medicine, University of Porto, Porto; ^3^Rheumatology, Centro Hospitalar Vila Nova de Gaia/Espinho, Porto Portugal, Gaia; ^4^Pediatric, Centro Hospitalar Entre Douro e Vouga, Santa Maria da Feira; ^5^Pediatric and Young Adult Rheumatology Unit, Centro Hospitalar e Universitário São João, Porto, Portugal

##### **Correspondence:** R. Nicolau

*Pediatric Rheumatology 2023*, **21(Suppl 2):**P414

**Introduction:** Uveitis is a serious extra-articular manifestation of juvenile idiopathic arthritis (JIA) associated with a risk of blindness. Evidence suggests that uveitis may persist up to adulthood in some cases, potentially causing severe visual impairment. We aimed to highlight the importance of pediatric rheumatologists and transitional care in preventing blindness due to JIA-U.

**Objectives:** To evaluate the characteristics of ocular disease in patients with JIA-associated uveitis (JIA-U) who still exhibit uveitis in adulthood. Data on clinical features, treatment, complications and visual outcomes were collected.

**Methods:** We conducted a retrospective study on a series of patients aged 18 years or older with JIA-related active uveitis who had transitioned from pediatric to adult rheumatology in our Pediatric and Young Adult Rheumatology Unit. Data on clinical features, treatment, complications and visual outcomes were collected.

**Results:** 19 JIA-U patients were included (10 males, 9 females; median age 29 years, range 23–31 years). The onset of uveitis preceded the onset of arthritis in 7 (36.8%) patients and the median age of uveitis onset was 10 years (range 6.0–15.0 years).

Oligoarticular JIA was present in 11 (52.6%) patients, enthesitis related JIA in 8 (42.1%) and poliarticular JIA in 1 (5.3%). 11 (57.9%) patients had anti-nuclear antibody (ANA) positivity, all with a homogeneous or speckled-pattern subtype, and 7 (36.8%) had HLA-B27 positivity. 16 (84.2%) had at least one uveitis flare during the transitional care, mainly anterior (89.5%) and bilateral (68.4%) uveitis.

All patients were treated with topical glucocorticoid eye drops. Ten (52.6%) patients received methotrexate and 2 (10.5%) sulfasalazine. Monoclonal anti-TNF agents were administered in 11 (57.9%) cases while tocilizumab was used in 1 patient due to non-responsiveness to anti-TNF drugs. Two patients started Anti-TNF after transition. The most common ocular complications in our sample were cataract (68.4%), posterior synechiae (21.1%), band keratopathy (15.8%) and glaucoma (15.8%). Eleven (57.9%) patients underwent ophthalmic surgery for cataracts or glaucoma at an early age (≤ 18 years). One patient developed blindness after the transition period due to therapeutic noncompliance. 11 (57.9%) of patients were in remission for at least 2 years. An earlier onset of JIA-U was associated with the presence of ocular sequelae complications (*p* = 0.01).

**Conclusion:** The rate of complications of JIA-U has progressively declined over time due to extensive ophthalmological screening,highly effective treatments, and also due to an integrated multidisciplinary team which includes transition to adult care. This study highlights the importance of continuity of care for successful transitions, improving their health outcomes.

**Patient Consent**

Not applicable (there are no patient data)

**Disclosure of Interest**

None declared

## P415 The importance of the transitional care in pediatric Behçet’s syndrome: a retrospective series of 17 cases

### R. Nicolau^1,2^, T. Beirão^3^, F. Guimarães^4^, F. Aguiar^2,5^, S. Ganhão^5^, M. Rodrigues^2,5^, I. Brito^2,5^

#### ^1^Rheumatology, Centro Hospitalar Tondela-Viseu, Viseu; ^2^Medicine, Faculty of Medicine, University of Porto, Porto; ^3^Rheumatology, Centro Hospitalar Vila Nova de Gaia/Espinho, Gaia; ^4^Pediatric, Centro Hospitalar Entre Douro e Vouga, Santa Maria da Feira; ^5^Pediatric and Young Adult Rheumatology Unit, Centro Hospitalar e Universitário São João, Porto, Portugal

##### **Correspondence:** R. Nicolau

*Pediatric Rheumatology 2023*, **21(Suppl 2):**P415

**Introduction:** Behçets syndrome (BS) is characterized by relapses and remissions, and its management remains challenging due the wide clinical spectrum. Ocular involvement is one of the most common causes of morbidity, as well as neurological, vascular, and gastrointestinal involvement which may be life-threatening. Youth with rheumatic diseases often experience a gap in care or are lost to follow-up after transfer to adult rheumatology care. We aimed to highlight the importance of pediatric rheumatologists and transitional care in preventing comorbidities related to BS.

**Objectives:** To describe the transitional care experience in pediatric BS patients.

**Methods:** We conducted a retrospective study on a series of BS patients aged 18 years or older who hadtransitioned from pediatric to adult rheumatology and followed-up over a maximum 14-year period in our Pediatric and young adult Rheumatology Unit. Data on clinical features, treatment and complications were collected.

**Results:** 17 caucasian BS patients were included [11 females and 6 males; median age 26 years (IQR 24-34 years)]. Median age at symptom onset was 12 (IQR 8.5-14.5), while the median age at the diagnosis was 13 (IQR 12.5-17). HLA-B51 positivity was reported in 9 cases (52.9%).

All patients presented oral apthous ulcers, 15 (88.2%) genital ulcers, 8 (47.1%) pseudofolliculitis and 2 (11.8%) erythema nodosum. Concerning major organ-related manifestations, articular (5 patients, 29.4%) and ocular involvement (3 patients, 17.6%) were

the most common, while cardiac, gastrointestinal and neurological signs were present in only in 11.8%. 2 patients (11.8%) presented retinal vasculitis. Concerning the therapeutic approaches, colchicine was the most frequently prescribed medication (94.1%), followed by conventional disease modifying anti-rheumatic drugs (cDMARDs) (58.9%) - 40% methotrexate, 30% cyclosporine and 30% azathioprine. 2 patients were treated with Monoclonal Anti-TNF (Infliximab and Adalimumab). 3 patients received hypocoagulation (1 deep vein thrombosis, 1 venous CNS thrombosis, 1 intracardiac thrombosis). During the transitional care, 5 (29.4%) had at least one oral aphtous episode, 3 (17.6%) had recurrent arthralgias and 2 (11.8%) had pseudofollicutis episode. No ocular complications were reported. No patients were lost to follow-up.

**Conclusion:** Pediatric rheumatologists and transitional care play a major role in management of BS patients by improving the independence and skills of youth to meaningfully engage in their own care and ensure a good prognosis.

**Patient Consent**

Not applicable (there are no patient data)

**Disclosure of Interest**

None declared

## P416 Relapses of juvenile idiopathic arthritis in adulthood: a monocentric experience

### L. Scagnellato^1^, G. Cozzi^1^, M. Lorenzin^2^, G. Martini^3^, F. Zulian^4^, R. Ramonda^1^

#### ^1^Rheumatology, Department of Medicine, University of Padova; ^2^Rheumatology, Department of Medicine; ^3^Paediatric Rheumatology, Azienda Ospedaliera di Padova; ^4^Paediatric Rheumatology, University of Padova, Padova, Italy

##### **Correspondence:** L. Scagnellato

*Pediatric Rheumatology 2023*, **21(Suppl 2):**P416

**Introduction:** Classification and outcome of Juvenile Idiopathic Arthritis (JIA) is still debated in the paediatric era and the outcome of these patients in adulthood is even more obscure. A crucial moment in the definition of the JIA journey over time is the transition to the Adult Health Care Service, which happens in the very delicate moment of adolescence.

**Objectives:** The aim of the study is to describe a monocentric cohort of Young Adult JIA patients, analysing their risk of relapse after the transition to the adult Health Care Service.

**Methods:** Clinical, serological, and demographic data of young adult patients (< 30 years old) referring to the Transition clinic of a single Italian centre were retrospectively collected. Systemic onset JIA patients were excluded. Patients is persistent remission (NRe) according to Wallace criteria were compared to those who relapsed (Re). Cox proportional hazards models were applied to identify risk factors for relapse.

**Results:** Fifty patients with age 18-30 years old were enrolled in the study and followed for a median of 30 months. According to the 2004 ILAR Classification Criteria, 31 (62%) patients were affected by oligoarticular JIA, 14 (28%) by polyarticular JIA, 4 (8%) by Enthesitis Related Arthritis, and 2 (2%) by Psoriatic Arthritis. Thirty-seven patients (74%) were ANA positive, 1 (2%) was rheumatoid factor positive, 3 (6%) were anti-CCP antibodies positive. Sixteen (32%) patients presented chronic anterior uveitis and 3 (6%) were affected by inflammatory bowel disease. The median disease duration at transition was 15 years. Most patients (46, 92%) were treated with csDMARDs during their life, while only 20 (40%) were on treatment through adulthood. Thirty-eight patients (76%) were on bDMARD in adulthood. Twenty-three patients relapsed after the transition to the Adult Clinic after a median follow-up time of 9 months (IQR 0-46.5). Most relapses involved the knees (69.6%). The history of monoarthritis was more frequent in relapsing patients (Re 55.5% vs NRe 87.0%, p=0.0174) and their follow-up was longer (Re 48 months IQR 24-66 vs NRe median 30 months IQR 12-42 p=0.0109). Chronic NSAID use was more frequent in the relapsing group compared with the non-relapsing one (56.7% vs 91.3%, p=0.0057), while the use of systemic glucocorticoids, csDMARDs, and bDMARDs did not differ substantially. The multivariate analysis identified monoarthritis (HR 2.26, p value 0.0281) and rheumatoid factor positivity (HR 4.53, p-value 0.0022) as risk factors for relapse within the first 36 months of follow-up, while the exposure to at least one course of treatment with bDMARDs was protective (HR 0.323 p-value 0.028).

**Conclusion:** This analysis highlights that in JIA patients the risk of relapse after the transition to Adult healthcare is still high, irrespective of disease subtype, requiring the continuation of bDMARD treatment. The main risk factors for the early occurrence of articular activity are rheumatoid factor positivity and monoarticular involvement.

**Patient Consent**

Yes, I received consent

**Disclosure of Interest**

None declared

## P417 Multidisciplinary model for the care of patients with autoinflammatory diseases: a pilot experience in a reference center in Madrid

### C. Udaondo^1,2^, A. Remesal^1^, R. Alcobendas ^1^, M. Bravo Garcia-Morato^3^, C. Millan-Longo^1^, A. Robles^4^, M. Feito^5^, C. Camara^3^, I. Castillo^6^, L. Nuño Nuño^7^, C. Plasencia^7^, E. Lopez Granados^3^, A. Balsa^7^ on behalf of EMMA (Equipo Multidisciplinar de enfermedades autoinflamatorias pediátricas), Hospital La Paz, Madrid, Spain.

#### ^1^Paediatric Rheumatology Unit, Hospital Universitario La Paz; ^2^IDIPAZ; ^3^Immunology; ^4^Internal Medicine; ^5^Dermatology; ^6^Farmacology; ^7^Rheumatology, Hospital Universitario La Paz, Madrid, Spain

##### **Correspondence:** C. Udaondo

*Pediatric Rheumatology 2023*, **21(Suppl 2):**P417

**Introduction:** Autoinflammatory diseases are rare conditions that require a high level of expertise for their diagnosis and treatment. These diseases often require consultations with multiple specialists, which highlights the importance of coordination among different healthcare services and increases the number of hospital visits. This can pose a significant challenge for patients and their families.

**Objectives:** In this study, we aim to present a patient-centred model of care for the multidisciplinary review of autoinflammatory disease patients at a referral hospital in Madrid, Spain.

**Methods:** We outline the process of creating and organizing the multidisciplinary consultation, as well as provide details about the patients who have been reviewed since its establishment in september 2022.

**Results:** The hospital is highly specialized, and its paediatric rheumatology and immunology unit established a committee called EMMA (Multidisciplinary Autoinflammatory Team, in Spanish *Equipo Multidisciplinar de Autoinflamatorias*). The committee includes key personnel from paediatric rheumatology, adult rheumatology, internal medicine, immunology, immunogenetics, dermatology, gastroenterology, cardiology, and pharmacy. The teams are organized into working groups focusing on general coordination, quality of care and quality of life scales, databases, and clinical organization. The committee meets monthly, and during each session, an average of four patients are assessed in-person in a multidisciplinary manner. This shared space is also utilized for non-presential discussions regarding patient cases, sharing of patients transitioning from pediatric to adult care, and approval of treatment changes. Since its inception in September 2022, a total of 24 patients (39% male) with a median age of 13.58 years have been assessed in person. The patients and their families reported high satisfaction with the quality of the consultation (93% overall satisfaction rate).

**Conclusion:** In conclusion, we present a pilot model that enhances both the actual quality of care and the perceived quality of care by patients. We recommend implementing this patient-centred multidisciplinary model for the care of autoinflammatory diseases in routine clinical practice in referral centers. The establishment of databases will facilitate future improvements in care, registries, and research projects.

**Patient Consent**

Not applicable (there are no patient data)

**Disclosure of Interest**

None declared

## P418 Safety of Canakinumab in pediatric rheumatology: a single center experience

### E. Kılıç Könte, A. Adrovic, K. Ucak, A. Günalp, M. Yildiz, F. Haslak, E. I. Derelioglu, D. Gurleyik, E. Aslan, S. Sahin, K. Barut, O. Kasapcopur

#### Pediatric Rheumatology, Istanbul University, Cerrahpasa, Istanbul, Türkiye

##### **Correspondence:** E. Kılıç Könte

*Pediatric Rheumatology 2023*, **21(Suppl 2):**P418

**Introduction:** Canakinumab is an IL-1β selective human monoclonal antibody in IgG structure used in autoinflammatory diseases and systemic juvenile idiopathic arthritis (SJIA) treatment.

**Objectives:** We aimed to show the drug response and the safety profile of canakinumab in our clinic practice.

**Methods:** A total of 215 patients using canakinumab between 2015-2022 were reviewed, retrospectively. Efficacy and side effects were questioned during patients' routine clinical examinations or by telephone call. One patient was excluded due to irregular follow-up and 3 patients died in the meantime. Finally, 189 were included in the study.

**Results:** Among 189 patients, 115 (60.8%) were females and 74 (39.2%) were males. The mean age of symptom onset was 2 (1-17) years, and the mean age at diagnosis was 4 (1-17) years. The indications for canakinumab treatment were: colchicine-resistant familial Mediterranean fever (FMF) in 106 (56.1%) patients, local reactions to Anakinra in 11 (5.8%) and recurrent autoinflammatory diseases other than FMF in 72 (38%) patients. The mean cumulative dose of canakinumab was 2400 (240-18000) mg. There was at least one hospitalization in 30 (15.9%) patients, systemic reactions in 6 (3.17%), local reactions in 15 (7.9%), protein-purified derived (PPD) positivity in 18 (9.5%) and a history of serious COVID infection in 10 (5.3%) patients during the canakinumab usage. Of the patients, 124 (65.6%) were treated in the prepubertal-pubertal period and 2 (1.6%) were followed up due to delayed puberty. There was not significant correlation between the frequency of infections and the cumulative dose of canakinumab. Erythrocyte sedimentation rate, C-reactive protein (CRP), proteinuria and physician/family general activity score (GAS) significantly decreased after the canakinumab treatment. In 70 patients corticosteroids were used concomitanly with canakinumab. During the follow-up of canakinumab treatment, 3 patients were diagnosed with inflammatory bowel disease, 1 patient with systemic lupus erythematosus, 2 patients with lymphoma, 1 patient with ovarian mass, 1 patient with alopecia areata, and 2 patients with epilepsy. Two SJIA patients were resistant to canakinumab. The post-treatment diagnoses of the patients were not dose-dependent.

**Conclusion:** In our study, improvement in initial symptoms and a statistically significant decrease in acute phase markers indicate the efficacy of canakinumab treatment. Limited number of side effects supports the safety of the drug in pediatric population.

**Patient Consent**

Yes, I received consent

**Disclosure of Interest**

None declared

**References**

1. De Benedetti, F., et al., *Canakinumab for treating Autoinflammatory Recurrent Fever Syndromes.* N Engl J Med, 2018. **378**(20): p. 1908-1919.
2. Del Giudice, E., et al., *Off-label use of canakinumab in pediatric rheumatology and rare diseases.* Frontiers in Medicine, 2022. **9**.
3. Hur, P., et al., *Reasons for canakinumab initiation among patients with periodic fever syndromes: a retrospective medical chart review from the United States.* Pediatr Rheumatol Online J, 2021. **19**(1): p. 143.
4. Kurt, T., et al., *Effect of anti-interleukin-1 treatment on quality of life in children with colchicine-resistant familial Mediterranean fever: A single-center experience.* Int J Rheum Dis, 2020. **23**(7): p. 977-981.

## P419 The Turkish translation and cross-cultural adaptation of the gait outcomes assessments list (for children with lower-limb difference) in patient with juvenile idiopathic arthritis

### A. Namli Seker^1,2^, N. Arman^3^, A. Albayrak^1,4^, A. Yekdaneh^1,5^, F. Demirkan^6^, O. Akgun^6^, N. Aktay Ayaz^6^

#### ^1^Physiotherapy and Rehabilitation Doctorate Program, Institute of Graduate Studies, Istanbul University-Cerrahpasa, Istanbul; ^2^Podology Program, Aydin Adnan Menderes University, Nazilli Vocational School of Health Services, Aydin; ^3^Department of Physiotherapy and Rehabilitation, Istanbul University-Cerrahpaşa Faculty of Health Sciences; ^4^Department of Physiotherapy and Rehabilitation, Istanbul Kent University, Faculty of Health Sciences; ^5^Physiotherapy English Program, Fenerbahçe University Vocational School of Health Services; ^6^Department of Pediatric Rheumatology, Istanbul University, Istanbul Faculty of Medicine, Istanbul, Türkiye

##### **Correspondence:** A. Namli Seker

*Pediatric Rheumatology 2023*, **21(Suppl 2):**P419

**Introduction:** Juvenile idiopathic arthritis (JIA) is the most common chronic inflammatory rheumatic disease. Lower extremity involvement in JIA leads to walking problems. Childhood Health Assessment Questionnaire (CHAQ) as the most commonly used scale has a walking subscale, but the walking subscale is not sufficiently detailed and sensitive. The Gait Outcomes Assessments List (For Children with Lower-Limb Difference) (GOAL-LD) was developed for adequately assessing different aspects of walking in different patient groups with lower extremity problems. The GOAL-LD is suited for use in clinical decision making, especially to implement a treat-to-target approach and follow up.

**Objectives:** The aim of the study was to translate and culturally adapt the GOAL-LD child and parent version into Turkish in patients with JIA.

**Methods:** The GOAL-LD was translated into Turkish by 4 bilingual translators, native speakers of Turkish. The consensus on forward translation was reached by the team. Backward-translation into English was performed by 1 native speaker of English and who were blinded to the original GOAL-LD version. After the review of the Turkish version was prepared by an expert committee that included translators, one patient and the research team a pre-final version. This version was used in a field-test with cognitive debriefing and involved a sample of 11 patients with JIA with variation in gender, age, disease duration, and educational background. The final Turkish GOAL-LD version was reached after the patients were interviewed to check understandability, interpretation and cultural relevance of the translation. The whole process was performed according to the Beaton method. The CHAQ was used to estimate validity.

**Results:** After the forward-backward translation process, small incompatibilities were resolved during the expert committee meeting. 4 sentences and 3 words were revised by the team. Cognitive debriefing showed that items of the GOAL-LD are clear, relevant, understandable, and easy to complete. Two patients stated that they had difficulty in understanding the questions asked about the difficulty level of the activities asked. They said they did not understand whether to associate the complaint of difficulty in walking with fatigue or weakness. During the cognitive debriefing, 2 patients suggested a change in the wording of one item to make it more suitable to Turkish culture. Part E of the GOAL-LD includes the use of brace and assistive devices. Since this scale was developed for Cerebral Palsy with severe lower extremity involvement, this section was excluded because children with JIA do not use these devices. The mean age of the participants was 13.27 years. Significant correlation was found between walking score of the CHAQ and walking-getting around (r:-0.66), physical activities-games-recreation (r:-0,61), body image-self esteem (r:-0.67) and total scores (r:-0.60) of the GOAL-LD child version (p<0.05). Also, significant correlation was found between total score of CHAQ and walking-getting around (r:-0.67), physical activities-games-recreation (r:-0,64), gait appearance (r:-0.68), body image-self esteem (r:-0.60) and total scores (r:-0.67) of GOAL-LD parent version (p<0.05).

**Conclusion:** The final Turkish version of the GOAL-LD parent and child version is acceptable in patients with JIA. It was found there was a significant correlation between walking, physical activity, and body image-self esteem scores GOAL-LD for parents and child version and walking score of CHAQ. These consequences encompass the focus group results obtained from our ongoing study. Validation of GOAL-LD should requires studies with a large sample size encompassing individuals with varying degrees of involvement in patients with JIA.

**Trial registration identifying number:** NCT05846672

**Patient Consent**

Yes, I received consent

**Disclosure of Interest**

None declared

## P420 Clinical experience using interferon Gamma inhibitor emapalumab in treatment of pediatric patients with primary hemophagocytic lymphohistiocytosis (P-HLH)

### Y. Rodina, V. Burlakov, N. Kan, G. Novichkova, A. Shcherbina

#### Dmitry Rogachev National Medical Research Center of Pediatric Hematology, Oncology and Immunology, Moscow, Russian Federation

##### **Correspondence:** Y. Rodina

*Pediatric Rheumatology 2023*, **21(Suppl 2):**P420

**Introduction:** P-HLH is a group of genetically determined disorders with multi-organ damage due to systemic inflammation with high IFN-g production, and high mortality rate, despite extensive immunosuppressive treatment.

**Methods:** We retrospectively analyzed 7 patients with P-HLH (XLP2 – 3, FHLH3 – 2, FHLH1 – 2), treated with emapalumab. Preceding\concurrent immunosuppressive therapy included HLH-2004 protocol variations, with addition of tocilizumab in 3/7, JAKinibs - 4/7, kineret - 1/7. Emapalumab was administrated twice weekly for a median of 6.3 weeks (2-13 weeks), at an average dose of 1.7 mg/kg in 3 patients, and 7.2 mg/kg in 4 patients. The severity of the disease was assessed using adapted H-score, remission was registered at H-score<90.

**Results:** Significant clinical and laboratory improvement was documented after 2 weeks of emapalumab therapy in both groups (p<0.05), all patients reached remission by the end of the treatment. Yet, in high-dose group 2/4 patients reached remission by day 14, and 1/4 by day 28. In the low-dose group no remission was noted at day 14, 1/3 reached remission by day 28.

In the whole cohort addition of emapalumab allowed to reduce dexamethasone dose from 10±2 at baseline to 0.7± 0.4 mg/kg/day by week 4. No side effects or new infections were noted during the treatment. 6/7 patients underwent HSCT at the end of the treatment period, without relapse of HLH in the post-transplant period.

**Conclusion:** We demonstrate efficacy and safety of Emapalumab in treatment of severe P-HLH in a group of children. The optimal doses of emapalumab require further investigation.

**Patient Consent**

Not applicable (there are no patient data)

**Disclosure of Interest**

None declared

## P421 Increasing the etanercept dose in juvenile idiopathic arthritis patients: does it help reaching the treatment target? A Post-HOC analysis of the Best4Kids randomised clinical trial

### B. T. Van Dijk^1,2^, S. A. Bergstra^2^, J. M. van den Berg^3^, D. Schonenberg-Meinema^3^, L. W. van Suijlekom-Smit^4^, M. A. van Rossum^4,5^, Y. Koopman-Keemink^6^, R. ten Cate^1^, C. F. Allaart^2^, D. M. Brinkman^1^, P. C. Hissink-Muller^1^

#### ^1^Paediatrics, division of Paediatric Rheumatology, Willem Alexander Children's Hospital; ^2^Rheumatology, Leiden University Medical Centre (LUMC), Leiden; ^3^Paediatric Immunology, Rheumatology and Infectious Diseases; ^4^Paediatrics, Emma Children's Hospital, Amsterdam University Medical Centers; ^5^Paediatric Rheumatology, Amsterdam Rheumatology and Immunology Centre (Reade), Amsterdam; ^6^Paediatrics, HagaZiekenhuis Juliana Children's Hospital, the Hague, Netherlands

##### **Correspondence:** B. T. Van Dijk

*Pediatric Rheumatology 2023*, **21(Suppl 2):**P421

**Introduction:** Etanercept is a frequently used second-line therapy for juvenile idiopathic arthritis (JIA). It is unknown whether higher doses of etanercept result in better clinical outcomes, but most studies only evaluated doses up to the most commonly used 0.8 mg/kg/week (max 50mg/week).[1] Although higher doses are used off-label in clinical practice, the literature lacks an in-depth description of patients receiving such treatment.

**Objectives:** To describe the clinical course of JIA-patients that received high-dose etanercept (1.6 mg/kg/week; max 50mg/week) as part of the Best4Kids trial.

**Methods:** In a single-blinded treatment-strategy trial, patients with oligoarticular JIA, RF-negative polyarticular JIA or juvenile psoriatic arthritis were randomised across three treatment-strategy arms: (1) sequential DMARD-monotherapy (sulfasalazine or methotrexate (MTX)), (2) combination-therapy MTX+6 weeks prednisolone and (3) combination therapy MTX+etanercept.[2] A protocolised treat-to-target approach aiming for inactive disease was used during 24 months follow-up. Treatment was escalated in case of persistent disease-activity or tapered in case of inactive disease. In any treatment-arm patients could eventually escalate from regular to high-dose etanercept alongside MTX 10mg/m^2^/week. For comparison we studied patients who did not receive high-dose etanercept due to decisions overriding the trial-protocol by the treating paediatrician and/or the patient/parents.

**Results:** Of the 94 randomised patients, 32 received high-dose etanercept (69% female, median age 6 years (IQR 4-10), median time from baseline 10 months (7-16), median dose 1.3 mg/kg/week (1.1-1.5)). Follow-up was up to 2 years from baseline (median follow-up 24.6 months). Clinical measures of disease-activity decreased largely within 3 months: median VAS-physician from 12 to 4 (p=0.022), VAS-patient/parent from 38.5 to 13 (p=0.003), VAS pain from 35.5 to 15 (p=0.030), number of active joints from 2 to 0.5 (p=0.12) and JADAS10 from 7.2 to 2.8 (p=0.008). Functional status (CHAQ-score) improved more gradually and ESR remained stable. A comparable pattern of clinical parameters over time was observed in 11 patients (73% girls, median age 8 (IQR 6-9)) who were not escalated to high-dose etanercept despite eligibility according to trial-protocol. In both the high-dose and the comparison group the percentage of patients with inactive disease 6 months after eligibility for dose-increase was 56%. In the high-dose group, 18 out of 32 patients (56%) experienced 26 infectious adverse events (AEs, on average 0.20 events per visit following dose-increase). No serious AEs (SAEs) were recorded after dose-increase. Among the 11 patients who did not receive high-dose etanercept, 4 patients (36%) subsequently experienced 5 infectious AEs (on average 0.11 events per visit); this included one SAE requiring hospitalisation. Findings were similar when one patient who switched to infliximab, instead of maintaining the regular etanercept dose, was excluded from the comparison group.

**Conclusion:** Escalation to high-dose etanercept was generally followed by meaningful clinical improvement within 3 months. However, non-escalators experienced comparable improvement. These data do not suggest superior clinical outcomes after etanercept dose increase, while there was a potential trend for more (non-severe) infectious AEs. Larger studies are needed to more closely examine outcomes, adverse events and cost-effectiveness of high dose etanercept.

**Trial registration identifying number:** Netherlands Trial Register NL1504

**Patient Consent**

Not applicable (there are no patient data)

**Disclosure of Interest**

None declared

**References**

1. Verstegen RHJ et al. Arthritis Care Res (Hoboken). 2022. doi: 10.1002/acr.24859
2. Hissink Muller P et al. Ann Rheum Dis. 2019;78(1):51-9. doi: 10.1136/annrheumdis-2018-213902.

## P422 Bone density in children with hypermobility spectrum disorder compared to children with benign hypermobility

### S. Barzamini, K. Rahmani, R. Shiari

#### Department of Pediatric Rheumatology, Faculty of Medicine, Shahid Beheshti University of Medical Sciences, Tehran, Iran, Islamic Republic Of

##### **Correspondence:** S. Barzamini

*Pediatric Rheumatology 2023*, **21(Suppl 2):**P422

**Introduction:** Hypermobility spectrum disorder (HSD) which is probably a multifactorial disorder determined by musculoskeletal pain, joint instability, and a reduced bone mineral density (BMD)[1]. Benign joint hypermobility syndrome (BJHS) is a type of connective tissue disorder with hypermobility in which widespread musculoskeletal symptoms are observed without rheumatological findings. So, we decided to determine and compare bone density in children with hypermobility spectrum disorder (HSD) and children with benign hypermobility.

**Objectives:** This case-control study was conducted on 73 children with hypermobility spectrum disorder and children with generalized hypermobility referred to the Rheumatology clinic of Mofid Hospital, in Tehran in 2022. Sampling was done by convenience method. The inclusion criteria of patients required a medically documented diagnosis, and a Beighton score of ≥4 and be between 3-16 years of age. The exclusion criteria were known rheumatological disease, other forms of connective tissue diseases, any contraindications to a DXA scan and parents' dissatisfaction with their child's participation in the study.

**Methods:** The quantitative and qualitative variables were indicated as mean±SD and number (percentage), respectively. Kolmogorov–Smirnov and, Shapiro–Wilk tests were used to test for the distribution. The comparison of bone density in two groups was done with independent t-test. The chi-square test was used to compare the qualitative data between two groups. Linear regression and logistic modeling were used to control confounding variables. P<0.05 was considered significant.

**Results:** The mean BMD Z-score of the spine and the whole body of total patients were -0.8±1.2 and -1.8±1.5, respectively. AP Spine (L1-L4) BMD was significantly different between benign and HSD groups (P=0.001). Moreover, whole body BMD was remarkably different between the two groups (P=0.008), While there was no considerable difference in whole body BMC between the two groups (P=0.06).

Based on the BMD Z-score of the spine; in the HSD group 18 patients (81.8%) had normal BMD, and 4 patients (18.2%) had low bone density, osteoporosis was not observed in HSD group patients. In the Benign Hypermobility group, according to the BMD Z-score of the spine, normal condition, low bone density and osteoporosis were observed in 36.5%, 56.2% and 7.3% of patients, respectively. Based on BMD Z-score of the spine, two groups had a significant difference in terms of the frequency distribution of the spine (P=0.002). Furthermore, a surprising statistical difference was observed in terms of frequency distribution of whole body bone condition based on BMD Z-score (P=0.04)

**Conclusion:** Considering the high rate of osteoporosis in benign patients, the tendency to osteopenia should be considered when treating benign patients, and this issue may One of the limitations of this study was the small sample size because the study was conducted during the COVID-19 pandemic and patients did not go to the clinic with any musculoskeletal pain.

**Patient Consent**

Yes, I received consent

**Disclosure of Interest**

None declared

**References**

1. Banica, T., et al., *Higher fracture prevalence and smaller bone size in patients with hEDS/HSD—a prospective cohort study.* Osteoporosis International, 2020. **31**: p. 849-856.
2. Gulbahar, S., et al., *Hypermobility syndrome increases the risk for low bone mass.* Clinical rheumatology, 2006. **25**: p. 511-514.

## P423 The journey to diagnosis of CRMO: a single-centre case series

### L. Crosby^1^, R. Geetala^2^, K. Armon^1^

#### ^1^Cambridge University Hospitals NHS Foundation Trust; ^2^Cambridge University, Cambridge, United Kingdom

##### **Correspondence:** L. Crosby

*Pediatric Rheumatology 2023*, **21(Suppl 2):**P423

**Introduction:** Chronic recurrent multifocal osteomyelitis (CRMO) is an autoinflammatory bone disease seen in the paediatric population. Delayed diagnosis may result in unnecessary admissions, futile treatment for different pathologies and failure to treat symptoms that impact on quality of life.

**Objectives:** To describe the journey to diagnosis for children with CRMO at our tertiary paediatric rheumatology centre, examining their demographics and comparing to the current literature.

**Methods:** The electronic medical records of children (<16 years) with a diagnosis of CRMO currently under the care of the paediatric rheumatology team at Cambridge University Hospitals were included in the review.

**Results:** 17 children were identified, 82% were female. Median age at symptom onset was 9 years (range 4-15) and median age at diagnosis was 10 years (range 5-14). All patients were seen by paediatric rheumatology, 59% by orthopaedics, 41% by paediatric oncology, 35% by general paediatrics, 6% by neurosurgery. 2 children with psoriasis or pustular skin conditions were seen by dermatology. Prior to diagnosis of CRMO, 18% of children received a diagnosis of an infective pathology. Langerhans cell histiocytosis was the suspected or working diagnosis in 12%, and sarcoma in 12%. Median time to diagnosis from symptom onset was 259 days (range 34-1418). CRMO was diagnosed by paediatric rheumatology in 71%, orthopaedics in 24%, and oncology in 6%. Children who saw paediatric rheumatology as their first tertiary referral had the shortest median time from first appointment to diagnosis (31 days) compared to oncology (35) or orthopaedics (50). The number of specialties involved inversely correlated to time between symptom onset and diagnosis, with a median 482 days for those seen by 1 secondary care specialty, and 100 days for those seen by 4. 100% of children had a whole-body MRI. The most common site of disease on whole body MRI was tibia and fibula (75% of patients), clavicle (59%) and pelvis (47%).

**Conclusion:** Diagnosing CRMO requires exclusion of serious and similarly-presenting pathologies and a multi-specialty approach is essential. This is supported by the inverse correlation between number of specialty teams involved and time to diagnosis in this series. The median time from symptom onset to diagnosis in our report is shorter than previously reported, with a greater proportion referred directly to paediatric rheumatology(1). This may reflect an increasing awareness of this rare but important condition. Long bone metaphyses were the most frequent site of disease. The pelvis was over-represented in our patient population in comparison to other patient series(1). Paediatric rheumatologists caring for children with CRMO should be mindful of the often protracted and complicated journey prior to arrival in their care.

**Patient Consent**

Not applicable (there are no patient data)

**Disclosure of Interest**

None declared

**References**

1. Roderick MR, Shah R, Rogers V, Finn A, Ramanan A V. Chronic recurrent multifocal osteomyelitis ( CRMO ) – advancing the diagnosis. Pediatr Rheumatol [Internet]. 2016;1–5. Available from: 10.1186/s12969-016-0109-1

## P424 Clinico-radiological features in 30 patients with chronic non- bacterial osteitis (CNO) from a centre in Mumbai, India

### D. Ramadoss^1^, A. Khan^1^, B. Jankharia^2^, H. Panwala^3^, S. Garg^3^, R. Khubchandani^1^

#### ^1^Pediatric Rheumatology, SRCC Childrens Hospital; ^2^Radiology, Picture This; ^3^Pediatric Radiology, SRCC Childrens Hospital, Mumbai, India

##### **Correspondence:** R. Khubchandani

*Pediatric Rheumatology 2023*, **21(Suppl 2):**P424

**Introduction:** CNO-an acquired auto inflammatory bone disease affects children / young adults

**Objectives:** Our experience with CNO between Nov 2016-Feb 2023

**Methods:** Retrospective demographic and clinical /radiologic analysis

**Results:** Eighteen were males and 12 females. The mean age at presentation was 9 years (Range 4-14 years). Median time from onset of complaints to referral was 8 months (Range1m- 132m). Pediatricians referred 16(53%), pediatric orthopedicians 9(30%), radiologists 3(10%) and one each by a pediatric oncologist and adult rheumatologist. Time to diagnosis from referral ranged from 0-40 months. At presentation 22(73%) patients were suspected. Delay occurred in our first case with ulcerating skin and those with atypical features. Presenting features were painful gait (93%), local bone pains (77%) and symptomatic arthritis (10%). Palmoplantar psoriasis preceded skeletal complaints in 2 while 5 had associated psoriasis at presentation. Two had acne and evolved into SAPHO and one evolved from trigger thumb in infancy to twenty nail dystrophy at 4 years and CNO at 6 years. Fever was seen in 2 and erythema nodosum, urticaria, lymphadenopathy and proximal myopathy in 1 each. Family history of psoriasis was present in 5, and HLA B27 associated arthritis in 3. Median laboratory values were (Range in brackets) Hemoglobin 12.1g/dL (10.8- 13.6 gm/dL), Leukocyte count 8700x10^9^/L (5450-16530x10^9^/L), Platelet count 3.65x10^9^/L (2.1- 6.04 x10^9^/L), ESR 30 mm/hr(2-72 mm/hr), and CRP 6mg/dL (0.1- 46 mg/dL).Whole- body MRI was performed in 28 and a bone scan in 2. Eleven out of 13 bone biopsies performed prior to referral could have been avoided applying Bristol criteria. We biopsied 2 patients ,1 with a raised ESR/CRP and the other with a monostotic lesion in the tibial diaphysis to rule out malignancy. The mean count of bony lesions / patient on MRI was 6 (Range:1-15) with 2 having single lesions in sacrum and tibia. The most common site was tibia (44/173), followed by femur (33/173). Axial and appendicular lesions were seen in 18 (60%), appendicular in 7(23%), axial in 5(17%) and 1 had a mandibular lesion. Cranial bones were spared. Meta-diaphyseal involvement was seen in 3 Fifteen (50%) patients had associated arthritis on MRI (monoarticular -7, polyarticular-8) of which 6 had an ESR >30 mm/hr.

**Conclusion:** Awareness of CNO and Bristol criteria can reduce latency of diagnosis and reduce biopsies though atypical cases may receive delayed diagnosis. Associated psoriasis or a family history of psoriasis/HLA B27 arthritis and our infant who evolved from trigger thumb and twenty nail dystrophy inspire genetic study. Cranial bones were not involved and 15 (50%) had arthritis on MRI with symptomatic presenting arthritis in 3.

**Patient Consent**

Not applicable (there are no patient data)

**Disclosure of Interest**

None declared

## P425 The prospects for using of bisphosphonates in children with avascular necrosis of the femoral head course in form of osteoarthritis

### A. Kozhevnikov^1^, N. Pozdeeva^2^, D. Barsukov^1^, A. Gubaeva^1^, G. Novik^3^

#### ^1^H.Turner National Medical Research Center for Children's Orthopedics and Trauma Surgery; ^2^State Budgetary Healthcare Institution Children's Out-Patient Department №49; ^3^Saint-Petersburg State Pediatric Medical University, Saint-Petersburg, Russian Federation

##### **Correspondence:** A. Kozhevnikov

*Pediatric Rheumatology 2023*, **21(Suppl 2):**P425

**Introduction:** Avascular necrosis of the femoral head (AVN) in children remains a focus in specialists despite long historical of study. AVN is a multifactorial disease with non-inflammatory nature. The disease occurs in young and middle-aged children, and proceeds through several stages characterized by clinical and instrumental picture. Some children develop more aggressive course disease in form of osteoarthritis (OA) related to the hyperactivity osteoclasts. The prevalence of chronic inflammatory in children with AVN can leads to the osteoarthritis. Osteoarthritis characterized by chronic synovial tissue inflammation differs from autoimmunity diseases. Some researchers reported that the chronic synovitis increased focus of osteonecrosis, leads to femoral head subluxation and no allow surgical treatment. The use of bisphosphonate ibandronic acid (IA) is aimed to inhibit osteoclasts and reduce inflammatory.

**Objectives:** The proposed this study directed to evaluate the efficacy of IA treatment of OA developed in children with AVN (OA/AVN).

**Methods:** The proposed control trial recruiting 20 children with OA/AVN assigned IA. Control group 20 children with OA/AVN treated by standard care. The outcome measure will radiographic deformity index (DI) and hip inflammation MRI scoring system (HIMRISS) assessed at 12 months following of IA treatment (3-monthly doses of IA 1,5 mg at baseline children before 7 yr and 2,0 mg - older, 3, 6, 9 and 12 months). VAS score will used to measure pain. Laboratory blood inflammatory tests included ESR, CRP, ANA, IL6, TNF-alpha and markers of bone metabolisms.

**Results:** Now 9 children with OA/AVN included in the study received only one intravenous of IA, 4 children - at least get 2 infusions of IA. Pre results indicated reduce joint pain after first infusion of IA and significant decrease bone marrow edema and synovitis by MRI imaging after second dosage. Serum concentration of IL6, TNF-alpha and CRP were normally. ANA were positive in 77,8%. Vitamin D were 18 ng/ml (±4,2).

**Conclusion:** Use of bisphophonates in OA developed in children with AVN had shown promise to treatment. But this first data requires further detailed study.

**Patient Consent**

Not applicable (there are no patient data)

**Disclosure of Interest**

None declared

## P426 Isotretinoin in SAPHO syndrome: a triggering factor for acute onset?

### G. Minca^1^, A. Meneghel, M. L. Cagnato, A. Agazzi, F. Tirelli, G. Martini, F. Zulian

#### Pediatric Rheumatology Unit, Department for Women's and Child's Health, University Hospital of Padova, Padova, Italy

##### **Correspondence:** G. Minca

*Pediatric Rheumatology 2023*, **21(Suppl 2):**P426

**Introduction:** SAPHOs (Synovitis Acne Pustulosis Hyperostosis Osteitis syndrome) is a disorder characterized by skin and bone symptoms with a relapsing-remitting chronic course and mono- or multifocal involvement. Isotretinoin (ITN), an oral retinoid used in severe acne, can trigger a syndrome (Acne Fulminans with systemic symptoms, AF) characterized by fever, fatigue and bone involvement. Differential diagnosis between SAPHOs and AF may be difficult.

**Objectives:** We report two patients developing osteoarticular symptoms coherent with SAPHOs during ITN therapy.

**Methods:** Case series

**Results:** Case1: A 13 year-old boy with nodulocystic acne started ITN. After 6 weeks, he developed acute lower limbs pain and fever. Lab tests showed: ESR 93 mm/h, CRP 58 mg/L, negative autoantibodies and HLAB27. ITN induced AF was suspected and corticosteroid (CS) treatment was started. After CS withdrawal, fever relapsed and osteoarticular symptoms persisted with a migratory pattern so ITN was discontinued and a whole body MRI was performed demonstrating osteitic lesions in femurs, tibias and fibulas. SAPHOs was suspected and intravenous bisphosphonates were started with pain resolution and lab tests normalization. Case2: A 15 year-old girl started ITN for nodulocystic acne; after 1 month she reported sudden onset of low back pain with fever and gait impairment. Lab tests showed: ESR 56 mm/h, CRP 9 mg/L, negative HLAB27. Pelvis MRI demonstrated bilateral sacroiliitis. A Tc 99 bone scan confirmed sacroiliitis and revealed an abnormal uptake in the sternum. Suspecting SAPHOs, intravenous biphosphonates were started with rapid clinical and lab tests improvement.

**Conclusion:** The role of ITN as a triggering factor for development of osteoarticular manifestations in patients with isolated cutaneous symptoms of SAPHO has been described (2,3,4). Some Authors reported the acute onset of systemic symptoms (fever, musculoskeletal pain) during ITN therapy, ascribing them to a different clinical entity, AF, that doesn’t routinely require isotretinoin discontinuation, being usually responsive to systemic steroids. Whether this syndrome represents the unrecognized counterpart of SAPHOs is difficult to establish, given the rarity of both conditions. Therefore, we suggest to perform musculoskeletal imaging in patients treated with ITN developing bone involvement, to rule out a SAPHOs and properly target the therapy.

**Patient Consent**

Yes, I received consent

**Disclosure of Interest**

None declared

**References**

1. Luzzati M,Simonini G,Filippeschi C et al. SAPHO syndrome: the supposed trigger by isotretinoin, the efficacy of adalimumab and the specter of depressive disorder: a case report. Ital J Pediatr.202;46(1):169.
2. Shahada OO,Kurdi AS,Aljawi AF et al. Synovitis, Acne, Pustulosis, Hyperostosis, and Osteitis Syndrome Diagnosis in Adolescent and Isotretinoin as a Possible Serious Exacerbating Factor.Cureus.2022;14(3):e22776.
3. Karatas Togral A,Yıldızgoren MT,Koryurek OM et al. Can Isotretinoin Induce Articular Symptoms in SAPHO Syndrome?West Indian Med J.2015;64(2):167-8.

## P427 Progressive osseous heteroplasia as a cause of pathological ossification in children: small case-series report

### S. Arsenyeva, I. Nikishina, V. Matkava, A. Arefieva

#### Paediatric, V.A. Nasonova Scientific Research Institute of Rheumatology, Moscow, Russian Federation

##### **Correspondence:** I. Nikishina

*Pediatric Rheumatology 2023*, **21(Suppl 2):**P427

**Introduction:** Progressive osseous heteroplasia (POH) is an ultrarare genetic disorder characterized by progressive heterotopic ossification (HO). Most cases of POH are caused by heterozygous inactivating mutations in the GNAS gene and usually presents in infancy. It starts in the dermis and progresses into the deep connective tissue and skeletal muscle, which may leads to loss of affected areas mobility and disability. POH is often misdiagnosed due to the lack of awareness among doctors and interpretation of HO as calcification incorrectly.

**Objectives:** To describe case series of POH and analyze the main clinical features and variety of GNAS mutation in patients (pts).

**Methods:** Among the children examined in our center with different types of HO, including fibrodysplasia ossificans progressive and traumatic HO, we identified 6 pts with POH. All pts met diagnostic criteria for POH (the presence of superficial/deep HO in the absence of other Albright hereditary osteodystrophy features and without hormone resistance). Genetic tests (3-whole-exome sequencing, 3-target sequencing) performed in all pts and GNAS mutation was confirmed. Radiological assessment was done in all pts, whole-body CT in 1 pt.

**Results:** We presented the data of 6 pts with POH (4 male/2 female); the average onset age was 22.1 months (from 2 weeks to 4 years). All of them had diagnostic delay from 2,5 to 20 years. Skin biopsy was done in 4 pts and led to progression of HO, but did not help in disease diagnostic. In the most pts ossification was seen in the extremities (2 pts-lower, 2 pts-upper) and 2 pts had small ossifications in different part of the body (head, abdomen, upper gingiva). Unilateral involvement without midline crossing was detected in 3 pts (50%), 2 had severe skin damage connected with HO. These pts had severe limitation of joint motion with ankylosis (2-shoulder, elbow, wrist;1-hip, knee, ankle) and significant limb shortening in 2 cases. Bilateral asymmetrical involvement with superficial widespread HO was detected in 3 pts (50%), without typical skin lesions, apart skin perforation by HO. The serum levels of calcium, phosphorus, and alkaline phosphates were in normal range. No parathyroid endocrine abnormalities were detected. Also we noted delay of neuropsychiatric development in 3 pts, which may be considered as comorbidity. All pts had different GNAS mutations, 3 pts-de novo, in 3 cases parents were not investigated yet. In our latest pt mutation c.137T > C,p.Leu46Pro was identified in exon 2 of the GNAS gene that has not previously been described, but it was combined with POH features, so we supposed it can be pathogenic mutation.

**Conclusion:** Our data allowed us to divide pts into 2 groups with distinct POH phenotypes: unilateral dermomyotomal distribution with deep ossification, severe skin lesions, disability and group with mosaic chaotic distribution of superficial HO with no functional impairment. Further research is needed to understand the relationship between the type of GNAS mutation and variability of POH phenotypes.

**Patient Consent**

Not applicable (there are no patient data)

**Disclosure of Interest**

None declared

## P428 Unusual limping child: aneurysmal bone cyst

### F. Paciello^1^, G. Federico^2^, F. Orlando^3^, P. Guida^2^, L. Martemucci^3^, M. Tardi^3^

#### ^1^Pediatric Emergency and Short Stay Unit; ^2^Department of Orthopaedic Surgery; ^3^Pediatric Rheumatology Unit, Department of General and Emergency Pediatrics, Santobono-Pausilipon Children's Hospital, Naples, Italy

##### **Correspondence:** F. Paciello

*Pediatric Rheumatology 2023*, **21(Suppl 2):**P428

**Introduction:** Limping is a common chief complaint in the Pediatric Emergency Department. It is important to differentiate among the multitude of diagnoses evaluating associated symptoms, diagnostic tests, physical examination, and patient’s age.

**Objectives:** To present a case of unusual limping child.

**Methods:** A two-year-old male admitted to our Unit due to a month history of right-sided limping not relieved by NSAIDS. No history of trauma or fever in his medical history except for recent coryzal symptoms.

**Results:** On the clinical evaluation, the child was afebrile with minimal limitation of internal and external rotation of the right hip. Bilateral hip ultrasound showed right intracapsular effusion. Blood tests including full blood count and C reactive protein were normal. Pelvis X-ray revealed an oval area (maximum diameter 25 x 17 mm) of bone erosion with irregular margins involving the proximal right femoral diaphysis. CT scan confirmed the lytic lesion surrounded by an irregular, discontinuous and scalloped bone border. MRI better defined multiloculated appearance with multiple cyst-like areolae containing fluid levels. All those findings were consistent with an aneurysmal bone cyst (ABC), confirmed by biopsy. Curettage of the cyst was performed, and the cystic space was filled with an injectable bone sostitute.

**Conclusion:** ABC is a non-neoplastic osteolytic lesion that may exist as a primary bone cyst or as a secondary lesion arising from other osseous conditions such as giant cell tumors or unicameral bone cysts. ABC is exceptionally rare with a reported incidence of 0.14 per 100,000 individuals. Lesions occur primarily in the first and second decades of life. The long bones (especially the tibiae and femur) and vertebrae are the most common sites. Pain is the most common clinical symptom at presentation. The radiographic differential diagnosis includes unicameral bone cysts, giant cell tumors, osteosarcoma, and osteoblastoma (in vertebral lesions). The diagnosis must be based on histopathologic evidence.Local recurrence rate after classic surgical procedures (curettage and grafting) is about 11.8-30.8%. ABC is an uncommon cause of limping in children but should be considered in the patient with a persistent limp after more common causes have been excluded. The combination of different imaging modalities including plain radiography, CT scan, MRI, and bone scans helps to accurately establish and confirm the diagnosis.

**Patient Consent**

Yes, I received consent

**Disclosure of Interest**

None declared

## P429 Mucolipidosis mimicking juvenile idiopathic arthritis: the first reported 3 siblings in Libya

### A. M. A. Abushhaiwia^1^, Y. Elfawires^1^, A. Ateeq^2^

#### ^1^Pediatrics Rheumatology, University of Tripoli, Faculty of Medicine, Tripoli Children's Hospital; ^2^Pediatrics Rheumatology, Tripoli Children's Hospital, Tripoli, Libya

##### **Correspondence:** A. M. A. Abushhaiwia

*Pediatric Rheumatology 2023*, **21(Suppl 2):**P429

**Introduction:** Mucolipidosis type III gamma (MC III) is an extremely rare, inherited, progressive lysosomal storage disease that is characterized by a progressive slowing of the growth rate in early childhood; stiffness and pain in shoulders, hips, and finger joints; a gradual, mild coarsening of facial features. Due to its rarity, it can be often misdiagnosed as juvenile idiopathic arthritis (JIA). Hereby, we present 3 cases of in 3 siblings who was initially managed as JIA. Our cases represents the first reported cases in the state of Libya.

**Objectives:** To highlights the importance of considering such rare genetic disease entities.

**Methods:** sequencing including NGS based CNV analysis was done for one the elder two twin sibling, a homozygous pathogenic variant was identified in the GNPTG gene. the result is consistent with the genetic diagnosis of autosomal recessive mucolipidosis III gamma

**Results:** 3 siblings ( the elder two are twins and 8 years old , the youngest one is 5 years old ) have presented to our pediatric Rheumatology clinic as a referral case from the private sector with the complaint of progressive joint contractures affecting multiple joints. They are a product of a non Consanguineous marriage, with other healthy siblings, one girl and two boys and history of one sibling died at age of 33 days due to RDS? (a boy ) before the birth of these three boys. All of them have no significant medical nor surgical history worth mentioning except for the progressive joint contractures involving ( hips, knees, ankles, neck, back inform of mild scoliosis with winging of scapulae, hands, wrist, shoulder and neck ) in form of restriction in range of movement, tenderness, muscle wasting and loss of function affecting their daily activity, school performance and play. The features were noted soon after the child starts to cruise around furniture in early infancy. The problem is also associated with subtle dysmorphic features and a degree of intellectual disability and delayed milestones. The problem is affecting them with varying degrees and little one or youngest one being the least affected. Other systemic examination is free including Neuro, cardio, abdominal, chest and eye examinations, and hearing assessment. They were evaluated for the possibility of CACP and the disease was excluded with negative gene test ( one of older one was tested with molecular genetic analysis of the PRG4 gene )Also for the possibility of MPS, the enzyme activities were above cut off values and therefore Mucopolysaccharidosis type I, II, IIIB, IVA, VI, and VII were unlikely in the dried blood spot test. ( tandem mass spectrometry) . All three siblings were tested. Echocardiography done with no significant anomaly seen, skeletal survey to show extent of joint damage, routine blood tests, neurology and metabolic specialty consultation done as part of the workup. diagnosis of mucolipidosis (type III) which was confirmed on genetic testing.

**Conclusion:** Our 3 sibling cases represents an extremely rare lysosomal disorder that can be easily misdiagnosed as JIA. Genetic test is a very essential test in the evaluation and diagnosis of the condition this family is suffering from in.

**Patient Consent**

Yes, I received consent

**Disclosure of Interest**

None declared

## P430 Antibiotic exposure in prenatal and early life and risk of juvenile idiopathic arthritis: a nationwide register-based cohort study

### S. Hestetun^1,2^, S. Andersen^2,3^, H. Sanner^1,4^, K. Størdal^5,6^

#### ^1^Department of Rheumatology , Oslo University Hospital; ^2^Institute of Clinical Medicine, University of Oslo, Oslo; ^3^Pediatric Department, Vestfold Hospital Trust, Tønsberg; ^4^Oslo New University College; ^5^Pediatric department, Oslo University Hospital; ^6^Pediatric research Institute, Institute of Clinical Medicine, University of Oslo , Oslo , Norway

##### **Correspondence:** S. Hestetun

*Pediatric Rheumatology 2023*, **21(Suppl 2):**P430

**Introduction:** Early antibiotic exposure influences the gut microbiota which is believed to be involved in the pathogenesis of juvenile idiopathic arthritis (JIA) (1-4).

**Objectives:** To investigate the association between systemic antibiotics in prenatal and early life and risk of JIA.

**Methods:** We conducted a register-based cohort study including all children born in Norway from 2004 through 2012. The children were followed until December 31, 2020. Main exposures were dispensed antibiotics to the mother during pregnancy and to the child during 0-24 months of age. The outcome was defined by diagnostic codes indicating JIA. Multivariate logistic regression analyses were performed to estimate the association between antibiotic exposure and JIA.

**Results:** We included 535 294 children and their mothers in the analyses; 1011 cases were identified. We found an association between exposure to systemic antibiotics during 0-24 months and JIA (adjusted OR (aOR) 1.40, 95% CI 1.24 to 1.59), with a stronger association for >1 course (aOR 1.50, 95% CI 1.29 to 1.74) vs. one course (aOR 1.31, 95% CI 1.13 to 1.53). Sub-analyses showed significant associations in all age periods except 0-6 months, and stronger association with sulfonamides/trimethoprim and broad-spectrum antibiotics. There was no association between prenatal antibiotic exposure and JIA.

**Conclusion:** The novel observation of no association with prenatal antibiotic exposure and JIA suggests that the association between antibiotics in early life and JIA is unlikely to be confounded by shared family factors. This may indicate that exposure to antibiotics in early life is an independent risk factor for JIA.

**Patient Consent**

Not applicable (there are no patient data)

**Disclosure of Interest**

S. Hestetun: None declared, S. Andersen Grant / Research Support with: has received travel funding from Ferring pharmaceuticals, H. Sanner : None declared, K. Størdal : None declared

**References**

1. McDonnell L, Gilkes A, Ashworth M, Rowland V, Harries TH, Armstrong D, et al. Association between antibiotics and gut microbiome dysbiosis in children: systematic review and meta-analysis. Gut Microbes. 2021;13(1):1-18.
2. De Filippo C, Di Paola M, Giani T, Tirelli F, Cimaz R. Gut microbiota in children and altered profiles in juvenile idiopathic arthritis. J Autoimmun. 2019;98:1-12.
3. Xin L, He F, Li S, Zhou ZX, Ma XL. Intestinal microbiota and juvenile idiopathic arthritis: current understanding and future prospective. World J Pediatr. 2021;17(1):40-51.
4. Arvonen M, Berntson L, Pokka T, Karttunen TJ, Vahasalo P, Stoll ML. Gut microbiota-host interactions and juvenile idiopathic arthritis. Pediatr Rheumatol Online J. 2016;14(1):44.

## P431 Comprehensive multiomic profiling of disrupted regulatory networks in COVID19 associated multisystem inflammatory syndrome in children

### B. Jenko Bizjan^1,2^, R. Sket^1,2^, T. Tesovnik^1,2^, J. Kovac^1,2^, M. Debeljak^1,2^, M. Zajc Avramovic^3^, N. Emersic^3^, K. Črepinšek^1^, B. Slapnik^1^, B. Vrhovsek^1^, T. Avcin^2,3^

#### ^1^Clinical Institute of Special Laboratory Diagnostics, University Children’s Hospital, UMC; ^2^Faculty of Medicine, University of Ljubljana; ^3^Department of Allergology, Rheumatology and Clinical Immunology, University Children’s Hospital, UMC, Ljubljana, Slovenia

##### **Correspondence:** B. Jenko Bizjan

*Pediatric Rheumatology 2023*, **21(Suppl 2):**P431

**Introduction:** Children infected with severe acute respiratory syndrome coronavirus 2 (SARS-CoV-2) usually present minimal symptoms or are asymptomatic. Nevertheless, a subset of children 2-6 weeks after the initial SARS-CoV-2 infection develops a postinfectious SARS-CoV-2-related multisystem inflammatory syndrome in (MIS-C). Recently, transient expansion of TRBV11-2 T cell clonotypes in MIS-C was associated with signatures of inflammation and T cell activation, however the underlying pathophysiology of MIS-C is not fully understood [1].

**Objectives:** The purpose of our project is to characterize the complexity of cell populations and capture cellular heterogeneity to uncover the regulatory networks that are disrupted during MIS-C flare with simultaneous profiling of gene expression and open chromatin regions. Moreover, we are exploring gene regulatory interactions driving inflammation in MIS-C.

**Methods:** Samples of peripheral blood mononuclear cells from patients with MIS-C diagnosed at the University Children’s Hospital, University Medical Center Ljubljana, were collected during the initial presentation before any treatment and at 6-12 months in remission. Primary aim is to identify which regulatory networks are disrupted during MIS-C flare, for which we are performing single cell Multiome ATAC + Gene Expression Sequencing. To enable simultaneous profiling of epigenomic landscape and gene expression from the same nuclei, we are using Chromium Next GEM Single Cell Multiome ATAC + Gene Expression kit from 10X Genomics.

**Results:** We included 50 patients with MIS-C from whom we collected paired blood samples during the initial presentation before treatment and at 6-12 months in remission. All samples were included into bulk transcriptomic profiling (RNA sequencing). Additionally, we have included 10 patients, with the most viable cell count prior cryopreservation into single cell multiomic experiment. All samples that were included into multiomic single cell analysis have 75% - 99% viability prior cryopreservation. In the protocol the key is to remove remaining granulocytes causing high mitochondrial RNA burden and extensively optimise the dilution factor of lysis buffer and the length of cell lysis step in order to get intact nuclei with no significant blebbing. Data are undergoing pairwise analysis to compare the cell population heterogeneity, expression profile and open chromatin landscape in the time the initial presentation of MIS-C and in the remission, with Cell ranger software as well as with R package scREG [2], and custom scripting.

**Conclusion:** The results of this project are expected to enlighten the underlying pathophysiology of MIS-C flare and thus support clinical decision on more targeted treatment. The identified disrupted networks during MIS-C flare could lead the way to establish an early diagnosis and improve long-term outcome, including prevention of myocardial and neuropsychological impairment.

**Patient Consent**

Not applicable (there are no patient data)

**Disclosure of Interest**

None declared

**References**

1. Sacco, K., Castagnoli, R., Vakkilainen, S. *et al.* Immunopathological signatures in multisystem inflammatory syndrome in children and pediatric COVID-19. *Nat Med* **28**, 1050–1062 (2022).
2. Duren, Z., Chang, F., Naqing, F. *et al.* Regulatory analysis of single cell multiome gene expression and chromatin accessibility data with scREG. *Genome Biol* **23**, 114 (2022).

## P432 New heterozygous variant of socs1 associated with early-onset multiple autoimmunity and ALPS-like syndrome

### S. Palmeri^1,2^, I. Prigione^2^, F. Schena^2^, M. Miano^3^, R. Caorsi^2^, S. Volpi^1,2^, M. Gattorno^2^

#### ^1^DINOGMI, University of Genova; ^2^Centro Malattie Autoinfiammatorie e Immunodeficienze, IRCCS Istituto G. Gaslini; ^3^UO Ematologia, IRCCS Istituto G. Gaslini, Genova, Italy

##### **Correspondence:** S. Palmeri

*Pediatric Rheumatology 2023*, **21(Suppl 2):**P432

**Introduction:** We report a case of early onset polyautoimmunity associated with a novel heterozygous SOCS1 gene variant. Other cases of multiple autoimmunity have recently been associated with SOCS1 gene mutation and subsequent lack of downregulation of the intracellular cytokine signal, due to a lack of inhibition of the JAK-STAT pathway. (1)

Case report: female patient born in 2009, evaluated for the first time in our hospital in 2020. The clinical history is characterized by recurrent fevers starting at three years of age, associated with multiple autoimmune diseases, including celiac disease, diabetes mellitus, gastritis erosive autoimmune, autoimmune thyroiditis, arthritis, limbic encephalitis due to anti-GAD antibodies. The patient was initially classified as ALPS-like phenotype, also supported by the increase of double negative alpha/beta lymphocytes, the reduction of CD19+CD27+ cells, modest increase of vitamin B12 and heterozygous CASP10 mutation. After a therapeutic attempt with mycophenolate mofetil, which resulted ineffective, sirolimus was started with good control over the febrile component, then associated with methotrexate to control the arthritic symptomatology.

NGS analysis detected a heterozygous variant of the SOCS1 gene, not yet described in the literature, and inherited from her father, affected by multiple sclerosis.

**Objectives:** to validate the pathogenicity of the SOCS1 gene variant (c.208G>C) with functional laboratory tests.

**Methods:** peripheral blood samples of the patient were collected after obtaining parental informed consent. Patient and healthy donor (HD) PBMCs were isolated and were performed: i) surface and intracellular staining for FoxP3 and CD25 markers; ii) membrane staining for CD3 and CD19 and intracytoplasmic staining for pSTAT5 in the absence or in the presence of IL-2 stimulation; iii) after expansion of T blasts from the patient and HD, a proliferation assay was performed with and without IL-2 stimulation.

**Results:** Functional tests revealed: i) lower CD4+ CD25+ FoxP3+ regulatory T cells frequency compared to HD; ii) higher IL-2 induced pSTAT5 phosphorylation in CD3+ T cells of patient compared to HD; iii) increased proliferation of patient-derived T blasts in response to IL-2 stimulation in patient compared to HD.

**Conclusion:** The functional tests performed displayed a result consistent with lymphocyte hyperactivity in response to cytokine stimulus, compatible with SOCS1 haploinsufficiency. These tests, associated with further functional assays, could validate a new pathogenetic variant of SOCS1 and suggest a new therapeutic approach in the treatment of our patient (use of JAK-inhibitors).

**Patient Consent**

Yes, I received consent

**Disclosure of Interest**

None declared

**References**

1. Hadjadj J, Castro CN, Tusseau M, et al. Early-onset autoimmunity associated with SOCS1 haploinsufficiency. Nat Commun. 2020;11(1):5341. 2020 Oct 21.

## P433 Arthralgia and nutritional deficiency

### S. Jiménez^1^, R. E. Vela^1^, M. M. Corolla^2^, L. G. Urbina^3^, V. Cavazos^3^, L. C. Jimenez^3^

#### ^1^Pediatric Rheumatology; ^2^Pediatric Hematology; ^3^Pediatrics, Instituto Mexicano Del Seguro Social, Monterrey, Mexico

##### **Correspondence:** S. Jiménez

*Pediatric Rheumatology 2023*, **21(Suppl 2):**P433

**Introduction:** Scurvy, produced by de deficiency of vitamin C, due to insufficient intake, this condition has been associated with specific conditions such as developmental delay, autism and malnourish. Patients develop symptoms like fatigue, gastrointestinal and neurological disorders. Musculoskeletal manifestations appear in 80% of patients, predominantly in lower limbs with arthralgias, myalgias, frog leg position and muscular hemorrhages.

According to National Health and Nutrition survey in Mexico the prevalence of micronutrients deficiency is frequent, 30% of children with vitamin C deficiency.

**Objectives:** Present a case report of scurvy in a patient with arthralgias.

**Methods:** 4-year-old male was admitted to the pediatric department, with history of 3 months of joint pain. He had a history of autism spectrum and picky eater, and family history of autoimmunity with arthritis and hypothyroidism. Arthralgias were describe as bilateral knee pain after period of rest, interrupts sleep 2 nights a week, decreased weight bearing, and IV Steinbrocker functional classification. Upon physical examination the patient’s had low weight for age, pale, pull hair test positive, poor dental hygiene, gingivitis, cervical and inguinal lymphadenopathies, hips in flexion with limited extension, knees in flexion with tenderness but no synovial thickening. Laboratories Hgb 8.3 g/dL, MCV 75.9 fL, MCH 23.3pg, platelets 264.2 Leukocytes 4806 Lymphocytes 2000, Neutrophils 2300, ESR de 6 mm/h, CPR de 0.8, ferritine 21.76 ng/ml, LDH 192 U/L, Rheumatoid factor negative, antinuclear antibodies negative, infectious disease where rule out. Bone marrow aspirate was performed with cytometry for leukemia and lymphoma, the result came negative. Due to lack of clinical findings of arthritis and studies negative for malignancy, a vitamin deficiency was considered, taking serological determinations of vitamin C and D. Empiric treatment was initiated with reposition of vitamin C.

**Results:** Within three days of treatment the pain and symptoms improved. A month later the result was vitamin D 35.6 ng/ml (normal) and Vitamin C with -0.1 mg/dL, confirming the diagnosis of scurvy.

**Conclusion:** Scurvy, despite being a disease with small incidence, should be considered in patients with joint pain like inflammatory arthritis, without evidence of arthritis and negative acute reactants, considering nutritional context.

According to the National Health and Nutrition survey in Mexico, the diet of a large proportion of Mexicans is deficient in micronutrients that are key to the health and development of children and adolescents, thus affecting their growth and development, this defiency can have musculoskeletal manifestations that can mimmick malignancies and autoimmune diseases, therefore the importance of adecuate anamnesis. Although currently the incidence of scurvy is rare, it is an important differential diagnosis in patients with risk factors such as place of residence, dietary habits, and comorbidities like developmental disorders, and autism.

**Patient Consent**

Yes, I received consent

**Disclosure of Interest**

None declared

**References**

1. Perkins A, Sontheimer C, Otjen JP, Shenoi S. Scurvy Masquerading as Juvenile Idiopathic Arthritis or Vasculitis with Elevated Inflammatory Markers: A Case Series. J Pediatr. 2020 Mar;218:234-237.e2. doi: 10.1016/j.jpeds.2019.10.059. Epub 2019 Dec 13. PMID: 31843213.
2. García OP, Ronquillo D, del Carmen Caamaño M, Martínez G, Camacho M, López V, Rosado JL. Zinc, iron and vitamins A, C and e are associated with obesity, inflammation, lipid profile and insulin resistance in Mexican school-aged children. Nutrients. 2013 Dec 10;5(12):5012-30. doi: 10.3390/nu5125012. PMID: 24335710; PMCID: PMC3875915.
3. Jain DS, Agrawal T, Malviya PK. Scurvy Masquerading as Septic Arthritis in a Case of Cerebral Palsy. J Orthop Case Rep. 2021 Aug;11(8):107-110. doi: 10.13107/jocr.2021.v11.i08.2388. PMID: 35004388; PMCID: PMC8686502

## P434 The potential role of TIM3/GAL9 pathway in primary Raynaud’s diseases

### O. Boluk^1^, E. Yaglikara^1^, Y. Bayindir^2^, Y. Bilginer^2^, S. Ozen^2,3^, E. Sag^1,3^

#### ^1^Pediatric Rheumatology, Ankara Training and Research Hospital; ^2^Pediatric Rheumatology; ^3^Translational Medicine Laboratory, Pediatric Rheumatology Unit, Hacettepe University, Ankara, Türkiye

##### **Correspondence:** O. Boluk

*Pediatric Rheumatology 2023*, **21(Suppl 2):**P434

**Introduction:** Primary Raynaud’s disease (RD) is an episodic, reversible vasospasm of the peripheral arteries (usually digital) causing pallor, followed by cyanosis and/or redness, often with pain and paresthesia, occurring in the absence of an underlying disease. Inhibition of checkpoint proteins are the novel treatment strategies for cancer treatment, in which some patients developed Raynaud’s like phenomenon as a side effect.

**Objectives:** In this study we report the role of checkpoint proteins in the pathogenesis of primary Raynaud’s disease.

**Methods:** We included 26 consecutive primary RD patients admitted to our clinic and as for the control group age and sex matched patients with secondary RD due to SLE (n=4), SLE patients without RD (n=16) and healthy controls (HC, n=10) were included, as well. We measured the soluble plasma levels of co-IRs (IL-2, Gal-9, TIM-3, LAG-3, PD-1, PD-L1, CTLA-4, CD86 and 4-1BB) by commercial pre-defined cytometric bead array kits.

**Results:** There were no significant difference between groups in terms of age (RD 14.1±1.6 yrs; SLE 14.8±2.7 yrs; HC 14.1±2.7yrs p=0.52) and sex (RD 65.4% male; SLE 60% male; HC 60% male p=0.426). Vitamin D, ESR and CRP levels, WBC and platelet counts were comparable between groups however hemoglobin, C3, C4 levels were lower in SLE group as expected. In RD group, TIM-3 (2304.4±22505.2 pg/mL vs 1359.8±667.7 pg/mL; p=0.04) and Gal-9 (17802.1±7991.3 pg/mL vs 10439.2±4784.4 pg/mL; p=0.009) levels were significantly higher than healthy controls. Gal-9 levels were the highest in SLE group (25356.2±13624 pg/mL). The levels of nearly all of the checkpoint proteins were higher in SLE patients, however only Gal-9 achieved statistical significance. In RD patients the levels of all checkpoint inhibitors were significantly correlated with each other. Furthermore, TIM-3 levels were negatively correlated with vitamin D levels (r=-0.48, p=0.04), PD-1 levels were negatively correlated with hemoglobin (r=-0.43, p=0.03) and Gal-9 levels were negatively correlated with platelet counts (r=-0.4, p=0.035). Any of the checkpoint protein levels were not correlated with Raynaud Condition Score.

**Conclusion:** This is the first study evaluating the role of different checkpoint proteins in Raynaud’s Disease. Gal-9 is an important marker for IFN type-I pathway and its levels have been shown to be associated with SLE disease activity, SLEDAI.^1^ Gal-9 levels of our RD patients were in between SLE patients and HCs. Tim-3 levels were higher than HC and they were negatively correlated with vitamin D levels. There are studies reporting that low levels of vitamin D may cause Raynaud disease and resolves after replacement treatment.^2^ Gal-9 is a ligand for TIM-3. Since the interaction of them and the balance in TIM3/Gal-9 pathway is important in both cancer and autoimmunity, TIM-3 and Gal-9 might have a role in Raynaud’s disease pathogenesis.

**Patient Consent**

Not applicable (there are no patient data)

**Disclosure of Interest**

None declared

**References**

1. Yuksel K, Sag E, Demir S, Özdel S, Kaya UA, Atalay E, Cuceoglu MK, Topaloglu R, Bilginer Y, Ozen S. Plasma checkpoint protein levels and galectin-9 in juvenile systemic lupus erythematosus. Lupus. 2021 May;30(6):998-1004.
2. Hélou J, Moutran R, Maatouk I, Haddad F. Raynaud's phenomenon and vitamin D. Rheumatol Int. 2013 Mar;33(3):751-5.

## P435 Distribution of cells in the normal synovium during the postnatal period

### A. Fedotchenko^1,2^

#### ^1^Asklepios Klinik Altona , Hamburg, Germany; ^2^Zaporizhzhia State Medical and Pharmaceutical University, Zaporizhzhia, Ukraine

##### **Correspondence:** A. Fedotchenko

*Pediatric Rheumatology 2023*, **21(Suppl 2):**P435

**Introduction:** The synovium is an important component of the tissues that form the synovial joint and is essential for its normal functioning. Synovial cells are involved in several processes aimed to maintain homeostasis of the synovium. It is known that cells of the inflamed or damaged synovium play a pivotal role in the initiation of an array of joint disorders. It seems necessary to study changes in their distribution in the normal synovium and compare them with that seen in the pathology. Despite numerous literature data on the cellular composition of the synovium, it is extremely difficult to trace the patterns of distribution of synovial cells in the postnatal period in the human body. Laboratory rats are commonly used as the model of choice for such studies.

**Objectives:** Cells distribution (fibroblasts, fibrocytes, mast cells, macrophages and lymphocytes) in the normal synovial subintima from birth till the 90th day was studied using the hip joints of Wistar rats.

**Methods:** Sampling was carried out on the first, seventh, 14th, 30th, 45th, 60th and 90th day (six rats in each group, 42 in total). The formalin-fixed paraffin-embedded tissue sections were stained with hematoxylin and eosin, alcian blue (0.2 M MgCl2). The light microscopy, morphometrical and statistical (p<0.05) methods with subsequent extrapolation to humans [1] were used.

**Results:** The maximum number of young fibroblasts was observed on the first day (37.7±1.99). It decreased till the 14th day (early infancy of human life) practically fourfold (11.0±1.72), followed by increases on the 30th (14.0±1.53; approximately 9 human years) and 90th days (11.11±1.45; approximately 18-19 human years). The maximum quantity of mature fibroblasts was on the seventh day (early neonatal period of human life). Subsequently, it gradually decreased. On the 90th day, it was almost two times less compared to the seventh day (50.1±2.93 vs 110.2±3.31, p<0.01). The maximum number of fibrocytes was on the seventh (22.22±1.31), 30th (23.48±2.16) and 90th days (30.1±2.03). The figure on the 90th day exceeded that of newborns almost three times (30.1±2.03 vs 9.3±1.31, p<0.01). The quantity of mast cells increased till the 30th day (6.55±0.12 vs 4.2±0.17 on the first day, p<0.01) with a subsequent gradual decrease (6.55±0.12 vs 3.3±0.05 on the 90th day, p<0.01). The maximum number of macrophages was on the seventh day (6.56±0.41 vs 5.05±0.31 on the first day, p=0.017). It decreased subsequently till the 45th day (6.56±0.41 vs 2.96±0.23, p<0.01) and further did not vary significantly (2.96±0.23 vs 2.79±0.18 vs 2.88±0.2 on the 45th, 60th and 90th days respectively, p>0.05). The number of lymphocytes increased till the 30th day (6.69±0.29 vs 2.78±0.07 on the first day, p<0.01) with a subsequent gradual decrease (6.69±0.29 vs 4.8±0.17 on the 90th day, p<0.01).

**Conclusion:** The distribution of cells in the synovial subintima has mainly an undulatory pattern, reflecting the complex cell-cell and cell-matrix relationship within the joint capsule. Understanding the normal synovium's morphogenetic patterns will help better understand its pathological changes.

**Patient Consent**

Not applicable (there are no patient data)

**Disclosure of Interest**

A. Fedotchenko Employee with: no conflict of interest

**References**

1. Sengupta P. The Laboratory Rat: Relating Its Age With Human's. Int J Prev Med. 2013 Jun;4(6):624-30.

## P436 The distribution of mannose-binding lectins in the normal articular cartilage of the hip joint during the postnatal period

### A. Fedotchenko^1,2^

#### ^1^Asklepios Klinik Altona, Hamburg, Germany; ^2^Zaporizhzhia State Medical and Pharmaceutical University, Zaporizhzhia, Ukraine

##### **Correspondence:** A. Fedotchenko

*Pediatric Rheumatology 2023*, **21(Suppl 2):**P436

**Introduction:** The mannose-binding lectin (MBL) is an important element of the innate immune system. MBL deficiency is associated with a high risk of the development of rheumatoid arthritis and its poor prognosis.

**Objectives:** The distribution of MBLs in the normal articular cartilage during the postnatal period was studied using the hip joint of Wistar rats.

**Methods:** Sampling was carried out on the first, seventh, 14th, 30th, 45th, 60th, and 90th days (n=6). The joints were fixed, decalcified, dehydrated, embedded in paraffin, stained with mannose-specific lectins conjugated with horseradish peroxidase (HRP): lens culinaris (LCA-HRP) and vicia sativa (VSA-HRP), analyzed by light microscopy. The intensity of staining was evaluated by a semi-quantitative assessment. There were no substantial differences in the distribution of the lectins between the femoral head and the acetabulum as well as the degree of expression between the studied lectins.

**Results:** The most superficial zone of the articular cartilage could be seen as a cells-matrix layer. It was clearly separated from the adjacent tangential zone by a continuous lamina with a strong expression of lectins (from +++ to ++++) which didn't change significantly. The nuclei of chondrocytes expressed different staining (from weak (+) to strong (+++)). The articular cartilage showed a moderate (++) reaction on the first day. The marginal cartilage showed weak (+) staining till the seventh day. On the seventh day, the tangential and middle zones were weakly marked (+); the deep zone was moderately (++) stained. On the 14th day, the tangential zone, upper layers of the middle zone and the marginal cartilage expressed almost no lectin-binding sites. The deeper layers of the middle zone and basal zone showed a weak reaction (+). Subsequently, a strong (+++) lectins affinity was found in the tangential zone (30th-45th day), marginal cartilage (30th-90th day), capsule of the lacuna of chondrocytes (30th-90th day), deep zone, and metaphyseal growth cartilage, especially in its proliferative zone (60th-90th day). A weak reaction showed the middle zone (from the 30th day) and the tangential zone (from the 60th day). The lectin affinity of the subchondral bone, emerging in the middle zone, was similar to the surrounding cartilage. The resting zone of the metaphyseal cartilage had no lectin affinity.

**Conclusion:** The distribution of MBLs in the normal articular cartilage changes dynamically throughout the entire lifespan, reflecting the morphogenesis of its structural components. The obtained data are proposed as a pattern for the evaluation of pathologies or premorbid conditions of the joint.

**Patient Consent**

Not applicable (there are no patient data)

**Disclosure of Interest**

A. Fedotchenko Employee with: no conflict of interest

## P437 Immunoadsorption as effective treatment of pediatric autoimmune disease-related catatonia, is-it associated to increased cerebro spinal fluid interferon alpha?

### A. Felix^1,2^, V. Bondet^3^, D. Duffy^3^, A. NTORKOU^4^, T. Kwon^5^, Y. Crow^6^, P. Ellul^7^, I. Melki^1^

#### ^1^Reference Center for Rheumatologic and Autoimmune Diseases, Robert Debré University Hospital, Paris, France; ^2^Reference Center for Rheumatologic and Autoimmune Diseases, Martinique University hospital, Fort-De-France, Martinique; ^3^Institut Pasteur; ^4^Radiology; ^5^Nephrology, Robert Debré University Hospital; ^6^Institut Imagine; ^7^Child Psychiatry, Robert Debré University Hospital, Paris, France

##### **Correspondence:** A. Felix

*Pediatric Rheumatology 2023*, **21(Suppl 2):**P437

**Introduction:** Catatonia is an underdiagnosed neuropsychiatric syndrome due to inflammatory brain disorders in more than 50% of patients in acute medical department.

**Objectives:** However, the pathophysiology underlying catatonia, as well as optimal treatments, is still to be determined.

**Methods:** Retrospective study between 2017 and 2022 in a tertiary centre for Rheumatic, Autoimmune and Systemic diseases in children in France. Patients were evaluated by a multidisciplinary team including a psychologist, paediatric psychiatrist, neurologist, and rheumatologist. Cognitive tests, Montreal Cognitive Assessment scale (MoCA), blood and CSF assessments and radiological investigations were performed. Type I interferon (IFN I) pathway was assessed for every patient (IFN activity, IFN signature and Simoa).

**Results:** We identified five paediatric catatonic patients diagnosed with juvenile systemic lupus erythematosus (n=3), type 1 interferonopathy (Aicardi-Goutières Syndrome, n=1) and juvenile dermatomyositis (n=1) with median follow-up of 2 years. They suffered mostly from agitated catatonia. Four out of five were resistant to steroid and cyclophosphamide pulses and benefitted from immuno-adsorption (IA) sessions. Their MoCA scales were significantly low at onset (median 8/30, normal ^3^ 26), their brain MRI mostly showed aspecific white matter hypersignals, and lumbar puncture were normal to subnormal (isolated hyperproteinorachia 0.3-1.1g/l). All of them had high levels of circulating IFN and highly positive IFN Simoa and assay in the cerebrospinal fluid (CSF). They all had increased neopterin and decreased biopterin in the CSF. The median number of IA sessions was 22 (20-25) over 5-8 weeks and was related to dramatic improvement without sequalae and fast clearance of blood and CSF IFN.

**Conclusion:** We report a series of five patients, affected with three different types of paediatric autoimmune / autoinflammatory diseases biologically mediated by IFN I, who benefited from IA sessions. Our study provides further evidence for the immuno-physiopathology of catatonia. The effectiveness of IA should be explored in further studies.

**Patient Consent**

Yes, I received consent

**Disclosure of Interest**

None declared

## P439 A new actinopathy associated with hemizygot mutation in FLNA gene

### S. Erdem^1^, A. Paç Kısaarslan^1,2^, M. E. Doğan^3^, S. Özdemir Çiçek^1,2^, A. Özcan^4^, S. Haskoloğlu^5^, F. Doğu^5^, K. A. İkincioğulları^5^, E. Ünal^1,4^, A. Eken^1^

#### ^1^GENKÖK Genom and Stem Cell Center; ^2^Pediatric Rheumatology, Erciyes University; ^3^Genetics, Kayseri City Hospital; ^4^Pediatric Heamatology and Oncology, Erciyes University, Kayseri; ^5^Pediatric Immunology, Ankara University, Ankara, Türkiye

##### **Correspondence:** A. Paç Kısaarslan

*Pediatric Rheumatology 2023*, **21(Suppl 2):**P439

**Introduction:** Filamin A, encoded by its gene *FLNA* on X chromosome, is a cytoskeletal protein which binds actin filaments in orthogonal networks and makes the junction of cytoskeleton and membrane proteins. There are no studies examining the behavior of immune cells in a patient with *FLNA* mutation in real life.

**Objectives:** The molecular changes were characterized in T and NK cells caused by a *FLNA* mutation in a patient for the first time.

**Methods:** T and NK cells of the patient who had a c.7405C>T(p.Pro2469Ser) hemizygous mutation in the FLNA gene detected with whole-exome sequencing analysis were investigated by flow cytometric and molecular biology techniques.

**Results:** The patient’s complaints started at 14th month after birth with CMV colitis and recurrent pneumonia. After 2 years of age, recurrent fevers were accompanied by cervical lymphadenitis and oral aphthae. The patient had persistently elevated acute phase rectans throughout this period. Sistemic steroid and IL-1 blocker were initiated because of autoinflammation.

Treg cell frequency and FOXP3 expression (MFI) data from ex vivo differentiation test from naive T cells and the counts in the peripheral blood revealed statistically significant reduction. Additionally, CD25, CD69 surface expression decreased dramatically in T cells cultured in the presence of IL-15. After culture with PHA, both CD4+ and CD8+ T cell-derived IFN-γ, TNFα, IL-2 productions decreased. CD4+ and CD8+ cells were more likely to undergo apoptosis in culture, regardless of CD3/CD28 stimulation. Very high expression of BAX and BCL2 expression in CD8+ T cells, and decreased BCL2 expression was observed in CD4+ T cells compared to control. The basal pSTAT5 levels of the patient's PBMCs were higher than the controls, but showed significantly reduced STAT5 phosphorylation after IL-2 stimulation. After CD3/CD28 stimulation, sligthyly decreased p38, pZAP70, pLCK were observed in patient T cells. Similarly, the patient's NK cells were more apoptotic than the control, even with the addition of IL-15, and decreased BCL2 levels were observed. The cytotoxicity of the patient's NK cells against K562 target cells was reduced, the amount of GznB produced by NK cells cultured with K562s decreased. Finally, in vitro transwell migration of patient PBMCs was uneventful, although patients peripheral blood leucocytes were always high.

**Conclusion:** Collectively, these data indicate that the *FLNA* c.7405C>T (p.Pro2469Ser) mutation may be pathogenic, and that this mutation may cause combined immunodeficiency via Treg, CD3 cell signaling and NK cell function, also autoinflammation through an unidentified mechanism.

**Patient Consent**

Yes, I received consent

**Disclosure of Interest**

None declared

## P440 Respiratory involvement in juvenile idiopathic inflammatory myopathy: a systematic review

### S. Abu Rumeileh, E. Marrani, V. Maniscalco, I. Maccora, I. Pagnini, M. V. Mastrolia, G. Simonini

#### Pediatric Rheumatology, AOU Meyer IRCCS, Firenze, Italy

##### **Correspondence:** S. Abu Rumeileh

*Pediatric Rheumatology 2023*, **21(Suppl 2):**P440

**Introduction:** Juvenile idiopathic inflammatory myopathies (JIIM) are a group of connective tissue diseases characterized by muscle inflammation and variable systemic involvement including interstitial lung disease (ILD)(1,2). Available data on JIIM-associated ILD is very limited.

**Objectives:** This review aims to identify clinical, laboratory, and radiological features and outcomes of patients with JIIM-associated ILD.

**Methods:** A systematic literature review was performed in accordance with Preferred Reporting Items for Systematic Reviews and Meta-Analyses (PRISMA) statement. The electronic bibliographic databases MEDLINE via PubMed and EMBASE were used with the following inclusion criteria: studies including patients with a JIIM diagnosis made before 18 years of age and reporting a radiological or histological diagnosis of ILD.

**Results:** Of the 1916 retrieved publications, 37 papers were identified for a total of 86 patients. Of these, 76.7% had JDM, 10.4% amyopathic JDM, 8.1% anti-synthetase syndrome, 3.4% overlap syndrome, and 1.16% juvenile polymyositis. 62.8% of patients were female and most (55.8%) were from East Asia. Lung involvement was reported at a median age of 10 years old (IQR 7-14.4), with a median interval between myositis and ILD diagnosis of 5 weeks (IQR 0.25-22). Anti-melanoma differentiation-associated gene 5 (MDA-5/CADM-140) was the most frequently reported (30.2%,) myositis-specific antibody. Ninety-three % of patients showed skin involvement, 84.9% muscular involvement, and 41.9% reported fever. At diagnosis of ILD, respiratory symptoms were reported in 54.6%. 25.6 % of patients were admitted to the intensive care unit (ICU); 30.2% of patients died, and all deaths were due to ILD, with a median interval of 2 months (IQR 1.5-4.7) between the onset of respiratory symptoms and death. Patients with rapidly progressive (RP) lung disease (33.7%) were more likely to be MDA5/CADM140 positive (ρ.387, p 0.001), middle Eastern/north African (ρ.525, p 0.006), and far East Asian (ρ.412, p 0.003), to have fever (ρ.501, p<0.001), to be admitted in ICU (ρ.601, p <0.001) and to die (ρ.690, p <0.001). While patients with an acute/subacute pattern (11.6%) were more likely to have an overlap syndrome (ρ.313, p 0.011), to be Caucasian (ρ.409, p 0.038) or black (ρ.431, p 0.032), to show a higher level of aldolase (ρ.828, p 0.042), to have a general improvement of symptoms (ρ.289, p 0.033) and they were less likely to die because of ILD (ρ-650.313, p <0.001).

**Conclusion:** ILD is a rare but severe manifestation among the spectrum of visceral involvement in JIIM, with a high rate of ICU admission and mortality. Early recognition and aggressive treatment are needed to prevent a severe outcome. East Asian patients are at higher risk of developing ILD. MDA-5/CADM-14 is certainly related to ILD and RP-ILD. Patients admitted to ICU are more frequently Asiatic male patients, presenting with fever, respiratory symptoms, a rapidly progressive pattern, a progression of radiologic features, and a higher blood level of KL-6.

**Patient Consent**

Not applicable (there are no patient data)

**Disclosure of Interest**

None declared

**References**

1. Sanner H, Aaløkken TM, Gran JT, Sjaastad I, Johansen B, Flatø B. Pulmonary outcome in juvenile dermatomyositis: a case-control study. Ann Rheum Dis. 2011;70(1):86-91.
2. Robinson AB, Reed AM. Clinical features, pathogenesis and treatment of juvenile and adult dermatomyositis. Nat Rev Rheumatol. 2011;7(11):664-675.

## P441 Catching up with calcinosis: analysis of clinical characteristics, myositis specific antibodies and response to therapy in juvenile dermatomyositis in a cohort from a tertiary care centre in North India

### R. Aggarwal, A. Sil, A. Dod, R. K. Pilania, M. Dhaliwal, S. Sharma, A. K. Jindal, D. Suri, A. Rawat, P. Vignesh, S. Singh

#### Pediatrics, Advanced Pediatric Center, Post Graduate Institute of Medical Education and Research, Chandigarh, India

##### **Correspondence:** R. Aggarwal

*Pediatric Rheumatology 2023*, **21(Suppl 2):**P441

**Introduction:** Juvenile dermatomyositis (JDM) is the most common type of inflammatory myopathy seen in children with dystrophic calcification being a classic, sometimes unpredictable and difficult to treat complication.

**Objectives:** To report and compare demography, clinical features, myositis specific antibody profile, therapy and response in patients of JDM with and without calcinosis. To analyse the risk factors for development of extensive calcinosis, and categories of drugs used for improvement in calcinosis.

**Methods:** Retrospective data were collected from clinic files of patients with JDM registered at the Pediatric Rheumatology Clinic at PGIMER, Chandigarh during the period of January 1992 to April 2023. Diagnosis was made based on Modified Bohn and Peter criteria. Severity of calcinosis was defined as mild (single site, single subcutaneous nodule), moderate (more than 1 site, discrete subcutaneous nodules) and extensive (Calcinosis universalis, tumoral calcinosis, exoskeleton formation). Drugs used were classified into disease modifying anti-rheumatic agents, intravenous immunoglobulin, systemic steroids, biologics and drugs affecting calcium (Ca)/phosphorus (P) metabolism. Outcome was defined as reduction in calcinosis vs static/progressive calcinosis. Statistical analysis was done using the SPSS v.29 software.

**Results:** Of the 157 patients with JDM in our cohort calcinosis was noted in 42 patients (27%). Age at onset and age at diagnosis of JDM were comparable in patients with and without calcinosis (Median: 4.80 years vs 6.90 years, p = 0.07; Median: 6.60 years vs 7.40 yrs, p = 0.89). However, significant difference was found in the median delay in diagnosis and NXP-2 autoantibody positivity between patients with and without calcinosis (Median: 12 months vs 5 months, **p = 0.0001**; NXP-2 positivity: 40.9% vs 17.5%, **p = 0.04**). Lipodystrophy was seen more in the calcinosis group (39% vs 5%, **p = 0.0001**).

Mild calcinosis was noted in 10 patients (24%), moderate in 19 patients (45%) and severe in 13 patients (31%). No statistical difference was found in median delay in diagnosis of JDM between calcinosis groups (Mild, Moderate, Severe = 7, 18,12 months; p = 0.15). However, an early age of onset of JDM was noted in the severe calcinosis group (Mild, Moderate, Severe = 6, 9, 2 years; **p = 0.001**). Binary logistic regression analysis done on various groups of drugs while keeping the degree of calcinosis constant revealed that only drugs affecting Ca/P metabolism were significantly associated with improvement in calcinosis (**p = 0.035**).

**Conclusion:** Calcinosis was present in approximately 1/3^rd^ of our patients with JDM. Delay in diagnosis and NXP-2 positivity were identified as risk factors for development of calcinosis. An earlier age of onset of JDM predicted more severe calcinosis. Drugs that act on Ca/P metabolism may be more effective than anti-inflammatory drugs for reducing calcinosis.

**Patient Consent**

Yes, I received consent

**Disclosure of Interest**

None declared

## P442 Myositis specific and associated auto antibodies in juvenile dermatomyositis: clinical implications

### N. K. Bagri^1^ on behalf of Working group: Alen Joe Joseph1, Baehat Dhakal 1, Sathvik Reddy Erla1, Yogendra Singh2, Narendra Bagri2#, Ashish D Upadhyay3, Narendra K Bagri2#, SK Kabra21MBBS Students, AIIMS, New Delhi, 2Department of Pediatrics, AIIMS, New Delhi,3Clinical Research Uni

#### ^1^Pediatrics, All India Institute of Medical Sciences, New Delhi, India

##### **Correspondence:** N. K. Bagri

*Pediatric Rheumatology 2023*, **21(Suppl 2):**P442

**Introduction:** JDM is associated with various autoantibodies categorised as Myositis-Specific Autoantibodies (MSA) or Myositis-Associated Autoantibodies (MAA). The knowledge of clinical variables associated with a poor outcome and the clinical profile associated with a particular MSA may lead to a better understanding of the disease and may help in tailoring management. However, the prevalence and clinical association studies of MSAs/MAAs in different geographic and ethnic populations have shown some differences, suggesting a role for genetic and environmental factors in the genesis of MSAs. Given these differences in the prevalence of MSA/MAAs in different geographical regions, we aimed to study them in our setting and correlate them with characteristic clinical phenotypes.

**Objectives:** In this single-centre study, we aimed to characterize the profile of MSA/MAA in JDM patients and correlate them with clinical features and outcomes.

**Methods:** Forty-six children with JDM with ≥6 months’ follow-up in the Pediatric Rheumatology Clinic, Department of Pediatrics, All India Institute of Medical Sciences, New Delhi, were enrolled between February 2021 and December 2022. The clinical details (presentation, course, and outcome) were noted in a predesigned Performa. Serum samples were tested for sixteen MSA/MAA by line immunoassay [Euroimmun, Germany]. MSA/MAAs were correlated with clinical features and outcome (defined as a complete clinical response [≥6 months disease inactivity on medication] or complete remission [≥6 months inactivity off all drugs]. *Disease flare* was defined as an increase in muscle weakness or skin rash that required restarting of treatment or an increase in the dose of steroids.

**Results:** Thirty-seven (80%) subjects had at least one MSA/MAA. Eight (17.4%) had more than one antibody. Most common antibodies were anti-NXP2 (n=13, 28.3%), anti-TIF1γ (n = 10, 21.7%) and anti-MDA5 (n=8, 17.4%). No patient had anti-PL-12, anti-EJ, anti-OJ or anti-Ro 52 antibodies. Thirty-three (71.7%) patients showed a complete clinical response, among which 6 (13%) achieved complete remission. 12/33 (36.3%) had at least one disease flare after attaining a complete response. Anti-TIF1γ was associated with younger age of onset (≤ 36 months) [OR 8.0, 95%CI 1.59-40.33, p=0.012] and disease flares after attaining complete response [OR 9.0, 95%CI 1.42-57.11, p= 0.02]. Patients with anti-NXP2 had higher odds of severe muscular weakness [OR 3.68, 95% CI 0.96-14.08, p= 0.057] and truncal weakness [OR 3.94, 95%CI 1.00-15.97, p= 0.051]. One child with anti-MDA5 positivity had evidence of interstitial lung disease on HRCT on the chest. There was no association between the MSA/MAA profile and disease response or remission.

**Conclusion:** MAA/MSAs in JDM are associated with certain peculiar disease characteristics and may help in predicting the disease flare.

**Patient Consent**

Yes, I received consent

**Disclosure of Interest**

None declared

**References**

1. Brouwer R, Hengstman GJ, Vree EW, Ehrfeld H, Bozic B, Ghirardello A et al. (2001) Autoantibody profiles in the sera of European patients with myositis. Ann Rheum Dis 60:116–123
2. Hamaguchi Y, Kuwana M, Hoshino K, Hasegawa M, Kaji K, Matsushita T et al. (2011) Clinical Correlations With Dermatomyositis- Specific Autoantibodies in Adult Japanese Patients With Dermatomyositis. Arch Dermatol 142:391–398
3. Tansley, Sarah L., Stefania Simou, Gavin Shaddick, Zoe E. Betteridge, Beverley Almeida, Harsha Gunawardena, Wendy Thomson, et al. “Autoantibodies in Juvenile-Onset Myositis: Their Diagnostic Value and Associated Clinical Phenotype in a Large UK Cohort.” *Journal of Autoimmunity* 84 (November 2017): 55–64.

## P443 Cardiac study of juvenile dermatomyositis patients: deep diving in disease with speckle-tracking-echocardiography

### R. Dedeoglu^1,2^, N. Ulug^1^, A. Gunalp^3^, Y. I. Coskun^1^, E. Kilic Konte^2^, E. Aslan^2^, F. Haslak^2^, M. Yildiz^2^, S. Sahin^2^, A. Adrovic^2^, K. Barut^2^, F. Oztunc^1^, O. Kasapcopur^2^

#### ^1^Pediatric Cardiology, Istanbul University Cerrahpaşa Medical School; ^2^Pediatric Rheumatology, Istanbul University Cerrahpasa Medical School; ^3^Pediatric Rheumatology, Istanbul University Cerrahpaşa Medical School, Istanbul, Türkiye

##### **Correspondence:** R. Dedeoglu

*Pediatric Rheumatology 2023*, **21(Suppl 2):**P443

**Introduction:** Juvenile dermatomyositis (JDM) is a rheumatic disease of childhood that mainly targets skin and muscles, but may also affect internal organs, such as the heart and lungs.

**Objectives:** This study aimed to investigate subclinical left ventricle (LV) systolic dysfunction in juvenile dermatomyositis (JDM) using two-dimensional speckle-tracking echocardiography (2DST).

**Methods:** Twenty-six consecutive JDM patients without cardiac symptoms and 20 healthy volunteers were enrolled. Clinical data were collected from medical records, and echocardiograms were performed by a pediatric cardiologist who is unaware of patients’ condition. Possible associations between LV deformation impairment and disease activity were also evaluated

**Results:** Patients and control group had similar age (13.1 ± 5.3 vs.13.8 ± 4.76; p = 0.787). Mean of JDM duration was 5.9 ± 3.9 (1.5–19) years. Conventional echocardiogram revealed preserved LV ejection fraction (EF) (≥ 55%) in all individuals. In JDM, 2DST identified reduction of LV longitudinal in patients and control subjects (−23 ± 4.9 % vs. −28 ± 4.) %; p = 0.001). There was a negative correlation between LV global PLSS and the age at diagnosis (r = −0.499; p = 0.011). Additionally similar negative correlation between E/E’ ratio, a parameter which is considered to be a prognostic factor for the development of cardiovascular disease and the age at diagnosis (r = −0.469; p = 0.018). There was a negative correlation between RV global strain and age at diagnosis (r = −0.443; p = 0.024).

**Conclusion:** LV 2DST detected early systolic myocardial compromise in asymptomatic pediatric JDM patients, with preserved EF. Longitudinal strain impairment was associated with age at diagnosis, whereas right ventricular strain impairment was also associated exclusively with age at diagnosis.

**Patient Consent**

Yes, I received consent

**Disclosure of Interest**

None declared

## P444 Alteration of peripheral B cell subsets in juvenile dermatomyositis: a disease biomarker

### A. Dod, K. Sharma, P. Vignesh, S. Sharma, A. Rawat

#### Advanced Pediatrics Center, Post Graduate Institute of Medical Education and Research, Chandigarh, India

##### **Correspondence:** A. Dod

*Pediatric Rheumatology 2023*, **21(Suppl 2):**P444

**Introduction:** JDM is a rare autoimmune myositis that produces muscular weakness and a rash on the skin. B cells continue to play a role in pathogenesis by producing pathogenic antibodies known as myositis-specific autoantibodies (MSAs). However, the alterations in peripheral blood B lymphocyte homeostasis in JDM patients remain unresolved.

**Objectives:** The aim of our study was to look at phenotypic alterations in B cell subsets and how they relate to the clinical manifestations.

**Methods:** Blood was drawn from 19 JDM patients (16 On-Treatment, 3 Pre-treatment) and 10 healthy controls. Based on their surface markers, peripheral B cell subsets were categorised into non-switched memory B cells (CD19+CD27+lgD+), Class switched memory B cells (CD19+CD27+lgD-), double-negative (CD19+CD27-lgD-), naive B cells (CD19+CD27-lgD+) and transitional B cell (CD19+CD27-IgD+CD10+). We obtained the age, sex, ethnicity, disease activity score, CMAS, and MSA for the included patients. Mann Whitney testing was used to compare the above parameters between the high and normal B cell groups.

**Results:** We have studied the characteristics of 19 JDM patients (16 On-Treatment and 3 Pre-Treatment) and 10 healthy controls. The male: female ratio is 7:8. Frequencies of CD19+ B cells were increased marginally in Pre-treatment JDM patients compared with healthy controls (median value 15.1 and 13.9 respectively) and the frequency of Class switched memory B cells was decreased as compared to control (Median value On-treatment-13.5 and Control-17.8). There were no statistically significant differences in Age, DAS total, CMAS. However, patients had significantly higher double negative B cells proportion in JDM individual (p=0.032; Median Control-57, On-treatment-64.1). Patients who were not receiving treatment had elevated levels of CD19+ B cells and dual negative B cell along with reduced class-switched memory B cells and non-switched B cell compared with patients who were receiving treatment.

**Conclusion:** Our findings suggest that the homeostasis of B cell subsets is altered in JDM patients, resulting in increased CD19+ and dual negative B cells with marginally diminished class switched memory B cells.

**Patient Consent**

Yes, I received consent

**Disclosure of Interest**

None declared

## P445 A rare case of anti MDA5 dermatomyositis

### O. Gaidarji^1^, N. Revenco^1^, R. Eremciuc^1^, E. Nedealcova^1^, S. Foca^2^

#### ^1^Pediatrics, SUMPh Nicolae Testemitanu; ^2^Rheumatology, Institute of Mother and Childcare, Chisinau, Moldova, Republic of

##### **Correspondence:** R. Eremciuc

*Pediatric Rheumatology 2023*, **21(Suppl 2):**P445

**Introduction:** Anti-MDA5 dermatomyositis is an uncommon form of juvenile idiopathic inflammatory myopathies. Due to non-specific cutaneous features and mild muscular involvment it could mimic other conditions, thus causing significant diagnostic delays.

**Objectives:** To report a case of a pediatric patient with anti-DMA5 positive dermatomyositis, who was initially misdiagnosed as skin psoriasis.

**Methods:** Case presentation

**Results:** A 15-year-old adolescent was admitted to the Rheumatology department with inflammatory arthritis, extensive cutaneous lesions, hair loss, and weight loss. She was treated for psoriasis for 2 years before developing arthritis and now was referred to the rheumatologist for psoriatic arthritis.

Skin examination revealed multiple erythemo-squamous lesions on the extensor surfaces of the hands, elbows, knees (Gottron papules), and on the shin. Also, pitting scars, skin ulcerations, and cuticle overgrowth were noted. On musculoskeletal examination, there was symmetrical arthritis of the small joints and proximal muscular weakness with a CMAS 29/55. She had inflammatory anemia, normal ESR and CRP, normal CK, but slightly increased ALT - 84,8 U/L (N – 0-41 U/L), AST 213 U/L (N 0 – 40 U/L), LDH 536 U/L (120-300 U/L), CK-MB 30 U/L (N – 0-25 U/L). She was positive for antinuclear antibodies on the Hep2 cell line, with nuclear fine speckled staining (AC-4), negative for RF and anti-CCP, and positive for anti-MDA5 antibodies. Capillaroscopy revealed the presence of giant capillaries, and hemorrhages, consistent with a scleroderma capillaroscopic pattern. A diagnosis of amyopathic juvenile dermatomyositis was made according to Bohan and Peter's criteria.

Due to MDA5 positivity, which is associated with a high risk of severe interstitial lung disease, extensive respiratory workup was done. She had a restrictive pattern on spirometry, DLCO of 47% of predicted (N>80%), and normal result of high-resolution computed tomography. She was started on methylprednisolone (pulse therapy), oral prednisolone, and methotrexate with a good response.

**Conclusion:** Juvenile dermatomyositis is rare a condition in childhood, and could be a diagnostic challenge when presenting with non-specific features.

**Patient Consent**

Yes, I received consent

**Disclosure of Interest**

None declared

**References**

1. Kobayashi I, Akioka S, Kobayashi N, Iwata N, Takezaki S, Nakaseko H, Sato S, Nishida Y, Nozawa T, Yamasaki Y, Yamazaki K, Arai S, Nishino I, Mori M. Clinical practice guidance for juvenile dermatomyositis (JDM) 2018-Update. Mod Rheumatol. 2020 May;30(3):411-423. doi: 10.1080/14397595.2020.1718866. Epub 2020 Feb 3. Erratum in: Mod Rheumatol. 2020 May;30(3):607. PMID: 31955618.
2. Li D, Tansley SL. Juvenile Dermatomyositis-Clinical Phenotypes. Curr Rheumatol Rep. 2019 Dec 11;21(12):74. doi: 10.1007/s11926-019-0871-4. PMID: 31828535; PMCID: PMC6906215.
3. Nombel A, Fabien N, Coutant F. Dermatomyositis With Anti-MDA5 Antibodies: Bioclinical Features, Pathogenesis and Emerging Therapies. *Front Immunol*. 2021;12:773352. Published 2021 Oct 20. doi:10.3389/fimmu.2021.773352

## P446 Long-standing juvenile dermatomyositis: a case report in a marginalized area of Northeast Mexico

### A. K. Leos Leija^1^, A. V. Villarreal Treviño^1,2^

#### ^1^Pediatric Rheumatology, Hospital Universitario "Dr. José Eleuterio González", Monterrey; ^2^Pediatric Rheumatology, Hospital Regional Materno Infantil de Alta Especialidad, Guadalupe, Mexico

##### **Correspondence:** A. K. Leos Leija

*Pediatric Rheumatology 2023*, **21(Suppl 2):**P446

**Introduction:** According to the Childhood Arthritis and Rheumatology Research Alliance Legacy Registry (CLR), there’s a more than 1-year diagnostic delay in the diagnosis of juvenile dermatomyositis (JDM) in 14% of cases. Among the risk factors for delayed diagnosis, younger age at disease onset and a greater distance to the nearest pediatric rheumatologist have been reported.

**Objectives:** We aimed to describe the case of an 11-year-old girl with 9 years of evolution with JDM.

**Methods:** We retrospectively analyzed the case of a patient with long-standing JDM.

**Results:** An 11-year-old female from Matamoros, Mexico, was referred to the outpatient clinic of a third-level care center for 9 years of evolution with muscle weakness and dermatosis under chronic follow-up without previously established diagnosis or treatment. Past medical and family history were unremarkable. On physical examination, she was emaciated, weighing 26 kg (<3rd percentile for age), and height of 135 cm (10th percentile). Skin examination with heliotrope and malar rash, and superficial calcinosis on the left upper eyelid, elbows, and knees. Gottron's papules on metacarpophalangeal, proximal and distal interphalangeal joints. Proximal muscular weakness of the shoulder and pelvic girdle, symmetrical with limitation to functional class and need to walk assisted by a family member and use of a wheelchair, MMT8 score 68 points. On evaluation, complete blood count without changes, elevated lactate dehydrogenase (LDH) level 353 U/L and aspartate aminotransferase (AST) 45 U/L, and normal creatine phosphokinase (CPK). Normal hepatic, renal, and metabolic evaluation. Chest X-ray, echocardiogram and esophagogastroduodenal series without alterations. Magnetic resonance imaging of the pelvic limbs with atrophic changes with symmetrical and bilateral fat replacement, without alteration in signal intensity. A panel of autoimmune inflammatory myopathies was positive for TIF1gamma. Video capillaroscopic evaluation with the presence of tortuous and arborized mega capillaries and microhemorrhages. Treatment was started with methylprednisolone pulses for 3 days, switching to prednisone, methotrexate, and hydroxychloroquine.

**Conclusion:** It is important to increase awareness of the existence of rheumatic diseases in children both in training programs in medical schools and in pediatric residency programs to optimize and improve early recognition. It is essential to improve the use of technologies such as telemedicine to facilitate the assessment of these patients by certified pediatric rheumatologists in remote areas.

**Patient Consent**

Yes, I received consent

**Disclosure of Interest**

None declared

**References**

1. Neely J, et. al. Childhood Arthritis and Rheumatology Research Alliance Investigators. Access to care and diagnostic delays in juvenile dermatomyositis: Results from the Childhood Arthritis and Rheumatology Research Alliance Legacy Registry. ACR Open Rheumatol. 2021;3(5):349–54
2. Namsrai T, et al. Diagnostic delay of myositis: an integrated systematic review. Orphanet J Rare Dis. 2022;17(1):420.

## P447 Severe calcinosis in NXP2-positive juvenile dermatomyositis despite intensive anti-inflammatory treatment – a case report

### E. Maria^1^, S. Berg^2^

#### ^1^Pediatric Department, Futurum Academy of Health and Care, Region Jonkoping County, Jonkoping; ^2^Pediatric Department, Institute of Clinical Sciences, University of Gothenburg, Gothenburg, Sweden

##### **Correspondence:** E. Maria

*Pediatric Rheumatology 2023*, **21(Suppl 2):**P447

**Introduction:** An 8-year-old girl presented with muscular pain, proximal weakness and exanthema of the face. There was a moderate increase in muscle enzymes, positive ANA but no myositis antibodies. Findings in MRI were typical for juvenile dermatomyositis (JDM). The result on Childhood Myositis Assessment Scale (CMAS), was reduced, 30/52.

Treatment was initiated according to PRINTO protocol with intravenous steroid pulses followed by oral prednisolone together with subcutaneous methotrexate. Due to poor effect, intravenous immunoglobulin (IVIG) was added after 3 months. Deterioration continued with increasing stiffness and contractures in the calf muscles and also a fracture of the humerus after a minor trauma. Six months after diagnosis TNF inhibition (infliximab) was started and given for almost six months but without any obvious effect. During this period the girl again had another fracture after a minor trauma, this time in her distal femur. The rash of the face was very intense. The muscle weakness increased as shown by declining results of CMAS, 18/52 as the lowest. A new strategy was initiated with mycofenolate mofetil (MMF), rituximab and hydroxychlorokine. Around that time, one year after diagnosis, painful lumps around the elbows and torso started to appear. Calcium deposits could be visualized on x-ray. Calcinosis is reported in 20-40% of all JDM.

Due to severe gastrointestinal side effects MMF was stopped already after 2 months. Janus kinase inhibitor (JAKi) baricitinib was added for 6 months but had no obvious effect. Another JAKi, tofacitinib, was started together with takrolimus, IVIG, and prednisolone. Pamidronate (bisphosphonate) was added in order to reduce the calcinosis. Topical treatment with sodium thiosulphate has been tried twice daily for about 6 months without any effect. In addition to intensive anti-inflammatory treatment, physiotherapy was conducted to maintain muscle strength and mobility and reduce contractures.

Two years after onset, autoantibodies to the nuclear matrix protein 2 (NXP-2) were found. NXP-2 antibodies have been observed in about 25% of all JDM and are associated with a higher risk of developing calcinosis and severe muscle symptoms. A variety of treatment strategies have been suggested for calcinosis in JDM but evidence is limited.

In May 2023, four years after diagnosis, there are signs of over-all improvement with less inflammation. The rash of the face has disappeared and the muscle strength is improving with latest value of CMAS 30. The calcinosis remains a major problem with painful inflammations and ulcerations.

**Conclusion:** This case demonstrates that calcinosis remains a substantial clinical problem despite early and aggressive anti-inflammatory therapy. There is no consensus regarding specific treatment against calcinosis, but pamidronate might be worth considering.

**Patient Consent**

Yes, I received consent

**Disclosure of Interest**

E. Maria: None declared, S. Berg Speaker Bureau with: Has received a speakers fee from Sobi.

## P448 Rituximab as adjunctive therapy for childhood-onset anti-HMGCR positive necrotizing myositis

### E. Marrani^1^, M. V. Mastrolia^1^, I. Maccora^1^, I. Pagnini^1^, V. Maniscalco^1^, S. Abu Rumeileh^1^, I. Bertacca^2^, G. Simonini^1^

#### ^1^Pediatric Rheumatology; ^2^Pediatrics, AOU Meyer IRCCS, Firenze, Italy

##### **Correspondence:** E. Marrani

*Pediatric Rheumatology 2023*, **21(Suppl 2):**P448

**Introduction:** Myositis-specific antibodies (MSA) such as anti-3-hydroxy-3-methylglutaryl-coenzyme A reductase (HMGCR) have been recently associated with Immune-mediated necrotizing myopathy (IMNM) also in pediatric age. Children suffering from anti-HMGCR necrotizing myopathy often present with a chronic progressive course with poor response to standard therapy. Thus, treatment of this disease is often difficult. we report two cases of anti-HMGCR antibody-related myositis, successfully treated with Rituximab and we suggest a promising role of anti-CD20 treatment in this condition

**Results:** Case 1: A 14-year-old girl was referred due to high CPK level (8036 IU/L). Awhole-body MRI performed at onset showed 12/42 muscle group involvement, with an inflammation degree of 25/84. The autoantibody screen detected anti-nucleus antibody (ANA; titer 1:1280) and anti-HMGCR antibody positivity. The diagnosis of anti-HMGCR antibody-related myositis was established. Intravenous immunoglobulin and corticosteroid therapy were administered. Rituximab treatment was then performed with clinical, laboratory, and radiological improvement. At 7 months from onset, background therapy with Mycophenolate mofetil was started and corticosteroid therapy was discontinued.

Case 2: A 14-year-old girl already suffering from autoimmune polyendocrinopathy (type 1 diabetes mellitus and hypothyroidism), was referred for high CPK levels (2736 IU/L). Whole-body MRI performed at the presentation revealed inflammatory myositis The autoantibody screen revealed the positivity of ANA (titer 1:320) and anti-HMGCR antibody. Thus, intravenous immunoglobulin and mycophenolate mofetil were started. Due to partial clinical response and persistence of hyperCkemia, at +10 months from diagnosis, Rituximab treatment was started. Whole-body MRI documented a clear improvement, as well as CPK levels.

**Conclusion:** In pediatric age, Anti-HMGCR should be detected especially in case of myositis with an indolent course, severe presentation, or treatment refractoriness. This disease is ofter refractory to medication. We hypotized a possible role for Rituximab in such patients.

**Patient Consent**

Yes, I received consent

**Disclosure of Interest**

None declared

**References**

1. Tansley SL, Betteridge ZE, Simou S, et al. Anti-HMGCR autoantibodies in juvenile idiopathic inflammatory myopathies identify a rare but clinically important subset of patients. J Rheumatol. 2017;44(4):488-492. doi:10.3899/jrheum.160871

## P449 The use of baricitinib in treating refractory juvenile dermatomyositis: five year experience at a paediatric rheumatology tertiary centre

### A. Mohammed, M. Al Obaidi, S. Lacassagne, Y. Glackin, C. Papadopoulou

#### Rheumatology, Great Ormond Street Hospital, London, United Kingdom

##### **Correspondence:** A. Mohammed

*Pediatric Rheumatology 2023*, **21(Suppl 2):**P449

**Introduction:** Juvenile Dermatomyositis is a rare, autoimmune disease causing skin and muscle inflammation in children. In recent years our understanding of Juvenile Dermatomyositis has greatly expanded, with treatments being more targeted and morbidity therefore improving. In some cases, the disease does not respond adequately to currently available immunosuppression and is considered refractory, causing recurrent severe flares. Interferon Type 1 is known to be implicated in the pathophysiology of Juvenile Dermatomyositis. Several case reports have shown Baricitinib, a Jak STAT inhibitor, to be used in refractory disease with data suggesting good safety and efficacy.

**Objectives:** To evaluate the use of Baricitinib in treating muscle and skin disease in refractory Juvenile Dermatomyositis.

**Methods:** We retrospectively reviewed all patients with a diagnosis of refractory Juvenile Dermatomyositis who were treated with Baricitinib at Great Ormond Street Hospital for Children NHS Foundation Trust over the last five years. Nine patients were identified and data were collected retrospectively to include patient demographics, antibody profile, previous response to medication, laboratory results, skin manifestations of disease and muscle involvement. Data were collected at baseline on starting baricitinib treatment and then followed up at 3 and 6 month time intervals and at the last follow-up visit. To assess safety and overall efficacy of baricitinib, side effects of treatment and time to remission were also noted.

**Results:** Nine patients were identified (2 male, 7 female) as having refractory Juvenile Dermatomyositis and treated with Baricitinib with a median age of onset of 5 years (range 2-17 years). Two patients were found to have negative myositis specific antibodies at diagnosis, with the remaining having one or more of the following: anti TIF1-γ, anti NXP2, anti-MDA 5, anti PMScl75, anti PMScl100. Baricitinib was administered as per monogenic interferonopathies dose. Majority of patients had tried several DMARDs including methotrexate, IVIG, hydroxychloroquine, rituximab, cyclophosphamide, MMF, infliximab and adalimumab. Patients responded well to Baricitinib and showed significant improvement in both skin and muscle disease with no significant side effects being reported.

**Conclusion:** Baricitinib seems to be an effective treatment in refractory Juvenile Dermatomyositis. Clinical trials are needed to confirm the efficacy and safety of the medication.

**Patient Consent**

Not applicable (there are no patient data)

**Disclosure of Interest**

None declared

## P450 Spectrum of clinical phenotypes associated with myositis-specific autoantibodies in juvenile idiopathic inflammatory myositis: our experience from North-India

### V. Pandiarajan, A. Rawat, S. Basu, A. Dod, R. Kumrah, R. Garg, M. Dhaliwal, S. Sharma, R. Pilania, A. Jindal, D. Suri, S. Singh

#### Pediatrics, Postgraduate Institute of Medical Education and Research, Chandigarh, India

##### **Correspondence:** V. Pandiarajan

*Pediatric Rheumatology 2023*, **21(Suppl 2):**P450

**Introduction:** Idiopathic Inflammatory Myositis (IIM) are a heterogenous group of disorders with distinct clinical phenotypes associated with specific myositis-specific antibodies (MSA).

**Objectives:** To evaluate the frequency, pattern, and associations of MSA with clinical phenotypes in a large Indian cohort of juvenile idiopathic inflammatory myositis (JIIM).

**Methods:** A review of medical records of all patients diagnosed to have JIIM during the period January 1992 - April 2023 in Pediatric Rheumatology Clinic, Advanced Pediatrics Centre, Postgraduate Institute of Medical Education and Research, Chandigarh, India was done. Case records of children with JIIM who had significant positivity for MSA by myositis immunoblot were analyzed in detail.

**Results:** Of the 157 children with JIIM, MSA immunoblot was carried out in 77 patients. Positivity for myositis autoantibodies was seen in 58/77 (75.3%) cases, and, 15 of them were positive for multiple antibodies. Most common MSA was anti-NXP2 (n=21, 27%) followed by anti-MDA5 (n=11, 14.3%) and anti-TIF-gamma (n=10, 13%). Anti-Mi2, anti-Ro52, anti-PM-Scl positivity was found in 7 (9%), 8 (10.4%), and 8 (10.4%) cases, respectively. We observed 4 (5.2%) cases with positive anti-SAE antibody and all of them having cutaneous disease predating muscle disease and the myositis responded briskly to immunosuppressants. Calcinosis-predominant presentation with no clinical muscle involvement at presentation was seen in 4/21 (19%) cases with anti-NXP2 antibody group. While severe and relapsing cutaneous disease is more commonly noted in anti-TIF-gamma group, cutaneous ulcers, arthritis and interstitial lung disease (ILD) were noted at higher rates in anti-MDA5 group. However, we have not noted amyopathic form in anti-MDA5 JDM in our cohort.

**Conclusion:** Spectrum of MSAs and clinical phenotypes in our cohort varies from other reported cohorts from Eastern and Western world. Anti-NXP2 is the commonest MSA in our cohort with 19% of them presenting as calcinosis-predominant clinically amyopathic form. Anti-MDA5 subgroup had more arthritis-predominant presentation and no amyopathic form was noted in this group.

**Patient Consent**

Yes, I received consent

**Disclosure of Interest**

None declared

## P451 Understanding the scope of JDM burden in Africa: a survey of AFLAR and PAFLAR members

### J. Perfetto^1^, L. B. Lewandowski^2^, D. M. Wahezi^1^, K. Webb^3^, C. Scott^3^, A. Migowa^4^

#### ^1^Pediatric Rheumatology, The Children's Hospital at Montefiore, Bronx; ^2^Lupus Genomics and Global Health Disparities Unit, National Institute of Arthritis and Musculoskeletal and Skin Diseases, National Institutes of Health, Bethesda, United States; ^3^Paediatric Rheumatology, Red Cross War Memorial Children's Hospital, Cape Town, South Africa; ^4^Paediatric Rheumatology, Aga Khan Medical College East Africa, Nairobi, Kenya

##### **Correspondence:** J. Perfetto

*Pediatric Rheumatology 2023*, **21(Suppl 2):**P451

**Introduction:** There are few studies of juvenile dermatomyositis (JDM) in low and middle-income countries (LMIC), including Africa. Most are small single-center reports; many demonstrate higher mortality rates than those from studies in high-income countries, delays to diagnosis and treatment, and high prevalence of severe disease manifestations including cutaneous ulceration, global weakness, calcinosis, vasculitis, and pulmonary involvement. Despite this, 81% of African countries are without a pediatric rheumatologist. There is a need to study JDM in African children and increase awareness of JDM.

**Objectives:** The objectives of this survey are to understand the prevalence and severity of JDM in Africa, gauge availability and accessibility of diagnostic tools and medications, and identify challenges in care of children with JDM in Africa.

**Methods:** We administered a survey in English and French via WhatsApp groups to members of the African League of Associations for Rheumatology (AFLAR; n=233) and Paediatric Society of the African League Against Rheumatism (PAFLAR; n=130) from November 2022-January 2023. Topics included respondent specialty, number of JDM patients followed within the past 10 years, severe manifestations, available diagnostic tools and medications (with and without consideration of cost), and challenges in care.

**Results:** Forty-three (12%) providers answered the survey; 4 (9%) partially and 16 (37%) fully completed it. Most respondents were adult and/or pediatric rheumatologists (n=19; 95%). While all African regions were represented, most respondents were from Northern Africa (n=12; 60%). Respondents described 216 children with JDM seen within the last 10 years. They reported high prevalence of calcinosis and interstitial lunge disease (ILD), and higher mortality rates in Kenya (n=6; 14%) and Zambia (n=2; 29%) than the typical 1-3% mortality rates reported in studies of high-income countries. Almost half the queried diagnostics and medications (n=13; 48%) were accessible to ≤50% of respondents, mostly in Northern or Southern Africa (n=9; 69%). All respondents were interested in future collaboration.

**Conclusion:** This is the first multi-national exploration of JDM across the African continent. Our study supports existing reports describing severe manifestations and poor outcomes in African children with JDM, with prevalence of calcinosis and mortality higher than in North American and European studies. Respondents identified 216 children with JDM seen within the last 10 years, surpassing the 196 African children with JDM seen within the last 25 years reported to date, most of whom are from only 2 countries, Egypt and South Africa. This likely still under-represents true prevalence and does not account for children who are undiagnosed due to under-recognition of JDM. Differences in accessibility of diagnostics and medications by region parallel known disparities in the continent. These potential differences in JDM severity warrant further study, and inclusion of children and providers from African and other LMIC in global collaborative research is critical to ensure equitability and generalizability of research and improve outcomes.

**Patient Consent**

Not applicable (there are no patient data)

**Disclosure of Interest**

None declared

## P452 Towards the development of composite parent-centered disease activity scores for juvenile dermatomyositis

### S. Rosina^1^, A. I. Rebollo Giménez^1^, L. Tarantola^2^, R. Naddei^3^, A. Consolaro^1^, A. Pistorio^4^, A. Ravelli^4^

#### ^1^UOC Reumatologia e Malattie Autoinfiammatorie, IRCCS Istituto Giannina Gaslini; ^2^Dipartimento di Neuroscienze, Riabilitazione, Oftalmologia, Genetica e Scienze Materno-Infantili (DiNOGMI), Università degli Studi di Genova, Genoa; ^3^Dipartimento di Scienze Mediche Traslazionali, Università degli Studi di Napoli Federico II, Napoli; ^4^Direzione Scientifica, IRCCS Istituto Giannina Gaslini , Genoa, Italy

##### **Correspondence:** S. Rosina

*Pediatric Rheumatology 2023*, **21(Suppl 2):**P452

**Introduction:** Increasing attention has been recently paid to the development of parent- and child-centered composite DAS for the assessment of health status of children with rheumatic diseases.

**Objectives:** To develop and test an entirely parent-centered composite DAS for JDM, named parent Juvenile DermatoMyositis Activity Index (parJDMAI). Two versions of the score were evaluated.

**Methods:** Both parJDMAI1 and parJDMAI2 include the following items: 1) parent assessment of skin disease activity (Parent Skin Scale) on a 0-5 scale, by giving 1 point to the presence of each of: i) rash on eyelids, ii) nose/cheeks, iii) knuckles, iv) trunk/arms, v) skin ulceration; 2) parent assessment of muscle disease activity (Parent Muscle Scale) on a 0-5 scale, by giving 1 point to the presence of each of: i) fatigue/discomfort, ii) muscle weakness, iii) muscle pain, iv) voice change, v) difficulty swallowing; 3) parent assessment of child’s fatigue on a 0-10 VAS (0 =no fatigue; 10 = maximum fatigue). As fourth item, the parJDMAI1 includes the parent global assessment of child’s wellbeing on a 0-10 VAS (0 = best; 10 = worst), whereas the parJDMAI2 includes the parent global assessment of disease activity on a 0-10 VAS (0 = no activity; 10 = maximum activity). To give the 4 components of the tools the same weight, the scores of the Parent Skin and Muscle Scales were doubled. Thus, total score of both instruments ranges from 0 to 40. Initial validation was conducted on a multicentric prospective sample of 213 patients followed in standard clinical care, and on a monocentric sample including 50 patients, all assessed at baseline and 32 also assessed after a median of 3.9 months. Construct validity was assessed in both cohorts by calculating the correlations between parJDMAI1 and parJDMAI2 with: i) physician-centered JDM outcome measures; ii) the global composite DAS for JDM named JDMAI1 and JDMAI2; iii) reduced versions of JDMAI1 and JDMAI2, not including parent global assessment of child’s wellbeing. Responsiveness to change, internal consistency and discriminant ability were assessed in the monocentric sample.

**Results:** Correlations between parJDMAI1 and parJDMAI2 with physician-centered JDM outcome measures, as well as with the original and reduced versions of JDMAI1 and JDMAI2, were moderate in the multicentric sample and strong in the monocentric sample. Responsiveness to change was found to be good for both versions, while internal consistency turned out to be substantial. Both parJDMAI1 and parJDMAI2 showed satisfactory discriminant ability.

**Conclusion:** The new parent-centered composite DAS revealed satisfactory measurement properties. The proposed tools should be further tested in different clinical and cultural environments before their widespread use can be recommended.

**Patient Consent**

Not applicable (there are no patient data)

**Disclosure of Interest**

None declared

**References**

1. Rosina S, Consolaro A, van Dijkhuizen P, et al. Development and validation of a composite disease activity score for measurement of muscle and skin involvement in juvenile dermatomyositis. Rheumatology (Oxford) 2019;58(7):1196-1205.

## P453 Prevalence of pulmonary involvement and myositis-specific and myositis-associated antibodies pattern in a cohort of patients with juvenile dermatomyositis

### M. Rossano^1^, C. Portulano^2^, D. Montin^2^, F. Minoia^1^, F. Licciardi^2^, G. Filocamo^1^

#### ^1^Fondazione IRCCS Ca’ Granda Ospedale Maggiore Policlinico, Milan; ^2^Ospedale Regina Margherita, Turin, Italy

##### **Correspondence:** M. Rossano

*Pediatric Rheumatology 2023*, **21(Suppl 2):**P453

**Introduction:** Juvenile dermatomyositis (JDM) is a rare systemic vasculitis primarily affecting muscles and skin, but it can involve multiple organs, including pulmonary manifestations. Myositis-specific and myositis-associated antibodies (MSA and MAA) have been associated with different clinical patterns of JDM. In particular, anti MDA-5 positive JDM patients presented an higher risk of interstitial lung disease (ILD), which was further increased by the combined association with anti-Ro-52 antibodies.

**Objectives:** To investigate the prevalence of subclinical signs of ILD in a cohort of patients with JDM and to evaluate the clinical features and autoantibodies pattern in our cohort.

**Methods:** Patients with JDM regularly followed up in two Italian pediatric tertiary care centers were enrolled in this retrospective observational cohort study. Patients with no available data on MSA, MAA and pulmonary functioning tests, including DLCO were excluded. Descriptive analyses were reported as mean and interquartile range for continuous variables and absolute frequencies and percentages for categorical variables.

**Results:** Thirty three patients (67% female) were enrolled, with a median age at disease onset of 7.0 years (IQR 3.13-9.63) and a median follow up of 4 years (IQR 2.14 – 8.09). All patients presented skin involvement at disease onset, except one patients with anti-NXP2 Ab, and 28 (87%) had clinical and laboratory signs of myositis. Quantitative assessment of muscle strength at onset was performed in 15 (45%) patients (hybrid CMAS-MMT score) with median score of 75/100 (IQR 57/100-91/100). MSA were positive in 22 patients (70%) and MAA in 7 (22%). Anti-NXP2 were the most frequently reported Ab in our cohort (32%), foru patients were MDA5 positive (12%). In 5 patients positivity of MAA was associated with at least one MSA; 1 patient presented isolated Anti-Ro52 positivity. Among patients classified as clinical amyopathic dermatomyositis (12%), 2 were positive for anti-MDA5, 1 for anti-NPX2 and 1 for TIFI1γ.

In our cohort, 2 patients (6%) where diagnosed with ILD, without any clinical symptoms. The pulmonary involvement was evidenced with a severe reduction of DLCO and signs of restrictive syndrome, after that CT scan showed ground glass lesions in both patients. Both had combined anti-MDA5 and Ro SSa (52) positivity. No respiratory involvement was identified in the other patients.

**Conclusion:** In children with JDM, ILD represents a negative and severe prognostic factor. In light of the significant muscular impairment at onset, it can be challenging to identify clinical signs of pulmonary involvement, so testing MSA and MAA could improve the identification of patients at risk and guide the evaluation with respiratory function tests and, if necessary, with a CT. Further studies on larger cohorts of patients are needed to define the real significance of MSA patterns in disease prognosis, optimizing tailored therapeutic strategies and follow-up.

**Patient Consent**

Yes, I received consent

**Disclosure of Interest**

None declared

**References**

1. Mathiesen PR, et al (2014) Pulmonary function and autoantibodies in a long-term follow-up of juvenile dermatomyositis patients. Rheumatology (Oxford) 53:644–649

## P454 From paediatric to adult rheumatology care: how are JDM patients doing?

### P. Šeferna^1^, K. Šingelová^1^, N. Vinšová^1^, L. Lajczyková^2^, J. Vencovský^3^, P. Doležalová^1^

#### ^1^Centre for Paediatric Rheumatology and Autoinflammatory Diseases ERN-RITA, Department of Paediatrics and Inherited Metabolic Disorders, General University Hospital and 1st Faculty of Medicine, Charles University, Prague; ^2^Department of Paediatric Medicine, Medical Faculty of the Ostrava University, Ostrava; ^3^Institution of Rheumatology, ERN ReCONNET, Prague, Czech Republic

##### **Correspondence:** P. Šeferna

*Pediatric Rheumatology 2023*, **21(Suppl 2):**P454

**Introduction:** Juvenile dermatomyositis (JDM) is one of the rare paediatric rheumatic diseases within the portfolio of European Reference Network ERN-RITA for rare immunological disorders with a close link to ERN ReCONNET, which covers, among others, adult idiopathic inflammatory myopathies. As the large proportion of JDM patients in the country are seen at our centre their transition to the partner ERN institution for adult patients has been established.

**Objectives:** To evaluate patient-reported outcomes of JDM patients after their transfer.

**Methods:** Data on paediatric patients with JDM attending our clinics since the electronic hospital record system was established in 2004 were retrieved and their characteristics analysed. They were asked to complete an online questionnaire after an approval to analyse and present their anonymised data. Questions addressed following topics: 1. Logistics, pluses and minuses of the transition. 2. Current healthcare, health issues, therapies. 3. Education and vocation. 4. Socioeconomic area.

**Results:** Total of 34 JDM patients were identified, 12 currently <18 years old. In 22 adults following therapies were prescribed during the paediatric care: glucocorticoids (GC 14; 63.6%), methotrexate (MTX,18; 81.8%), cyclosporine A (CyA 12; 52%),

hydroxychloroquine (HCQ 10; 41.6%), IVIG (4; 17.9%), mycophenolate mofetil (4; 17.9%). 12 (54,5%) were transferred to the partner institution, 2 (9,1%) to regional centres, 3 (13,6%) who are just 18 remain with us, 3 (13,6%) with monocyclic course did not require transfer, and 3 (13,6%) have been lost to F/U. Disease duration until the last paediatric visit was 7.1 years (SD4.2), time from the transfer to date 6.7 yrs (SD4.8). 16 patients completed the survey (mean age 23.9 yrs, SD4.9). Transition itself was considered smooth by all. Following current health issues were reported: no problems in 7 (43.8%), skin rash, mild muscle weakness and fatigue in 3 cases (18.8%) each, heart issues in 2 (12.5%), osteoporosis, lipoatrophy, Raynaud phenomenon, neurological problems and hypertension in 1 case each (6.3%). 9 patients (56.3%) are off treatment, following numbers being on therapy with: GC (4; 25%), MTX (3;18.7%), azathioprine (2;12.5%), IVIG (2;12.5%), HCQ (2; 12.5%). Although 6 patients (37.5%) reported having their school performance affected by the disease, 2 reached master’s degree (12.5 %), 4 finished high school (25%) and 1 completed basic 9-year education only (6.3%). 9 currently study (56.3%) and 6 (37.5%) are employed, 2 (12.5%) have children.

**Conclusion:** We asked our grown-up JDM patients about their transfer to adult care, current condition and socioeconomic issues. After more than 13 years of disease majority are doing well without major health issues, all but one study or are employed. More than half are off therapy, only 25% receive GC. In order to better capture evolution of disease activity and damage we will have to harmonize relevant tools used for paediatric and adult disease.

**Acknowledgements**

Supported by the Czech Health Research Council (AZV CR) grant NU21-05-00522 and SVV260523.

**Patient Consent**

Yes, I received consent

**Disclosure of Interest**

None declared

## P455 Mortality in juvenile dermatomyositis: analysis of clinical experience of over three decades from a tertiary care centre in North India

### A. Sil, R. Aggarwal, A. Dod, R. K. Pilania, M. Dhaliwal, S. Sharma, A. K. Jindal, D. Suri, A. Rawat, P. Vignesh, S. Singh

#### Allergy Immunology Unit, Department of Pediatrics, Post Graduate Institute of Medical Education and Research, Chandigarh, India

##### **Correspondence:** A. Sil

*Pediatric Rheumatology 2023*, **21(Suppl 2):**P455

**Introduction:** Juvenile dermatomyositis (JDM), a chronic, idiopathic inflammatory myositis, has been associated with significant morbidity and mortality in children despite improvement in management protocol.

**Objectives:** To report demography, clinical features, laboratory profile and treatment in patients with JDM who had mortality and compare them with the survival group. To analyse risk factors for mortality in children with JDM.

**Methods:** Data were collated from clinic files of patients registered at the Pediatric Rheumatology clinic, Advanced Pediatrics Centre, Post Graduate Institute of Medical Education and Research, Chandigarh, India from January 1993 to April 2023. Modified Bohan and Peter criteria was used for diagnosis of JDM. Patients were divided into ‘mortality group’ and ‘survival group.’ A detailed analysis of demographic, clinical, and laboratory parameters was done in both groups to find a comparison and predict risk factors for mortality.

**Results:** There were 157 children diagnosed to have JDM over a period of 30 years and mortality was reported in 15 (9.55%) of them. Previously published mortality rate from our centre was 11.1% (7/63). Seven deaths were reported till 2013, and over the last 10 years 8 more deaths were encountered. No significant difference was noted in age at onset (Median: 7.50 vs 6.50 years), age at diagnosis (Median: 8 vs 7 years) and median delay in diagnosis (Median: 3 vs 6 months) between the mortality and survival group. The median age at death was 9 years (range: 4–21), with a male to female ratio of 1:1.1. Aspiration pneumonia was the commonest cause of death, reported in 6/15 patients (40%). Rapidly progressive interstitial lung disease (RP-ILD) (3), gastrointestinal (GI) vasculitis (3), pneumonia (2) and rituximab-induced lung injury (1), were other important causes.

Respiratory muscle weakness **(p=0.004)** and GI vasculitis **(p=0.0002)** were present in significantly greater number of patients in mortality group as compared to the survival group. The median AST, ALT and CK-NAC was significantly higher in the mortality vs survival group **(p= 0.028, 0.006, 0.006)**. Myositis specific autoantibodies (MSA) was found to be positive in 3 patients (NXP-2:1, MDA-5:1, Ku/Ro52:1) in the mortality group. Injection cyclophosphamide **(p=0.0081)** and intravenous immunoglobulin **(0.0003)** were used in significantly more number of patients in the mortality group as compared to the survival group, indicating severity of illness in the former. Binary logistic regression analysis done on all clinical features, showed that respiratory muscle weakness was significantly associated with mortality **(p=0.024).**

**Conclusion:** Despite advances in treatment, the mortality rate in JDM at our centre in North India has not changed over last 10 years. This is probably due to the increased recognition of severe and atypical forms of disease (RP-ILD) which might have been missed in the past. Aspiration pneumonia was the commonest cause of death. Respiratory muscle weakness came out to be an important predictive factor for mortality from our study.

**Patient Consent**

Yes, I received consent

**Disclosure of Interest**

None declared

## P456 Juvenile dermatomyositis : clinical characteristics, myositis specific antibody profile and disease course in a tertiary care centre in South India

### A. K. Tennelli^1^, B. Krishna^2^, A. P. Rao^3^

#### ^1^Paediatric Rheumatology, Manipal Hospital; ^2^Paediatric; ^3^Paediatric Rheumatology, Manipal, Bangalore, India

##### **Correspondence:** A. K. Tennelli

*Pediatric Rheumatology 2023*, **21(Suppl 2):**P456

**Introduction:** Juvenile dermatomyositis (JDM) is the most common inflammatory myopathy of childhood and is characterised by proximal muscle weakness and pathognomic skin rashes. It can also involve various other organ systems with significant mortality from cardiovascular, respiratory and gastrointestinal sequelae of the disease.

**Objectives:** To study the clinical characteristics, laboratory findings, myositis specific antibody profile and disease course in patients with Juvenile dermatomyositis.

**Methods:** A prospective, observational study of 20 children with JDM fulfilling the Bohan and Peter criteria were included and observed over a period of 2 years. Data on demographic, clinical features, laboratory investigations, treatment and disease course were recorded.

**Results**

The mean age at onset of disease was 7.4 years. There were 55%(11) males and 45% (9) females. The mean duration from time of onset of symptoms to diagnosis was 4 months. All patients had skin rashes typical of JDM. Proximal muscle weakness seen in 90%(18) patients. The most common systemic manifestations being weight loss 75%(15), Arthralgia 70%(14), Arthritis 50%(10), Fever 55%(11). Other features like Gastrointestinal involvement 15%(3), Respiratory involvement 25%(5), Calcinosis 55%(11) and Lipodystrophy 30%(6) were noted. All patients presented with at least one of the four (AST,ALT,CPK,LDH) serum muscle enzymes elevated. Muscle MRI was performed in 80%(16) and was abnormal in all. Out of 20 patients tested, 35%(7) had autoantibodies with MDA5 followed by NXP2 25%(5), TIF1g 5% (1) and RO52 5% (1). All patients received Corticosteroids and Methotrexate. Other immunosuppressive drugs used in case of inadequate response were HCQ 60%(12), Cyclophosphamide 35%(7), MMF 20%(4), Tofacitinib 15%(3). 20%(4) refractory cases treated with Rituximab. A monophasic course was seen in 65%(13), polyphasic course in 25%(5) and a chronic progressive course in 10%(2) patients.

**Conclusion:** JDM should always be considered in the differential diagnosis of any child with skin rash and muscle weakness. MDA5 positivity was associated with the complication of Calcinosis and Interstitial lung disease. Early diagnosis and appropriate treatment may minimise sequelae in patients with JDM.

**Patient Consent**

Yes, I received consent

**Disclosure of Interest**

None declared

**References**

1. Saghafi, M., Rezaieyazdi, Z., & Hashemzadeh, K. (2014). *Juvenile dermatomyositis, clinical manifestations and outcome in an Iranian cohort. Egyptian Pediatric Association Gazette, 62(2), 46–51.*doi:10.1016/j.epag.2014.06.001
2. Sag E, Demir S, Bilginer Y, Talim B, Haliloglu G, Topaloglu H, Ozen S. Clinical features, muscle biopsy scores, myositis specific antibody profiles and outcome in juvenile dermatomyositis. Semin Arthritis Rheum. 2021 Feb;51(1):95-100. doi: 10.1016/j.semarthrit.2020.10.007. Epub 2020 Dec 22. PMID: 33360233.
3. Valenzuela A, Chung L, Casciola-Rosen L, Fiorentino D. Identification of clinical features and autoantibodies associated with calcinosis in dermatomyositis. JAMA Dermatol 2014;150(7):724–9.

## P457 Infections in juvenile dermatomyositis: a cohort from a tertiary care centre in North India

### R. Tyagi, R. Aggarwal, S. Basu, A. Dod, S. Sharma, M. Dhaliwal, R. K. Pilania, A. K. Jindal, A. Rawat, D. Suri, P. Vignesh, S. Singh

#### Pediatrics, Postgraduate Institute of Medical Education and Research, Chandigarh, India

##### **Correspondence:** R. Tyagi

*Pediatric Rheumatology 2023*, **21(Suppl 2):**P457

**Introduction:** Juvenile dermatomyositis (JDM) is the most common type of inflammatory myopathy seen in children. There is paucity of data describing infections in these patients. This is the first retrospective study analysing the incidence of infections in patients with JDM.

**Objectives:** To report and compare various demographic details and disease symptoms in patients of JDM with and without infections and to analyse the risk factors for development of infections.

**Methods:** Retrospective data were collected from clinic files of patients with JDM registered at the Pediatric Rheumatology Clinic at PGIMER, Chandigarh during the period of January 1992 to April 2023. Statistical analysis was done using the SPSS v.29 software.

**Results:** Out of 157 patients with JDM, we recorded infections in 31 patients (19.7%) with a total 44 episodes of infections. Out of 31 patients with infections, 27 received intravenous (IV) pulse steroids, 30 received oral steroids, 23 received methotrexate, 5 mycophenolate mofetil, 6 received IV immunoglobulin and 7 received cyclophosphamide.

Significant difference was found in the male to female ratio(M:F – 1:1.2 vs M:F – 2.1:1, p = 0.018; %,). No statistical difference was found between the age at onset and age at diagnosis in years between patients with and without infections (Median: 6.00 years vs 7.50 years, p=0.268; Median: 7.00 years vs 8.50 years, p = 0.575). Neck weakness, absent gag reflex and gastrointestinal vasculitis was significantly higher in patients with infections as compared to those without. (47.6% vs 67.7%, p=0.049; 33.3% vs 61.3%, p=0.007; 2.4% vs 16.1%, p= 0.015).

Pneumonia (3 community acquired and 3 ventilator associated) was seen in 22.6% (7/31) patients, skin infections (tinea versicolor, pyoderma, scabies, folliculitis, cellulitis, intravenous site abscess) in 58% (18/31) and viral infections (1 CMV, 1 H1N1, 1 Parvovirus, 1 Hepatitis B, 1 Dengue, 1 Herpes zoster and 1 varicella zoster) in 22.6% (7/31). Pneumonia was seen significantly more in patients with neck flexor weakness (p=0.044), skin and viral infections had no significant association with calcinosis, interstitial lung disease or use of drugs like steroids, disease modifying agents. Bacterial infections were the most common cause of infections in our cohort (18/31, 58%) out of which 14 were due to Staphylococcus aureus, 2 were Salmonella typhi, 1 was Acinetobacter baumanii and 1 was Escherichia coli. Fungal infections of the skin (Malassezia furfur, candidiasis and tinea capitis) were found in 22.6% (7/31) patients. Tuberculosis was seen in 9.6% (3/31) and scabies was seen in 9.6% (3/31) patients only. Most of these patients recovered from the infections except 2 patients who succumbed to ventilator associated pneumonia caused by Acinetobacter baumanii.

**Conclusion:** Infections although rare in patients of JDM are a cause of significant morbidity. Infections are significantly higher in patients with neck flexor weakness, absent gag reflex and GI vasculitis. Bacterial infections mainly staphylococcus aureus are the most common cause of infections in our cohort with skin being the most common organ involved.

**Patient Consent**

Yes, I received consent

**Disclosure of Interest**

None declared

## P458 Can nailfold capillaroscopy be used as a method for diagnosing and evaluating the activity of juvenile dermatomyositis?

### N. Yudkina, A. Volkov, M. Kaleda, I. Nikishina

#### V.A. Nasonova Research Institute of Rheumatology , Moscow, Russian Federation

##### **Correspondence:** I. Nikishina

*Pediatric Rheumatology 2023*, **21(Suppl 2):**P458

**Introduction:** Nailfold videocapillaroscopy (NVC) of the periunguar bed is a non-invasive, simple and affordable method of screening for systemic connective tissue diseases in adults and children. In systemic sclerosis, typical changes were made to the 2013 ACR-EULAR criteria as an important diagnostic feature. In juvenile dermatomyositis (jDM), the role of capillaroscopy in order to diagnose the disease and assess the activity of the disease is controversial, ambiguous and is still being studied.

**Objectives:** To evaluate the possibilities of videocapillaroscopy of the periunguar bed for the diagnosis and evaluation of the activity of the jDM.

**Methods:** The study included 25 patients with a reliable diagnosis jDM on the Bohan and Peter criteria for DM. All patients had skin manifestations of the disease (vasculitis of the periunguar folds, erythema/papules of the Gottron), proximal muscle weakness of varying severity, an increase in the level of creatine phosphokinase. Each patient underwent NVC using a stereomicroscope with a 20-fold magnification at the time of diagnosis of jDM, against the background of therapy with glucocorticosteroids and methotrexate after 6, 12, 24 months. The test assessed the presence of capillaroscopic changes of the periunguar bed on 2-5 fingers of both hands. The normal capillaroscopic pattern was characterized by the presence of 7-11 hairpin-shaped capillaries per 1 mm. Pathological patterns included morphological and structural changes, such as enlarged, giant and bushy capillaries, hemorrhages, avascular areas, disorganization. The pattern of capillaroscopy of changes was determined qualitatively. Decreased capillary density, dilated, giant, bush-shaped capillaries, hemorrhages were evaluated semi-quantitatively.

**Results:** Changes in the capillaries of the nailfold at the time of diagnosis were found in each of the 25 patients with jDM and were the same on all fingers, quite pronounced and bright, in most patients they were easy to detect even with the naked eye. Changes in the capillaries of the periunguar bed correlated with the presence of skin changes, but had no relationship with the severity of the muscle weakness. More severe changes (giant and bushy capillaries, avascular areas, violation of architectonics) were observed with a long duration of the disease in the absence of therapy. Against the background of ongoing treatment with glucocorticosteroids and methotrexate, after 6, 12 and 24 months, all patients achieved a decrease in the activity of the disease, which was reflected in a change in the capillaroscopic picture: the disappearance of giant capillaries, a decrease in the number of bushy capillaries, normalization of architectonics, gradual regeneration of capillaries.

**Conclusion:** NVC can be useful as an additional method of diagnosing jDM, but is not recommended as a diagnostic criterion, since the type of capillaroscopic changes is non-specific and similar to SSc, correlates only with the skin process, differs in the instability of changes.

**Patient Consent**

Not applicable (there are no patient data)

**Disclosure of Interest**

None declared

## P459 Comparison of the juvenile SLE outcomes with early and late rituximab administration

### E. Kalashnikova, R. Raupov, E. Isupova, E. Gaidar, V. Masalova, I. Chikova, K. Belozerov, L. Sorokina, M. Kaneva, O. Kalashnikova, V. Chasnyk, M. Kostik

#### Pediatric, Saint-Petersburg State Pediatric Medical University, Saint Petersburg, Russian Federation

##### **Correspondence:** M. Kostik

*Pediatric Rheumatology 2023*, **21(Suppl 2):**P459

**Introduction:** Juvenile systemic lupus erythematosus (jSLE) is the most common tissue disease in children that has serious prognosis [1,2]. Glucocorticosteroids (GCS) are considered the base treatment of SLE [3]. The toxicity of GCS and their long-term use requires steroid-sparing therapy administration [4]. There are no pediatric standardized recommendations for corticosteroid tapering and withdrawal despite the EULAR recommendations to try to minimize corticosteroid therapy [5]. Rituximab (RTX) still hasn’t been approved in jSLE treatment. Early biological therapy may improve the disease’s outcomes [2,4].

**Objectives:** to compare advantage between early rituximab administration to late.

**Methods:** In the retrospective cohort study were included 36 jSLE patients, who get early (less the 6 months from onset) RTX administration (ERA) and late (more than 1 year) RTX administration (LRA). The patient’s characteristics on the baseline (BL) - the start of RTX, after 12 months - end of study (EOS), outcomes, and adverse events of the treatment were assessed.

**Results:** The main differences at BL were higher disease activity, MAS frequency, WBC levels and higher daily dose of GCS in the ERA group to LRA. Also there were higher ANA-titer, lower C3 and C4 complement levels in ERA group with borderline significance.

At the EOS, there were significant differences in the main treatment outcomes (ANA titer, anti-ds DNA, complement C3 and C4, SLEDAI score, WBC, HGB and PLT levels) in both groups, except for the higher level of platelets in the ERA group. There was a higher frequency of patients with active LN in the ERA group (31% vs 11%), but the data were non-significant (p=0.150). There was no difference in SLE outcomes at EOS. The main benefits of the ERA were a shorter interval to achieve low daily GCS dose (≤0.2 mg/kg) - 1.2 (0.9; 1.4) years compared to 2.8 (2.3; 4.0) years (p=0.000001), higher probability to achieve this low dose (HR=57.8 [95%CI: 7.2; 463.2], p=0.00001) and achieve of the remission (SLEDAI=0); HR=37.6 [95%CI: 4.45; 333.3], p=0.00001).

There were no differences in adverse events during RTX treatment, including adverse events. About half of the patients in each group got co-trimoxasol profilaxis and the IgG level was the same, but frequently of low IgG levels and higher proportion of B-cell depletion were in ERA group.

**Conclusion:** ERA showed effectiveness in controlling jSLE activity and achievement of GCS’s low dose. Randomized controlled trials are required.

**Patient Consent**

Not applicable (there are no patient data)

**Disclosure of Interest**

None declared

**References**

1. Watson L, Beresford MW. et al. The indications, efficacy and adverse events of rituximab in a large cohort of patients with juvenile-onset SLE. Lupus. 2015 Jan;24(1):10-7.
2. Smith EMD, Lythgoe H. et al Current views on lupus in children. Curr Opin Rheumatol. 2023 Mar 1;35(2):68-81.
3. Thakral A, Klein-Gitelman MS. An Update on Treatment and Management of Pediatric Systemic Lupus Erythematosus. Rheumatol Ther. 2016 Dec;3(2):209-219.
4. Fanouriakis A, Tziolos N et al. Update οn the diagnosis and management of systemic lupus erythematosus. Ann Rheum Dis. 2021 Jan;80(1):14-25.
5. Trindade VC, Carneiro-Sampaio M. et al. An Update on the Management of Childhood-Onset Systemic Lupus Erythematosus. Paediatr Drugs. 2021 Jul;23(4):331-347.

## P460 Assessment of complementary regulatory protein CD55 and CD46 in patients with pediatric-onset systemic lupus erythematous: a preliminary study

### A. Kumar, B. Hariachen, K. Arora, A. Rawat, A. Gupta, S. Singh

#### Department of Pediatrics, Advanced Pediatrics Centre, Post Graduate Institute of Medical Education & Research, Chandigarh, Chandigarh, India

##### **Correspondence:** A. Kumar

*Pediatric Rheumatology 2023*, **21(Suppl 2):**P460

**Introduction:** Systemic lupus erythematosus (SLE) is a multifactorial autoimmune disorder characterized by production of various autoantibodies. Role of complement regulatory proteins in patients with SLE has been studied mainly in adult SLE. There is a paucity of literature in pediatric SLE (pSLE) and more so from developing countries like India. We evaluated complementary regulatory proteins CD55 and CD46 in patients with pSLE and to see correlation with disease activity.

**Methods:** This prospective observational study from the period of July 2016 -December 2017, was carried out at the Pediatric Rheumatology Clinic, Postgraduate Institute of Medical Education and Research, Chandigarh, India in patients with pSLE (onset of disease before 18 years) and fulfil the SLICC 2012 for diagnosis. Disease activity was measured by SLEDAI score.

**Results:** A total of 32 patients (24 girls; 8 boys) of pSLE were enrolled. The study group comprised of 2 groups–active (12 patients; mean age 11 years) and inactive SLE (20 patients, mean age 12 years). Median fluorescent index (MFI) of CD46 in lymphocytes, monocytes and neutrophils were higher in cases compared to healthy controls. MFI of expression on neutrophils was higher in active SLE group comparing to inactive disease. Percentage of CD55 expression on lymphocytes was reduced in case compared to controls. Percentage of CD55 expression on neutrophils was lower in active group comparing to inactive group. Percentage expression of CD55 on red blood cells was higher in SLE patients (both active and inactive disease) compared to controls. There was no significant correlation between anti double standard DNA and MFI expression on leucocytes and RBC.

**Conclusion:** There was significant association of CD46 expression on all leucocytes on pSLE cases compared to age matched healthy controls. Study showed significant association of CD55 expression on neutrophils and disease activity in pSLE patients. However, in view of small samples size results of this study need to be confirmed on larger cohort.

**Patient Consent**

Yes, I received consent

**Disclosure of Interest**

None declared

## P461 Development of a multi-disciplinary (MDT) juvenile-onset systemic lupus erythematosus (JSLE) clinic to improve standards of care and patient journey and introduce routine fitness testing

### H. Lythgoe^1^, V. Cuthbert^1^, A. Hollows^1^, A. McGovern^1^, M. Morrisroe^1^, D. Nicholson^1^, M. Sim^1^, E. Smith^2,3^, P. Riley^1^

#### ^1^Paediatric Rheumatology, Royal Manchester Children's Hospital, Manchester; ^2^Institute of Life Course & Medical Sciences (Child Health), University of Liverpool; ^3^Paediatric Rheumatology, Alder Hey Children's Hospital, Liverpool, United Kingdom

##### **Correspondence:** H. Lythgoe

*Pediatric Rheumatology 2023*, **21(Suppl 2):**P461

**Introduction:** We used national and international guidelines (including Single Hub and Access point for paedatric Rheumatology in Europe (SHARE) guidelines) and local expertise from paediatric rheumatologists, immunologists, endocrinologists and allied health professionals to describe gold standard of care for JSLE patients managed in our tertiary service. These standards cover medical assessment/management, modifiable lifestyle factors, transitional care, vaccination and functional and physical assessments with the aim of optimising long-term health outcomes. Audit data demonstrated that these standards were not being consistently met within current clinical practice.

**Objectives:** To plan and set up an annual review MDT lupus clinic designed to improve attainment of standards including structured monitoring and management, screening for comorbidities and health promotion whilst improving the patient journey through reducing the overall number of hospital visits. To identify an appropriate cardiorespiratory fitness test and evaluate delivery of it within the clinic.

**Methods:** Clinic and team capacity was assessed to identify how the clinic could be delivered using existing resources. Standardised proformas for the electronic patient record were designed. Exercise testing methods were reviewed for a simple, practical and reproducible test to be used as part of the physical assessment. Paper and online feedback questionnaires were designed to collect patient and parent/carer feedback.

**Results:** Under-utilised capacity was identified within which to deliver this clinic quarterly. Two clinics have now been successfully delivered with ten patients included. During the appointment each patient is rotated around clinician, clinical nurse specialist, occupational therapist and physiotherapist. The clinic is also supported by a research practitioner to ensure efficient data collection for existing studies and to share new research study opportunities with families. Trainees are invited to attend to support their learning regarding JSLE and conducting research. The Kasch pulse recovery step test has been introduced as a standard of care to assess fitness and to support patients to improve their physical fitness. Feedback from 7 patient/carers has been positive with families wanting to attend the JSLE clinic again, enjoying the fitness testing and most (6/7) preferring to see multiple health professionals within one appointment and only 2/7 feeling that the appointment was too long.

**Conclusion:** An MDT lupus clinic can be successfully implemented within a busy tertiary service using existing resources. The clinic and routine fitness testing were well-received by patients and families. Future work will re-audit the standards of care following implementation of the clinic, continue to review clinic processes to ensure maximum efficiency, assess the utility of fitness testing as part of routine care to improve levels of physical activity and fitness for JSLE patients and consider how the clinic can used to further develop the lupus service, for example, through delivering a treat to target approach.

**Patient Consent**

Not applicable (there are no patient data)

**Disclosure of Interest**

None declared

## P462 Lupus nephritis in childhood: the analysis of a cohort in the transitional age

### M. M. D'Alessandro^1^, G. Corsello^2,3^, B. Gramaglia^2^, G. Pavone^1^, C. Corrado^1^, M. C. Sapia^1^, R. Cusumano^1^, M. C. Maggio^2,3^

#### ^1^ARNAS, Palermo, O.U. of Paediatric Nephrology and Dialysis, Children Hospital “G. di Cristina”; ^2^University Department PROMISE “G. D’Alessandro”, University of Palermo; ^3^ARNAS, Palermo, Paediatric Clinic, Children Hospital "G. Di Cristina", Palermo, Italy

##### **Correspondence:** M. C. Maggio

*Pediatric Rheumatology 2023*, **21(Suppl 2):**P462

**Introduction:** Lupus nephritis (LN) is the onset of systemic lupus erythematosus (SLE) in 50-80% of children; otherwise, LN appears, in most patients, in the first two years of illness. Some patients have nephritis as the only manifestation of SLE. Histological diagnosis confirms renal involvement, defines the damage extent, guides treatment, based on the six classes of nephritis.

**Objectives:** The aim of our work is to compare the effectiveness of the different therapeutic schemes for LN in a single center cohort, to ensure the appropriate time for transition from the paediatric to the adult Rheumatology care.

**Methods:** A retrospective analysis of SLE patients followed in the period 2000-2023, selected 9 patients (8F;1M: age: 9-16years); mean age at the LN onset: 12 years.

**Results:** LN was the first sign of SLE in 5/9 (56%), after 0.5-2 years in 4 (44%). At the onset, 100% presented proteinuria >500 mg/24h, in 2 with microhaematuria. 6/9 (66%) had a nephrotic syndrome, 4 of whom needed haemodialysis treatment for acute renal failure. One patient presented antiphospholipid antibody syndrome with thrombotic manifestations. Four (44%) had hypertension. Positive autoantibodies (ANA, ENA, anti-dsDNA, anti-nucleosome) were detected in 8/9 (88%), hypocomplementemia in 4/9 (44%). The diagnosis of LN was confirmed by histology: 6 (67%) had class IV, 3 (33%) class V nephritis. All patients were treated with intravenous methylprednisolone boluses, followed by oral prednisone, with a slow dose tapering at the remission. Induction therapy, in patients with diffuse proliferative glomerulonephritis, was done with cyclophosphamide, in combination with steroid (1-2 mg/kg/day) in 5/6 (83%), with mycophenolate in 1. 8/9 (88%) received maintenance treatment with mycophenolate; 1 patient with cyclosporine, obtaining in most clinical improvement and normalization of proteinuria. One patient, non-responder to therapy, needed haemodialysis treatment and inclusion in the kidney transplant list. All patients with membranous glomerulonephritis received mycophenolate in induction and in maintenance, except 1, treated in induction with prednisone and azathioprine. The analysis of the follow-up data showed lower frequency of relapses with mycophenolate. The transitional care was started only for patients in remission, to a Rheumatology center with a multispecialistic team.

**Conclusion:** Our patients received a different therapeutic approach, depending on the year of the diagnosis. Our case series confirms the importance of LN histological diagnosis, to personalize therapy and improve quality of life in the perspective of transition (1). It is desirable that the patient faces the transition into a phase of remission of the disease, to a Rheumatology center with a multispecialistic team, to abate relapses risk and to improve long-term outcome.

**Patient Consent**

Yes, I received consent

**Disclosure of Interest**

None declared

**References**

1. Brunner HI, et al. American College of Rheumatology Provisional Criteria for Clinically Relevant Improvement in Children and Adolescents With Childhood-Onset Systemic Lupus Erythematosus. Arthritis Care Res (Hoboken). 2019;71(5):579-590.

## P463 Effect of immunomodulatory therapies on antiphospholipid antibodies titers in children with antiphospholipid syndrome

### P. Morán Álvarez, V. Messia, E. Marasco, F. De Benedetti, C. Bracaglia

#### Pediatric Rheumatology, Ospedale Pediatrico Bambino Gesù, Rome, Italy

##### **Correspondence:** P. Morán Álvarez

*Pediatric Rheumatology 2023*, **21(Suppl 2):**P463

**Introduction:** Pediatric Antiphospolipid Syndrome (APS) is an autoimmune disease characterized by thrombotic events (TE) associated with 2 consecutive positive determinations of antiphospholipid antibodies (aPL) ^1^. High levels of aPL are associated with increased thrombotic risk. The use of immunomodulatory therapies with the aim of reducing aPL titers may represent a treatment strategy. Therefore, monitoring of aPL levels may represent a strategy to evaluate both disease activity and response to therapies.

**Objectives:** To investigate the trend over time of aPL titers in children with APS, comparing patients under immunomodulatory therapies and those without them.

**Methods:** A descriptive, observational, cross-sectional study was carried out in children with APS. aPLs were tested from diagnosis every 3-4 months for 2 years. Interferon Gene Signature (IGS) was also assessed. Laboratory, clinical and demographic data was retrieved and analyzed.

**Results:** Sixteen children with a diagnosis of APS were included. The median age at disease onset was 11.5 years (range: 6 months – 17 years); 63% were girls; 88% Caucasians. Thirteen patients (81%) had a diagnosis of primary APS and 3 (19%) secondary APS.

Regarding clinical manifestations, 11 children developed at least one TE (7 arterial and 5 venous). Cerebral territory was the most frequently involved (5), followed by 3 deep vein thrombosis , 1 pulmonary thromboembolism and 2 arterial renal thrombosis. 13 and 8 patients received an antiaggregant and/or anticoagulant therapy, respectively. Twelve (75%) children developed at least one non-criterion manifestation, 44% of which were cardiac (Libman-Sacks endocarditis or valvular heart disease), 44% neurological (chorea or white matter lesions), 38% hematological (thrombocytopenia, hemolytic anemia or Evans syndrome) and 4% renal ( thrombotic microangiopathy).

The rates of aPLs recorded were: 25% single positivity, 42% double and 44% triple; showing the highest rates IgG aβ2GP (88%) and IgG aCL (81%). Lower rates were identified for LA (44%), IgM aCL (19%) and IgM aB2GP (13%). Four (25%) were ANA positive (3 secondary APS).

Regarding the therapy, 13 children (81%) received at least one immunomodulatory drug (13 mycophenolate; 4 rituximab) and 3 (19%) did not receive any treatment.

During the 2-year-follow-up, 11 patients (69%) showed a reduction of aPL titers, becoming 9 (56%) negative (Fig 1). Of those who were negative, 8 received an immunomodulatory therapy. Five patients (31%) showed stable titers during follow-up. Of them, 2 were not treated and 2 were under mycophenolate but with poor compliance (a reduction of titers with therapy resumption was observed).

IGS at diasease onset was also evaluated in 11 patients, resulting positive in 9 (82%).

**Conclusion:** Our data suggest the possible effect of immunomodulatory therapies in reducing antibody titers in APS. Therefore, it may represent a strategy to control the disease activity leading to a better prognosis. Further studies are needed to confirm and expand our data.

**Patient Consent**

Yes, I received consent

**Disclosure of Interest**

None declared

**References**

1. Schreiber, K., Sciascia, S., de Groot, P. et al. Antiphospholipid syndrome. Nat Rev Dis Primers 4, 17103 (2018).

## P464 Different patterns of longitudinal changes in antinuclear antibodies titres in children with systemic lupus erythematosus and other connective tissue diseases

### P. Morán Álvarez, C. Bracaglia, V. Messia, L. Giovannelli, I. Caiello, F. De Benedetti, E. Marasco

#### Ospedale Pediatrico Bambino Gesù, Rome, Italy

##### **Correspondence:** P. Morán Álvarez

*Pediatric Rheumatology 2023*, **21(Suppl 2):**P464

**Introduction:** Systemic lupus erythematosus (SLE) is an autoimmune disease characterized by the presence of antinuclear antibodies (ANA). Monitoring of anti-DNA antibody levels may reflect disease activity, by contrast a single anti-RBP antibody determination is thought to suffice for clinical purposes. Recently, evidence suggests that ANA levels in SLE and other connective tissue diseases (CTD) may decrease over time secondary to the natural history of the disease or the effect of treatments.

**Objectives:** To investigate the trend of autoantibodies titers over time, during a 2-year-follow up, in children with a diagnosis of pediatric onset SLE and primary Sjögren syndrome (SjS).

**Methods:** We enrolled 15 children with a diagnosis of SLE with disease onset under the age of 18 years, followed at Ospedale Pediatrico Bambino Gesù (italian tertiary hospital). We also enrolled 21 children with primary SjS. Antibodies monitoring was carried out from diagnosis every 3-4 months for 2 years. Interferon Gene Signature (IGS) was also assessed. ANA were defined as negative for titers <1:80. Laboratory parameters, clinical and demographic data was retrieved and analyzed.

**Results:** At 2 years of follow-up, all patients showed ANA titers lower than at time of SLE onset, p=0.0001. After 2 years of follow-up, 11 patients (73%) still showed positive ANA (group 1), while 4 patients (26%) became ANA negative (group 0). Assessing the change over time in ANA titers, the 2 groups of patients showed two different patterns: in group 0, ANA titers quickly declined and disappeared in the first 6 months after diagnosis; in group 1, ANA titers declined more slowly, remaining positive at 2-year follow-up. No statistically differences in clinical and demographic features were found between the groups. Both C3 and C4 increased in the follow-up, with no different patterns between the 2 groups, p= 0.4. Similarly, anti-dsDNA antibodies titers declined over time with no clear different patterns between the groups, p= 0.7. We analyzed the levels of IGS at last follow-up and no significant differences were found between the groups, p= 0.53.

Twenty-one children with primary SjS were included. At disease onset all patients were ANA and anti-Ro antibody positive and 14 (66%) were anti-La positive. After 2 years of follow-up, all patients remained ANA positive; however, ANA titers were significantly reduced at follow-up (Wilcoxon test, p=0.04). Titers of anti-Ro and anti-La antibodies did not change over time and no patient became negative (anti-Ro, p=0.29; anti-La,p=0.67).

**Conclusion:** Our analysis showed 2 different patterns in the reduction of ANA titers over time in children with SLE. Around 25% of patients became ANA negative after 6 months from diagnosis and remained persistently negative at 2 year-follow-up. On the other hand, all children with a diagnosis of SjS remained both ANA and ENA (anti-Ro and anti-La) positive at 2-year-follow-up. Our data showed different patterns of longitudinal changes in ANA and ENA in children with SLE and primary SjS. This fact may represent different pathogenetic pathways among CTD. At the same time, the seronegative state shown in SLE patients, may be the expression of mechanisms independent of autoantibodies role. Large cohort studies may be undergone to confirm our data.

**Patient Consent**

Yes, I received consent

**Disclosure of Interest**

None declared

## P465 Pediatric lupus with a severe atypical cardiac complication

### E. Pardo Campo^1^, S. Murias Loza^2^, J. Rodriguez Suárez^2^, S. Burger^1^, P. Gonzalez del Pozo ^1^, I. Braña Abascal ^3^, S. Alonso Castro ^3^, R. Queiro Silva ^1^, M. Alperi López^1^, A. Brandy-Garcia^4^, S. Bueno Pardo ^5^

#### ^1^Rheumatology; ^2^Pediatrics; ^3^Hospital Universitario Central De Asturias, OVIEDO; ^4^Hospital Universitario De Cabueñes , Gijon, Spain; ^5^Pediatrics, Hospital Universitario De Cabueñes, Gijon, Spain

##### **Correspondence:** E. Pardo Campo

*Pediatric Rheumatology 2023*, **21(Suppl 2):**P465

**Introduction:** 12-year-old girl, from Ecuador, with a history of hypothyroidism, who in May 2022 presents with clinical manifestations of arthralgias, amenorrhea and alopecia accompanied by erythematous-violaceous lesions on both hands and feet. The biopsy was compatible with chronic cutaneous Lupus. In the blood work we found ANA+ 1/2560 mottled pattern, antiSM+, as well as antiDNA+ 104 U/ml and hypocomplementemia C3 0.63g/L with normal urine analysis and urine quotient. In addition, positivity for lupus anticoagulant was detected. Based on this, the patient was diagnosed with systemic lupus erythematosus. Further studies were carried out, normal echocardiogram and a capillaroscopy with megacapillaries. In addition, treatment was initiated with methotrexate and hydroxychloroquine.

**Objectives:** DESCRIPTION OF THE CASE

In September 2022, she went to the emergency room 3 times for precordial chest pain. She was admitted, and an electrocardiogram was performed (image 1), showing ST elevation in V2-V3 associated with repolarization anomalies, as well as troponin elevation. An urgent echocardiogram was requested (image 2), showing apical hypokinesia and an aneurysm in the left common trunk (LCT) of about 7mm. Further testing was done via angiography and CT coronary angiography (image 3) confirming permeability of the right coronary artery (RCA), with total obstruction of the anterior descending artery with aneurysmal dilatation and collateral circulation.

**Results:** OUTCOME

We have a patient with known SLE, clinical presentation of chest pain, with confirmed findings of anterior descending obstruction, LCT aneurysms and secondary STEACS. During admission she presented clinical and analytical lupus activity with negativization of lupus anticoagulant. Doppler ultrasound body scan was performed, without finding signs of thrombosis. Multidisciplinary management was decided, performing coronary artery bypass with left internal mammary artery (LAMI). Intensive corticosteroid therapy was started, in addition to, acetylsalicylic acid, beta-blocker, statin and oral anticoagulation at therapeutic doses. Methotrexate was replaced by mycophenolate mofetil.

**Conclusion:** CLINICAL SIGNIFICANCE

The presence of cardiac involvement in pediatric SLE is approximately 30-40%. Among the most characteristic manifestations are pericarditis, and to a lesser extent myocarditis, endocarditis and Libman-Sacks type valvular lesions.

It is known that the presentation of pediatric lupus is more aggressive than in the adult population. Coronary artery disease, as well as AMI, is extremely rare in patients with pSLE. In children, late diagnosis is common despite anginal symptoms, as it can lead to confusion with other more common conditions. Therefore, we must not forget about these complications, which, although infrequent, compromise the lives of our patients. Control of cardiovascular risk factors and periodic echocardiography are recommended.

**Patient Consent**

Yes, I received consent

**Disclosure of Interest**

None declared

## P467 Diagnostic accuracy and clinical utility of the FEIA and the CLIFT anti-dsDNA assays in childhood onset systemic lupus erythematosus

### J. Postmes^1^, M. Verkaaik^1^, M. J. Wahadat^1^, A. Mubarak^1^, M. Gruijters^1^, A. van Dijk-Hummelman^1^, S. Bakx^1^, M. Schreurs^2^, S. Veenbergen^2^, S. Kamphuis^1^

#### ^1^Department of Pediatric Rheumatology; ^2^Laboratory Medical Immunology, Department of Immunology, Erasmus University Medical Center, Rotterdam, Netherlands

##### **Correspondence:** M. Verkaaik

*Pediatric Rheumatology 2023*, **21(Suppl 2):**P467

**Introduction:** Childhood onset Systemic lupus erythematosus (cSLE) is a severe heterogeneous autoimmune disease. Antibodies against double stranded DNA (anti-dsDNA) are present in 50-70% of cSLE patients and play an important role in diagnosing cSLE. Limited information is available on the diagnostic accuracy of anti-dsDNA assays in children and it is not known whether these tests fulfill the ACR-EULAR requirement for anti-dsDNA assays to achieve ≥90% specificity in cSLE. The Fluorescence Enzyme-immunoassay (FEIA) anti-dsDNA assay is the most often used test. In our centre, an additional Crithidia Luciliae ImmunoFluorescence (CLIFT) anti-dsDNA assay is performed when the FEIA delivers a borderline or positive result (≥10 IU/ml).

**Objectives:** To determine the diagnostic accuracy and clinical utility for diagnosing cSLE of 1) the FEIA anti-dsDNA assay, and 2) the combined FEIA/CLIFT approach.

**Methods:** This single center prospective cohort study included all children < 18 years with a suspected autoimmune rheumatic disease in whom an FEIA anti-dsDNA assay was performed between December 2018 and December 2022. These children were divided into a cSLE patient group and a control group. Additionally, all children diagnosed with cSLE between March 2010 and November 2018 were included. Sensitivity, specificity, positive predictive value (PPV) and negative predictive value (NPV) were calculated for FEIA and the combined FEIA/CLIFT approach, whilst applying different cutoff values for FEIA positivity.

**Results:** Fifty-seven cSLE patients (27 between 2018-2022) and 380 controls were included. When applying the manufacturer’s advised cutoff value for positivity (≥15 IU/ml), the FEIA showed a low sensitivity (43.9%) and low PPV (43.8%) and a high specificity (95.0%) and high NPV (96.4%) for the diagnosis of cSLE. False positive results occurred in 18/380 of controls and, given the rarity of SLE, these outnumbered the true positive results (14/27) in cSLE in the same time period. When the FEIA cutoff value was adjusted to ≥40 IU/ml, the PPV increased (75.0%), whereas the NPV (96.0%) remained the same. The diagnostic accuracy of the combined FEIA/CLIFT approach was optimal when a CLIFT was performed if the FEIA result was ≥15 IU/ml. This combination achieved a comparable sensitivity (44.4%) and NPV (96.0%) compared to FEIA, but a substantially increased specificity (99.5%) and PPV (85.7%).

**Conclusion:** The FEIA anti-dsDNA assay fulfills the ACR-EULAR requirement for anti-dsDNA assays to achieve ≥90% specificity in cSLE, which supports its use for diagnosing cSLE. The occurrence of false positive results can be reduced by either increasing FEIA’s cutoff value for positivity to ≥40 IU/ml, or applying a combined FEIA/CLIFT approach.

**Patient Consent**

Yes, I received consent

**Disclosure of Interest**

None declared

## P468 Outcome and spectrum of neuropsychiatric manifestations in juvenile onset sle: single centre experience from India

### A. Prabhudesai, A. Lawrence, D. P. Misra, V. Agarwal, A. Aggarwal

#### Clinical Immunology & Rheumatology, Sanjay Gandhi Postgraduate Institute of Medical Sciences, Lucknow, India

##### **Correspondence:** A. Prabhudesai

*Pediatric Rheumatology 2023*, **21(Suppl 2):**P468

**Introduction:** Juvenile onset systemic lupus erythematosus (JSLE) is a rare disease with significant morbidity and mortality. Though renal disease has received a lot of attention, neuropsychiatric (NP) manifestations have been less studied. NPSLE has varied spectrum from minimal cognitive dysfunction to serious NP manifestations like stroke, and transverse myelitis.

**Methods:** Retrospective review of case records of JSLE patients seen at a tertiary care rheumatology clinic over the last 25 years was done and patients having NPSLE were identified. Details of patients with NPSLE including demographic, clinical and laboratory data was retrieved from clinical file and hospital information system.

**Results:** Among 338 JSLE patients seen over this period, 88 (26.03%) had NP manifestations. The mean age at presentation was 15.7 (range 8-18) years and among them 8 were boys. Seizures were present in 53 (60.22%), psychosis in 15 (17.04%), mood disorders in 12 (13.63%), headaches in 11 (12.50%) and transverse myelitis in 3 (3.40%) patients. Neuropathy was uncommon with 3 children having peripheral nerve mononeuropathy, 1 having polyneuropathy and 3 had cranial neuropathy.

The most common SLE features in these patients were mucocutaneous manifestations in 79 (89.77%), musculoskeletal in 63 (71.59%) and nephritis in 62 (70.45%). Anti-dsDNA antibodies were present in 69 (78.40%) patients, anti-Sm in 12/41 (29.26%), anti-Ribosomal P in 8/51 (15.68%). Direct coombs test was positive in 25/63 (39.6%) patients while 6/65 patients (9.23%) had positive antiphospholipid antibodies. Most patients received corticosteroids along with immunosuppressive drugs besides supportive care.

Nineteen patients did not follow up at our centre, in the remaining 69 at an average follow up of 6.6 years, 48 (69.6%) had no recurrence of NP features while 2 (2.9%) had recurrent seizures, 2 (2.9%) had intermittent psychotic episode and 2 (2.9%) had recurrent transverse myelitis. The NP manifestations led to morbidity in 4 (5.8%) patients [ residual moderate sensory neural hearing loss in 1, partial foot drop in 1, residual quadriparesis in 2 patients]. 4 patients died [2 due to status epilepticus, 2 due to non-neurological causes).

**Conclusion:** NP manifestations occur in a quarter of patients with JSLE and in about 10% each, they are associated with recurrences and significant morbidity.

**Patient Consent**

Yes, I received consent

**Disclosure of Interest**

None declared

## P469 Outcomes of juvenile systemic lupus erythematosus in young adulthood

### M. Niwa^1,2^, A. Radziszewska^1,2^, J. Peng^1,2^, H. Peckham^1,2^, M. Butt^1,2^, C. Ciurtin^1,2^

#### ^1^Centre for Adolescent Rheumatology Versus Arthritis at UCL UCLH and GOSH; ^2^Centre for Rheumatology Research, Division of Medicine, University College London, London, United Kingdom

##### **Correspondence:** A. Radziszewska

*Pediatric Rheumatology 2023*, **21(Suppl 2):**P469

**Introduction:** Juvenile systemic lupus erythematosus (JSLE) is characterised by a more severe clinical presentation than the adult phenotype, as well as increased risk for damage accrual and comorbidities. Most of the published clinical research in JSLE is focused on the first few years of the disease course, and therefore there is an unmet need to investigate JSLE outcomes in adulthood.

**Objectives:** To characterise the medium-term outcomes of young adults (YA) with JSLE followed up in our centre to identify disease outcomes and predictors of damage.

**Methods:** Detailed patient and disease characteristics, including demographics, clinical and serological parameters, type of organ involvement and medication, were collected from medical records. Disease activity and damage were assessed using validated scores (SLEDAI-2000 and pBILAG, and PedSDI, respectively). Results are presented using descriptive statistics and regression analysis.

**Results:** Complete datasets were available for 89 JSLE patients (75 females and 14 males) with a mean age of 26 ± 4.18 years and disease duration of 13.5± 4.71 years (31.4% White, 24.7% Black, 22.4% Asian, and 21.5% Other). The most common manifestations were: mucocutaneous/haematological (94.8%), musculoskeletal (82%), constitutional (75.2%), while 39.33% had lupus nephritis (LN) and 15.7% neuropsychiatric manifestations. There were no sex differences between patients who accrued damage (27%) vs. not (73%), but patients with damage were older (27±3 vs. 25±4 years, p=0.026), had more renal (50% vs. 27.6%, p=0.048) and neurological involvement (33.3% vs. 13.8%, p= 0.038), and higher median global BILAG (1±1.7 vs. 0.9±3.3, p= 0.048) but not SLEDAI (2±2.21 vs. 2±2.9, p=0.09) at the last assessment. Patients without damage were more frequently treated with azathioprine (73.8% vs. 50%, p=0.033). Overall, non-White vs. White patients had higher SLEDAI scores at last assessment (p=0.005). Neurological manifestations and presence of anti-La antibodies were predictors of damage when accounting for age, sex, ethnicity, renal disease, disease duration, and disease activity at last assessment (p=0.04, p=0.01, respectively).

**Conclusion:** This analysis, of the largest YA JSLE cohort in the UK, highlights ethnic disparities in JSLE outcomes in young adulthood. Neurological manifestations, LN, older age, more active disease at the last assessment were the main predictors of damage. The relevance of anti-La seropositivity in predicting damage accrual requires further investigation.

**Patient Consent**

Not applicable (there are no patient data)

**Disclosure of Interest**

None declared

## P470 Urinary NGAL as a biomarker in paediatric systemic lupus erythematosus

### F. Rajão Martins^1^, R. Nicolau^2^, T. Beirão^3^, S. Ganhão^4^, F. Aguiar^4^, M. Rodrigues^4,5^, C. Ferreras^6^, S. Martins^7,8^, R. Farinha^7,8^, I. Brito^4,5^

#### ^1^Rheumatology, University Hospital Centre of Algarve, Faro; ^2^Rheumatology, University Hospital Centre of Tondela-Viseu, Viseu; ^3^Rheumatology, University Hospital Centre of Vila Nova de Gaia / Espinho, Vila Nova de Gaia; ^4^Paediatric and Young Adult Rheumatology Unit, University Hospital Centre São João; ^5^Faculty of Medicine, University of Porto; ^6^Paediatrics; ^7^Clinical Pathology, University Hospital Centre São João; ^8^EPIUnit/ITR - Institute of Public Health and Laboratory for Integrative and Translational Research on Public Health, University of Porto, Porto, Portugal

##### **Correspondence:** R. Nicolau

*Pediatric Rheumatology 2023*, **21(Suppl 2):**P470

**Introduction:** Lupus nephritis (LN) is a serious renal manifestation of systemic lupus erythematosus (SLE), constituting the leading cause of chronic kidney disease in these patients. Absence of reliable serum or urine biomarkers for early diagnosis of LN is troublesome in clinical practice. Several urinary cytokines such as the TNF-like weak inducer of apoptosis (TWEAK), monocyte chemoattractant protein-1 (MCP-1) and neutrophil gelatinase-associated lipocalin (NGAL) have been proposed as early indicators of renal involvement in SLE^1,2^.

**Objectives:** We aim to describe associations between clinical and laboratorial variables and urine TWEAK, MCP-1 and NGAL levels in paediatric SLE patients.

**Methods:** Patients with a definite SLE diagnosis before the age of 21 years-old were consecutively recruited at a tertiary hospital’s paediatric rheumatology unit. Sociodemographic, clinical and laboratorial data was collected. Differences between groups were assessed using T-Test for independent variables and Mann-Whitney-U and Kruskall Wallis tests. Association between variables was assessed using Pearson correlation. A p-value <0.05 was considered as significant.

**Results:** From an initial group of 50 individuals, 36 were included in the analysis, mostly female (97.2%), average age at diagnosis 13.9 ± 3.8 years and average age at analysis 24.2 ± 7.7 years. Mucocutaneous (n=23, 63.9%), articular (n=21, 58.3%), haematological (n=21, 58.3%) and LN (n=15, 41.7%) were the most frequent manifestations of the disease. Class 4 LN was the most frequent (n=9, 60%). Most patients were under corticosteroids (n=28, 77.8%) and hydroxychloroquine (n=29, 80.60%), with a significant proportion treated with mycophenolic acid (n=15, 41.7%). At time of analysis, most patients were either in remission (SLEDAI=0, n=13, 36.1%) or in a low disease activity state (SLEDAI <4, n= 9, 25%), and damage accrual was low (average SLICC damage index 0.17 ± 0.38).

Significant differences between groups were observed for TWEAK (higher in non-NL patients, p=0.01; lower in patients receiving steroids, p= 0.04) and MCP-1 levels (higher in anti-P-ribossome antibody positive patients, p = 0.04). NGAL was negatively correlated with erythrocyte sedimentation rate (ρ= - 0.47, p <0.01), C3 (ρ= - 0.51, p < 0.01), C4 (ρ= - 0.35, p=0.04) and AST (ρ= - 0.37, p = 0.03). No statistically significant correlations were observed with other biomarkers, nor were any on them associated with urine parameters. No differences in biomarker levels were observed according to other therapeutic regimens.

**Conclusion:** NGAL urine levels may represent a surrogate marker for global disease activity in paediatric SLE patients. Small population size and absence of a control group limit interpretation of the findings. Further studies are required for biomarker validation.

**Patient Consent**

Yes, I received consent

**Disclosure of Interest**

None declared

**References**

1. Costa-Reis P, Maurer K, Petri MA, et al. Urinary HER2, TWEAK and VCAM-1 levels are associated with new-onset proteinuria in paediatric lupus nephritis. *Lupus Sci Med*. 2022;9(1). doi:10.1136/LUPUS-2022-000719
2. Dong X, Zheng Z, Luo X, et al. Combined utilization of untimed single urine of MCP-1 and TWEAK as a potential indicator for proteinuria in lupus nephritis: A case-control study. *Med (United States)*. 2018;97(16). doi:10.1097/MD.0000000000010343

## P471 Detection of genetic mutations underlying early-onset systemic lupus erythematosus

### S. Sener^1^, E. Sag^2^, X. Han^3^, Y. Bilginer^1^, Q. Zhou^3^, S. Ozen^1^

#### ^1^Pediatric Rheumatology, Hacettepe University; ^2^Pediatric Rheumatology, Ankara Research and Training Hospital, Ankara, Türkiye; ^3^Zhejiang University, Life Sciences Institute, Zhejiang, China

##### **Correspondence:** S. Sener

*Pediatric Rheumatology 2023*, **21(Suppl 2):**P471

**Introduction:** The discovery of new genes with common or rare variants and the detection of different gene mutations may guide us in understanding and better management of monogenic lupus patients.

**Objectives:** We aimed to investigate the presence of monogenic causes of systemic lupus erythematosus (SLE) in our early-onset SLE patients.

**Methods:** Fifteen pediatric SLE cases who had early disease onset (before 6 years of age) were enrolled in this study. All patients fulfilled the Systemic Lupus International Collaborating Clinics (SLICC) criteria. Genomic DNA was extracted from whole blood, then it was used for whole exome sequencing (WES). Pathogenic variants were confirmed by Sanger sequencing.

**Results:** The median age at diagnosis of 15 early-onset SLE patients included in the study was 4 (2-6) years (F/M=12/3). Significant gene mutations were detected in five of these patients (33.3%). Patients 1 and 2 with homozygous *DNASE1L3* mutations [*c.320+4_320+7del* and *G188A (c.563G>C)* variants] had skin involvement and oral ulcers. One of them (patient 1) had arthritis and nephritis, and another (patient 2) had nonscarring alopecia and thrombocytopenia. They are currently clinically inactive but have positive serological findings. Patient 3 with homozygous pathogenic *ACP5* mutation [*G109R (c.325G>A)* variant] had arthritis, nephritis, short stature, and skeletal dysplasia. Patient 4 with a heterozygote novel *IFIH1* mutation [*L809F (c.2425C>T)* variant] had skin findings and leukopenia. Patient 5 with the novel *C1S* variant [homozygous *C147W (c.441C>G)* variant] had marked skin findings, oral ulcers, nonscarring alopecia, and pancytopenia. She also had a low total hemolytic complement CH50 level. All patients have responded to the treatments and have low Systemic Lupus Erythematosus Disease Activity Index (SLEDAI) scores, on therapy.

**Conclusion:** Genetic causes should be investigated in early-onset SLE, for better management and genetic counseling. On the other hand, multicenter studies may help to further define genotype-phenotype associations.

**Patient Consent**

Yes, I received consent

**Disclosure of Interest**

None declared

**References**

1. Batu ED, Koşukcu C, Taşkıran E, Sahin S, Akman S, Sözeri B, Ünsal E, Bilginer Y, Kasapcopur O, Alikaşifoğlu M, Ozen S. Whole Exome Sequencing in Early-onset Systemic Lupus Erythematosus. J Rheumatol. 2018 Dec;45(12):1671-1679. doi: 10.3899/jrheum.171358.
2. Demirkaya, E.; Sahin, S.; Romano, M.; Zhou, Q.; Aksentijevich, I. New Horizons in the Genetic Etiology of Systemic Lupus Erythematosus and Lupus-Like Disease: Monogenic Lupus and Beyond. J. Clin. Med. 2020, 9, 712. 10.3390/jcm9030712.

## P472 Natriuretic peptide and catecholamines in adolescents with systemic lupus erythematosus

### T. O. Golovko^1,2^, L. F. Bohmat^1^, N. S. Shevchenko^1,2^

#### ^1^Department of Rheumatology and Comorbid Conditions, S.I."Institute for Children and Adolescents Health Care of the National Academy of Medical Sciences of Ukraine"; ^2^Department of Pediatrics , V. N. Karazin Kharkiv National University , Kharkiv, Ukraine

##### **Correspondence:** N. S. Shevchenko

*Pediatric Rheumatology 2023*, **21(Suppl 2):**P472

**Introduction:** Systemic lupus erythematosus (SLE) is an autoimmune chronic disease of uncertain etiology, in which all organs and systems of the body, including the cardiovascular system, are involved in the pathological process. The spectrum of cardiac pathology is diverse and all structures of the heart can be involved in the pathological process, but myocarditis occupies the first place in frequency. It is observed in almost all children with SLE. In recent decades, the prognosis of the disease has improved significantly due to the use of active immunosuppressive therapy. At the same time, long-term use of glucocorticosteroids and cytostatic drugs creates additional conditions for the development of changes in the cardiovascular system and contributes to the formation of irreversible changes.

**Objectives:** To determine the level of brain natriuretic peptide (NT-proBNP) in the blood and the level of excretion of catecholamines (epinephrine and norepinephrine) in daily urine in patients with SLE.

**Methods:** There were examined 15 adolescents with SLE (14 girls and 1 guy) aged 14.13±0.64 years with a disease duration of 52.00±12.56 months who received basic immunosuppressive therapy for more than 1 year or more (azathioprine 28,73±5.50 months, hydroxychloroquine 26.93±5.58 months, methylprednisolone 32.87±9.64 months). At the time of the examination, the doses of basic therapy drugs were as follows: azathioprine 2.09±0.19 mg/kg, hydroxychloroquine 4.98±0.49 mg/kg, methylprednisolone 0.18±0.03 mg/kg. The control group consisted of 16 healthy girls aged 13.70±0.38 years.

To assess the state of the sympathoadrenal system, the content of catecholamines (epinephrine and norepinephrine) in the daily urine was studied by iodine oxidation with the formation of fluorescent trihydroxyindoles (adrenolutin and noradrenolutin) and subsequent fluorimetry using a Fluorat 02-ABLF-T analyzer. The study of NT-proBNP in the blood was carried out by competitive immunoassay on an IMMULITE 2000 Siemens analyzer. Statistical processing of the obtained data was carried out using the SPSS17 application software package (license 4а180844250981ae3dae-s/nSPSS17) on an IBM PC Pentium-4. The arithmetic mean and standard error were calculated for all indicators. Comparison of indicators was carried out with similar indicators of adolescents in the control group.

**Results:** In patients with SLE, the level of NT-proBNP was within the age norms (47.34±8.87 pg/l), but was significantly higher than in the girls of the control group (29.27±5.23 pg/l, p = 0.02). The level of excretion of catecholamines in the daily urine was also within the age norms, while the level of epinephrine was significantly lower than in healthy peers (epinephrine 24.88±3.22 nmol/l vs 33.34±3.07 nmol/l of adolescents in the control group), p = 0.02; norepinephrine 85.78±9.75 nmol/l vs 104.51±7.95 nmol/l in adolescents in the control group, p = 0.07). The epinephrine /norepinephrine ratio did not change significantly (0.29 ± 0.02 versus 0.32±0.01, р = 0.2).

**Conclusion:** An increase in the level of brain natriuretic peptide in the blood and a decrease in the excretion of epinephrine in the daily urine in patients with systemic lupus erythematosus may affect the formation of cardiovascular disorders

**Patient Consent**

Not applicable (there are no patient data)

**Disclosure of Interest**

None declared

## P473 Clinical features, management, and outcomes of neuropsychiatric lupus in Saudi children, a multicentric study

### R. Shigdar^1^, H. Shogdar^2^, F. Althubaiti^3^, R. Albakry^4^, M. Muzaffer^5^, L. Akbar^6^, M. Nashawi^5^

#### ^1^Faculty of Medicine, King Abdulaziz University; ^2^Emergency Medicine, King Abdulaziz Hospital; ^3^Pediatric Neurology, Faculty of Medicine, King Abdulaziz University; ^4^Pediatric Rheumatology, Jeddah East Hospital; ^5^Pediatric Rheumatology, Faculty of Medicine, King Abdulaziz University, Jeddah; ^6^Pediatrics, College of Medicine, Imam Abdulrahman Bin Faisal University, Dammam, Saudi Arabia

##### **Correspondence:** M. Nashawi

*Pediatric Rheumatology 2023*, **21(Suppl 2):**P473

**Introduction:** Systemic lupus erythematosus (SLE) is a complex autoimmune disorder characterized by multisystem involvement, including the nervous system [1]. Approximately one-third of all children with SLE will display neuropsychiatric manifestations at some point during the disease. Early diagnosis and prompt intervention are critical for a better prognosis [2].

**Objectives:** The goal of this research is to describe the most common clinical features, laboratory and radiological findings, management, and outcomes of pediatric neuropsychiatric lupus in Saudi Arabia.

**Methods:** Data of all children with SLE and evidence of neuropsychiatric manifestations were obtained from the database of 3 tertiary centers in Saudi Arabia. NPSLE is defined by the presence SLE in addition to at least one of the following: cerebrovascular accident (CVA), movement disorder, seizure, cognitive dysfunction, psychiatric or spinal cord manifestations. Patients with isolated headache or concomitant diseases were exluded.

**Results:** Our study included 21 patients (19 females and 2 males) diagnosed at mean age of 10.3 years. Their clinical features were headache (52.4%), seizure disorders (52.4%), CVA (28.6%), movement disorders (9.5%), acute confusional state (28.6%), cognitive dysfunction (23.8%), mood disorder (38.1%), and GBS (4.8%). ANA titer was positive in 19/21, and dsDNA was high in 20/21. MRI /MRA showed positive findings in 9/15. The management consisted of a variety of regimens. However, most patients were given methylprednisolone pulse therapy in addition to cyclophosphamide or rituximab, and mycophenolate as maintenance. In the 24-month follow-up of 13/21 patients, neuropsychiatric symptoms had resolved in most while 2 patients were deceased.

**Conclusion:** This study shows the heterogenicity of NPSLE management in children. A larger study including all regions in Saudi Arabia is needed to estimate the exact prevalence of the disease in our region. Finally, a national consensus recommendation regarding treatment is necessary for better disease outcome.

**Patient Consent**

Not applicable (there are no patient data)

**Disclosure of Interest**

None declared

**References**

1. Aringer M, Costenbader K, Daikh D, Brinks R, Mosca M, Ramsey-Goldman R, et al. 2019 European League Against Rheumatism/American College of Rheumatology classification criteria for systemic lupus erythematosus. Arthritis Rheumatol [Internet]. 2019 [cited 2023 May 23];71(9):1400–12. Available from: https://pubmed.ncbi.nlm.nih.gov/31385462/
2. Charras A, Smith E, Hedrich CM. Systemic lupus erythematosus in children and young people. Curr Rheumatol Rep [Internet]. 2021 [cited 2023 May 23];23(3):20. Available from: https://pubmed.ncbi.nlm.nih.gov/33569643/

## P474 Chronicles of an announced complication: the risk of undiagnosed SLE treatment after abdominal surgery in a 12 year old female

### L. I. Sifuentes-Aguilar^1^, O. O. Loya-Guerrero^1^, A. V. Villarreal-Treviño^2^

#### ^1^Pediatrics; ^2^Pediatric Rheumatology, Tecnologico de Monterrey, Monterrey, Mexico

##### **Correspondence:** L. I. Sifuentes-Aguilar

*Pediatric Rheumatology 2023*, **21(Suppl 2):**P474

**Introduction:** We present the case of a 12 year old female, who came to the emergency department with acute complicated appendicitis and long term symptoms of Systemic Lupus Erythematosus (malar rash, joint pain and kidney involvement). As an important medical background, she was diagnosed with juvenile arthritis 3 months earlier, though she did not receive any rheumatology evaluation nor she had any serological exams performed; she had been treated with prednisone since her diagnosis. At arrival to our center, she was treated with conventional appendectomy, antibiotics, oral prednisone and hydroxychloroquine. 5 days later she presented fecaloid discharge at the surgical site. She was again subjected to surgery where a resection of the affected site was performed, requiring an ileostomy. Meanwhile, SLE treatment had to be delayed, even though we already had serological confirmation of SLE with ANAs (1:640, fine speckled by IIF), Anti-DNAs (>666.9 UI/mL, reference value <27UI/ml) and AntiSm (106.3 CU, reference value <20 CU) antibodies; this decision had to be made because there was high risk of surgical site dehiscence and because we obtained a positive QuantifFERON™ - TB Gold Test. Tuberculosis prophylaxis was provided after. 72 hours later, she presented with a mood disorder and multiple seizures, as well as proteinuria and hematuria. After the seizures were controlled, treatment with cyclophosphamide started. Many complications occurred in the following days, including abdominal sepsis, pleural effusion, hypovolemic shock, pseudomembranous colitis and suspected rectovaginal fistula.

**Conclusion:** Currently in Mexico there is a delay of Systemic Lupus Erythematosus diagnosis, especially in the pediatric population; this case represents this statement as she had symptoms compatible with SLE for almost eight months. She came to our center purely as an abdominal emergency, but when digging a bit deeper, we could diagnose and start appropriate treatment for her base pathology. It is of great importance to raise awareness of SLE in the pediatric population, so they can receive the appropriate treatment at the appropriate time and avoid at the most possible complications.

**Patient Consent**

Yes, I received consent

**Disclosure of Interest**

None declared

## P475 Neonatal lupus erythematosus – a lesson learnt over 11 years of experience from a tertiary care centre of North India

### A. Sil^1^, A. Thangaraj^1^, M. Arora^1^, R. K. Pilania^1^, D. Suri^1^, A. Rawat^1^, A. K. Baranwal^2^, S. Singh^1^

#### ^1^Allergy Immunology Unit, Department of Pediatrics, Advanced Pediatrics Centre; ^2^High Dependency Facility (HDF), Department of Pediatrics, Advanced Pediatrics Centre, Post Graduate Institute of Medical Education and Research, Chandigarh, India

##### **Correspondence:** A. Sil

*Pediatric Rheumatology 2023*, **21(Suppl 2):**P475

**Introduction:** Neonatal lupus erythematosus (NLE) is a passively acquired autoimmunity leading to various manifestations in neonates and infants. The most significant complication of NLE is cardiac dysrhythmia.

**Objectives:** To report the clinical spectrum, laboratory features (including the autoantibody profile of mothers), treatment, and outcomes in an NLE cohort from North India.

**Methods:** Data were collated from clinic files of patients registered at the Pediatric Rheumatology Clinic, Advanced Pediatrics Centre, Postgraduate Institute of Medical Education and Research, Chandigarh, India, from 2011 to 2022. Demographic features, clinical profiles, and laboratory parameters of the children were analyzed in detail, including the autoantibody profiles of the mothers. The statistical analysis was done using SPSS version 29.

**Results:** Fifteen patients were found to have features of NLE. The median age at diagnosis was 60 days (1 day to 1440 days), with a male to female ratio of 1.14:1. Cutaneous manifestations were reported in 9 (60%) patients, which included rash over the face (especially in the periorbital area), scalp, trunk, back, chest, and groin areas. Rash was maculopapular in nature in 3 patients, annular in 1, discoid in 1, and macular with central clearing or scaling in the rest. Hepatomegaly and splenomegaly were noted in 9 (60%) and 2 (13.3%) patients, respectively. Elevated transaminases were found in 6 (40%) patients; one patient had features of cholestatic jaundice that subsided over a few weeks. Haematological manifestations described in our cohort were bicytopenia (5), pancytopenia (2), anaemia (2), and thrombocytopenia (3), which resolved over a period of a few weeks. However, one patient required intravenous immunoglobulin (IV Ig) therapy. Neurological manifestations included seizures, developmental delay, phrenic palsy, and macrocephaly in one patient each. IV Ig was required for the patient with phrenic nerve palsy.

Cardiac involvement was reported in 7 (46.6%) patients: 3rd degree heart block in 5, 2nd degree heart block in 1, and PR prolongation in 1. Of the 5 patients with 3rd-degree heart block, 4 were managed with epicardial pacemaker insertion. One patient required reimplantation due to infective endocarditis, and one patient was lost to follow-up. Congenital hydrops was seen in two patients, both of whom required respiratory support, and recovery was uneventful. Antinuclear antibody (ANA) was found to be positive in 9 (60%) babies. On investigating the mother, 9 (60%) were found to be symptomatic, and 4 had a history of recurrent pregnancy loss. ANA records of mothers were available in 11 cases, and all of them were positive. ANA immunoblot showed both anti-Ro and anti-La positivity in 4 and only anti-Ro positivity in 4. No mortality was reported in this cohort.

**Conclusion:** Neonatal lupus is associated with significant morbidity. Nearly 50% had cardiac manifestations, of which 70% had 3rd-degree heart block, which required pacemaker insertion. Other rare clinical manifestations, like phrenic nerve palsy and seizures, were noticed in our cohort.

**Patient Consent**

Yes, I received consent

**Disclosure of Interest**

None declared

## P476 Assessing standards of care for juvenile systemic lupus erythematosus in an outpatient tertiary paediatric setting

### M. Sim^1^, A. Chieng^2^, V. Cuthbert^1^, A. Hollows^1^, M. Morrisroe^1^, D. Nicholson^1^, P. Riley^1^, H. Lythgoe^1^

#### ^1^Paediatric Rheumatology Department; ^2^Paediatric Rheumatology, Manchester University NHS Foundation Trust, Manchester, United Kingdom

##### **Correspondence:** M. Sim

*Pediatric Rheumatology 2023*, **21(Suppl 2):**P476

**Introduction:** Juvenile systemic lupus erythematosus (JSLE) is a complex, multi-system inflammatory condition. Ongoing care and outpatient management of JSLE must be multi-dimensional to meet their varied and dynamic needs.

**Objectives:** We sought to evaluate current outpatient management of children with JSLE against standards of care formulated from international, national, and local recommendations.

**Methods:** Standards of care were formulated from British Society for Rheumatology, European League Against Rheumatism, Paediatric Rheumatology European Society, and Single Hub and Access point for Rheumatology in Europe guidelines. Local expertise was also sought from paediatric rheumatologists, immunologists, and endocrinologists as well as specialist nurses, occupational and physiotherapists. Management of children with JSLE in a tertiary paediatric setting was audited against these standards in 2022.

**Results:** A total of 20 JSLE patients were included. The median number of clinics attended by those newly diagnosed (2021-2022; n=7) was 5 (IQR: 3-7.5), with a mean interval of 2.1 months between clinics. Those diagnosed prior to 2021 (n=13) attended a median of 3 clinics/year (IQR 2-4), with a mean interval of 3.6 months between clinics.

Medications were reviewed at every appointment for 100% of patients; however, discussions about medication compliance was explicitly documented and assessed at every clinic in only 5% (reviewed at least annually in 40%). A total of 95% were treated with hydroxychloroquine, however, annual ophthalmology review was achieved in only 45%.

Standardised measures of disease activity (pBILAG/SLEDAI) and damage (SLICC SDI) was not recorded in clinical letters for use in clinical practice (0%), although most patients are participants of the UK JSLE Cohort Study so likely had them completed for research purposes. Laboratory markers of disease activity (FBC, ESR, anti-dsDNA, C3, C4) were completed at most visits (≥75% for each).

Important lifestyle factors were also considered. Exercise was discussed at least annually in 60% of patients and bone health considered in 50%, but documented discussion of other important factors such as smoking (5%), alcohol and drugs (20%), pregnancy/fertility/contraception (30%), sunscreen (25%), mental health (20%) was less reliable.

**Conclusion:** These findings highlight the challenges in delivering holistic, comprehensive care within busy outpatient settings. Lifestyle factors, in particular, are less reliably discussed in clinic, with exercise levels not always explored despite its role in the non-pharmacological management of fatigue and mood. We have developed a dedicated, multi-disciplinary lupus clinic as a potential way to address these needs by increasing proactive input from allied healthcare professionals whilst reducing the number of separate appointments required for patients and their families. Future work will re-evaluate these standards following initiation of the clinic along with patient feedback.

**Patient Consent**

Not applicable (there are no patient data)

**Disclosure of Interest**

None declared

## P477 Early onset systemic lupus erythematosus: a case series of four patients with C1Q deficiency

### N. Singh^1^, J. Janardhanan^1^, S. Ramprakash^2^, R. C. P^2^, S. Bhattad^1^

#### ^1^Pediatric Immunology and Rheumatology; ^2^Pediatric Hematoncology and BMT, Aster CMI Hospital, Bengaluru, India

##### **Correspondence:** N. Singh

*Pediatric Rheumatology 2023*, **21(Suppl 2):**P477

**Introduction:** Primary C1q deficiency is associated with recurrent infections and systemic lupus erythematosus (SLE). Inherited in an autosomal recessive manner, the onset of the illness is in early childhood. The most common presentation is cutaneous involvement, followed by renal and cerebral involvement.

**Objectives:** To describe the clinical profile, the treatment, and the outcome of four early onset SLE patients with C1q deficiency.

**Methods:** Three hundred nineteen patients with various Inborn errors of Immunity (IEIs) were diagnosed in the Paediatric Immunology and Rheumatology Unit of our hospital during the study period of February 2017 to January 2023. Six patients from five kindred had early onset SLE, out of which four had biallelic mutations in *C1q* gene, one patient had a biallelic loss-of-function mutation in *protein kinase C δ (PKCδ)* gene and whole exome sequencing was negative in one patient. We analysed the available clinical data of four patients with C1q deficiency.

**Results:** The mean age of onset was 1.4 years and mean age at presentation was 11 years. Consanguinity was noted in two families and there was history of sibling deaths in two families. Male to female ratio was 1:1. The most common manifestation was mucocutaneous involvement including oral aphthosis(4), alopecia(4), vitiligo (n=1) and Raynaud’s phenomena (n=1), followed by autoimmune cytopenia (n=1) and renal involvement(n=1). P4 presented with rapidly progressive glomerulonephritis, and the renal biopsy showed Class IV Nephritis (Activity Score 8/24, Chronicity Score 0/12). P2 had neutropenia while P3 had thrombocytosis. The spectrum of infections included otitis media (n=4), pseudomonas sepsis(n=1) and subdural empyema (n=1). Anti-nuclear antibody by immunofluorescence was negative in P3 and P4. Whole exome sequencing in all four patients showed deletions. While P1 had a frameshift mutation(*C1QA* chr 1:22964153delT c.44delT p.lle15AnsfsTer7), P2 had a terminal deletion [*C1QB* Exon 2 c.171del(p.Gly58GlufsTer86)], P3 and P4 had a novel mutation [*C1QC* Exon 3 c.643_645del (p.Glu216del)]. Two patients were on fresh frozen plasma therapy to correct C1q deficiency and one patient underwent a hematopoietic stem cell transplant and succumbed to post-transplant complications. Two patients were treated with azathioprine, and one patient with mycophenolate mofetil. Overall, three patients remain under close follow-up.

**Conclusion:** We present our single-center experience of C1q deficiency from the Indian subcontinent. A significant delay in the diagnosis of these patients was observed in our cohort.

**Patient Consent**

Yes, I received consent

**Disclosure of Interest**

None declared

## P478 A patient with SLE and extremely high levels of CSF protein—disease manifestation or hydroxychloroquine toxicity?

### A. T. Suchi^1,2^, V. Gotloib^3^, Y. Uziel^1,2^, K. Geva^2,4^, R. Haviv^1,2^, A. Ziv^1,2^

#### ^1^Pediatric Rheumatology Unit, Department of Pediatrics, Meir Medical Center, Kfar Saba; ^2^sackler school of medicine, Tel Aviv University, Tel Aviv; ^3^Pediatrics, Haemek Medical Center, Afula; ^4^Pediatric neurology unit, Department of Pediatrics, Meir Medical Center, Kfar Saba, Israel

##### **Correspondence:** A. T. Suchi

*Pediatric Rheumatology 2023*, **21(Suppl 2):**P478

**Introduction:** This case report discusses a patient with SLE, neuropathy, and myopathy who had exceptionally high CSF protein levels while taking hydroxychloroquine.

Case presentation

A 15.5-year-old girl with morbid obesity and a history of arthralgia and alopecia, presented with extreme weakness, and severe Coombs positive hemolytic anemia (Hb 5 gm).

Based on positive SLE autoantibodies and high erythrocyte sedimentation rate, she was diagnosed with SLE. Corticosteroids and hydroxychloroquine (HCQ) stabilized her symptoms. However, 9 months later, while receiving HCQ only, she developed muscle weakness and paresthesia. Ophthalmological examination revealed severe papilledema and visual field impairment. Brain and spinal magnetic resonance imaging (MRI) demonstrated increased intracranial fluid and contrast enhancement near the optic nerves. Diffuse polyradiculopathy with adjacent muscle fat infiltration of the paraspinal muscles was seen on MRI of the muscles. Lumbar puncture found high intracranial pressure (36.5 cm/H_2_O), (normal range 6 to 25 cmH_2_O) extremely high levels of protein, 900 mg/dl (normal range 15 to 60 milligrams per deciliter (mg/dL)), No WBC

Due to suspicion of HCQ toxicity, muscle biopsy was performed, with findings of nonspecific inflammatory changes. No vacuoles were found. Myositis panel was negative. Electrophysiology showed axonal damage and not nerve demyelination, which is inconsistent with a diagnosis of chronic inflammatory demyelinating polyneuropathy (CIDP). Electromyography -nerve conduction studies did not support CIDP either. Due to a differential diagnosis of HCQ toxicity, the drug was stopped. Acetazolamide was started with partial improvement. High-dose steroids and Belimumab were added. After a neurology consultation, it was agreed to proceed with a therapeutic trial of intravenous immunoglobulin despite lacking a clear-cut diagnosis. Symptoms of papilledema, muscle strength and visual field abnormalities gradually improved, but the specific cause remained uncertain.

**Conclusion:** This report presents a girl with SLE who presented with myopathy and neuropathy, initially suspected to be due to HCQ toxicity; a known but very rare complication. However, the extremely high CSF protein levels and lack of significant improvement after stopping the drug raised the possibility of a rare neurologic manifestation of SLE. While HCQ toxicity remains the most likely diagnosis, CIDP could not be completely ruled out.

Notably, there are no cases in the literature with such high CSF protein levels in SLE patients, adding to the complexity of the diagnosis.

**Patient Consent**

Yes, I received consent

**Disclosure of Interest**

None declared

## P479 Antiphospholipid syndrome in a newborn: case report in a patient with neonatal lupus, IGG and IGA antibodies and multiple thrombotic events

### A. Fonseca G Da Silva^1^, F. R. Sztajnbok^2^

#### ^1^Pediatrics, HUGG/UNIRIO; ^2^Pediatrics, IPPMG/UFRJ, Rio de Janeiro, Brazil

##### **Correspondence:** A. Fonseca G Da Silva

*Pediatric Rheumatology 2023*, **21(Suppl 2):**P479

**Introduction:** Antiphospholipid syndrome is a systemic autoimmune disease characterized by an increased risk of venous and arterial thrombosis. Pediatric syndrome is a poorly treated condition. In newborns, the most frequent etiology is the transplacental passage of maternal antibodies of the IgG subtype.

**Objectives:** Case description of APS in neonatal lupus, with negative maternal antibodies, multiple thrombotic lesions and positivity of antiphospholipid antibodies, with the presence of IgG and IgA subtypes.

**Methods:** Case description

**Results:** Female patient, 3 months and 12 days old, born after in vitro fertilization, by cesarean section, preterm, admitted after birth in ICU due to intrauterine diagnosis of complete AV block. Her mother was followed for primary Sjogren's Syndrome, with ANA and anti-Ro in high titers, and antiphospholipid antibodies negative from the beginning of the follow-up to the pospartum period; she has used of hydroxychloroquine and low-dose prednisone during the all pregnacy; no history of COVID infection. A permanent pacemaker was implanted with 2500g of weight. On the 80th day, she developed venous thrombosis of the left external jugular, right brachiocephalic and subclavian branches, and low molecular weight heparin was started for treatment. On the 94th day of hospitalization, a thrombus was seen in the left iliac and inferior vena cava, with an increase in the dose of heparin. During the investigation, she presented negative ANA, anticardiolipin and anti beta2 glycoprotein IgG+, with negative IgM; anti Ro and anti-RNP positives, anti-La, lupus anticoagulant and COVID Ag were negative. Doses of low molecular weight heparin were increased to 4mg/kg/day, with stabilization in the formation and expansion of thrombi. The patient was discharged for outpatient follow-up. These antibodies remain present until the age of 18 months.

**Conclusion:** The production of IgM class antibodies in neonates has already been described in a mother diagnosed with lupus and aPL +, suggesting the “de novo” antibodies production by the fetus. The origin of these antibodies may be related to an autoimmune disorder or an infection. Anticardiolipin and anti-β2GPI antibodies were detected in newborns and corresponded to the mother's antibody profile. In Rolim's study, there were no cases of thrombosis or neonatal lupus from the 134 children born from mothers with APS.

In the present case, the mother presented negative aPL research results on more than one occasion, with no possibility of this mechanism in the newborn. Further studies on isolated positivity in newborns from mothers with non-reactive aPL are necessary. In the case in question, in vitro fertilization as well as vaccination against COVID are possibilities to be investigated, despite the transplacental passage of mRNA from the administered vaccine having no evidence in the most recent studies.

**Patient Consent**

Yes, I received consent

**Disclosure of Interest**

None declared

## P481 Systemic lupus erythematosus presenting as optic perineuritis

### O. Vougiouka on behalf of Eleni Statha, Anna Mourgela, Stavroula Oikonomou, Eirini Sakou, Maria Tsolia

#### 2nd Department of Paediatrics, National Kapodistrian University "P. A. Kyriakou" children's Hospital, Athens, Greece

##### **Correspondence:** O. Vougiouka

*Pediatric Rheumatology 2023*, **21(Suppl 2):**P481

**Introduction:** Neuroophthalmologic manifestations are reported in about 0,5% childhood SLE with a mean onset age 12,6 ± 1 years. Optic neuritis (OP) can be the initial sign. Optic Perineuritis (OPN) is even rarer corresponding to a handful of case reports in literature.

**Objectives:** The presentation of pathophysiology, laboratory investigation, diagnosis and treatment of a patient with SLE/OPN.

**Methods:** Case report of an adolescent with asymptomatic bilateral OPN as an initial sign of SLE.

**Results:** A 14 years old, male adolescent with unremarkable past history, presented to the Emergency Department with three days’ fever, arthralgia and left extremity limping. Clinical examination revealed frank left knee and ankle arthritis. Initial laboratory findings were unremarkable. Left knee arthrocentesis was sterile. The investigation expanded to inflammatory arthritis. Ophthalmological examination revealed grade 2 optic disc oedema but intact visual acuity and optic fields. OCT showed bilaterally extremely swollen optic fibers and brain/orbital MRI highlighted enhancement around the optic nerve sheath (Tram-Track sign). Lumbar puncture unfolded pleocytosis (WBC 75/L), normal pressure, cytology, biochemistry, immunology, culture. The serum autoimmune profile disclosed positive ANA 1/320, low C3 70 mg/dL (90-180), C4 3,2 mg/dL (13-75) CH50 45% (74-124) and negative anti ds DNA, antiphospholipides, anti DNASE, normal body specimen cultures. During hospitalization our patient developed migratory arthritis. Based on clinical and laboratory findings he fulfilled the International Lupus criteria (SLICC 2012, EULAR 2019). Because of the ophthalmological findings pulse Methylprednisolone was administered with dramatic ophthalmological improvement besides general condition. Azathioprine immunomodulation was added to continuing corticosteroid slow tapering.

**Conclusion:** OPN is a rare heterogeneous inflammatory process of the optic nerve sheath with common manifestations to ON, when symptomatic, such as unilateral central vision loss and orbital pain, central scotoma. It has been rarely reported in SLE literature. The diagnosis is confirmed by orbital MRI imaging and optic nerve OCT. Prompt high corticosteroid administration is related to good prognosis.

**Patient Consent**

Yes, I received consent

**Disclosure of Interest**

None declared

**References**

1. Hongyang Li, Hang Zhou. Optic Perineuritis and its Association With Autoimmunne Diseases. Front Neurol 2021 (11) 1-9
2. Zahid S, Iqbal M. Systemic LUPUS Erythematosus presenting as Optic Neuropathy: a case report. Curius 2019 11 (6) e4806
3. Silpa-Archa, JJ Lee. Ocular manifestations in Systemic Lupus Erythematosus. Int Rheum Dis 2014; 17: 494-501

## P482 Cracking the enigma: unveiling the overlapping shadows of TTP, CAPS, and MAS in pediatric lupus – a diagnostic quest

### A. Ziv^1^, R. Haviv^1^, J. Yacobovich^2^, Y. Uziel^1^

#### ^1^Pediatrics, Meir Medical Center, Kfar Saba; ^2^Pediatrics, Schneider Children's Medical Center, Petach Tikva, Israel

##### **Correspondence:** A. Ziv

*Pediatric Rheumatology 2023*, **21(Suppl 2):**P482

**Introduction:** Systemic lupus erythematosus (SLE) can be associated with life-threatening complications, such as thrombotic thrombocytopenic purpura (TTP), catastrophic antiphospholipid syndrome (CAPS), and macrophage activation syndrome (MAS). Timely treatment decisions are often required in these acute and potentially fatal situations, even before a definitive diagnosis is established.

**Case presentation:** A 14-year-old girl, previously healthy, presented with fever, abdominal pain, and vomiting. Physical examination revealed a moderate ill appearance, with abdominal tenderness, livedo reticularis on her legs, oral aphthous ulcers and signs indicative of shock, including profound pallor and tachycardia. Laboratory findings demonstrated severe pancytopenia (WBC 2,100, hemoglobin 5.5, platelets 4,000), positive Coombs, schistocytes on blood smear, and acute renal impairment. Serological tests were positive for SLE, including ANA, dsDNA, low complement levels and triple antiphospholipid antibodies (APLA) positivity. Additional laboratory results showed undetectable ADAMTS13 activity (0) and positive ADAMTS13 antibodies. Natural Killer (NK) cell activity was substantially reduced. Abdominal CT revealed a large spleen infarct and polyserositis. Considering the diagnostic challenge, TTP, CAPS, and MAS were considered in the differential diagnosis.

Upon arrival, she was admitted to the pediatric intensive care unit for immediate resuscitation and intensive monitoring. She received pulse steroids and intravenous immunoglobulin (IVIG), resulting in clinical improvement. Due to severe thrombocytopenia, anticoagulant therapy was deferred. A multidisciplinary discussion involving rheumatology, hematology, nephrology, and the plasma exchange team was conducted. As there was no central nervous system involvement, plasmapheresis was not performed, and the decision was made to initiate rituximab therapy.

Rituximab treatment led to rapid improvement, allowing the initiation of low molecular weight heparin (LMWH). The patient was discharged and continued with rituximab, transitioning to belimumab. LMWH was switched to warfarin. The patient is currently in remission.

**Conclusion:** This case emphasizes the diagnostic challenges encountered in distinguishing severe SLE complications like TTP, CAPS, and MAS. A multidisciplinary approach involving specialists from various fields is essential for optimal management. Early initiation of targeted therapies, such as rituximab, can lead to favorable outcomes in complex pediatric SLE cases.

**Patient Consent**

Yes, I received consent

**Disclosure of Interest**

None declared

## P483 Rare genetic condition in patients of unusual course of juvenile arthritis

### I. Nikishina^1^, A. Arefeva^1^, S. Arsenyeva^1^, V. Matkava^1^, M. Kaleda^1^, T. Markova^2^

#### ^1^Paediatric, V.A. Nasonova Scientific Research Institute of Rheumatology; ^2^Research Centre for Medical Genetics, Moscow, Russian Federation

##### **Correspondence:** A. Arefeva

*Pediatric Rheumatology 2023*, **21(Suppl 2):**P483

**Introduction:** Multimorbid conditions substantially complicate the verification of the diagnosis and require multidisciplinary coordination and efforts of the medical team. Concomitant diseases and infections impair the patient (pts) management.

**Objectives:** To describe 2 clinical cases of adolescents with congenital insensitivity to pain due to SCN9A gene mutation: boy with chronic nonbacterial osteomyelitis (CNO) and JIA who have been observed in Pediatric department of V.A. Nasonova Research Institute of Rheumatology.

**Methods:** Description of 2 clinical cases of adolescents of 17 and 14 y.o. with the same mutation in SCN9A gene who have been observed in our clinic. One boy with CNO and history of sepsis and another boy with JIA and Ehlers-Danlos syndrome.

**Results:** 17 y.o. boy has history of synovitis of knees, elbows and ankles, generalized joint hypermobility and indifference to pain, e.g. recurrent home accident traumas and injuries. CT and whole-body MRI images demonstrate osteonecrosis femoral head, pelvic lesion, multiple osteitis, lumbar spine destruction and knee osteoarthritis. ENMG shows distal sensory aksonopathy of lower extremities. JIA and CNO were verified so NSAID and Etanercept (ETA) were administrated with good response. Genetic test showed mutations in the SCN9A gene (hereditary sensory and autonomic neuropathy type IID) encoding the sodium channel which is associated with loss of pain sensation and selective immunity depression to Staphylacoccus aureus (SA). In January 2021 he had SA sepsis with high intensive pain (the first time in his life) in his back and wrists. Antibacterial, fungicide therapy, norepinephrine, red blood cell and albumin transfusion, opioid analgesis were administrated. It took 6 months for recovery after surgical treatment of bilateral abscess of m. iliopsoas, purulent shoulder synovitis, L1-L2 corporectomy and spine implant instillation. Due to intensive septical condition treatment and gap in antirheumatic therapy polyarthritis had exacerbated and psorias de novo appeared. JAK-inhibitor, Tofacitinib (TOFA), was administrated with good efficacy.

**Conclusion:** This 2 clinical cases demonstrate the necessity of careful manage of pts with rare rheumatic diseases and severe comorbid problems such as hereditary sensory and autonomic neuropathy type IID caused by SCNA9A gene mutation and sepsis. Success of pts management depends on effective coordination of multidisciplinary medical team.

**Patient Consent**

Yes, I received consent

**Disclosure of Interest**

None declared

## P484 Clinical spectrum and outcome of children with deficiency of adenosine deaminase 2 (DADA2): multicentric experience from India

### N. K. Bagri^1^, S. Kumar^2^, A. Chugh^1^, C. Bhat^3^, P. Patra ^4^, V. Viswanathan^5^, A. Gupta^1^, P. Hari ^1^, A. V. Ramanan^6^, L. Lingappa ^7^

#### ^1^Pediatrics , All India Institute of Medical Sciences , New Delhi; ^2^Pediatrics, Christian Medical College, Vellore; ^3^Pediatric Rheumatology Services, Rainbow Children's Hospital, Banglore; ^4^Pediatrics, All India Institute of Medical Sciences, Patna ; ^5^Pediatrics, Jupiter Hospital, Thane, India; ^6^Pediatric Rheumatology, Bristol Royal Hospital for Children, Bristol, United Kingdom; ^7^Neurosciences, Rainbow Children's Hospital, Hyderabad, India

##### **Correspondence:** N. K. Bagri

*Pediatric Rheumatology 2023*, **21(Suppl 2):**P484

**Introduction:** Deficiency of adenosine deaminase-2 (DADA2) is a monogenic form of polyarteritis nodosa (PAN) with a wide spectrum of clinical presentation and can present to pediatric care providers across various sub-specialities. Whilst, cyclophosphamide, mycophenolate mofetil, and azathioprine are the usual DMARDs for the management of classic PAN, most children with DADA2 would respond to anti-tumour necrosis factor (anti-TNF) agents. The identification and differentiation of DADA2 from classic PAN is pivotal to reducing disease-related damage and for the timely initiation of the most appropriate therapy. We aim to describe the clinical spectrum and outcome of Indian children with DADA2 through this case series.

**Objectives:** To study the clinical spectrum and outcome of children with DADA-2.

**Methods:** A formal invitation to participate in the study through email along with the relevant Performa was sent to various centres managing complex rheumatic disorders including DADA2. The participating centres were asked to share deidentified data (through emails) collected during the management of these children (2014 – till date). The deidentified data from all participating centres were entered in an Excel sheet and the coordinating centre (AIIMS, New Delhi) screened the data for accuracy and completeness, and the respective sites were contacted in case of discrepancies. The cleaned dataset was analysed using Stata software to study predefined objectives.

**Results:** We enrolled 15 children (10 females) in the study; the mean (SD) age at the time of onset of symptoms for males and females was 46.2 (47) and 73.6 (50.4) months respectively. The diagnosis of DADA2 was confirmed with the clinical exome in all children. The most common clinical feature in this cohort was fever and rash which was present in 80% of patients. More than half of children n, (%) [8, (53%)] had a CNS stroke. The other clinical features were hypertension [5,(33%)], anaemia [3,(20%)] and arthralgia/arthritis in 4(26%) children. One child developed procedure-related thrombosis of the femoral artery. One child had pure red cell aplasia. The mean duration of follow-up in this cohort was 32 months. These children were managed with various immunomodulators; steroids [13, (86%)], anti-TNF agents [(12, (80%)], cyclophosphamide [2 (13%)] and mycophenolate mofetil [3,(20%)]. 14 children showed clinical improvement and achieved remission at the last follow-up. None of the patients had recurrent strokes after the initiation of anti-TNF drugs. One child with PRCA underwent a hematopoietic stem cell transplant and succumbed to procedure-related morbidities. None of the subjects developed a flare of latent tuberculosis or any other serious adverse event related to therapy.

**Conclusion:** DADA-2 closely resembles PAN; early age of onset and CNS stroke are striking differentiating features from classic PAN. Most children respond well to anti-TNF agents without any serious adverse events.

**Patient Consent**

Yes, I received consent

**Disclosure of Interest**

None declared

**References**

1. Kasap Cuceoglu M, Sener S, Batu ED, Kaya Akca U, Demir S, Sag E, et al. Systematic review of childhood-onset polyarteritis nodosa and DADA2. Seminars in Arthritis and Rheumatism. 2021 Jun;51(3):559–64.
2. Sharma A, Naidu G, Sharma V, Jha S, Dhooria A, Dhir V, et al. Deficiency of adenosine deaminase 2 (DADA2) in Adults and Children: Experience from India. Arthritis Rheumatol. 2021 Feb;73(2):276–85

## P486 Obvious only in retrospect: a cohort of Sting Associated Vasculopathy in Infancy (SAVI) without typical rash

### I. Maccora^1,2,3^, P. Vega-Fernandez^3,4^, K. Risma^4,5^, G. Schulert^3,4^

#### ^1^Rheumatology Unit, ERN ReConnet Center, Meyer Children's Hospital IRCCS; ^2^NeuroFARBA Department, University of Florence, Florence, Italy; ^3^Division of Rheumatology, Cincinnati Children's Hospital, Medical Centre; ^4^Department of Pediatrics, University of Cincinnati College of Medicine; ^5^Division of Allergy and Immunology, Cincinnati Children's Hospital Medical Center, Cincinnati, United States

##### **Correspondence:** I. Maccora

*Pediatric Rheumatology 2023*, **21(Suppl 2):**P486

**Introduction:** STING-associated vasculopathy with onset in infancy (SAVI) is characterized by systemic inflammation, skin vasculopathy and interstitial lung disease. However, since the initial description knowledge and clinical spectrum of this disease has substantially broadened.

**Objectives:** Here we present the clinical and laboratory characteristics of 2 families with SAVI presenting with autoimmunity and lung disease without cutaneous vasculopathy.

**Methods:** We performed a retrospective chart review of patients with a genetic diagnosis of SAVI followed at the Rheumatology division of Cincinnati Children’s Hospital Medical Center (CCHMC). We collected demographic, clinical, laboratory and radiological information.

**Results:** We identified 9 patients from 2 families. *Family A* consisted of 7 individuals (the father, 3 sons and 3 daughters) carrying the heterozygous variant in STING1 c.463G>A, previously reported as cohort with undiagnosed interstitial lung disease, arthritis, and atopic dermatitis. The diagnosis was confirmed reviewing their medical records and performing genetic testing for SAVI. *Family B* consisted of 2 individuals (father and daughter), carrying the c.842G>A variant. The median age at onset of symptoms in these 9 patients was 18 months (range 1-adulthood). The first symptom was eczema in 5 (55.5%), lung disease in 3 (33.3%), failure to thrive in 2 (22.2%), and arthritis in 1 (11.1%). Eight patients showed skin involvement (7 eczema and alopecia and 1 blistering rash), 7/9 lung involvement, 3/9 arthritis, 7/9 arthralgia and 1/9 increased liver enzymes. Seven patients underwent to lung CT that showed an interstitial lung disease of different severity characterized by minimal ground glass or nodular lesion up to diffuse cystic lesions to complete disruption of parenchyma. Lung biopsy performed in 5 patients showed chronic inflammatory bronchiolitis. Laboratory tests were available in 8 patients: all had increased ESR, 75% ANA and rheumatoid factor positivity, and 62.5% hypergammaglobulinemia. Lymphocyte subset showed expanded B cells in 75% (median 38.5% Range 31-53%), with decreased CD8+T in 71% (median 14%, R 10-38%) and 57% with increased CD4+/CD8. Plasma cytokines performed in 7 patients, showed increased value of IL1b (1/7), IL2 (2/7), IL4 (2/7), IL5 (4/7), IL8 (3/7) IL6 (5/7), TNFa (4/7). One patient of *Family A* died at age 9 years because of lung disease. The daughter of *family B,* after failing anti-TNF, received a JAK-inhibitor upadacitinib, with resolution of arthritis and improvement of lung function (spirometry) after 1.5 months of treatment.

**Conclusion:** The phenotype of SAVI may be extremely variable even within the same family. It is essential to reconsider prior cases with familial ILD and autoimmunity in order to identify the correct diagnosis and tailored treatment.

**Patient Consent**

Yes, I received consent

**Disclosure of Interest**

None declared

## P487 Chaple syndrome (complement hyperactivation, angiopathic thrombosis and protein losing enteropathy) managed with investigational C5 inhibitor, pozelimab

### N. P. Maldar^1^, A. Khan^1^, A. Prabhudesai^1^, A. Ozen^2^, R. Khubchandani^1^

#### ^1^Pediatric Rheumatology, SRCC Childrens Hospital, Mumbai, India; ^2^Department of Pediatrics, Division of Allergy and Immunology, Marmara University, School of Medicine, Istanbul, Türkiye

##### **Correspondence:** A. Prabhudesai

*Pediatric Rheumatology 2023*, **21(Suppl 2):**P487

**Introduction:** Described in 2017 (Ozen), this is an ultra-rare autosomal recessive disorder that occurs due to mutation in the CD55 gene. The phenotype consists of **APLE** (**a**ngiopathic thrombosis, **p**rotein losing enteropathy leading to abdominal pain, diarrhoea, hypoproteinaemia, edema, and malabsorption). Patients exhibit bowel inflammation, recurrent infections due to hypogammaglobulinemia and/or angiopathic thromboembolic phenomena.

**Objectives:** We describe probably the first case of CHAPLE disease from Southeast Asia and our experience with Pozelimab, a C5 inhibitor, obtained through the compassionate use programme of Regeneron

**Results:** A 4.5 year female, (Weight 13.7kg with severe edema, Height:93.5cm) born of a non-consanguineous marriage was referred to us with recurrent severe abdominal pain, diarrhoea, anasarca, failure to thrive and grade 4 clubbing. She had severe anaemia and hypoproteinaemia,with normal urinalysis and inflammatory markers. Onset of illness was at 8 months of age. Abdominal radio-imaging showed ascites, small bowel wall thickening and dilatation with thrombosis in the distal superior mesenteric vein and its branches. Right atrial and left pulmonary artery thrombosis was seen on 2DECHO. Gastroduodenoscopy and colonoscopy suggested active inflammation confirmed on biopsy.

An OMIM search with keywords ‘ thromboembolism and protein losing enteropathy’ led to a presumptive diagnosis of CHAPLE syndrome. Whole exome sequencing confirmed **a homozygous c.148G>T;p.Glu50Ter CD 55** 'likely pathogenic' mutation. Prednisolone (0.5 mg/kg) was started in the interim. Overactivity of the complement system in CHAPLE has been the basis for using C5 inhibitors, eg Eculizumab. Cost constraints prevented usage and Pozelimab, an investigational C5 inhibitor was initiated as a single loading dose (30 mg/kg IV) followed by 200mg subcutaneously weekly after parental consent, ethics approval and meningococcal vaccine. After 8 months of treatment the child was active, playful with mild pallor on clinical examination and a weight gain of 1.8 kg and height gain of 3.5 cms. Laboratory findings showed an increase in Hb from a nadir of 5.6 gms/dl to 9.6g/dL, platelet count reduced from 625 (x10^9^/L) to 368 (x10^9^/L). Total proteins increased from 2.5g/L (Alb 1.2 g/L/Glob 1.3 g/L) to 8.3 g/L (Alb 4.6 g/L/Glob 3.7 g/L) without albumin infusions and S. Iron increased to 26 ug/dL from 2 ug/dL. Vit B12 levels improved from <159pg/mL to 680 pg/mL. Steroids were discontinued by the 14th week of pozelimab therapy. One unexplained seizure occurred in the seventh week of treatment.

**Conclusion:** Our patient exhibited prototypic features and a previously described mutation. Pozelimab led to a dramatic anthropometric, clinical and laboratory improvement after a 8 month follow up.

**Patient Consent**

Yes, I received consent

**Disclosure of Interest**

N. Maldar: None declared, A. Khan: None declared, A. Prabhudesai: None declared, A. Ozen Conflict with: A.O. has a pending patent on C5 inhibitor treatment of CHAPLE. , R. Khubchandani: None declared

## P488 Perception and attitudes about physical activity in juvenile idiopathic arthritis

### A. K. Leos Leija^1^, F. García Rodríguez^1^, I. Pelaez Ballestas^2^, G. Burgos^3^, A. Loyola Sanchez^4^, A. V. Villarreal Treviño^1^, N. E. Rubio Pérez^1^

#### ^1^Pediatric Rheumatology, Hospital Universitario "Dr. José Eleuterio González", Monterrey; ^2^Hospital General de Mexico "Dr. Eduardo Liceaga", Ciudad de México, Mexico; ^3^Rheumatology, Alberta Health Services; ^4^Division of Physical Medicine and Rehabilitation, Faculty of Medicine and Dentistry, University of Alberta, Alberta, Canada

##### **Correspondence:** A. K. Leos Leija

*Pediatric Rheumatology 2023*, **21(Suppl 2):**P488

**Introduction:** The inability to maintain sufficient physical activity, in conjunction with increased sedentary behavior, is commonly observed in rheumatic diseases. It has been observed that pediatric patients with rheumatic diseases do not reach the minimum physical activity requirements per day and that physical training programs are able to improve general health and thus prevent disease-related symptoms without increasing the activity of the disease.

**Objectives:** To assess the different perspectives, fears, obstacles, facilitators, knowledge, and attitudes about physical activity in children with juvenile idiopathic arthritis (JIA).

**Methods:** A prospective study was carried out, using mixed methods and sequential evaluative design, including qualitative techniques. The patients and caregivers were included during their routine visits and the most common problems related to physical activity were intended to be detected. With this, we create an interview guide that was discussed by a group of experts and evaluated in a pilot study to confirm its relevance and understanding. The interview guide was divided into several domains and aimed at evaluating the participants' general knowledge about physical activity and exercise, their general adherence to it, as well as motivations, preferences, barriers, obstacles, and facilitators for their performance.

**Results:** Seventeen patients were included, of which 58% were male, with a median age of 12 years, the most common classifications in patients were rheumatoid factor-negative polyarthritis (6/17) and enthesitis-related arthritis (5/17). Ten patients (58%) had high disease activity at the time of evaluation, and 23% were in remission. Sixty-five percent of the patients did not exercise regularly, with disease status being the main reason. Of the 6 patients who did exercise regularly (35%), half reported doing so for pleasure and the other half to improve disease symptoms. Seventy percent of the patients had received information on the importance of exercising, in most cases given by physicians, of whom 42% already exercised regularly and 25% began to do so, however, they did not continue because considering that disease activity was an impediment. Evaluating the change since the time of diagnosis, 58% of the patients exercised before disease onset, however, half stopped doing so due to disease activity.

**Conclusion:** In our study, we observed that most of our patients with JIA do not exercise regularly, the pain and high activity of their disease being the main obstacle to doing so. Even though a smaller percentage of patients did acquire the habit of exercising, we observed that having found an interesting and fun activity for them was a factor that conferred success in continuing it in the long term. We consider it important to provide extensive information to our patients to encourage good habits and maintain multidisciplinary management.

**Patient Consent**

Not applicable (there are no patient data)

**Disclosure of Interest**

None declared

**References**

1. Pinto AJ, Yazigi Solis M, de Sá Pinto AL, Silva CA, Maluf Elias Sallum A, Roschel H, et al. Physical (in) activity and its influence on disease-related features, physical capacity, and health-related quality of life in a cohort of chronic juvenile dermatomyositis patients. Semin Arthritis Rheum. 2016 Aug 1;46(1):64–70.
2. Bohr AH, Nielsen S, Müller K, Karup Pedersen F, Andersen LB. Reduced physical activity in children and adolescents with Juvenile Idiopathic Arthritis despite satisfactory control of inflammation. Pediatric Rheumatology. 2015 Dec 10;13(1).
3. Henderson CJ, Lovell DJ, Specker BL, Campaigne BN. Physical Activity in Children with Juvenile Rheumatoid Arthritis: Quantification and Evaluation.

## P489 Do adolescents with familial mediterranean fever fulfill the daily physical activity recommendations?

### C. Uludag^1^, D. C. Sarac^2^, I. Inac^1^, B. Kasap Demir^3^, O. Altug Gucenmez^4^, D. Bayraktar^2^

#### ^1^Institute of Health Sciences, Department of Physiotherapy and Rehabilitation; ^2^Faculty of Health Sciences, Department of Physiotherapy and Rehabilitation; ^3^Faculty of Medicine, Department of Pediatrics, Izmir Katip Celebi University; ^4^Dr. Ozge Altug Gucenmez Clinic, Izmir, Türkiye

##### **Correspondence:** D. Bayraktar

*Pediatric Rheumatology 2023*, **21(Suppl 2):**P489

**Introduction:** Engaging in at least 60 minutes of moderate-vigorous physical activity daily is recommended for children and adolescents. The role of regular physical activity in disease management is well-documented in several previous studies on various rheumatic/inflammatory diseases. However, no previous study investigated the level of PA in adolescents with familial Mediterranean fever (FMF) which is a chronic auto-inflammatory disease.

**Objectives:** The aim of this cross-sectional study was to investigate the rates of adolescents fulfilling the daily physical activity recommendations.

**Methods:** Thirty-two adolescents (age: 15.4±1.7 years, 16 female) with FMF were evaluated regarding physical (sex, age, body-mass index) and disease-related characteristics (disease duration, medication). Disease activity was assessed with Pras Score. Physical activity was measured with a 3-axial accelerometer (Actigraph wGT3X-BT) which was worn for six days. Total moderate-vigorous physical activity (MVPA) was calculated for each day using an equation [moderate activity in minutes+(vigorous activity in minutes*2)]. The number of days fulfilling recommended physical activity level (60 minutes MVPA) was calculated.

**Results:** Mean disease duration was 8.3±4.2 years. Twenty-nine patients were using colchicine (91.6%), while three patients were on biologics (9.4%). The median Pras score was 8.0 (7.0/9.0) indicating a low disease activity. Median number of days for fulfilling daily recommendation was 1.0 (0.0/2.0). Thirteen patients did not perform recommended MVPA on any of the measured days.

**Conclusion:** Our results suggest that adolescents with FMF did not achieve daily recommended physical activity. Thus, any effort seems crucial for increasing the level of MVPA in adolescents with FMF.

**Patient Consent**

Yes, I received consent

**Disclosure of Interest**

None declared

## P490 Correlation of disease activity and psychiatric burden of juvenile idiopathic arthritis-interim analysis of a prospective observational study

### N. Singh^1^, J. Janardhanan^1^, S. Gopalan^2^, S. Bhattad^1^

#### ^1^Pediatric Immunology and Rheumatology; ^2^Clinical Psychology, Aster CMI Hospital, Bengaluru, India

##### **Correspondence:** N. Singh

*Pediatric Rheumatology 2023*, **21(Suppl 2):**P490

**Introduction:** Juvenile idiopathic arthritis (JIA) is the most common chronic rheumatic illness in children. Initial symptoms may be vague and its psychological impact often goes unnoticed in clinical practice. Hence objective measurement of disease activity and psychiatric burden in children with JIA is essential for optimizing treatment, disease monitoring and assessing efficacy of therapeutic interventions.

**Objectives:** We aimed to evaluate the association between disease activity and psychiatric burden of children with JIA.

**Methods:** The study is an ongoing observational study carried out in the Pediatric Rheumatology Division of Aster CMI Hospital, Bengaluru initiated in October 2022. An interim analysis was carried out in April 2023. Demographic data were collected from patient’s records, and disease activity was measured using the Juvenile Arthritis Disease Activity Score (JADAS)27 score (1). The patients underwent a psychiatric assessment by a trained psychologist using the Mini-International Neuropsychiatric Interview for Children and Adolescents (MINI kid) screen Kid & Kid Parent Version 7.0.2 (2). Those who tested positive, were subjected to the standard MINI Kid interview.

**Results:** Thirty-two outpatient JIA patients were included and classified as per the Pediatric Rheumatology International Trials Organization (PRINTO) Classification Criteria for JIA, 2019 into systemic JIA(n=12), early onset ANA positive JIA(n=9), Enthesitis/Spondylitis JIA (n=9), and RF Positive JIA(n=2).

The mean age at presentation was 120 months for SJIA, 84 months for early onset JIA, 160 months for Enthesitis/Spondylitis JIA and 113 months for RF Positive JIA. During disease measurement, four SJIA, seven Early Onset ANA Positive JIA, three Enthesitis/Spondylitis JIA and one RF positive JIA had disease remission and hence were not included in calculating the mean disease activity. Eight SJIA patients had a mean JADAS27 score of 20, two Early Onset ANA positive patients had a mean JADAS27 score of 8.5, six Enthesitis/Spondylitis JIA patients had a mean JADAS27 score of 9.5, and one RF positive JIA patient had JADAS27 score 23, respectively.

Of the thirty-two patients screened by the MINI Kid patient/parent version, two SJIA patients had depression. Both children were males, were in remission, and had developed these symptoms over two months with mean disease duration of three months. The identified triggers were child and parental stress and anxiety due to symptoms impacting the daily lifestyle and fear of recurrence of disease.

The depression was mild and both children were initiated on psychological therapy. Families were also included for providing psychoeducation and taught strategies for home management. The children are under follow-up and effect of intervention is yet to be assessed.

**Conclusion:** The interim analysis failed to show correlation between ongoing disease activity and psychiatric burden of the disease. However, the small sample size and lack of data on follow-up visits is a limitation. The definitive conclusion can be ascertained after culmination of the study.

**Patient Consent**

Yes, I received consent

**Disclosure of Interest**

None declared

**References**

1. Consolaro A, Ruperto N, Bazso A, Pistorio A, Magni-Manzoni S, Filocamo G, et al. Development and validation of a composite disease activity score for juvenile idiopathic arthritis. Arthritis Rheum. 2009;61(5):658-66.
2. Sheehan DV, Sheehan KH, Shytle RD, Janavs J, Bannon Y, Rogers JE et al. Reliability and Validity of the Mini International Neuropsychiatric Interview for Children and Adolescents (MINI–KID). J Clin Psychiatry; 2010;71(3):313-326.

## P491 Pain perception and sleep disturbances of children suffering from Chronic Non-bacterial Osteomyelitis (CNO)

### Y. Butbul Aviel^1^, A. Miller^1^, Y. H. Butbul Aviel^1^, R. Haviv^2^, G. Amarylio^3^, I. Tirosh^4^, S. Shpielman^4^, R. Semo Oz^3^, M. Heshin Bekenstein^5^

#### ^1^Department of Pediatric B, Ruth Rappaport Children's Hospital, Rambam Medical Center, Haifa; ^2^Pediatric Rheumatology Unit, Department of Pediatrics, Meir Medical Center, Kfar Saba; ^3^Pediatric Rheumatology Unit, Schneider Children's Medical Center of Israel, Petach Tikva; ^4^Edmond and Lily Safra Children’sHospital, Sheba Medical Center, Tel Hashomer, Deaptment of Pediatric A; ^5^Pediatric Rheumatology Service, Dana Children’s Hospital of Tel Aviv Medical center, Tel Aviv, Israel

##### **Correspondence:** Y. Butbul Aviel

*Pediatric Rheumatology 2023*, **21(Suppl 2):**P491

**Introduction:** Chronic nonbacterial osteomyelitis (CNO) is an inflammatory bone disorder that most frequently affects children and adolescents, pain is being a major symptom of CNO, affecting the quality of life of these children. Major research was done regarding the relation of chronic pediatric disease and its effect on sleep patterns and quality of life, yet no research was done for CNO affected individuals.

The aim of this study was to assess the pain perception in patients with CNO, its correlation to disease activity and the effect of the pain on quality of life and sleep disturbance.

**Objectives:** This was a prospective study enrolling thirty-six patients diagnosed with CNO in 5 pediatric rheumatology centers in Israel.

Pain was assessed by PROMIS (Patient-Reported Outcomes Measurement Information System) pain module questionnaires. Sleep disturbance was measured by Pittsburgh sleep quality index (PSQI), and quality of life was assessed by PedsQOL (The Pediatric Quality of Life Inventory) and CHAQ (Childhood health assessment questionnaire). Demographic as well as clinical data including laboratory findings, affected areas by MRI, and Physician global assessment were collected.

**Methods:** This was a prospective study enrolling thirty-six patients diagnosed with CNO in 5 pediatric rheumatology centers in Israel.

Pain was assessed by PROMIS (Patient-Reported Outcomes Measurement Information System) pain module questionnaires. Sleep disturbance was measured by Pittsburgh sleep quality index (PSQI), and quality of life was assessed by PedsQOL (The Pediatric Quality of Life Inventory) and CHAQ (Childhood health assessment questionnaire). Demographic as well as clinical data including laboratory findings, affected areas by MRI, and Physician global assessment were collected.

**Results:** 13/36 patients suffered from other autoimmune diseases such as psoriasis and IBD. Most of the patients (77%) were treated with NSAIDS and 2 patients (6%) were treated with anti TNF during the study.

The most affected areas were the lower limb (74%) and the pelvis (64.5%), and the median Physician global assessment was 1.

Seven patients (19%) reported disturbed sleep (PROMIS score>5), the median pain score was 2 at the time of filling the questionnaire (range 0-9) and median worst pain reported was 3.4 (0-10) .

Poor reported sleep highly correlated with physician global assessment and patient global assessment (p-0.03 and P- 0.029 respectively), and also correlated with high CRP (p-0.04) and pain at the time of fulfilling the questionnaire (p-0.048).

Although more patients with sleep disturbance reported abnormal CHAQ the differences didn’t reach significance (28.6% vs 13.8% p-0.57).

**Conclusion:** Sleep disturbance are prevalent among children with CNO. Sleep disturbance a strongly associated with increased pain and disease activity. Strategies aimed at improving sleep and reducing pain should be studied as possible ways of improving quality of life for children with CNO.

**Trial registration identifying number:** not applicable

**Patient Consent**

Not applicable (there are no patient data)

**Disclosure of Interest**

None declared

## P492 Young peoples’ perception of systemic disease risk stratification in primary care

### N. Goss^1^, M. Beresford^1,2^, C. Pain^2^

#### ^1^Department of Women's and Children's Health , University of Liverpool, Institution of Life Course and Medicine; ^2^Department of Paediatric Rheumatology , Alder Hey Children’s NHS Foundation Trust, Liverpool, United Kingdom

##### **Correspondence:** N. Goss

*Pediatric Rheumatology 2023*, **21(Suppl 2):**P492

**Introduction:** Patient and Public Involvement and Engagement (PPIE) research was conducted to directly inform a cohort study of primary care data assessing the risk of systemic disease in children and young people who present to primary care with mouth ulcers. However the findings gave insight into young people's (YPs) experiences of systemic disease, including Behcet's, systemic lupus erythematous (SLE) and inflammatory bowel disease (IBD); as well as their perceptions of risk stratification in primary care generally.

**Objectives:** Systematic PPIE research aimed to discover the views of YP on informing patients and their families of the risk of systemic disease given specific symptoms and demographics, in a primary care setting. Additional objectives were met relating to YPs insights into systemic disease experience through investigation and diagnosis specifically.

**Methods:** A multi-phase approach was adopted to address the aims. An initial meeting with 8 members of GenR Liverpool (an advisory group for YP interested in contributing to health care research), established important outcomes and language to be used in the project as a whole. Next, an online survey was distributed via social media posts and mailing lists, facilitated by various charities involved with each systemic disease (SLE, IBD and Behcet’s). A total of 218 responses provided further insight into the perception of proposed outcomes and language from both patients and parents. Finally, a meeting with six YP involved with YourRheum (a young person’s advisory group for patients with rheumatic conditions) discussed the results of the primary care cohort study in terms of acceptability and dissemination of the findings.

**Results:** In the first phase meeting, YP felt that it may be reassuring and empowering to know the systemic disease risk associated with symptoms and demographics. However, they stressed that there must be a balance between ‘realistic and scary’ when communicating risk, and that knowledge is only helpful if there is action that can be taken.

In the second phase, ‘red flag’ information and advice on symptomatic treatment were deemed to be most beneficial at the early stages of diagnosis. YP and their parents felt that a risk calculation would: raise awareness of rare conditions; reduce time to diagnosis; allow preparation time; validate presenting symptoms; provide reassurance; and potentially allow earlier action to be taken. Concerns regarding risk calculation in primary care included: oversimplifying complex presentations; causing unnecessary worry; the risk of misdiagnosis; and the potential to demoralise patients and their families.

In the final phase meeting, results of the primary care mouth ulcer cohort study were presented and it was deemed that since the risk was ‘low’ (in this study) so it would be unhelpful to be informed of the exact risk of systemic disease given the presence of mouth ulcers. However, some patients/ parents may be reassured. Nevertheless, since the COVID-19 pandemic, YP with experience of rheumatic conditions were keen to highlight the mental health impact of being labelled ‘at risk’. YP preferred age- appropriate, positive language, ideally communicated ‘face-to-face’ by consultants, when receiving information about risk.

**Conclusion:** Overall this PPIE research provides insight into the thoughts, feelings and opinions of YP around the risk stratification of systemic disease in primary care. These views must be considered in any aspect of care for YP with systemic disease, particularly during early stage diagnosis or cases of diagnostic uncertainty.

**Patient Consent**

Not applicable (there are no patient data)

**Disclosure of Interest**

None declared

## P493 Newly diagnosed children and adolescents with Juvenile Idiopathic Arthritis (JIA): development, implementation and evaluation of a dialogue tool

### C. S. Jensen, K. S. Graarup

#### Department of Paediatrics and Adolescent Medicine, Aarhus University Hospital, Aarhus, Denmark

##### **Correspondence:** C. S. Jensen

*Pediatric Rheumatology 2023*, **21(Suppl 2):**P493

**Introduction:** Studies have shown that information about illness is not always well understood by patients. We wished to improve the quality of the information offered to newly diagnosed children and adolescents with JIA and their parents. We wished to convey homogenous and systematic information about JIA 1-2 months after the diagnosis as well as offer an individual dialogue focusing on individual needs and concerns.

**Objectives:** To develop, implement and evaluate a dialogue tool to ensure homogenous, systematic and age-relevant information to newly diagnosed children and adolescents with JIA and their parents. Information should focus on JIA, treatment and psychosocial aspects.

**Methods:** This study was a quality improvement study. We developed a dialogue tool based on literature, our clinical knowledge and semistructured interviews with nurses in paediatric rheumatology (N=5). The tool was tested in our clinical setting and evaluated using a qualitative approach. We evaluated the usefulness and acceptability using informal dialogue with children (N=8) and adolescents (N=7) and their parents (N=19). Data was analysed using content analysis.

**Results:** We identified six themes as essential: What is top of mind for you?, What is JIA?, Symptoms, Medicine, Living with JIA, and Hospital appointments. To ensure information was systematic and homogenous, we developed a written tool to be used by all nurses. Children, adolescents, their parents and nurses were positive and only minor adjustments were made to the tool. Based on their feedback we also found that having a structured dialogue 1-2 months after the diagnosis was too early, and that outcome was better for most children and adolescents when the dialogue took place 3-6 months after the diagnosis.

**Conclusion:** Our dialogue tool has ensured a systematic and homogenous approach and were positively evaluated by both children, adolescents, parents as well as nurses . All newly diagnosed children and adolescents with JIA and their parents are now offered a 1-hour nurse-led dialogue 3-6 months after the diagnosis in connection with a doctor-led appointment at the hospital.

A further development of the tool could preferably include adjustment of the tool to visually and verbally accommodate different age groups.

**Patient Consent**

Not applicable (there are no patient data)

**Disclosure of Interest**

None declared

## P494 Sleep problems in children with juvenile idiopathic arthritis - a cross-sectional and analytical study

### J. M. Nascimento^1^, J. Silva^2^, S. Ferreira^3^, M. Salgado^1^, P. Estanqueiro^1^

#### ^1^Pediatric Rheumatology; ^2^Faculty of Medicine; ^3^Pediatric Pulmonology and Sleep Medicine, Centro Hospitalar e Universitário Coimbra, Coimbra, Portugal

##### **Correspondence:** J. M. Nascimento

*Pediatric Rheumatology 2023*, **21(Suppl 2):**P494

**Introduction:** Children with juvenile idiopathic arthritis (JIA) and their parents report significantly more cases of nocturnal awakenings, parasomnias, excessive daytime sleepiness and sleep-related anxiety. Otherwise, sleep problems can increase pain reports and be responsible for a worse control of chronic diseases.

**Objectives:** Our aim was to analyze the quality and quantity of sleep in patients with JIA and determine the diagnostic and sleep therapeutic approaches that should be implemented in these patients.

**Methods:** A cross-sectional and analytical study of the quality and quantity of sleep was carried out in children aged 2 to 17 years with JIA, who attended outpatient pediatric rheumatology appointment from June 2022 to March 2023. The following scales/inquiries were used in the study: JADAS-27 (to assess disease activity), CSHQ-PT (qualitative assessment of sleep) and BEARS (screening questionnaire for sleep disorders). The patients underwent an objective sleep assessment using a non-invasive technique, actigraphy. Parental consent and ethical approval by ethics committee from Centro Hospitalar e Universitário de Coimbra were obtained.

**Results:** We evaluated data from 22 patients (16 females) aged between 2 and 17 years. 50% were persistent oligoarticular and 72.7% were on methotrexate. Two were on biologics (anti-TNF). We had actigraphy data from 8 patients. Our first outcome point was to compare the sleep duration in JIA patients with the recommended sleep duration for each age defined by Portuguese Pediatric Society. It was found that the median of the observed total sleep time (491.0 minutes) differed in a statistically significant manner from the median of the midpoint (630.0 minutes).The associations of disease activity with total sleep time and wake after sleep onset (WASO) proved to be positive and moderate (rs= 0.552 and rs=0.561 respectively), but without statistical significance. A marginal association of resistance to going to bed with a higher CSHQ-PT score (p=0.063) was detected. No statistically significant differences were observed when comparing the three diagnoses of Extended Oligoarticular, Persistent Oligoarticular and Polyarticular (negative rheumatoid factor) in the CSHQ-PT questionnaires (p=0.232). Younger kids (onset ≤ 5 years) with JIA seem to have had more anxiety related with sleep and bedtime resistance in CSHQ-PT score but the results from actigraphy showed a short sleep onset latency in all 8 patients (p=0.560).

**Conclusion:** To our knowlegde this is the first portuguese study who tried to analyze the quality and quantity of sleep in a group of JIA patients. A higher disease activity was related with less total sleep time and more sleep fragmentation. However, we did not find any relation between sleep problems and number of involved joints or the use of anti-TNF (only two patients). Inconsistent findings in the various evaluated parameters highlight a complex bidirectional relationship between JIA and sleep, which deserves to be addressed in future longitudinal studies with a larger sample size. Nevertheless, we propose a sleep screening diagnostic algorithm for JIA patients.

**Patient Consent**

Yes, I received consent

**Disclosure of Interest**

None declared

**References**

1. Saidi O, Rochette E, Bourdier P, Ratel S, Merlin E, Pereira B, Duché P. Sleep in children and adolescents with juvenile idiopathic arthritis: a systematic review and meta-analysis of case-control studies. Sleep. 2022 Feb 14;45(2):zsab233. doi: 10.1093/sleep/zsab233. PMID: 34525202.

## P495 Comparison of the frequency of juvenile primer fibromyalgia syndrome in pediatric patients presenting with juvenile idiopathic arthritis and familial mediterranean fever with the healthy control group

### G. Özomay Baykal, B. Sözeri

#### Pediatric Rheumatology, Umraniye Training and Research Hospital, Istanbul, Türkiye

##### **Correspondence:** G. Özomay Baykal

*Pediatric Rheumatology 2023*, **21(Suppl 2):**P495

**Introduction:** Juvenile Idiopathic Arthritis (JIA) and Familial Mediterranean Fever (FMF) are chronic diseases that constitute an important part of childhood rheumatic diseases and are characterized by joint pain, swelling, and morning stiffness. The coexistence of Fibromyalgia with inflammatory rheumatic diseases has been shown in adult patients, and it has been reported that FM may accompany Rheumatoid Arthritis (RA) in 13% of patients.

**Objectives:** The aim of this study is to evaluate the frequency of Juvenile Primer Fibromyalgia (JPFS) in patients diagnosed with childhood inflammatory arthritis (JIA) and FMF with joint symptoms.

**Methods:** Patients who applied to Pediatric Rheumatology Clinic at Umraniye Training and Research Hospital between September-December 2022 with JIA and FMF diagnoses, and healthy control group who applied to general pediatrics on the same dates were included. A total of 121 patients and 60 healthy individuals who met these criteria were evaluated. Eligible patients between the ages of 12 and 18 were evaluated according to the ACR 2010 diagnostic criteria. The pain and symptom assessment scale (PSAT) used for JPFS diagnosis was applied and output data were analyzed with the widespread pain index (WPI) and symptom severity scale (SS).

**Results:** A total of 121 patients (52 JIA, 69 FMF) and 60 healthy controls were included in the study. Of the patient group, 57% (69) were followed up with a diagnosis of FMF, and 43% (52) were followed up with a diagnosis of JIA. Of the JIA patients, 53.8% (28) were oligoarticular, 7.7% (4) were polyarticular, and 38.5% (20) were diagnosed with spondyloarthropathy. The median disease duration was 4 years (1-16). When the PSAT measurements of the patient and control groups were compared with those diagnosed with JPFS, 12% (14) of the patient group and 2% (1) of the control group were diagnosed with JPFS and statistical significance was detected (p<0.05). JPFS diagnosis was made in 19% (10) of the 52 patients diagnosed with JIA, 5% (4) of the 69 patients diagnosed with FMF, and 1% (1) of the healthy control group. The median age of patients diagnosed with JPFS was 16 (12-18) years. 87% (13) of those diagnosed with JPFS were female and 13% (2) were male, and a statistically significant difference was found (p<0.05). 40% (4) of JIA patients diagnosed with JPFS were oligoarticular, 60% (6) were spondyloarthropathy, and 58% (25) of FMF patients diagnosed with JPFS met the diagnostic criteria. The most common symptoms in JPFS patients were myalgia, fatigue, muscle weakness, headache, and irritability.

**Conclusion:** In patients with JIA and FMF who complain of chronic musculoskeletal pain, fatigue, and weakness, JPFS diagnosis should always be considered in clinical evaluation. It is intended to point the importance of evaluation for JPFS diagnosis for patients who apply to the pediatric rheumatology clinic with chronic, musculoskeletal pain.

**Patient Consent**

Not applicable (there are no patient data)

**Disclosure of Interest**

None declared

**References**

1. Kashikar-Zuck S, Ting TV. Juvenile fibromyalgia: current status of research and future developments. Nat Rev Rheumatol. 2014 Feb;10(2):89-96. doi: 10.1038/nrrheum.2013.177. Epub 2013 Nov 26. PMID: 24275966; PMCID: PMC4470499.
2. Coles ML, Weissmann R, Uziel Y. Juvenile primary Fibromyalgia Syndrome: epidemiology, etiology, pathogenesis, clinical manifestations and diagnosis. Pediatr Rheumatol Online J. 2021 Mar 1;19(1):22. doi: 10.1186/s12969-021-00493-6. PMID: 33648522; PMCID: PMC7923821.

## P496 School well-being of children with juvenile idiopathic arthritis – a population-wide study

### M. J. Pedersen^1^, C. Høst^2^, S. N. Hansen^1^, J. Klotsche^3^, K. Minden^3^, B. Deleuran^4,5^, B. H. Bech^1^

#### ^1^Department of Public Health, Aarhus University; ^2^Department of Paediatric and Adolescent Medicine, Aarhus University Hospital, Aarhus, Denmark; ^3^Deutsche Reuma-Forschungszentrum Berlin, Berlin, Germany; ^4^Department of Biomedicine, Aarhus University; ^5^Department of Rheumatology, Aarhus University Hospital, Aarhus, Denmark

##### **Correspondence:** M. J. Pedersen

*Pediatric Rheumatology 2023*, **21(Suppl 2):**P496

**Introduction:** Multiple studies have reported on the quality of life (QoL) and well-being of children with juvenile idiopathic arthritis (JIA). Most of these studies have found impaired QoL and well-being among these children both at the time of diagnosis and into adulthood. No study has to our knowledge compared the school well-being (SWB) of children with JIA to the general population on a national level.

**Objectives:** We aimed to compare the SWB of children with JIA to the rest of the Danish population within different categories of school well-being. Of specific interest was the possible effect of socioeconomic status (SES) on the association between JIA and SWB.

**Methods:** We performed a cross-sectional population-wide register-based study. The study population was defined as all children participating in the annual National Well-being Survey (NWS) from the 4^th^ to 9^th^ grades from 2015-2019. At least five hospital contacts with a diagnosis code of JIA (ICD-10 codes DM08 and DM09) was used to define JIA patients in our study. The outcome of our study was the results of the 8 best validated questions within different domains of SWB within the 40 questions in the NWS. We performed ordinal logistic regression of the specific questions of the NWS comparing children with JIA to children with no JIA.

**Results:** Children with JIA reported similar SWB compared to their peers. The only question with significant lower reporting among children with JIA was on the question “I perform well in school”. Questions concerning stomach-ache, headache, loneliness, student support, and the ability to achieve goals did not significantly differ between groups. Children with JIA of parents with short education or low income tended to report lower SWB compared to peers, however, only few questions showed statistically significant differences.

**Conclusion:** We found no significant differences on SWB between children with JIA and their peers except for their own perception of school performance. We found, however, a tendency towards lower SWB among children with JIA of parents with low income and short education.

**Patient Consent**

Not applicable (there are no patient data)

**Disclosure of Interest**

None declared

## P497 A theoretical model with a cellular pathway for the mechanism of "Juvenile Fibromyalgia Syndrome" and psychosomatic syndromes ("Fascial Armoring" as a disease of western lifestyle)

### S. Plaut

#### Department of Basic and Clinical Sciences, University of Nicosia, Nicosia, Cyprus

##### **Correspondence:** S. Plaut

*Pediatric Rheumatology 2023*, **21(Suppl 2):**P497

**Introduction:** Fibromyalgia is a prevalent condition with significant burden and morbidity. While juvenile fibromyalgia (JF) occurs in ~2-6 percent of the pediatric population, current theories for its etiopathogenesis over-rely on psychological factors. Biopsychosocial theories describe this disease as the misfortunate fate of traumatized and stressed individuals that have behavioral, cognitive, social, and/or genetic predisposition for an infinite positive-feedback of pain with no peripheral organic lesion/injury. Meanwhile, a recent study suggested a mechanism involving myofascial myofibroblasts (MF) in the pathophysiology of fibromyalgia.^1^

**Objectives:** This work offers a theoretical model to help explain the mechanism of JF and psychosomatic syndromes, based on cross-disciplinary empirical studies.

**Methods:** Systematic and scoping literature search on fibromyalgia, JF, fascia, and MF, searching multiple phrases in MEDLINE, EMBASE, COCHRANE, PEDro, and medRxiv, majority with no time limit. Inclusion/exclusion based on title/abstract, then full text inspection.

**Results:** Fascia is a complex continuous interconnected tissue network that has qualities of tensegrity as well as myofascial chains.^1,2^ MF form a collaborating network of contractile cells that create mechanical tension.^1,3^ Chronic changes in extracellular matrix remodeling pathways and dysregulated MF activation contribute to various disease states, and there is empirical evidence to support fascia as having a role in the pathophysiology of fibromyalgia.^1^ Factors such as obesity, immobility, mechanical stimuli, hypermobility syndrome, pollution, and diet, can affect MF biology.

**Conclusion:** 'JF' is suggested to be the result of myofascial MFs that have contractile activity which may help explain findings in JF/fibromyalgia such as pain, decreased pressure pain threshold, trigger/tender points, chronic fatigue, cardiovascular and metabolic abnormalities, autonomic abnormalities, various psychosomatic symptoms, silent imaging investigations, and other phenomena (e.g., close association with joint hypermobility). Alpha smooth muscle actin and transforming growth factor β-1 pathways are at the core of this mechanism. 'Functional'/'psychosomatic' conditions and JF are suggested to share a common mechanism (myofibroblast-generated-tensegrity-tension) resembling a mild-moderate global exertional chronic compartment-like syndrome.^1^ The theoreical model of "fascial armoring" links between unhealthy lifestyle (eg, sedentarism, western diet, obesity, pollution and toxins/herbicides, and mechanical factors such as tight clothes) and pain and suffering.

**Patient Consent**

Not applicable (there are no patient data)

**Disclosure of Interest**

None declared

**References**

1. Plaut S. Scoping review and interpretation of myofascial pain/fibromyalgia syndrome: An attempt to assemble a medical puzzle. PLoS ONE 2022;17(2): e0263087.
2. Wilke et al. Not merely a protective packing organ? A review of fascia and its force transmission capacity. J Appl Physiol (1985). 2018 Jan 1;124(1):234-244.
3. Tomasek et al. Myofibroblasts and mechano-regulation of connective tissue remodelling. Nat Rev Mol Cell Biol. (2002) 3(5): 349-63.

## P498 An unexpected consequence of ebv infection: the need for a multidisciplinary approach - a case report

### I. Radoš^1^, K. Rubelj^1^, S. Roglić^2^, N. Rajačić^3^, I. Sitaš^1^, M. Vidović^1^

#### ^1^Pediatrics, University Clinical Hospital Center Sestre Milosrdnice; ^2^Pediatrics, University Hospital for Infectious Diseases; ^3^Oncology, Children`s Hospital Zagreb, Zagreb, Croatia

##### **Correspondence:** I. Radoš

*Pediatric Rheumatology 2023*, **21(Suppl 2):**P498

**Introduction:** Epstein-Barr virus (EBV) infection in young children is usually a self-limiting condition, but in adolescents, it can cause clinical symptoms typical of infectious mononucleosis (IM) with fever, pharyngitis, lymphadenopathy, splenomegaly and fatigue. Nodular changes in the lungs are rare and raise suspicion of other conditions, including autoimmune diseases.

**Objectives:** We present a case of a patient with EBV infection and nodular changes in the lungs, the differential diagnosis, and the outcome.

**Methods:** A 13-year-old boy was admitted to the Clinical Hospital Center Sestre Milosrdnice, Department of Pediatrics with a cough, sore throat, and fatigue without fever. Anemia, lymphocytosis with reactive lymphocytes, slightly elevated inflammatory parameters, and elevated liver enzymes were observed. Clinical findings included a hyperemic pharynx, tonsils covered with fibrin, normal auscultation, and palpatory mild spleen enlargement. A chest radiogram showed two lung nodules in the right lung, and azithromycin was introduced. Despite antimicrobial therapy, repeated laboratory findings showed elevated inflammatory parameters, transaminases, and lymphocytosis. Due to persistent changes in the lungs, a CT scan of the chest was performed, which showed 5 nodular lesions in addition to mild splenomegaly.

**Results:** Differential diagnoses of our patient with nonspecific symptoms and nodular changes in the lungs included various infections and other conditions like neoplasms and autoimmune diseases. Malignant disease was ruled out by a hematological workup. Due to the positive finding of extractable nuclear antigen (ENA) patient was screened for hypersensitivity pneumonitis, sarcoidosis, SLE and Sjögren syndrome and all results came up negative. In the meantime. An acute EBV infection was confirmed by a serological test, and in consultation with an infectious disease specialist, symptomatic treatment and regular follow-up by a hemato-oncologist and a rheumatologist were agreed. CT performed after 6 months showed complete regression of the changes with normal laboratory findings including immunological panel and clinical status.

**Conclusion:** Multiple pulmonary lung lesions as part of EBV infection in immunocompetent patients are extremely rare. The probable pathophysiological mechanism is an immune-mediated reaction, the therapeutic approach is conservative, but monitoring is necessary due to the documented link between EBV infection and autoimmune disease development, SLE being one of them.

**Patient Consent**

Yes, I received consent

**Disclosure of Interest**

None declared

## P499 How to identify probable fatigue in patients with juvenile idiopathic arthritis using the Juvenile Arthritis Multidimensional Assessment Report (JAMAR)

### A. Vroegindeweij^1^, S. A. Musterd^1^, E. M. Van De Putte^2,3^, N. M. Wulffraat^1,3^, M. Albertella^4^, A. Consolaro^5^, S. L. Nijhof^2,3^, J. F. Swart^1,3^

#### ^1^Pediatric Rheumatology/Immunology; ^2^Social Pediatrics, Wilhelmina Children's Hospital, University Medical Center Utrecht; ^3^Medicine, Utrecht University, Utrecht, Netherlands; ^4^Medicine; ^5^Neurosciences, Rehabilitation, Ophtalmology, Genetics and Maternal-Infantile Sciences, University of Genoa, Genoa, Italy

##### **Correspondence:** A. Vroegindeweij

*Pediatric Rheumatology 2023*, **21(Suppl 2):**P499

**Introduction:** One out of four patients with Juvenile Idiopathic Arthritis (JIA) suffers from severe fatigue (1). The Juvenile Arthritis Multidimensional Assessment Report (JAMAR) is widely used in clinical care to monitor JIA patients’ well-being (2) but does not include direct assessment of fatigue.

**Objectives:** We evaluated whether two quality of life (QoL) items that are already included in the JAMAR can be used together as a proxy to identify probable fatigue.

**Methods:** 105 JIA patients at the Wilhelmina Children’s Hospital completed the JAMAR and Checklist Individual Strength-8 (CIS-8), a validated questionnaire on fatigue (3). Principal Component Analysis (PCA) was used on all JAMAR QoL items to evaluate which items had the highest factor loadings on fatigue. We expected that the two items selected as proxy would have the highest loadings, which are items on experiencing issues with energy and concentration, each scaled from 0 (never) to 3 (every day). With binary logistic regressions and ROC analyses, the predictive value and favored cut-off of the proxy were determined and compared to the most predictive CIS-8 item. The proxy was re-used in the EPOCA dataset (*N* = 8618) (4) for validation.

**Results:** The CIS-8 identified 30 patients as severely fatigued (28.6%). The PCA indicated that only the item on energy issues had a high factor loading on fatigue of .716, but additional analyses showed that using the two items combined outperformed the use of one or other QoL items. The binary logistic regression indicated that controlled for age, sex, and disease activity, the proxy was a significant predictor of fatigue (*p* < .001). From the ROC followed an Area Under the Curve (AUC) of .911, sensitivity of 90.0%, and specificity of 69.6% for favored cut-off ≥1. The proxy neared the performance of the most predictive CIS-8 item (i.e., sensitivity 93.3%, specificity 78.0%). With ≥1 as cut-off, 61 out of 105 patients (58.1%) were identified with probable fatigue. An equivalent percentage was identified in the large EPOCA dataset, namely 56.6%.

**Conclusion:** The proxy can be used in clinical practice to identify probable fatigue. If patients indicate to experience energy or concentration issues sometimes to every day, administering an ad-hoc questionnaire on fatigue is recommended to quantify fatigue severity.

**Patient Consent**

Yes, I received consent

**Disclosure of Interest**

None declared

**References**

1. Nijhof LN, van de Putte EM, Wulffraat NM, Nijhof SL. Prevalence of severe fatigue among adolescents with pediatric rheumatic diseases. Arthritis Care Res (Hoboken). 2016;68(1):108–14.
2. Filocamo G, Consolaro A, Schiappapietra B, Dalprà S, Lattanzi B, Magni-Manzoni S, et al. A new approach to clinical care of juvenile idiopathic arthritis: the Juvenile Arthritis Multidimensional Assessment Report. J Rheumatol. 2011;38(5):938–53.
3. Hewlett S, Dures E, Almeida C. Measures of fatigue: Bristol rheumatoid arthritis fatigue multi-dimensional questionnaire (BRAF MDQ), Bristol rheumatoid arthritis fatigue numerical rating scales (BRAF NRS) for severity, effect, and coping, chalder fatigue questionnaire (CFQ), checklist. Arthritis Care Res (Hoboken). 2011;63(S11):S263–86.
4. Consolaro A, Ruperto N, Filocamo G, Lanni S, Bracciolini G, Garrone M, et al. Seeking insights into the EPidemiology, treatment and Outcome of Childhood Arthritis through a multinational collaborative effort: Introduction of the EPOCA study. Pediatric rheumatology. 2012;10(1):1–6.

